# An advanced draft genome assembly of a *desi* type chickpea (*Cicer arietinum* L.)

**DOI:** 10.1038/srep12806

**Published:** 2015-08-11

**Authors:** Sabiha Parween, Kashif Nawaz, Riti Roy, Anil K. Pole, B. Venkata Suresh, Gopal Misra, Mukesh Jain, Gitanjali Yadav, Swarup K. Parida, Akhilesh K. Tyagi, Sabhyata Bhatia, Debasis Chattopadhyay

**Affiliations:** 1 National Institute of Plant Genome Research (NIPGR), Aruna Asaf Ali Marg, New Delhi 110067, India

## Abstract

Chickpea (*Cicer arietinum* L.) is an important pulse legume crop. We previously reported a draft genome assembly of the *desi* chickpea cultivar ICC 4958. Here we report an advanced version of the ICC 4958 genome assembly (version 2.0) generated using additional sequence data and an improved genetic map. This resulted in 2.7-fold increase in the length of the pseudomolecules and substantial reduction of sequence gaps. The genome assembly covered more than 94% of the estimated gene space and predicted the presence of 30,257 protein-coding genes including 2230 and 133 genes encoding potential transcription factors (TF) and resistance gene homologs, respectively. Gene expression analysis identified several TF and chickpea-specific genes with tissue-specific expression and displayed functional diversification of the paralogous genes. Pairwise comparison of pseudomolecules in the *desi* (ICC 4958) and the earlier reported *kabuli* (CDC Frontier) chickpea assemblies showed an extensive local collinearity with incongruity in the placement of large sequence blocks along the linkage groups, apparently due to use of different genetic maps. Single nucleotide polymorphism (SNP)-based mining of intra-specific polymorphism identified more than four thousand SNPs differentiating a *desi* group and a *kabuli* group of chickpea genotypes.

Chickpea (*Cicer arietinum L.*) ranks third after common bean (*Phaseolus vulgaris*) and peas (*Pisum sativum*) in grain legume production and the only large-scale cultivated crop within *Cicer* genus^1^. It has been cultivated for about 7500 years and has suffered a series of evolutionary bottlenecks due to fungus infection and during domestication resulting in a narrow genetic base^2^,^3^. Cultivation in marginal lands and other reasons such as fungal infection and pod borer devastation resulted in an average productivity of 0.9 ton/hectare, far below than its potential of 6 ton/hectare^4^,^5^. Cultivated chickpea is of two types according to seed morphology, *desi* type with wrinkled, dark-brown colored small seeds and *kabuli* type with light-brown colored bold seeds.

Due to its high nutritional value and commercial importance, significant efforts are currently being made recently to improve the chickpea genomics resources. A series of reports describing high-density inter- and intra-species genetic map of chickpea using simple sequence repeat (SSR) and single nucleotide polymorphism (SNP) markers have been published^6^,^7^,^8^. In addition to the traditional polymerase chain reaction (PCR)-based marker genotyping assays, high -throughput technology platforms such as GoldenGate/Infinium, Diversity arrays technology (DArT), KBioscience competitive allele-specific polymerase chain reaction (KASPar) and genotyping-by-sequencing (GBS), exploiting mostly SNPs have been used to construct comprehensive genetic maps and understand the domestication process of chickpea^9^,^10^,^11^,^12^,^13^,^14^. Similarly, several reports describing transcriptomes of cultivated *desi* and *kabuli* genotypes of chickpea as well as its wild progenitor *C. reticulatum* are also now available^15^,^16^,^17^,^18^. High-throughput tissue-specific transcript profiling during vegetative, reproductive and root nodule development and under stress treatments have been reported to facilitate functional dissection of chickpea plant development and nitrogen fixation^19^,^20^,^21^,^22^. Functionally relevant markers are being developed utilizing transcriptome-based SSRs and SNPs for genetic and association mapping of traits^23^,^24^. Gene expression is post-transcriptionally regulated by microRNAs. High-throughput small RNA sequencing studies to discover tissue-specific and stress-responsive expression profile of chickpea microRNAs have been reported^25^,^26^.

All these efforts have brought about a significant enrichment of the in enriching chickpea genomic information, which are certainly useful for the marker-trait association, comparative sequence analyses of orthologous genes and improvement of chickpea by genetic transformation. On the other hand, effective anchoring of sequence scaffolds to the genetic linkage groups based on genetic markers to have a chromosome-level genome assembly is essential to identify genetic loci governing traits followed by marker-assisted breeding and comparative mapping with other species. To this end, two independent initiatives have published two draft sequences, one each of a *kabuli* type (CDC Frontier) and a *desi* type (ICC 4958) chickpea^27^,^28^. Draft sequences are continuously being used to identify trait-linked loci and comparative mapping of genetic markers^29^,^30^. Draft sequences are subject to correction depending on the advancement of genetic and physical maps and chromosomal genomics data. Further sequencing of new genomic DNA libraries is also required to fill the sequence gaps within the sequence scaffolds so that genes present in the gaps could be annotated. High-density genetic and physical maps constructed using various technologies are continuously being made available. In this study, an advanced draft of a *desi* type chickpea genome assembly is reported. We sequenced new genomic DNA libraries to improve scaffold length and fill the sequence gaps. We used a recently developed high-density genetic map, constructed using an inter-specific bi-parental mapping population (*C. arietinum* cv. ICC 4958 X *C. reticulatum* cv. PI 489777), to anchor more sequence scaffolds of the cultivar ICC4958 to the linkage groups, which resulted in 2.7-fold increase in the total pseudomolecule length in comparison to the previous assembly. Moreover, gene prediction and annotation were carried out using advanced tools and databases resulting in the annotation of 2686 more genes. Comparison of the genome assemblies of *desi* and *kabuli* type chickpeas revealed an overall collinearity with some structural deviations, some of which might have resulted from the use of different genetic maps for anchoring.

## Results and Discussion

### Sequencing and reassembly

We previously reported the draft genome assembly of the *desi* type chickpea cultivar ICC 4958 using 13.4 Gb and 43.7 Gb of sequence data generated by 454/Roche GS FLX Titanium and Illumina GAIIx platforms, respectively^27^. We further generated 1.01 Gb sequence data from two mate pair (MP) libraries with insert size of 20 kb as well as 23.93 Gb paired-end (PE) sequence data from a whole genome shotgun (WGS) library with insert size of 320–620 bp using 454/Roche and Illumina Hiseq1000 platforms, respectively (Supplementary Table S1). The new MP sequence data was used to improve the scaffold size of the previous assembly using NEWBLER v2.6 tool. Scaffolds and contigs of size less than 1 kb were removed. As described previously, publicly available bacterial artificial chromosome (BAC)-end sequences were used for further scaffolding^14^,^27^. All these approaches reduced the number of scaffolds from 182,734 to 42,978. Sequence gaps total of 27,381,655 bases within the scaffolds were filled with PE sequence data generated by the Illumina platform using SOAP *de novo* gap closure tool version 1.1 and, thereby, reduced the N-content from 13.8% in the previous assembly to 8.68%, much lower than the CDC Frontier assembly (15.20%)^31^,^28^. A sequence span of all the scaffolds was 511,022,194 bases, of which scaffolds larger than 5 kb constituted more than 88% of the assembly. Sequences of bacterial origin were removed or trimmed by us and additionally by National Center for Biotechnology Information (NCBI). After removal, the scaffolds were assigned to their corresponding linkage groups (LGs) using marker sequences of an advanced inter-specific genetic linkage map (manuscript submitted) constructed using a bi-parental mapping population of *C. arietinum* cv. ICC 4958 X *C. reticulatum* cv. PI 489777. Total span of the advanced draft sequence was 510,879,539 bases, of which sixty-five percent (333,999,488 bases) of total assembly was assigned to eight linkage groups with ~340 kb/cM recombination rate resulting in 2.7-fold improvement in length from the previous assembly. Length of the smallest scaffold of the fewest set representing 50% of total assembled bases (N50) was improved from 77.31 kb to 39.90 Mb and N50-index was decreased to 6 from 931 in the previous assembly (Table 1). Seventy-five percent of total assembled sequence was contributed by about 1000 fragments (Supplementary Fig. S1). Sizes of the pseudomolecules were increased considerably, with LG4 being the largest (54.99 Mb) (Table 1). We mapped back the sequence reads to the assembly to ensure the absence of any spurious assembly artifact (Supplementary Table S2).

Transcriptome coverage in the advanced draft assembly was calculated using two data sets; the 34,760 assembled transcripts of ICC 4958 and the 46,369 assembled transcripts (CaTAv2) generated from different tissues and development stages of more than seventeen chickpea genotypes^18^,^20^. A BLAT search of these sequences in the chickpea genome showed that 95.50% of ICC 4958 transcripts and 92.30% of CaTAv2 transcripts were covered with more than 90% identity and 80% coverage, whereas 95.1% of ICC 4958 transcripts and 90.18% of combined transcripts showed more than 90% identity and 90% coverage (Supplementary Table S3). An analysis of abundance of core eukaryotic orthologous groups (KOG) using CEGMA (Core eukaryotic gene mapping approach) pipeline showed the presence of 453 (98.90%) out of 458 KOGs in the advanced draft genome (Supplementary Table S4)^32^. Overall, these results indicated that about 94% of the presently available transcript sequences are covered in the present genome assembly.

### Gene annotation

Using repeat-masked genome assembly, we annotated protein-coding and noncoding genes. We annotated protein-coding genes based on *ab initio*, homology-based and EST mapping approaches followed by generating a non-redundant consensus set of genes by merging their prediction results. We predicted a total of 30,257 non-redundant consensus genes spanning 91.80 Mb with an average gene length of 3034 bases and 5.9 genes per 100 kb of the genome assembly. 417 genes were predicted to have multiple transcript isoforms with an average of 2.02 resulting in a prediction of 30,686 protein-coding sequences (CDS). Of all, 28,193 CDSs did not show any ambiguous base (N) in their sequences and the rest possessed <10% ‘N’. Total CDS length was 35.50 Mb with an average CDS length of 1173 bases and an average GC content of 41.60%. The average number of exons per gene was 5.1 with an average exon length 253 bases and the average intron length was predicted as 432 bases (Table 1). At least 25,986 (85.88%) predicted genes were supported by gene expression data (transcripts and/or RNAseq reads) (Supplementary Table S5). 449 KOGs out of 458 could be aligned on the annotated genes with significant homology (≤1e-10) (Supplementary Table S4). We predicted 27,571 gene models in the previous assembly and considering gene-space coverage of about 85%, we projected that chickpea genome is expected to encode about 32,000 genes^27^. In the present assembly, we predicted an average of 94% coverage of gene-space by mapping two transcriptome datasets, which further reflected the presence of about 32000 genes in chickpea.

Based on BLAST searches, 29,553 gene models (97.67%) showed significant similarity (<1e-5) with at least one of the public protein databases analyzed (Supplementary Table S6). 76% (23,003) were assigned a gene ontology (GO) term using Blast2GO pipeline. ATP-binding activity (16.4%), protein phosphorylation (4%) and nucleus (25.6%) were the most abundant GO categories under molecular function, biological process and cellular component categories, respectively (Supplementary Fig. S2).

Annotation of non-coding RNAs was performed following methods described previously^27^. A total of 753 tRNA loci spanning 56,564 bp with an average length of 75 bp/locus was identified. The number of rRNA loci decreased from 249 in the previous draft to 229 in this draft, while the total length was increased from 40.85 kb to 52.22 kb due to reduced gap within the scaffolds. A total of 414 snoRNA loci and 555 microRNA loci spanning 32,932 bp and 61,394 bp, respectively were identified (Supplementary Table S7).

### Genome features

**Figure 1:**
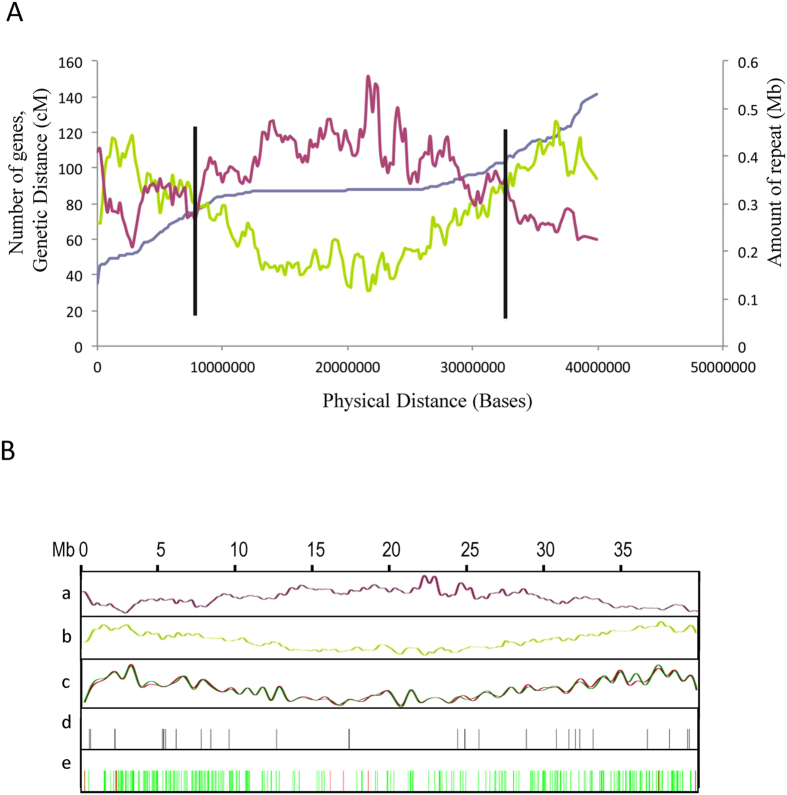
Multi-dimensional topography of chickpea linkage group 1. (**A**) Pericentromeric region of linkage group 1 was identified based on comparison of physical distance (X-axis) with gene density (green line, left Y-axis), repeat density (maroon line, right Y-axis) and average genetic distance (purple line, Y-axis). Vertical bars indicate positions of transition from euchromatic arms to pericentromeric arms. (**B**) Distribution of repeats (**a**), genes (**b**), RNAseq reads from vegetative tissues (young root and shoot, matured leaf) (green) and reproductive tissues (flower, young pod) (red) (**c**), microRNA loci (**d**), gene models encoding transcription factors (green) and R-genes (red) (**e**). All measurements are performed in 1 Mb sliding window increasing every 200 kb (other LGs are shown in supplementary figure S4).

We attempted to identify pericentromeric regions in the genome assembly by comparing genetic and physical distances, and mapping gene and repeat densities together with genetic and physical distances (Fig. 1A and Supplementary Fig. S3). Pericentrometric regions in this assembly were found primarily rich in repeat sequences and poor in gene content as observed in soybean (*Glycine max*) and common bean (*Phaseolus vulgaris* L.) genomes with average gene density of 5.57/100 kb in contrast to 8.63/100 kb in the euchromatic region^33^,^34^. Average recombination rate (1691 kb/cM) in this region was about 9-fold lower than that in the euchromatic regions (193 kb/cM) (Supplementary Table S8). Recombination rate in the pericentromeric region in this assembly was much higher than that observed for common bean (4350 kb/cM)^34^. This was most probably due to less anchoring of sequence scaffolds in the pericentromeric region in this assembly. Average gene density per 100 kb of the pseudomolecules was 7.07 while, that of the unanchored scaffolds was 3.73. This suggested that most of the unanchored scaffolds were gene-poor and probably belonged to pericentromeric regions. Distribution of repeats, genes and expression reads of protein-coding genes in vegetative and reproductive tissues along the linkage groups are shown in Fig. 1B and Supplementary Fig. S4.

**Figure 2:**
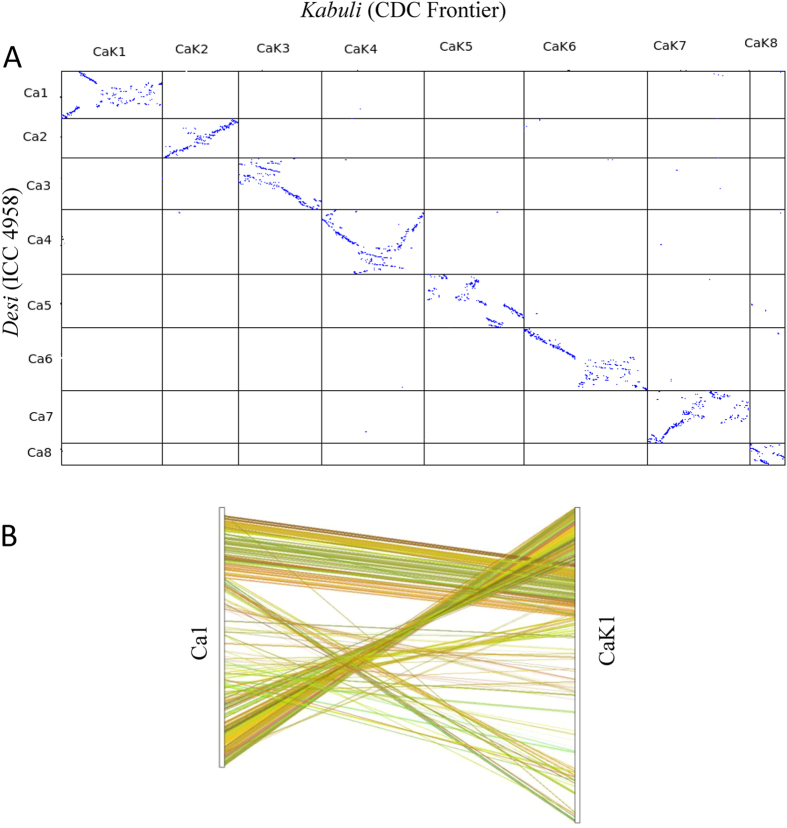
Comparison of *desi* and *kabuli* chickpea genome assemblies. (**A**) A dot-plot matrix comparing the *desi* (ICC 4958) and the *kabuli* (CDC Frontier) draft assemblies of the pseudomolecules corresponding to each linkage group. Pairwise comparison of all the pseudomolecules of *desi* (Ca1-Ca8) and *kabuli* (CaK1-CaK8) chickpea draft genome assemblies were performed using synteny blocks and anchor filtering algorithms of tool SyMap v4.0. (**B**) Pairwise collinearity analysis of orthologous genes present in linkage group 1 of *desi* (Ca1) and *kabuli* (CaK1) draft assemblies. Analysis was performed using default parameters of tool MCScanX. Comparison of other LGs is shown in supplementary figure S6.

We performed a pairwise comparison between the draft genome assemblies of *desi* chickpea genotype (ICC 4958) and the *kabuli* chickpea genotype (CDC Frontier) (Fig. 2A). Sequence blocks within the pseudomolecules in ICC 4958 assembly matched with those of the corresponding pseudomolecules in the CDC Frontier assembly. However, large segments within certain pseudomolecules in the ICC 4958 assembly were inverted in comparison to the CDC Frontier assembly, which could be primarily due to use of different marker maps. For example, most of the LG6 pseudomolecule in both the assemblies showed similar orientation, but LG2 of *desi* assembly was entirely inverted in comparison to the corresponding LG in the *kabuli* assembly. In case of LG4, half of the pseudomolecule of ICC 4958 showed forward orientation with respect to the CDC Frontier, whereas the other half showed inverted orientation. We performed pairwise collinearity analysis of orthologous genes in the two assemblies. Although, extensive local gene collinearity was observed in all the pseudomolecules, an incongruity in the positions of large gene blocks within the corresponding pseudomolecules of the two assemblies was evident and in concurrence with the observed pair-wise pseudomolecule alignment (Fig. 2B and Supplementary Fig. S5). A comparison of the length and ‘N’-content of the individual pseudomolecules of ICC 4948 and CDC Frontier assemblies is presented in the supplementary table S9. Sequence-based physical assembly is more reliable than the genetic marker-dependent assembly as positions of genetic markers change with maps with variable marker densities. Therefore, generation of larger sequence reads and chromosomal genomics approach are required to further improve the genome assemblies^35^.

### Multispecies comparison of homologous gene families

**Figure 3:**
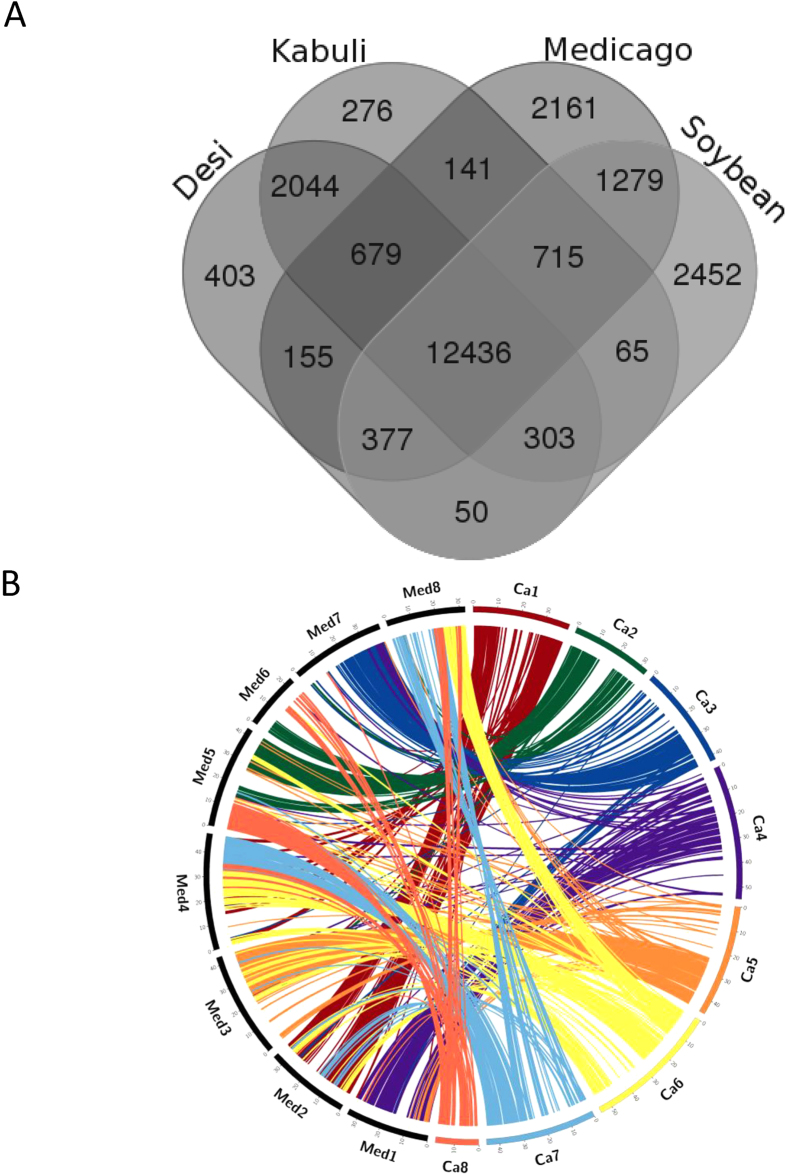
Comparative features of chickpea protein-coding genes. (**A**). Venn diagram showing distribution of gene families among *desi* (ICC 4958) and *kabuli* (CDC Frontier) type chickpea, *Medicago truncatula* and soybean. (**B**). Circos diagram presenting syntenic relationship between chickpea (Ca) and *Medicago truncatula* (Mt) pseudomolecules. Mt pseudomolecules are shown in black and labeled as Med1–5. Chickpea pseudomolecules are labeled in different colours and labeled as Ca1-8. Colinear blocks are coloured according to the colour of the corresponding chickpea pseudomolecule.

Predicted proteome in this assembly was compared with those from *Medicago truncatula* (*Medicago*), soybean and *kabuli* chickpea assembly. A total of 25,086 *desi* chickpea genes clustered with 107,423 genes from three other plants in 23,536 gene families of two or more members (Fig. 3A and Supplementary Fig. S6). Among these, 12,436 gene families containing 93,506 genes were conserved in all four plants, whereas 2044 families containing 5273 genes were restricted to both chickpea assemblies. A total of 403 families representing 1143 genes were unique to *desi* chickpea assembly, while 1028 genes belonging to 276 families were unique to the *kabuli* chickpea assembly. *Desi* chickpea shared 155 gene families representing 946 genes with *Medicago* only in comparison to 141 gene families representing 405 genes of *kabuli* type chickpea. Several previous analyses demonstrated extensive synteny and conservation of gene orders between chickpea and *Medicago* as they belong to galegoid clade of Papilinoidae subfamily of legumes^27^,^36^. Total of 25,919 *desi* chickpea genes showed considerable sequence homology (≤1e-5) with *Medicago* genes. Out of these, 9909 genes exist in 677 collinear blocks between *desi* chickpea and *Medicago* genome assemblies. Overall all the chickpea LGs showed extensive collinearity with different *Medicago* chromosomes. Large collinear gene blocks were observed between chickpea LG5 (CaLG5) and *Medicago* chromosome 3 (Med3) with 1196 orthologous genes in 71 collinear blocks, followed by 1082 genes in 59 blocks between CaLG1 and Med2, and 1046 genes in 53 blocks between CaLG3 and Med7. The largest collinear block of 115 genes was observed between CaLG1 and Med2 (Fig. 3B).

We compared the annotated CDS and peptide sequences of the *desi* and *kabuli* chickpea assemblies. 26,673 (94.35%) of 28,269 CDSs in the *kabuli* assembly showed high sequence identity (<1e-10) with those in the *desi* assembly. However, on detail analysis, 20,200 *kabuli* CDSs (71.45%) showed >90% coverage with >95% identity and 24,908 *kabuli* CDSs (88.11%) showed >50% coverage with >95% identity with the *desi* CDSs. 14,023 (~50%) *kabuli* CDSs showed no nucleotide variation with the *desi* CDSs. 27,486 (97.12%) predicted peptides in the *kabuli* assembly showed high sequence similarity (<1e-10) with the deduced *desi* proteome while, 19,029 (67.31%) of those exhibited >90% coverage with >90% identity. This apparent lack of extensive sequence identity seems to have arisen due to the use of different annotation pipelines and sequencing gaps and errors. For example, Ca_08927.1 (Ca_LG4: 11231442-11236902) in our assembly codes for a protein of 479 amino acid (aa) in length, but in *kabuli* assembly the corresponding gene Ca_07691 (Ca4: 589600-596516) encoded a protein of 523 aa. At a closer view, the alignment of these two gene sequences showed 100% sequence identity except a stretch of extra 111 ‘N’s within the *kabuli* gene model (Ca_07691) probably added to stitch two scaffolds. Likewise, Ca_08931.1 (Ca_LG4: 11287627-11291734) in desi assembly encoded a protein of 853 aa, while the corresponding gene Ca_07695 (Ca4: 647346-650769) in *kabuli* assembly encoded a protein of 811 aa. Both the gene sequences showed 100% sequence identity in the corresponding region. However, annotation of Ca_08931.1 included 126 bp more at the 5′-end, which was present in the *kabuli* genome assembly, but was not included in the annotation. We compared sequence identity between *desi* (ICC4958) and *kabuli* (ICCV2) chickpea transcriptomes^20^,^15^. 24,332 (70%) of 34,760 *desi* chickpea transcripts could be aligned with >90% coverage and >90% identity with *kabuli* transcripts showing a result similar to the comparison of annotated CDS.

### Gene families

We identified 2230 transcription factor (TF) proteins by following the pipeline used for plant transcription factor database version 3.0 using default parameters and classified those into 57 families (Supplementary Table S10)^37^. 2131 TF gene loci were assigned to LGs. Previously, we reported 1784 genes belonging to 84 transcription factor/regulator families^27^. However, according to a recent classification of plant transcription factors excluding transcription regulators, there are 60 DNA-binding domain specific TF families in plants to date^37^. According to parameters used in this pipeline, soybean genome encoded 5069 TF genes (3714 loci/7.99%) belonging to 57 families, while other sequenced legumes reported fewer TF genes (*Cajanus cajan* 1886, *M. truncatula* 1663, *Lotus japonicas* 1311) than chickpea (7.37% of all protein-coding genes)^37^. Recently sequenced mung bean (*Vigna radiata*) and adzuki bean (*Vigna angularis*) genomes also encode similar proportion of TFs (8.2% and 9.9%, respectively)^38^,^39^. TF bHLH represented the largest single family in chickpea with 193 genes, as in soybean with 480 genes. When related TF families were considered, consistent with other sequenced legumes, MYB (125 genes)/MYB-related (121 genes) and AP2 (35 genes)/ERF (159 genes) families represented more genes in chickpea. Among different TF families, the numbers of genes encoding SAP (Sterile Apetalla), STAT and S1Fa-like families of proteins were unusually higher in chickpea (24, 3 and 8, respectively), even more than those in soybean (2, 1, and 4, respectively) and other legumes we compared (Supplementary Table S10). Distribution of TF-genes on the linkage groups was shown in Fig. 1B and Supplementary Fig. S4. We mapped the previously reported tissue-specific RNAseq reads on the annotated TF mRNAs^20^. Normalized expression values were plotted in a heatmap. Tissue-preferential expression was considered when the expression of a gene in a tissue was at least three-fold higher than its expression in all other tissues. 518 TF-encoding genes showed tissue-preferential expression. Most of these TF genes preferentially expressed in flower (262) followed by root (142), young pod (91) and leaf (23), indicating their specialized functions in a reproductive organ (Supplementary Fig. S7). Similarly, mapping of previously reported abiotic stress-responsive transcriptome data showed that as many as 144 TF-genes were preferentially expressed in response to salt treatment in 10 day-old roots followed by 28 genes in dehydration-treated root according to the criteria described above (Supplementary Fig. S8)^40^.

We used stringent criteria for identification of resistance gene homologs (RGHs). Hidden Markov Model (HMM) profiles for different *R* gene families were generated using *R* gene sequences present in plant resistance genes database^41^. Initial screening with chickpea deduced proteome resulted in the identification of 1559 peptide models, including receptor-like-kinases. These peptide sequences were screened for the presence of specific R-protein domains using InterPro scan and NCBI conserved domain search to select 133 high-probability RGH and 623 receptor-like kinases^42^,^43^. One hundred eight of these were assigned to different LGs and were found to be distributed all over the LGs like those in *Medicago*^44^. (Supplementary Fig. S4). Previously, the *desi* and the *kabuli* chickpea draft genome assemblies reported the presence of 86 (excluding TTR-WRKY) and 187 RGHs, respectively^27^,^28^. The presence of *R*-genes in chickpea in comparison to other sequenced legumes and non-legumes is surprisingly low (Supplementary Table S11). A sequence comparison with PCR-derived NBS (nucleotide binding site) amplicon of chickpea genome also suggested a low number of RGH in chickpea genome^28^. However, the number of receptor-like kinases associated with disease tolerance in chickpea is comparable with the other species analyzed.

### Chickpea-specific and orphan genes

Seven hundred four predicted gene models did not show significant sequence similarity (<1e-5) with any of the nucleotide or protein databases. These genes were considered as orphan genes. Average lengths of these genes and CDSs are much smaller (592 and 314 bases, respectively) in comparison to the overall averages (3034 and 1173 bases, respectively). On average, these gene models showed the presence of only two exons and high GC content (43.32%) (Supplementary Table S12). Three hundred four of these genes showed expression evidence when mapped with available RNAseq reads. Twenty-nine of these orphan genes showed the presence of some conserved protein domains when searched in the NCBI conserved domain database. These domains are of various types, including PRM_SF, present in RNA processing proteins, Myc target 1- and MIP- and Fe-Alcohol dehydrogenase superfamilies (Supplementary Table S13).

A total of 3296 gene models showed significant sequence similarity with only chickpea genes present in various databases and, therefore, considered as chickpea-specific genes. This is about 10% of all the predicted gene models and similar to the predictions based on the previous draft assembly and transcriptome data^20^,^27^. Two thousand two hundred fifty-eight of these genes had expression evidence and orthologs of 2918 of these genes are present in the annotated *kabuli* gene models. Average lengths of these genes and CDSs are smaller than the overall averages (Supplementary Table S12), however, longer than those of the orphan genes. 223 chickpea- specific genes showed tissue-specific expression according to a previously reported tissue-specific RNAseq data^20^. Most of these genes displayed exclusive expression in flower (110) followed by young pod (77), root (23) and leaf (13) (Supplementary Fig. S9). Preferential expression of chickpea-specific genes in flower and young pod suggests that these genes contribute some characters to these organs, which are different from other species. One thousand eight hundred fifty-one chickpea-specific peptide models showed the presence of conserved domains (Supplementary Table S14). The most abundant conserved domain superfamilies were zf-CCHC (NCBI CDD accession: cl22700), RNaseH-like (cl14782), pepsin-retropepsin-like (cl11403) and gag (cl04237). These domains are related to or derived from retrotransposons or retroviral gag proteins and, therefore, appear to be originated by the movement of transposable elements, which is common in eukaryotes^45^. The fifth most abundant conserved domain present in chickpea-specific genes is ABC-transporter superfamily (cl21455) (Supplementary Fig S10). ABC-transporter proteins are membrane-bound proteins responsible for the transport of various metabolic products across the membranes and may provide specialized functions or unique biological properties^46^.

### Functional diversity of paralogous genes and genome evolution

**Figure 4:**
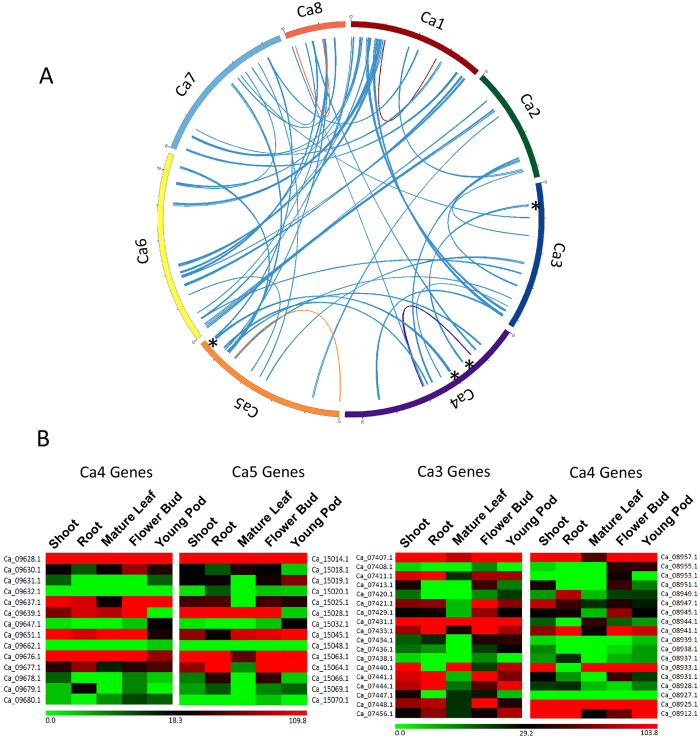
Whole genome duplication and functional diversification analyses. (**A**). Duplicated blocks in *desi* chickpea genome. All eight linkage groups are shown in different colours. The inter-linkage group blocks are shown in blue connecting lines and the intra-linkage group blocks are marked by connecting lines of same colour of the linkage groups. (**B**). Heatmap showing expression of genes present in the paralogous blocks (marked by * in fig. 4A) in various tissue samples. Gene IDs and tissues are mentioned at the top and sides, respectively. Corresponding linkage group numbers are mentioned at the top. The colour scale represents the number of unique reads mapped per million reads (RPM) values.

Evidences provided in several reports suggested a common whole genome duplication (WGD) event in most of the legumes approximately 58-60 million years ago preceded by paleopolyploidization shared with other plants^27^,^47^. WGD followed by gene loss resulted in the existence of paralogous gene blocks. 914 pairs of genes in 110 paralogous blocks of five and more genes were identified in the present draft assembly. Four of these blocks were intrachromosomal (LGs 1, 4, 5 and 8). LG1 shared a maximum of 30 paralogous blocks with four linkage groups (LG1, LG4, LG5 and LG8), including 16 paralogous blocks containing 152 gene pairs with LG6 (Fig. 4A). Functional redundancy could be one of the reasons for gene loss after WGD. Functional diversification or acquiring a new essential function would retain a gene after duplication. We analyzed tissue-specific expression profile of genes present in two large paralogous gene blocks using RNAseq data reported before (Fig. 4B)^20^. Overall, there was a general trend of differential tissue-specific expression of the gene-pairs present in those paralogous gene blocks except Ca_09628.1 and Ca_15014.1; Ca_09647.1 and Ca_15032.1 pairs. For example, in a paralogous block between Ca3 and Ca4, both Ca_07448.1 and Ca_08925.1 encode 26S proteasome subunit 4 proteins. While Ca_08925.1 displayed a high expression in all the tested tissues, Ca_07448.1 expression was significantly low in mature leaf and young pod. A similar differential expression was observed for Ca_07431.1 and Ca_08944.1 encoding alcohol dehydrogenase class 3 proteins. In paralogous blocks between Ca4 and Ca5, Ca_9677.1 and Ca_015064.1, both encoding serine-rich adhesion proteins, and Ca_09637.1 and Ca_15025.1 encoding transcription repressor LEUNIG-like proteins exhibited contrasting expression patterns depicting functional diversification of paralogous genes.

*Kabuli* type chickpea or the race macrosperma is considered as a derivative of *desi* type or microsperma obtained through selection for increased seed size using a narrow genetic basis in recent times after domestication of the crop^48^. Distribution of rate of synonymous substitution (Ks) within the orthologous gene pairs of *desi* and *kabuli* chickpeas showed a peak at a Ks value of 0.0001 suggesting a divergence of about 8000 years between two chickpea types supporting the hypothesis of recent selection of *kabuli* type (Supplementary Fig. S11). In addition, a diffused secondary peak at the range of Ks 0.002 to 0.003 was also visible indicating an initial divergence of the founder cultivars of *kabuli* type from the *desi* cultivars about 0.16 to 0.25 million years ago.

### SNP-based intra-specific polymorphism

**Figure 5:**
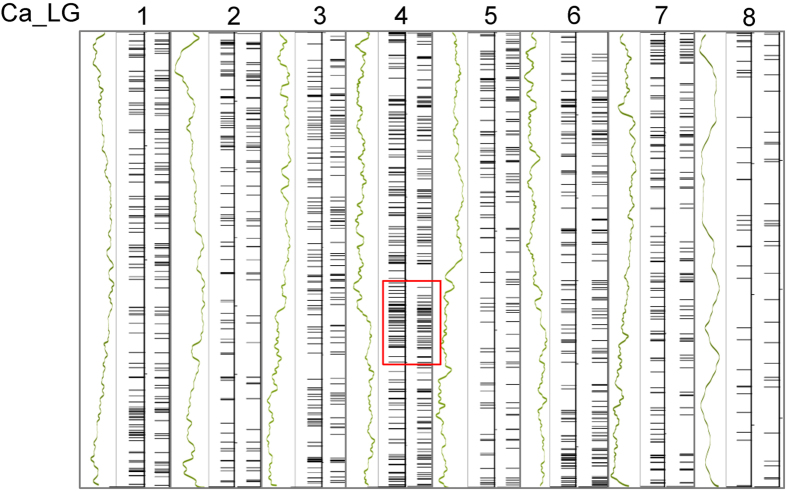
Distribution of SNPs within the *desi* group (39 desi genotypes) and the *kabuli* group (53 *kabuli* genotypes) of chickpea along the pseudomolecules (Ca_LG1-8). Distribution of genes (green) is followed by the SNPs within the *desi* group and the *kabuli* group of chickpea geneotypes, respectively. Red rectangle denotes genomic region with maximum variation in chickpea genotypes.

The NGS-based high-throughput GBS assay is a well-established strategy for simultaneous SNP discovery and genotyping of SNPs at a genome-wide scale that have been employed recently for multi-dimensional large-scale genotyping applications in crop plants, including chickpea^10^,^11^,^49^. However, the success of this approach for efficient mining and genotyping of genome-wide SNPs relies upon availability of a high-quality well-assembled reference genome sequence, on which the sequence reads generated from diverse crop genotypes through GBS assay can be effectively mapped. Henceforth, to assess the efficiency of our presently assembled improved version of *desi* genome sequence as a reference for SNPs discovery in chickpea, we uniquely mapped the de-multiplexed high-quality sequence reads generated individually from 39 *desi* and 53 *kabuli* genotypes onto the present genome sequence^11^. This reference genome-based GBS analysis identified 2150 (mean SNP map density 141.9 kb) and 2199 (138.7 kb) SNPs among the genotypes belonging to *desi* and *kabuli* cultivar groups, respectively (Supplementary Tables 15 and 16). The slight variation in the SNP density and thus diversity between *desi* and *kabuli* is possibly due to a more recent secondary bottleneck in *kabuli* chickpea^28^. A maximum variability and density of SNPs identified between the *desi* and *kabuli* genotypes specifically in 30–40 Mb genomic region of chromosome 4 were observed (Fig. 5), which is agreed well with the previous documentation^28^. This highly variable genomic location could be a target region of interest for further understanding the domestication-led evolutionary patterns in *desi* and *kabuli* chickpea. Numerous informative genome-wide well-distributed genetic markers with high intra-specific polymorphic potential are required for marker-assisted genetic improvement of the chickpea genome with a narrow genetic base. In this context, two groups each of more than two thousands SNPs differentiating the *desi* genotypes and the *kabuli* genotypes identified in our study will be of significance in the construction of high-density integrated genetic and physical maps. This will be also useful for genetic/association mapping for efficient delineation of genes/QTLs governing important agronomic traits leading to genetic enhancement studies in chickpea. Moreover, 2150 and 2199 SNPs showing polymorphism within *desi* and *kabuli* groups was derived from 715 and 757 annotated protein-coding genes, respectively. These gene-based SNPs could be utilized for establishing rapid marker trait linkages and targeted mapping of genes harboring QTLs associated with important traits in chickpea. Collectively, the aforementioned outcomes reflect the broader applicability of the present *desi* genome assembly as a reference for multiple NGS-based low-scale resequencing and GBS-derived SNP mining and genotyping-related analyses in diverse *desi* and *kabuli* chickpea genotypes.

## Conclusion

We have attempted to improve and advance the previously reported genome assembly of a *desi* type chickpea cultivar (ICC 4958) using an improved high throughput genetic marker map. Pseudomolecules corresponding to eight linkage groups were increased by 2.7 fold in size. Filling of sequence gaps amounting 27 Mb with the newly generated short read data reduced N-content of the previous assembly by almost 50% and resulted in annotation of 30,257 protein-coding genes, 2686 more than the previous assembly and 1988 more than the CDC Frontier assembly. These genes accounted for about 95% of the chickpea gene space. The gene density analysis in the predicted pericentromeric regions suggested a low anchoring of repeat-rich sequences due to unavailability of genetic markers in these regions. This can only be improved by producing longer sequence reads and increasing scaffold sizes. As expected, the annotated protein-coding genes in both the chickpea genome assemblies displayed an overall sequence similarity. However, the lack of extensive sequence identity between ~30% genes may have resulted due to sequence errors or gaps and use of different annotation pipelines in the two assemblies. About 8% of all the CDSs in this assembly encoded for transcription factors. This is similar to the estimates in recently sequenced legume genomes, however, higher than those predicted in the *Medicago* and *Lotus japonicus*. Species-specific genes are originated due to gene duplication, recombination and movement of transposable elements, and encode proteins with specialized functions for the new species. The most abundant conserved domains observed in the chickpea-specific genes had the signatures of transposable elements indicating that the genesis of at least some of these genes was due to movement of transposable elements. Divergence of *desi* and *kabuli* chickpea types about 8000 years ago as assessed through the rate of synonymous substitution is consistent with the proposition that the *kabuli* type was derived from the *desi* types after domestication on the basis of traits. This reference *desi* genome assembly could serve as an efficient genomic resource for mining SNPs at a genome-wide scale in diverse *desi* and *kabuli* genotypes and thus would expedite genomics-assisted breeding applications and genetic enhancement targeting multiple qualitative and quantitative traits in chickpea. Finally, a pair wise comparison between the pseudomolecules of the *desi* and *kabuli* chickpea assemblies displayed extensive local collinearity in spite of some large structural variations. These variations might be due to the use of different genetic maps in the two assemblies, which necessitates the creation of a reference chickpea genetic map. The apparent disagreement between the two chickpea genome assemblies would limit their use in the genetic improvement of the crop. Therefore, a further improvement with the help of larger sequence reads and physically mapped BAC library is necessary to produce a reference genome assembly of chickpea^50^.

## Methods

### Sequencing and assembly

DNA samples from the chickpea cultivar ICC 4958 were used to prepare genomic DNA libraries as described previously^27^. The sequence reads reported in the previous draft assembly and the new MP reads (Supplementary Table S1) from two libraries of 20 kb insert size generated by 454/Roche GS FLX Titanium platform (http://www.454.com) were assembled following the method described previously. 100 base long PE reads were generated using a whole genome shotgun library of insert size 320–620 bases through Illumna HiSeq1000 platform (Illumina Inc., San Diego, CA, USA). High-quality sequence reads were quality filtered using NGS toolkit and further used to fill the sequence gaps using SOAP *de novo* gap closure tool version 1.1 with default parameters^51^. The sequence scaffolds were screened for mitochondrial, chloroplast, cloning vectors and bacterial DNA contamination following methods described before and additionally by NCBI^23^. Finally, the scaffolds and contigs were anchored to corresponding linkage groups using marker sequences of a genetic map constructed with a biparental mapping population *C. arietinum* cv. ICC 4958 × *C. reticulatum* cv. PI 489777 (http://nipgr.res.in/CGAP2/home.php). The assembly was submitted to NCBI under BioProject ID:PRJNA78951 with a submission ID:SUB713178. The newly generated reads were submitted to NCBI sequence read archive (SRA) database (SRR1632264, SRR1647666, −67, −69 and −70).

### Gene annotation

Repeat sequences were identified by software packages REPEATMODELLER, PILER, REPEATSCOUT and LTR_FINDER and masked by REPEATMASKER (http://repeatmasker.org) and REPEATPROTEIN MASK^52^,^53^,^54^,^55^. Repeat-masked genome assembly was used for annotating protein-coding genes by three approaches, *ab initio*, homology-based and EST-based. Augustus and GENESCAN tools with parameters trained on *Arabidopsis* were used for *ab initio* prediction^56^,^57^. We used EXONERATE (http://www.ebi.ac.uk/~guy/exonerate/) for homology-based prediction using protein sequences predicted in *kabuli* and *desi* chickpea draft assemblies^58^. For EST-based approach, we aligned chickpea transcriptome reads for spliced alignment using PASA^20^,^58^. Outputs from these three approaches were integrated by EVIDENCEMODELLER (EVM) to generate consensus gene set^59^. Finally, the EVM output was run through PASA for identification of spliced variants and prediction of untranslated regions and identified 30,649 gene models and 31,078 transcripts. We filtered out 392 gene models on the basis of small coding sequences (<150 bases) and high N-content ( ≥10%) finally to predict at 30,257 gene models and 30,686 CDSs. Genes encoding Ribosomal RNAs, transfer RNAs, micro RNAs and small nuclear RNAs were annotated following the previously described methods^27^.

### Functional annotation

Functional annotation was done by sequence alignment with TAIR10 protein database using BLASTP with cut off E value <1e-5. Proteins, which did not show significant similarity with TAIR database, were used to align with *M. truncatula* deduced proteome for functional annotation. Gene ontology terms were assigned using Blast2GO pipeline^60^. Methods for identifying genes encoding transcription factor proteins and *R*-genes are described in the Result section. Tissue-specific gene expression in four tissues was performed using 454 RNAseq data described previously^20^. Reads were quality-filtered using NGS toolkit. Quality filtered reads were mapped on annotated mRNA sequences using GSMapper (Roche Applied Sciences, http://www.roche-applied-science.com) with default parameters. Uniquely mapped reads per million total reads (RPM) were used to generate expression profiles. To generate the tissue-specific expression profile, genes with 0 RPM value in all, but one tissue were considered. For tissue-preferential expression of transcription factor (TF)-encoding genes, RPM values of a gene in all four tissues were converted to fold expression values by normalizing with its highest RPM value, thus converting the highest RPM value of a gene to 1. Fold expression values of TF-encoding genes in four different tissues were plotted as heatmap. Tissue-preferential expression was considered if the expression of a gene in a particular tissue was at least three-fold higher than its expression in all other tissues.

### Pair-wise sequence comparison and synteny

Pair wise sequence comparison of pseudomolecules was performed using algorithms in SyMap v4.0^61^. A whole genome dot plot with eight linkage groups of *desi* and *kabuli* chickpea was generated by selecting hits with >90% identity. Inter/intra-species gene synteny and collinearity were detected by MCScanX using default parameters considering pBLAST ≤1e-5. Links of collinear blocks between sets of LGs/chromosomes are shown by dual-synteny plot or circle plot^62^. Peptide sequences of orthologous and paralogous gene-pairs were aligned using ClustalW^63^. The output alignment file and the corresponding CDS sequences were used to determine synonymous substitution rates (Ks) using PAL2NAL and CODEML in PAML 4.5^64^,^65^. Species divergence was calculated using formula T = Ks/2r. The value of r was taken as 6.1 × 10^−9^ per year^66^.

### SNP-mining

GBS reads were quality-filtered using NGS tool kit^49^. Reads from 39 desi and 53 kabuli genotypes were grouped separately and mapped on chickpea pseudomolecules using BWA^11^,^67^. The sam files were converted to bam files by using samtools and then to .vcf format to identify SNPs. Only unique non-reference allele supported by at least three reads was considered.

## 

## Additional Information

**Accession codes** Genome assembly, annotation data and all the supplementary figures, tables, data are available for viewing and downloading at http://nipgr.res.in/CGAP2/home.php We will open it for public for view and download. User Id and password will not be required. Genome assembly and annotation are available at the National Centre for Biotechnology Information (NCBI) as Bioproject ID PRJNA78951. Additional sequence data generated for advanced draft assembly is submitted to NCBI (SRA) database (SRR1632264, SRR1647666, –67, –69 and –70).

**How to cite this article**: Parween, S. *et al.* An advanced draft genome assembly of a *desi* type chickpea (*Cicer arietinum* L.). *Sci. Rep.* **5**, 12806; doi: 10.1038/srep12806 (2015).

## Supplementary Material

Supplementary Information

## Figures and Tables

**Table 1:** Salient features of the advance draft of *desi* chickpea (ICC4958) genome assembly.

Total size (base)	510,879,539
Number of scaffolds	38,513
Minimum scaffold length(bp)	1000
Maximum scaffold length(bp)	54,992,815
Average scaffold length(bp)	13,265
N50 length(bp)	39,901,017
N50 index	6
GC content (%)	27.95
Length of pseudomolecules (bp)	
Ca_LG_1	39,901,017
Ca_LG_2	33,233,457
Ca_LG_3	42,267,542
Ca_LG_4	54,992,815
Ca_LG_5	45,819,701
Ca_LG_6	54,841,389
Ca_LG_7	45,279,478
Ca_LG_8	17,664,089
Protein-coding gene annotation	
Number of genes	30,257
Total gene length	91,806,333
Total CDS length	35,503,303
Number of predicted mRNA	30,686
Average gene length (bp)	3034
Average coding sequence (CDS) length (bp)	1173
Average exon length (bp)	253
Average intron length (bp)	432
Average number of exons per gene	5.1
GC content in CDS	41.60%
