# Supplementary material for: An advanced draft genome assembly of a desi type chickpea (Cicer arietinum L.)
Source: Sci Rep. 2015 Aug 11;5:12806. doi: 10.1038/srep12806 (PMC4531285; doi:10.1038/srep12806)
Supplement: Supplementary Information [file srep12806-s1.pdf]

An advanced draft genome assembly of a *desi* type chickpea (*Cicer arietinum* L.)

Sabiha Parween[1], Kashif Nawaz[1], Riti Roy[1], Anil K. Pole, B. Venkata Suresh, Gopal Misra, Mukesh Jain, Gitanjali Yadav, Swarup K. Parida, Akhilesh K. Tyagi, Sabhyata Bhatia, Debasis Chattopadhyay*

National Institute of Plant Genome Research (NIPGR), Aruna Asaf Ali Marg, New Delhi 110067, India

*Corresponding author

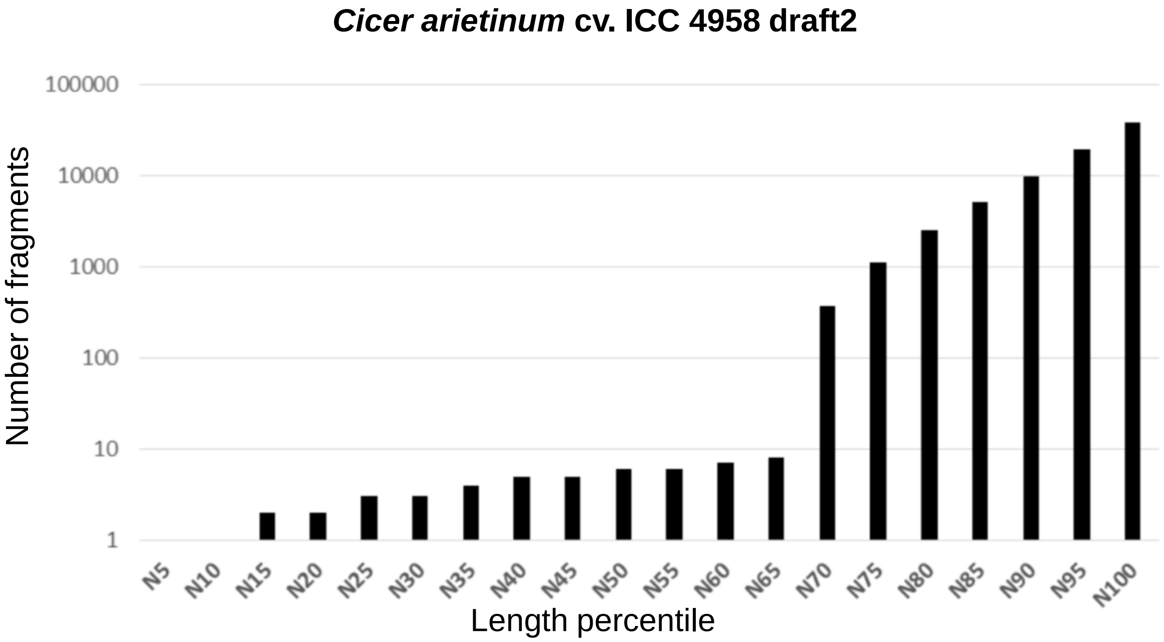

Supplementary Figure S1: Fragment distribution of *desi* chickpea var. ICC 4958 genome assembly. Number of fragments covering different percentile of the assembly plotted against different length percentile.

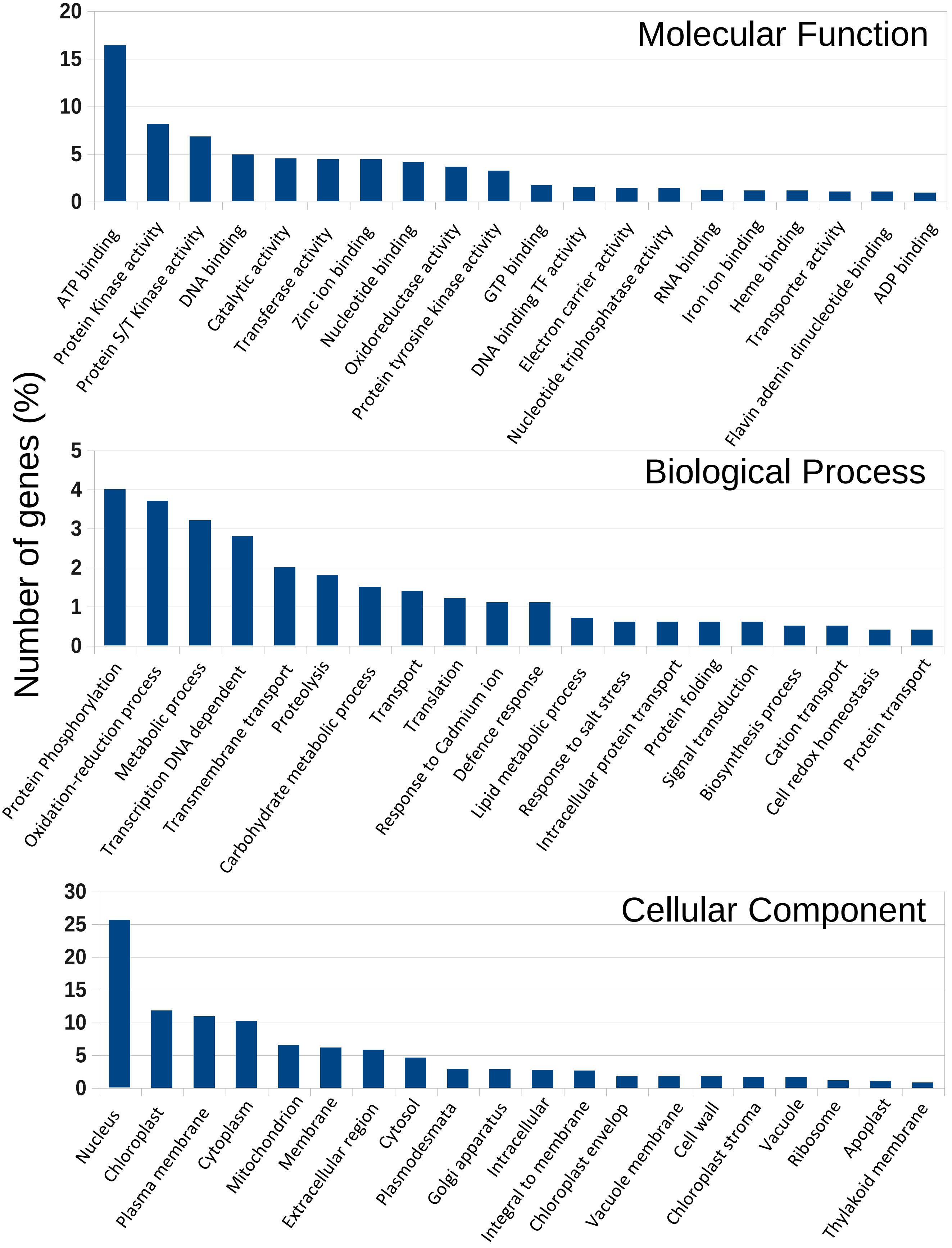

Supplementary Figure S2. Top 20 GO terms represented in chickpea geneset. GO terms were assigned using Blast2Go pipeline.

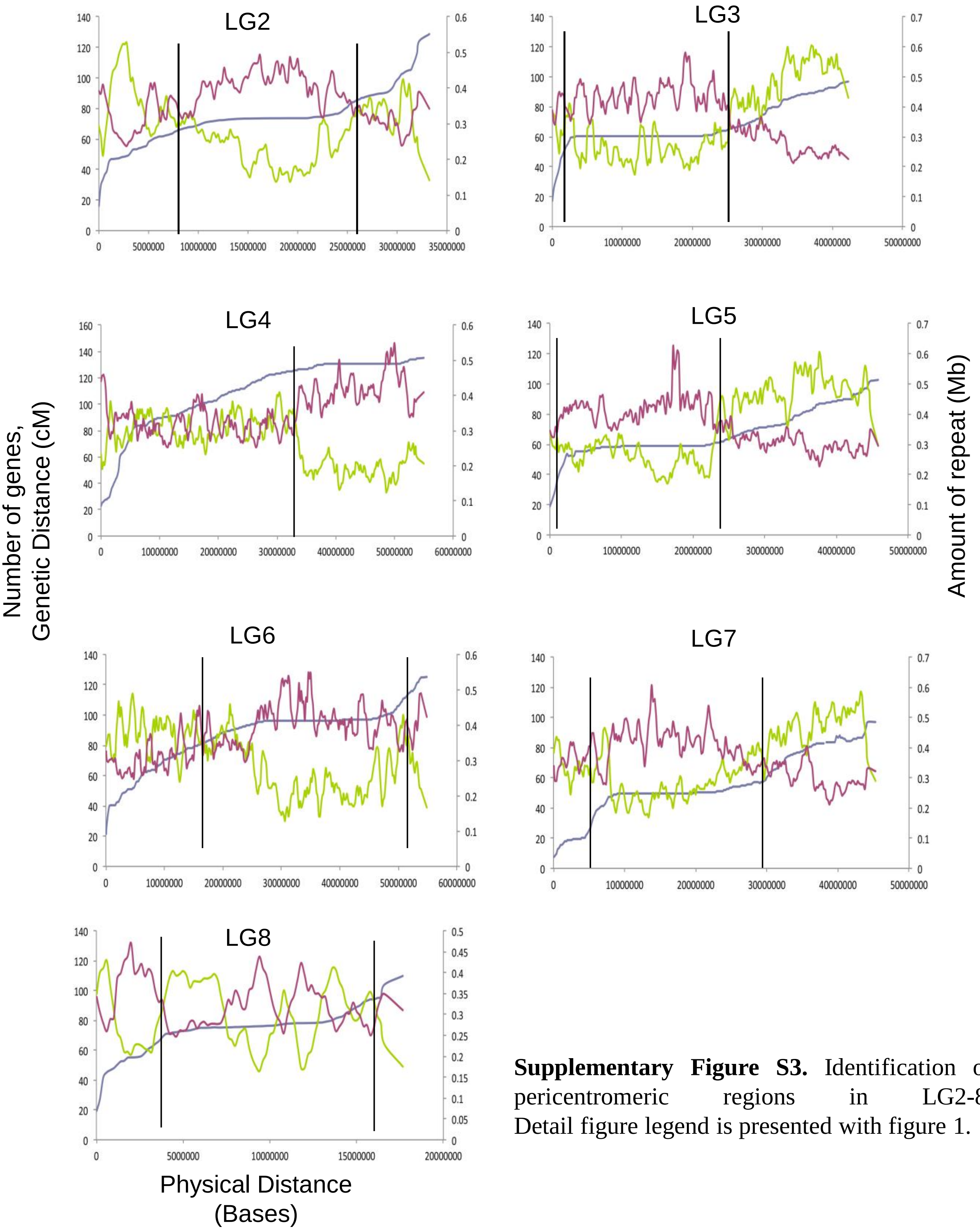

Supplementary Figure S3. Identification of pericentromeric regions in LG2-8. Detail figure legend is presented with figure 1.

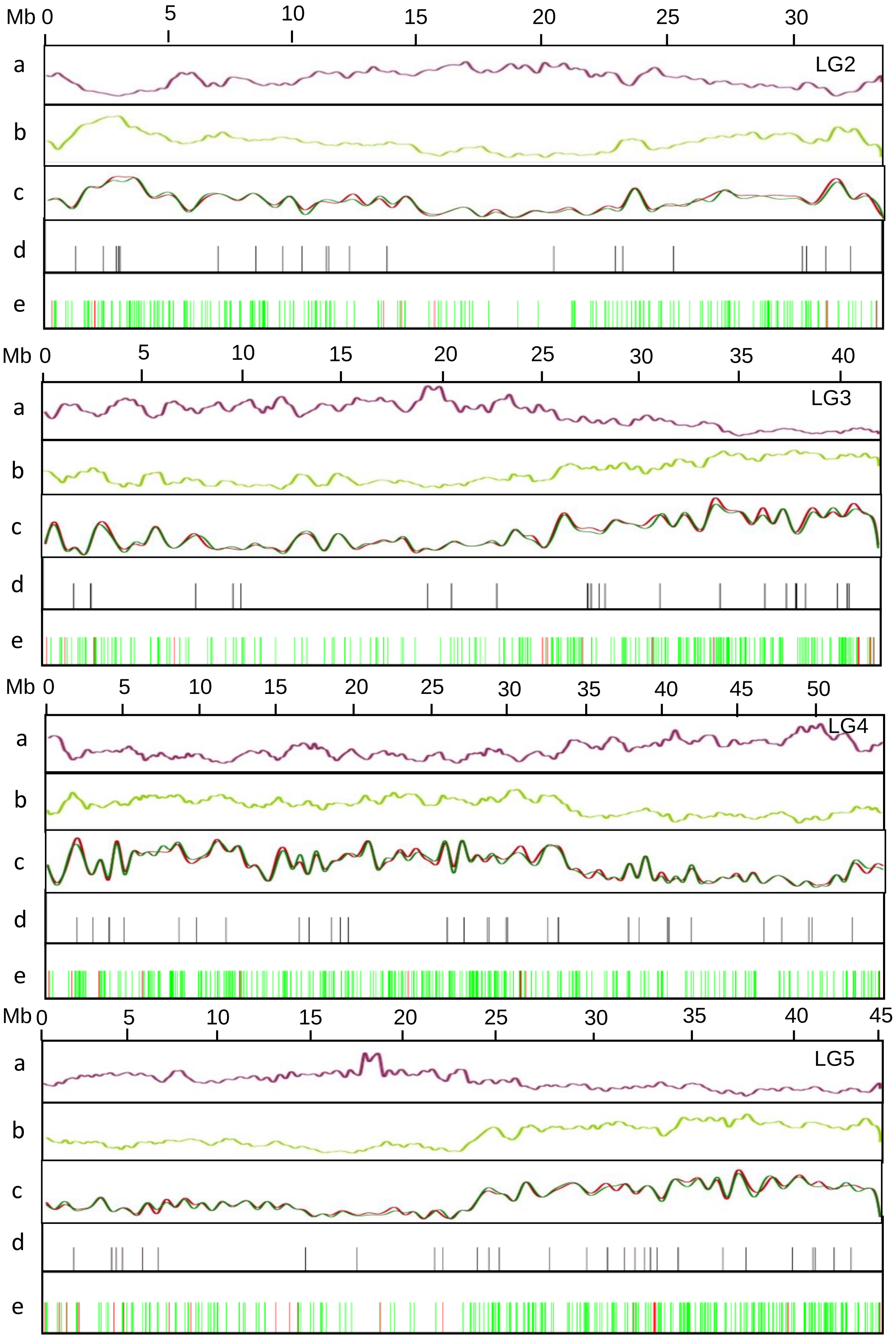

Mb 0 5 10 15 20 25 30
LG2
a
b
c
d
e
Mb 0 5 10 15 20 25 30 35 40
LG3
a
b
c
d
e
Mb 0 5 10 15 20 25 30 35 40 45 50
LG4
a
b
c
d
e
Mb 0 5 10 15 20 25 30 35 40 45
LG5
a
b
c
d
e

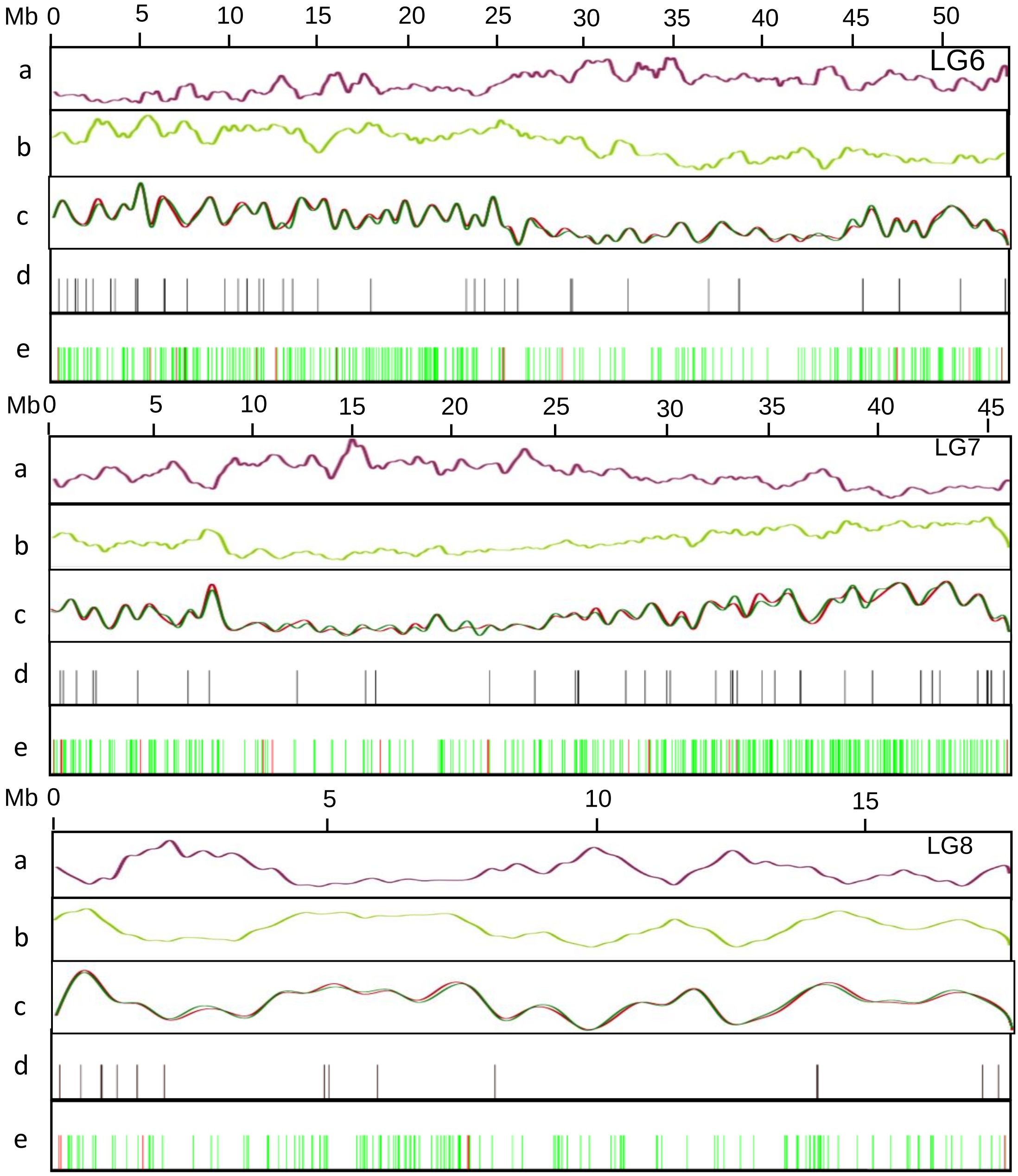

Supplementary Figure S4. Multidimentional topography of chickpea LG2-8. Distribution of repeats (a), genes (b), RNAseq reads from vegetative tissues (root, shoot, matured leaf) (green) and reproductive tissues (flower, young pod) (red) (c), microRNAs (d), gene models encoding transcription factors (green) and *R*-genes (red) (e). Detail figure legend is presented with figure 1B.

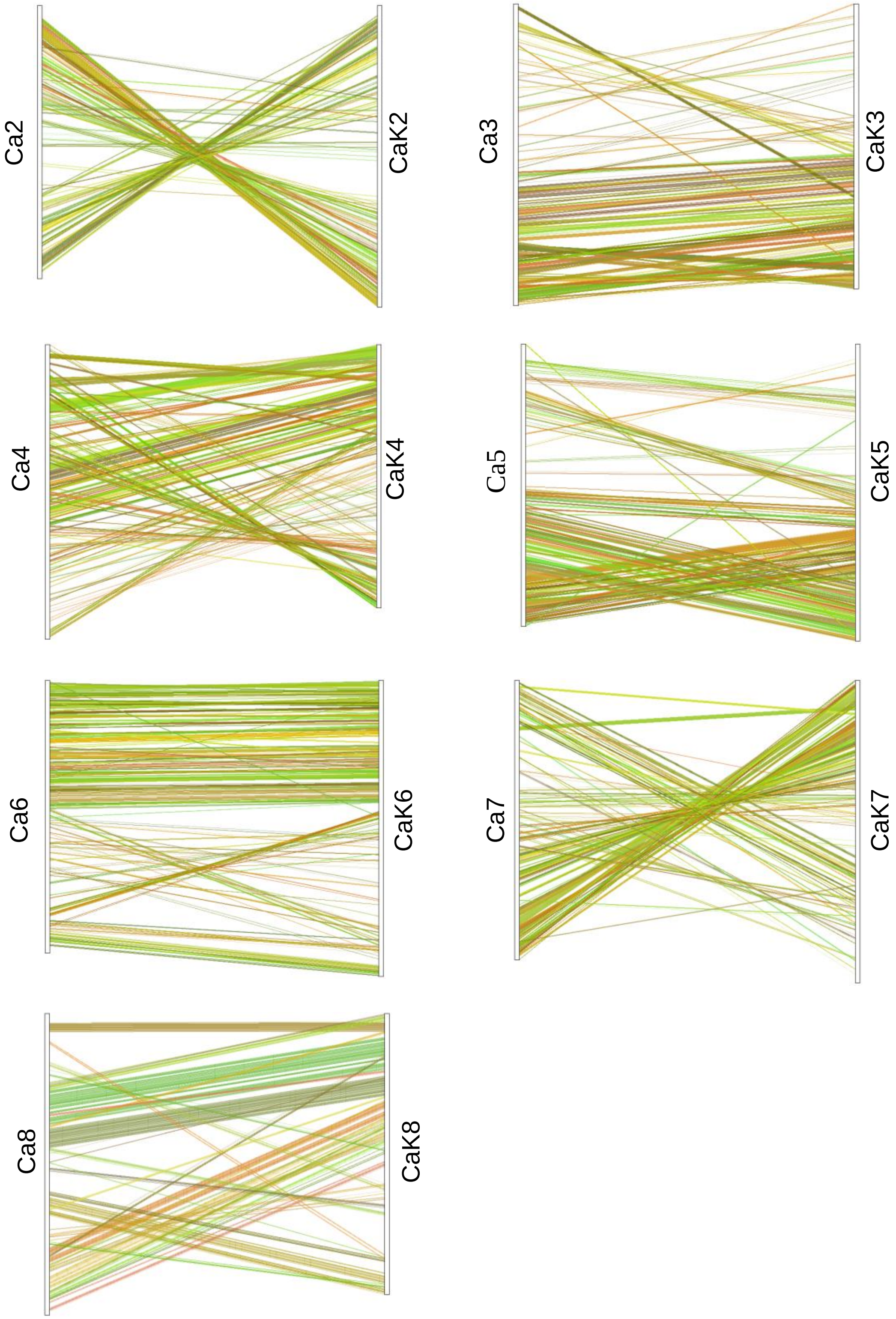

Supplementary Figure S5. Pairwise colinearity analysis of orthologous genes present in pseudomolecules of LG2-8 of *desi* (Ca) and *kabuli* (CaK) draft assemblies. Figure for LG1 is presented in figure 2B.

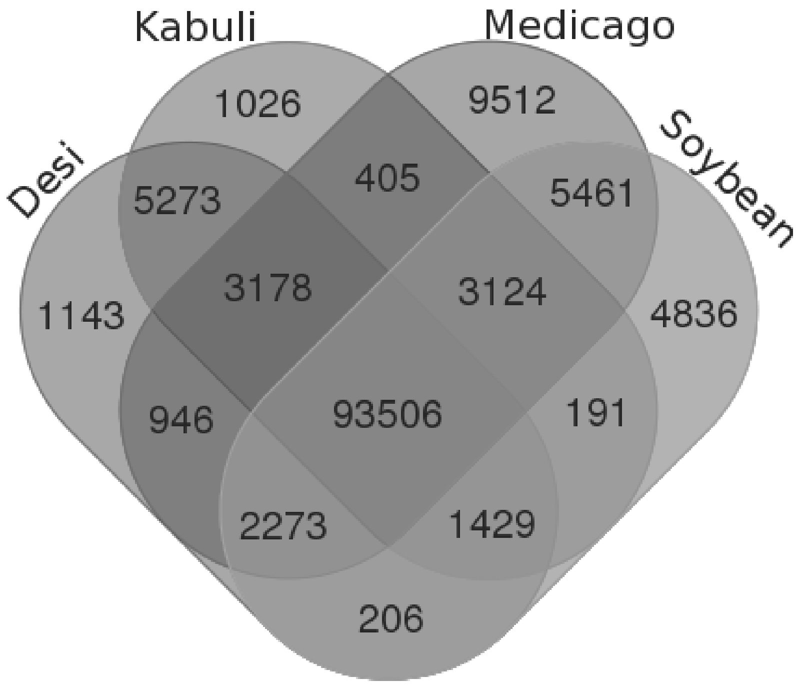

Supplementary Figure S6. Comparative features of chickpea protein-coding genes. Venn diagram showing distribution of genes among *desi* (ICC 4958) and *kabuli* (CDC Frontier) type chickpea, *Medicago truncatula* and soybean.

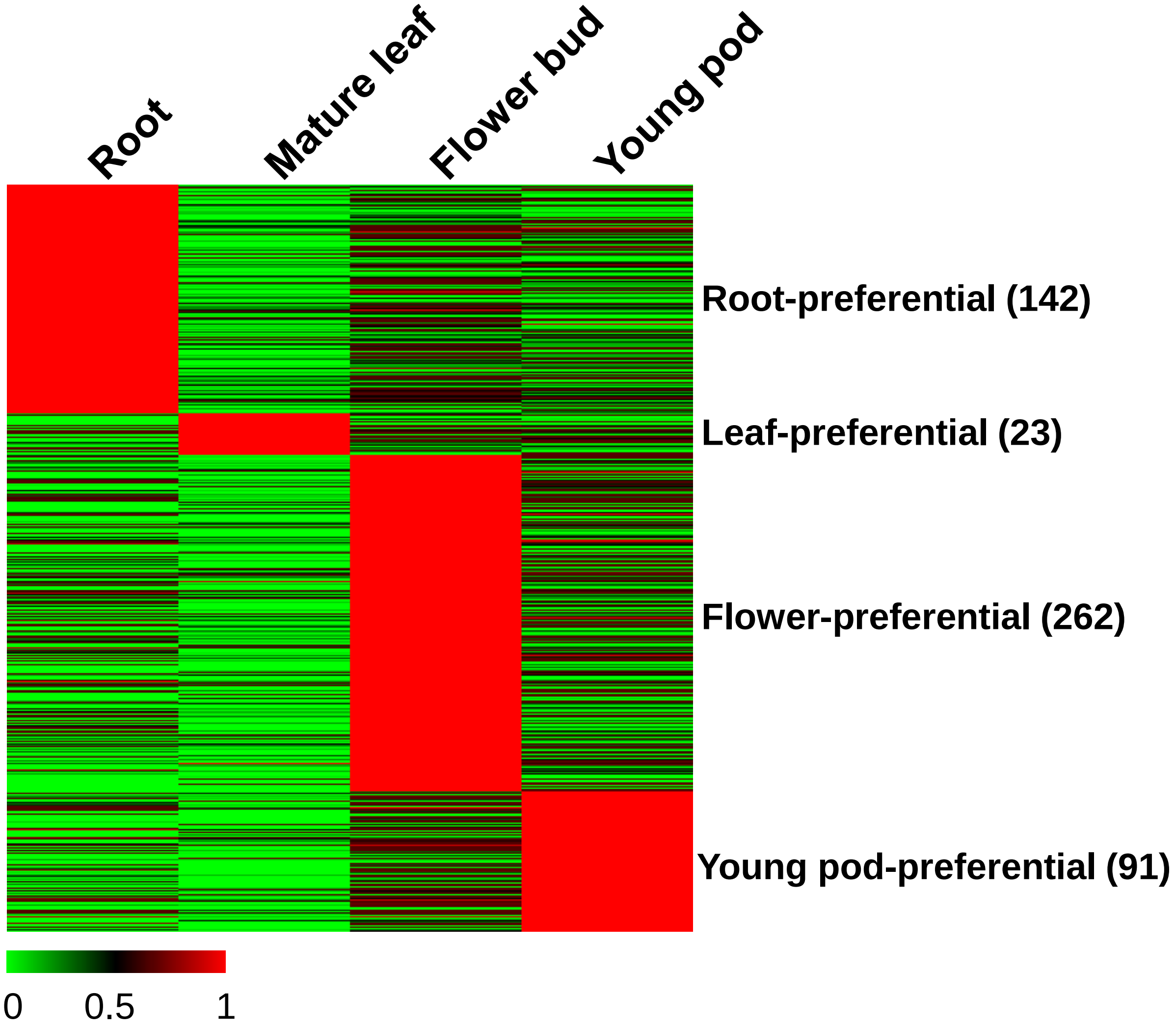

Supplementary Figure S7. Heatmap showing tissue-preferential expression of transcription factor-encoding genes. The colour scale at the bottom refers to normalized RPM (reads per million) values of genes between 0 and 1. Tissue-preferential expression was considered if the expression of a gene in a particular tissue was at least three fold higher than its expression in all other tissues. Number of genes showing tissue-preferential expression was shown in the right side. Tissues were mentioned at the top.

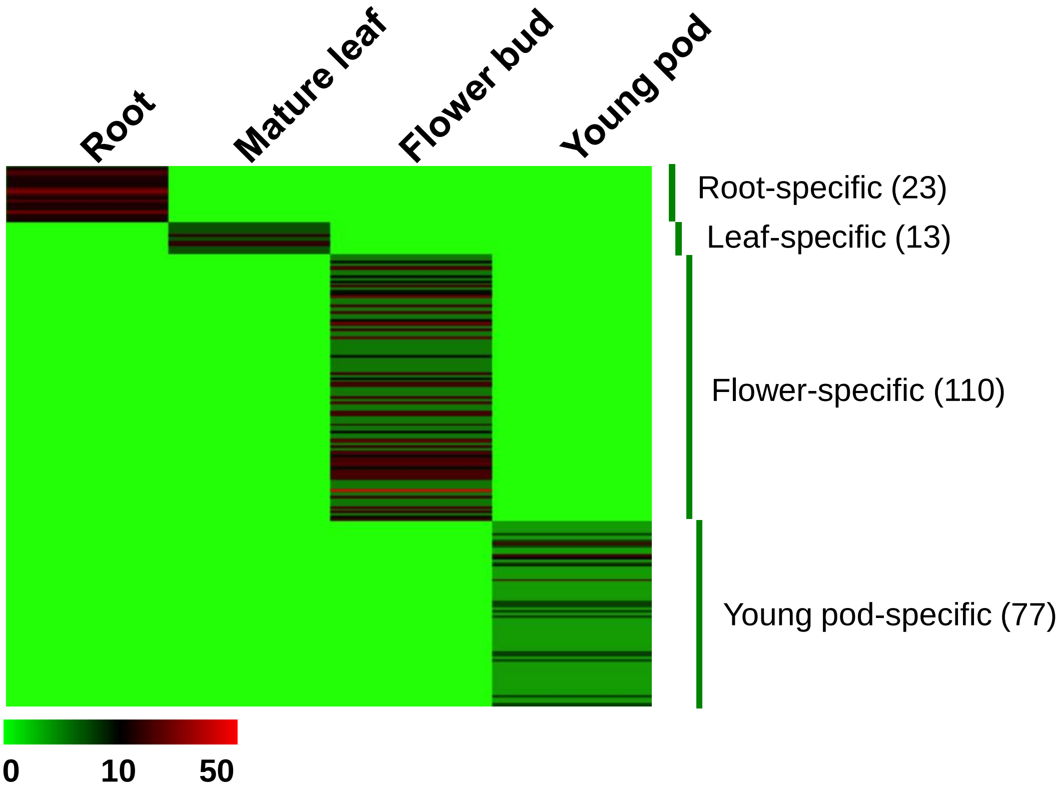

Supplementary Figure S8. Heatmap showing tissue-specific expression of chickpea-specific genes. The number of genes showing tissue-specific expression are mentioned on the right side. Tissues are mentioned at the top. The colour scale represents RPM (reads per million) values. Transcripts with >50 RPM values were not distinguished from those with 50 RPM by colour scale.

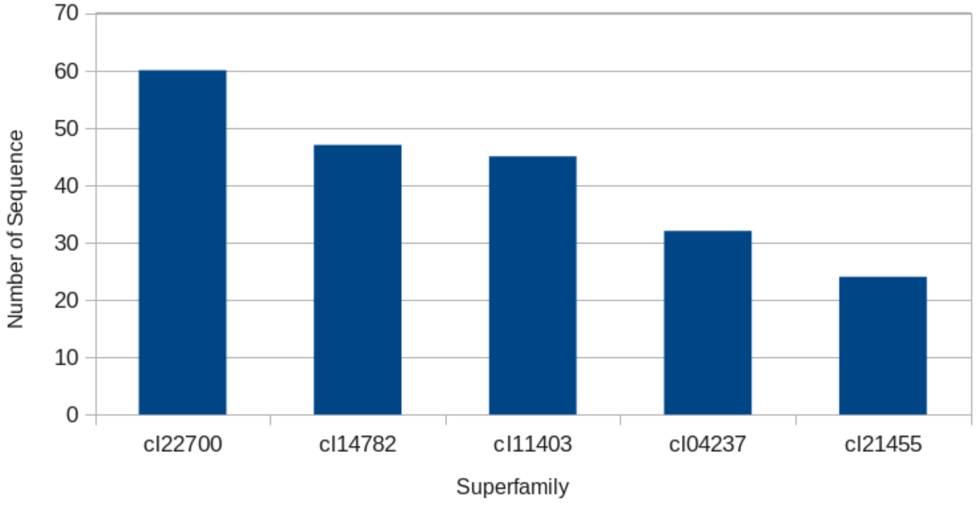

Supplementary Figure S9. Top 5 conserved domain superfamilies present in chickpea-specific geneset. NCBI CDD (conserved domain database) accession terms were assigned using domain search.

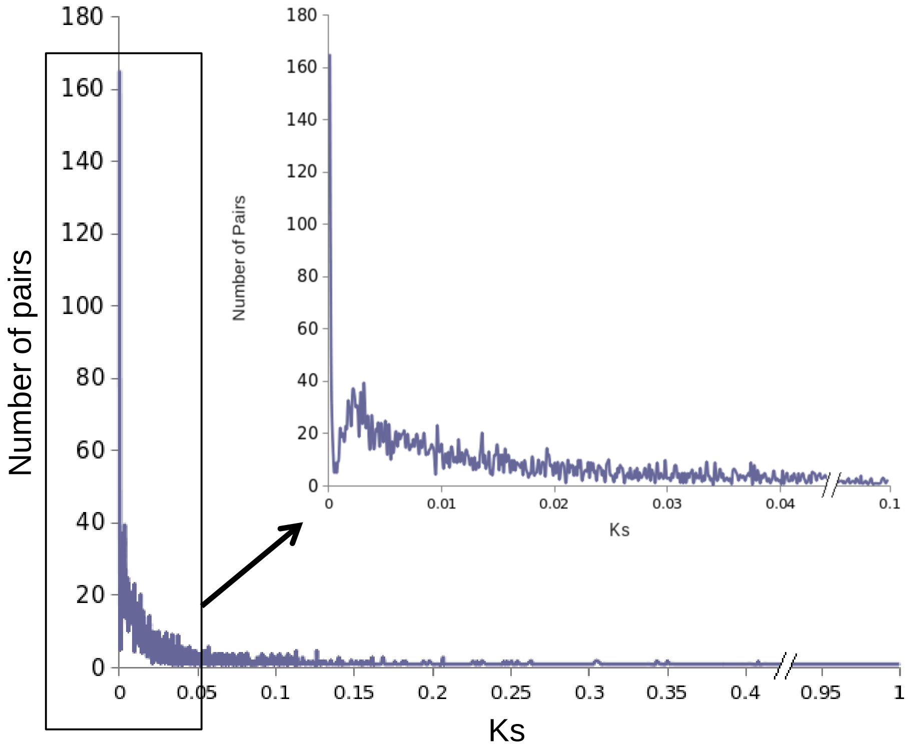

Supplementary Figure S10. Distribution of Ks of orthologous gene pairs between *desi* (ICC 4958) and *kabuli* (CDC Frontier) chickpea plotted against number of gene pairs. Inset shows distribution of Ks up to 0.1.

Supplementary Table S1. Chickpea (ICC4958) genome sequence data generated to improve assembly

Sequence generated using 454/Roche GSFLX+ platform

**Library type**	**Insert length**	**Avg. read length (bases)**	**filtered reads**	**Bases**
MP	20 kb	390	1,459,999	570,444,270
MP	20 kb	408	1,076135	439,769,976

NCBI submission ID: SUB48968, Bioproject ID: PRJNA78951, Sequence Read Archive (SRA) Nos: SRR1647666, SRR1647667, SRR1647669, SRR1647670

Paired-end sequence using Illumina/HiSeq1000 platform

**Library type**	**Insert Length (bp)**	**Read length (bases)**	**Filtered reads**	**Bases**
WGS	320-620	100PE	236,940,924	23,931,033,324

NCBI submission ID: SUB48968, Bioproject ID: PRJNA78951, Sequence Read Archive (SRA) No: SRR1632264

Supplementary Table S2. Result of mapping pseudomolecules with sequence reads

**LGs**	**Total Length (bases)**	**'N's (bases)**	**Mapped bases**
Ca _LG _1	39901017	3287587	36613430
Ca _LG _2	33233457	3230595	30002862
Ca _LG _3	42267542	3476199	38791343
Ca _LG _4	54992815	4754373	50238442
Ca _LG _5	45819701	4119109	41700592
Ca _LG _6	54841389	4808745	50032644
Ca _LG _7	45279478	3785643	41493835
Ca _LG _8	17664089	1458292	16205797

Supplementary Table S3. Transcriptome coverage in chickpea var. ICC 4958 genome assembly

Dataset	Number	With >90% identity and >80% coverage		With >= 90% identity and >90% coverage	
		**Number**	**Percentage**	**Number**	**Percentage**
**ICC 4958 Transcripts**					
All	34,760	33,197	95.50%	33,080	95.17%
>500 bp	21,399	20,174	94.28%	20,165	94.23%
>1000 bp	13,803	12,955	93.86%	12,952	93.83%
>2000 bp	4,594	4,293	93.45%	4,292	93.43%
**CaTA v2 Transcripts**					
All	46,369	42,802	92.31%	41,805	90.16%
>500 bp	25,197	22,828	90.60%	22,217	88.17%
>1000 bp	17,604	15,890	90.26%	15,643	88.86%
>2000 bp	6,138	5,751	93.70%	5,734	93.42%

Supplementary Table S4. Assessment of gene prediction using CEGMA pipeline

**Characteristics**	**Number**	**Percentage**
Total KOGs	458	
Total KOGs detected in the genome using CEGMA pipeline	453	98.90%
Total KOGs aligned on genes (E-value >= 1E-10)	449	98.03%
KOGs aligned (coverage >= 80%)	437	95.41%
KOGs aligned (coverage >= 50%)	444	96.94%
KOGs detected in the genome but not aligned to predicted gene set	4	0.008%

Supplementary Table S5. Experimental evidence of expression of protein-coding genes by mapping assembled transcripts and RNAseq data

	**Gene models (Total 30257)**	
**Dataset**	**Number**	**Percentage**
ICC4958 Transcripts	24843	82.1%
CaTA v2 Transcripts	24312	80.3%
At least one	25770	85.17%
RNAseq reads (NCBI SRA accession nos.)		
SRR(627762-627772)<br>SRR(063783-063785)<br>SRR(252664-252665)	25986	85.88%
At least one dataset	25986	85.88%

Supplementary Table S6. Functional annotation of the predicted protein-coding genes

**Database**	**Number**	**Percentage**
NCBI ntdb	28,231	93.30%
Swissprot	20,612	68.12%
TrEMBL	27,419	90.62%
UniRef100	28,904	95.52%
TAIR	23,734	78.44%
GO	23,003	76.02%
KEGG	9,252	30.57%
Total annotated[1]	29,553	97.67%

[1]number of unique genes showing hit with at least one of the databases

Supplementary Table S7. Non-coding RNA genes in the chickpea advanced draft genome assembly

**Type**		**Number of total loci**	**Average length (bases)**	**Length (bases)**
tRNA		753	75.12	56564
rRNA	18S	72	340.79	24537
	28S	33	223.79	7385
	5.8S	44	227.43	10007
	5S	80	128.61	10289
	Total rRNA	229	228.03	52218
miRNA		555	110.62	61394
snoRNA		414	79.55	32932

Supplementary Table S8. Comparison of different genomic features of linkage groups, pericentromeric and euchromatic regions in chickpea advanced draft genome assembly

	Linkage Groups			
	**Physical length (bases)**	**Genetic length (cM)**	**kb/cM per chromosome**	**Number of genes**
Chr1	39,901,017	146.63	272.12	2872
Chr2	33,233,457	151.36	219.57	2177
Chr3	42,267,542	100.61	420.11	2977
Chr4	54,992,815	137.34	400.41	3853
Chr5	45,819,701	114.46	400.31	3312
Chr6	54,841,389	125.45	437.16	3794
Chr7	45,279,478	102.22	442.96	3174
Chr8	17,664,089	138.96	127.12	1456
**Total**	**333,999,488**			**23615**
**Average**			**339.97**	

	Pericentromeric region			
	**Physical Length (base)**	**Genetic Length (cM)**	**Kb/cM per chromosome**	**No. of Genes**
Chr1	23400000	21.1	1109.004739	1311
Chr2	17200000	16.847	1020.953285	926
Chr3	23400000	5.25	4457.142857	1228
Chr4	21390000	8.97	2384.615385	1100
Chr5	24800000	32.092	772.7782625	1322
Chr6	28400000	18.429	1541.049433	1578
Chr7	21400000	14.222	1504.711011	1169
Chr8	10200000	13.777	740.3643754	848
**Total**	**170190000**			**9482**
**Average**			**1691.327419**	

	Euchromatic region			
	**Physical Length (base)**	**Genetic Length (cM)**	**kb/cM euchromatic arms**	**No. of genes**
Chr1	16501017	125.53	131.4507847	1561
Chr2	16033457	134.513	119.1963379	1251
Chr3	18867542	95.36	197.8559354	1749
Chr4	33602815	128.37	261.7653268	2753
Chr5	21019701	82.368	255.1925626	1990
Chr6	26441389	107.021	247.0672952	2216
Chr7	23879478	87.998	271.3638719	2005
Chr8	7464089	125.183	59.62542038	608
**Total**	**163809488**			**14133**
**Average**			**192.9396919**	

Supplementary Table S9. Comparison of length and 'N'-content of the pseudomolecules in ICC 4958 and CDC Frontier assemblies

LGs	ICC 4958			CDC Frontier		
	**Bases**	**%N**	**Valid bases (A+T+G+C)**	**Bases**	**%N**	**Valid bases (A+T+G+C)**
LG1	39901017	8.24	36613434	49165945	10.47	44019308
LG2	33233457	9.72	30002866	37245437	11.31	33033172
LG3	42267542	8.22	38791347	40655487	8.31	37277311
LG4	54992815	8.64	50238446	50011546	7.48	44075125
LG5	45819701	8.99	41700596	48971958	12.12	43035537
LG6	54841389	8.77	50032648	60454965	10.13	54328525
LG7	45279478	8.36	41493839	49777588	8.24	45675118
LG8	17664089	8.25	16205801	16751926	12.79	14608853

Supplementary Table S10. Transcription factor (TF) families in *desi* chickpea genome assembly and comparison with other sequenced legumes. Total number of TFs in each assembly is mentioned in bracket

TF Families	*Cicer arietinum* (2230)	*Glycine max* (5069)	*Cajanus cajan* (1886)	*Medicago truncatula* (1663)	*Lotus japonicas* (1311)
AP2	35	76	25	20	14
ARF	31	85	26	26	11
ARR-B	12	42	15	15	10
B3	56	112	47	85	30
BBR/BPC	5	22	5	2	4
BES1	11	19	6	6	4
bHLH	193	480	174	133	96
bZIP	97	266	69	66	39
C2H2	135	267	120	112	85
C3H	90	136	41	44	40
CAMTA	9	23	10	8	4
CO-like	14	32	10	9	8
CPP	5	19	6	6	5
DBB	7	36	11	7	6
Dof	67	93	39	26	24
E2F-DP	17	28	7	7	6
EIL	7	12	6	12	4
ERF	159	330	148	106	123
FAR1	87	103		124	10
G2-like	53	164	49	35	31
GATA	49	70	33	36	19
GeBP	11	11	7	4	3
GRAS	61	139	60	61	50
GRF	14	31	10	4	4
HB-other	14	31	7	16	14
HB-PHD	3	11	2	1	2
HD-ZIP	79	140	53	34	28
HRT-like	2	1	1	3	1
HSF	31	61	27	21	15
LBD	46	111	54	33	45
LFY	1	2	1	1	2
LSD	3	17	7	3	4
M-type	39	88	58	73	56
MIKC	30	160	28	16	19
MYB	125	369	179	101	82
MYB-related	121	265	94	84	86
NAC	131	247	96	75	98
NF-X1	3	8	1	3	4
NF-YA	10	57	13	8	6
NF-YB	20	46	23	12	11
NF-YC	22	35	14	13	9
Nin-like	14	45	16	8	11
RAV	2	5	2	3	1
S1Fa-like	8	4	2	4	3
SAP	24	2	1	1	1

**TF Families**	***Cicer arietinum* (2230)**	***Glycine max* (5069)**	***Cajanus cajan* (1886)**	***Medicago truncatula* (1663)**	***Lotus japonicas* (1311)**
SBP	23	73	24	13	14
SRS	11	33	10	10	9
STAT	3	1	1		
TALE	28	101	32	13	13
TCP	30	71	28	15	21
Trihelix	39	93	38	28	23
VOZ	1	20	2	1	1
Whirly	3	13	3	1	2
WOX	17	42	19	11	10
WRKY	103	233	97	81	66
YABBY	7	34	9	8	4
zf-HD	12	54	20	15	20

Supplementary Table S11. Comparison of *R*-gene family in chickpea draft genome with other sequenced plant genomes

***R*-gene families**	**Chickpea**	**Common bean**	***M. truncatula***	***Arabidopsis***	**Grape**
CC-NBS-LRR	26	104	124	25	15
TIR-NBS-LRR	14	93	298	125	57
NBS-LRR	29	155	147	8	60
CC-NBS	12	4	129	15	164
NBS	27	7	111	12	37
TIR	15		10	31	2
TIR-NBS	10	13	9	17	1
Total	133	537	828	233	336

Receptor like kinases	623	1131	579	586	527

Supplementary Table S12. General features of chickpea-specific and orphan genes

**Features**	**Chickpea-specific genes**	**Orphan genes**
Number of genes	3296	704
Total gene length (bases)	4541393	417053
Average gene length (bases)	1377.85	592.40
Total CDS length (bases)	2333238	221028
Average CDS length (bases)	707.89	313.96
Average exon length (bases)	262.44	159.15
Average no. of exon per gene	2.78	1.99
GC content in CDS	42.04%	43.32%

Supplementary Table S13. Orphan genes in chickpea advanced draft assembly and conserved domains present in the presented peptide models

Hit type	PSSM-ID	From	To	E-Value	Bitscore	Accession	Short name
superfamily	276192	12	36	0.00652517	30.6314	cl17169	RRM_SF superfamily
superfamily	276192	12	36	0.00652517	30.6314	cl17169	RRM_SF superfamily
multi-dom	178945	3	84	0.00194572	34.0559	PRK00247	PRK00247
multi-dom	139913	64	101	0.00458659	33.6775	PRK13853	PRK13853
superfamily	275482	32	89	0.00928501	32.1414	cl00200	MIP superfamily
multi-dom	181883	49	75	0.0073492	34.1794	PRK09468	ompR
superfamily	271750	7	65	1.0286e-05	40.398	cl14603	C2 superfamily
multi-dom	131106	5	78	0.00597543	31.9682	TIGR02051	Mercuric_resistance_operon_ regulatory_protein
superfamily	276217	17	44	0.000955868	35.3425	cl18945	AAT_I superfamily
multi-dom	223021	77	293	0.000714026	39.9217	PHA03247	PHA03247
multi-dom	215541	54	141	0.000888409	37.3464	PLN03020	PLN03020
superfamily	255361	10	58	0.00845726	31.0814	cl09671	Wbp11 superfamily
multi-dom	215138	62	84	0.00987916	32.3101	PLN02248	PLN02248
superfamily	275532	213	280	0.00458096	36.3044	cl00325	Ribosomal_L4 superfamily
superfamily	265803	56	92	0.00806492	32.9141	cl15846	Phage_F superfamily
superfamily	256162	35	70	0.00422656	31.4803	cl11371	XhlA superfamily
superfamily	271754	35	84	4.01203e-06	40.3753	cl14782	RNase_H_like superfamily
superfamily	265821	49	79	0.000839791	34.4705	cl15874	UBN2 superfamily
superfamily	275783	1	53	0.00966805	31.7884	cl02872	DHQ_Fe-ADH superfamily
multi-dom	215130	14	66	0.00357307	33.1388	PLN02217	PLN02217
multi-dom	113902	82	124	0.00799925	33.8597	pfam05149	Flagellar_rod
superfamily	268208	39	85	0.00505596	33.0564	cl19855	DUF1501 superfamily
superfamily	268208	39	85	0.00505596	33.0564	cl19855	DUF1501 superfamily
superfamily	268208	39	85	0.00505596	33.0564	cl19855	DUF1501 superfamily
multi-dom	182457	15	91	0.00195234	34.6629	PRK10434	srlR
superfamily	271925	39	94	0.00651585	32.4812	cl21505	SpoU_methylase superfamily
superfamily	259313	3	63	0.00841096	33.2795	cl21044	Myc_target_1 superfamily
superfamily	275580	15	59	0.00592992	31.4771	cl00447	Nudix_Hydrolase superfamily
multi-dom	113902	82	124	0.00799925	33.8597	pfam05149	Flagellar_rod

Supplementary Table S14. Chickpea-specific genes in advanced draft assembly and conserved domains present in presented peptide models

Query	Types	PSSM-ID	From	To	E-Value	Bitscore	Accession	Short name	Superfamily
Q#1 - >Ca_00014.1|Ca_	specific	238166	2	76	1.15038e-16	72.6779	cd00266	MADS_SRF_like	cl00109
Q#1 - >Ca_00014.1|Ca_	superfamily	241616	2	76	1.15038e-16	72.6779	cl00109	MADS superfamily	-
Q#3 - >Ca_00019.1|Ca_	specific	260006	275	382	8.64961e-46	160.351	cd09274	RNase_HI_RT_Ty3	cl14782
Q#3 - >Ca_00019.1|Ca_	superfamily	271754	275	382	8.64961e-46	160.351	cl14782	RNase_H_like superfamily	-
Q#3 - >Ca_00019.1|Ca_	specific	255851	74	152	5.69625e-37	134.441	pfam10252	PP28	cl10936
Q#3 - >Ca_00019.1|Ca_	superfamily	255851	74	152	5.69625e-37	134.441	cl10936	PP28 superfamily	-
Q#3 - >Ca_00019.1|Ca_	specific	250040	510	596	1.06425e-08	53.8765	pfam00665	rve	cl21549
Q#3 - >Ca_00019.1|Ca_	superfamily	276355	510	596	1.06425e-08	53.8765	cl21549	rve superfamily	-
Q#5 - >Ca_00023.1|Ca_	specific	132921	26	185	3.72253e-91	275.527	cd07038	TPP_PYR_PDC_IPDC_like	cl11410
Q#5 - >Ca_00023.1|Ca_	superfamily	264357	26	185	3.72253e-91	275.527	cl11410	TPP_enzyme_PYR superfamily	-
Q#5 - >Ca_00023.1|Ca_	specific	238963	248	413	7.28811e-82	252.07	cd02005	TPP_PDC_IPDC	cl01629
Q#5 - >Ca_00023.1|Ca_	superfamily	275737	248	413	7.28811e-82	252.07	cl01629	TPP_enzymes superfamily	-
Q#5 - >Ca_00023.1|Ca_	multi-dom	215311	8	430	0	897.138	PLN02573	PLN02573	-
Q#8 - >Ca_00053.1|Ca_	specific	250476	7	188	3.73443e-102	294.937	pfam01251	Ribosomal_S7e	cl03141
Q#8 - >Ca_00053.1|Ca_	superfamily	261606	7	188	3.73443e-102	294.937	cl03141	Ribosomal_S7e superfamily	-
Q#10 - >Ca_00072.1|Ca_	specific	252797	155	206	4.19388e-08	52.4246	pfam04782	DUF632	cl04746
Q#10 - >Ca_00072.1|Ca_	superfamily	252797	155	206	4.19388e-08	52.4246	cl04746	DUF632 superfamily	-
Q#10 - >Ca_00072.1|Ca_	superfamily	191091	1	20	0.00568743	34.0905	cl04747	DUF630 superfamily	-
Q#11 - >Ca_00080.1|Ca_	superfamily	275603	378	471	9.08566e-16	76.9873	cl00490	EEP superfamily	-
Q#11 - >Ca_00080.1|Ca_	superfamily	258218	25	65	4.53211e-06	44.8978	cl16506	zf-RVT superfamily	-
Q#13 - >Ca_00107.1|Ca_	specific	254925	14	166	1.17066e-40	136.266	pfam08617	CGI-121	cl00698
Q#13 - >Ca_00107.1|Ca_	superfamily	260578	14	166	1.17066e-40	136.266	cl00698	CGI-121 superfamily	-
Q#14 - >Ca_00111.1|Ca_	specific	214815	141	199	7.35076e-33	116.047	smart00774	WRKY	cl03892
Q#14 - >Ca_00111.1|Ca_	superfamily	243565	141	199	7.35076e-33	116.047	cl03892	WRKY superfamily	-
Q#15 - >Ca_00130.1|Ca_	specific	254721	52	89	2.17773e-08	46.3883	pfam08312	cwf21	cl07067
Q#15 - >Ca_00130.1|Ca_	superfamily	254721	52	89	2.17773e-08	46.3883	cl07067	cwf21 superfamily	-
Q#16 - >Ca_00132.1|Ca_	specific	254721	52	89	2.4037e-08	46.0031	pfam08312	cwf21	cl07067
Q#16 - >Ca_00132.1|Ca_	superfamily	254721	52	89	2.4037e-08	46.0031	cl07067	cwf21 superfamily	-
Q#17 - >Ca_00169.1|Ca_	specific	257351	40	70	4.28439e-09	51.1961	pfam12854	PPR_1	cl03252
Q#17 - >Ca_00169.1|Ca_	superfamily	275791	40	70	4.28439e-09	51.1961	cl03252	PPR superfamily	-
Q#17 - >Ca_00169.1|Ca_	specific	257351	109	141	2.87364e-05	40.4105	pfam12854	PPR_1	cl03252
Q#17 - >Ca_00169.1|Ca_	superfamily	275791	109	141	2.87364e-05	40.4105	cl03252	PPR superfamily	-
Q#17 - >Ca_00169.1|Ca_	multi-dom	257477	113	160	6.94784e-12	59.3386	pfam13041	PPR_2	-
Q#17 - >Ca_00169.1|Ca_	multi-dom	257477	183	232	1.82325e-10	55.4866	pfam13041	PPR_2	-

Query	Types	PSSM-ID	From	To	E-Value	Bitscore	Accession	Short name	Superfamily
Q#17 - >Ca_00169.1|Ca_	multi-dom	257477	78	127	3.57778e-09	52.0198	pfam13041	PPR_2	-
Q#17 - >Ca_00169.1|Ca_	multi-dom	257477	43	89	3.68565e-09	51.6346	pfam13041	PPR_2	-
Q#17 - >Ca_00169.1|Ca_	multi-dom	257477	22	57	1.01252e-06	45.0862	pfam13041	PPR_2	-
Q#18 - >Ca_00170.1|Ca_	specific	250966	23	86	1.81201e-08	47.2117	pfam01918	Alba	cl00682
Q#18 - >Ca_00170.1|Ca_	superfamily	260569	23	86	1.81201e-08	47.2117	cl00682	Alba superfamily	-
Q#23 - >Ca_00209.1|Ca_	specific	238204	19	236	3.59945e-64	202.098	cd00333	MIP	cl00200
Q#23 - >Ca_00209.1|Ca_	superfamily	275482	19	236	3.59945e-64	202.098	cl00200	MIP superfamily	-
Q#26 - >Ca_00324.1|Ca_	specific	238008	11	75	1.43985e-16	67.9581	cd00051	EFh	cl08302
Q#26 - >Ca_00324.1|Ca_	superfamily	275859	11	75	1.43985e-16	67.9581	cl08302	EFh superfamily	-
Q#26 - >Ca_00324.1|Ca_	multi-dom	257819	19	74	1.18073e-10	52.0009	pfam13499	EF-hand_7	-
Q#29 - >Ca_00380.1|Ca_	superfamily	265821	33	60	1.74871e-05	37.9373	cl15874	UBN2 superfamily	-
Q#30 - >Ca_00382.1|Ca_	specific	202502	1	153	1.95162e-77	236.774	pfam02992	Transposase_21	cl03823
Q#30 - >Ca_00382.1|Ca_	superfamily	202502	1	153	1.95162e-77	236.774	cl03823	Transposase_21 superfamily	-
Q#31 - >Ca_00384.1|Ca_	specific	237985	1	37	2.08743e-15	67.6819	cd00018	AP2	cl00033
Q#31 - >Ca_00384.1|Ca_	superfamily	260146	1	37	2.08743e-15	67.6819	cl00033	AP2 superfamily	-
Q#32 - >Ca_00402.1|Ca_	specific	238003	219	307	2.99934e-17	76.5084	cd00043	CYCLIN	cl00050
Q#32 - >Ca_00402.1|Ca_	superfamily	271412	219	307	2.99934e-17	76.5084	cl00050	CYCLIN superfamily	-
Q#32 - >Ca_00402.1|Ca_	specific	238003	316	404	6.72064e-16	72.6564	cd00043	CYCLIN	cl00050
Q#32 - >Ca_00402.1|Ca_	superfamily	271412	316	404	6.72064e-16	72.6564	cl00050	CYCLIN superfamily	-
Q#33 - >Ca_00412.1|Ca_	superfamily	260768	22	84	2.04426e-19	79.9669	cl01053	SGNH_hydrolase superfamily	-
Q#34 - >Ca_00413.1|Ca_	multi-dom	131079	143	212	0.00790341	35.5053	TIGR02024	FtcD	-
Q#35 - >Ca_00415.1|Ca_	superfamily	260768	14	177	1.23053e-41	144.295	cl01053	SGNH_hydrolase superfamily	-
Q#38 - >Ca_00461.1|Ca_	superfamily	271591	33	156	2.21158e-34	121.12	cl02544	VHS_ENTH_ANTH superfamily	-
Q#40 - >Ca_00469.1|Ca_	superfamily	276328	4	61	2.23557e-13	62.0202	cl21496	2OG-FeII_Oxy superfamily	-
Q#41 - >Ca_00474.1|Ca_	specific	238029	23	128	2.47109e-65	197.172	cd00074	H2A	cl00074
Q#41 - >Ca_00474.1|Ca_	superfamily	275467	23	128	2.47109e-65	197.172	cl00074	H2A superfamily	-
Q#41 - >Ca_00474.1|Ca_	multi-dom	177758	23	135	6.86812e-69	207.01	PLN00157	PLN00157	-
Q#42 - >Ca_00475.1|Ca_	superfamily	276299	15	172	3.41978e-95	277.227	cl21455	ABC_ATPase superfamily	-
Q#42 - >Ca_00475.1|Ca_	multi-dom	249560	19	171	1.13111e-76	230.085	pfam00071	Ras	-
Q#43 - >Ca_00477.1|Ca_	specific	257687	82	250	5.1557e-34	122.741	pfam13359	DDE_Tnp_4	cl21562
Q#43 - >Ca_00477.1|Ca_	superfamily	271982	82	250	5.1557e-34	122.741	cl21562	DDE_Tnp_1_6 superfamily	-
Q#44 - >Ca_00482.1|Ca_	specific	271353	2	82	4.48115e-10	56.4763	cd14798	RX-CC_like	cl22426
Q#44 - >Ca_00482.1|Ca_	superfamily	271353	2	82	4.48115e-10	56.4763	cl22426	RX-CC_like superfamily	-
Q#45 - >Ca_00533.1|Ca_	specific	251315	2	127	2.97052e-15	68.8228	pfam02469	Fasciclin	cl02663
Q#45 - >Ca_00533.1|Ca_	superfamily	261399	2	127	2.97052e-15	68.8228	cl02663	Fasciclin superfamily	-
Q#47 - >Ca_00564.1|Ca_	superfamily	275781	21	96	1.86789e-30	112.165	cl02777	chaperonin_like superfamily	-

Query	Types	PSSM-ID	From	To	E-Value	Bitscore	Accession	Short name	Superfamily
Q#48 - >Ca_00594.1|Ca_	specific	238088	15	113	2.81544e-19	78.3865	cd00156	REC	cl19078
Q#48 - >Ca_00594.1|Ca_	superfamily	271833	15	113	2.81544e-19	78.3865	cl19078	REC superfamily	-
Q#49 - >Ca_00633.1|Ca_	superfamily	272026	14	116	2.82458e-19	83.1789	cl21606	PLN02247 superfamily	-
Q#51 - >Ca_00659.1|Ca_	superfamily	275544	11	138	3.11262e-37	125.907	cl00350	Ribosomal_S19 superfamily	-
Q#52 - >Ca_00660.1|Ca_	superfamily	265404	1	92	1.85248e-32	110.173	cl13983	DUF3774 superfamily	-
Q#54 - >Ca_00756.1|Ca_	specific	256054	283	337	2.67221e-07	47.4557	pfam10551	MULE	cl19537
Q#54 - >Ca_00756.1|Ca_	superfamily	276269	283	337	2.67221e-07	47.4557	cl19537	Transposase_mut superfamily	-
Q#54 - >Ca_00756.1|Ca_	superfamily	190526	192	235	0.000250665	38.3526	cl07846	DBD_Tnp_Mut superfamily	-
Q#56 - >Ca_00800.1|Ca_	specific	211390	122	175	1.97198e-07	46.4246	cd11378	DUF296	cl00720
Q#56 - >Ca_00800.1|Ca_	superfamily	260588	122	175	1.97198e-07	46.4246	cl00720	DUF296 superfamily	-
Q#57 - >Ca_00841.1|Ca_	superfamily	252133	129	199	1.19736e-08	50.4149	cl04237	Retrotrans_gag superfamily	-
Q#58 - >Ca_00842.1|Ca_	specific	257974	12	73	2.73786e-24	93.0423	pfam13668	Ferritin_2	cl00264
Q#58 - >Ca_00842.1|Ca_	superfamily	275506	12	73	2.73786e-24	93.0423	cl00264	Ferritin_like superfamily	-
Q#59 - >Ca_00843.1|Ca_	superfamily	276299	82	132	4.21097e-18	77.1397	cl21455	ABC_ATPase superfamily	-
Q#59 - >Ca_00843.1|Ca_	superfamily	271701	1	43	1.86212e-13	63.1276	cl10459	Peptidases_S8_S53 superfamily	-
Q#60 - >Ca_00856.1|Ca_	specific	238029	9	121	1.6096e-66	199.484	cd00074	H2A	cl00074
Q#60 - >Ca_00856.1|Ca_	superfamily	275467	9	121	1.6096e-66	199.484	cl00074	H2A superfamily	-
Q#60 - >Ca_00856.1|Ca_	multi-dom	177758	1	128	3.72775e-75	222.033	PLN00157	PLN00157	-
Q#61 - >Ca_00860.1|Ca_	specific	197943	1	48	1.37534e-05	41.5581	smart00875	BACK	cl06652
Q#61 - >Ca_00860.1|Ca_	superfamily	271628	1	48	1.37534e-05	41.5581	cl06652	BACK_like superfamily	-
Q#63 - >Ca_00879.1|Ca_	multi-dom	273328	39	134	3.25344e-07	46.9327	TIGR00898	organic_anion_transporter	-
Q#65 - >Ca_00973.1|Ca_	specific	250639	81	156	1.92954e-21	83.7552	pfam01466	Skp1	cl03226
Q#65 - >Ca_00973.1|Ca_	superfamily	250639	81	156	1.92954e-21	83.7552	cl03226	Skp1 superfamily	-
Q#65 - >Ca_00973.1|Ca_	superfamily	271586	9	106	1.29573e-14	65.7777	cl02518	BTB superfamily	-
Q#65 - >Ca_00973.1|Ca_	multi-dom	227528	10	154	5.17793e-22	87.3068	COG5201	SKP1	-
Q#67 - >Ca_01023.1|Ca_	superfamily	271686	14	119	8.92408e-30	108.106	cl10012	DnaQ_like_exo superfamily	-
Q#68 - >Ca_01048.1|Ca_	superfamily	253938	28	52	0.000663403	32.3609	cl06070	zf-GRF superfamily	-
Q#71 - >Ca_01058.1|Ca_	specific	260011	8	134	1.92907e-45	146.464	cd09279	RNase_HI_like	cl14782
Q#71 - >Ca_01058.1|Ca_	superfamily	271754	8	134	1.92907e-45	146.464	cl14782	RNase_H_like superfamily	-
Q#72 - >Ca_01082.1|Ca_	multi-dom	258434	10	78	3.17377e-22	85.0974	pfam14244	UBN2_3	-
Q#75 - >Ca_01094.1|Ca_	superfamily	256046	41	144	2.62129e-27	110.177	cl11168	PMD superfamily	-
Q#76 - >Ca_01103.1|Ca_	superfamily	256046	87	150	1.36972e-07	48.1596	cl11168	PMD superfamily	-
Q#80 - >Ca_01121.1|Ca_	superfamily	276194	73	349	5.33851e-92	281.184	cl17173	AdoMet_MTases superfamily	-
Q#81 - >Ca_01124.1|Ca_	multi-dom	173561	103	163	0.000263079	38.6345	PTZ00368	PTZ00368	-
Q#84 - >Ca_01153.1|Ca_	multi-dom	258434	59	83	0.000155329	36.5622	pfam14244	UBN2_3	-
Q#86 - >Ca_01160.1|Ca_	superfamily	265821	1	50	1.11422e-08	52.5749	cl15874	UBN2 superfamily	-

Query	Types	PSSM-ID	From	To	E-Value	Bitscore	Accession	Short name	Superfamily
Q#88 - >Ca_01191.1|Ca_	superfamily	271849	121	163	4.18008e-11	59.1591	cl19250	Sulfate_transp superfamily	-
Q#91 - >Ca_01208.1|Ca_	multi-dom	223021	182	337	5.61812e-06	48.0109	PHA03247	PHA03247	-
Q#96 - >Ca_01232.1|Ca_	multi-dom	179712	42	81	0.0022504	35.6123	PRK04019	rplP0	-
Q#101 - >Ca_01287.1|Ca_	superfamily	275481	21	104	0.0096214	34.4991	cl00192	ribokinase_pfkB_like superfamily	-
Q#102 - >Ca_01302.1|Ca_	specific	252681	16	109	5.35928e-32	117.395	pfam04576	Zein-binding	cl04604
Q#102 - >Ca_01302.1|Ca_	superfamily	252681	16	109	5.35928e-32	117.395	cl04604	Zein-binding superfamily	-
Q#103 - >Ca_01330.1|Ca_	superfamily	275922	4	91	4.39072e-19	78.3317	cl10555	Chorismate_bind superfamily	-
Q#104 - >Ca_01343.1|Ca_	specific	250639	79	153	3.19665e-23	87.9924	pfam01466	Skp1	cl03226
Q#104 - >Ca_01343.1|Ca_	superfamily	250639	79	153	3.19665e-23	87.9924	cl03226	Skp1 superfamily	-
Q#104 - >Ca_01343.1|Ca_	superfamily	271586	6	104	2.4644e-14	65.0073	cl02518	BTB superfamily	-
Q#104 - >Ca_01343.1|Ca_	multi-dom	227528	6	152	1.4727e-25	96.5515	COG5201	SKP1	-
Q#105 - >Ca_01344.1|Ca_	specific	147159	14	255	6.92432e-90	269.505	pfam04857	CAF1	cl10012
Q#105 - >Ca_01344.1|Ca_	superfamily	271686	14	255	6.92432e-90	269.505	cl10012	DnaQ_like_exo superfamily	-
Q#107 - >Ca_01350.1|Ca_	superfamily	250320	73	130	0.00116355	36.6283	cl20213	Vinculin superfamily	-
Q#109 - >Ca_01370.1|Ca_	multi-dom	227952	327	475	0.00792382	37.3438	COG5665	NOT5	-
Q#112 - >Ca_01394.1|Ca_	multi-dom	273731	44	270	1.50822e-70	232.988	TIGR01647	Plasma_membrane_ATPase	-
Q#112 - >Ca_01394.1|Ca_	multi-dom	173412	6	63	0.000211892	41.2799	PTZ00121	PTZ00121	-
Q#114 - >Ca_01426.1|Ca_	multi-dom	251763	58	199	0.000445763	41.5964	pfam03154	Atrophin-1	-
Q#117 - >Ca_01439.1|Ca_	specific	260004	63	186	1.17918e-64	198.846	cd09272	RNase_HI_RT_Ty1	cl14782
Q#117 - >Ca_01439.1|Ca_	superfamily	271754	63	186	1.17918e-64	198.846	cl14782	RNase_H_like superfamily	-
Q#119 - >Ca_01466.1|Ca_	specific	257687	291	448	7.64284e-40	142.001	pfam13359	DDE_Tnp_4	cl21562
Q#119 - >Ca_01466.1|Ca_	superfamily	271982	291	448	7.64284e-40	142.001	cl21562	DDE_Tnp_1_6 superfamily	-
Q#121 - >Ca_01489.1|Ca_	multi-dom	215145	121	214	0.00349285	37.757	PLN02258	PLN02258	-
Q#121 - >Ca_01489.1|Ca_	multi-dom	185628	42	173	0.00942904	36.5914	PTZ00449	PTZ00449	-
Q#122 - >Ca_01494.1|Ca_	superfamily	206228	31	84	0.00394504	36.2077	cl16577	PcfK superfamily	-
Q#123 - >Ca_01497.1|Ca_	specific	239719	45	270	7.15882e-153	434.057	cd03750	proteasome_alpha_type_2	cl00467
Q#123 - >Ca_01497.1|Ca_	superfamily	275593	45	270	7.15882e-153	434.057	cl00467	Ntn_hydrolase superfamily	-
Q#123 - >Ca_01497.1|Ca_	multi-dom	223711	43	268	7.70982e-75	234.885	COG0638	PRE1	-
Q#125 - >Ca_01509.1|Ca_	specific	258213	20	48	1.03363e-06	45.1094	pfam13961	DUF4219	cl22382
Q#125 - >Ca_01509.1|Ca_	superfamily	258213	20	48	1.03363e-06	45.1094	cl22382	DUF4219 superfamily	-
Q#125 - >Ca_01509.1|Ca_	specific	197667	97	113	0.00808398	33.956	smart00343	ZnF_C2HC	cl22700
Q#125 - >Ca_01509.1|Ca_	superfamily	276654	97	113	0.00808398	33.956	cl22700	zf-CCHC superfamily	-
Q#127 - >Ca_01529.1|Ca_	specific	238166	2	76	1.3776e-16	73.0631	cd00266	MADS_SRF_like	cl00109
Q#127 - >Ca_01529.1|Ca_	superfamily	241616	2	76	1.3776e-16	73.0631	cl00109	MADS superfamily	-
Q#127 - >Ca_01529.1|Ca_	superfamily	271918	241	303	0.00316208	35.3302	cl21498	SANT superfamily	-
Q#129 - >Ca_01532.1|Ca_	superfamily	271593	1	259	2.523e-23	95.8648	cl02567	WD40 superfamily	-

Query	Types	PSSM-ID	From	To	E-Value	Bitscore	Accession	Short name	Superfamily
Q#129 - >Ca_01532.1|Ca_	multi-dom	225201	21	273	2.39613e-19	86.2959	COG2319	COG2319	-
Q#134 - >Ca_01561.1|Ca_	multi-dom	237030	69	144	0.00918946	34.8662	PRK12270	kgd	-
Q#135 - >Ca_01567.1|Ca_	specific	119398	49	127	1.42122e-16	70.6923	cd06530	S26_SPase_I	cl10465
Q#135 - >Ca_01567.1|Ca_	superfamily	275919	49	127	1.42122e-16	70.6923	cl10465	Peptidase_S24_S26 superfamily	-
Q#136 - >Ca_01597.1|Ca_	superfamily	254039	130	270	3.51082e-57	186.743	cl06203	DUF1336 superfamily	-
Q#137 - >Ca_01598.1|Ca_	superfamily	271753	39	142	1.14392e-15	69.6745	cl14643	SRPBCC superfamily	-
Q#139 - >Ca_01621.1|Ca_	specific	251717	78	262	3.59851e-63	200.248	pfam03080	DUF239	cl03877
Q#139 - >Ca_01621.1|Ca_	superfamily	251717	78	262	3.59851e-63	200.248	cl03877	DUF239 superfamily	-
Q#139 - >Ca_01621.1|Ca_	superfamily	206533	47	78	1.74304e-13	64.6026	cl16830	DUF4409 superfamily	-
Q#140 - >Ca_01622.1|Ca_	superfamily	276297	2	45	7.73114e-19	78.9487	cl21453	PKc_like superfamily	-
Q#141 - >Ca_01623.1|Ca_	specific	206130	50	166	3.82809e-76	231.324	pfam13960	DUF4218	cl16501
Q#141 - >Ca_01623.1|Ca_	superfamily	206130	50	166	3.82809e-76	231.324	cl16501	DUF4218 superfamily	-
Q#141 - >Ca_01623.1|Ca_	superfamily	258207	201	232	1.74034e-12	61.4431	cl16495	DUF4216 superfamily	-
Q#142 - >Ca_01627.1|Ca_	multi-dom	178748	296	460	0.00311692	38.7539	PLN03209	PLN03209	-
Q#143 - >Ca_01642.1|Ca_	superfamily	275563	24	125	3.43923e-31	108.31	cl00388	Thioredoxin_like superfamily	-
Q#144 - >Ca_01651.1|Ca_	specific	238284	22	122	1.58573e-57	176.181	cd00513	Ribosomal_L32_L32e	cl00748
Q#144 - >Ca_01651.1|Ca_	superfamily	260601	22	122	1.58573e-57	176.181	cl00748	Ribosomal_L32_L32e superfamily	-
Q#145 - >Ca_01664.1|Ca_	specific	260011	3	129	1.47901e-45	146.849	cd09279	RNase_HI_like	cl14782
Q#145 - >Ca_01664.1|Ca_	superfamily	271754	3	129	1.47901e-45	146.849	cl14782	RNase_H_like superfamily	-
Q#147 - >Ca_01675.1|Ca_	superfamily	252133	100	199	2.98528e-10	55.8077	cl04237	Retrotrans_gag superfamily	-
Q#149 - >Ca_01692.1|Ca_	specific	238167	35	209	6.16312e-81	247.013	cd00268	DEADc	cl21455
Q#149 - >Ca_01692.1|Ca_	superfamily	276299	35	209	6.16312e-81	247.013	cl21455	ABC_ATPase superfamily	-
Q#149 - >Ca_01692.1|Ca_	specific	238034	209	296	2.06549e-31	115.413	cd00079	HELICc	cl21455
Q#149 - >Ca_01692.1|Ca_	superfamily	276299	209	296	2.06549e-31	115.413	cl21455	ABC_ATPase superfamily	-
Q#149 - >Ca_01692.1|Ca_	multi-dom	185609	10	327	2.29204e-142	411.142	PTZ00424	PTZ00424	-
Q#150 - >Ca_01704.1|Ca_	superfamily	275873	31	92	0.00187057	33.6016	cl09238	CY superfamily	-
Q#151 - >Ca_01714.1|Ca_	superfamily	276301	62	134	4.53391e-20	83.7445	cl21457	TIM_phosphate_binding superfamily	-
Q#152 - >Ca_01715.1|Ca_	specific	237985	16	76	9.34244e-27	98.1126	cd00018	AP2	cl00033
Q#152 - >Ca_01715.1|Ca_	superfamily	260146	16	76	9.34244e-27	98.1126	cl00033	AP2 superfamily	-
Q#153 - >Ca_01716.1|Ca_	specific	237985	16	76	1.04481e-27	100.809	cd00018	AP2	cl00033
Q#153 - >Ca_01716.1|Ca_	superfamily	260146	16	76	1.04481e-27	100.809	cl00033	AP2 superfamily	-
Q#154 - >Ca_01719.1|Ca_	specific	212549	25	129	4.91315e-38	126.956	cd11711	GINS_A_Sld5	cl17012
Q#154 - >Ca_01719.1|Ca_	superfamily	266497	25	129	4.91315e-38	126.956	cl17012	GINS_A superfamily	-
Q#155 - >Ca_01724.1|Ca_	superfamily	261399	214	300	0.000576643	38.1119	cl02663	Fasciclin superfamily	-
Q#157 - >Ca_01761.1|Ca_	multi-dom	257477	136	180	0.000310548	37.3822	pfam13041	PPR_2	-
Q#159 - >Ca_01791.1|Ca_	multi-dom	259534	129	206	0.00545072	38.5866	pfam15402	Spc7_N	-

Query	Types	PSSM-ID	From	To	E-Value	Bitscore	Accession	Short name	Superfamily
Q#160 - >Ca_01810.1|Ca_	specific	251760	1	118	6.22805e-22	86.4552	pfam03151	TPT	cl00910
Q#160 - >Ca_01810.1|Ca_	superfamily	275677	1	118	6.22805e-22	86.4552	cl00910	EamA superfamily	-
Q#161 - >Ca_01815.1|Ca_	specific	133136	133	191	1.03177e-09	54.2648	cd00303	retropepsin_like	cl11403
Q#161 - >Ca_01815.1|Ca_	superfamily	275927	133	191	1.03177e-09	54.2648	cl11403	pepsin_retropepsin_like superfamily	-
Q#162 - >Ca_01840.1|Ca_	specific	250455	41	123	3.61466e-36	120.74	pfam01221	Dynein_light	cl03131
Q#162 - >Ca_01840.1|Ca_	superfamily	261601	41	123	3.61466e-36	120.74	cl03131	Dynein_light superfamily	-
Q#163 - >Ca_01845.1|Ca_	superfamily	268118	6	254	4.22858e-21	89.8167	cl19765	mTERF superfamily	-
Q#164 - >Ca_01851.1|Ca_	superfamily	261287	51	121	0.000394717	37.1663	cl02440	DAGK_acc superfamily	-
Q#165 - >Ca_01853.1|Ca_	superfamily	261287	51	121	0.000394717	37.1663	cl02440	DAGK_acc superfamily	-
Q#166 - >Ca_01859.1|Ca_	specific	250455	38	122	5.09731e-37	122.666	pfam01221	Dynein_light	cl03131
Q#166 - >Ca_01859.1|Ca_	superfamily	261601	38	122	5.09731e-37	122.666	cl03131	Dynein_light superfamily	-
Q#167 - >Ca_01864.1|Ca_	specific	99751	37	83	2.67403e-16	68.3409	cd06257	DnaJ	cl02542
Q#167 - >Ca_01864.1|Ca_	superfamily	271590	37	83	2.67403e-16	68.3409	cl02542	DnaJ superfamily	-
Q#170 - >Ca_01918.1|Ca_	superfamily	271686	59	207	1.1579e-27	103.812	cl10012	DnaQ_like_exo superfamily	-
Q#171 - >Ca_01928.1|Ca_	superfamily	276194	1	97	6.9426e-57	181.023	cl17173	AdoMet_MTases superfamily	-
Q#172 - >Ca_01945.1|Ca_	specific	239926	65	131	1.48649e-14	64.5808	cd04480	RPA1_DBD_A_like	cl09930
Q#172 - >Ca_01945.1|Ca_	superfamily	275906	65	131	1.48649e-14	64.5808	cl09930	RPA_2b-aaRSs_OBF_like superfamily	-
Q#173 - >Ca_01946.1|Ca_	superfamily	276175	5	53	1.22248e-25	100.494	cl17037	NBD_sugar-kinase_HSP70_actin superfamily	-
Q#175 - >Ca_01983.1|Ca_	specific	176884	28	257	3.99839e-72	229.85	cd08875	START_ArGLABRA2_like	cl14643
Q#175 - >Ca_01983.1|Ca_	superfamily	271753	28	257	3.99839e-72	229.85	cl14643	SRPBCC superfamily	-
Q#176 - >Ca_02004.1|Ca_	superfamily	259620	36	72	0.00884933	33.1999	cl21304	CTC1 superfamily	-
Q#177 - >Ca_02007.1|Ca_	specific	144416	1	138	2.76511e-32	114.964	pfam00810	ER_lumen_recept	cl02155
Q#177 - >Ca_02007.1|Ca_	superfamily	242908	1	138	2.76511e-32	114.964	cl02155	ER_lumen_recept superfamily	-
Q#178 - >Ca_02035.1|Ca_	specific	249964	3	100	6.22436e-23	96.2847	pfam00566	RabGAP-TBC	cl02495
Q#178 - >Ca_02035.1|Ca_	superfamily	261308	3	100	6.22436e-23	96.2847	cl02495	RabGAP-TBC superfamily	-
Q#178 - >Ca_02035.1|Ca_	superfamily	115072	315	422	0.00800319	36.2785	cl21644	MAT1 superfamily	-
Q#181 - >Ca_02118.1|Ca_	specific	260004	186	322	8.69332e-69	214.254	cd09272	RNase_HI_RT_Ty1	cl14782
Q#181 - >Ca_02118.1|Ca_	superfamily	271754	186	322	8.69332e-69	214.254	cl14782	RNase_H_like superfamily	-
Q#184 - >Ca_02136.1|Ca_	specific	190559	8	108	1.09202e-53	165.828	pfam03195	DUF260	cl03929
Q#184 - >Ca_02136.1|Ca_	superfamily	190559	8	108	1.09202e-53	165.828	cl03929	DUF260 superfamily	-
Q#185 - >Ca_02139.1|Ca_	superfamily	276194	90	289	2.67608e-128	377.815	cl17173	AdoMet_MTases superfamily	-
Q#186 - >Ca_02162.1|Ca_	superfamily	276061	38	187	1.49548e-39	133.927	cl14783	DOMON_like superfamily	-
Q#187 - >Ca_02178.1|Ca_	superfamily	275812	20	67	9.97828e-13	58.9494	cl04571	MARVEL superfamily	-
Q#188 - >Ca_02221.1|Ca_	superfamily	275467	1	46	4.35216e-10	51.4576	cl00074	H2A superfamily	-
Q#190 - >Ca_02234.1|Ca_	superfamily	275882	21	91	0.00761723	33.1361	cl09782	Cas6-I-III superfamily	-
Q#191 - >Ca_02256.1|Ca_	superfamily	276263	125	282	2.10369e-13	66.9992	cl19514	FBA_1 superfamily	-

Query	Types	PSSM-ID	From	To	E-Value	Bitscore	Accession	Short name	Superfamily
Q#194 - >Ca_02272.1|Ca_	superfamily	276192	83	116	1.74152e-08	48.2992	cl17169	RRM_SF superfamily	-
Q#195 - >Ca_02366.1|Ca_	specific	132916	171	287	3.44353e-47	157.218	cd07033	TPP_PYR_DXS_TK_like	cl11410
Q#195 - >Ca_02366.1|Ca_	superfamily	264357	171	287	3.44353e-47	157.218	cl11410	TPP_enzyme_PYR superfamily	-
Q#195 - >Ca_02366.1|Ca_	superfamily	275737	74	127	2.21601e-20	86.0612	cl01629	TPP_enzymes superfamily	-
Q#196 - >Ca_02404.1|Ca_	specific	132921	26	185	8.46931e-92	275.527	cd07038	TPP_PYR_PDC_IPDC_like	cl11410
Q#196 - >Ca_02404.1|Ca_	superfamily	264357	26	185	8.46931e-92	275.527	cl11410	TPP_enzyme_PYR superfamily	-
Q#196 - >Ca_02404.1|Ca_	superfamily	275737	248	376	4.23793e-49	165.785	cl01629	TPP_enzymes superfamily	-
Q#196 - >Ca_02404.1|Ca_	multi-dom	215311	8	393	0	783.889	PLN02573	PLN02573	-
Q#198 - >Ca_02460.1|Ca_	specific	238204	27	236	3.13497e-62	197.476	cd00333	MIP	cl00200
Q#198 - >Ca_02460.1|Ca_	superfamily	275482	27	236	3.13497e-62	197.476	cl00200	MIP superfamily	-
Q#199 - >Ca_02461.1|Ca_	specific	238204	27	236	3.45614e-63	200.172	cd00333	MIP	cl00200
Q#199 - >Ca_02461.1|Ca_	superfamily	275482	27	236	3.45614e-63	200.172	cl00200	MIP superfamily	-
Q#201 - >Ca_02608.1|Ca_	superfamily	275927	86	344	1.02927e-33	126.609	cl11403	pepsin_retropepsin_like superfamily	-
Q#201 - >Ca_02608.1|Ca_	multi-dom	249520	86	338	1.09725e-11	63.8475	pfam00026	Asp	-
Q#202 - >Ca_02620.1|Ca_	specific	240181	27	163	1.75424e-63	193.618	cd05381	SCP_PR-1_like	cl00133
Q#202 - >Ca_02620.1|Ca_	superfamily	260208	27	163	1.75424e-63	193.618	cl00133	SCP superfamily	-
Q#203 - >Ca_02622.1|Ca_	specific	240181	11	147	2.71049e-60	184.759	cd05381	SCP_PR-1_like	cl00133
Q#203 - >Ca_02622.1|Ca_	superfamily	260208	11	147	2.71049e-60	184.759	cl00133	SCP superfamily	-
Q#204 - >Ca_02746.1|Ca_	superfamily	271550	39	139	0.00323566	35.2459	cl01255	DAGK_cat superfamily	-
Q#205 - >Ca_02766.1|Ca_	superfamily	263558	2	330	8.7678e-138	400.937	cl08270	Peptidase_S10 superfamily	-
Q#206 - >Ca_02768.1|Ca_	superfamily	263558	3	175	3.63525e-63	199.562	cl08270	Peptidase_S10 superfamily	-
Q#208 - >Ca_02770.1|Ca_	multi-dom	185628	357	551	1.82494e-05	46.6066	PTZ00449	PTZ00449	-
Q#209 - >Ca_02774.1|Ca_	superfamily	110290	35	121	3.53782e-10	53.1185	cl03145	Oleosin superfamily	-
Q#210 - >Ca_02790.1|Ca_	superfamily	252826	44	208	5.35808e-68	216.064	cl04787	COBRA superfamily	-
Q#211 - >Ca_02818.1|Ca_	specific	251775	156	227	2.63437e-15	69.3607	pfam03171	2OG-FeII_Oxy	cl21496
Q#211 - >Ca_02818.1|Ca_	superfamily	276328	156	227	2.63437e-15	69.3607	cl21496	2OG-FeII_Oxy superfamily	-
Q#211 - >Ca_02818.1|Ca_	specific	258418	16	88	5.6406e-14	66.1098	pfam14226	DIOX_N	cl21672
Q#211 - >Ca_02818.1|Ca_	superfamily	272092	16	88	5.6406e-14	66.1098	cl21672	DIOX_N superfamily	-
Q#212 - >Ca_02862.1|Ca_	specific	237980	43	106	2.54915e-13	61.6739	cd00010	AAI_LTSS	cl07890
Q#212 - >Ca_02862.1|Ca_	superfamily	275845	43	106	2.54915e-13	61.6739	cl07890	AAI_LTSS superfamily	-
Q#219 - >Ca_02908.1|Ca_	multi-dom	227217	8	74	0.00611752	33.7394	COG4880	COG4880	-
Q#220 - >Ca_02910.1|Ca_	superfamily	276663	126	205	1.74801e-08	51.5592	cl22709	AMN1 superfamily	-
Q#220 - >Ca_02910.1|Ca_	specific	257418	45	74	0.00176702	34.7953	pfam12937	F-box-like	cl02535
Q#220 - >Ca_02910.1|Ca_	superfamily	261329	45	74	0.00176702	34.7953	cl02535	F-box superfamily	-
Q#222 - >Ca_02930.1|Ca_	superfamily	276299	88	158	0.00296971	35.9699	cl21455	ABC_ATPase superfamily	-
Q#223 - >Ca_02937.1|Ca_	superfamily	254387	171	310	7.86555e-71	222.332	cl06662	RVT_2 superfamily	-

<table>
<tr><th>Query</th><th>Types</th><th>PSSM-ID</th><th>From</th><th>To</th><th>E-Value</th><th>Bitscore</th><th>Accession</th><th>Short name</th><th>Superfamily</th></tr>
<tr><td>Q#225 - >Ca_02944.1|Ca_</td><td>superfamily</td><td>252133</td><td>100</td><td>176</td><td>2.27113e-11</td><td>56.9633</td><td>cl04237</td><td>Retrotrans_gag superfamily</td><td>-</td></tr>
<tr><td>Q#229 - >Ca_02979.1|Ca_</td><td>specific</td><td>250854</td><td>85</td><td>129</td><td>1.42736e-17</td><td>73.0006</td><td>pfam01775</td><td>Ribosomal_L18ae</td><td>cl00945</td></tr>
<tr><td>Q#229 - >Ca_02979.1|Ca_</td><td>superfamily</td><td>260714</td><td>85</td><td>129</td><td>1.42736e-17</td><td>73.0006</td><td>cl00945</td><td>Ribosomal_L18ae superfamily</td><td>-</td></tr>
<tr><td>Q#231 - >Ca_02997.1|Ca_</td><td>specific</td><td>238219</td><td>17</td><td>70</td><td>5.82926e-06</td><td>41.8225</td><td>cd00371</td><td>HMA</td><td>cl00207</td></tr>
<tr><td>Q#231 - >Ca_02997.1|Ca_</td><td>superfamily</td><td>260261</td><td>17</td><td>70</td><td>5.82926e-06</td><td>41.8225</td><td>cl00207</td><td>HMA superfamily</td><td>-</td></tr>
<tr><td>Q#232 - >Ca_03082.1|Ca_</td><td>specific</td><td>198296</td><td>90</td><td>208</td><td>3.48308e-59</td><td>184.353</td><td>cd03187</td><td>GST_C_Phi</td><td>cl02776</td></tr>
<tr><td>Q#232 - >Ca_03082.1|Ca_</td><td>superfamily</td><td>275780</td><td>90</td><td>208</td><td>3.48308e-59</td><td>184.353</td><td>cl02776</td><td>GST_C_family superfamily</td><td>-</td></tr>
<tr><td>Q#232 - >Ca_03082.1|Ca_</td><td>specific</td><td>239351</td><td>2</td><td>76</td><td>2.01536e-35</td><td>121.604</td><td>cd03053</td><td>GST_N_Phi</td><td>cl00388</td></tr>
<tr><td>Q#232 - >Ca_03082.1|Ca_</td><td>superfamily</td><td>275563</td><td>2</td><td>76</td><td>2.01536e-35</td><td>121.604</td><td>cl00388</td><td>Thioredoxin_like superfamily</td><td>-</td></tr>
<tr><td>Q#232 - >Ca_03082.1|Ca_</td><td>multi-dom</td><td>166036</td><td>1</td><td>215</td><td>2.45299e-129</td><td>366.496</td><td>PLN02395</td><td>PLN02395</td><td>-</td></tr>
<tr><td>Q#234 - >Ca_03112.1|Ca_</td><td>superfamily</td><td>276020</td><td>1</td><td>200</td><td>2.98926e-56</td><td>187.711</td><td>cl12078</td><td>p450 superfamily</td><td>-</td></tr>
<tr><td>Q#235 - >Ca_03152.1|Ca_</td><td>specific</td><td>240513</td><td>52</td><td>105</td><td>2.40618e-14</td><td>63.6878</td><td>cd06089</td><td>KOW_RPL26</td><td>cl00354</td></tr>
<tr><td>Q#235 - >Ca_03152.1|Ca_</td><td>superfamily</td><td>275547</td><td>52</td><td>105</td><td>2.40618e-14</td><td>63.6878</td><td>cl00354</td><td>KOW superfamily</td><td>-</td></tr>
<tr><td>Q#236 - >Ca_03178.1|Ca_</td><td>multi-dom</td><td>215561</td><td>3</td><td>82</td><td>1.50227e-17</td><td>76.4269</td><td>PLN03077</td><td>PLN03077</td><td>-</td></tr>
<tr><td>Q#238 - >Ca_03261.1|Ca_</td><td>specific</td><td>211390</td><td>79</td><td>198</td><td>1.66872e-12</td><td>62.2178</td><td>cd11378</td><td>DUF296</td><td>cl00720</td></tr>
<tr><td>Q#238 - >Ca_03261.1|Ca_</td><td>superfamily</td><td>260588</td><td>79</td><td>198</td><td>1.66872e-12</td><td>62.2178</td><td>cl00720</td><td>DUF296 superfamily</td><td>-</td></tr>
<tr><td>Q#239 - >Ca_03262.1|Ca_</td><td>superfamily</td><td>254980</td><td>72</td><td>141</td><td>2.11295e-16</td><td>70.8074</td><td>cl07355</td><td>Fcf2 superfamily</td><td>-</td></tr>
<tr><td>Q#240 - >Ca_03273.1|Ca_</td><td>superfamily</td><td>259385</td><td>44</td><td>170</td><td>0.00124475</td><td>37.5705</td><td>cl21110</td><td>DUF4588 superfamily</td><td>-</td></tr>
<tr><td>Q#241 - >Ca_03287.1|Ca_</td><td>specific</td><td>238008</td><td>85</td><td>147</td><td>4.46755e-24</td><td>93.3813</td><td>cd00051</td><td>EFh</td><td>cl08302</td></tr>
<tr><td>Q#241 - >Ca_03287.1|Ca_</td><td>superfamily</td><td>275859</td><td>85</td><td>147</td><td>4.46755e-24</td><td>93.3813</td><td>cl08302</td><td>EFh superfamily</td><td>-</td></tr>
<tr><td>Q#241 - >Ca_03287.1|Ca_</td><td>specific</td><td>238008</td><td>12</td><td>74</td><td>3.17179e-22</td><td>88.3737</td><td>cd00051</td><td>EFh</td><td>cl08302</td></tr>
<tr><td>Q#241 - >Ca_03287.1|Ca_</td><td>superfamily</td><td>275859</td><td>12</td><td>74</td><td>3.17179e-22</td><td>88.3737</td><td>cl08302</td><td>EFh superfamily</td><td>-</td></tr>
<tr><td>Q#241 - >Ca_03287.1|Ca_</td><td>specific</td><td>250134</td><td>275</td><td>353</td><td>3.02786e-17</td><td>75.3939</td><td>pfam00789</td><td>UBX</td><td>cl00155</td></tr>
<tr><td>Q#241 - >Ca_03287.1|Ca_</td><td>superfamily</td><td>275476</td><td>275</td><td>353</td><td>3.02786e-17</td><td>75.3939</td><td>cl00155</td><td>UBQ superfamily</td><td>-</td></tr>
<tr><td>Q#241 - >Ca_03287.1|Ca_</td><td>multi-dom</td><td>185504</td><td>1</td><td>149</td><td>1.2763e-98</td><td>291.28</td><td>PTZ00184</td><td>PTZ00184</td><td>-</td></tr>
<tr><td>Q#242 - >Ca_03313.1|Ca_</td><td>specific</td><td>240687</td><td>17</td><td>93</td><td>1.48453e-51</td><td>159.704</td><td>cd12241</td><td>RRM_SF3B14</td><td>cl17169</td></tr>
<tr><td>Q#242 - >Ca_03313.1|Ca_</td><td>superfamily</td><td>276192</td><td>17</td><td>93</td><td>1.48453e-51</td><td>159.704</td><td>cl17169</td><td>RRM_SF superfamily</td><td>-</td></tr>
<tr><td>Q#242 - >Ca_03313.1|Ca_</td><td>multi-dom</td><td>214636</td><td>20</td><td>89</td><td>3.40965e-19</td><td>76.4783</td><td>smart00360</td><td>RRM</td><td>-</td></tr>
<tr><td>Q#243 - >Ca_03323.1|Ca_</td><td>superfamily</td><td>261191</td><td>7</td><td>129</td><td>2.22325e-16</td><td>72.8974</td><td>cl02130</td><td>Got1 superfamily</td><td>-</td></tr>
<tr><td>Q#243 - >Ca_03323.1|Ca_</td><td>superfamily</td><td>261351</td><td>222</td><td>255</td><td>0.00459529</td><td>35.6166</td><td>cl02570</td><td>RhoGAP superfamily</td><td>-</td></tr>
<tr><td>Q#244 - >Ca_03324.1|Ca_</td><td>specific</td><td>133136</td><td>4</td><td>51</td><td>1.02778e-06</td><td>42.7088</td><td>cd00303</td><td>retropepsin_like</td><td>cl11403</td></tr>
<tr><td>Q#244 - >Ca_03324.1|Ca_</td><td>superfamily</td><td>275927</td><td>4</td><td>51</td><td>1.02778e-06</td><td>42.7088</td><td>cl11403</td><td>pepsin_retropepsin_like superfamily</td><td>-</td></tr>
<tr><td>Q#245 - >Ca_03338.1|Ca_</td><td>specific</td><td>189387</td><td>375</td><td>388</td><td>0.00827183</td><td>34.7373</td><td>pfam00098</td><td>zf-CCHC</td><td>cl22700</td></tr>
<tr><td>Q#245 - >Ca_03338.1|Ca_</td><td>superfamily</td><td>276654</td><td>375</td><td>388</td><td>0.00827183</td><td>34.7373</td><td>cl22700</td><td>zf-CCHC superfamily</td><td>-</td></tr>
<tr><td>Q#245 - >Ca_03338.1|Ca_</td><td>specific</td><td>189387</td><td>411</td><td>424</td><td>0.00953202</td><td>34.3521</td><td>pfam00098</td><td>zf-CCHC</td><td>cl22700</td></tr>
<tr><td>Q#245 - >Ca_03338.1|Ca_</td><td>superfamily</td><td>276654</td><td>411</td><td>424</td><td>0.00953202</td><td>34.3521</td><td>cl22700</td><td>zf-CCHC superfamily</td><td>-</td></tr>
</table>

Query	Types	PSSM-ID	From	To	E-Value	Bitscore	Accession	Short name	Superfamily
Q#245 - >Ca_03338.1|Ca_	multi-dom	173561	264	427	3.75547e-23	96.0292	PTZ00368	PTZ00368	-
Q#252 - >Ca_03384.1|Ca_	specific	176359	13	99	1.11433e-50	157.009	cd01763	Sumo	cl00155
Q#252 - >Ca_03384.1|Ca_	superfamily	275476	13	99	1.11433e-50	157.009	cl00155	UBQ superfamily	-
Q#255 - >Ca_03458.1|Ca_	multi-dom	178439	31	157	0.00775447	35.6217	PLN02847	PLN02847	-
Q#256 - >Ca_03459.1|Ca_	superfamily	276192	11	49	2.61367e-19	74.6367	cl17169	RRM_SF superfamily	-
Q#257 - >Ca_03460.1|Ca_	specific	257367	39	61	0.000266024	35.1748	pfam12874	zf-met	cl22457
Q#257 - >Ca_03460.1|Ca_	superfamily	276411	39	61	0.000266024	35.1748	cl22457	zf-C2H2_jaz superfamily	-
Q#258 - >Ca_03493.1|Ca_	specific	252801	138	233	1.606e-37	132.703	pfam04788	DUF620	cl04752
Q#258 - >Ca_03493.1|Ca_	superfamily	252801	138	233	1.606e-37	132.703	cl04752	DUF620 superfamily	-
Q#259 - >Ca_03495.1|Ca_	multi-dom	224495	28	85	0.00199829	34.6487	COG1579	COG1579	-
Q#260 - >Ca_03496.1|Ca_	multi-dom	215130	132	251	0.000672595	39.6872	PLN02217	PLN02217	-
Q#261 - >Ca_03504.1|Ca_	superfamily	275586	21	98	1.19527e-05	42.1615	cl00456	SLC5-6-like_sbd superfamily	-
Q#262 - >Ca_03507.1|Ca_	specific	253025	96	158	2.049e-24	96.1074	pfam05127	Helicase_RecD	cl21455
Q#262 - >Ca_03507.1|Ca_	superfamily	276299	96	158	2.049e-24	96.1074	cl21455	ABC_ATPase superfamily	-
Q#262 - >Ca_03507.1|Ca_	superfamily	276297	32	73	1.02481e-08	53.3834	cl21453	PKc_like superfamily	-
Q#263 - >Ca_03524.1|Ca_	multi-dom	258434	80	138	2.69333e-16	70.4598	pfam14244	UBN2_3	-
Q#264 - >Ca_03533.1|Ca_	specific	238037	201	254	0.000116615	39.1392	cd00084	HMG-box	cl00082
Q#264 - >Ca_03533.1|Ca_	superfamily	260179	201	254	0.000116615	39.1392	cl00082	HMG-box superfamily	-
Q#264 - >Ca_03533.1|Ca_	superfamily	271573	156	220	5.20013e-12	62.1836	cl02008	2-oxoacid_dh superfamily	-
Q#266 - >Ca_03541.1|Ca_	superfamily	190526	214	250	4.35286e-08	48.753	cl07846	DBD_Tnp_Mut superfamily	-
Q#268 - >Ca_03575.1|Ca_	superfamily	192059	83	143	2.7432e-20	81.8381	cl07235	Whirly superfamily	-
Q#269 - >Ca_03587.1|Ca_	specific	238096	91	136	1.42939e-09	52.1926	cd00167	SANT	cl21498
Q#269 - >Ca_03587.1|Ca_	superfamily	271918	91	136	1.42939e-09	52.1926	cl21498	SANT superfamily	-
Q#271 - >Ca_03670.1|Ca_	specific	214764	139	247	3.73387e-67	206.329	smart00653	eIF2B_5	cl17014
Q#271 - >Ca_03670.1|Ca_	superfamily	266499	139	247	3.73387e-67	206.329	cl17014	eIF-5_eIF-2B superfamily	-
Q#273 - >Ca_03706.1|Ca_	specific	258207	34	107	7.49195e-27	98.0371	pfam13952	DUF4216	cl16495
Q#273 - >Ca_03706.1|Ca_	superfamily	258207	34	107	7.49195e-27	98.0371	cl16495	DUF4216 superfamily	-
Q#274 - >Ca_03722.1|Ca_	specific	211390	73	189	1.15713e-14	67.9958	cd11378	DUF296	cl00720
Q#274 - >Ca_03722.1|Ca_	superfamily	260588	73	189	1.15713e-14	67.9958	cl00720	DUF296 superfamily	-
Q#276 - >Ca_03741.1|Ca_	superfamily	276341	2	140	3.71253e-05	43.7675	cl21520	Asp-Al_Ex superfamily	-
Q#276 - >Ca_03741.1|Ca_	multi-dom	215608	3	348	3.1721e-49	177.004	PLN03159	PLN03159	-
Q#277 - >Ca_03749.1|Ca_	specific	100107	85	184	0.000989257	36.6391	cd02440	AdoMet_MTases	cl17173
Q#277 - >Ca_03749.1|Ca_	superfamily	276194	85	184	0.000989257	36.6391	cl17173	AdoMet_MTases superfamily	-
Q#278 - >Ca_03751.1|Ca_	superfamily	275474	3	66	4.83291e-14	66.4829	cl00130	PseudoU_synth superfamily	-
Q#280 - >Ca_03773.1|Ca_	specific	251140	20	77	7.85135e-10	51.0679	pfam02179	BAG	cl02539
Q#280 - >Ca_03773.1|Ca_	superfamily	261331	20	77	7.85135e-10	51.0679	cl02539	BAG superfamily	-

Query	Types	PSSM-ID	From	To	E-Value	Bitscore	Accession	Short name	Superfamily
Q#281 - >Ca_03779.1|Ca_	specific	237987	4	76	1.54642e-08	51.1611	cd00020	ARM	cl22454
Q#281 - >Ca_03779.1|Ca_	superfamily	276408	4	76	1.54642e-08	51.1611	cl22454	ARM superfamily	-
Q#283 - >Ca_03792.1|Ca_	specific	211390	24	105	1.39098e-13	63.3734	cd11378	DUF296	cl00720
Q#283 - >Ca_03792.1|Ca_	superfamily	260588	24	105	1.39098e-13	63.3734	cl00720	DUF296 superfamily	-
Q#285 - >Ca_03801.1|Ca_	superfamily	251718	396	618	1.24476e-18	86.626	cl03878	Exo70 superfamily	-
Q#286 - >Ca_03807.1|Ca_	specific	211390	85	190	3.16276e-18	77.2406	cd11378	DUF296	cl00720
Q#286 - >Ca_03807.1|Ca_	superfamily	260588	85	190	3.16276e-18	77.2406	cl00720	DUF296 superfamily	-
Q#289 - >Ca_03858.1|Ca_	multi-dom	237863	14	156	0.000132533	42.4048	PRK14949	PRK14949	-
Q#290 - >Ca_03876.1|Ca_	multi-dom	258434	40	108	5.10718e-22	88.179	pfam14244	UBN2_3	-
Q#291 - >Ca_03895.1|Ca_	specific	238205	15	151	7.55915e-34	117.207	cd00336	Ribosomal_L22	cl00327
Q#291 - >Ca_03895.1|Ca_	superfamily	275534	15	151	7.55915e-34	117.207	cl00327	Ribosomal_L22 superfamily	-
Q#292 - >Ca_03903.1|Ca_	specific	260006	96	215	1.99935e-26	101.415	cd09274	RNase_HI_RT_Ty3	cl14782
Q#292 - >Ca_03903.1|Ca_	superfamily	271754	96	215	1.99935e-26	101.415	cl14782	RNase_H_like superfamily	-
Q#294 - >Ca_03914.1|Ca_	superfamily	275485	63	132	0.00168603	39.4857	cl00210	Isoprenoid_Biosyn_C1 superfamily	-
Q#296 - >Ca_03960.1|Ca_	specific	173887	30	254	7.25024e-79	240.209	cd06899	lectin_legume_LecRK_Arcelin_ConA	cl14058
Q#296 - >Ca_03960.1|Ca_	superfamily	265423	30	254	7.25024e-79	240.209	cl14058	lectin_L-type superfamily	-
Q#297 - >Ca_03963.1|Ca_	superfamily	271701	83	154	7.77452e-24	97.6688	cl10459	Peptidases_S8_S53 superfamily	-
Q#297 - >Ca_03963.1|Ca_	superfamily	271701	16	64	9.58191e-05	40.0895	cl10459	Peptidases_S8_S53 superfamily	-
Q#298 - >Ca_03966.1|Ca_	specific	133136	60	147	3.0747e-16	72.3692	cd00303	retropepsin_like	cl11403
Q#298 - >Ca_03966.1|Ca_	superfamily	275927	60	147	3.0747e-16	72.3692	cl11403	pepsin_retropepsin_like superfamily	-
Q#298 - >Ca_03966.1|Ca_	superfamily	261455	267	314	1.16903e-16	75.3245	cl02808	RT_like superfamily	-
Q#300 - >Ca_03976.1|Ca_	superfamily	263558	9	93	1.30965e-13	66.1395	cl08270	Peptidase_S10 superfamily	-
Q#304 - >Ca_04011.1|Ca_	superfamily	276297	11	224	8.61995e-84	254.67	cl21453	PKc_like superfamily	-
Q#306 - >Ca_04018.1|Ca_	superfamily	275927	125	195	0.00682176	33.464	cl11403	pepsin_retropepsin_like superfamily	-
Q#307 - >Ca_04022.1|Ca_	specific	153145	200	272	1.26182e-06	45.2317	cd04873	ACT_UUR-ACR-like	cl09141
Q#307 - >Ca_04022.1|Ca_	superfamily	275868	200	272	1.26182e-06	45.2317	cl09141	ACT superfamily	-
Q#307 - >Ca_04022.1|Ca_	specific	238036	127	166	0.000181826	38.7343	cd00083	HLH	cl00081
Q#307 - >Ca_04022.1|Ca_	superfamily	260178	127	166	0.000181826	38.7343	cl00081	HLH superfamily	-
Q#308 - >Ca_04026.1|Ca_	specific	238825	370	499	3.76417e-54	183.18	cd01647	RT_LTR	cl02808
Q#308 - >Ca_04026.1|Ca_	superfamily	261455	370	499	3.76417e-54	183.18	cl02808	RT_like superfamily	-
Q#308 - >Ca_04026.1|Ca_	multi-dom	249567	386	499	6.02694e-14	70.0522	pfam00078	RVT_1	-
Q#310 - >Ca_04045.1|Ca_	superfamily	251669	10	58	0.00193334	35.4681	cl03830	Transposase_24 superfamily	-
Q#314 - >Ca_04074.1|Ca_	superfamily	256046	89	281	2.99408e-22	94.7688	cl11168	PMD superfamily	-
Q#316 - >Ca_04081.1|Ca_	superfamily	276310	25	60	3.39217e-11	54.8779	cl21469	HDc superfamily	-
Q#321 - >Ca_04097.1|Ca_	specific	273253	31	64	0.00259782	32.8123	TIGR00756	PPR	cl03252
Q#321 - >Ca_04097.1|Ca_	superfamily	275791	31	64	0.00259782	32.8123	cl03252	PPR superfamily	-

Query	Types	PSSM-ID	From	To	E-Value	Bitscore	Accession	Short name	Superfamily
Q#321 - >Ca_04097.1|Ca_	multi-dom	257477	28	77	5.80521e-11	53.9458	pfam13041	PPR_2	-
Q#321 - >Ca_04097.1|Ca_	multi-dom	257477	63	100	0.00775265	31.6042	pfam13041	PPR_2	-
Q#328 - >Ca_04114.1|Ca_	superfamily	256046	61	102	0.00957513	33.522	cl11168	PMD superfamily	-
Q#330 - >Ca_04124.1|Ca_	superfamily	275485	63	132	0.00700945	37.5597	cl00210	Isoprenoid_Biosyn_C1 superfamily	-
Q#331 - >Ca_04125.1|Ca_	superfamily	252133	129	199	8.95095e-07	45.0221	cl04237	Retrotrans_gag superfamily	-
Q#335 - >Ca_04149.1|Ca_	superfamily	256495	1	28	6.40079e-08	44.5787	cl13039	DUF3223 superfamily	-
Q#337 - >Ca_04159.1|Ca_	superfamily	267728	131	179	0.000521226	39.309	cl19375	HemX superfamily	-
Q#338 - >Ca_04168.1|Ca_	multi-dom	258434	1	75	1.78423e-07	47.733	pfam14244	UBN2_3	-
Q#340 - >Ca_04173.1|Ca_	superfamily	256046	86	189	3.60126e-06	46.2336	cl11168	PMD superfamily	-
Q#341 - >Ca_04200.1|Ca_	specific	249868	31	405	4.84448e-175	497.948	pfam00450	Peptidase_S10	cl08270
Q#341 - >Ca_04200.1|Ca_	superfamily	263558	31	405	4.84448e-175	497.948	cl08270	Peptidase_S10 superfamily	-
Q#342 - >Ca_04210.1|Ca_	specific	189387	248	263	0.000363656	37.4337	pfam00098	zf-CCHC	cl22700
Q#342 - >Ca_04210.1|Ca_	superfamily	276654	248	263	0.000363656	37.4337	cl22700	zf-CCHC superfamily	-
Q#343 - >Ca_04211.1|Ca_	superfamily	256046	82	191	1.1651e-14	73.1976	cl11168	PMD superfamily	-
Q#344 - >Ca_04216.1|Ca_	specific	133136	229	317	2.76246e-16	72.7544	cd00303	retropepsin_like	cl11403
Q#344 - >Ca_04216.1|Ca_	superfamily	275927	229	317	2.76246e-16	72.7544	cl11403	pepsin_retropepsin_like superfamily	-
Q#344 - >Ca_04216.1|Ca_	superfamily	252133	10	80	2.57705e-10	56.5781	cl04237	Retrotrans_gag superfamily	-
Q#344 - >Ca_04216.1|Ca_	multi-dom	259374	119	176	0.00021955	39.941	pfam15240	Pro-rich	-
Q#345 - >Ca_04222.1|Ca_	superfamily	203599	312	450	0.0034059	38.6694	cl06322	Phytoreo_S7 superfamily	-
Q#348 - >Ca_04241.1|Ca_	superfamily	275905	54	83	3.46873e-14	65.7487	cl09928	Molybdopterin-Binding superfamily	-
Q#350 - >Ca_04248.1|Ca_	superfamily	256046	39	247	5.58179e-34	126.355	cl11168	PMD superfamily	-
Q#351 - >Ca_04263.1|Ca_	specific	240537	2	306	3.26983e-110	332.23	cd13132	MATE_eukaryotic	cl09326
Q#351 - >Ca_04263.1|Ca_	superfamily	271660	2	306	3.26983e-110	332.23	cl09326	MATE_like superfamily	-
Q#351 - >Ca_04263.1|Ca_	multi-dom	273273	1	221	2.92267e-18	83.4484	TIGR00797	Multidrug_resistance_protein_HmrM	-
Q#352 - >Ca_04275.1|Ca_	superfamily	251669	184	235	2.98831e-05	41.2461	cl03830	Transposase_24 superfamily	-
Q#353 - >Ca_04288.1|Ca_	specific	203765	153	202	2.50362e-17	73.1122	pfam07797	DUF1639	cl06707
Q#353 - >Ca_04288.1|Ca_	superfamily	203765	153	202	2.50362e-17	73.1122	cl06707	DUF1639 superfamily	-
Q#354 - >Ca_04290.1|Ca_	superfamily	259584	28	183	0.00975406	37.0454	cl21271	Pilt superfamily	-
Q#359 - >Ca_04312.1|Ca_	superfamily	276020	86	297	4.83887e-73	235.287	cl12078	p450 superfamily	-
Q#360 - >Ca_04360.1|Ca_	superfamily	268118	4	194	1.18478e-35	129.492	cl19765	mTERF superfamily	-
Q#367 - >Ca_04391.1|Ca_	specific	178710	1	96	1.29611e-54	168.184	PLN03166	PLN03166	cl00955
Q#367 - >Ca_04391.1|Ca_	superfamily	242219	1	96	1.29611e-54	168.184	cl00955	Ribosomal_L34e superfamily	-
Q#368 - >Ca_04406.1|Ca_	specific	251251	14	142	4.00388e-74	220.539	pfam02365	NAM	cl03558
Q#368 - >Ca_04406.1|Ca_	superfamily	251251	14	142	4.00388e-74	220.539	cl03558	NAM superfamily	-
Q#369 - >Ca_04414.1|Ca_	superfamily	276412	1	144	1.1099e-60	189.257	cl22458	RICIN superfamily	-
Q#371 - >Ca_04432.1|Ca_	superfamily	276299	78	152	2.27316e-17	75.2137	cl21455	ABC_ATPase superfamily	-

Query	Types	PSSM-ID	From	To	E-Value	Bitscore	Accession	Short name	Superfamily
Q#371 - >Ca_04432.1|Ca_	superfamily	271701	4	47	4.90551e-09	50.416	cl10459	Peptidases_S8_S53 superfamily	-
Q#372 - >Ca_04433.1|Ca_	superfamily	276313	27	161	2.67079e-19	83.1318	cl21472	MFS superfamily	-
Q#374 - >Ca_04478.1|Ca_	specific	99960	156	273	1.06423e-27	109.76	cd03784	GT1_Gtf_like	cl10013
Q#374 - >Ca_04478.1|Ca_	superfamily	275912	156	273	1.06423e-27	109.76	cl10013	Glycosyltransferase_GTB_type superfamily	-
Q#375 - >Ca_04510.1|Ca_	specific	258114	55	215	5.88352e-41	142.579	pfam13839	PC-Esterase	cl01053
Q#375 - >Ca_04510.1|Ca_	superfamily	260768	55	215	5.88352e-41	142.579	cl01053	SGNH_hydrolase superfamily	-
Q#376 - >Ca_04511.1|Ca_	specific	258114	50	167	1.53289e-33	121.008	pfam13839	PC-Esterase	cl01053
Q#376 - >Ca_04511.1|Ca_	superfamily	260768	50	167	1.53289e-33	121.008	cl01053	SGNH_hydrolase superfamily	-
Q#377 - >Ca_04514.1|Ca_	specific	258114	1	152	4.48017e-40	137.572	pfam13839	PC-Esterase	cl01053
Q#377 - >Ca_04514.1|Ca_	superfamily	260768	1	152	4.48017e-40	137.572	cl01053	SGNH_hydrolase superfamily	-
Q#378 - >Ca_04532.1|Ca_	superfamily	263970	14	253	2.01196e-40	142.125	cl10447	GH18_chitinase-like superfamily	-
Q#378 - >Ca_04532.1|Ca_	multi-dom	250070	19	202	3.82451e-07	49.0405	pfam00704	Glyco_hydro_18	-
Q#380 - >Ca_04585.1|Ca_	superfamily	271660	36	295	1.73911e-64	211.662	cl09326	MATE_like superfamily	-
Q#381 - >Ca_04605.1|Ca_	superfamily	254466	2	104	2.67012e-40	133.989	cl06746	DUF1637 superfamily	-
Q#382 - >Ca_04614.1|Ca_	multi-dom	224557	25	176	1.5597e-34	129.362	COG1643	HrpA	-
Q#391 - >Ca_04658.1|Ca_	specific	238825	89	188	4.83243e-29	111.533	cd01647	RT_LTR	cl02808
Q#391 - >Ca_04658.1|Ca_	superfamily	261455	89	188	4.83243e-29	111.533	cl02808	RT_like superfamily	-
Q#392 - >Ca_04665.1|Ca_	specific	238230	26	139	5.79839e-53	167.682	cd00392	Ribosomal_L13	cl00333
Q#392 - >Ca_04665.1|Ca_	superfamily	260361	26	139	5.79839e-53	167.682	cl00333	Ribosomal_L13 superfamily	-
Q#393 - >Ca_04690.1|Ca_	superfamily	256046	5	106	4.50424e-20	82.4424	cl11168	PMD superfamily	-
Q#395 - >Ca_04717.1|Ca_	superfamily	256028	19	41	0.00175775	36.2447	cl20401	zf-4CXXC_R1 superfamily	-
Q#395 - >Ca_04717.1|Ca_	superfamily	243137	131	190	0.00202406	35.6862	cl02674	DDT superfamily	-
Q#398 - >Ca_04751.1|Ca_	superfamily	271848	27	362	2.26977e-27	110.554	cl19241	Condensation superfamily	-
Q#399 - >Ca_04773.1|Ca_	superfamily	191657	109	207	4.96244e-15	69.2657	cl06155	DUF1298 superfamily	-
Q#406 - >Ca_04800.1|Ca_	specific	260004	86	158	4.14928e-29	112.562	cd09272	RNase_HI_RT_Ty1	cl14782
Q#406 - >Ca_04800.1|Ca_	superfamily	271754	86	158	4.14928e-29	112.562	cl14782	RNase_H_like superfamily	-
Q#407 - >Ca_04818.1|Ca_	superfamily	252133	100	199	1.75404e-12	61.5857	cl04237	Retrotrans_gag superfamily	-
Q#409 - >Ca_04827.1|Ca_	superfamily	256046	16	80	8.5177e-06	42.7668	cl11168	PMD superfamily	-
Q#410 - >Ca_04832.1|Ca_	superfamily	275799	61	133	1.74706e-42	141.042	cl04011	DPBB_1 superfamily	-
Q#410 - >Ca_04832.1|Ca_	specific	250557	149	226	4.35897e-34	119.211	pfam01357	Pollen_allerg_1	cl08320
Q#410 - >Ca_04832.1|Ca_	superfamily	263589	149	226	4.35897e-34	119.211	cl08320	Pollen_allerg_1 superfamily	-
Q#410 - >Ca_04832.1|Ca_	multi-dom	165628	1	240	6.93866e-119	342.398	PLN00050	PLN00050	-
Q#413 - >Ca_04932.1|Ca_	superfamily	271593	47	298	9.82634e-22	91.6276	cl02567	WD40 superfamily	-
Q#413 - >Ca_04932.1|Ca_	multi-dom	225201	3	289	1.7527e-11	63.1839	COG2319	COG2319	-
Q#414 - >Ca_04940.1|Ca_	specific	177799	2	140	3.2673e-99	283.924	PLN00210	PLN00210	cl00334
Q#414 - >Ca_04940.1|Ca_	superfamily	260362	2	140	3.2673e-99	283.924	cl00334	Ribosomal_S9 superfamily	-

Query	Types	PSSM-ID	From	To	E-Value	Bitscore	Accession	Short name	Superfamily
Q#415 - >Ca_04941.1|Ca_	superfamily	260362	4	78	6.45616e-51	159.889	cl00334	Ribosomal_S9 superfamily	-
Q#418 - >Ca_05014.1|Ca_	superfamily	276020	86	297	4.83887e-73	235.287	cl12078	p450 superfamily	-
Q#419 - >Ca_05017.1|Ca_	superfamily	256046	1	112	9.51039e-13	63.9528	cl11168	PMD superfamily	-
Q#420 - >Ca_05018.1|Ca_	superfamily	276299	82	170	8.16041e-29	106.8	cl21455	ABC_ATPase superfamily	-
Q#420 - >Ca_05018.1|Ca_	superfamily	271701	1	46	4.23922e-14	65.4388	cl10459	Peptidases_S8_S53 superfamily	-
Q#421 - >Ca_05020.1|Ca_	specific	214719	200	283	1.03695e-07	48.5123	smart00554	FAS1	cl02663
Q#421 - >Ca_05020.1|Ca_	superfamily	261399	200	283	1.03695e-07	48.5123	cl02663	Fasciclin superfamily	-
Q#421 - >Ca_05020.1|Ca_	superfamily	261399	29	138	0.000230874	38.7773	cl02663	Fasciclin superfamily	-
Q#423 - >Ca_05035.1|Ca_	superfamily	261404	265	358	1.40341e-09	54.9639	cl02677	POX superfamily	-
Q#424 - >Ca_05040.1|Ca_	superfamily	259501	62	104	0.00327077	34.867	cl21207	KIAA1328 superfamily	-
Q#425 - >Ca_05049.1|Ca_	superfamily	256046	67	133	0.00680484	35.0628	cl11168	PMD superfamily	-
Q#428 - >Ca_05063.1|Ca_	superfamily	130699	161	215	0.00143704	35.9474	cl11766	hypothetical_protein superfamily	-
Q#429 - >Ca_05070.1|Ca_	superfamily	275525	7	117	1.21507e-56	173.847	cl00314	Ribosomal_S10 superfamily	-
Q#432 - >Ca_05086.1|Ca_	specific	259878	24	122	2.43235e-39	130.458	cd04216	Phytocyanin	cl19115
Q#432 - >Ca_05086.1|Ca_	superfamily	276230	24	122	2.43235e-39	130.458	cl19115	Cupredoxin superfamily	-
Q#433 - >Ca_05113.1|Ca_	superfamily	251325	17	127	5.28883e-33	117.369	cl12266	Branch superfamily	-
Q#434 - >Ca_05126.1|Ca_	superfamily	251669	61	129	2.96012e-08	47.7945	cl03830	Transposase_24 superfamily	-
Q#435 - >Ca_05133.1|Ca_	superfamily	271593	107	391	1.07688e-24	102.798	cl02567	WD40 superfamily	-
Q#435 - >Ca_05133.1|Ca_	multi-dom	225201	93	414	6.25127e-17	82.0587	COG2319	COG2319	-
Q#437 - >Ca_05149.1|Ca_	multi-dom	258434	46	108	1.98723e-10	53.8962	pfam14244	UBN2_3	-
Q#438 - >Ca_05151.1|Ca_	superfamily	276020	1	195	5.85555e-79	247.032	cl12078	p450 superfamily	-
Q#439 - >Ca_05153.1|Ca_	specific	189387	102	116	0.00194457	35.1225	pfam00098	zf-CCHC	cl22700
Q#439 - >Ca_05153.1|Ca_	superfamily	276654	102	116	0.00194457	35.1225	cl22700	zf-CCHC superfamily	-
Q#443 - >Ca_05165.1|Ca_	specific	252073	33	111	2.15564e-37	128.552	pfam03634	TCP	cl15990
Q#443 - >Ca_05165.1|Ca_	superfamily	252073	33	111	2.15564e-37	128.552	cl15990	TCP superfamily	-
Q#445 - >Ca_05194.1|Ca_	superfamily	275467	31	120	1.48925e-10	54.3112	cl00074	H2A superfamily	-
Q#446 - >Ca_05211.1|Ca_	multi-dom	275232	26	63	0.00444373	33.9084	TIGR04439	hypothetical_protein	-
Q#449 - >Ca_05250.1|Ca_	specific	197608	5	29	0.00236294	35.4902	smart00256	FBOX	cl02535
Q#449 - >Ca_05250.1|Ca_	superfamily	261329	5	29	0.00236294	35.4902	cl02535	F-box superfamily	-
Q#451 - >Ca_05281.1|Ca_	specific	250720	34	167	2.41693e-37	134.338	pfam01582	TIR	cl22462
Q#451 - >Ca_05281.1|Ca_	superfamily	250720	34	167	2.41693e-37	134.338	cl22462	TIR superfamily	-
Q#451 - >Ca_05281.1|Ca_	superfamily	276299	230	328	0.00101159	37.9276	cl21455	ABC_ATPase superfamily	-
Q#452 - >Ca_05296.1|Ca_	specific	237985	61	111	1.6677e-18	74.6155	cd00018	AP2	cl00033
Q#452 - >Ca_05296.1|Ca_	superfamily	260146	61	111	1.6677e-18	74.6155	cl00033	AP2 superfamily	-
Q#453 - >Ca_05299.1|Ca_	multi-dom	223021	268	418	0.000375687	42.2329	PHA03247	PHA03247	-
Q#454 - >Ca_05300.1|Ca_	superfamily	254387	202	239	4.52675e-11	60.5479	cl06662	RVT_2 superfamily	-

Query	Types	PSSM-ID	From	To	E-Value	Bitscore	Accession	Short name	Superfamily
Q#455 - >Ca_05301.1|Ca_	specific	238039	17	79	1.70182e-13	61.8756	cd00086	homeodomain	cl00084
Q#455 - >Ca_05301.1|Ca_	superfamily	275470	17	79	1.70182e-13	61.8756	cl00084	homeodomain superfamily	-
Q#458 - >Ca_05326.1|Ca_	superfamily	252977	1	185	1.42789e-47	166.544	cl04913	DUF674 superfamily	-
Q#459 - >Ca_05332.1|Ca_	specific	214842	231	295	5.03146e-12	60.289	smart00831	Cation_ATPase_N	cl02930
Q#459 - >Ca_05332.1|Ca_	superfamily	261517	231	295	5.03146e-12	60.289	cl02930	Cation_ATPase_N superfamily	-
Q#460 - >Ca_05339.1|Ca_	superfamily	256046	1	89	8.64875e-26	104.399	cl11168	PMD superfamily	-
Q#460 - >Ca_05339.1|Ca_	superfamily	256046	66	141	0.00300803	37.374	cl11168	PMD superfamily	-
Q#461 - >Ca_05348.1|Ca_	specific	249719	1	39	1.74724e-14	63.7994	pfam00254	FKBP_C	cl19519
Q#461 - >Ca_05348.1|Ca_	superfamily	267872	1	39	1.74724e-14	63.7994	cl19519	FKBP_C superfamily	-
Q#461 - >Ca_05348.1|Ca_	superfamily	267872	63	101	0.00895711	31.4426	cl19519	FKBP_C superfamily	-
Q#467 - >Ca_05374.1|Ca_	superfamily	271701	1	40	7.4525e-15	69.164	cl10459	Peptidases_S8_S53 superfamily	-
Q#468 - >Ca_05380.1|Ca_	specific	212558	39	89	1.23915e-16	69.1331	cd11660	SANT_TRF	cl21498
Q#468 - >Ca_05380.1|Ca_	superfamily	271918	39	89	1.23915e-16	69.1331	cl21498	SANT superfamily	-
Q#468 - >Ca_05380.1|Ca_	superfamily	276065	91	117	0.00152377	35.9492	cl14813	GluZincin superfamily	-
Q#469 - >Ca_05383.1|Ca_	superfamily	243129	4	106	4.51815e-18	75.7529	cl02653	MA3 superfamily	-
Q#470 - >Ca_05389.1|Ca_	multi-dom	223066	243	372	0.00128582	40.0417	PHA03379	PHA03379	-
Q#471 - >Ca_05395.1|Ca_	superfamily	271919	2	73	6.54228e-21	80.7751	cl21499	SPX superfamily	-
Q#472 - >Ca_05423.1|Ca_	superfamily	276334	1	98	7.13561e-53	168.066	cl21511	PEMT superfamily	-
Q#473 - >Ca_05430.1|Ca_	specific	176653	52	226	8.83169e-62	193.179	cd06141	WRN_exo	cl10012
Q#473 - >Ca_05430.1|Ca_	superfamily	271686	52	226	8.83169e-62	193.179	cl10012	DnaQ_like_exo superfamily	-
Q#475 - >Ca_05445.1|Ca_	specific	132921	45	181	1.53651e-72	224.681	cd07038	TPP_PYR_PDC_IPDC_like	cl11410
Q#475 - >Ca_05445.1|Ca_	superfamily	264357	45	181	1.53651e-72	224.681	cl11410	TPP_enzyme_PYR superfamily	-
Q#475 - >Ca_05445.1|Ca_	superfamily	275737	228	329	2.1511e-38	136.51	cl01629	TPP_enzymes superfamily	-
Q#476 - >Ca_05462.1|Ca_	multi-dom	215633	45	91	5.84827e-05	42.9395	PLN03210	PLN03210	-
Q#476 - >Ca_05462.1|Ca_	multi-dom	215633	101	134	0.0017176	38.3171	PLN03210	PLN03210	-
Q#477 - >Ca_05468.1|Ca_	specific	239511	18	101	1.11858e-32	111.092	cd03419	GRX_GRXh_1_2_like	cl00388
Q#477 - >Ca_05468.1|Ca_	superfamily	275563	18	101	1.11858e-32	111.092	cl00388	Thioredoxin_like superfamily	-
Q#479 - >Ca_05498.1|Ca_	multi-dom	240227	1	86	9.36477e-10	54.0302	PTZ00009	PTZ00009	-
Q#480 - >Ca_05502.1|Ca_	specific	197667	201	216	0.00474611	33.5708	smart00343	ZnF_C2HC	cl22700
Q#480 - >Ca_05502.1|Ca_	superfamily	276654	201	216	0.00474611	33.5708	cl22700	zf-CCHC superfamily	-
Q#482 - >Ca_05523.1|Ca_	specific	250720	74	159	1.38949e-21	85.4181	pfam01582	TIR	cl22462
Q#482 - >Ca_05523.1|Ca_	superfamily	250720	74	159	1.38949e-21	85.4181	cl22462	TIR superfamily	-
Q#483 - >Ca_05528.1|Ca_	superfamily	252977	1	383	1.21788e-44	161.922	cl04913	DUF674 superfamily	-
Q#484 - >Ca_05529.1|Ca_	superfamily	252977	1	356	1.14311e-44	161.152	cl04913	DUF674 superfamily	-
Q#485 - >Ca_05535.1|Ca_	specific	133136	363	453	3.34658e-18	80.0731	cd00303	retropepsin_like	cl11403
Q#485 - >Ca_05535.1|Ca_	superfamily	275927	363	453	3.34658e-18	80.0731	cl11403	pepsin_retropepsin_like superfamily	-

Query	Types	PSSM-ID	From	To	E-Value	Bitscore	Accession	Short name	Superfamily
Q#485 - >Ca_05535.1|Ca_	superfamily	275586	150	314	1.71896e-17	83.1161	cl00456	SLC5-6-like_sbd superfamily	-
Q#486 - >Ca_05558.1|Ca_	specific	258838	17	179	6.58088e-43	148.609	pfam14700	RPOL_N	cl20638
Q#486 - >Ca_05558.1|Ca_	superfamily	258838	17	179	6.58088e-43	148.609	cl20638	RPOL_N superfamily	-
Q#487 - >Ca_05561.1|Ca_	specific	133136	208	296	5.4793e-11	58.1168	cd00303	retropepsin_like	cl11403
Q#487 - >Ca_05561.1|Ca_	superfamily	275927	208	296	5.4793e-11	58.1168	cl11403	pepsin_retropepsin_like superfamily	-
Q#490 - >Ca_05570.1|Ca_	specific	133136	168	223	5.45015e-07	46.5608	cd00303	retropepsin_like	cl11403
Q#490 - >Ca_05570.1|Ca_	superfamily	275927	168	223	5.45015e-07	46.5608	cl11403	pepsin_retropepsin_like superfamily	-
Q#491 - >Ca_05581.1|Ca_	specific	250692	54	84	0.00355442	33.9665	pfam01535	PPR	cl03252
Q#491 - >Ca_05581.1|Ca_	superfamily	275791	54	84	0.00355442	33.9665	cl03252	PPR superfamily	-
Q#491 - >Ca_05581.1|Ca_	multi-dom	257477	82	126	6.48768e-07	44.701	pfam13041	PPR_2	-
Q#491 - >Ca_05581.1|Ca_	multi-dom	257477	190	232	5.57303e-05	39.3082	pfam13041	PPR_2	-
Q#491 - >Ca_05581.1|Ca_	multi-dom	257477	113	156	0.00135505	35.4562	pfam13041	PPR_2	-
Q#492 - >Ca_05589.1|Ca_	specific	238104	27	201	1.30016e-34	122.066	cd00178	STI	cl11466
Q#492 - >Ca_05589.1|Ca_	superfamily	264382	27	201	1.30016e-34	122.066	cl11466	STI superfamily	-
Q#493 - >Ca_05590.1|Ca_	specific	238104	27	201	1.30016e-34	122.066	cd00178	STI	cl11466
Q#493 - >Ca_05590.1|Ca_	superfamily	264382	27	201	1.30016e-34	122.066	cl11466	STI superfamily	-
Q#496 - >Ca_05594.1|Ca_	superfamily	265686	54	114	5.60775e-09	51.5218	cl15397	DUF89 superfamily	-
Q#500 - >Ca_05602.1|Ca_	specific	238825	570	686	3.66704e-56	190.499	cd01647	RT_LTR	cl02808
Q#500 - >Ca_05602.1|Ca_	superfamily	261455	570	686	3.66704e-56	190.499	cl02808	RT_like superfamily	-
Q#500 - >Ca_05602.1|Ca_	specific	133136	374	464	8.94007e-20	85.4659	cd00303	retropepsin_like	cl11403
Q#500 - >Ca_05602.1|Ca_	superfamily	275927	374	464	8.94007e-20	85.4659	cl11403	pepsin_retropepsin_like superfamily	-
Q#500 - >Ca_05602.1|Ca_	superfamily	252133	131	221	9.46325e-10	56.5781	cl04237	Retrotrans_gag superfamily	-
Q#500 - >Ca_05602.1|Ca_	multi-dom	249567	588	686	3.53511e-15	74.6746	pfam00078	RVT_1	-
Q#501 - >Ca_05607.1|Ca_	superfamily	253337	44	87	3.84019e-09	52.9081	cl05327	Auxin_canalis superfamily	-
Q#502 - >Ca_05614.1|Ca_	specific	238239	2	131	8.53096e-67	201.936	cd00412	pyrophosphatase	cl00217
Q#502 - >Ca_05614.1|Ca_	superfamily	260270	2	131	8.53096e-67	201.936	cl00217	pyrophosphatase superfamily	-
Q#507 - >Ca_05631.1|Ca_	superfamily	252133	100	199	8.12755e-11	56.1929	cl04237	Retrotrans_gag superfamily	-
Q#509 - >Ca_05645.1|Ca_	superfamily	256046	34	166	6.26541e-20	83.9832	cl11168	PMD superfamily	-
Q#510 - >Ca_05649.1|Ca_	specific	214614	438	598	5.78473e-30	114.739	smart00317	SET	cl02566
Q#510 - >Ca_05649.1|Ca_	superfamily	261347	438	598	5.78473e-30	114.739	cl02566	SET superfamily	-
Q#510 - >Ca_05649.1|Ca_	specific	252958	329	430	1.55311e-26	104.421	pfam05033	Pre-SET	cl02622
Q#510 - >Ca_05649.1|Ca_	superfamily	261377	329	430	1.55311e-26	104.421	cl02622	Pre-SET superfamily	-
Q#510 - >Ca_05649.1|Ca_	superfamily	261375	173	305	1.8862e-19	85.7673	cl02620	SAD_SRA superfamily	-
Q#511 - >Ca_05653.1|Ca_	specific	239356	8	81	1.11796e-39	132.787	cd03058	GST_N_Tau	cl00388
Q#511 - >Ca_05653.1|Ca_	superfamily	275563	8	81	1.11796e-39	132.787	cl00388	Thioredoxin_like superfamily	-
Q#511 - >Ca_05653.1|Ca_	specific	198294	91	212	1.35341e-39	134.228	cd03185	GST_C_Tau	cl02776

Query	Types	PSSM-ID	From	To	E-Value	Bitscore	Accession	Short name	Superfamily
Q#511 - >Ca_05653.1|Ca_	superfamily	275780	91	212	1.35341e-39	134.228	cl02776	GST_C_family superfamily	-
Q#511 - >Ca_05653.1|Ca_	multi-dom	223698	8	185	3.97952e-27	103.736	COG0625	Gst	-
Q#512 - >Ca_05654.1|Ca_	specific	198294	91	217	1.77128e-48	157.34	cd03185	GST_C_Tau	cl02776
Q#512 - >Ca_05654.1|Ca_	superfamily	275780	91	217	1.77128e-48	157.34	cl02776	GST_C_family superfamily	-
Q#512 - >Ca_05654.1|Ca_	specific	239356	8	81	9.59141e-39	130.476	cd03058	GST_N_Tau	cl00388
Q#512 - >Ca_05654.1|Ca_	superfamily	275563	8	81	9.59141e-39	130.476	cl00388	Thioredoxin_like superfamily	-
Q#512 - >Ca_05654.1|Ca_	multi-dom	223698	9	213	3.58734e-24	96.0321	COG0625	Gst	-
Q#513 - >Ca_05657.1|Ca_	multi-dom	259534	525	654	5.13264e-05	45.5202	pfam15402	Spc7_N	-
Q#513 - >Ca_05657.1|Ca_	multi-dom	223021	2	294	0.00916236	38.3809	PHA03247	PHA03247	-
Q#514 - >Ca_05663.1|Ca_	specific	260004	3	121	2.32863e-59	182.283	cd09272	RNase_HI_RT_Ty1	cl14782
Q#514 - >Ca_05663.1|Ca_	superfamily	271754	3	121	2.32863e-59	182.283	cl14782	RNase_H_like superfamily	-
Q#515 - >Ca_05672.1|Ca_	specific	238096	68	111	0.000255739	36.0142	cd00167	SANT	cl21498
Q#515 - >Ca_05672.1|Ca_	superfamily	271918	68	111	0.000255739	36.0142	cl21498	SANT superfamily	-
Q#517 - >Ca_05679.1|Ca_	specific	119339	2	189	1.32208e-30	113.039	cd06558	crotonase-like	cl21466
Q#517 - >Ca_05679.1|Ca_	superfamily	276308	2	189	1.32208e-30	113.039	cl21466	crotonase-like superfamily	-
Q#518 - >Ca_05686.1|Ca_	specific	260004	283	420	1.29549e-59	193.068	cd09272	RNase_HI_RT_Ty1	cl14782
Q#518 - >Ca_05686.1|Ca_	superfamily	271754	283	420	1.29549e-59	193.068	cl14782	RNase_H_like superfamily	-
Q#518 - >Ca_05686.1|Ca_	superfamily	254387	142	275	1.59951e-50	172.641	cl06662	RVT_2 superfamily	-
Q#520 - >Ca_05702.1|Ca_	specific	189387	80	97	0.000991497	37.8189	pfam00098	zf-CCHC	cl22700
Q#520 - >Ca_05702.1|Ca_	superfamily	276654	80	97	0.000991497	37.8189	cl22700	zf-CCHC superfamily	-
Q#523 - >Ca_05710.1|Ca_	superfamily	276175	1	103	4.91897e-52	175.993	cl17037	NBD_sugar-kinase_HSP70_actin superfamily	-
Q#523 - >Ca_05710.1|Ca_	multi-dom	240227	1	277	9.98682e-87	273.979	PTZ00009	PTZ00009	-
Q#529 - >Ca_05731.1|Ca_	specific	214614	134	186	6.77991e-17	73.9079	smart00317	SET	cl02566
Q#529 - >Ca_05731.1|Ca_	superfamily	261347	134	186	6.77991e-17	73.9079	cl02566	SET superfamily	-
Q#531 - >Ca_05743.1|Ca_	multi-dom	257262	202	302	0.00201087	39.3973	pfam12737	Mating_C	-
Q#535 - >Ca_05770.1|Ca_	specific	133437	17	69	4.96451e-15	68.2974	cd01667	TGS_ThrRS_N	cl00155
Q#535 - >Ca_05770.1|Ca_	superfamily	275476	17	69	4.96451e-15	68.2974	cl00155	UBQ superfamily	-
Q#540 - >Ca_05818.1|Ca_	superfamily	275947	126	328	1.73782e-15	76.1065	cl11493	PQQ_DH_like superfamily	-
Q#540 - >Ca_05818.1|Ca_	multi-dom	257688	8	320	5.34266e-12	63.5632	pfam13360	PQQ_2	-
Q#541 - >Ca_05822.1|Ca_	superfamily	276297	110	206	4.05655e-24	96.9582	cl21453	PKc_like superfamily	-
Q#545 - >Ca_05899.1|Ca_	superfamily	275518	13	141	0.00524399	36.1248	cl00304	TP_methylase superfamily	-
Q#546 - >Ca_05909.1|Ca_	superfamily	271754	96	194	6.278e-06	44.0205	cl14782	RNase_H_like superfamily	-
Q#548 - >Ca_05921.1|Ca_	multi-dom	147601	224	365	0.00129917	39.5664	pfam05505	Ebola_NP	-
Q#549 - >Ca_05928.1|Ca_	specific	250356	6	141	2.81582e-76	225.46	pfam01090	Ribosomal_S19e	cl00969
Q#549 - >Ca_05928.1|Ca_	superfamily	260724	6	141	2.81582e-76	225.46	cl00969	Ribosomal_S19e superfamily	-
Q#550 - >Ca_05931.1|Ca_	multi-dom	223021	70	286	0.000316623	42.6181	PHA03247	PHA03247	-

Query	Types	PSSM-ID	From	To	E-Value	Bitscore	Accession	Short name	Superfamily
Q#551 - >Ca_05932.1|Ca_	multi-dom	165099	214	338	0.000634276	40.5065	PHA02732	PHA02732	-
Q#557 - >Ca_05963.1|Ca_	specific	250770	1	156	1.41689e-59	191.349	pfam01650	Peptidase_C13	cl00042
Q#557 - >Ca_05963.1|Ca_	superfamily	271411	1	156	1.41689e-59	191.349	cl00042	CASc superfamily	-
Q#559 - >Ca_05969.1|Ca_	superfamily	275753	3	206	1.1926e-83	253.303	cl01911	Pectinesterase superfamily	-
Q#561 - >Ca_06000.1|Ca_	superfamily	276263	124	349	1.41485e-16	77.0144	cl19514	FBA_1 superfamily	-
Q#561 - >Ca_06000.1|Ca_	specific	197608	14	54	4.60795e-08	49.3574	smart00256	FBOX	cl02535
Q#561 - >Ca_06000.1|Ca_	superfamily	261329	14	54	4.60795e-08	49.3574	cl02535	F-box superfamily	-
Q#564 - >Ca_06024.1|Ca_	specific	239926	33	99	1.79034e-13	64.1956	cd04480	RPA1_DBD_A_like	cl09930
Q#564 - >Ca_06024.1|Ca_	superfamily	275906	33	99	1.79034e-13	64.1956	cl09930	RPA_2b-aaRSs_OBF_like superfamily	-
Q#564 - >Ca_06024.1|Ca_	superfamily	275906	114	214	4.46057e-08	50.3843	cl09930	RPA_2b-aaRSs_OBF_like superfamily	-
Q#566 - >Ca_06038.1|Ca_	superfamily	251810	3	68	4.37107e-15	65.0379	cl03943	Yippee-Mis18 superfamily	-
Q#568 - >Ca_06051.1|Ca_	superfamily	202502	1	94	5.65115e-44	152.415	cl03823	Transposase_21 superfamily	-
Q#568 - >Ca_06051.1|Ca_	superfamily	258207	233	264	5.86987e-13	63.3691	cl16495	DUF4216 superfamily	-
Q#570 - >Ca_06071.1|Ca_	superfamily	223036	45	109	0.00457884	36.1899	cl19802	PHA03295 superfamily	-
Q#572 - >Ca_06073.1|Ca_	specific	211392	5	197	1.59641e-58	183.105	cd11380	Ribosomal_S8e_like	cl00880
Q#572 - >Ca_06073.1|Ca_	superfamily	260676	5	197	1.59641e-58	183.105	cl00880	Ribosomal_S8e_like superfamily	-
Q#572 - >Ca_06073.1|Ca_	multi-dom	240292	1	213	1.39556e-115	331.186	PTZ00148	PTZ00148	-
Q#574 - >Ca_06075.1|Ca_	multi-dom	273330	22	104	7.20584e-11	57.5174	TIGR00906	Low_affinity_cationic_amino_acid_transporter_2	-
Q#575 - >Ca_06099.1|Ca_	multi-dom	254474	1	76	1.07026e-06	43.3452	pfam07859	Abhydrolase_3	-
Q#581 - >Ca_06116.1|Ca_	superfamily	276192	28	89	2.52934e-20	80.35	cl17169	RRM_SF superfamily	-
Q#581 - >Ca_06116.1|Ca_	multi-dom	214636	28	92	1.71111e-15	67.2335	smart00360	RRM	-
Q#582 - >Ca_06124.1|Ca_	multi-dom	223021	180	326	9.31424e-05	43.3885	PHA03247	PHA03247	-
Q#584 - >Ca_06140.1|Ca_	multi-dom	227709	419	622	0.00091726	41.4151	COG5422	ROM1	-
Q#587 - >Ca_06162.1|Ca_	specific	203773	6	174	1.65243e-55	179.094	pfam07819	PGAP1	cl21494
Q#587 - >Ca_06162.1|Ca_	superfamily	276326	6	174	1.65243e-55	179.094	cl21494	Esterase_lipase superfamily	-
Q#587 - >Ca_06162.1|Ca_	multi-dom	257231	30	115	0.000519071	38.6841	pfam12697	Abhydrolase_6	-
Q#588 - >Ca_06168.1|Ca_	specific	239089	120	185	4.67287e-19	78.2871	cd02396	PCBP_like_KH	cl00098
Q#588 - >Ca_06168.1|Ca_	superfamily	275471	120	185	4.67287e-19	78.2871	cl00098	KH-I superfamily	-
Q#588 - >Ca_06168.1|Ca_	superfamily	275507	1	82	4.46826e-50	168.147	cl00268	class_II_aaRS-like_core superfamily	-
Q#588 - >Ca_06168.1|Ca_	superfamily	275471	206	227	0.00034678	37.0708	cl00098	KH-I superfamily	-
Q#591 - >Ca_06187.1|Ca_	superfamily	260768	2	49	5.43647e-12	57.6475	cl01053	SGNH_hydrolase superfamily	-
Q#592 - >Ca_06189.1|Ca_	superfamily	275485	64	133	0.00323878	38.3301	cl00210	Isoprenoid_Biosyn_C1 superfamily	-
Q#593 - >Ca_06190.1|Ca_	superfamily	254387	6	136	2.55172e-36	128.343	cl06662	RVT_2 superfamily	-
Q#596 - >Ca_06274.1|Ca_	specific	249606	246	396	4.12022e-21	91.8614	pfam00122	E1-E2_ATPase	cl02811
Q#596 - >Ca_06274.1|Ca_	superfamily	249606	246	396	4.12022e-21	91.8614	cl02811	E1-E2_ATPase superfamily	-
Q#596 - >Ca_06274.1|Ca_	specific	257602	398	482	1.15631e-14	69.9501	pfam13246	Hydrolase_like2	cl16182

Query	Types	PSSM-ID	From	To	E-Value	Bitscore	Accession	Short name	Superfamily
Q#596 - >Ca_06274.1|Ca_	superfamily	257602	398	482	1.15631e-14	69.9501	cl16182	Hydrolase_like2 superfamily	-
Q#599 - >Ca_06284.1|Ca_	specific	251775	17	115	1.38961e-35	121.363	pfam03171	2OG-FeII_Oxy	cl21496
Q#599 - >Ca_06284.1|Ca_	superfamily	276328	17	115	1.38961e-35	121.363	cl21496	2OG-FeII_Oxy superfamily	-
Q#600 - >Ca_06285.1|Ca_	specific	258418	56	163	6.91656e-34	117.727	pfam14226	DIOX_N	cl21672
Q#600 - >Ca_06285.1|Ca_	superfamily	272092	56	163	6.91656e-34	117.727	cl21672	DIOX_N superfamily	-
Q#601 - >Ca_06318.1|Ca_	superfamily	275507	75	162	1.23192e-53	179.318	cl00268	class_II_aaRS-like_core superfamily	-
Q#601 - >Ca_06318.1|Ca_	superfamily	275471	256	277	0.00730537	33.604	cl00098	KH-I superfamily	-
Q#604 - >Ca_06363.1|Ca_	superfamily	260211	24	293	5.63713e-104	311.28	cl00137	SERPIN superfamily	-
Q#608 - >Ca_06416.1|Ca_	specific	254181	1	84	1.5354e-21	82.6557	pfam07386	DUF1499	cl01841
Q#608 - >Ca_06416.1|Ca_	superfamily	261091	1	84	1.5354e-21	82.6557	cl01841	DUF1499 superfamily	-
Q#609 - >Ca_06455.1|Ca_	superfamily	256046	154	224	1.38634e-06	47.7744	cl11168	PMD superfamily	-
Q#612 - >Ca_06481.1|Ca_	specific	189907	1	81	9.54977e-49	150.819	pfam01249	Ribosomal_S21e	cl03140
Q#612 - >Ca_06481.1|Ca_	superfamily	189907	1	81	9.54977e-49	150.819	cl03140	Ribosomal_S21e superfamily	-
Q#613 - >Ca_06518.1|Ca_	superfamily	275799	47	127	2.032e-05	38.6967	cl04011	DPBB_1 superfamily	-
Q#614 - >Ca_06520.1|Ca_	superfamily	275799	85	154	0.00713809	32.5335	cl04011	DPBB_1 superfamily	-
Q#615 - >Ca_06527.1|Ca_	specific	252718	1	248	1.68748e-151	426.705	pfam04646	DUF604	cl21608
Q#615 - >Ca_06527.1|Ca_	superfamily	272028	1	248	1.68748e-151	426.705	cl21608	Galactosyl_T superfamily	-
Q#618 - >Ca_06568.1|Ca_	superfamily	260203	81	180	2.10966e-10	59.2623	cl00120	PP2Cc superfamily	-
Q#618 - >Ca_06568.1|Ca_	specific	214625	289	401	4.09264e-10	58.5405	smart00332	PP2Cc	cl00120
Q#618 - >Ca_06568.1|Ca_	superfamily	260203	289	401	4.09264e-10	58.5405	cl00120	PP2Cc superfamily	-
Q#619 - >Ca_06571.1|Ca_	multi-dom	165099	416	547	0.0020835	39.7361	PHA02732	PHA02732	-
Q#620 - >Ca_06577.1|Ca_	superfamily	275460	255	387	2.0304e-58	189.392	cl00015	nt_trans superfamily	-
Q#620 - >Ca_06577.1|Ca_	multi-dom	215227	255	403	4.96001e-81	257.303	PLN02406	PLN02406	-
Q#622 - >Ca_06585.1|Ca_	superfamily	256046	83	239	6.24505e-15	73.1976	cl11168	PMD superfamily	-
Q#623 - >Ca_06595.1|Ca_	superfamily	276299	2	89	6.36148e-30	106.8	cl21455	ABC_ATPase superfamily	-
Q#624 - >Ca_06630.1|Ca_	superfamily	252133	113	163	2.75471e-11	58.5041	cl04237	Retrotrans_gag superfamily	-
Q#624 - >Ca_06630.1|Ca_	superfamily	275927	178	214	0.000489223	38.1695	cl11403	pepsin_retropepsin_like superfamily	-
Q#625 - >Ca_06635.1|Ca_	specific	133437	76	137	3.66739e-20	84.0906	cd01667	TGS_ThrRS_N	cl00155
Q#625 - >Ca_06635.1|Ca_	superfamily	275476	76	137	3.66739e-20	84.0906	cl00155	UBQ superfamily	-
Q#626 - >Ca_06643.1|Ca_	superfamily	254387	42	132	9.21529e-24	92.5195	cl06662	RVT_2 superfamily	-
Q#627 - >Ca_06646.1|Ca_	superfamily	276297	7	133	3.29817e-34	121.031	cl21453	PKc_like superfamily	-
Q#630 - >Ca_06719.1|Ca_	superfamily	260771	88	121	6.31766e-05	36.89	cl01066	Trm112p superfamily	-
Q#632 - >Ca_06775.1|Ca_	superfamily	253337	3	43	2.34534e-07	44.4337	cl05327	Auxin_canalis superfamily	-
Q#634 - >Ca_06860.1|Ca_	specific	258161	98	120	0.00280576	34.9172	pfam13894	zf-C2H2_4	cl20498
Q#634 - >Ca_06860.1|Ca_	superfamily	258161	98	120	0.00280576	34.9172	cl20498	zf-C2H2_4 superfamily	-
Q#634 - >Ca_06860.1|Ca_	multi-dom	257361	109	207	0.00847616	34.8609	pfam12868	DUF3824	-

Query	Types	PSSM-ID	From	To	E-Value	Bitscore	Accession	Short name	Superfamily
Q#635 - >Ca_06862.1|Ca_	superfamily	253250	157	180	5.11852e-05	38.2409	cl05229	DUF761 superfamily	-
Q#636 - >Ca_06864.1|Ca_	specific	238002	47	119	8.03282e-20	81.3664	cd00042	CY	cl09238
Q#636 - >Ca_06864.1|Ca_	superfamily	275873	47	119	8.03282e-20	81.3664	cl09238	CY superfamily	-
Q#636 - >Ca_06864.1|Ca_	superfamily	275873	142	187	0.00470303	34.372	cl09238	CY superfamily	-
Q#638 - >Ca_06933.1|Ca_	specific	132916	6	79	2.94779e-28	107.142	cd07033	TPP_PYR_DXS_TK_like	cl11410
Q#638 - >Ca_06933.1|Ca_	superfamily	264357	6	79	2.94779e-28	107.142	cl11410	TPP_enzyme_PYR superfamily	-
Q#638 - >Ca_06933.1|Ca_	superfamily	276298	81	146	2.98037e-18	81.467	cl21454	SDR superfamily	-
Q#640 - >Ca_06960.1|Ca_	superfamily	260927	53	103	5.33351e-17	72.2898	cl01412	Alpha-L-AF_C superfamily	-
Q#641 - >Ca_06964.1|Ca_	superfamily	276299	69	315	1.01596e-143	413.141	cl21455	ABC_ATPase superfamily	-
Q#644 - >Ca_07049.1|Ca_	specific	254692	24	62	1.14954e-07	47.7138	pfam08263	LRRNT_2	cl08472
Q#644 - >Ca_07049.1|Ca_	superfamily	254692	24	62	1.14954e-07	47.7138	cl08472	LRRNT_2 superfamily	-
Q#644 - >Ca_07049.1|Ca_	superfamily	276664	121	297	5.37522e-06	46.1946	cl22710	LRR_RI superfamily	-
Q#644 - >Ca_07049.1|Ca_	multi-dom	258126	119	179	1.67892e-07	47.5325	pfam13855	LRR_8	-
Q#644 - >Ca_07049.1|Ca_	multi-dom	258126	215	272	0.00498645	34.4357	pfam13855	LRR_8	-
Q#645 - >Ca_07075.1|Ca_	specific	238125	243	295	0.000198019	40.0595	cd00204	ANK	cl02529
Q#645 - >Ca_07075.1|Ca_	superfamily	275769	243	295	0.000198019	40.0595	cl02529	ANK superfamily	-
Q#645 - >Ca_07075.1|Ca_	specific	258214	340	452	1.7224e-25	101.029	pfam13962	PGG	cl16503
Q#645 - >Ca_07075.1|Ca_	superfamily	258214	340	452	1.7224e-25	101.029	cl16503	PGG superfamily	-
Q#645 - >Ca_07075.1|Ca_	multi-dom	257303	243	285	3.66053e-05	41.8579	pfam12796	Ank_2	-
Q#646 - >Ca_07079.1|Ca_	specific	249651	9	82	1.43266e-36	122.307	pfam00173	Cyt-b5	cl02041
Q#646 - >Ca_07079.1|Ca_	superfamily	261158	9	82	1.43266e-36	122.307	cl02041	Cyt-b5 superfamily	-
Q#648 - >Ca_07119.1|Ca_	specific	238705	4	167	5.9273e-76	228.389	cd01417	Ribosomal_L19e_E	cl00232
Q#648 - >Ca_07119.1|Ca_	superfamily	260284	4	167	5.9273e-76	228.389	cl00232	Ribosomal_L19e superfamily	-
Q#649 - >Ca_07125.1|Ca_	multi-dom	273731	47	161	1.64521e-23	97.0125	TIGR01647	Plasma_membrane_ATPase	-
Q#650 - >Ca_07128.1|Ca_	superfamily	204810	30	75	1.939e-15	64.3025	cl13460	DUF3511 superfamily	-
Q#651 - >Ca_07142.1|Ca_	specific	214614	303	392	5.05441e-20	85.0787	smart00317	SET	cl02566
Q#651 - >Ca_07142.1|Ca_	superfamily	261347	303	392	5.05441e-20	85.0787	cl02566	SET superfamily	-
Q#652 - >Ca_07143.1|Ca_	superfamily	252005	8	185	3.42235e-38	135.864	cl15987	GRAS superfamily	-
Q#653 - >Ca_07157.1|Ca_	specific	206717	18	176	1.28842e-125	359.799	cd04150	Arf1_5_like	cl21455
Q#653 - >Ca_07157.1|Ca_	superfamily	276299	18	176	1.28842e-125	359.799	cl21455	ABC_ATPase superfamily	-
Q#653 - >Ca_07157.1|Ca_	multi-dom	197556	15	177	7.78909e-31	115.803	smart00178	SAR	-
Q#654 - >Ca_07191.1|Ca_	specific	239111	122	334	6.01334e-103	305.734	cd02620	Peptidase_C1A_CathepsinB	cl00296
Q#654 - >Ca_07191.1|Ca_	superfamily	275516	122	334	6.01334e-103	305.734	cl00296	Peptidase_C1 superfamily	-
Q#654 - >Ca_07191.1|Ca_	specific	203856	40	82	2.26522e-06	44.1188	pfam08127	Propeptide_C1	cl06937
Q#654 - >Ca_07191.1|Ca_	superfamily	203856	40	82	2.26522e-06	44.1188	cl06937	Propeptide_C1 superfamily	-
Q#655 - >Ca_07199.1|Ca_	specific	249667	64	210	6.60983e-39	132.787	pfam00190	Cupin_1	cl21464

Query	Types	PSSM-ID	From	To	E-Value	Bitscore	Accession	Short name	Superfamily
Q#655 - >Ca_07199.1|Ca_	superfamily	276307	64	210	6.60983e-39	132.787	cl21464	ABD superfamily	-
Q#656 - >Ca_07225.1|Ca_	specific	211390	86	197	4.67553e-29	106.131	cd11378	DUF296	cl00720
Q#656 - >Ca_07225.1|Ca_	superfamily	260588	86	197	4.67553e-29	106.131	cl00720	DUF296 superfamily	-
Q#657 - >Ca_07240.1|Ca_	specific	189034	53	187	6.67219e-106	303.391	cd09864	PIN_Fcf1	cl14812
Q#657 - >Ca_07240.1|Ca_	superfamily	276064	53	187	6.67219e-106	303.391	cl14812	PIN_SF superfamily	-
Q#660 - >Ca_07281.1|Ca_	specific	99751	55	99	9.70152e-07	41.7621	cd06257	DnaJ	cl02542
Q#660 - >Ca_07281.1|Ca_	superfamily	271590	55	99	9.70152e-07	41.7621	cl02542	DnaJ superfamily	-
Q#662 - >Ca_07331.1|Ca_	multi-dom	215633	8	218	9.74763e-09	54.1103	PLN03210	PLN03210	-
Q#664 - >Ca_07356.1|Ca_	specific	251895	16	143	2.46865e-31	113.488	pfam03357	Snf7	cl21588
Q#664 - >Ca_07356.1|Ca_	superfamily	272008	16	143	2.46865e-31	113.488	cl21588	Snf7 superfamily	-
Q#666 - >Ca_07380.1|Ca_	specific	238083	62	359	7.68476e-42	149.014	cd00143	PP2Cc	cl00120
Q#666 - >Ca_07380.1|Ca_	superfamily	260203	62	359	7.68476e-42	149.014	cl00120	PP2Cc superfamily	-
Q#667 - >Ca_07381.1|Ca_	specific	238083	79	384	7.26535e-47	163.266	cd00143	PP2Cc	cl00120
Q#667 - >Ca_07381.1|Ca_	superfamily	260203	79	384	7.26535e-47	163.266	cl00120	PP2Cc superfamily	-
Q#668 - >Ca_07382.1|Ca_	specific	238083	42	358	6.7613e-44	154.792	cd00143	PP2Cc	cl00120
Q#668 - >Ca_07382.1|Ca_	superfamily	260203	42	358	6.7613e-44	154.792	cl00120	PP2Cc superfamily	-
Q#669 - >Ca_07383.1|Ca_	specific	238083	46	365	3.871e-32	122.05	cd00143	PP2Cc	cl00120
Q#669 - >Ca_07383.1|Ca_	superfamily	260203	46	365	3.871e-32	122.05	cl00120	PP2Cc superfamily	-
Q#671 - >Ca_07417.1|Ca_	specific	252304	10	180	3.12577e-28	106.305	pfam03998	Utp11	cl02164
Q#671 - >Ca_07417.1|Ca_	superfamily	261203	10	180	3.12577e-28	106.305	cl02164	Utp11 superfamily	-
Q#672 - >Ca_07435.1|Ca_	specific	238088	15	113	2.81544e-19	78.3865	cd00156	REC	cl19078
Q#672 - >Ca_07435.1|Ca_	superfamily	271833	15	113	2.81544e-19	78.3865	cl19078	REC superfamily	-
Q#673 - >Ca_07446.1|Ca_	multi-dom	254113	130	211	7.30104e-16	74.3924	pfam07227	DUF1423	-
Q#673 - >Ca_07446.1|Ca_	multi-dom	235600	5	106	0.00888062	35.2884	PRK05771	PRK05771	-
Q#674 - >Ca_07484.1|Ca_	specific	206540	556	655	1.4416e-43	152.313	pfam14372	DUF4413	cl16834
Q#674 - >Ca_07484.1|Ca_	superfamily	206540	556	655	1.4416e-43	152.313	cl16834	DUF4413 superfamily	-
Q#674 - >Ca_07484.1|Ca_	specific	214746	322	373	3.39482e-10	56.6672	smart00614	ZnF_BED	cl02703
Q#674 - >Ca_07484.1|Ca_	superfamily	261413	322	373	3.39482e-10	56.6672	cl02703	zf-BED superfamily	-
Q#675 - >Ca_07513.1|Ca_	specific	238068	16	149	2.14331e-21	84.7382	cd00121	MATH	cl02446
Q#675 - >Ca_07513.1|Ca_	superfamily	261289	16	149	2.14331e-21	84.7382	cl02446	MATH superfamily	-
Q#676 - >Ca_07535.1|Ca_	superfamily	276193	12	77	0.00506982	32.0166	cl17171	PH-like superfamily	-
Q#677 - >Ca_07554.1|Ca_	specific	238068	16	149	2.14331e-21	84.7382	cd00121	MATH	cl02446
Q#677 - >Ca_07554.1|Ca_	superfamily	261289	16	149	2.14331e-21	84.7382	cl02446	MATH superfamily	-
Q#678 - >Ca_07575.1|Ca_	superfamily	271982	180	344	4.08435e-19	83.065	cl21562	DDE_Tnp_1_6 superfamily	-
Q#678 - >Ca_07575.1|Ca_	superfamily	276302	87	133	0.00366426	34.9938	cl21459	HTH_XRE superfamily	-
Q#679 - >Ca_07583.1|Ca_	superfamily	271982	180	344	4.08435e-19	83.065	cl21562	DDE_Tnp_1_6 superfamily	-

Query	Types	PSSM-ID	From	To	E-Value	Bitscore	Accession	Short name	Superfamily
Q#679 - >Ca_07583.1|Ca_	superfamily	276302	87	133	0.00366426	34.9938	cl21459	HTH_XRE superfamily	-
Q#681 - >Ca_07635.1|Ca_	specific	191032	1	167	1.59386e-69	210.646	pfam04573	SPC22	cl04601
Q#681 - >Ca_07635.1|Ca_	superfamily	194922	1	167	1.59386e-69	210.646	cl04601	SPC22 superfamily	-
Q#683 - >Ca_07703.1|Ca_	specific	146016	5	109	1.18459e-27	98.8024	pfam03179	V-ATPase_G	cl03922
Q#683 - >Ca_07703.1|Ca_	superfamily	155755	5	109	1.18459e-27	98.8024	cl03922	V-ATPase_G superfamily	-
Q#684 - >Ca_07723.1|Ca_	superfamily	276175	9	71	1.15627e-51	166.749	cl17037	NBD_sugar-kinase_HSP70_actin superfamily	-
Q#685 - >Ca_07725.1|Ca_	specific	250855	17	124	6.1339e-43	138.894	pfam01776	Ribosomal_L22e	cl03348
Q#685 - >Ca_07725.1|Ca_	superfamily	261695	17	124	6.1339e-43	138.894	cl03348	Ribosomal_L22e superfamily	-
Q#686 - >Ca_07783.1|Ca_	superfamily	275544	27	132	5.2651e-35	119.744	cl00350	Ribosomal_S19 superfamily	-
Q#687 - >Ca_07793.1|Ca_	superfamily	241616	9	74	8.08791e-11	56.1143	cl00109	MADS superfamily	-
Q#688 - >Ca_07928.1|Ca_	superfamily	275544	39	110	6.24107e-34	115.08	cl00350	Ribosomal_S19 superfamily	-
Q#689 - >Ca_07979.1|Ca_	superfamily	275483	11	233	2.33618e-143	414.145	cl00204	PFK superfamily	-
Q#690 - >Ca_07999.1|Ca_	multi-dom	226444	1	146	5.00315e-05	41.4215	COG3934	COG3934	-
Q#691 - >Ca_08012.1|Ca_	specific	275442	17	169	1.80262e-41	138.725	cd15798	PMEI-like_3	cl04375
Q#691 - >Ca_08012.1|Ca_	superfamily	275808	17	169	1.80262e-41	138.725	cl04375	PMEI_like superfamily	-
Q#695 - >Ca_08034.1|Ca_	superfamily	275582	38	202	1.0973e-24	105.269	cl00451	MoCF_BD superfamily	-
Q#695 - >Ca_08034.1|Ca_	superfamily	275582	238	301	9.75277e-09	56.3482	cl00451	MoCF_BD superfamily	-
Q#696 - >Ca_08035.1|Ca_	specific	250027	23	70	1.47322e-08	49.8957	pfam00646	F-box	cl02535
Q#696 - >Ca_08035.1|Ca_	superfamily	261329	23	70	1.47322e-08	49.8957	cl02535	F-box superfamily	-
Q#696 - >Ca_08035.1|Ca_	superfamily	276263	142	263	0.00135126	38.1092	cl19514	FBA_1 superfamily	-
Q#697 - >Ca_08036.1|Ca_	superfamily	265652	127	188	8.94896e-09	49.9869	cl15242	BfiI_C_EcoRII_N_B3 superfamily	-
Q#698 - >Ca_08037.1|Ca_	specific	260004	474	518	1.27905e-14	72.1157	cd09272	RNase_HI_RT_Ty1	cl14782
Q#698 - >Ca_08037.1|Ca_	superfamily	271754	474	518	1.27905e-14	72.1157	cl14782	RNase_H_like superfamily	-
Q#698 - >Ca_08037.1|Ca_	specific	197383	116	194	8.2786e-13	65.8121	cd10017	B3_DNA	cl15242
Q#698 - >Ca_08037.1|Ca_	superfamily	265652	116	194	8.2786e-13	65.8121	cl15242	BfiI_C_EcoRII_N_B3 superfamily	-
Q#698 - >Ca_08037.1|Ca_	specific	237987	522	645	1.37013e-08	53.8575	cd00020	ARM	cl22454
Q#698 - >Ca_08037.1|Ca_	superfamily	276408	522	645	1.37013e-08	53.8575	cl22454	ARM superfamily	-
Q#698 - >Ca_08037.1|Ca_	specific	237987	696	852	6.08298e-08	51.5463	cd00020	ARM	cl22454
Q#698 - >Ca_08037.1|Ca_	superfamily	276408	696	852	6.08298e-08	51.5463	cl22454	ARM superfamily	-
Q#698 - >Ca_08037.1|Ca_	specific	237987	631	722	6.46772e-07	48.4647	cd00020	ARM	cl22454
Q#698 - >Ca_08037.1|Ca_	superfamily	276408	631	722	6.46772e-07	48.4647	cl22454	ARM superfamily	-
Q#698 - >Ca_08037.1|Ca_	specific	191545	222	302	8.00312e-35	128.52	pfam06507	Auxin_resp	cl05824
Q#698 - >Ca_08037.1|Ca_	superfamily	191545	222	302	8.00312e-35	128.52	cl05824	Auxin_resp superfamily	-
Q#699 - >Ca_08054.1|Ca_	specific	275443	2	156	1.49578e-21	87.8507	cd15799	PMEI-like_4	cl04375
Q#699 - >Ca_08054.1|Ca_	superfamily	275808	2	156	1.49578e-21	87.8507	cl04375	PMEI_like superfamily	-
Q#699 - >Ca_08054.1|Ca_	superfamily	275753	195	242	9.65376e-16	73.4152	cl01911	Pectinesterase superfamily	-

Query	Types	PSSM-ID	From	To	E-Value	Bitscore	Accession	Short name	Superfamily
Q#700 - >Ca_08056.1|Ca_	specific	176255	4	341	0	534.977	cd08295	double_bond_reductase_like	cl16912
Q#700 - >Ca_08056.1|Ca_	superfamily	276140	4	341	0	534.977	cl16912	MDR superfamily	-
Q#700 - >Ca_08056.1|Ca_	multi-dom	215606	2	344	8.71424e-153	436.579	PLN03154	PLN03154	-
Q#704 - >Ca_08070.1|Ca_	multi-dom	173561	254	290	7.47494e-08	49.8053	PTZ00368	PTZ00368	-
Q#711 - >Ca_08111.1|Ca_	specific	214587	9	134	4.58603e-31	115.498	smart00255	TIR	cl22440
Q#711 - >Ca_08111.1|Ca_	superfamily	276394	9	134	4.58603e-31	115.498	cl22440	TIR_2 superfamily	-
Q#711 - >Ca_08111.1|Ca_	specific	214842	256	322	1.36633e-13	65.2966	smart00831	Cation_ATPase_N	cl02930
Q#711 - >Ca_08111.1|Ca_	superfamily	261517	256	322	1.36633e-13	65.2966	cl02930	Cation_ATPase_N superfamily	-
Q#711 - >Ca_08111.1|Ca_	multi-dom	256504	157	260	6.13788e-05	41.519	pfam11559	ADIP	-
Q#712 - >Ca_08115.1|Ca_	specific	258178	33	59	2.54613e-05	40.2888	pfam13912	zf-C2H2_6	cl16471
Q#712 - >Ca_08115.1|Ca_	superfamily	258178	33	59	2.54613e-05	40.2888	cl16471	zf-C2H2_6 superfamily	-
Q#713 - >Ca_08118.1|Ca_	superfamily	261455	84	142	1.44784e-19	83.0285	cl02808	RT_like superfamily	-
Q#714 - >Ca_08119.1|Ca_	superfamily	276194	64	100	1.36934e-07	48.0595	cl17173	AdoMet_MTases superfamily	-
Q#718 - >Ca_08177.1|Ca_	superfamily	241616	2	84	1.50985e-13	62.2775	cl00109	MADS superfamily	-
Q#718 - >Ca_08177.1|Ca_	superfamily	276299	91	136	0.00156539	36.0977	cl21455	ABC_ATPase superfamily	-
Q#719 - >Ca_08226.1|Ca_	superfamily	276297	1	91	3.82637e-45	156.23	cl21453	PKc_like superfamily	-
Q#719 - >Ca_08226.1|Ca_	superfamily	276297	157	212	1.23207e-32	122.718	cl21453	PKc_like superfamily	-
Q#721 - >Ca_08229.1|Ca_	superfamily	271609	203	295	0.00424301	36.5159	cl03075	GrpE superfamily	-
Q#722 - >Ca_08244.1|Ca_	superfamily	260890	119	199	4.04324e-23	94.5112	cl01342	Peptidase_A22B superfamily	-
Q#722 - >Ca_08244.1|Ca_	superfamily	271849	32	106	1.88359e-19	83.0415	cl19250	Sulfate_transp superfamily	-
Q#723 - >Ca_08248.1|Ca_	superfamily	271754	177	247	1.87739e-12	64.0509	cl14782	RNase_H_like superfamily	-
Q#723 - >Ca_08248.1|Ca_	superfamily	261455	136	166	0.000141922	41.4269	cl02808	RT_like superfamily	-
Q#728 - >Ca_08314.1|Ca_	superfamily	252133	99	198	5.67843e-12	59.2745	cl04237	Retrotrans_gag superfamily	-
Q#731 - >Ca_08327.1|Ca_	specific	185450	2	107	1.02533e-63	191.059	PTZ00106	PTZ00106	cl00600
Q#731 - >Ca_08327.1|Ca_	superfamily	275633	2	107	1.02533e-63	191.059	cl00600	Ribosomal_L7Ae superfamily	-
Q#732 - >Ca_08333.1|Ca_	multi-dom	215445	81	114	1.07481e-12	62.8005	PLN02831	PLN02831	-
Q#734 - >Ca_08338.1|Ca_	multi-dom	178496	62	94	8.87558e-05	38.599	PLN02908	PLN02908	-
Q#736 - >Ca_08382.1|Ca_	superfamily	260576	23	90	0.000127307	38.7488	cl00693	CM_2 superfamily	-
Q#736 - >Ca_08382.1|Ca_	superfamily	147916	119	189	0.00590783	33.9954	cl19316	Chordopox_A30L superfamily	-
Q#736 - >Ca_08382.1|Ca_	multi-dom	177978	8	260	9.69278e-136	387.042	PLN02344	PLN02344	-
Q#737 - >Ca_08414.1|Ca_	specific	239904	6	65	1.41962e-26	92.6983	cd04457	S1_S28E	cl09927
Q#737 - >Ca_08414.1|Ca_	superfamily	275904	6	65	1.41962e-26	92.6983	cl09927	S1_like superfamily	-
Q#738 - >Ca_08421.1|Ca_	superfamily	275485	63	133	0.00184899	39.1005	cl00210	Isoprenoid_Biosyn_C1 superfamily	-
Q#739 - >Ca_08428.1|Ca_	superfamily	276297	7	99	1.48873e-32	114.867	cl21453	PKc_like superfamily	-
Q#742 - >Ca_08493.1|Ca_	superfamily	276034	5	58	6.52274e-26	102.826	cl13995	MPP_superfamily superfamily	-
Q#743 - >Ca_08537.1|Ca_	specific	253452	250	445	3.31612e-89	275.019	pfam05910	DUF868	cl05466

Query	Types	PSSM-ID	From	To	E-Value	Bitscore	Accession	Short name	Superfamily
Q#743 - >Ca_08537.1|Ca_	superfamily	253452	250	445	3.31612e-89	275.019	cl05466	DUF868 superfamily	-
Q#743 - >Ca_08537.1|Ca_	specific	253452	40	220	1.21064e-81	255.373	pfam05910	DUF868	cl05466
Q#743 - >Ca_08537.1|Ca_	superfamily	253452	40	220	1.21064e-81	255.373	cl05466	DUF868 superfamily	-
Q#744 - >Ca_08543.1|Ca_	superfamily	271453	13	84	4.27304e-15	66.4864	cl00269	cytidine_deaminase-like superfamily	-
Q#748 - >Ca_08779.1|Ca_	superfamily	205348	144	210	2.82929e-09	51.716	cl16145	GTP-bdg_N superfamily	-
Q#749 - >Ca_08782.1|Ca_	superfamily	275927	87	192	4.34551e-11	59.3612	cl11403	pepsin_retropepsin_like superfamily	-
Q#750 - >Ca_08806.1|Ca_	superfamily	256046	81	227	2.58686e-18	82.8276	cl11168	PMD superfamily	-
Q#751 - >Ca_08808.1|Ca_	superfamily	276299	1	107	3.50037e-30	108.491	cl21455	ABC_ATPase superfamily	-
Q#752 - >Ca_08822.1|Ca_	specific	197383	26	115	2.29799e-18	77.3681	cd10017	B3_DNA	cl15242
Q#752 - >Ca_08822.1|Ca_	superfamily	265652	26	115	2.29799e-18	77.3681	cl15242	BfiI_C_EcoRII_N_B3 superfamily	-
Q#753 - >Ca_08826.1|Ca_	superfamily	276194	133	180	3.43646e-05	41.1422	cl17173	AdoMet_MTases superfamily	-
Q#754 - >Ca_08827.1|Ca_	superfamily	276193	77	127	1.60025e-28	102.647	cl17171	PH-like superfamily	-
Q#755 - >Ca_08840.1|Ca_	specific	238287	9	186	2.40036e-32	117.577	cd00519	Lipase_3	cl21494
Q#755 - >Ca_08840.1|Ca_	superfamily	276326	9	186	2.40036e-32	117.577	cl21494	Esterase_lipase superfamily	-
Q#756 - >Ca_08849.1|Ca_	superfamily	275625	111	175	0.00746701	35.3185	cl00555	SAF superfamily	-
Q#757 - >Ca_08867.1|Ca_	specific	258132	191	378	2.45708e-58	190.883	pfam13862	BCIP	cl16439
Q#757 - >Ca_08867.1|Ca_	superfamily	258132	191	378	2.45708e-58	190.883	cl16439	BCIP superfamily	-
Q#758 - >Ca_08894.1|Ca_	specific	258478	101	268	2.65098e-72	221.288	pfam14299	PP2	cl16784
Q#758 - >Ca_08894.1|Ca_	superfamily	258478	101	268	2.65098e-72	221.288	cl16784	PP2 superfamily	-
Q#758 - >Ca_08894.1|Ca_	specific	250027	4	48	8.26197e-06	41.8066	pfam00646	F-box	cl02535
Q#758 - >Ca_08894.1|Ca_	superfamily	261329	4	48	8.26197e-06	41.8066	cl02535	F-box superfamily	-
Q#759 - >Ca_08919.1|Ca_	superfamily	275467	33	139	1.09773e-53	167.175	cl00074	H2A superfamily	-
Q#760 - >Ca_08946.1|Ca_	specific	250208	112	298	2.43623e-82	251.4	pfam00891	Methyltransf_2	cl17173
Q#760 - >Ca_08946.1|Ca_	superfamily	276194	112	298	2.43623e-82	251.4	cl17173	AdoMet_MTases superfamily	-
Q#760 - >Ca_08946.1|Ca_	specific	254619	39	94	1.35873e-09	52.9742	pfam08100	Dimerization	cl06920
Q#760 - >Ca_08946.1|Ca_	superfamily	254619	39	94	1.35873e-09	52.9742	cl06920	Dimerization superfamily	-
Q#761 - >Ca_09007.1|Ca_	specific	185442	7	149	7.30551e-91	263.038	PTZ00096	PTZ00096	cl00350
Q#761 - >Ca_09007.1|Ca_	superfamily	275544	7	149	7.30551e-91	263.038	cl00350	Ribosomal_S19 superfamily	-
Q#762 - >Ca_09011.1|Ca_	specific	256943	63	134	5.87879e-28	105.779	pfam12265	CAF1C_H4-bd	cl13671
Q#762 - >Ca_09011.1|Ca_	superfamily	256943	63	134	5.87879e-28	105.779	cl13671	CAF1C_H4-bd superfamily	-
Q#762 - >Ca_09011.1|Ca_	superfamily	271593	155	444	4.90631e-27	108.962	cl02567	WD40 superfamily	-
Q#762 - >Ca_09011.1|Ca_	multi-dom	225201	152	448	4.95516e-22	97.0815	COG2319	COG2319	-
Q#764 - >Ca_09014.1|Ca_	superfamily	262827	18	138	4.85511e-55	171.228	cl06243	PsbW superfamily	-
Q#766 - >Ca_09053.1|Ca_	specific	253452	23	288	1.51866e-130	374.785	pfam05910	DUF868	cl05466
Q#766 - >Ca_09053.1|Ca_	superfamily	253452	23	288	1.51866e-130	374.785	cl05466	DUF868 superfamily	-
Q#770 - >Ca_09112.1|Ca_	specific	257351	100	133	4.74569e-06	43.4921	pfam12854	PPR_1	cl03252

Query	Types	PSSM-ID	From	To	E-Value	Bitscore	Accession	Short name	Superfamily
Q#770 - >Ca_09112.1|Ca_	superfamily	275791	100	133	4.74569e-06	43.4921	cl03252	PPR superfamily	-
Q#770 - >Ca_09112.1|Ca_	multi-dom	257477	104	151	1.16486e-09	53.9458	pfam13041	PPR_2	-
Q#770 - >Ca_09112.1|Ca_	multi-dom	257477	34	83	3.28482e-06	43.9306	pfam13041	PPR_2	-
Q#771 - >Ca_09120.1|Ca_	specific	238219	171	224	9.54015e-12	58.0009	cd00371	HMA	cl00207
Q#771 - >Ca_09120.1|Ca_	superfamily	260261	171	224	9.54015e-12	58.0009	cl00207	HMA superfamily	-
Q#772 - >Ca_09125.1|Ca_	superfamily	276297	81	222	3.03226e-34	127.389	cl21453	PKc_like superfamily	-
Q#774 - >Ca_09159.1|Ca_	specific	252855	71	235	1.61376e-82	249.888	pfam04884	DUF647	cl04821
Q#774 - >Ca_09159.1|Ca_	superfamily	252855	71	235	1.61376e-82	249.888	cl04821	DUF647 superfamily	-
Q#775 - >Ca_09161.1|Ca_	specific	275443	18	117	6.45516e-19	77.4503	cd15799	PMEI-like_4	cl04375
Q#775 - >Ca_09161.1|Ca_	superfamily	275808	18	117	6.45516e-19	77.4503	cl04375	PMEI_like superfamily	-
Q#776 - >Ca_09162.1|Ca_	superfamily	275922	59	178	6.90133e-39	135.726	cl10555	Chorismate_bind superfamily	-
Q#776 - >Ca_09162.1|Ca_	superfamily	272091	38	56	7.06699e-06	44.3977	cl21671	GHMP_kinases_N superfamily	-
Q#778 - >Ca_09168.1|Ca_	specific	260004	144	282	3.79957e-76	231.588	cd09272	RNase_HI_RT_Ty1	cl14782
Q#778 - >Ca_09168.1|Ca_	superfamily	271754	144	282	3.79957e-76	231.588	cl14782	RNase_H_like superfamily	-
Q#778 - >Ca_09168.1|Ca_	specific	250040	1	45	4.89077e-05	40.7797	pfam00665	rve	cl21549
Q#778 - >Ca_09168.1|Ca_	superfamily	276355	1	45	4.89077e-05	40.7797	cl21549	rve superfamily	-
Q#779 - >Ca_09183.1|Ca_	specific	260004	321	453	2.71715e-66	218.492	cd09272	RNase_HI_RT_Ty1	cl14782
Q#779 - >Ca_09183.1|Ca_	superfamily	271754	321	453	2.71715e-66	218.492	cl14782	RNase_H_like superfamily	-
Q#779 - >Ca_09183.1|Ca_	specific	99707	188	322	1.29025e-14	72.5639	cd00009	AAA	cl21455
Q#779 - >Ca_09183.1|Ca_	superfamily	276299	188	322	1.29025e-14	72.5639	cl21455	ABC_ATPase superfamily	-
Q#779 - >Ca_09183.1|Ca_	multi-dom	223540	151	330	4.09701e-24	106.061	COG0464	SpoVK	-
Q#782 - >Ca_09192.1|Ca_	specific	202502	101	243	2.15206e-92	274.523	pfam02992	Transposase_21	cl03823
Q#782 - >Ca_09192.1|Ca_	superfamily	202502	101	243	2.15206e-92	274.523	cl03823	Transposase_21 superfamily	-
Q#783 - >Ca_09196.1|Ca_	superfamily	190526	13	46	7.69619e-07	44.901	cl07846	DBD_Tnp_Mut superfamily	-
Q#784 - >Ca_09200.1|Ca_	specific	258478	101	268	2.65098e-72	221.288	pfam14299	PP2	cl16784
Q#784 - >Ca_09200.1|Ca_	superfamily	258478	101	268	2.65098e-72	221.288	cl16784	PP2 superfamily	-
Q#784 - >Ca_09200.1|Ca_	specific	250027	4	48	8.26197e-06	41.8066	pfam00646	F-box	cl02535
Q#784 - >Ca_09200.1|Ca_	superfamily	261329	4	48	8.26197e-06	41.8066	cl02535	F-box superfamily	-
Q#786 - >Ca_09211.1|Ca_	multi-dom	223021	100	337	3.69308e-05	44.9293	PHA03247	PHA03247	-
Q#787 - >Ca_09279.1|Ca_	specific	239990	214	351	3.65948e-21	87.6815	cd04618	CBS_pair_5	cl15354
Q#787 - >Ca_09279.1|Ca_	superfamily	271773	214	351	3.65948e-21	87.6815	cl15354	CBS_pair superfamily	-
Q#787 - >Ca_09279.1|Ca_	specific	199889	24	100	3.72812e-19	81.4912	cd02859	E_set_AMPKbeta_like_N	cl09101
Q#787 - >Ca_09279.1|Ca_	superfamily	271649	24	100	3.72812e-19	81.4912	cl09101	E_set superfamily	-
Q#787 - >Ca_09279.1|Ca_	superfamily	271773	395	434	1.12101e-09	55.6958	cl15354	CBS_pair superfamily	-
Q#787 - >Ca_09279.1|Ca_	multi-dom	223591	387	435	0.000272291	39.3769	COG0517	COG0517	-
Q#788 - >Ca_09305.1|Ca_	superfamily	257165	1	126	1.54387e-43	142.703	cl13989	LdpA_C superfamily	-

Query	Types	PSSM-ID	From	To	E-Value	Bitscore	Accession	Short name	Superfamily
Q#791 - >Ca_09363.1|Ca_	superfamily	276064	35	54	1.01609e-09	54.5518	cl14812	PIN_SF superfamily	-
Q#792 - >Ca_09364.1|Ca_	specific	238088	12	139	2.09526e-26	97.2613	cd00156	REC	cl19078
Q#792 - >Ca_09364.1|Ca_	superfamily	271833	12	139	2.09526e-26	97.2613	cl19078	REC superfamily	-
Q#793 - >Ca_09374.1|Ca_	superfamily	271754	28	101	3.34029e-13	64.0509	cl14782	RNase_H_like superfamily	-
Q#797 - >Ca_09457.1|Ca_	multi-dom	258434	1	45	3.58542e-13	62.7558	pfam14244	UBN2_3	-
Q#798 - >Ca_09459.1|Ca_	multi-dom	258434	48	110	6.37335e-17	71.2302	pfam14244	UBN2_3	-
Q#799 - >Ca_09481.1|Ca_	superfamily	275737	66	101	1.09771e-05	40.9806	cl01629	TPP_enzymes superfamily	-
Q#800 - >Ca_09505.1|Ca_	superfamily	252855	37	98	5.09121e-09	50.3548	cl04821	DUF647 superfamily	-
Q#801 - >Ca_09506.1|Ca_	specific	197383	101	206	6.59375e-16	70.0493	cd10017	B3_DNA	cl15242
Q#801 - >Ca_09506.1|Ca_	superfamily	265652	101	206	6.59375e-16	70.0493	cl15242	BfiI_C_EcoRII_N_B3 superfamily	-
Q#802 - >Ca_09511.1|Ca_	superfamily	275576	6	216	3.50327e-47	160.25	cl00437	Zip superfamily	-
Q#804 - >Ca_09537.1|Ca_	superfamily	275475	15	70	1.63829e-09	50.3704	cl00154	UBCc superfamily	-
Q#805 - >Ca_09559.1|Ca_	specific	250692	158	188	9.82547e-06	40.9001	pfam01535	PPR	cl03252
Q#805 - >Ca_09559.1|Ca_	superfamily	275791	158	188	9.82547e-06	40.9001	cl03252	PPR superfamily	-
Q#805 - >Ca_09559.1|Ca_	specific	273253	57	90	9.63933e-05	38.2051	TIGR00756	PPR	cl03252
Q#805 - >Ca_09559.1|Ca_	superfamily	275791	57	90	9.63933e-05	38.2051	cl03252	PPR superfamily	-
Q#805 - >Ca_09559.1|Ca_	multi-dom	257477	155	205	8.795e-09	49.3234	pfam13041	PPR_2	-
Q#808 - >Ca_09590.1|Ca_	specific	257687	331	488	5.36847e-40	142.771	pfam13359	DDE_Tnp_4	cl21562
Q#808 - >Ca_09590.1|Ca_	superfamily	271982	331	488	5.36847e-40	142.771	cl21562	DDE_Tnp_1_6 superfamily	-
Q#809 - >Ca_09686.1|Ca_	superfamily	276015	1	186	2.60145e-61	191.026	cl12022	Ribosomal_L18e superfamily	-
Q#810 - >Ca_09687.1|Ca_	superfamily	276015	1	186	2.60145e-61	191.026	cl12022	Ribosomal_L18e superfamily	-
Q#811 - >Ca_09725.1|Ca_	specific	237985	6	64	2.22195e-34	116.987	cd00018	AP2	cl00033
Q#811 - >Ca_09725.1|Ca_	superfamily	260146	6	64	2.22195e-34	116.987	cl00033	AP2 superfamily	-
Q#811 - >Ca_09725.1|Ca_	superfamily	275487	54	128	0.00407239	35.2196	cl00215	Aconitase_swivel superfamily	-
Q#812 - >Ca_09732.1|Ca_	superfamily	276218	5	102	1.03511e-24	93.9739	cl18951	GGCT_like superfamily	-
Q#814 - >Ca_09764.1|Ca_	superfamily	276194	1	189	3.75027e-74	232.912	cl17173	AdoMet_MTases superfamily	-
Q#817 - >Ca_09773.1|Ca_	superfamily	276194	192	276	1.86038e-25	105.411	cl17173	AdoMet_MTases superfamily	-
Q#819 - >Ca_09815.1|Ca_	specific	176653	19	194	7.75176e-33	117.294	cd06141	WRN_exo	cl10012
Q#819 - >Ca_09815.1|Ca_	superfamily	271686	19	194	7.75176e-33	117.294	cl10012	DnaQ_like_exo superfamily	-
Q#820 - >Ca_09816.1|Ca_	superfamily	271686	46	176	8.25434e-20	83.3967	cl10012	DnaQ_like_exo superfamily	-
Q#821 - >Ca_09817.1|Ca_	superfamily	271686	53	167	2.4418e-11	57.5883	cl10012	DnaQ_like_exo superfamily	-
Q#822 - >Ca_09840.1|Ca_	specific	173962	28	118	1.19718e-38	127.647	cd07978	TAF13	cl02174
Q#822 - >Ca_09840.1|Ca_	superfamily	242920	28	118	1.19718e-38	127.647	cl02174	TAF13 superfamily	-
Q#824 - >Ca_09851.1|Ca_	specific	214487	91	227	4.95348e-24	94.2858	smart00046	DAGKc	cl01255
Q#824 - >Ca_09851.1|Ca_	superfamily	271550	91	227	4.95348e-24	94.2858	cl01255	DAGK_cat superfamily	-
Q#825 - >Ca_09858.1|Ca_	specific	250692	101	128	0.000101484	38.2037	pfam01535	PPR	cl03252

Query	Types	PSSM-ID	From	To	E-Value	Bitscore	Accession	Short name	Superfamily
Q#825 - >Ca_09858.1|Ca_	superfamily	275791	101	128	0.000101484	38.2037	cl03252	PPR superfamily	-
Q#825 - >Ca_09858.1|Ca_	specific	250692	7	37	0.00131608	35.1221	pfam01535	PPR	cl03252
Q#825 - >Ca_09858.1|Ca_	superfamily	275791	7	37	0.00131608	35.1221	cl03252	PPR superfamily	-
Q#825 - >Ca_09858.1|Ca_	superfamily	276297	35	62	0.00721883	35.1422	cl21453	PKc_like superfamily	-
Q#825 - >Ca_09858.1|Ca_	multi-dom	257477	35	79	4.38073e-07	44.701	pfam13041	PPR_2	-
Q#825 - >Ca_09858.1|Ca_	multi-dom	257477	7	49	2.83249e-05	40.0786	pfam13041	PPR_2	-
Q#825 - >Ca_09858.1|Ca_	multi-dom	257477	169	211	0.000250911	37.3822	pfam13041	PPR_2	-
Q#825 - >Ca_09858.1|Ca_	multi-dom	257477	66	110	0.000385055	36.6118	pfam13041	PPR_2	-
Q#826 - >Ca_09878.1|Ca_	specific	212470	2	77	1.63407e-59	181.235	cd01723	LSm4	cl00259
Q#826 - >Ca_09878.1|Ca_	superfamily	275503	2	77	1.63407e-59	181.235	cl00259	Sm_like superfamily	-
Q#827 - >Ca_09906.1|Ca_	specific	238705	4	167	1.87499e-74	224.537	cd01417	Ribosomal_L19e_E	cl00232
Q#827 - >Ca_09906.1|Ca_	superfamily	260284	4	167	1.87499e-74	224.537	cl00232	Ribosomal_L19e superfamily	-
Q#831 - >Ca_10029.1|Ca_	superfamily	276297	88	247	4.27201e-60	192.873	cl21453	PKc_like superfamily	-
Q#835 - >Ca_10099.1|Ca_	superfamily	276263	144	338	3.0488e-24	99.3559	cl19514	FBA_1 superfamily	-
Q#835 - >Ca_10099.1|Ca_	specific	250027	48	90	1.28045e-07	48.3549	pfam00646	F-box	cl02535
Q#835 - >Ca_10099.1|Ca_	superfamily	261329	48	90	1.28045e-07	48.3549	cl02535	F-box superfamily	-
Q#836 - >Ca_10100.1|Ca_	superfamily	276263	143	337	4.52548e-23	95.8891	cl19514	FBA_1 superfamily	-
Q#836 - >Ca_10100.1|Ca_	specific	197608	48	84	2.34565e-08	50.1278	smart00256	FBOX	cl02535
Q#836 - >Ca_10100.1|Ca_	superfamily	261329	48	84	2.34565e-08	50.1278	cl02535	F-box superfamily	-
Q#837 - >Ca_10157.1|Ca_	superfamily	276297	21	140	8.84148e-21	85.7874	cl21453	PKc_like superfamily	-
Q#839 - >Ca_10172.1|Ca_	superfamily	265821	31	80	8.88677e-06	42.9449	cl15874	UBN2 superfamily	-
Q#841 - >Ca_10242.1|Ca_	superfamily	251669	162	302	1.48671e-08	52.0316	cl03830	Transposase_24 superfamily	-
Q#842 - >Ca_10262.1|Ca_	superfamily	275485	52	133	0.00126881	39.1005	cl00210	Isoprenoid_Biosyn_C1 superfamily	-
Q#844 - >Ca_10340.1|Ca_	superfamily	276192	45	146	4.76353e-21	82.276	cl17169	RRM_SF superfamily	-
Q#844 - >Ca_10340.1|Ca_	multi-dom	214636	45	109	8.52039e-16	68.3891	smart00360	RRM	-
Q#847 - >Ca_10371.1|Ca_	specific	214654	35	97	5.22917e-30	104.676	smart00415	HSF	cl12113
Q#847 - >Ca_10371.1|Ca_	superfamily	264552	35	97	5.22917e-30	104.676	cl12113	HSF_DNA-bind superfamily	-
Q#848 - >Ca_10389.1|Ca_	specific	258215	13	40	9.5522e-08	45.396	pfam13963	Transpos_assoc	cl16504
Q#848 - >Ca_10389.1|Ca_	superfamily	258215	13	40	9.5522e-08	45.396	cl16504	Transpos_assoc superfamily	-
Q#850 - >Ca_10420.1|Ca_	specific	250801	98	242	9.30103e-44	148.525	pfam01694	Rhomboid	cl21536
Q#850 - >Ca_10420.1|Ca_	superfamily	276350	98	242	9.30103e-44	148.525	cl21536	Rhomboid superfamily	-
Q#851 - >Ca_10436.1|Ca_	multi-dom	274008	43	176	5.59507e-05	41.5807	TIGR02168	Chromosome_partition_protein_Smc	-
Q#852 - >Ca_10470.1|Ca_	superfamily	276298	1	197	2.49512e-74	227.329	cl21454	SDR superfamily	-
Q#852 - >Ca_10470.1|Ca_	multi-dom	273001	1	215	4.33201e-53	173.485	TIGR00292	Putative_ribose_15-bisphosphate_isomerase	-
Q#853 - >Ca_10471.1|Ca_	superfamily	276298	52	155	2.00416e-36	126.792	cl21454	SDR superfamily	-
Q#854 - >Ca_10475.1|Ca_	superfamily	276298	1	70	1.03997e-24	92.8941	cl21454	SDR superfamily	-

Query	Types	PSSM-ID	From	To	E-Value	Bitscore	Accession	Short name	Superfamily
Q#855 - >Ca_10476.1|Ca_	superfamily	276298	1	204	5.67269e-77	234.262	cl21454	SDR superfamily	-
Q#855 - >Ca_10476.1|Ca_	multi-dom	273001	1	222	3.33134e-55	179.263	TIGR00292	Putative_ribose_15-bisphosphate_isomerase	-
Q#856 - >Ca_10478.1|Ca_	specific	249708	10	70	3.7762e-10	53.3978	pfam00240	ubiquitin	cl00155
Q#856 - >Ca_10478.1|Ca_	superfamily	275476	10	70	3.7762e-10	53.3978	cl00155	UBQ superfamily	-
Q#857 - >Ca_10479.1|Ca_	specific	176364	16	57	1.42428e-06	44.1802	cd01769	UBL	cl00155
Q#857 - >Ca_10479.1|Ca_	superfamily	275476	16	57	1.42428e-06	44.1802	cl00155	UBQ superfamily	-
Q#858 - >Ca_10495.1|Ca_	superfamily	276298	1	70	1.03997e-24	92.8941	cl21454	SDR superfamily	-
Q#859 - >Ca_10496.1|Ca_	superfamily	276298	1	204	5.67269e-77	234.262	cl21454	SDR superfamily	-
Q#859 - >Ca_10496.1|Ca_	multi-dom	273001	1	222	3.33134e-55	179.263	TIGR00292	Putative_ribose_15-bisphosphate_isomerase	-
Q#860 - >Ca_10498.1|Ca_	specific	249708	10	70	3.7762e-10	53.3978	pfam00240	ubiquitin	cl00155
Q#860 - >Ca_10498.1|Ca_	superfamily	275476	10	70	3.7762e-10	53.3978	cl00155	UBQ superfamily	-
Q#861 - >Ca_10499.1|Ca_	specific	176364	16	57	1.42428e-06	44.1802	cd01769	UBL	cl00155
Q#861 - >Ca_10499.1|Ca_	superfamily	275476	16	57	1.42428e-06	44.1802	cl00155	UBQ superfamily	-
Q#862 - >Ca_10504.1|Ca_	superfamily	276298	22	223	6.47687e-69	213.462	cl21454	SDR superfamily	-
Q#862 - >Ca_10504.1|Ca_	multi-dom	273001	18	224	8.26172e-42	144.209	TIGR00292	Putative_ribose_15-bisphosphate_isomerase	-
Q#863 - >Ca_10510.1|Ca_	superfamily	276298	22	253	8.45042e-88	263.923	cl21454	SDR superfamily	-
Q#863 - >Ca_10510.1|Ca_	multi-dom	273001	18	271	4.13233e-62	198.908	TIGR00292	Putative_ribose_15-bisphosphate_isomerase	-
Q#864 - >Ca_10551.1|Ca_	superfamily	241616	22	81	4.82397e-22	85.2965	cl00109	MADS superfamily	-
Q#865 - >Ca_10552.1|Ca_	specific	238165	9	77	1.77106e-33	117.268	cd00265	MADS_MEF2_like	cl00109
Q#865 - >Ca_10552.1|Ca_	superfamily	241616	9	77	1.77106e-33	117.268	cl00109	MADS superfamily	-
Q#866 - >Ca_10553.1|Ca_	specific	238165	9	78	2.17885e-35	121.891	cd00265	MADS_MEF2_like	cl00109
Q#866 - >Ca_10553.1|Ca_	superfamily	241616	9	78	2.17885e-35	121.891	cl00109	MADS superfamily	-
Q#867 - >Ca_10554.1|Ca_	specific	238165	9	77	8.09544e-36	123.431	cd00265	MADS_MEF2_like	cl00109
Q#867 - >Ca_10554.1|Ca_	superfamily	241616	9	77	8.09544e-36	123.431	cl00109	MADS superfamily	-
Q#868 - >Ca_10555.1|Ca_	specific	238165	9	77	2.62138e-32	114.187	cd00265	MADS_MEF2_like	cl00109
Q#868 - >Ca_10555.1|Ca_	superfamily	241616	9	77	2.62138e-32	114.187	cl00109	MADS superfamily	-
Q#869 - >Ca_10558.1|Ca_	superfamily	276298	5	221	2.99973e-86	258.53	cl21454	SDR superfamily	-
Q#870 - >Ca_10573.1|Ca_	specific	252603	17	218	1.70345e-80	242.201	pfam04450	BSP	cl14813
Q#870 - >Ca_10573.1|Ca_	superfamily	276065	17	218	1.70345e-80	242.201	cl14813	GluZincin superfamily	-
Q#873 - >Ca_10739.1|Ca_	superfamily	276193	105	211	4.71116e-56	175.994	cl17171	PH-like superfamily	-
Q#877 - >Ca_10824.1|Ca_	specific	258415	1	77	1.08617e-17	76.4572	pfam14223	UBN2	cl15874
Q#877 - >Ca_10824.1|Ca_	superfamily	265821	1	77	1.08617e-17	76.4572	cl15874	UBN2 superfamily	-
Q#881 - >Ca_10853.1|Ca_	superfamily	276192	18	78	1.13984e-16	68.4766	cl17169	RRM_SF superfamily	-
Q#881 - >Ca_10853.1|Ca_	multi-dom	214636	17	78	6.61123e-15	63.7667	smart00360	RRM	-
Q#883 - >Ca_10878.1|Ca_	specific	238034	103	225	2.29423e-28	106.553	cd00079	HELICc	cl21455
Q#883 - >Ca_10878.1|Ca_	superfamily	276299	103	225	2.29423e-28	106.553	cl21455	ABC_ATPase superfamily	-

Query	Types	PSSM-ID	From	To	E-Value	Bitscore	Accession	Short name	Superfamily
Q#885 - >Ca_10912.1|Ca_	specific	253333	126	209	3.09741e-12	60.7645	pfam05699	Dimer_Tnp_hAT	cl05324
Q#885 - >Ca_10912.1|Ca_	superfamily	253333	126	209	3.09741e-12	60.7645	cl05324	Dimer_Tnp_hAT superfamily	-
Q#885 - >Ca_10912.1|Ca_	superfamily	203127	1	41	1.73703e-07	48.4339	cl04853	DUF659 superfamily	-
Q#887 - >Ca_10948.1|Ca_	superfamily	265434	2	70	3.76848e-11	55.2987	cl14616	Cyt_b561 superfamily	-
Q#888 - >Ca_10954.1|Ca_	specific	153419	79	270	3.37673e-117	337.065	cd07914	IGPD	cl00341
Q#888 - >Ca_10954.1|Ca_	superfamily	260369	79	270	3.37673e-117	337.065	cl00341	IGPD superfamily	-
Q#888 - >Ca_10954.1|Ca_	multi-dom	215430	43	272	7.33537e-136	387.267	PLN02800	PLN02800	-
Q#892 - >Ca_10981.1|Ca_	superfamily	276020	2	71	6.4616e-05	38.6418	cl12078	p450 superfamily	-
Q#893 - >Ca_10994.1|Ca_	specific	252073	33	111	2.15564e-37	128.552	pfam03634	TCP	cl15990
Q#893 - >Ca_10994.1|Ca_	superfamily	252073	33	111	2.15564e-37	128.552	cl15990	TCP superfamily	-
Q#894 - >Ca_10999.1|Ca_	superfamily	256046	120	186	0.000160182	41.226	cl11168	PMD superfamily	-
Q#895 - >Ca_11000.1|Ca_	multi-dom	178496	45	77	0.00218877	33.9766	PLN02908	PLN02908	-
Q#896 - >Ca_11006.1|Ca_	specific	238125	23	138	7.72944e-33	114.403	cd00204	ANK	cl02529
Q#896 - >Ca_11006.1|Ca_	superfamily	275769	23	138	7.72944e-33	114.403	cl02529	ANK superfamily	-
Q#896 - >Ca_11006.1|Ca_	multi-dom	257303	24	116	1.17609e-24	92.319	pfam12796	Ank_2	-
Q#897 - >Ca_11010.1|Ca_	superfamily	252133	36	134	1.84692e-09	51.5705	cl04237	Retrotrans_gag superfamily	-
Q#898 - >Ca_11067.1|Ca_	superfamily	190526	259	301	0.00396017	35.6562	cl07846	DBD_Tnp_Mut superfamily	-
Q#898 - >Ca_11067.1|Ca_	superfamily	276269	343	383	0.00482511	35.5145	cl19537	Transposase_mut superfamily	-
Q#900 - >Ca_11093.1|Ca_	specific	257687	97	263	1.03803e-33	122.355	pfam13359	DDE_Tnp_4	cl21562
Q#900 - >Ca_11093.1|Ca_	superfamily	271982	97	263	1.03803e-33	122.355	cl21562	DDE_Tnp_1_6 superfamily	-
Q#900 - >Ca_11093.1|Ca_	superfamily	257926	14	64	0.00141076	35.8294	cl16321	HTH_Tnp_4 superfamily	-
Q#901 - >Ca_11101.1|Ca_	superfamily	252977	1	236	1.63938e-30	116.854	cl04913	DUF674 superfamily	-
Q#902 - >Ca_11110.1|Ca_	multi-dom	181749	7	65	0.00643269	33.3461	PRK09276	PRK09276	-
Q#903 - >Ca_11112.1|Ca_	superfamily	275927	86	317	1.58608e-33	125.068	cl11403	pepsin_retropepsin_like superfamily	-
Q#904 - >Ca_11138.1|Ca_	superfamily	256046	65	195	4.26216e-07	47.7744	cl11168	PMD superfamily	-
Q#905 - >Ca_11141.1|Ca_	superfamily	276020	1	285	8.66939e-53	181.163	cl12078	p450 superfamily	-
Q#906 - >Ca_11151.1|Ca_	superfamily	271600	16	147	4.74588e-27	103.129	cl02666	KU superfamily	-
Q#907 - >Ca_11152.1|Ca_	superfamily	190729	8	80	5.83538e-10	52.0298	cl04236	Ku_C superfamily	-
Q#907 - >Ca_11152.1|Ca_	specific	251054	104	137	1.1043e-06	41.5988	pfam02037	SAP	cl02640
Q#907 - >Ca_11152.1|Ca_	superfamily	261387	104	137	1.1043e-06	41.5988	cl02640	SAP superfamily	-
Q#908 - >Ca_11165.1|Ca_	superfamily	271593	17	301	1.24262e-24	101.258	cl02567	WD40 superfamily	-
Q#908 - >Ca_11165.1|Ca_	multi-dom	225201	3	324	2.20886e-17	82.8291	COG2319	COG2319	-
Q#909 - >Ca_11189.1|Ca_	multi-dom	177618	161	268	0.000550748	39.6088	PHA03381	PHA03381	-
Q#910 - >Ca_11207.1|Ca_	superfamily	256046	98	368	7.09414e-42	152.549	cl11168	PMD superfamily	-
Q#913 - >Ca_11241.1|Ca_	specific	147159	15	250	2.15464e-87	265.267	pfam04857	CAF1	cl10012
Q#913 - >Ca_11241.1|Ca_	superfamily	271686	15	250	2.15464e-87	265.267	cl10012	DnaQ_like_exo superfamily	-

Query	Types	PSSM-ID	From	To	E-Value	Bitscore	Accession	Short name	Superfamily
Q#914 - >Ca_11247.1|Ca_	superfamily	271592	172	290	2.74349e-35	130.529	cl02553	Peptidase_C19 superfamily	-
Q#914 - >Ca_11247.1|Ca_	superfamily	252977	3	71	0.000825033	39.4286	cl04913	DUF674 superfamily	-
Q#915 - >Ca_11253.1|Ca_	specific	251772	59	162	9.84023e-09	49.672	pfam03168	LEA_2	cl12118
Q#915 - >Ca_11253.1|Ca_	superfamily	264557	59	162	9.84023e-09	49.672	cl12118	LEA_2 superfamily	-
Q#918 - >Ca_11274.1|Ca_	superfamily	276192	28	97	1.71033e-38	128.435	cl17169	RRM_SF superfamily	-
Q#918 - >Ca_11274.1|Ca_	multi-dom	214636	28	93	1.73781e-16	70.7003	smart00360	RRM	-
Q#919 - >Ca_11276.1|Ca_	superfamily	276301	45	248	7.17855e-115	333.532	cl21457	TIM_phosphate_binding superfamily	-
Q#923 - >Ca_11288.1|Ca_	multi-dom	173561	120	165	0.00293285	35.5529	PTZ00368	PTZ00368	-
Q#923 - >Ca_11288.1|Ca_	multi-dom	227414	148	191	0.00910949	34.4412	COG5082	AIR1	-
Q#928 - >Ca_11325.1|Ca_	specific	173823	1	46	8.3443e-10	52.541	cd00314	plant_peroxidase_like	cl00196
Q#928 - >Ca_11325.1|Ca_	superfamily	260251	1	46	8.3443e-10	52.541	cl00196	plant_peroxidase_like superfamily	-
Q#930 - >Ca_11327.1|Ca_	superfamily	275927	78	349	5.97566e-32	121.986	cl11403	pepsin_retropepsin_like superfamily	-
Q#930 - >Ca_11327.1|Ca_	superfamily	275927	80	174	6.16978e-08	50.0711	cl11403	pepsin_retropepsin_like superfamily	-
Q#930 - >Ca_11327.1|Ca_	multi-dom	249520	77	343	1.73984e-23	98.1302	pfam00026	Asp	-
Q#931 - >Ca_11328.1|Ca_	specific	252656	31	169	2.88325e-29	107.395	pfam04535	DUF588	cl04571
Q#931 - >Ca_11328.1|Ca_	superfamily	275812	31	169	2.88325e-29	107.395	cl04571	MARVEL superfamily	-
Q#932 - >Ca_11339.1|Ca_	specific	147159	15	250	7.59773e-88	264.112	pfam04857	CAF1	cl10012
Q#932 - >Ca_11339.1|Ca_	superfamily	271686	15	250	7.59773e-88	264.112	cl10012	DnaQ_like_exo superfamily	-
Q#938 - >Ca_11395.1|Ca_	multi-dom	215061	1	126	1.05965e-12	64.0988	PLN00113	PLN00113	-
Q#939 - >Ca_11396.1|Ca_	specific	238143	70	278	1.93926e-54	177.113	cd00232	HemeO	cl15243
Q#939 - >Ca_11396.1|Ca_	superfamily	276101	70	278	1.93926e-54	177.113	cl15243	HemeO superfamily	-
Q#940 - >Ca_11397.1|Ca_	superfamily	261455	276	329	3.28389e-13	65.6945	cl02808	RT_like superfamily	-
Q#940 - >Ca_11397.1|Ca_	superfamily	252133	82	150	5.02923e-06	43.8665	cl04237	Retrotrans_gag superfamily	-
Q#941 - >Ca_11401.1|Ca_	specific	258415	104	220	7.47981e-34	120.755	pfam14223	UBN2	cl15874
Q#941 - >Ca_11401.1|Ca_	superfamily	265821	104	220	7.47981e-34	120.755	cl15874	UBN2 superfamily	-
Q#942 - >Ca_11419.1|Ca_	superfamily	276007	148	307	4.57758e-51	175.315	cl11961	ALDH-SF superfamily	-
Q#942 - >Ca_11419.1|Ca_	superfamily	276282	1	46	5.31722e-19	81.5037	cl19911	CBM_4_9 superfamily	-
Q#946 - >Ca_11451.1|Ca_	superfamily	276235	147	218	3.28277e-17	78.7673	cl19188	Pectate_lyase_3 superfamily	-
Q#947 - >Ca_11453.1|Ca_	specific	197667	121	137	0.00279985	33.1856	smart00343	ZnF_C2HC	cl22700
Q#947 - >Ca_11453.1|Ca_	superfamily	276654	121	137	0.00279985	33.1856	cl22700	zf-CCHC superfamily	-
Q#949 - >Ca_11455.1|Ca_	superfamily	276214	22	105	4.21793e-36	126.153	cl18310	NHL superfamily	-
Q#952 - >Ca_11466.1|Ca_	multi-dom	273330	38	120	6.85207e-11	57.9026	TIGR00906	Low_affinity_cationic_amino_acid_transporter_2	-
Q#955 - >Ca_11478.1|Ca_	superfamily	275481	89	118	0.00479095	33.9004	cl00192	ribokinase_pfkB_like superfamily	-
Q#956 - >Ca_11481.1|Ca_	superfamily	275927	132	178	3.80832e-05	41.2511	cl11403	pepsin_retropepsin_like superfamily	-
Q#957 - >Ca_11483.1|Ca_	specific	214842	144	208	1.0996e-15	69.919	smart00831	Cation_ATPase_N	cl02930
Q#957 - >Ca_11483.1|Ca_	superfamily	261517	144	208	1.0996e-15	69.919	cl02930	Cation_ATPase_N superfamily	-

Query	Types	PSSM-ID	From	To	E-Value	Bitscore	Accession	Short name	Superfamily
Q#959 - >Ca_11501.1|Ca_	multi-dom	223021	261	359	7.22167e-07	50.3221	PHA03247	PHA03247	-
Q#961 - >Ca_11504.1|Ca_	superfamily	253224	62	103	0.00822675	32.9112	cl05191	DMAP1 superfamily	-
Q#961 - >Ca_11504.1|Ca_	superfamily	258949	89	119	0.00971398	31.5459	cl20728	PBP1_TM superfamily	-
Q#962 - >Ca_11507.1|Ca_	superfamily	252133	100	161	0.000218508	37.3181	cl04237	Retrotrans_gag superfamily	-
Q#963 - >Ca_11512.1|Ca_	superfamily	276194	43	104	0.00174046	34.9034	cl17173	AdoMet_MTases superfamily	-
Q#966 - >Ca_11530.1|Ca_	specific	249696	5	30	0.000769751	35.1994	pfam00226	DnaJ	cl02542
Q#966 - >Ca_11530.1|Ca_	superfamily	271590	5	30	0.000769751	35.1994	cl02542	DnaJ superfamily	-
Q#968 - >Ca_11545.1|Ca_	specific	260004	144	241	6.25358e-42	141.837	cd09272	RNase_HI_RT_Ty1	cl14782
Q#968 - >Ca_11545.1|Ca_	superfamily	271754	144	241	6.25358e-42	141.837	cl14782	RNase_H_like superfamily	-
Q#968 - >Ca_11545.1|Ca_	superfamily	254387	1	129	4.07292e-63	200.375	cl06662	RVT_2 superfamily	-
Q#969 - >Ca_11547.1|Ca_	specific	260004	1	63	7.91489e-24	94.8425	cd09272	RNase_HI_RT_Ty1	cl14782
Q#969 - >Ca_11547.1|Ca_	superfamily	271754	1	63	7.91489e-24	94.8425	cl14782	RNase_H_like superfamily	-
Q#970 - >Ca_11548.1|Ca_	superfamily	254387	2	105	9.83276e-63	193.057	cl06662	RVT_2 superfamily	-
Q#971 - >Ca_11550.1|Ca_	superfamily	261455	214	294	9.27e-20	86.8805	cl02808	RT_like superfamily	-
Q#971 - >Ca_11550.1|Ca_	specific	250040	448	529	2.11948e-11	61.1953	pfam00665	rve	cl21549
Q#971 - >Ca_11550.1|Ca_	superfamily	276355	448	529	2.11948e-11	61.1953	cl21549	rve superfamily	-
Q#971 - >Ca_11550.1|Ca_	superfamily	252133	71	112	0.000290182	39.6293	cl04237	Retrotrans_gag superfamily	-
Q#972 - >Ca_11552.1|Ca_	multi-dom	223021	284	418	0.000841574	40.6921	PHA03247	PHA03247	-
Q#973 - >Ca_11562.1|Ca_	superfamily	256046	1	152	2.86239e-19	83.598	cl11168	PMD superfamily	-
Q#975 - >Ca_11570.1|Ca_	superfamily	276299	71	184	1.40205e-42	143.394	cl21455	ABC_ATPase superfamily	-
Q#975 - >Ca_11570.1|Ca_	superfamily	271701	1	48	1.45339e-16	72.7576	cl10459	Peptidases_S8_S53 superfamily	-
Q#976 - >Ca_11571.1|Ca_	superfamily	260768	1	114	7.2374e-15	68.2309	cl01053	SGNH_hydrolase superfamily	-
Q#977 - >Ca_11573.1|Ca_	specific	251675	25	214	1.7959e-36	131.417	pfam03016	Exostosin	cl20239
Q#977 - >Ca_11573.1|Ca_	superfamily	251675	25	214	1.7959e-36	131.417	cl20239	Exostosin superfamily	-
Q#979 - >Ca_11584.1|Ca_	superfamily	252884	222	318	0.000220391	42.7322	cl15563	Herpes_DNAp_acc superfamily	-
Q#980 - >Ca_11591.1|Ca_	specific	238825	339	455	3.95125e-53	177.788	cd01647	RT_LTR	cl02808
Q#980 - >Ca_11591.1|Ca_	superfamily	261455	339	455	3.95125e-53	177.788	cl02808	RT_like superfamily	-
Q#980 - >Ca_11591.1|Ca_	multi-dom	249567	356	455	1.32895e-17	80.4526	pfam00078	RVT_1	-
Q#981 - >Ca_11605.1|Ca_	superfamily	215546	5	85	0.00284645	35.8944	cl14573	PLN03031 superfamily	-
Q#982 - >Ca_11615.1|Ca_	superfamily	276020	12	209	2.89435e-66	213.716	cl12078	p450 superfamily	-
Q#983 - >Ca_11617.1|Ca_	specific	211392	8	106	8.88222e-37	123.399	cd11380	Ribosomal_S8e_like	cl00880
Q#983 - >Ca_11617.1|Ca_	superfamily	260676	8	106	8.88222e-37	123.399	cl00880	Ribosomal_S8e_like superfamily	-
Q#985 - >Ca_11624.1|Ca_	specific	254557	91	114	0.00751112	30.8752	pfam07974	EGF_2	cl21504
Q#985 - >Ca_11624.1|Ca_	superfamily	276330	91	114	0.00751112	30.8752	cl21504	EGF_CA superfamily	-
Q#986 - >Ca_11626.1|Ca_	specific	133136	149	239	6.55754e-20	83.1547	cd00303	retropepsin_like	cl11403
Q#986 - >Ca_11626.1|Ca_	superfamily	275927	149	239	6.55754e-20	83.1547	cl11403	pepsin_retropepsin_like superfamily	-

Query	Types	PSSM-ID	From	To	E-Value	Bitscore	Accession	Short name	Superfamily
Q#987 - >Ca_11633.1|Ca_	superfamily	252133	104	190	4.99347e-08	49.2593	cl04237	Retrotrans_gag superfamily	-
Q#989 - >Ca_11636.1|Ca_	multi-dom	223021	249	382	0.00188077	39.9217	PHA03247	PHA03247	-
Q#990 - >Ca_11638.1|Ca_	specific	238068	69	192	2.45141e-33	121.717	cd00121	MATH	cl02446
Q#990 - >Ca_11638.1|Ca_	superfamily	261289	69	192	2.45141e-33	121.717	cl02446	MATH superfamily	-
Q#991 - >Ca_11645.1|Ca_	multi-dom	223880	49	107	0.00162704	38.2281	COG0810	TonB	-
Q#992 - >Ca_11648.1|Ca_	superfamily	251669	97	172	1.60711e-09	52.4168	cl03830	Transposase_24 superfamily	-
Q#995 - >Ca_11664.1|Ca_	superfamily	251669	205	277	5.44295e-08	50.1057	cl03830	Transposase_24 superfamily	-
Q#996 - >Ca_11677.1|Ca_	specific	250639	81	155	2.30995e-18	75.2808	pfam01466	Skp1	cl03226
Q#996 - >Ca_11677.1|Ca_	superfamily	250639	81	155	2.30995e-18	75.2808	cl03226	Skp1 superfamily	-
Q#996 - >Ca_11677.1|Ca_	superfamily	271586	9	106	1.4709e-14	65.3925	cl02518	BTB superfamily	-
Q#996 - >Ca_11677.1|Ca_	multi-dom	227528	11	154	4.28136e-21	84.6104	COG5201	SKP1	-
Q#997 - >Ca_11687.1|Ca_	specific	271246	20	207	2.0581e-131	369.96	cd14867	uS7_Eukaryote	cl00313
Q#997 - >Ca_11687.1|Ca_	superfamily	275524	20	207	2.0581e-131	369.96	cl00313	uS7 superfamily	-
Q#999 - >Ca_11705.1|Ca_	specific	238093	208	252	1.66815e-10	55.1444	cd00162	RING	cl17238
Q#999 - >Ca_11705.1|Ca_	superfamily	276201	208	252	1.66815e-10	55.1444	cl17238	RING superfamily	-
Q#999 - >Ca_11705.1|Ca_	specific	258540	6	37	1.35954e-06	44.0842	pfam14369	zf-RING_3	cl16832
Q#999 - >Ca_11705.1|Ca_	superfamily	258540	6	37	1.35954e-06	44.0842	cl16832	zf-RING_3 superfamily	-
Q#999 - >Ca_11705.1|Ca_	multi-dom	257215	204	249	4.00913e-10	54.7503	pfam12678	zf-rbx1	-
Q#1000 - >Ca_11717.1|Ca_	superfamily	262404	22	155	1.85986e-17	77.4383	cl05005	TAF4 superfamily	-
Q#1001 - >Ca_11742.1|Ca_	superfamily	253284	33	124	6.18756e-43	144.452	cl05279	DUF789 superfamily	-
Q#1002 - >Ca_11756.1|Ca_	superfamily	275927	79	145	0.000201365	38.9456	cl11403	pepsin_retropepsin_like superfamily	-
Q#1003 - >Ca_11764.1|Ca_	specific	238039	26	82	3.05479e-15	67.2684	cd00086	homeodomain	cl00084
Q#1003 - >Ca_11764.1|Ca_	superfamily	275470	26	82	3.05479e-15	67.2684	cl00084	homeodomain superfamily	-
Q#1003 - >Ca_11764.1|Ca_	superfamily	276553	81	118	0.00397968	33.6371	cl22599	HALZ superfamily	-
Q#1006 - >Ca_11828.1|Ca_	superfamily	256046	65	195	4.26216e-07	47.7744	cl11168	PMD superfamily	-
Q#1007 - >Ca_11841.1|Ca_	superfamily	271792	48	74	0.00887538	33.8127	cl17040	ADDz superfamily	-
Q#1008 - >Ca_11847.1|Ca_	superfamily	271793	89	256	4.52546e-95	291.475	cl17068	AFD_class_I superfamily	-
Q#1008 - >Ca_11847.1|Ca_	superfamily	271848	16	97	1.32984e-20	89.3676	cl19241	Condensation superfamily	-
Q#1009 - >Ca_11867.1|Ca_	superfamily	261371	7	48	0.00183788	33.2473	cl02611	G-patch superfamily	-
Q#1011 - >Ca_11885.1|Ca_	superfamily	252763	1	172	3.65486e-103	304.327	cl04706	Glyco_transf_17 superfamily	-
Q#1013 - >Ca_11898.1|Ca_	specific	206656	13	239	2.66453e-92	272.647	cd01863	Rab18	cl21455
Q#1013 - >Ca_11898.1|Ca_	superfamily	276299	13	239	2.66453e-92	272.647	cl21455	ABC_ATPase superfamily	-
Q#1013 - >Ca_11898.1|Ca_	multi-dom	215587	6	273	5.26764e-111	322.005	PLN03118	PLN03118	-
Q#1018 - >Ca_11946.1|Ca_	superfamily	252133	100	219	1.48884e-05	43.8665	cl04237	Retrotrans_gag superfamily	-
Q#1018 - >Ca_11946.1|Ca_	specific	189387	326	342	0.000206832	39.7449	pfam00098	zf-CCHC	cl22700
Q#1018 - >Ca_11946.1|Ca_	superfamily	276654	326	342	0.000206832	39.7449	cl22700	zf-CCHC superfamily	-

Query	Types	PSSM-ID	From	To	E-Value	Bitscore	Accession	Short name	Superfamily
Q#1020 - >Ca_11977.1|Ca_	superfamily	276020	1	189	8.44784e-61	198.882	cl12078	p450 superfamily	-
Q#1022 - >Ca_12008.1|Ca_	superfamily	256046	62	316	7.52344e-20	87.8352	cl11168	PMD superfamily	-
Q#1023 - >Ca_12014.1|Ca_	superfamily	260768	80	145	1.2598e-16	73.8038	cl01053	SGNH_hydrolase superfamily	-
Q#1023 - >Ca_12014.1|Ca_	superfamily	260768	30	80	2.64996e-14	67.6406	cl01053	SGNH_hydrolase superfamily	-
Q#1024 - >Ca_12022.1|Ca_	specific	214587	8	142	4.63031e-30	107.794	smart00255	TIR	cl22440
Q#1024 - >Ca_12022.1|Ca_	superfamily	276394	8	142	4.63031e-30	107.794	cl22440	TIR_2 superfamily	-
Q#1026 - >Ca_12030.1|Ca_	multi-dom	172376	301	416	0.000101566	42.9672	PRK13855	PRK13855	-
Q#1029 - >Ca_12040.1|Ca_	specific	254636	3	69	2.79283e-13	60.2659	pfam08156	NOP5NT	cl06964
Q#1029 - >Ca_12040.1|Ca_	superfamily	254636	3	69	2.79283e-13	60.2659	cl06964	NOP5NT superfamily	-
Q#1030 - >Ca_12043.1|Ca_	specific	238125	167	260	4.20426e-13	66.253	cd00204	ANK	cl02529
Q#1030 - >Ca_12043.1|Ca_	superfamily	275769	167	260	4.20426e-13	66.253	cl02529	ANK superfamily	-
Q#1030 - >Ca_12043.1|Ca_	specific	201968	492	531	5.38151e-23	92.3699	pfam01779	Ribosomal_L29e	cl22619
Q#1030 - >Ca_12043.1|Ca_	superfamily	276573	492	531	5.38151e-23	92.3699	cl22619	Ribosomal_L29e superfamily	-
Q#1030 - >Ca_12043.1|Ca_	specific	251571	377	484	2.25068e-19	84.1513	pfam02852	Pyr_redox_dim	cl08372
Q#1030 - >Ca_12043.1|Ca_	superfamily	251571	377	484	2.25068e-19	84.1513	cl08372	Pyr_redox_dim superfamily	-
Q#1030 - >Ca_12043.1|Ca_	multi-dom	257303	173	263	1.36581e-06	46.4803	pfam12796	Ank_2	-
Q#1033 - >Ca_12064.1|Ca_	specific	197383	20	112	1.49701e-08	51.1745	cd10017	B3_DNA	cl15242
Q#1033 - >Ca_12064.1|Ca_	superfamily	265652	20	112	1.49701e-08	51.1745	cl15242	BfiI_C_EcoRII_N_B3 superfamily	-
Q#1033 - >Ca_12064.1|Ca_	superfamily	265652	178	242	0.000257762	38.4629	cl15242	BfiI_C_EcoRII_N_B3 superfamily	-
Q#1034 - >Ca_12092.1|Ca_	superfamily	256046	47	314	1.47559e-54	186.446	cl11168	PMD superfamily	-
Q#1038 - >Ca_12108.1|Ca_	specific	143480	32	147	2.97779e-46	149.707	cd06664	IscU_like	cl00528
Q#1038 - >Ca_12108.1|Ca_	superfamily	275619	32	147	2.97779e-46	149.707	cl00528	IscU_like superfamily	-
Q#1039 - >Ca_12112.1|Ca_	superfamily	263970	1	196	4.99302e-93	275.657	cl10447	GH18_chitinase-like superfamily	-
Q#1039 - >Ca_12112.1|Ca_	multi-dom	250070	1	187	1.12872e-11	60.9817	pfam00704	Glyco_hydro_18	-
Q#1041 - >Ca_12117.1|Ca_	specific	257306	77	115	3.77525e-05	39.7756	pfam12799	LRR_4	cl19302
Q#1041 - >Ca_12117.1|Ca_	superfamily	257306	77	115	3.77525e-05	39.7756	cl19302	LRR_4 superfamily	-
Q#1041 - >Ca_12117.1|Ca_	superfamily	276664	52	132	0.00428566	36.5646	cl22710	LRR_RI superfamily	-
Q#1041 - >Ca_12117.1|Ca_	multi-dom	258126	77	132	3.11879e-08	48.6881	pfam13855	LRR_8	-
Q#1045 - >Ca_12180.1|Ca_	specific	250027	23	70	1.47322e-08	49.8957	pfam00646	F-box	cl02535
Q#1045 - >Ca_12180.1|Ca_	superfamily	261329	23	70	1.47322e-08	49.8957	cl02535	F-box superfamily	-
Q#1045 - >Ca_12180.1|Ca_	superfamily	276263	142	263	0.00135126	38.1092	cl19514	FBA_1 superfamily	-
Q#1049 - >Ca_12194.1|Ca_	superfamily	276192	102	176	1.42967e-18	77.2291	cl17169	RRM_SF superfamily	-
Q#1049 - >Ca_12194.1|Ca_	superfamily	260390	11	52	3.00992e-05	41.7902	cl00376	Ribosomal_L10_P0 superfamily	-
Q#1050 - >Ca_12205.1|Ca_	multi-dom	274346	7	67	0.00601655	34.8641	TIGR02907	Stage_VI_sporulation_protein_D	-
Q#1051 - >Ca_12214.1|Ca_	specific	260006	72	183	4.7618e-53	171.136	cd09274	RNase_HI_RT_Ty3	cl14782
Q#1051 - >Ca_12214.1|Ca_	superfamily	271754	72	183	4.7618e-53	171.136	cl14782	RNase_H_like superfamily	-

Query	Types	PSSM-ID	From	To	E-Value	Bitscore	Accession	Short name	Superfamily
Q#1054 - >Ca_12240.1|Ca_	specific	238002	5	143	4.25827e-12	58.6396	cd00042	CY	cl09238
Q#1054 - >Ca_12240.1|Ca_	superfamily	275873	5	143	4.25827e-12	58.6396	cl09238	CY superfamily	-
Q#1055 - >Ca_12247.1|Ca_	superfamily	251850	169	213	5.32562e-16	74.316	cl03979	PAE superfamily	-
Q#1055 - >Ca_12247.1|Ca_	specific	249704	16	62	3.51512e-15	68.76	pfam00235	Profilin	cl00123
Q#1055 - >Ca_12247.1|Ca_	superfamily	260204	16	62	3.51512e-15	68.76	cl00123	PROF superfamily	-
Q#1055 - >Ca_12247.1|Ca_	superfamily	252798	133	182	1.44523e-13	63.6761	cl04748	DUF547 superfamily	-
Q#1059 - >Ca_12274.1|Ca_	superfamily	276301	116	199	4.12662e-15	71.4181	cl21457	TIM_phosphate_binding superfamily	-
Q#1060 - >Ca_12276.1|Ca_	superfamily	275922	4	91	4.39072e-19	78.3317	cl10555	Chorismate_bind superfamily	-
Q#1063 - >Ca_12306.1|Ca_	multi-dom	223021	58	213	2.54409e-05	44.9293	PHA03247	PHA03247	-
Q#1064 - >Ca_12315.1|Ca_	superfamily	276335	1	167	1.43592e-93	282.666	cl21512	Glycos_transf_1 superfamily	-
Q#1065 - >Ca_12319.1|Ca_	multi-dom	254113	177	394	4.32149e-91	283.941	pfam07227	DUF1423	-
Q#1067 - >Ca_12364.1|Ca_	superfamily	254499	72	219	1.66444e-27	107.824	cl20350	Frigida superfamily	-
Q#1068 - >Ca_12365.1|Ca_	superfamily	276241	61	134	5.51215e-14	66.1817	cl19217	Mem_trans superfamily	-
Q#1069 - >Ca_12371.1|Ca_	multi-dom	236669	419	572	2.0958e-05	46.6167	PRK10263	PRK10263	-
Q#1072 - >Ca_12404.1|Ca_	specific	189387	191	208	0.000231532	37.4337	pfam00098	zf-CCHC	cl22700
Q#1072 - >Ca_12404.1|Ca_	superfamily	276654	191	208	0.000231532	37.4337	cl22700	zf-CCHC superfamily	-
Q#1075 - >Ca_12439.1|Ca_	superfamily	276298	194	275	1.70544e-12	63.5084	cl21454	SDR superfamily	-
Q#1077 - >Ca_12458.1|Ca_	multi-dom	215561	1	196	9.00848e-31	119.569	PLN03077	PLN03077	-
Q#1084 - >Ca_12478.1|Ca_	multi-dom	223021	14	91	0.00610337	36.0697	PHA03247	PHA03247	-
Q#1086 - >Ca_12493.1|Ca_	specific	238825	5	181	2.77737e-94	285.643	cd01647	RT_LTR	cl02808
Q#1086 - >Ca_12493.1|Ca_	superfamily	261455	5	181	2.77737e-94	285.643	cl02808	RT_like superfamily	-
Q#1086 - >Ca_12493.1|Ca_	multi-dom	249567	22	181	4.25694e-35	129.758	pfam00078	RVT_1	-
Q#1088 - >Ca_12541.1|Ca_	specific	238005	27	156	1.99463e-19	80.074	cd00046	DEXDc	cl21455
Q#1088 - >Ca_12541.1|Ca_	superfamily	276299	27	156	1.99463e-19	80.074	cl21455	ABC_ATPase superfamily	-
Q#1089 - >Ca_12543.1|Ca_	superfamily	276299	82	170	2.23028e-29	108.341	cl21455	ABC_ATPase superfamily	-
Q#1089 - >Ca_12543.1|Ca_	superfamily	271701	1	43	1.53744e-09	52.342	cl10459	Peptidases_S8_S53 superfamily	-
Q#1093 - >Ca_12583.1|Ca_	superfamily	276196	4	144	6.00691e-12	59.7353	cl17185	LPLAT superfamily	-
Q#1095 - >Ca_12587.1|Ca_	multi-dom	227596	88	162	0.00861367	36.1304	COG5271	MDN1	-
Q#1097 - >Ca_12603.1|Ca_	superfamily	271701	1	39	1.10667e-11	59.534	cl10459	Peptidases_S8_S53 superfamily	-
Q#1098 - >Ca_12606.1|Ca_	specific	133136	609	653	6.45612e-06	44.6348	cd00303	retropepsin_like	cl11403
Q#1098 - >Ca_12606.1|Ca_	superfamily	275927	609	653	6.45612e-06	44.6348	cl11403	pepsin_retropepsin_like superfamily	-
Q#1098 - >Ca_12606.1|Ca_	specific	252133	347	446	1.2981e-12	64.6673	pfam03732	Retrotrans_gag	cl04237
Q#1098 - >Ca_12606.1|Ca_	superfamily	252133	347	446	1.2981e-12	64.6673	cl04237	Retrotrans_gag superfamily	-
Q#1098 - >Ca_12606.1|Ca_	superfamily	252133	100	199	1.6211e-11	61.5857	cl04237	Retrotrans_gag superfamily	-
Q#1098 - >Ca_12606.1|Ca_	specific	189387	306	322	0.000494263	38.5893	pfam00098	zf-CCHC	cl22700
Q#1098 - >Ca_12606.1|Ca_	superfamily	276654	306	322	0.000494263	38.5893	cl22700	zf-CCHC superfamily	-

Query	Types	PSSM-ID	From	To	E-Value	Bitscore	Accession	Short name	Superfamily
Q#1098 - >Ca_12606.1|Ca_	specific	189387	552	569	0.00734757	35.1225	pfam00098	zf-CCHC	cl22700
Q#1098 - >Ca_12606.1|Ca_	superfamily	276654	552	569	0.00734757	35.1225	cl22700	zf-CCHC superfamily	-
Q#1099 - >Ca_12607.1|Ca_	specific	240712	17	120	8.95055e-28	103.198	cd12266	RRM_like_XS	cl17169
Q#1099 - >Ca_12607.1|Ca_	superfamily	276192	17	120	8.95055e-28	103.198	cl17169	RRM_SF superfamily	-
Q#1099 - >Ca_12607.1|Ca_	superfamily	255922	129	209	6.51496e-28	107.751	cl11011	DUF2419 superfamily	-
Q#1100 - >Ca_12609.1|Ca_	specific	260004	143	279	7.00705e-64	200.002	cd09272	RNase_HI_RT_Ty1	cl14782
Q#1100 - >Ca_12609.1|Ca_	superfamily	271754	143	279	7.00705e-64	200.002	cl14782	RNase_H_like superfamily	-
Q#1100 - >Ca_12609.1|Ca_	superfamily	254387	1	57	2.49633e-16	75.1855	cl06662	RVT_2 superfamily	-
Q#1102 - >Ca_12611.1|Ca_	specific	252133	100	199	1.80834e-12	63.1265	pfam03732	Retrotrans_gag	cl04237
Q#1102 - >Ca_12611.1|Ca_	superfamily	252133	100	199	1.80834e-12	63.1265	cl04237	Retrotrans_gag superfamily	-
Q#1102 - >Ca_12611.1|Ca_	superfamily	275927	362	406	0.000114105	40.0124	cl11403	pepsin_retropepsin_like superfamily	-
Q#1102 - >Ca_12611.1|Ca_	specific	197667	286	302	0.0058625	34.7264	smart00343	ZnF_C2HC	cl22700
Q#1102 - >Ca_12611.1|Ca_	superfamily	276654	286	302	0.0058625	34.7264	cl22700	zf-CCHC superfamily	-
Q#1103 - >Ca_12616.1|Ca_	specific	238825	569	699	3.07576e-62	207.448	cd01647	RT_LTR	cl02808
Q#1103 - >Ca_12616.1|Ca_	superfamily	261455	569	699	3.07576e-62	207.448	cl02808	RT_like superfamily	-
Q#1103 - >Ca_12616.1|Ca_	specific	133136	362	449	1.05659e-17	79.6879	cd00303	retropepsin_like	cl11403
Q#1103 - >Ca_12616.1|Ca_	superfamily	275927	362	449	1.05659e-17	79.6879	cl11403	pepsin_retropepsin_like superfamily	-
Q#1103 - >Ca_12616.1|Ca_	specific	252133	100	199	1.2121e-12	65.0525	pfam03732	Retrotrans_gag	cl04237
Q#1103 - >Ca_12616.1|Ca_	superfamily	252133	100	199	1.2121e-12	65.0525	cl04237	Retrotrans_gag superfamily	-
Q#1103 - >Ca_12616.1|Ca_	specific	189387	305	322	0.000639691	38.5893	pfam00098	zf-CCHC	cl22700
Q#1103 - >Ca_12616.1|Ca_	superfamily	276654	305	322	0.000639691	38.5893	cl22700	zf-CCHC superfamily	-
Q#1103 - >Ca_12616.1|Ca_	multi-dom	249567	586	690	2.96105e-18	83.9194	pfam00078	RVT_1	-
Q#1103 - >Ca_12616.1|Ca_	multi-dom	173561	287	366	3.9549e-09	55.5832	PTZ00368	PTZ00368	-
Q#1104 - >Ca_12625.1|Ca_	superfamily	276194	5	182	1.32161e-24	97.3057	cl17173	AdoMet_MTases superfamily	-
Q#1107 - >Ca_12646.1|Ca_	specific	119392	78	149	4.85028e-05	40.7601	cd06174	MFS	cl21472
Q#1107 - >Ca_12646.1|Ca_	superfamily	276313	78	149	4.85028e-05	40.7601	cl21472	MFS superfamily	-
Q#1108 - >Ca_12650.1|Ca_	specific	212030	105	150	3.18627e-07	45.5541	cd00118	LysM	cl00107
Q#1108 - >Ca_12650.1|Ca_	superfamily	275472	105	150	3.18627e-07	45.5541	cl00107	LysM superfamily	-
Q#1108 - >Ca_12650.1|Ca_	specific	212030	170	197	5.24548e-05	39.3909	cd00118	LysM	cl00107
Q#1108 - >Ca_12650.1|Ca_	superfamily	275472	170	197	5.24548e-05	39.3909	cl00107	LysM superfamily	-
Q#1109 - >Ca_12659.1|Ca_	specific	189387	52	65	0.00573415	30.5001	pfam00098	zf-CCHC	cl22700
Q#1109 - >Ca_12659.1|Ca_	superfamily	276654	52	65	0.00573415	30.5001	cl22700	zf-CCHC superfamily	-
Q#1110 - >Ca_12698.1|Ca_	superfamily	276263	125	281	1.07887e-14	70.466	cl19514	FBA_1 superfamily	-
Q#1110 - >Ca_12698.1|Ca_	specific	197608	16	56	8.95177e-09	50.513	smart00256	FBOX	cl02535
Q#1110 - >Ca_12698.1|Ca_	superfamily	261329	16	56	8.95177e-09	50.513	cl02535	F-box superfamily	-
Q#1112 - >Ca_12712.1|Ca_	specific	238093	116	155	2.71555e-10	52.448	cd00162	RING	cl17238

Query	Types	PSSM-ID	From	To	E-Value	Bitscore	Accession	Short name	Superfamily
Q#1112 - >Ca_12712.1|Ca_	superfamily	276201	116	155	2.71555e-10	52.448	cl17238	RING superfamily	-
Q#1114 - >Ca_12718.1|Ca_	specific	258415	63	179	4.40194e-38	129.23	pfam14223	UBN2	cl15874
Q#1114 - >Ca_12718.1|Ca_	superfamily	265821	63	179	4.40194e-38	129.23	cl15874	UBN2 superfamily	-
Q#1115 - >Ca_12728.1|Ca_	superfamily	256046	1	69	6.147e-17	78.2052	cl11168	PMD superfamily	-
Q#1123 - >Ca_12781.1|Ca_	multi-dom	259534	189	351	0.00193241	38.9718	pfam15402	Spc7_N	-
Q#1126 - >Ca_12799.1|Ca_	multi-dom	173561	69	139	0.000870104	36.3233	PTZ00368	PTZ00368	-
Q#1127 - >Ca_12801.1|Ca_	specific	133136	362	406	3.37941e-06	44.6348	cd00303	retropepsin_like	cl11403
Q#1127 - >Ca_12801.1|Ca_	superfamily	275927	362	406	3.37941e-06	44.6348	cl11403	pepsin_retropepsin_like superfamily	-
Q#1127 - >Ca_12801.1|Ca_	specific	252133	100	199	3.61717e-13	65.4377	pfam03732	Retrotrans_gag	cl04237
Q#1127 - >Ca_12801.1|Ca_	superfamily	252133	100	199	3.61717e-13	65.4377	cl04237	Retrotrans_gag superfamily	-
Q#1127 - >Ca_12801.1|Ca_	specific	189387	305	322	0.00272599	35.5077	pfam00098	zf-CCHC	cl22700
Q#1127 - >Ca_12801.1|Ca_	superfamily	276654	305	322	0.00272599	35.5077	cl22700	zf-CCHC superfamily	-
Q#1128 - >Ca_12802.1|Ca_	specific	202502	196	351	2.89721e-87	277.22	pfam02992	Transposase_21	cl03823
Q#1128 - >Ca_12802.1|Ca_	superfamily	202502	196	351	2.89721e-87	277.22	cl03823	Transposase_21 superfamily	-
Q#1128 - >Ca_12802.1|Ca_	specific	258207	624	692	1.35346e-19	84.5551	pfam13952	DUF4216	cl16495
Q#1128 - >Ca_12802.1|Ca_	superfamily	258207	624	692	1.35346e-19	84.5551	cl16495	DUF4216 superfamily	-
Q#1128 - >Ca_12802.1|Ca_	superfamily	206130	432	472	1.44579e-08	53.3615	cl16501	DUF4218 superfamily	-
Q#1133 - >Ca_12819.1|Ca_	specific	251090	6	116	2.77479e-48	152.063	pfam02109	DAD	cl03452
Q#1133 - >Ca_12819.1|Ca_	superfamily	251090	6	116	2.77479e-48	152.063	cl03452	DAD superfamily	-
Q#1138 - >Ca_12839.1|Ca_	superfamily	252133	100	199	7.51495e-12	60.4301	cl04237	Retrotrans_gag superfamily	-
Q#1138 - >Ca_12839.1|Ca_	specific	189387	253	269	5.31894e-05	39.7449	pfam00098	zf-CCHC	cl22700
Q#1138 - >Ca_12839.1|Ca_	superfamily	276654	253	269	5.31894e-05	39.7449	cl22700	zf-CCHC superfamily	-
Q#1140 - >Ca_12850.1|Ca_	specific	253333	27	89	1.98064e-12	59.6089	pfam05699	Dimer_Tnp_hAT	cl05324
Q#1140 - >Ca_12850.1|Ca_	superfamily	253333	27	89	1.98064e-12	59.6089	cl05324	Dimer_Tnp_hAT superfamily	-
Q#1141 - >Ca_12851.1|Ca_	superfamily	252133	1	94	5.55704e-09	49.6445	cl04237	Retrotrans_gag superfamily	-
Q#1144 - >Ca_12895.1|Ca_	multi-dom	237865	5	89	0.00518056	33.1493	PRK14951	PRK14951	-
Q#1145 - >Ca_12896.1|Ca_	specific	251988	257	295	2.26694e-11	58.3502	pfam03478	DUF295	cl04101
Q#1145 - >Ca_12896.1|Ca_	superfamily	251988	257	295	2.26694e-11	58.3502	cl04101	DUF295 superfamily	-
Q#1145 - >Ca_12896.1|Ca_	multi-dom	178754	21	295	9.08056e-12	64.2012	PLN03215	PLN03215	-
Q#1148 - >Ca_12913.1|Ca_	specific	133136	230	316	6.01344e-13	64.6652	cd00303	retropepsin_like	cl11403
Q#1148 - >Ca_12913.1|Ca_	superfamily	275927	230	316	6.01344e-13	64.6652	cl11403	pepsin_retropepsin_like superfamily	-
Q#1150 - >Ca_12926.1|Ca_	multi-dom	236912	32	127	0.000216887	38.3941	PRK11448	hsdR	-
Q#1153 - >Ca_12939.1|Ca_	multi-dom	259534	266	309	0.00300128	38.5866	pfam15402	Spc7_N	-
Q#1156 - >Ca_12942.1|Ca_	superfamily	191549	3	118	1.39773e-51	163.53	cl05834	PAR1 superfamily	-
Q#1157 - >Ca_12950.1|Ca_	specific	238002	5	143	4.25827e-12	58.6396	cd00042	CY	cl09238
Q#1157 - >Ca_12950.1|Ca_	superfamily	275873	5	143	4.25827e-12	58.6396	cl09238	CY superfamily	-

Query	Types	PSSM-ID	From	To	E-Value	Bitscore	Accession	Short name	Superfamily
Q#1158 - >Ca_12957.1|Ca_	multi-dom	249572	101	166	1.13399e-09	54.9994	pfam00083	Sugar_tr	-
Q#1159 - >Ca_12964.1|Ca_	specific	133136	191	248	1.53923e-07	49.6424	cd00303	retropepsin_like	cl11403
Q#1159 - >Ca_12964.1|Ca_	superfamily	275927	191	248	1.53923e-07	49.6424	cl11403	pepsin_retropepsin_like superfamily	-
Q#1159 - >Ca_12964.1|Ca_	specific	250040	476	581	1.29748e-10	59.2693	pfam00665	rve	cl21549
Q#1159 - >Ca_12964.1|Ca_	superfamily	276355	476	581	1.29748e-10	59.2693	cl21549	rve superfamily	-
Q#1160 - >Ca_12967.1|Ca_	superfamily	276269	162	195	0.00265073	34.7441	cl19537	Transposase_mut superfamily	-
Q#1161 - >Ca_12971.1|Ca_	specific	133136	327	383	8.05327e-06	43.8644	cd00303	retropepsin_like	cl11403
Q#1161 - >Ca_12971.1|Ca_	superfamily	275927	327	383	8.05327e-06	43.8644	cl11403	pepsin_retropepsin_like superfamily	-
Q#1163 - >Ca_12982.1|Ca_	specific	252133	100	199	4.32356e-13	62.7413	pfam03732	Retrotrans_gag	cl04237
Q#1163 - >Ca_12982.1|Ca_	superfamily	252133	100	199	4.32356e-13	62.7413	cl04237	Retrotrans_gag superfamily	-
Q#1164 - >Ca_12985.1|Ca_	specific	258114	80	129	1.76504e-13	63.9987	pfam13839	PC-Esterase	cl01053
Q#1164 - >Ca_12985.1|Ca_	superfamily	260768	80	129	1.76504e-13	63.9987	cl01053	SGNH_hydrolase superfamily	-
Q#1166 - >Ca_12993.1|Ca_	superfamily	271754	96	163	1.71872e-14	69.4437	cl14782	RNase_H_like superfamily	-
Q#1169 - >Ca_13026.1|Ca_	superfamily	261455	70	104	7.22118e-07	43.7381	cl02808	RT_like superfamily	-
Q#1169 - >Ca_13026.1|Ca_	superfamily	275927	1	50	0.0098759	31.1528	cl11403	pepsin_retropepsin_like superfamily	-
Q#1173 - >Ca_13046.1|Ca_	specific	260004	9	145	2.282e-71	213.869	cd09272	RNase_HI_RT_Ty1	cl14782
Q#1173 - >Ca_13046.1|Ca_	superfamily	271754	9	145	2.282e-71	213.869	cl14782	RNase_H_like superfamily	-
Q#1174 - >Ca_13049.1|Ca_	specific	260004	176	281	2.32575e-49	162.638	cd09272	RNase_HI_RT_Ty1	cl14782
Q#1174 - >Ca_13049.1|Ca_	superfamily	271754	176	281	2.32575e-49	162.638	cl14782	RNase_H_like superfamily	-
Q#1175 - >Ca_13068.1|Ca_	superfamily	275593	4	137	9.57652e-86	257.635	cl00467	Ntn_hydrolase superfamily	-
Q#1183 - >Ca_13239.1|Ca_	multi-dom	273258	2	102	0.00432262	33.8115	TIGR00763	Lon_protease	-
Q#1184 - >Ca_13241.1|Ca_	superfamily	271701	1	40	2.26709e-09	53.3708	cl10459	Peptidases_S8_S53 superfamily	-
Q#1184 - >Ca_13241.1|Ca_	superfamily	276299	97	137	8.1937e-07	44.9272	cl21455	ABC_ATPase superfamily	-
Q#1185 - >Ca_13254.1|Ca_	specific	238083	11	184	2.27225e-63	198.319	cd00143	PP2Cc	cl00120
Q#1185 - >Ca_13254.1|Ca_	superfamily	260203	11	184	2.27225e-63	198.319	cl00120	PP2Cc superfamily	-
Q#1187 - >Ca_13268.1|Ca_	specific	119339	1	147	4.17994e-20	84.9196	cd06558	crotonase-like	cl21466
Q#1187 - >Ca_13268.1|Ca_	superfamily	276308	1	147	4.17994e-20	84.9196	cl21466	crotonase-like superfamily	-
Q#1187 - >Ca_13268.1|Ca_	superfamily	262145	150	230	1.59742e-06	46.2306	cl04410	DFP superfamily	-
Q#1190 - >Ca_13385.1|Ca_	specific	238096	22	65	3.35983e-14	64.519	cd00167	SANT	cl21498
Q#1190 - >Ca_13385.1|Ca_	superfamily	271918	22	65	3.35983e-14	64.519	cl21498	SANT superfamily	-
Q#1190 - >Ca_13385.1|Ca_	specific	238096	74	116	5.82993e-11	55.6594	cd00167	SANT	cl21498
Q#1190 - >Ca_13385.1|Ca_	superfamily	271918	74	116	5.82993e-11	55.6594	cl21498	SANT superfamily	-
Q#1191 - >Ca_13391.1|Ca_	specific	256054	352	420	5.35998e-09	53.6189	pfam10551	MULE	cl19537
Q#1191 - >Ca_13391.1|Ca_	superfamily	276269	352	420	5.35998e-09	53.6189	cl19537	Transposase_mut superfamily	-
Q#1191 - >Ca_13391.1|Ca_	superfamily	190526	261	304	1.40104e-05	42.975	cl07846	DBD_Tnp_Mut superfamily	-
Q#1193 - >Ca_13432.1|Ca_	specific	238112	177	236	3.27471e-05	40.8264	cd00189	TPR	cl22441

Query	Types	PSSM-ID	From	To	E-Value	Bitscore	Accession	Short name	Superfamily
Q#1193 - >Ca_13432.1|Ca_	superfamily	276395	177	236	3.27471e-05	40.8264	cl22441	TPR superfamily	-
Q#1193 - >Ca_13432.1|Ca_	multi-dom	257757	177	238	5.25163e-06	42.6776	pfam13432	TPR_16	-
Q#1197 - >Ca_13503.1|Ca_	specific	251485	56	116	2.96141e-18	73.4743	pfam02704	GASA	cl03667
Q#1197 - >Ca_13503.1|Ca_	superfamily	251485	56	116	2.96141e-18	73.4743	cl03667	GASA superfamily	-
Q#1198 - >Ca_13507.1|Ca_	superfamily	276299	7	189	1.1936e-40	140.004	cl21455	ABC_ATPase superfamily	-
Q#1198 - >Ca_13507.1|Ca_	multi-dom	251682	7	193	1.95792e-79	239.19	pfam03029	ATP_bind_1	-
Q#1199 - >Ca_13535.1|Ca_	superfamily	241616	2	52	4.11144e-17	72.585	cl00109	MADS superfamily	-
Q#1200 - >Ca_13568.1|Ca_	superfamily	260277	13	157	1.83851e-39	139.253	cl00224	PLPDE_IV superfamily	-
Q#1200 - >Ca_13568.1|Ca_	superfamily	276392	205	243	0.00208904	35.6278	cl22438	TMF_TATA_bd superfamily	-
Q#1202 - >Ca_13645.1|Ca_	superfamily	276235	4	353	2.77818e-110	330.805	cl19188	Pectate_lyase_3 superfamily	-
Q#1202 - >Ca_13645.1|Ca_	multi-dom	249749	14	338	4.23677e-88	270.779	pfam00295	Glyco_hydro_28	-
Q#1204 - >Ca_13665.1|Ca_	specific	133437	143	182	2.83046e-09	51.3486	cd01667	TGS_ThrRS_N	cl00155
Q#1204 - >Ca_13665.1|Ca_	superfamily	275476	143	182	2.83046e-09	51.3486	cl00155	UBQ superfamily	-
Q#1206 - >Ca_13697.1|Ca_	superfamily	275516	37	123	1.55144e-16	72.2302	cl00296	Peptidase_C1 superfamily	-
Q#1207 - >Ca_13771.1|Ca_	specific	175973	11	119	4.25198e-06	42.4391	cd00030	C2	cl14603
Q#1207 - >Ca_13771.1|Ca_	superfamily	271750	11	119	4.25198e-06	42.4391	cl14603	C2 superfamily	-
Q#1208 - >Ca_13773.1|Ca_	specific	175973	11	119	3.44057e-06	42.8243	cd00030	C2	cl14603
Q#1208 - >Ca_13773.1|Ca_	superfamily	271750	11	119	3.44057e-06	42.8243	cl14603	C2 superfamily	-
Q#1209 - >Ca_13774.1|Ca_	specific	175973	15	123	2.58074e-07	45.9059	cd00030	C2	cl14603
Q#1209 - >Ca_13774.1|Ca_	superfamily	271750	15	123	2.58074e-07	45.9059	cl14603	C2 superfamily	-
Q#1210 - >Ca_13780.1|Ca_	superfamily	262303	16	105	4.86619e-36	133.414	cl04813	EIN3 superfamily	-
Q#1211 - >Ca_13797.1|Ca_	specific	250513	6	184	6.23031e-94	274.78	pfam01294	Ribosomal_L13e	cl01799
Q#1211 - >Ca_13797.1|Ca_	superfamily	261075	6	184	6.23031e-94	274.78	cl01799	Ribosomal_L13e superfamily	-
Q#1213 - >Ca_13858.1|Ca_	superfamily	275586	2	56	1.43928e-11	59.4703	cl00456	SLC5-6-like_sbd superfamily	-
Q#1214 - >Ca_13859.1|Ca_	superfamily	275586	2	56	1.43928e-11	59.4703	cl00456	SLC5-6-like_sbd superfamily	-
Q#1215 - >Ca_13860.1|Ca_	superfamily	275586	2	56	1.22517e-11	59.4703	cl00456	SLC5-6-like_sbd superfamily	-
Q#1216 - >Ca_13870.1|Ca_	superfamily	276241	33	145	1.62263e-12	62.3297	cl19217	Mem_trans superfamily	-
Q#1217 - >Ca_13876.1|Ca_	superfamily	276098	61	208	1.50854e-94	287.244	cl14876	Zinc_peptidase_like superfamily	-
Q#1220 - >Ca_13901.1|Ca_	superfamily	252133	193	267	1.02211e-08	51.9557	cl04237	Retrotrans_gag superfamily	-
Q#1221 - >Ca_13902.1|Ca_	specific	238096	16	62	5.13671e-07	42.1774	cd00167	SANT	cl21498
Q#1221 - >Ca_13902.1|Ca_	superfamily	271918	16	62	5.13671e-07	42.1774	cl21498	SANT superfamily	-
Q#1222 - >Ca_13948.1|Ca_	superfamily	275582	18	98	0.0019868	35.5447	cl00451	MoCF_BD superfamily	-
Q#1223 - >Ca_13954.1|Ca_	superfamily	253129	161	260	2.46068e-08	52.1479	cl12213	PEARLI-4 superfamily	-
Q#1226 - >Ca_13964.1|Ca_	superfamily	276007	103	147	0.00129902	36.6166	cl11961	ALDH-SF superfamily	-
Q#1227 - >Ca_14007.1|Ca_	specific	251731	62	136	3.3725e-21	87.3607	pfam03101	FAR1	cl03890
Q#1227 - >Ca_14007.1|Ca_	superfamily	251731	62	136	3.3725e-21	87.3607	cl03890	FAR1 superfamily	-

Query	Types	PSSM-ID	From	To	E-Value	Bitscore	Accession	Short name	Superfamily
Q#1227 - >Ca_14007.1|Ca_	superfamily	262858	302	402	3.05105e-06	44.8627	cl06336	Commd superfamily	-
Q#1228 - >Ca_14025.1|Ca_	specific	250966	19	83	7.42128e-19	77.6425	pfam01918	Alba	cl00682
Q#1228 - >Ca_14025.1|Ca_	superfamily	260569	19	83	7.42128e-19	77.6425	cl00682	Alba superfamily	-
Q#1230 - >Ca_14046.1|Ca_	specific	238099	32	177	2.74858e-41	138.287	cd00170	SEC14	cl15787
Q#1230 - >Ca_14046.1|Ca_	superfamily	265785	32	177	2.74858e-41	138.287	cl15787	SEC14 superfamily	-
Q#1231 - >Ca_14080.1|Ca_	superfamily	276263	114	326	1.55879e-24	99.7411	cl19514	FBA_1 superfamily	-
Q#1231 - >Ca_14080.1|Ca_	specific	250027	19	62	1.91946e-07	47.5845	pfam00646	F-box	cl02535
Q#1231 - >Ca_14080.1|Ca_	superfamily	261329	19	62	1.91946e-07	47.5845	cl02535	F-box superfamily	-
Q#1232 - >Ca_14097.1|Ca_	specific	215093	1	109	4.26149e-62	188.043	PLN00186	PLN00186	cl01993
Q#1232 - >Ca_14097.1|Ca_	superfamily	261144	1	109	4.26149e-62	188.043	cl01993	Ribosomal_S26e superfamily	-
Q#1233 - >Ca_14219.1|Ca_	superfamily	252763	1	172	3.65486e-103	304.327	cl04706	Glyco_transf_17 superfamily	-
Q#1235 - >Ca_14260.1|Ca_	superfamily	275868	57	84	2.48048e-09	49.659	cl09141	ACT superfamily	-
Q#1236 - >Ca_14344.1|Ca_	superfamily	275846	1	193	3.27819e-67	219.802	cl07893	AmyAc_family superfamily	-
Q#1236 - >Ca_14344.1|Ca_	multi-dom	166698	1	446	1.06626e-135	414.016	PLN03059	PLN03059	-
Q#1237 - >Ca_14351.1|Ca_	superfamily	261217	283	354	0.0018555	38.2493	cl02219	Bap31 superfamily	-
Q#1238 - >Ca_14373.1|Ca_	superfamily	275846	1	180	1.54886e-69	225.58	cl07893	AmyAc_family superfamily	-
Q#1238 - >Ca_14373.1|Ca_	multi-dom	166698	1	433	4.8801e-140	424.802	PLN03059	PLN03059	-
Q#1239 - >Ca_14382.1|Ca_	superfamily	271424	52	158	1.91219e-22	87.715	cl00140	SNc superfamily	-
Q#1240 - >Ca_14395.1|Ca_	specific	237991	33	82	1.32653e-12	61.1259	cd00024	CHROMO	cl17459
Q#1240 - >Ca_14395.1|Ca_	superfamily	271815	33	82	1.32653e-12	61.1259	cl17459	CHROMO superfamily	-
Q#1241 - >Ca_14408.1|Ca_	specific	238246	41	131	1.07071e-19	81.4323	cd00432	Ribosomal_L18_L5e	cl00379
Q#1241 - >Ca_14408.1|Ca_	superfamily	275559	41	131	1.07071e-19	81.4323	cl00379	Ribosomal_L18_L5e superfamily	-
Q#1241 - >Ca_14408.1|Ca_	specific	258399	153	228	5.0479e-30	108.791	pfam14204	Ribosomal_L18_c	cl16705
Q#1241 - >Ca_14408.1|Ca_	superfamily	258399	153	228	5.0479e-30	108.791	cl16705	Ribosomal_L18_c superfamily	-
Q#1241 - >Ca_14408.1|Ca_	multi-dom	240254	1	231	1.78845e-109	320.477	PTZ00069	PTZ00069	-
Q#1242 - >Ca_14422.1|Ca_	specific	212558	282	330	3.09507e-17	74.1407	cd11660	SANT_TRF	cl21498
Q#1242 - >Ca_14422.1|Ca_	superfamily	271918	282	330	3.09507e-17	74.1407	cl21498	SANT superfamily	-
Q#1245 - >Ca_14433.1|Ca_	specific	251348	10	89	1.29417e-32	110.857	pfam02519	Auxin_inducible	cl03633
Q#1245 - >Ca_14433.1|Ca_	superfamily	261812	10	89	1.29417e-32	110.857	cl03633	Auxin_inducible superfamily	-
Q#1246 - >Ca_14437.1|Ca_	specific	251348	2	90	1.26506e-34	115.865	pfam02519	Auxin_inducible	cl03633
Q#1246 - >Ca_14437.1|Ca_	superfamily	261812	2	90	1.26506e-34	115.865	cl03633	Auxin_inducible superfamily	-
Q#1247 - >Ca_14438.1|Ca_	specific	251348	21	84	1.14929e-32	110.857	pfam02519	Auxin_inducible	cl03633
Q#1247 - >Ca_14438.1|Ca_	superfamily	261812	21	84	1.14929e-32	110.857	cl03633	Auxin_inducible superfamily	-
Q#1248 - >Ca_14440.1|Ca_	specific	251348	21	77	5.66725e-23	85.434	pfam02519	Auxin_inducible	cl03633
Q#1248 - >Ca_14440.1|Ca_	superfamily	261812	21	77	5.66725e-23	85.434	cl03633	Auxin_inducible superfamily	-
Q#1249 - >Ca_14477.1|Ca_	superfamily	276192	7	75	8.52366e-26	91.6807	cl17169	RRM_SF superfamily	-

Query	Types	PSSM-ID	From	To	E-Value	Bitscore	Accession	Short name	Superfamily
Q#1249 - >Ca_14477.1|Ca_	multi-dom	214636	7	75	3.45277e-24	87.6491	smart00360	RRM	-
Q#1250 - >Ca_14494.1|Ca_	specific	211390	46	144	1.3509e-16	72.233	cd11378	DUF296	cl00720
Q#1250 - >Ca_14494.1|Ca_	superfamily	260588	46	144	1.3509e-16	72.233	cl00720	DUF296 superfamily	-
Q#1251 - >Ca_14505.1|Ca_	superfamily	276299	82	140	1.00651e-22	89.4661	cl21455	ABC_ATPase superfamily	-
Q#1251 - >Ca_14505.1|Ca_	superfamily	271701	3	46	1.01097e-13	63.898	cl10459	Peptidases_S8_S53 superfamily	-
Q#1252 - >Ca_14519.1|Ca_	specific	237987	349	456	5.29194e-11	60.7911	cd00020	ARM	cl22454
Q#1252 - >Ca_14519.1|Ca_	superfamily	276408	349	456	5.29194e-11	60.7911	cl22454	ARM superfamily	-
Q#1252 - >Ca_14519.1|Ca_	specific	237987	426	550	1.30197e-09	56.5539	cd00020	ARM	cl22454
Q#1252 - >Ca_14519.1|Ca_	superfamily	276408	426	550	1.30197e-09	56.5539	cl22454	ARM superfamily	-
Q#1252 - >Ca_14519.1|Ca_	superfamily	191545	38	80	1.28509e-09	56.1029	cl05824	Auxin_resp superfamily	-
Q#1252 - >Ca_14519.1|Ca_	superfamily	201951	127	215	0.00302	36.5898	cl03339	IBB superfamily	-
Q#1254 - >Ca_14556.1|Ca_	superfamily	271848	20	149	1.32784e-28	108.224	cl19241	Condensation superfamily	-
Q#1255 - >Ca_14558.1|Ca_	superfamily	271848	20	149	1.35435e-28	108.224	cl19241	Condensation superfamily	-
Q#1256 - >Ca_14594.1|Ca_	specific	237987	349	456	4.7759e-11	60.7911	cd00020	ARM	cl22454
Q#1256 - >Ca_14594.1|Ca_	superfamily	276408	349	456	4.7759e-11	60.7911	cl22454	ARM superfamily	-
Q#1256 - >Ca_14594.1|Ca_	specific	237987	426	550	1.2193e-09	56.5539	cd00020	ARM	cl22454
Q#1256 - >Ca_14594.1|Ca_	superfamily	276408	426	550	1.2193e-09	56.5539	cl22454	ARM superfamily	-
Q#1256 - >Ca_14594.1|Ca_	superfamily	191545	38	80	1.27049e-09	56.1029	cl05824	Auxin_resp superfamily	-
Q#1256 - >Ca_14594.1|Ca_	superfamily	201951	127	215	0.00308414	36.5898	cl03339	IBB superfamily	-
Q#1257 - >Ca_14661.1|Ca_	specific	237991	33	82	3.56395e-12	60.3555	cd00024	CHROMO	cl17459
Q#1257 - >Ca_14661.1|Ca_	superfamily	271815	33	82	3.56395e-12	60.3555	cl17459	CHROMO superfamily	-
Q#1257 - >Ca_14661.1|Ca_	superfamily	260153	261	318	8.24925e-15	67.7372	cl00044	ChSh superfamily	-
Q#1260 - >Ca_14690.1|Ca_	superfamily	275527	104	168	4.2407e-08	50.3297	cl00316	Cation_efflux superfamily	-
Q#1262 - >Ca_14760.1|Ca_	specific	238924	57	141	6.76163e-24	89.6952	cd01958	HPS_like	cl07890
Q#1262 - >Ca_14760.1|Ca_	superfamily	275845	57	141	6.76163e-24	89.6952	cl07890	AAI_LTSS superfamily	-
Q#1263 - >Ca_14770.1|Ca_	specific	238008	42	99	4.08601e-11	54.8613	cd00051	EFh	cl08302
Q#1263 - >Ca_14770.1|Ca_	superfamily	275859	42	99	4.08601e-11	54.8613	cl08302	EFh superfamily	-
Q#1263 - >Ca_14770.1|Ca_	superfamily	275859	74	140	5.11558e-06	40.6089	cl08302	EFh superfamily	-
Q#1263 - >Ca_14770.1|Ca_	multi-dom	257819	46	98	1.08362e-05	39.6745	pfam13499	EF-hand_7	-
Q#1263 - >Ca_14770.1|Ca_	multi-dom	257819	79	139	0.000688582	35.0521	pfam13499	EF-hand_7	-
Q#1266 - >Ca_14789.1|Ca_	multi-dom	236712	100	147	0.00978512	34.3203	PRK10547	PRK10547	-
Q#1267 - >Ca_14816.1|Ca_	specific	238036	211	241	0.000168457	38.3491	cd00083	HLH	cl00081
Q#1267 - >Ca_14816.1|Ca_	superfamily	260178	211	241	0.000168457	38.3491	cl00081	HLH superfamily	-
Q#1268 - >Ca_14830.1|Ca_	superfamily	275516	14	69	1.53778e-12	61.0594	cl00296	Peptidase_C1 superfamily	-
Q#1269 - >Ca_14845.1|Ca_	specific	188824	6	68	4.39462e-38	127.581	cd09440	LIM1_SF3	cl02475
Q#1269 - >Ca_14845.1|Ca_	superfamily	261300	6	68	4.39462e-38	127.581	cl02475	LIM superfamily	-

Query	Types	PSSM-ID	From	To	E-Value	Bitscore	Accession	Short name	Superfamily
Q#1269 - >Ca_14845.1|Ca_	specific	188825	110	170	3.16713e-35	119.883	cd09441	LIM2_SF3	cl02475
Q#1269 - >Ca_14845.1|Ca_	superfamily	261300	110	170	3.16713e-35	119.883	cl02475	LIM superfamily	-
Q#1270 - >Ca_14886.1|Ca_	superfamily	271848	2	80	9.41854e-07	44.2992	cl19241	Condensation superfamily	-
Q#1271 - >Ca_14942.1|Ca_	specific	252797	109	147	8.43994e-09	51.6542	pfam04782	DUF632	cl04746
Q#1271 - >Ca_14942.1|Ca_	superfamily	252797	109	147	8.43994e-09	51.6542	cl04746	DUF632 superfamily	-
Q#1272 - >Ca_14944.1|Ca_	specific	250027	27	64	9.9391e-06	42.577	pfam00646	F-box	cl02535
Q#1272 - >Ca_14944.1|Ca_	superfamily	261329	27	64	9.9391e-06	42.577	cl02535	F-box superfamily	-
Q#1273 - >Ca_14951.1|Ca_	superfamily	276299	39	162	4.78239e-51	166.526	cl21455	ABC_ATPase superfamily	-
Q#1273 - >Ca_14951.1|Ca_	multi-dom	224054	39	201	6.78144e-38	134.357	COG1131	CcmA	-
Q#1274 - >Ca_15024.1|Ca_	multi-dom	177993	63	114	5.51991e-06	42.4708	PLN02365	PLN02365	-
Q#1274 - >Ca_15024.1|Ca_	multi-dom	178576	4	66	0.000463187	36.8932	PLN02997	PLN02997	-
Q#1275 - >Ca_15034.1|Ca_	superfamily	276192	3	64	2.79845e-25	92.6834	cl17169	RRM_SF superfamily	-
Q#1275 - >Ca_15034.1|Ca_	specific	197667	65	79	0.000849717	34.3412	smart00343	ZnF_C2HC	cl22700
Q#1275 - >Ca_15034.1|Ca_	superfamily	276654	65	79	0.000849717	34.3412	cl22700	zf-CCHC superfamily	-
Q#1275 - >Ca_15034.1|Ca_	multi-dom	214636	3	64	1.72377e-13	61.4555	smart00360	RRM	-
Q#1276 - >Ca_15037.1|Ca_	specific	214815	214	272	4.09869e-34	119.899	smart00774	WRKY	cl03892
Q#1276 - >Ca_15037.1|Ca_	superfamily	243565	214	272	4.09869e-34	119.899	cl03892	WRKY superfamily	-
Q#1276 - >Ca_15037.1|Ca_	specific	192617	167	212	1.99983e-16	71.2531	pfam10533	Plant_zn_clust	cl11166
Q#1276 - >Ca_15037.1|Ca_	superfamily	192617	167	212	1.99983e-16	71.2531	cl11166	Plant_zn_clust superfamily	-
Q#1277 - >Ca_15039.1|Ca_	specific	176359	10	95	2.15472e-35	117.719	cd01763	Sumo	cl00155
Q#1277 - >Ca_15039.1|Ca_	superfamily	275476	10	95	2.15472e-35	117.719	cl00155	UBQ superfamily	-
Q#1278 - >Ca_15051.1|Ca_	superfamily	276192	3	64	3.43591e-26	94.9946	cl17169	RRM_SF superfamily	-
Q#1278 - >Ca_15051.1|Ca_	specific	197667	65	79	0.000543118	34.7264	smart00343	ZnF_C2HC	cl22700
Q#1278 - >Ca_15051.1|Ca_	superfamily	276654	65	79	0.000543118	34.7264	cl22700	zf-CCHC superfamily	-
Q#1278 - >Ca_15051.1|Ca_	multi-dom	214636	3	64	1.31037e-13	61.8407	smart00360	RRM	-
Q#1279 - >Ca_15054.1|Ca_	specific	192617	167	212	7.05684e-16	68.9419	pfam10533	Plant_zn_clust	cl11166
Q#1279 - >Ca_15054.1|Ca_	superfamily	192617	167	212	7.05684e-16	68.9419	cl11166	Plant_zn_clust superfamily	-
Q#1279 - >Ca_15054.1|Ca_	specific	214815	214	235	2.67493e-10	54.0296	smart00774	WRKY	cl03892
Q#1279 - >Ca_15054.1|Ca_	superfamily	243565	214	235	2.67493e-10	54.0296	cl03892	WRKY superfamily	-
Q#1280 - >Ca_15056.1|Ca_	specific	176359	10	95	2.15472e-35	117.719	cd01763	Sumo	cl00155
Q#1280 - >Ca_15056.1|Ca_	superfamily	275476	10	95	2.15472e-35	117.719	cl00155	UBQ superfamily	-
Q#1281 - >Ca_15097.1|Ca_	specific	107221	53	143	4.39701e-28	101.862	cd06464	ACD_sHsps-like	cl00175
Q#1281 - >Ca_15097.1|Ca_	superfamily	271432	53	143	4.39701e-28	101.862	cl00175	alpha-crystallin-Hsps_p23-like superfamily	-
Q#1281 - >Ca_15097.1|Ca_	multi-dom	223149	1	143	1.05742e-21	86.6618	COG0071	IbpA	-
Q#1282 - >Ca_15117.1|Ca_	superfamily	276299	9	50	4.75607e-20	81.726	cl21455	ABC_ATPase superfamily	-
Q#1282 - >Ca_15117.1|Ca_	superfamily	276299	62	92	2.47482e-06	44.4456	cl21455	ABC_ATPase superfamily	-

Query	Types	PSSM-ID	From	To	E-Value	Bitscore	Accession	Short name	Superfamily
Q#1284 - >Ca_15145.1|Ca_	superfamily	260211	10	79	1.60584e-14	66.6778	cl00137	SERPIN superfamily	-
Q#1285 - >Ca_15180.1|Ca_	multi-dom	215633	49	212	1.08165e-19	93.4006	PLN03210	PLN03210	-
Q#1286 - >Ca_15187.1|Ca_	superfamily	251669	202	312	4.51767e-15	72.8324	cl03830	Transposase_24 superfamily	-
Q#1286 - >Ca_15187.1|Ca_	superfamily	276263	431	625	4.70523e-14	71.2364	cl19514	FBA_1 superfamily	-
Q#1287 - >Ca_15209.1|Ca_	specific	206660	11	175	1.31899e-124	352.633	cd01868	Rab11_like	cl21455
Q#1287 - >Ca_15209.1|Ca_	superfamily	276299	11	175	1.31899e-124	352.633	cl21455	ABC_ATPase superfamily	-
Q#1287 - >Ca_15209.1|Ca_	multi-dom	178657	4	217	1.12898e-118	339.597	PLN03110	PLN03110	-
Q#1288 - >Ca_15222.1|Ca_	specific	251348	10	87	2.46914e-38	125.11	pfam02519	Auxin_inducible	cl03633
Q#1288 - >Ca_15222.1|Ca_	superfamily	261812	10	87	2.46914e-38	125.11	cl03633	Auxin_inducible superfamily	-
Q#1289 - >Ca_15225.1|Ca_	superfamily	275846	1	176	3.48482e-59	189.756	cl07893	AmyAc_family superfamily	-
Q#1290 - >Ca_15267.1|Ca_	specific	252637	176	269	1.22374e-30	113.154	pfam04504	DUF573	cl04551
Q#1290 - >Ca_15267.1|Ca_	superfamily	252637	176	269	1.22374e-30	113.154	cl04551	DUF573 superfamily	-
Q#1291 - >Ca_15295.1|Ca_	specific	191945	66	84	3.06668e-06	39.8617	pfam08137	DVL	cl06945
Q#1291 - >Ca_15295.1|Ca_	superfamily	191945	66	84	3.06668e-06	39.8617	cl06945	DVL superfamily	-
Q#1292 - >Ca_15319.1|Ca_	superfamily	258203	41	138	8.8164e-06	42.6047	cl16494	GUB_WAK_bind superfamily	-
Q#1293 - >Ca_15323.1|Ca_	superfamily	258203	41	138	8.8164e-06	42.6047	cl16494	GUB_WAK_bind superfamily	-
Q#1294 - >Ca_15390.1|Ca_	superfamily	252881	287	387	1.46716e-11	61.513	cl08427	PAP_RNA-bind superfamily	-
Q#1294 - >Ca_15390.1|Ca_	superfamily	276009	50	98	1.81199e-08	51.7891	cl11966	Rel-Spo_like superfamily	-
Q#1294 - >Ca_15390.1|Ca_	multi-dom	252883	14	281	1.82984e-108	326.536	pfam04928	PAP_central	-
Q#1298 - >Ca_15494.1|Ca_	specific	99731	1	101	3.16895e-37	130.418	cd06410	PB1_UP2	cl02720
Q#1298 - >Ca_15494.1|Ca_	superfamily	261422	1	101	3.16895e-37	130.418	cl02720	PB1 superfamily	-
Q#1299 - >Ca_15510.1|Ca_	specific	253452	40	235	3.05155e-92	275.019	pfam05910	DUF868	cl05466
Q#1299 - >Ca_15510.1|Ca_	superfamily	253452	40	235	3.05155e-92	275.019	cl05466	DUF868 superfamily	-
Q#1300 - >Ca_15537.1|Ca_	specific	252003	3	96	9.04325e-61	186.252	pfam03501	S10_plectin	cl02102
Q#1300 - >Ca_15537.1|Ca_	superfamily	261176	3	96	9.04325e-61	186.252	cl02102	S10_plectin superfamily	-
Q#1301 - >Ca_15555.1|Ca_	specific	214823	224	404	8.26857e-54	180.469	smart00799	DENN	cl11519
Q#1301 - >Ca_15555.1|Ca_	superfamily	264408	224	404	8.26857e-54	180.469	cl11519	DENN superfamily	-
Q#1301 - >Ca_15555.1|Ca_	superfamily	262011	1	90	4.39243e-07	47.7145	cl04085	uDENN superfamily	-
Q#1302 - >Ca_15603.1|Ca_	specific	251348	4	84	4.43205e-23	86.5896	pfam02519	Auxin_inducible	cl03633
Q#1302 - >Ca_15603.1|Ca_	superfamily	261812	4	84	4.43205e-23	86.5896	cl03633	Auxin_inducible superfamily	-
Q#1303 - >Ca_15666.1|Ca_	specific	199209	9	92	2.44852e-22	84.5432	cd00463	Ribosomal_L31e	cl00921
Q#1303 - >Ca_15666.1|Ca_	superfamily	260697	9	92	2.44852e-22	84.5432	cl00921	Ribosomal_L31e superfamily	-
Q#1305 - >Ca_15713.1|Ca_	superfamily	276192	11	79	1.5839e-17	73.4164	cl17169	RRM_SF superfamily	-
Q#1305 - >Ca_15713.1|Ca_	multi-dom	214636	11	79	1.45945e-12	59.5295	smart00360	RRM	-
Q#1306 - >Ca_15720.1|Ca_	specific	250365	1	142	1.91761e-19	80.3884	pfam01105	EMP24_GP25L	cl03099
Q#1306 - >Ca_15720.1|Ca_	superfamily	250365	1	142	1.91761e-19	80.3884	cl03099	EMP24_GP25L superfamily	-

Query	Types	PSSM-ID	From	To	E-Value	Bitscore	Accession	Short name	Superfamily
Q#1307 - >Ca_15787.1|Ca_	specific	250941	54	230	9.44565e-60	188.906	pfam01885	PTS_2-RNA	cl19470
Q#1307 - >Ca_15787.1|Ca_	superfamily	267823	54	230	9.44565e-60	188.906	cl19470	PTS_2-RNA superfamily	-
Q#1308 - >Ca_15813.1|Ca_	specific	238093	139	185	2.26836e-09	50.9072	cd00162	RING	cl17238
Q#1308 - >Ca_15813.1|Ca_	superfamily	276201	139	185	2.26836e-09	50.9072	cl17238	RING superfamily	-
Q#1311 - >Ca_15962.1|Ca_	superfamily	253129	190	405	2.2047e-17	79.8823	cl12213	PEARLI-4 superfamily	-
Q#1314 - >Ca_16035.1|Ca_	superfamily	276192	32	60	1.50736e-09	49.9427	cl17169	RRM_SF superfamily	-
Q#1314 - >Ca_16035.1|Ca_	multi-dom	214636	21	59	1.20326e-06	41.8104	smart00360	RRM	-
Q#1319 - >Ca_16076.1|Ca_	specific	107229	50	141	4.94813e-54	167.867	cd06472	ACD_ScHsp26_like	cl00175
Q#1319 - >Ca_16076.1|Ca_	superfamily	271432	50	141	4.94813e-54	167.867	cl00175	alpha-crystallin-Hsps_p23-like superfamily	-
Q#1319 - >Ca_16076.1|Ca_	multi-dom	223149	38	155	4.36581e-27	100.144	COG0071	IbpA	-
Q#1320 - >Ca_16104.1|Ca_	superfamily	275808	33	78	4.04782e-08	47.4329	cl04375	PMEI_like superfamily	-
Q#1321 - >Ca_16117.1|Ca_	superfamily	215579	1	469	7.1205e-114	345.524	cl15944	TCP2 superfamily	-
Q#1322 - >Ca_16139.1|Ca_	specific	250972	193	283	0.00850439	35.6431	pfam01925	TauE	cl21514
Q#1322 - >Ca_16139.1|Ca_	superfamily	276337	193	283	0.00850439	35.6431	cl21514	TauE superfamily	-
Q#1325 - >Ca_16210.1|Ca_	specific	214845	14	154	1.80791e-21	88.1063	smart00835	Cupin_1	cl21464
Q#1325 - >Ca_16210.1|Ca_	superfamily	276307	14	154	1.80791e-21	88.1063	cl21464	ABD superfamily	-
Q#1325 - >Ca_16210.1|Ca_	specific	214845	202	302	2.07719e-15	71.1575	smart00835	Cupin_1	cl21464
Q#1325 - >Ca_16210.1|Ca_	superfamily	276307	202	302	2.07719e-15	71.1575	cl21464	ABD superfamily	-
Q#1326 - >Ca_16231.1|Ca_	specific	197867	33	117	4.80438e-36	122.364	smart00768	X8	cl06842
Q#1326 - >Ca_16231.1|Ca_	superfamily	263067	33	117	4.80438e-36	122.364	cl06842	X8 superfamily	-
Q#1328 - >Ca_16248.1|Ca_	specific	238284	22	122	2.61473e-57	175.796	cd00513	Ribosomal_L32_L32e	cl00748
Q#1328 - >Ca_16248.1|Ca_	superfamily	260601	22	122	2.61473e-57	175.796	cl00748	Ribosomal_L32_L32e superfamily	-
Q#1330 - >Ca_16325.1|Ca_	specific	273253	119	152	0.00431077	33.1975	TIGR00756	PPR	cl03252
Q#1330 - >Ca_16325.1|Ca_	superfamily	275791	119	152	0.00431077	33.1975	cl03252	PPR superfamily	-
Q#1330 - >Ca_16325.1|Ca_	multi-dom	257477	5	64	0.000503853	35.8414	pfam13041	PPR_2	-
Q#1331 - >Ca_16326.1|Ca_	superfamily	276007	254	434	5.1581e-64	213.449	cl11961	ALDH-SF superfamily	-
Q#1331 - >Ca_16326.1|Ca_	superfamily	276282	1	46	4.93504e-17	77.6517	cl19911	CBM_4_9 superfamily	-
Q#1331 - >Ca_16326.1|Ca_	superfamily	275583	184	238	5.40333e-16	76.702	cl00452	AAK superfamily	-
Q#1331 - >Ca_16326.1|Ca_	superfamily	276282	81	170	0.00790505	35.8037	cl19911	CBM_4_9 superfamily	-
Q#1332 - >Ca_16327.1|Ca_	specific	189387	216	232	0.00118217	35.8929	pfam00098	zf-CCHC	cl22700
Q#1332 - >Ca_16327.1|Ca_	superfamily	276654	216	232	0.00118217	35.8929	cl22700	zf-CCHC superfamily	-
Q#1332 - >Ca_16327.1|Ca_	multi-dom	173561	156	232	0.00120071	37.4789	PTZ00368	PTZ00368	-
Q#1333 - >Ca_16363.1|Ca_	specific	206660	11	194	7.76953e-120	341.077	cd01868	Rab11_like	cl21455
Q#1333 - >Ca_16363.1|Ca_	superfamily	276299	11	194	7.76953e-120	341.077	cl21455	ABC_ATPase superfamily	-
Q#1333 - >Ca_16363.1|Ca_	multi-dom	178657	3	235	5.64848e-112	323.034	PLN03110	PLN03110	-
Q#1335 - >Ca_16385.1|Ca_	superfamily	264523	85	314	2.9116e-37	138.639	cl12050	TraB superfamily	-

Query	Types	PSSM-ID	From	To	E-Value	Bitscore	Accession	Short name	Superfamily
Q#1337 - >Ca_16499.1|Ca_	specific	238037	19	70	1.23096e-07	44.1468	cd00084	HMG-box	cl00082
Q#1337 - >Ca_16499.1|Ca_	superfamily	260179	19	70	1.23096e-07	44.1468	cl00082	HMG-box superfamily	-
Q#1338 - >Ca_16544.1|Ca_	multi-dom	275319	28	92	0.0043385	33.8385	TIGR04526	predic_Ig_block	-
Q#1339 - >Ca_16553.1|Ca_	specific	258843	15	79	4.32376e-30	103.332	pfam14705	Costars	cl20642
Q#1339 - >Ca_16553.1|Ca_	superfamily	258843	15	79	4.32376e-30	103.332	cl20642	Costars superfamily	-
Q#1341 - >Ca_16589.1|Ca_	superfamily	252966	40	161	3.62391e-70	215.351	cl10635	Caleosin superfamily	-
Q#1341 - >Ca_16589.1|Ca_	superfamily	276193	1	34	2.34953e-11	58.349	cl17171	PH-like superfamily	-
Q#1341 - >Ca_16589.1|Ca_	specific	258173	206	228	0.00023029	37.6087	pfam13906	AA_permease_C	cl18287
Q#1341 - >Ca_16589.1|Ca_	superfamily	258173	206	228	0.00023029	37.6087	cl18287	AA_permease_C superfamily	-
Q#1343 - >Ca_16597.1|Ca_	specific	206660	11	194	7.76953e-120	341.077	cd01868	Rab11_like	cl21455
Q#1343 - >Ca_16597.1|Ca_	superfamily	276299	11	194	7.76953e-120	341.077	cl21455	ABC_ATPase superfamily	-
Q#1343 - >Ca_16597.1|Ca_	multi-dom	178657	3	235	5.64848e-112	323.034	PLN03110	PLN03110	-
Q#1344 - >Ca_16601.1|Ca_	superfamily	276241	29	121	2.34018e-05	40.6794	cl19217	Mem_trans superfamily	-
Q#1345 - >Ca_16627.1|Ca_	superfamily	276298	1	108	4.2355e-34	121.143	cl21454	SDR superfamily	-
Q#1346 - >Ca_16629.1|Ca_	superfamily	276298	9	52	2.01301e-25	98.8009	cl21454	SDR superfamily	-
Q#1347 - >Ca_16640.1|Ca_	superfamily	276305	60	105	1.86364e-08	46.9224	cl21462	bZIP superfamily	-
Q#1348 - >Ca_16657.1|Ca_	specific	254686	68	108	5.58859e-06	41.5949	pfam08242	Methyltransf_12	cl17173
Q#1348 - >Ca_16657.1|Ca_	superfamily	276194	68	108	5.58859e-06	41.5949	cl17173	AdoMet_MTases superfamily	-
Q#1349 - >Ca_16739.1|Ca_	specific	253250	162	186	3.06839e-09	49.7969	pfam05553	DUF761	cl05229
Q#1349 - >Ca_16739.1|Ca_	superfamily	253250	162	186	3.06839e-09	49.7969	cl05229	DUF761 superfamily	-
Q#1350 - >Ca_16751.1|Ca_	superfamily	256046	68	179	1.33016e-24	97.4652	cl11168	PMD superfamily	-
Q#1351 - >Ca_16765.1|Ca_	specific	107229	54	145	2.22479e-54	169.023	cd06472	ACD_ScHsp26_like	cl00175
Q#1351 - >Ca_16765.1|Ca_	superfamily	271432	54	145	2.22479e-54	169.023	cl00175	alpha-crystallin-Hsps_p23-like superfamily	-
Q#1351 - >Ca_16765.1|Ca_	multi-dom	223149	55	159	1.5693e-26	98.9882	COG0071	IbpA	-
Q#1352 - >Ca_16770.1|Ca_	superfamily	275927	6	228	4.14612e-35	128.623	cl11403	pepsin_retropepsin_like superfamily	-
Q#1353 - >Ca_16838.1|Ca_	superfamily	253839	1	94	5.20961e-39	131.707	cl05927	ATP11 superfamily	-
Q#1355 - >Ca_16847.1|Ca_	specific	238287	2	184	4.3832e-31	116.037	cd00519	Lipase_3	cl21494
Q#1355 - >Ca_16847.1|Ca_	superfamily	276326	2	184	4.3832e-31	116.037	cl21494	Esterase_lipase superfamily	-
Q#1356 - >Ca_16858.1|Ca_	multi-dom	235600	16	96	0.00245734	37.2144	PRK05771	PRK05771	-
Q#1357 - >Ca_16885.1|Ca_	superfamily	256046	92	218	8.31057e-19	82.4424	cl11168	PMD superfamily	-
Q#1358 - >Ca_16889.1|Ca_	specific	238166	2	85	9.13862e-22	86.1599	cd00266	MADS_SRF_like	cl00109
Q#1358 - >Ca_16889.1|Ca_	superfamily	241616	2	85	9.13862e-22	86.1599	cl00109	MADS superfamily	-
Q#1359 - >Ca_16927.1|Ca_	specific	250253	131	199	1.92441e-31	111.862	pfam00957	Synaptobrevin	cl09522
Q#1359 - >Ca_16927.1|Ca_	superfamily	250253	131	199	1.92441e-31	111.862	cl09522	Synaptobrevin superfamily	-
Q#1359 - >Ca_16927.1|Ca_	specific	258059	35	116	1.14951e-27	101.867	pfam13774	Longin	cl16386
Q#1359 - >Ca_16927.1|Ca_	superfamily	258059	35	116	1.14951e-27	101.867	cl16386	Longin superfamily	-

Query	Types	PSSM-ID	From	To	E-Value	Bitscore	Accession	Short name	Superfamily
Q#1360 - >Ca_16929.1|Ca_	specific	258183	221	259	4.8177e-07	45.4571	pfam13920	zf-C3HC4_3	cl17238
Q#1360 - >Ca_16929.1|Ca_	superfamily	276201	221	259	4.8177e-07	45.4571	cl17238	RING superfamily	-
Q#1362 - >Ca_16984.1|Ca_	specific	258478	1	148	2.33113e-56	175.45	pfam14299	PP2	cl16784
Q#1362 - >Ca_16984.1|Ca_	superfamily	258478	1	148	2.33113e-56	175.45	cl16784	PP2 superfamily	-
Q#1363 - >Ca_17024.1|Ca_	specific	206660	11	175	3.02049e-123	349.166	cd01868	Rab11_like	cl21455
Q#1363 - >Ca_17024.1|Ca_	superfamily	276299	11	175	3.02049e-123	349.166	cl21455	ABC_ATPase superfamily	-
Q#1363 - >Ca_17024.1|Ca_	multi-dom	178657	4	218	3.06242e-122	348.457	PLN03110	PLN03110	-
Q#1364 - >Ca_17031.1|Ca_	superfamily	252569	17	82	2.79384e-18	74.1413	cl04499	DUF538 superfamily	-
Q#1365 - >Ca_17039.1|Ca_	multi-dom	223021	15	165	0.000701616	39.9217	PHA03247	PHA03247	-
Q#1366 - >Ca_17063.1|Ca_	superfamily	251724	5	304	1.82019e-80	258.083	cl03887	Mlo superfamily	-
Q#1367 - >Ca_17074.1|Ca_	specific	238045	84	134	1.94281e-09	49.859	cd00093	HTH_XRE	cl21459
Q#1367 - >Ca_17074.1|Ca_	superfamily	276302	84	134	1.94281e-09	49.859	cl21459	HTH_XRE superfamily	-
Q#1367 - >Ca_17074.1|Ca_	specific	117100	8	78	1.30788e-18	75.4704	pfam08523	MBF1	cl07226
Q#1367 - >Ca_17074.1|Ca_	superfamily	117100	8	78	1.30788e-18	75.4704	cl07226	MBF1 superfamily	-
Q#1368 - >Ca_17080.1|Ca_	specific	253297	10	75	8.26415e-15	65.045	pfam05641	Agenet	cl02573
Q#1368 - >Ca_17080.1|Ca_	superfamily	275771	10	75	8.26415e-15	65.045	cl02573	TUDOR superfamily	-
Q#1368 - >Ca_17080.1|Ca_	superfamily	275771	81	138	0.00632809	32.2957	cl02573	TUDOR superfamily	-
Q#1369 - >Ca_17082.1|Ca_	specific	191112	6	138	4.52038e-76	226.253	pfam04852	DUF640	cl04800
Q#1369 - >Ca_17082.1|Ca_	superfamily	191112	6	138	4.52038e-76	226.253	cl04800	DUF640 superfamily	-
Q#1370 - >Ca_17085.1|Ca_	specific	238036	48	97	7.30268e-09	50.2903	cd00083	HLH	cl00081
Q#1370 - >Ca_17085.1|Ca_	superfamily	260178	48	97	7.30268e-09	50.2903	cl00081	HLH superfamily	-
Q#1371 - >Ca_17113.1|Ca_	specific	239904	6	65	1.41962e-26	92.6983	cd04457	S1_S28E	cl09927
Q#1371 - >Ca_17113.1|Ca_	superfamily	275904	6	65	1.41962e-26	92.6983	cl09927	S1_like superfamily	-
Q#1372 - >Ca_17115.1|Ca_	superfamily	276298	1	44	2.37364e-15	65.9301	cl21454	SDR superfamily	-
Q#1376 - >Ca_17149.1|Ca_	superfamily	253938	28	58	0.000106607	35.4425	cl06070	zf-GRF superfamily	-
Q#1377 - >Ca_17150.1|Ca_	specific	214632	53	77	6.09366e-05	38.7645	smart00356	ZnF_C3H1	cl11592
Q#1377 - >Ca_17150.1|Ca_	superfamily	264437	53	77	6.09366e-05	38.7645	cl11592	zf-CCCH superfamily	-
Q#1379 - >Ca_17186.1|Ca_	specific	252179	29	102	5.94899e-13	59.9385	pfam03810	IBN_N	cl04271
Q#1379 - >Ca_17186.1|Ca_	superfamily	262079	29	102	5.94899e-13	59.9385	cl04271	IBN_N superfamily	-
Q#1380 - >Ca_17197.1|Ca_	superfamily	275925	109	166	2.92765e-05	44.1002	cl11394	Glyco_tranf_GTA_type superfamily	-
Q#1380 - >Ca_17197.1|Ca_	superfamily	275925	364	404	0.000536882	40.2482	cl11394	Glyco_tranf_GTA_type superfamily	-
Q#1380 - >Ca_17197.1|Ca_	multi-dom	215138	30	486	0	685.994	PLN02248	PLN02248	-
Q#1381 - >Ca_17223.1|Ca_	specific	237985	99	156	5.94799e-32	111.595	cd00018	AP2	cl00033
Q#1381 - >Ca_17223.1|Ca_	superfamily	260146	99	156	5.94799e-32	111.595	cl00033	AP2 superfamily	-
Q#1382 - >Ca_17243.1|Ca_	superfamily	249606	137	178	0.00041914	39.0891	cl02811	E1-E2_ATPase superfamily	-
Q#1383 - >Ca_17249.1|Ca_	superfamily	275563	14	46	4.52631e-07	43.0076	cl00388	Thioredoxin_like superfamily	-

Query	Types	PSSM-ID	From	To	E-Value	Bitscore	Accession	Short name	Superfamily
Q#1384 - >Ca_17255.1|Ca_	superfamily	275846	4	48	1.82123e-12	59.607	cl07893	AmyAc_family superfamily	-
Q#1385 - >Ca_17260.1|Ca_	superfamily	276298	178	243	3.17767e-11	58.1156	cl21454	SDR superfamily	-
Q#1386 - >Ca_17274.1|Ca_	superfamily	275586	6	125	0.000106397	41.1294	cl00456	SLC5-6-like_sbd superfamily	-
Q#1386 - >Ca_17274.1|Ca_	superfamily	275586	108	193	0.00483892	36.1218	cl00456	SLC5-6-like_sbd superfamily	-
Q#1387 - >Ca_17307.1|Ca_	superfamily	265660	295	378	0.00675228	36.3721	cl15270	FinO_conjug_rep superfamily	-
Q#1396 - >Ca_17441.1|Ca_	multi-dom	255846	42	103	0.008474	36.4162	pfam10243	MIP-T3	-
Q#1401 - >Ca_17463.1|Ca_	multi-dom	273953	5	87	0.000881976	36.881	TIGR02072	Malonyl-_O-methyltransferase	-
Q#1403 - >Ca_17480.1|Ca_	superfamily	275482	1	174	6.41868e-68	211.259	cl00200	MIP superfamily	-
Q#1404 - >Ca_17484.1|Ca_	superfamily	276663	184	296	0.00177073	37.692	cl22709	AMN1 superfamily	-
Q#1405 - >Ca_17492.1|Ca_	specific	251348	1	91	9.72599e-38	123.954	pfam02519	Auxin_inducible	cl03633
Q#1405 - >Ca_17492.1|Ca_	superfamily	261812	1	91	9.72599e-38	123.954	cl03633	Auxin_inducible superfamily	-
Q#1406 - >Ca_17494.1|Ca_	specific	251348	1	90	3.13583e-36	120.102	pfam02519	Auxin_inducible	cl03633
Q#1406 - >Ca_17494.1|Ca_	superfamily	261812	1	90	3.13583e-36	120.102	cl03633	Auxin_inducible superfamily	-
Q#1407 - >Ca_17497.1|Ca_	specific	251348	2	89	3.42054e-40	130.117	pfam02519	Auxin_inducible	cl03633
Q#1407 - >Ca_17497.1|Ca_	superfamily	261812	2	89	3.42054e-40	130.117	cl03633	Auxin_inducible superfamily	-
Q#1408 - >Ca_17504.1|Ca_	specific	251348	2	89	3.43082e-37	122.413	pfam02519	Auxin_inducible	cl03633
Q#1408 - >Ca_17504.1|Ca_	superfamily	261812	2	89	3.43082e-37	122.413	cl03633	Auxin_inducible superfamily	-
Q#1409 - >Ca_17505.1|Ca_	specific	251348	9	84	2.16967e-35	117.406	pfam02519	Auxin_inducible	cl03633
Q#1409 - >Ca_17505.1|Ca_	superfamily	261812	9	84	2.16967e-35	117.406	cl03633	Auxin_inducible superfamily	-
Q#1413 - >Ca_17544.1|Ca_	specific	110442	2	74	6.09413e-14	60.9935	pfam01439	Metallothio_2	cl03212
Q#1413 - >Ca_17544.1|Ca_	superfamily	110442	2	74	6.09413e-14	60.9935	cl03212	Metallothio_2 superfamily	-
Q#1414 - >Ca_17546.1|Ca_	multi-dom	237864	320	500	0.00865265	37.8668	PRK14950	PRK14950	-
Q#1417 - >Ca_17556.1|Ca_	specific	133136	362	406	3.37941e-06	44.6348	cd00303	retropepsin_like	cl11403
Q#1417 - >Ca_17556.1|Ca_	superfamily	275927	362	406	3.37941e-06	44.6348	cl11403	pepsin_retropepsin_like superfamily	-
Q#1417 - >Ca_17556.1|Ca_	specific	252133	100	199	3.61717e-13	65.4377	pfam03732	Retrotrans_gag	cl04237
Q#1417 - >Ca_17556.1|Ca_	superfamily	252133	100	199	3.61717e-13	65.4377	cl04237	Retrotrans_gag superfamily	-
Q#1417 - >Ca_17556.1|Ca_	specific	189387	305	322	0.00272599	35.5077	pfam00098	zf-CCHC	cl22700
Q#1417 - >Ca_17556.1|Ca_	superfamily	276654	305	322	0.00272599	35.5077	cl22700	zf-CCHC superfamily	-
Q#1420 - >Ca_17576.1|Ca_	multi-dom	178496	70	136	1.73076e-34	126.425	PLN02908	PLN02908	-
Q#1421 - >Ca_17579.1|Ca_	multi-dom	258434	48	123	1.76611e-16	73.1562	pfam14244	UBN2_3	-
Q#1422 - >Ca_17589.1|Ca_	specific	258213	19	47	5.63124e-05	36.2498	pfam13961	DUF4219	cl22382
Q#1422 - >Ca_17589.1|Ca_	superfamily	258213	19	47	5.63124e-05	36.2498	cl22382	DUF4219 superfamily	-
Q#1426 - >Ca_17603.1|Ca_	superfamily	253938	28	58	0.000123695	35.4425	cl06070	zf-GRF superfamily	-
Q#1427 - >Ca_17616.1|Ca_	specific	250692	116	146	4.92544e-06	40.5149	pfam01535	PPR	cl03252
Q#1427 - >Ca_17616.1|Ca_	superfamily	275791	116	146	4.92544e-06	40.5149	cl03252	PPR superfamily	-
Q#1427 - >Ca_17616.1|Ca_	superfamily	275791	5	33	0.000205708	36.1733	cl03252	PPR superfamily	-

Query	Types	PSSM-ID	From	To	E-Value	Bitscore	Accession	Short name	Superfamily
Q#1427 - >Ca_17616.1|Ca_	multi-dom	257477	115	156	1.92482e-07	44.3158	pfam13041	PPR_2	-
Q#1427 - >Ca_17616.1|Ca_	multi-dom	257477	4	47	4.60547e-05	38.1526	pfam13041	PPR_2	-
Q#1427 - >Ca_17616.1|Ca_	multi-dom	257477	74	127	0.00672543	32.3746	pfam13041	PPR_2	-
Q#1430 - >Ca_17650.1|Ca_	specific	258415	90	199	1.83886e-47	156.194	pfam14223	UBN2	cl15874
Q#1430 - >Ca_17650.1|Ca_	superfamily	265821	90	199	1.83886e-47	156.194	cl15874	UBN2 superfamily	-
Q#1430 - >Ca_17650.1|Ca_	specific	258213	13	37	1.39825e-05	41.2574	pfam13961	DUF4219	cl22382
Q#1430 - >Ca_17650.1|Ca_	superfamily	258213	13	37	1.39825e-05	41.2574	cl22382	DUF4219 superfamily	-
Q#1432 - >Ca_17653.1|Ca_	superfamily	256046	9	96	0.000286617	39.3	cl11168	PMD superfamily	-
Q#1433 - >Ca_17668.1|Ca_	superfamily	271593	125	266	9.92599e-18	80.4568	cl02567	WD40 superfamily	-
Q#1433 - >Ca_17668.1|Ca_	multi-dom	225201	79	334	4.6213e-16	77.4363	COG2319	COG2319	-
Q#1438 - >Ca_17710.1|Ca_	superfamily	271581	53	88	0.00497574	35.4114	cl02432	CLECT superfamily	-
Q#1440 - >Ca_17714.1|Ca_	superfamily	251718	127	343	1.28145e-33	128.228	cl03878	Exo70 superfamily	-
Q#1441 - >Ca_17722.1|Ca_	superfamily	271432	47	131	1.0918e-24	91.5976	cl00175	alpha-crystallin-Hsps_p23-like superfamily	-
Q#1442 - >Ca_17723.1|Ca_	superfamily	252825	1	126	6.3715e-30	107.342	cl04786	SOUL superfamily	-
Q#1443 - >Ca_17725.1|Ca_	superfamily	256046	16	118	3.80765e-24	99.006	cl11168	PMD superfamily	-
Q#1446 - >Ca_17740.1|Ca_	superfamily	275476	87	112	1.09529e-07	45.4175	cl00155	UBQ superfamily	-
Q#1446 - >Ca_17740.1|Ca_	superfamily	261398	6	33	0.000135072	36.7696	cl02662	SEP superfamily	-
Q#1450 - >Ca_17781.1|Ca_	superfamily	276299	1	115	1.45441e-45	153.541	cl21455	ABC_ATPase superfamily	-
Q#1451 - >Ca_17786.1|Ca_	superfamily	275753	8	132	8.95319e-75	230.191	cl01911	Pectinesterase superfamily	-
Q#1453 - >Ca_17796.1|Ca_	superfamily	252133	64	119	0.00128378	35.7773	cl04237	Retrotrans_gag superfamily	-
Q#1454 - >Ca_17798.1|Ca_	superfamily	261455	626	665	3.5369e-12	64.9241	cl02808	RT_like superfamily	-
Q#1454 - >Ca_17798.1|Ca_	superfamily	252133	222	295	8.31914e-09	53.4965	cl04237	Retrotrans_gag superfamily	-
Q#1454 - >Ca_17798.1|Ca_	specific	189387	398	414	0.000106715	40.5153	pfam00098	zf-CCHC	cl22700
Q#1454 - >Ca_17798.1|Ca_	superfamily	276654	398	414	0.000106715	40.5153	cl22700	zf-CCHC superfamily	-
Q#1454 - >Ca_17798.1|Ca_	superfamily	275927	463	507	0.000956026	38.5547	cl11403	pepsin_retropepsin_like superfamily	-
Q#1454 - >Ca_17798.1|Ca_	specific	197667	379	395	0.00349076	36.2672	smart00343	ZnF_C2HC	cl22700
Q#1454 - >Ca_17798.1|Ca_	superfamily	276654	379	395	0.00349076	36.2672	cl22700	zf-CCHC superfamily	-
Q#1454 - >Ca_17798.1|Ca_	superfamily	271610	17	119	0.00383089	38.352	cl03114	RNase_PH superfamily	-
Q#1457 - >Ca_17807.1|Ca_	superfamily	251669	1	44	0.00154678	33.9273	cl03830	Transposase_24 superfamily	-
Q#1459 - >Ca_17824.1|Ca_	multi-dom	223021	45	331	4.02224e-07	52.6333	PHA03247	PHA03247	-
Q#1460 - >Ca_17837.1|Ca_	specific	238088	39	129	3.79996e-13	62.5933	cd00156	REC	cl19078
Q#1460 - >Ca_17837.1|Ca_	superfamily	271833	39	129	3.79996e-13	62.5933	cl19078	REC superfamily	-
Q#1460 - >Ca_17837.1|Ca_	superfamily	271918	162	211	3.19284e-11	55.8729	cl21498	SANT superfamily	-
Q#1461 - >Ca_17838.1|Ca_	superfamily	254387	56	186	1.73542e-66	213.087	cl06662	RVT_2 superfamily	-
Q#1463 - >Ca_17843.1|Ca_	superfamily	252133	130	223	9.64232e-11	57.3485	cl04237	Retrotrans_gag superfamily	-
Q#1466 - >Ca_17847.1|Ca_	specific	235532	2	79	6.67326e-32	108.703	PRK05610	rpsQ	cl00351

Query	Types	PSSM-ID	From	To	E-Value	Bitscore	Accession	Short name	Superfamily
Q#1466 - >Ca_17847.1|Ca_	superfamily	275545	2	79	6.67326e-32	108.703	cl00351	Ribosomal_S17 superfamily	-
Q#1468 - >Ca_17857.1|Ca_	superfamily	276299	78	128	9.83913e-22	87.2098	cl21455	ABC_ATPase superfamily	-
Q#1469 - >Ca_17864.1|Ca_	superfamily	276192	33	68	2.02756e-09	53.3068	cl17169	RRM_SF superfamily	-
Q#1469 - >Ca_17864.1|Ca_	superfamily	165431	200	286	0.00153727	38.5304	cl20105	PHA03160 superfamily	-
Q#1472 - >Ca_17880.1|Ca_	specific	251676	44	185	5.58277e-66	202.821	pfam03018	Dirigent	cl03841
Q#1472 - >Ca_17880.1|Ca_	superfamily	251676	44	185	5.58277e-66	202.821	cl03841	Dirigent superfamily	-
Q#1473 - >Ca_17881.1|Ca_	specific	251676	51	197	6.84168e-65	198.969	pfam03018	Dirigent	cl03841
Q#1473 - >Ca_17881.1|Ca_	superfamily	251676	51	197	6.84168e-65	198.969	cl03841	Dirigent superfamily	-
Q#1475 - >Ca_17886.1|Ca_	specific	145969	71	129	4.6139e-37	126.191	pfam03106	WRKY	cl03892
Q#1475 - >Ca_17886.1|Ca_	superfamily	243565	71	129	4.6139e-37	126.191	cl03892	WRKY superfamily	-
Q#1477 - >Ca_17905.1|Ca_	specific	133136	185	257	7.30886e-09	51.1832	cd00303	retropepsin_like	cl11403
Q#1477 - >Ca_17905.1|Ca_	superfamily	275927	185	257	7.30886e-09	51.1832	cl11403	pepsin_retropepsin_like superfamily	-
Q#1481 - >Ca_17935.1|Ca_	superfamily	258207	104	162	8.15581e-08	49.8871	cl16495	DUF4216 superfamily	-
Q#1481 - >Ca_17935.1|Ca_	superfamily	251669	368	421	1.94808e-05	43.1721	cl03830	Transposase_24 superfamily	-
Q#1483 - >Ca_17938.1|Ca_	superfamily	251669	151	234	9.02104e-13	63.2024	cl03830	Transposase_24 superfamily	-
Q#1484 - >Ca_17940.1|Ca_	multi-dom	253298	28	115	0.00130485	40.0495	pfam05642	Sporozoite_P67	-
Q#1485 - >Ca_17946.1|Ca_	superfamily	263454	29	84	9.03076e-13	59.5652	cl07747	Aha1_N superfamily	-
Q#1487 - >Ca_17972.1|Ca_	superfamily	275927	83	125	6.93301e-06	44.6695	cl11403	pepsin_retropepsin_like superfamily	-
Q#1487 - >Ca_17972.1|Ca_	superfamily	275927	147	177	0.00102369	38.1211	cl11403	pepsin_retropepsin_like superfamily	-
Q#1488 - >Ca_17973.1|Ca_	superfamily	272028	1	166	7.03442e-46	158.155	cl21608	Galactosyl_T superfamily	-
Q#1493 - >Ca_18038.1|Ca_	superfamily	271701	1	56	7.13659e-22	85.4692	cl10459	Peptidases_S8_S53 superfamily	-
Q#1493 - >Ca_18038.1|Ca_	superfamily	276299	95	119	1.88721e-05	40.5458	cl21455	ABC_ATPase superfamily	-
Q#1499 - >Ca_18085.1|Ca_	specific	238686	111	168	2.83466e-07	45.3114	cd01390	HMGB-UBF_HMG-box	cl00082
Q#1499 - >Ca_18085.1|Ca_	superfamily	260179	111	168	2.83466e-07	45.3114	cl00082	HMG-box superfamily	-
Q#1503 - >Ca_18175.1|Ca_	specific	260004	870	994	3.15381e-55	190.372	cd09272	RNase_HI_RT_Ty1	cl14782
Q#1503 - >Ca_18175.1|Ca_	superfamily	271754	870	994	3.15381e-55	190.372	cl14782	RNase_H_like superfamily	-
Q#1503 - >Ca_18175.1|Ca_	superfamily	254387	622	787	7.95547e-76	252.763	cl06662	RVT_2 superfamily	-
Q#1503 - >Ca_18175.1|Ca_	specific	258415	75	193	1.03231e-33	127.689	pfam14223	UBN2	cl15874
Q#1503 - >Ca_18175.1|Ca_	superfamily	265821	75	193	1.03231e-33	127.689	cl15874	UBN2 superfamily	-
Q#1504 - >Ca_18178.1|Ca_	specific	251988	271	329	8.4423e-08	48.7202	pfam03478	DUF295	cl04101
Q#1504 - >Ca_18178.1|Ca_	superfamily	251988	271	329	8.4423e-08	48.7202	cl04101	DUF295 superfamily	-
Q#1505 - >Ca_18183.1|Ca_	specific	100103	1	54	1.28415e-28	101.88	cd00472	Ribosomal_L24e_L24	cl00909
Q#1505 - >Ca_18183.1|Ca_	superfamily	275676	1	54	1.28415e-28	101.88	cl00909	Ribosomal_L24e_L24 superfamily	-
Q#1506 - >Ca_18185.1|Ca_	superfamily	276007	35	234	1.65454e-52	176.47	cl11961	ALDH-SF superfamily	-
Q#1507 - >Ca_18186.1|Ca_	superfamily	260240	25	105	0.00368999	34.6193	cl00182	Mth938-like superfamily	-
Q#1508 - >Ca_18191.1|Ca_	multi-dom	223021	60	235	1.80902e-07	52.6333	PHA03247	PHA03247	-

Query	Types	PSSM-ID	From	To	E-Value	Bitscore	Accession	Short name	Superfamily
Q#1509 - >Ca_18202.1|Ca_	specific	257351	22	50	3.79912e-06	42.3365	pfam12854	PPR_1	cl03252
Q#1509 - >Ca_18202.1|Ca_	superfamily	275791	22	50	3.79912e-06	42.3365	cl03252	PPR superfamily	-
Q#1509 - >Ca_18202.1|Ca_	multi-dom	257477	21	66	4.13406e-10	53.5606	pfam13041	PPR_2	-
Q#1511 - >Ca_18207.1|Ca_	superfamily	256046	63	160	6.81995e-19	85.1388	cl11168	PMD superfamily	-
Q#1511 - >Ca_18207.1|Ca_	specific	258228	258	322	3.90546e-13	63.9152	pfam13976	gag_pre-integrs	cl16514
Q#1511 - >Ca_18207.1|Ca_	superfamily	258228	258	322	3.90546e-13	63.9152	cl16514	gag_pre-integrs superfamily	-
Q#1513 - >Ca_18227.1|Ca_	superfamily	271660	58	113	1.64623e-13	65.2866	cl09326	MATE_like superfamily	-
Q#1514 - >Ca_18237.1|Ca_	superfamily	275927	28	146	3.24905e-05	41.5956	cl11403	pepsin_retropepsin_like superfamily	-
Q#1516 - >Ca_18247.1|Ca_	superfamily	256046	179	395	4.46101e-23	98.6208	cl11168	PMD superfamily	-
Q#1521 - >Ca_18277.1|Ca_	multi-dom	215601	1	58	1.86601e-16	72.911	PLN03142	PLN03142	-
Q#1523 - >Ca_18287.1|Ca_	specific	260004	276	331	1.79347e-27	105.243	cd09272	RNase_HI_RT_Ty1	cl14782
Q#1523 - >Ca_18287.1|Ca_	superfamily	271754	276	331	1.79347e-27	105.243	cl14782	RNase_H_like superfamily	-
Q#1523 - >Ca_18287.1|Ca_	specific	250040	13	89	7.04094e-13	63.8917	pfam00665	rve	cl21549
Q#1523 - >Ca_18287.1|Ca_	superfamily	276355	13	89	7.04094e-13	63.8917	cl21549	rve superfamily	-
Q#1523 - >Ca_18287.1|Ca_	superfamily	254387	241	259	0.00180296	37.8212	cl06662	RVT_2 superfamily	-
Q#1524 - >Ca_18290.1|Ca_	multi-dom	258434	46	109	1.37077e-18	78.549	pfam14244	UBN2_3	-
Q#1525 - >Ca_18294.1|Ca_	specific	238926	27	115	1.18686e-27	98.5761	cd01960	nsLTP1	cl07890
Q#1525 - >Ca_18294.1|Ca_	superfamily	275845	27	115	1.18686e-27	98.5761	cl07890	AAI_LTSS superfamily	-
Q#1527 - >Ca_18300.1|Ca_	specific	260004	9	145	3.65566e-66	200.772	cd09272	RNase_HI_RT_Ty1	cl14782
Q#1527 - >Ca_18300.1|Ca_	superfamily	271754	9	145	3.65566e-66	200.772	cl14782	RNase_H_like superfamily	-
Q#1530 - >Ca_18345.1|Ca_	specific	119392	78	225	1.7823e-15	75.0429	cd06174	MFS	cl21472
Q#1530 - >Ca_18345.1|Ca_	superfamily	276313	78	225	1.7823e-15	75.0429	cl21472	MFS superfamily	-
Q#1531 - >Ca_18349.1|Ca_	superfamily	276298	36	109	9.11242e-36	125.409	cl21454	SDR superfamily	-
Q#1533 - >Ca_18371.1|Ca_	specific	251315	7	121	5.69134e-17	73.4452	pfam02469	Fasciclin	cl02663
Q#1533 - >Ca_18371.1|Ca_	superfamily	261399	7	121	5.69134e-17	73.4452	cl02663	Fasciclin superfamily	-
Q#1534 - >Ca_18407.1|Ca_	specific	107229	49	140	3.60043e-55	170.563	cd06472	ACD_ScHsp26_like	cl00175
Q#1534 - >Ca_18407.1|Ca_	superfamily	271432	49	140	3.60043e-55	170.563	cl00175	alpha-crystallin-Hsps_p23-like superfamily	-
Q#1534 - >Ca_18407.1|Ca_	multi-dom	223149	13	154	1.15306e-27	101.685	COG0071	IbpA	-
Q#1535 - >Ca_18413.1|Ca_	specific	252240	56	96	1.07517e-14	63.3582	pfam03911	Sec61_beta	cl09194
Q#1535 - >Ca_18413.1|Ca_	superfamily	275871	56	96	1.07517e-14	63.3582	cl09194	Sec61_beta superfamily	-
Q#1536 - >Ca_18430.1|Ca_	specific	240513	52	105	7.1955e-14	62.5322	cd06089	KOW_RPL26	cl00354
Q#1536 - >Ca_18430.1|Ca_	superfamily	275547	52	105	7.1955e-14	62.5322	cl00354	KOW superfamily	-
Q#1537 - >Ca_18445.1|Ca_	specific	238117	17	159	1.03127e-59	184.328	cd00195	UBCc	cl00154
Q#1537 - >Ca_18445.1|Ca_	superfamily	275475	17	159	1.03127e-59	184.328	cl00154	UBCc superfamily	-
Q#1537 - >Ca_18445.1|Ca_	multi-dom	227410	18	162	4.21487e-61	188.24	COG5078	COG5078	-
Q#1539 - >Ca_18508.1|Ca_	specific	275442	4	100	2.37353e-15	67.4633	cd15798	PMEI-like_3	cl04375

Query	Types	PSSM-ID	From	To	E-Value	Bitscore	Accession	Short name	Superfamily
Q#1539 - >Ca_18508.1|Ca_	superfamily	275808	4	100	2.37353e-15	67.4633	cl04375	PMEI_like superfamily	-
Q#1540 - >Ca_18511.1|Ca_	specific	238036	47	91	1.06438e-10	54.5275	cd00083	HLH	cl00081
Q#1540 - >Ca_18511.1|Ca_	superfamily	260178	47	91	1.06438e-10	54.5275	cl00081	HLH superfamily	-
Q#1542 - >Ca_18548.1|Ca_	superfamily	252133	100	176	1.60972e-10	54.2669	cl04237	Retrotrans_gag superfamily	-
Q#1543 - >Ca_18549.1|Ca_	specific	237985	138	198	2.89841e-34	121.225	cd00018	AP2	cl00033
Q#1543 - >Ca_18549.1|Ca_	superfamily	260146	138	198	2.89841e-34	121.225	cl00033	AP2 superfamily	-
Q#1544 - >Ca_18559.1|Ca_	specific	252408	32	132	1.39449e-20	82.7289	pfam04145	Ctr	cl04417
Q#1544 - >Ca_18559.1|Ca_	superfamily	252408	32	132	1.39449e-20	82.7289	cl04417	Ctr superfamily	-
Q#1545 - >Ca_18579.1|Ca_	specific	253297	10	75	8.26415e-15	65.045	pfam05641	Agenet	cl02573
Q#1545 - >Ca_18579.1|Ca_	superfamily	275771	10	75	8.26415e-15	65.045	cl02573	TUDOR superfamily	-
Q#1545 - >Ca_18579.1|Ca_	superfamily	275771	81	138	0.00632809	32.2957	cl02573	TUDOR superfamily	-
Q#1546 - >Ca_18602.1|Ca_	superfamily	252005	261	318	2.98711e-14	71.9204	cl15987	GRAS superfamily	-
Q#1547 - >Ca_18608.1|Ca_	superfamily	256046	3	76	1.47224e-17	74.3532	cl11168	PMD superfamily	-
Q#1552 - >Ca_18626.1|Ca_	specific	252408	32	132	1.39449e-20	82.7289	pfam04145	Ctr	cl04417
Q#1552 - >Ca_18626.1|Ca_	superfamily	252408	32	132	1.39449e-20	82.7289	cl04417	Ctr superfamily	-
Q#1553 - >Ca_18641.1|Ca_	superfamily	249757	2	235	1.07923e-61	206.522	cl09510	Lipoxygenase superfamily	-
Q#1555 - >Ca_18665.1|Ca_	specific	250692	95	125	3.65643e-05	38.9741	pfam01535	PPR	cl03252
Q#1555 - >Ca_18665.1|Ca_	superfamily	275791	95	125	3.65643e-05	38.9741	cl03252	PPR superfamily	-
Q#1555 - >Ca_18665.1|Ca_	multi-dom	257477	61	106	3.99001e-05	38.923	pfam13041	PPR_2	-
Q#1556 - >Ca_18670.1|Ca_	superfamily	256046	1	152	1.59899e-08	52.782	cl11168	PMD superfamily	-
Q#1557 - >Ca_18675.1|Ca_	superfamily	253952	13	239	1.05695e-24	99.7438	cl06089	RNA_pol_I_A49 superfamily	-
Q#1560 - >Ca_18734.1|Ca_	superfamily	271753	85	111	5.85807e-06	40.623	cl14643	SRPBCC superfamily	-
Q#1561 - >Ca_18736.1|Ca_	specific	238096	21	65	6.18604e-08	44.8738	cd00167	SANT	cl21498
Q#1561 - >Ca_18736.1|Ca_	superfamily	271918	21	65	6.18604e-08	44.8738	cl21498	SANT superfamily	-
Q#1567 - >Ca_18818.1|Ca_	superfamily	256046	38	167	5.78085e-22	89.7612	cl11168	PMD superfamily	-
Q#1568 - >Ca_18841.1|Ca_	superfamily	271593	115	348	1.40876e-25	103.569	cl02567	WD40 superfamily	-
Q#1568 - >Ca_18841.1|Ca_	multi-dom	225201	73	353	3.65163e-21	93.2295	COG2319	COG2319	-
Q#1570 - >Ca_18849.1|Ca_	superfamily	251719	53	291	1.29765e-42	147.557	cl03884	DUF241 superfamily	-
Q#1571 - >Ca_18851.1|Ca_	superfamily	251719	53	292	1.6174e-38	136.772	cl03884	DUF241 superfamily	-
Q#1572 - >Ca_18866.1|Ca_	superfamily	276298	24	54	0.000518503	36.0983	cl21454	SDR superfamily	-
Q#1575 - >Ca_18918.1|Ca_	multi-dom	215061	22	119	0.00549017	34.0533	PLN00113	PLN00113	-
Q#1578 - >Ca_18944.1|Ca_	superfamily	256046	86	179	5.24884e-08	50.0856	cl11168	PMD superfamily	-
Q#1579 - >Ca_18948.1|Ca_	superfamily	261174	8	242	3.48988e-118	347.021	cl02098	14-3-3 superfamily	-
Q#1580 - >Ca_18950.1|Ca_	specific	133136	451	490	6.05726e-06	44.6348	cd00303	retropepsin_like	cl11403
Q#1580 - >Ca_18950.1|Ca_	superfamily	275927	451	490	6.05726e-06	44.6348	cl11403	pepsin_retropepsin_like superfamily	-
Q#1580 - >Ca_18950.1|Ca_	superfamily	252884	19	111	0.000191595	42.347	cl15563	Herpes_DNAp_acc superfamily	-

Query	Types	PSSM-ID	From	To	E-Value	Bitscore	Accession	Short name	Superfamily
Q#1581 - >Ca_18951.1|Ca_	specific	260004	92	228	1.82972e-69	212.328	cd09272	RNase_HI_RT_Ty1	cl14782
Q#1581 - >Ca_18951.1|Ca_	superfamily	271754	92	228	1.82972e-69	212.328	cl14782	RNase_H_like superfamily	-
Q#1582 - >Ca_18952.1|Ca_	superfamily	256046	146	415	1.40483e-29	117.881	cl11168	PMD superfamily	-
Q#1585 - >Ca_19003.1|Ca_	specific	238096	28	72	5.95192e-09	52.1926	cd00167	SANT	cl21498
Q#1585 - >Ca_19003.1|Ca_	superfamily	271918	28	72	5.95192e-09	52.1926	cl21498	SANT superfamily	-
Q#1585 - >Ca_19003.1|Ca_	specific	238096	81	119	1.80565e-07	47.9554	cd00167	SANT	cl21498
Q#1585 - >Ca_19003.1|Ca_	superfamily	271918	81	119	1.80565e-07	47.9554	cl21498	SANT superfamily	-
Q#1586 - >Ca_19035.1|Ca_	superfamily	276175	2	122	9.46335e-18	77.4616	cl17037	NBD_sugar-kinase_HSP70_actin superfamily	-
Q#1587 - >Ca_19044.1|Ca_	superfamily	252955	10	171	1.72489e-51	171.345	cl04895	PARG_cat superfamily	-
Q#1587 - >Ca_19044.1|Ca_	superfamily	271859	82	137	0.000470415	39.056	cl19419	DUF2263 superfamily	-
Q#1589 - >Ca_19066.1|Ca_	superfamily	276297	62	319	3.12299e-75	236.015	cl21453	PKc_like superfamily	-
Q#1589 - >Ca_19066.1|Ca_	multi-dom	214567	61	318	5.47527e-45	156.151	smart00220	S_TKc	-
Q#1590 - >Ca_19075.1|Ca_	superfamily	275907	58	137	2.7207e-21	82.7612	cl09936	PP-binding superfamily	-
Q#1591 - >Ca_19096.1|Ca_	superfamily	276335	18	370	8.16218e-78	249.975	cl21512	Glycos_transf_1 superfamily	-
Q#1592 - >Ca_19097.1|Ca_	superfamily	276335	18	377	3.49774e-75	243.426	cl21512	Glycos_transf_1 superfamily	-
Q#1593 - >Ca_19108.1|Ca_	superfamily	271592	130	213	1.1508e-16	75.8305	cl02553	Peptidase_C19 superfamily	-
Q#1593 - >Ca_19108.1|Ca_	superfamily	252977	8	61	9.44641e-05	41.7398	cl04913	DUF674 superfamily	-
Q#1594 - >Ca_19110.1|Ca_	superfamily	276299	73	208	3.38316e-36	128.136	cl21455	ABC_ATPase superfamily	-
Q#1596 - >Ca_19129.1|Ca_	superfamily	252133	73	142	1.71815e-08	50.4149	cl04237	Retrotrans_gag superfamily	-
Q#1597 - >Ca_19151.1|Ca_	multi-dom	114603	236	303	1.43647e-05	43.024	pfam05887	Trypan_PARP	-
Q#1599 - >Ca_19186.1|Ca_	superfamily	276299	153	189	6.57975e-21	87.7483	cl21455	ABC_ATPase superfamily	-
Q#1599 - >Ca_19186.1|Ca_	multi-dom	250205	6	177	4.29248e-26	104.245	pfam00888	Cullin	-
Q#1602 - >Ca_19222.1|Ca_	superfamily	276412	1	142	1.87032e-69	210.443	cl22458	RICIN superfamily	-
Q#1603 - >Ca_19253.1|Ca_	superfamily	115928	160	223	0.0013738	35.7276	cl06365	Occludin_ELL superfamily	-
Q#1604 - >Ca_19255.1|Ca_	superfamily	115928	98	194	0.000219822	37.6536	cl06365	Occludin_ELL superfamily	-
Q#1605 - >Ca_19259.1|Ca_	multi-dom	258434	1	47	7.03994e-12	58.9038	pfam14244	UBN2_3	-
Q#1606 - >Ca_19273.1|Ca_	specific	257351	33	56	9.08668e-07	41.5661	pfam12854	PPR_1	cl03252
Q#1606 - >Ca_19273.1|Ca_	superfamily	275791	33	56	9.08668e-07	41.5661	cl03252	PPR superfamily	-
Q#1606 - >Ca_19273.1|Ca_	multi-dom	257477	33	75	8.30432e-12	55.4866	pfam13041	PPR_2	-
Q#1607 - >Ca_19274.1|Ca_	superfamily	261198	26	79	1.51295e-07	44.9835	cl02154	YL1_C superfamily	-
Q#1608 - >Ca_19275.1|Ca_	superfamily	276020	3	265	3.06174e-68	221.997	cl12078	p450 superfamily	-
Q#1610 - >Ca_19289.1|Ca_	multi-dom	215563	3	109	3.38578e-19	82.2257	PLN03081	PLN03081	-
Q#1611 - >Ca_19297.1|Ca_	superfamily	260442	5	98	3.47024e-38	125.962	cl00466	ATP-synt_C superfamily	-
Q#1612 - >Ca_19343.1|Ca_	specific	238947	33	183	9.066e-40	140.874	cd01989	STK_N	cl00292
Q#1612 - >Ca_19343.1|Ca_	superfamily	275515	33	183	9.066e-40	140.874	cl00292	AANH_like superfamily	-
Q#1612 - >Ca_19343.1|Ca_	superfamily	276297	336	455	7.86308e-40	144.217	cl21453	PKc_like superfamily	-

Query	Types	PSSM-ID	From	To	E-Value	Bitscore	Accession	Short name	Superfamily
Q#1613 - >Ca_19345.1|Ca_	superfamily	206472	123	156	0.000190914	36.3221	cl16788	CSTF_C superfamily	-
Q#1615 - >Ca_19381.1|Ca_	superfamily	252133	55	129	1.88204e-07	45.7925	cl04237	Retrotrans_gag superfamily	-
Q#1616 - >Ca_19387.1|Ca_	multi-dom	226022	124	299	1.47276e-11	63.1535	COG3491	PcbC	-
Q#1619 - >Ca_19482.1|Ca_	superfamily	252133	101	176	0.000147588	39.6293	cl04237	Retrotrans_gag superfamily	-
Q#1620 - >Ca_19561.1|Ca_	superfamily	276299	131	203	3.66289e-23	92.6977	cl21455	ABC_ATPase superfamily	-
Q#1623 - >Ca_19570.1|Ca_	superfamily	275593	1	135	1.76524e-75	225.601	cl00467	Ntn_hydrolase superfamily	-
Q#1623 - >Ca_19570.1|Ca_	multi-dom	223711	1	131	3.76968e-20	82.7308	COG0638	PRE1	-
Q#1626 - >Ca_19588.1|Ca_	superfamily	251669	19	139	1.09771e-11	58.9652	cl03830	Transposase_24 superfamily	-
Q#1627 - >Ca_19589.1|Ca_	specific	191112	28	144	3.91322e-78	231.646	pfam04852	DUF640	cl04800
Q#1627 - >Ca_19589.1|Ca_	superfamily	191112	28	144	3.91322e-78	231.646	cl04800	DUF640 superfamily	-
Q#1628 - >Ca_19593.1|Ca_	superfamily	275460	125	196	1.5969e-36	132.507	cl00015	nt_trans superfamily	-
Q#1628 - >Ca_19593.1|Ca_	specific	214605	87	135	0.000841546	35.6524	smart00298	CHROMO	cl17459
Q#1628 - >Ca_19593.1|Ca_	superfamily	271815	87	135	0.000841546	35.6524	cl17459	CHROMO superfamily	-
Q#1630 - >Ca_19596.1|Ca_	superfamily	114645	218	320	0.0011341	39.3568	cl05479	MCLC superfamily	-
Q#1631 - >Ca_19597.1|Ca_	multi-dom	223021	68	223	8.97805e-05	43.0033	PHA03247	PHA03247	-
Q#1633 - >Ca_19614.1|Ca_	multi-dom	184900	28	128	0.00338405	38.7776	PRK14907	rplD	-
Q#1644 - >Ca_19675.1|Ca_	superfamily	271500	244	331	1.35226e-15	75.4072	cl00549	ABC_membrane superfamily	-
Q#1644 - >Ca_19675.1|Ca_	superfamily	265821	1	41	2.36414e-06	45.6413	cl15874	UBN2 superfamily	-
Q#1644 - >Ca_19675.1|Ca_	superfamily	131187	47	173	0.00298268	37.4997	cl11779	PHA_synthase_subunit_PhaR superfamily	-
Q#1644 - >Ca_19675.1|Ca_	multi-dom	224055	193	331	8.46355e-08	53.2014	COG1132	MdlB	-
Q#1645 - >Ca_19676.1|Ca_	superfamily	275927	125	167	1.27787e-09	53.1923	cl11403	pepsin_retropepsin_like superfamily	-
Q#1646 - >Ca_19683.1|Ca_	superfamily	275510	78	158	1.89287e-39	136.084	cl00279	APP_MetAP superfamily	-
Q#1647 - >Ca_19684.1|Ca_	superfamily	275510	1	55	7.51209e-29	102.957	cl00279	APP_MetAP superfamily	-
Q#1648 - >Ca_19688.1|Ca_	superfamily	271753	3	151	6.38832e-39	130.832	cl14643	SRPBCC superfamily	-
Q#1650 - >Ca_19717.1|Ca_	multi-dom	223021	70	286	1.01865e-05	47.6257	PHA03247	PHA03247	-
Q#1652 - >Ca_19726.1|Ca_	specific	111399	31	75	5.38013e-05	37.4435	pfam02496	ABA_WDS	cl03622
Q#1652 - >Ca_19726.1|Ca_	superfamily	111399	31	75	5.38013e-05	37.4435	cl03622	ABA_WDS superfamily	-
Q#1656 - >Ca_19758.1|Ca_	specific	238825	390	511	4.73236e-59	195.122	cd01647	RT_LTR	cl02808
Q#1656 - >Ca_19758.1|Ca_	superfamily	261455	390	511	4.73236e-59	195.122	cl02808	RT_like superfamily	-
Q#1656 - >Ca_19758.1|Ca_	specific	252133	100	199	6.30171e-12	62.3561	pfam03732	Retrotrans_gag	cl04237
Q#1656 - >Ca_19758.1|Ca_	superfamily	252133	100	199	6.30171e-12	62.3561	cl04237	Retrotrans_gag superfamily	-
Q#1656 - >Ca_19758.1|Ca_	superfamily	275927	248	290	1.043e-08	53.1923	cl11403	pepsin_retropepsin_like superfamily	-
Q#1656 - >Ca_19758.1|Ca_	multi-dom	249567	406	515	1.18351e-18	83.9194	pfam00078	RVT_1	-
Q#1657 - >Ca_19768.1|Ca_	specific	250356	6	141	1.64284e-78	231.238	pfam01090	Ribosomal_S19e	cl00969
Q#1657 - >Ca_19768.1|Ca_	superfamily	260724	6	141	1.64284e-78	231.238	cl00969	Ribosomal_S19e superfamily	-
Q#1661 - >Ca_19776.1|Ca_	superfamily	177577	61	254	0.00793335	37.6293	cl19709	PHA03292 superfamily	-

Query	Types	PSSM-ID	From	To	E-Value	Bitscore	Accession	Short name	Superfamily
Q#1662 - >Ca_19781.1|Ca_	specific	260006	209	321	3.7442e-37	130.69	cd09274	RNase_HI_RT_Ty3	cl14782
Q#1662 - >Ca_19781.1|Ca_	superfamily	271754	209	321	3.7442e-37	130.69	cl14782	RNase_H_like superfamily	-
Q#1662 - >Ca_19781.1|Ca_	superfamily	252133	67	166	3.7944e-09	53.1113	cl04237	Retrotrans_gag superfamily	-
Q#1664 - >Ca_19796.1|Ca_	superfamily	276305	15	46	9.30607e-05	37.664	cl21462	bZIP superfamily	-
Q#1666 - >Ca_19809.1|Ca_	superfamily	262303	23	298	5.99325e-105	329.865	cl04813	EIN3 superfamily	-
Q#1667 - >Ca_19811.1|Ca_	superfamily	275753	3	216	1.44739e-144	408.924	cl01911	Pectinesterase superfamily	-
Q#1669 - >Ca_19820.1|Ca_	multi-dom	259534	578	685	9.69898e-05	44.7498	pfam15402	Spc7_N	-
Q#1669 - >Ca_19820.1|Ca_	multi-dom	237874	128	252	0.00588332	38.6031	PRK14971	PRK14971	-
Q#1671 - >Ca_19828.1|Ca_	specific	109342	9	52	7.51278e-12	57.3264	pfam00281	Ribosomal_L5	cl08254
Q#1671 - >Ca_19828.1|Ca_	superfamily	109342	9	52	7.51278e-12	57.3264	cl08254	Ribosomal_L5 superfamily	-
Q#1675 - >Ca_19855.1|Ca_	superfamily	271838	50	133	9.36145e-38	135.995	cl19114	RNAP_largest_subunit_N superfamily	-
Q#1677 - >Ca_19870.1|Ca_	specific	130620	36	92	2.56977e-23	91.3113	TIGR01557	unnamed_protein_product	cl21498
Q#1677 - >Ca_19870.1|Ca_	superfamily	271918	36	92	2.56977e-23	91.3113	cl21498	SANT superfamily	-
Q#1677 - >Ca_19870.1|Ca_	specific	206547	126	173	1.70854e-20	83.0631	pfam14379	Myb_CC_LHEQLE	cl16840
Q#1677 - >Ca_19870.1|Ca_	superfamily	206547	126	173	1.70854e-20	83.0631	cl16840	Myb_CC_LHEQLE superfamily	-
Q#1682 - >Ca_19898.1|Ca_	specific	238825	380	525	9.19648e-75	237.108	cd01647	RT_LTR	cl02808
Q#1682 - >Ca_19898.1|Ca_	superfamily	261455	380	525	9.19648e-75	237.108	cl02808	RT_like superfamily	-
Q#1682 - >Ca_19898.1|Ca_	specific	260004	1	101	1.06856e-47	163.793	cd09272	RNase_HI_RT_Ty1	cl14782
Q#1682 - >Ca_19898.1|Ca_	superfamily	271754	1	101	1.06856e-47	163.793	cl14782	RNase_H_like superfamily	-
Q#1682 - >Ca_19898.1|Ca_	specific	133136	230	317	9.70219e-18	78.9175	cd00303	retropepsin_like	cl11403
Q#1682 - >Ca_19898.1|Ca_	superfamily	275927	230	317	9.70219e-18	78.9175	cl11403	pepsin_retropepsin_like superfamily	-
Q#1682 - >Ca_19898.1|Ca_	specific	189387	174	189	0.000232356	38.9745	pfam00098	zf-CCHC	cl22700
Q#1682 - >Ca_19898.1|Ca_	superfamily	276654	174	189	0.000232356	38.9745	cl22700	zf-CCHC superfamily	-
Q#1682 - >Ca_19898.1|Ca_	specific	197667	154	169	0.00121328	37.0376	smart00343	ZnF_C2HC	cl22700
Q#1682 - >Ca_19898.1|Ca_	superfamily	276654	154	169	0.00121328	37.0376	cl22700	zf-CCHC superfamily	-
Q#1682 - >Ca_19898.1|Ca_	multi-dom	249567	392	525	6.14778e-30	115.891	pfam00078	RVT_1	-
Q#1683 - >Ca_19899.1|Ca_	specific	260006	1	88	4.18985e-44	149.18	cd09274	RNase_HI_RT_Ty3	cl14782
Q#1683 - >Ca_19899.1|Ca_	superfamily	271754	1	88	4.18985e-44	149.18	cl14782	RNase_H_like superfamily	-
Q#1683 - >Ca_19899.1|Ca_	specific	250040	247	327	1.2104e-09	54.6469	pfam00665	rve	cl21549
Q#1683 - >Ca_19899.1|Ca_	superfamily	276355	247	327	1.2104e-09	54.6469	cl21549	rve superfamily	-
Q#1689 - >Ca_19937.1|Ca_	specific	238825	96	172	5.65306e-30	111.918	cd01647	RT_LTR	cl02808
Q#1689 - >Ca_19937.1|Ca_	superfamily	261455	96	172	5.65306e-30	111.918	cl02808	RT_like superfamily	-
Q#1690 - >Ca_19939.1|Ca_	specific	133136	215	275	7.97242e-08	48.872	cd00303	retropepsin_like	cl11403
Q#1690 - >Ca_19939.1|Ca_	superfamily	275927	215	275	7.97242e-08	48.872	cl11403	pepsin_retropepsin_like superfamily	-
Q#1690 - >Ca_19939.1|Ca_	superfamily	252133	11	110	1.20988e-10	56.9633	cl04237	Retrotrans_gag superfamily	-
Q#1691 - >Ca_19940.1|Ca_	specific	133136	362	449	4.17687e-18	79.6879	cd00303	retropepsin_like	cl11403

Query	Types	PSSM-ID	From	To	E-Value	Bitscore	Accession	Short name	Superfamily
Q#1691 - >Ca_19940.1|Ca_	superfamily	275927	362	449	4.17687e-18	79.6879	cl11403	pepsin_retropepsin_like superfamily	-
Q#1691 - >Ca_19940.1|Ca_	specific	252133	100	199	5.47504e-13	65.0525	pfam03732	Retrotrans_gag	cl04237
Q#1691 - >Ca_19940.1|Ca_	superfamily	252133	100	199	5.47504e-13	65.0525	cl04237	Retrotrans_gag superfamily	-
Q#1691 - >Ca_19940.1|Ca_	specific	189387	305	322	0.00173046	36.2781	pfam00098	zf-CCHC	cl22700
Q#1691 - >Ca_19940.1|Ca_	superfamily	276654	305	322	0.00173046	36.2781	cl22700	zf-CCHC superfamily	-
Q#1692 - >Ca_19942.1|Ca_	multi-dom	259534	393	500	0.00132813	40.5126	pfam15402	Spc7_N	-
Q#1695 - >Ca_19961.1|Ca_	superfamily	276298	8	223	6.07135e-17	75.7951	cl21454	SDR superfamily	-
Q#1696 - >Ca_19965.1|Ca_	multi-dom	259534	10	103	1.57949e-06	47.061	pfam15402	Spc7_N	-
Q#1697 - >Ca_19966.1|Ca_	multi-dom	223021	44	297	0.000319606	41.8477	PHA03247	PHA03247	-
Q#1698 - >Ca_19978.1|Ca_	superfamily	252133	100	199	6.05372e-12	59.2745	cl04237	Retrotrans_gag superfamily	-
Q#1699 - >Ca_19979.1|Ca_	superfamily	256046	3	81	3.83385e-18	76.2792	cl11168	PMD superfamily	-
Q#1700 - >Ca_19988.1|Ca_	specific	189387	52	65	0.00471906	30.8853	pfam00098	zf-CCHC	cl22700
Q#1700 - >Ca_19988.1|Ca_	superfamily	276654	52	65	0.00471906	30.8853	cl22700	zf-CCHC superfamily	-
Q#1701 - >Ca_19998.1|Ca_	superfamily	275737	1	81	2.2485e-14	68.3908	cl01629	TPP_enzymes superfamily	-
Q#1701 - >Ca_19998.1|Ca_	superfamily	276301	125	279	0.000302107	39.9355	cl21457	TIM_phosphate_binding superfamily	-
Q#1703 - >Ca_20031.1|Ca_	superfamily	130628	141	198	1.3456e-21	84.5351	cl22771	unnamed_protein_product superfamily	-
Q#1703 - >Ca_20031.1|Ca_	specific	252791	41	97	1.03429e-20	82.0313	pfam04770	ZF-HD_dimer	cl04737
Q#1703 - >Ca_20031.1|Ca_	superfamily	262275	41	97	1.03429e-20	82.0313	cl04737	ZF-HD_dimer superfamily	-
Q#1704 - >Ca_20039.1|Ca_	superfamily	260253	11	121	1.8259e-71	220.996	cl00198	Phosphoglycerate_kinase superfamily	-
Q#1705 - >Ca_20044.1|Ca_	specific	260004	1	115	3.75423e-57	176.505	cd09272	RNase_HI_RT_Ty1	cl14782
Q#1705 - >Ca_20044.1|Ca_	superfamily	271754	1	115	3.75423e-57	176.505	cl14782	RNase_H_like superfamily	-
Q#1706 - >Ca_20046.1|Ca_	specific	258415	1	77	4.37703e-17	75.6868	pfam14223	UBN2	cl15874
Q#1706 - >Ca_20046.1|Ca_	superfamily	265821	1	77	4.37703e-17	75.6868	cl15874	UBN2 superfamily	-
Q#1708 - >Ca_20049.1|Ca_	specific	252133	158	255	1.62088e-15	71.2157	pfam03732	Retrotrans_gag	cl04237
Q#1708 - >Ca_20049.1|Ca_	superfamily	252133	158	255	1.62088e-15	71.2157	cl04237	Retrotrans_gag superfamily	-
Q#1708 - >Ca_20049.1|Ca_	specific	189387	336	352	7.38647e-05	39.7449	pfam00098	zf-CCHC	cl22700
Q#1708 - >Ca_20049.1|Ca_	superfamily	276654	336	352	7.38647e-05	39.7449	cl22700	zf-CCHC superfamily	-
Q#1711 - >Ca_20060.1|Ca_	specific	211392	2	150	3.21499e-38	129.177	cd11380	Ribosomal_S8e_like	cl00880
Q#1711 - >Ca_20060.1|Ca_	superfamily	260676	2	150	3.21499e-38	129.177	cl00880	Ribosomal_S8e_like superfamily	-
Q#1712 - >Ca_20066.1|Ca_	specific	250692	101	128	0.00014139	37.8185	pfam01535	PPR	cl03252
Q#1712 - >Ca_20066.1|Ca_	superfamily	275791	101	128	0.00014139	37.8185	cl03252	PPR superfamily	-
Q#1712 - >Ca_20066.1|Ca_	specific	250692	202	232	0.000384998	36.6629	pfam01535	PPR	cl03252
Q#1712 - >Ca_20066.1|Ca_	superfamily	275791	202	232	0.000384998	36.6629	cl03252	PPR superfamily	-
Q#1712 - >Ca_20066.1|Ca_	specific	250692	41	68	0.0012566	35.1221	pfam01535	PPR	cl03252
Q#1712 - >Ca_20066.1|Ca_	superfamily	275791	41	68	0.0012566	35.1221	cl03252	PPR superfamily	-
Q#1712 - >Ca_20066.1|Ca_	specific	250692	7	37	0.00803278	32.8109	pfam01535	PPR	cl03252

Query	Types	PSSM-ID	From	To	E-Value	Bitscore	Accession	Short name	Superfamily
Q#1712 - >Ca_20066.1|Ca_	superfamily	275791	7	37	0.00803278	32.8109	cl03252	PPR superfamily	-
Q#1712 - >Ca_20066.1|Ca_	multi-dom	257477	35	79	1.61366e-06	43.5454	pfam13041	PPR_2	-
Q#1712 - >Ca_20066.1|Ca_	multi-dom	257477	7	49	2.46214e-05	40.0786	pfam13041	PPR_2	-
Q#1712 - >Ca_20066.1|Ca_	multi-dom	257477	66	110	0.00026578	37.3822	pfam13041	PPR_2	-
Q#1712 - >Ca_20066.1|Ca_	multi-dom	257477	169	211	0.00121937	35.4562	pfam13041	PPR_2	-
Q#1715 - >Ca_20078.1|Ca_	multi-dom	215061	7	155	1.32988e-18	82.5884	PLN00113	PLN00113	-
Q#1716 - >Ca_20081.1|Ca_	superfamily	256046	149	229	5.94152e-19	87.0648	cl11168	PMD superfamily	-
Q#1717 - >Ca_20087.1|Ca_	multi-dom	240271	2	113	0.00563259	38.1037	PTZ00108	PTZ00108	-
Q#1721 - >Ca_20114.1|Ca_	superfamily	252133	55	131	3.38399e-11	57.7337	cl04237	Retrotrans_gag superfamily	-
Q#1723 - >Ca_20146.1|Ca_	superfamily	252133	273	341	1.44088e-08	51.5705	cl04237	Retrotrans_gag superfamily	-
Q#1726 - >Ca_20162.1|Ca_	multi-dom	223021	68	395	6.01986e-05	44.5441	PHA03247	PHA03247	-
Q#1728 - >Ca_20169.1|Ca_	specific	251669	133	254	5.00326e-23	92.0924	pfam03004	Transposase_24	cl03830
Q#1728 - >Ca_20169.1|Ca_	superfamily	251669	133	254	5.00326e-23	92.0924	cl03830	Transposase_24 superfamily	-
Q#1729 - >Ca_20171.1|Ca_	specific	189387	170	186	0.00029605	37.0485	pfam00098	zf-CCHC	cl22700
Q#1729 - >Ca_20171.1|Ca_	superfamily	276654	170	186	0.00029605	37.0485	cl22700	zf-CCHC superfamily	-
Q#1729 - >Ca_20171.1|Ca_	superfamily	251582	6	75	0.00513424	34.1779	cl03753	MurB_C superfamily	-
Q#1731 - >Ca_20184.1|Ca_	superfamily	276263	122	317	9.91522e-17	77.3996	cl19514	FBA_1 superfamily	-
Q#1731 - >Ca_20184.1|Ca_	specific	250027	18	58	5.84284e-06	43.3474	pfam00646	F-box	cl02535
Q#1731 - >Ca_20184.1|Ca_	superfamily	261329	18	58	5.84284e-06	43.3474	cl02535	F-box superfamily	-
Q#1732 - >Ca_20188.1|Ca_	multi-dom	236669	38	83	0.00461602	35.0607	PRK10263	PRK10263	-
Q#1733 - >Ca_20191.1|Ca_	superfamily	251754	32	219	1.80258e-53	178.217	cl03911	DUF247 superfamily	-
Q#1737 - >Ca_20223.1|Ca_	multi-dom	185628	3	91	0.00432085	38.9026	PTZ00449	PTZ00449	-
Q#1738 - >Ca_20226.1|Ca_	superfamily	256046	18	230	5.74714e-32	120.192	cl11168	PMD superfamily	-
Q#1739 - >Ca_20230.1|Ca_	specific	258213	19	47	6.53855e-05	36.2498	pfam13961	DUF4219	cl22382
Q#1739 - >Ca_20230.1|Ca_	superfamily	258213	19	47	6.53855e-05	36.2498	cl22382	DUF4219 superfamily	-
Q#1740 - >Ca_20233.1|Ca_	superfamily	261492	44	108	3.92304e-35	122.977	cl02879	Chloroa_b-bind superfamily	-
Q#1741 - >Ca_20238.1|Ca_	superfamily	276679	184	259	0.000553693	36.7387	cl22725	FBD superfamily	-
Q#1743 - >Ca_20263.1|Ca_	specific	238125	37	169	1.56074e-12	61.6306	cd00204	ANK	cl02529
Q#1743 - >Ca_20263.1|Ca_	superfamily	275769	37	169	1.56074e-12	61.6306	cl02529	ANK superfamily	-
Q#1743 - >Ca_20263.1|Ca_	superfamily	275769	144	205	0.00289069	35.4371	cl02529	ANK superfamily	-
Q#1743 - >Ca_20263.1|Ca_	multi-dom	257303	76	181	1.52102e-06	44.1691	pfam12796	Ank_2	-
Q#1744 - >Ca_20265.1|Ca_	specific	252681	1	63	2.34891e-23	89.2752	pfam04576	Zein-binding	cl04604
Q#1744 - >Ca_20265.1|Ca_	superfamily	252681	1	63	2.34891e-23	89.2752	cl04604	Zein-binding superfamily	-
Q#1745 - >Ca_20276.1|Ca_	superfamily	271547	1	96	6.79516e-35	122.487	cl01132	FA_hydroxylase superfamily	-
Q#1746 - >Ca_20283.1|Ca_	superfamily	276020	2	121	1.08421e-22	92.9637	cl12078	p450 superfamily	-
Q#1747 - >Ca_20289.1|Ca_	superfamily	242911	29	152	8.34546e-72	219.792	cl02160	Rcd1 superfamily	-

Query	Types	PSSM-ID	From	To	E-Value	Bitscore	Accession	Short name	Superfamily
Q#1750 - >Ca_20325.1|Ca_	specific	260004	63	128	3.50546e-21	90.9905	cd09272	RNase_HI_RT_Ty1	cl14782
Q#1750 - >Ca_20325.1|Ca_	superfamily	271754	63	128	3.50546e-21	90.9905	cl14782	RNase_H_like superfamily	-
Q#1751 - >Ca_20336.1|Ca_	superfamily	256046	6	117	1.43639e-09	56.634	cl11168	PMD superfamily	-
Q#1752 - >Ca_20337.1|Ca_	specific	238117	3	142	7.82276e-72	214.374	cd00195	UBCc	cl00154
Q#1752 - >Ca_20337.1|Ca_	superfamily	275475	3	142	7.82276e-72	214.374	cl00154	UBCc superfamily	-
Q#1752 - >Ca_20337.1|Ca_	multi-dom	227410	1	147	7.84604e-76	225.22	COG5078	COG5078	-
Q#1754 - >Ca_20345.1|Ca_	multi-dom	223021	200	350	3.45511e-06	48.3961	PHA03247	PHA03247	-
Q#1754 - >Ca_20345.1|Ca_	multi-dom	172376	304	398	0.00761924	37.1892	PRK13855	PRK13855	-
Q#1755 - >Ca_20349.1|Ca_	superfamily	256046	117	240	1.3006e-25	102.088	cl11168	PMD superfamily	-
Q#1756 - >Ca_20353.1|Ca_	superfamily	276020	4	179	3.79946e-39	141.872	cl12078	p450 superfamily	-
Q#1757 - >Ca_20374.1|Ca_	superfamily	275926	185	276	0.000165259	40.8902	cl11399	HP superfamily	-
Q#1758 - >Ca_20384.1|Ca_	specific	250513	6	184	3.56059e-93	272.854	pfam01294	Ribosomal_L13e	cl01799
Q#1758 - >Ca_20384.1|Ca_	superfamily	261075	6	184	3.56059e-93	272.854	cl01799	Ribosomal_L13e superfamily	-
Q#1760 - >Ca_20424.1|Ca_	specific	237985	60	126	4.7969e-18	76.1563	cd00018	AP2	cl00033
Q#1760 - >Ca_20424.1|Ca_	superfamily	260146	60	126	4.7969e-18	76.1563	cl00033	AP2 superfamily	-
Q#1760 - >Ca_20424.1|Ca_	superfamily	260146	160	223	3.87675e-12	60.3565	cl00033	AP2 superfamily	-
Q#1761 - >Ca_20431.1|Ca_	superfamily	276241	13	184	1.16819e-29	113.561	cl19217	Mem_trans superfamily	-
Q#1762 - >Ca_20444.1|Ca_	superfamily	275467	5	51	8.41303e-12	55.0816	cl00074	H2A superfamily	-
Q#1763 - >Ca_20468.1|Ca_	superfamily	252133	101	203	8.03505e-09	53.1113	cl04237	Retrotrans_gag superfamily	-
Q#1763 - >Ca_20468.1|Ca_	superfamily	252133	402	489	4.47353e-08	50.8001	cl04237	Retrotrans_gag superfamily	-
Q#1763 - >Ca_20468.1|Ca_	superfamily	275927	245	272	0.00316997	36.6287	cl11403	pepsin_retropepsin_like superfamily	-
Q#1765 - >Ca_20471.1|Ca_	superfamily	276235	5	215	5.28879e-72	227.187	cl19188	Pectate_lyase_3 superfamily	-
Q#1766 - >Ca_20479.1|Ca_	superfamily	276297	1	174	4.29527e-57	184.013	cl21453	PKc_like superfamily	-
Q#1767 - >Ca_20480.1|Ca_	superfamily	268118	4	194	3.4875e-07	48.6004	cl19765	mTERF superfamily	-
Q#1768 - >Ca_20484.1|Ca_	specific	197608	10	34	0.0092703	33.9494	smart00256	FBOX	cl02535
Q#1768 - >Ca_20484.1|Ca_	superfamily	261329	10	34	0.0092703	33.9494	cl02535	F-box superfamily	-
Q#1771 - >Ca_20490.1|Ca_	specific	133143	188	453	6.33986e-52	178.611	cd05476	pepsin_A_like_plant	cl11403
Q#1771 - >Ca_20490.1|Ca_	superfamily	275927	188	453	6.33986e-52	178.611	cl11403	pepsin_retropepsin_like superfamily	-
Q#1771 - >Ca_20490.1|Ca_	specific	258418	42	118	2.99205e-15	71.8878	pfam14226	DIOX_N	cl21672
Q#1771 - >Ca_20490.1|Ca_	superfamily	272092	42	118	2.99205e-15	71.8878	cl21672	DIOX_N superfamily	-
Q#1771 - >Ca_20490.1|Ca_	specific	251775	150	174	7.94333e-05	40.8559	pfam03171	2OG-FeII_Oxy	cl21496
Q#1771 - >Ca_20490.1|Ca_	superfamily	276328	150	174	7.94333e-05	40.8559	cl21496	2OG-FeII_Oxy superfamily	-
Q#1778 - >Ca_20532.1|Ca_	superfamily	263970	14	261	4.21822e-49	164.852	cl10447	GH18_chitinase-like superfamily	-
Q#1778 - >Ca_20532.1|Ca_	multi-dom	250070	38	199	3.6745e-08	52.1221	pfam00704	Glyco_hydro_18	-
Q#1779 - >Ca_20534.1|Ca_	multi-dom	223021	35	218	0.000225342	42.2329	PHA03247	PHA03247	-
Q#1782 - >Ca_20597.1|Ca_	superfamily	261228	1	91	7.88538e-37	130.738	cl02262	UPF0261 superfamily	-

Query	Types	PSSM-ID	From	To	E-Value	Bitscore	Accession	Short name	Superfamily
Q#1783 - >Ca_20602.1|Ca_	superfamily	256046	91	237	1.14846e-09	57.0192	cl11168	PMD superfamily	-
Q#1785 - >Ca_20629.1|Ca_	superfamily	275791	80	112	4.40916e-05	38.4845	cl03252	PPR superfamily	-
Q#1785 - >Ca_20629.1|Ca_	superfamily	275791	118	136	0.00262513	33.4769	cl03252	PPR superfamily	-
Q#1785 - >Ca_20629.1|Ca_	specific	273253	45	85	0.00424445	33.1975	TIGR00756	PPR	cl03252
Q#1785 - >Ca_20629.1|Ca_	superfamily	275791	45	85	0.00424445	33.1975	cl03252	PPR superfamily	-
Q#1785 - >Ca_20629.1|Ca_	multi-dom	257477	83	133	2.43476e-13	61.2646	pfam13041	PPR_2	-
Q#1785 - >Ca_20629.1|Ca_	multi-dom	257477	43	97	5.32778e-09	48.9382	pfam13041	PPR_2	-
Q#1786 - >Ca_20642.1|Ca_	superfamily	256046	49	160	1.64184e-07	50.4708	cl11168	PMD superfamily	-
Q#1788 - >Ca_20652.1|Ca_	specific	190559	5	105	1.97023e-43	145.413	pfam03195	DUF260	cl03929
Q#1788 - >Ca_20652.1|Ca_	superfamily	190559	5	105	1.97023e-43	145.413	cl03929	DUF260 superfamily	-
Q#1789 - >Ca_20653.1|Ca_	specific	190559	64	164	5.56688e-47	156.969	pfam03195	DUF260	cl03929
Q#1789 - >Ca_20653.1|Ca_	superfamily	190559	64	164	5.56688e-47	156.969	cl03929	DUF260 superfamily	-
Q#1789 - >Ca_20653.1|Ca_	superfamily	190037	142	224	0.00145525	38.4736	cl20137	Zein superfamily	-
Q#1791 - >Ca_20678.1|Ca_	superfamily	276020	1	253	2.4911e-151	435.446	cl12078	p450 superfamily	-
Q#1792 - >Ca_20683.1|Ca_	superfamily	261170	1	20	0.000160861	35.7968	cl02092	Clat_adaptor_s superfamily	-
Q#1793 - >Ca_20693.1|Ca_	superfamily	276020	1	253	2.4911e-151	435.446	cl12078	p450 superfamily	-
Q#1794 - >Ca_20698.1|Ca_	superfamily	261170	1	20	0.000160861	35.7968	cl02092	Clat_adaptor_s superfamily	-
Q#1795 - >Ca_20706.1|Ca_	multi-dom	224022	1	74	0.000217098	37.3215	COG1097	RRP4	-
Q#1796 - >Ca_20710.1|Ca_	specific	238239	36	162	9.2681e-68	205.788	cd00412	pyrophosphatase	cl00217
Q#1796 - >Ca_20710.1|Ca_	superfamily	260270	36	162	9.2681e-68	205.788	cl00217	pyrophosphatase superfamily	-
Q#1800 - >Ca_20751.1|Ca_	superfamily	252133	100	199	6.37781e-12	59.6597	cl04237	Retrotrans_gag superfamily	-
Q#1801 - >Ca_20771.1|Ca_	superfamily	271701	114	167	2.60273e-14	69.164	cl10459	Peptidases_S8_S53 superfamily	-
Q#1807 - >Ca_20851.1|Ca_	specific	271034	1	167	1.92157e-124	355.311	cd14132	STKc_CK2_alpha	cl21453
Q#1807 - >Ca_20851.1|Ca_	superfamily	276297	1	167	1.92157e-124	355.311	cl21453	PKc_like superfamily	-
Q#1808 - >Ca_20852.1|Ca_	superfamily	276297	29	74	5.71318e-22	86.4417	cl21453	PKc_like superfamily	-
Q#1809 - >Ca_20859.1|Ca_	superfamily	276194	2	190	9.84498e-105	303.839	cl17173	AdoMet_MTases superfamily	-
Q#1810 - >Ca_20865.1|Ca_	specific	238036	158	189	7.28659e-06	41.8159	cd00083	HLH	cl00081
Q#1810 - >Ca_20865.1|Ca_	superfamily	260178	158	189	7.28659e-06	41.8159	cl00081	HLH superfamily	-
Q#1816 - >Ca_20994.1|Ca_	superfamily	271494	8	126	6.07083e-51	163.162	cl00473	BI-1-like superfamily	-
Q#1817 - >Ca_20999.1|Ca_	multi-dom	254113	134	309	5.31849e-70	226.161	pfam07227	DUF1423	-
Q#1818 - >Ca_21000.1|Ca_	superfamily	276307	88	148	9.55408e-16	68.8616	cl21464	ABD superfamily	-
Q#1819 - >Ca_21053.1|Ca_	superfamily	271660	1	88	3.03267e-24	96.1025	cl09326	MATE_like superfamily	-
Q#1819 - >Ca_21053.1|Ca_	superfamily	271660	100	129	0.0066227	34.4706	cl09326	MATE_like superfamily	-
Q#1820 - >Ca_21063.1|Ca_	superfamily	257142	1	35	0.000129628	39.5415	cl13955	TPPII superfamily	-
Q#1823 - >Ca_21154.1|Ca_	superfamily	271593	173	279	1.87168e-15	73.138	cl02567	WD40 superfamily	-
Q#1823 - >Ca_21154.1|Ca_	multi-dom	225201	173	280	5.27207e-06	45.8499	COG2319	COG2319	-

Query	Types	PSSM-ID	From	To	E-Value	Bitscore	Accession	Short name	Superfamily
Q#1825 - >Ca_21200.1|Ca_	multi-dom	223619	38	66	0.00122675	33.9278	COG0545	FkpA	-
Q#1826 - >Ca_21202.1|Ca_	superfamily	276307	88	148	9.55408e-16	68.8616	cl21464	ABD superfamily	-
Q#1827 - >Ca_21203.1|Ca_	multi-dom	254113	134	309	5.31849e-70	226.161	pfam07227	DUF1423	-
Q#1828 - >Ca_21294.1|Ca_	specific	260004	21	148	3.91303e-61	188.061	cd09272	RNase_HI_RT_Ty1	cl14782
Q#1828 - >Ca_21294.1|Ca_	superfamily	271754	21	148	3.91303e-61	188.061	cl14782	RNase_H_like superfamily	-
Q#1831 - >Ca_21313.1|Ca_	specific	252791	29	80	3.5968e-23	85.1129	pfam04770	ZF-HD_dimer	cl04737
Q#1831 - >Ca_21313.1|Ca_	superfamily	262275	29	80	3.5968e-23	85.1129	cl04737	ZF-HD_dimer superfamily	-
Q#1832 - >Ca_21324.1|Ca_	specific	250548	96	137	5.22811e-05	39.1851	pfam01344	Kelch_1	cl02701
Q#1832 - >Ca_21324.1|Ca_	superfamily	261412	96	137	5.22811e-05	39.1851	cl02701	Kelch_3 superfamily	-
Q#1832 - >Ca_21324.1|Ca_	specific	128874	60	102	0.00014203	38.3083	smart00612	Kelch	cl02701
Q#1832 - >Ca_21324.1|Ca_	superfamily	261412	60	102	0.00014203	38.3083	cl02701	Kelch_3 superfamily	-
Q#1833 - >Ca_21400.1|Ca_	specific	238125	73	210	3.91555e-17	74.3422	cd00204	ANK	cl02529
Q#1833 - >Ca_21400.1|Ca_	superfamily	275769	73	210	3.91555e-17	74.3422	cl02529	ANK superfamily	-
Q#1833 - >Ca_21400.1|Ca_	multi-dom	257947	110	164	4.00896e-06	42.6228	pfam13637	Ank_4	-
Q#1833 - >Ca_21400.1|Ca_	multi-dom	257947	146	210	0.000302822	37.23	pfam13637	Ank_4	-
Q#1833 - >Ca_21400.1|Ca_	multi-dom	257947	76	130	0.00191034	34.9188	pfam13637	Ank_4	-
Q#1833 - >Ca_21400.1|Ca_	multi-dom	257303	13	92	0.00235364	34.9243	pfam12796	Ank_2	-
Q#1834 - >Ca_21430.1|Ca_	superfamily	275906	5	104	7.70259e-21	81.9854	cl09930	RPA_2b-aaRSs_OBF_like superfamily	-
Q#1836 - >Ca_21462.1|Ca_	specific	250507	82	111	3.84585e-10	51.7527	pfam01287	eIF-5a	cl09927
Q#1836 - >Ca_21462.1|Ca_	superfamily	275904	82	111	3.84585e-10	51.7527	cl09927	S1_like superfamily	-
Q#1838 - >Ca_21536.1|Ca_	specific	238008	139	202	1.39209e-12	59.8689	cd00051	EFh	cl08302
Q#1838 - >Ca_21536.1|Ca_	superfamily	275859	139	202	1.39209e-12	59.8689	cl08302	EFh superfamily	-
Q#1838 - >Ca_21536.1|Ca_	multi-dom	257819	145	202	3.26702e-07	45.0673	pfam13499	EF-hand_7	-
Q#1840 - >Ca_21586.1|Ca_	specific	257687	269	426	2.81368e-38	137.378	pfam13359	DDE_Tnp_4	cl21562
Q#1840 - >Ca_21586.1|Ca_	superfamily	271982	269	426	2.81368e-38	137.378	cl21562	DDE_Tnp_1_6 superfamily	-
Q#1841 - >Ca_21639.1|Ca_	superfamily	261267	29	57	0.00092405	36.0936	cl02381	Tim17 superfamily	-
Q#1842 - >Ca_21693.1|Ca_	specific	149534	60	85	0.00230147	34.9859	pfam08513	LisH	cl22501
Q#1842 - >Ca_21693.1|Ca_	superfamily	276455	60	85	0.00230147	34.9859	cl22501	LisH superfamily	-
Q#1843 - >Ca_21694.1|Ca_	specific	238121	45	325	4.38302e-54	180.609	cd00200	WD40	cl02567
Q#1843 - >Ca_21694.1|Ca_	superfamily	271593	45	325	4.38302e-54	180.609	cl02567	WD40 superfamily	-
Q#1843 - >Ca_21694.1|Ca_	multi-dom	225201	45	325	2.04901e-27	110.178	COG2319	COG2319	-
Q#1844 - >Ca_21701.1|Ca_	superfamily	275516	146	204	2.03838e-17	76.0822	cl00296	Peptidase_C1 superfamily	-
Q#1845 - >Ca_21745.1|Ca_	specific	257126	9	56	5.63482e-20	75.6825	pfam12554	MOZART1	cl13929
Q#1845 - >Ca_21745.1|Ca_	superfamily	257126	9	56	5.63482e-20	75.6825	cl13929	MOZART1 superfamily	-
Q#1846 - >Ca_21796.1|Ca_	specific	238002	10	67	1.54941e-09	49.78	cd00042	CY	cl09238
Q#1846 - >Ca_21796.1|Ca_	superfamily	275873	10	67	1.54941e-09	49.78	cl09238	CY superfamily	-

Query	Types	PSSM-ID	From	To	E-Value	Bitscore	Accession	Short name	Superfamily
Q#1847 - >Ca_21810.1|Ca_	superfamily	275925	75	271	2.34538e-54	179.157	cl11394	Glyco_tranf_GTA_type superfamily	-
Q#1848 - >Ca_21849.1|Ca_	superfamily	275467	25	97	9.8567e-12	57.0076	cl00074	H2A superfamily	-
Q#1853 - >Ca_21921.1|Ca_	specific	275440	28	169	3.52258e-48	155.215	cd15796	CIF_like	cl04375
Q#1853 - >Ca_21921.1|Ca_	superfamily	275808	28	169	3.52258e-48	155.215	cl04375	PMEI_like superfamily	-
Q#1854 - >Ca_21925.1|Ca_	specific	275440	26	172	1.81771e-44	145.971	cd15796	CIF_like	cl04375
Q#1854 - >Ca_21925.1|Ca_	superfamily	275808	26	172	1.81771e-44	145.971	cl04375	PMEI_like superfamily	-
Q#1855 - >Ca_21930.1|Ca_	multi-dom	223489	23	165	2.65455e-14	67.0204	COG0412	COG0412	-
Q#1857 - >Ca_21978.1|Ca_	specific	275440	28	169	3.52258e-48	155.215	cd15796	CIF_like	cl04375
Q#1857 - >Ca_21978.1|Ca_	superfamily	275808	28	169	3.52258e-48	155.215	cl04375	PMEI_like superfamily	-
Q#1858 - >Ca_21979.1|Ca_	superfamily	276335	146	318	8.91226e-35	131.905	cl21512	Glycos_transf_1 superfamily	-
Q#1858 - >Ca_21979.1|Ca_	superfamily	276335	50	133	5.41304e-17	80.2886	cl21512	Glycos_transf_1 superfamily	-
Q#1863 - >Ca_22025.1|Ca_	superfamily	275467	31	120	1.48925e-10	54.3112	cl00074	H2A superfamily	-
Q#1865 - >Ca_22032.1|Ca_	specific	240792	7	75	5.96314e-37	122.797	cd12346	RRM3_NGR1_NAM8_like	cl17169
Q#1865 - >Ca_22032.1|Ca_	superfamily	276192	7	75	5.96314e-37	122.797	cl17169	RRM_SF superfamily	-
Q#1865 - >Ca_22032.1|Ca_	multi-dom	214636	6	72	5.65677e-22	84.1823	smart00360	RRM	-
Q#1866 - >Ca_22033.1|Ca_	specific	240791	157	236	3.07553e-46	150.892	cd12345	RRM2_SECp43_like	cl17169
Q#1866 - >Ca_22033.1|Ca_	superfamily	276192	157	236	3.07553e-46	150.892	cl17169	RRM_SF superfamily	-
Q#1866 - >Ca_22033.1|Ca_	specific	240790	66	146	5.41106e-46	150.491	cd12344	RRM1_SECp43_like	cl17169
Q#1866 - >Ca_22033.1|Ca_	superfamily	276192	66	146	5.41106e-46	150.491	cl17169	RRM_SF superfamily	-
Q#1866 - >Ca_22033.1|Ca_	multi-dom	214636	159	232	7.7449e-18	75.3227	smart00360	RRM	-
Q#1866 - >Ca_22033.1|Ca_	multi-dom	214636	66	131	4.70897e-16	70.3151	smart00360	RRM	-
Q#1867 - >Ca_22035.1|Ca_	superfamily	191530	22	140	5.74674e-47	154.899	cl05782	DUF1084 superfamily	-
Q#1868 - >Ca_22066.1|Ca_	specific	240792	7	75	5.96314e-37	122.797	cd12346	RRM3_NGR1_NAM8_like	cl17169
Q#1868 - >Ca_22066.1|Ca_	superfamily	276192	7	75	5.96314e-37	122.797	cl17169	RRM_SF superfamily	-
Q#1868 - >Ca_22066.1|Ca_	multi-dom	214636	6	72	5.65677e-22	84.1823	smart00360	RRM	-
Q#1869 - >Ca_22067.1|Ca_	specific	240791	157	236	1.21594e-46	152.048	cd12345	RRM2_SECp43_like	cl17169
Q#1869 - >Ca_22067.1|Ca_	superfamily	276192	157	236	1.21594e-46	152.048	cl17169	RRM_SF superfamily	-
Q#1869 - >Ca_22067.1|Ca_	specific	240790	66	146	1.78802e-45	149.335	cd12344	RRM1_SECp43_like	cl17169
Q#1869 - >Ca_22067.1|Ca_	superfamily	276192	66	146	1.78802e-45	149.335	cl17169	RRM_SF superfamily	-
Q#1869 - >Ca_22067.1|Ca_	multi-dom	214636	159	232	8.0554e-18	75.3227	smart00360	RRM	-
Q#1869 - >Ca_22067.1|Ca_	multi-dom	214636	66	131	4.9375e-16	70.3151	smart00360	RRM	-
Q#1870 - >Ca_22069.1|Ca_	superfamily	191530	22	140	5.74674e-47	154.899	cl05782	DUF1084 superfamily	-
Q#1871 - >Ca_22132.1|Ca_	superfamily	271922	26	232	1.25832e-128	370.117	cl21502	CTP_transf_1 superfamily	-
Q#1872 - >Ca_22143.1|Ca_	multi-dom	184955	16	60	0.00707963	31.7684	PRK14993	PRK14993	-
Q#1873 - >Ca_22148.1|Ca_	superfamily	252407	13	132	5.98611e-28	103.074	cl04416	SCAMP superfamily	-
Q#1874 - >Ca_22153.1|Ca_	specific	116798	12	158	4.21224e-59	182.877	pfam08212	Lipocalin_2	cl21528

Query	Types	PSSM-ID	From	To	E-Value	Bitscore	Accession	Short name	Superfamily
Q#1874 - >Ca_22153.1|Ca_	superfamily	271948	12	158	4.21224e-59	182.877	cl21528	Lipocalin superfamily	-
Q#1876 - >Ca_22165.1|Ca_	specific	250805	80	208	2.40409e-22	89.9688	pfam01699	Na_Ca_ex	cl08331
Q#1876 - >Ca_22165.1|Ca_	superfamily	250805	80	208	2.40409e-22	89.9688	cl08331	Na_Ca_ex superfamily	-
Q#1877 - >Ca_22180.1|Ca_	specific	176858	5	150	5.96747e-45	146.18	cd07816	Bet_v1-like	cl14643
Q#1877 - >Ca_22180.1|Ca_	superfamily	271753	5	150	5.96747e-45	146.18	cl14643	SRPBCC superfamily	-
Q#1878 - >Ca_22181.1|Ca_	specific	238096	107	151	1.6441e-11	57.9706	cd00167	SANT	cl21498
Q#1878 - >Ca_22181.1|Ca_	superfamily	271918	107	151	1.6441e-11	57.9706	cl21498	SANT superfamily	-
Q#1878 - >Ca_22181.1|Ca_	specific	238096	13	59	6.60381e-10	53.7334	cd00167	SANT	cl21498
Q#1878 - >Ca_22181.1|Ca_	superfamily	271918	13	59	6.60381e-10	53.7334	cl21498	SANT superfamily	-
Q#1880 - >Ca_22216.1|Ca_	specific	249979	84	168	1.6392e-12	59.9678	pfam00583	Acetyltransf_1	cl17182
Q#1880 - >Ca_22216.1|Ca_	superfamily	276195	84	168	1.6392e-12	59.9678	cl17182	NAT_SF superfamily	-
Q#1880 - >Ca_22216.1|Ca_	multi-dom	257639	23	168	5.88864e-34	119.376	pfam13302	Acetyltransf_3	-
Q#1881 - >Ca_22271.1|Ca_	specific	114091	6	132	3.00885e-47	151.067	pfam05348	UMP1	cl05088
Q#1881 - >Ca_22271.1|Ca_	superfamily	114091	6	132	3.00885e-47	151.067	cl05088	UMP1 superfamily	-
Q#1882 - >Ca_22292.1|Ca_	superfamily	253337	19	109	1.79357e-17	74.8644	cl05327	Auxin_canalis superfamily	-
Q#1885 - >Ca_22316.1|Ca_	superfamily	275588	28	91	1.48238e-18	77.2846	cl00459	MIT_CorA-like superfamily	-
Q#1886 - >Ca_22325.1|Ca_	superfamily	276263	143	337	4.52548e-23	95.8891	cl19514	FBA_1 superfamily	-
Q#1886 - >Ca_22325.1|Ca_	specific	197608	48	84	2.34565e-08	50.1278	smart00256	FBOX	cl02535
Q#1886 - >Ca_22325.1|Ca_	superfamily	261329	48	84	2.34565e-08	50.1278	cl02535	F-box superfamily	-
Q#1887 - >Ca_22326.1|Ca_	superfamily	276263	144	338	3.0488e-24	99.3559	cl19514	FBA_1 superfamily	-
Q#1887 - >Ca_22326.1|Ca_	specific	250027	48	90	1.28045e-07	48.3549	pfam00646	F-box	cl02535
Q#1887 - >Ca_22326.1|Ca_	superfamily	261329	48	90	1.28045e-07	48.3549	cl02535	F-box superfamily	-
Q#1890 - >Ca_22377.1|Ca_	specific	250967	801	857	0.00122553	38.367	pfam01920	Prefoldin_2	cl09111
Q#1890 - >Ca_22377.1|Ca_	superfamily	271651	801	857	0.00122553	38.367	cl09111	Prefoldin superfamily	-
Q#1890 - >Ca_22377.1|Ca_	multi-dom	258126	284	340	0.000178923	40.5989	pfam13855	LRR_8	-
Q#1890 - >Ca_22377.1|Ca_	multi-dom	227223	58	259	1.00448e-05	47.6554	COG4886	COG4886	-
Q#1891 - >Ca_22378.1|Ca_	multi-dom	215633	7	310	5.02584e-31	129.995	PLN03210	PLN03210	-
Q#1891 - >Ca_22378.1|Ca_	multi-dom	224117	723	845	0.000924323	41.6236	COG1196	Smc	-
Q#1892 - >Ca_22382.1|Ca_	multi-dom	223021	346	433	0.000644875	41.4625	PHA03247	PHA03247	-
Q#1893 - >Ca_22387.1|Ca_	specific	211390	69	184	2.03767e-15	69.1514	cd11378	DUF296	cl00720
Q#1893 - >Ca_22387.1|Ca_	superfamily	260588	69	184	2.03767e-15	69.1514	cl00720	DUF296 superfamily	-
Q#1894 - >Ca_22395.1|Ca_	specific	253883	64	205	1.72032e-35	125.927	pfam06733	DEAD_2	cl21455
Q#1894 - >Ca_22395.1|Ca_	superfamily	276299	64	205	1.72032e-35	125.927	cl21455	ABC_ATPase superfamily	-
Q#1894 - >Ca_22395.1|Ca_	superfamily	252704	201	252	0.00163156	37.3432	cl14959	Pex19 superfamily	-
Q#1895 - >Ca_22401.1|Ca_	multi-dom	223021	170	320	5.25481e-07	51.0925	PHA03247	PHA03247	-
Q#1896 - >Ca_22409.1|Ca_	superfamily	276326	69	138	3.78389e-08	50.0353	cl21494	Esterase_lipase superfamily	-

Query	Types	PSSM-ID	From	To	E-Value	Bitscore	Accession	Short name	Superfamily
Q#1897 - >Ca_22415.1|Ca_	superfamily	271586	14	115	3.66717e-14	65.3925	cl02518	BTB superfamily	-
Q#1897 - >Ca_22415.1|Ca_	superfamily	250639	89	161	1.23952e-10	55.2504	cl03226	Skp1 superfamily	-
Q#1897 - >Ca_22415.1|Ca_	multi-dom	227528	15	153	8.32009e-10	54.5648	COG5201	SKP1	-
Q#1898 - >Ca_22416.1|Ca_	superfamily	271592	29	155	4.92089e-41	141.314	cl02553	Peptidase_C19 superfamily	-
Q#1900 - >Ca_22432.1|Ca_	superfamily	276020	1	192	1.71025e-121	354.046	cl12078	p450 superfamily	-
Q#1901 - >Ca_22437.1|Ca_	superfamily	271793	3	107	1.67803e-55	182.067	cl17068	AFD_class_I superfamily	-
Q#1903 - >Ca_22453.1|Ca_	specific	133136	293	360	1.78187e-07	48.4868	cd00303	retropepsin_like	cl11403
Q#1903 - >Ca_22453.1|Ca_	superfamily	275927	293	360	1.78187e-07	48.4868	cl11403	pepsin_retropepsin_like superfamily	-
Q#1903 - >Ca_22453.1|Ca_	superfamily	252133	118	151	0.000781946	37.3181	cl04237	Retrotrans_gag superfamily	-
Q#1905 - >Ca_22457.1|Ca_	superfamily	256046	15	136	1.7084e-34	123.659	cl11168	PMD superfamily	-
Q#1906 - >Ca_22470.1|Ca_	specific	250692	2	30	0.000374965	37.8185	pfam01535	PPR	cl03252
Q#1906 - >Ca_22470.1|Ca_	superfamily	275791	2	30	0.000374965	37.8185	cl03252	PPR superfamily	-
Q#1906 - >Ca_22470.1|Ca_	specific	273253	72	104	0.00390497	34.7383	TIGR00756	PPR	cl03252
Q#1906 - >Ca_22470.1|Ca_	superfamily	275791	72	104	0.00390497	34.7383	cl03252	PPR superfamily	-
Q#1906 - >Ca_22470.1|Ca_	multi-dom	257477	2	43	8.85514e-07	45.4714	pfam13041	PPR_2	-
Q#1908 - >Ca_22514.1|Ca_	multi-dom	224117	157	397	1.1508e-06	49.7128	COG1196	Smc	-
Q#1909 - >Ca_22569.1|Ca_	superfamily	271838	25	108	1.74447e-45	156.026	cl19114	RNAP_largest_subunit_N superfamily	-
Q#1911 - >Ca_22586.1|Ca_	specific	255296	123	240	4.46994e-11	57.2965	pfam09335	SNARE_assoc	cl00429
Q#1911 - >Ca_22586.1|Ca_	superfamily	260418	123	240	4.46994e-11	57.2965	cl00429	SNARE_assoc superfamily	-
Q#1912 - >Ca_22613.1|Ca_	specific	250380	1	143	1.3191e-79	234.099	pfam01125	G10	cl02138
Q#1912 - >Ca_22613.1|Ca_	superfamily	261194	1	143	1.3191e-79	234.099	cl02138	G10 superfamily	-
Q#1913 - >Ca_22631.1|Ca_	superfamily	260403	50	247	3.58995e-103	300.248	cl00395	FMT_core superfamily	-
Q#1914 - >Ca_22654.1|Ca_	superfamily	276297	5	200	1.65893e-126	362.935	cl21453	PKc_like superfamily	-
Q#1914 - >Ca_22654.1|Ca_	multi-dom	214567	6	201	3.4422e-61	195.826	smart00220	S_TKc	-
Q#1915 - >Ca_22663.1|Ca_	superfamily	255867	213	287	2.93913e-09	54.67	cl10951	Tmemb_185A superfamily	-
Q#1917 - >Ca_22715.1|Ca_	superfamily	276298	1	142	6.29593e-79	238.543	cl21454	SDR superfamily	-
Q#1919 - >Ca_22746.1|Ca_	specific	145700	6	141	2.40234e-69	208.177	pfam02681	DUF212	cl00474
Q#1919 - >Ca_22746.1|Ca_	superfamily	275595	6	141	2.40234e-69	208.177	cl00474	PAP2_like superfamily	-
Q#1921 - >Ca_22757.1|Ca_	specific	239035	46	107	3.96978e-17	73.2167	cd02120	PA_subtilisin_like	cl10459
Q#1921 - >Ca_22757.1|Ca_	superfamily	271701	46	107	3.96978e-17	73.2167	cl10459	Peptidases_S8_S53 superfamily	-
Q#1921 - >Ca_22757.1|Ca_	superfamily	271701	98	156	2.28692e-06	44.9158	cl10459	Peptidases_S8_S53 superfamily	-
Q#1921 - >Ca_22757.1|Ca_	specific	253461	1	51	3.76512e-06	41.5183	pfam05922	Inhibitor_I9	cl05470
Q#1921 - >Ca_22757.1|Ca_	superfamily	253461	1	51	3.76512e-06	41.5183	cl05470	Inhibitor_I9 superfamily	-
Q#1922 - >Ca_22771.1|Ca_	superfamily	276335	67	197	4.70257e-42	148.232	cl21512	Glycos_transf_1 superfamily	-
Q#1922 - >Ca_22771.1|Ca_	superfamily	275912	9	43	0.00150248	37.3424	cl10013	Glycosyltransferase_GTB_type superfamily	-
Q#1923 - >Ca_22828.1|Ca_	specific	190559	8	108	1.37279e-51	166.984	pfam03195	DUF260	cl03929

Query	Types	PSSM-ID	From	To	E-Value	Bitscore	Accession	Short name	Superfamily
Q#1923 - >Ca_22828.1|Ca_	superfamily	190559	8	108	1.37279e-51	166.984	cl03929	DUF260 superfamily	-
Q#1924 - >Ca_22869.1|Ca_	specific	173833	20	286	4.36464e-89	268.265	cd00685	Trans_IPPS_HT	cl00210
Q#1924 - >Ca_22869.1|Ca_	superfamily	275485	20	286	4.36464e-89	268.265	cl00210	Isoprenoid_Biosyn_C1 superfamily	-
Q#1926 - >Ca_22926.1|Ca_	specific	175973	46	108	2.32115e-10	52.8395	cd00030	C2	cl14603
Q#1926 - >Ca_22926.1|Ca_	superfamily	271750	46	108	2.32115e-10	52.8395	cl14603	C2 superfamily	-
Q#1928 - >Ca_22933.1|Ca_	multi-dom	173561	43	79	1.26856e-07	46.3385	PTZ00368	PTZ00368	-
Q#1930 - >Ca_22947.1|Ca_	superfamily	271660	1	234	2.38479e-89	273.68	cl09326	MATE_like superfamily	-
Q#1931 - >Ca_22983.1|Ca_	specific	214842	207	264	8.33526e-06	42.1846	smart00831	Cation_ATPase_N	cl02930
Q#1931 - >Ca_22983.1|Ca_	superfamily	261517	207	264	8.33526e-06	42.1846	cl02930	Cation_ATPase_N superfamily	-
Q#1931 - >Ca_22983.1|Ca_	superfamily	118909	49	112	0.0027812	36.198	cl11053	YkuI_C superfamily	-
Q#1932 - >Ca_22990.1|Ca_	superfamily	275485	64	133	0.00716117	37.1745	cl00210	Isoprenoid_Biosyn_C1 superfamily	-
Q#1934 - >Ca_23019.1|Ca_	multi-dom	109787	30	85	0.000157051	39.4043	pfam00743	FMO-like	-
Q#1935 - >Ca_23029.1|Ca_	superfamily	254489	3	164	3.01788e-81	245.707	cl06775	Calmodulin_bind superfamily	-
Q#1937 - >Ca_23054.1|Ca_	superfamily	252804	8	102	2.12659e-13	67.8568	cl04754	LMBR1 superfamily	-
Q#1938 - >Ca_23062.1|Ca_	specific	250431	134	207	8.73405e-43	140.336	pfam01191	RNA_pol_Rpb5_C	cl00883
Q#1938 - >Ca_23062.1|Ca_	superfamily	260678	134	207	8.73405e-43	140.336	cl00883	RNA_pol_Rpb5_C superfamily	-
Q#1938 - >Ca_23062.1|Ca_	specific	252217	4	90	3.70647e-28	103.111	pfam03871	RNA_pol_Rpb5_N	cl04305
Q#1938 - >Ca_23062.1|Ca_	superfamily	252217	4	90	3.70647e-28	103.111	cl04305	RNA_pol_Rpb5_N superfamily	-
Q#1938 - >Ca_23062.1|Ca_	superfamily	275614	53	133	0.000768621	36.7422	cl00516	Mrr_cat superfamily	-
Q#1938 - >Ca_23062.1|Ca_	multi-dom	215582	1	207	3.74085e-102	296.425	PLN03111	PLN03111	-
Q#1939 - >Ca_23097.1|Ca_	superfamily	256046	2	246	4.9058e-28	112.103	cl11168	PMD superfamily	-
Q#1940 - >Ca_23157.1|Ca_	superfamily	206228	42	104	0.0054256	35.8225	cl16577	PcfK superfamily	-
Q#1941 - >Ca_23159.1|Ca_	multi-dom	223021	164	341	0.000471182	41.4625	PHA03247	PHA03247	-
Q#1946 - >Ca_23178.1|Ca_	specific	257351	180	212	8.90444e-12	58.1297	pfam12854	PPR_1	cl03252
Q#1946 - >Ca_23178.1|Ca_	superfamily	275791	180	212	8.90444e-12	58.1297	cl03252	PPR superfamily	-
Q#1946 - >Ca_23178.1|Ca_	specific	257351	110	142	2.36845e-07	46.1885	pfam12854	PPR_1	cl03252
Q#1946 - >Ca_23178.1|Ca_	superfamily	275791	110	142	2.36845e-07	46.1885	cl03252	PPR superfamily	-
Q#1946 - >Ca_23178.1|Ca_	specific	257351	215	247	1.34671e-06	43.8773	pfam12854	PPR_1	cl03252
Q#1946 - >Ca_23178.1|Ca_	superfamily	275791	215	247	1.34671e-06	43.8773	cl03252	PPR superfamily	-
Q#1946 - >Ca_23178.1|Ca_	specific	257351	36	66	3.90198e-05	40.0253	pfam12854	PPR_1	cl03252
Q#1946 - >Ca_23178.1|Ca_	superfamily	275791	36	66	3.90198e-05	40.0253	cl03252	PPR superfamily	-
Q#1946 - >Ca_23178.1|Ca_	superfamily	275791	79	107	0.00594231	33.4769	cl03252	PPR superfamily	-
Q#1946 - >Ca_23178.1|Ca_	specific	273253	152	186	0.00805079	33.1975	TIGR00756	PPR	cl03252
Q#1946 - >Ca_23178.1|Ca_	superfamily	275791	152	186	0.00805079	33.1975	cl03252	PPR superfamily	-
Q#1946 - >Ca_23178.1|Ca_	superfamily	275791	1	31	0.00855428	33.0917	cl03252	PPR superfamily	-
Q#1946 - >Ca_23178.1|Ca_	multi-dom	257477	184	233	1.14634e-19	79.7542	pfam13041	PPR_2	-

Query	Types	PSSM-ID	From	To	E-Value	Bitscore	Accession	Short name	Superfamily
Q#1946 - >Ca_23178.1|Ca_	multi-dom	257477	151	198	2.8071e-13	62.4202	pfam13041	PPR_2	-
Q#1946 - >Ca_23178.1|Ca_	multi-dom	257477	81	128	1.42996e-10	55.1014	pfam13041	PPR_2	-
Q#1946 - >Ca_23178.1|Ca_	multi-dom	257477	6	54	1.37056e-08	49.7086	pfam13041	PPR_2	-
Q#1951 - >Ca_23232.1|Ca_	multi-dom	185628	181	286	0.000683249	40.8286	PTZ00449	PTZ00449	-
Q#1954 - >Ca_23361.1|Ca_	specific	214704	5	105	1.72e-42	138.58	smart00512	Skp1	cl02518
Q#1954 - >Ca_23361.1|Ca_	superfamily	271586	5	105	1.72e-42	138.58	cl02518	BTB superfamily	-
Q#1954 - >Ca_23361.1|Ca_	specific	250639	78	155	1.93019e-40	132.676	pfam01466	Skp1	cl03226
Q#1954 - >Ca_23361.1|Ca_	superfamily	250639	78	155	1.93019e-40	132.676	cl03226	Skp1 superfamily	-
Q#1954 - >Ca_23361.1|Ca_	multi-dom	227528	4	153	1.23734e-44	145.857	COG5201	SKP1	-
Q#1955 - >Ca_23363.1|Ca_	specific	144426	2	193	4.22968e-107	308.327	pfam00827	Ribosomal_L15e	cl02977
Q#1955 - >Ca_23363.1|Ca_	superfamily	243258	2	193	4.22968e-107	308.327	cl02977	Ribosomal_L15e superfamily	-
Q#1957 - >Ca_23425.1|Ca_	specific	250776	19	112	1.18088e-24	94.0635	pfam01657	Stress-antifung	cl03296
Q#1957 - >Ca_23425.1|Ca_	superfamily	250776	19	112	1.18088e-24	94.0635	cl03296	Stress-antifung superfamily	-
Q#1957 - >Ca_23425.1|Ca_	specific	250776	108	180	4.98237e-11	56.3139	pfam01657	Stress-antifung	cl03296
Q#1957 - >Ca_23425.1|Ca_	superfamily	250776	108	180	4.98237e-11	56.3139	cl03296	Stress-antifung superfamily	-
Q#1958 - >Ca_23458.1|Ca_	superfamily	275904	28	133	1.98041e-24	90.7815	cl09927	S1_like superfamily	-
Q#1960 - >Ca_23481.1|Ca_	specific	251731	88	161	3.91689e-27	103.539	pfam03101	FAR1	cl03890
Q#1960 - >Ca_23481.1|Ca_	superfamily	251731	88	161	3.91689e-27	103.539	cl03890	FAR1 superfamily	-
Q#1960 - >Ca_23481.1|Ca_	specific	252595	241	263	3.53839e-05	41.2487	pfam04434	SWIM	cl19527
Q#1960 - >Ca_23481.1|Ca_	superfamily	276267	241	263	3.53839e-05	41.2487	cl19527	SWIM superfamily	-
Q#1961 - >Ca_23501.1|Ca_	multi-dom	258434	50	100	5.27222e-12	57.7482	pfam14244	UBN2_3	-
Q#1962 - >Ca_23505.1|Ca_	superfamily	271750	15	86	4.96611e-17	71.8783	cl14603	C2 superfamily	-
Q#1963 - >Ca_23541.1|Ca_	specific	215086	1	189	5.90791e-81	251.793	PLN00168	PLN00168	cl12078
Q#1963 - >Ca_23541.1|Ca_	superfamily	276020	1	189	5.90791e-81	251.793	cl12078	p450 superfamily	-
Q#1964 - >Ca_23560.1|Ca_	specific	250513	6	184	6.23031e-94	274.78	pfam01294	Ribosomal_L13e	cl01799
Q#1964 - >Ca_23560.1|Ca_	superfamily	261075	6	184	6.23031e-94	274.78	cl01799	Ribosomal_L13e superfamily	-
Q#1965 - >Ca_23610.1|Ca_	superfamily	276656	279	328	5.00189e-10	55.8497	cl22702	CaM_binding superfamily	-
Q#1966 - >Ca_23614.1|Ca_	multi-dom	274765	27	102	0.00712997	33.8474	TIGR03752	conj_TIGR03752	-
Q#1971 - >Ca_24201.1|sca	superfamily	275927	346	421	2.12636e-07	49.3403	cl11403	pepsin_retropepsin_like superfamily	-
Q#1973 - >Ca_24205.1|sca	multi-dom	258434	42	111	5.56821e-13	62.3706	pfam14244	UBN2_3	-
Q#1974 - >Ca_24206.1|sca	superfamily	252133	100	199	2.70841e-11	59.2745	cl04237	Retrotrans_gag superfamily	-
Q#1974 - >Ca_24206.1|sca	specific	189387	307	323	0.00125844	36.2781	pfam00098	zf-CCHC	cl22700
Q#1974 - >Ca_24206.1|sca	superfamily	276654	307	323	0.00125844	36.2781	cl22700	zf-CCHC superfamily	-
Q#1975 - >Ca_24208.1|sca	specific	173887	32	220	1.20039e-54	176.651	cd06899	lectin_legume_LecRK_Arcelin_ConA	cl14058
Q#1975 - >Ca_24208.1|sca	superfamily	265423	32	220	1.20039e-54	176.651	cl14058	lectin_L-type superfamily	-
Q#1976 - >Ca_24216.1|sca	superfamily	271736	2	52	0.000102402	37.4207	cl12018	Peptidase_M48 superfamily	-

Query	Types	PSSM-ID	From	To	E-Value	Bitscore	Accession	Short name	Superfamily
Q#1977 - >Ca_24218.1|sca	superfamily	271736	21	217	2.55597e-09	54.3695	cl12018	Peptidase_M48 superfamily	-
Q#1981 - >Ca_24235.1|sca	superfamily	252133	103	177	1.8114e-11	57.7337	cl04237	Retrotrans_gag superfamily	-
Q#1983 - >Ca_24261.1|sca	superfamily	256046	1	203	5.09401e-13	66.264	cl11168	PMD superfamily	-
Q#1985 - >Ca_24263.1|sca	specific	185489	1	142	5.89276e-89	262.65	PTZ00160	PTZ00160	cl12022
Q#1985 - >Ca_24263.1|sca	superfamily	276015	1	142	5.89276e-89	262.65	cl12022	Ribosomal_L18e superfamily	-
Q#1985 - >Ca_24263.1|sca	specific	259377	155	206	1.92197e-12	60.8142	pfam15243	ANAPC15	cl21102
Q#1985 - >Ca_24263.1|sca	superfamily	259377	155	206	1.92197e-12	60.8142	cl21102	ANAPC15 superfamily	-
Q#1986 - >Ca_24267.1|sca	superfamily	262404	3	138	6.72839e-18	78.5938	cl05005	TAF4 superfamily	-
Q#1987 - >Ca_24269.1|sca	specific	185489	1	147	2.10787e-83	244.161	PTZ00160	PTZ00160	cl12022
Q#1987 - >Ca_24269.1|sca	superfamily	276015	1	147	2.10787e-83	244.161	cl12022	Ribosomal_L18e superfamily	-
Q#1990 - >Ca_24307.1|sca	multi-dom	179712	51	91	0.0043621	33.6863	PRK04019	rplP0	-
Q#1992 - >Ca_24313.1|sca	superfamily	252133	112	159	1.38614e-07	46.9481	cl04237	Retrotrans_gag superfamily	-
Q#1994 - >Ca_24326.1|sca	superfamily	271848	19	147	1.18011e-25	100.134	cl19241	Condensation superfamily	-
Q#1996 - >Ca_24328.1|sca	superfamily	256046	157	384	1.69385e-24	102.858	cl11168	PMD superfamily	-
Q#1997 - >Ca_24329.1|sca	superfamily	271848	18	439	1.56685e-82	262.707	cl19241	Condensation superfamily	-
Q#1998 - >Ca_24330.1|sca	superfamily	256046	83	242	1.01655e-20	88.2204	cl11168	PMD superfamily	-
Q#2001 - >Ca_24367.1|sca	specific	214842	249	316	8.39042e-12	60.6742	smart00831	Cation_ATPase_N	cl02930
Q#2001 - >Ca_24367.1|sca	superfamily	261517	249	316	8.39042e-12	60.6742	cl02930	Cation_ATPase_N superfamily	-
Q#2001 - >Ca_24367.1|sca	superfamily	265821	1	44	0.000695974	37.9373	cl15874	UBN2 superfamily	-
Q#2002 - >Ca_24376.1|sca	specific	254692	37	73	2.73612e-09	52.7214	pfam08263	LRRNT_2	cl08472
Q#2002 - >Ca_24376.1|sca	superfamily	254692	37	73	2.73612e-09	52.7214	cl08472	LRRNT_2 superfamily	-
Q#2002 - >Ca_24376.1|sca	superfamily	276297	344	377	2.64623e-05	44.1382	cl21453	PKc_like superfamily	-
Q#2002 - >Ca_24376.1|sca	superfamily	276664	208	342	3.89581e-05	43.8834	cl22710	LRR_RI superfamily	-
Q#2002 - >Ca_24376.1|sca	multi-dom	258126	183	243	0.00855724	34.0505	pfam13855	LRR_8	-
Q#2003 - >Ca_24378.1|sca	superfamily	206130	254	360	5.53007e-45	156.98	cl16501	DUF4218 superfamily	-
Q#2003 - >Ca_24378.1|sca	specific	258215	1	78	7.06328e-20	85.0716	pfam13963	Transpos_assoc	cl16504
Q#2003 - >Ca_24378.1|sca	superfamily	258215	1	78	7.06328e-20	85.0716	cl16504	Transpos_assoc superfamily	-
Q#2003 - >Ca_24378.1|sca	superfamily	251669	539	622	2.90241e-13	67.4396	cl03830	Transposase_24 superfamily	-
Q#2006 - >Ca_24386.1|sca	specific	260004	458	501	1.54884e-15	73.6565	cd09272	RNase_HI_RT_Ty1	cl14782
Q#2006 - >Ca_24386.1|sca	superfamily	271754	458	501	1.54884e-15	73.6565	cl14782	RNase_H_like superfamily	-
Q#2006 - >Ca_24386.1|sca	superfamily	254387	260	404	2.80388e-79	250.066	cl06662	RVT_2 superfamily	-
Q#2007 - >Ca_24389.1|sca	specific	252133	56	154	2.25333e-12	64.2821	pfam03732	Retrotrans_gag	cl04237
Q#2007 - >Ca_24389.1|sca	superfamily	252133	56	154	2.25333e-12	64.2821	cl04237	Retrotrans_gag superfamily	-
Q#2007 - >Ca_24389.1|sca	superfamily	259520	575	627	0.00354503	38.6491	cl21225	FAM117 superfamily	-
Q#2009 - >Ca_24392.1|sca	specific	133136	228	313	2.68652e-15	71.2136	cd00303	retropepsin_like	cl11403
Q#2009 - >Ca_24392.1|sca	superfamily	275927	228	313	2.68652e-15	71.2136	cl11403	pepsin_retropepsin_like superfamily	-

Query	Types	PSSM-ID	From	To	E-Value	Bitscore	Accession	Short name	Superfamily
Q#2009 - >Ca_24392.1|sca	superfamily	261455	418	454	0.00516879	36.942	cl02808	RT_like superfamily	-
Q#2010 - >Ca_24395.1|sca	superfamily	275693	28	96	1.19143e-21	90.0299	cl01046	BglB superfamily	-
Q#2013 - >Ca_24400.1|sca	specific	258214	42	153	1.82822e-20	82.9251	pfam13962	PGG	cl16503
Q#2013 - >Ca_24400.1|sca	superfamily	258214	42	153	1.82822e-20	82.9251	cl16503	PGG superfamily	-
Q#2014 - >Ca_24409.1|sca	multi-dom	139494	188	261	2.20644e-05	45.117	PRK13335	PRK13335	-
Q#2018 - >Ca_24423.1|sca	superfamily	261492	142	161	5.89006e-08	49.4035	cl02879	Chloroa_b-bind superfamily	-
Q#2018 - >Ca_24423.1|sca	superfamily	192471	90	128	9.77254e-08	46.2202	cl10872	DUF2372 superfamily	-
Q#2024 - >Ca_24444.1|sca	superfamily	275586	59	309	9.6515e-48	167.09	cl00456	SLC5-6-like_sbd superfamily	-
Q#2025 - >Ca_24448.1|sca	multi-dom	106330	315	371	0.00070389	40.394	PRK13372	pcmA	-
Q#2026 - >Ca_24450.1|sca	specific	260004	63	176	3.2543e-51	173.808	cd09272	RNase_HI_RT_Ty1	cl14782
Q#2026 - >Ca_24450.1|sca	superfamily	271754	63	176	3.2543e-51	173.808	cl14782	RNase_H_like superfamily	-
Q#2027 - >Ca_24451.1|sca	superfamily	254387	87	214	1.4301e-75	230.421	cl06662	RVT_2 superfamily	-
Q#2033 - >Ca_24469.1|sca	superfamily	113458	14	136	3.83057e-29	106.089	cl04677	YABBY superfamily	-
Q#2034 - >Ca_24480.1|sca	specific	238825	201	326	1.75404e-51	171.624	cd01647	RT_LTR	cl02808
Q#2034 - >Ca_24480.1|sca	superfamily	261455	201	326	1.75404e-51	171.624	cl02808	RT_like superfamily	-
Q#2034 - >Ca_24480.1|sca	specific	189387	10	27	0.00350975	35.1225	pfam00098	zf-CCHC	cl22700
Q#2034 - >Ca_24480.1|sca	superfamily	276654	10	27	0.00350975	35.1225	cl22700	zf-CCHC superfamily	-
Q#2034 - >Ca_24480.1|sca	superfamily	275927	48	88	0.00498516	35.4731	cl11403	pepsin_retropepsin_like superfamily	-
Q#2034 - >Ca_24480.1|sca	multi-dom	249567	235	333	2.12173e-15	73.1338	pfam00078	RVT_1	-
Q#2036 - >Ca_24482.1|sca	superfamily	260676	104	246	5.046e-21	86.0342	cl00880	Ribosomal_S8e_like superfamily	-
Q#2036 - >Ca_24482.1|sca	superfamily	264552	2	35	2.98893e-08	49.8058	cl12113	HSF_DNA-bind superfamily	-
Q#2039 - >Ca_24485.1|sca	multi-dom	148271	197	249	0.00436535	37.2802	pfam06566	Chon_Sulph_att	-
Q#2040 - >Ca_24490.1|sca	multi-dom	173561	54	86	0.000286887	35.9381	PTZ00368	PTZ00368	-
Q#2043 - >Ca_24508.1|sca	multi-dom	165099	286	394	0.00431771	38.1953	PHA02732	PHA02732	-
Q#2046 - >Ca_24533.1|sca	multi-dom	258434	47	139	8.39674e-24	90.4902	pfam14244	UBN2_3	-
Q#2047 - >Ca_24535.1|sca	specific	133136	250	315	4.90609e-06	43.4792	cd00303	retropepsin_like	cl11403
Q#2047 - >Ca_24535.1|sca	superfamily	275927	250	315	4.90609e-06	43.4792	cl11403	pepsin_retropepsin_like superfamily	-
Q#2049 - >Ca_24539.1|sca	multi-dom	223066	8	158	0.00142382	38.8861	PHA03379	PHA03379	-
Q#2051 - >Ca_24552.1|sca	multi-dom	223021	144	302	0.00211368	39.1513	PHA03247	PHA03247	-
Q#2053 - >Ca_24575.1|sca	multi-dom	185628	198	388	0.000386821	41.599	PTZ00449	PTZ00449	-
Q#2054 - >Ca_24594.1|sca	superfamily	252133	101	198	3.39416e-11	58.1189	cl04237	Retrotrans_gag superfamily	-
Q#2057 - >Ca_24613.1|sca	superfamily	276241	76	157	2.74681e-10	56.4726	cl19217	Mem_trans superfamily	-
Q#2061 - >Ca_24624.1|sca	superfamily	252637	89	182	1.00424e-20	84.6496	cl04551	DUF573 superfamily	-
Q#2062 - >Ca_24626.1|sca	specific	197772	55	121	2.80044e-13	61.0541	smart00526	H15	cl00073
Q#2062 - >Ca_24626.1|sca	superfamily	260173	55	121	2.80044e-13	61.0541	cl00073	H15 superfamily	-
Q#2063 - >Ca_24629.1|sca	superfamily	260327	1	73	6.74033e-37	134.03	cl00286	Motor_domain superfamily	-

Query	Types	PSSM-ID	From	To	E-Value	Bitscore	Accession	Short name	Superfamily
Q#2065 - >Ca_24633.1|sca	superfamily	271773	53	80	2.86883e-08	46.8412	cl15354	CBS_pair superfamily	-
Q#2066 - >Ca_24634.1|sca	multi-dom	185628	181	278	0.00144386	39.673	PTZ00449	PTZ00449	-
Q#2070 - >Ca_24654.1|sca	superfamily	256635	66	151	0.00276962	36.4592	cl20423	DUF3326 superfamily	-
Q#2072 - >Ca_24665.1|sca	superfamily	190526	213	246	1.49873e-06	44.1306	cl07846	DBD_Tnp_Mut superfamily	-
Q#2073 - >Ca_24678.1|sca	specific	238825	646	744	8.14337e-43	153.135	cd01647	RT_LTR	cl02808
Q#2073 - >Ca_24678.1|sca	superfamily	261455	646	744	8.14337e-43	153.135	cl02808	RT_like superfamily	-
Q#2073 - >Ca_24678.1|sca	specific	133136	289	346	6.01759e-06	45.02	cd00303	retropepsin_like	cl11403
Q#2073 - >Ca_24678.1|sca	superfamily	275927	289	346	6.01759e-06	45.02	cl11403	pepsin_retropepsin_like superfamily	-
Q#2073 - >Ca_24678.1|sca	specific	252133	100	199	3.74869e-12	63.5117	pfam03732	Retrotrans_gag	cl04237
Q#2073 - >Ca_24678.1|sca	superfamily	252133	100	199	3.74869e-12	63.5117	cl04237	Retrotrans_gag superfamily	-
Q#2073 - >Ca_24678.1|sca	specific	189387	233	249	0.000227378	39.7449	pfam00098	zf-CCHC	cl22700
Q#2073 - >Ca_24678.1|sca	superfamily	276654	233	249	0.000227378	39.7449	cl22700	zf-CCHC superfamily	-
Q#2074 - >Ca_24683.1|sca	specific	258183	300	339	3.65975e-08	49.3091	pfam13920	zf-C3HC4_3	cl17238
Q#2074 - >Ca_24683.1|sca	superfamily	276201	300	339	3.65975e-08	49.3091	cl17238	RING superfamily	-
Q#2075 - >Ca_24685.1|sca	superfamily	252133	93	163	4.82645e-09	52.3409	cl04237	Retrotrans_gag superfamily	-
Q#2076 - >Ca_24686.1|sca	superfamily	256046	10	86	1.16924e-16	75.1236	cl11168	PMD superfamily	-
Q#2077 - >Ca_24687.1|sca	superfamily	276299	1	107	5.0169e-56	176.62	cl21455	ABC_ATPase superfamily	-
Q#2077 - >Ca_24687.1|sca	superfamily	261449	115	148	5.93727e-07	44.0355	cl02787	Translation_Factor_II_like superfamily	-
Q#2078 - >Ca_24691.1|sca	superfamily	276301	49	103	8.1098e-29	106.214	cl21457	TIM_phosphate_binding superfamily	-
Q#2079 - >Ca_24704.1|sca	specific	258418	7	121	5.87979e-19	79.977	pfam14226	DIOX_N	cl21672
Q#2079 - >Ca_24704.1|sca	superfamily	272092	7	121	5.87979e-19	79.977	cl21672	DIOX_N superfamily	-
Q#2079 - >Ca_24704.1|sca	specific	251775	167	268	5.30248e-16	71.6718	pfam03171	2OG-FeII_Oxy	cl21496
Q#2079 - >Ca_24704.1|sca	superfamily	276328	167	268	5.30248e-16	71.6718	cl21496	2OG-FeII_Oxy superfamily	-
Q#2079 - >Ca_24704.1|sca	multi-dom	177993	4	297	3.36909e-35	130.296	PLN02365	PLN02365	-
Q#2081 - >Ca_24714.1|sca	specific	175973	25	60	0.000920459	32.8091	cd00030	C2	cl14603
Q#2081 - >Ca_24714.1|sca	superfamily	271750	25	60	0.000920459	32.8091	cl14603	C2 superfamily	-
Q#2082 - >Ca_24720.1|sca	specific	251669	157	290	3.20769e-32	118.671	pfam03004	Transposase_24	cl03830
Q#2082 - >Ca_24720.1|sca	superfamily	251669	157	290	3.20769e-32	118.671	cl03830	Transposase_24 superfamily	-
Q#2083 - >Ca_24724.1|sca	superfamily	261353	1	146	6.94355e-22	88.9778	cl02572	PIPKc superfamily	-
Q#2084 - >Ca_24728.1|sca	specific	252133	11	111	1.37947e-16	72.3713	pfam03732	Retrotrans_gag	cl04237
Q#2084 - >Ca_24728.1|sca	superfamily	252133	11	111	1.37947e-16	72.3713	cl04237	Retrotrans_gag superfamily	-
Q#2084 - >Ca_24728.1|sca	specific	189387	194	210	0.000139376	38.2041	pfam00098	zf-CCHC	cl22700
Q#2084 - >Ca_24728.1|sca	superfamily	276654	194	210	0.000139376	38.2041	cl22700	zf-CCHC superfamily	-
Q#2084 - >Ca_24728.1|sca	specific	197667	174	190	0.00874262	33.1856	smart00343	ZnF_C2HC	cl22700
Q#2084 - >Ca_24728.1|sca	superfamily	276654	174	190	0.00874262	33.1856	cl22700	zf-CCHC superfamily	-
Q#2085 - >Ca_24730.1|sca	superfamily	252825	1	114	1.85093e-26	97.7115	cl04786	SOUL superfamily	-

Query	Types	PSSM-ID	From	To	E-Value	Bitscore	Accession	Short name	Superfamily
Q#2087 - >Ca_24736.1|sca	specific	133136	362	406	3.37941e-06	44.6348	cd00303	retropepsin_like	cl11403
Q#2087 - >Ca_24736.1|sca	superfamily	275927	362	406	3.37941e-06	44.6348	cl11403	pepsin_retropepsin_like superfamily	-
Q#2087 - >Ca_24736.1|sca	specific	252133	100	199	3.83404e-13	65.0525	pfam03732	Retrotrans_gag	cl04237
Q#2087 - >Ca_24736.1|sca	superfamily	252133	100	199	3.83404e-13	65.0525	cl04237	Retrotrans_gag superfamily	-
Q#2087 - >Ca_24736.1|sca	specific	189387	305	322	0.0016594	36.2781	pfam00098	zf-CCHC	cl22700
Q#2087 - >Ca_24736.1|sca	superfamily	276654	305	322	0.0016594	36.2781	cl22700	zf-CCHC superfamily	-
Q#2091 - >Ca_24770.1|sca	superfamily	254387	385	514	1.27591e-66	218.095	cl06662	RVT_2 superfamily	-
Q#2091 - >Ca_24770.1|sca	specific	258213	20	48	2.76101e-06	44.7242	pfam13961	DUF4219	cl22382
Q#2091 - >Ca_24770.1|sca	superfamily	258213	20	48	2.76101e-06	44.7242	cl22382	DUF4219 superfamily	-
Q#2092 - >Ca_24778.1|sca	superfamily	267823	89	143	3.56284e-10	53.7007	cl19470	PTS_2-RNA superfamily	-
Q#2095 - >Ca_24796.1|sca	superfamily	250720	52	82	0.000174419	36.1125	cl22462	TIR superfamily	-
Q#2096 - >Ca_24803.1|sca	superfamily	256046	93	196	0.0053294	36.2184	cl11168	PMD superfamily	-
Q#2099 - >Ca_24824.1|sca	multi-dom	258063	12	65	0.00379391	33.7262	pfam13779	DUF4175	-
Q#2102 - >Ca_24832.1|sca	superfamily	276297	309	329	9.88766e-05	41.7372	cl21453	PKc_like superfamily	-
Q#2103 - >Ca_24835.1|sca	multi-dom	147601	279	451	1.60811e-05	46.1148	pfam05505	Ebola_NP	-
Q#2104 - >Ca_24836.1|sca	specific	223739	1	164	2.63821e-24	96.2426	COG0667	Tas	cl00470
Q#2104 - >Ca_24836.1|sca	superfamily	260445	1	164	2.63821e-24	96.2426	cl00470	Aldo_ket_red superfamily	-
Q#2106 - >Ca_24856.1|sca	superfamily	276192	1	63	5.66299e-36	118.15	cl17169	RRM_SF superfamily	-
Q#2106 - >Ca_24856.1|sca	multi-dom	214636	8	59	7.46326e-13	57.9887	smart00360	RRM	-
Q#2107 - >Ca_24857.1|sca	multi-dom	259434	221	379	0.000983357	39.9059	pfam15301	SLAIN	-
Q#2108 - >Ca_24858.1|sca	multi-dom	215369	64	86	0.000424349	37.2889	PLN02685	PLN02685	-
Q#2109 - >Ca_24869.1|sca	superfamily	261317	18	94	5.31122e-28	103.475	cl02511	GH64-TLP-SF superfamily	-
Q#2110 - >Ca_24872.1|sca	specific	260004	68	206	6.2055e-71	226.196	cd09272	RNase_HI_RT_Ty1	cl14782
Q#2110 - >Ca_24872.1|sca	superfamily	271754	68	206	6.2055e-71	226.196	cl14782	RNase_H_like superfamily	-
Q#2113 - >Ca_24887.1|sca	specific	250692	26	49	0.00521543	33.1961	pfam01535	PPR	cl03252
Q#2113 - >Ca_24887.1|sca	superfamily	275791	26	49	0.00521543	33.1961	cl03252	PPR superfamily	-
Q#2115 - >Ca_24894.1|sca	specific	252133	145	244	3.48588e-13	64.6673	pfam03732	Retrotrans_gag	cl04237
Q#2115 - >Ca_24894.1|sca	superfamily	252133	145	244	3.48588e-13	64.6673	cl04237	Retrotrans_gag superfamily	-
Q#2120 - >Ca_24908.1|sca	specific	238825	569	714	1.36952e-76	246.738	cd01647	RT_LTR	cl02808
Q#2120 - >Ca_24908.1|sca	superfamily	261455	569	714	1.36952e-76	246.738	cl02808	RT_like superfamily	-
Q#2120 - >Ca_24908.1|sca	specific	133136	362	449	1.06159e-17	79.6879	cd00303	retropepsin_like	cl11403
Q#2120 - >Ca_24908.1|sca	superfamily	275927	362	449	1.06159e-17	79.6879	cl11403	pepsin_retropepsin_like superfamily	-
Q#2120 - >Ca_24908.1|sca	specific	252133	100	199	1.52828e-12	64.6673	pfam03732	Retrotrans_gag	cl04237
Q#2120 - >Ca_24908.1|sca	superfamily	252133	100	199	1.52828e-12	64.6673	cl04237	Retrotrans_gag superfamily	-
Q#2120 - >Ca_24908.1|sca	specific	189387	305	322	0.000629681	38.5893	pfam00098	zf-CCHC	cl22700
Q#2120 - >Ca_24908.1|sca	superfamily	276654	305	322	0.000629681	38.5893	cl22700	zf-CCHC superfamily	-

Query	Types	PSSM-ID	From	To	E-Value	Bitscore	Accession	Short name	Superfamily
Q#2120 - >Ca_24908.1|sca	multi-dom	249567	586	713	4.15236e-26	107.031	pfam00078	RVT_1	-
Q#2120 - >Ca_24908.1|sca	multi-dom	173561	287	366	3.76199e-09	55.5832	PTZ00368	PTZ00368	-
Q#2121 - >Ca_24911.1|sca	specific	238083	17	155	2.221e-27	105.101	cd00143	PP2Cc	cl00120
Q#2121 - >Ca_24911.1|sca	superfamily	260203	17	155	2.221e-27	105.101	cl00120	PP2Cc superfamily	-
Q#2124 - >Ca_24937.1|sca	specific	273253	17	46	0.000364343	33.5827	TIGR00756	PPR	cl03252
Q#2124 - >Ca_24937.1|sca	superfamily	275791	17	46	0.000364343	33.5827	cl03252	PPR superfamily	-
Q#2125 - >Ca_24940.1|sca	superfamily	254387	83	212	2.7056e-78	237.355	cl06662	RVT_2 superfamily	-
Q#2126 - >Ca_24952.1|sca	superfamily	252133	79	177	5.65183e-11	57.7337	cl04237	Retrotrans_gag superfamily	-
Q#2127 - >Ca_24954.1|sca	superfamily	252133	80	122	8.77246e-07	43.0961	cl04237	Retrotrans_gag superfamily	-
Q#2128 - >Ca_24955.1|sca	multi-dom	182581	4	48	0.00279425	33.8756	PRK10600	PRK10600	-
Q#2131 - >Ca_24974.1|sca	specific	251717	116	300	6.46531e-62	198.322	pfam03080	DUF239	cl03877
Q#2131 - >Ca_24974.1|sca	superfamily	251717	116	300	6.46531e-62	198.322	cl03877	DUF239 superfamily	-
Q#2131 - >Ca_24974.1|sca	specific	206533	2	111	3.95714e-40	137.791	pfam14365	DUF4409	cl16830
Q#2131 - >Ca_24974.1|sca	superfamily	206533	2	111	3.95714e-40	137.791	cl16830	DUF4409 superfamily	-
Q#2132 - >Ca_24978.1|sca	multi-dom	258063	28	128	0.000850227	38.3486	pfam13779	DUF4175	-
Q#2133 - >Ca_24979.1|sca	specific	189387	23	40	0.00192563	36.2781	pfam00098	zf-CCHC	cl22700
Q#2133 - >Ca_24979.1|sca	superfamily	276654	23	40	0.00192563	36.2781	cl22700	zf-CCHC superfamily	-
Q#2136 - >Ca_24987.1|sca	superfamily	252133	100	157	0.00138541	35.0069	cl04237	Retrotrans_gag superfamily	-
Q#2138 - >Ca_25000.1|sca	specific	238093	130	177	4.94347e-10	52.0628	cd00162	RING	cl17238
Q#2138 - >Ca_25000.1|sca	superfamily	276201	130	177	4.94347e-10	52.0628	cl17238	RING superfamily	-
Q#2138 - >Ca_25000.1|sca	multi-dom	257215	130	174	6.72205e-11	54.7503	pfam12678	zf-rbx1	-
Q#2139 - >Ca_25004.1|sca	superfamily	251669	2	90	0.00152159	34.6977	cl03830	Transposase_24 superfamily	-
Q#2140 - >Ca_25029.1|sca	superfamily	275737	49	84	7.2939e-06	40.9806	cl01629	TPP_enzymes superfamily	-
Q#2145 - >Ca_25048.1|sca	superfamily	276235	30	244	5.31455e-70	222.949	cl19188	Pectate_lyase_3 superfamily	-
Q#2149 - >Ca_25067.1|sca	superfamily	275859	149	200	1.27271e-05	42.1497	cl08302	EFh superfamily	-
Q#2149 - >Ca_25067.1|sca	superfamily	275859	230	291	5.15665e-05	40.2237	cl08302	EFh superfamily	-
Q#2149 - >Ca_25067.1|sca	superfamily	275859	109	166	0.00157538	35.9865	cl08302	EFh superfamily	-
Q#2149 - >Ca_25067.1|sca	multi-dom	185504	138	290	4.46029e-05	41.6706	PTZ00184	PTZ00184	-
Q#2152 - >Ca_25086.1|sca	superfamily	275927	220	287	6.28854e-08	50.1107	cl11403	pepsin_retropepsin_like superfamily	-
Q#2152 - >Ca_25086.1|sca	specific	189387	154	170	0.00271695	35.1225	pfam00098	zf-CCHC	cl22700
Q#2152 - >Ca_25086.1|sca	superfamily	276654	154	170	0.00271695	35.1225	cl22700	zf-CCHC superfamily	-
Q#2153 - >Ca_25087.1|sca	superfamily	254387	126	168	1.75115e-15	70.1779	cl06662	RVT_2 superfamily	-
Q#2155 - >Ca_25109.1|sca	specific	212548	49	150	5.97559e-26	96.5552	cd11710	GINS_A_psf1	cl17012
Q#2155 - >Ca_25109.1|sca	superfamily	266497	49	150	5.97559e-26	96.5552	cl17012	GINS_A superfamily	-
Q#2156 - >Ca_25110.1|sca	multi-dom	273731	23	162	1.94532e-30	117.043	TIGR01647	Plasma_membrane_ATPase	-
Q#2158 - >Ca_25113.1|sca	specific	189387	198	214	0.000889631	36.2781	pfam00098	zf-CCHC	cl22700

Query	Types	PSSM-ID	From	To	E-Value	Bitscore	Accession	Short name	Superfamily
Q#2158 - >Ca_25113.1|sca	superfamily	276654	198	214	0.000889631	36.2781	cl22700	zf-CCHC superfamily	-
Q#2159 - >Ca_25120.1|sca	specific	214842	357	409	3.94014e-07	47.1922	smart00831	Cation_ATPase_N	cl02930
Q#2159 - >Ca_25120.1|sca	superfamily	261517	357	409	3.94014e-07	47.1922	cl02930	Cation_ATPase_N superfamily	-
Q#2159 - >Ca_25120.1|sca	superfamily	265821	1	44	1.93166e-05	42.5597	cl15874	UBN2 superfamily	-
Q#2159 - >Ca_25120.1|sca	superfamily	271847	245	332	0.000930894	39.9289	cl19219	DUF342 superfamily	-
Q#2159 - >Ca_25120.1|sca	specific	189387	110	126	0.00257208	35.5077	pfam00098	zf-CCHC	cl22700
Q#2159 - >Ca_25120.1|sca	superfamily	276654	110	126	0.00257208	35.5077	cl22700	zf-CCHC superfamily	-
Q#2161 - >Ca_25123.1|sca	superfamily	256046	13	42	0.0047966	35.0628	cl11168	PMD superfamily	-
Q#2162 - >Ca_25126.1|sca	superfamily	276303	60	199	4.20153e-22	90.08	cl21460	HAD_like superfamily	-
Q#2163 - >Ca_25133.1|sca	superfamily	252593	43	96	3.15232e-07	44.1429	cl04514	FrhB_FdhB_C superfamily	-
Q#2164 - >Ca_25135.1|sca	multi-dom	183854	236	294	0.00198461	38.4605	PRK13042	PRK13042	-
Q#2168 - >Ca_25153.1|sca	superfamily	271754	1	57	3.81581e-23	91.0149	cl14782	RNase_H_like superfamily	-
Q#2170 - >Ca_25158.1|sca	multi-dom	227278	66	139	0.000444283	40.0905	COG4942	COG4942	-
Q#2171 - >Ca_25160.1|sca	superfamily	251669	22	75	0.000203678	38.5497	cl03830	Transposase_24 superfamily	-
Q#2172 - >Ca_25162.1|sca	superfamily	256046	52	108	3.02865e-06	46.2336	cl11168	PMD superfamily	-
Q#2172 - >Ca_25162.1|sca	superfamily	256046	104	169	8.32792e-06	44.6928	cl11168	PMD superfamily	-
Q#2173 - >Ca_25164.1|sca	specific	273253	33	65	4.87349e-07	42.0571	TIGR00756	PPR	cl03252
Q#2173 - >Ca_25164.1|sca	superfamily	275791	33	65	4.87349e-07	42.0571	cl03252	PPR superfamily	-
Q#2173 - >Ca_25164.1|sca	multi-dom	257477	33	75	5.56295e-12	56.257	pfam13041	PPR_2	-
Q#2175 - >Ca_25176.1|sca	multi-dom	256956	269	429	0.00577934	37.6455	pfam12287	Caprin-1_C	-
Q#2179 - >Ca_25190.1|sca	superfamily	191114	151	196	0.00243829	35.408	cl04804	DUF641 superfamily	-
Q#2182 - >Ca_25201.1|sca	superfamily	275507	12	91	2.46404e-33	121.888	cl00268	class_II_aaRS-like_core superfamily	-
Q#2182 - >Ca_25201.1|sca	superfamily	271451	134	182	1.72046e-11	57.1285	cl00266	HGTP_anticodon superfamily	-
Q#2186 - >Ca_25210.1|sca	superfamily	251850	122	196	4.14024e-19	82.7904	cl03979	PAE superfamily	-
Q#2188 - >Ca_25214.1|sca	superfamily	276263	122	246	9.52901e-15	70.466	cl19514	FBA_1 superfamily	-
Q#2188 - >Ca_25214.1|sca	specific	197608	16	56	3.63046e-09	51.6686	smart00256	FBOX	cl02535
Q#2188 - >Ca_25214.1|sca	superfamily	261329	16	56	3.63046e-09	51.6686	cl02535	F-box superfamily	-
Q#2189 - >Ca_25215.1|sca	specific	238926	28	116	2.83601e-32	110.517	cd01960	nsLTP1	cl07890
Q#2189 - >Ca_25215.1|sca	superfamily	275845	28	116	2.83601e-32	110.517	cl07890	AAI_LTSS superfamily	-
Q#2190 - >Ca_25220.1|sca	superfamily	251669	527	599	3.19791e-17	79.3808	cl03830	Transposase_24 superfamily	-
Q#2190 - >Ca_25220.1|sca	superfamily	206130	1	27	2.80085e-13	67.6139	cl16501	DUF4218 superfamily	-
Q#2190 - >Ca_25220.1|sca	superfamily	258207	172	202	4.15002e-05	42.1831	cl16495	DUF4216 superfamily	-
Q#2190 - >Ca_25220.1|sca	superfamily	251500	664	718	0.00548506	36.8942	cl03682	SKIP_SNW superfamily	-
Q#2191 - >Ca_25225.1|sca	superfamily	252133	100	166	2.02937e-08	48.1037	cl04237	Retrotrans_gag superfamily	-
Q#2194 - >Ca_25233.1|sca	superfamily	254387	314	453	3.81495e-81	253.533	cl06662	RVT_2 superfamily	-
Q#2195 - >Ca_25236.1|sca	superfamily	254387	689	745	1.05099e-13	70.5631	cl06662	RVT_2 superfamily	-

Query	Types	PSSM-ID	From	To	E-Value	Bitscore	Accession	Short name	Superfamily
Q#2199 - >Ca_25259.1|sca	superfamily	276192	1	63	5.66299e-36	118.15	cl17169	RRM_SF superfamily	-
Q#2199 - >Ca_25259.1|sca	multi-dom	214636	8	59	7.46326e-13	57.9887	smart00360	RRM	-
Q#2200 - >Ca_25262.1|sca	specific	275441	2	155	2.12844e-29	106.352	cd15797	PMEI	cl04375
Q#2200 - >Ca_25262.1|sca	superfamily	275808	2	155	2.12844e-29	106.352	cl04375	PMEI_like superfamily	-
Q#2201 - >Ca_25270.1|sca	specific	251775	199	276	0.00775007	34.3075	pfam03171	2OG-FeII_Oxy	cl21496
Q#2201 - >Ca_25270.1|sca	superfamily	276328	199	276	0.00775007	34.3075	cl21496	2OG-FeII_Oxy superfamily	-
Q#2201 - >Ca_25270.1|sca	multi-dom	226022	7	312	1.14561e-09	57.7607	COG3491	PcbC	-
Q#2204 - >Ca_25279.1|sca	specific	189387	145	160	0.000360289	36.2781	pfam00098	zf-CCHC	cl22700
Q#2204 - >Ca_25279.1|sca	superfamily	276654	145	160	0.000360289	36.2781	cl22700	zf-CCHC superfamily	-
Q#2205 - >Ca_25285.1|sca	specific	133136	98	151	1.85738e-08	48.4868	cd00303	retropepsin_like	cl11403
Q#2205 - >Ca_25285.1|sca	superfamily	275927	98	151	1.85738e-08	48.4868	cl11403	pepsin_retropepsin_like superfamily	-
Q#2208 - >Ca_25292.1|sca	specific	250208	3	170	2.1013e-68	210.183	pfam00891	Methyltransf_2	cl17173
Q#2208 - >Ca_25292.1|sca	superfamily	276194	3	170	2.1013e-68	210.183	cl17173	AdoMet_MTases superfamily	-
Q#2209 - >Ca_25294.1|sca	specific	100107	99	187	0.000938287	36.6391	cd02440	AdoMet_MTases	cl17173
Q#2209 - >Ca_25294.1|sca	superfamily	276194	99	187	0.000938287	36.6391	cl17173	AdoMet_MTases superfamily	-
Q#2209 - >Ca_25294.1|sca	multi-dom	131763	15	205	1.14519e-16	76.2431	TIGR02716	C-20_methyltransferase	-
Q#2211 - >Ca_25303.1|sca	superfamily	252133	101	199	1.19117e-11	58.1189	cl04237	Retrotrans_gag superfamily	-
Q#2213 - >Ca_25307.1|sca	multi-dom	253927	109	176	0.0021752	36.7954	pfam06818	Fez1	-
Q#2214 - >Ca_25316.1|sca	superfamily	253459	18	57	4.3676e-07	44.6849	cl05469	Mitovir_RNA_pol superfamily	-
Q#2216 - >Ca_25320.1|sca	multi-dom	178748	251	423	9.09929e-06	46.8431	PLN03209	PLN03209	-
Q#2217 - >Ca_25321.1|sca	superfamily	252133	100	199	1.0322e-10	56.5781	cl04237	Retrotrans_gag superfamily	-
Q#2218 - >Ca_25322.1|sca	specific	257592	408	593	5.58946e-56	191.239	pfam13234	rRNA_proc-arch	cl00354
Q#2218 - >Ca_25322.1|sca	superfamily	275547	408	593	5.58946e-56	191.239	cl00354	KOW superfamily	-
Q#2218 - >Ca_25322.1|sca	specific	258228	232	277	2.28117e-05	42.7292	pfam13976	gag_pre-integrs	cl16514
Q#2218 - >Ca_25322.1|sca	superfamily	258228	232	277	2.28117e-05	42.7292	cl16514	gag_pre-integrs superfamily	-
Q#2219 - >Ca_25323.1|sca	specific	238825	442	517	5.76148e-35	131.564	cd01647	RT_LTR	cl02808
Q#2219 - >Ca_25323.1|sca	superfamily	261455	442	517	5.76148e-35	131.564	cl02808	RT_like superfamily	-
Q#2219 - >Ca_25323.1|sca	superfamily	275927	243	342	2.29282e-10	58.9703	cl11403	pepsin_retropepsin_like superfamily	-
Q#2220 - >Ca_25325.1|sca	superfamily	276034	80	123	0.00810461	33.6287	cl13995	MPP_superfamily superfamily	-
Q#2223 - >Ca_25329.1|sca	superfamily	252133	45	153	2.26032e-07	46.1777	cl04237	Retrotrans_gag superfamily	-
Q#2224 - >Ca_25334.1|sca	superfamily	271753	6	126	1.11416e-19	80.6965	cl14643	SRPBCC superfamily	-
Q#2225 - >Ca_25336.1|sca	specific	176858	5	152	4.35507e-55	172.374	cd07816	Bet_v1-like	cl14643
Q#2225 - >Ca_25336.1|sca	superfamily	271753	5	152	4.35507e-55	172.374	cl14643	SRPBCC superfamily	-
Q#2228 - >Ca_25358.1|sca	superfamily	252133	45	115	3.56967e-11	56.1929	cl04237	Retrotrans_gag superfamily	-
Q#2230 - >Ca_25363.1|sca	multi-dom	258434	48	162	1.11129e-26	102.046	pfam14244	UBN2_3	-
Q#2232 - >Ca_25372.1|sca	superfamily	260145	5	43	0.00082873	34.2135	cl00030	CH superfamily	-

Query	Types	PSSM-ID	From	To	E-Value	Bitscore	Accession	Short name	Superfamily
Q#2233 - >Ca_25374.1|sca	superfamily	275927	325	424	8.58443e-12	62.4371	cl11403	pepsin_retropepsin_like superfamily	-
Q#2233 - >Ca_25374.1|sca	superfamily	252133	72	170	4.43595e-11	59.6597	cl04237	Retrotrans_gag superfamily	-
Q#2233 - >Ca_25374.1|sca	specific	197667	286	302	0.00970516	34.3412	smart00343	ZnF_C2HC	cl22700
Q#2233 - >Ca_25374.1|sca	superfamily	276654	286	302	0.00970516	34.3412	cl22700	zf-CCHC superfamily	-
Q#2233 - >Ca_25374.1|sca	multi-dom	173561	268	333	5.51066e-08	50.9609	PTZ00368	PTZ00368	-
Q#2238 - >Ca_25396.1|sca	superfamily	276301	67	99	3.77602e-11	57.2941	cl21457	TIM_phosphate_binding superfamily	-
Q#2240 - >Ca_25403.1|sca	superfamily	260992	79	117	0.00647323	35.763	cl01545	DUF1853 superfamily	-
Q#2241 - >Ca_25406.1|sca	superfamily	256046	84	262	9.40136e-20	87.45	cl11168	PMD superfamily	-
Q#2243 - >Ca_25418.1|sca	specific	189387	52	65	0.00517489	30.8853	pfam00098	zf-CCHC	cl22700
Q#2243 - >Ca_25418.1|sca	superfamily	276654	52	65	0.00517489	30.8853	cl22700	zf-CCHC superfamily	-
Q#2247 - >Ca_25432.1|sca	superfamily	206130	1	81	5.57512e-51	163.529	cl16501	DUF4218 superfamily	-
Q#2247 - >Ca_25432.1|sca	superfamily	258207	112	150	1.16113e-10	54.8947	cl16495	DUF4216 superfamily	-
Q#2249 - >Ca_25446.1|sca	superfamily	276192	32	60	1.50736e-09	49.9427	cl17169	RRM_SF superfamily	-
Q#2249 - >Ca_25446.1|sca	multi-dom	214636	21	59	1.20326e-06	41.8104	smart00360	RRM	-
Q#2250 - >Ca_25449.1|sca	superfamily	252147	97	212	2.84515e-19	80.4565	cl15991	DUF313 superfamily	-
Q#2251 - >Ca_25453.1|sca	superfamily	276299	262	294	1.79445e-08	53.0352	cl21455	ABC_ATPase superfamily	-
Q#2253 - >Ca_25460.1|sca	superfamily	251669	22	120	6.00164e-10	54.728	cl03830	Transposase_24 superfamily	-
Q#2255 - >Ca_25469.1|sca	specific	238825	569	714	2.04382e-78	250.59	cd01647	RT_LTR	cl02808
Q#2255 - >Ca_25469.1|sca	superfamily	261455	569	714	2.04382e-78	250.59	cl02808	RT_like superfamily	-
Q#2255 - >Ca_25469.1|sca	specific	133136	362	449	9.16636e-18	79.6879	cd00303	retropepsin_like	cl11403
Q#2255 - >Ca_25469.1|sca	superfamily	275927	362	449	9.16636e-18	79.6879	cl11403	pepsin_retropepsin_like superfamily	-
Q#2255 - >Ca_25469.1|sca	specific	252133	100	199	1.07514e-12	65.0525	pfam03732	Retrotrans_gag	cl04237
Q#2255 - >Ca_25469.1|sca	superfamily	252133	100	199	1.07514e-12	65.0525	cl04237	Retrotrans_gag superfamily	-
Q#2255 - >Ca_25469.1|sca	specific	189387	305	322	0.000843218	37.8189	pfam00098	zf-CCHC	cl22700
Q#2255 - >Ca_25469.1|sca	superfamily	276654	305	322	0.000843218	37.8189	cl22700	zf-CCHC superfamily	-
Q#2255 - >Ca_25469.1|sca	multi-dom	249567	586	713	2.15428e-27	110.113	pfam00078	RVT_1	-
Q#2255 - >Ca_25469.1|sca	multi-dom	173561	287	366	8.39881e-09	54.4276	PTZ00368	PTZ00368	-
Q#2262 - >Ca_25486.1|sca	specific	258650	83	159	5.85683e-18	75.3214	pfam14510	ABC_trans_N	cl20552
Q#2262 - >Ca_25486.1|sca	superfamily	258650	83	159	5.85683e-18	75.3214	cl20552	ABC_trans_N superfamily	-
Q#2263 - >Ca_25489.1|sca	superfamily	256046	5	127	1.09792e-36	132.133	cl11168	PMD superfamily	-
Q#2264 - >Ca_25495.1|sca	specific	133136	362	406	3.37941e-06	44.6348	cd00303	retropepsin_like	cl11403
Q#2264 - >Ca_25495.1|sca	superfamily	275927	362	406	3.37941e-06	44.6348	cl11403	pepsin_retropepsin_like superfamily	-
Q#2264 - >Ca_25495.1|sca	specific	252133	100	199	3.61717e-13	65.4377	pfam03732	Retrotrans_gag	cl04237
Q#2264 - >Ca_25495.1|sca	superfamily	252133	100	199	3.61717e-13	65.4377	cl04237	Retrotrans_gag superfamily	-
Q#2264 - >Ca_25495.1|sca	specific	189387	305	322	0.00272599	35.5077	pfam00098	zf-CCHC	cl22700
Q#2264 - >Ca_25495.1|sca	superfamily	276654	305	322	0.00272599	35.5077	cl22700	zf-CCHC superfamily	-

Query	Types	PSSM-ID	From	To	E-Value	Bitscore	Accession	Short name	Superfamily
Q#2265 - >Ca_25507.1|sca	specific	211392	218	365	2.26991e-31	116.465	cd11380	Ribosomal_S8e_like	cl00880
Q#2265 - >Ca_25507.1|sca	superfamily	260676	218	365	2.26991e-31	116.465	cl00880	Ribosomal_S8e_like superfamily	-
Q#2265 - >Ca_25507.1|sca	superfamily	276193	66	129	9.1328e-30	111.892	cl17171	PH-like superfamily	-
Q#2265 - >Ca_25507.1|sca	specific	258173	193	219	0.000275784	38.3791	pfam13906	AA_permease_C	cl18287
Q#2265 - >Ca_25507.1|sca	superfamily	258173	193	219	0.000275784	38.3791	cl18287	AA_permease_C superfamily	-
Q#2267 - >Ca_25511.1|sca	specific	273253	90	123	2.45228e-05	41.6719	TIGR00756	PPR	cl03252
Q#2267 - >Ca_25511.1|sca	superfamily	275791	90	123	2.45228e-05	41.6719	cl03252	PPR superfamily	-
Q#2267 - >Ca_25511.1|sca	multi-dom	215561	41	504	6.53627e-50	183.127	PLN03077	PLN03077	-
Q#2268 - >Ca_25523.1|sca	superfamily	275753	1	90	1.49469e-50	166.634	cl01911	Pectinesterase superfamily	-
Q#2271 - >Ca_25547.1|sca	superfamily	261267	30	93	1.07598e-13	65.2445	cl02381	Tim17 superfamily	-
Q#2271 - >Ca_25547.1|sca	superfamily	276297	183	245	3.51613e-08	51.5046	cl21453	PKc_like superfamily	-
Q#2274 - >Ca_25564.1|sca	superfamily	275482	3	176	1.34922e-86	260.95	cl00200	MIP superfamily	-
Q#2275 - >Ca_25567.1|sca	superfamily	276263	103	333	9.62548e-18	80.4812	cl19514	FBA_1 superfamily	-
Q#2275 - >Ca_25567.1|sca	specific	197608	11	47	1.13579e-06	45.1202	smart00256	FBOX	cl02535
Q#2275 - >Ca_25567.1|sca	superfamily	261329	11	47	1.13579e-06	45.1202	cl02535	F-box superfamily	-
Q#2278 - >Ca_25574.1|sca	superfamily	276194	2	129	9.51274e-65	207.489	cl17173	AdoMet_MTases superfamily	-
Q#2281 - >Ca_25600.1|sca	superfamily	275466	29	189	3.34886e-32	116.15	cl00064	ZnMc superfamily	-
Q#2281 - >Ca_25600.1|sca	multi-dom	249842	35	189	3.0051e-32	116.191	pfam00413	Peptidase_M10	-
Q#2282 - >Ca_25601.1|sca	superfamily	276192	72	118	3.52328e-10	52.9615	cl17169	RRM_SF superfamily	-
Q#2282 - >Ca_25601.1|sca	superfamily	276192	7	36	2.54222e-08	47.5288	cl17169	RRM_SF superfamily	-
Q#2284 - >Ca_25605.1|sca	superfamily	252133	100	199	2.97451e-12	61.9709	cl04237	Retrotrans_gag superfamily	-
Q#2284 - >Ca_25605.1|sca	specific	189387	276	293	0.00120837	36.2781	pfam00098	zf-CCHC	cl22700
Q#2284 - >Ca_25605.1|sca	superfamily	276654	276	293	0.00120837	36.2781	cl22700	zf-CCHC superfamily	-
Q#2285 - >Ca_25621.1|sca	superfamily	252133	100	199	1.42335e-12	61.9709	cl04237	Retrotrans_gag superfamily	-
Q#2290 - >Ca_25630.1|sca	superfamily	252133	75	174	1.10663e-08	49.6445	cl04237	Retrotrans_gag superfamily	-
Q#2291 - >Ca_25635.1|sca	superfamily	275753	93	148	6.49635e-15	68.7928	cl01911	Pectinesterase superfamily	-
Q#2291 - >Ca_25635.1|sca	superfamily	275808	10	53	2.18458e-09	51.2567	cl04375	PMEI_like superfamily	-
Q#2292 - >Ca_25638.1|sca	specific	258215	3	83	3.67493e-22	88.5384	pfam13963	Transpos_assoc	cl16504
Q#2292 - >Ca_25638.1|sca	superfamily	258215	3	83	3.67493e-22	88.5384	cl16504	Transpos_assoc superfamily	-
Q#2292 - >Ca_25638.1|sca	superfamily	202502	292	335	2.48368e-20	87.3162	cl03823	Transposase_21 superfamily	-
Q#2293 - >Ca_25643.1|sca	specific	237985	18	76	1.36927e-30	108.128	cd00018	AP2	cl00033
Q#2293 - >Ca_25643.1|sca	superfamily	260146	18	76	1.36927e-30	108.128	cl00033	AP2 superfamily	-
Q#2294 - >Ca_25646.1|sca	specific	211392	6	75	1.21195e-27	101.057	cd11380	Ribosomal_S8e_like	cl00880
Q#2294 - >Ca_25646.1|sca	superfamily	260676	6	75	1.21195e-27	101.057	cl00880	Ribosomal_S8e_like superfamily	-
Q#2294 - >Ca_25646.1|sca	multi-dom	240292	6	148	1.7711e-60	187.891	PTZ00148	PTZ00148	-
Q#2295 - >Ca_25647.1|sca	superfamily	271701	91	141	6.67952e-11	59.534	cl10459	Peptidases_S8_S53 superfamily	-

Query	Types	PSSM-ID	From	To	E-Value	Bitscore	Accession	Short name	Superfamily
Q#2297 - >Ca_25662.1|sca	superfamily	276241	2	158	7.72076e-29	108.939	cl19217	Mem_trans superfamily	-
Q#2298 - >Ca_25665.1|sca	specific	252735	1	200	3.5326e-126	359.72	pfam04674	Phi_1	cl04664
Q#2298 - >Ca_25665.1|sca	superfamily	252735	1	200	3.5326e-126	359.72	cl04664	Phi_1 superfamily	-
Q#2303 - >Ca_25684.1|sca	multi-dom	114603	121	178	0.00160607	38.0164	pfam05887	Trypan_PARP	-
Q#2308 - >Ca_25720.1|sca	superfamily	252133	101	175	7.36136e-09	49.6445	cl04237	Retrotrans_gag superfamily	-
Q#2309 - >Ca_25726.1|sca	specific	258415	89	181	5.296e-12	60.664	pfam14223	UBN2	cl15874
Q#2309 - >Ca_25726.1|sca	superfamily	265821	89	181	5.296e-12	60.664	cl15874	UBN2 superfamily	-
Q#2309 - >Ca_25726.1|sca	multi-dom	258434	22	131	0.000548304	37.7178	pfam14244	UBN2_3	-
Q#2311 - >Ca_25737.1|sca	specific	252133	168	267	1.28929e-13	65.4377	pfam03732	Retrotrans_gag	cl04237
Q#2311 - >Ca_25737.1|sca	superfamily	252133	168	267	1.28929e-13	65.4377	cl04237	Retrotrans_gag superfamily	-
Q#2315 - >Ca_25744.1|sca	specific	250209	96	219	7.16666e-06	43.0007	pfam00892	EamA	cl00910
Q#2315 - >Ca_25744.1|sca	superfamily	275677	96	219	7.16666e-06	43.0007	cl00910	EamA superfamily	-
Q#2317 - >Ca_25758.1|sca	specific	260004	211	349	3.19122e-62	206.55	cd09272	RNase_HI_RT_Ty1	cl14782
Q#2317 - >Ca_25758.1|sca	superfamily	271754	211	349	3.19122e-62	206.55	cl14782	RNase_H_like superfamily	-
Q#2321 - >Ca_25793.1|sca	superfamily	190526	188	221	7.38592e-08	48.753	cl07846	DBD_Tnp_Mut superfamily	-
Q#2322 - >Ca_25799.1|sca	superfamily	251768	6	58	1.96775e-14	66.2558	cl20246	Mon1 superfamily	-
Q#2323 - >Ca_25805.1|sca	specific	258415	1	114	1.17208e-26	105.347	pfam14223	UBN2	cl15874
Q#2323 - >Ca_25805.1|sca	superfamily	265821	1	114	1.17208e-26	105.347	cl15874	UBN2 superfamily	-
Q#2324 - >Ca_25806.1|sca	superfamily	206130	1	80	6.3703e-44	144.269	cl16501	DUF4218 superfamily	-
Q#2324 - >Ca_25806.1|sca	superfamily	258207	116	147	9.90027e-13	60.2875	cl16495	DUF4216 superfamily	-
Q#2325 - >Ca_25811.1|sca	multi-dom	258126	22	78	0.00930288	33.2801	pfam13855	LRR_8	-
Q#2326 - >Ca_25814.1|sca	superfamily	276297	117	276	1.35848e-28	115.063	cl21453	PKc_like superfamily	-
Q#2326 - >Ca_25814.1|sca	superfamily	260198	1	40	2.17129e-08	52.0534	cl00112	PAN_APPLE superfamily	-
Q#2327 - >Ca_25815.1|sca	specific	238531	124	196	1.5174e-21	88.6474	cd01098	PAN_AP_plant	cl00112
Q#2327 - >Ca_25815.1|sca	superfamily	260198	124	196	1.5174e-21	88.6474	cl00112	PAN_APPLE superfamily	-
Q#2327 - >Ca_25815.1|sca	superfamily	276297	236	426	6.84677e-47	164.753	cl21453	PKc_like superfamily	-
Q#2327 - >Ca_25815.1|sca	superfamily	201524	28	87	6.45696e-20	85.0244	cl03036	S_locus_glycop superfamily	-
Q#2330 - >Ca_25827.1|sca	superfamily	271686	34	114	7.83731e-22	86.5347	cl10012	DnaQ_like_exo superfamily	-
Q#2332 - >Ca_25835.1|sca	superfamily	151721	5	57	0.00506596	30.6283	cl12822	DUF3081 superfamily	-
Q#2333 - >Ca_25836.1|sca	superfamily	271793	89	256	5.0378e-95	291.475	cl17068	AFD_class_I superfamily	-
Q#2333 - >Ca_25836.1|sca	superfamily	271848	18	97	7.29073e-21	90.138	cl19241	Condensation superfamily	-
Q#2336 - >Ca_25857.1|sca	superfamily	265686	68	122	3.20935e-09	52.2922	cl15397	DUF89 superfamily	-
Q#2337 - >Ca_25862.1|sca	superfamily	275582	280	440	1.92647e-24	106.039	cl00451	MoCF_BD superfamily	-
Q#2338 - >Ca_25864.1|sca	specific	252133	75	174	5.82098e-15	68.1341	pfam03732	Retrotrans_gag	cl04237
Q#2338 - >Ca_25864.1|sca	superfamily	252133	75	174	5.82098e-15	68.1341	cl04237	Retrotrans_gag superfamily	-
Q#2338 - >Ca_25864.1|sca	superfamily	275927	223	265	2.91522e-09	53.1923	cl11403	pepsin_retropepsin_like superfamily	-

Query	Types	PSSM-ID	From	To	E-Value	Bitscore	Accession	Short name	Superfamily
Q#2340 - >Ca_25871.1|sca	specific	252133	100	199	3.31173e-13	66.2081	pfam03732	Retrotrans_gag	cl04237
Q#2340 - >Ca_25871.1|sca	superfamily	252133	100	199	3.31173e-13	66.2081	cl04237	Retrotrans_gag superfamily	-
Q#2340 - >Ca_25871.1|sca	specific	189387	306	322	0.000146311	40.1301	pfam00098	zf-CCHC	cl22700
Q#2340 - >Ca_25871.1|sca	superfamily	276654	306	322	0.000146311	40.1301	cl22700	zf-CCHC superfamily	-
Q#2347 - >Ca_25904.1|sca	specific	237985	54	111	2.2699e-17	73.0747	cd00018	AP2	cl00033
Q#2347 - >Ca_25904.1|sca	superfamily	260146	54	111	2.2699e-17	73.0747	cl00033	AP2 superfamily	-
Q#2348 - >Ca_25905.1|sca	superfamily	276214	19	138	3.26185e-46	154.657	cl18310	NHL superfamily	-
Q#2351 - >Ca_25915.1|sca	specific	238096	89	133	2.06063e-13	61.8226	cd00167	SANT	cl21498
Q#2351 - >Ca_25915.1|sca	superfamily	271918	89	133	2.06063e-13	61.8226	cl21498	SANT superfamily	-
Q#2353 - >Ca_25918.1|sca	superfamily	252133	145	210	0.000144058	40.0145	cl04237	Retrotrans_gag superfamily	-
Q#2353 - >Ca_25918.1|sca	specific	189387	338	354	0.000968286	37.0485	pfam00098	zf-CCHC	cl22700
Q#2353 - >Ca_25918.1|sca	superfamily	276654	338	354	0.000968286	37.0485	cl22700	zf-CCHC superfamily	-
Q#2356 - >Ca_25949.1|sca	superfamily	276298	1	104	2.11741e-28	104.999	cl21454	SDR superfamily	-
Q#2357 - >Ca_25957.1|sca	superfamily	255867	185	260	5.30899e-06	45.04	cl10951	Tmemb_185A superfamily	-
Q#2363 - >Ca_25975.1|sca	specific	237985	121	166	3.52891e-12	58.4371	cd00018	AP2	cl00033
Q#2363 - >Ca_25975.1|sca	superfamily	260146	121	166	3.52891e-12	58.4371	cl00033	AP2 superfamily	-
Q#2363 - >Ca_25975.1|sca	superfamily	260146	67	91	2.71539e-07	44.9485	cl00033	AP2 superfamily	-
Q#2364 - >Ca_25986.1|sca	specific	254387	399	566	4.22582e-94	299.757	pfam07727	RVT_2	cl06662
Q#2364 - >Ca_25986.1|sca	superfamily	254387	399	566	4.22582e-94	299.757	cl06662	RVT_2 superfamily	-
Q#2364 - >Ca_25986.1|sca	specific	258228	84	132	1.01669e-10	59.2928	pfam13976	gag_pre-integrs	cl16514
Q#2364 - >Ca_25986.1|sca	superfamily	258228	84	132	1.01669e-10	59.2928	cl16514	gag_pre-integrs superfamily	-
Q#2364 - >Ca_25986.1|sca	specific	250040	145	221	1.22037e-09	56.9581	pfam00665	rve	cl21549
Q#2364 - >Ca_25986.1|sca	superfamily	276355	145	221	1.22037e-09	56.9581	cl21549	rve superfamily	-
Q#2365 - >Ca_25989.1|sca	superfamily	260527	1	185	9.32532e-43	142.939	cl00607	PUA superfamily	-
Q#2371 - >Ca_26004.1|sca	specific	258215	3	83	3.55763e-23	88.5384	pfam13963	Transpos_assoc	cl16504
Q#2371 - >Ca_26004.1|sca	superfamily	258215	3	83	3.55763e-23	88.5384	cl16504	Transpos_assoc superfamily	-
Q#2374 - >Ca_26015.1|sca	multi-dom	185628	153	306	0.004247	37.747	PTZ00449	PTZ00449	-
Q#2375 - >Ca_26021.1|sca	multi-dom	185575	1	74	1.70717e-17	75.1927	PTZ00361	PTZ00361	-
Q#2378 - >Ca_26028.1|sca	superfamily	252919	18	70	0.00569504	35.7468	cl20284	NMD3 superfamily	-
Q#2378 - >Ca_26028.1|sca	multi-dom	224416	17	224	2.02333e-07	50.08	COG1499	NMD3	-
Q#2381 - >Ca_26035.1|sca	specific	99751	61	115	5.898e-15	65.6445	cd06257	DnaJ	cl02542
Q#2381 - >Ca_26035.1|sca	superfamily	271590	61	115	5.898e-15	65.6445	cl02542	DnaJ superfamily	-
Q#2383 - >Ca_26047.1|sca	specific	253443	13	287	3.09746e-61	197.184	pfam05890	Ebp2	cl05454
Q#2383 - >Ca_26047.1|sca	superfamily	253443	13	287	3.09746e-61	197.184	cl05454	Ebp2 superfamily	-
Q#2386 - >Ca_26056.1|sca	superfamily	253334	1	73	1.13919e-16	70.9541	cl05325	BCAS2 superfamily	-
Q#2387 - >Ca_26058.1|sca	superfamily	267999	11	68	0.00899345	34.6765	cl19646	DUF4138 superfamily	-

Query	Types	PSSM-ID	From	To	E-Value	Bitscore	Accession	Short name	Superfamily
Q#2388 - >Ca_26060.1|sca	multi-dom	258434	10	106	2.53535e-23	93.1866	pfam14244	UBN2_3	-
Q#2391 - >Ca_26089.1|sca	specific	260004	402	540	4.05467e-78	244.685	cd09272	RNase_HI_RT_Ty1	cl14782
Q#2391 - >Ca_26089.1|sca	superfamily	271754	402	540	4.05467e-78	244.685	cl14782	RNase_H_like superfamily	-
Q#2391 - >Ca_26089.1|sca	specific	254387	92	320	1.32807e-128	379.108	pfam07727	RVT_2	cl06662
Q#2391 - >Ca_26089.1|sca	superfamily	254387	92	320	1.32807e-128	379.108	cl06662	RVT_2 superfamily	-
Q#2399 - >Ca_26112.1|sca	superfamily	256046	1	66	3.92438e-06	43.9224	cl11168	PMD superfamily	-
Q#2401 - >Ca_26116.1|sca	superfamily	252133	71	170	6.26507e-11	57.3485	cl04237	Retrotrans_gag superfamily	-
Q#2405 - >Ca_26152.1|sca	superfamily	275927	57	266	2.80596e-26	104.652	cl11403	pepsin_retropepsin_like superfamily	-
Q#2408 - >Ca_26163.1|sca	specific	238825	623	753	4.51624e-62	207.448	cd01647	RT_LTR	cl02808
Q#2408 - >Ca_26163.1|sca	superfamily	261455	623	753	4.51624e-62	207.448	cl02808	RT_like superfamily	-
Q#2408 - >Ca_26163.1|sca	specific	133136	416	503	1.11908e-17	79.6879	cd00303	retropepsin_like	cl11403
Q#2408 - >Ca_26163.1|sca	superfamily	275927	416	503	1.11908e-17	79.6879	cl11403	pepsin_retropepsin_like superfamily	-
Q#2408 - >Ca_26163.1|sca	specific	252133	154	253	1.14707e-12	65.4377	pfam03732	Retrotrans_gag	cl04237
Q#2408 - >Ca_26163.1|sca	superfamily	252133	154	253	1.14707e-12	65.4377	cl04237	Retrotrans_gag superfamily	-
Q#2408 - >Ca_26163.1|sca	specific	189387	359	376	0.000990351	37.8189	pfam00098	zf-CCHC	cl22700
Q#2408 - >Ca_26163.1|sca	superfamily	276654	359	376	0.000990351	37.8189	cl22700	zf-CCHC superfamily	-
Q#2408 - >Ca_26163.1|sca	multi-dom	249567	640	744	2.65916e-18	83.9194	pfam00078	RVT_1	-
Q#2408 - >Ca_26163.1|sca	multi-dom	173561	341	420	9.96651e-09	54.4276	PTZ00368	PTZ00368	-
Q#2410 - >Ca_26166.1|sca	specific	255575	85	122	1.71592e-06	43.1941	pfam09811	Yae1_N	cl10784
Q#2410 - >Ca_26166.1|sca	superfamily	255575	85	122	1.71592e-06	43.1941	cl10784	Yae1_N superfamily	-
Q#2411 - >Ca_26170.1|sca	superfamily	276335	3	219	3.66059e-54	181.794	cl21512	Glycos_transf_1 superfamily	-
Q#2415 - >Ca_26183.1|sca	superfamily	261228	1	91	7.88538e-37	130.738	cl02262	UPF0261 superfamily	-
Q#2416 - >Ca_26184.1|sca	superfamily	251669	47	145	2.90941e-11	58.58	cl03830	Transposase_24 superfamily	-
Q#2417 - >Ca_26185.1|sca	superfamily	256046	1	85	0.00772976	36.2184	cl11168	PMD superfamily	-
Q#2420 - >Ca_26196.1|sca	specific	133136	362	449	1.46399e-17	79.6879	cd00303	retropepsin_like	cl11403
Q#2420 - >Ca_26196.1|sca	superfamily	275927	362	449	1.46399e-17	79.6879	cl11403	pepsin_retropepsin_like superfamily	-
Q#2420 - >Ca_26196.1|sca	specific	252133	100	199	1.75392e-12	65.0525	pfam03732	Retrotrans_gag	cl04237
Q#2420 - >Ca_26196.1|sca	superfamily	252133	100	199	1.75392e-12	65.0525	cl04237	Retrotrans_gag superfamily	-
Q#2420 - >Ca_26196.1|sca	specific	189387	305	322	0.000378053	39.3597	pfam00098	zf-CCHC	cl22700
Q#2420 - >Ca_26196.1|sca	superfamily	276654	305	322	0.000378053	39.3597	cl22700	zf-CCHC superfamily	-
Q#2420 - >Ca_26196.1|sca	multi-dom	173561	287	366	6.31536e-10	58.2796	PTZ00368	PTZ00368	-
Q#2423 - >Ca_26220.1|sca	specific	238825	495	629	1.94456e-49	174.706	cd01647	RT_LTR	cl02808
Q#2423 - >Ca_26220.1|sca	superfamily	261455	495	629	1.94456e-49	174.706	cl02808	RT_like superfamily	-
Q#2423 - >Ca_26220.1|sca	specific	260006	651	758	2.29292e-42	152.262	cd09274	RNase_HI_RT_Ty3	cl14782
Q#2423 - >Ca_26220.1|sca	superfamily	271754	651	758	2.29292e-42	152.262	cl14782	RNase_H_like superfamily	-
Q#2423 - >Ca_26220.1|sca	specific	133136	228	317	9.48406e-15	71.984	cd00303	retropepsin_like	cl11403

Query	Types	PSSM-ID	From	To	E-Value	Bitscore	Accession	Short name	Superfamily
Q#2423 - >Ca_26220.1|sca	superfamily	275927	228	317	9.48406e-15	71.984	cl11403	pepsin_retropepsin_like superfamily	-
Q#2423 - >Ca_26220.1|sca	specific	250040	887	1000	2.19972e-16	77.3736	pfam00665	rve	cl21549
Q#2423 - >Ca_26220.1|sca	superfamily	276355	887	1000	2.19972e-16	77.3736	cl21549	rve superfamily	-
Q#2423 - >Ca_26220.1|sca	superfamily	252133	22	58	0.000997008	38.8589	cl04237	Retrotrans_gag superfamily	-
Q#2423 - >Ca_26220.1|sca	multi-dom	249567	510	629	6.50462e-13	68.5114	pfam00078	RVT_1	-
Q#2424 - >Ca_26223.1|sca	specific	258478	1	148	2.33113e-56	175.45	pfam14299	PP2	cl16784
Q#2424 - >Ca_26223.1|sca	superfamily	258478	1	148	2.33113e-56	175.45	cl16784	PP2 superfamily	-
Q#2426 - >Ca_26232.1|sca	specific	260004	420	475	2.39782e-24	97.9241	cd09272	RNase_HI_RT_Ty1	cl14782
Q#2426 - >Ca_26232.1|sca	superfamily	271754	420	475	2.39782e-24	97.9241	cl14782	RNase_H_like superfamily	-
Q#2426 - >Ca_26232.1|sca	specific	258415	74	190	5.62728e-36	129.615	pfam14223	UBN2	cl15874
Q#2426 - >Ca_26232.1|sca	superfamily	265821	74	190	5.62728e-36	129.615	cl15874	UBN2 superfamily	-
Q#2426 - >Ca_26232.1|sca	superfamily	254387	352	412	4.29393e-29	114.091	cl06662	RVT_2 superfamily	-
Q#2429 - >Ca_26249.1|sca	specific	250720	21	154	2.05068e-39	132.798	pfam01582	TIR	cl22462
Q#2429 - >Ca_26249.1|sca	superfamily	250720	21	154	2.05068e-39	132.798	cl22462	TIR superfamily	-
Q#2430 - >Ca_26250.1|sca	superfamily	276192	31	65	8.54141e-13	60.2404	cl17169	RRM_SF superfamily	-
Q#2430 - >Ca_26250.1|sca	multi-dom	258445	32	95	0.00513043	32.9238	pfam14259	RRM_6	-
Q#2433 - >Ca_26266.1|sca	superfamily	252133	131	205	2.53625e-07	46.1777	cl04237	Retrotrans_gag superfamily	-
Q#2434 - >Ca_26267.1|sca	superfamily	251922	32	75	8.09427e-15	64.8664	cl04048	Ist1 superfamily	-
Q#2435 - >Ca_26280.1|sca	specific	253297	8	68	9.59881e-17	70.0526	pfam05641	Agenet	cl02573
Q#2435 - >Ca_26280.1|sca	superfamily	275771	8	68	9.59881e-17	70.0526	cl02573	TUDOR superfamily	-
Q#2435 - >Ca_26280.1|sca	superfamily	275771	80	133	4.2824e-06	40.7701	cl02573	TUDOR superfamily	-
Q#2436 - >Ca_26291.1|sca	specific	238213	48	106	1.54013e-17	71.7654	cd00353	Ribosomal_S15p_S13e	cl00349
Q#2436 - >Ca_26291.1|sca	superfamily	260373	48	106	1.54013e-17	71.7654	cl00349	S15_NS1_EPRS_RNA-bind superfamily	-
Q#2438 - >Ca_26301.1|sca	superfamily	263558	87	135	2.68701e-12	61.9023	cl08270	Peptidase_S10 superfamily	-
Q#2440 - >Ca_26314.1|sca	specific	133136	328	415	3.36922e-16	73.91	cd00303	retropepsin_like	cl11403
Q#2440 - >Ca_26314.1|sca	superfamily	275927	328	415	3.36922e-16	73.91	cl11403	pepsin_retropepsin_like superfamily	-
Q#2440 - >Ca_26314.1|sca	specific	252133	66	165	1.71091e-12	63.5117	pfam03732	Retrotrans_gag	cl04237
Q#2440 - >Ca_26314.1|sca	superfamily	252133	66	165	1.71091e-12	63.5117	cl04237	Retrotrans_gag superfamily	-
Q#2440 - >Ca_26314.1|sca	specific	189387	271	288	5.29682e-05	40.9005	pfam00098	zf-CCHC	cl22700
Q#2440 - >Ca_26314.1|sca	superfamily	276654	271	288	5.29682e-05	40.9005	cl22700	zf-CCHC superfamily	-
Q#2441 - >Ca_26315.1|sca	superfamily	276301	11	156	3.63549e-39	138.443	cl21457	TIM_phosphate_binding superfamily	-
Q#2443 - >Ca_26321.1|sca	superfamily	254387	84	169	4.28012e-33	118.713	cl06662	RVT_2 superfamily	-
Q#2445 - >Ca_26325.1|sca	multi-dom	234750	82	140	0.00311866	35.958	PRK00409	PRK00409	-
Q#2446 - >Ca_26327.1|sca	superfamily	242911	3	206	2.62099e-104	305.306	cl02160	Rcd1 superfamily	-
Q#2447 - >Ca_26329.1|sca	specific	258213	19	47	4.43549e-05	36.635	pfam13961	DUF4219	cl22382
Q#2447 - >Ca_26329.1|sca	superfamily	258213	19	47	4.43549e-05	36.635	cl22382	DUF4219 superfamily	-

Query	Types	PSSM-ID	From	To	E-Value	Bitscore	Accession	Short name	Superfamily
Q#2449 - >Ca_26336.1|sca	superfamily	191258	18	106	0.00301338	37.6974	cl05080	CSF-1 superfamily	-
Q#2450 - >Ca_26337.1|sca	multi-dom	237862	32	101	0.000600283	39.9461	PRK14948	PRK14948	-
Q#2452 - >Ca_26347.1|sca	superfamily	252133	89	158	6.5651e-05	38.4737	cl04237	Retrotrans_gag superfamily	-
Q#2453 - >Ca_26349.1|sca	specific	260004	24	114	2.13436e-38	128.355	cd09272	RNase_HI_RT_Ty1	cl14782
Q#2453 - >Ca_26349.1|sca	superfamily	271754	24	114	2.13436e-38	128.355	cl14782	RNase_H_like superfamily	-
Q#2455 - >Ca_26379.1|sca	superfamily	271848	2	206	1.258e-31	119.181	cl19241	Condensation superfamily	-
Q#2458 - >Ca_26382.1|sca	specific	238088	38	130	5.52874e-10	54.1189	cd00156	REC	cl19078
Q#2458 - >Ca_26382.1|sca	superfamily	271833	38	130	5.52874e-10	54.1189	cl19078	REC superfamily	-
Q#2458 - >Ca_26382.1|sca	superfamily	271918	169	221	1.47554e-13	62.8065	cl21498	SANT superfamily	-
Q#2459 - >Ca_26384.1|sca	superfamily	275476	17	86	1.58223e-23	88.9066	cl00155	UBQ superfamily	-
Q#2459 - >Ca_26384.1|sca	specific	251140	85	142	1.71367e-09	50.6827	pfam02179	BAG	cl02539
Q#2459 - >Ca_26384.1|sca	superfamily	261331	85	142	1.71367e-09	50.6827	cl02539	BAG superfamily	-
Q#2460 - >Ca_26387.1|sca	multi-dom	203922	14	134	0.00231799	35.5094	pfam08377	MAP2_projctn	-
Q#2464 - >Ca_26411.1|sca	specific	252958	117	218	5.5668e-27	102.495	pfam05033	Pre-SET	cl02622
Q#2464 - >Ca_26411.1|sca	superfamily	261377	117	218	5.5668e-27	102.495	cl02622	Pre-SET superfamily	-
Q#2464 - >Ca_26411.1|sca	specific	214614	226	296	2.70503e-13	65.0483	smart00317	SET	cl02566
Q#2464 - >Ca_26411.1|sca	superfamily	261347	226	296	2.70503e-13	65.0483	cl02566	SET superfamily	-
Q#2464 - >Ca_26411.1|sca	superfamily	261375	31	91	2.09722e-10	57.2625	cl02620	SAD_SRA superfamily	-
Q#2466 - >Ca_26420.1|sca	superfamily	254730	198	278	4.18102e-06	45.0021	cl07077	WLM superfamily	-
Q#2468 - >Ca_26430.1|sca	superfamily	271500	3	152	6.0312e-26	99.6748	cl00549	ABC_membrane superfamily	-
Q#2468 - >Ca_26430.1|sca	multi-dom	224055	2	152	5.01559e-18	79.7802	COG1132	MdlB	-
Q#2469 - >Ca_26432.1|sca	specific	176228	2	184	3.73335e-53	173.941	cd08267	MDR1	cl16912
Q#2469 - >Ca_26432.1|sca	superfamily	276140	2	184	3.73335e-53	173.941	cl16912	MDR superfamily	-
Q#2469 - >Ca_26432.1|sca	multi-dom	223677	6	171	2.77658e-27	105.122	COG0604	Qor	-
Q#2470 - >Ca_26433.1|sca	superfamily	265821	6	48	7.44703e-06	40.6337	cl15874	UBN2 superfamily	-
Q#2472 - >Ca_26439.1|sca	specific	260004	165	220	6.55365e-31	112.176	cd09272	RNase_HI_RT_Ty1	cl14782
Q#2472 - >Ca_26439.1|sca	superfamily	271754	165	220	6.55365e-31	112.176	cl14782	RNase_H_like superfamily	-
Q#2473 - >Ca_26440.1|sca	specific	250692	169	198	2.80387e-05	39.3593	pfam01535	PPR	cl03252
Q#2473 - >Ca_26440.1|sca	superfamily	275791	169	198	2.80387e-05	39.3593	cl03252	PPR superfamily	-
Q#2473 - >Ca_26440.1|sca	specific	250692	68	98	5.74238e-05	38.5889	pfam01535	PPR	cl03252
Q#2473 - >Ca_26440.1|sca	superfamily	275791	68	98	5.74238e-05	38.5889	cl03252	PPR superfamily	-
Q#2477 - >Ca_26451.1|sca	superfamily	252344	100	149	1.22697e-08	52.6354	cl04384	DUF383 superfamily	-
Q#2477 - >Ca_26451.1|sca	specific	250181	213	262	5.25194e-06	43.6411	pfam00856	SET	cl02566
Q#2477 - >Ca_26451.1|sca	superfamily	261347	213	262	5.25194e-06	43.6411	cl02566	SET superfamily	-
Q#2480 - >Ca_26480.1|sca	multi-dom	165564	282	441	3.61129e-05	45.2298	PHA03309	PHA03309	-
Q#2481 - >Ca_26486.1|sca	superfamily	272138	7	80	3.21147e-27	103.802	cl21718	RNA_pol_B_RPB2 superfamily	-

Query	Types	PSSM-ID	From	To	E-Value	Bitscore	Accession	Short name	Superfamily
Q#2483 - >Ca_26504.1|sca	specific	202502	175	397	1.64462e-118	352.719	pfam02992	Transposase_21	cl03823
Q#2483 - >Ca_26504.1|sca	superfamily	202502	175	397	1.64462e-118	352.719	cl03823	Transposase_21 superfamily	-
Q#2483 - >Ca_26504.1|sca	superfamily	206130	534	568	7.30661e-05	41.4203	cl16501	DUF4218 superfamily	-
Q#2489 - >Ca_26522.1|sca	superfamily	253459	43	83	1.33684e-07	46.9961	cl05469	Mitovir_RNA_pol superfamily	-
Q#2491 - >Ca_26527.1|sca	specific	250208	21	253	1.48066e-78	239.844	pfam00891	Methyltransf_2	cl17173
Q#2491 - >Ca_26527.1|sca	superfamily	276194	21	253	1.48066e-78	239.844	cl17173	AdoMet_MTases superfamily	-
Q#2493 - >Ca_26536.1|sca	specific	238093	128	175	1.39723e-08	47.8256	cd00162	RING	cl17238
Q#2493 - >Ca_26536.1|sca	superfamily	276201	128	175	1.39723e-08	47.8256	cl17238	RING superfamily	-
Q#2493 - >Ca_26536.1|sca	multi-dom	257215	128	172	9.32542e-10	51.6687	pfam12678	zf-rbx1	-
Q#2497 - >Ca_26542.1|sca	specific	260004	86	224	2.18716e-76	230.048	cd09272	RNase_HI_RT_Ty1	cl14782
Q#2497 - >Ca_26542.1|sca	superfamily	271754	86	224	2.18716e-76	230.048	cl14782	RNase_H_like superfamily	-
Q#2498 - >Ca_26543.1|sca	superfamily	252133	97	171	3.5442e-08	47.7185	cl04237	Retrotrans_gag superfamily	-
Q#2499 - >Ca_26546.1|sca	superfamily	276020	1	181	2.60172e-60	197.341	cl12078	p450 superfamily	-
Q#2503 - >Ca_26568.1|sca	superfamily	254387	118	247	9.9855e-80	242.362	cl06662	RVT_2 superfamily	-
Q#2504 - >Ca_26569.1|sca	superfamily	256046	3	81	8.62494e-15	67.0344	cl11168	PMD superfamily	-
Q#2508 - >Ca_26578.1|sca	specific	260004	434	542	8.85622e-46	161.482	cd09272	RNase_HI_RT_Ty1	cl14782
Q#2508 - >Ca_26578.1|sca	superfamily	271754	434	542	8.85622e-46	161.482	cl14782	RNase_H_like superfamily	-
Q#2508 - >Ca_26578.1|sca	specific	254387	107	352	4.4693e-135	406.457	pfam07727	RVT_2	cl06662
Q#2508 - >Ca_26578.1|sca	superfamily	254387	107	352	4.4693e-135	406.457	cl06662	RVT_2 superfamily	-
Q#2509 - >Ca_26579.1|sca	specific	273253	176	210	2.14633e-07	46.6795	TIGR00756	PPR	cl03252
Q#2509 - >Ca_26579.1|sca	superfamily	275791	176	210	2.14633e-07	46.6795	cl03252	PPR superfamily	-
Q#2509 - >Ca_26579.1|sca	specific	250692	74	103	8.06898e-05	39.3593	pfam01535	PPR	cl03252
Q#2509 - >Ca_26579.1|sca	superfamily	275791	74	103	8.06898e-05	39.3593	cl03252	PPR superfamily	-
Q#2509 - >Ca_26579.1|sca	multi-dom	257477	143	185	0.0011981	36.2266	pfam13041	PPR_2	-
Q#2509 - >Ca_26579.1|sca	multi-dom	257477	175	220	9.42368e-09	50.8642	pfam13041	PPR_2	-
Q#2515 - >Ca_26595.1|sca	superfamily	252133	100	174	3.36241e-10	55.8077	cl04237	Retrotrans_gag superfamily	-
Q#2515 - >Ca_26595.1|sca	specific	189387	264	280	0.00164553	35.5077	pfam00098	zf-CCHC	cl22700
Q#2515 - >Ca_26595.1|sca	superfamily	276654	264	280	0.00164553	35.5077	cl22700	zf-CCHC superfamily	-
Q#2519 - >Ca_26603.1|sca	superfamily	271411	1	114	6.17901e-38	134.725	cl00042	CASc superfamily	-
Q#2523 - >Ca_26626.1|sca	specific	238825	254	408	4.99393e-85	259.45	cd01647	RT_LTR	cl02808
Q#2523 - >Ca_26626.1|sca	superfamily	261455	254	408	4.99393e-85	259.45	cl02808	RT_like superfamily	-
Q#2523 - >Ca_26626.1|sca	specific	133136	47	134	1.14513e-16	74.6804	cd00303	retropepsin_like	cl11403
Q#2523 - >Ca_26626.1|sca	superfamily	275927	47	134	1.14513e-16	74.6804	cl11403	pepsin_retropepsin_like superfamily	-
Q#2523 - >Ca_26626.1|sca	multi-dom	249567	271	406	3.71197e-30	115.121	pfam00078	RVT_1	-
Q#2525 - >Ca_26628.1|sca	superfamily	252133	1	94	5.60437e-12	58.1189	cl04237	Retrotrans_gag superfamily	-
Q#2528 - >Ca_26635.1|sca	specific	260004	79	217	2.05125e-78	234.67	cd09272	RNase_HI_RT_Ty1	cl14782

Query	Types	PSSM-ID	From	To	E-Value	Bitscore	Accession	Short name	Superfamily
Q#2528 - >Ca_26635.1|sca	superfamily	271754	79	217	2.05125e-78	234.67	cl14782	RNase_H_like superfamily	-
Q#2529 - >Ca_26651.1|sca	specific	238875	1	258	8.05838e-89	269.485	cd01837	SGNH_plant_lipase_like	cl01053
Q#2529 - >Ca_26651.1|sca	superfamily	260768	1	258	8.05838e-89	269.485	cl01053	SGNH_hydrolase superfamily	-
Q#2532 - >Ca_26661.1|sca	specific	260004	59	173	5.44216e-54	182.668	cd09272	RNase_HI_RT_Ty1	cl14782
Q#2532 - >Ca_26661.1|sca	superfamily	271754	59	173	5.44216e-54	182.668	cl14782	RNase_H_like superfamily	-
Q#2533 - >Ca_26662.1|sca	superfamily	271701	1	40	4.35736e-12	61.46	cl10459	Peptidases_S8_S53 superfamily	-
Q#2535 - >Ca_26678.1|sca	superfamily	256046	3	59	1.29365e-12	62.412	cl11168	PMD superfamily	-
Q#2536 - >Ca_26680.1|sca	superfamily	249757	297	414	1.79157e-36	140.267	cl09510	Lipoxygenase superfamily	-
Q#2537 - >Ca_26681.1|sca	superfamily	276326	33	57	1.89722e-05	41.0005	cl21494	Esterase_lipase superfamily	-
Q#2537 - >Ca_26681.1|sca	multi-dom	215216	2	233	9.40043e-108	317.079	PLN02385	PLN02385	-
Q#2538 - >Ca_26682.1|sca	specific	238083	17	156	9.89483e-25	98.1674	cd00143	PP2Cc	cl00120
Q#2538 - >Ca_26682.1|sca	superfamily	260203	17	156	9.89483e-25	98.1674	cl00120	PP2Cc superfamily	-
Q#2539 - >Ca_26688.1|sca	superfamily	276192	9	78	4.11539e-27	95.9179	cl17169	RRM_SF superfamily	-
Q#2539 - >Ca_26688.1|sca	multi-dom	214636	9	78	2.45672e-24	88.8047	smart00360	RRM	-
Q#2540 - >Ca_26691.1|sca	specific	260004	136	269	8.47877e-72	230.433	cd09272	RNase_HI_RT_Ty1	cl14782
Q#2540 - >Ca_26691.1|sca	superfamily	271754	136	269	8.47877e-72	230.433	cl14782	RNase_H_like superfamily	-
Q#2540 - >Ca_26691.1|sca	superfamily	254387	1	99	2.96967e-56	192.286	cl06662	RVT_2 superfamily	-
Q#2543 - >Ca_26712.1|sca	superfamily	202502	154	181	5.34265e-10	57.6558	cl03823	Transposase_21 superfamily	-
Q#2544 - >Ca_26713.1|sca	superfamily	252133	73	172	8.87429e-12	60.4301	cl04237	Retrotrans_gag superfamily	-
Q#2544 - >Ca_26713.1|sca	specific	189387	250	265	0.000114591	38.9745	pfam00098	zf-CCHC	cl22700
Q#2544 - >Ca_26713.1|sca	superfamily	276654	250	265	0.000114591	38.9745	cl22700	zf-CCHC superfamily	-
Q#2547 - >Ca_26721.1|sca	specific	252133	100	199	3.12811e-13	63.8969	pfam03732	Retrotrans_gag	cl04237
Q#2547 - >Ca_26721.1|sca	superfamily	252133	100	199	3.12811e-13	63.8969	cl04237	Retrotrans_gag superfamily	-
Q#2549 - >Ca_26727.1|sca	specific	206651	22	220	3.1041e-79	241.285	cd01852	AIG1	cl21455
Q#2549 - >Ca_26727.1|sca	superfamily	276299	22	220	3.1041e-79	241.285	cl21455	ABC_ATPase superfamily	-
Q#2554 - >Ca_26754.1|sca	specific	238166	3	85	2.13116e-26	97.3307	cd00266	MADS_SRF_like	cl00109
Q#2554 - >Ca_26754.1|sca	superfamily	241616	3	85	2.13116e-26	97.3307	cl00109	MADS superfamily	-
Q#2555 - >Ca_26755.1|sca	specific	238166	3	85	2.11753e-29	105.805	cd00266	MADS_SRF_like	cl00109
Q#2555 - >Ca_26755.1|sca	superfamily	241616	3	85	2.11753e-29	105.805	cl00109	MADS superfamily	-
Q#2556 - >Ca_26761.1|sca	multi-dom	223021	203	349	7.23266e-05	44.1589	PHA03247	PHA03247	-
Q#2557 - >Ca_26770.1|sca	superfamily	275633	1	87	1.27062e-17	75.8907	cl00600	Ribosomal_L7Ae superfamily	-
Q#2558 - >Ca_26772.1|sca	superfamily	254533	6	53	0.006492	36.8522	cl20352	DUF1682 superfamily	-
Q#2559 - >Ca_26784.1|sca	superfamily	271701	85	144	7.08635e-17	77.2532	cl10459	Peptidases_S8_S53 superfamily	-
Q#2561 - >Ca_26794.1|sca	superfamily	190526	94	133	2.5924e-05	38.3526	cl07846	DBD_Tnp_Mut superfamily	-
Q#2562 - >Ca_26796.1|sca	superfamily	276263	129	193	8.48414e-07	46.9688	cl19514	FBA_1 superfamily	-
Q#2563 - >Ca_26797.1|sca	superfamily	252133	81	174	7.05988e-11	56.1929	cl04237	Retrotrans_gag superfamily	-

Query	Types	PSSM-ID	From	To	E-Value	Bitscore	Accession	Short name	Superfamily
Q#2565 - >Ca_26805.1|sca	multi-dom	223533	8	190	0.000238961	39.8293	COG0457	NrfG	-
Q#2571 - >Ca_26822.1|sca	superfamily	147759	217	361	0.00423839	38.0672	cl21892	ECM1 superfamily	-
Q#2573 - >Ca_26834.1|sca	superfamily	261455	155	216	3.60691e-12	61.98	cl02808	RT_like superfamily	-
Q#2575 - >Ca_26839.1|sca	multi-dom	223079	160	212	0.00403141	36.4646	PHA03419	PHA03419	-
Q#2577 - >Ca_26853.1|sca	specific	257351	167	200	3.62894e-09	50.8109	pfam12854	PPR_1	cl03252
Q#2577 - >Ca_26853.1|sca	superfamily	275791	167	200	3.62894e-09	50.8109	cl03252	PPR superfamily	-
Q#2577 - >Ca_26853.1|sca	specific	257351	39	70	3.23353e-07	45.8033	pfam12854	PPR_1	cl03252
Q#2577 - >Ca_26853.1|sca	superfamily	275791	39	70	3.23353e-07	45.8033	cl03252	PPR superfamily	-
Q#2577 - >Ca_26853.1|sca	specific	257351	132	164	5.09825e-06	42.3365	pfam12854	PPR_1	cl03252
Q#2577 - >Ca_26853.1|sca	superfamily	275791	132	164	5.09825e-06	42.3365	cl03252	PPR superfamily	-
Q#2577 - >Ca_26853.1|sca	specific	257351	202	233	3.75385e-05	40.0253	pfam12854	PPR_1	cl03252
Q#2577 - >Ca_26853.1|sca	superfamily	275791	202	233	3.75385e-05	40.0253	cl03252	PPR superfamily	-
Q#2577 - >Ca_26853.1|sca	specific	273253	11	45	0.00045247	36.6643	TIGR00756	PPR	cl03252
Q#2577 - >Ca_26853.1|sca	superfamily	275791	11	45	0.00045247	36.6643	cl03252	PPR superfamily	-
Q#2577 - >Ca_26853.1|sca	multi-dom	257477	136	185	2.79293e-17	73.2058	pfam13041	PPR_2	-
Q#2577 - >Ca_26853.1|sca	multi-dom	257477	171	219	4.83678e-15	67.0426	pfam13041	PPR_2	-
Q#2577 - >Ca_26853.1|sca	multi-dom	257477	207	254	1.05162e-14	66.2722	pfam13041	PPR_2	-
Q#2577 - >Ca_26853.1|sca	multi-dom	257477	8	56	2.04899e-12	60.109	pfam13041	PPR_2	-
Q#2577 - >Ca_26853.1|sca	multi-dom	257477	79	149	1.4543e-10	55.1014	pfam13041	PPR_2	-
Q#2578 - >Ca_26860.1|sca	specific	211390	95	209	1.10561e-15	70.6922	cd11378	DUF296	cl00720
Q#2578 - >Ca_26860.1|sca	superfamily	260588	95	209	1.10561e-15	70.6922	cl00720	DUF296 superfamily	-
Q#2579 - >Ca_26862.1|sca	superfamily	256046	9	145	9.29148e-21	85.524	cl11168	PMD superfamily	-
Q#2581 - >Ca_26870.1|sca	multi-dom	223021	107	265	2.84966e-05	44.9293	PHA03247	PHA03247	-
Q#2584 - >Ca_26883.1|sca	specific	258415	104	223	3.68623e-34	127.689	pfam14223	UBN2	cl15874
Q#2584 - >Ca_26883.1|sca	superfamily	265821	104	223	3.68623e-34	127.689	cl15874	UBN2 superfamily	-
Q#2584 - >Ca_26883.1|sca	specific	258213	19	47	0.000789178	38.1758	pfam13961	DUF4219	cl22382
Q#2584 - >Ca_26883.1|sca	superfamily	258213	19	47	0.000789178	38.1758	cl22382	DUF4219 superfamily	-
Q#2584 - >Ca_26883.1|sca	specific	189387	294	311	0.00619724	35.8929	pfam00098	zf-CCHC	cl22700
Q#2584 - >Ca_26883.1|sca	superfamily	276654	294	311	0.00619724	35.8929	cl22700	zf-CCHC superfamily	-
Q#2585 - >Ca_26885.1|sca	multi-dom	273731	34	220	2.74165e-32	123.976	TIGR01647	Plasma_membrane_ATPase	-
Q#2587 - >Ca_26895.1|sca	superfamily	275753	68	281	1.94318e-82	253.303	cl01911	Pectinesterase superfamily	-
Q#2589 - >Ca_26903.1|sca	superfamily	275485	1	71	0.00796187	37.1745	cl00210	Isoprenoid_Biosyn_C1 superfamily	-
Q#2593 - >Ca_26922.1|sca	specific	252648	160	284	1.59869e-38	134.085	pfam04520	Senescence_reg	cl04561
Q#2593 - >Ca_26922.1|sca	superfamily	252648	160	284	1.59869e-38	134.085	cl04561	Senescence_reg superfamily	-
Q#2593 - >Ca_26922.1|sca	superfamily	259367	65	121	0.00874688	34.8403	cl21095	SYCE1 superfamily	-
Q#2594 - >Ca_26924.1|sca	specific	240687	17	90	1.63356e-46	146.607	cd12241	RRM_SF3B14	cl17169

Query	Types	PSSM-ID	From	To	E-Value	Bitscore	Accession	Short name	Superfamily
Q#2594 - >Ca_26924.1|sca	superfamily	276192	17	90	1.63356e-46	146.607	cl17169	RRM_SF superfamily	-
Q#2594 - >Ca_26924.1|sca	multi-dom	214636	20	86	1.09533e-16	69.5447	smart00360	RRM	-
Q#2596 - >Ca_26932.1|sca	superfamily	256046	4	171	2.94281e-54	177.587	cl11168	PMD superfamily	-
Q#2598 - >Ca_26938.1|sca	specific	238825	257	406	1.48246e-79	247.509	cd01647	RT_LTR	cl02808
Q#2598 - >Ca_26938.1|sca	superfamily	261455	257	406	1.48246e-79	247.509	cl02808	RT_like superfamily	-
Q#2598 - >Ca_26938.1|sca	specific	133136	50	137	2.3387e-16	74.6804	cd00303	retropepsin_like	cl11403
Q#2598 - >Ca_26938.1|sca	superfamily	275927	50	137	2.3387e-16	74.6804	cl11403	pepsin_retropepsin_like superfamily	-
Q#2598 - >Ca_26938.1|sca	multi-dom	249567	274	409	7.06969e-29	112.039	pfam00078	RVT_1	-
Q#2599 - >Ca_26939.1|sca	superfamily	252133	66	165	1.14986e-10	56.1929	cl04237	Retrotrans_gag superfamily	-
Q#2600 - >Ca_26940.1|sca	superfamily	275485	36	105	0.00675498	36.4041	cl00210	Isoprenoid_Biosyn_C1 superfamily	-
Q#2602 - >Ca_26948.1|sca	superfamily	276298	70	150	2.41831e-32	116.779	cl21454	SDR superfamily	-
Q#2602 - >Ca_26948.1|sca	superfamily	261368	1	63	4.22797e-23	89.0408	cl02608	BAH superfamily	-
Q#2603 - >Ca_26950.1|sca	specific	100107	11	90	7.0088e-07	44.3431	cd02440	AdoMet_MTases	cl17173
Q#2603 - >Ca_26950.1|sca	superfamily	276194	11	90	7.0088e-07	44.3431	cl17173	AdoMet_MTases superfamily	-
Q#2604 - >Ca_26951.1|sca	multi-dom	223021	35	225	0.00241462	39.9217	PHA03247	PHA03247	-
Q#2605 - >Ca_26959.1|sca	specific	260004	215	353	4.13687e-76	244.3	cd09272	RNase_HI_RT_Ty1	cl14782
Q#2605 - >Ca_26959.1|sca	superfamily	271754	215	353	4.13687e-76	244.3	cl14782	RNase_H_like superfamily	-
Q#2605 - >Ca_26959.1|sca	superfamily	254387	79	176	1.02168e-51	180.73	cl06662	RVT_2 superfamily	-
Q#2606 - >Ca_26978.1|sca	superfamily	252133	100	199	1.81007e-12	60.4301	cl04237	Retrotrans_gag superfamily	-
Q#2607 - >Ca_26983.1|sca	specific	249634	44	143	1.84425e-18	76.1339	pfam00153	Mito_carr	cl02813
Q#2607 - >Ca_26983.1|sca	superfamily	249634	44	143	1.84425e-18	76.1339	cl02813	Mito_carr superfamily	-
Q#2611 - >Ca_27002.1|sca	specific	260004	39	90	2.77563e-21	89.4497	cd09272	RNase_HI_RT_Ty1	cl14782
Q#2611 - >Ca_27002.1|sca	superfamily	271754	39	90	2.77563e-21	89.4497	cl14782	RNase_H_like superfamily	-
Q#2615 - >Ca_27018.1|sca	superfamily	268115	42	70	1.55357e-08	48.8329	cl19762	RNB superfamily	-
Q#2616 - >Ca_27024.1|sca	multi-dom	183854	290	378	0.000239386	41.9273	PRK13042	PRK13042	-
Q#2622 - >Ca_27040.1|sca	specific	239089	172	237	2.49557e-18	76.7463	cd02396	PCBP_like_KH	cl00098
Q#2622 - >Ca_27040.1|sca	superfamily	275471	172	237	2.49557e-18	76.7463	cl00098	KH-I superfamily	-
Q#2622 - >Ca_27040.1|sca	superfamily	275507	39	104	6.30546e-33	123.464	cl00268	class_II_aaRS-like_core superfamily	-
Q#2622 - >Ca_27040.1|sca	superfamily	275471	258	278	0.00151585	35.53	cl00098	KH-I superfamily	-
Q#2623 - >Ca_27045.1|sca	specific	249667	59	210	1.00414e-34	122.002	pfam00190	Cupin_1	cl21464
Q#2623 - >Ca_27045.1|sca	superfamily	276307	59	210	1.00414e-34	122.002	cl21464	ABD superfamily	-
Q#2625 - >Ca_27066.1|sca	superfamily	275868	141	189	5.783e-05	39.3262	cl09141	ACT superfamily	-
Q#2625 - >Ca_27066.1|sca	superfamily	275868	29	90	0.00134827	35.4035	cl09141	ACT superfamily	-
Q#2626 - >Ca_27067.1|sca	specific	251346	8	63	8.10264e-08	44.8563	pfam02517	Abi	cl00558
Q#2626 - >Ca_27067.1|sca	superfamily	271502	8	63	8.10264e-08	44.8563	cl00558	Abi superfamily	-
Q#2628 - >Ca_27072.1|sca	superfamily	276335	5	245	5.3973e-61	200.234	cl21512	Glycos_transf_1 superfamily	-

Query	Types	PSSM-ID	From	To	E-Value	Bitscore	Accession	Short name	Superfamily
Q#2631 - >Ca_27085.1|sca	superfamily	276306	5	53	1.37768e-20	78.6372	cl21463	UBA_like_SF superfamily	-
Q#2632 - >Ca_27091.1|sca	superfamily	244811	5	188	1.81177e-111	324.885	cl07847	RGP superfamily	-
Q#2636 - >Ca_27105.1|sca	specific	273253	122	155	4.98853e-06	44.3683	TIGR00756	PPR	cl03252
Q#2636 - >Ca_27105.1|sca	superfamily	275791	122	155	4.98853e-06	44.3683	cl03252	PPR superfamily	-
Q#2636 - >Ca_27105.1|sca	specific	273253	444	477	4.98853e-06	44.3683	TIGR00756	PPR	cl03252
Q#2636 - >Ca_27105.1|sca	superfamily	275791	444	477	4.98853e-06	44.3683	cl03252	PPR superfamily	-
Q#2636 - >Ca_27105.1|sca	multi-dom	215561	91	658	1.16044e-70	246.685	PLN03077	PLN03077	-
Q#2643 - >Ca_27141.1|sca	superfamily	276263	119	273	3.26073e-14	69.3104	cl19514	FBA_1 superfamily	-
Q#2644 - >Ca_27143.1|sca	superfamily	268118	2	255	1.22324e-14	71.7123	cl19765	mTERF superfamily	-
Q#2650 - >Ca_27167.1|sca	superfamily	253337	6	55	7.85663e-13	60.9973	cl05327	Auxin_canalis superfamily	-
Q#2654 - >Ca_27177.1|sca	specific	256046	41	278	2.26673e-64	214.951	pfam10536	PMD	cl11168
Q#2654 - >Ca_27177.1|sca	superfamily	256046	41	278	2.26673e-64	214.951	cl11168	PMD superfamily	-
Q#2657 - >Ca_27181.1|sca	specific	189387	290	306	6.89262e-05	39.7449	pfam00098	zf-CCHC	cl22700
Q#2657 - >Ca_27181.1|sca	superfamily	276654	290	306	6.89262e-05	39.7449	cl22700	zf-CCHC superfamily	-
Q#2657 - >Ca_27181.1|sca	specific	197667	270	286	0.00523381	34.3412	smart00343	ZnF_C2HC	cl22700
Q#2657 - >Ca_27181.1|sca	superfamily	276654	270	286	0.00523381	34.3412	cl22700	zf-CCHC superfamily	-
Q#2658 - >Ca_27182.1|sca	superfamily	252133	72	158	4.01006e-07	46.1777	cl04237	Retrotrans_gag superfamily	-
Q#2661 - >Ca_27190.1|sca	superfamily	260768	34	88	1.47296e-28	105.005	cl01053	SGNH_hydrolase superfamily	-
Q#2668 - >Ca_27231.1|sca	specific	189387	227	244	0.000516225	37.0485	pfam00098	zf-CCHC	cl22700
Q#2668 - >Ca_27231.1|sca	superfamily	276654	227	244	0.000516225	37.0485	cl22700	zf-CCHC superfamily	-
Q#2668 - >Ca_27231.1|sca	superfamily	252133	96	140	0.000796355	36.9329	cl04237	Retrotrans_gag superfamily	-
Q#2670 - >Ca_27236.1|sca	superfamily	276298	1	130	2.36276e-58	185.83	cl21454	SDR superfamily	-
Q#2673 - >Ca_27239.1|sca	superfamily	252133	118	165	0.000625595	36.9329	cl04237	Retrotrans_gag superfamily	-
Q#2675 - >Ca_27246.1|sca	specific	260004	247	385	5.9884e-75	231.974	cd09272	RNase_HI_RT_Ty1	cl14782
Q#2675 - >Ca_27246.1|sca	superfamily	271754	247	385	5.9884e-75	231.974	cl14782	RNase_H_like superfamily	-
Q#2675 - >Ca_27246.1|sca	superfamily	254387	1	165	3.39468e-84	259.696	cl06662	RVT_2 superfamily	-
Q#2679 - >Ca_27252.1|sca	superfamily	271848	1	205	6.6869e-23	98.2272	cl19241	Condensation superfamily	-
Q#2679 - >Ca_27252.1|sca	superfamily	271848	248	382	4.21228e-20	90.138	cl19241	Condensation superfamily	-
Q#2681 - >Ca_27260.1|sca	multi-dom	251763	371	560	3.01296e-05	45.8336	pfam03154	Atrophin-1	-
Q#2683 - >Ca_27263.1|sca	superfamily	264349	72	136	8.21122e-19	80.8452	cl11396	Patatin_and_cPLA2 superfamily	-
Q#2686 - >Ca_27289.1|sca	specific	252133	100	199	2.20795e-13	63.8969	pfam03732	Retrotrans_gag	cl04237
Q#2686 - >Ca_27289.1|sca	superfamily	252133	100	199	2.20795e-13	63.8969	cl04237	Retrotrans_gag superfamily	-
Q#2690 - >Ca_27298.1|sca	multi-dom	178748	206	412	0.00114448	39.9095	PLN03209	PLN03209	-
Q#2692 - >Ca_27310.1|sca	superfamily	252133	79	177	9.42165e-12	58.5041	cl04237	Retrotrans_gag superfamily	-
Q#2697 - >Ca_27338.1|sca	specific	260004	64	202	6.14599e-73	233.514	cd09272	RNase_HI_RT_Ty1	cl14782
Q#2697 - >Ca_27338.1|sca	superfamily	271754	64	202	6.14599e-73	233.514	cl14782	RNase_H_like superfamily	-

Query	Types	PSSM-ID	From	To	E-Value	Bitscore	Accession	Short name	Superfamily
Q#2699 - >Ca_27345.1|sca	specific	258415	1	89	1.68318e-25	97.6432	pfam14223	UBN2	cl15874
Q#2699 - >Ca_27345.1|sca	superfamily	265821	1	89	1.68318e-25	97.6432	cl15874	UBN2 superfamily	-
Q#2699 - >Ca_27345.1|sca	specific	258415	70	163	9.60311e-22	87.2428	pfam14223	UBN2	cl15874
Q#2699 - >Ca_27345.1|sca	superfamily	265821	70	163	9.60311e-22	87.2428	cl15874	UBN2 superfamily	-
Q#2700 - >Ca_27346.1|sca	specific	133136	300	387	1.59051e-16	75.4508	cd00303	retropepsin_like	cl11403
Q#2700 - >Ca_27346.1|sca	superfamily	275927	300	387	1.59051e-16	75.4508	cl11403	pepsin_retropepsin_like superfamily	-
Q#2700 - >Ca_27346.1|sca	superfamily	252133	65	164	6.70612e-11	59.2745	cl04237	Retrotrans_gag superfamily	-
Q#2700 - >Ca_27346.1|sca	specific	189387	244	259	0.00102281	37.4337	pfam00098	zf-CCHC	cl22700
Q#2700 - >Ca_27346.1|sca	superfamily	276654	244	259	0.00102281	37.4337	cl22700	zf-CCHC superfamily	-
Q#2700 - >Ca_27346.1|sca	specific	197667	224	239	0.00919433	34.7264	smart00343	ZnF_C2HC	cl22700
Q#2700 - >Ca_27346.1|sca	superfamily	276654	224	239	0.00919433	34.7264	cl22700	zf-CCHC superfamily	-
Q#2703 - >Ca_27350.1|sca	superfamily	252884	217	289	0.00112128	40.421	cl15563	Herpes_DNAp_acc superfamily	-
Q#2706 - >Ca_27360.1|sca	superfamily	275737	4	27	0.000926681	36.7557	cl01629	TPP_enzymes superfamily	-
Q#2708 - >Ca_27369.1|sca	multi-dom	223021	225	323	2.62841e-05	45.3145	PHA03247	PHA03247	-
Q#2711 - >Ca_27375.1|sca	superfamily	252133	100	199	2.83954e-10	55.0373	cl04237	Retrotrans_gag superfamily	-
Q#2712 - >Ca_27379.1|sca	superfamily	271660	1	123	8.52118e-26	101.11	cl09326	MATE_like superfamily	-
Q#2713 - >Ca_27381.1|sca	multi-dom	215556	1	117	2.80185e-39	139.932	PLN03064	PLN03064	-
Q#2721 - >Ca_27408.1|sca	superfamily	253639	84	193	3.07252e-17	79.3688	cl20322	Zw10 superfamily	-
Q#2726 - >Ca_27426.1|sca	specific	133136	444	501	5.36502e-06	44.6348	cd00303	retropepsin_like	cl11403
Q#2726 - >Ca_27426.1|sca	superfamily	275927	444	501	5.36502e-06	44.6348	cl11403	pepsin_retropepsin_like superfamily	-
Q#2726 - >Ca_27426.1|sca	specific	252133	100	199	3.16596e-13	66.2081	pfam03732	Retrotrans_gag	cl04237
Q#2726 - >Ca_27426.1|sca	superfamily	252133	100	199	3.16596e-13	66.2081	cl04237	Retrotrans_gag superfamily	-
Q#2726 - >Ca_27426.1|sca	specific	189387	306	320	0.000286042	38.9745	pfam00098	zf-CCHC	cl22700
Q#2726 - >Ca_27426.1|sca	superfamily	276654	306	320	0.000286042	38.9745	cl22700	zf-CCHC superfamily	-
Q#2729 - >Ca_27440.1|sca	multi-dom	223021	140	221	0.000218542	41.4625	PHA03247	PHA03247	-
Q#2729 - >Ca_27440.1|sca	multi-dom	237940	187	279	0.00574238	37.0858	PRK15313	PRK15313	-
Q#2736 - >Ca_27471.1|sca	specific	252133	102	201	2.41089e-13	62.7413	pfam03732	Retrotrans_gag	cl04237
Q#2736 - >Ca_27471.1|sca	superfamily	252133	102	201	2.41089e-13	62.7413	cl04237	Retrotrans_gag superfamily	-
Q#2738 - >Ca_27476.1|sca	superfamily	275753	3	177	1.26664e-57	184.738	cl01911	Pectinesterase superfamily	-
Q#2739 - >Ca_27477.1|sca	superfamily	255867	225	316	1.66601e-16	75.856	cl10951	Tmemb_185A superfamily	-
Q#2743 - >Ca_27495.1|sca	superfamily	276298	1	66	4.81569e-18	74.5631	cl21454	SDR superfamily	-
Q#2744 - >Ca_27496.1|sca	specific	275442	58	199	1.20685e-17	76.3229	cd15798	PMEI-like_3	cl04375
Q#2744 - >Ca_27496.1|sca	superfamily	275808	58	199	1.20685e-17	76.3229	cl04375	PMEI_like superfamily	-
Q#2745 - >Ca_27497.1|sca	multi-dom	223021	216	345	0.00175602	39.5365	PHA03247	PHA03247	-
Q#2748 - >Ca_27510.1|sca	superfamily	275485	43	132	0.00064773	40.2561	cl00210	Isoprenoid_Biosyn_C1 superfamily	-
Q#2752 - >Ca_27524.1|sca	specific	251717	119	295	2.69564e-60	194.084	pfam03080	DUF239	cl03877

Query	Types	PSSM-ID	From	To	E-Value	Bitscore	Accession	Short name	Superfamily
Q#2752 - >Ca_27524.1|sca	superfamily	251717	119	295	2.69564e-60	194.084	cl03877	DUF239 superfamily	-
Q#2752 - >Ca_27524.1|sca	specific	206533	11	112	6.52902e-28	105.434	pfam14365	DUF4409	cl16830
Q#2752 - >Ca_27524.1|sca	superfamily	206533	11	112	6.52902e-28	105.434	cl16830	DUF4409 superfamily	-
Q#2757 - >Ca_27546.1|sca	multi-dom	257477	8	88	8.15171e-05	37.7674	pfam13041	PPR_2	-
Q#2757 - >Ca_27546.1|sca	multi-dom	257477	114	146	0.00392421	33.145	pfam13041	PPR_2	-
Q#2757 - >Ca_27546.1|sca	multi-dom	257477	81	126	0.00542816	32.7598	pfam13041	PPR_2	-
Q#2758 - >Ca_27553.1|sca	superfamily	252133	100	199	3.40139e-12	59.6597	cl04237	Retrotrans_gag superfamily	-
Q#2759 - >Ca_27554.1|sca	superfamily	261306	25	78	8.30617e-06	38.7389	cl02489	SWIB superfamily	-
Q#2760 - >Ca_27559.1|sca	superfamily	271343	177	241	2.01288e-12	63.0789	cl22423	NBR1_like superfamily	-
Q#2760 - >Ca_27559.1|sca	superfamily	261422	4	41	8.95729e-07	46.249	cl02720	PB1 superfamily	-
Q#2760 - >Ca_27559.1|sca	superfamily	276306	346	378	1.75623e-06	44.7291	cl21463	UBA_like_SF superfamily	-
Q#2763 - >Ca_27564.1|sca	multi-dom	223066	141	254	0.00801143	36.1897	PHA03379	PHA03379	-
Q#2764 - >Ca_27565.1|sca	superfamily	275927	1	36	0.00188996	34.2344	cl11403	pepsin_retropepsin_like superfamily	-
Q#2765 - >Ca_27566.1|sca	superfamily	276297	81	308	7.49371e-64	205.584	cl21453	PKc_like superfamily	-
Q#2767 - >Ca_27570.1|sca	specific	275441	21	174	3.4658e-29	106.352	cd15797	PMEI	cl04375
Q#2767 - >Ca_27570.1|sca	superfamily	275808	21	174	3.4658e-29	106.352	cl04375	PMEI_like superfamily	-
Q#2773 - >Ca_27588.1|sca	specific	258207	22	95	1.3951e-21	87.2515	pfam13952	DUF4216	cl16495
Q#2773 - >Ca_27588.1|sca	superfamily	258207	22	95	1.3951e-21	87.2515	cl16495	DUF4216 superfamily	-
Q#2775 - >Ca_27593.1|sca	specific	260004	2	46	2.47225e-23	94.8425	cd09272	RNase_HI_RT_Ty1	cl14782
Q#2775 - >Ca_27593.1|sca	superfamily	271754	2	46	2.47225e-23	94.8425	cl14782	RNase_H_like superfamily	-
Q#2776 - >Ca_27594.1|sca	specific	258207	34	107	7.81156e-28	100.733	pfam13952	DUF4216	cl16495
Q#2776 - >Ca_27594.1|sca	superfamily	258207	34	107	7.81156e-28	100.733	cl16495	DUF4216 superfamily	-
Q#2778 - >Ca_27597.1|sca	multi-dom	257477	100	148	6.72309e-09	52.7902	pfam13041	PPR_2	-
Q#2778 - >Ca_27597.1|sca	multi-dom	257477	408	457	1.32967e-06	46.2418	pfam13041	PPR_2	-
Q#2778 - >Ca_27597.1|sca	multi-dom	257477	3	45	0.00147847	36.997	pfam13041	PPR_2	-
Q#2780 - >Ca_27617.1|sca	multi-dom	223021	23	200	0.000750329	39.5365	PHA03247	PHA03247	-
Q#2781 - >Ca_27621.1|sca	multi-dom	223066	212	342	0.00405666	37.7305	PHA03379	PHA03379	-
Q#2784 - >Ca_27632.1|sca	superfamily	263790	243	351	0.00153294	38.5596	cl09506	catalase_like superfamily	-
Q#2788 - >Ca_27646.1|sca	multi-dom	183854	263	341	0.000245673	41.5421	PRK13042	PRK13042	-
Q#2789 - >Ca_27648.1|sca	superfamily	275485	63	133	0.00759832	37.1745	cl00210	Isoprenoid_Biosyn_C1 superfamily	-
Q#2790 - >Ca_27654.1|sca	specific	192898	1	47	4.4561e-14	63.4893	pfam11961	DUF3475	cl13408
Q#2790 - >Ca_27654.1|sca	superfamily	192898	1	47	4.4561e-14	63.4893	cl13408	DUF3475 superfamily	-
Q#2791 - >Ca_27655.1|sca	specific	238825	124	211	6.40858e-29	108.837	cd01647	RT_LTR	cl02808
Q#2791 - >Ca_27655.1|sca	superfamily	261455	124	211	6.40858e-29	108.837	cl02808	RT_like superfamily	-
Q#2792 - >Ca_27656.1|sca	specific	252133	102	201	4.43126e-13	63.1265	pfam03732	Retrotrans_gag	cl04237
Q#2792 - >Ca_27656.1|sca	superfamily	252133	102	201	4.43126e-13	63.1265	cl04237	Retrotrans_gag superfamily	-

Query	Types	PSSM-ID	From	To	E-Value	Bitscore	Accession	Short name	Superfamily
Q#2794 - >Ca_27663.1|sca	superfamily	256046	65	290	4.6699e-36	135.6	cl11168	PMD superfamily	-
Q#2795 - >Ca_27664.1|sca	multi-dom	227361	591	703	0.00463724	39.0046	COG5028	COG5028	-
Q#2798 - >Ca_27668.1|sca	superfamily	260250	6	56	2.19103e-12	58.4628	cl00195	SIR2 superfamily	-
Q#2800 - >Ca_27675.1|sca	multi-dom	223066	215	327	0.000197124	42.3529	PHA03379	PHA03379	-
Q#2803 - >Ca_27686.1|sca	superfamily	252133	82	143	0.000758428	35.3921	cl04237	Retrotrans_gag superfamily	-
Q#2804 - >Ca_27687.1|sca	superfamily	275485	64	135	0.00468125	37.9449	cl00210	Isoprenoid_Biosyn_C1 superfamily	-
Q#2805 - >Ca_27689.1|sca	multi-dom	215130	214	296	0.00082444	40.4576	PLN02217	PLN02217	-
Q#2806 - >Ca_27690.1|sca	specific	249850	6	215	1.85603e-70	219.315	pfam00425	Chorismate_bind	cl10555
Q#2806 - >Ca_27690.1|sca	superfamily	275922	6	215	1.85603e-70	219.315	cl10555	Chorismate_bind superfamily	-
Q#2807 - >Ca_27694.1|sca	superfamily	275927	194	231	7.80319e-05	40.0124	cl11403	pepsin_retropepsin_like superfamily	-
Q#2807 - >Ca_27694.1|sca	superfamily	261455	289	308	0.00144621	37.5749	cl02808	RT_like superfamily	-
Q#2808 - >Ca_27696.1|sca	specific	252133	71	162	2.26098e-13	64.2821	pfam03732	Retrotrans_gag	cl04237
Q#2808 - >Ca_27696.1|sca	superfamily	252133	71	162	2.26098e-13	64.2821	cl04237	Retrotrans_gag superfamily	-
Q#2808 - >Ca_27696.1|sca	specific	189387	244	260	8.12703e-05	39.3597	pfam00098	zf-CCHC	cl22700
Q#2808 - >Ca_27696.1|sca	superfamily	276654	244	260	8.12703e-05	39.3597	cl22700	zf-CCHC superfamily	-
Q#2810 - >Ca_27703.1|sca	superfamily	276020	1	203	1.32956e-69	222.764	cl12078	p450 superfamily	-
Q#2811 - >Ca_27704.1|sca	specific	258213	19	47	7.4799e-05	35.4794	pfam13961	DUF4219	cl22382
Q#2811 - >Ca_27704.1|sca	superfamily	258213	19	47	7.4799e-05	35.4794	cl22382	DUF4219 superfamily	-
Q#2816 - >Ca_27731.1|sca	superfamily	147759	136	262	0.00163064	38.8376	cl21892	ECM1 superfamily	-
Q#2817 - >Ca_27736.1|sca	superfamily	276217	42	120	1.33481e-46	160.579	cl18945	AAT_I superfamily	-
Q#2818 - >Ca_27742.1|sca	specific	107229	54	145	1.81297e-56	174.415	cd06472	ACD_ScHsp26_like	cl00175
Q#2818 - >Ca_27742.1|sca	superfamily	271432	54	145	1.81297e-56	174.415	cl00175	alpha-crystallin-Hsps_p23-like superfamily	-
Q#2818 - >Ca_27742.1|sca	multi-dom	223149	21	159	2.10945e-27	100.914	COG0071	IbpA	-
Q#2819 - >Ca_27749.1|sca	superfamily	276192	1	35	7.58591e-13	58.7789	cl17169	RRM_SF superfamily	-
Q#2819 - >Ca_27749.1|sca	superfamily	265686	46	98	7.793e-06	41.5066	cl15397	DUF89 superfamily	-
Q#2819 - >Ca_27749.1|sca	multi-dom	214636	1	35	1.00244e-09	50.2847	smart00360	RRM	-
Q#2820 - >Ca_27751.1|sca	multi-dom	223021	9	156	0.00103148	39.1513	PHA03247	PHA03247	-
Q#2824 - >Ca_27764.1|sca	superfamily	276297	160	380	1.50414e-47	164.753	cl21453	PKc_like superfamily	-
Q#2824 - >Ca_27764.1|sca	superfamily	201524	36	115	8.87326e-08	49.5861	cl03036	S_locus_glycop superfamily	-
Q#2828 - >Ca_27784.1|sca	superfamily	252804	1	59	7.57498e-08	46.6708	cl04754	LMBR1 superfamily	-
Q#2829 - >Ca_27791.1|sca	superfamily	275904	2	84	2.83737e-29	103.878	cl09927	S1_like superfamily	-
Q#2831 - >Ca_27796.1|sca	superfamily	252133	100	199	4.95265e-12	60.0449	cl04237	Retrotrans_gag superfamily	-
Q#2833 - >Ca_27804.1|sca	superfamily	252133	100	199	9.22368e-13	61.9709	cl04237	Retrotrans_gag superfamily	-
Q#2834 - >Ca_27809.1|sca	specific	128458	195	230	3.4766e-15	66.5737	smart00153	VHP	cl02491
Q#2834 - >Ca_27809.1|sca	superfamily	207613	195	230	3.4766e-15	66.5737	cl02491	VHP superfamily	-
Q#2835 - >Ca_27810.1|sca	specific	119392	14	175	1.46578e-08	52.3161	cd06174	MFS	cl21472

Query	Types	PSSM-ID	From	To	E-Value	Bitscore	Accession	Short name	Superfamily
Q#2835 - >Ca_27810.1|sca	superfamily	276313	14	175	1.46578e-08	52.3161	cl21472	MFS superfamily	-
Q#2836 - >Ca_27814.1|sca	specific	238531	1	89	1.5879e-22	89.4178	cd01098	PAN_AP_plant	cl00112
Q#2836 - >Ca_27814.1|sca	superfamily	260198	1	89	1.5879e-22	89.4178	cl00112	PAN_APPLE superfamily	-
Q#2836 - >Ca_27814.1|sca	superfamily	276297	147	257	2.37605e-19	84.4693	cl21453	PKc_like superfamily	-
Q#2836 - >Ca_27814.1|sca	superfamily	256698	258	303	0.000777029	36.533	cl13343	DUF3403 superfamily	-
Q#2840 - >Ca_27825.1|sca	superfamily	276175	9	71	1.15627e-51	166.749	cl17037	NBD_sugar-kinase_HSP70_actin superfamily	-
Q#2843 - >Ca_27836.1|sca	multi-dom	237862	186	255	0.000240363	41.8721	PRK14948	PRK14948	-
Q#2844 - >Ca_27837.1|sca	superfamily	271941	1	115	1.746e-19	82.9652	cl21521	PEPcase superfamily	-
Q#2846 - >Ca_27844.1|sca	specific	252133	100	199	1.14401e-14	66.5933	pfam03732	Retrotrans_gag	cl04237
Q#2846 - >Ca_27844.1|sca	superfamily	252133	100	199	1.14401e-14	66.5933	cl04237	Retrotrans_gag superfamily	-
Q#2847 - >Ca_27847.1|sca	superfamily	276297	138	185	9.28535e-09	55.2975	cl21453	PKc_like superfamily	-
Q#2853 - >Ca_27867.1|sca	multi-dom	148271	291	330	0.00275199	37.6654	pfam06566	Chon_Sulph_att	-
Q#2858 - >Ca_27883.1|sca	superfamily	276301	1	39	1.11002e-16	69.6455	cl21457	TIM_phosphate_binding superfamily	-
Q#2859 - >Ca_27884.1|sca	superfamily	263970	14	258	4.89393e-44	151.37	cl10447	GH18_chitinase-like superfamily	-
Q#2860 - >Ca_27888.1|sca	superfamily	251754	26	317	3.86047e-48	167.816	cl03911	DUF247 superfamily	-
Q#2861 - >Ca_27895.1|sca	superfamily	252133	100	199	6.57717e-12	59.6597	cl04237	Retrotrans_gag superfamily	-
Q#2862 - >Ca_27896.1|sca	superfamily	275846	1	174	4.59038e-57	184.364	cl07893	AmyAc_family superfamily	-
Q#2864 - >Ca_27903.1|sca	multi-dom	227414	176	243	0.000529669	38.6784	COG5082	AIR1	-
Q#2866 - >Ca_27910.1|sca	specific	238093	103	145	1.22317e-10	53.2184	cd00162	RING	cl17238
Q#2866 - >Ca_27910.1|sca	superfamily	276201	103	145	1.22317e-10	53.2184	cl17238	RING superfamily	-
Q#2867 - >Ca_27913.1|sca	specific	133136	426	513	5.4004e-16	73.91	cd00303	retropepsin_like	cl11403
Q#2867 - >Ca_27913.1|sca	superfamily	275927	426	513	5.4004e-16	73.91	cl11403	pepsin_retropepsin_like superfamily	-
Q#2867 - >Ca_27913.1|sca	specific	133136	336	366	3.59856e-05	42.3236	cd00303	retropepsin_like	cl11403
Q#2867 - >Ca_27913.1|sca	superfamily	275927	336	366	3.59856e-05	42.3236	cl11403	pepsin_retropepsin_like superfamily	-
Q#2867 - >Ca_27913.1|sca	superfamily	252133	100	199	1.91674e-11	61.2005	cl04237	Retrotrans_gag superfamily	-
Q#2868 - >Ca_27915.1|sca	specific	251775	40	139	1.25096e-33	117.125	pfam03171	2OG-FeII_Oxy	cl21496
Q#2868 - >Ca_27915.1|sca	superfamily	276328	40	139	1.25096e-33	117.125	cl21496	2OG-FeII_Oxy superfamily	-
Q#2870 - >Ca_27922.1|sca	multi-dom	178748	269	383	0.00667748	37.5983	PLN03209	PLN03209	-
Q#2871 - >Ca_27923.1|sca	superfamily	275927	119	155	1.83512e-05	41.2511	cl11403	pepsin_retropepsin_like superfamily	-
Q#2873 - >Ca_27929.1|sca	superfamily	276326	4	48	0.000194863	37.6503	cl21494	Esterase_lipase superfamily	-
Q#2874 - >Ca_27931.1|sca	specific	238825	254	430	4.25273e-98	294.503	cd01647	RT_LTR	cl02808
Q#2874 - >Ca_27931.1|sca	superfamily	261455	254	430	4.25273e-98	294.503	cl02808	RT_like superfamily	-
Q#2874 - >Ca_27931.1|sca	specific	133136	47	134	9.2065e-18	78.1471	cd00303	retropepsin_like	cl11403
Q#2874 - >Ca_27931.1|sca	superfamily	275927	47	134	9.2065e-18	78.1471	cl11403	pepsin_retropepsin_like superfamily	-
Q#2874 - >Ca_27931.1|sca	multi-dom	249567	271	430	8.50085e-37	134.381	pfam00078	RVT_1	-
Q#2878 - >Ca_27935.1|sca	superfamily	252133	67	143	9.46571e-11	54.6521	cl04237	Retrotrans_gag superfamily	-

Query	Types	PSSM-ID	From	To	E-Value	Bitscore	Accession	Short name	Superfamily
Q#2882 - >Ca_27946.1|sca	superfamily	261267	30	93	6.18467e-16	69.0965	cl02381	Tim17 superfamily	-
Q#2885 - >Ca_27957.1|sca	superfamily	275758	28	95	0.00462985	36.6986	cl02170	Sec62 superfamily	-
Q#2887 - >Ca_27962.1|sca	specific	258415	78	187	1.40912e-30	110.74	pfam14223	UBN2	cl15874
Q#2887 - >Ca_27962.1|sca	superfamily	265821	78	187	1.40912e-30	110.74	cl15874	UBN2 superfamily	-
Q#2887 - >Ca_27962.1|sca	specific	258213	19	46	2.62283e-05	40.1018	pfam13961	DUF4219	cl22382
Q#2887 - >Ca_27962.1|sca	superfamily	258213	19	46	2.62283e-05	40.1018	cl22382	DUF4219 superfamily	-
Q#2890 - >Ca_27977.1|sca	specific	237985	1	37	2.43723e-15	67.6819	cd00018	AP2	cl00033
Q#2890 - >Ca_27977.1|sca	superfamily	260146	1	37	2.43723e-15	67.6819	cl00033	AP2 superfamily	-
Q#2891 - >Ca_27982.1|sca	specific	273253	175	208	3.53686e-05	39.7459	TIGR00756	PPR	cl03252
Q#2891 - >Ca_27982.1|sca	superfamily	275791	175	208	3.53686e-05	39.7459	cl03252	PPR superfamily	-
Q#2891 - >Ca_27982.1|sca	specific	250692	86	115	5.99337e-05	38.9741	pfam01535	PPR	cl03252
Q#2891 - >Ca_27982.1|sca	superfamily	275791	86	115	5.99337e-05	38.9741	cl03252	PPR superfamily	-
Q#2891 - >Ca_27982.1|sca	multi-dom	257477	82	126	2.63051e-10	53.9458	pfam13041	PPR_2	-
Q#2891 - >Ca_27982.1|sca	multi-dom	257477	171	218	4.7797e-09	50.479	pfam13041	PPR_2	-
Q#2891 - >Ca_27982.1|sca	multi-dom	257477	141	183	2.54397e-05	40.4638	pfam13041	PPR_2	-
Q#2891 - >Ca_27982.1|sca	multi-dom	257477	54	96	0.000346058	36.997	pfam13041	PPR_2	-
Q#2893 - >Ca_27986.1|sca	specific	275441	24	171	3.9991e-28	103.656	cd15797	PMEI	cl04375
Q#2893 - >Ca_27986.1|sca	superfamily	275808	24	171	3.9991e-28	103.656	cl04375	PMEI_like superfamily	-
Q#2894 - >Ca_27988.1|sca	superfamily	276034	1	27	1.31798e-09	53.1353	cl13995	MPP_superfamily superfamily	-
Q#2895 - >Ca_27989.1|sca	specific	133136	136	223	4.86862e-19	79.3027	cd00303	retropepsin_like	cl11403
Q#2895 - >Ca_27989.1|sca	superfamily	275927	136	223	4.86862e-19	79.3027	cl11403	pepsin_retropepsin_like superfamily	-
Q#2896 - >Ca_27990.1|sca	specific	252133	103	182	1.66088e-12	62.3561	pfam03732	Retrotrans_gag	cl04237
Q#2896 - >Ca_27990.1|sca	superfamily	252133	103	182	1.66088e-12	62.3561	cl04237	Retrotrans_gag superfamily	-
Q#2900 - >Ca_28000.1|sca	superfamily	256046	20	109	1.20843e-17	75.894	cl11168	PMD superfamily	-
Q#2901 - >Ca_28003.1|sca	multi-dom	257383	19	94	2.44639e-18	75.0006	pfam12895	Apc3	-
Q#2904 - >Ca_28016.1|sca	specific	239926	57	123	2.36746e-15	66.5068	cd04480	RPA1_DBD_A_like	cl09930
Q#2904 - >Ca_28016.1|sca	superfamily	275906	57	123	2.36746e-15	66.5068	cl09930	RPA_2b-aaRSs_OBF_like superfamily	-
Q#2905 - >Ca_28017.1|sca	multi-dom	252721	135	282	0.000682983	40.0461	pfam04652	DUF605	-
Q#2907 - >Ca_28024.1|sca	superfamily	251836	78	153	0.00628199	36.7942	cl03966	CDK5_activator superfamily	-
Q#2908 - >Ca_28026.1|sca	specific	189387	65	80	0.00445285	32.4261	pfam00098	zf-CCHC	cl22700
Q#2908 - >Ca_28026.1|sca	superfamily	276654	65	80	0.00445285	32.4261	cl22700	zf-CCHC superfamily	-
Q#2912 - >Ca_28040.1|sca	specific	206754	1	83	5.58723e-07	45.0792	cd06223	PRTases_typeI	cl00309
Q#2912 - >Ca_28040.1|sca	superfamily	275521	1	83	5.58723e-07	45.0792	cl00309	PRTases_typeI superfamily	-
Q#2914 - >Ca_28046.1|sca	superfamily	276034	56	98	0.00460621	33.6287	cl13995	MPP_superfamily superfamily	-
Q#2915 - >Ca_28047.1|sca	specific	133136	202	291	1.59748e-18	79.3027	cd00303	retropepsin_like	cl11403
Q#2915 - >Ca_28047.1|sca	superfamily	275927	202	291	1.59748e-18	79.3027	cl11403	pepsin_retropepsin_like superfamily	-

Query	Types	PSSM-ID	From	To	E-Value	Bitscore	Accession	Short name	Superfamily
Q#2916 - >Ca_28050.1|sca	multi-dom	223021	190	325	0.000543979	41.0773	PHA03247	PHA03247	-
Q#2917 - >Ca_28057.1|sca	superfamily	252133	100	164	2.87324e-05	39.6293	cl04237	Retrotrans_gag superfamily	-
Q#2918 - >Ca_28059.1|sca	superfamily	276299	133	161	6.01617e-11	58.394	cl21455	ABC_ATPase superfamily	-
Q#2923 - >Ca_28082.1|sca	superfamily	275753	68	169	3.12148e-52	170.1	cl01911	Pectinesterase superfamily	-
Q#2925 - >Ca_28090.1|sca	superfamily	252133	78	155	2.43662e-10	53.4965	cl04237	Retrotrans_gag superfamily	-
Q#2926 - >Ca_28094.1|sca	superfamily	271660	1	88	5.23043e-23	91.0949	cl09326	MATE_like superfamily	-
Q#2928 - >Ca_28097.1|sca	superfamily	276007	297	356	0.00998279	36.4259	cl11961	ALDH-SF superfamily	-
Q#2929 - >Ca_28099.1|sca	superfamily	252133	100	198	3.31438e-12	59.2745	cl04237	Retrotrans_gag superfamily	-
Q#2930 - >Ca_28102.1|sca	superfamily	275476	91	116	2.18116e-05	40.1778	cl00155	UBQ superfamily	-
Q#2932 - >Ca_28108.1|sca	multi-dom	173561	157	191	2.08208e-07	47.1089	PTZ00368	PTZ00368	-
Q#2933 - >Ca_28109.1|sca	superfamily	253337	3	43	2.34534e-07	44.4337	cl05327	Auxin_canalis superfamily	-
Q#2934 - >Ca_28110.1|sca	specific	275443	2	156	3.3277e-21	86.3099	cd15799	PMEI-like_4	cl04375
Q#2934 - >Ca_28110.1|sca	superfamily	275808	2	156	3.3277e-21	86.3099	cl04375	PMEI_like superfamily	-
Q#2934 - >Ca_28110.1|sca	superfamily	275753	195	228	4.96816e-12	62.6296	cl01911	Pectinesterase superfamily	-
Q#2935 - >Ca_28112.1|sca	superfamily	252384	8	61	0.00014607	37.7782	cl04404	Gpi16 superfamily	-
Q#2939 - >Ca_28121.1|sca	superfamily	276194	21	223	1.12717e-33	123.114	cl17173	AdoMet_MTases superfamily	-
Q#2940 - >Ca_28124.1|sca	superfamily	256046	9	96	2.28708e-10	55.8636	cl11168	PMD superfamily	-
Q#2942 - >Ca_28126.1|sca	superfamily	276332	228	244	3.45695e-05	40.7097	cl21508	Ribosomal_P1_P2_L12p superfamily	-
Q#2943 - >Ca_28127.1|sca	specific	119408	3	129	2.7798e-36	126.905	cd06660	Aldo_ket_red	cl00470
Q#2943 - >Ca_28127.1|sca	superfamily	260445	3	129	2.7798e-36	126.905	cl00470	Aldo_ket_red superfamily	-
Q#2945 - >Ca_28138.1|sca	specific	252133	100	199	9.39482e-14	64.2821	pfam03732	Retrotrans_gag	cl04237
Q#2945 - >Ca_28138.1|sca	superfamily	252133	100	199	9.39482e-14	64.2821	cl04237	Retrotrans_gag superfamily	-
Q#2949 - >Ca_28144.1|sca	superfamily	253682	93	205	0.00307402	36.9619	cl09408	Med13_C superfamily	-
Q#2950 - >Ca_28149.1|sca	specific	189387	197	212	0.00933641	32.8113	pfam00098	zf-CCHC	cl22700
Q#2950 - >Ca_28149.1|sca	superfamily	276654	197	212	0.00933641	32.8113	cl22700	zf-CCHC superfamily	-
Q#2951 - >Ca_28150.1|sca	multi-dom	223021	190	316	4.73094e-06	48.0109	PHA03247	PHA03247	-
Q#2955 - >Ca_28159.1|sca	superfamily	251724	47	116	2.91737e-31	114.788	cl03887	Mlo superfamily	-
Q#2958 - >Ca_28169.1|sca	multi-dom	185271	15	57	0.00637176	32.3564	PRK15373	PRK15373	-
Q#2963 - >Ca_28193.1|sca	specific	189387	112	128	0.00141157	33.9669	pfam00098	zf-CCHC	cl22700
Q#2963 - >Ca_28193.1|sca	superfamily	276654	112	128	0.00141157	33.9669	cl22700	zf-CCHC superfamily	-
Q#2964 - >Ca_28194.1|sca	superfamily	190526	135	171	4.21966e-09	49.9086	cl07846	DBD_Tnp_Mut superfamily	-
Q#2972 - >Ca_28226.1|sca	specific	189387	165	180	0.00163548	35.5077	pfam00098	zf-CCHC	cl22700
Q#2972 - >Ca_28226.1|sca	superfamily	276654	165	180	0.00163548	35.5077	cl22700	zf-CCHC superfamily	-
Q#2973 - >Ca_28232.1|sca	superfamily	256046	27	91	6.53297e-06	43.5372	cl11168	PMD superfamily	-
Q#2976 - >Ca_28241.1|sca	multi-dom	250737	15	134	4.32711e-32	118.094	pfam01602	Adaptin_N	-
Q#2978 - >Ca_28250.1|sca	superfamily	251669	2	89	1.33829e-09	52.0316	cl03830	Transposase_24 superfamily	-

Query	Types	PSSM-ID	From	To	E-Value	Bitscore	Accession	Short name	Superfamily
Q#2978 - >Ca_28250.1|sca	superfamily	112929	93	168	0.0049279	34.4769	cl04414	Sec34 superfamily	-
Q#2981 - >Ca_28255.1|sca	superfamily	275607	1	123	8.14106e-54	174.861	cl00504	Cytochrom_C_asm superfamily	-
Q#2984 - >Ca_28266.1|sca	superfamily	271754	2	34	9.96283e-14	64.8213	cl14782	RNase_H_like superfamily	-
Q#2984 - >Ca_28266.1|sca	superfamily	276355	196	241	0.00884116	33.8461	cl21549	rve superfamily	-
Q#2985 - >Ca_28267.1|sca	superfamily	260250	6	56	2.19103e-12	58.4628	cl00195	SIR2 superfamily	-
Q#2987 - >Ca_28270.1|sca	superfamily	256046	73	137	0.000841002	37.7592	cl11168	PMD superfamily	-
Q#2996 - >Ca_28296.1|sca	multi-dom	223202	2	67	0.00671267	32.1979	COG0124	HisS	-
Q#2997 - >Ca_28299.1|sca	specific	258213	19	47	1.28241e-05	37.4054	pfam13961	DUF4219	cl22382
Q#2997 - >Ca_28299.1|sca	superfamily	258213	19	47	1.28241e-05	37.4054	cl22382	DUF4219 superfamily	-
Q#3002 - >Ca_28328.1|sca	specific	271182	914	1091	3.86279e-65	218.679	cd00801	INT_P4_C	cl00213
Q#3002 - >Ca_28328.1|sca	superfamily	271437	914	1091	3.86279e-65	218.679	cl00213	DNA_BRE_C superfamily	-
Q#3002 - >Ca_28328.1|sca	specific	256705	65	315	4.53248e-129	395.841	pfam11893	DUF3413	cl13352
Q#3002 - >Ca_28328.1|sca	superfamily	256705	65	315	4.53248e-129	395.841	cl13352	DUF3413 superfamily	-
Q#3002 - >Ca_28328.1|sca	specific	237471	1	68	3.40808e-35	130.102	PRK13689	PRK13689	cl01175
Q#3002 - >Ca_28328.1|sca	superfamily	242344	1	68	3.40808e-35	130.102	cl01175	DUF1414 superfamily	-
Q#3002 - >Ca_28328.1|sca	specific	257685	705	793	9.28554e-26	103.401	pfam13356	DUF4102	cl15867
Q#3002 - >Ca_28328.1|sca	superfamily	257685	705	793	9.28554e-26	103.401	cl15867	DUF4102 superfamily	-
Q#3002 - >Ca_28328.1|sca	superfamily	276316	341	562	5.33382e-21	94.4145	cl21476	alkPPc superfamily	-
Q#3002 - >Ca_28328.1|sca	specific	258797	803	854	0.00213392	37.508	pfam14659	Phage_int_SAM_3	cl12235
Q#3002 - >Ca_28328.1|sca	superfamily	276027	803	854	0.00213392	37.508	cl12235	Phage_int_SAM_1 superfamily	-
Q#3002 - >Ca_28328.1|sca	multi-dom	225625	68	642	0	793.554	COG3083	COG3083	-
Q#3002 - >Ca_28328.1|sca	multi-dom	170049	698	1098	0	557.717	PRK09692	PRK09692	-
Q#3003 - >Ca_28348.1|sca	specific	107262	309	566	1.16764e-75	243.291	cd06267	PBP1_LacI_sugar_binding_like	cl10011
Q#3003 - >Ca_28348.1|sca	superfamily	275911	309	566	1.16764e-75	243.291	cl10011	Periplasmic_Binding_Protein_Type_1 superfamily	-
Q#3003 - >Ca_28348.1|sca	specific	143331	253	304	8.28961e-18	78.2189	cd01392	HTH_LacI	cl21459
Q#3003 - >Ca_28348.1|sca	superfamily	276302	253	304	8.28961e-18	78.2189	cl21459	HTH_XRE superfamily	-
Q#3003 - >Ca_28348.1|sca	specific	119392	9	223	2.29096e-09	58.0941	cd06174	MFS	cl21472
Q#3003 - >Ca_28348.1|sca	superfamily	276313	9	223	2.29096e-09	58.0941	cl21472	MFS superfamily	-
Q#3003 - >Ca_28348.1|sca	multi-dom	224525	249	573	4.59952e-98	304.232	COG1609	PurR	-
Q#3004 - >Ca_28367.1|sca	specific	238923	216	450	9.19237e-85	269.03	cd01948	EAL	cl00290
Q#3004 - >Ca_28367.1|sca	superfamily	260330	216	450	9.19237e-85	269.03	cl00290	EAL superfamily	-
Q#3004 - >Ca_28367.1|sca	specific	119392	499	685	1.57199e-19	89.2953	cd06174	MFS	cl21472
Q#3004 - >Ca_28367.1|sca	superfamily	276313	499	685	1.57199e-19	89.2953	cl21472	MFS superfamily	-
Q#3004 - >Ca_28367.1|sca	specific	257300	1	189	4.51611e-21	92.2117	pfam12792	CSS-motif	cl15094
Q#3004 - >Ca_28367.1|sca	superfamily	257300	1	189	4.51611e-21	92.2117	cl15094	CSS-motif superfamily	-
Q#3005 - >Ca_28375.1|sca	specific	119392	493	866	1.81621e-06	49.6197	cd06174	MFS	cl21472

Query	Types	PSSM-ID	From	To	E-Value	Bitscore	Accession	Short name	Superfamily
Q#3005 - >Ca_28375.1|sca	superfamily	276313	493	866	1.81621e-06	49.6197	cl21472	MFS superfamily	-
Q#3005 - >Ca_28375.1|sca	superfamily	275586	35	390	5.2749e-05	45.5076	cl00456	SLC5-6-like_sbd superfamily	-
Q#3005 - >Ca_28375.1|sca	multi-dom	182324	1	439	0	807.641	PRK10238	PRK10238	-
Q#3005 - >Ca_28375.1|sca	multi-dom	225121	471	929	7.37555e-160	480.208	COG2211	MelB	-
Q#3006 - >Ca_28378.1|sca	specific	119394	96	326	3.32586e-17	77.3188	cd06261	TM_PBP2	cl00427
Q#3006 - >Ca_28378.1|sca	superfamily	275575	96	326	3.32586e-17	77.3188	cl00427	TM_PBP2 superfamily	-
Q#3006 - >Ca_28378.1|sca	multi-dom	223674	1	326	1.4186e-126	367.585	COG0601	DppB	-
Q#3007 - >Ca_28381.1|sca	specific	249873	224	381	1.69238e-70	221.213	pfam00455	DeoRC	cl00339
Q#3007 - >Ca_28381.1|sca	superfamily	275540	224	381	1.69238e-70	221.213	cl00339	SugarP_isomerase superfamily	-
Q#3007 - >Ca_28381.1|sca	specific	223777	20	152	1.20751e-29	114.484	COG0705	COG0705	cl21536
Q#3007 - >Ca_28381.1|sca	superfamily	276350	20	152	1.20751e-29	114.484	cl21536	Rhomboid superfamily	-
Q#3007 - >Ca_28381.1|sca	specific	116806	155	210	4.6084e-21	85.7669	pfam08220	HTH_DeoR	cl21459
Q#3007 - >Ca_28381.1|sca	superfamily	276302	155	210	4.6084e-21	85.7669	cl21459	HTH_XRE superfamily	-
Q#3007 - >Ca_28381.1|sca	multi-dom	182827	150	401	0	544.068	PRK10906	PRK10906	-
Q#3008 - >Ca_28382.1|sca	specific	257938	152	287	1.54478e-40	139.259	pfam13628	DUF4142	cl21583
Q#3008 - >Ca_28382.1|sca	superfamily	272003	152	287	1.54478e-40	139.259	cl21583	DUF305 superfamily	-
Q#3008 - >Ca_28382.1|sca	specific	226252	1	60	4.40606e-18	76.3617	COG3729	GsiB	cl01514
Q#3008 - >Ca_28382.1|sca	superfamily	226252	1	60	4.40606e-18	76.3617	cl01514	GsiB superfamily	-
Q#3009 - >Ca_28386.1|sca	specific	119394	47	221	4.27382e-10	55.7476	cd06261	TM_PBP2	cl00427
Q#3009 - >Ca_28386.1|sca	superfamily	275575	47	221	4.27382e-10	55.7476	cl00427	TM_PBP2 superfamily	-
Q#3009 - >Ca_28386.1|sca	multi-dom	273441	4	228	9.58879e-06	44.0696	TIGR01097	PhnE	-
Q#3010 - >Ca_28387.1|sca	specific	214628	28	136	1.20707e-28	103.75	smart00344	HTH_ASNC	cl21459
Q#3010 - >Ca_28387.1|sca	superfamily	276302	28	136	1.20707e-28	103.75	cl21459	HTH_XRE superfamily	-
Q#3010 - >Ca_28387.1|sca	multi-dom	224439	24	176	1.56442e-37	128.657	COG1522	Lrp	-
Q#3011 - >Ca_28402.1|sca	specific	99990	2	330	1.10246e-89	274.855	cd03820	GT1_amsD_like	cl10013
Q#3011 - >Ca_28402.1|sca	superfamily	275912	2	330	1.10246e-89	274.855	cl10013	Glycosyltransferase_GTB_type superfamily	-
Q#3011 - >Ca_28402.1|sca	multi-dom	223515	20	320	7.92469e-38	139.698	COG0438	RfaG	-
Q#3012 - >Ca_28411.1|sca	specific	249873	77	233	7.60856e-62	193.864	pfam00455	DeoRC	cl00339
Q#3012 - >Ca_28411.1|sca	superfamily	275540	77	233	7.60856e-62	193.864	cl00339	SugarP_isomerase superfamily	-
Q#3012 - >Ca_28411.1|sca	specific	197714	6	58	5.29504e-19	78.0329	smart00420	HTH_DEOR	cl21459
Q#3012 - >Ca_28411.1|sca	superfamily	276302	6	58	5.29504e-19	78.0329	cl21459	HTH_XRE superfamily	-
Q#3012 - >Ca_28411.1|sca	multi-dom	224268	1	253	5.59663e-95	281.815	COG1349	GlpR	-
Q#3013 - >Ca_28414.1|sca	superfamily	254749	47	154	7.21849e-29	106.523	cl10076	PAS_6 superfamily	-
Q#3013 - >Ca_28414.1|sca	specific	257646	176	239	6.86882e-19	77.8687	pfam13309	HTH_22	cl21459
Q#3013 - >Ca_28414.1|sca	superfamily	276302	176	239	6.86882e-19	77.8687	cl21459	HTH_XRE superfamily	-
Q#3013 - >Ca_28414.1|sca	multi-dom	225512	43	243	1.19888e-52	171.736	COG2964	COG2964	-

Query	Types	PSSM-ID	From	To	E-Value	Bitscore	Accession	Short name	Superfamily
Q#3014 - >Ca_28442.1|sca	superfamily	275533	93	175	1.35269e-25	94.9239	cl00326	Ribosomal_L23 superfamily	-
Q#3014 - >Ca_28442.1|sca	specific	252262	60	87	4.79455e-09	49.2214	pfam03939	Ribosomal_L23eN	cl04335
Q#3014 - >Ca_28442.1|sca	superfamily	252262	60	87	4.79455e-09	49.2214	cl04335	Ribosomal_L23eN superfamily	-
Q#3014 - >Ca_28442.1|sca	multi-dom	185507	60	175	2.53165e-53	168.725	PTZ00191	PTZ00191	-
Q#3016 - >Ca_28450.1|sca	specific	271182	216	393	1.01884e-68	217.138	cd00801	INT_P4_C	cl00213
Q#3016 - >Ca_28450.1|sca	superfamily	271437	216	393	1.01884e-68	217.138	cl00213	DNA_BRE_C superfamily	-
Q#3016 - >Ca_28450.1|sca	specific	257685	8	94	9.83303e-26	99.1637	pfam13356	DUF4102	cl15867
Q#3016 - >Ca_28450.1|sca	superfamily	257685	8	94	9.83303e-26	99.1637	cl15867	DUF4102 superfamily	-
Q#3016 - >Ca_28450.1|sca	multi-dom	170049	1	394	0	578.518	PRK09692	PRK09692	-
Q#3017 - >Ca_28452.1|sca	superfamily	242867	3	133	2.94072e-26	100.334	cl02074	DUF2000 superfamily	-
Q#3017 - >Ca_28452.1|sca	specific	151248	197	256	1.52005e-19	79.7608	pfam10798	YmgB	cl12389
Q#3017 - >Ca_28452.1|sca	superfamily	151248	197	256	1.52005e-19	79.7608	cl12389	YmgB superfamily	-
Q#3018 - >Ca_28453.1|sca	specific	238923	2	124	5.92047e-49	159.248	cd01948	EAL	cl00290
Q#3018 - >Ca_28453.1|sca	superfamily	260330	2	124	5.92047e-49	159.248	cl00290	EAL superfamily	-
Q#3019 - >Ca_28455.1|sca	specific	119392	1	162	2.45505e-07	48.0789	cd06174	MFS	cl21472
Q#3019 - >Ca_28455.1|sca	superfamily	276313	1	162	2.45505e-07	48.0789	cl21472	MFS superfamily	-
Q#3020 - >Ca_28465.1|sca	specific	213181	3	217	1.83405e-69	213.836	cd03214	ABC_Iron-Siderophores_B12_Hemin	cl21455
Q#3020 - >Ca_28465.1|sca	superfamily	276299	3	217	1.83405e-69	213.836	cl21455	ABC_ATPase superfamily	-
Q#3020 - >Ca_28465.1|sca	multi-dom	224045	2	247	7.36724e-88	263.655	COG1120	FepC	-
Q#3021 - >Ca_28471.1|sca	specific	107318	15	284	1.08645e-119	346.528	cd06323	PBP1_ribose_binding	cl10011
Q#3021 - >Ca_28471.1|sca	superfamily	275911	15	284	1.08645e-119	346.528	cl10011	Periplasmic_Binding_Protein_Type_1 superfamily	-
Q#3021 - >Ca_28471.1|sca	multi-dom	257732	17	271	1.55015e-52	174.026	pfam13407	Peripla_BP_4	-
Q#3022 - >Ca_28478.1|sca	superfamily	275527	55	183	1.0982e-88	264.613	cl00316	Cation_efflux superfamily	-
Q#3022 - >Ca_28478.1|sca	specific	226252	1	58	2.00951e-08	48.2422	COG3729	GsiB	cl01514
Q#3022 - >Ca_28478.1|sca	superfamily	226252	1	58	2.00951e-08	48.2422	cl01514	GsiB superfamily	-
Q#3023 - >Ca_28490.1|sca	specific	254025	27	91	2.23299e-27	99.7025	pfam07022	Phage_CI_repr	cl21459
Q#3023 - >Ca_28490.1|sca	superfamily	276302	27	91	2.23299e-27	99.7025	cl21459	HTH_XRE superfamily	-
Q#3024 - >Ca_28491.1|sca	superfamily	148487	39	189	1.05322e-45	150.18	cl06105	Phage_CP76 superfamily	-
Q#3025 - >Ca_28493.1|sca	specific	213181	19	236	1.04427e-82	247.734	cd03214	ABC_Iron-Siderophores_B12_Hemin	cl21455
Q#3025 - >Ca_28493.1|sca	superfamily	276299	19	236	1.04427e-82	247.734	cl21455	ABC_ATPase superfamily	-
Q#3025 - >Ca_28493.1|sca	multi-dom	224047	19	243	1.29664e-51	169.744	COG1122	CbiO	-
Q#3026 - >Ca_28504.1|sca	superfamily	276194	19	289	0	532.375	cl17173	AdoMet_MTases superfamily	-
Q#3027 - >Ca_28505.1|sca	specific	257619	31	88	1.77893e-10	54.078	pfam13276	HTH_21	cl16206
Q#3027 - >Ca_28505.1|sca	superfamily	257619	31	88	1.77893e-10	54.078	cl16206	HTH_21 superfamily	-
Q#3028 - >Ca_28507.1|sca	specific	173858	642	992	0	539.072	cd08493	PBP2_DppA_like	cl01709
Q#3028 - >Ca_28507.1|sca	superfamily	275744	642	992	0	539.072	cl01709	PBP2_NikA_DppA_OppA_like superfamily	-

Query	Types	PSSM-ID	From	To	E-Value	Bitscore	Accession	Short name	Superfamily
Q#3028 - >Ca_28507.1|sca	specific	270421	29	257	4.38004e-109	339.61	cd13703	PBP2_HisJ_LAO	cl21456
Q#3028 - >Ca_28507.1|sca	superfamily	276300	29	257	4.38004e-109	339.61	cl21456	Periplasmic_Binding_Protein_Type_2 superfamily	-
Q#3028 - >Ca_28507.1|sca	multi-dom	273440	26	257	1.17934e-75	249.581	TIGR01096	ABC_transporter_arginine-binding_protein	-
Q#3029 - >Ca_28510.1|sca	specific	99982	23	344	8.1941e-57	198.33	cd03811	GT1_WabH_like	cl10013
Q#3029 - >Ca_28510.1|sca	superfamily	275912	23	344	8.1941e-57	198.33	cl10013	Glycosyltransferase_GTB_type superfamily	-
Q#3029 - >Ca_28510.1|sca	specific	252887	555	707	1.55253e-08	53.5527	pfam04932	Wzy_C	cl04850
Q#3029 - >Ca_28510.1|sca	superfamily	275818	555	707	1.55253e-08	53.5527	cl04850	Wzy_C superfamily	-
Q#3029 - >Ca_28510.1|sca	multi-dom	182148	23	367	8.34637e-104	324.742	PRK09922	PRK09922	-
Q#3029 - >Ca_28510.1|sca	multi-dom	225844	367	736	3.30688e-12	67.9137	COG3307	RfaL	-
Q#3030 - >Ca_28511.1|sca	superfamily	260705	1	149	2.07085e-37	136.663	cl00934	CDH superfamily	-
Q#3030 - >Ca_28511.1|sca	specific	256198	243	365	2.65208e-34	124.067	pfam10908	DUF2778	cl12489
Q#3030 - >Ca_28511.1|sca	superfamily	256198	243	365	2.65208e-34	124.067	cl12489	DUF2778 superfamily	-
Q#3031 - >Ca_28517.1|sca	specific	236728	1	108	1.32511e-47	157.472	PRK10629	PRK10629	cl08136
Q#3031 - >Ca_28517.1|sca	superfamily	263534	1	108	1.32511e-47	157.472	cl08136	SecD-TM1 superfamily	-
Q#3031 - >Ca_28517.1|sca	superfamily	275750	225	298	2.04844e-21	86.6103	cl01888	DUF883 superfamily	-
Q#3031 - >Ca_28517.1|sca	specific	253754	151	219	1.24687e-17	76.149	pfam06476	DUF1090	cl05801
Q#3031 - >Ca_28517.1|sca	superfamily	253754	151	219	1.24687e-17	76.149	cl05801	DUF1090 superfamily	-
Q#3032 - >Ca_28520.1|sca	specific	107296	137	408	2.46474e-130	379.265	cd06301	PBP1_rhizopine_binding_like	cl10011
Q#3032 - >Ca_28520.1|sca	superfamily	275911	137	408	2.46474e-130	379.265	cl10011	Periplasmic_Binding_Protein_Type_1 superfamily	-
Q#3032 - >Ca_28520.1|sca	multi-dom	257732	138	395	5.91361e-53	179.419	pfam13407	Peripla_BP_4	-
Q#3033 - >Ca_28521.1|sca	superfamily	275911	1	226	8.66665e-130	375.453	cl10011	Periplasmic_Binding_Protein_Type_1 superfamily	-
Q#3034 - >Ca_28526.1|sca	specific	189752	9	119	1.10638e-34	117.377	pfam00877	NLPC_P60	cl21534
Q#3034 - >Ca_28526.1|sca	superfamily	276349	9	119	1.10638e-34	117.377	cl21534	NLPC_P60 superfamily	-
Q#3034 - >Ca_28526.1|sca	multi-dom	223862	1	118	3.92314e-40	133.951	COG0791	Spr	-
Q#3035 - >Ca_28589.1|sca	superfamily	275722	6	194	1.83478e-23	92.7097	cl01416	Fimbrial superfamily	-
Q#3036 - >Ca_28593.1|sca	specific	119394	1	176	1.4234e-08	50.7401	cd06261	TM_PBP2	cl00427
Q#3036 - >Ca_28593.1|sca	superfamily	275575	1	176	1.4234e-08	50.7401	cl00427	TM_PBP2 superfamily	-
Q#3037 - >Ca_28596.1|sca	specific	213229	3	202	8.31058e-117	343.743	cd03262	ABC_HisP_GlnQ	cl21455
Q#3037 - >Ca_28596.1|sca	superfamily	276299	3	202	8.31058e-117	343.743	cl21455	ABC_ATPase superfamily	-
Q#3037 - >Ca_28596.1|sca	superfamily	275458	228	448	7.41102e-93	291.838	cl00013	Lyase_I_like superfamily	-
Q#3037 - >Ca_28596.1|sca	multi-dom	130234	1	187	3.45014e-31	118.295	TIGR01166	membrane_associated_cobalt_transport_protein_CbiO	-
Q#3038 - >Ca_28602.1|sca	superfamily	276065	24	184	9.60354e-35	124.71	cl14813	GluZincin superfamily	-
Q#3039 - >Ca_28606.1|sca	specific	257986	257	323	4.25286e-26	99.1528	pfam13683	rve_3	cl21549
Q#3039 - >Ca_28606.1|sca	superfamily	276355	257	323	4.25286e-26	99.1528	cl21549	rve superfamily	-
Q#3039 - >Ca_28606.1|sca	specific	250040	170	286	1.3841e-22	90.8556	pfam00665	rve	cl21549
Q#3039 - >Ca_28606.1|sca	superfamily	276355	170	286	1.3841e-22	90.8556	cl21549	rve superfamily	-

Query	Types	PSSM-ID	From	To	E-Value	Bitscore	Accession	Short name	Superfamily
Q#3039 - >Ca_28606.1|sca	specific	250686	2	72	5.27859e-20	82.3816	pfam01527	HTH_Tnp_1	cl21459
Q#3039 - >Ca_28606.1|sca	superfamily	276302	2	72	5.27859e-20	82.3816	cl21459	HTH_XRE superfamily	-
Q#3039 - >Ca_28606.1|sca	specific	257619	102	157	4.19079e-07	46.374	pfam13276	HTH_21	cl16206
Q#3039 - >Ca_28606.1|sca	superfamily	257619	102	157	4.19079e-07	46.374	cl16206	HTH_21 superfamily	-
Q#3039 - >Ca_28606.1|sca	multi-dom	237792	103	338	1.18649e-06	47.8043	PRK14702	PRK14702	-
Q#3039 - >Ca_28606.1|sca	multi-dom	225511	1	111	0.00306055	35.6685	COG2963	COG2963	-
Q#3040 - >Ca_28611.1|sca	specific	271177	333	476	6.8137e-30	116.272	cd00796	INT_Rci_Hp1_C	cl00213
Q#3040 - >Ca_28611.1|sca	superfamily	271437	333	476	6.8137e-30	116.272	cl00213	DNA_BRE_C superfamily	-
Q#3040 - >Ca_28611.1|sca	specific	183471	509	670	1.21317e-117	351.561	PRK12359	PRK12359	cl00438
Q#3040 - >Ca_28611.1|sca	superfamily	275577	509	670	1.21317e-117	351.561	cl00438	FMN_red superfamily	-
Q#3040 - >Ca_28611.1|sca	specific	254025	13	76	1.7159e-24	97.7765	pfam07022	Phage_CI_repr	cl21459
Q#3040 - >Ca_28611.1|sca	superfamily	276302	13	76	1.7159e-24	97.7765	cl21459	HTH_XRE superfamily	-
Q#3040 - >Ca_28611.1|sca	multi-dom	222904	168	491	3.48216e-155	454.571	PHA02601	int	-
Q#3041 - >Ca_28612.1|sca	superfamily	148487	56	211	1.01815e-54	176.759	cl06105	Phage_CP76 superfamily	-
Q#3041 - >Ca_28612.1|sca	superfamily	151341	244	287	3.33923e-10	54.9151	cl12476	DUF2724 superfamily	-
Q#3042 - >Ca_28613.1|sca	specific	240022	1	108	2.46853e-26	102.815	cd04664	Nudix_Hydrolase_7	cl00447
Q#3042 - >Ca_28613.1|sca	superfamily	275580	1	108	2.46853e-26	102.815	cl00447	Nudix_Hydrolase superfamily	-
Q#3042 - >Ca_28613.1|sca	specific	234640	139	377	7.77455e-160	454.924	PRK00110	PRK00110	cl00361
Q#3042 - >Ca_28613.1|sca	superfamily	275551	139	377	7.77455e-160	454.924	cl00361	Transcrip_reg superfamily	-
Q#3042 - >Ca_28613.1|sca	superfamily	271902	376	462	2.10879e-41	146.028	cl21482	RuvC_resolvase superfamily	-
Q#3043 - >Ca_28620.1|sca	specific	259853	15	75	4.61837e-33	110.559	cd13831	HU	cl00257
Q#3043 - >Ca_28620.1|sca	superfamily	275502	15	75	4.61837e-33	110.559	cl00257	HU_IHF superfamily	-
Q#3044 - >Ca_28621.1|sca	specific	189752	45	144	4.89093e-35	118.918	pfam00877	NLPC_P60	cl21534
Q#3044 - >Ca_28621.1|sca	superfamily	276349	45	144	4.89093e-35	118.918	cl21534	NLPC_P60 superfamily	-
Q#3045 - >Ca_28622.1|sca	specific	270248	41	255	1.92417e-65	212.11	cd13530	PBP2_peptides_like	cl21456
Q#3045 - >Ca_28622.1|sca	superfamily	276300	41	255	1.92417e-65	212.11	cl21456	Periplasmic_Binding_Protein_Type_2 superfamily	-
Q#3045 - >Ca_28622.1|sca	multi-dom	249906	41	256	1.22794e-64	210.209	pfam00497	SBP_bac_3	-
Q#3046 - >Ca_28623.1|sca	specific	213223	34	275	1.03272e-98	291.779	cd03256	ABC_PhnC_transporter	cl21455
Q#3046 - >Ca_28623.1|sca	superfamily	276299	34	275	1.03272e-98	291.779	cl21455	ABC_ATPase superfamily	-
Q#3046 - >Ca_28623.1|sca	multi-dom	226164	31	273	1.05516e-112	328.058	COG3638	COG3638	-
Q#3047 - >Ca_28626.1|sca	superfamily	275744	89	278	5.58988e-84	262.114	cl01709	PBP2_NikA_DppA_OppA_like superfamily	-
Q#3048 - >Ca_28667.1|sca	specific	251221	48	134	4.51736e-10	53.2857	pfam02321	OEP	cl22292
Q#3048 - >Ca_28667.1|sca	superfamily	251221	48	134	4.51736e-10	53.2857	cl22292	OEP superfamily	-
Q#3049 - >Ca_28674.1|sca	specific	213224	316	540	2.82327e-81	256.279	cd03257	ABC_NikE_OppD_transporters	cl21455
Q#3049 - >Ca_28674.1|sca	superfamily	276299	316	540	2.82327e-81	256.279	cl21455	ABC_ATPase superfamily	-
Q#3049 - >Ca_28674.1|sca	specific	213224	3	242	3.6299e-77	245.493	cd03257	ABC_NikE_OppD_transporters	cl21455

Query	Types	PSSM-ID	From	To	E-Value	Bitscore	Accession	Short name	Superfamily
Q#3049 - >Ca_28674.1|sca	superfamily	276299	3	242	3.6299e-77	245.493	cl21455	ABC_ATPase superfamily	-
Q#3049 - >Ca_28674.1|sca	specific	254752	239	306	2.80542e-19	82.4708	pfam08352	oligo_HPY	cl07097
Q#3049 - >Ca_28674.1|sca	superfamily	263181	239	306	2.80542e-19	82.4708	cl07097	oligo_HPY superfamily	-
Q#3049 - >Ca_28674.1|sca	superfamily	263181	537	561	0.00490169	35.4764	cl07097	oligo_HPY superfamily	-
Q#3049 - >Ca_28674.1|sca	multi-dom	185049	1	317	0	688.847	PRK15093	PRK15093	-
Q#3049 - >Ca_28674.1|sca	multi-dom	185067	307	566	0	540.917	PRK15112	PRK15112	-
Q#3050 - >Ca_28683.1|sca	superfamily	276300	19	249	8.74032e-114	329.301	cl21456	Periplasmic_Binding_Protein_Type_2 superfamily	-
Q#3050 - >Ca_28683.1|sca	multi-dom	249906	30	250	1.41155e-43	148.577	pfam00497	SBP_bac_3	-
Q#3051 - >Ca_28688.1|sca	superfamily	271437	277	297	0.00918845	35.3878	cl00213	DNA_BRE_C superfamily	-
Q#3052 - >Ca_28702.1|sca	superfamily	275490	1	141	1.99081e-30	115.269	cl00222	lysozyme_like superfamily	-
Q#3053 - >Ca_28703.1|sca	specific	119392	1	144	8.86403e-06	42.6861	cd06174	MFS	cl21472
Q#3053 - >Ca_28703.1|sca	superfamily	276313	1	144	8.86403e-06	42.6861	cl21472	MFS superfamily	-
Q#3054 - >Ca_28705.1|sca	specific	249873	77	236	7.22193e-50	163.048	pfam00455	DeoRC	cl00339
Q#3054 - >Ca_28705.1|sca	superfamily	275540	77	236	7.22193e-50	163.048	cl00339	SugarP_isomerase superfamily	-
Q#3054 - >Ca_28705.1|sca	superfamily	276302	10	63	7.84787e-09	50.3286	cl21459	HTH_XRE superfamily	-
Q#3054 - >Ca_28705.1|sca	multi-dom	182644	3	254	6.89264e-143	404.079	PRK10681	PRK10681	-
Q#3055 - >Ca_28713.1|sca	specific	100119	541	599	6.96549e-11	58.655	cd06171	Sigma70_r4	cl22432
Q#3055 - >Ca_28713.1|sca	superfamily	276386	541	599	6.96549e-11	58.655	cl22432	Sigma70_r4 superfamily	-
Q#3055 - >Ca_28713.1|sca	specific	252662	139	348	8.41866e-89	276.763	pfam04546	Sigma70_ner	cl04578
Q#3055 - >Ca_28713.1|sca	superfamily	252662	139	348	8.41866e-89	276.763	cl04578	Sigma70_ner superfamily	-
Q#3055 - >Ca_28713.1|sca	specific	146934	458	535	3.8888e-26	102.678	pfam04539	Sigma70_r3	cl04574
Q#3055 - >Ca_28713.1|sca	superfamily	146934	458	535	3.8888e-26	102.678	cl04574	Sigma70_r3 superfamily	-
Q#3055 - >Ca_28713.1|sca	specific	252292	1	73	3.29306e-24	96.963	pfam03979	Sigma70_r1_1	cl04353
Q#3055 - >Ca_28713.1|sca	superfamily	252292	1	73	3.29306e-24	96.963	cl04353	Sigma70_r1_1 superfamily	-
Q#3055 - >Ca_28713.1|sca	specific	252661	379	449	4.51312e-24	96.4913	pfam04542	Sigma70_r2	cl08419
Q#3055 - >Ca_28713.1|sca	superfamily	252661	379	449	4.51312e-24	96.4913	cl08419	Sigma70_r2 superfamily	-
Q#3055 - >Ca_28713.1|sca	specific	249622	97	128	7.59466e-10	55.0809	pfam00140	Sigma70_r1_2	cl02812
Q#3055 - >Ca_28713.1|sca	superfamily	249622	97	128	7.59466e-10	55.0809	cl02812	Sigma70_r1_2 superfamily	-
Q#3055 - >Ca_28713.1|sca	multi-dom	235549	1	613	0	1034.75	PRK05658	PRK05658	-
Q#3056 - >Ca_28721.1|sca	specific	119394	100	254	2.01086e-21	89.6452	cd06261	TM_PBP2	cl00427
Q#3056 - >Ca_28721.1|sca	superfamily	275575	100	254	2.01086e-21	89.6452	cl00427	TM_PBP2 superfamily	-
Q#3056 - >Ca_28721.1|sca	superfamily	276299	300	366	7.60979e-31	117.245	cl21455	ABC_ATPase superfamily	-
Q#3056 - >Ca_28721.1|sca	multi-dom	223836	79	261	2.62162e-57	188.097	COG0765	HisM	-
Q#3056 - >Ca_28721.1|sca	multi-dom	224051	299	366	4.53998e-34	126.854	COG1126	GlnQ	-
Q#3057 - >Ca_28729.1|sca	specific	107296	13	280	5.60689e-123	355.383	cd06301	PBP1_rhizopine_binding_like	cl10011
Q#3057 - >Ca_28729.1|sca	superfamily	275911	13	280	5.60689e-123	355.383	cl10011	Periplasmic_Binding_Protein_Type_1 superfamily	-

Query	Types	PSSM-ID	From	To	E-Value	Bitscore	Accession	Short name	Superfamily
Q#3057 - >Ca_28729.1|sca	multi-dom	257732	13	267	6.65035e-58	188.279	pfam13407	Peripla_BP_4	-
Q#3058 - >Ca_28743.1|sca	specific	237084	1	236	1.7521e-135	383.768	PRK12378	PRK12378	cl00361
Q#3058 - >Ca_28743.1|sca	superfamily	275551	1	236	1.7521e-135	383.768	cl00361	Transcrip_reg superfamily	-
Q#3059 - >Ca_28762.1|sca	specific	251221	754	931	1.66531e-22	96.8132	pfam02321	OEP	cl22292
Q#3059 - >Ca_28762.1|sca	superfamily	251221	754	931	1.66531e-22	96.8132	cl22292	OEP superfamily	-
Q#3059 - >Ca_28762.1|sca	superfamily	273899	280	354	0.000232958	40.7841	cl11690	VCBS_repeat superfamily	-
Q#3059 - >Ca_28762.1|sca	superfamily	251221	958	1063	0.000488352	40.9593	cl22292	OEP superfamily	-
Q#3060 - >Ca_28773.1|sca	specific	270418	20	241	7.49706e-127	361.377	cd13700	PBP2_Arg_STM4351	cl21456
Q#3060 - >Ca_28773.1|sca	superfamily	276300	20	241	7.49706e-127	361.377	cl21456	Periplasmic_Binding_Protein_Type_2 superfamily	-
Q#3060 - >Ca_28773.1|sca	multi-dom	184969	1	241	1.17871e-121	349.33	PRK15007	PRK15007	-
Q#3061 - >Ca_28774.1|sca	specific	119394	48	181	2.22858e-14	69.2296	cd06261	TM_PBP2	cl00427
Q#3061 - >Ca_28774.1|sca	superfamily	275575	48	181	2.22858e-14	69.2296	cl00427	TM_PBP2 superfamily	-
Q#3062 - >Ca_28777.1|sca	specific	206779	307	506	3.36221e-54	183.207	cd11386	MCP_signal	cl21547
Q#3062 - >Ca_28777.1|sca	superfamily	206779	307	506	3.36221e-54	183.207	cl21547	MCP_signal superfamily	-
Q#3062 - >Ca_28777.1|sca	specific	238075	37	127	9.38463e-10	56.1023	cd00130	PAS	cl21578
Q#3062 - >Ca_28777.1|sca	superfamily	271998	37	127	9.38463e-10	56.1023	cl21578	PAS superfamily	-
Q#3062 - >Ca_28777.1|sca	multi-dom	214599	280	526	8.79089e-67	218.694	smart00283	MA	-
Q#3062 - >Ca_28777.1|sca	multi-dom	254805	43	128	2.23638e-13	66.2495	pfam08447	PAS_3	-
Q#3063 - >Ca_28781.1|sca	superfamily	151248	26	73	1.7398e-05	37.774	cl12389	YmgB superfamily	-
Q#3064 - >Ca_28789.1|sca	specific	254749	17	127	3.58446e-50	161.606	pfam08348	PAS_6	cl10076
Q#3064 - >Ca_28789.1|sca	superfamily	254749	17	127	3.58446e-50	161.606	cl10076	PAS_6 superfamily	-
Q#3064 - >Ca_28789.1|sca	specific	257646	154	217	1.15333e-21	85.1875	pfam13309	HTH_22	cl21459
Q#3064 - >Ca_28789.1|sca	superfamily	276302	154	217	1.15333e-21	85.1875	cl21459	HTH_XRE superfamily	-
Q#3064 - >Ca_28789.1|sca	multi-dom	225512	8	224	8.29221e-107	310.023	COG2964	COG2964	-
Q#3065 - >Ca_28811.1|sca	specific	250596	282	412	5.16346e-32	120.776	pfam01408	GFO_IDH_MocA	cl21454
Q#3065 - >Ca_28811.1|sca	superfamily	276298	282	412	5.16346e-32	120.776	cl21454	SDR superfamily	-
Q#3065 - >Ca_28811.1|sca	specific	250484	32	260	2.31234e-23	98.6283	pfam01261	AP_endonuc_2	cl21457
Q#3065 - >Ca_28811.1|sca	superfamily	276301	32	260	2.31234e-23	98.6283	cl21457	TIM_phosphate_binding superfamily	-
Q#3065 - >Ca_28811.1|sca	specific	251601	424	547	6.55702e-09	54.0829	pfam02894	GFO_IDH_MocA_C	cl21649
Q#3065 - >Ca_28811.1|sca	superfamily	272069	424	547	6.55702e-09	54.0829	cl21649	GFO_IDH_MocA_C superfamily	-
Q#3065 - >Ca_28811.1|sca	multi-dom	223745	280	652	3.94477e-61	208.946	COG0673	MviM	-
Q#3066 - >Ca_28819.1|sca	specific	116942	84	202	9.91149e-61	187.936	pfam08361	TetR_C_2	cl22496
Q#3066 - >Ca_28819.1|sca	superfamily	276450	84	202	9.91149e-61	187.936	cl22496	TetR_C_6 superfamily	-
Q#3066 - >Ca_28819.1|sca	specific	249860	16	62	1.20407e-15	67.8186	pfam00440	TetR_N	cl21459
Q#3066 - >Ca_28819.1|sca	superfamily	276302	16	62	1.20407e-15	67.8186	cl21459	HTH_XRE superfamily	-
Q#3066 - >Ca_28819.1|sca	multi-dom	182632	1	208	3.66593e-141	396.299	PRK10668	PRK10668	-

Query	Types	PSSM-ID	From	To	E-Value	Bitscore	Accession	Short name	Superfamily
Q#3067 - >Ca_28828.1|sca	specific	213183	37	253	2.9075e-74	226.156	cd03216	ABC_Carb_Monos_I	cl21455
Q#3067 - >Ca_28828.1|sca	superfamily	276299	37	253	2.9075e-74	226.156	cl21455	ABC_ATPase superfamily	-
Q#3067 - >Ca_28828.1|sca	multi-dom	223488	34	253	1.65757e-53	175.838	COG0411	LivG	-
Q#3068 - >Ca_28832.1|sca	specific	270421	27	253	2.40224e-130	371.196	cd13703	PBP2_HisJ_LAO	cl21456
Q#3068 - >Ca_28832.1|sca	superfamily	276300	27	253	2.40224e-130	371.196	cl21456	Periplasmic_Binding_Protein_Type_2 superfamily	-
Q#3068 - >Ca_28832.1|sca	multi-dom	184972	1	260	1.49091e-156	439.056	PRK15010	PRK15010	-
Q#3069 - >Ca_28851.1|sca	specific	239242	180	491	0	532.84	cd02932	OYE_YqiM_FMN	cl21457
Q#3069 - >Ca_28851.1|sca	superfamily	276301	180	491	0	532.84	cl21457	TIM_phosphate_binding superfamily	-
Q#3069 - >Ca_28851.1|sca	superfamily	276311	1	104	2.65961e-36	131.646	cl21470	Peptidase_M14NE-CP-C_like superfamily	-
Q#3069 - >Ca_28851.1|sca	multi-dom	224814	176	491	1.57886e-123	368.883	COG1902	NemA	-
Q#3070 - >Ca_28864.1|sca	specific	119394	65	249	2.71925e-15	71.926	cd06261	TM_PBP2	cl00427
Q#3070 - >Ca_28864.1|sca	superfamily	275575	65	249	2.71925e-15	71.926	cl00427	TM_PBP2 superfamily	-
Q#3070 - >Ca_28864.1|sca	multi-dom	223674	1	259	1.19127e-95	289.004	COG0601	DppB	-
Q#3071 - >Ca_28866.1|sca	specific	206779	410	605	2.95102e-58	197.845	cd11386	MCP_signal	cl21547
Q#3071 - >Ca_28866.1|sca	superfamily	206779	410	605	2.95102e-58	197.845	cl21547	MCP_signal superfamily	-
Q#3071 - >Ca_28866.1|sca	specific	100122	332	373	1.08115e-07	49.941	cd06225	HAMP	cl01054
Q#3071 - >Ca_28866.1|sca	superfamily	260769	332	373	1.08115e-07	49.941	cl01054	HAMP superfamily	-
Q#3071 - >Ca_28866.1|sca	superfamily	260214	36	154	0.00312089	38.3604	cl00144	Tar_Tsr_LBD superfamily	-
Q#3071 - >Ca_28866.1|sca	multi-dom	223910	280	631	1.68832e-76	256.071	COG0840	Tar	-
Q#3072 - >Ca_28871.1|sca	specific	238213	8	85	9.93799e-31	104.893	cd00353	Ribosomal_S15p_S13e	cl00349
Q#3072 - >Ca_28871.1|sca	superfamily	260373	8	85	9.93799e-31	104.893	cl00349	S15_NS1_EPRS_RNA-bind superfamily	-
Q#3073 - >Ca_28873.1|sca	superfamily	276302	16	102	7.10306e-17	73.003	cl21459	HTH_XRE superfamily	-
Q#3073 - >Ca_28873.1|sca	specific	250746	129	252	8.12069e-15	68.079	pfam01614	IclR	cl08329
Q#3073 - >Ca_28873.1|sca	superfamily	250746	129	252	8.12069e-15	68.079	cl08329	IclR superfamily	-
Q#3073 - >Ca_28873.1|sca	multi-dom	224332	17	254	2.19256e-43	148.91	COG1414	IclR	-
Q#3074 - >Ca_28892.1|sca	specific	213224	318	552	1.46831e-112	338.326	cd03257	ABC_NikE_OppD_transporters	cl21455
Q#3074 - >Ca_28892.1|sca	superfamily	276299	318	552	1.46831e-112	338.326	cl21455	ABC_ATPase superfamily	-
Q#3074 - >Ca_28892.1|sca	specific	213224	21	259	2.48664e-107	324.844	cd03257	ABC_NikE_OppD_transporters	cl21455
Q#3074 - >Ca_28892.1|sca	superfamily	276299	21	259	2.48664e-107	324.844	cl21455	ABC_ATPase superfamily	-
Q#3074 - >Ca_28892.1|sca	specific	254752	549	595	1.6186e-10	57.818	pfam08352	oligo_HPY	cl07097
Q#3074 - >Ca_28892.1|sca	superfamily	263181	549	595	1.6186e-10	57.818	cl07097	oligo_HPY superfamily	-
Q#3074 - >Ca_28892.1|sca	specific	254752	256	298	1.6542e-08	52.04	pfam08352	oligo_HPY	cl07097
Q#3074 - >Ca_28892.1|sca	superfamily	263181	256	298	1.6542e-08	52.04	cl07097	oligo_HPY superfamily	-
Q#3074 - >Ca_28892.1|sca	multi-dom	224048	19	578	0	768.279	COG1123	COG1123	-
Q#3075 - >Ca_28902.1|sca	specific	143635	284	442	2.07908e-44	155.792	cd01949	GGDEF	cl11967
Q#3075 - >Ca_28902.1|sca	superfamily	276010	284	442	2.07908e-44	155.792	cl11967	Nucleotidyl_cyc_III superfamily	-

Query	Types	PSSM-ID	From	To	E-Value	Bitscore	Accession	Short name	Superfamily
Q#3075 - >Ca_28902.1|sca	superfamily	266863	8	267	0.000257592	42.047	cl17823	MASE1 superfamily	-
Q#3076 - >Ca_28904.1|sca	specific	239905	704	768	1.3331e-29	112.672	cd04458	CSP_CDS	cl09927
Q#3076 - >Ca_28904.1|sca	superfamily	275904	704	768	1.3331e-29	112.672	cl09927	S1_like superfamily	-
Q#3076 - >Ca_28904.1|sca	superfamily	263345	575	685	6.94513e-34	127.638	cl07428	Ivy superfamily	-
Q#3076 - >Ca_28904.1|sca	superfamily	276235	74	176	8.31832e-08	52.4347	cl19188	Pectate_lyase_3 superfamily	-
Q#3077 - >Ca_28914.1|sca	specific	99964	479	820	7.44415e-80	260.265	cd03789	GT1_LPS_heptosyltransferase	cl10013
Q#3077 - >Ca_28914.1|sca	superfamily	275912	479	820	7.44415e-80	260.265	cl10013	Glycosyltransferase_GTB_type superfamily	-
Q#3077 - >Ca_28914.1|sca	specific	132997	166	285	5.34118e-33	125.696	cd00761	Glyco_tranf_GTA_type	cl11394
Q#3077 - >Ca_28914.1|sca	superfamily	275925	166	285	5.34118e-33	125.696	cl11394	Glyco_tranf_GTA_type superfamily	-
Q#3077 - >Ca_28914.1|sca	superfamily	275925	1	85	1.84133e-19	88.4258	cl11394	Glyco_tranf_GTA_type superfamily	-
Q#3077 - >Ca_28914.1|sca	superfamily	149484	100	142	0.00988063	34.9804	cl07166	Glyco_transf_8C superfamily	-
Q#3077 - >Ca_28914.1|sca	multi-dom	182223	158	468	0	543.87	PRK10073	PRK10073	-
Q#3077 - >Ca_28914.1|sca	multi-dom	223928	477	823	6.4434e-75	248.826	COG0859	RfaF	-
Q#3077 - >Ca_28914.1|sca	multi-dom	224359	1	138	2.61311e-19	88.9138	COG1442	RfaJ	-
Q#3078 - >Ca_28924.1|sca	specific	153246	112	297	1.08003e-60	193.97	cd07984	LPLAT_LABLAT-like	cl17185
Q#3078 - >Ca_28924.1|sca	superfamily	276196	112	297	1.08003e-60	193.97	cl17185	LPLAT superfamily	-
Q#3079 - >Ca_28940.1|sca	specific	239905	5	68	8.52823e-34	113.828	cd04458	CSP_CDS	cl09927
Q#3079 - >Ca_28940.1|sca	superfamily	275904	5	68	8.52823e-34	113.828	cl09927	S1_like superfamily	-
Q#3080 - >Ca_28944.1|sca	superfamily	275586	15	176	1.65864e-05	45.3539	cl00456	SLC5-6-like_sbd superfamily	-
Q#3080 - >Ca_28944.1|sca	multi-dom	182566	5	452	0	784.394	PRK10580	proY	-
Q#3081 - >Ca_28948.1|sca	specific	100119	410	468	7.31409e-09	52.4918	cd06171	Sigma70_r4	cl22432
Q#3081 - >Ca_28948.1|sca	superfamily	276386	410	468	7.31409e-09	52.4918	cl22432	Sigma70_r4 superfamily	-
Q#3081 - >Ca_28948.1|sca	specific	250701	59	151	8.67584e-39	137.314	pfam01551	Peptidase_M23	cl18370
Q#3081 - >Ca_28948.1|sca	superfamily	250701	59	151	8.67584e-39	137.314	cl18370	Peptidase_M23 superfamily	-
Q#3081 - >Ca_28948.1|sca	specific	252661	248	318	2.25089e-22	91.0985	pfam04542	Sigma70_r2	cl08419
Q#3081 - >Ca_28948.1|sca	superfamily	252661	248	318	2.25089e-22	91.0985	cl08419	Sigma70_r2 superfamily	-
Q#3081 - >Ca_28948.1|sca	specific	146934	327	404	4.87035e-18	79.1811	pfam04539	Sigma70_r3	cl04574
Q#3081 - >Ca_28948.1|sca	superfamily	146934	327	404	4.87035e-18	79.1811	cl04574	Sigma70_r3 superfamily	-
Q#3081 - >Ca_28948.1|sca	specific	249622	210	241	5.43018e-08	49.6881	pfam00140	Sigma70_r1_2	cl02812
Q#3081 - >Ca_28948.1|sca	superfamily	249622	210	241	5.43018e-08	49.6881	cl02812	Sigma70_r1_2 superfamily	-
Q#3081 - >Ca_28948.1|sca	multi-dom	235548	158	481	0	601.174	PRK05657	PRK05657	-
Q#3082 - >Ca_28951.1|sca	superfamily	275750	48	137	1.59333e-31	109.951	cl01888	DUF883 superfamily	-
Q#3083 - >Ca_28954.1|sca	specific	270417	31	274	4.73817e-112	333.186	cd13699	PBP2_OccT_like	cl21456
Q#3083 - >Ca_28954.1|sca	superfamily	276300	31	274	4.73817e-112	333.186	cl21456	Periplasmic_Binding_Protein_Type_2 superfamily	-
Q#3083 - >Ca_28954.1|sca	specific	119394	278	472	4.06704e-19	85.0228	cd06261	TM_PBP2	cl00427
Q#3083 - >Ca_28954.1|sca	superfamily	275575	278	472	4.06704e-19	85.0228	cl00427	TM_PBP2 superfamily	-

Query	Types	PSSM-ID	From	To	E-Value	Bitscore	Accession	Short name	Superfamily
Q#3083 - >Ca_28954.1|sca	multi-dom	226670	276	491	6.77401e-81	253.339	COG4215	ArtQ	-
Q#3083 - >Ca_28954.1|sca	multi-dom	273440	25	274	1.14507e-61	204.128	TIGR01096	ABC_transporter_arginine-binding_protein	-
Q#3084 - >Ca_28955.1|sca	specific	234603	20	231	8.29635e-124	357.598	PRK00056	mtgA	cl19357
Q#3084 - >Ca_28955.1|sca	superfamily	276248	20	231	8.29635e-124	357.598	cl19357	Transgly superfamily	-
Q#3084 - >Ca_28955.1|sca	multi-dom	223815	20	231	2.2521e-67	224.544	COG0744	MrcB	-
Q#3085 - >Ca_28958.1|sca	specific	238923	272	509	5.05213e-82	257.474	cd01948	EAL	cl00290
Q#3085 - >Ca_28958.1|sca	superfamily	260330	272	509	5.05213e-82	257.474	cl00290	EAL superfamily	-
Q#3085 - >Ca_28958.1|sca	specific	257300	50	241	2.13031e-23	97.6045	pfam12792	CSS-motif	cl15094
Q#3085 - >Ca_28958.1|sca	superfamily	257300	50	241	2.13031e-23	97.6045	cl15094	CSS-motif superfamily	-
Q#3085 - >Ca_28958.1|sca	multi-dom	182541	6	515	0	729.867	PRK10551	PRK10551	-
Q#3086 - >Ca_28959.1|sca	specific	189752	80	184	5.08133e-40	133.556	pfam00877	NLPC_P60	cl21534
Q#3086 - >Ca_28959.1|sca	superfamily	276349	80	184	5.08133e-40	133.556	cl21534	NLPC_P60 superfamily	-
Q#3086 - >Ca_28959.1|sca	multi-dom	236773	2	191	9.49556e-122	345.211	PRK10838	spr	-
Q#3087 - >Ca_28961.1|sca	specific	235485	32	259	2.45779e-106	312.7	PRK05471	PRK05471	cl00934
Q#3087 - >Ca_28961.1|sca	superfamily	260705	32	259	2.45779e-106	312.7	cl00934	CDH superfamily	-
Q#3088 - >Ca_28962.1|sca	specific	213229	280	502	1.58813e-123	363.773	cd03262	ABC_HisP_GlnQ	cl21455
Q#3088 - >Ca_28962.1|sca	superfamily	276299	280	502	1.58813e-123	363.773	cl21455	ABC_ATPase superfamily	-
Q#3088 - >Ca_28962.1|sca	specific	119394	64	267	3.35369e-23	96.964	cd06261	TM_PBP2	cl00427
Q#3088 - >Ca_28962.1|sca	superfamily	275575	64	267	3.35369e-23	96.964	cl00427	TM_PBP2 superfamily	-
Q#3088 - >Ca_28962.1|sca	multi-dom	224051	280	526	3.23654e-135	394.567	COG1126	GlnQ	-
Q#3088 - >Ca_28962.1|sca	multi-dom	223836	47	279	3.41316e-78	247.033	COG0765	HisM	-
Q#3089 - >Ca_28973.1|sca	superfamily	275490	88	233	6.91212e-26	101.017	cl00222	lysozyme_like superfamily	-
Q#3090 - >Ca_28984.1|sca	specific	99964	238	540	4.00765e-37	139.698	cd03789	GT1_LPS_heptosyltransferase	cl10013
Q#3090 - >Ca_28984.1|sca	superfamily	275912	238	540	4.00765e-37	139.698	cl10013	Glycosyltransferase_GTB_type superfamily	-
Q#3090 - >Ca_28984.1|sca	superfamily	275578	118	171	2.7908e-26	110.536	cl00445	Iso_dh superfamily	-
Q#3090 - >Ca_28984.1|sca	multi-dom	223928	237	550	1.656e-36	139.429	COG0859	RfaF	-
Q#3091 - >Ca_29004.1|sca	specific	213224	257	480	7.53089e-68	219.299	cd03257	ABC_NikE_OppD_transporters	cl21455
Q#3091 - >Ca_29004.1|sca	superfamily	276299	257	480	7.53089e-68	219.299	cl21455	ABC_ATPase superfamily	-
Q#3091 - >Ca_29004.1|sca	specific	253506	8	246	0	529.55	pfam06007	PhnJ	cl01457
Q#3091 - >Ca_29004.1|sca	superfamily	260945	8	246	0	529.55	cl01457	PhnJ superfamily	-
Q#3091 - >Ca_29004.1|sca	superfamily	263181	477	497	9.05956e-05	40.484	cl07097	oligo_HPY superfamily	-
Q#3091 - >Ca_29004.1|sca	multi-dom	183280	252	497	0	517.171	PRK11701	phnK	-
Q#3092 - >Ca_29006.1|sca	specific	212491	7	189	5.70525e-43	145.89	cd05233	SDR_c	cl21454
Q#3092 - >Ca_29006.1|sca	superfamily	276298	7	189	5.70525e-43	145.89	cl21454	SDR superfamily	-
Q#3092 - >Ca_29006.1|sca	multi-dom	249592	5	172	1.30507e-24	95.6966	pfam00106	adh_short	-
Q#3093 - >Ca_29009.1|sca	specific	249860	15	61	4.2204e-13	60.885	pfam00440	TetR_N	cl21459

Query	Types	PSSM-ID	From	To	E-Value	Bitscore	Accession	Short name	Superfamily
Q#3093 - >Ca_29009.1|sca	superfamily	276302	15	61	4.2204e-13	60.885	cl21459	HTH_XRE superfamily	-
Q#3093 - >Ca_29009.1|sca	multi-dom	224228	1	155	1.70986e-13	65.3449	COG1309	AcrR	-
Q#3094 - >Ca_29031.1|sca	superfamily	276300	30	274	3.56654e-62	197.902	cl21456	Periplasmic_Binding_Protein_Type_2 superfamily	-
Q#3094 - >Ca_29031.1|sca	multi-dom	249906	31	274	7.59122e-64	202.12	pfam00497	SBP_bac_3	-
Q#3095 - >Ca_29034.1|sca	multi-dom	225926	37	118	3.19906e-08	49.5218	COG3391	COG3391	-
Q#3096 - >Ca_29036.1|sca	specific	259858	1	64	2.14023e-27	102.921	cd13836	IHF_B	cl00257
Q#3096 - >Ca_29036.1|sca	superfamily	275502	1	64	2.14023e-27	102.921	cl00257	HU_IHF superfamily	-
Q#3096 - >Ca_29036.1|sca	specific	257884	134	269	6.93563e-09	53.1502	pfam13567	DUF4131	cl16306
Q#3096 - >Ca_29036.1|sca	superfamily	257884	134	269	6.93563e-09	53.1502	cl16306	DUF4131 superfamily	-
Q#3097 - >Ca_29049.1|sca	specific	107251	49	329	5.51094e-147	419.308	cd01538	PBP1_ABC_xylose_binding	cl10011
Q#3097 - >Ca_29049.1|sca	superfamily	275911	49	329	5.51094e-147	419.308	cl10011	Periplasmic_Binding_Protein_Type_1 superfamily	-
Q#3097 - >Ca_29049.1|sca	multi-dom	257732	50	307	2.73101e-66	211.776	pfam13407	Peripla_BP_4	-
Q#3098 - >Ca_29052.1|sca	specific	238923	2	101	2.41579e-46	150.774	cd01948	EAL	cl00290
Q#3098 - >Ca_29052.1|sca	superfamily	260330	2	101	2.41579e-46	150.774	cl00290	EAL superfamily	-
Q#3099 - >Ca_29063.1|sca	specific	213224	1	214	3.50204e-100	292.487	cd03257	ABC_NikE_OppD_transporters	cl21455
Q#3099 - >Ca_29063.1|sca	superfamily	276299	1	214	3.50204e-100	292.487	cl21455	ABC_ATPase superfamily	-
Q#3099 - >Ca_29063.1|sca	multi-dom	224059	1	213	2.28304e-58	185.791	COG1136	SalX	-
Q#3100 - >Ca_29093.1|sca	superfamily	275540	71	185	6.71897e-25	95.6381	cl00339	SugarP_isomerase superfamily	-
Q#3100 - >Ca_29093.1|sca	superfamily	276302	4	56	1.30524e-13	62.2398	cl21459	HTH_XRE superfamily	-
Q#3100 - >Ca_29093.1|sca	multi-dom	224268	1	188	1.44374e-45	152.388	COG1349	GlpR	-
Q#3101 - >Ca_29101.1|sca	specific	238042	62	113	0.000126435	38.819	cd00090	HTH_ARSR	cl21459
Q#3101 - >Ca_29101.1|sca	superfamily	276302	62	113	0.000126435	38.819	cl21459	HTH_XRE superfamily	-
Q#3101 - >Ca_29101.1|sca	specific	250314	111	185	7.54436e-19	78.3447	pfam01037	AsnC_trans_reg	cl21459
Q#3101 - >Ca_29101.1|sca	superfamily	276302	111	185	7.54436e-19	78.3447	cl21459	HTH_XRE superfamily	-
Q#3101 - >Ca_29101.1|sca	multi-dom	224439	58	194	3.30361e-41	140.983	COG1522	Lrp	-
Q#3102 - >Ca_29104.1|sca	specific	132997	6	129	2.98774e-28	107.207	cd00761	Glyco_tranf_GTA_type	cl11394
Q#3102 - >Ca_29104.1|sca	superfamily	275925	6	129	2.98774e-28	107.207	cl11394	Glyco_tranf_GTA_type superfamily	-
Q#3102 - >Ca_29104.1|sca	multi-dom	224137	2	296	1.00115e-14	71.7463	COG1216	COG1216	-
Q#3103 - >Ca_29105.1|sca	specific	270233	17	253	2.93024e-113	328.068	cd01072	PBP2_SMa0082_like	cl21456
Q#3103 - >Ca_29105.1|sca	superfamily	276300	17	253	2.93024e-113	328.068	cl21456	Periplasmic_Binding_Protein_Type_2 superfamily	-
Q#3103 - >Ca_29105.1|sca	multi-dom	249906	31	247	1.18258e-55	180.164	pfam00497	SBP_bac_3	-
Q#3104 - >Ca_29134.1|sca	specific	173869	40	537	0	603.007	cd08504	PBP2_OppA	cl01709
Q#3104 - >Ca_29134.1|sca	superfamily	275744	40	537	0	603.007	cl01709	PBP2_NikA_DppA_OppA_like superfamily	-
Q#3104 - >Ca_29134.1|sca	multi-dom	185059	4	532	0	839.051	PRK15104	PRK15104	-
Q#3105 - >Ca_29138.1|sca	specific	143635	222	373	8.12315e-55	179.675	cd01949	GGDEF	cl11967
Q#3105 - >Ca_29138.1|sca	superfamily	276010	222	373	8.12315e-55	179.675	cl11967	Nucleotidyl_cyc_III superfamily	-

Query	Types	PSSM-ID	From	To	E-Value	Bitscore	Accession	Short name	Superfamily
Q#3106 - >Ca_29139.1|sca	specific	213224	3	236	7.64998e-109	317.14	cd03257	ABC_NikE_OppD_transporters	cl21455
Q#3106 - >Ca_29139.1|sca	superfamily	276299	3	236	7.64998e-109	317.14	cl21455	ABC_ATPase superfamily	-
Q#3106 - >Ca_29139.1|sca	superfamily	263181	231	275	1.30807e-12	61.6091	cl07097	oligo_HPY superfamily	-
Q#3106 - >Ca_29139.1|sca	multi-dom	224049	1	260	4.00626e-87	262.586	COG1124	DppF	-
Q#3107 - >Ca_29143.1|sca	specific	153118	53	196	1.23552e-55	180.462	cd07909	YciF	cl00264
Q#3107 - >Ca_29143.1|sca	superfamily	275506	53	196	1.23552e-55	180.462	cl00264	Ferritin_like superfamily	-
Q#3107 - >Ca_29143.1|sca	specific	226210	179	339	3.85838e-40	140.565	COG3685	COG3685	cl00264
Q#3107 - >Ca_29143.1|sca	superfamily	275506	179	339	3.85838e-40	140.565	cl00264	Ferritin_like superfamily	-
Q#3107 - >Ca_29143.1|sca	specific	226252	1	47	1.88708e-17	75.5913	COG3729	GsiB	cl01514
Q#3107 - >Ca_29143.1|sca	superfamily	226252	1	47	1.88708e-17	75.5913	cl01514	GsiB superfamily	-
Q#3108 - >Ca_29148.1|sca	superfamily	151248	50	98	6.64991e-11	53.182	cl12389	YmgB superfamily	-
Q#3109 - >Ca_29154.1|sca	specific	238045	128	181	4.01031e-10	53.3258	cd00093	HTH_XRE	cl21459
Q#3109 - >Ca_29154.1|sca	superfamily	276302	128	181	4.01031e-10	53.3258	cl21459	HTH_XRE superfamily	-
Q#3109 - >Ca_29154.1|sca	superfamily	276329	7	96	3.76577e-34	119.955	cl21503	Plasmid_stabil superfamily	-
Q#3110 - >Ca_29157.1|sca	specific	187632	265	501	2.19782e-86	269.486	cd05374	17beta-HSD-like_SDR_c	cl21454
Q#3110 - >Ca_29157.1|sca	superfamily	276298	265	501	2.19782e-86	269.486	cl21454	SDR superfamily	-
Q#3110 - >Ca_29157.1|sca	specific	212491	6	196	4.09647e-42	151.282	cd05233	SDR_c	cl21454
Q#3110 - >Ca_29157.1|sca	superfamily	276298	6	196	4.09647e-42	151.282	cl21454	SDR superfamily	-
Q#3110 - >Ca_29157.1|sca	multi-dom	235725	261	530	1.95657e-158	455.131	PRK06179	PRK06179	-
Q#3110 - >Ca_29157.1|sca	multi-dom	223377	1	258	1.55172e-82	260.257	COG0300	DltE	-
Q#3111 - >Ca_29159.1|sca	specific	119394	70	197	2.37973e-11	58.444	cd06261	TM_PBP2	cl00427
Q#3111 - >Ca_29159.1|sca	superfamily	275575	70	197	2.37973e-11	58.444	cl00427	TM_PBP2 superfamily	-
Q#3111 - >Ca_29159.1|sca	multi-dom	223673	8	197	6.35995e-65	202.838	COG0600	TauC	-
Q#3112 - >Ca_29160.1|sca	specific	153246	379	570	1.12218e-62	206.682	cd07984	LPLAT_LABLAT-like	cl17185
Q#3112 - >Ca_29160.1|sca	superfamily	276196	379	570	1.12218e-62	206.682	cl17185	LPLAT superfamily	-
Q#3112 - >Ca_29160.1|sca	specific	252148	1	151	1.21689e-59	197.36	pfam03755	YicC_N	cl04245
Q#3112 - >Ca_29160.1|sca	superfamily	252148	1	151	1.21689e-59	197.36	cl04245	YicC_N superfamily	-
Q#3112 - >Ca_29160.1|sca	specific	254742	198	282	1.01371e-45	156.431	pfam08340	DUF1732	cl07087
Q#3112 - >Ca_29160.1|sca	superfamily	254742	198	282	1.01371e-45	156.431	cl07087	DUF1732 superfamily	-
Q#3112 - >Ca_29160.1|sca	multi-dom	236993	1	282	1.83199e-151	439.968	PRK11820	PRK11820	-
Q#3113 - >Ca_29187.1|sca	superfamily	275744	37	156	3.24418e-55	182.369	cl01709	PBP2_NikA_DppA_OppA_like superfamily	-
Q#3114 - >Ca_29215.1|sca	specific	257986	78	144	2.25888e-34	116.872	pfam13683	rve_3	cl21549
Q#3114 - >Ca_29215.1|sca	superfamily	276355	78	144	2.25888e-34	116.872	cl21549	rve superfamily	-
Q#3114 - >Ca_29215.1|sca	specific	250040	1	107	2.88942e-19	78.9144	pfam00665	rve	cl21549
Q#3114 - >Ca_29215.1|sca	superfamily	276355	1	107	2.88942e-19	78.9144	cl21549	rve superfamily	-
Q#3114 - >Ca_29215.1|sca	multi-dom	225361	1	154	7.25516e-19	79.9992	COG2801	Tra5	-

Query	Types	PSSM-ID	From	To	E-Value	Bitscore	Accession	Short name	Superfamily
Q#3115 - >Ca_29217.1|sca	specific	250746	74	182	1.71283e-39	136.644	pfam01614	IclR	cl08329
Q#3115 - >Ca_29217.1|sca	superfamily	250746	74	182	1.71283e-39	136.644	cl08329	IclR superfamily	-
Q#3115 - >Ca_29217.1|sca	specific	258226	258	314	3.45583e-22	88.1801	pfam13974	YebO	cl16513
Q#3115 - >Ca_29217.1|sca	superfamily	258226	258	314	3.45583e-22	88.1801	cl16513	YebO superfamily	-
Q#3115 - >Ca_29217.1|sca	superfamily	276302	1	48	2.83764e-11	58.7506	cl21459	HTH_XRE superfamily	-
Q#3116 - >Ca_29243.1|sca	specific	119394	67	157	3.99381e-10	54.2069	cd06261	TM_PBP2	cl00427
Q#3116 - >Ca_29243.1|sca	superfamily	275575	67	157	3.99381e-10	54.2069	cl00427	TM_PBP2 superfamily	-
Q#3119 - >Ca_29266.1|sca	specific	258132	191	356	2.98467e-51	171.623	pfam13862	BCIP	cl16439
Q#3119 - >Ca_29266.1|sca	superfamily	258132	191	356	2.98467e-51	171.623	cl16439	BCIP superfamily	-
Q#3122 - >Ca_29279.1|sca	specific	253250	162	186	3.06839e-09	49.7969	pfam05553	DUF761	cl05229
Q#3122 - >Ca_29279.1|sca	superfamily	253250	162	186	3.06839e-09	49.7969	cl05229	DUF761 superfamily	-
Q#3123 - >Ca_29284.1|sca	specific	238926	30	118	1.24442e-36	122.073	cd01960	nsLTP1	cl07890
Q#3123 - >Ca_29284.1|sca	superfamily	275845	30	118	1.24442e-36	122.073	cl07890	AAI_LTSS superfamily	-
Q#3126 - >Ca_29301.1|sca	multi-dom	273731	47	161	1.64521e-23	97.0125	TIGR01647	Plasma_membrane_ATPase	-
Q#3127 - >Ca_29305.1|sca	superfamily	276299	43	120	3.38218e-21	84.5249	cl21455	ABC_ATPase superfamily	-
Q#3128 - >Ca_29306.1|sca	superfamily	276299	43	120	3.3713e-21	84.5249	cl21455	ABC_ATPase superfamily	-
Q#3129 - >Ca_29307.1|sca	multi-dom	177805	4	129	7.34861e-12	60.3542	PLN00411	PLN00411	-
Q#3130 - >Ca_29333.1|sca	superfamily	276299	55	107	2.58496e-22	87.1549	cl21455	ABC_ATPase superfamily	-
Q#3130 - >Ca_29333.1|sca	superfamily	271701	1	43	4.80874e-15	66.5944	cl10459	Peptidases_S8_S53 superfamily	-
Q#3131 - >Ca_29334.1|sca	multi-dom	250737	15	134	1.92015e-32	119.25	pfam01602	Adaptin_N	-
Q#3134 - >Ca_29379.1|sca	superfamily	271848	1	270	1.86611e-66	215.713	cl19241	Condensation superfamily	-
Q#3135 - >Ca_29382.1|sca	superfamily	260253	1	94	1.66593e-54	175.928	cl00198	Phosphoglycerate_kinase superfamily	-
Q#3136 - >Ca_29406.1|sca	multi-dom	250737	15	134	1.92015e-32	119.25	pfam01602	Adaptin_N	-
Q#3141 - >Ca_29470.1|sca	superfamily	271411	16	201	6.43146e-50	166.696	cl00042	CASc superfamily	-
Q#3142 - >Ca_29471.1|sca	specific	257949	69	93	1.53011e-05	37.7511	pfam13639	zf-RING_2	cl17238
Q#3142 - >Ca_29471.1|sca	superfamily	276201	69	93	1.53011e-05	37.7511	cl17238	RING superfamily	-
Q#3145 - >Ca_29520.1|sca	superfamily	271754	323	396	1.93748e-12	64.4361	cl14782	RNase_H_like superfamily	-
Q#3146 - >Ca_29525.1|sca	specific	257974	12	69	9.39505e-16	69.5451	pfam13668	Ferritin_2	cl00264
Q#3146 - >Ca_29525.1|sca	superfamily	275506	12	69	9.39505e-16	69.5451	cl00264	Ferritin_like superfamily	-
Q#3147 - >Ca_29526.1|sca	superfamily	276298	36	109	9.11242e-36	125.409	cl21454	SDR superfamily	-
Q#3148 - >Ca_29527.1|sca	specific	133136	101	154	2.52461e-06	44.2496	cd00303	retropepsin_like	cl11403
Q#3148 - >Ca_29527.1|sca	superfamily	275927	101	154	2.52461e-06	44.2496	cl11403	pepsin_retropepsin_like superfamily	-
Q#3151 - >Ca_29539.1|sca	superfamily	276656	171	220	4.77877e-12	59.7017	cl22702	CaM_binding superfamily	-
Q#3153 - >Ca_29545.1|sca	superfamily	256046	14	90	1.18483e-11	58.56	cl11168	PMD superfamily	-
Q#3159 - >Ca_29574.1|sca	superfamily	276297	32	139	3.18277e-43	145.831	cl21453	PKc_like superfamily	-
Q#3159 - >Ca_29574.1|sca	superfamily	276299	138	167	7.66819e-07	44.8759	cl21455	ABC_ATPase superfamily	-

Query	Types	PSSM-ID	From	To	E-Value	Bitscore	Accession	Short name	Superfamily
Q#3163 - >Ca_29612.1|sca	superfamily	275808	33	78	4.04782e-08	47.4329	cl04375	PMEI_like superfamily	-
Q#3165 - >Ca_29618.1|sca	superfamily	253682	93	205	0.00307402	36.9619	cl09408	Med13_C superfamily	-
Q#3166 - >Ca_29621.1|sca	specific	250040	157	262	3.26493e-11	59.2693	pfam00665	rve	cl21549
Q#3166 - >Ca_29621.1|sca	superfamily	276355	157	262	3.26493e-11	59.2693	cl21549	rve superfamily	-
Q#3168 - >Ca_29629.1|sca	superfamily	276313	27	161	2.67079e-19	83.1318	cl21472	MFS superfamily	-
Q#3170 - >Ca_29638.1|sca	superfamily	275633	1	87	1.27062e-17	75.8907	cl00600	Ribosomal_L7Ae superfamily	-
Q#3171 - >Ca_29643.1|sca	superfamily	251669	172	257	3.20071e-10	56.2688	cl03830	Transposase_24 superfamily	-
Q#3171 - >Ca_29643.1|sca	superfamily	258207	34	91	2.59274e-09	52.5835	cl16495	DUF4216 superfamily	-
Q#3172 - >Ca_29653.1|sca	superfamily	276299	55	168	7.58795e-43	143.394	cl21455	ABC_ATPase superfamily	-
Q#3172 - >Ca_29653.1|sca	superfamily	271701	1	51	2.41051e-13	63.1276	cl10459	Peptidases_S8_S53 superfamily	-
Q#3173 - >Ca_29657.1|sca	specific	238204	29	230	3.92147e-58	185.92	cd00333	MIP	cl00200
Q#3173 - >Ca_29657.1|sca	superfamily	275482	29	230	3.92147e-58	185.92	cl00200	MIP superfamily	-
Q#3174 - >Ca_29662.1|sca	superfamily	276263	122	317	9.91522e-17	77.3996	cl19514	FBA_1 superfamily	-
Q#3174 - >Ca_29662.1|sca	specific	250027	18	58	5.84284e-06	43.3474	pfam00646	F-box	cl02535
Q#3174 - >Ca_29662.1|sca	superfamily	261329	18	58	5.84284e-06	43.3474	cl02535	F-box superfamily	-
Q#3177 - >Ca_29666.1|sca	specific	206754	1	83	5.58723e-07	45.0792	cd06223	PRTases_typeI	cl00309
Q#3177 - >Ca_29666.1|sca	superfamily	275521	1	83	5.58723e-07	45.0792	cl00309	PRTases_typeI superfamily	-
Q#3178 - >Ca_29671.1|sca	specific	197867	26	106	2.02681e-31	107.726	smart00768	X8	cl06842
Q#3178 - >Ca_29671.1|sca	superfamily	263067	26	106	2.02681e-31	107.726	cl06842	X8 superfamily	-
Q#3179 - >Ca_29672.1|sca	specific	190559	6	106	5.45154e-47	155.428	pfam03195	DUF260	cl03929
Q#3179 - >Ca_29672.1|sca	superfamily	190559	6	106	5.45154e-47	155.428	cl03929	DUF260 superfamily	-
Q#3179 - >Ca_29672.1|sca	superfamily	190037	84	166	0.00453186	36.5476	cl20137	Zein superfamily	-
Q#3180 - >Ca_29676.1|sca	superfamily	252133	73	142	7.10934e-09	50.4149	cl04237	Retrotrans_gag superfamily	-
Q#3183 - >Ca_29695.1|sca	superfamily	275476	91	116	1.45792e-05	40.1778	cl00155	UBQ superfamily	-
Q#3187 - >Ca_29706.1|sca	superfamily	260792	1	84	9.11066e-35	119.63	cl01109	SYLF superfamily	-
Q#3190 - >Ca_29718.1|sca	multi-dom	223066	212	342	0.00405666	37.7305	PHA03379	PHA03379	-
Q#3197 - >Ca_29771.1|sca	specific	238008	85	147	1.47397e-21	82.9809	cd00051	EFh	cl08302
Q#3197 - >Ca_29771.1|sca	superfamily	275859	85	147	1.47397e-21	82.9809	cl08302	EFh superfamily	-
Q#3197 - >Ca_29771.1|sca	specific	238008	12	74	7.97083e-20	78.3585	cd00051	EFh	cl08302
Q#3197 - >Ca_29771.1|sca	superfamily	275859	12	74	7.97083e-20	78.3585	cl08302	EFh superfamily	-
Q#3197 - >Ca_29771.1|sca	multi-dom	185504	1	149	4.28342e-90	261.234	PTZ00184	PTZ00184	-
Q#3201 - >Ca_29800.1|sca	superfamily	253459	43	83	1.33684e-07	46.9961	cl05469	Mitovir_RNA_pol superfamily	-
Q#3202 - >Ca_29803.1|sca	multi-dom	223066	8	158	0.00717586	36.1897	PHA03379	PHA03379	-
Q#3205 - >Ca_29818.1|sca	superfamily	256046	3	58	2.73948e-12	62.0268	cl11168	PMD superfamily	-
Q#3205 - >Ca_29818.1|sca	superfamily	256046	81	145	1.20664e-07	48.1596	cl11168	PMD superfamily	-
Q#3206 - >Ca_29819.1|sca	superfamily	276297	200	369	1.21467e-60	198.266	cl21453	PKc_like superfamily	-

Query	Types	PSSM-ID	From	To	E-Value	Bitscore	Accession	Short name	Superfamily
Q#3206 - >Ca_29819.1|sca	superfamily	201524	44	121	2.67167e-07	48.0453	cl03036	S_locus_glycop superfamily	-
Q#3210 - >Ca_29829.1|sca	multi-dom	215061	7	155	1.45308e-18	82.2032	PLN00113	PLN00113	-
Q#3211 - >Ca_29830.1|sca	multi-dom	236669	18	157	0.00552525	36.2163	PRK10263	PRK10263	-
Q#3212 - >Ca_29833.1|sca	specific	238068	69	192	2.55244e-33	121.717	cd00121	MATH	cl02446
Q#3212 - >Ca_29833.1|sca	superfamily	261289	69	192	2.55244e-33	121.717	cl02446	MATH superfamily	-
Q#3216 - >Ca_29854.1|sca	specific	189387	174	190	0.000709348	35.8929	pfam00098	zf-CCHC	cl22700
Q#3216 - >Ca_29854.1|sca	superfamily	276654	174	190	0.000709348	35.8929	cl22700	zf-CCHC superfamily	-
Q#3219 - >Ca_29866.1|sca	multi-dom	173561	150	185	1.06394e-05	42.8717	PTZ00368	PTZ00368	-
Q#3222 - >Ca_29870.1|sca	superfamily	276299	82	170	2.23028e-29	108.341	cl21455	ABC_ATPase superfamily	-
Q#3222 - >Ca_29870.1|sca	superfamily	271701	1	43	1.53744e-09	52.342	cl10459	Peptidases_S8_S53 superfamily	-
Q#3226 - >Ca_29895.1|sca	multi-dom	258434	42	111	5.56821e-13	62.3706	pfam14244	UBN2_3	-
Q#3228 - >Ca_29903.1|sca	superfamily	276297	21	159	7.87969e-38	133.552	cl21453	PKc_like superfamily	-
Q#3228 - >Ca_29903.1|sca	superfamily	271773	160	177	0.00898074	33.6266	cl15354	CBS_pair superfamily	-
Q#3229 - >Ca_29906.1|sca	superfamily	276214	19	138	3.26185e-46	154.657	cl18310	NHL superfamily	-
Q#3230 - >Ca_29908.1|sca	superfamily	260792	1	80	3.86122e-33	114.238	cl01109	SYLF superfamily	-
Q#3232 - >Ca_29913.1|sca	superfamily	271754	96	194	8.02705e-06	43.6353	cl14782	RNase_H_like superfamily	-
Q#3233 - >Ca_29928.1|sca	multi-dom	258434	48	110	6.37335e-17	71.2302	pfam14244	UBN2_3	-
Q#3234 - >Ca_29929.1|sca	specific	189387	31	45	0.000842024	33.5817	pfam00098	zf-CCHC	cl22700
Q#3234 - >Ca_29929.1|sca	superfamily	276654	31	45	0.000842024	33.5817	cl22700	zf-CCHC superfamily	-
Q#3235 - >Ca_29930.1|sca	superfamily	258215	8	30	0.00012429	36.5364	cl16504	Transpos_assoc superfamily	-
Q#3236 - >Ca_29933.1|sca	specific	252648	113	237	1.96861e-38	132.544	pfam04520	Senescence_reg	cl04561
Q#3236 - >Ca_29933.1|sca	superfamily	252648	113	237	1.96861e-38	132.544	cl04561	Senescence_reg superfamily	-
Q#3237 - >Ca_29942.1|sca	superfamily	260211	24	293	5.63713e-104	311.28	cl00137	SERPIN superfamily	-
Q#3239 - >Ca_29970.1|sca	superfamily	250754	1	75	5.41821e-11	55.2739	cl03286	MutS_I superfamily	-
Q#3241 - >Ca_29978.1|sca	superfamily	276299	78	152	2.27316e-17	75.2137	cl21455	ABC_ATPase superfamily	-
Q#3241 - >Ca_29978.1|sca	superfamily	271701	4	47	4.90551e-09	50.416	cl10459	Peptidases_S8_S53 superfamily	-
Q#3243 - >Ca_29986.1|sca	specific	211392	20	158	1.40887e-45	149.207	cd11380	Ribosomal_S8e_like	cl00880
Q#3243 - >Ca_29986.1|sca	superfamily	260676	20	158	1.40887e-45	149.207	cl00880	Ribosomal_S8e_like superfamily	-
Q#3244 - >Ca_29989.1|sca	specific	214842	114	178	1.00212e-15	69.5338	smart00831	Cation_ATPase_N	cl02930
Q#3244 - >Ca_29989.1|sca	superfamily	261517	114	178	1.00212e-15	69.5338	cl02930	Cation_ATPase_N superfamily	-
Q#3245 - >Ca_29990.1|sca	specific	107229	54	145	1.81297e-56	174.415	cd06472	ACD_ScHsp26_like	cl00175
Q#3245 - >Ca_29990.1|sca	superfamily	271432	54	145	1.81297e-56	174.415	cl00175	alpha-crystallin-Hsps_p23-like superfamily	-
Q#3245 - >Ca_29990.1|sca	multi-dom	223149	21	159	2.10945e-27	100.914	COG0071	IbpA	-
Q#3246 - >Ca_29992.1|sca	superfamily	275927	86	317	1.58608e-33	125.068	cl11403	pepsin_retropepsin_like superfamily	-
Q#3247 - >Ca_29993.1|sca	specific	224739	137	169	0.00430742	34.668	COG1826	TatA	cl00788
Q#3247 - >Ca_29993.1|sca	superfamily	275655	137	169	0.00430742	34.668	cl00788	MttA_Hcf106 superfamily	-

Query	Types	PSSM-ID	From	To	E-Value	Bitscore	Accession	Short name	Superfamily
Q#3249 - >Ca_29996.1|sca	superfamily	271773	53	80	2.86883e-08	46.8412	cl15354	CBS_pair superfamily	-
Q#3251 - >Ca_30010.1|sca	specific	238037	19	70	1.23096e-07	44.1468	cd00084	HMG-box	cl00082
Q#3251 - >Ca_30010.1|sca	superfamily	260179	19	70	1.23096e-07	44.1468	cl00082	HMG-box superfamily	-
Q#3252 - >Ca_30016.1|sca	specific	211392	8	106	8.88222e-37	123.399	cd11380	Ribosomal_S8e_like	cl00880
Q#3252 - >Ca_30016.1|sca	superfamily	260676	8	106	8.88222e-37	123.399	cl00880	Ribosomal_S8e_like superfamily	-
Q#3253 - >Ca_30023.1|sca	multi-dom	258063	12	65	0.00379391	33.7262	pfam13779	DUF4175	-
Q#3254 - >Ca_30025.1|sca	superfamily	275544	27	132	5.2651e-35	119.744	cl00350	Ribosomal_S19 superfamily	-
Q#3258 - >Ca_30053.1|sca	superfamily	276299	57	93	1.19398e-05	40.5458	cl21455	ABC_ATPase superfamily	-
Q#3260 - >Ca_30059.1|sca	multi-dom	273328	39	134	3.25344e-07	46.9327	TIGR00898	organic_anion_transporter	-
Q#3262 - >Ca_30063.1|sca	superfamily	275586	92	269	3.94189e-37	137.429	cl00456	SLC5-6-like_sbd superfamily	-
Q#3263 - >Ca_30064.1|sca	superfamily	265686	12	57	8.40934e-07	43.8178	cl15397	DUF89 superfamily	-
Q#3264 - >Ca_30067.1|sca	specific	239914	86	156	1.58661e-29	104.542	cd04468	S1_eIF5A	cl09927
Q#3264 - >Ca_30067.1|sca	superfamily	275904	86	156	1.58661e-29	104.542	cl09927	S1_like superfamily	-
Q#3264 - >Ca_30067.1|sca	multi-dom	215580	1	159	3.83226e-113	320.888	PLN03107	PLN03107	-
Q#3267 - >Ca_30079.1|sca	superfamily	252133	76	125	0.000459528	37.3181	cl04237	Retrotrans_gag superfamily	-
Q#3268 - >Ca_30080.1|sca	superfamily	261267	30	93	9.67022e-15	65.6297	cl02381	Tim17 superfamily	-
Q#3269 - >Ca_30096.1|sca	superfamily	252344	100	149	5.76711e-09	53.0206	cl04384	DUF383 superfamily	-
Q#3269 - >Ca_30096.1|sca	specific	250181	213	249	0.00146491	36.3223	pfam00856	SET	cl02566
Q#3269 - >Ca_30096.1|sca	superfamily	261347	213	249	0.00146491	36.3223	cl02566	SET superfamily	-
Q#3271 - >Ca_30103.1|sca	superfamily	256046	33	155	9.94991e-36	130.207	cl11168	PMD superfamily	-
Q#3272 - >Ca_30104.1|sca	multi-dom	237862	1	80	0.0020385	38.0201	PRK14948	PRK14948	-
Q#3279 - >Ca_30162.1|sca	multi-dom	139494	3	85	6.20893e-06	44.3466	PRK13335	PRK13335	-
Q#3280 - >Ca_30173.1|sca	superfamily	271838	50	133	9.36145e-38	135.995	cl19114	RNAP_largest_subunit_N superfamily	-
Q#3282 - >Ca_30194.1|sca	multi-dom	215599	45	102	1.02123e-09	54.4669	PLN03140	PLN03140	-
Q#3286 - >Ca_30206.1|sca	superfamily	267728	131	179	0.00058621	38.9238	cl19375	HemX superfamily	-
Q#3289 - >Ca_30213.1|sca	multi-dom	223222	55	100	0.00207827	36.5989	COG0144	Sun	-
Q#3292 - >Ca_30237.1|sca	superfamily	256046	3	106	4.09398e-24	93.6132	cl11168	PMD superfamily	-
Q#3296 - >Ca_30257.1|sca	specific	133136	98	151	1.85738e-08	48.4868	cd00303	retropepsin_like	cl11403
Q#3296 - >Ca_30257.1|sca	superfamily	275927	98	151	1.85738e-08	48.4868	cl11403	pepsin_retropepsin_like superfamily	-

Supplementary Table S15. Intra-specific SNPs within 39 *desi* group and 53 *kabuli* group of chickpea genotypes as derived by mapping GBS sequence data on ICC 4958 genome assembly.

**LGs**	***Kabuli***	***Desi***
Ca_LG1	371	345
Ca_LG2	214	227
Ca_LG3	270	233
Ca_LG4	358	377
Ca_LG5	306	289
Ca_LG6	336	319
Ca_LG7	266	293
Ca_LG8	78	67
**Total**	**2199**	**2150**

Supplementary Table S16. SNPs differentiating the accessions belonging to *desi* and *kabuli* chickpea cultivars

**Positions**	**-100 bp flanking sequences**	**SNPs within *desi* cultivar**	**+100 bp flanking sequences**
Ca_LG_1:217652	TGCTTGCCGGTCGATATCTTCAATATCTTTAAAAGATAGCTTAATGCCTAAGTGCGCTTGTATTGCAGCAACATCCTGAACAACTGAATTCAGAGATGCT	T/C	TGGAGATTCGTCAAGGCAGCTTCCATTCTAGCCAGTGACTCTTGCATTTTGGCGTTGTGAGCCATCTGAACTTCCATAAATTCCAGCAGTTTTGCGACGT
Ca_LG_1:799854	CCTAAATTGTGTCTGCAATGCCGTTGTGTAGCCTTCAAAATCCGCAATATTGCGGCTGCAATTGTGGTTGCAGACCGCAATTTACAATTGTGTTGCAGTT	T/C	GAGTTCCACTTTGCACTGCGATTCCGCGCTACAGCGGCACGATTGACATCGTAGCTTAATTTCATACGTCGTTGCTCGTTTTCTGTGCTCTAAATTTTGA
Ca_LG_1:799879	GTGTAGCCTTCAAAATCCGCAATATTGCGGCTGCAATTGTGGTTGCAGACCGCAATTTACAATTGTGTTGCAGTTGAGTTCCACTTTGCACTGCGATTCC	C/T	GCGCTACAGCGGCACGATTGACATCGTAGCTTAATTTCATACGTCGTTGCTCGTTTTCTGTGCTCTAAATTTTGATTTCACATCCTTAGGTATCTATAGT
Ca_LG_1:806676	AAAATAAGTGACAATAACAAAATTATAAGCTGCTATGTCCTATATCAACCTTAAAATAATTCATGTTTCATAAGTCATATATCAAACTGACAGAAAAATC	C/G	GTTGCTTATACAAAGTCGACAAAAAAAAGTGTTTGCTGCACTAAGTTTAGGTGTTGTATTCATTAGAATGAATCAAGATTCAAGATGGAAGCCATGTCCA
Ca_LG_1:806694	AAAATTATAAGCTGCTATGTCCTATATCAACCTTAAAATAATTCATGTTTCATAAGTCATATATCAAACTGACAGAAAAATCGTTGCTTATACAAAGTCG	G/A	ACAAAAAAAAGTGTTTGCTGCACTAAGTTTAGGTGTTGTATTCATTAGAATGAATCAAGATTCAAGATGGAAGCCATGTCCAAATTGAATCAAGATACAA
Ca_LG_1:1187336	TATATATCACAAATACCACTATTTGTTGCAAGTTTAAGAGAGAGTTAATACAACTCCCAAGAACACAAAGTAGATGTTGGCTGCTTCCTAAATAGTAAGG	G/A	CATGATGGAGTCTCCTCCATCAGTGGGCAATCAAAGATCCACGTAAATGTTGGCAGCTTCCTAAATGGTAAGACATGATGGAGAGTAATAGCAAGGGACT
Ca_LG_1:1411903	GGAACTTGGTGTATCATCACTTGCAGATGCGATAACTTGCCGAATAGCTGCTCGGGATGCCTTGTAGAAGAATGATTGTGATATGCTTGTATACTGACTT	T/G	TTTTATTACCAAATCTAATTTGTCTTGGAATTTAATAAGCCTCTAGCTGCTAGCCTTGTAGCACAGCCTTATCTTGTAGGAATGAATTATTAGTTGATCT
Ca_LG_1:1411919	TCACTTGCAGATGCGATAACTTGCCGAATAGCTGCTCGGGATGCCTTGTAGAAGAATGATTGTGATATGCTTGTATACTGACTTTTTTATTACCAAATCT	T/C	AATTTGTCTTGGAATTTAATAAGCCTCTAGCTGCTAGCCTTGTAGCACAGCCTTATCTTGTAGGAATGAATTATTAGTTGATCTAGTATTAAACATGATT
Ca_LG_1:1434100	TGCAGCGTCCATTCCATTGGCTTTAGATGAAGCAGTTAGAAGTGGGAAAGTTAAGGCAGGACAAACTATTGCAGCTGCTGGCTTTGGTGCCGGTCTTACT	T/A	TGGGGTTCGGCAATTGTTCGTTGGGGCTGAGTCGTCTTTCTCAGCAGCATTGCATTTGCAAATAGCCGGCATTTTCAAGTTCAAGTGCAACTCTCTAAAG
Ca_LG_1:1434109	CATTCCATTGGCTTTAGATGAAGCAGTTAGAAGTGGGAAAGTTAAGGCAGGACAAACTATTGCAGCTGCTGGCTTTGGTGCCGGTCTTACTTGGGGTTCG	G/A	GCAATTGTTCGTTGGGGCTGAGTCGTCTTTCTCAGCAGCATTGCATTTGCAAATAGCCGGCATTTTCAAGTTCAAGTGCAACTCTCTAAAGAATCCAATT
Ca_LG_1:1436661	CTGCAAGGCATGTAGTTGCAAAAATATTATATTAATTAATATATTAAAGTGTCCATTGTAACACCATTTTGTCAGATTATCATCAGGAATTACAATAATC	C/A	CCTCGAGCAGCAACAACTGTTTCTAATGTGGAGGTATTTTATTTATTAAATAAATGATTTGCATGTGAATAAAGCATTATATAGTAATTGAATTTAAATA
Ca_LG_1:1464710	CTAATTTTCTTCTTATTACTAATGCAGCTACTTAGTTTTTCTTATGTTGCAATTGGATTTTGTTCATTCATCTTAATCTTAGAAATAGTATTTTTTTTTT	T/A	ATACGAAATCTAGACCTATATTTCCATTCTCGCTTATTAAAGATTTGTTGTTCGTTTTTGCTGCAGAATATGGTTGCCCCAAGTTCAAGTTCAGGTTCAG

Positions	-100 bp flanking sequences	SNPs within *desi* cultivar	+100 bp flanking sequences
Ca_LG_1:1464725	TTACTAATGCAGCTACTTAGTTTTTCTTATGTTGCAATTGGATTTTGTTCATTCATCTTAATCTTAGAAATAGTATTTTTTTTTTATACGAAATCTAGAC	C/A	CTATATTTCCATTCTCGCTTATTAAAGATTTGTTGTTCGTTTTTGCTGCAGAATATGGTTGCCCCAAGTTCAAGTTCAGGTTCAGGTTCAGGTTCAGGTT
Ca_LG_1:1464792	AAATAGTATTTTTTTTTTATACGAAATCTAGACCTATATTTCCATTCTCGCTTATTAAAGATTTGTTGTTCGTTTTTGCTGCAGAATATGGTTGCCCCAA	A/G	GTTCAAGTTCAGGTTCAGGTTCAGGTTCAGGTTCAGGGCAGCCACAATTCATTTCAAGCACTGGAAACAGAAACCTTTCCAATGCGCCACTCATTGAGAC
Ca_LG_1:1555688	ATATTCGTTCCTAGGAAATGGATCACGAAGTCACGGAATGTTTGAGCCATACCAACACCATCGATGAAGAGTTCTTCAAGATATCAATGCAGCAGCAACA	A/C	ACAATTGCCCTTAATCTTAACACTGCGACCGTTATTAATTCAGTATTTGACCTAATGAACTCATCCATCAATGTCTTTCATTCAACTCAACATGCTGCAG
Ca_LG_1:1555713	CGAAGTCACGGAATGTTTGAGCCATACCAACACCATCGATGAAGAGTTCTTCAAGATATCAATGCAGCAGCAACAACAATTGCCCTTAATCTTAACACTG	G/A	CGACCGTTATTAATTCAGTATTTGACCTAATGAACTCATCCATCAATGTCTTTCATTCAACTCAACATGCTGCAGAAAAGCCAATCTCAGTCACTAACAA
Ca_LG_1:1555715	AAGTCACGGAATGTTTGAGCCATACCAACACCATCGATGAAGAGTTCTTCAAGATATCAATGCAGCAGCAACAACAATTGCCCTTAATCTTAACACTGCG	G/C	ACCGTTATTAATTCAGTATTTGACCTAATGAACTCATCCATCAATGTCTTTCATTCAACTCAACATGCTGCAGAAAAGCCAATCTCAGTCACTAACAATA
Ca_LG_1:1564405	AACTTTTTGGCCAAATCATAAATTTTTAACACACAATACCATTTTGTAATAGCAAGATACCTCGTGTGCAGCGTAAATCTCAGTGTTAAAACCAATCTCG	G/T	TCGCCGTGTTCATTAGTACCAAAGCCTCTAGGTTTTCCAAAATAGATACCTGAATAACAAACAAAGCAGCAGCAAGACTGAGTTAGGTGAATTAGCTTCC
Ca_LG_1:1967926	GTGTTTGTTGGCAGCTGGTGTTGGCACAACTGTAACAGAGTTAAAAAAAAGAACAAAACATTGACAGCTAGTGTAAAGCACAACTGTGTTTGAAAGAAAA	A/T	AAAAAAGTCACTGAAAAACACATAGTGAAGGTTGTGCTTTTGAGGCCAGCTGTAGCGTCGTGGCTGGTTGCTAGAAAGACAGCCGGAGTTGATGGTGACA
Ca_LG_1:1967934	TGGCAGCTGGTGTTGGCACAACTGTAACAGAGTTAAAAAAAAGAACAAAACATTGACAGCTAGTGTAAAGCACAACTGTGTTTGAAAGAAAAAAAAAAGT	T/C	CACTGAAAAACACATAGTGAAGGTTGTGCTTTTGAGGCCAGCTGTAGCGTCGTGGCTGGTTGCTAGAAAGACAGCCGGAGTTGATGGTGACAGATGACTG
Ca_LG_1:1968008	ACTGTGTTTGAAAGAAAAAAAAAAGTCACTGAAAAACACATAGTGAAGGTTGTGCTTTTGAGGCCAGCTGTAGCGTCGTGGCTGGTTGCTAGAAAGACAG	G/A	CCGGAGTTGATGGTGACAGATGACTGTATGAAGCCAAAAAATTGAAAGACAGAAAAAGGTGGTGCTGTTGGCGGCATTTGCTGCCGCCGGCAGCGTTAGT
Ca_LG_1:1968018	AAAGAAAAAAAAAAGTCACTGAAAAACACATAGTGAAGGTTGTGCTTTTGAGGCCAGCTGTAGCGTCGTGGCTGGTTGCTAGAAAGACAGCCGGAGTTGA	A/G	TGGTGACAGATGACTGTATGAAGCCAAAAAATTGAAAGACAGAAAAAGGTGGTGCTGTTGGCGGCATTTGCTGCCGCCGGCAGCGTTAGTGAAGCACAAA
Ca_LG_1:1968021	GAAAAAAAAAAGTCACTGAAAAACACATAGTGAAGGTTGTGCTTTTGAGGCCAGCTGTAGCGTCGTGGCTGGTTGCTAGAAAGACAGCCGGAGTTGATGG	G/T	TGACAGATGACTGTATGAAGCCAAAAAATTGAAAGACAGAAAAAGGTGGTGCTGTTGGCGGCATTTGCTGCCGCCGGCAGCGTTAGTGAAGCACAAAGAC
Ca_LG_1:1968036	CTGAAAAACACATAGTGAAGGTTGTGCTTTTGAGGCCAGCTGTAGCGTCGTGGCTGGTTGCTAGAAAGACAGCCGGAGTTGATGGTGACAGATGACTGTA	A/G	TGAAGCCAAAAAATTGAAAGACAGAAAAAGGTGGTGCTGTTGGCGGCATTTGCTGCCGCCGGCAGCGTTAGTGAAGCACAAAGACGGTCACAAAATCAAA

**Positions**	**-100 bp flanking sequences**	**SNPs within *desi* cultivar**	**+100 bp flanking sequences**
Ca_LG_1:1968059	GTGCTTTTGAGGCCAGCTGTAGCGTCGTGGCTGGTTGCTAGAAAGACAGCCGGAGTTGATGGTGACAGATGACTGTATGAAGCCAAAAAATTGAAAGACA	A/T	GAAAAAGGTGGTGCTGTTGGCGGCATTTGCTGCCGCCGGCAGCGTTAGTGAAGCACAAAGACGGTCACAAAATCAAAACCTGCTCTGATACCATGTAGAA
Ca_LG_1:2116008	TCACAAAGTTTAGTAATCTTGATATTAATCTAAACATGTATAGGAACCAACCATAGGAGCTGCTTTTTTCACACAAATATTATCATTATCTGAAGCAACA	A/T	GTGAAGTTTGACATATGGGACACAGCAGGACAAGAAAGATATCATAGTTTGGCTCCTATGTACTACCGTGGTGCTGCAGCAGCAATTGTTGTTTATGATA
Ca_LG_1:2116014	AGTTTAGTAATCTTGATATTAATCTAAACATGTATAGGAACCAACCATAGGAGCTGCTTTTTTCACACAAATATTATCATTATCTGAAGCAACAGTGAAG	G/A	TTTGACATATGGGACACAGCAGGACAAGAAAGATATCATAGTTTGGCTCCTATGTACTACCGTGGTGCTGCAGCAGCAATTGTTGTTTATGATATCTCTA
Ca_LG_1:2116020	GTAATCTTGATATTAATCTAAACATGTATAGGAACCAACCATAGGAGCTGCTTTTTTCACACAAATATTATCATTATCTGAAGCAACAGTGAAGTTTGAC	C/T	ATATGGGACACAGCAGGACAAGAAAGATATCATAGTTTGGCTCCTATGTACTACCGTGGTGCTGCAGCAGCAATTGTTGTTTATGATATCTCTAGCATTG
Ca_LG_1:2116041	ACATGTATAGGAACCAACCATAGGAGCTGCTTTTTTCACACAAATATTATCATTATCTGAAGCAACAGTGAAGTTTGACATATGGGACACAGCAGGACAA	A/G	GAAAGATATCATAGTTTGGCTCCTATGTACTACCGTGGTGCTGCAGCAGCAATTGTTGTTTATGATATCTCTAGCATTGTAAGAAAATTCATATTGTTAA
Ca_LG_1:2116045	GTATAGGAACCAACCATAGGAGCTGCTTTTTTCACACAAATATTATCATTATCTGAAGCAACAGTGAAGTTTGACATATGGGACACAGCAGGACAAGAAA	A/C	GATATCATAGTTTGGCTCCTATGTACTACCGTGGTGCTGCAGCAGCAATTGTTGTTTATGATATCTCTAGCATTGTAAGAAAATTCATATTGTTAATTTT
Ca_LG_1:2116050	GGAACCAACCATAGGAGCTGCTTTTTTCACACAAATATTATCATTATCTGAAGCAACAGTGAAGTTTGACATATGGGACACAGCAGGACAAGAAAGATAT	T/C	CATAGTTTGGCTCCTATGTACTACCGTGGTGCTGCAGCAGCAATTGTTGTTTATGATATCTCTAGCATTGTAAGAAAATTCATATTGTTAATTTTGAAAA
Ca_LG_1:2229913	AGTAAAATTCAGCTGAATTCTTACGTGGCAATGGAAGTTTTCTGTTCTTATAAGCAGCAACAGTTACTTGTGCAAAACCAGTGAACAAGCTGGTTCTTTT	T/C	TTTAATCTTTACATAAAGAGGAGAAGCAAGAAAGAACAAGAGAGTAGACAAAAGCATGAGTGCTGCTGGAACTCCAAAACCAATTTTCCATCCAAGATGA
Ca_LG_1:3462003	CAAGTAATCATAACATAAAACAAACGGGTGCATGTGATAGAATGCATACAGTTCATTTGCAGCATACAGTTGTGCTTCATCTTTAGGCATGCTGCAATAA	A/C	AAGATCAAATTATAATTACTACTATTAGTATTATAAATGCAAGCATATGTGAAAATGAATATGATTGGTTTTCAAAAAAGCGCACCTGTAAAATATGTAA
Ca_LG_1:3462525	CAAGCCAAGCAAACCAAATGGATCAGGAGTAGATTGTGCAGTTTGCATAGACTTCATGCCATGATCCCTAAAGGACTGATTTGCAGCTGACATTTGCTGC	C/T	AGGCGAAACTGGGACTGGTTCTGGTGTTGTTGATACTGCTGGATGAGCTGGTCATATGAACCCGTACCAGAAACTGTATTTGGAGAATTTAGGGGCCTCA
Ca_LG_1:3473169	CCCTTCGGCCGTGAATAAGGACAATCCTAATGCTATTTTGAATGGACTAGAGATCATGAAAATGAACAATTCCATAGGTAGTCTTAGTGCTGCAGCAGCG	G/A	TCTGGTACTGGGGGTGATTCCTCTGGTTCGAGTTCTAGTAAGGTTGGCGTGATAGCAGGTGTGAGTGTAGGGATAGTGAGTGCATTGGTCTTGGCCGGTG
Ca_LG_1:3473190	CAATCCTAATGCTATTTTGAATGGACTAGAGATCATGAAAATGAACAATTCCATAGGTAGTCTTAGTGCTGCAGCAGCGTCTGGTACTGGGGGTGATTCC	C/T	TCTGGTTCGAGTTCTAGTAAGGTTGGCGTGATAGCAGGTGTGAGTGTAGGGATAGTGAGTGCATTGGTCTTGGCCGGTGTTTTCTGTGTAATGTGCAGGA

**Positions**	**-100 bp flanking sequences**	**SNPs within *desi* cultivar**	**+100 bp flanking sequences**
Ca_LG_1:3923732	TCCTTTGTGAGGGCTTTTCAGCCAAAAAATCAGCCATATATGAGGGTAAAGGCTGCCACAAAGGCAATCCATTTGTGACCGCCACAAAATCAGTATACCG	G/A	TTGCACTTTGTGGCTGCAAAGGCCACCACAAATACTCATTGTACCGTTGCCCGTTGTGGCTGCTTAGGCCGCTACAAATTCCTTCATTTCTGACCGTAAA
Ca_LG_1:4210820	GCAGAATCTTGATTGAGCCCGAGGTTCAGCCTTCTGACTTGGCTACCAGAATCTCAATCGAACCTGAGGATCAGCCTTCTGACTCGGCTAGCAGCATCTC	C/G	GATTGAACTCAAGGATCAGCCTCTTGACTTGGATGGAGATGATGCAACTAATAATTTGGCAGCATTTGAATATACTGATGAACTATACAATTTCTACAAG
Ca_LG_1:4250517	AAGATGAGGGGCTAGTAGTGCTATCCATATCATAAATGAGATTGTCTCTCAATTGTGGCAGCTGAGTTGCATTTGGAACACTGGCAGAGAAAGATGGTCG	G/A	GCGAAGCCTTGAAAGCTGCATAACCTGCAGTCAATTTTGAAAGAAGGCAGTAATATAAGAAGCATTTCAGATTTGTAGATAAGAAGCAATTCTTGTTTTG
Ca_LG_1:4359791	TTCTTTCCAATTCAATACAAACAAAAACCATAACTGACCTTTAATGACCCATCTACCTAATCTTCTCCTTTCCACTGATCCTATTAGCAGCTTCCCTAAT	T/C	TTTTTCAATATAACCCTCAATAGGAGGAGTAAGAGTAGCCATAAAACGATTCGCAGGAAAAGGTGAGAAATCAGGCACCGAAGCAGCTTCTTTCAAACCC
Ca_LG_1:4359834	ATGACCCATCTACCTAATCTTCTCCTTTCCACTGATCCTATTAGCAGCTTCCCTAATTTTTTCAATATAACCCTCAATAGGAGGAGTAAGAGTAGCCATA	A/G	AAACGATTCGCAGGAAAAGGTGAGAAATCAGGCACCGAAGCAGCTTCTTTCAAACCCTCAGGCAAAGCTTCAATGGCTTCCTTTTTCATCCTCAAAAGCG
Ca_LG_1:4359844	TACCTAATCTTCTCCTTTCCACTGATCCTATTAGCAGCTTCCCTAATTTTTTCAATATAACCCTCAATAGGAGGAGTAAGAGTAGCCATAAAACGATTCG	G/A	CAGGAAAAGGTGAGAAATCAGGCACCGAAGCAGCTTCTTTCAAACCCTCAGGCAAAGCTTCAATGGCTTCCTTTTTCATCCTCAAAAGCGTCGTTTCCGC
Ca_LG_1:4359845	ACCTAATCTTCTCCTTTCCACTGATCCTATTAGCAGCTTCCCTAATTTTTTCAATATAACCCTCAATAGGAGGAGTAAGAGTAGCCATAAAACGATTCGC	C/T	AGGAAAAGGTGAGAAATCAGGCACCGAAGCAGCTTCTTTCAAACCCTCAGGCAAAGCTTCAATGGCTTCCTTTTTCATCCTCAAAAGCGTCGTTTCCGCA
Ca_LG_1:4359858	CTTTCCACTGATCCTATTAGCAGCTTCCCTAATTTTTTCAATATAACCCTCAATAGGAGGAGTAAGAGTAGCCATAAAACGATTCGCAGGAAAAGGTGAG	G/C	AAATCAGGCACCGAAGCAGCTTCTTTCAAACCCTCAGGCAAAGCTTCAATGGCTTCCTTTTTCATCCTCAAAAGCGTCGTTTCCGCAGCTTGTCTCGCTC
Ca_LG_1:4359871	CTATTAGCAGCTTCCCTAATTTTTTCAATATAACCCTCAATAGGAGGAGTAAGAGTAGCCATAAAACGATTCGCAGGAAAAGGTGAGAAATCAGGCACCG	G/A	AAGCAGCTTCTTTCAAACCCTCAGGCAAAGCTTCAATGGCTTCCTTTTTCATCCTCAAAAGCGTCGTTTCCGCAGCTTGTCTCGCTCTATGCTTCCTCAT
Ca_LG_1:4359909	AATAGGAGGAGTAAGAGTAGCCATAAAACGATTCGCAGGAAAAGGTGAGAAATCAGGCACCGAAGCAGCTTCTTTCAAACCCTCAGGCAAAGCTTCAATG	G/A	GCTTCCTTTTTCATCCTCAAAAGCGTCGTTTCCGCAGCTTGTCTCGCTCTATGCTTCCTCATCAAAACCCTACTATACTCCTTCGCCAATCTCCTCCCAT
Ca_LG_1:4950774	TAAAAACAGAAAGCAGAAGCTGCATTGCTTTTTGCTGCCTGAAAATTGTATTTCAAGTAAATTGTTGATATCTTTGTCCTACGAACAATAAAGTTGTAAA	A/T	AATACGATTTTCAATCAAACTCAGTTTTGAGCAATCTATTGACGGCATATTTCACAAGTTGCTACTTTCCTGCACTTTTTTTCTTCCACTAACAGAAATT
Ca_LG_1:5103778	CAGTACTAATTGAGAGCAAACTAATTTGTGTTTGTATAGTTCTCAAGTAACTGTCATAAATCAATGCTTTCGGTGTTGACGGCGCCGCCGTAACTGGTGG	G/T	CATCTCCACCGGAGTTGTCTCATATTCCATTGCCTTGTGGAGCGGGTTGCTGCCTTTACCATTTGGGACTGGCCATGAGACATACATATATAACACGAAT

Positions	-100 bp flanking sequences	SNPs within *desi* cultivar	+100 bp flanking sequences
Ca_LG_1:5103786	ATTGAGAGCAAACTAATTTGTGTTTGTATAGTTCTCAAGTAACTGTCATAAATCAATGCTTTCGGTGTTGACGGCGCCGCCGTAACTGGTGGCATCTCCA	A/G	CCGGAGTTGTCTCATATTCCATTGCCTTGTGGAGCGGGTTGCTGCCTTTACCATTTGGGACTGGCCATGAGACATACATATATAACACGAATTATGATAT
Ca_LG_1:5212668	TATCTAATCAAGAAGAAGAGTGAAGTGTTGGCTGTGTGTAGAGGGTTCAAGCTAATGGCAAAAAAACAAAGTGGCTGCAATCTTAAAGGGATTAGAACAT	T/G	ATGGAGGTGGTGAGTATCAATCTTATGAATTTTATGACAAAGAAAATTATTCATGAAGTAACTGCACCATATATATCAGGACATAATAGAACTGCATAAA
Ca_LG_1:5212680	AAGAAGAGTGAAGTGTTGGCTGTGTGTAGAGGGTTCAAGCTAATGGCAAAAAAACAAAGTGGCTGCAATCTTAAAGGGATTAGAACATATGGAGGTGGTG	G/A	AGTATCAATCTTATGAATTTTATGACAAAGAAAATTATTCATGAAGTAACTGCACCATATATATCAGGACATAATAGAACTGCATAAAGGAGAAATAGAA
Ca_LG_1:6247039	TGATAGGATACTCAGACTCAGGAAAAGATGCGGGCCGTCTTTCAGCAGCTTGGGCACTATACAAGGCTCAAGAGGAACTCATAAATGTAGCAAAGGAGTT	T/C	TGGTGTTAAGCTAACAATGTTCCACGGCCGAGGAGGGACAGTTGGAAGAGGAGGAGGTCCTACTCATCTTGCTATATTGTCTCAGCCACCGGATACTATT
Ca_LG_1:6436331	CGCTATAGCTGAATCTAAGGATCTAGCAAAGATGACGACAGCAACATTATTTGGAAAATTAAGGGAACATGAAATGGAATTACAACGACTGGATGAATCA	A/C	GAAATGGAAAGTAGGAAAAAGAAAGGTTTATCTCTGAAGGTTCAAGCAAAGCAATCTAAACCTGAATCCGATAGCTACTCAAATGAATCCAGCAGTGAAA
Ca_LG_1:6822949	CAACAGCTATGCCAAGTTTCTCCCGGATAGAGGACACTCGTTGTTCTGAATGTTCGAGTTCATTTGCTTTCTCACGCAATTCAGTACGTGCAGCAGTAAT	T/G	AGCTTCCTCAGCTTGATGTAAGCTTTCCCTTAGAACAAATATTTCATTTTCATTCTCAAGACGCAGCATATCTTGACGATGCATCTTTTCCTTCAGTTGA
Ca_LG_1:6824151	GCAAGTTCGATGTTGGATGCTTCCAAATCTGAACAATGCTGCTTCAATCCTTCAAACTGGTCCTTGAGATCACTGTACTTTGCATCCTCAATTTTCCTAC	C/T	CGTCTCGTTCCCCCTTGAACAATACAGCTGCCCTCTGAACATCCAGCTTCCAATTTAAAAGCAATTTTCTCAAGTTTCCTACTTGCTCTTCAGTCAACAT
Ca_LG_1:6824153	AAGTTCGATGTTGGATGCTTCCAAATCTGAACAATGCTGCTTCAATCCTTCAAACTGGTCCTTGAGATCACTGTACTTTGCATCCTCAATTTTCCTACCG	G/A	TCTCGTTCCCCCTTGAACAATACAGCTGCCCTCTGAACATCCAGCTTCCAATTTAAAAGCAATTTTCTCAAGTTTCCTACTTGCTCTTCAGTCAACATAA
Ca_LG_1:6824162	GTTGGATGCTTCCAAATCTGAACAATGCTGCTTCAATCCTTCAAACTGGTCCTTGAGATCACTGTACTTTGCATCCTCAATTTTCCTACCGTCTCGTTCC	C/T	CCCTTGAACAATACAGCTGCCCTCTGAACATCCAGCTTCCAATTTAAAAGCAATTTTCTCAAGTTTCCTACTTGCTCTTCAGTCAACATAATCAATGAGT
Ca_LG_1:6826878	CAAATTAGTTTCCTCTCCTGTATCATTTTCCCTGACCGCTGACGACGAATCAACAGATGGTTCAACATTATCAGGAGCTGCTGAGCTTGCCAAAGACTGT	T/C	GACTCTAATGATTCGGTGATAACCACATTGGGGTCACTGTTGGTTTCAAGATTTCCATCAGTAACCTGTGATGATCTTGTTGAAATAGAGGTAACACTAG
Ca_LG_1:6826986	TGATTCGGTGATAACCACATTGGGGTCACTGTTGGTTTCAAGATTTCCATCAGTAACCTGTGATGATCTTGTTGAAATAGAGGTAACACTAGCAGCATCA	A/G	GAATCAGCATCGGACTCGGGTATCTGATGCTTGGAAGATTTTTTTGATGATTTCCCACGGCTACTACCACCTTTATTGTCCTTGTCCTTCTTTTGACGAT
Ca_LG_1:7051978	AGGAAACATGGGTTCTTCAGGCTGGTGCTGCTGACCATGTTCATCATCAAACTGATGTTGCTGCTGGTGATCATGTTCATCATCAAACACATGATCTTGT	T/C	TGGGGCTGGTCATCCTGTTCTTCATCAAACACAGGATTTTGCTGGGGCTGCTCATCCTGGGGCGCCTGTCTTTCTTCTTCATTTCTCTTTCTTCTTTTTT

**Positions**	**-100 bp flanking sequences**	**SNPs within *desi* cultivar**	**+100 bp flanking sequences**
Ca_LG_1:7226535	CAACCAAACGCGATCACGCAAGCAGCGACGGCTGAGCGGAAACCAATAAACAAGCAGCGAAAATGACCCAGAAACGCAGTAACGAGCAGCAGCGAGAATA	A/G	TAATGAGCAGCAGCGAGAATGTAATGAGCAGCAGCGAGAATGTAATGAGCAGCAGCGAGAATGTAATGAGCAGCAGCGAGAATGTAATGAGCAACAGCGA
Ca_LG_1:7226629	AGAATATAATGAGCAGCAGCGAGAATGTAATGAGCAGCAGCGAGAATGTAATGAGCAGCAGCGAGAATGTAATGAGCAGCAGCGAGAATGTAATGAGCAA	A/G	CAGCGAGAATGACCCAGAAACGCAGTAACGATCGGCAGCGAGAAAGACCCAGAAACGCAGTAACGAGCAGCAGCGAGAAACAACCCAAAAGCACAATCGC
Ca_LG_1:8617451	GTTGGAATTGAGGCAGAAGATGAGAAGATTGAGCTTCTCCTAACTGAAGTTAAGGGAAAAGATTTTGCCGAGCTTATTGCTAGTGGAAGAGAAAAGTTGT	T/G	CTTCAGTTCCTTCTGGTGGTGGTGCTGTTGCTGTTTCTGCTGCACCTGCCGGAGGTGCTGCAGCTGCCGCACCTGCTGCTGAAGCAAAGGAGGAGAAGAA
Ca_LG_1:8617453	TGGAATTGAGGCAGAAGATGAGAAGATTGAGCTTCTCCTAACTGAAGTTAAGGGAAAAGATTTTGCCGAGCTTATTGCTAGTGGAAGAGAAAAGTTGTCT	T/A	TCAGTTCCTTCTGGTGGTGGTGCTGTTGCTGTTTCTGCTGCACCTGCCGGAGGTGCTGCAGCTGCCGCACCTGCTGCTGAAGCAAAGGAGGAGAAGAAGG
Ca_LG_1:8617459	TGAGGCAGAAGATGAGAAGATTGAGCTTCTCCTAACTGAAGTTAAGGGAAAAGATTTTGCCGAGCTTATTGCTAGTGGAAGAGAAAAGTTGTCTTCAGTT	T/G	CCTTCTGGTGGTGGTGCTGTTGCTGTTTCTGCTGCACCTGCCGGAGGTGCTGCAGCTGCCGCACCTGCTGCTGAAGCAAAGGAGGAGAAGAAGGTCGAAG
Ca_LG_1:8617462	GGCAGAAGATGAGAAGATTGAGCTTCTCCTAACTGAAGTTAAGGGAAAAGATTTTGCCGAGCTTATTGCTAGTGGAAGAGAAAAGTTGTCTTCAGTTCCT	T/C	TCTGGTGGTGGTGCTGTTGCTGTTTCTGCTGCACCTGCCGGAGGTGCTGCAGCTGCCGCACCTGCTGCTGAAGCAAAGGAGGAGAAGAAGGTCGAAGAAA
Ca_LG_1:8617489	CCTAACTGAAGTTAAGGGAAAAGATTTTGCCGAGCTTATTGCTAGTGGAAGAGAAAAGTTGTCTTCAGTTCCTTCTGGTGGTGGTGCTGTTGCTGTTTCT	T/C	GCTGCACCTGCCGGAGGTGCTGCAGCTGCCGCACCTGCTGCTGAAGCAAAGGAGGAGAAGAAGGTCGAAGAAAAGGAAGAATCCGATGATGTAAGTTTTG
Ca_LG_1:8847149	GTCTTTACATTGGATGGTTTTGGGTTTTGATGATCCCTACCTTATTGACCGCAACTTTTGTATTTATTATCACTTTCATTCTTGCCCCTCTAGTAGATAG	G/T	TGACGGTATTCATGAGCCTATTTTCTGCATCTCTACTTTACAAAAACAATATTATTTTTGGTGCCATTATTCCTACTTCTGCGGCTATTGGTTTTAACTT
Ca_LG_1:8847161	GATGGTTTTGGGTTTTGATGATCCCTACCTTATTGACCGCAACTTTTGTATTTATTATCACTTTCATTCTTGCCCCTCTAGTAGATAGTGACGGTATTCA	A/G	TGAGCCTATTTTCTGCATCTCTACTTTACAAAAACAATATTATTTTTGGTGCCATTATTCCTACTTCTGCGGCTATTGGTTTTAACTTTTACCCAATATG
Ca_LG_1:8847177	GATGATCCCTACCTTATTGACCGCAACTTTTGTATTTATTATCACTTTCATTCTTGCCCCTCTAGTAGATAGTGACGGTATTCATGAGCCTATTTTCTGC	C/G	ATCTCTACTTTACAAAAACAATATTATTTTTGGTGCCATTATTCCTACTTCTGCGGCTATTGGTTTTAACTTTTACCCAATATGGGAAGTTGTATCAAGT
Ca_LG_1:8847190	TTATTGACCGCAACTTTTGTATTTATTATCACTTTCATTCTTGCCCCTCTAGTAGATAGTGACGGTATTCATGAGCCTATTTTCTGCATCTCTACTTTAC	C/T	AAAAACAATATTATTTTTGGTGCCATTATTCCTACTTCTGCGGCTATTGGTTTTAACTTTTACCCAATATGGGAAGTTGTATCAAGTTGATGAATGGTTA
Ca_LG_1:8847191	TATTGACCGCAACTTTTGTATTTATTATCACTTTCATTCTTGCCCCTCTAGTAGATAGTGACGGTATTCATGAGCCTATTTTCTGCATCTCTACTTTACA	A/G	AAAACAATATTATTTTTGGTGCCATTATTCCTACTTCTGCGGCTATTGGTTTTAACTTTTACCCAATATGGGAAGTTGTATCAAGTTGATGAATGGTTAT

**Positions**	**-100 bp flanking sequences**	**SNPs within *desi* cultivar**	**+100 bp flanking sequences**
Ca_LG_1:8847192	ATTGACCGCAACTTTTGTATTTATTATCACTTTCATTCTTGCCCCTCTAGTAGATAGTGACGGTATTCATGAGCCTATTTTCTGCATCTCTACTTTACAA	A/G	AAACAATATTATTTTTGGTGCCATTATTCCTACTTCTGCGGCTATTGGTTTTAACTTTTACCCAATATGGGAAGTTGTATCAAGTTGATGAATGGTTATA
Ca_LG_1:8847207	TGTATTTATTATCACTTTCATTCTTGCCCCTCTAGTAGATAGTGACGGTATTCATGAGCCTATTTTCTGCATCTCTACTTTACAAAAACAATATTATTTT	T/C	TGGTGCCATTATTCCTACTTCTGCGGCTATTGGTTTTAACTTTTACCCAATATGGGAAGTTGTATCAAGTTGATGAATGGTTATACAATGGCGATCTGTA
Ca_LG_1:8847244	GATAGTGACGGTATTCATGAGCCTATTTTCTGCATCTCTACTTTACAAAAACAATATTATTTTTGGTGCCATTATTCCTACTTCTGCGGCTATTGGTTTT	T/G	AACTTTTACCCAATATGGGAAGTTGTATCAAGTTGATGAATGGTTATACAATGGCGATCTGTATGAACTAATTGTTCTACACTTCTTACTTGGTGTAGCT
Ca_LG_1:8847245	ATAGTGACGGTATTCATGAGCCTATTTTCTGCATCTCTACTTTACAAAAACAATATTATTTTTGGTGCCATTATTCCTACTTCTGCGGCTATTGGTTTTA	A/C	ACTTTTACCCAATATGGGAAGTTGTATCAAGTTGATGAATGGTTATACAATGGCGATCTGTATGAACTAATTGTTCTACACTTCTTACTTGGTGTAGCTT
Ca_LG_1:8847253	GGTATTCATGAGCCTATTTTCTGCATCTCTACTTTACAAAAACAATATTATTTTTGGTGCCATTATTCCTACTTCTGCGGCTATTGGTTTTAACTTTTAC	C/T	CCAATATGGGAAGTTGTATCAAGTTGATGAATGGTTATACAATGGCGATCTGTATGAACTAATTGTTCTACACTTCTTACTTGGTGTAGCTTGTTACATG
Ca_LG_1:8847301	TATTTTTGGTGCCATTATTCCTACTTCTGCGGCTATTGGTTTTAACTTTTACCCAATATGGGAAGTTGTATCAAGTTGATGAATGGTTATACAATGGCGA	A/G	TCTGTATGAACTAATTGTTCTACACTTCTTACTTGGTGTAGCTTGTTACATGGTTCGTGAATGGGAACTTAGTTTTCGTCTGGGTATGTGCCCTTGGATT
Ca_LG_1:8847304	TTTTGGTGCCATTATTCCTACTTCTGCGGCTATTGGTTTTAACTTTTACCCAATATGGGAAGTTGTATCAAGTTGATGAATGGTTATACAATGGCGATCT	T/C	GTATGAACTAATTGTTCTACACTTCTTACTTGGTGTAGCTTGTTACATGGTTCGTGAATGGGAACTTAGTTTTCGTCTGGGTATGTGCCCTTGGATTGTT
Ca_LG_1:8847305	TTTGGTGCCATTATTCCTACTTCTGCGGCTATTGGTTTTAACTTTTACCCAATATGGGAAGTTGTATCAAGTTGATGAATGGTTATACAATGGCGATCTG	G/A	TATGAACTAATTGTTCTACACTTCTTACTTGGTGTAGCTTGTTACATGGTTCGTGAATGGGAACTTAGTTTTCGTCTGGGTATGTGCCCTTGGATTGTTG
Ca_LG_1:8847355	AATATGGGAAGTTGTATCAAGTTGATGAATGGTTATACAATGGCGATCTGTATGAACTAATTGTTCTACACTTCTTACTTGGTGTAGCTTGTTACATGGT	T/G	TCGTGAATGGGAACTTAGTTTTCGTCTGGGTATGTGCCCTTGGATTGTTGTTGCATATTCAACTCCTATTGCAATTGCTACTGTTTTCTTGATCTACCCA
Ca_LG_1:8847362	GAAGTTGTATCAAGTTGATGAATGGTTATACAATGGCGATCTGTATGAACTAATTGTTCTACACTTCTTACTTGGTGTAGCTTGTTACATGGTTCGTGAA	A/G	TGGGAACTTAGTTTTCGTCTGGGTATGTGCCCTTGGATTGTTGTTGCATATTCAACTCCTATTGCAATTGCTACTGTTTTCTTGATCTACCCAATAGGTC
Ca_LG_1:8847390	TACAATGGCGATCTGTATGAACTAATTGTTCTACACTTCTTACTTGGTGTAGCTTGTTACATGGTTCGTGAATGGGAACTTAGTTTTCGTCTGGGTATGT	T/C	GCCCTTGGATTGTTGTTGCATATTCAACTCCTATTGCAATTGCTACTGTTTTCTTGATCTACCCAATAGGTCAAGGAAGCTTTTTAGATGGTATTCCTCT
Ca_LG_1:8847403	TGTATGAACTAATTGTTCTACACTTCTTACTTGGTGTAGCTTGTTACATGGTTCGTGAATGGGAACTTAGTTTTCGTCTGGGTATGTGCCCTTGGATTGT	T/C	TGTTGCATATTCAACTCCTATTGCAATTGCTACTGTTTTCTTGATCTACCCAATAGGTCAAGGAAGCTTTTTAGATGGTATTCCTCTAGGAATTTATGGT

Positions	-100 bp flanking sequences	SNPs within *desi* cultivar	+100 bp flanking sequences
Ca_LG_1:8847417	GTTCTACACTTCTTACTTGGTGTAGCTTGTTACATGGTTCGTGAATGGGAACTTAGTTTTCGTCTGGGTATGTGCCCTTGGATTGTTGTTGCATATTCAA	A/G	CTCCTATTGCAATTGCTACTGTTTTCTTGATCTACCCAATAGGTCAAGGAAGCTTTTTAGATGGTATTCCTCTAGGAATTTATGGTACTTTAAACTTTAT
Ca_LG_1:8847423	CACTTCTTACTTGGTGTAGCTTGTTACATGGTTCGTGAATGGGAACTTAGTTTTCGTCTGGGTATGTGCCCTTGGATTGTTGTTGCATATTCAACTCCTA	A/G	TTGCAATTGCTACTGTTTTCTTGATCTACCCAATAGGTCAAGGAAGCTTTTTAGATGGTATTCCTCTAGGAATTTATGGTACTTTAAACTTTATGATTGT
Ca_LG_1:8847470	TAGTTTTCGTCTGGGTATGTGCCCTTGGATTGTTGTTGCATATTCAACTCCTATTGCAATTGCTACTGTTTTCTTGATCTACCCAATAGGTCAAGGAAGC	C/T	TTTTTAGATGGTATTCCTCTAGGAATTTATGGTACTTTAAACTTTATGATTGTATTTCAAGCTAAGCATAATATTCTTATGCACCCATTTCACATGTTAG
Ca_LG_1:8847475	TTCGTCTGGGTATGTGCCCTTGGATTGTTGTTGCATATTCAACTCCTATTGCAATTGCTACTGTTTTCTTGATCTACCCAATAGGTCAAGGAAGCTTTTT	T/C	AGATGGTATTCCTCTAGGAATTTATGGTACTTTAAACTTTATGATTGTATTTCAAGCTAAGCATAATATTCTTATGCACCCATTTCACATGTTAGGTGTA
Ca_LG_1:8847485	TATGTGCCCTTGGATTGTTGTTGCATATTCAACTCCTATTGCAATTGCTACTGTTTTCTTGATCTACCCAATAGGTCAAGGAAGCTTTTTAGATGGTATT	T/G	CCTCTAGGAATTTATGGTACTTTAAACTTTATGATTGTATTTCAAGCTAAGCATAATATTCTTATGCACCCATTTCACATGTTAGGTGTAGTTTGTGTAA
Ca_LG_1:8847499	TTGTTGTTGCATATTCAACTCCTATTGCAATTGCTACTGTTTTCTTGATCTACCCAATAGGTCAAGGAAGCTTTTTAGATGGTATTCCTCTAGGAATTTA	A/C	TGGTACTTTAAACTTTATGATTGTATTTCAAGCTAAGCATAATATTCTTATGCACCCATTTCACATGTTAGGTGTAGTTTGTGTAATCGGCGGCTCTTTA
Ca_LG_1:8847509	ATATTCAACTCCTATTGCAATTGCTACTGTTTTCTTGATCTACCCAATAGGTCAAGGAAGCTTTTTAGATGGTATTCCTCTAGGAATTTATGGTACTTTA	A/C	AACTTTATGATTGTATTTCAAGCTAAGCATAATATTCTTATGCACCCATTTCACATGTTAGGTGTAGTTTGTGTAATCGGCGGCTCTTTATTTAGTGTTA
Ca_LG_1:8847530	TGCTACTGTTTTCTTGATCTACCCAATAGGTCAAGGAAGCTTTTTAGATGGTATTCCTCTAGGAATTTATGGTACTTTAAACTTTATGATTGTATTTCAA	A/G	GCTAAGCATAATATTCTTATGCACCCATTTCACATGTTAGGTGTAGTTTGTGTAATCGGCGGCTCTTTATTTAGTGTTATGCACAGTTCCTTGGTAACTT
Ca_LG_1:8847534	ACTGTTTTCTTGATCTACCCAATAGGTCAAGGAAGCTTTTTAGATGGTATTCCTCTAGGAATTTATGGTACTTTAAACTTTATGATTGTATTTCAAGCTA	A/G	AGCATAATATTCTTATGCACCCATTTCACATGTTAGGTGTAGTTTGTGTAATCGGCGGCTCTTTATTTAGTGTTATGCACAGTTCCTTGGTAACTTCTAG
Ca_LG_1:8847682	TAATCGGCGGCTCTTTATTTAGTGTTATGCACAGTTCCTTGGTAACTTCTAGTTTGATCAGGGAAACAGCAAAAAATGAATCTACTAATGAAGGTTACAA	A/G	ATTTAGTCAAGAGGAAGAAACCTATAATATTGTAGCTGCTCATGGTTATTTTGGCCGATTGATCTTCCAATATGTGAGCTTCAACATTTCTCGTCTTTAC
Ca_LG_1:8847687	GGCGGCTCTTTATTTAGTGTTATGCACAGTTCCTTGGTAACTTCTAGTTTGATCAGGGAAACAGCAAAAAATGAATCTACTAATGAAGGTTACAAATTTA	A/G	GTCAAGAGGAAGAAACCTATAATATTGTAGCTGCTCATGGTTATTTTGGCCGATTGATCTTCCAATATGTGAGCTTCAACATTTCTCGTCTTTACATTTC
Ca_LG_1:8847757	ATGAATCTACTAATGAAGGTTACAAATTTAGTCAAGAGGAAGAAACCTATAATATTGTAGCTGCTCATGGTTATTTTGGCCGATTGATCTTCCAATATGT	T/C	GAGCTTCAACATTTCTCGTCTTTACATTTCTTTCTAGCTACTTGGCCTGTAGTAGGTATTTGGTTTACCGCCTTAGGTATCAACGCTATGAATTTCAATT

Positions	-100 bp flanking sequences	SNPs within *desi* cultivar	+100 bp flanking sequences
Ca_LG_1:8847761	ATCTACTAATGAAGGTTACAAATTTAGTCAAGAGGAAGAAACCTATAATATTGTAGCTGCTCATGGTTATTTTGGCCGATTGATCTTCCAATATGTGAGC	C/T	TTCAACATTTCTCGTCTTTACATTTCTTTCTAGCTACTTGGCCTGTAGTAGGTATTTGGTTTACCGCCTTAGGTATCAACGCTATGAATTTCAATTTAAA
Ca_LG_1:8847769	ATGAAGGTTACAAATTTAGTCAAGAGGAAGAAACCTATAATATTGTAGCTGCTCATGGTTATTTTGGCCGATTGATCTTCCAATATGTGAGCTTCAACAT	T/A	TTCTCGTCTTTACATTTCTTTCTAGCTACTTGGCCTGTAGTAGGTATTTGGTTTACCGCCTTAGGTATCAACGCTATGAATTTCAATTTAAATGGTTTCA
Ca_LG_1:8847797	AGAAACCTATAATATTGTAGCTGCTCATGGTTATTTTGGCCGATTGATCTTCCAATATGTGAGCTTCAACATTTCTCGTCTTTACATTTCTTTCTAGCTA	A/G	CTTGGCCTGTAGTAGGTATTTGGTTTACCGCCTTAGGTATCAACGCTATGAATTTCAATTTAAATGGTTTCAGTTTTAACCAATCCATAGTTGACAGTCA
Ca_LG_1:8847817	CTGCTCATGGTTATTTTGGCCGATTGATCTTCCAATATGTGAGCTTCAACATTTCTCGTCTTTACATTTCTTTCTAGCTACTTGGCCTGTAGTAGGTATT	T/C	TGGTTTACCGCCTTAGGTATCAACGCTATGAATTTCAATTTAAATGGTTTCAGTTTTAACCAATCCATAGTTGACAGTCAAGGTCGTGTAATTAATACAT
Ca_LG_1:8847829	ATTTTGGCCGATTGATCTTCCAATATGTGAGCTTCAACATTTCTCGTCTTTACATTTCTTTCTAGCTACTTGGCCTGTAGTAGGTATTTGGTTTACCGCC	C/G	TTAGGTATCAACGCTATGAATTTCAATTTAAATGGTTTCAGTTTTAACCAATCCATAGTTGACAGTCAAGGTCGTGTAATTAATACATGGATTGCTATTA
Ca_LG_1:8847840	TTGATCTTCCAATATGTGAGCTTCAACATTTCTCGTCTTTACATTTCTTTCTAGCTACTTGGCCTGTAGTAGGTATTTGGTTTACCGCCTTAGGTATCAA	A/G	CGCTATGAATTTCAATTTAAATGGTTTCAGTTTTAACCAATCCATAGTTGACAGTCAAGGTCGTGTAATTAATACATGGATTGCTATTATTAATCGTGTT
Ca_LG_1:8847842	GATCTTCCAATATGTGAGCTTCAACATTTCTCGTCTTTACATTTCTTTCTAGCTACTTGGCCTGTAGTAGGTATTTGGTTTACCGCCTTAGGTATCAACG	G/A	CTATGAATTTCAATTTAAATGGTTTCAGTTTTAACCAATCCATAGTTGACAGTCAAGGTCGTGTAATTAATACATGGATTGCTATTATTAATCGTGTTAA
Ca_LG_1:8847848	CCAATATGTGAGCTTCAACATTTCTCGTCTTTACATTTCTTTCTAGCTACTTGGCCTGTAGTAGGTATTTGGTTTACCGCCTTAGGTATCAACGCTATGA	A/G	ATTTCAATTTAAATGGTTTCAGTTTTAACCAATCCATAGTTGACAGTCAAGGTCGTGTAATTAATACATGGATTGCTATTATTAATCGTGTTAACCTTGG
Ca_LG_1:8847849	CAATATGTGAGCTTCAACATTTCTCGTCTTTACATTTCTTTCTAGCTACTTGGCCTGTAGTAGGTATTTGGTTTACCGCCTTAGGTATCAACGCTATGAA	A/C	TTTCAATTTAAATGGTTTCAGTTTTAACCAATCCATAGTTGACAGTCAAGGTCGTGTAATTAATACATGGATTGCTATTATTAATCGTGTTAACCTTGGT
Ca_LG_1:8847870	TCTCGTCTTTACATTTCTTTCTAGCTACTTGGCCTGTAGTAGGTATTTGGTTTACCGCCTTAGGTATCAACGCTATGAATTTCAATTTAAATGGTTTCAG	G/A	TTTTAACCAATCCATAGTTGACAGTCAAGGTCGTGTAATTAATACATGGATTGCTATTATTAATCGTGTTAACCTTGGTATGGAAGTTATGCATGAATGT
Ca_LG_1:8847884	TTCTTTCTAGCTACTTGGCCTGTAGTAGGTATTTGGTTTACCGCCTTAGGTATCAACGCTATGAATTTCAATTTAAATGGTTTCAGTTTTAACCAATCCA	A/G	TAGTTGACAGTCAAGGTCGTGTAATTAATACATGGATTGCTATTATTAATCGTGTTAACCTTGGTATGGAAGTTATGCATGAATGTAATGCTCATAATTT
Ca_LG_1:8847892	AGCTACTTGGCCTGTAGTAGGTATTTGGTTTACCGCCTTAGGTATCAACGCTATGAATTTCAATTTAAATGGTTTCAGTTTTAACCAATCCATAGTTGAC	C/T	AGTCAAGGTCGTGTAATTAATACATGGATTGCTATTATTAATCGTGTTAACCTTGGTATGGAAGTTATGCATGAATGTAATGCTCATAATTTCCCTATAG

Positions	-100 bp flanking sequences	SNPs within *desi* cultivar	+100 bp flanking sequences
Ca_LG_1:8847916	TTGGTTTACCGCCTTAGGTATCAACGCTATGAATTTCAATTTAAATGGTTTCAGTTTTAACCAATCCATAGTTGACAGTCAAGGTCGTGTAATTAATACA	A/C	TGGATTGCTATTATTAATCGTGTTAACCTTGGTATGGAAGTTATGCATGAATGTAATGCTCATAATTTCCCTATAGACCTAACTACGGTCGAGGTTCCAT
Ca_LG_1:8847920	TTTACCGCCTTAGGTATCAACGCTATGAATTTCAATTTAAATGGTTTCAGTTTTAACCAATCCATAGTTGACAGTCAAGGTCGTGTAATTAATACATGGA	A/G	TTGCTATTATTAATCGTGTTAACCTTGGTATGGAAGTTATGCATGAATGTAATGCTCATAATTTCCCTATAGACCTAACTACGGTCGAGGTTCCATCTAT
Ca_LG_1:8847921	TTACCGCCTTAGGTATCAACGCTATGAATTTCAATTTAAATGGTTTCAGTTTTAACCAATCCATAGTTGACAGTCAAGGTCGTGTAATTAATACATGGAT	T/C	TGCTATTATTAATCGTGTTAACCTTGGTATGGAAGTTATGCATGAATGTAATGCTCATAATTTCCCTATAGACCTAACTACGGTCGAGGTTCCATCTATA
Ca_LG_1:8847924	CCGCCTTAGGTATCAACGCTATGAATTTCAATTTAAATGGTTTCAGTTTTAACCAATCCATAGTTGACAGTCAAGGTCGTGTAATTAATACATGGATTGC	C/A	TATTATTAATCGTGTTAACCTTGGTATGGAAGTTATGCATGAATGTAATGCTCATAATTTCCCTATAGACCTAACTACGGTCGAGGTTCCATCTATAAAT
Ca_LG_1:8847934	TATCAACGCTATGAATTTCAATTTAAATGGTTTCAGTTTTAACCAATCCATAGTTGACAGTCAAGGTCGTGTAATTAATACATGGATTGCTATTATTAAT	T/C	CGTGTTAACCTTGGTATGGAAGTTATGCATGAATGTAATGCTCATAATTTCCCTATAGACCTAACTACGGTCGAGGTTCCATCTATAAATGGATAATATT
Ca_LG_1:8847939	ACGCTATGAATTTCAATTTAAATGGTTTCAGTTTTAACCAATCCATAGTTGACAGTCAAGGTCGTGTAATTAATACATGGATTGCTATTATTAATCGTGT	T/C	TAACCTTGGTATGGAAGTTATGCATGAATGTAATGCTCATAATTTCCCTATAGACCTAACTACGGTCGAGGTTCCATCTATAAATGGATAATATTTTAGT
Ca_LG_1:8847968	AGTTTTAACCAATCCATAGTTGACAGTCAAGGTCGTGTAATTAATACATGGATTGCTATTATTAATCGTGTTAACCTTGGTATGGAAGTTATGCATGAAT	T/C	GTAATGCTCATAATTTCCCTATAGACCTAACTACGGTCGAGGTTCCATCTATAAATGGATAATATTTTAGTTTTTAAGAAGGATACAAGTTTTTAAAAGT
Ca_LG_1:8847989	GACAGTCAAGGTCGTGTAATTAATACATGGATTGCTATTATTAATCGTGTTAACCTTGGTATGGAAGTTATGCATGAATGTAATGCTCATAATTTCCCTA	A/C	TAGACCTAACTACGGTCGAGGTTCCATCTATAAATGGATAATATTTTAGTTTTTAAGAAGGATACAAGTTTTTAAAAGTAAAGGGCGGGTTGATTTAAAA
Ca_LG_1:8848011	ATACATGGATTGCTATTATTAATCGTGTTAACCTTGGTATGGAAGTTATGCATGAATGTAATGCTCATAATTTCCCTATAGACCTAACTACGGTCGAGGT	T/C	TCCATCTATAAATGGATAATATTTTAGTTTTTAAGAAGGATACAAGTTTTTAAAAGTAAAGGGCGGGTTGATTTAAAAGTAAAATGCTCATAATCTCAAC
Ca_LG_1:8848055	GTTATGCATGAATGTAATGCTCATAATTTCCCTATAGACCTAACTACGGTCGAGGTTCCATCTATAAATGGATAATATTTTAGTTTTTAAGAAGGATACA	A/G	AGTTTTTAAAAGTAAAGGGCGGGTTGATTTAAAAGTAAAATGCTCATAATCTCAACAATGTTGTATATTAGTTCAAATGCCACTATCGGGTTGATTTACA
Ca_LG_1:10251478	TTCATCCCATTTTTTTGGGGATAGGCGGCGAATACAAATCTAATAAGTGAAGTAGTCGTCGTCTGACCAATTGGCTCGGACACCAGACCGCTCGTGCCCA	A/G	CCCGTTCTGTCTCGCCCTAAATGGAATGACTCTCTTAGTTACGATGCGCCCCGGTTTGAGTCCCCACTTCCACTCTTCTCTGCGCGCAACCCAAGAAGCT
Ca_LG_1:10251482	TCCCATTTTTTTGGGGATAGGCGGCGAATACAAATCTAATAAGTGAAGTAGTCGTCGTCTGACCAATTGGCTCGGACACCAGACCGCTCGTGCCCACCCG	G/A	TTCTGTCTCGCCCTAAATGGAATGACTCTCTTAGTTACGATGCGCCCCGGTTTGAGTCCCCACTTCCACTCTTCTCTGCGCGCAACCCAAGAAGCTGGCT

**Positions**	**-100 bp flanking sequences**	**SNPs within *desi* cultivar**	**+100 bp flanking sequences**
Ca_LG_1:10251507	GAATACAAATCTAATAAGTGAAGTAGTCGTCGTCTGACCAATTGGCTCGGACACCAGACCGCTCGTGCCCACCCGTTCTGTCTCGCCCTAAATGGAATGA	A/G	CTCTCTTAGTTACGATGCGCCCCGGTTTGAGTCCCCACTTCCACTCTTCTCTGCGCGCAACCCAAGAAGCTGGCTTTGCCAACACAACATTAGGGCCGTC
Ca_LG_1:10251534	CGTCGTCTGACCAATTGGCTCGGACACCAGACCGCTCGTGCCCACCCGTTCTGTCTCGCCCTAAATGGAATGACTCTCTTAGTTACGATGCGCCCCGGTT	T/C	TGAGTCCCCACTTCCACTCTTCTCTGCGCGCAACCCAAGAAGCTGGCTTTGCCAACACAACATTAGGGCCGTCCCCTTCATTCTATGCCGACCCCGGCCC
Ca_LG_1:10251546	AATTGGCTCGGACACCAGACCGCTCGTGCCCACCCGTTCTGTCTCGCCCTAAATGGAATGACTCTCTTAGTTACGATGCGCCCCGGTTTGAGTCCCCACT	T/G	TCCACTCTTCTCTGCGCGCAACCCAAGAAGCTGGCTTTGCCAACACAACATTAGGGCCGTCCCCTTCATTCTATGCCGACCCCGGCCCCGGGGCTGGCTTT
Ca_LG_1:10251550	GGCTCGGACACCAGACCGCTCGTGCCCACCCGTTCTGTCTCGCCCTAAATGGAATGACTCTCTTAGTTACGATGCGCCCCGGTTTGAGTCCCCACTTCCA	A/G	CTCTTCTCTGCGCGCAACCCAAGAAGCTGGCTTTGCCAACACAACATTAGGGCCGTCCCCTTCATTCTATGCCGACCCCGGCCCGGGGCTGGCTTTTTGG
Ca_LG_1:10251559	ACCAGACCGCTCGTGCCCACCCGTTCTGTCTCGCCCTAAATGGAATGACTCTCTTAGTTACGATGCGCCCCGGTTTGAGTCCCCACTTCCACTCTTCTCT	T/C	GCGCGCAACCCAAGAAGCTGGCTTTGCCAACACAACATTAGGGCCGTCCCCTTCATTCTATGCCGACCCCGGCCCGGGGCTGGCTTTTTGGGAAGCCCGT
Ca_LG_1:10251562	AGACCGCTCGTGCCCACCCGTTCTGTCTCGCCCTAAATGGAATGACTCTCTTAGTTACGATGCGCCCCGGTTTGAGTCCCCACTTCCACTCTTCTCTGCG	G/C	CGCAACCCAAGAAGCTGGCTTTGCCAACACAACATTAGGGCCGTCCCCTTCATTCTATGCCGACCCCGGCCCGGGGCTGGCTTTTTGGGAAGCCCGTTCC
Ca_LG_1:10251577	ACCCGTTCTGTCTCGCCCTAAATGGAATGACTCTCTTAGTTACGATGCGCCCCGGTTTGAGTCCCCACTTCCACTCTTCTCTGCGCGCAACCCAAGAAGC	C/T	TGGCTTTGCCAACACAACATTAGGGCCGTCCCCTTCATTCTATGCCGACCCCGGCCCGGGGCTGGCTTTTTGGGAAGCCCGTTCCCACCGCGCTCACGGC
Ca_LG_1:10761209	TTCACGTGAACCTTCTGATGCAAAGTTGCTGGTGAGTGATGCTGATGAAGAAAGGAATGCAGCTAAGACAACCCAATCTTCAATCCAGAATACTAATAGC	C/A	AGTGGTATAGATGTTGAAAAGTCCTGCAATGCTGCTACCACTGAAGCAGGCTTTAAAAGTTCATCAATAGAGAATAATTCAGGCAAAGTACATGATGAAT
Ca_LG_1:11501507	TGGATCCTACCAAGAATGGTCTGAAGAGAAAAGACAAGAATGGCTTTTGTCTGAGTTGAGTGGGAAAAGGCCATTGTTTGGTCCCGACCTTCCTCAAACC	C/T	GAAGAAATTCAAGATGTCTTGGAGACATTTCGTGTCATAGCAGAATTACCATCAGACAACTTTGGAGCCTATATCATATCTATGGCAACTGCACCATCTG
Ca_LG_1:11501517	CAAGAATGGTCTGAAGAGAAAAGACAAGAATGGCTTTTGTCTGAGTTGAGTGGGAAAAGGCCATTGTTTGGTCCCGACCTTCCTCAAACCGAAGAAATTC	C/A	AAGATGTCTTGGAGACATTTCGTGTCATAGCAGAATTACCATCAGACAACTTTGGAGCCTATATCATATCTATGGCAACTGCACCATCTGATGTGCTTGC
Ca_LG_1:11501518	AAGAATGGTCTGAAGAGAAAAGACAAGAATGGCTTTTGTCTGAGTTGAGTGGGAAAAGGCCATTGTTTGGTCCCGACCTTCCTCAAACCGAAGAAATTCA	A/G	AGATGTCTTGGAGACATTTCGTGTCATAGCAGAATTACCATCAGACAACTTTGGAGCCTATATCATATCTATGGCAACTGCACCATCTGATGTGCTTGCG
Ca_LG_1:11501525	GTCTGAAGAGAAAAGACAAGAATGGCTTTTGTCTGAGTTGAGTGGGAAAAGGCCATTGTTTGGTCCCGACCTTCCTCAAACCGAAGAAATTCAAGATGTC	C/T	TTGGAGACATTTCGTGTCATAGCAGAATTACCATCAGACAACTTTGGAGCCTATATCATATCTATGGCAACTGCACCATCTGATGTGCTTGCGGTCGAAC

Positions	-100 bp flanking sequences	SNPs within *desi* cultivar	+100 bp flanking sequences
Ca_LG_1:11501531	AGAGAAAAGACAAGAATGGCTTTTGTCTGAGTTGAGTGGGAAAAGGCCATTGTTTGGTCCCGACCTTCCTCAAACCGAAGAAATTCAAGATGTCTTGGAG	G/C	ACATTTCGTGTCATAGCAGAATTACCATCAGACAACTTTGGAGCCTATATCATATCTATGGCAACTGCACCATCTGATGTGCTTGCGGTCGAACTTCTTC
Ca_LG_1:11501534	GAAAAGACAAGAATGGCTTTTGTCTGAGTTGAGTGGGAAAAGGCCATTGTTTGGTCCCGACCTTCCTCAAACCGAAGAAATTCAAGATGTCTTGGAGACA	A/G	TTTCGTGTCATAGCAGAATTACCATCAGACAACTTTGGAGCCTATATCATATCTATGGCAACTGCACCATCTGATGTGCTTGCGGTCGAACTTCTTCAAC
Ca_LG_1:11501539	GACAAGAATGGCTTTTGTCTGAGTTGAGTGGGAAAAGGCCATTGTTTGGTCCCGACCTTCCTCAAACCGAAGAAATTCAAGATGTCTTGGAGACATTTCG	G/A	TGTCATAGCAGAATTACCATCAGACAACTTTGGAGCCTATATCATATCTATGGCAACTGCACCATCTGATGTGCTTGCGGTCGAACTTCTTCAACGCGAA
Ca_LG_1:11501553	TTGTCTGAGTTGAGTGGGAAAAGGCCATTGTTTGGTCCCGACCTTCCTCAAACCGAAGAAATTCAAGATGTCTTGGAGACATTTCGTGTCATAGCAGAAT	T/C	TACCATCAGACAACTTTGGAGCCTATATCATATCTATGGCAACTGCACCATCTGATGTGCTTGCGGTCGAACTTCTTCAACGCGAATGCCACATCAAGAA
Ca_LG_1:11501576	GCCATTGTTTGGTCCCGACCTTCCTCAAACCGAAGAAATTCAAGATGTCTTGGAGACATTTCGTGTCATAGCAGAATTACCATCAGACAACTTTGGAGCC	C/A	TATATCATATCTATGGCAACTGCACCATCTGATGTGCTTGCGGTCGAACTTCTTCAACGCGAATGCCACATCAAGAATCCGTTAAGAGTTGTACCGTTGT
Ca_LG_1:11501750	GAATCCGTTAAGAGTTGTACCGTTGTTTGAGAAGCTTGATGATCTTGAGGCTGCTCCTGCTGCGTTGGCTCGGCTGTTCTCAGTAGAATGGTACAGAAAC	C/T	CGGATCGATGGTAAGCAAGAAGTCATGATTGGATATTCGGATTCAGGAAAAGACGCCGGAAGGTTTTCTGCAGCATGGCAGCTATATAAGGCTCAAGAGG
Ca_LG_1:11501757	TTAAGAGTTGTACCGTTGTTTGAGAAGCTTGATGATCTTGAGGCTGCTCCTGCTGCGTTGGCTCGGCTGTTCTCAGTAGAATGGTACAGAAACCGGATCG	G/A	ATGGTAAGCAAGAAGTCATGATTGGATATTCGGATTCAGGAAAAGACGCCGGAAGGTTTTCTGCAGCATGGCAGCTATATAAGGCTCAAGAGGAACTCAG
Ca_LG_1:11501759	AAGAGTTGTACCGTTGTTTGAGAAGCTTGATGATCTTGAGGCTGCTCCTGCTGCGTTGGCTCGGCTGTTCTCAGTAGAATGGTACAGAAACCGGATCGAT	T/C	GGTAAGCAAGAAGTCATGATTGGATATTCGGATTCAGGAAAAGACGCCGGAAGGTTTTCTGCAGCATGGCAGCTATATAAGGCTCAAGAGGAACTCAGAA
Ca_LG_1:11501762	AGTTGTACCGTTGTTTGAGAAGCTTGATGATCTTGAGGCTGCTCCTGCTGCGTTGGCTCGGCTGTTCTCAGTAGAATGGTACAGAAACCGGATCGATGGT	T/G	AAGCAAGAAGTCATGATTGGATATTCGGATTCAGGAAAAGACGCCGGAAGGTTTTCTGCAGCATGGCAGCTATATAAGGCTCAAGAGGAACTCAGAAATG
Ca_LG_1:11501783	GCTTGATGATCTTGAGGCTGCTCCTGCTGCGTTGGCTCGGCTGTTCTCAGTAGAATGGTACAGAAACCGGATCGATGGTAAGCAAGAAGTCATGATTGGA	A/G	TATTCGGATTCAGGAAAAGACGCCGGAAGGTTTTCTGCAGCATGGCAGCTATATAAGGCTCAAGAGGAACTCAGAAATGTTGCTCAGAAATTCGGTATTA
Ca_LG_1:11501789	TGATCTTGAGGCTGCTCCTGCTGCGTTGGCTCGGCTGTTCTCAGTAGAATGGTACAGAAACCGGATCGATGGTAAGCAAGAAGTCATGATTGGATATTCG	G/T	GATTCAGGAAAAGACGCCGGAAGGTTTTCTGCAGCATGGCAGCTATATAAGGCTCAAGAGGAACTCAGAAATGTTGCTCAGAAATTCGGTATTAAGCTAA
Ca_LG_1:11501798	GGCTGCTCCTGCTGCGTTGGCTCGGCTGTTCTCAGTAGAATGGTACAGAAACCGGATCGATGGTAAGCAAGAAGTCATGATTGGATATTCGGATTCAGGA	A/T	AAAGACGCCGGAAGGTTTTCTGCAGCATGGCAGCTATATAAGGCTCAAGAGGAACTCAGAAATGTTGCTCAGAAATTCGGTATTAAGCTAACCATGTTCC

**Positions**	**-100 bp flanking sequences**	**SNPs within *desi* cultivar**	**+100 bp flanking sequences**
Ca_LG_1:11501804	TCCTGCTGCGTTGGCTCGGCTGTTCTCAGTAGAATGGTACAGAAACCGGATCGATGGTAAGCAAGAAGTCATGATTGGATATTCGGATTCAGGAAAAGAC	C/T	GCCGGAAGGTTTTCTGCAGCATGGCAGCTATATAAGGCTCAAGAGGAACTCAGAAATGTTGCTCAGAAATTCGGTATTAAGCTAACCATGTTCCACGGTC
Ca_LG_1:11502510	GATCTTTGCATGGACACAAACAAGGTTTCATCTTCCAGTATGGCTAGGATTTGGAGCAGCATTTAAACAAGTTATTGAGAAGGATGTTAAGAATCTTCAT	T/A	ATGCTGCAAGAGATGTACAATAAATGGCCTTTCTTTAGGGTCACAATTGATTTAGTTGAAATGGTGTTTGCCAAAGGAGACCCTGGAATTGCAGCTTTGA
Ca_LG_1:11502516	TGCATGGACACAAACAAGGTTTCATCTTCCAGTATGGCTAGGATTTGGAGCAGCATTTAAACAAGTTATTGAGAAGGATGTTAAGAATCTTCATATGCTG	G/A	CAAGAGATGTACAATAAATGGCCTTTCTTTAGGGTCACAATTGATTTAGTTGAAATGGTGTTTGCCAAAGGAGACCCTGGAATTGCAGCTTTGAATGATA
Ca_LG_1:11502532	AGGTTTCATCTTCCAGTATGGCTAGGATTTGGAGCAGCATTTAAACAAGTTATTGAGAAGGATGTTAAGAATCTTCATATGCTGCAAGAGATGTACAATA	A/C	AATGGCCTTTCTTTAGGGTCACAATTGATTTAGTTGAAATGGTGTTTGCCAAAGGAGACCCTGGAATTGCAGCTTTGAATGATAGGCTCCTAGTTTCACA
Ca_LG_1:11502555	AGGATTTGGAGCAGCATTTAAACAAGTTATTGAGAAGGATGTTAAGAATCTTCATATGCTGCAAGAGATGTACAATAAATGGCCTTTCTTTAGGGTCACA	A/T	ATTGATTTAGTTGAAATGGTGTTTGCCAAAGGAGACCCTGGAATTGCAGCTTTGAATGATAGGCTCCTAGTTTCACAGGATCTGTGGCCATTTGGTGAGC
Ca_LG_1:11502567	AGCATTTAAACAAGTTATTGAGAAGGATGTTAAGAATCTTCATATGCTGCAAGAGATGTACAATAAATGGCCTTTCTTTAGGGTCACAATTGATTTAGTT	T/G	GAAATGGTGTTTGCCAAAGGAGACCCTGGAATTGCAGCTTTGAATGATAGGCTCCTAGTTTCACAGGATCTGTGGCCATTTGGTGAGCAGTTGAGGAACA
Ca_LG_1:11502585	TGAGAAGGATGTTAAGAATCTTCATATGCTGCAAGAGATGTACAATAAATGGCCTTTCTTTAGGGTCACAATTGATTTAGTTGAAATGGTGTTTGCCAAA	A/G	GGAGACCCTGGAATTGCAGCTTTGAATGATAGGCTCCTAGTTTCACAGGATCTGTGGCCATTTGGTGAGCAGTTGAGGAACAAATATGAAGAAACTAAGA
Ca_LG_1:11503094	AAGGATCCGCGATCCAAACTACAGCGTGACGGTGCGCCCTCACATTTCAAGAGAGTCCGTGGAAACAAGTAAACCAGCCGATGAACTTGTAACACTGAAT	T/C	CCAACAAGTGAATTTGGTCCTGGATTGGAAGACACACTCATTCTTACCATGAAGGGTATTGCTGCTGGAATGCAGAACACTGGTTAAATTTTTGGTTACT
Ca_LG_1:11503106	TCCAAACTACAGCGTGACGGTGCGCCCTCACATTTCAAGAGAGTCCGTGGAAACAAGTAAACCAGCCGATGAACTTGTAACACTGAATCCAACAAGTGAA	A/G	TTTGGTCCTGGATTGGAAGACACACTCATTCTTACCATGAAGGGTATTGCTGCTGGAATGCAGAACACTGGTTAAATTTTTGGTTACTTTGTATTTGTTT
Ca_LG_1:11503108	CAAACTACAGCGTGACGGTGCGCCCTCACATTTCAAGAGAGTCCGTGGAAACAAGTAAACCAGCCGATGAACTTGTAACACTGAATCCAACAAGTGAATT	T/A	TGGTCCTGGATTGGAAGACACACTCATTCTTACCATGAAGGGTATTGCTGCTGGAATGCAGAACACTGGTTAAATTTTTGGTTACTTTGTATTTGTTTCT
Ca_LG_1:11503111	ACTACAGCGTGACGGTGCGCCCTCACATTTCAAGAGAGTCCGTGGAAACAAGTAAACCAGCCGATGAACTTGTAACACTGAATCCAACAAGTGAATTTGG	G/C	TCCTGGATTGGAAGACACACTCATTCTTACCATGAAGGGTATTGCTGCTGGAATGCAGAACACTGGTTAAATTTTTGGTTACTTTGTATTTGTTTCTTAA
Ca_LG_1:11503112	CTACAGCGTGACGGTGCGCCCTCACATTTCAAGAGAGTCCGTGGAAACAAGTAAACCAGCCGATGAACTTGTAACACTGAATCCAACAAGTGAATTTGGT	T/A	CCTGGATTGGAAGACACACTCATTCTTACCATGAAGGGTATTGCTGCTGGAATGCAGAACACTGGTTAAATTTTTGGTTACTTTGTATTTGTTTCTTAAT

Positions	-100 bp flanking sequences	SNPs within *desi* cultivar	+100 bp flanking sequences
Ca_LG_1:11503118	CGTGACGGTGCGCCCTCACATTTCAAGAGAGTCCGTGGAAACAAGTAAACCAGCCGATGAACTTGTAACACTGAATCCAACAAGTGAATTTGGTCCTGGA	A/T	TTGGAAGACACACTCATTCTTACCATGAAGGGTATTGCTGCTGGAATGCAGAACACTGGTTAAATTTTTGGTTACTTTGTATTTGTTTCTTAATGTAAGT
Ca_LG_1:11503127	GCGCCCTCACATTTCAAGAGAGTCCGTGGAAACAAGTAAACCAGCCGATGAACTTGTAACACTGAATCCAACAAGTGAATTTGGTCCTGGATTGGAAGAC	C/T	ACACTCATTCTTACCATGAAGGGTATTGCTGCTGGAATGCAGAACACTGGTTAAATTTTTGGTTACTTTGTATTTGTTTCTTAATGTAAGTGTGCTAAGA
Ca_LG_1:11503130	CCCTCACATTTCAAGAGAGTCCGTGGAAACAAGTAAACCAGCCGATGAACTTGTAACACTGAATCCAACAAGTGAATTTGGTCCTGGATTGGAAGACACA	A/T	CTCATTCTTACCATGAAGGGTATTGCTGCTGGAATGCAGAACACTGGTTAAATTTTTGGTTACTTTGTATTTGTTTCTTAATGTAAGTGTGCTAAGATAT
Ca_LG_1:11503139	TTCAAGAGAGTCCGTGGAAACAAGTAAACCAGCCGATGAACTTGTAACACTGAATCCAACAAGTGAATTTGGTCCTGGATTGGAAGACACACTCATTCTT	T/C	ACCATGAAGGGTATTGCTGCTGGAATGCAGAACACTGGTTAAATTTTTGGTTACTTTGTATTTGTTTCTTAATGTAAGTGTGCTAAGATATCATAAATAC
Ca_LG_1:11989386	GATTGCTGTTAAACAGCTCTCTTCGAAATCAAAGCAAGGGAACCGCGAATTCGTTAACGAAATAGGCATGATATCTGCTTTGCAACATCCAAATCTTGTC	C/T	AAGCTTTATGGCTGTTGCATTGAAGGAAACCAATTGCTGCTTGTATATGAATACATGGAGAACAATAGTCTTGCTCGCGCTCTTTTCGGTAAATGCTTCA
Ca_LG_1:12494130	GAGTATGGACAAGAGCTCCATCTACTGCTGATCATGCACAATCCAGTGCTGATCAAGCCCAAGCGAGTGCCCAACCACCTGCATCAGCTGAAGCATCTTT	T/C	TGACATTATGACGAAGCTGCTGGAGTTCATGAAGATTCAAGCTGCCCACAACGAAAGAGTGGAAGCCTCTCTTCGAAAGCTCCAATCAACTTTGAACTCT
Ca_LG_1:12557788	ATTCCTTTAGTTTCTGGCAGTATGAAGTATACAAAGATTGTCATAGTCACAAGCGGCAAAGAATATGAACAATCCAAACTTCATATGGCAGAGCATGGTT	T/C	AAGAAACATTGTGCCACAAGAAACTTGTGCTCTATGCTTCCTTTCCCGCAATTTTCTCAAAGGCTGTTCAACTCTCGTTGAAGCTATACTCGCTGCCACA
Ca_LG_1:13171929	TGGGAATGAGTATCTCATTAATCTCATTGATTCACCTGGACACGTTGACTTCTCATCTGAGGTTACTGCTGCACTCCGTATTACTGATGGAGCACTAGTG	G/T	GTGGTGGATTGTGTTGAGGGTGTTTGTGTCCAAACTGAAACTGTTCTGCGACAGGCTCTTGGTGAAAGGATCAGGCCTGTTCTGACTGTTAACAAGATGG
Ca_LG_1:14432014	ATAGTATCAACTGACATACCTAAAATATGTATGTGTGCAGTGCTGCTATGATAAACCAAATAACGAAGGTTGCTGCTAGCTACAACAGTGAAGTCAAGAC	C/A	AGTCTATCAACAAAGGAAACAAAAAAAAAACTTCATCAATAAATAATAAATGATTTAATTAGCAACAAAGTAGTAGAAAAAAATAGAGAAAGAGTTGAAA
Ca_LG_1:14432018	TATCAACTGACATACCTAAAATATGTATGTGTGCAGTGCTGCTATGATAAACCAAATAACGAAGGTTGCTGCTAGCTACAACAGTGAAGTCAAGACAGTC	C/A	TATCAACAAAGGAAACAAAAAAAAAACTTCATCAATAAATAATAAATGATTTAATTAGCAACAAAGTAGTAGAAAAAAATAGAGAAAGAGTTGAAATTGA
Ca_LG_1:14432023	ACTGACATACCTAAAATATGTATGTGTGCAGTGCTGCTATGATAAACCAAATAACGAAGGTTGCTGCTAGCTACAACAGTGAAGTCAAGACAGTCTATCA	A/T	ACAAAGGAAACAAAAAAAAAACTTCATCAATAAATAATAAATGATTTAATTAGCAACAAAGTAGTAGAAAAAAATAGAGAAAGAGTTGAAATTGAATAAC
Ca_LG_1:14432048	GTGCAGTGCTGCTATGATAAACCAAATAACGAAGGTTGCTGCTAGCTACAACAGTGAAGTCAAGACAGTCTATCAACAAAGGAAACAAAAAAAAAACTTC	C/A	ATCAATAAATAATAAATGATTTAATTAGCAACAAAGTAGTAGAAAAAAATAGAGAAAGAGTTGAAATTGAATAACAAAAAAAATTACCGTTTCCAACACC

**Positions**	**-100 bp flanking sequences**	**SNPs within *desi* cultivar**	**+100 bp flanking sequences**
Ca_LG_1:14432057	TGCTATGATAAACCAAATAACGAAGGTTGCTGCTAGCTACAAC AGTGAAGTCAAGACAGTCTATCAACAAAGGAAACAAAAAAAAA ACTTCATCAATAAA	A/G	TAATAAATGATTTAATTAGCAACAAAGTAGTAGAAAAAAATAG AGAAAGAGTTGAAATTGAATAACAAAAAAAATTACCGTTTCC AACACCAAAAATGGA
Ca_LG_1:14432122	AACAAAGGAAACAAAAAAAAACTTCATCAATAAATAATAAAT GATTTAATTAGCAACAAAGTAGTAGAAAAAAATAGAGAAAGAG TTGAAATTGAATAA	A/C	CAAAAAAAATTACCGTTTCCAACACCAAAAATGGAAAAATGC AGTACATTGGTCAACACAGTAGTACAACAATGCAACAAAAAT GCAGAAAATGCACCAA
Ca_LG_1:14432126	AAGGAAACAAAAAAAAACTTCATCAATAAATAATAAATGATTT AATTAGCAACAAAGTAGTAGAAAAAAATAGAGAAAGAGTTGAA ATTGAATAACAAA	A/G	AAAAATTACCGTTTCCAACACCAAAAATGGAAAAATGCAGTA CATTGGTCAACACAGTAGTACAACAATGCAACAAAAATGCAG AAAATGCACCAAAAAT
Ca_LG_1:14648077	GGAATGAAGAAACCAGACTCAATGGCAACTCAATTCTTGGCC CAAAAATCATTTGCGCAAACGCAATTAGCAGCAACAAAATTCG AAGAGGAATACTTTG	G/A	ACATCTTGGCCCAACTTCTCAAAAGCAAGGTCCAATCCTCAA AACCTTTCAAAGGGCAGCGCTTCGGAAGACACCATTAATTTG GAAAATGACAACGAAG
Ca_LG_1:14648083	AAGAAACCAGACTCAATGGCAACTCAATTCTTGGCCCAAAAAT CATTTGCGCAAACGCAATTAGCAGCAACAAAATTCGAAGAGG AATACTTTGACATCT	T/A	TGGCCCAACTTCTCAAAAGCAAGGTCCAATCCTCAAAACCTT TCAAAGGGCAGCGCTTCGGAAGACACCATTAATTTGGAAAA TGACAACGAAGACGATT
Ca_LG_1:15325954	TTTGTATCTTTCAGCAACCATACCAAATATAATGTTTATTGCTA GCTTACCTTCCAGGTTAATGCATTCTCCAAGCCAAGTCCACCT TGGTGCAGCAAAA	A/G	GGAGTTTAAAGGTACTTAAAAGGTACAATTCATTTTGACTTG TGTGTTTTTGAAAAATAGAAGTGGAGACCTCCAAGGTTATGC AGATAGTGATTGAGCA
Ca_LG_1:15325955	TTGTATCTTTCAGCAACCATACCAAATATAATGTTTATTGCTAG CTTACCTTCCAGGTTAATGCATTCTCCAAGCCAAGTCCACCTT GGTGCAGCAAAAG	G/A	GAGTTTAAAGGTACTTAAAAGGTACAATTCATTTTGACTTGT GTGTTTTTGAAAAATAGAAGTGGAGACCTCCAAGGTTATGCA GATAGTGATTGAGCAG
Ca_LG_1:15325962	TTTCAGCAACCATACCAAATATAATGTTTATTGCTAGCTTACCT TCCAGGTTAATGCATTCTCCAAGCCAAGTCCACCTTGGTGCA GCAAAAGGAGTTTA	A/T	AAGGTACTTAAAAGGTACAATTCATTTTGACTTGTGTGTTTTT GAAAAATAGAAGTGGAGACCTCCAAGGTTATGCAGATAGTG ATTGAGCAGGAAGTGT
Ca_LG_1:15365785	GGTTTAGAGAGAAGTTAGAAGTGAAAAATGATAAGAGGAGTG GAGTAATATATATAGAATTTGGTTGATCCTTTGTGGCGGCTAA AGCAGCCACAATGAG	G/A	CAATGGATATATGGCGTTTGTGGCGGCCAAAACGGTCAGAA ATGCAGGGATTTGTGGCGGCCTGGGCGGCCACAACTGACA ACAGTCATTTGGGTGTTTG
Ca_LG_1:15365787	TTTAGAGAGAAGTTAGAAGTGAAAAATGATAAGAGGAGTGGA GTAATATATATAGAATTTGGTTGATCCTTTGTGGCGGCTAAAG CAGCCACAATGAGCA	A/G	ATGGATATATGGCGTTTGTGGCGGCCAAAACGGTCAGAAAT GCAGGGATTTGTGGCGGCCTGGGCGGCCACAACTGACAAC AGTCATTTGGGTGTTTGTG
Ca_LG_1:15365789	TAGAGAGAAGTTAGAAGTGAAAAATGATAAGAGGAGTGGAGT AATATATATAGAATTTGGTTGATCCTTTGTGGCGGCTAAAGCA GCCACAATGAGCAAT	T/C	GGATATATGGCGTTTGTGGCGGCCAAAACGGTCAGAAATGC AGGGATTTGTGGCGGCCTGGGCGGCCACAACTGACAACAG TCATTTGGGTGTTTGTGGC
Ca_LG_1:15365791	GAGAGAAGTTAGAAGTGAAAAATGATAAGAGGAGTGGAGTAA TATATATAGAATTTGGTTGATCCTTTGTGGCGGCTAAAGCAGC CACAATGAGCAATGG	G/A	ATATATGGCGTTTGTGGCGGCCAAAACGGTCAGAAATGCAG GGATTTGTGGCGGCCTGGGCGGCCACAACTGACAACAGTC ATTTGGGTGTTTGTGGCGG

**Positions**	**-100 bp flanking sequences**	**SNPs within *desi* cultivar**	**+100 bp flanking sequences**
Ca_LG_1:15582244	CCTCACAAATTTTTAAATATTTAACTGGATTTTGTGAGGGCTTTTACAGCCACAAAAAATCAATTTTGTGAGGGCCAAATCCGTCACAAAGGCAAACGGG	G/A	ACAAGTGGCATTTGTGGCTGCAAAGGCCGCCACAAAGGAGGCCCAATACAGCTGGTGTTTGTGGCCGCTTAGGCCGTCACAACTGATTGTACCGTTGGAT
Ca_LG_1:15582253	TTTTTAAATATTTAACTGGATTTTGTGAGGGCTTTTACAGCCACAAAAAATCAATTTTGTGAGGGCCAAATCCGTCACAAAGGCAAACGGGACAAGTGGC	C/A	ATTTGTGGCTGCAAAGGCCGCCACAAAGGAGGCCCAATACAGCTGGTGTTTGTGGCCGCTTAGGCCGTCACAACTGATTGTACCGTTGGATTTTCTGGCG
Ca_LG_1:16125628	GACTAGAGCTCATCCGTCTACTGAACAAGTGCAGCCAACTGCAGAGCAAGCTGAGTCAGTCGAAGCACATTATGTGGTCTTAATGAAGCTGCTGGAATTC	C/T	ATGGAGATTCAAGTTGCTCATAATGCAAGAGTATAAGCATCTCTCAAGACTCTCCAGGCATCGCTTGATTCCGTCGTTCAGGATGTGGCTGCAATACAAG
Ca_LG_1:16125652	ACAAGTGCAGCCAACTGCAGAGCAAGCTGAGTCAGTCGAAGCACATTATGTGGTCTTAATGAAGCTGCTGGAATTCATGGAGATTCAAGTTGCTCATAAT	T/C	GCAAGAGTATAAGCATCTCTCAAGACTCTCCAGGCATCGCTTGATTCCGTCGTTCAGGATGTGGCTGCAATACAAGAACATCTAGGCCTTAAGATGTCAT
Ca_LG_1:16153461	AAATATAAGAAAAAATATAAAATCAAATCAACTTTTAATTCTAGAGTGGAAAAAAAAATAGAGAGAGAGGTGGGGGCGGCTGTATAGAAACAGAGTTGCTG	G/C	GAAAAAGAAAGAAAAGAGAGGAAGAAGAAAAAAGAAAGGAAAGGAAAAGCATAGAAAAGAAATAGAGGACTTGCAGTGCGGATGTTGATCGCGCAATGGC
Ca_LG_1:16153474	AATATAAAATCAAATCAACTTTTAATTCTAGAGTGGAAAAAAAATAGAGAGAGAGGTGGGGGCGGCTGTATAGAAACAGAGTTGCTGGAAAAAGAAAGAA	A/G	AAGAGAGGAAGAAGAAAAAAGAAAGGAAAGGAAAAGCATAGAAAAGAAATAGAGGACTTGCAGTGCGGATGTTGATCGCGCAATGGCGGCAGGAGGGATG
Ca_LG_1:16153477	ATAAAATCAAATCAACTTTTAATTCTAGAGTGGAAAAAAAAATAGAGAGAGAGGTGGGGGCGGCTGTATAGAAACAGAGTTGCTGGAAAAAGAAAGAAAAG	G/T	AGAGGAAGAAGAAAAAAGAAAGGAAAGGAAAAGCATAGAAAAGAAATAGAGGACTTGCAGTGCGGATGTTGATCGCGCAATGGCGGCAGGAGGGATGCTC
Ca_LG_1:16153479	AAAATCAAATCAACTTTTAATTCTAGAGTGGAAAAAAAAATAGAGAGAGAGGTGGGGGCGGCTGTATAGAAACAGAGTTGCTGGAAAAAGAAAGAAAAGAG	G/A	AGGAAGAAGAAAAAAGAAAGGAAAGGAAAAGCATAGAAAAGAAATAGAGGACTTGCAGTGCGGATGTTGATCGCGCAATGGCGGCAGGAGGGATGCTCTT
Ca_LG_1:16265728	NNNNNNNNNNNNNNNNNNNNNNNNNNNNNAGCCACAATGAGCAACGGATATATGGCTTTTGTGGCGGCCAAAACGGTCAGAAAGGCATGCGTTTGTGGCGG	G/A	CTTAGGCGGCCACAAATGGCAGCGGTCCTTTTGGAGTTTGGGGCGCCCTTTGCAGACACAAAAGGTAACGACTATAAGCGTTTGTGGCGGCCTGGGCGGC
Ca_LG_1:16265888	AAAGGTAACGACTATAAGCGTTTGTGGCGGCCTGGGCGGCCACAAATGGCAACGTCACTTTGGGATTTTGTGGCGGCTTTTCCAGCCACAAAAGGTAACG	G/A	ACTCAGGCTTTTGTGGCGGCCTTTGCAGCCACAAAAGGTAACGTCACTTTTGGATTTTGTGGCGGCCTTTACCCTCACAAAGGGTTGATTTTGTGGCTGA
Ca_LG_1:16759724	ATTCTCGTCCATCTAGATGTTGCGCCAATTTCTTGTCTCAACCATCTAACGAGATAGCTGCACAGATAGTAGCATTTGAGGAGCGAGCAAAAGTGGCAGA	A/C	AAACGAGGCACGAGAGGCACAAGAGGAGCTAAGGAAAGTAGAGCAACGTCGACAAGAGGAAGTTCAACAAACAGAGCAACACACTACAGAGCTATAGATG
Ca_LG_1:16759726	TCTCGTCCATCTAGATGTTGCGCCAATTTCTTGTCTCAACCATCTAACGAGATAGCTGCACAGATAGTAGCATTTGAGGAGCGAGCAAAAGTGGCAGAAA	A/C	ACGAGGCACGAGAGGCACAAGAGGAGCTAAGGAAAGTAGAGCAACGTCGACAAGAGGAAGTTCAACAAACAGAGCAACACACTACAGAGCTATAGATGCA

Positions	-100 bp flanking sequences	SNPs within *desi* cultivar	+100 bp flanking sequences
Ca_LG_1:16759735	TCTAGATGTTGCGCCAATTTCTTGTCTCAACCATCTAACGAGATAGCTGCACAGATAGTAGCATTTGAGGAGCGAGCAAAAGTGGCAGAAAACGAGGCAC	C/T	GAGAGGCACAAGAGGAGCTAAGGAAAGTAGAGCAACGTCGACAAGAGGAAGTTCAACAAACAGAGCAACACACTACAGAGCTATAGATGCAGCTAGCAAC
Ca_LG_1:16759738	AGATGTTGCGCCAATTTCTTGTCTCAACCATCTAACGAGATAGCTGCACAGATAGTAGCATTTGAGGAGCGAGCAAAAGTGGCAGAAAACGAGGCACGAG	G/A	AGGCACAAGAGGAGCTAAGGAAAGTAGAGCAACGTCGACAAGAGGAAGTTCAACAAACAGAGCAACACACTACAGAGCTATAGATGCAGCTAGCAACATT
Ca_LG_1:16759763	AACCATCTAACGAGATAGCTGCACAGATAGTAGCATTTGAGGAGCGAGCAAAAGTGGCAGAAAACGAGGCACGAGAGGCACAAGAGGAGCTAAGGAAAGT	T/A	AGAGCAACGTCGACAAGAGGAAGTTCAACAAACAGAGCAACACACTACAGAGCTATAGATGCAGCTAGCAACATTGGCAAAACGTGTTGCTTCCATACAG
Ca_LG_1:16759803	GGAGCGAGCAAAAGTGGCAGAAAACGAGGCACGAGAGGCACAAGAGGAGCTAAGGAAAGTAGAGCAACGTCGACAAGAGGAAGTTCAACAAACAGAGCAA	A/G	CACACTACAGAGCTATAGATGCAGCTAGCAACATTGGCAAAACGTGTTGCTTCCATACAGGCCGAGTCATCTCGTCGTCGTCGTCATCCAGATTATGACG
Ca_LG_1:16759805	AGCGAGCAAAAGTGGCAGAAAACGAGGCACGAGAGGCACAAGAGGAGCTAAGGAAAGTAGAGCAACGTCGACAAGAGGAAGTTCAACAAACAGAGCAACA	A/G	CACTACAGAGCTATAGATGCAGCTAGCAACATTGGCAAAACGTGTTGCTTCCATACAGGCCGAGTCATCTCGTCGTCGTCGTCATCCAGATTATGACGAG
Ca_LG_1:17238435	CCAGGTGAAATACCAGCAGCATTACGCTCTTTCTCAGCCCATTGAATATGCAATGCCTCAAGTTGGCTATGTAAGATTTTATTCTGAAAATATGATGTGG	G/T	AGTTGATTGTGTTAATGAAACTAGCAGCCAACATGGTATAATATAAGACATACACATAAGTAAAAGCATGCAACTATGATGACTATGGATTTCTGACTTC
Ca_LG_1:17567993	AAACATGGGTTCATCAGGCTGGTGCTGCTGACCATGTTCATCATCAAAATGTTGCTGCTGCTGGTCATATTCATCATAAAACACATGATCGTGCTGGGGT	T/C	TGCTCATCCTGGGGCTGCTCATCCTGTTCTGCATCAAACATAAGATTTTGCTGTGGCTGCTCATCCTGGGGCTGCTCGTCCTGAGGCGCCTGCCTTTCCT
Ca_LG_1:18689505	GATATAACCACCATTGACTAAATTGCTACTGTGGGATTCTTAAAGTATGATTCCTCGTTCTCATGTCATTCCTCCTGTAGCCTGTATTATCACTCCATCG	G/A	TTTACAAAGTAAAAACCTTAAAACACATAAACTGTTCAAATATAGACCACAAGGACTGCCCTAAAAATCAACTAATTTAATATTAATCAAATAAATATAT
Ca_LG_1:18689527	TTGCTACTGTGGGATTCTTAAAGTATGATTCCTCGTTCTCATGTCATTCCTCCTGTAGCCTGTATTATCACTCCATCGTTTACAAAGTAAAAACCTTAAA	A/C	ACACATAAACTGTTCAAATATAGACCACAAGGACTGCCCTAAAAATCAACTAATTTAATATTAATCAAATAAATATATATAATTATATGTTACAAATTTT
Ca_LG_1:18691570	ATGCGACCACATTAAGGGAGGCGGCCAGGCAGTTCAGAACAAACTCAATGGCTAGGCAACGAACAAAAAGAAGCGAAAACGAAGAAGGACGAAGAAACGA	A/G	CGAGCAGTGACAACGGCGCGCAGTGACGACGGCGAGCAGTGGCGACGGCGAGCAGCGACCACGACGAGCAGCGACCACGGAGAGCAAGTGACCAGGACGA
Ca_LG_1:18898195	TCGATCACATTTTGTTGCTTCTCTTGAGTCGGGTAGTGAGAAGAATACGCCATTTGGCTAACACCTGGTGCAAATTTCATCACATTATGTTGCTGCAGTT	T/G	GCTGCTCTTGAGTCTGGTAGTGAGAAGAATTTGCCATTTGGCTAGCACTTGGTACAGATTCCATCAAATGTTGTCGCTCCTGTAGCTGCTCTGGAGTCTG
Ca_LG_1:18898227	GTAGTGAGAAGAATACGCCATTTGGCTAACACCTGGTGCAAATTTCATCACATTATGTTGCTGCAGTTGCTGCTCTTGAGTCTGGTAGTGAGAAGAATTT	T/C	GCCATTTGGCTAGCACTTGGTACAGATTCCATCAAATGTTGTCGCTCCTGTAGCTGCTCTGGAGTCTGGTTTTGAGAAAATACACCATTTGGCTAGCCAC

**Positions**	**-100 bp flanking sequences**	**SNPs within *desi* cultivar**	**+100 bp flanking sequences**
Ca_LG_1:18898367	GTCGCTCCTGTAGCTGCTCTGGAGTCTGGTTTTGAGAAAATACACCATTTGGCTAGCCACTGGTGCAGACTTCATCCCATTTTGTTGCTGCTCTTGAGTG	G/C	TGGTAGTGAGAAGAATTCGCCATTTCGCTAGCACCTGGTAGAGATTCCATCACATGTTGTTGCTGCTGTTGAGGCTCTTGAGTTGGGTTTTGAGAAGAAT
Ca_LG_1:18898372	TCCTGTAGCTGCTCTGGAGTCTGGTTTTGAGAAAATACACCATTTGGCTAGCCACTGGTGCAGACTTCATCCCATTTTGTTGCTGCTCTTGAGTGTGGTA	A/G	GTGAGAAGAATTCGCCATTTCGCTAGCACCTGGTAGAGATTCCATCACATGTTGTTGCTGCTGTTGAGGCTCTTGAGTTGGGTTTTGAGAAGAATACACC
Ca_LG_1:18898393	TGGTTTTGAGAAAATACACCATTTGGCTAGCCACTGGTGCAGACTTCATCCCATTTTGTTGCTGCTCTTGAGTGTGGTAGTGAGAAGAATTCGCCATTTC	C/G	GCTAGCACCTGGTAGAGATTCCATCACATGTTGTTGCTGCTGTTGAGGCTCTTGAGTTGGGTTTTGAGAAGAATACACCATTTGGCTAACACCTGGTGCA
Ca_LG_1:18898399	TGAGAAAATACACCATTTGGCTAGCCACTGGTGCAGACTTCATCCCATTTTGTTGCTGCTCTTGAGTGTGGTAGTGAGAAGAATTCGCCATTTCGCTAGC	C/T	ACCTGGTAGAGATTCCATCACATGTTGTTGCTGCTGTTGAGGCTCTTGAGTTGGGTTTTGAGAAGAATACACCATTTGGCTAACACCTGGTGCAAATTTC
Ca_LG_1:18898414	TTTGGCTAGCCACTGGTGCAGACTTCATCCCATTTTGTTGCTGCTCTTGAGTGTGGTAGTGAGAAGAATTCGCCATTTCGCTAGCACCTGGTAGAGATTC	C/T	CATCACATGTTGTTGCTGCTGTTGAGGCTCTTGAGTTGGGTTTTGAGAAGAATACACCATTTGGCTAACACCTGGTGCAAATTTCATCCCATTTTGTTGC
Ca_LG_1:18898554	TTTTGAGAAGAATACACCATTTGGCTAACACCTGGTGCAAATTTCATCCCATTTTGTTGCTGCAGTGGCTGCTCTTGAGTCTGGTAGTGAGAAGAATTCA	A/G	CCATTTGGCTAGCACTTGGTACAGATTCCATCACATGTTGCTGCTCTTGAGTTGGGTGGTGAGAAGAATACGCCATTTGGCTAACACCTGGTGCAAATTT
Ca_LG_1:18996048	GGCAGGCAAGAGACAACCTGGCGAACTGAAACATCTTAGTAGCCAGAGGAAAAGAAAGCAAAAGCGATTCCCGTAGTAGCGGCGAGCGAAATGGGAACAA	A/G	CCTAAACCGTGAAAACGGGAATGTGGGAGAGCAATACAAGCGTCGTGCTGCTAGGCGAAGCAATCGAATGTTGCACCCTAGATGGTGAAAGTCCAGTAGC
Ca_LG_1:18996068	GCGAACTGAAACATCTTAGTAGCCAGAGGAAAAGAAAGCAAAAGCGATTCCCGTAGTAGCGGCGAGCGAAATGGGAACAACCTAAACCGTGAAAACGGGA	A/G	ATGTGGGAGAGCAATACAAGCGTCGTGCTGCTAGGCGAAGCAATCGAATGTTGCACCCTAGATGGTGAAAGTCCAGTAGCCGAAAGCATCACTAGCTTAT
Ca_LG_1:18996069	CGAACTGAAACATCTTAGTAGCCAGAGGAAAAGAAAGCAAAAGCGATTCCCGTAGTAGCGGCGAGCGAAATGGGAACAACCTAAACCGTGAAAACGGGAA	A/T	TGTGGGAGAGCAATACAAGCGTCGTGCTGCTAGGCGAAGCAATCGAATGTTGCACCCTAGATGGTGAAAGTCCAGTAGCCGAAAGCATCACTAGCTTATG
Ca_LG_1:18996157	TGAAAACGGGAATGTGGGAGAGCAATACAAGCGTCGTGCTGCTAGGCGAAGCAATCGAATGTTGCACCCTAGATGGTGAAAGTCCAGTAGCCGAAAGCAT	T/G	CACTAGCTTATGCTCTAACCCGAGTAGCAAGGACCACCTTGCAAGGCTAAATACTCCTGGGTGACCGATAGTGAAGTAGTACTGTTAGGGAAAGGTGAAA
Ca_LG_1:18996779	GAGGCAAACTCTGAATACTAGATATGACCCCAAAATAAGAAGGGTCAAGGTCGGCCAGTGAGACGATGGGGGATAAGCTTCATCGTCGAGAGGGAAACAA	A/G	CCCAGATATCCAGCTAAGGCCCATAAATGATCGCTAAGTGATAAAGGAGGTAGGAGTGCAGAGACAACCAGGAGGTTTGCCTAGAAGCAGCCACCCTTCA
Ca_LG_1:18996787	CTCTGAATACTAGATATGACCCCAAAATAAGAAGGGTCAAGGTCGGCCAGTGAGACGATGGGGGATAAGCTTCATCGTCGAGAGGGAAACAACCCAGATA	A/C	TCCAGCTAAGGCCCATAAATGATCGCTAAGTGATAAAGGAGGTAGGAGTGCAGAGACAACCAGGAGGTTTGCCTAGAAGCAGCCACCCTTCAAAGAGTGC

Positions	-100 bp flanking sequences	SNPs within *desi* cultivar	+100 bp flanking sequences
Ca_LG_1:18996788	TCTGAATACTAGATATGACCCCAAAATAAGAAGGGTCAAGGTCGGCCAGTGAGACGATGGGGGATAAGCTTCATCGTCGAGAGGGAAACAACCCAGATAT	T/A	CCAGCTAAGGCCCATAAATGATCGCTAAGTGATAAAGGAGGTAGGAGTGCAGAGACAACCAGGAGGTTTGCCTAGAAGCAGCCACCCTTCAAAGAGTGCG
Ca_LG_1:18996802	ATGACCCCAAAATAAGAAGGGTCAAGGTCGGCCAGTGAGACGATGGGGGATAAGCTTCATCGTCGAGAGGGAAACAACCCAGATATCCAGCTAAGGCCCA	A/C	TAAATGATCGCTAAGTGATAAAGGAGGTAGGAGTGCAGAGACAACCAGGAGGTTTGCCTAGAAGCAGCCACCCTTCAAAGAGTGCGTAATAGCTCACTGA
Ca_LG_1:18996846	GGGGGATAAGCTTCATCGTCGAGAGGGAAACAACCCAGATATCCAGCTAAGGCCCATAAATGATCGCTAAGTGATAAAGGAGGTAGGAGTGCAGAGACAA	A/G	CCAGGAGGTTTGCCTAGAAGCAGCCACCCTTCAAAGAGTGCGTAATAGCTCACTGATCGAGCGCTCTTGCGCCGAAGATGAACGGGGCTAAGCGATCTGC
Ca_LG_1:19240783	GCCGTAAGAAGGCAGCAGACTTAAGCACCAGAAATAAGAAGGCAGCAGACTAGAAGCCCTAAGAACGCAGCAGACGAAAGTACCAGAAATAAGAAGGCAG	G/A	CAGACTAGAAGCCATAAGAAGGAAGCTGTACAGGCAGCAAGAAACAGTAGAGAAAGCACCAGAAATAGTGGGACCCACTGACCAAACTTGGCCAAAACAG
Ca_LG_1:19712226	TAACCGATTTAAATGAAATTTTTATGTGGTTAAAACTTCAATGCTGCTCATAGAGCCTCTGTCAAATATCAATAGAGTCCTATCTTTAGTTCTCCAAAAC	C/T	GAAAGACTGAATGGAGGGTCTAATGATAATACAGAATCTCAGACTTTTGTAATTGCTGCTAAAACAAAGAAAGGTTCTTGGAAAGGAAAGGAAAATTCCA
Ca_LG_1:19744738	CTGCAACAAGTCCTCTGCATCACTTTTTGGGTCATGTGCAGCCTCCACATCATTAAATTTATCTTAACCATCATGTTCTGGTTTAACCAGCAGCACATCC	C/T	TCTCCCTCACTTTCTGTTGTTTTTCCATTTCTTCTTTTGATCACTATGGTGCAAGCTCCCTCAGCTTCCAAAGTTCTATCATCAGCAAGCCTGGTCTTGC
Ca_LG_1:20380005	CTTTCCTTGTGTGTTTTTTTCAATGACTTTTCTTTTGTTCTTGATATGTCCATTCATCAATCACCTGTTTGAAGTTAGTGAATGCAGCGTCATCTTCAGC	C/T	GGTAAACGGGATGCCAACAGATGATTTCATATGCTCCAACAAATCCTGCTTGGCAGCAAGAAGCTCATGGCACTGCTCTTTCAAGGTTTAGGTCACACCA
Ca_LG_1:20380006	TTTCCTTGTGTGTTTTTTTCAATGACTTTTCTTTTGTTCTTGATATGTCCATTCATCAATCACCTGTTTGAAGTTAGTGAATGCAGCGTCATCTTCAGCG	G/A	GTAAACGGGATGCCAACAGATGATTTCATATGCTCCAACAAATCCTGCTTGGCAGCAAGAAGCTCATGGCACTGCTCTTTCAAGGTTTAGGTCACACCAA
Ca_LG_1:20666328	CAAAGGGGCTATTTGTGGCGGCCTAAGGCAGCCACAAATAAGCTGGTCACAAACTTTTGTGAGGGCTTTTGCAGCCACAAAATGTGGAAAACACGTTGGC	C/T	ATTTGTGAGGGTTTTTGCAGCCACAAAGTGTGGAAAACACGTTGGCATTTGTGAGGGCTTTTGTAGCCACAAAAGGTTGCCTTTGTGAGGGCATAGGCCG
Ca_LG_1:20666340	TTGTGGCGGCCTAAGGCAGCCACAAATAAGCTGGTCACAAACTTTTGTGAGGGCTTTTGCAGCCACAAAATGTGGAAAACACGTTGGCATTTGTGAGGGT	T/C	TTTTGCAGCCACAAAGTGTGGAAAACACGTTGGCATTTGTGAGGGCTTTTGTAGCCACAAAAGGTTGCCTTTGTGAGGGCATAGGCCGCCACAAATGACA
Ca_LG_1:21469407	TTTTTCTTCGTGGTAATTTGTTATGGTGATTTTCCTTTGTGTACCAAAATCGGCCATCTAAAATTGCAGCGAATCAAGCAACGAAATCACGCACCATCTC	C/T	CACTCAACTGAGAATTTTCGATTGAAATAATTGCGGTTTGCGAGAACAGAGATTTAGGCTGCATACCCTTACGTTGATCCAAAAAAACAAATACAGAATC
Ca_LG_1:21705993	CACAAACTGATTATTTTATGTCATGTCATCCTTTGTGGTGGCCTAAACCCTCACAAAAGACAACTTTTGTGGCGGCTTTGGCCCTCACAAACGCTAACTG	G/A	TTTTAAACTTCTTAGTCTTTTGTGGCGGACTTTTCTGTCACAGTTTTGTGAAGGCTTAAGACGTCACAAATGGTAACAAAAAAAAACTGAATTTTGTGGT

Positions	-100 bp flanking sequences	SNPs within *desi* cultivar	+100 bp flanking sequences
Ca_LG_1:21706004	TATTTTATGTCATGTCATCCTTTGTGGTGGCCTAAACCCTCACAAAAGACAACTTTTGTGGCGGCTTTGGCCCTCACAAACGCTAACTGTTTTAAACTTC	C/T	TTAGTCTTTTGTGGCGGACTTTTCTGTCACAGTTTTGTGAAGGCTTAAGACGTCACAAATGGTAACAAAAAAAAACTGAATTTTGTGGTTGTTTTGGCCG
Ca_LG_1:24118226	CCATCACAAATACAAAAATTGAAATTGGTCTTTATTTGTAGCTGAAAAAGCTCTCACAAATTATTAAAAATTTAGGTGGAATTTATGGCTGGCTAAGCCT	T/C	TCACAAAATCACAACGTTGTGATTTTGTAAGGGCTTAAGCAGCCACAAATTCCGCCTAATTTTTTAAGATTTTTGTGAGGGTTTTTGCAGCTACAAAGTC
Ca_LG_1:24118255	CTTTATTTGTAGCTGAAAAAGCTCTCACAAATTATTAAAAATTTAGGTGGAATTTATGGCTGGCTAAGCCTTCACAAAATCACAACGTTGTGATTTTGTA	A/G	AGGGCTTAAGCAGCCACAAATTCCGCCTAATTTTTTAAGATTTTTGTGAGGGTTTTTGCAGCTACAAAGTCTAAAATTTGTGAGGGTCAAGTCCGTCACA
Ca_LG_1:24118280	CACAAATTATTAAAAATTTAGGTGGAATTTATGGCTGGCTAAGCCTTCACAAAATCACAACGTTGTGATTTTGTAAGGGCTTAAGCAGCCACAAATTCCG	G/A	CCTAATTTTTTAAGATTTTTGTGAGGGTTTTTGCAGCTACAAAGTCTAAAATTTGTGAGGGTCAAGTCCGTCACAAAAGCCTGAAATGACATGTCAATTT
Ca_LG_1:24188231	TTTGTTTATGCCACAAGTTCTAATACAAGAAAGCTGCAGGCTGCATATTTTTAGCATCAAATAGAAAGCTGCAGGCTGCATATTTTTTAGCATCAAATAG	G/T	AAAGCTGCAGGCTGCATATTTTTAGCATCAAATATAGGATGAGGGTGACTTGTGTGCAGGCTTAGGAGTTTGGTAATTCCGTTTTGCAGTCTTCTTCATC
Ca_LG_1:24188236	TTATGCCACAAGTTCTAATACAAGAAAGCTGCAGGCTGCATATTTTTAGCATCAAATAGAAAGCTGCAGGCTGCATATTTTTTAGCATCAAATAGAAAGC	C/T	TGCAGGCTGCATATTTTTAGCATCAAATATAGGATGAGGGTGACTTGTGTGCAGGCTTAGGAGTTTGGTAATTCCGTTTTGCAGTCTTCTTCATCAAATA
Ca_LG_1:24464785	CATATATTTGGTCAATATGGGATTTTAACACATCCCTTCAAGTCCAGCACAATGAAGGTGCCGAGGCCAAAACGAAGGCAGCTGCAATGCAGGACGATAC	C/G	GGTTTCCAACAACAGTTTTAAAAAGAAAAAAGTAAACATAGGAAAAATCTCACCGTCAAATTTTCGAAAGCACTTTTGAAGCGCTGCAGAAGAAGTTGCA
Ca_LG_1:24728837	ATTTCCCTCTATAGACTTGGTTAGAACAAATAGAGTTTTACAACTAGTTCACATGGACCTTTTTGGACCTGCCCAAGTTAAGAGTTTAGGTGGAAACCTG	G/T	TATGGATATGTGCTGGTTGATGATTACTCAAGGTTCACATGGACTTATTTTCTGGCTCATAAGAGTGATACATTCTCAATTTTTAAAAAATTTGCTGCTT
Ca_LG_1:24728861	AACAAATAGAGTTTTACAACTAGTTCACATGGACCTTTTTGGACCTGCCCAAGTTAAGAGTTTAGGTGGAAACCTGTATGGATATGTGCTGGTTGATGAT	T/C	TACTCAAGGTTCACATGGACTTATTTTCTGGCTCATAAGAGTGATACATTCTCAATTTTTAAAAAATTTGCTGCTTTAGTTCAAAATGAGAATGATCAGA
Ca_LG_1:24838360	ATTGATCACTTCTGCCATGCTCGTTGGCCTTCGGTTTACATACACGGTAAAACCGGGCGTTTCTTCCTTAGCTTCTTGTTTACCAATTGCCATTTCTCTG	G/A	TGGCTTGATTCAATATGACCTGCAGCTTGAGATTTTGATACTTCAAAACTAGCACTGCCATGCACTTGCTGTGAATCTTTGGCATCGTGCTCGACTTCGG
Ca_LG_1:25536421	AAAAAGATGTTAGTTTCACCTTCGATGACAAATGCAAGAAGGCGTTTGATTTCTTAAAGGCAACACTAACCTCTACTCCCATAATTCAGCCACCCAATTG	G/A	GACCCTCCCTTTTGAAATTATGTGTGATGTATCTAACTATATCGTGGGAGCAGTCCTTGCACATAGGGTTGGTAAGGTCGCCCTAGATTCTGCACATGCT
Ca_LG_1:25536465	TTTGATTTCTTAAAGGCAACACTAACCTCTACTCCCATAATTCAGCCACCCAATTGGACCCTCCCTTTTGAAATTATGTGTGATGTATCTAACTATATCG	G/A	TGGGAGCAGTCCTTGCACATAGGGTTGGTAAGGTCGCCCTAGATTCTGCACATGCTAATTATACTACCACAAAAAAAGGATCTTTTAGCTATTGTTTTTG

Positions	-100 bp flanking sequences	SNPs within *desi* cultivar	+100 bp flanking sequences
Ca_LG_1:26421258	GTGATTTTGTGAGGGCTTAGGCAACCACGAAATCCAGTTAAATTTTTAAGAATTTTGTGAGGGCTTTTACAGCCACAAAAAGTAAATTTTGTGAGGGGCA	A/T	GATCCGTCACAAATGACTGAAATGACATGGCAATTTGTGGCTACATAGGCCGCCACAAAAGCCTATGACCGTTACCTTTTGTGGCTGCAAAAGCCGCCAC
Ca_LG_1:26421301	TTTTAAGAATTTTGTGAGGGCTTTTACAGCCACAAAAAGTAAATTTTGTGAGGGGCAGATCCGTCACAAATGACTGAAATGACATGGCAATTTGTGGCTA	A/G	CATAGGCCGCCACAAAAGCCTATGACCGTTACCTTTTGTGGCTGCAAAAGCCGCCACAAAGGATTAAATATGTAACTGATTTTCTGGCCGCCCAGGCCGT
Ca_LG_1:26421302	TTTAAGAATTTTGTGAGGGCTTTTACAGCCACAAAAAGTAAATTTTGTGAGGGGCAGATCCGTCACAAATGACTGAAATGACATGGCAATTTGTGGCTAC	C/T	ATAGGCCGCCACAAAAGCCTATGACCGTTACCTTTTGTGGCTGCAAAAGCCGCCACAAAGGATTAAATATGTAACTGATTTTCTGGCCGCCCAGGCCGTC
Ca_LG_1:28120207	AGTCAACAATTTCTTAAAAAAAAAAAACAAGAGCAGCAAAAAAAGTCTACCGAAAAAAGTCTTATTATTATTTTTTTAAGATTAAACAAAAGGATTGGCT	T/A	AATTAAAGTGCTAATGCTACAACCAGCCCATAAATAGTTAAAGCTTCCATAAAAGCCAGACTAAGTAATAAAGTACCCTTTATTTTGTCCTCTGCTTCCG
Ca_LG_1:28120211	AACAATTTCTTAAAAAAAAAAAACAAGAGCAGCAAAAAAAGTCTACCGAAAAAAGTCTTATTATTATTTTTTTAAGATTAAACAAAAGGATTGGCTAATT	T/A	AAAGTGCTAATGCTACAACCAGCCCATAAATAGTTAAAGCTTCCATAAAAGCCAGACTAAGTAATAAAGTACCCTTTATTTTGTCCTCTGCTTCCGGTTT
Ca_LG_1:28120216	TTTCTTAAAAAAAAAAAACAAGAGCAGCAAAAAAAGTCTACCGAAAAAAGTCTTATTATTATTTTTTTAAGATTAAACAAAAGGATTGGCTAATTAAAGT	T/C	GCTAATGCTACAACCAGCCCATAAATAGTTAAAGCTTCCATAAAAGCCAGACTAAGTAATAAAGTACCCTTTATTTTGTCCTCTGCTTCCGGTTTTCGCA
Ca_LG_1:28120286	GATTAAACAAAAGGATTGGCTAATTAAAGTGCTAATGCTACAACCAGCCCATAAATAGTTAAAGCTTCCATAAAAGCCAGACTAAGTAATAAAGTACCCT	T/C	TTATTTTGTCCTCTGCTTCCGGTTTTCGCACAATCCCTTCCATATTCGGTAGCCATTTCATATTTAGTAGTCGTTCCATATTCGGTAGCCATGCAATTAG
Ca_LG_1:28120311	AAAGTGCTAATGCTACAACCAGCCCATAAATAGTTAAAGCTTCCATAAAAGCCAGACTAAGTAATAAAGTACCCTTTATTTTGTCCTCTGCTTCCGGTTT	T/G	TCGCACAATCCCTTCCATATTCGGTAGCCATTTCATATTTAGTAGTCGTTCCATATTCGGTAGCCATGCAATTAGTCAATAACTGAGTAGTCCTTCCATA
Ca_LG_1:28120315	TGCTAATGCTACAACCAGCCCATAAATAGTTAAAGCTTCCATAAAAGCCAGACTAAGTAATAAAGTACCCTTTATTTTGTCCTCTGCTTCCGGTTTTCGC	C/T	ACAATCCCTTCCATATTCGGTAGCCATTTCATATTTAGTAGTCGTTCCATATTCGGTAGCCATGCAATTAGTCAATAACTGAGTAGTCCTTCCATATTTG
Ca_LG_1:28120316	GCTAATGCTACAACCAGCCCATAAATAGTTAAAGCTTCCATAAAAGCCAGACTAAGTAATAAAGTACCCTTTATTTTGTCCTCTGCTTCCGGTTTTCGCA	A/G	CAATCCCTTCCATATTCGGTAGCCATTTCATATTTAGTAGTCGTTCCATATTCGGTAGCCATGCAATTAGTCAATAACTGAGTAGTCCTTCCATATTTGT
Ca_LG_1:29061769	GACAGAGTACATAAAAAAAATGCATTAATACTTGACAGAATCAAAATTTGAATCACCCTTTTCATCTATGCTAATTTTCTAATGGTCTAGACAATCAAAA	A/T	TTTAATTTTTGTATAGAGTATTCTTTGAAAAGATATCGATGATCCATATGCGGTTGAAAATAATGTAATATAAACCTGAAACGCGATAGAACAAGTTGAA
Ca_LG_1:29061783	AAAAAATGCATTAATACTTGACAGAATCAAAATTTGAATCACCCTTTTCATCTATGCTAATTTTCTAATGGTCTAGACAATCAAAATTTAATTTTTGTAT	T/A	AGAGTATTCTTTGAAAAGATATCGATGATCCATATGCGGTTGAAAATAATGTAATATAAACCTGAAACGCGATAGAACAAGTTGAAAGATTCCATGGTTC

Positions	-100 bp flanking sequences	SNPs within *desi* cultivar	+100 bp flanking sequences
Ca_LG_1:29442116	CGTCAAAGATAGAATTGATGGTTGCACTTTTTCCAACTCCTGTCTTTCCAAGAACCATTATTGTACAAGAGAAATCAAGTGGTTCTTGACCCACTGATTC	C/G	AAGTTGCTCGGCCATTGCACTAGCACGATCAAAGCTAAAAGCGCCAACACGGCTGCCATTCCTCCCTCTGAGTTGTTCAGCCAGTCCTAATCTATACAGA
Ca_LG_1:29442138	TGCACTTTTTCCAACTCCTGTCTTTCCAAGAACCATTATTGTACAAGAGAAATCAAGTGGTTCTTGACCCACTGATTCAAGTTGCTCGGCCATTGCACTA	A/T	GCACGATCAAAGCTAAAAGCGCCAACACGGCTGCCATTCCTCCCTCTGAGTTGTTCAGCCAGTCCTAATCTATACAGAACTTGTGCTACAACAACATTGT
Ca_LG_1:29442164	CAAGAACCATTATTGTACAAGAGAAATCAAGTGGTTCTTGACCCACTGATTCAAGTTGCTCGGCCATTGCACTAGCACGATCAAAGCTAAAAGCGCCAAC	C/A	ACGGCTGCCATTCCTCCCTCTGAGTTGTTCAGCCAGTCCTAATCTATACAGAACTTGTGCTACAACAACATTGTGGGGTGTCTGCCCAAGCCTATTAGCT
Ca_LG_1:29442376	TTCACCCTAATCATTTGAAGTTTCTCACGAGTCTCGTCATACTCCTCAGCCTCCCCAGATGAGGACTCGTCCAATTTTTGGGACTGCAAATTAGATACAG	G/C	TATTATTGACCCGCGGCTGCTGCACTACCCGTGGAGCAGGTTTCAATAATGGAGCTGCAGGTCCAAGGCCAGCAGGAGGAACAAGAGTGGTGGTAGTGGC
Ca_LG_1:30253632	GTGTAGATGATGTTGTCCTTGTTGGTGGCTCTTCTAGGATTCCCAAAGTGAAGGAGTTGTGCGAGAGCATTAATCCTGATGAGGCTGTGGCTTATGGGGG	G/C	AGCTGTTCAGGCTGCATTGTTGAGTGAAGGCTTTAACAATATTCCAAAGCTGGTGTTGCAGGATGTCACACCTTTGTCTCTTGGTAGGTCAGTATTAGGA
Ca_LG_1:30280699	CCTGATACCAATGTTACTCAATATGATAAGCCGCCTCCCTTGGCACCACCTGAGCCTGCTGCTTCTGCGCCGAGTTTAGCGCCTATACCGGTTGCTCATT	T/C	CGATGACACCTGCTGGAGTGGTGCAACAGCATGGGCAACAGATGATGCAAGTGCATTCCTCACAACAACAGCAGGGGAGTCACTTTGCTCAACAGCATGG
Ca_LG_1:30284273	GGCGATCTTCTTGTTAATCCTGTTCAGGTTAACATTGGAAATGTAGACGAGCTTGCTGCAAATAAATCCATCACACAGGTATTCAATTAATGCCCACTTA	A/G	TTTAAAATCTCAAAGTTGCTGCTAAATGGACAGTTCTTTTTTTTATCTTGCTTCGTCAATGTTTTTGAAATCTCAAAGTTGTCATTGTGGTCAACTTGTA
Ca_LG_1:30310231	ATGTCAGATTTCCTAAACTAAACGTCGTTACTTTCAATAAAGAAGGTCTACAACACATGTTCTTCCAAAGGAAGGCTTTGCTGCAGCACCTACAAGGGTC	C/T	CATATTCCACTCTTGTAAGTCACAGGGATATTTTGAAGGCTGCTATTGATAGTCTGCTCAGAAAAACACATTAAAATCCTTGTGAACAAACTGAGAAGAG
Ca_LG_1:31415384	TGACAGATGTTGCTATGACAAATACAATAGCAGCCTTTGAATGATGTGAATAACTTGCTTATTAGAACTACCTCTTGCTGCTGCAGCATTTCGAGATTCC	C/A	TTGGGATACAATTCATCATAGCGTTTACCAGACTTCTCAGGAGCAATTATATCTGACCATAGTGAAGCACAGACAATTGTTCCAACAGAGACCAACCACA
Ca_LG_1:31415401	ACAAATACAATAGCAGCCTTTGAATGATGTGAATAACTTGCTTATTAGAACTACCTCTTGCTGCTGCAGCATTTCGAGATTCCTTGGGATACAATTCATC	C/T	ATAGCGTTTACCAGACTTCTCAGGAGCAATTATATCTGACCATAGTGAAGCACAGACAATTGTTCCAACAGAGACCAACCACAGCAATGTAACTGGGAAG
Ca_LG_1:31415407	ACAATAGCAGCCTTTGAATGATGTGAATAACTTGCTTATTAGAACTACCTCTTGCTGCTGCAGCATTTCGAGATTCCTTGGGATACAATTCATCATAGCG	G/A	TTTACCAGACTTCTCAGGAGCAATTATATCTGACCATAGTGAAGCACAGACAATTGTTCCAACAGAGACCAACCACAGCAATGTAACTGGGAAGTCTAAA
Ca_LG_1:31415427	ATGTGAATAACTTGCTTATTAGAACTACCTCTTGCTGCTGCAGCATTTCGAGATTCCTTGGGATACAATTCATCATAGCGTTTACCAGACTTCTCAGGAG	G/A	CAATTATATCTGACCATAGTGAAGCACAGACAATTGTTCCAACAGAGACCAACCACAGCAATGTAACTGGGAAGTCTAAAAGTGGTTGAGGTGGTGCATA

Positions	-100 bp flanking sequences	SNPs within *desi* cultivar	+100 bp flanking sequences
Ca_LG_1:31415430	TGAATAACTTGCTTATTAGAACTACCTCTTGCTGCTGCAGCATTTCGAGATTCCTTGGGATACAATTCATCATAGCGTTTACCAGACTTCTCAGGAGCAA	A/G	TTATATCTGACCATAGTGAAGCACAGACAATTGTTCCAACAGAGACCAACCACAGCAATGTAACTGGGAAGTCTAAAAGTGGTTGAGGTGGTGCATATAA
Ca_LG_1:31415439	TGCTTATTAGAACTACCTCTTGCTGCTGCAGCATTTCGAGATTCCTTGGGATACAATTCATCATAGCGTTTACCAGACTTCTCAGGAGCAATTATATCTG	G/T	ACCATAGTGAAGCACAGACAATTGTTCCAACAGAGACCAACCACAGCAATGTAACTGGGAAGTCTAAAAGTGGTTGAGGTGGTGCATATAACTAAACTTC
Ca_LG_1:31926195	ACGCATGGCAGCAACGTGAAGCACTGTGTACCCTCGAGAATTCCGAAGATTGACATTAGCCATACCCAACCCAAGTACTTCGGAAACGACCTTGGGGTCG	G/A	CAGTAGGCTGCTGCGTAATGGAGAGCACCGGCTTCGTCTAAAGTAATGTCCGACTCATTTAAAAGAAGTTTAACAAGCTCGACATCATCCGAGTCCAATG
Ca_LG_1:32434343	TGAATTTCATTCGGTTGGCAATCAAGACCAACAGCTGCACCTGCAGGCTGCAGCTATTATATGATTCTGAATCATTTGCCTGCTCATGAAATGAACTTAC	C/T	TGGTTCCTGAATTGTGACAATACTGCGATATAAACTTTGTTTATAGGGACATGCTTGGATTAGTAGAGCATCAAGAAGTCGAAATTTAGTCTGGAAAGTA
Ca_LG_1:32434370	CCAACAGCTGCACCTGCAGGCTGCAGCTATTATATGATTCTGAATCATTTGCCTGCTCATGAAATGAACTTACTGGTTCCTGAATTGTGACAATACTGCG	G/C	ATATAAACTTTGTTTATAGGGACATGCTTGGATTAGTAGAGCATCAAGAAGTCGAAATTTAGTCTGGAAAGTAAGCGAAAAACAACCAGGTAAGCTGCTT
Ca_LG_1:32452598	CAATACTGGCTTCTTCTGTTTCGTTGCATAAGATGGCAGCATGATCAGGAGAATCTAATGTAGGCAACTCAGGTGTCTCTGTCTTTCTAACTTGCAGTCC	C/T	GGTAGAGCGTGCAAGGTCAATCCATTGCTGCAGCAATTTACAGAAAAACCAAATTTGAAAAATCCTAGTCAAATTTAGAAACTATATGTAGATGAATGAG
Ca_LG_1:32886545	CCACTTTGTTGGTCTCTAGTAAGTAGTAACAAAGAGAGGCAGCAAATTTCAAAACGACAAGTCAGAAATCGCTATCTCAAACCAAACTGCGATTGACCAC	C/T	AGCAGCAATTTCTAGTTTTAGTGACACGATGGAGATGTGACTCGGCGTGCCCGATTTTCATCCCAAAACTACGTGTTTACACGCTGGAACCATTATTATA
Ca_LG_1:33038368	AAGTTTTGGTTGAGACAATTTGCGATGTTCTTGATGAGTTATACACAATTTCTTTAGGGATTCAAACATGTTCCCGTTTGCAAGGAAAGCTGCTGAGTTG	G/C	TTTGCAACAGGACACTGAAGCACATGGAACAAGACGTTGTGCTGTCCTAGTATGTACATTATTCAGCTCCTTGGGAATAATGTTCCTTCTATGCTGCACA
Ca_LG_1:33038427	ATTCAAACATGTTCCCGTTTGCAAGGAAAGCTGCTGAGTTGTTTGCAACAGGACACTGAAGCACATGGAACAAGACGTTGTGCTGTCCTAGTATGTACAT	T/A	TATTCAGCTCCTTGGGAATAATGTTCCTTCTATGCTGCACAATTTTCATTATTAGGTTAGCCATACACTTCCAAGTTTCTGCATTTGGCAAGCTATTGGG
Ca_LG_1:33043165	TCACAGAATTCAAACGCAACGATTTTGCTGCACTACCTGAAATTTTTCAAGAACATATTCAAACTAATAATAATCTAGATGTCAAATATTGCAAAATAAG	G/A	CATTTCTCTTTACCCAAATAACTACTCACCAAAATCACCAACTGATGCATCTTCCTTTGTTTTCAACGCAGCTGCAATCTGATGATGATTACTAGCAGTA
Ca_LG_1:33043281	AATAACTACTCACCAAAATCACCAACTGATGCATCTTCCTTTGTTTTCAACGCAGCTGCAATCTGATGATGATTACTAGCAGTATCAACCACTGAACCAG	G/A	TAAGGTTTCTTGCAAGATGTCTAATTTGATTTAGACGCAGCCTTGGTAAATTATTTAGCTTACAAGCTAGAGTTTCCATGTTAATTGATGATCCTATTGT
Ca_LG_1:33049332	AATCGTCTGTTCTTGATCGAACAATCGACACAATATGACTGCGTGCTGAGTTGATACATAAAATAGTATGCAATTCACTTTGCAGCAGCTATTACATCAA	A/G	AAAATAATCTCAAACTATATACAGTGGAAGCACTCTACAAAAAAAGCTTATTAAGAAAAAATAAAATAATTAGCAGTTAAAGAAAAATATAAAACGCGTG

**Positions**	**-100 bp flanking sequences**	**SNPs within *desi* cultivar**	**+100 bp flanking sequences**
Ca_LG_1:33106822	TTGCTCCTGCTGCATCAAGTTTTGGTTCAGAACAACCAAAATTTTCCTCCATCGCCAACTTTTCCTCGCTGCCATCTCCTCCAGTGTTGTCCACATCATT	T/C	GCCATTGTTCTCAGTTCCCTCATCACTGCTACTACCAGAGTTACGATCCCTGTCTTTATCAATGGCATGGAGTGGTTTGATTTCATCGTCTACATTGTCA
Ca_LG_1:33240913	CAAAAAAAATGAAGATTCTCTTGATAACAAAGAATCAAATGAGAGTTATTGTCAAGACATAGACTTGAAAATCAAGCAGCTATCGTTCGAGAAAGAAAAC	C/T	GCGAAAAAAGAAGAAGAAGAGATGAGAATAAAAGCTCAGGAACTGAAACAAGAAGCTGACAAATCAAGAGTTTTGTCTGAAGAAATAGAAGGGAAACTAG
Ca_LG_1:33240916	AAAAAATGAAGATTCTCTTGATAACAAAGAATCAAATGAGAGTTATTGTCAAGACATAGACTTGAAAATCAAGCAGCTATCGTTCGAGAAAGAAAACGCG	G/A	AAAAAAGAAGAAGAAGAGATGAGAATAAAAGCTCAGGAACTGAAACAAGAAGCTGACAAATCAAGAGTTTTGTCTGAAGAAATAGAAGGGAAACTAGAGC
Ca_LG_1:33256282	GTGGGTAAGAAAATTAACAGTGGTGTAACTTGTATACATTCTTTCAGGATATGATTATGGGGAGAAAAAGCGAATTCCTGACTGGTGTGATAGAATAATG	G/A	TATCGTGATACTCGGGCAGCTGCCGTGTCTGACTGCAATTTAGACTGTCCTGCTGTGTCTTCAATTTTACAGTATGTTTCATGGTCAAAATTGTTTTCTA
Ca_LG_1:33289544	GGCGGTGTTTGTTGATTTTGGAGCGCACTAGAAAAGAGGCAGCACAGCTGTAACAGAGTAAAAAAAAAACAAAACAGTGACAGCTGGTGTAAAGCACAGC	C/T	TGTGTTTGAAAGAAAAAAAAATCACTGGAAAAACACACAGTGAAGGCGGTGCTTTTGAGGCTAGCTGTAGTGTCGTGGCTGATTGCTAGAAAGGCAGCCG
Ca_LG_1:33289576	AAAGAGGCAGCACAGCTGTAACAGAGTAAAAAAAAAACAAAACAGTGACAGCTGGTGTAAAGCACAGCTGTGTTTGAAAGAAAAAAAAATCACTGGAAAA	A/C	ACACACAGTGAAGGCGGTGCTTTTGAGGCTAGCTGTAGTGTCGTGGCTGATTGCTAGAAAGGCAGCCGGAGTTGTTGGTGACAGATGGCTGTATGAAGCC
Ca_LG_1:33417688	GGACTTGTCCCGCGAACACACGAACAATCTTGCTTGTAGAATTCCCTTTTCCTCTTCAAATTCTTACTTATGTAGGTTTCCCGGTAATAGGAATATCTGT	T/C	GAAAACTCTTACCCATATCTTACCATTTTTTTGGAATTGTTCGCTCATAGAACGATGGAAGTGTCCAATTATAGCCCAACATGTTGCTTCAATGGCTCGA
Ca_LG_1:33417720	CTTGTAGAATTCCCTTTTCCTCTTCAAATTCTTACTTATGTAGGTTTCCCGGTAATAGGAATATCTGTGAAAACTCTTACCCATATCTTACCATTTTTTT	T/C	GGAATTGTTCGCTCATAGAACGATGGAAGTGTCCAATTATAGCCCAACATGTTGCTTCAATGGCTCGATATGAAAGCGATCAGCTCTACAACTTTTAGTT
Ca_LG_1:33417729	TTCCCTTTTCCTCTTCAAATTCTTACTTATGTAGGTTTCCCGGTAATAGGAATATCTGTGAAAACTCTTACCCATATCTTACCATTTTTTTGGAATTGTT	T/C	CGCTCATAGAACGATGGAAGTGTCCAATTATAGCCCAACATGTTGCTTCAATGGCTCGATATGAAAGCGATCAGCTCTACAACTTTTAGTTTCATATCTT
Ca_LG_1:33417739	CTCTTCAAATTCTTACTTATGTAGGTTTCCCGGTAATAGGAATATCTGTGAAAACTCTTACCCATATCTTACCATTTTTTTGGAATTGTTCGCTCATAGA	A/C	ACGATGGAAGTGTCCAATTATAGCCCAACATGTTGCTTCAATGGCTCGATATGAAAGCGATCAGCTCTACAACTTTTAGTTTCATATCTTCCAAAACCTA
Ca_LG_1:33417755	TTATGTAGGTTTCCCGGTAATAGGAATATCTGTGAAAACTCTTACCCATATCTTACCATTTTTTTGGAATTGTTCGCTCATAGAACGATGGAAGTGTCCA	A/G	ATTATAGCCCAACATGTTGCTTCAATGGCTCGATATGAAAGCGATCAGCTCTACAACTTTTAGTTTCATATCTTCCAAAACCTAGTTTTGTTATGTCTAG
Ca_LG_1:33545581	CGCTATTGTGTAGCGGAATTCGAACAAATTACTATTGTTCTGCTGCACGTATTTAGTAGTAAGTGTTATCAAATTGCGGCGCGGTAGCACTGTAGCATAT	T/G	CAGAATTTGAACAATCCACTATTTTCCATGATTTGCGATTTGCAGCACTGTTGTCTGCCTTAACGTTTCCATCCCTGTAAATAAGTTGCTGGATTGATTG

Positions	-100 bp flanking sequences	SNPs within *desi* cultivar	+100 bp flanking sequences
Ca_LG_1:33749072	ATTTGGTAAGTATGTGTAATGTAATATATATTTAGCAGCAGCAAGGAAGAATAATATGGCACTATTAGGTTCCACAGAATCCTCCTCAGCTGTTGTTCCG	G/A	AGCTGCGGTACGGAAGATCTTGCTCGAACCCAAACTCAACGGTACATTTATTTTCTTTTTTTTATTTTTTATTTTTACCTCTCTCTTTTATGGTGCTTAT
Ca_LG_1:33805261	GGAACCCCAACCTTGTGATTCTGAATCCGAACTTTCTTCCCTACCATTTTTACCATTGTTATTTCTAACTATGTGATCTCTTGTTGTTCCAGCTGCTTCG	G/A	ATCTGAGGTGTGTTTGATCTAGTTCTTTCATCAGATGAAGAATTCGATACTTGATCATCTACTTCTGCTGCTGTACCACTTGGACCTATCTCAAGAAATT
Ca_LG_1:33948838	ACCTACGGCCCCATTCTCGTCACTTCTGCAGCTCAAATGATGTCTATAACCAATACACATGGGAACATTCAGATTCAAAACCCTAGCTTTCGCTCCGCCG	G/T	CCGGGAACCACCTTGCTCCGGCGACTCGCGGCGGTTTCTCTCTTTAACGCTTTCTCGATGTTTGTCGTTTGATGCTGCTCAGAGTTTTTGGAACCGCCGC
Ca_LG_1:34019029	ATATTTATTTAAGATAAACAAATGTGTAACACATTATCAATTACTTATTCCACGGCCACGAATAGGCATCCCTATTTGTTACCCTAATTGGTCCTTTTTC	C/G	GCTGATAACCCAATTACATTCAGAACATGGATCTCTAAGACCTACATATATGTCAAACCAGTGAAATTCTCCTTCCCACTTAAAGGAACAATAGAATAGT
Ca_LG_1:34019037	TTAAGATAAACAAATGTGTAACACATTATCAATTACTTATTCCACGGCCACGAATAGGCATCCCTATTTGTTACCCTAATTGGTCCTTTTTCGCTGATAA	A/T	CCCAATTACATTCAGAACATGGATCTCTAAGACCTACATATATGTCAAACCAGTGAAATTCTCCTTCCCACTTAAAGGAACAATAGAATAGTGTTGTGCC
Ca_LG_1:34464880	GCGATTTCTTCATTTGGCTGGCCAAACATTGGAGGGCCTGGCTGCAGGTTGTCCATGGCCTTGCATGCCAATTGTAGCTTCCTTGTGGACCCAAAAGGCG	G/A	AAACGTTGGAGTGATTTCCTTATTTTCTCAGCATCTAGAACTGTCTTCCTTCACAACAGCGATGCAGTAGTTCAACTTGTTAAAAGCTGCTTCACAGCTA
Ca_LG_1:34634667	GTCCTTTGATGGAGATTCAAGTGGAGTGCTATCCTTAGAGTCCGCATTTTGGGTGGATATATAAAATAGCAACTCACCCAAAGCAGCCATAGAAAACCTC	C/T	CTCACTTTTTCATGTCTGTCTCTAAGGCCATCAGTCAGTGAACCTAGAATTCCAGAGTTAGCCAAACTGTCGTCAACAAAAGTAGAGTGTCTAATCAACA
Ca_LG_1:34939157	TCTGAAGTGAATCTCCAGATACCAAAACCGGTTGCTCTATGTTGGTTTTGGCTCCTCTTGTGTTCATCTGATGTCCAGGGTCATTTTGATTTGTTCTACC	C/A	ATCTGCTGCCTGAAGTATACGTGGGAAGGTAGTGGAAAAATGCTATTATGATTTATTCAGCAGTAAATAAAGCGGTTTAAATAGAGAGATTACAATAAAG
Ca_LG_1:35091220	TAACATCTTATTCAATGTTTAATTAGTAATTTGAAGAAACACCTGTTTAATTTAAATTATTCATGAGTGTAGATTGAGAGCAGCTGGTAGATCAAATGCC	C/T	GACGGTGTCAAGAAATCTCGTCCGCGTGAGCGAGTAGGGCGTGGAATTCCAGTTCCAGATGCCAATGCTGAACTTCAAGCAAATATTGATGTATGTCTGA
Ca_LG_1:35156572	AAATCCTCCTATTGATCCTTCTTCCACAGTGATTAAAACCTCATGTGATTTGGCTAGGCTGCGGATGAGGGAACGATCCAATGGCTTGCAGAAACGTGCG	G/A	TCGGCCACTGTTACATGTAAGCCGTGATGCTCCAACAAGGAAGCTGCAGCTAGACAGTTCTGAACAGCTGATCCATAGCCCAAAAGGGCCACTCTTTCCC
Ca_LG_1:35157909	AGCCAGACCTGCAGCAAATGTAACAGCATGCTGTTCTGCTATCCCCACGTCAAAGCATCTTGCAGGGAAACGGCGGAGGAAGAGATTCATGCCAGTTCCG	G/A	CCTCCCATTGCAGCATGGATTCCAACAATGTTTTTGTCAGCTTCTGCTTCTGCAATCAAAGCCTCAGCAAAGTATGTTGTGTATGACTGGGTGGGAGCCT
Ca_LG_1:35310394	GTCCATGGCAGCGATGGCTAAAATCCGCTATTTTTTTGTCTACATGTCCGCCATAGCCAACATCATGTGTGAAATGGCCATTGATGAAATCCCGCCACCA	A/G	ATATCCGCCGTGGCTTCCATTTGATAACACTGGCTGCAATTGTGTGTAGGGATGAGAATAGGGTGCACCGAGCTAGACTATTGCATAAATTGGTCTATTA

**Positions**	**-100 bp flanking sequences**	**SNPs within *desi* cultivar**	**+100 bp flanking sequences**
Ca_LG_1:35325367	CTCTAAGTTTGCGTTGCTGCCTCCCTCCCTTACCTCTCTATCGCTAACCTATATGTCAAGTTTGCACACGTTGGACTGCACGGAGCTTCTCCACCTCACG	G/T	TCCCTCCAACACTTAGCAATTTTTACTTGTTACAAGCTGGAGAATATGGCAGGTGAAAGGCTGCCTGCTACTCTAACACAACTTCAAATCATTGAGTGTC
Ca_LG_1:35327575	TTAGCTCTATCTTTCTGTAACATCGCTTTAATCTCATGTAAATCTATATAATAATGTAGCAATTATTATGGTTCAGGCAAATTGCTATGGTTATGCTGCT	T/A	TCACCTTAGAAGTCCCAAGTGCTGTGCTTTCAAAGATTCATCCCACAATGCCCACAACATCACTTCACCTTCAAAAAACCTTGTCCAAAACATGTCGTCT
Ca_LG_1:35327606	TCTCATGTAAATCTATATAATAATGTAGCAATTATTATGGTTCAGGCAAATTGCTATGGTTATGCTGCTTCACCTTAGAAGTCCCAAGTGCTGTGCTTTC	C/G	AAAGATTCATCCCACAATGCCCACAACATCACTTCACCTTCAAAAAACCTTGTCCAAAACATGTCGTCTGGAAGCATTGCAGTGTCAAAATGATGTGCAG
Ca_LG_1:35327638	TATTATGGTTCAGGCAAATTGCTATGGTTATGCTGCTTCACCTTAGAAGTCCCAAGTGCTGTGCTTTCAAAGATTCATCCCACAATGCCCACAACATCAC	C/T	TTCACCTTCAAAAAACCTTGTCCAAAACATGTCGTCTGGAAGCATTGCAGTGTCAAAATGATGTGCAGTATTTCTACATTAGTTGTTTGCTGCCTATGTT
Ca_LG_1:35327670	CTGCTTCACCTTAGAAGTCCCAAGTGCTGTGCTTTCAAAGATTCATCCCACAATGCCCACAACATCACTTCACCTTCAAAAAACCTTGTCCAAAACATGT	T/A	CGTCTGGAAGCATTGCAGTGTCAAAATGATGTGCAGTATTTCTACATTAGTTGTTTGCTGCCTATGTTATGAATTCTGTTTTGGGGTGATAGGCTCTTAT
Ca_LG_1:35327671	TGCTTCACCTTAGAAGTCCCAAGTGCTGTGCTTTCAAAGATTCATCCCACAATGCCCACAACATCACTTCACCTTCAAAAAACCTTGTCCAAAACATGTC	C/G	GTCTGGAAGCATTGCAGTGTCAAAATGATGTGCAGTATTTCTACATTAGTTGTTTGCTGCCTATGTTATGAATTCTGTTTTGGGGTGATAGGCTCTTATG
Ca_LG_1:35327680	TTAGAAGTCCCAAGTGCTGTGCTTTCAAAGATTCATCCCACAATGCCCACAACATCACTTCACCTTCAAAAAACCTTGTCCAAAACATGTCGTCTGGAAG	G/A	CATTGCAGTGTCAAAATGATGTGCAGTATTTCTACATTAGTTGTTTGCTGCCTATGTTATGAATTCTGTTTTGGGGTGATAGGCTCTTATGCATGAGAAT
Ca_LG_1:35327688	CCCAAGTGCTGTGCTTTCAAAGATTCATCCCACAATGCCCACAACATCACTTCACCTTCAAAAAACCTTGTCCAAAACATGTCGTCTGGAAGCATTGCAG	G/A	TGTCAAAATGATGTGCAGTATTTCTACATTAGTTGTTTGCTGCCTATGTTATGAATTCTGTTTTGGGGTGATAGGCTCTTATGCATGAGAATACTGAAGT
Ca_LG_1:35389631	ACAAGCTTTGAATGGCTTAGAGCCAACAAGGACTATTGCCAAGAAGCACTACAGAGGTGTGAGGCGCCGTCCTTGGGGGAAATACGCAGCCGAGATTCGT	T/C	GACTCGGCTCGACATGGAACAAGAATATGGCTTGGAACATTCCAAACAGCTGAAGAAGCTGCTTTGGCTTATGATAGAGCTGCTTTTAAAATGAGAGGTT
Ca_LG_1:35590217	ATACCAAATGTTCTAAGGTTTTAGATTATATGTGAATCAAAATTTCTTAGTGATCTTGAAGCATTTTGATAATTGCTGCTAACGCTGTCCAAATTCTCCC	C/T	GACAAATTTTAATTCCGAGGATACAAGTAAAAAATTGCTTATACAAAATTCACCAACATAGCAGCATAATTGACTTATATTTATCAAAACAAACAGGTTA
Ca_LG_1:35590238	TAGATTATATGTGAATCAAAATTTCTTAGTGATCTTGAAGCATTTTGATAATTGCTGCTAACGCTGTCCAAATTCTCCCGACAAATTTTAATTCCGAGGA	A/G	TACAAGTAAAAAATTGCTTATACAAAATTCACCAACATAGCAGCATAATTGACTTATATTTATCAAAACAAACAGGTTATCCCATATTTTTTTATTGGTA
Ca_LG_1:35855878	ATCTGTATGGTCCAAGCTTCGATTTCGAGATTGGAACAATCATTGAAAGTGGCTCAGCAGCAGCAGCCTCTGAAGAAGGCCTGCAAAACATAGCATGCAG	G/A	CAATGTACATCAGAATACATATGTGAATATGTGATACATTTTAAACCAATGGAGCAAGGCCAACAAAAATAATAAACACATGAAAAATATTCATTATCAA

**Positions**	**-100 bp flanking sequences**	**SNPs within *desi* cultivar**	**+100 bp flanking sequences**
Ca_LG_1:36808803	ATTGGTTTTTGCAGCCTTAATGCTTTTTGCTGGTTGGTTTCATTATCCCAAAGCTGCTCCAAAATTGGCTTCGTTCCAAGATGTAGAATCCATGTTGAAC	C/T	CACCATTTGGCAGGGCTACTAGGACTTGGGTCTCTTTCTTGGGCGGGGAATCAAGTACATGTATCTTTACCAATTAACCAATTTCTAAATGTTGGAGTAG
Ca_LG_1:36917177	ATCTCCAACAAGGCAATTTCCTTCAATCGTTCATTAGAGACAGATAAACCAGACACATCCGAGTTAGTGGCAACAATCTGTCGTTGATCCTGCTCCTCGC	C/T	GGATAACACCTGCAGACTGCAACATAACCAGTTAACCTCCAGATTAAAATCATAACAGATCAGCAGCAGCAGCAGGGTTACTTACATTTTCAGCAGTCAC
Ca_LG_1:36917207	TCATTAGAGACAGATAAACCAGACACATCCGAGTTAGTGGCAACAATCTGTCGTTGATCCTGCTCCTCGCGGATAACACCTGCAGACTGCAACATAACCA	A/C	GTTAACCTCCAGATTAAAATCATAACAGATCAGCAGCAGCAGCAGGGTTACTTACATTTTCAGCAGTCACTCTCTGTTCCTGCAGTACATTTTCACTCCG
Ca_LG_1:36917221	TAAACCAGACACATCCGAGTTAGTGGCAACAATCTGTCGTTGATCCTGCTCCTCGCGGATAACACCTGCAGACTGCAACATAACCAGTTAACCTCCAGAT	T/A	TAAAATCATAACAGATCAGCAGCAGCAGCAGGGTTACTTACATTTTCAGCAGTCACTCTCTGTTCCTGCAGTACATTTTCACTCCGATGACTGATTTGTA
Ca_LG_1:37591211	AATCACAGGGGTTGTGCCTCAAAAAAGAATTGGTCTTTCCCTTTAAGGATGTAGCTGCTATGTATTCCCGCTGCACTCCCATGACAACATTGCTGGTAAG	G/A	GGTCTGCAAATTGTGACATGATGGTTAGTGTGGGCTGTGCGGTGGTTGTGTGGGTGTTGGTGGATCAATAATTGATCGAGCCCAGTTACTCATGTGAGAC
Ca_LG_1:37591244	TCTTTCCCTTTAAGGATGTAGCTGCTATGTATTCCCGCTGCACTCCCATGACAACATTGCTGGTAAGGGTCTGCAAATTGTGACATGATGGTTAGTGTGG	G/T	GCTGTGCGGTGGTTGTGTGGGTGTTGGTGGATCAATAATTGATCGAGCCCAGTTACTCATGTGAGACATACTCATTGTTGTGCTGAACCTTTTTGCTCTG
Ca_LG_1:37591259	ATGTAGCTGCTATGTATTCCCGCTGCACTCCCATGACAACATTGCTGGTAAGGGTCTGCAAATTGTGACATGATGGTTAGTGTGGGCTGTGCGGTGGTTG	G/C	TGTGGGTGTTGGTGGATCAATAATTGATCGAGCCCAGTTACTCATGTGAGACATACTCATTGTTGTGCTGAACCTTTTTGCTCTGCCAGTAACGTCACAC
Ca_LG_1:37735245	TGATGGACATGCTCACCGATCTTAGAATTGGAAAAGTATGTGATGGTGTGGAGTCTTTCCCCAATAAGGATTTTGCGTTGCTGCCTCCCTCCCTTACCTC	C/A	TCTATCGCTAAGCAATATGCCAAGTTTGCACACGTTGGACTGCACGGAGCTTCTCCACCTCACGTCCCTCCAACAATTAGAAATTGACAGTTGTTCAAAT
Ca_LG_1:38253475	GTGAGGGCTTTTGCAGCCACAAAAGACAGCAGTTTGTGAGGGCTTTTGCAGCCACAAACGTCTGCCTTTGTGAGGGCTTAGGCCGCCACAAAGGAGAGAC	C/G	TGATTCTGAAATCAACGTTTGTGAGGGCCCAGGCCGCCACAAACTCTCTCCTTTGTGACGGCTTAGGCCGCCACAAATGACATCCCGTTTAAAGTATTTT
Ca_LG_1:38256174	CGTGCGCGTTCCTTTGTGGCGGCCTTTGCAGCCACAAATGCCACGTGCGCGTTCCTTTGTGGCGGCCTTTGCAGCCACAAATGTCCACTTGTCCCGTCCG	G/T	CCTTTGTGACGGATTTGGCCCTCACAAAATTGATTATTTGTGGCTGTAAAAGCCCTCACAAAATCCTTAAATATTTAACTGGATTTTGTGGCTGCCCAAG
Ca_LG_1:38409310	CGTTGATAGCGGGGGATAAGTTAAGGTCAAGCGGATAAGATTCATCGGCGGCGGCGTCACGGTCGCGGCTGGAGGATTCAACGGTGCTGCTTTGGCTAGT	T/G	ACTTTGGATCTTTATATTTTCGGAAGGGTAAGGGAAGTTAGTTTTAGCCTTAGAACCACGGAAATCACGTGCGGCGGTGTCGTAGGCTCGTGCAGCCTCT
Ca_LG_1:38494973	TTCGGGAGAAGAGTATTTGCGTGACAAGGCAATGACTTTATATTAACACATTTGTAGATATCCATTGATCTCAAGTTGGGCGCAGACAATCCATCTCTTC	C/G	CAAATGAAACAAATTTGGGGCAGTCGTAAATGTCAAAACTGTTGAGATTTTGAAGAATCTTCGAAGCGGATATACATTCTAGATTTTCACAGTGCCAGAT

Positions	-100 bp flanking sequences	SNPs within *desi* cultivar	+100 bp flanking sequences
Ca_LG_1:38525016	GCACAAGAACATTGTGAAACTATGGTGTTGTTGTACCACTAGGGATTGCAAGCTATTGGTTTATGAGTATATGCCAAATGGTAGTCTTGGTGATTTGCTG	G/T	CATAGCAATAAAGGAGGGTTGTTGGATTGGCCAACAAGGTATAAGATAGCTCTTGATGCTGCAGAAGGGCTCTCTTATCTGCATCATGACTGTGTCCCTC
Ca_LG_1:38525022	GAACATTGTGAAACTATGGTGTTGTTGTACCACTAGGGATTGCAAGCTATTGGTTTATGAGTATATGCCAAATGGTAGTCTTGGTGATTTGCTGCATAGC	C/T	AATAAAGGAGGGTTGTTGGATTGGCCAACAAGGTATAAGATAGCTCTTGATGCTGCAGAAGGGCTCTCTTATCTGCATCATGACTGTGTCCCTCCTATTG
Ca_LG_1:38525024	ACATTGTGAAACTATGGTGTTGTTGTACCACTAGGGATTGCAAGCTATTGGTTTATGAGTATATGCCAAATGGTAGTCTTGGTGATTTGCTGCATAGCAA	A/C	TAAAGGAGGGTTGTTGGATTGGCCAACAAGGTATAAGATAGCTCTTGATGCTGCAGAAGGGCTCTCTTATCTGCATCATGACTGTGTCCCTCCTATTGTT
Ca_LG_1:38525055	TAGGGATTGCAAGCTATTGGTTTATGAGTATATGCCAAATGGTAGTCTTGGTGATTTGCTGCATAGCAATAAAGGAGGGTTGTTGGATTGGCCAACAAGG	G/A	TATAAGATAGCTCTTGATGCTGCAGAAGGGCTCTCTTATCTGCATCATGACTGTGTCCCTCCTATTGTTCATAGAGATGTGAAATCTAATAACATCTTGT
Ca_LG_1:38525058	GGATTGCAAGCTATTGGTTTATGAGTATATGCCAAATGGTAGTCTTGGTGATTTGCTGCATAGCAATAAAGGAGGGTTGTTGGATTGGCCAACAAGGTAT	T/C	AAGATAGCTCTTGATGCTGCAGAAGGGCTCTCTTATCTGCATCATGACTGTGTCCCTCCTATTGTTCATAGAGATGTGAAATCTAATAACATCTTGTTGG
Ca_LG_1:38525068	CTATTGGTTTATGAGTATATGCCAAATGGTAGTCTTGGTGATTTGCTGCATAGCAATAAAGGAGGGTTGTTGGATTGGCCAACAAGGTATAAGATAGCTC	C/G	TTGATGCTGCAGAAGGGCTCTCTTATCTGCATCATGACTGTGTCCCTCCTATTGTTCATAGAGATGTGAAATCTAATAACATCTTGTTGGACGAGGACTT
Ca_LG_1:38564469	TCTCTCCTTCAACCACAAGAGCCAAACCCACCTGTATTATTCGGAAATCTCTTGGAGAATATTAGTGTGTAGTGTCAGAATTCAATCTAATATAATTGTA	A/T	ATGTCACACAAGTTAGCTCAGCTGGCAGCTGCTAATACAGTTGCAATGGACAATGATAGTTAGTATCTAAAAGCTTCTAATAACAGCTGCGTCAGTAGTA
Ca_LG_1:38564525	GAATATTAGTGTGTAGTGTCAGAATTCAATCTAATATAATTGTAATGTCACACAAGTTAGCTCAGCTGGCAGCTGCTAATACAGTTGCAATGGACAATGA	A/G	TAGTTAGTATCTAAAAGCTTCTAATAACAGCTGCGTCAGTAGTAGGAGAGTTGGTTAGAATAACAGTTGTAGAATGTTTAGCATGTGTTTGTATAAAAAA
Ca_LG_1:38879515	GGTGCAGAAATTGCTGGCCTGGGTGTAGAGTTTGCTAGCCTAGGTGCAACAGTTGTTGGACTAGGTGCAGAAGTTGCTGGATTGGGTGCAGTGGTTGCTA	A/G	GACTGGGTTCTGCACCTGGGGCAGGAAAAAACATGACAGAGCTGGACTCTAAATTAGGAGTGGAAGATGTTGGAATTGTTGAATTAAAAATGTAGCATGT
Ca_LG_1:38879532	CCTGGGTGTAGAGTTTGCTAGCCTAGGTGCAACAGTTGTTGGACTAGGTGCAGAAGTTGCTGGATTGGGTGCAGTGGTTGCTAGACTGGGTTCTGCACCT	T/A	GGGGCAGGAAAAAACATGACAGAGCTGGACTCTAAATTAGGAGTGGAAGATGTTGGAATTGTTGAATTAAAAATGTAGCATGTGCCAGCAGTGTGATGAG
Ca_LG_1:38879544	GTTTGCTAGCCTAGGTGCAACAGTTGTTGGACTAGGTGCAGAAGTTGCTGGATTGGGTGCAGTGGTTGCTAGACTGGGTTCTGCACCTGGGGCAGGAAAA	A/T	AACATGACAGAGCTGGACTCTAAATTAGGAGTGGAAGATGTTGGAATTGTTGAATTAAAAATGTAGCATGTGCCAGCAGTGTGATGAGGAATAATGCTAA
Ca_LG_1:38879546	TTGCTAGCCTAGGTGCAACAGTTGTTGGACTAGGTGCAGAAGTTGCTGGATTGGGTGCAGTGGTTGCTAGACTGGGTTCTGCACCTGGGGCAGGAAAAAA	A/G	CATGACAGAGCTGGACTCTAAATTAGGAGTGGAAGATGTTGGAATTGTTGAATTAAAAATGTAGCATGTGCCAGCAGTGTGATGAGGAATAATGCTAATG

Positions	-100 bp flanking sequences	SNPs within *desi* cultivar	+100 bp flanking sequences
Ca_LG_1:38925020	ATATTCTCCAGCTTGTAACAATTAAAAATTTCTAATTTTTGGAGGGACGTGAGGTGGAAAAGCTCCGTGCAGTCCAACGTGTGCAAGCTTGACATATTGC	C/T	GTAGCGTTAGAGAGGTAAGGGAGGGAGGCAGCAACGCAAAACCCTTATTGGGGAAGGACTCCACACCATCACAACTACCAATTGTAAGATGGGTGAGCAT
Ca_LG_1:39657941	TGTCTCAGTAGTCTTTCATTCTTAAACTCTAAGTTCACAACACTACATTGATCATCATCAACTCCAAACACTACACTGATGCGTCTACTGCATCTTGATT	T/G	CTCCACAATGACGGAATCACAATCACCGTTAGTTTATTATCTTCCATTTAATCATCTTTCTAATTTACCAAATTCATTTTCGGATCTCTAATTTCTTTGC
Ca_LG_1:39657948	GTAGTCTTTCATTCTTAAACTCTAAGTTCACAACACTACATTGATCATCATCAACTCCAAACACTACACTGATGCGTCTACTGCATCTTGATTCTCCACA	A/G	ATGACGGAATCACAATCACCGTTAGTTTATTATCTTCCATTTAATCATCTTTCTAATTTACCAAATTCATTTTCGGATCTCTAATTTCTTTGCTTTTTTT
Ca_LG_1:39657960	TCTTAAACTCTAAGTTCACAACACTACATTGATCATCATCAACTCCAAACACTACACTGATGCGTCTACTGCATCTTGATTCTCCACAATGACGGAATCA	A/G	CAATCACCGTTAGTTTATTATCTTCCATTTAATCATCTTTCTAATTTACCAAATTCATTTTCGGATCTCTAATTTCTTTGCTTTTTTTTTTGTAGGCGGA
Ca_LG_1:39658091	ATCATCTTTCTAATTTACCAAATTCATTTTCGGATCTCTAATTTCTTTGCTTTTTTTTTTGTAGGCGGAGAAGATTAACACTATCTTGAAGGCTGCTGGG	G/C	GTCACTGTCGAATCATACTGGCCAAGCTTATTCGCCAAACTTGCTCAGAATAAGAGCATTGACGATCTCGTTTTGAACGATGGCGCTGCCGGTGGTGCCG
Ca_LG_1:39658193	CACTGTCGAATCATACTGGCCAAGCTTATTCGCCAAACTTGCTCAGAATAAGAGCATTGACGATCTCGTTTTGAACGATGGCGCTGCCGGTGGTGCCGCT	T/C	GTTGCCGTATCTGCTCCTGCCGCTGCTGCTGGTGGTGGAGCCGCTGCAGCTGCCGCACCTGCTGTCGAGGCCAAGAAGGTACTGTTAGTAGGACCATTTA
Ca_LG_1:39658203	TCATACTGGCCAAGCTTATTCGCCAAACTTGCTCAGAATAAGAGCATTGACGATCTCGTTTTGAACGATGGCGCTGCCGGTGGTGCCGCTGTTGCCGTAT	T/G	CTGCTCCTGCCGCTGCTGCTGGTGGTGGAGCCGCTGCAGCTGCCGCACCTGCTGTCGAGGCCAAGAAGGTACTGTTAGTAGGACCATTTAGACTTAAATA
Ca_LG_1:39658218	TTATTCGCCAAACTTGCTCAGAATAAGAGCATTGACGATCTCGTTTTGAACGATGGCGCTGCCGGTGGTGCCGCTGTTGCCGTATCTGCTCCTGCCGCTG	G/T	CTGCTGGTGGTGGAGCCGCTGCAGCTGCCGCACCTGCTGTCGAGGCCAAGAAGGTACTGTTAGTAGGACCATTTAGACTTAAATATCATATCTTGCATCA
Ca_LG_1:39690391	TACTACGCAACAGAATAATATTTATAAGCTACATACATGCTTTATTATATCTTCATGCAGAGTTTTGGCTTTCTGCTCAAGCTCCAGCTGCGAAACCCAG	G/A	GTGGTTATCATTTGGTCACATTTTTCTACAAATCCAATCAAATATTGTTTAACAAATAAAATTCTTACTCATAAGGAACACCCATAAAATCAGCGTCGTC
Ca_LG_2:541984	GGAATATTCTTGCTGCTATTTTACTATTAATATTTTCATAATCAGTTTCGTTTTTATTTCCCATATACTTGCTCTGGAAACCATGGAAAGAAAGTCATTA	A/T	AAGTTAATATATGTAGAGCTGCCGAATTAGAATGCACAAAAGAGACAAAGAAATGAAAAAAATTGATGGAAAAATCGAACAAGTTGTGTAAAAACAACCA
Ca_LG_2:541995	GCTGCTATTTTACTATTAATATTTTCATAATCAGTTTCGTTTTTATTTCCCATATACTTGCTCTGGAAACCATGGAAAGAAAGTCATTAAAGTTAATATA	A/G	TGTAGAGCTGCCGAATTAGAATGCACAAAAGAGACAAAGAAATGAAAAAAATTGATGGAAAAATCGAACAAGTTGTGTAAAAACAACCAATTTAGCAAAA
Ca_LG_2:547943	TGGCTATTTTGATGAGGTGTAATTTCAGTAATAGTCAAAGCTCAATTAAATCAGTGGAGGTGAATCATGTTGCTTCCTTTCACCTCAATTTGAAAGTTAG	G/C	AATAAATATTAGGCAGCAATTTTACTGCTAAAAAAATTTACCTTAACTCCGCAATTATACCTTTACCTCACGGAACTTTCAATTTTGTCATTCCTAGTAA

Positions	-100 bp flanking sequences	SNPs within *desi* cultivar	+100 bp flanking sequences
Ca_LG_2:674698	AGATTGAACTAGCTGAAGACTTTAACACATTACATTCATATCTTGAACTTTCCTTTGATAATCTCTTACTGCATTAGCAGCAGCACCACCTGCAAGGACG	G/T	GCCTCTTCTAGCTCCCTTACAGTTTGGGTGAGCTTTTCAACCTCTGCCACCTTTTGTCTATGCATTTTGTCCAAAATTTTATTTTCCTCCTGAGAGAAAT
Ca_LG_2:674700	ATTGAACTAGCTGAAGACTTTAACACATTACATTCATATCTTGAACTTTCCTTTGATAATCTCTTACTGCATTAGCAGCAGCACCACCTGCAAGGACGGC	C/T	CTCTTCTAGCTCCCTTACAGTTTGGGTGAGCTTTTCAACCTCTGCCACCTTTTGTCTATGCATTTTGTCCAAAATTTTATTTTCCTCCTGAGAGAAATGG
Ca_LG_2:674716	ACTTTAACACATTACATTCATATCTTGAACTTTCCTTTGATAATCTCTTACTGCATTAGCAGCAGCACCACCTGCAAGGACGGCCTCTTCTAGCTCCCTT	T/C	ACAGTTTGGGTGAGCTTTTCAACCTCTGCCACCTTTTGTCTATGCATTTTGTCCAAAATTTTATTTTCCTCCTGAGAGAAATGGTGATGAAACAAGAAGT
Ca_LG_2:733624	TGGCCCATGAGTTGTTGGAAGAAGGGATTTAATCCCTAAATCTCTATATATAAACAGATACTGCTGCAGCAGCAACATAAGCAGAGAGATCATTGCAACA	A/C	TAGAAGAAGAAAATTGGGTTCCGGCGGTGGATGATTGGTCAGTAATCTTGTTAATTTTCATATGCTGTTCTTATCGCGACTTTGATTATTGATATATTTT
Ca_LG_2:733683	ACTGCTGCAGCAGCAACATAAGCAGAGAGATCATTGCAACATAGAAGAAGAAAATTGGGTTCCGGCGGTGGATGATTGGTCAGTAATCTTGTTAATTTTC	C/A	ATATGCTGTTCTTATCGCGACTTTGATTATTGATATATTTTATTAGTAATTTTGTATTCTCTTTTGAATCGTGATTCAAAGAGAATACAAAACTACGATA
Ca_LG_2:765882	AACCAAAATCTGAAAGCTTAGCATTGTAATCCTGAAAAAAATAAAATGAGTGTGTTCATGTCACTTTATGCTGCCTCTATTTGATCAACATATATTAATT	T/C	GAGTGCATAATGAATGTGTACTTACAGCATCTAATAGAATGTTTGAGGCTTTAATATCTCTATAGATAACTGGTTTCTCTTCTTCATGAAGAAAAGCTAG
Ca_LG_2:765883	ACCAAAATCTGAAAGCTTAGCATTGTAATCCTGAAAAAAATAAAATGAGTGTGTTCATGTCACTTTATGCTGCCTCTATTTGATCAACATATATTAATTG	G/T	AGTGCATAATGAATGTGTACTTACAGCATCTAATAGAATGTTTGAGGCTTTAATATCTCTATAGATAACTGGTTTCTCTTCTTCATGAAGAAAAGCTAGT
Ca_LG_2:765989	TAATGAATGTGTACTTACAGCATCTAATAGAATGTTTGAGGCTTTAATATCTCTATAGATAACTGGTTTCTCTTCTTCATGAAGAAAAGCTAGTCCTTTA	A/T	GCAGCTCCAATTGCAATTTTGATTCTTGTTAACCAAGGCAAGGTTGCTAAATAAGCTGCATTTTCACAACCATTTTGTAAGAAAATATTGAAACAACATT
Ca_LG_2:813151	CGGCTATGGCTGCAGCACAAGGATTGAGCAACTGCTCAGCCCACCTACCATCCTTGTTATTTCCACTGTTACAGTTACTTCCATTGGCCTTAGCTGCACC	C/T	CTTCTTGTTTCCACCTGGTTTTCTAACCACTGTCCCTTCAAGAGCTGCATCTCCATTATCAGCTTCATTCATCGAACGACTCTTGATTCTCTTGTTCTTT
Ca_LG_2:874868	AGACCAAGACCATTGAGAGCTGGAAATTGTGCAACAAATGCTGGTGTCACTGCTGCATTTATGATGTTTGTTGTGTTTCCTGCTTCAATTAGGCCTGAGA	A/T	ATGCAAAGTCATCTGATGTTACTGTTGTTGCTGCTTTGCAAGGGTATCCTGCTGGTGTGTCTGAGGCTTTTATGTCTGCTACACAGAAATCTTGGACTGA
Ca_LG_2:875871	GATTTTAAATTAAAGTCATAATCGCATTATAATTATAATTCTTGATAGGTCAATGCAGCTGCAAGACACAAAAATACCGGGATTAAGATTTTAAATTGCG	G/A	GTCACAGTCAAATTCTAATTCTTGATAGGGGGTGCAGCTGCAAGACATAAAAACTATTGATAACACAATCCAACGGATGAGGACCTGTTCTCAATATCCT
Ca_LG_2:875878	AATTAAAGTCATAATCGCATTATAATTATAATTCTTGATAGGTCAATGCAGCTGCAAGACACAAAAATACCGGGATTAAGATTTTAAATTGCGGTCACAG	G/A	TCAAATTCTAATTCTTGATAGGGGGTGCAGCTGCAAGACATAAAAACTATTGATAACACAATCCAACGGATGAGGACCTGTTCTCAATATCCTGACCAAA

Positions	-100 bp flanking sequences	SNPs within *desi* cultivar	+100 bp flanking sequences
Ca_LG_2:1099766	CTCATCCCCCTTAAACTTGGGAGGATTGTAACGACGGAAGTCTTCTAATCCTCTTGACTCTGCAGCACGGATCTCTCTTCCCCTCTTTTCAAGATCGCGT	T/C	TGAGTCTTGGCAGCAGTCTGTGCAGCAACAAAAGCAGCCATGTTATTCATAGCCTCCGCCATTTGATCATCCCTATTAGCATTGGCTCTCGGAGCGTCAT
Ca_LG_2:1476711	CGAAGGAGTTCAGCTACTGATATCTCTTCTCGGTCTTTCGTCAGAACAGCAGCAAGAATGTGCAGTTGCCTTGCTTTGCCTTTTATCTAATGAAAATGAC	C/T	GAAAGTAAATGGGCAATTACTGCTGCTGGTGGTATACCTCCACTTGTTCAAATTTTGGAGACAGGATCTGCAAAAGCAAAGGAAGATTCTGCAAGAATCC
Ca_LG_2:1480091	TCTTACGAAGTATCTTTCCCTCGGTCCACAAGATGCAACCGAAGAAGCTGCTACAGATCTGCTAGGAATTCTGTTTAGTAGTGCTGATATACGAAAACAC	C/T	GACTCTGCATTTGGTGCTGTTACTCAGCTTGTAGCTGTCCTACGTTTAGGAGGAAGAGGGGCAAGATATAGTGCTGCAAAAGCATTGGAAAGTTTATTTT
Ca_LG_2:1480391	TGCTGCATTGGTTGGTTTACTGAGTGAAAATCCGTCAAGAGCACTTGCTGTTGCAGATGTTGAGATGAATGCAGTGGATGTTCTTTGTAGGATCCTTTCG	G/T	TCGAATTGTTCAATGGACCTCAAAGCGGATGCTGCTGAATTATGCTGTGCTCTTTTTGGAAATACAAGGATTCGGTCCACGATGGCTGCTGAACGATGTG
Ca_LG_2:1480418	AAATCCGTCAAGAGCACTTGCTGTTGCAGATGTTGAGATGAATGCAGTGGATGTTCTTTGTAGGATCCTTTCGTCGAATTGTTCAATGGACCTCAAAGCG	G/A	GATGCTGCTGAATTATGCTGTGCTCTTTTTGGAAATACAAGGATTCGGTCCACGATGGCTGCTGAACGATGTGTTGAACCATTAGTCTCTCTCCTTGCAA
Ca_LG_2:1480472	TCTTTGTAGGATCCTTTCGTCGAATTGTTCAATGGACCTCAAAGCGGATGCTGCTGAATTATGCTGTGCTCTTTTTGGAAATACAAGGATTCGGTCCACG	G/A	ATGGCTGCTGAACGATGTGTTGAACCATTAGTCTCTCTCCTTGCAACTGAGTTTAGTTCCGCTCATCATTCGGTTGTCCGGGCATTGGATAGGCTTGTTG
Ca_LG_2:1577314	CTATCCTCTATTGCTCTGTTATTTGCTAAACGTTCACTAATGATTTGTTCATTATTCTCCACAGTCTACACTGAGGTATAAATAGTAGAACAATGCAAAA	A/C	ATATAGTTGTTTGATTAGTACGGAAAGCAGCATCAAGCAAAGTCATAAATTAATAGCTGAATTCTGATGATAATCACCATCAGATTGTAGCATATCAACA
Ca_LG_2:1577335	TTTGCTAAACGTTCACTAATGATTTGTTCATTATTCTCCACAGTCTACACTGAGGTATAAATAGTAGAACAATGCAAAAATATAGTTGTTTGATTAGTAC	C/T	GGAAAGCAGCATCAAGCAAAGTCATAAATTAATAGCTGAATTCTGATGATAATCACCATCAGATTGTAGCATATCAACAGTTTGGTTATAAAAGTCTCTA
Ca_LG_2:2090337	TTAGTGCTGTCAAACACAGATCACGGGAAATGACATTTGTTCAAATTCTGCTACACTACAATGCTATAGCTGCTATTTGACTACACTTTGTACTAAATCG	G/A	GGCATCGTGCAACAATAGTGGTTTGTCAATATTCCACTTCGCAGCAAAAATTAATCAAACCCCCTGCTTAACGAAAGATATATGAGCATGTTCTGAAGCA
Ca_LG_2:2148375	AGATGAGGTGGTGTCGAGCAACCCATGGCCTTGCAAAGTTTGTTGGGGAAGTGTAAACAATTAGTGGATGGGTTACTACTATCCACAAAGATGTTTTTGT	T/C	TGGCTGATGATAATGATAAAGAGTTAGGACAACAGCTAAAAGATGAATTGAATAGGGAATTGCAAGAGCATTTGGAATGCTACACTGCAAGGGCTACCTC
Ca_LG_2:2285962	TTATAACATACTGAAGAATATTTAGATGATACTTAAAGATAGAATGCCCAACCTCCGTATCTAGCATTAAGAACAAAGGGCTGTAATGTTCTGGCAGCTA	A/G	CAACGGTAAAATTCCTAGAATTTGTCATCTGGCACAAATCCCCTTTGCAGCATTTCATCATGGACACGGAATGCCTCTTTTAGGTTGTCGTTCGAGACAT
Ca_LG_2:2285980	TATTTAGATGATACTTAAAGATAGAATGCCCAACCTCCGTATCTAGCATTAAGAACAAAGGGCTGTAATGTTCTGGCAGCTACAACGGTAAAATTCCTAG	G/A	AATTTGTCATCTGGCACAAATCCCCTTTGCAGCATTTCATCATGGACACGGAATGCCTCTTTTAGGTTGTCGTTCGAGACATACCCATTTATTAGTAATG

**Positions**	**-100 bp flanking sequences**	**SNPs within *desi* cultivar**	**+100 bp flanking sequences**
Ca_LG_2:2727162	GGTAATCCACCGTTGGTCATAGCACATGGTGGTTTTTCGGGGATATTTCCCGATTCAAGTGGTGCTGCTTATAGTTTGGCCTTACAAACGAGCTTACCGG	G/A	ATGTTGCTCTATGGTGTGATGTGCAATTGACAAAAGATGCAGCTGGGATTTGCCTTCCAAATGTCAACCTTTTAAATTCTACTTACGTTTCAATTGCTAT
Ca_LG_2:2727167	TCCACCGTTGGTCATAGCACATGGTGGTTTTTCGGGGATATTTCCCGATTCAAGTGGTGCTGCTTATAGTTTGGCCTTACAAACGAGCTTACCGGATGTT	T/C	GCTCTATGGTGTGATGTGCAATTGACAAAAGATGCAGCTGGGATTTGCCTTCCAAATGTCAACCTTTTAAATTCTACTTACGTTTCAATTGCTATCCCAA
Ca_LG_2:2730020	GACCTATAAATGCTCTGTTTCAAATTTAATTTTCTCTTATTTTCCATTTAATAATATTATGTTGTGTTCTTCTCTTATCAGAATGCAGCCTATCTTGCAG	G/A	AAAACCAAGGATTTAGTGTGACCGATAAGGTCATTGATGCTTTGCGCAAAGCAGGTTATGATAAACCAGGGAGCCAAAAGGTTTATATTCAATCCACTAA
Ca_LG_2:2730134	AGTGTGACCGATAAGGTCATTGATGCTTTGCGCAAAGCAGGTTATGATAAACCAGGGAGCCAAAAGGTTTATATTCAATCCACTAATAGCTCTGTACTTC	C/T	TGAAATTCAAGGAGAAAACCAATTACGAGCTTGTCTACAAGATTGACGAGACTGTCAGTGATGCTGCCAACGCAGCTGTTGAGGATATAAAAACGTTTGC
Ca_LG_2:2730179	GATAAACCAGGGAGCCAAAAGGTTTATATTCAATCCACTAATAGCTCTGTACTTCTGAAATTCAAGGAGAAAACCAATTACGAGCTTGTCTACAAGATTG	G/T	ACGAGACTGTCAGTGATGCTGCCAACGCAGCTGTTGAGGATATAAAAACGTTTGCGGGTTCTGTGGTTATCAACAAGGACTCGGTATTTCCTCAGAATAG
Ca_LG_2:2730193	CCAAAAGGTTTATATTCAATCCACTAATAGCTCTGTACTTCTGAAATTCAAGGAGAAAACCAATTACGAGCTTGTCTACAAGATTGACGAGACTGTCAGT	T/C	GATGCTGCCAACGCAGCTGTTGAGGATATAAAAACGTTTGCGGGTTCTGTGGTTATCAACAAGGACTCGGTATTTCCTCAGAATAGTGGCTTCATAACGG
Ca_LG_2:2830949	ATCATGGTTGGTTAAGAACCAAACGAATCGCTCTTCCTCTGTCAGACAAACGAAACGCTTCCGACAAACGAATCACGAATCACTCCTGTGCTCGCTGCGG	G/A	GCCCGCGGCCGCCTCTGCCTCCGTGGCCGTTCGTCGCTCTCGCCTCTCTGTTCGCCGCAACTCTCCAGATCGCGTTGCTGCTTTGTTCAAAAGGTTCGAA
Ca_LG_2:2989538	GCAATGATCGGTCCTATGTATATGGTTGAATGGAAGTCGAGTCATGTGTAACTAGCTCCTAATGTTTTCTCAAGTATATGAGCTGTTAAGCATAATTAAC	C/T	TCTTCTTGGATTCAGCTTTTGTTATGTAAAAGTGAGCAGCAGTGGGGGAGAGTAAAATAACAGACTATTTTTTGTATGTATTTATATATGAATGTCGTGG
Ca_LG_2:3001274	AGCAGCAGAGGCTAGTATACTTCATCAGATTAGAGTCTTATCCGAAAGTACTAGTGCTGCTCGCACCTCAACATCTGAATCTGGTGCTAGAATTACAATA	A/C	TCGAAGGAGGAATTTGATTCCTTAAGCCGCAAGGTTGAGGAATCCGATAAACTAGCAAGCATCAAAGTAGCTGCTTCCACAGCACAGGTTGAAGCTGTGA
Ca_LG_2:3001822	GTATTAGTGCCATCTTTCATAAGAAAAAGAGGCTGCATGTTGAGAAAGGATATCCTTCTTACCTTCCAGGTGAAAATCCTTTGTGAAATTTTGTTTTAGC	C/T	TTTTGTGCAGCTCTTGCATGTGAGAGATTAAACATATTGGTTTATGTGAAAGTTGAGGCCTATGTGATCAGATTTTTGTCATAATCATAGCTTGAGAGAA
Ca_LG_2:3081515	TGGATGAATCTTTTAGGTGTTTACCCTCTTGATAAAAGTCAGTGTTGTTAAATGGTGGTCATGGCGGTTCTATAGGCTGCAATGGCATGTTTATACGGCG	G/A	GATTTTTGTCCTTCCGCCACCGGCAACACTGATCAAAGATTACTTATTTTGTTCATTATTGTAATCTGTTGTGTAACCCGCTTGAGCTGCCACTGTGTCA
Ca_LG_2:3134304	GGCCTCCTACACAATCAAGTAGGAGAGCTGCTGTTGAAAAAAGGAGAAAGAGAAATGAGGAAAAGCAGGCGCCCCAAGATGAGTAGCCTCAGCAAAATCC	C/T	TATGTTTGATGCAGAACATGATGAGCAGCCCCATGATGAGCAGCCCCAGCAAGATCATGTGTTTGATGATGAACATGATCAGCAGCAGCAACAGTTTGAT

Positions	-100 bp flanking sequences	SNPs within *desi* cultivar	+100 bp flanking sequences
Ca_LG_2:3153069	ATAACAATAAAATAAAAACCCTAATCAATCAATGGTTTGCATGATATCTTCAGGAGCAAACTTAGTACCCTTTTCCTTATCAGCAGCAGCACGACCCTTC	C/T	GCCTTGCGGTCAAGCAACGACTTACGATCCTTATCCAAACGGAGTTTAGTGATAACAACTTTGGAAGGGTTGACTCCAACGTTGACTGTTGAACCGTTAA
Ca_LG_2:3153078	AAATAAAAACCCTAATCAATCAATGGTTTGCATGATATCTTCAGGAGCAAACTTAGTACCCTTTTCCTTATCAGCAGCAGCACGACCCTTCGCCTTGCGG	G/A	TCAAGCAACGACTTACGATCCTTATCCAAACGGAGTTTAGTGATAACAACTTTGGAAGGGTTGACTCCAACGTTGACTGTTGAACCGTTAACTTTTTCTC
Ca_LG_2:3158445	CCATTTTCCCCCTAATAATTATCAAATTTTTGTAAGCAGCTTATATTTATTCAATTTACCAATAACTATGACCATCAATTATAATGAAGCTTCTAAAGCA	A/G	CGGACAATCTCTCTTATTGCTTAATGGCTAGCTGCATTCAATTTGAAAGACCACGTCCACCTAAAGCATGCTGGTACTTTCGTACAATATATGAATATCA
Ca_LG_2:3158619	TACTTTCGTACAATATATGAATATCATGTCACCACACACTACTGATCCCGTGCCAAGTGTCAAATTCATTGTAATGCAGCTTAAACTCAAATTTTTCTCA	A/G	TGTCTAGCTACCCCGTGAATCAGTAGTAGTGATGTAGTTCCGCTGCATAGTGAAGCACAACTTACCCCAAAATGATAAAATAGAATGACATAGGCCAAGT
Ca_LG_2:3158634	TATGAATATCATGTCACCACACACTACTGATCCCGTGCCAAGTGTCAAATTCATTGTAATGCAGCTTAAACTCAAATTTTTCTCATGTCTAGCTACCCCG	G/A	TGAATCAGTAGTAGTGATGTAGTTCCGCTGCATAGTGAAGCACAACTTACCCCAAAATGATAAAATAGAATGACATAGGCCAAGTTTTGGTTAACTTTTG
Ca_LG_2:4140265	NNNNNNNNNNNNNNNNNNNNNNNNNNNNNNNNNNNNNNNNNNNNNATACAACCTAATCATCACCGCAATATATTTCACAACAATAACAACAACAACAAAA	A/G	TTACAAGTACTATTCATTGAATTTCATATTGAAGAATGAAATCCATTAACATATAAACATTATTTGAAACCAGAATAAATGGCGACATCAAAAACCATCT
Ca_LG_2:4658907	AGAGGTTGCATATTTTTATTAGTGGAGTCTGAAGATACTCCATTTGTAATTGCTGCTTTTGTATAGTTAAAATCTTTTTCATCAGTTTTGGGTTGGTTTG	G/A	GAGAAGGAACAGGTTTTGTTTGTGGCCTTGGATTCTGACTAACAACTCCTGACTGCTGCTTACCATTGTTGGTGATAGGAGGATCACCAGTTTCCTCTGG
Ca_LG_2:4705113	TGATAAAATTTTAGTATGAACTTATAAATTGTTTAGAAAGACATACATTACAGTCAAATGCAAATCTCCTTGCAAGCATGATGGCTGCATTCCTTTCTCG	G/T	CTCTGATTCAAAAGCTAATACAAAAGAATGTCCTTGTTTCGGCTGCCAAAATAGTGCCTGCGCCGCAGCATTTCCACCGCCTCGAACTCCACAAAGCTAA
Ca_LG_2:4705138	AAATTGTTTAGAAAGACATACATTACAGTCAAATGCAAATCTCCTTGCAAGCATGATGGCTGCATTCCTTTCTCGCTCTGATTCAAAAGCTAATACAAAA	A/T	GAATGTCCTTGTTTCGGCTGCCAAAATAGTGCCTGCGCCGCAGCATTTCCACCGCCTCGAACTCCACAAAGCTAAAGAACACATTACCAAAGCACATTGG
Ca_LG_2:4705141	TTGTTTAGAAAGACATACATTACAGTCAAATGCAAATCTCCTTGCAAGCATGATGGCTGCATTCCTTTCTCGCTCTGATTCAAAAGCTAATACAAAAGAA	A/G	TGTCCTTGTTTCGGCTGCCAAAATAGTGCCTGCGCCGCAGCATTTCCACCGCCTCGAACTCCACAAAGCTAAAGAACACATTACCAAAGCACATTGGTTT
Ca_LG_2:4705142	TGTTTAGAAAGACATACATTACAGTCAAATGCAAATCTCCTTGCAAGCATGATGGCTGCATTCCTTTCTCGCTCTGATTCAAAAGCTAATACAAAAGAAT	T/A	GTCCTTGTTTCGGCTGCCAAAATAGTGCCTGCGCCGCAGCATTTCCACCGCCTCGAACTCCACAAAGCTAAAGAACACATTACCAAAGCACATTGGTTTC
Ca_LG_2:4705153	ACATACATTACAGTCAAATGCAAATCTCCTTGCAAGCATGATGGCTGCATTCCTTTCTCGCTCTGATTCAAAAGCTAATACAAAAGAATGTCCTTGTTTC	C/T	GGCTGCCAAAATAGTGCCTGCGCCGCAGCATTTCCACCGCCTCGAACTCCACAAAGCTAAAGAACACATTACCAAAGCACATTGGTTTCCCAAATTCAAG

**Positions**	**-100 bp flanking sequences**	**SNPs within *desi* cultivar**	**+100 bp flanking sequences**
Ca_LG_2:4705156	TACATTACAGTCAAATGCAAATCTCCTTGCAAGCATGATGGCTGCATTCCTTTCTCGCTCTGATTCAAAAGCTAATACAAAAGAATGTCCTTGTTTCGGC	C/T	TGCCAAAATAGTGCCTGCGCCGCAGCATTTCCACCGCCTCGAACTCCACAAAGCTAAAGAACACATTACCAAAGCACATTGGTTTCCCAAATTCAAGTAG
Ca_LG_2:4765841	AAAAAATAAGGAAAAGACAGAAAAAGAACATTGCAGTAATCTGCATAGTAAAAGAGAACATAAATTGAATCCTAATTTCGGTTAGAGAAATAAAAATACA	A/G	TTAGACATAAAACACAGGATCTGAAAAAGGGATGAAATAGCATGCGGAAAAAGATAACCTTGTAATCATAAAGATCGCTACCAGCATAACCACTACTTTC
Ca_LG_2:4765859	AGAAAAAGAACATTGCAGTAATCTGCATAGTAAAAGAGAACATAAATTGAATCCTAATTTCGGTTAGAGAAATAAAAATACATTAGACATAAAACACAGG	G/A	ATCTGAAAAAGGGATGAAATAGCATGCGGAAAAAGATAACCTTGTAATCATAAAGATCGCTACCAGCATAACCACTACTTTCACTTAATTTTCCGCTGTG
Ca_LG_2:4765863	AAAGAACATTGCAGTAATCTGCATAGTAAAAGAGAACATAAATTGAATCCTAATTTCGGTTAGAGAAATAAAAATACATTAGACATAAAACACAGGATCT	T/C	GAAAAAGGGATGAAATAGCATGCGGAAAAAGATAACCTTGTAATCATAAAGATCGCTACCAGCATAACCACTACTTTCACTTAATTTTCCGCTGTGAACA
Ca_LG_2:4765864	AAGAACATTGCAGTAATCTGCATAGTAAAAGAGAACATAAATTGAATCCTAATTTCGGTTAGAGAAATAAAAATACATTAGACATAAAACACAGGATCTG	G/A	AAAAAGGGATGAAATAGCATGCGGAAAAAGATAACCTTGTAATCATAAAGATCGCTACCAGCATAACCACTACTTTCACTTAATTTTCCGCTGTGAACAC
Ca_LG_2:4765873	GCAGTAATCTGCATAGTAAAAGAGAACATAAATTGAATCCTAATTTCGGTTAGAGAAATAAAAATACATTAGACATAAAACACAGGATCTGAAAAAGGGA	A/T	TGAAATAGCATGCGGAAAAAGATAACCTTGTAATCATAAAGATCGCTACCAGCATAACCACTACTTTCACTTAATTTTCCGCTGTGAACACGTTCAACAT
Ca_LG_2:4765970	GGATGAAATAGCATGCGGAAAAAGATAACCTTGTAATCATAAAGATCGCTACCAGCATAACCACTACTTTCACTTAATTTTCCGCTGTGAACACGTTCAA	A/G	CATCTTCATCTGTCTCTTGATCAACTTCATTCTCTGCATCATGGTAACCATTTCCTTTCACATCTATATTGTCGTCATCGGTCGAATCTTCGGTCCCGCT
Ca_LG_2:5178762	TGTTTTCTTATATTGATTCAGCATAATCCTTTGAAATGAATGATTTGGGCACAATGTTGTCAAATAGCAGCTATATAGCATAGCGGATTTTGATGAATTT	T/C	GCTATGAAAGCCTTGATGCTATCCTAGTTTCGCTACTAGGACAAAAGTGGCCGCTATTCTAGTTTCTTGTAGCAGCAATAGCGGGAATTAAAAATCAAAT
Ca_LG_2:5178815	ATGTTGTCAAATAGCAGCTATATAGCATAGCGGATTTTGATGAATTTGCTATGAAAGCCTTGATGCTATCCTAGTTTCGCTACTAGGACAAAAGTGGCCG	G/A	CTATTCTAGTTTCTTGTAGCAGCAATAGCGGGAATTAAAAATCAAATTTTAGGTATTTTAAAATAATAAAACTGAAAATTTTGCACCGAGTGCCAGACGT
Ca_LG_2:5831254	TTCTTCCCTTGAATTAGATTGTGTTGAACCTGTTGCTAGCCCTCTTCCTAAAATGATCATTGTAGCGTCCGTCGCTGCTGGTGTTCAGTTTGGATGGGCG	G/A	CTACAGCTCTCATTGTTGACCCCTTACGTGCAGACTCTTGGCGTTCCGCACATATGGGCATCTTTTATTTGGCTCTGTGGTCCAATGTCGGGTCTTCTTG
Ca_LG_2:5831260	CCTTGAATTAGATTGTGTTGAACCTGTTGCTAGCCCTCTTCCTAAAATGATCATTGTAGCGTCCGTCGCTGCTGGTGTTCAGTTTGGATGGGCGCTACAG	G/A	CTCTCATTGTTGACCCCTTACGTGCAGACTCTTGGCGTTCCGCACATATGGGCATCTTTTATTTGGCTCTGTGGTCCAATGTCGGGTCTTCTTGTTCAAC
Ca_LG_2:5831649	GATTCTGGATCCTTGACGTAGCAAACAACATGCTCCAGGGACCATGCCGTGCTTTCCTCGCCGACTTAGCAGCCAGCGATGAAAAAATGACAAGAACAGG	G/C	TAACGCGTTCTTCTCGTTCTTCATGGCTGTCGGAAACATTTTGGGATACGCAGCTGGTTCATACAGCAGCCTATACAAAATCTTCCCATTCACCGAAACC

**Positions**	**-100 bp flanking sequences**	**SNPs within *desi* cultivar**	**+100 bp flanking sequences**
Ca_LG_2:5831656	GATCCTTGACGTAGCAAACAACATGCTCCAGGGACCATGCCGTGCTTTCCTCGCCGACTTAGCAGCCAGCGATGAAAAAATGACAAGAACAGGTAACGCG	G/A	TTCTTCTCGTTCTTCATGGCTGTCGGAAACATTTTGGGATACGCAGCTGGTTCATACAGCAGCCTATACAAAATCTTCCCATTCACCGAAACCGCGGCAT
Ca_LG_2:5831662	TGACGTAGCAAACAACATGCTCCAGGGACCATGCCGTGCTTTCCTCGCCGACTTAGCAGCCAGCGATGAAAAAATGACAAGAACAGGTAACGCGTTCTTC	C/T	TCGTTCTTCATGGCTGTCGGAAACATTTTGGGATACGCAGCTGGTTCATACAGCAGCCTATACAAAATCTTCCCATTCACCGAAACCGCGGCATGCAACA
Ca_LG_2:5831665	CGTAGCAAACAACATGCTCCAGGGACCATGCCGTGCTTTCCTCGCCGACTTAGCAGCCAGCGATGAAAAAATGACAAGAACAGGTAACGCGTTCTTCTCG	G/A	TTCTTCATGGCTGTCGGAAACATTTTGGGATACGCAGCTGGTTCATACAGCAGCCTATACAAAATCTTCCCATTCACCGAAACCGCGGCATGCAACATTT
Ca_LG_2:5831668	AGCAAACAACATGCTCCAGGGACCATGCCGTGCTTTCCTCGCCGACTTAGCAGCCAGCGATGAAAAAATGACAAGAACAGGTAACGCGTTCTTCTCGTTC	C/T	TTCATGGCTGTCGGAAACATTTTGGGATACGCAGCTGGTTCATACAGCAGCCTATACAAAATCTTCCCATTCACCGAAACCGCGGCATGCAACATTTTCT
Ca_LG_2:5831677	CATGCTCCAGGGACCATGCCGTGCTTTCCTCGCCGACTTAGCAGCCAGCGATGAAAAAATGACAAGAACAGGTAACGCGTTCTTCTCGTTCTTCATGGCT	T/C	GTCGGAAACATTTTGGGATACGCAGCTGGTTCATACAGCAGCCTATACAAAATCTTCCCATTCACCGAAACCGCGGCATGCAACATTTTCTGCGCCAATC
Ca_LG_2:5873513	TCATTTTCATTCATATTGTATGGCAGCCTAGAAATTTTTATGCAGCCAGAAATATTAAGCTTTTTAAGAGATTTCATGTTGCAGACAAAAGTTGGTATGT	T/C	AAAGAAGATTTTTGCAATCCTCCAAACTAAGACTTGATAAGTATTTCATATTCTTTCCAAACTTTGGAAGCTTTTTTACTTTTTTGCAGCCTGAAAAAAT
Ca_LG_2:5873610	TGTAAAGAAGATTTTTGCAATCCTCCAAACTAAGACTTGATAAGTATTTCATATTCTTTCCAAACTTTGGAAGCTTTTTTACTTTTTTGCAGCCTGAAAA	A/G	AATCAATTCCTCTAGAGAATCCATTTCCAATTTTGTTGGAAGGGTTTGAAGATTTATGCAATCTTTCAAGTTCAGTTTGACAAGCTTCTTGTGTTTTCCA
Ca_LG_2:5873617	AAGATTTTTGCAATCCTCCAAACTAAGACTTGATAAGTATTTCATATTCTTTCCAAACTTTGGAAGCTTTTTTACTTTTTTGCAGCCTGAAAAAATCAAT	T/C	TCCTCTAGAGAATCCATTTCCAATTTTGTTGGAAGGGTTTGAAGATTTATGCAATCTTTCAAGTTCAGTTTGACAAGCTTCTTGTGTTTTCCAACAGATT
Ca_LG_2:5873622	TTTTGCAATCCTCCAAACTAAGACTTGATAAGTATTTCATATTCTTTCCAAACTTTGGAAGCTTTTTTACTTTTTTGCAGCCTGAAAAAATCAATTCCTC	C/T	TAGAGAATCCATTTCCAATTTTGTTGGAAGGGTTTGAAGATTTATGCAATCTTTCAAGTTCAGTTTGACAAGCTTCTTGTGTTTTCCAACAGATTTGTGA
Ca_LG_2:5873638	ACTAAGACTTGATAAGTATTTCATATTCTTTCCAAACTTTGGAAGCTTTTTTACTTTTTTGCAGCCTGAAAAAATCAATTCCTCTAGAGAATCCATTTCC	C/A	AATTTTGTTGGAAGGGTTTGAAGATTTATGCAATCTTTCAAGTTCAGTTTGACAAGCTTCTTGTGTTTTCCAACAGATTTGTGAACCTTAACAAGGCTTG
Ca_LG_2:5892888	TGCGTGAGACCGCGAGTTCGTGTTCGTCTCCCCCGTGCGCCGTGCTGCTGCTGCGTCCGCCGTCCAGCAGTCTCTGTTCTGCGTGAGTATTCTTCACTGC	C/T	CATTTTGCACTTGATTTGTTGTTGAATTTTCTGTTATCATTATTCATTGCTGCACTTGATTTTCTGTTGGGAGTTGAATTGTTATATCATTGAATTGAAT
Ca_LG_2:5893006	TTGTTGAATTTTCTGTTATCATTATTCATTGCTGCACTTGATTTTCTGTTGGGAGTTGAATTGTTATATCATTGAATTGAATTGAATTGTTTTGAACATG	G/C	TTATCATTATTCATTGCTGCTCTTGATTTTCTGTTGTTGCGCTAAACTTGTTCTCTTATCATTGATGCAAATGAATTGTTGCGAGCTGAACTGAGTTTTG

Positions	-100 bp flanking sequences	SNPs within *desi* cultivar	+100 bp flanking sequences
Ca_LG_2:6112086	TTGCGCATTTCTATGGATGATGACTCAGTAGCCTCTGATCAAGACAACTTAGAGAAAGGAAAATATAATGAACAGAACCCTCAAATTCGACTTGCTTATG	G/A	ACATTGCTGCTTCAGCTGCCTCCTATGTTCAATTGCAAACAAAGAACCTCTTATCCCTTGCTGCCAAGTCACAACAAAGTGATAATGAAGATTCCAATGA
Ca_LG_2:6263590	GGTTGCGGCGGCGGTGCATTTAGGTTGCTCGTTGAAGGTTGGGGCGGCAGCGGCGGCGGTGAGGTTGCGGCGGTGAGGTTGCTCGTTGAAGGTTGCAGCG	G/A	GCGGCGGCGGTGAGGTTGCGGCGGTGCGTTTAGGGATTTTGCAGGAGAGAAGGGTAAGTTGAAGCAGCGCAGCTTGTAAAAGAGGATTTAGGTATTTGGC
Ca_LG_2:6263626	GGTTGGGGCGGCAGCGGCGGCGGTGAGGTTGCGGCGGTGAGGTTGCTCGTTGAAGGTTGCAGCGGCGGCGGCGGTGAGGTTGCGGCGGTGCGTTTAGGGA	A/G	TTTTGCAGGAGAGAAGGGTAAGTTGAAGCAGCGCAGCTTGTAAAAGAGGATTTAGGTATTTGGCAGCGGGAACTCAAAGTTTTGAGGCGGGACTGAATTT
Ca_LG_2:6263632	GGCGGCAGCGGCGGCGGTGAGGTTGCGGCGGTGAGGTTGCTCGTTGAAGGTTGCAGCGGCGGCGGCGGTGAGGTTGCGGCGGTGCGTTTAGGGATTTTGC	C/T	AGGAGAGAAGGGTAAGTTGAAGCAGCGCAGCTTGTAAAAGAGGATTTAGGTATTTGGCAGCGGGAACTCAAAGTTTTGAGGCGGGACTGAATTTTTTATT
Ca_LG_2:6472192	TTCTTTTGACAAATTCCACCTTGCAAATATCTAAACTGTAACTGTCCAATGGGATGGTGCAGCTTCAAATTCACTTTTTCAAGCGTATAACACCGTGTGT	T/A	CATCCGTGTGTCTCTATCCCTAGGACAAACCCGCGTGAACACCTTTGACTTAGGATCATGATTTACTGCGGTTGAAATCCCTTACATGAACGCAGTAACT
Ca_LG_2:6472207	CCACCTTGCAAATATCTAAACTGTAACTGTCCAATGGGATGGTGCAGCTTCAAATTCACTTTTTCAAGCGTATAACACCGTGTGTCATCCGTGTGTCTCT	T/A	ATCCCTAGGACAAACCCGCGTGAACACCTTTGACTTAGGATCATGATTTACTGCGGTTGAAATCCCTTACATGAACGCAGTAACTCGTCGGTAATCGTTC
Ca_LG_2:6472211	CTTGCAAATATCTAAACTGTAACTGTCCAATGGGATGGTGCAGCTTCAAATTCACTTTTTCAAGCGTATAACACCGTGTGTCATCCGTGTGTCTCTATCC	C/G	CTAGGACAAACCCGCGTGAACACCTTTGACTTAGGATCATGATTTACTGCGGTTGAAATCCCTTACATGAACGCAGTAACTCGTCGGTAATCGTTCGATC
Ca_LG_2:6610438	GAAACATGAAAGCAAACCAGATTTTCACATTCTTCTTATTGACTGCCAGAAGTAGTTATAGTTTAATGTGTTAATTTAGAGGCAATATTTCCATCCTCTC	C/T	GGATACGATGGTGTTTGTTATCTTGTTTGCATACATGAAAGTAATAGCAGTAACAGAAAGGAAGCAGCTGCATATTTTCAATCCTTGACTCCATATCTCT
Ca_LG_2:6779817	ACTCCACTCCTAACTCCGCCACCTCAGCCACCAAGCCAACTTTATATCCCCACCTCTTCCTATCAGTGTTGTTAAATGGCAGCCACCGCAGAATGGCGCT	T/G	GTGGATTTCTTTGACAAGGGTCATAGGCAATTTATGAAGGGATGCCTGCCGTGACTTAAAATAAATTGTACATCTGCCTCATCTTTTTTTTAACCCTCTA
Ca_LG_2:6779831	TCCGCCACCTCAGCCACCAAGCCAACTTTATATCCCCACCTCTTCCTATCAGTGTTGTTAAATGGCAGCCACCGCAGAATGGCGCTGTGGATTTCTTTGA	A/G	CAAGGGTCATAGGCAATTTATGAAGGGATGCCTGCCGTGACTTAAAATAAATTGTACATCTGCCTCATCTTTTTTTTAACCCTCTAGCTCCCAGAGTAAT
Ca_LG_2:6779862	ATCCCCACCTCTTCCTATCAGTGTTGTTAAATGGCAGCCACCGCAGAATGGCGCTGTGGATTTCTTTGACAAGGGTCATAGGCAATTTATGAAGGGATGC	C/T	CTGCCGTGACTTAAAATAAATTGTACATCTGCCTCATCTTTTTTTTAACCCTCTAGCTCCCAGAGTAATCCAAAGTTCAAATGGTAGGTAAGTAAAACCT
Ca_LG_2:6936604	TCCACTAGCAACATCCAAGATTAAGGGGTTAAGTCGCATTTGGGGTTGCCTAACTGATTTTACCCCATCCTCCAATAGTATCCTATGCATAATATGGACG	G/T	GACTAATACCAGGAATATCAGCCAATGTCCATCCGATTGCTTTCCTGTGTTTTTTCAAAATTTGCAGCAACTTATCTTCCTGTTCATCTTCAAGTTTGGC

Positions	-100 bp flanking sequences	SNPs within *desi* cultivar	+100 bp flanking sequences
Ca_LG_2:6936630	GGTTAAGTCGCATTTGGGGTTGCCTAACTGATTTTACCCCATCCTCCAATAGTATCCTATGCATAATATGGACGGACTAATACCAGGAATATCAGCCAAT	T/G	GTCCATCCGATTGCTTTCCTGTGTTTTTTCAAAATTTGCAGCAACTTATCTTCCTGTTCATCTTCAAGTTTGGCTGAAATAATCACATGTAATTTTCCAC
Ca_LG_2:7573082	AATTATTTTCAAATAATGAAGTAAGATTTGATGTGTATATACCATTAGCATCTTTGAGACATTGCTGGCACCAAAAATTTTGTGTACAGAAGCAAACTTC	C/T	TGAGGCTCATGAGGAGAGAAATATGGAGAAAAAGGGCATTCTTGTGCACACCTTCTTCTCAAAAGCTTACATGCTGCACAAGGTGTTGTTGTATTTAATG
Ca_LG_2:7573094	ATAATGAAGTAAGATTTGATGTGTATATACCATTAGCATCTTTGAGACATTGCTGGCACCAAAAATTTTGTGTACAGAAGCAAACTTCTGAGGCTCATGA	A/T	GGAGAGAAATATGGAGAAAAAGGGCATTCTTGTGCACACCTTCTTCTCAAAAGCTTACATGCTGCACAAGGTGTTGTTGTATTTAATGTTCCAGAACCTC
Ca_LG_2:7573097	ATGAAGTAAGATTTGATGTGTATATACCATTAGCATCTTTGAGACATTGCTGGCACCAAAAATTTTGTGTACAGAAGCAAACTTCTGAGGCTCATGAGGA	A/T	GAGAAATATGGAGAAAAAGGGCATTCTTGTGCACACCTTCTTCTCAAAAGCTTACATGCTGCACAAGGTGTTGTTGTATTTAATGTTCCAGAACCTCCTA
Ca_LG_2:7573100	AAGTAAGATTTGATGTGTATATACCATTAGCATCTTTGAGACATTGCTGGCACCAAAAATTTTGTGTACAGAAGCAAACTTCTGAGGCTCATGAGGAGAG	G/A	AAATATGGAGAAAAAGGGCATTCTTGTGCACACCTTCTTCTCAAAAGCTTACATGCTGCACAAGGTGTTGTTGTATTTAATGTTCCAGAACCTCCTAACA
Ca_LG_2:7573106	GATTTGATGTGTATATACCATTAGCATCTTTGAGACATTGCTGGCACCAAAAATTTTGTGTACAGAAGCAAACTTCTGAGGCTCATGAGGAGAGAAATAT	T/A	GGAGAAAAAGGGCATTCTTGTGCACACCTTCTTCTCAAAAGCTTACATGCTGCACAAGGTGTTGTTGTATTTAATGTTCCAGAACCTCCTAACATTCTTC
Ca_LG_2:7573118	ATATACCATTAGCATCTTTGAGACATTGCTGGCACCAAAAATTTTGTGTACAGAAGCAAACTTCTGAGGCTCATGAGGAGAGAAATATGGAGAAAAAGGG	G/A	CATTCTTGTGCACACCTTCTTCTCAAAAGCTTACATGCTGCACAAGGTGTTGTTGTATTTAATGTTCCAGAACCTCCTAACATTCTTCCCTTTTTAAAAC
Ca_LG_2:7573124	CATTAGCATCTTTGAGACATTGCTGGCACCAAAAATTTTGTGTACAGAAGCAAACTTCTGAGGCTCATGAGGAGAGAAATATGGAGAAAAAGGGCATTCT	T/C	TGTGCACACCTTCTTCTCAAAAGCTTACATGCTGCACAAGGTGTTGTTGTATTTAATGTTCCAGAACCTCCTAACATTCTTCCCTTTTTAAAACAACCAT
Ca_LG_2:7573126	TTAGCATCTTTGAGACATTGCTGGCACCAAAAATTTTGTGTACAGAAGCAAACTTCTGAGGCTCATGAGGAGAGAAATATGGAGAAAAAGGGCATTCTTG	G/C	TGCACACCTTCTTCTCAAAAGCTTACATGCTGCACAAGGTGTTGTTGTATTTAATGTTCCAGAACCTCCTAACATTCTTCCCTTTTTAAAACAACCATCT
Ca_LG_2:7573127	TAGCATCTTTGAGACATTGCTGGCACCAAAAATTTTGTGTACAGAAGCAAACTTCTGAGGCTCATGAGGAGAGAAATATGGAGAAAAAGGGCATTCTTGT	T/A	GCACACCTTCTTCTCAAAAGCTTACATGCTGCACAAGGTGTTGTTGTATTTAATGTTCCAGAACCTCCTAACATTCTTCCCTTTTTAAAACAACCATCTC
Ca_LG_2:7587499	TATTGAATAAGAAGATGAAGAAGGAGAGACTTTTTCTTGAGAGAACACGGGAGAATCTACAATCAAATTTTGCAGCTATCTCTGCTCAAAATGTGGTCCA	A/G	GAGAGAAGCAAGGTCAAAGCCACCACCAGCAGCTTCTGTAGAAAAGAGTTTTCATACCCCCCAAAATATTGTGAGGGATTTAAAAATTGATGCTGAACAG
Ca_LG_2:7657110	TTATATATTAACTGGATTTACAGAACTATGGTTGATGAATTCCTGATTTAGTTTCTTTGGCATTTTTTATGACTGAATTACAATATACTGTTGTTCCTAA	A/G	ATATGTTGAAACATAGTTCGCTGCATAGATATGTTGTATTGTCTTATGTATCCCTGAACAAAAGTTTTTTATTTGGCTTGAGGTATGATCTAGGTTTTTA

Positions	-100 bp flanking sequences	SNPs within *desi* cultivar	+100 bp flanking sequences
Ca_LG_2:7769703	CATTAAATCGTTCAAAAGCTGTAAAGCCTCTTGATCATATTCA GCTTGTTCTTCCATCATTCTTTGGTATTGCAATGCTTCCATCTG CATTGCTGCTTTT	T/C	TCTTCTTGCAACCTTGTTATCATTGCCATTGTTTGGTTTGCAG CTATTGCTGAGGCACTTCTCTCTTCTTCTAGTTCTTGATATAT AGCACTTAGAGCTT
Ca_LG_2:7769707	AAATCGTTCAAAAGCTGTAAAGCCTCTTGATCATATTCAGCTT GTTCTTCCATCATTCTTTGGTATTGCAATGCTTCCATCTGCATT GCTGCTTTTTCTT	T/G	CTTGCAACCTTGTTATCATTGCCATTGTTTGGTTTGCAGCTAT TGCTGAGGCACTTCTCTCTTCTTCTAGTTCTTGATATATAGCA CTTAGAGCTTTTTG
Ca_LG_2:8024030	ACCAAGATGTTTTCCTTCTGTGTTTCAATGTAAAAGCTTTTGAA TTTTCCTTGCATGTCAGACATCAGTGCAACTCTTAATTGAAAAT TTTGCCTCTTTA	A/G	AGGCATCATATGTTACTACACCATCATTGCACAATTGTTGCA ACTCATAAGCGGCTGTAAATACACATCAATTTTACCTTTTGG ATTGTACCTGGAATAT
Ca_LG_2:8024032	CAAGATGTTTTCCTTCTGTGTTTCAATGTAAAAGCTTTTGAATT TTCCTTGCATGTCAGACATCAGTGCAACTCTTAATTGAAAATTT TGCCTCTTTAAG	G/A	GCATCATATGTTACTACACCATCATTGCACAATTGTTGCAAC TCATAAGCGGCTGTAAATACACATCAATTTTACCTTTTGGATT GTACCTGGAATATTC
Ca_LG_2:8024059	GTAAAAGCTTTTGAATTTTCCTTGCATGTCAGACATCAGTGCA ACTCTTAATTGAAAATTTTGCCTCTTTAAGGCATCATATGTTAC TACACCATCATTG	G/C	CACAATTGTTGCAACTCATAAGCGGCTGTAAATACACATCAA TTTTACCTTTTGGATTGTACCTGGAATATTCAATGACAAGAAC ATATAAGGCATCATC
Ca_LG_2:8244404	GATTGGAAAATTCCGCACATAATGGAAGTGGTTGTGTTGGAA AATGTTTCCAGCAGCGACGACAGACAGCACCAGACGGCGGC GGCGGCAGTAGCAGACG	G/A	GGGCAGCAGCGGTGATTGTGGGCAAAAGTGACAGCAGTCA CAAAAAGGAACGATCATGCTAGAAAAGAGAAACTATGATTAT ATATTCTCAAACTATTGG
Ca_LG_2:8314607	CATAAGCAACAACATCACTAGCCAAACACCAGGCGAAATCTTT AGAAGCAAACACGAAAATCTTATAAGTAAAAGCAGCGAGTGG CTAAAAGACACATCT	T/C	GAATCATGCTCCGTCGTGGCAACGCTCGTTGCTGGTGTTTC CTTCGCCACAACGAATTCTGTCCCCGGCGGAACAACAGATG AAGGCACACTAACACTAG
Ca_LG_2:8314629	CAAACACCAGGCGAAATCTTTAGAAGCAAACACGAAAATCTTA TAAGTAAAAGCAGCGAGTGGCTAAAAGACACATCTGAATCAT GCTCCGTCGTGGCAA	A/G	CGCTCGTTGCTGGTGTTTCCTTCGCCACAACGAATTCTGTCC CCGGCGGAACAACAGATGAAGGCACACTAACACTAGAAGGC AAACCCGCATTTGATAT
Ca_LG_2:8345011	TGGAAGTAGTAAACAAGTTACTAGAATATTGTCCAGATCCAAA AGAAATGTTTGACACGTGTCATGACCGAAATATTCTTCACATT GCTGCAAACTATGA	A/G	AAAATATGAATTGGTACAAAATATATTACAAAGTCAAATTCGT GAACATCATACGATGATAAATCAAAAGGATAAAGAAGGAAAT ACACCTTTGCATTTG
Ca_LG_2:8345045	AGATCCAAAAGAAATGTTTGACACGTGTCATGACCGAAATATT CTTCACATTGCTGCAAACTATGAAAAATATGAATTGGTACAAA ATATATTACAAAGT	T/C	CAAATTCGTGAACATCATACGATGATAAATCAAAAGGATAAA GAAGGAAATACACCTTTGCATTTGGCCGCAAGATCATGTCAT CCCACAAGTGTTTACT
Ca_LG_2:8345050	CAAAAGAAATGTTTGACACGTGTCATGACCGAAATATTCTTCA CATTGCTGCAAACTATGAAAAATATGAATTGGTACAAAATATAT TACAAAGTCAAAT	T/C	TCGTGAACATCATACGATGATAAATCAAAAGGATAAAGAAGG AAATACACCTTTGCATTTGGCCGCAAGATCATGTCATCCCAC AAGTGTTTACTACTTA
Ca_LG_2:8345053	AAGAAATGTTTGACACGTGTCATGACCGAAATATTCTTCACAT TGCTGCAAACTATGAAAAATATGAATTGGTACAAAATATATTAC AAAGTCAAATTCG	G/C	TGAACATCATACGATGATAAATCAAAAGGATAAAGAAGGAAA TACACCTTTGCATTTGGCCGCAAGATCATGTCATCCCACAAG TGTTTACTACTTAATT

Positions	-100 bp flanking sequences	SNPs within *desi* cultivar	+100 bp flanking sequences
Ca_LG_2:8345059	TGTTTGACACGTGTCATGACCGAAATATTCTTCACATTGCTGCAAACTATGAAAAATATGAATTGGTACAAAATATATTACAAAGTCAAATTCGTGAACA	A/T	TCATACGATGATAAATCAAAAGGATAAAGAAGGAAATACACCTTTGCATTTGGCCGCAAGATCATGTCATCCCACAAGTGTTTACTACTTAATTTGGAAT
Ca_LG_2:8345065	ACACGTGTCATGACCGAAATATTCTTCACATTGCTGCAAACTATGAAAAATATGAATTGGTACAAAATATATTACAAAGTCAAATTCGTGAACATCATAC	C/A	GATGATAAATCAAAAGGATAAAGAAGGAAATACACCTTTGCATTTGGCCGCAAGATCATGTCATCCCACAAGTGTTTACTACTTAATTTGGAATGAAAGA
Ca_LG_2:8348680	AAACACGTTGGCATTTGTGAGAGTTTTTGCAGCCACAAAAGGCTGCCTTTGTGAGGGCCTTGCAGCCACAAAAGTTTGCCTTTGTGAGGGCATAGGCCGT	T/C	CACAAAGGACAAACTGATTTTAAAAAATTCAGCCTTTGTGAGGGCCCAAGTTGCCACAAAATCCCTTCTTTGTGAGGGTTTAGGCCGCCACAAAATACCT
Ca_LG_2:8348682	ACACGTTGGCATTTGTGAGAGTTTTTGCAGCCACAAAAGGCTGCCTTTGTGAGGGCCTTGCAGCCACAAAAGTTTGCCTTTGTGAGGGCATAGGCCGTCA	A/G	CAAAGGACAAACTGATTTTAAAAAATTCAGCCTTTGTGAGGGCCCAAGTTGCCACAAAATCCCTTCTTTGTGAGGGTTTAGGCCGCCACAAAATACCTCC
Ca_LG_2:8348692	ATTTGTGAGAGTTTTTGCAGCCACAAAAGGCTGCCTTTGTGAGGGCCTTGCAGCCACAAAAGTTTGCCTTTGTGAGGGCATAGGCCGTCACAAAGGACAA	A/G	ACTGATTTTAAAAAATTCAGCCTTTGTGAGGGCCCAAGTTGCCACAAAATCCCTTCTTTGTGAGGGTTTAGGCCGCCACAAAATACCTCCAGATTAAAAA
Ca_LG_2:8567991	ACATGCACCGGCAGCTGGGTTTTGGGGCAGCAGATAAAACTTCGTTTATGAGAGACATTAAGATGTCAACAATTCACATCTTGGCGTAAAAAAAAAATAT	T/G	CCCCAAGAAAAGGTCTTATCCATTAAGACAAGGTTTCTATCAGTGTTGTCAGATAGCATCTATAGCGGCGCTATAGTGCTATAACATAGCAGAAATTGAA
Ca_LG_2:9885743	GTACTTCAGATGTCTTTTGCATTTTTCTTTGTGAAGATCTAGTTTTCGCTATAGCTGCATTTGGTTTCAGTGATTCAATTTTTGCTTCCTCTTTTGGCTG	G/T	GGAGTCCGGAGTTGTAGGTACTTCAGACATCTTTTCCATTTTTCTCTGTGAAGATCTAGTTTTCGCTATAGCTGCCTTTGGTTTCAGTGATTCAATTTTT
Ca_LG_2:9885752	ATGTCTTTTGCATTTTTCTTTGTGAAGATCTAGTTTTCGCTATAGCTGCATTTGGTTTCAGTGATTCAATTTTTGCTTCCTCTTTTGGCTGGGAGTCCGG	G/A	AGTTGTAGGTACTTCAGACATCTTTTCCATTTTTCTCTGTGAAGATCTAGTTTTCGCTATAGCTGCCTTTGGTTTCAGTGATTCAATTTTTGCTTCCTCT
Ca_LG_2:9885771	TTGTGAAGATCTAGTTTTCGCTATAGCTGCATTTGGTTTCAGTGATTCAATTTTTGCTTCCTCTTTTGGCTGGGAGTCCGGAGTTGTAGGTACTTCAGAC	C/T	ATCTTTTCCATTTTTCTCTGTGAAGATCTAGTTTTCGCTATAGCTGCCTTTGGTTTCAGTGATTCAATTTTTGCTTCCTCTTTTGGCTCAGAGTCTGGAG
Ca_LG_2:9885779	ATCTAGTTTTCGCTATAGCTGCATTTGGTTTCAGTGATTCAATTTTTGCTTCCTCTTTTGGCTGGGAGTCCGGAGTTGTAGGTACTTCAGACATCTTTTC	C/G	CATTTTTCTCTGTGAAGATCTAGTTTTCGCTATAGCTGCCTTTGGTTTCAGTGATTCAATTTTTGCTTCCTCTTTTGGCTCAGAGTCTGGAGTTGTAGGT
Ca_LG_2:9885861	TACTTCAGACATCTTTTCCATTTTTCTCTGTGAAGATCTAGTTTTCGCTATAGCTGCCTTTGGTTTCAGTGATTCAATTTTTGCTTCCTCTTTTGGCTCA	A/G	GAGTCTGGAGTTGTAGGTACTTCAGATATCTTTTGCATTTTCTTCTGAGAAGACCTAGTTTTCCTTATAGTTGTCTCTAGTTTCAGTGTTTCAACTTTTG
Ca_LG_2:9885862	ACTTCAGACATCTTTTCCATTTTTCTCTGTGAAGATCTAGTTTTCGCTATAGCTGCCTTTGGTTTCAGTGATTCAATTTTTGCTTCCTCTTTTGGCTCAG	G/T	AGTCTGGAGTTGTAGGTACTTCAGATATCTTTTGCATTTTCTTCTGAGAAGACCTAGTTTTCCTTATAGTTGTCTCTAGTTTCAGTGTTTCAACTTTTGC

Positions	-100 bp flanking sequences	SNPs within *desi* cultivar	+100 bp flanking sequences
Ca_LG_2:9885863	CTTCAGACATCTTTTCCATTTTTCTCTGTGAAGATCTAGTTTTCGCTATAGCTGCCTTTGGTTTCAGTGATTCAATTTTTGCTTCCTCTTTTGGCTCAGA	A/T	GTCTGGAGTTGTAGGTACTTCAGATATCTTTTGCATTTTCTTCTGAGAAGACCTAGTTTTCCTTATAGTTGTCTCTAGTTTCAGTGTTTCAACTTTTGCT
Ca_LG_2:9885867	AGACATCTTTTCCATTTTTCTCTGTGAAGATCTAGTTTTCGCTATAGCTGCCTTTGGTTTCAGTGATTCAATTTTTGCTTCCTCTTTTGGCTCAGAGTCT	T/C	GGAGTTGTAGGTACTTCAGATATCTTTTGCATTTTCTTCTGAGAAGACCTAGTTTTCCTTATAGTTGTCTCTAGTTTCAGTGTTTCAACTTTTGCTTCCT
Ca_LG_2:10141465	ACATTGTTCATTTTAAGGATATGAGTGCTAGTCAATTACCTCGATAAGTGTTTACCGACTCCTGCTTTAGTTGCATCTCCACCAAAAATTATAGGATATA	A/T	GTTTTCCTTGAAGGTCAAATGTGTTCAAAGAAATACCCTAAACATTAAACAAATATATATTAAAATTATTGTTGACGTTATTATTATAAATAAAAAAAGA
Ca_LG_2:10141471	TTCATTTTAAGGATATGAGTGCTAGTCAATTACCTCGATAAGTGTTTACCGACTCCTGCTTTAGTTGCATCTCCACCAAAAATTATAGGATATAGTTTTC	C/T	CTTGAAGGTCAAATGTGTTCAAAGAAATACCCTAAACATTAAACAAATATATATTAAAATTATTGTTGACGTTATTATTATAAATAAAAAAAGAGTTTCT
Ca_LG_2:10141473	CATTTTAAGGATATGAGTGCTAGTCAATTACCTCGATAAGTGTTTACCGACTCCTGCTTTAGTTGCATCTCCACCAAAAATTATAGGATATAGTTTTCCT	T/C	TGAAGGTCAAATGTGTTCAAAGAAATACCCTAAACATTAAACAAATATATATTAAAATTATTGTTGACGTTATTATTATAAATAAAAAAAGAGTTTCTTA
Ca_LG_2:10141475	TTTTAAGGATATGAGTGCTAGTCAATTACCTCGATAAGTGTTTACCGACTCCTGCTTTAGTTGCATCTCCACCAAAAATTATAGGATATAGTTTTCCTTG	G/C	AAGGTCAAATGTGTTCAAAGAAATACCCTAAACATTAAACAAATATATATTAAAATTATTGTTGACGTTATTATTATAAATAAAAAAAGAGTTTCTTATT
Ca_LG_2:10141494	AGTCAATTACCTCGATAAGTGTTTACCGACTCCTGCTTTAGTTGCATCTCCACCAAAAATTATAGGATATAGTTTTCCTTGAAGGTCAAATGTGTTCAAA	A/G	GAAATACCCTAAACATTAAACAAATATATATTAAAATTATTGTTGACGTTATTATTATAAATAAAAAAAGAGTTTCTTATTCAAGATTCACATCATAAGC
Ca_LG_2:10141497	CAATTACCTCGATAAGTGTTTACCGACTCCTGCTTTAGTTGCATCTCCACCAAAAATTATAGGATATAGTTTTCCTTGAAGGTCAAATGTGTTCAAAGAA	A/G	ATACCCTAAACATTAAACAAATATATATTAAAATTATTGTTGACGTTATTATTATAAATAAAAAAAGAGTTTCTTATTCAAGATTCACATCATAAGCTTA
Ca_LG_2:10141499	ATTACCTCGATAAGTGTTTACCGACTCCTGCTTTAGTTGCATCTCCACCAAAAATTATAGGATATAGTTTTCCTTGAAGGTCAAATGTGTTCAAAGAAAT	T/C	ACCCTAAACATTAAACAAATATATATTAAAATTATTGTTGACGTTATTATTATAAATAAAAAAAGAGTTTCTTATTCAAGATTCACATCATAAGCTTATA
Ca_LG_2:10142007	AATATACATTGTTTTCTGAATCTTTTCCAATTGAGACAGACAATATATCGACCCCATCAGCAATAGCATCGTCAAATGCAGCAAGAATATTTGCATCATT	T/C	GCAACCACTAGACCAACATGCTTTGTAGACAGCAATGCGAGCTGAGGGAGCACCACCTCTTGCTGTTCCTTGTGCAAAGCCTAGCATGCTTGCCTGGCTA
Ca_LG_2:10142008	ATATACATTGTTTTCTGAATCTTTTCCAATTGAGACAGACAATATATCGACCCCATCAGCAATAGCATCGTCAAATGCAGCAAGAATATTTGCATCATTG	G/A	CAACCACTAGACCAACATGCTTTGTAGACAGCAATGCGAGCTGAGGGAGCACCACCTCTTGCTGTTCCTTGTGCAAAGCCTAGCATGCTTGCCTGGCTAA
Ca_LG_2:10142011	TACATTGTTTTCTGAATCTTTTCCAATTGAGACAGACAATATATCGACCCCATCAGCAATAGCATCGTCAAATGCAGCAAGAATATTTGCATCATTGCAA	A/C	CCACTAGACCAACATGCTTTGTAGACAGCAATGCGAGCTGAGGGAGCACCACCTCTTGCTGTTCCTTGTGCAAAGCCTAGCATGCTTGCCTGGCTAACCG

Positions	-100 bp flanking sequences	SNPs within *desi* cultivar	+100 bp flanking sequences
Ca_LG_2:10142017	GTTTTCTGAATCTTTTCCAATTGAGACAGACAATATATCGACCCCATCAGCAATAGCATCGTCAAATGCAGCAAGAATATTTGCATCATTGCAACCACTA	A/G	GACCAACATGCTTTGTAGACAGCAATGCGAGCTGAGGGAGCACCACCTCTTGCTGTTCCTTGTGCAAAGCCTAGCATGCTTGCCTGGCTAACCGGGTTCC
Ca_LG_2:10142018	TTTTCTGAATCTTTTCCAATTGAGACAGACAATATATCGACCCCATCAGCAATAGCATCGTCAAATGCAGCAAGAATATTTGCATCATTGCAACCACTAG	G/A	ACCAACATGCTTTGTAGACAGCAATGCGAGCTGAGGGAGCACCACCTCTTGCTGTTCCTTGTGCAAAGCCTAGCATGCTTGCCTGGCTAACCGGGTTCCC
Ca_LG_2:10142027	TCTTTTCCAATTGAGACAGACAATATATCGACCCCATCAGCAATAGCATCGTCAAATGCAGCAAGAATATTTGCATCATTGCAACCACTAGACCAACATG	G/A	CTTTGTAGACAGCAATGCGAGCTGAGGGAGCACCACCTCTTGCTGTTCCTTGTGCAAAGCCTAGCATGCTTGCCTGGCTAACCGGGTTCCCTGCTGCAGT
Ca_LG_2:11196986	ATAATCGACAATCGAAGTGAGAAAATGCATCCAAAACAGGAGAGACAGTGGTTGTTTCGGAAAACTTTTCTAGCAGCGATGGCAGGCAGCAGCAGCAAAA	A/C	GGCAGCAAACGGCGGCAACGGCAGTAGCAGACGGGGCAGCAGCGACGATTGTGGACGGAAGTGATAGTAGTCAGAAAAAGGAATGATCATGCTAGAAAAG
Ca_LG_2:11764861	ATATACTTAACAGTTTTTCTCTTAATCTCAGATAGCTTTCACATGTTTAGTATATCCCTGCCTAATTTTGGCATACATGGGTGAGGCTGCATTTCTCTCC	C/T	AAGCATCATTATGACATTCAGAGAAGTTTCTACAAAGCCATACCAGGCAAGAATTTACTACTTTAAGAAGCCACAAAAATGTCAATATTATTTATAAGCT
Ca_LG_2:11764913	TATCCCTGCCTAATTTTGGCATACATGGGTGAGGCTGCATTTCTCTCCAAGCATCATTATGACATTCAGAGAAGTTTCTACAAAGCCATACCAGGCAAGA	A/G	ATTTACTACTTTAAGAAGCCACAAAAATGTCAATATTATTTATAAGCTCTCTAGTTTAACTGTGCCACAATTTTTTATGCAGAAGCTGTATTTTGGCCAG
Ca_LG_2:12751879	CTTGCTCTTTATCCTGATGAGCTTGTCTTGCAATGATAGTTTGCAGAGCATCAAAAATACTGGGAATGTTCTTATAAGCTCTTGAGGATTCCTTTTCCAG	G/A	TTTGGTCCTTTTAGAGGGACGTTGATGATCATCAATAGGAGTGCTTTGGGAAGGAGAAACAGAGGTACAAAAAGTGGATAGAATGGCAAAAGTAGTATTG
Ca_LG_2:12751901	TTGTCTTGCAATGATAGTTTGCAGAGCATCAAAAATACTGGGAATGTTCTTATAAGCTCTTGAGGATTCCTTTTCCAGTTTGGTCCTTTTAGAGGGACGT	T/A	TGATGATCATCAATAGGAGTGCTTTGGGAAGGAGAAACAGAGGTACAAAAAGTGGATAGAATGGCAAAAGTAGTATTGATGTGAGGAAGAGAGTTGGGAG
Ca_LG_2:12751917	GTTTGCAGAGCATCAAAAATACTGGGAATGTTCTTATAAGCTCTTGAGGATTCCTTTTCCAGTTTGGTCCTTTTAGAGGGACGTTGATGATCATCAATAG	G/A	GAGTGCTTTGGGAAGGAGAAACAGAGGTACAAAAAGTGGATAGAATGGCAAAAGTAGTATTGATGTGAGGAAGAGAGTTGGGAGCGGAGTGATGGGGGAA
Ca_LG_2:13321366	AGCTAGTTTGTTTTCTCATCTAATTTTTTGTCCGGTGAAGCAGCGTGCTCCAGAACCCCAGGTTAAGCAGCAGCCAAAGGCATTGCCACCACCTAAAGAC	C/T	GAGGAAGAAGAACTGGACGAGGAAGATGAAGATGAACACGGAGAGACCTTATGCGGTGCATGCGGAGAGCATTATGGCACTGACGAATTCTGGATTTGCT
Ca_LG_2:13460400	TCTGCCCCTAGTTCTAATTGTTTTTTATTTCTTTCTATTTTTTGTATGGCAGTATTAACAGTCTCTCCCACATTTTTAACCATCTTTTCAGCCTCCTTGC	C/T	TTTTCACCTTTACAGCTGAAAATTATCCTTGTATACACGTCAAATTTCTAGCCTTCTTTAGCCTCTTTTGCATTTCTCTTTTTTCCCCAAAACAAATGCA
Ca_LG_2:13460411	TTCTAATTGTTTTTTATTTCTTTCTATTTTTTGTATGGCAGTATTAACAGTCTCTCCCACATTTTTAACCATCTTTTCAGCCTCCTTGCTTTTCACCTTT	T/C	ACAGCTGAAAATTATCCTTGTATACACGTCAAATTTCTAGCCTTCTTTAGCCTCTTTTGCATTTCTCTTTTTTCCCCAAAACAAATGCAATTTTTTCCTT

Positions	-100 bp flanking sequences	SNPs within *desi* cultivar	+100 bp flanking sequences
Ca_LG_2:13593546	AAATCACCAACACTAAGCTTTGGTTTTGCGAAACAGAGGCAG GGAAGAAGAAACAGTTCTCGCGAGAAGGAGGAAATGTTGTTC TTTCACTAACACTTGG	G/A	TTTTCTTGAAGCCAAAGAAAGAAAAAGAAAGGAGAAGAAGAA AGGATTCGCGAGCAGGGAGGAAACGTTTCTGCTTTCTTCCT TTTCTTATCCTTGCTTC
Ca_LG_2:13593566	TGGTTTTGCGAAACAGAGGCAGGGAAGAAGAAACAGTTCTCG CGAGAAGGAGGAAATGTTGTTCTTTCACTAACACTTGGTTTTC TTGAAGCCAAAGAAA	A/G	GAAAAAGAAAGGAGAAGAAGAAAGGATTCGCGAGCAGGGA GGAAACGTTTCTGCTTTCTTCCTTTTCTTATCCTTGCTTCTGA TATAACTATGTTTATAC
Ca_LG_2:13593589	GAAGAAGAAACAGTTCTCGCGAGAAGGAGGAAATGTTGTTCT TTCACTAACACTTGGTTTTCTTGAAGCCAAAGAAAGAAAAAGA AAGGAGAAGAAGAAA	A/G	GGATTCGCGAGCAGGGAGGAAACGTTTCTGCTTTCTTCCTTT TCTTATCCTTGCTTCTGATATAACTATGTTTATACATTACTTAC TTACTAAGTAATAT
Ca_LG_2:13593592	GAAGAAACAGTTCTCGCGAGAAGGAGGAAATGTTGTTCTTTC ACTAACACTTGGTTTTCTTGAAGCCAAAGAAAGAAAAAGAAAG GAGAAGAAGAAAGGA	A/T	TTCGCGAGCAGGGAGGAAACGTTTCTGCTTTCTTCCTTTTCT TATCCTTGCTTCTGATATAACTATGTTTATACATTACTTACTTA CTAAGTAATATATA
Ca_LG_2:13744687	TCTCACTCATCTCGGATGTATCTTTGATGATGACCTCCATTGC CTTGAACTCCATCTCGTCCTCTTTTAGTTGTGCCAGTTTTTCTA AGGCTGCTACTTC	C/T	TTTATTCATTGTATCTTGCACAACATTATTAATAAGAGGTGTT TCAACCGCCTTTGCCTTGCCCATTCTTTTTGTTTCCTTTTGTC CCATTAGACGCTCC
Ca_LG_2:13744713	ATGATGACCTCCATTGCCTTGAACTCCATCTCGTCCTCTTTTA GTTGTGCCAGTTTTTCTAAGGCTGCTACTTCTTTATTCATTGTA TCTTGCACAACAT	T/G	TATTAATAAGAGGTGTTTCAACCGCCTTTGCCTTGCCCATTC TTTTTGTTTCCTTTTGTCCCATTAGACGCTCCATTGGTGATGT TGAGTCAATTTCATA
Ca_LG_2:14097659	TGGATAGAATCTTCATTTCATTTTTAATCCACCATTGTCACTCA TGAATAACCTATGTAGGATTGATTAATGAAGCCAAACATGTGC TGCATATATTTCG	G/A	TTTCTGCTTACTCATAGGGTGTTTATGCTCTTCACCTGTCATG ATGCAGGTCTCTATGTTCTTGCTGCTGAATCTGTGCAAATTC CTTGGTTTGATTTTG
Ca_LG_2:15317808	ATTCAACAGTGGTTTGGAACCTTCCAATTTGAATGTCAAACTT CGCACACTTCTCTGTTATGGCCACACGGGAACCGTTTCCAAA TTTTGCGTTCATAAT	T/C	TGGGATCTGTTCTACTGGAAGCTGCAGATCTGCAACAATTTG AGGAGTCACAAAATTGGGAGTAGCACAAGTGTCATCCATCA TCATGACCGGTATGCCC
Ca_LG_2:15620884	CATGGTAAGGAAGAAAGAAGAACAAGCACACTTGGAGAGCG CATTACAATGGATAGTCGTATGTTGCGTTCGAAAGGATAAATC ATTCCTGAAAAGGAAA	A/T	CTATTGATTCTCTCCCAATTGGATGGACCGTAGGTGTGATGA TTTACATCATGGGTGAGGTCTTAGTTTTAAATTCAGGATGGC CCAACTGCGCCAAGGA
Ca_LG_2:15620893	GAAGAAAGAAGAACAAGCACACTTGGAGAGCGCATTACAATG GATAGTCGTATGTTGCGTTCGAAAGGATAAATCATTCCTGAAA AGGAAACTATTGATT	T/C	CTCTCCCAATTGGATGGACCGTAGGTGTGATGATTTACATCA TGGGTGAGGTCTTAGTTTTAAATTCAGGATGGCCCAACTGC GCCAAGGAAAAGAATAA
Ca_LG_2:15620921	AGCGCATTACAATGGATAGTCGTATGTTGCGTTCGAAAGGAT AAATCATTCCTGAAAAGGAAACTATTGATTCTCTCCCAATTGG ATGGACCGTAGGTGT	T/C	GATGATTTACATCATGGGTGAGGTCTTAGTTTTAAATTCAGG ATGGCCCAACTGCGCCAAGGAAAAGAATAAAAGACTAATTT GACTTTTTCATGCATGC
Ca_LG_2:15631359	AGTGGCATCGTTTAAAGTGTCAATAAGTGGCATCATATAATCC AAGTAGTTATTATTGTTTAATATTCTGCTGCAGGTTAAGGTTG GAATCCAAGGGGCT	T/C	GAAGAGTTTGGTGAGTTTGAGGAATGGTTCAATTACAGGCTA TCAAGAGCACGAGAGCAGCACCTGAAACATGTGCTGATTTC CTTGGAAGTTTCATAGC

Positions	-100 bp flanking sequences	SNPs within *desi* cultivar	+100 bp flanking sequences
Ca_LG_2:15632518	TACTCTGCTGGGATTGTACTCCTGCAAATTGCAATACCAACCTTAAAGTCTCCTGTTGCTTTGAAGAATTTCAATTTGGAAATAAAAACATGTGGATATC	C/G	ATTTGAAAAAATGGAGGAGAACACTCGCCTCAGACCCGACTTTCAAATTCTTGATAGTGAATCTGGTAGAGGGTGGGACTTAGCAACAAAGCTTATCTCT
Ca_LG_2:15632561	TAAAGTCTCCTGTTGCTTTGAAGAATTTCAATTTGGAAATAAAAACATGTGGATATCATTTGAAAAAATGGAGGAGAACACTCGCCTCAGACCCGACTTT	T/C	CAAATTCTTGATAGTGAATCTGGTAGAGGGTGGGACTTAGCAACAAAGCTTATCTCTAAGAGAGGTCCCTTAAGAAGAGGACGTTTATCTGCTGCTTCAG
Ca_LG_2:16592886	AGAGATAACTGGAGGTTGGTATCATGTTGATAAGATTTATGGCCGGCAGCAGAGAGTGAAGATTGCAGCGGTGACGACCGGTGAGGTTTCTCGTTGAAGA	A/G	AAGGTTGTTAGATGAAGATTGATGCGGTAGCGGCGGCGGCGAGGTTGCTCGTTAAAGGAAGATTGCTCATTGAAGATTGCAGCGGCGGCGGCGGCGAGGT
Ca_LG_2:17538199	ACTCCAGCAGCTTCGTCATAATGTCAGAAGATGCTTCAACTGGTGCAGATGGTTGGGCACTTGCTTGGGCTTGATCAGCACTGGGTTGTGCATGATCAGT	T/C	AGTAGATGGAGTTCTTGTCCATACTCCATTAATGCGTCGCAGCTGCATTTGGTTCACAATTGATTCACCAAATGTCAGTGTGGTTTTGAGAGATTCTTCA
Ca_LG_2:17568987	TAGTGTGACAACACGCTTTGGAATTTTCTACATATAATCTTCATCAACAACTTCACTATTAAAGTCTTGCAACAATTCTCATCAACACAGATCCAACAAT	T/C	CTTTAGGTTCGATTTTATATTCAATCAATGGCTGCATCACTCAAAGAAATGACTTCTCATTTTGCGAAGTTGGAGAAATTCGATGGATGAAACTTTATTT
Ca_LG_2:17579108	AACTGAGAGGCGTCAAAACGCGCCGTCACACGCTGTGGAGACGGCGGTGCGTGAGATCTACTCGCAAGAGAGATTAGCAGCGTGTGAGAGCGCATGTGAC	C/T	GGCTTTTGGCGACGCGCTTTCGAGCATGACCTGACCTAGAGATGACGATGCTATTCGCTGGAAAATTTGCTGAAAATAGTGGCAGTAACAGCTGCAATAG
Ca_LG_2:17629707	CTCAAAGGCAGCAGCCCAAGTTTCATAATTTGCAGCACCTACCAATTTTTTCAAAAAGAATCAAAGAGTAAAGAAAGTGAAAGCGAAAGCGAAAGATGAA	A/G	GAAGCAGCAAATCCGGTGTCGGACATGAAGTTCTATGTGAGATGAAGAAGTTTGTTGTAAAATCATCAAAAGATCAAAAACACCTCCTAATGTGGCATAG
Ca_LG_2:17629794	AGCGAAAGATGAAGAAGCAGCAAATCCGGTGTCGGACATGAAGTTCTATGTGAGATGAAGAAGTTTGTTGTAAAATCATCAAAAGATCAAAAACACCTCC	C/T	TAATGTGGCATAGAGGGTTCTGCAGTGGTCAGCACAAGAAGCTGCTTGCTGGAGTGGTGCTGCGTATGTTTCTGGTGGTGGCGCATGGATCCCATGCACA
Ca_LG_2:19565764	TGGCTAGTGGAAGGAATGACGCTGCAATTGTTGAGGCTCTAGAGTCCATGACGAGAGTTAGAGTACAAGCTCTCATGGCTTTGGCTGAATCACAAGCTAT	T/C	TGCGCAAGCTGCTACTCAAGTTGCACAAGTTGCTGCTCAAGCTACTACTTACGCTGGTGGTCAAGCAAATGTGCAAATAAATGAGTTCATGGTGATGGAT
Ca_LG_2:19686028	TTTTTGCGGCGAAAGCAGTCACAAAATCCAACGGTAACTAAAGCCTTTGTGGCAGTTTTTGTGGTCAGAAAACAAGGAATTTGTGGTGGCCTAAGGAGCC	C/T	AGAACTAGCAACGGTCCATTGGGCATTTGTGGTGGCCTTTGCAGCCCCAACGGGTAACGGTCCACTGATTTTGTGGCGGTATTTACCCTCATAAATTGGT
Ca_LG_2:20527624	TGATCGGTTCCCCAAGGAAGAAACGACATGACTGAGGCAATCCAAGCCATGAATGCAATAGCCGCAGCAATGGCCCAACAAGCTGCGATCCAGGCTCAAT	T/C	GAGATGCACAGAGGGATCAGAGGGATGAAGCAGCCAGTGCAGCAAGAGCATTGAATGAATTTTGTCGACAAGATTCGCCTAAATTCAAAGGGGAACATGA
Ca_LG_2:21042303	TGGAAAATCCTACAATCAATTAGCAGAGGAAACAGGACTCACAAACGTCTATGTTGCTCAGCTTCTAAGAAGACAAGCTCAGCTCAAACCTGACACTGTG	G/T	CCTAAACTCCGGGCAGCACTGCCCAACCTGCCCGAACAACTTATCCATGAGATGATGAAACCGCCTTTGAGGTCTTATGACCCTAATCTCATCCAAGACC

Positions	-100 bp flanking sequences	SNPs within *desi* cultivar	+100 bp flanking sequences
Ca_LG_2:21042312	CTACAATCAATTAGCAGAGGAAACAGGACTCACAAACGTCTATGTTGCTCAGCTTCTAAGAAGACAAGCTCAGCTCAAACCTGACACTGTGCCTAAACTC	C/T	CGGGCAGCACTGCCCAACCTGCCCGAACAACTTATCCATGAGATGATGAAACCGCCTTTGAGGTCTTATGACCCTAATCTCATCCAAGACCCCACTATTT
Ca_LG_2:21224300	AGAGAATAAACTCTCAATTTTTTACTCAATCAAATTGTAATTTTATTCAATCACAATTCATATACAAGTAGCATGCCATAACTTTTTGTCTTCCTAAACA	A/G	TAAGACTTAAAACGCCAATTGAATGTCTAATTGATCACTTTAATGGCTACACTTCAATTGGAAATTAACTAGTGCCATAAAGTGTAAGTTACATTTCTCC
Ca_LG_2:21224326	CAATCAAATTGTAATTTTATTCAATCACAATTCATATACAAGTAGCATGCCATAACTTTTTGTCTTCCTAAACATAAGACTTAAAACGCCAATTGAATGT	T/C	CTAATTGATCACTTTAATGGCTACACTTCAATTGGAAATTAACTAGTGCCATAAAGTGTAAGTTACATTTCTCCAACAATCATATCTTGCGTAAAATAAA
Ca_LG_2:21224364	CAAGTAGCATGCCATAACTTTTTGTCTTCCTAAACATAAGACTTAAAACGCCAATTGAATGTCTAATTGATCACTTTAATGGCTACACTTCAATTGGAAA	A/G	TTAACTAGTGCCATAAAGTGTAAGTTACATTTCTCCAACAATCATATCTTGCGTAAAATAAACATACAACGGCTAGTATTATACCTTTCATCTAAAAAAC
Ca_LG_2:21224392	CCTAAACATAAGACTTAAAACGCCAATTGAATGTCTAATTGATCACTTTAATGGCTACACTTCAATTGGAAATTAACTAGTGCCATAAAGTGTAAGTTAC	C/G	ATTTCTCCAACAATCATATCTTGCGTAAAATAAACATACAACGGCTAGTATTATACCTTTCATCTAAAAAACTTAAAATGCTACTAATGAATAAAAAAAG
Ca_LG_2:21224394	TAAACATAAGACTTAAAACGCCAATTGAATGTCTAATTGATCACTTTAATGGCTACACTTCAATTGGAAATTAACTAGTGCCATAAAGTGTAAGTTACAT	T/C	TTCTCCAACAATCATATCTTGCGTAAAATAAACATACAACGGCTAGTATTATACCTTTCATCTAAAAAACTTAAAATGCTACTAATGAATAAAAAAAGGA
Ca_LG_2:21663534	ATGACATCTTAAGGCCTAGATGTTCTTGTATTGCAGCCACATCCTGAGCGACGGAATCAAGCGATGCCTGGAGAGTCTTGAGAGATGCTTCTACTCTTGT	T/C	ATTATGAGCAGCTTGAATCTCCATGAATTCCAGCAGCTTTGTCAAGACCACAGAATGTGCTTCGACTGGCTCAGCTTGCTCTGCAGTTGGCTGCGCTTGT
Ca_LG_2:22428916	GGAATCCAGTTGAGTAGTCAAAAACTGAGGTTTCAAAGTTCATTCCAAATATTCCTGCCACGGTGGCAAATACTGCAGCTACCAATGTAGCTGCTGTAAA	A/G	AAGCAACTCAAACTGTATCAGGCGGTTTTGAATATTTCCCTGTAATAGATAGTGTGTCAAGTGATGGTCCCTGATTTATATAGTTTAGAATATATCAAAC
Ca_LG_2:22661498	CAATAGTTGTTTCGGAAAACTTGCTGGAAAACTTTTCCAGCAGCGACAGTAGATGGAAAGCAGCAGGAGACGGCGGATCTTGAAGGTAGTAGGTGTAACT	T/C	GTAGCCCGAAACTAGAATACGAGGGCAGATTTGAAATCGCGAGGTTGAGGCGTGATTGACTGACCAAGCAGCAACTGAGAGGCGTCAAAATGCGCCAAAA
Ca_LG_2:22661503	GTTGTTTCGGAAAACTTGCTGGAAAACTTTTCCAGCAGCGACAGTAGATGGAAAGCAGCAGGAGACGGCGGATCTTGAAGGTAGTAGGTGTAACTGTAGC	C/G	CCGAAACTAGAATACGAGGGCAGATTTGAAATCGCGAGGTTGAGGCGTGATTGACTGACCAAGCAGCAACTGAGAGGCGTCAAAATGCGCCAAAATGTTG
Ca_LG_2:22661547	TAGATGGAAAGCAGCAGGAGACGGCGGATCTTGAAGGTAGTAGGTGTAACTGTAGCCCGAAACTAGAATACGAGGGCAGATTTGAAATCGCGAGGTTGAG	G/A	GCGTGATTGACTGACCAAGCAGCAACTGAGAGGCGTCAAAATGCGCCAAAATGTTGGAAAACAGAAACTATAATTATATTCCCTAATTGTTGGGCTCTGA
Ca_LG_2:23435161	CGCTTCCTCTAGAACTTTTTTATCCTTGTTCAGAAAAATATTGAAAGCCATAACTTTCTCTTCTTTCCTTGATCCCACGAAGAGATGACTTAATGTCTTA	A/G	AATTTTAGACGCTATTTGATGGGAAGTGATCACATTTGAGATGAACTGACCAATCTTATGGTGTAATCACAACAATTATTTTTGTAGATACATTGCATTT

**Positions**	**-100 bp flanking sequences**	**SNPs within *desi* cultivar**	**+100 bp flanking sequences**
Ca_LG_2:23522965	CAAATCCAAATCTCCAATAACATTAGCAATTAACATTGTTCAAACTCATGGGGTATAGTTTTAGTATCCATCGTTTTTCCCTTTTTGAAGGTTTGATTCC	C/T	TCTTTATAGTTGTTACAATAAAATGAGAAAACTAGTATGCATCATTTTCCTTTGTTTTAGTGTCGACATCATTATTAGCTAGATCTGCTGCCGTGTTTCG
Ca_LG_2:25186109	CTTTCTTGCATTTATAGTTTTGAACTTTGACAGGTAAGTCAAACAAAAGCTGATAAAATTACAATAGCACTCACATTGTACCACCTGCAGCAGGACCAAT	T/C	AGCTTTGAAAAAAGACATAGCTGTCATAGAAATTCCATTAGCTGCCCCTCTTTGGTGTTGTTCCTGTTTTCCAAATCAACATGTCATATACATATGTCAC
Ca_LG_2:25186117	CATTTATAGTTTTGAACTTTGACAGGTAAGTCAAACAAAAGCTGATAAAATTACAATAGCACTCACATTGTACCACCTGCAGCAGGACCAATAGCTTTGA	A/C	AAAAAGACATAGCTGTCATAGAAATTCCATTAGCTGCCCCTCTTTGGTGTTGTTCCTGTTTTCCAAATCAACATGTCATATACATATGTCACAATTCCAA
Ca_LG_2:25204180	AGTGTAGTCGGGAGTTAGTTTTGGAATTATTCAAAGTGGTGTTAACATTCTTCTTGATGATGATTTTATAGGAAAACCATGCCGCCAAGAAGGAATGACG	G/C	CTCCAAGAGTCAGTGTCAATAGGGACGATCAAATGGCGGATGCTATGAATAACATGGCTGCTTCTTTTGCTGCCCAAACTATTGCAAAGACTCGACGGGA
Ca_LG_2:25204221	TTAACATTCTTCTTGATGATGATTTTATAGGAAAACCATGCCGCCAAGAAGGAATGACGCTCCAAGAGTCAGTGTCAATAGGGACGATCAAATGGCGGAT	T/A	GCTATGAATAACATGGCTGCTTCTTTTGCTGCCCAAACTATTGCAAAGACTCGACGGGATCTAGAAAAGAGGGAAAGAGAGATCCGTGCTGCTGAGTCAA
Ca_LG_2:25204235	GATGATGATTTTATAGGAAAACCATGCCGCCAAGAAGGAATGACGCTCCAAGAGTCAGTGTCAATAGGGACGATCAAATGGCGGATGCTATGAATAACAT	T/C	GGCTGCTTCTTTTGCTGCCCAAACTATTGCAAAGACTCGACGGGATCTAGAAAAGAGGGAAAGAGAGATCCGTGCTGCTGAGTCAAGATGATTAGAAGAT
Ca_LG_2:25204246	TATAGGAAAACCATGCCGCCAAGAAGGAATGACGCTCCAAGAGTCAGTGTCAATAGGGACGATCAAATGGCGGATGCTATGAATAACATGGCTGCTTCTT	T/G	TTGCTGCCCAAACTATTGCAAAGACTCGACGGGATCTAGAAAAGAGGGAAAGAGAGATCCGTGCTGCTGAGTCAAGATGATTAGAAGATTTTCGTTGTTA
Ca_LG_2:25204269	AAGGAATGACGCTCCAAGAGTCAGTGTCAATAGGGACGATCAAATGGCGGATGCTATGAATAACATGGCTGCTTCTTTTGCTGCCCAAACTATTGCAAAG	G/C	ACTCGACGGGATCTAGAAAAGAGGGAAAGAGAGATCCGTGCTGCTGAGTCAAGATGATTAGAAGATTTTCGTTGTTACAATCCTCCCAAATTTAAGGGTG
Ca_LG_2:25204274	ATGACGCTCCAAGAGTCAGTGTCAATAGGGACGATCAAATGGCGGATGCTATGAATAACATGGCTGCTTCTTTTGCTGCCCAAACTATTGCAAAGACTCG	G/A	ACGGGATCTAGAAAAGAGGGAAAGAGAGATCCGTGCTGCTGAGTCAAGATGATTAGAAGATTTTCGTTGTTACAATCCTCCCAAATTTAAGGGTGATGAA
Ca_LG_2:25261328	TTAGCTTAGGATTTTAATTAAGGGTTTTATTTATTCTCCCTTCCTTATTGTAATATTGTTGTTGCAATGAAATTTATGTATATGAAATAATTGTGATTTT	T/C	TTTTGGGTCAATGTGAATTGTTCGATTGTAAATAAGTTATTTCTGTCGAAGCCATTTTAATGATGCAACAAAAATTCAAATCTTCATTTTTGAAACATAA
Ca_LG_2:25261347	AAGGGTTTTATTTATTCTCCCTTCCTTATTGTAATATTGTTGTTGCAATGAAATTTATGTATATGAAATAATTGTGATTTTTTTTGGGTCAATGTGAATT	T/G	GTTCGATTGTAAATAAGTTATTTCTGTCGAAGCCATTTTAATGATGCAACAAAAATTCAAATCTTCATTTTTGAAACATAATTTTACAATAAAATATTTT
Ca_LG_2:25261360	ATTCTCCCTTCCTTATTGTAATATTGTTGTTGCAATGAAATTTATGTATATGAAATAATTGTGATTTTTTTTGGGTCAATGTGAATTGTTCGATTGTAAA	A/G	TAAGTTATTTCTGTCGAAGCCATTTTAATGATGCAACAAAAATTCAAATCTTCATTTTTGAAACATAATTTTACAATAAAATATTTTACTTTGATTTAAA

Positions	-100 bp flanking sequences	SNPs within *desi* cultivar	+100 bp flanking sequences
Ca_LG_2:25419006	ATTTTATTGGTTTTCCGGAACAAGTACAAAGGAAAAATTATGAAAGCAATGTTATCGTTGATATAATTCAAATATTCTTGCTGCATTCGATGATGCAATA	A/T	GTAGATGGGGTTGATATATTATCAGTCTCAATGGCATCAGAGTCACCGCCTCCCATTTTAGAGATGGAATTTCCATTGGAGCATTACATGCTATGCAACG
Ca_LG_2:25419008	TTTATTGGTTTTCCGGAACAAGTACAAAGGAAAAATTATGAAAGCAATGTTATCGTTGATATAATTCAAATATTCTTGCTGCATTCGATGATGCAATAGT	T/C	AGATGGGGTTGATATATTATCAGTCTCAATGGCATCAGAGTCACCGCCTCCCATTTTAGAGATGGAATTTCCATTGGAGCATTACATGCTATGCAACGTG
Ca_LG_2:26761243	TATTCTTTTCATTACCAAAACCAAGTTGTTTATCATGGAAACTGTGTTGAAGATGTTCATGAAGTTTGTTATGTTATTTGTTGTTGTTGCAGCTGCTATA	A/G	GTTGGTAACGTACAAGGTCTCCCACCTTGTTGCCCAGCAGATAAATCATTAGAATGTCAACCAATTCACTGTCCTAAGCTCCCATGTTGCATCCCTCCAA
Ca_LG_2:26761252	CATTACCAAAACCAAGTTGTTTATCATGGAAACTGTGTTGAAGATGTTCATGAAGTTTGTTATGTTATTTGTTGTTGTTGCAGCTGCTATAGTTGGTAAC	C/T	GTACAAGGTCTCCCACCTTGTTGCCCAGCAGATAAATCATTAGAATGTCAACCAATTCACTGTCCTAAGCTCCCATGTTGCATCCCTCCAACCAGTGACG
Ca_LG_2:26761268	TTGTTTATCATGGAAACTGTGTTGAAGATGTTCATGAAGTTTGTTATGTTATTTGTTGTTGTTGCAGCTGCTATAGTTGGTAACGTACAAGGTCTCCCAC	C/T	CTTGTTGCCCAGCAGATAAATCATTAGAATGTCAACCAATTCACTGTCCTAAGCTCCCATGTTGCATCCCTCCAACCAGTGACGTGAATCAGTAAAATTC
Ca_LG_2:26839927	ATTATAGAAGATCCATGAGAGTTTGCAGCAATGTCCTTAAGGCCTTGCATGTGTCTTAGTTGCCTTGGACTCACGTCGTCAGAGACATTGTGTCTGGCCG	G/A	CAGCTACATTACAATCTTCAGCTGACAAATTTGGAGGAGTTGAGCAATTAATATATTGCTTTTGTATGCAAAAAGAAGCTGCTTCCCCAAAAACTGCAGT
Ca_LG_2:26873511	TATAATATAATGTGGCCATAAAATTTATTTTCTTTATAAAATTTCATCAGGAATACTTGCAGAATTTCCTATGAATCTAATAAACACAAACTTTGCTGCC	C/G	GAGTTTATCTTCGGAAAATTTCCGCATATAGTTCTTGGAAAAAGCTAGTGTTTGCTGCGGATATACTGTCGTATGTGAATCCAAAGAAAACAAAAAAAAT
Ca_LG_2:28581416	TGTGGTATTATGGAGCTGCTTGTTATGATCCAAAATAGCACATGAAGCATTACACTTAATAAAAGAAAATCATTTGAGGAAATGGGACAGGTGGTTTTAG	G/A	ATTCAATTATTCCAGGAGTCTATCAATAATTGTGCAGCTTTTGACCAAGTTATCATTCTGAAATCTCCTCCCTCTGTACCTTACTCTTGCTCTCTCTGTT
Ca_LG_2:29298268	TTCAATTTTTGGGCTAACACGTGCATAGATTTTAAACCCTCCTATGTTTGTGTCAATTCTGCAGGGATAAGCTAGCAGAATTGATAGCCAAACAATCTGA	A/C	ATCTCATGATATAGCTGCAGCTGTGGAAAGCAACAAAATTCAATGAATCTTTGAAATAGTTAGTAAATAAGCTTATAAGAACTGTAAATTTAAGAATAGC
Ca_LG_2:29951000	GTTTTAATAAGGACTTTGGCCTACTCCATTTTAAGTATTTTAATTTGCTACTTTTAGCACCTAGGTAATTGCTAGCTGCACCCCCCACCACAAATTAATG	G/A	ACCAGCATCGCAGTCTTTTTCCTGAGGCAATCTTCTTCCACTCTAAACGATGCCGCCATTGAAATTCTCACACATCTCTCTCCTCTTCATTTCGATGAAC
Ca_LG_2:29951011	GACTTTGGCCTACTCCATTTTAAGTATTTTAATTTGCTACTTTTAGCACCTAGGTAATTGCTAGCTGCACCCCCCACCACAAATTAATGACCAGCATCGC	C/T	AGTCTTTTTCCTGAGGCAATCTTCTTCCACTCTAAACGATGCCGCCATTGAAATTCTCACACATCTCTCTCCTCTTCATTTCGATGAACTATTTAAAGGA
Ca_LG_2:30430402	ACATCATCACTAACCTTAGTTCCAACCTCAAAATTACCAATACCCTTTTCGAATTGCGAATCCTCGTCAGTCGCAGCACCACCATCCTCGCCGGCGGTGG	G/A	AAAAGGGTCTTGTGGGGAGGGTTCTATCACCGGAAAGGTTTAAACTTTGTGAGAAACTAGTGATACAACGAGGGTTCAACAAATGGGTCGAAAAGGGACG

Positions	-100 bp flanking sequences	SNPs within *desi* cultivar	+100 bp flanking sequences
Ca_LG_2:30430828	GTTTTTTAGTTGAAATTTGAAAATTTTGAAGAATGGAATTGAAGAAGAAAAAGATGGGGTTTAGGTGAATTGAAAATTTATTGGTGAAGGAGAGTGAAGG	G/A	GAGGGAAAAAAGTTTGGTTAGGGTTAAAGTGTCAACAAGGGTTGGTTGATTATGACATGTGCCGCCATTGCAAAGTCATTGTTTTTTTGTGCATTTTTTG
Ca_LG_2:30430860	ATGGAATTGAAGAAGAAAAAGATGGGGTTTAGGTGAATTGAAAATTTATTGGTGAAGGAGAGTGAAGGGAGGGAAAAAAGTTTGGTTAGGGTTAAAGTGT	T/C	CAACAAGGGTTGGTTGATTATGACATGTGCCGCCATTGCAAAGTCATTGTTTTTTTGTGCATTTTTTGGTGTGGGAGGTGCTAACTACACCCTTTATTTT
Ca_LG_2:30430867	TGAAGAAGAAAAAGATGGGGTTTAGGTGAATTGAAAATTTATTGGTGAAGGAGAGTGAAGGGAGGGAAAAAAGTTTGGTTAGGGTTAAAGTGTCAACAAG	G/T	GGTTGGTTGATTATGACATGTGCCGCCATTGCAAAGTCATTGTTTTTTTGTGCATTTTTTGGTGTGGGAGGTGCTAACTACACCCTTTATTTTGATATGC
Ca_LG_2:30430879	AGATGGGGTTTAGGTGAATTGAAAATTTATTGGTGAAGGAGAGTGAAGGGAGGGAAAAAAGTTTGGTTAGGGTTAAAGTGTCAACAAGGGTTGGTTGATT	T/A	ATGACATGTGCCGCCATTGCAAAGTCATTGTTTTTTTGTGCATTTTTTGGTGTGGGAGGTGCTAACTACACCCTTTATTTTGATATGCACACCGCTACTT
Ca_LG_2:30603825	AAACAATACACTAAAAAAATAATAATTTATCCTTTCTCAAAATATAATCATAGTTTTAAAATTGCGGCCACAATATATAATTTATGCAATATCTACGACG	G/C	GCAATTGTAGCTGCATTATTCTCATTTTGTAAATGTTCTGCAGCGTAGCTGCAACTACAATTTAAAATTATTGATAGATGAAATGGTAGAATTTACGAAT
Ca_LG_2:30603876	AGTTTTAAAATTGCGGCCACAATATATAATTTATGCAATATCTACGACGGCAATTGTAGCTGCATTATTCTCATTTTGTAAATGTTCTGCAGCGTAGCTG	G/A	CAACTACAATTTAAAATTATTGATAGATGAAATGGTAGAATTTACGAATTGAAATTCTCACCTGTGCCTTGTGATGATGTTGATGATGAAGCAGGCATAG
Ca_LG_2:31287895	ACAAACGCGCGGCCGCCTCTGCTCCTCTGCTCGCTGCGGGCCTGTGGCCGCCTCTGTCTGACTGCCTCCATGGCCGTTCATCGCTCTCGCCTCTCTGTTT	T/C	GCCGCAGATCGCGTTGCTGCTTTGTTCAAAAGGTTCGGATTTTTATTTTGTTAGTGCTGATTTTTCTTAGTTAGTGCTGATTTTTGTTAGTTTTGATTTT
Ca_LG_2:31336725	TAGCCTAATGGTATGTCTTTTCTATTCTTGTATTTTGGTTAAAAATGGCTCAAGCACAAGATTTTATGTGATGGGCATATATGCAGCACATAATGATTTA	A/C	ACTAAATAACAGGTAGAGAAAACCAAAAGAAAGGTGGAGTAACACCTTTTTAGCCCGCTCAAACCTGACTTCTATCAATAATTTTTTTATGAATTTAATC
Ca_LG_2:31336739	GTCTTTTCTATTCTTGTATTTTGGTTAAAAATGGCTCAAGCACAAGATTTTATGTGATGGGCATATATGCAGCACATAATGATTTAACTAAATAACAGGT	T/C	AGAGAAAACCAAAAGAAAGGTGGAGTAACACCTTTTTAGCCCGCTCAAACCTGACTTCTATCAATAATTTTTTTATGAATTTAATCGTCCATTAACCTTC
Ca_LG_2:31336780	ACAAGATTTTATGTGATGGGCATATATGCAGCACATAATGATTTAACTAAATAACAGGTAGAGAAAACCAAAAGAAAGGTGGAGTAACACCTTTTTAGCC	C/T	CGCTCAAACCTGACTTCTATCAATAATTTTTTTATGAATTTAATCGTCCATTAACCTTCATCTCTAGTTCTTAATATTACTGCTGCCAGGCGTTGGTGTT
Ca_LG_2:31336781	CAAGATTTTATGTGATGGGCATATATGCAGCACATAATGATTTAACTAAATAACAGGTAGAGAAAACCAAAAGAAAGGTGGAGTAACACCTTTTTAGCCC	C/T	GCTCAAACCTGACTTCTATCAATAATTTTTTTATGAATTTAATCGTCCATTAACCTTCATCTCTAGTTCTTAATATTACTGCTGCCAGGCGTTGGTGTTT
Ca_LG_2:31821808	AATTGACATGACATCGATGAGTCCGTTGGCTCTTAGTTCCTCATCTTTGCCTATGTCAACTGTTTCAACACCTCCCATGTCTCCCTTGGCAGCTTCATCG	G/A	TCTCCCAAGAGTGGAAACTTGTGGCAGAACAAACTAAACTTCACTCCACCATCATTGCAGCTCTCAGGTAGCCGCTTGAAGAGTGCTTTGAGTGCCAGGG

Positions	-100 bp flanking sequences	SNPs within *desi* cultivar	+100 bp flanking sequences
Ca_LG_2:32056193	CCCAATAATACCATCTTGTAATAGCAAGATACCTCGTGTGCAGTGTAAATCTCAGTGTTAAAACCAATCTCTTCCCCATGTCCATTAGTACCAAAGCCCC	C/T	TAGGTTTTCCAAAATAGATACCTGAATAACAAACAAAGCAGCAGCAAGACTGAGTTAGGTGAACTAGCTTCCAATATTATCAATCAAATGAAATGAATGA
Ca_LG_2:32056215	AGCAAGATACCTCGTGTGCAGTGTAAATCTCAGTGTTAAAACCAATCTCTTCCCCATGTCCATTAGTACCAAAGCCCCTAGGTTTTCCAAAATAGATACC	C/A	TGAATAACAAACAAAGCAGCAGCAAGACTGAGTTAGGTGAACTAGCTTCCAATATTATCAATCAAATGAAATGAATGAAGAGTTAATATTCAAAATTTTA
Ca_LG_2:32808956	AAACAGGGAGAGATAGAGAGAGAAAAAAGTACCTGTGCAGAAGACCCGAAAGCGTTTCTTATTATGTGCAGCCAGTTTAAGAACTTCAAAGACAACTCTA	A/G	GAAAAACCATGAACCAAAATGGTACAGCCATCAAATATAAAATCTTGACTAAGCATTGCAATAATCTTGCGAGCCTGCAAAAAATATAGGAATAATCAAT
Ca_LG_2:32863801	AAGGCGAAGGGAAATGAGCCGAGAAGTCAATGCACCAGATCCCGGTCCTTTAGCCACCTAAGAAAATGGAGTTGAAGGAAAGCAGAATCATACCGAACAA	A/G	CCAAAGCCATTAAATTCTGTTTGCTTTGACAGCAGCTTAACCAAAAGGGGAGGTCTCGCTCTTTTTGCTCTTCTATAGGATAGGATCTTGGGGGAGACTT
Ca_LG_2:32863813	AATGAGCCGAGAAGTCAATGCACCAGATCCCGGTCCTTTAGCCACCTAAGAAAATGGAGTTGAAGGAAAGCAGAATCATACCGAACAACCAAAGCCATTA	A/C	AATTCTGTTTGCTTTGACAGCAGCTTAACCAAAAGGGGAGGTCTCGCTCTTTTTGCTCTTCTATAGGATAGGATCTTGGGGGAGACTTTCTAATGGCTTT
Ca_LG_2:32863861	AGAAAATGGAGTTGAAGGAAAGCAGAATCATACCGAACAACCAAAGCCATTAAATTCTGTTTGCTTTGACAGCAGCTTAACCAAAAGGGGAGGTCTCGCT	T/C	CTTTTTGCTCTTCTATAGGATAGGATCTTGGGGGAGACTTTCTAATGGCTTTGATAGTTTAGAGTTGGACTTAGACTTTCCGAATCTTCTTTGGAAGATA
Ca_LG_2:33232664	CTTGATGCATTTAGGACTGCTGCTATATACTTGTTATTAGGACTGCTTCTATTTGGTCTGCTTGTTATTTGGACTGCTGCTATATTTTGGACTGCTATAT	T/C	TTTGGACAACTGCTATATTTACTTGTTAGGACTGCTGCTATATACTTGATGCATTTAGGACTGCTGCTATATACTTGTTATTTAGGACTACTATATACTT
Ca_LG_2:33232754	ACTGCTATATTTTGGACAACTGCTATATTTACTTGTTAGGACTGCTGCTATATACTTGATGCATTTAGGACTGCTGCTATATACTTGTTATTTAGGACTA	A/G	CTATATACTTTATATTTAGGACTACTGCTATATTTACTTGTTAGGACTGCTACTATATACTTGATGCATTTAGGACTGCTGCTATATACTTGTTATTTGG
Ca_LG_3:23775	TTCTATACAATGTGGTACTTTAGTATTTATAGAGTTGAAAAATACTCAAAATGTGAGATCTTTTCAATGTGGGACTTCAATGCGCTACTTCACTTCCTCC	C/T	GAAATCACCTTCTCAAGGTATCTATCTCTTCCGATCTGATCTGTTTTCTCTCTCTCTCTAGTATTATGCACTTGTAATGTAGTTAGGTTACTCAATGTTA
Ca_LG_3:493207	GCTCTTGAAATCGTTCCTTTCGTTCTTGGTCCTTGCTTGTTTCGTGATTACGTTGCGAACTCTCAATCATAGTTGCATGCGGTTGCTTGTTCGACTCTTG	G/T	GTTTCATCTTCGCGATTCGTTATTGTCTTTTACAATTTTTCGGTGTTGAGATTGTGCTTCTGGGTTGTTTCTCGCTGCTGCTCGATCGTGATTCAGGATT
Ca_LG_3:831538	AAATGCTAAACCAAATCTAAAAAATAAAAATAAAAAAATCTAGCAGCAAAAAAAGAGTAAAAGAGAGATCCACTAACACTGACGGTTACGTTACGGCCTC	C/T	CAAAAATCTAGCAGATTGGGCAAAATGGAAATAAAAGTTAGTGTTGGAATTCAATAAAATTTCAAGCAGCAAATAATATATAAGTAACTAGGATGAAACA
Ca_LG_3:870976	GTACAGAAAAACTGATATTTGGGCTTGTGTTTGTTAGGTAAATGAGTTTGTCGATAAGTCGTCGGTAGATGGATTGATCTTGATAGGAGTACCATTGGCG	G/A	TTGCTGAGTTTAGTGTCTTTCATCATTGGAGTATTGGCAAGTTTGACAGCAAGCAAACCGGTGTCAGATAATAAATCTAGAGTGTATTTTGTTTGACATA

Positions	-100 bp flanking sequences	SNPs within *desi* cultivar	+100 bp flanking sequences
Ca_LG_3:2643898	TGCAGAAGAGACAGCTACGTGAGACTGTGCGCTATATGGAGCTGATGTTGTAATTAATATAAAAGGTTAATGGGTCGGTGTCACAGGTCATGACTGCAGC	C/T	GGTGGAGCCAGCTTTGGATACAGAGGATAAGGCTAGCTGCTGATTTTGGTTCTGAGTTAGCAATGAATCCGTACCTGGTGCTGTGTTTTCTGTCGTGTGT
Ca_LG_3:2643914	ACGTGAGACTGTGCGCTATATGGAGCTGATGTTGTAATTAATATAAAAGGTTAATGGGTCGGTGTCACAGGTCATGACTGCAGCGGTGGAGCCAGCTTTG	G/A	GATACAGAGGATAAGGCTAGCTGCTGATTTTGGTTCTGAGTTAGCAATGAATCCGTACCTGGTGCTGTGTTTTCTGTCGTGTGTTGCTCAGGTGGTTAGG
Ca_LG_3:2722825	TAACCCGGAGGACTTTTGGGGGAGACACATAAACTTGTTAGAGGTGTTAGTCCGAAATCTGATATGCTGCCTTGAAGATCTATTGAGAGAAGCACATTGG	G/A	ATGATTTTATATTGCCATGGACAAGTTTCCTTCCATTGGCTGAGTGGATATGAGCAATGCCCTTAGCAGCTCCTGCCATGATCTTCAATCTGGAATCCCA
Ca_LG_3:2722826	AACCCGGAGGACTTTTGGGGGAGACACATAAACTTGTTAGAGGTGTTAGTCCGAAATCTGATATGCTGCCTTGAAGATCTATTGAGAGAAGCACATTGGA	A/T	TGATTTTATATTGCCATGGACAAGTTTCCTTCCATTGGCTGAGTGGATATGAGCAATGCCCTTAGCAGCTCCTGCCATGATCTTCAATCTGGAATCCCAA
Ca_LG_3:2793667	TGCAGCTGTGGCTAGCTGCATATTTGGAGCCACTGATAGCTGGCATGGTTCTCTTGCAGCAGCTGCCATAATTGGATTTATGGGTCCGATATCAGCAATC	C/T	GGAGCTATTCTCAGTGGTATTGATTATAGTGGTCTTGATCACATAATGGTTCTTGCCTGTGGTGGATTAATTCCTAGCTTCGGAAGTGTAGTTAAGAGAT
Ca_LG_3:3253166	CCAAAGCAACTGGATAATCAAAAAGCATAATCACTAAACAGAAAACATAATCATAAAAACATAATCATTCTCCCCCTGAATTTGGCAGCAGCAAAAAGAA	A/C	ATAATAACAGATTTAATCTGAGGAGGCTGGGTCATCTGATGAGGATGAAGGAGATGGAACATCAGAGTTTGGCTGGGCTGGTAGCTCCGGAGGATGATCT
Ca_LG_3:3375551	CCCTTCCCATCCTACGGTTGAGAAATGATTTTGTGTTGCTTCGACGCTTTTAAGAGACATAGCTTTGGAAAAAACGTATTAGTAGCTTATATGCCGTGGA	A/G	AAGGTTACAATTCTGAAGATGCTGTACTTAGTGAGCGTTTGGCCTATAAAGATATTTATACTTTTTTCACATAACGAAATATGAATTACTAATAAAATTC
Ca_LG_3:4133484	GAACACAAAATGAAGGATAGTAGTAGGTTTTGGTACAAAAGATGCTTTAGTGATATTTGATGGTCTGAACAAGGAACTCTGTGTGGGGATCCATCTTGTA	A/T	GTTATTCGCATTGTTCACCTTGGTTGAAATCTTGTTGGTTGTTATGCACTTTATATTTTAAGCAAGAAGCTTCATCCTTGATGATGGCCTATGGTGGTGA
Ca_LG_3:4133517	TACAAAAGATGCTTTAGTGATATTTGATGGTCTGAACAAGGAACTCTGTGTGGGGATCCATCTTGTAGTTATTCGCATTGTTCACCTTGGTTGAAATCTT	T/G	GTTGGTTGTTATGCACTTTATATTTTAAGCAAGAAGCTTCATCCTTGATGATGGCCTATGGTGGTGATGAATTGGGAATGGTTTTTCACCCTCCATTCAA
Ca_LG_3:4133519	CAAAAGATGCTTTAGTGATATTTGATGGTCTGAACAAGGAACTCTGTGTGGGGATCCATCTTGTAGTTATTCGCATTGTTCACCTTGGTTGAAATCTTGT	T/G	TGGTTGTTATGCACTTTATATTTTAAGCAAGAAGCTTCATCCTTGATGATGGCCTATGGTGGTGATGAATTGGGAATGGTTTTTCACCCTCCATTCAAAA
Ca_LG_3:4133528	CTTTAGTGATATTTGATGGTCTGAACAAGGAACTCTGTGTGGGGATCCATCTTGTAGTTATTCGCATTGTTCACCTTGGTTGAAATCTTGTTGGTTGTTA	A/C	TGCACTTTATATTTTAAGCAAGAAGCTTCATCCTTGATGATGGCCTATGGTGGTGATGAATTGGGAATGGTTTTTCACCCTCCATTCAAAATGTTTTGTT
Ca_LG_3:4133532	AGTGATATTTGATGGTCTGAACAAGGAACTCTGTGTGGGGATCCATCTTGTAGTTATTCGCATTGTTCACCTTGGTTGAAATCTTGTTGGTTGTTATGCA	A/G	CTTTATATTTTAAGCAAGAAGCTTCATCCTTGATGATGGCCTATGGTGGTGATGAATTGGGAATGGTTTTTCACCCTCCATTCAAAATGTTTTGTTTGTT

Positions	-100 bp flanking sequences	SNPs within *desi* cultivar	+100 bp flanking sequences
Ca_LG_3:5318863	CGTGAAAGAGCTGAATTCAGAGAGAGAGGCAAAGAGAAAAGTGAGAGAGGAAGAGCAAACCCTATTTTCGCATAAACAGAGCAGCTGCACTAGATTCGCG	G/C	ATTTCTCCTTTGTTCACCGTCGGATCGGGCTGAAATTTGAACAGCAGGTTCGTGACTCGTGGTACTTCATTCTGACCGTTTGGATCGTCGATCAGAGGTC
Ca_LG_3:5848239	GTGAGATCAATAAATCCAGCTCCCTTAAACTCTGCTGTCCCCATCAATGCGGAAGAAGCATCTTGTTCAACACAAGAAGCAGCTCTGTCTGACCTAGACG	G/A	ACTTAGATAAACAATTTGTTGAGAACATTTTTGGTCTAATGCAGCAAGAGCAAACATTTTCTGGCCAGGAGAAATTGATGGAGTGGATATTGACGATACA
Ca_LG_3:5963739	ACAGCAAAGGGGACACCAACGTCTCCACTTAAACTACCACTGAATGGTACAAAGGAGGAAAAATTCACTCAGACAATTTTTTATAAGCATCATTAAATCT	T/A	TCTTGCACTGCAAGGTTATTATGTGCTTGGAACAGCTTCTTTACTCTCTGTAGCTGCCTCACCAGCACCATCGCTCTTGCCAGCAGCATCTTGCTCACCA
Ca_LG_3:5963809	AGACAATTTTTTATAAGCATCATTAAATCTTCTTGCACTGCAAGGTTATTATGTGCTTGGAACAGCTTCTTTACTCTCTGTAGCTGCCTCACCAGCACCA	A/G	TCGCTCTTGCCAGCAGCATCTTGCTCACCAGGCTTCGACACTTCTTGCTCTATACAATTGCCAAACATAACAATTAGCAGGCAAATTTACCCGTTGATTA
Ca_LG_3:5963895	CCTCACCAGCACCATCGCTCTTGCCAGCAGCATCTTGCTCACCAGGCTTCGACACTTCTTGCTCTATACAATTGCCAAACATAACAATTAGCAGGCAAAT	T/A	TTACCCGTTGATTAGGAATACTATTTTCCAAAAAAATACCAAAATCTTGATGATACTGTACAGCTGCAGCATGTCATTCTAAACCACATATTCATCACGG
Ca_LG_3:5963900	CCAGCACCATCGCTCTTGCCAGCAGCATCTTGCTCACCAGGCTTCGACACTTCTTGCTCTATACAATTGCCAAACATAACAATTAGCAGGCAAATTTACC	C/T	CGTTGATTAGGAATACTATTTTCCAAAAAAATACCAAAATCTTGATGATACTGTACAGCTGCAGCATGTCATTCTAAACCACATATTCATCACGGGCAAT
Ca_LG_3:6000021	AAAATAAACAAAAAAAAATTATGAACAATTGAATGAATTTTTAAACTTTTGAATGAATATATAGTGCATTACCAATACGTAGAGGCAATTCTAGCAGCTT	T/C	TAGCTGTGAAAGAAACATAGCTGATGCTATAGAGTTGCTTGTTAATTGGGATAGCTGCGATTTTCCATTTTTTTGATGTATTGATTCTTTCTAACTCTTG
Ca_LG_3:6000033	AAAAAATTATGAACAATTGAATGAATTTTTAAACTTTTGAATGAATATATAGTGCATTACCAATACGTAGAGGCAATTCTAGCAGCTTTAGCTGTGAAAG	G/C	AAACATAGCTGATGCTATAGAGTTGCTTGTTAATTGGGATAGCTGCGATTTTCCATTTTTTTGATGTATTGATTCTTTCTAACTCTTGACCAAACTGATC
Ca_LG_3:6434728	AGAGAGAGAGAGCTGAATTCAGAGAGTGAGGCAAAGAGAAAAGTGAGAGAGGAAGAGCAAAACCCATTTTCGCATAAAACAGAGCAGCTGCACTAGATTC	C/T	GCAATTTCTCCTTCGTTCACCGTCGGATCGGGCTGAAATTTGGACAGCAGGTTAGTGACTCATGGTACTTCAATCTGACCGGTTGGGTCGTCGATCAGAG
Ca_LG_3:6454994	CAGATGCAACCAGATAACCCTAAGCCCTTCTTCTTGTCTGAACCCGGTGCCGCATTAGGTTAGGGAGCTGGGCTTTTTTCGCTCCTCTCACATTCATTCA	A/G	TTCGAGCTGCTTCGCTCCTCCGCATCCCAAAGCGCGCAGCCGTCCTACGCGCGAGACACTAAGTAATCCAAGCCAACCCACATTACTGACTAACGGTGGA
Ca_LG_3:7068872	CACAATTTGAAAAGTTGTGGAGCTATCCAAGCCAGTAGATATTTCATCCATTAATAGAACTTTTGCTGCTCCAACCAACATCTCTCCTGTTGTAACTCTT	T/C	TTCCTTTGTCCGCCCGAGATACCTCGCCGCATTCCATCCCCCACAATAGTATCACTACATACATCCAACCCCAAGATCTGCAAAATTTTATTTTATTGTA
Ca_LG_3:7068994	CTCGCCGCATTCCATCCCCCACAATAGTATCACTACATACATCCAACCCCAAGATCTGCAAAATTTTATTTTATTGTAGCTAAATTTTTCGTCAATAATT	T/C	CAACTTTTTTTTGGCACTAAAGAACCTTATTCTCTAGTATTACCTTCAGCATATAATCTATTACCAGACTTTCCTCCTGGCCTCCAGCTGCAGCTGCCTG

Positions	-100 bp flanking sequences	SNPs within *desi* cultivar	+100 bp flanking sequences
Ca_LG_3:7069058	TTTATTTTATTGTAGCTAAATTTTTCGTCAATAATTCAACTTTTTTTTGGCACTAAAGAACCTTATTCTCTAGTATTACCTTCAGCATATAATCTATTAC	C/T	CAGACTTTCCTCCTGGCCTCCAGCTGCAGCTGCCTGGTATATCAAAAGTCAATGAGATGCACACATACGCCCTCCGGTCATTACAGTAAACGAAAATAAC
Ca_LG_3:7069070	TAGCTAAATTTTTCGTCAATAATTCAACTTTTTTTTGGCACTAAAGAACCTTATTCTCTAGTATTACCTTCAGCATATAATCTATTACCAGACTTTCCTC	C/A	CTGGCCTCCAGCTGCAGCTGCCTGGTATATCAAAAGTCAATGAGATGCACACATACGCCCTCCGGTCATTACAGTAAACGAAAATAACTACTTTTTCTCA
Ca_LG_3:7371113	ACCTTTGGTGAAGAAATCTGCAATCTGATCAGTGCTGGAGACAAGGAGTAGCTTCATGGTTCCATTGTTGGCTTTCGCTATAACAATATGACAATCAATG	G/A	TCAAGATGTTTGGTTCTCTCATGAAAAACTGGATTGGCTGCTATATGAAGGGTGCTTCCATTGTCACAATAAATAGTTGGTGTCTTGATATAAGTAATCT
Ca_LG_3:7371116	TTTGGTGAAGAAATCTGCAATCTGATCAGTGCTGGAGACAAGGAGTAGCTTCATGGTTCCATTGTTGGCTTTCGCTATAACAATATGACAATCAATGTCA	A/T	AGATGTTTGGTTCTCTCATGAAAAACTGGATTGGCTGCTATATGAAGGGTGCTTCCATTGTCACAATAAATAGTTGGTGTCTTGATATAAGTAATCTTTA
Ca_LG_3:7371125	GAAATCTGCAATCTGATCAGTGCTGGAGACAAGGAGTAGCTTCATGGTTCCATTGTTGGCTTTCGCTATAACAATATGACAATCAATGTCAAGATGTTTG	G/T	GTTCTCTCATGAAAAACTGGATTGGCTGCTATATGAAGGGTGCTTCCATTGTCACAATAAATAGTTGGTGTCTTGATATAAGTAATCTTTAAGTCCTCAA
Ca_LG_3:7819804	CATCATAGGAATTGCTGCAAAAGCTTCAACCAAACCAGACAAAGCAAACTGTGGCACAAGCCACCAAATTCTTGTAGATGATTCAAATGTTCCATTTTTT	T/C	GCAGCACCGCGGCGGTGAATCTCAAATAGTCCTGAAACAACCATGCAAACGATAGATAAAATGATTCCTATTAATAACCTATGTTCAATTGACAACCTTT
Ca_LG_3:7871963	TCAAAAAAATTGGAGCACTTGGAAACAAGACAACCAAGTTTTCATTATACTCCCTTGCACTAACACTGTTTACTGAGAATTCTTATCATGAAGGTATCTC	C/A	ATTAACTTCATTATATGCTAGAAAAACAAGCAAATTACAATTAGAACTAAACAGTTTTGTTGAATAGCAGCACTATAGTGTAGCGGAATTTAAACAAAAT
Ca_LG_3:7871979	ACTTGGAAACAAGACAACCAAGTTTTCATTATACTCCCTTGCACTAACACTGTTTACTGAGAATTCTTATCATGAAGGTATCTCATTAACTTCATTATAT	T/A	GCTAGAAAAACAAGCAAATTACAATTAGAACTAAACAGTTTTGTTGAATAGCAGCACTATAGTGTAGCGGAATTTAAACAAAATGCTATTGTTCTGCGAT
Ca_LG_3:7901135	TCCACTCAAATGTTATTATTGCAAGCAGCCAGGCCACAAAGAGGCTAGCTGCTGGAAGAAAGAGGCAGATGAACAAAAGGGTGATCAAAAAATCCAATTTC	C/T	GTGGAGCATGAGCAAAAATTGTTCTTAGCACAAAGTGCAGCTAGCAATGATGCAGGAGGTGGCATGTGGTATGTTGACAGTGGATGCTCCAACCATATGT
Ca_LG_3:8166303	TGTGGACTTGTCATATCCCAATCCACAAATAGTATCAGCTTATATGCATATATAATATAATGTTTTGGTAAAAAAATGTATATAATGCAAAAATTCAAGC	C/T	TATGTAGCAAATGACAGAATGTTAGCCACCAAATCACTTTAGTAGCTTTACCTATTTTATCATTTCCCCATACATTCTTTTTCATGTGTTGACCTACATG
Ca_LG_3:8376496	GATGCATTACTTCTCAACCCTAGTTCCATCATCAATTCTACCAGCCACACGGCTTGTCAAATACCCATTGATGCAGCCATATATTCAGCTTCACAAGTGG	G/A	ACAATACTACTACACTTAGTTTCCTTCAACACCATGAGATTGGTGACTAATTCAGCATGAATTAATAGCCTGTGGTGCTCTTCCTTTCATCTTTATCTCC
Ca_LG_3:8554819	TTTCTTGCATTGATTTGTGGCTCAGCAACCACTCCAATTGTCCGATTTGCCGCGCCACCATTGCCGCCGCCGTTAAACGTTCCGATTCTAACAGAGAGGA	A/G	TGATCTGCAGCAGGGTCACCGTGACGCCTCTTCTATGGTTTGATTTTATTTTATTTTTTAATTTTTGTTATCATAAATGTGAGTTTTTTTTTATTACTAT

**Positions**	**-100 bp flanking sequences**	**SNPs within *desi* cultivar**	**+100 bp flanking sequences**
Ca_LG_3:8860616	TGTAACCCAGATGCTCCAGGTCAACTGATAATCTCAGTGTCCAATATCCTCTTTTTACATCACAAGTGTAAACATTTTGCAGCCACTTAAGTAACTCAAC	C/T	TGTGTCCCTGTACCTGAGAAACAGAAGTTGCTTTTTTTCACCATCTGTAGTACACAACATGATGAATAAATTGCAACCATAATACCATATCTACATTAAG
Ca_LG_3:8860619	AACCCAGATGCTCCAGGTCAACTGATAATCTCAGTGTCCAATATCCTCTTTTTACATCACAAGTGTAAACATTTTGCAGCCACTTAAGTAACTCAACTGT	T/C	GTCCCTGTACCTGAGAAACAGAAGTTGCTTTTTTTCACCATCTGTAGTACACAACATGATGAATAAATTGCAACCATAATACCATATCTACATTAAGTAG
Ca_LG_3:8860693	TGCAGCCACTTAAGTAACTCAACTGTGTCCCTGTACCTGAGAAACAGAAGTTGCTTTTTTTCACCATCTGTAGTACACAACATGATGAATAAATTGCAAC	C/T	CATAATACCATATCTACATTAAGTAGGTAGTTAATCCCAGATGGTGGTGCGTCGCAACCAACCTCAAAAAATGGGATAGCAGGATATCCTGGTTTGCCAC
Ca_LG_3:9101461	GAAATGACCTTCTGCATATCGATCTACATTTGAGGGAGCAGGACTTCCAGTTTTCTCTTTCTCCGCTTCTTGAGGTAGTCTCCGGAAGAAATCCACAGTG	G/A	AGATACGAAGATTCCATATCTACAAGCCGGAGAGTTGTCTTCCTACCTTCTTCACGAAACCTTTCTAAAGCCTCATTAGCCGCTGCAGCTAGTTCAGCTT
Ca_LG_3:9101482	ATCTACATTTGAGGGAGCAGGACTTCCAGTTTTCTCTTTCTCCGCTTCTTGAGGTAGTCTCCGGAAGAAATCCACAGTGAGATACGAAGATTCCATATCT	T/C	ACAAGCCGGAGAGTTGTCTTCCTACCTTCTTCACGAAACCTTTCTAAAGCCTCATTAGCCGCTGCAGCTAGTTCAGCTTGAAAAGTTGGGAATCGCTTCA
Ca_LG_3:9101491	TGAGGGAGCAGGACTTCCAGTTTTCTCTTTCTCCGCTTCTTGAGGTAGTCTCCGGAAGAAATCCACAGTGAGATACGAAGATTCCATATCTACAAGCCGG	G/A	AGAGTTGTCTTCCTACCTTCTTCACGAAACCTTTCTAAAGCCTCATTAGCCGCTGCAGCTAGTTCAGCTTGAAAAGTTGGGAATCGCTTCAGTTCCTGCT
Ca_LG_3:9101493	AGGGAGCAGGACTTCCAGTTTTCTCTTTCTCCGCTTCTTGAGGTAGTCTCCGGAAGAAATCCACAGTGAGATACGAAGATTCCATATCTACAAGCCGGAG	G/T	AGTTGTCTTCCTACCTTCTTCACGAAACCTTTCTAAAGCCTCATTAGCCGCTGCAGCTAGTTCAGCTTGAAAAGTTGGGAATCGCTTCAGTTCCTGCTCA
Ca_LG_3:9461435	TTAAATTTTATGCATGAGGTTGTTGCGGTAGATGTACATACATTTTGGTCGCACATTGTAAATGCTTTTGTTCGATTTAGGATATTTATTTCCTACACAT	T/G	ACTGTAGCAGCATTTAGATCAAACTTAACCAATTTTGTTCTCTAAATTTAGTTGCTTTGACTTGTTATATTGGTTTAAATTTAATCGATCTAATAGATTA
Ca_LG_3:9485023	AGTTCAGCAGAAAATGAGCAGTTTATGGCCATAATGGCCATTACTGCCAGACAGTAATAGGTGCAGCATCAAAAGGTGAAGAAATTATGGCCATAATGGT	T/G	CATTACTACCAGGCAGTAATAGGTGCAGCATTAGAAGATGAACATATTATGACCATAATGGTCATTACTGTCAAGCAGTATTCCTTGCTGACTTGAAGTA
Ca_LG_3:9723383	CAAATTCAGCAACAGCGTGTGAGGTCTTTGATGTTATAAATTTGGTTGTTTTACTTAATGGATGCAAACTTGTTGATTTAGTTTTGGGTGTTTAAAAAAT	T/C	CAGTTTAGTATCTTGGAGTTATTACAGACAATTGGTTGTAAGAACTTTGTTGTAGTTGGCTAATCTTTGCACTACTTGTCCATGGACTAGTGGTATTAAT
Ca_LG_3:9723395	CAGCGTGTGAGGTCTTTGATGTTATAAATTTGGTTGTTTTACTTAATGGATGCAAACTTGTTGATTTAGTTTTGGGTGTTTAAAAAATCAGTTTAGTATC	C/T	TTGGAGTTATTACAGACAATTGGTTGTAAGAACTTTGTTGTAGTTGGCTAATCTTTGCACTACTTGTCCATGGACTAGTGGTATTAATTCACGTTTCTTG
Ca_LG_3:9723418	ATAAATTTGGTTGTTTTACTTAATGGATGCAAACTTGTTGATTTAGTTTTGGGTGTTTAAAAAATCAGTTTAGTATCTTGGAGTTATTACAGACAATTGG	G/A	TTGTAAGAACTTTGTTGTAGTTGGCTAATCTTTGCACTACTTGTCCATGGACTAGTGGTATTAATTCACGTTTCTTGTGTTTCATAGTGGTATTAATTCA

Positions	-100 bp flanking sequences	SNPs within *desi* cultivar	+100 bp flanking sequences
Ca_LG_3:9723435	ACTTAATGGATGCAAACTTGTTGATTTAGTTTTGGGTGTTTAAAAAATCAGTTTAGTATCTTGGAGTTATTACAGACAATTGGTTGTAAGAACTTTGTTG	G/A	TAGTTGGCTAATCTTTGCACTACTTGTCCATGGACTAGTGGTATTAATTCACGTTTCTTGTGTTTCATAGTGGTATTAATTCAGAAAAATTGTTAACTTG
Ca_LG_3:9723439	AATGGATGCAAACTTGTTGATTTAGTTTTGGGTGTTTAAAAAATCAGTTTAGTATCTTGGAGTTATTACAGACAATTGGTTGTAAGAACTTTGTTGTAGT	T/C	TGGCTAATCTTTGCACTACTTGTCCATGGACTAGTGGTATTAATTCACGTTTCTTGTGTTTCATAGTGGTATTAATTCAGAAAAATTGTTAACTTGTGTT
Ca_LG_3:9986238	CGTCCCCCGACCGGCACGCGGCGACCCGCTCTCGCCGCGGGAGCAGCTCAAGCAGTCCACCAACAGCCGACGGGTTCGGAACTGGGACCCCCGTGCCCAG	G/A	CCCTCAGAGCCAATCCTTTTCCCGAGGTTACGGATCCATTTTGCCGACTTCCCTTGCCTACATTGTTCCATCGACCAGAGGCTGTTCACCTTGGAGACCT
Ca_LG_3:9986264	CGCTCTCGCCGCGGGAGCAGCTCAAGCAGTCCACCAACAGCCGACGGGTTCGGAACTGGGACCCCCGTGCCCAGCCCTCAGAGCCAATCCTTTTCCCGAG	G/A	GTTACGGATCCATTTTGCCGACTTCCCTTGCCTACATTGTTCCATCGACCAGAGGCTGTTCACCTTGGAGACCTGATGCGGTTATGAGTACGACCGGGCA
Ca_LG_3:9986275	CGGGAGCAGCTCAAGCAGTCCACCAACAGCCGACGGGTTCGGAACTGGGACCCCCGTGCCCAGCCCTCAGAGCCAATCCTTTTCCCGAGGTTACGGATCC	C/T	ATTTTGCCGACTTCCCTTGCCTACATTGTTCCATCGACCAGAGGCTGTTCACCTTGGAGACCTGATGCGGTTATGAGTACGACCGGGCATGGAAGGCACT
Ca_LG_3:9986294	CCACCAACAGCCGACGGGTTCGGAACTGGGACCCCCGTGCCCAGCCCTCAGAGCCAATCCTTTTCCCGAGGTTACGGATCCATTTTGCCGACTTCCCTTG	G/A	CCTACATTGTTCCATCGACCAGAGGCTGTTCACCTTGGAGACCTGATGCGGTTATGAGTACGACCGGGCATGGAAGGCACTCGGTCCTCCGGATTTTCAA
Ca_LG_3:9986295	CACCAACAGCCGACGGGTTCGGAACTGGGACCCCCGTGCCCAGCCCTCAGAGCCAATCCTTTTCCCGAGGTTACGGATCCATTTTGCCGACTTCCCTTGC	C/T	CTACATTGTTCCATCGACCAGAGGCTGTTCACCTTGGAGACCTGATGCGGTTATGAGTACGACCGGGCATGGAAGGCACTCGGTCCTCCGGATTTTCAAG
Ca_LG_3:9986307	ACGGGTTCGGAACTGGGACCCCCGTGCCCAGCCCTCAGAGCCAATCCTTTTCCCGAGGTTACGGATCCATTTTGCCGACTTCCCTTGCCTACATTGTTCC	C/T	ATCGACCAGAGGCTGTTCACCTTGGAGACCTGATGCGGTTATGAGTACGACCGGGCATGGAAGGCACTCGGTCCTCCGGATTTTCAAGGGCCGCCAGGGG
Ca_LG_3:9986311	GTTCGGAACTGGGACCCCCGTGCCCAGCCCTCAGAGCCAATCCTTTTCCCGAGGTTACGGATCCATTTTGCCGACTTCCCTTGCCTACATTGTTCCATCG	G/A	ACCAGAGGCTGTTCACCTTGGAGACCTGATGCGGTTATGAGTACGACCGGGCATGGAAGGCACTCGGTCCTCCGGATTTTCAAGGGCCGCCAGGGGCGCA
Ca_LG_3:9986314	CGGAACTGGGACCCCCGTGCCCAGCCCTCAGAGCCAATCCTTTTCCCGAGGTTACGGATCCATTTTGCCGACTTCCCTTGCCTACATTGTTCCATCGACC	C/T	AGAGGCTGTTCACCTTGGAGACCTGATGCGGTTATGAGTACGACCGGGCATGGAAGGCACTCGGTCCTCCGGATTTTCAAGGGCCGCCAGGGGCGCACCG
Ca_LG_3:10753824	AACAAATAGAATATATAATAATCCTTAATTGTTTTGATTTTTCAATGAGATTGATTGCTTTTGTTTTTTGTTTCAGCCTGATGTAACTGCGCCAGGTGTA	A/G	AACATACTTGCTGCCTATTCATTGTTTGCAAGTGCATCTAATTTACTAACAGATACTCGTCGAGGTTTTCCATTCAATGTAATGCAGGGAACTTCTATGT
Ca_LG_3:10753950	TGCAAGTGCATCTAATTTACTAACAGATACTCGTCGAGGTTTTCCATTCAATGTAATGCAGGGAACTTCTATGTCTTGCCCTCATGTCGCTGGCATTGCA	A/T	GGACTTATCAAAACACTTCATCCTAATTGGAGTCCAGCTGCTATTAAATCAGCTATCATGACCACGGGTATTGTCTCTCTCATCACTCACACAGACACTT

Positions	-100 bp flanking sequences	SNPs within *desi* cultivar	+100 bp flanking sequences
Ca_LG_3:11242017	CTCTGATAATTAAAGTTTGGATCCAATGTTTCTCTCAGTAGTATCTTCAACGGCCCTAATCACGTGACCCAAAATCTTCTTCCGATTCGCAGCGTACACA	A/G	TGTCCATCCCATCCACGCTTCACCAAATCAACGGCCAAACCAACCGCAGCCTTAACCGTCTCCTCAACGCTATCATGTGCCGAGTCGATTATCCCCACCC
Ca_LG_3:11242029	AAGTTTGGATCCAATGTTTCTCTCAGTAGTATCTTCAACGGCCCTAATCACGTGACCCAAAATCTTCTTCCGATTCGCAGCGTACACATGTCCATCCCAT	T/G	CCACGCTTCACCAAATCAACGGCCAAACCAACCGCAGCCTTAACCGTCTCCTCAACGCTATCATGTGCCGAGTCGATTATCCCCACCCGCACCGCCTCCT
Ca_LG_3:11242062	TTCAACGGCCCTAATCACGTGACCCAAAATCTTCTTCCGATTCGCAGCGTACACATGTCCATCCCATCCACGCTTCACCAAATCAACGGCCAAACCAACC	C/A	GCAGCCTTAACCGTCTCCTCAACGCTATCATGTGCCGAGTCGATTATCCCCACCCGCACCGCCTCCTCAGCAGTCATCTTCTCCGCCTGCATCAGGATCC
Ca_LG_3:11242084	CCCAAAATCTTCTTCCGATTCGCAGCGTACACATGTCCATCCCATCCACGCTTCACCAAATCAACGGCCAAACCAACCGCAGCCTTAACCGTCTCCTCAA	A/G	CGCTATCATGTGCCGAGTCGATTATCCCCACCCGCACCGCCTCCTCAGCAGTCATCTTCTCCGCCTGCATCAGGATCCGACGGCGTACCGCAGGTGCTCC
Ca_LG_3:11242095	CTTCCGATTCGCAGCGTACACATGTCCATCCCATCCACGCTTCACCAAATCAACGGCCAAACCAACCGCAGCCTTAACCGTCTCCTCAACGCTATCATGT	T/C	GCCGAGTCGATTATCCCCACCCGCACCGCCTCCTCAGCAGTCATCTTCTCCGCCTGCATCAGGATCCGACGGCGTACCGCAGGTGCTCCGACCTTAGCAT
Ca_LG_3:11689294	TTTTTCTTGTAGTGGTATAACCATTCATCAATGGATGCAGCTTCCCATATCGAGTATAAGTGCAAACCAATAGTCGCAAAAGTATAAATAATGGCACCAT	T/G	AAATAATATTGTTTCCGTAAAGTAGAGATCTAGAAACATGCTCACGAATACCATCAATATCTACAGGAGGGGCAACAACGAAACCATAATAAATACATAA
Ca_LG_3:11689311	TAACCATTCATCAATGGATGCAGCTTCCCATATCGAGTATAAGTGCAAACCAATAGTCGCAAAAGTATAAATAATGGCACCATAAATAATATTGTTTCCG	G/A	TAAAGTAGAGATCTAGAAACATGCTCACGAATACCATCAATATCTACAGGAGGGGCAACAACGAAACCATAATAAATACATAAGTTGTTGTCAATAAGGT
Ca_LG_3:11689325	TGGATGCAGCTTCCCATATCGAGTATAAGTGCAAACCAATAGTCGCAAAAGTATAAATAATGGCACCATAAATAATATTGTTTCCGTAAAGTAGAGATCT	T/C	AGAAACATGCTCACGAATACCATCAATATCTACAGGAGGGGCAACAACGAAACCATAATAAATACATAAGTTGTTGTCAATAAGGTAGCATGTATATAAA
Ca_LG_3:11689333	GCTTCCCATATCGAGTATAAGTGCAAACCAATAGTCGCAAAAGTATAAATAATGGCACCATAAATAATATTGTTTCCGTAAAGTAGAGATCTAGAAACAT	T/G	GCTCACGAATACCATCAATATCTACAGGAGGGGCAACAACGAAACCATAATAAATACATAAGTTGTTGTCAATAAGGTAGCATGTATATAAAATTGCATA
Ca_LG_3:11689359	ACCAATAGTCGCAAAAGTATAAATAATGGCACCATAAATAATATTGTTTCCGTAAAGTAGAGATCTAGAAACATGCTCACGAATACCATCAATATCTACA	A/T	GGAGGGGCAACAACGAAACCATAATAAATACATAAGTTGTTGTCAATAAGGTAGCATGTATATAAAATTGCATAGTTATGATAAAATCTCAATTAATTAA
Ca_LG_3:11689369	GCAAAAGTATAAATAATGGCACCATAAATAATATTGTTTCCGTAAAGTAGAGATCTAGAAACATGCTCACGAATACCATCAATATCTACAGGAGGGGCAA	A/G	CAACGAAACCATAATAAATACATAAGTTGTTGTCAATAAGGTAGCATGTATATAAAATTGCATAGTTATGATAAAATCTCAATTAATTAAAAGGGGTATG
Ca_LG_3:11737488	CAATTGACCTTATTGTATACAAAAGATTGGTTAAAAAGCAAATTGGAAAAATTATGAGATGTCTAACCAGAGAATGGCAGCCCAAAATAACAGCCATAGT	T/C	TGAATCTAAAGATCTAGCAAAGATGACAACTGCAACATTGTTTGGAAAACTAAGGGAACATGAAATGGAACTGCATAGGCTGGACGAATCAGAAATGGAA

**Positions**	**-100 bp flanking sequences**	**SNPs within *desi* cultivar**	**+100 bp flanking sequences**
Ca_LG_3:12179531	TTTACTTTTAAAGCCAAAGTGTCCATTTGAATAACTGATAGCCGCTACCAGCTATCAGTTATGAGAAGGTTATATCCGCTACCAGCTATCAGCTATGAGA	A/G	AGGCGATAGCCTCTACCAGCTATCAGCTATGAGAAGGCATACCAGCTAGCTAGCTGCTTTATGATAGCCAATAGCTGATGGCCGACAGAGGATATCACCT
Ca_LG_3:12179532	TTACTTTTAAAGCCAAAGTGTCCATTTGAATAACTGATAGCCGCTACCAGCTATCAGTTATGAGAAGGTTATATCCGCTACCAGCTATCAGCTATGAGAA	A/C	GGCGATAGCCTCTACCAGCTATCAGCTATGAGAAGGCATACCAGCTAGCTAGCTGCTTTATGATAGCCAATAGCTGATGGCCGACAGAGGATATCACCTA
Ca_LG_3:12179543	GCCAAAGTGTCCATTTGAATAACTGATAGCCGCTACCAGCTATCAGTTATGAGAAGGTTATATCCGCTACCAGCTATCAGCTATGAGAAGGCGATAGCCT	T/G	CTACCAGCTATCAGCTATGAGAAGGCATACCAGCTAGCTAGCTGCTTTATGATAGCCAATAGCTGATGGCCGACAGAGGATATCACCTATCAGATATCAA
Ca_LG_3:12179555	ATTTGAATAACTGATAGCCGCTACCAGCTATCAGTTATGAGAAGGTTATATCCGCTACCAGCTATCAGCTATGAGAAGGCGATAGCCTCTACCAGCTATC	C/G	AGCTATGAGAAGGCATACCAGCTAGCTAGCTGCTTTATGATAGCCAATAGCTGATGGCCGACAGAGGATATCACCTATCAGATATCAAGATATCAGATTT
Ca_LG_3:12644514	CACCTGCCCTTCTGGAGAATGAGTTCAAGAAGTTTGGACCTATAAAGAGTGGTGGTATCCAAGTTAGGGCGCAAAAGGTATTTCACTTGGTAATTCACTA	A/G	ATTTTTATTGTTGGCTGCTAGCTAATACCTTTTTGGCATATTAATGGGTTTATCAGGGATTTTACTTTGGCTTTGTGGAGTTTGAAGTGGCTAGTGCTGT
Ca_LG_3:13412210	CTCGCCGTCAAACCAGTCGCGCCGTCGAACCAGTCGCGCCGTCAAACCAGTCGCGCCGTCGAACCAGTCGCGCCGTCAAACCAGTCGCGCCGTCGAACCG	G/C	GAGCTGCACTCGCCGTCGAACCAGTCGTGCCTTCGAACCCGAGCTGCACTCACCGTCGTTCTCCCCGTTCTCGCACCACTGGTAATTTCTTGAACCACCA
Ca_LG_3:15424931	TTACTAATCATGATTTAAAGTTTGTTTGTTTGTATGTTATAAATTTAATGCAAGCCTTGAATGCAATGGCCACTGCAATGGCCCAACAGGCTCAGCAGGC	C/T	TGCAAACCAGGTTTAACTTAATGCACAAAAGGACCAGAGGGAGGAAGCAGCGATTGCTATGATAGGACTGACTAAATTCTGTCGACAAGATCCGCCAAAA
Ca_LG_3:15424937	ATCATGATTTAAAGTTTGTTTGTTTGTATGTTATAAATTTAATGCAAGCCTTGAATGCAATGGCCACTGCAATGGCCCAACAGGCTCAGCAGGCTGCAAA	A/T	CCAGGTTTAACTTAATGCACAAAAGGACCAGAGGGAGGAAGCAGCGATTGCTATGATAGGACTGACTAAATTCTGTCGACAAGATCCGCCAAAATTTAAA
Ca_LG_3:15424943	ATTTAAAGTTTGTTTGTTTGTATGTTATAAATTTAATGCAAGCCTTGAATGCAATGGCCACTGCAATGGCCCAACAGGCTCAGCAGGCTGCAAACCAGGT	T/C	TTAACTTAATGCACAAAAGGACCAGAGGGAGGAAGCAGCGATTGCTATGATAGGACTGACTAAATTCTGTCGACAAGATCCGCCAAAATTTAAAGGGGAG
Ca_LG_3:15424953	TGTTTGTTTGTATGTTATAAATTTAATGCAAGCCTTGAATGCAATGGCCACTGCAATGGCCCAACAGGCTCAGCAGGCTGCAAACCAGGTTTAACTTAAT	T/A	GCACAAAAGGACCAGAGGGAGGAAGCAGCGATTGCTATGATAGGACTGACTAAATTCTGTCGACAAGATCCGCCAAAATTTAAAGGGGAGCATGACCCTG
Ca_LG_3:15424957	TGTTTGTATGTTATAAATTTAATGCAAGCCTTGAATGCAATGGCCACTGCAATGGCCCAACAGGCTCAGCAGGCTGCAAACCAGGTTTAACTTAATGCAC	C/A	AAAAGGACCAGAGGGAGGAAGCAGCGATTGCTATGATAGGACTGACTAAATTCTGTCGACAAGATCCGCCAAAATTTAAAGGGGAGCATGACCCTGACAA
Ca_LG_3:15424961	TGTATGTTATAAATTTAATGCAAGCCTTGAATGCAATGGCCACTGCAATGGCCCAACAGGCTCAGCAGGCTGCAAACCAGGTTTAACTTAATGCACAAAA	A/T	GGACCAGAGGGAGGAAGCAGCGATTGCTATGATAGGACTGACTAAATTCTGTCGACAAGATCCGCCAAAATTTAAAGGGGAGCATGACCCTGACAAGGCT

Positions	-100 bp flanking sequences	SNPs within *desi* cultivar	+100 bp flanking sequences
Ca_LG_3:15424984	GCCTTGAATGCAATGGCCACTGCAATGGCCCAACAGGCTCAGCAGGCTGCAAACCAGGTTTAACTTAATGCACAAAAGGACCAGAGGGAGGAAGCAGCGA	A/G	TTGCTATGATAGGACTGACTAAATTCTGTCGACAAGATCCGCCAAAATTTAAAGGGGAGCATGACCCTGACAAGGCTGATCTCTGGCAGCAGGAGATTGA
Ca_LG_3:15424991	ATGCAATGGCCACTGCAATGGCCCAACAGGCTCAGCAGGCTGCAAACCAGGTTTAACTTAATGCACAAAAGGACCAGAGGGAGGAAGCAGCGATTGCTAT	T/C	GATAGGACTGACTAAATTCTGTCGACAAGATCCGCCAAAATTTAAAGGGGAGCATGACCCTGACAAGGCTGATCTCTGGCAGCAGGAGATTGAGAAGATA
Ca_LG_3:15424992	TGCAATGGCCACTGCAATGGCCCAACAGGCTCAGCAGGCTGCAAACCAGGTTTAACTTAATGCACAAAAGGACCAGAGGGAGGAAGCAGCGATTGCTATG	G/A	ATAGGACTGACTAAATTCTGTCGACAAGATCCGCCAAAATTTAAAGGGGAGCATGACCCTGACAAGGCTGATCTCTGGCAGCAGGAGATTGAGAAGATAT
Ca_LG_3:15776921	TCCTCTGATGTTCCTGTTCCATCTCCTTCATCCTCGTTTGAAGAGTCATCCTCCTCCGAGTAACTTTGTGTTTCCTTCTTTTTGCTGCTGCCAAATTCAG	G/A	GGGGAGATAGATCATGATTATCTAGTAGTTGTCTTTGTGTTGAACTAGCTGTTGAGTTGATTTGTTTTGTTTTGTTGCCTATGTATCTTGTTTGATGTTT
Ca_LG_3:15776941	ATCTCCTTCATCCTCGTTTGAAGAGTCATCCTCCTCCGAGTAACTTTGTGTTTCCTTCTTTTTGCTGCTGCCAAATTCAGGGGGAGATAGATCATGATTA	A/T	TCTAGTAGTTGTCTTTGTGTTGAACTAGCTGTTGAGTTGATTTGTTTTGTTTTGTTGCCTATGTATCTTGTTTGATGTTTATCTTGTGTTGAACAAACTT
Ca_LG_3:16372227	ACAAAAAAGTTAAAAGTTAGTAACCCTAAAAATAATTGCACACTTGAAGGCACAACAAAGCGGCTGTGTTTGCAGAAAGCACAAGTGCAGCAGCAGCTTA	A/G	ACGGACGCGGCAACGGCTGGGTGTTTTGGTGACACGGCGGACATCCTTAGCGGCTTGGATCCTCAAAACTTGATCATCCAGCTTTAACCGCTCTGAGGCT
Ca_LG_3:16372247	TAACCCTAAAAATAATTGCACACTTGAAGGCACAACAAAGCGGCTGTGTTTGCAGAAAGCACAAGTGCAGCAGCAGCTTAACGGACGCGGCAACGGCTGG	G/T	GTGTTTTGGTGACACGGCGGACATCCTTAGCGGCTTGGATCCTCAAAACTTGATCATCCAGCTTTAACCGCTCTGAGGCTTTCCATACGGCCGCCATCTT
Ca_LG_3:16372258	ATAATTGCACACTTGAAGGCACAACAAAGCGGCTGTGTTTGCAGAAAGCACAAGTGCAGCAGCAGCTTAACGGACGCGGCAACGGCTGGGTGTTTTGGTG	G/A	ACACGGCGGACATCCTTAGCGGCTTGGATCCTCAAAACTTGATCATCCAGCTTTAACCGCTCTGAGGCTTTCCATACGGCCGCCATCTTTATCATTGTTC
Ca_LG_3:16372260	AATTGCACACTTGAAGGCACAACAAAGCGGCTGTGTTTGCAGAAAGCACAAGTGCAGCAGCAGCTTAACGGACGCGGCAACGGCTGGGTGTTTTGGTGAC	C/T	ACGGCGGACATCCTTAGCGGCTTGGATCCTCAAAACTTGATCATCCAGCTTTAACCGCTCTGAGGCTTTCCATACGGCCGCCATCTTTATCATTGTTCTC
Ca_LG_3:16372261	ATTGCACACTTGAAGGCACAACAAAGCGGCTGTGTTTGCAGAAAGCACAAGTGCAGCAGCAGCTTAACGGACGCGGCAACGGCTGGGTGTTTTGGTGACA	A/G	CGGCGGACATCCTTAGCGGCTTGGATCCTCAAAACTTGATCATCCAGCTTTAACCGCTCTGAGGCTTTCCATACGGCCGCCATCTTTATCATTGTTCTCC
Ca_LG_3:16372262	TTGCACACTTGAAGGCACAACAAAGCGGCTGTGTTTGCAGAAAGCACAAGTGCAGCAGCAGCTTAACGGACGCGGCAACGGCTGGGTGTTTTGGTGACAC	C/T	GGCGGACATCCTTAGCGGCTTGGATCCTCAAAACTTGATCATCCAGCTTTAACCGCTCTGAGGCTTTCCATACGGCCGCCATCTTTATCATTGTTCTCCA
Ca_LG_3:16372266	ACACTTGAAGGCACAACAAAGCGGCTGTGTTTGCAGAAAGCACAAGTGCAGCAGCAGCTTAACGGACGCGGCAACGGCTGGGTGTTTTGGTGACACGGCG	G/A	GACATCCTTAGCGGCTTGGATCCTCAAAACTTGATCATCCAGCTTTAACCGCTCTGAGGCTTTCCATACGGCCGCCATCTTTATCATTGTTCTCCAAGGT

Positions	-100 bp flanking sequences	SNPs within *desi* cultivar	+100 bp flanking sequences
Ca_LG_3:16372289	GCTGTGTTTGCAGAAAGCACAAGTGCAGCAGCAGCTTAACGGACGCGGCAACGGCTGGGTGTTTTGGTGACACGGCGGACATCCTTAGCGGCTTGGATCC	C/T	TCAAAACTTGATCATCCAGCTTTAACCGCTCTGAGGCTTTCCATACGGCCGCCATCTTTATCATTGTTCTCCAAGGTTGTAAGAGAAAATCTCCATTGCT
Ca_LG_3:16372308	CAAGTGCAGCAGCAGCTTAACGGACGCGGCAACGGCTGGGTGTTTTGGTGACACGGCGGACATCCTTAGCGGCTTGGATCCTCAAAACTTGATCATCCAG	G/A	CTTTAACCGCTCTGAGGCTTTCCATACGGCCGCCATCTTTATCATTGTTCTCCAAGGTTGTAAGAGAAAATCTCCATTGCTCGAATTTGAGAGAGAGAGA
Ca_LG_3:16372321	AGCTTAACGGACGCGGCAACGGCTGGGTGTTTTGGTGACACGGCGGACATCCTTAGCGGCTTGGATCCTCAAAACTTGATCATCCAGCTTTAACCGCTCT	T/C	GAGGCTTTCCATACGGCCGCCATCTTTATCATTGTTCTCCAAGGTTGTAAGAGAAAATCTCCATTGCTCGAATTTGAGAGAGAGAGAGAGAGTGCAACGA
Ca_LG_3:16372322	GCTTAACGGACGCGGCAACGGCTGGGTGTTTTGGTGACACGGCGGACATCCTTAGCGGCTTGGATCCTCAAAACTTGATCATCCAGCTTTAACCGCTCTG	G/A	AGGCTTTCCATACGGCCGCCATCTTTATCATTGTTCTCCAAGGTTGTAAGAGAAAATCTCCATTGCTCGAATTTGAGAGAGAGAGAGAGAGTGCAACGAC
Ca_LG_3:16590388	AAGACTCTACTTGATGATGGTTTTATAGGAAACCATGCTGCCAAGAAGAAATGACGCTCCGAGAGCCAACGCTAATAGGAATGATCAAATGGCGGAGGCT	T/C	ATGAATAACATGGCTGCTTCTGTTGCTGCACAGACTGCTGCCAAGACTCAAAGGGATCTTGAAAAGAGGGGAAGAGAGATCCGTGCGGCAGAGTCAAGAG
Ca_LG_3:16697127	AATTGAAGAAAGTGACAATGTTTGAATATGTGCGTATTTAATATTGCTACACTGTAATTTTATTAATGAACATCTAAATCACCTAATTCCATAGCAGAAT	T/C	CCGACCTTGGAATTTAATGTGTTAAGCATATGATTTTTCCGTCTTATCCAAAGTGGTCAGAAATGTGAGACAGCTTTTTTGTACTCTATATTTTTTGTAT
Ca_LG_3:16697142	CAATGTTTGAATATGTGCGTATTTAATATTGCTACACTGTAATTTTATTAATGAACATCTAAATCACCTAATTCCATAGCAGAATCCGACCTTGGAATTT	T/G	AATGTGTTAAGCATATGATTTTTCCGTCTTATCCAAAGTGGTCAGAAATGTGAGACAGCTTTTTTGTACTCTATATTTTTTGTATGAATTTCTGGTTCTG
Ca_LG_3:16854692	TTTTTCTTTGTCTTTAATGTTAGGAAAAAATAGCTAAGCAAGCTATATAACATACCAAAGCCTGAACTCCGCCAGCTGCTGCAATGGCCTCTCGATTTCT	T/C	TTCGTCAAATGACAAATGACAAATTCCAAAATGAACCAGCAGCTTCTTGCCTATAGAAATACAACAATGTCAAATCAACACTGTAATGAATGTAATATTT
Ca_LG_3:17088567	GCTGCAAAGCCACAAAATCCCAAAGTCTCGTTGCCATTTGTGGCCACCACAAACGCATTGAGCCATTACCCTTTGTGGCTGCAAAGGCCGCCACAAATTT	T/C	CCATAGTTCCGTTGCCATTTGTGGCCGCCCAGGCCGCCACAAACGCCTGCCTTTCTGACCGTTTTGGCCGCCACAAAATTCATAGATCCGTTGCACTTTG
Ca_LG_3:17088569	TGCAAAGCCACAAAATCCCAAAGTCTCGTTGCCATTTGTGGCCACCACAAACGCATTGAGCCATTACCCTTTGTGGCTGCAAAGGCCGCCACAAATTTCC	C/A	ATAGTTCCGTTGCCATTTGTGGCCGCCCAGGCCGCCACAAACGCCTGCCTTTCTGACCGTTTTGGCCGCCACAAAATTCATAGATCCGTTGCACTTTGTG
Ca_LG_3:17088576	CCACAAAATCCCAAAGTCTCGTTGCCATTTGTGGCCACCACAAACGCATTGAGCCATTACCCTTTGTGGCTGCAAAGGCCGCCACAAATTTCCATAGTTC	C/T	CGTTGCCATTTGTGGCCGCCCAGGCCGCCACAAACGCCTGCCTTTCTGACCGTTTTGGCCGCCACAAAATTCATAGATCCGTTGCACTTTGTGGCTGCTT
Ca_LG_3:17088593	CTCGTTGCCATTTGTGGCCACCACAAACGCATTGAGCCATTACCCTTTGTGGCTGCAAAGGCCGCCACAAATTTCCATAGTTCCGTTGCCATTTGTGGCC	C/T	GCCCAGGCCGCCACAAACGCCTGCCTTTCTGACCGTTTTGGCCGCCACAAAATTCATAGATCCGTTGCACTTTGTGGCTGCTTTGGCCGCCACAAAGGGT

**Positions**	**-100 bp flanking sequences**	**SNPs within *desi* cultivar**	**+100 bp flanking sequences**
Ca_LG_3:17088604	TTGTGGCCACCACAAACGCATTGAGCCATTACCCTTTGTGGCTGCAAAGGCCGCCACAAATTTCCATAGTTCCGTTGCCATTTGTGGCCGCCCAGGCCGC	C/T	CACAAACGCCTGCCTTTCTGACCGTTTTGGCCGCCACAAAATTCATAGATCCGTTGCACTTTGTGGCTGCTTTGGCCGCCACAAAGGGTCAACCATTTTC
Ca_LG_3:17426256	CGGTGGTGAGGTTGATATTCGATTACGTGTGTGGTGGTGGTGGAAAGTGGTGGTTCTTCTGGTTCGCGGCGGTGGAAATTACGGTGGCAGCCACATGCGG	G/A	CGCATTTTATGGAAGTTGGGTCGGCGGATGTTGCGGTGGGAGATGTCATGAATTCGCCGCAACCGTCGAGGGCGTGGCCACCTAAGTTGGCTGCGTGGTT
Ca_LG_3:17621916	GTTTAATATCTAAATCAAACAGGTCACATATTAGTTTTAAAGTTGCATGTGACAAGAAATAGGTTTAATGCATCGCGAAATTCAGCTGCTGCAGCAGTGG	G/T	GAATATCGCGTCAGCAGCGTTTTGCGCAGCAGCAGGAGCCGCTGAAAAGATAGCAGTCGCAAAATGACAGTGCTGCCAACCTCTGACTTCCGTAGATTAG
Ca_LG_3:17680851	TATTTGGAGGTCCTTGTCAATTGCATTGAGATCTAAGAGAGACATCATTTTATCAGTGGTATCAAGCGAAATTGCAGGTTTACTTATTCTTGGAGGTAGG	G/A	CCTGCGCATTCAAGGTTCTCCATACCATTAGAAGTTGACGAATATTCCATTTGCAGCACAACTTAGAATAGTCCACTAACATAATTGATTGCAAAAACAA
Ca_LG_3:17680852	ATTTGGAGGTCCTTGTCAATTGCATTGAGATCTAAGAGAGACATCATTTTATCAGTGGTATCAAGCGAAATTGCAGGTTTACTTATTCTTGGAGGTAGGC	C/A	CTGCGCATTCAAGGTTCTCCATACCATTAGAAGTTGACGAATATTCCATTTGCAGCACAACTTAGAATAGTCCACTAACATAATTGATTGCAAAAACAAA
Ca_LG_3:17680857	GAGGTCCTTGTCAATTGCATTGAGATCTAAGAGAGACATCATTTTATCAGTGGTATCAAGCGAAATTGCAGGTTTACTTATTCTTGGAGGTAGGCCTGCG	G/A	CATTCAAGGTTCTCCATACCATTAGAAGTTGACGAATATTCCATTTGCAGCACAACTTAGAATAGTCCACTAACATAATTGATTGCAAAAACAAAGCTCA
Ca_LG_3:17680866	GTCAATTGCATTGAGATCTAAGAGAGACATCATTTTATCAGTGGTATCAAGCGAAATTGCAGGTTTACTTATTCTTGGAGGTAGGCCTGCGCATTCAAGG	G/A	TTCTCCATACCATTAGAAGTTGACGAATATTCCATTTGCAGCACAACTTAGAATAGTCCACTAACATAATTGATTGCAAAAACAAAGCTCATTATATTAG
Ca_LG_3:17680890	AGACATCATTTTATCAGTGGTATCAAGCGAAATTGCAGGTTTACTTATTCTTGGAGGTAGGCCTGCGCATTCAAGGTTCTCCATACCATTAGAAGTTGAC	C/T	GAATATTCCATTTGCAGCACAACTTAGAATAGTCCACTAACATAATTGATTGCAAAAACAAAGCTCATTATATTAGAAGAAGCACCAATGACTTATAAAC
Ca_LG_3:17680899	TTTATCAGTGGTATCAAGCGAAATTGCAGGTTTACTTATTCTTGGAGGTAGGCCTGCGCATTCAAGGTTCTCCATACCATTAGAAGTTGACGAATATTCC	C/A	ATTTGCAGCACAACTTAGAATAGTCCACTAACATAATTGATTGCAAAAACAAAGCTCATTATATTAGAAGAAGCACCAATGACTTATAAACATTGTTTTC
Ca_LG_3:18077530	TTTTTTAGACATCTAGAACAGATTAGTCTGAAGAGCCTCAATCACTGACAATCTTTCGCAGCTTGCATTTACAGGTTCAACTGATACTGGAAAAGCCGTT	T/C	CTTCAACTGGCAGCAAAAAGCAATCTTAAACCTGTAACTTTGGAGCTTGGTGGAAAATCTCCTTTTATTGTATGCGAAGATGCTGATGTAGATCAAGCTG
Ca_LG_3:18314872	GAAAATAGTATCAATCCAATATCTTGTGCTATTTGTTCTACCACATTCAAATAAAATGGCAAGAGCAGTAATGATCATGCTTTGACCATCATATGATTCC	C/A	AACGTCATTTTCTTCAATCTCACTTCCATAAGATTTGGAGTTACTATTATCATTTACACTTGGTGTAGAGCTAGCAGTGCTGCTTGTGGCATGCGGTGAA
Ca_LG_3:18314875	AATAGTATCAATCCAATATCTTGTGCTATTTGTTCTACCACATTCAAATAAAATGGCAAGAGCAGTAATGATCATGCTTTGACCATCATATGATTCCAAC	C/A	GTCATTTTCTTCAATCTCACTTCCATAAGATTTGGAGTTACTATTATCATTTACACTTGGTGTAGAGCTAGCAGTGCTGCTTGTGGCATGCGGTGAATCA

**Positions**	**-100 bp flanking sequences**	**SNPs within *desi* cultivar**	**+100 bp flanking sequences**
Ca_LG_3:19643788	TTGGTGAGGGTTATGATCCTTTAAATAGTTCATCAAAATGAAGAGGAGAGATATGTGTGAGAATTTCAATGGCGGCAATGTCTAGAGTGGAAGAAGACTA	A/G	CATCAGGAAGAAGACTGTGATGCTGATTTTTAATTTGTGGTGGGGGTGCAGCTAGCGATTACCTAGGTGCCAAAAGTAGCAGATTAAAAGACTTAAAATG
Ca_LG_3:19643806	CTTTAAATAGTTCATCAAAATGAAGAGGAGAGATATGTGTGAGAATTTCAATGGCGGCAATGTCTAGAGTGGAAGAAGACTACATCAGGAAGAAGACTGT	T/C	GATGCTGATTTTTAATTTGTGGTGGGGGTGCAGCTAGCGATTACCTAGGTGCCAAAAGTAGCAGATTAAAAGACTTAAAATGGAGTGGGCCAAAGTCCAT
Ca_LG_3:20663037	TCCACGATTAAATAATATTTCATTCTGGTTGTTGCCGCCAAGTCTCTTGCTCCTATTAAGCTCAGCCTTAGTAGAGGTGGGTAGCGGCACTGGGTGGACA	A/G	GTCTATCCGCCCTTAAGTGGTATTACCAGCCATTCTGGAGGAGCAGTTGATTCAGCAATTTCTAGTCTTCATCTATCTGGTGTTTCATCCATTTTAGGTT
Ca_LG_3:20663045	TAAATAATATTTCATTCTGGTTGTTGCCGCCAAGTCTCTTGCTCCTATTAAGCTCAGCCTTAGTAGAGGTGGGTAGCGGCACTGGGTGGACAGTCTATCC	C/T	GCCCTTAAGTGGTATTACCAGCCATTCTGGAGGAGCAGTTGATTCAGCAATTTCTAGTCTTCATCTATCTGGTGTTTCATCCATTTTAGGTTCTATCAAT
Ca_LG_3:20877268	CACATGGAACTTGACATTTTCTTTGTCAAATATATCGTTCTTAGCAAGTCCTTAATTGTTTCCTATGTTCCTTTATGAAGCTGCTTCCCAATTCTTAGTT	T/G	CTATACTTTATGAAACAGACTCATGGAGGGTTCTAAGCATCGACCAGCTCCCAACCTGAGTTTGTGGGGGGACTGATAATGTATATAGAGTACTTAAACT
Ca_LG_3:20877282	CATTTTCTTTGTCAAATATATCGTTCTTAGCAAGTCCTTAATTGTTTCCTATGTTCCTTTATGAAGCTGCTTCCCAATTCTTAGTTCTATACTTTATGAA	A/G	ACAGACTCATGGAGGGTTCTAAGCATCGACCAGCTCCCAACCTGAGTTTGTGGGGGGACTGATAATGTATATAGAGTACTTAAACTCTTTAATATTCTAT
Ca_LG_3:20877369	TATACTTTATGAAACAGACTCATGGAGGGTTCTAAGCATCGACCAGCTCCCAACCTGAGTTTGTGGGGGGACTGATAATGTATATAGAGTACTTAAACTC	C/G	TTTAATATTCTATTGCATTGTATTGCTTCCAGCTCAGCAGCCAGCTGTATGCCAGCTCATCAGTTAGTTACGGCTGTCTCTTTTTGTTTAAGGCAGTTAC
Ca_LG_3:21245355	ATCCCTTAACAGAGTTATGAATTGTTTTAAAGGATTGAGATTTATTAGCAACTATTATTTCCTCTTTTTTATTTTTCCAATGCTTGTAGCAGCATATTGA	A/T	TCCTTTTCTATTTTTTATTTCCTTCATTTTCTGTCATAGCACTGAAACAAGTCTCATGATAAGTTTAATGTTGCTGTTTGGTCTGAACTAAGAGTGGTTG
Ca_LG_3:21245410	TATTTCCTCTTTTTTATTTTTCCAATGCTTGTAGCAGCATATTGATCCTTTTCTATTTTTTATTTCCTTCATTTTCTGTCATAGCACTGAAACAAGTCTC	C/T	ATGATAAGTTTAATGTTGCTGTTTGGTCTGAACTAAGAGTGGTTGCAAATTGAAAGTTTACTTTGTAGGATTGTTATGACATGTAGAATTTAACACAAGT
Ca_LG_3:21245412	TTTCCTCTTTTTTATTTTTCCAATGCTTGTAGCAGCATATTGATCCTTTTCTATTTTTTATTTCCTTCATTTTCTGTCATAGCACTGAAACAAGTCTCAT	T/G	GATAAGTTTAATGTTGCTGTTTGGTCTGAACTAAGAGTGGTTGCAAATTGAAAGTTTACTTTGTAGGATTGTTATGACATGTAGAATTTAACACAAGTTA
Ca_LG_3:21245418	CTTTTTTATTTTTCCAATGCTTGTAGCAGCATATTGATCCTTTTCTATTTTTTATTTCCTTCATTTTCTGTCATAGCACTGAAACAAGTCTCATGATAAG	G/A	TTTAATGTTGCTGTTTGGTCTGAACTAAGAGTGGTTGCAAATTGAAAGTTTACTTTGTAGGATTGTTATGACATGTAGAATTTAACACAAGTTAGTTTAA
Ca_LG_3:21245426	TTTTTCCAATGCTTGTAGCAGCATATTGATCCTTTTCTATTTTTTATTTCCTTCATTTTCTGTCATAGCACTGAAACAAGTCTCATGATAAGTTTAATGT	T/C	TGCTGTTTGGTCTGAACTAAGAGTGGTTGCAAATTGAAAGTTTACTTTGTAGGATTGTTATGACATGTAGAATTTAACACAAGTTAGTTTAACATAATTG

Positions	-100 bp flanking sequences	SNPs within *desi* cultivar	+100 bp flanking sequences
Ca_LG_3:21245440	GTAGCAGCATATTGATCCTTTTCTATTTTTTATTTCCTTCATTTTCTGTCATAGCACTGAAACAAGTCTCATGATAAGTTTAATGTTGCTGTTTGGTCTG	G/A	AACTAAGAGTGGTTGCAAATTGAAAGTTTACTTTGTAGGATTGTTATGACATGTAGAATTTAACACAAGTTAGTTTAACATAATTGAAAATTGTAATGTC
Ca_LG_3:21786287	CTCTTGCACTTTCAACTTATGTTGATGCTAATTGGGGCAGCATTGTGGATACTAGAAGATCCACTACTGGTTTTTGCCTCTTCATTGGTGATGCTTTAGC	C/T	TTCTTGGAAGGCAAAAAAAAAAAAAACAACTTACAGTCTCTAAATCTTCTGTTGCAGCTAAGTATAGGGCATTGTCTTCTACTGCTAGTGAATTTGTTTGG
Ca_LG_3:21788117	CTCTCTAGAGCAAGAAAGTAAAGAATCCGAAGAAAACCCATGAAAGCAAACAACAAAAAGAATGAACCTGTGCGACCACCAGCACCAGTAACAAGCACAA	A/G	AACTCTTTGAATTTTCAGCAGCCATAGCAATAACAAGACTTGTTCTGATTCCAACCCTTTTCAGACCCTTATAATTCCTCAACGTTGCAAGCGAAATCAA
Ca_LG_3:22567722	GTTTCCACCTCTAACCCTAAGTCATTATTAGAATTGGAGCATCCCTGAATCCATGGTTTTGAATTAGCAATAGAAGTAGGGGCACCATCAACAACAACAA	A/G	CATGTTCCTTAAGAGCTTTGATGGAATGATTCTGCCTGTTAGATGCTGAAAAAGTTGGAGCACATTTTGATGACTTCTTTGAAAATATTACAGATTTTGA
Ca_LG_3:22567727	CACCTCTAACCCTAAGTCATTATTAGAATTGGAGCATCCCTGAATCCATGGTTTTGAATTAGCAATAGAAGTAGGGGCACCATCAACAACAACAACATGT	T/C	TCCTTAAGAGCTTTGATGGAATGATTCTGCCTGTTAGATGCTGAAAAAGTTGGAGCACATTTTGATGACTTCTTTGAAAATATTACAGATTTTGAAGGAG
Ca_LG_3:22618579	TAGAATAACTAATTGGTGTCAATCGATTTATTCGGTGTCAATCGATTTATTCTAAGGTGTTTCTAGTGCTATTAGAATTGTGAATATTTTGTTTCCTACA	A/G	TGCGTCGCTCTTATTCTTTTTGAGGCTAAATGTTTGAGCGGCGGCTATCAGGCAAGGGTTTGTAATAGAGGAAAATAGTATGTTCTAGCACAATTGCCTA
Ca_LG_3:22618582	AATAACTAATTGGTGTCAATCGATTTATTCGGTGTCAATCGATTTATTCTAAGGTGTTTCTAGTGCTATTAGAATTGTGAATATTTTGTTTCCTACATGC	C/T	GTCGCTCTTATTCTTTTTGAGGCTAAATGTTTGAGCGGCGGCTATCAGGCAAGGGTTTGTAATAGAGGAAAATAGTATGTTCTAGCACAATTGCCTAATA
Ca_LG_3:22618586	ACTAATTGGTGTCAATCGATTTATTCGGTGTCAATCGATTTATTCTAAGGTGTTTCTAGTGCTATTAGAATTGTGAATATTTTGTTTCCTACATGCGTCG	G/A	CTCTTATTCTTTTTGAGGCTAAATGTTTGAGCGGCGGCTATCAGGCAAGGGTTTGTAATAGAGGAAAATAGTATGTTCTAGCACAATTGCCTAATAATAC
Ca_LG_3:22672155	AATAGGAGCTGGATCAGACGTATTTCCTGATAAGCAACTCAATCCTGAAGCAGCATACAAAGAAATGGACACCATGAAACTTAATTTGATATAATAATCT	T/C	CTTGCTTAACTTTATGATATAATAAAAATAATAAAATTTCAGCTATGTTTTGTTCTCTTATTCTTGCACTTGAATGGACAGTGCAGGCAATCAAATAAAT
Ca_LG_3:22672159	GGAGCTGGATCAGACGTATTTCCTGATAAGCAACTCAATCCTGAAGCAGCATACAAAGAAATGGACACCATGAAACTTAATTTGATATAATAATCTCTTG	G/A	CTTAACTTTATGATATAATAAAAATAATAAAATTTCAGCTATGTTTTGTTCTCTTATTCTTGCACTTGAATGGACAGTGCAGGCAATCAAATAAATAATC
Ca_LG_3:22672188	GCAACTCAATCCTGAAGCAGCATACAAAGAAATGGACACCATGAAACTTAATTTGATATAATAATCTCTTGCTTAACTTTATGATATAATAAAAATAATA	A/T	AAATTTCAGCTATGTTTTGTTCTCTTATTCTTGCACTTGAATGGACAGTGCAGGCAATCAAATAAATAATCTCAGGAGGACAAATAGAAAAGGGGCAATT
Ca_LG_3:22715307	AAAAGAACTGTCAATCACATTGGCACCATTAGGGGCAATTTTTTGCTCTCCTATTTGTCTCTCTTTTAACCCCTTCTGTCGGGATACTACTACTGCTGCT	T/C	ATCTCTTTTTAGGCCTTATTGTGACAAATATCATTGATGCGGTGTGTAAGTTGAGTTTGAGTTTGTTAAATAGCTCCATATATGGTTTTCTTTAATTGTG

Positions	-100 bp flanking sequences	SNPs within *desi* cultivar	+100 bp flanking sequences
Ca_LG_3:23082584	CAGAAATTGGAAATTGCGGAAAATATATATTTATTTATATATTTTTATTTATTTAATATATTCCCATGGATCTTAGGCTATATGGCAGCATCTAATTTAC	C/G	CCCTAAACAAAGATTAGGTGTGAAGATGTCATGACGCTGCTCTTTTTCTTTAATTAGTTGCACCAATGTTATTGGTCACACTTTCAAAAACAAATTGTCC
Ca_LG_3:23135840	ATCGTAGACTAGCAGCCATGCCTTGAATTATCTTCTCCAATTTACTCTTTCTTCATCAATGGACTTCATTGCGCTAGTTATCACACATTGCAAGTATCAA	A/T	CTCTGCAGCTCCTTTCCAATGCACATCGACTTTGTTCTCATTTTCCTTCCTTATCGCAACACCACTCCGTTTCTTCTCCGAGTTAAATGTTTCAACATGA
Ca_LG_3:23233656	AATTACCAGTGGTGCGAGAACGGTGAGAACGACGGTGAGTGCAGCTCGGGTTCGACGGCGCGACTGGTTCGACGGCGAGTGCAGCTCCGGTTCGACGGCG	G/A	AGTGCGCGACTGGTTTGACGGCGCGACTGGTTCGACGGTGAGTGCAGCTGGTTGATAGACGACGAGAATGGTGAGAACGACCGTTTTAAGTGAGAACGGC
Ca_LG_3:23233672	AGAACGGTGAGAACGACGGTGAGTGCAGCTCGGGTTCGACGGCGCGACTGGTTCGACGGCGAGTGCAGCTCCGGTTCGACGGCGAGTGCGCGACTGGTTT	T/C	GACGGCGCGACTGGTTCGACGGTGAGTGCAGCTGGTTGATAGACGACGAGAATGGTGAGAACGACCGTTTTAAGTGAGAACGGCGAGAAGGAGAAGACAG
Ca_LG_3:23818016	ATGAATGGGAAAATGGTGTTGGACAATCGGAGGAGAGCGCAAATAACGCATGGGCAGCAACACAACAACATTTTAGCCGCAACAACATTCGATGGAAAAA	A/G	GATTGAGAGTGAATCGTCTAAAGTGTAATATTGAAGCTGCTTTCTCTTTTCAGCACAAAATATTAAACGCATTAATAAAATAAATTTTAATTTAAATATT
Ca_LG_3:23888880	AAAATTAAGTAATTAACTTACATGCTTGATATGCTGTCATTTTAGTTGTCATTTTTCTGCAGTTCAGATCCCTGTGATTTGAACATTTGCTGCTGTGATG	G/T	ATGAGGATCGGAGAACCAAAAACAACTGCACTTATCTTTGCATCTGGCAAAATGGTATCTTTGCATTTGGCGTTTTATTGCAATAGCTAAATAGTTTAGT
Ca_LG_3:23888913	CTGTCATTTTAGTTGTCATTTTTCTGCAGTTCAGATCCCTGTGATTTGAACATTTGCTGCTGTGATGATGAGGATCGGAGAACCAAAAACAACTGCACTT	T/C	ATCTTTGCATCTGGCAAAATGGTATCTTTGCATTTGGCGTTTTATTGCAATAGCTAAATAGTTTAGTCCTTTTACTGTTGTTGGCTACCTTGGTTTTAGT
Ca_LG_3:23888922	TAGTTGTCATTTTTCTGCAGTTCAGATCCCTGTGATTTGAACATTTGCTGCTGTGATGATGAGGATCGGAGAACCAAAAACAACTGCACTTATCTTTGCA	A/G	TCTGGCAAAATGGTATCTTTGCATTTGGCGTTTTATTGCAATAGCTAAATAGTTTAGTCCTTTTACTGTTGTTGGCTACCTTGGTTTTAGTTGGGTGCCT
Ca_LG_3:23888931	TTTTTCTGCAGTTCAGATCCCTGTGATTTGAACATTTGCTGCTGTGATGATGAGGATCGGAGAACCAAAAACAACTGCACTTATCTTTGCATCTGGCAAA	A/G	ATGGTATCTTTGCATTTGGCGTTTTATTGCAATAGCTAAATAGTTTAGTCCTTTTACTGTTGTTGGCTACCTTGGTTTTAGTTGGGTGCCTTAAATTCCA
Ca_LG_3:24333072	GATCAAACGAAGCAAGTTTGCTACACACATGTTACCACCACCGGAACCAAACCCTTATTTTTCTTCTCTGGCAGTCGTTCTCTTTGTTCTTCTACATTCA	A/G	GATGTAACATCCGAGCCTAGGAAGGAAGAGGGAAGGCAAGGGTCCCAGGAGCTATCAGTATTAGCTGCTGACTGGTGACGAGAGGATCATATAGTTTGAG
Ca_LG_3:24735898	TCCTATTTTAGTTGAATCGCGAAAGTAGTCAACTTATTCTAGCAAAAGCGATTCCCATAGTAGCGGCGAGCGAAATGAGAACAGCCTAAACCGTGAAAAT	T/C	GGGATTGTGGGAGAGCAATACAAGCGTTGTGTTGTTAGGCTTTCTCTCCAGTTTTGGTCCAAAACTTGATAAAATATCATTTTTTTAAGCCACACCACAT
Ca_LG_3:24735902	ATTTTAGTTGAATCGCGAAAGTAGTCAACTTATTCTAGCAAAAGCGATTCCCATAGTAGCGGCGAGCGAAATGAGAACAGCCTAAACCGTGAAAATGGGA	A/G	TTGTGGGAGAGCAATACAAGCGTTGTGTTGTTAGGCTTTCTCTCCAGTTTTGGTCCAAAACTTGATAAAATATCATTTTTTTAAGCCACACCACATCATT

Positions	-100 bp flanking sequences	SNPs within *desi* cultivar	+100 bp flanking sequences
Ca_LG_3:24942309	CTAGAAGAAACTAATCTTCAATGTTTTCATTAACAGCAATGGATCAAATGTTCGTGGTTACTTCATTTGGGCAGCATTTGATACATTTGAATTTCAAGCG	G/A	GGATATTCAGGACATTGGGGACTTTATCATGTTGATTTTAATGATAATCTCAAGCGTATACCAAAGGACTCTGCTGAATGGTATAGAAAGTTTCTGATAC
Ca_LG_3:24942312	GAAGAAACTAATCTTCAATGTTTTCATTAACAGCAATGGATCAAATGTTCGTGGTTACTTCATTTGGGCAGCATTTGATACATTTGAATTTCAAGCGGGA	A/C	TATTCAGGACATTGGGGACTTTATCATGTTGATTTTAATGATAATCTCAAGCGTATACCAAAGGACTCTGCTGAATGGTATAGAAAGTTTCTGATACCAG
Ca_LG_3:24942318	ACTAATCTTCAATGTTTTCATTAACAGCAATGGATCAAATGTTCGTGGTTACTTCATTTGGGCAGCATTTGATACATTTGAATTTCAAGCGGGATATTCA	A/T	GGACATTGGGGACTTTATCATGTTGATTTTAATGATAATCTCAAGCGTATACCAAAGGACTCTGCTGAATGGTATAGAAAGTTTCTGATACCAGAGTGTG
Ca_LG_3:24942322	ATCTTCAATGTTTTCATTAACAGCAATGGATCAAATGTTCGTGGTTACTTCATTTGGGCAGCATTTGATACATTTGAATTTCAAGCGGGATATTCAGGAC	C/A	ATTGGGGACTTTATCATGTTGATTTTAATGATAATCTCAAGCGTATACCAAAGGACTCTGCTGAATGGTATAGAAAGTTTCTGATACCAGAGTGTGGACA
Ca_LG_3:25396854	TCCCCCATCGTCTCACTGGCCGACCTTGACCCTTCTTATTTTGGGGTCATATCTAGTATTCAGAGTTTGCCTCGATTTGGTACCGCTCTCGCGGCCCGCA	A/G	CTGAAACAGTGCTTTACCCCTAGATGTCCAGTCAACTGCTGCGCCTCAACGCATTTCGGGGAGAACCAGCTAGCTCTGGGTTCGAGTGGCATTTCACCGC
Ca_LG_3:25396856	CCCCATCGTCTCACTGGCCGACCTTGACCCTTCTTATTTTGGGGTCATATCTAGTATTCAGAGTTTGCCTCGATTTGGTACCGCTCTCGCGGCCCGCACT	T/C	GAAACAGTGCTTTACCCCTAGATGTCCAGTCAACTGCTGCGCCTCAACGCATTTCGGGGAGAACCAGCTAGCTCTGGGTTCGAGTGGCATTTCACCGCTA
Ca_LG_3:25419260	CAACGACAATGGGGATGATCAAATGGCTGAAGCAATGAATAACATGGTTGCTTCTGTTGCTGCACAGACTACTGCATAGACTCTGCGAGATCTGGAGAAA	A/G	AGGGAAATAGAGATATGTGCTGTTGCGTCAAGGGGATTAGAAGATTTTAGTCGTTATCATCCTCCAAAGTTCAAGGGTGATGAGAACTCAGAAAACGCGG
Ca_LG_3:27326825	TAAAATTATTTTAAAAATTAAATTTCATAATTATTGAAACAAATTCTTTTTTTAATTGTGATAGTTAATTTAAAACAACTAATATCATTTGTAAAATCTT	T/C	CATCTTAAAATCAATACTACCTACTAGTACTAGTAGTGGTTGGTGTATCATAAATTCTAATAGCAACCTTTCTATAGTTGCCAGTTTCATTTATTAAATC
Ca_LG_3:27326866	AATTCTTTTTTTAATTGTGATAGTTAATTTAAAACAACTAATATCATTTGTAAAATCTTCATCTTAAAATCAATACTACCTACTAGTACTAGTAGTGGTT	T/A	GGTGTATCATAAATTCTAATAGCAACCTTTCTATAGTTGCCAGTTTCATTTATTAAATCCAAATTATTAATCATTAAAATATGCATAGAGTAGTAGTATA
Ca_LG_3:27394533	AGGCGTCGATGGAGCCAGTCCATCTACTGCTATCAGAACAGTAGGTCTCGAAAGAGTTCAAATGATGACGATACATCTTGCATCTGTTGGAGAAACCTAT	T/C	GCTCAGGCCAGCTTCGATGAAGCTGCTTTAAAGATATCGCGACCGACTCTACTGAAAGATACCCCAAAAGATGTATGAAATTTCTTTTAAAAAAGCCCTC
Ca_LG_3:27394549	AGTCCATCTACTGCTATCAGAACAGTAGGTCTCGAAAGAGTTCAAATGATGACGATACATCTTGCATCTGTTGGAGAAACCTATGCTCAGGCCAGCTTCG	G/C	ATGAAGCTGCTTTAAAGATATCGCGACCGACTCTACTGAAAGATACCCCAAAAGATGTATGAAATTTCTTTTAAAAAAGCCCTCTAATTATTGTTGATTT
Ca_LG_3:27875755	ATCGATAAGAGCAACAACATCAGTGGTGATTATGTCCACATATTTATTTATGAAAATCCCAAGACCTCTTATTAGGTAGAAGTCCAATCCTATAGCTTCC	C/T	TCAATGCTAGGACGTTGCACCTTGACAGCCACGAGTTTACCCGAATACTTCAACCGAGCTTTATAAACTTGACCTAAACTAGCTGCAGCCATAGGTGATG

Positions	-100 bp flanking sequences	SNPs within *desi* cultivar	+100 bp flanking sequences
Ca_LG_3:27875758	GATAAGAGCAACAACATCAGTGGTGATTATGTCCACATATTTATTTATGAAAATCCCAAGACCTCTTATTAGGTAGAAGTCCAATCCTATAGCTTCCTCA	A/G	ATGCTAGGACGTTGCACCTTGACAGCCACGAGTTTACCCGAATACTTCAACCGAGCTTTATAAACTTGACCTAAACTAGCTGCAGCCATAGGTGATGGTG
Ca_LG_3:27875763	GAGCAACAACATCAGTGGTGATTATGTCCACATATTTATTTATGAAAATCCCAAGACCTCTTATTAGGTAGAAGTCCAATCCTATAGCTTCCTCAATGCT	T/C	AGGACGTTGCACCTTGACAGCCACGAGTTTACCCGAATACTTCAACCGAGCTTTATAAACTTGACCTAAACTAGCTGCAGCCATAGGTGATGGTGTTATA
Ca_LG_3:27875767	AACAACATCAGTGGTGATTATGTCCACATATTTATTTATGAAAATCCCAAGACCTCTTATTAGGTAGAAGTCCAATCCTATAGCTTCCTCAATGCTAGGA	A/G	CGTTGCACCTTGACAGCCACGAGTTTACCCGAATACTTCAACCGAGCTTTATAAACTTGACCTAAACTAGCTGCAGCCATAGGTGATGGTGTTATAGAAG
Ca_LG_3:27875785	TATGTCCACATATTTATTTATGAAAATCCCAAGACCTCTTATTAGGTAGAAGTCCAATCCTATAGCTTCCTCAATGCTAGGACGTTGCACCTTGACAGCC	C/A	ACGAGTTTACCCGAATACTTCAACCGAGCTTTATAAACTTGACCTAAACTAGCTGCAGCCATAGGTGATGGTGTTATAGAAGAAAAAATGGAGTCAAGGG
Ca_LG_3:27875788	GTCCACATATTTATTTATGAAAATCCCAAGACCTCTTATTAGGTAGAAGTCCAATCCTATAGCTTCCTCAATGCTAGGACGTTGCACCTTGACAGCCACG	G/A	AGTTTACCCGAATACTTCAACCGAGCTTTATAAACTTGACCTAAACTAGCTGCAGCCATAGGTGATGGTGTTATAGAAGAAAAAATGGAGTCAAGGGATA
Ca_LG_3:27875794	ATATTTATTTATGAAAATCCCAAGACCTCTTATTAGGTAGAAGTCCAATCCTATAGCTTCCTCAATGCTAGGACGTTGCACCTTGACAGCCACGAGTTTA	A/C	CCCGAATACTTCAACCGAGCTTTATAAACTTGACCTAAACTAGCTGCAGCCATAGGTGATGGTGTTATAGAAGAAAAAATGGAGTCAAGGGATAATCCTA
Ca_LG_3:27875797	TTTATTTATGAAAATCCCAAGACCTCTTATTAGGTAGAAGTCCAATCCTATAGCTTCCTCAATGCTAGGACGTTGCACCTTGACAGCCACGAGTTTACCC	C/A	GAATACTTCAACCGAGCTTTATAAACTTGACCTAAACTAGCTGCAGCCATAGGTGATGGTGTTATAGAAGAAAAAATGGAGTCAAGGGATAATCCTAATT
Ca_LG_3:27875800	ATTTATGAAAATCCCAAGACCTCTTATTAGGTAGAAGTCCAATCCTATAGCTTCCTCAATGCTAGGACGTTGCACCTTGACAGCCACGAGTTTACCCGAA	A/G	TACTTCAACCGAGCTTTATAAACTTGACCTAAACTAGCTGCAGCCATAGGTGATGGTGTTATAGAAGAAAAAATGGAGTCAAGGGATAATCCTAATTCCC
Ca_LG_3:27875802	TTATGAAAATCCCAAGACCTCTTATTAGGTAGAAGTCCAATCCTATAGCTTCCTCAATGCTAGGACGTTGCACCTTGACAGCCACGAGTTTACCCGAATA	A/T	CTTCAACCGAGCTTTATAAACTTGACCTAAACTAGCTGCAGCCATAGGTGATGGTGTTATAGAAGAAAAAATGGAGTCAAGGGATAATCCTAATTCCCTT
Ca_LG_3:27875824	TATTAGGTAGAAGTCCAATCCTATAGCTTCCTCAATGCTAGGACGTTGCACCTTGACAGCCACGAGTTTACCCGAATACTTCAACCGAGCTTTATAAACT	T/C	TGACCTAAACTAGCTGCAGCCATAGGTGATGGTGTTATAGAAGAAAAAATGGAGTCAAGGGATAATCCTAATTCCCTTTCAATGCATGCAAAGGCCTCCT
Ca_LG_3:28434842	CTGCTCTCATGGTTCTTCAAATACAAATTGAAGGAGAGAGCATTAACTTTCATTACTTTGATTCACAAGGTTCAATATTGTATCTTCCATCAGTTCTGCA	A/T	AGAATAGCGTCGCCCATTTCAAAACCAACTGTTTCAGCATCGAATCGAAGGTCCATCCATGTTCCATTTTTTGCCAAGTCTTTCTTAACAATTTGTTCCA
Ca_LG_3:28434848	TCATGGTTCTTCAAATACAAATTGAAGGAGAGAGCATTAACTTTCATTACTTTGATTCACAAGGTTCAATATTGTATCTTCCATCAGTTCTGCAAGAATA	A/G	GCGTCGCCCATTTCAAAACCAACTGTTTCAGCATCGAATCGAAGGTCCATCCATGTTCCATTTTTTGCCAAGTCTTTCTTAACAATTTGTTCCAAAGTAT

Positions	-100 bp flanking sequences	SNPs within *desi* cultivar	+100 bp flanking sequences
Ca_LG_3:28434849	CATGGTTCTTCAAATACAAATTGAAGGAGAGAGCATTAACTTTCATTACTTTGATTCACAAGGTTCAATATTGTATCTTCCATCAGTTCTGCAAGAATAG	G/A	CGTCGCCCATTTCAAAACCAACTGTTTCAGCATCGAATCGAAGGTCCATCCATGTTCCATTTTTTGCCAAGTCTTTCTTAACAATTTGTTCCAAAGTATG
Ca_LG_3:28434851	TGGTTCTTCAAATACAAATTGAAGGAGAGAGCATTAACTTTCATTACTTTGATTCACAAGGTTCAATATTGTATCTTCCATCAGTTCTGCAAGAATAGCG	G/A	TCGCCCATTTCAAAACCAACTGTTTCAGCATCGAATCGAAGGTCCATCCATGTTCCATTTTTTGCCAAGTCTTTCTTAACAATTTGTTCCAAAGTATGCG
Ca_LG_3:28434857	TTCAAATACAAATTGAAGGAGAGAGCATTAACTTTCATTACTTTGATTCACAAGGTTCAATATTGTATCTTCCATCAGTTCTGCAAGAATAGCGTCGCCC	C/T	ATTTCAAAACCAACTGTTTCAGCATCGAATCGAAGGTCCATCCATGTTCCATTTTTTGCCAAGTCTTTCTTAACAATTTGTTCCAAAGTATGCGGTGGAG
Ca_LG_3:28434878	AGAGCATTAACTTTCATTACTTTGATTCACAAGGTTCAATATTGTATCTTCCATCAGTTCTGCAAGAATAGCGTCGCCCATTTCAAAACCAACTGTTTCA	A/G	GCATCGAATCGAAGGTCCATCCATGTTCCATTTTTTGCCAAGTCTTTCTTAACAATTTGTTCCAAAGTATGCGGTGGAGGCAAAGGAAGGACATGCCAGT
Ca_LG_3:28434881	GCATTAACTTTCATTACTTTGATTCACAAGGTTCAATATTGTATCTTCCATCAGTTCTGCAAGAATAGCGTCGCCCATTTCAAAACCAACTGTTTCAGCA	A/T	TCGAATCGAAGGTCCATCCATGTTCCATTTTTTGCCAAGTCTTTCTTAACAATTTGTTCCAAAGTATGCGGTGGAGGCAAAGGAAGGACATGCCAGTGCA
Ca_LG_3:28434884	TTAACTTTCATTACTTTGATTCACAAGGTTCAATATTGTATCTTCCATCAGTTCTGCAAGAATAGCGTCGCCCATTTCAAAACCAACTGTTTCAGCATCG	G/A	AATCGAAGGTCCATCCATGTTCCATTTTTTGCCAAGTCTTTCTTAACAATTTGTTCCAAAGTATGCGGTGGAGGCAAAGGAAGGACATGCCAGTGCACTC
Ca_LG_3:28966924	AACAATTTTTTTGAGGTACAATCTAGTACGAAAGGTGTTACACAATGAAAACTTGCTTATATAATGAAAATAAATCACCTCAGCAGCCTCTGAAGTTGAT	T/A	GAAAGATACTGATAATAATAATGCCGCAACGCATCAGGTACGGATGCAGGTGTGTTGTGCCAAATGTCCAAGTTCTGGTCAGTAACCTGCAGCAATAGTT
Ca_LG_3:28966973	AACTTGCTTATATAATGAAAATAAATCACCTCAGCAGCCTCTGAAGTTGATGAAAGATACTGATAATAATAATGCCGCAACGCATCAGGTACGGATGCAG	G/A	GTGTGTTGTGCCAAATGTCCAAGTTCTGGTCAGTAACCTGCAGCAATAGTTATAGTATAGAATAAGAAATCACTAAAATTAAGCACCAAATAGGAAGGGC
Ca_LG_3:28967003	TCAGCAGCCTCTGAAGTTGATGAAAGATACTGATAATAATAATGCCGCAACGCATCAGGTACGGATGCAGGTGTGTTGTGCCAAATGTCCAAGTTCTGGT	T/C	CAGTAACCTGCAGCAATAGTTATAGTATAGAATAAGAAATCACTAAAATTAAGCACCAAATAGGAAGGGCAAAACCATCACTAAAATTGTATCCATGTCC
Ca_LG_3:29822260	TATTTTCATATTGTTACTACATATAATTGTCTTGTTACCGTAAGATTTTGAACGAAGTCTCTAGATTGATTAAGAAATAATTTTCTTCGCCATTAAGCAA	A/G	CATCCTTTAAGTGAGTAGGGGTGCGTCGGACTTCCGACGAATAATAGATACCCCCACACCCCAGGCAGCATTAGTGAGCCGTGAAATAGACCATTCTTCA
Ca_LG_3:29822286	TTGTCTTGTTACCGTAAGATTTTGAACGAAGTCTCTAGATTGATTAAGAAATAATTTTCTTCGCCATTAAGCAACATCCTTTAAGTGAGTAGGGGTGCGT	T/C	CGGACTTCCGACGAATAATAGATACCCCCACACCCCAGGCAGCATTAGTGAGCCGTGAAATAGACCATTCTTCACCTAAATGACTTAATTCACTATCAAT
Ca_LG_3:29822292	TGTTACCGTAAGATTTTGAACGAAGTCTCTAGATTGATTAAGAAATAATTTTCTTCGCCATTAAGCAACATCCTTTAAGTGAGTAGGGGTGCGTCGGACT	T/C	TCCGACGAATAATAGATACCCCCACACCCCAGGCAGCATTAGTGAGCCGTGAAATAGACCATTCTTCACCTAAATGACTTAATTCACTATCAATGAATGA

Positions	-100 bp flanking sequences	SNPs within *desi* cultivar	+100 bp flanking sequences
Ca_LG_3:29822293	GTTACCGTAAGATTTTGAACGAAGTCTCTAGATTGATTAAGAAATAATTTTCTTCGCCATTAAGCAACATCCTTTAAGTGAGTAGGGGTGCGTCGGACTT	T/G	CCGACGAATAATAGATACCCCCACACCCCAGGCAGCATTAGTGAGCCGTGAAATAGACCATTCTTCACCTAAATGACTTAATTCACTATCAATGAATGAC
Ca_LG_3:29822308	TGAACGAAGTCTCTAGATTGATTAAGAAATAATTTTCTTCGCCATTAAGCAACATCCTTTAAGTGAGTAGGGGTGCGTCGGACTTCCGACGAATAATAGA	A/G	TACCCCCACACCCCAGGCAGCATTAGTGAGCCGTGAAATAGACCATTCTTCACCTAAATGACTTAATTCACTATCAATGAATGACATACACATAAGGAAT
Ca_LG_3:29822329	TTAAGAAATAATTTTCTTCGCCATTAAGCAACATCCTTTAAGTGAGTAGGGGTGCGTCGGACTTCCGACGAATAATAGATACCCCCACACCCCAGGCAGC	C/A	ATTAGTGAGCCGTGAAATAGACCATTCTTCACCTAAATGACTTAATTCACTATCAATGAATGACATACACATAAGGAATACAAGGTTGGTTTGGTGGTTG
Ca_LG_3:29822330	TAAGAAATAATTTTCTTCGCCATTAAGCAACATCCTTTAAGTGAGTAGGGGTGCGTCGGACTTCCGACGAATAATAGATACCCCCACACCCCAGGCAGCA	A/G	TTAGTGAGCCGTGAAATAGACCATTCTTCACCTAAATGACTTAATTCACTATCAATGAATGACATACACATAAGGAATACAAGGTTGGTTTGGTGGTTGA
Ca_LG_3:29822344	CTTCGCCATTAAGCAACATCCTTTAAGTGAGTAGGGGTGCGTCGGACTTCCGACGAATAATAGATACCCCCACACCCCAGGCAGCATTAGTGAGCCGTGA	A/T	AATAGACCATTCTTCACCTAAATGACTTAATTCACTATCAATGAATGACATACACATAAGGAATACAAGGTTGGTTTGGTGGTTGATAAGCAACAAACTT
Ca_LG_3:30020584	AAAGAAAGATGAAGAGGAAAGAAGAACAGAGATTGCACAGAGCAGAGGTGCATGCAGCAATGTCAATTGCAAGTGTTGCAGCAACACTTTCTGCTATTGC	C/T	TTCTGAAAATTCAAAAAAGGAGGAATCAATGAAGATAGAGATGCAACAATAGCTTCTGCTTCTATTTTGGTTGCTGTCCAGTGTGCAATAGTAGCTGAAG
Ca_LG_3:32243619	ATGCGCACGTGGCATTTGTGGCTGCAAAGGCCGCCACAAAGGAGACCCAATACAGCTGGCGTTTGTGGCCGCTTAGGCCGTCACAAACGTGATGTAACCG	G/A	TTGGCATTGTGGCTGCTTTGGCCGCCACAAATGCTCAACGTGACCCTATATATATATTTTTCTCCCCTCCTCCTTCATTTTTTCACATCCAATTTCTCTCTA
Ca_LG_3:32785958	TACATCAGTAATGAGTGGATTAAGCTTGGAAATCTCATTTGTTAAGTAGCAGCATGTGCTAATGTTTTGGTGCAAGATTGGTGATAGGTTTTTGTTTTTT	T/C	TTCGTGCTAGCAATTTTGTGGTGCTAGTTTTTTTTAGCAGCAGCATGTGCTCTAGGTTTAGACGAGTGATGGAGTTTCGTATTTTGTTCCCTGGACTCTA
Ca_LG_3:33631943	GCCAAGCTTGATGAGAAGAAAAATATTAGGGATTTGATTTCTAGTGCTGCTGGCGGTGCAGCTCCAGCCGCTGCCGCAGCCTCAGCCGCTGCCGCAGCCC	C/T	CAGCCGCTGCCGCCAGCCGCTGCCGCTGCTCCAGCAGCTGAGGAGAAGAAGAAGGTAATCCCTTAGTGAAATTTATTTTCCTTTTCTTTTCAACTTTGAC
Ca_LG_3:33815213	TATCAAATCACCGTTCCTCACTGTAATTCCTCCAAACTCACTATTAATCCAACATGCAGCACGAATTACCGGAATCAAAGATTGGACAGAGGGCAACTCG	G/A	GAGGAACTAACTGTATTGGTACACACATTGGATGTCAAACAGCTGCAGCAACTGACACAATTAATTAATTAATTAATTAATTAATCAATCAATTATACAT
Ca_LG_3:33926065	CATGTAGCATTCAGCAAGGAATCCTTGAGGGGATGCGCGGCGACCATCCACAATTTTTGAGAAGTGGCGGATGGCTTCTGAGTAGAGACCGGCGTCGAGG	G/A	GCAGCGAGTGCTGCTGTGCGTCGGCGGAGGAGGAATTTGATGTGTGTGAGGAGTTGTGTTACGCCTTCTGTGTAGTTGAGAGGGACTCGCGGTGGTGTGG
Ca_LG_3:34134919	CACTATTCGTTGGTTTGAACAAAGGATAGGCTAGGCCTCAAACATGATTTTAAGATCCTAGCTACGCCATACGCGTTCCATACATAATCTGGAAAGCTGT	T/C	AGGGATGACAAAAAGAGGGCGCTTTCATGTGTTGCACAGAACACAGATAAAAGAAAGAAACCTTACTAAATAATAGAGAAGATGGATCATATCTCCTTTA

Positions	-100 bp flanking sequences	SNPs within *desi* cultivar	+100 bp flanking sequences
Ca_LG_3:34134946	AGGCTAGGCCTCAAACATGATTTTAAGATCCTAGCTACGCCATACGCGTTCCATACATAATCTGGAAAGCTGTAGGGATGACAAAAAGAGGGCGCTTTCA	A/C	TGTGTTGCACAGAACACAGATAAAAGAAAGAAACCTTACTAAATAATAGAGAAGATGGATCATATCTCCTTTATCGAACACTAAATGGTAAATCCTACCT
Ca_LG_3:34337575	ATATCTATCTATCATAAAAATTGTTAGCAAAACAAAGAGAGAAAAAAATCGCTGACCAACCTGGATTGCAGCAGGTACAGGGGCTTTTTCTTTAAGCTCG	G/A	TGCACAGTGTTCGACTGCACTGCTATTGCTGCCTGAGATCAACATTCAAAAGCAATATAGAAACAAAAATCATTGACCAAATAATGGACACATGATGTTA
Ca_LG_3:34647021	GAGCTTGGCTCGACAAGCTTTGTGGGCAAGTGGTGCGGCAGCCTGTGATCCCTCCACTGTTAGGAGCCGAAGGTTTTCGCTGCTCTGTGAGTCCAGCCTT	T/C	ACGCTATACCCTCCAGTCTAGGAGCCAAGAGACCCGAGCGTTCGCCCAAGTCCCGTGACTCAACTCTCAAATTGGAGCGGGCAGGCTTGCACAGTGCAGC
Ca_LG_3:34647061	GCCTGTGATCCCTCCACTGTTAGGAGCCGAAGGTTTTCGCTGCTCTGTGAGTCCAGCCTTACGCTATACCCTCCAGTCTAGGAGCCAAGAGACCCGAGCG	G/C	TTCGCCCAAGTCCCGTGACTCAACTCTCAAATTGGAGCGGGCAGGCTTGCACAGTGCAGCCACCAACCCAACCAAACAAGCCAAAGCTATGTTCCATCAC
Ca_LG_3:34647092	GGTTTTCGCTGCTCTGTGAGTCCAGCCTTACGCTATACCCTCCAGTCTAGGAGCCAAGAGACCCGAGCGTTCGCCCAAGTCCCGTGACTCAACTCTCAAA	A/C	TTGGAGCGGGCAGGCTTGCACAGTGCAGCCACCAACCCAACCAAACAAGCCAAAGCTATGTTCCATCACCATTTTGCCCCCGCTTGGGTCACAAGTTGGA
Ca_LG_3:35916630	TGTGATGGTAGGAGCACTCCCAGCTGCATTGACTTACTACTGGCGGATGAAGATGCCCGAAACTGCTCGTTACACCGCTCTAGTTGCTAAGAACACGGCG	G/A	CAGGCTGCAGCAGATATGTCTAAGGTTCTGCAAGTCGAGATCCAAGCTGAACCAAAGACAAATGAGCAGAAAAAGTCTTATGGTTTATTCTCAAAGGAAT
Ca_LG_3:36501498	ATTTGCAATTCCAAATGGTGATTTCAATGTCCGCTAGGCCACTCAGAAATACAGATAACAGCTGCCATACCATCAAATTGCGTGCAAAGGGAGAGAACCG	G/A	TGGTCTGCAGAAAGGCAGCCATTAATGTCCCCAATCTGTACATAATTAAGTCGAGCCACTTTCATCCTAATTGTGCTTTAACTTTTATGTTTTATTCAAA
Ca_LG_3:36765441	TTGTCTTTCTTGCTTAATCTTTGGGGTTGTGAAAACTGAAAACTCTTCATATGTCATGCAGCAACAATTGCAATCTTTACTTGAAACTTTAAGTTCCACC	C/T	GAGCCACACTACATTCGATGTGTGAAGCCCAATAATCTTCTTAAGCCAGCTATTTTCGAGAATAAAAATGTTTTGCTGCAACTGCGCTGTGGGGTAAGAC
Ca_LG_3:36858079	GCAAGGTCCAAAGGGAACTCTGGAAGTTGTTCAGAATTTTGAGAAGGTAATGAAGTTGTTTTTCCAGCACTTGGATGATCCTCACCATAAAGTTGCGCAG	G/A	GCTGCTCTTTCAACACTAGCAGATATTGTTCCAACATGTCGAAAGCCTTTCGAGGGTTACATGGAAAGAATATTACCACATGTGTTTTCTCGGTTAATTG
Ca_LG_3:37471313	CATCGCTTCCACCGCCGCCTTCGACGCTGTGTACGCGCCGTAACCTGGCTTAAACGCCGCTGCTAATGAAGTCGTCAGAAGTATGATTCTCCCTCCTCCG	G/A	CCGCGCTTTAACCGGTTCGCTGCTTCCTTCCCGCACAGAAACGATCCCCTTGCGTTCACGTTCATGATGCGATCGAAGGTTTCAACGGTTGTGTTCGCTA
Ca_LG_3:39665887	CTAATCAGTATTGTCTGTTGTGAATGCTTGCCTGCCTTGCAGTGTGCTGTCGGATATTGAATAGAAACTTGAGATTACGGTCGTTAGGTTCCTGGTCTCC	C/G	GAGCAATATAACGTTATGTCTTGGTCAGCAGCTGGGCAGTCAGCATAACGTTTGGGCGACATAACGTTTGGGGACAGGTCTTTTCTGGAACCAAGTCTTC
Ca_LG_3:39837598	CTTTCTTCTTTTTCATTTTTATTTTTATTTTTATTTTTCCGATGCGATTCCAGCGCGATTTGCTGCTGCAAAAGAGAATTTCGTCGCAGCAGAGCAAAGT	T/C	TCCACATCGCACTGTGATTCTGCGATTGCGCGCTATAGCTGCGCCATTGAAAACATAGCTTTTCTGGCTATTTATAGTAATTGATTTTCCCATTACATGT

Positions	-100 bp flanking sequences	SNPs within *desi* cultivar	+100 bp flanking sequences
Ca_LG_3:39887505	TCAATTATCGAGACTCGGCCCAGGTTCTGCTACCTCCTCGAGGCTGGCTGCAATTAAGGGACTCTCTTTAGATGATGGAGCCAATAATGAAAAGTGCACG	G/A	GCAATCACTAATGCCGAGGCATTAAATCCAAAGTACGAGTTTCCGAACATGAAAACAGCTGCTATCATATCAGACCAGAAGACACTGAAGGTTCGAATCA
Ca_LG_3:39940982	CTCAATCATAAACTTTGCTGCGGATTTTCCTGCGGAGTTTGCATGGAATTTGCTGCGGAAATTTGCCGCAGATAAATCCTATGGAAAAAGATGAACTTTG	G/A	CTGCGGAAATATAGTCGTATGTAAATCCGCAGCAAAAGAAAACGTTACCTGCCGTTTTACCTGCGGATAGTGATACCGTAGGAAAAGTCTTAAGATAATT
Ca_LG_3:40071610	AATATCAAGTACCATGAAGATAAGAGCTAAGTCAATACAAACAAAATGGCAGCTAATCATTACAGTGTTACCTGCATAAAGCACACCCACTAAGCTCAGT	T/C	GTCAATGTCTTTGACTATGCGGATGCATCTCTTGGAGATCTGTTCAATAGGATCAACTCCTTCAACCCTTCCAGCAGCCCAAGCATTGACATTGTAGGCC
Ca_LG_3:40076986	CTTGACTGTTGTAGGCTTCTTATTGACCACAATCTTCTTCGACATCTTCATCTCCAACTTGTTTCTCGGAGCAGCTAATGCACCATTGATATCACTCGCC	C/A	ACAACAGCACCTTCTTGTTCTAACTCCGTCACCATCTCCTCACCAGACTCAGCATTCGTTTTCATGGCTGTCGATCGTGTAATCCGCTTGAACGTCTTCA
Ca_LG_3:40919804	ACATAAACAGATATGTAATTTAAAATCTTGTTTTCTAAATGGGGCAAACAAATTTCCATTTTTACATTTGGATGATGATAGTCACCAAAATGTGCAGCAC	C/T	AATACAACGTTAGCAACATCATAATTATCATTAGCATAGCTCTTAATTAGATATAGCTGGACTACATTCAACCTAAAATACATAGCACATAGCGCAGCTA
Ca_LG_3:40927037	ACAGTAAAGCTAACCTGTGACAAGTGAGAGCATGCAGCCTCAACTGCATTGGCAGACCATGACTTCCCAAGGGATTTTCTGACAATAAACCTCAAAGTCT	T/A	TGACTGGTAACAATCCTAACGATGCTATTAAGTAGCTAGCAGCAGCAGCTAGAATTTGTGATCTGCAATCGCATTAACCATTAGTCATTTAGAATAACAT
Ca_LG_3:40927058	AAGTGAGAGCATGCAGCCTCAACTGCATTGGCAGACCATGACTTCCCAAGGGATTTTCTGACAATAAACCTCAAAGTCTTGACTGGTAACAATCCTAACG	G/A	ATGCTATTAAGTAGCTAGCAGCAGCAGCTAGAATTTGTGATCTGCAATCGCATTAACCATTAGTCATTTAGAATAACATATAGAACTTTTAAATGAAAAT
Ca_LG_3:41429764	GATTGACGACTTACCAATCCAATGACGTTCGCACACAACATTTCAAAAAAGGGGTACTCTATTCTCGTAATTCCATAATAGATGCCTGTTGGCATGCCAA	A/G	CCTTCCTTCTCCTTTCATGACCTATCCGAAAACAAAGAAAATTCTTTACTTAATTAGTGATGAATATCGGAATAGGACAAACCATCCCGTGGATCTCTTT
Ca_LG_3:41429771	GACTTACCAATCCAATGACGTTCGCACACAACATTTCAAAAAAGGGGTACTCTATTCTCGTAATTCCATAATAGATGCCTGTTGGCATGCCAACCTTCCT	T/C	TCTCCTTTCATGACCTATCCGAAAACAAAGAAAATTCTTTACTTAATTAGTGATGAATATCGGAATAGGACAAACCATCCCGTGGATCTCTTTGTTTGCT
Ca_LG_3:41429774	TTACCAATCCAATGACGTTCGCACACAACATTTCAAAAAAGGGGTACTCTATTCTCGTAATTCCATAATAGATGCCTGTTGGCATGCCAACCTTCCTTCT	T/C	CCTTTCATGACCTATCCGAAAACAAAGAAAATTCTTTACTTAATTAGTGATGAATATCGGAATAGGACAAACCATCCCGTGGATCTCTTTGTTTGCTTCA
Ca_LG_3:41755896	TAGTTTACAATTCGTTGGGCACTAAGCAATATATTGAGAGCAGTGGTTTCAAGAGAGATGTGCCAAATTTGGAAGAAGTTGTTATTCAAGAAGGTGTTGT	T/C	TCACTTCAACAACCAAGAAGCTGCTGAGGATATCACCAATCACATTTATGAATTTATCAAGAAGTTCTGATTTTGTCACTTGTTTTAGTTAAAAATCATC
Ca_LG_3:41755903	CAATTCGTTGGGCACTAAGCAATATATTGAGAGCAGTGGTTTCAAGAGAGATGTGCCAAATTTGGAAGAAGTTGTTATTCAAGAAGGTGTTGTTCACTTC	C/T	AACAACCAAGAAGCTGCTGAGGATATCACCAATCACATTTATGAATTTATCAAGAAGTTCTGATTTTGTCACTTGTTTTAGTTAAAAATCATCTTCTAAT

**Positions**	**-100 bp flanking sequences**	**SNPs within *desi* cultivar**	**+100 bp flanking sequences**
Ca_LG_3:41925198	AATGAATGAAAAACATGTTAGTGGTCACGAAACAGATAAGGCAAGCCACGAAAATTTCTTCCCTGACACAGTTCGCTTCCCACCCCACCCATACCATACT	T/C	ATCATTCCGTGCAAGAAATTCAAAAGCATTACATGAATTTGTGATAATAAAATTAAAATATATTATCGAATAAGTTGAGTTGAGTGATTCAGCATGGCCA
Ca_LG_4:94968	TTCTATTAGCAGCAGCAGCAAGCACATCAATGTGCATATTATCAGCAAAAAGGATTGACGATGGATGTGATGAGTGTTGACTATCAGCATACCTGACCCC	C/T	AACTAGCAATTGTGATTTCTCATCCCTTGTAGCAATTGACAATTCATTTTTTTAGTACAAAGAAAGTTGCAAAAGTCTTAACCTAATGGAATGATTTATC
Ca_LG_4:197030	AACAGAGTAACCGATGGTTGAGGTAAACCAGAAGCTAGTAATCTCGCTCAAACAACTGAACAGACTCCAAGTCCTCTCCTTTCACTCAATTCACTGACAG	G/C	AATGTTGTGGTGGTGGTGATGATGATGATGATCATAATAATGATGATGATACATCTAAATGCAGCGGCATCATAAGAAAAAATGTAAGGGAAAATCACCC
Ca_LG_4:197244	CAAAAATTCTGGAAGGAAGAATATCGAAAAGAAGATCATAAGAAAAAAAACCAACAAAAACATTAAATGTTGAATTATAGAGGTAAATTTTCCAGTAAGT	T/C	CATTCAAGCTGATAATGATGACAAATCATTTCAAAGCTACTGAGATGCTTGAGTTCTTCAGTGCTTTAATTTTAAACTTTCTCTATTGATGAAAGCTTAC
Ca_LG_4:197270	AAAAGAAGATCATAAGAAAAAAAACCAACAAAAACATTAAATGTTGAATTATAGAGGTAAATTTTCCAGTAAGTCATTCAAGCTGATAATGATGACAAAT	T/C	CATTTCAAAGCTACTGAGATGCTTGAGTTCTTCAGTGCTTTAATTTTAAACTTTCTCTATTGATGAAAGCTTACATGCCAAAATTTTGCAAACATTCAAA
Ca_LG_4:284435	TCCGAAGCGACAAAAAGCTATTGCAGACAAATGCACCTCATATTTTTGATGTTGTAGTTGCAGCTGATGGTACTGGAGATTTTACTACAGTGATGGAGGT	T/C	GGTGCATGCAGCCCCAAGCTCTAGTGAGAAGCGTTATGTCATATTTATAAAGAGTAAAGTTTATATGGAGAATTTTGAGGTTGACAATGATTGGAGAGGG
Ca_LG_4:330167	GCGAATCGTCGTTGCTGCCCATATTCCTTTTTACTTGGATGTCAAGACACGCCCCGTATTTAGCAAGGCATGACTGACTTATTTTAGGAGCAGCAGCGAC	C/T	CGATTGTCCCCGATCCAGTTTTCTAGCTGCAACTTTGGGAGCTTTATTACCCTCCAGCTTTTGTAGTTTGCCAGCTCTATTACCCTCCAATTTTTGAGGT
Ca_LG_4:488486	TTCCTCAGTTGCTTGTCGACCAATATGCGCTATGTCTTCAAAAGATAGCTTGAGGCCGAGGTGTGCCTGAATTGCAGCAACATCCTGAACAACTGATTTT	T/C	AGGGATGTTTGGATAGATGTCAAAGTAGCTTCCATCCTTGCATTTGACTCTAGTATTTTGGCGTTGTGTTCCATTTGAGCTGCCATAAATTCCATCAGTT
Ca_LG_4:488531	TAGCTTGAGGCCGAGGTGTGCCTGAATTGCAGCAACATCCTGAACAACTGATTTTAGGGATGTTTGGATAGATGTCAAAGTAGCTTCCATCCTTGCATTT	T/A	GACTCTAGTATTTTGGCGTTGTGTTCCATTTGAGCTGCCATAAATTCCATCAGTTTTGCAACAAATGCAGGAGTCTCAGCTGCTGATTCAGAGGCTTGTG
Ca_LG_4:488592	GTTTGGATAGATGTCAAAGTAGCTTCCATCCTTGCATTTGACTCTAGTATTTTGGCGTTGTGTTCCATTTGAGCTGCCATAAATTCCATCAGTTTTGCAA	A/G	CAAATGCAGGAGTCTCAGCTGCTGATTCAGAGGCTTGTGGAGGAGTTGGTTCAACATTTGGCATTGGTCAAATCCAAACATTATCTACTTTCTTCAGTTT
Ca_LG_4:488605	TCAAAGTAGCTTCCATCCTTGCATTTGACTCTAGTATTTTGGCGTTGTGTTCCATTTGAGCTGCCATAAATTCCATCAGTTTTGCAACAAATGCAGGAGT	T/C	CTCAGCTGCTGATTCAGAGGCTTGTGGAGGAGTTGGTTCAACATTTGGCATTGGTCAAATCCAAACATTATCTACTTTCTTCAGTTTCATCTGATTCACA
Ca_LG_4:1005491	AGAGGGGATATTGGTCAAGTACAACTTTATTCTCAGAATAGACAGCATCAAACATCTCTATATCCTGCACATGTCAGCTATACCTCAATAAATAATTGAA	A/G	CTTGTCAGCAGCATTGCTGAGTGGAAAATATATAGATAAAAACAACAATTAAACCGTGTGAACAGGGAGAGATTCACCCAATCTTGTGCAGAAACTGATT

**Positions**	**-100 bp flanking sequences**	**SNPs within *desi* cultivar**	**+100 bp flanking sequences**
Ca_LG_4:1005509	GTACAACTTTATTCTCAGAATAGACAGCATCAAACATCTCTAT ATCCTGCACATGTCAGCTATACCTCAATAAATAATTGAACTTG TCAGCAGCATTGCT	T/C	GAGTGGAAAATATATAGATAAAAACAACAATTAAACCGTGTG AACAGGGAGAGATTCACCCAATCTTGTGCAGAAACTGATTCA ATAAACTAATATGATA
Ca_LG_4:1011657	TTTGATGATTATTACATTGCAGGAAGAAAAGATATCGGTCAGT TACGACTTTTGTTTCAATGCGCAGCATCAAACATTTCGACATC CTTAACCTCTTTCA	A/T	CGTCTCCTATGCCTCAATAAACACAAAAAATGAAGTTTTCAG CAGCCATGGGAAAATGTATTATGAAATGCTTACCATTTCCTG AGGTGTTTTGGGGACT
Ca_LG_4:1011793	TTTCAGCAGCCATGGGAAAATGTATTATGAAATGCTTACCATT TCCTGAGGTGTTTTGGGGACTTTTTCCTCTATTTCTCCCGCAT TGTACTCTGGACTC	C/T	TCCAAATCATCGGGCTGCAATGAAAACAAAGGCACCAAGAT AGTAAAAACACTGCTAAAAAACAAGTATATGCAAAACGTTCA AGATACTTATTTTGTAA
Ca_LG_4:1044969	AGGATTAAATGCGTAACTGATTTTCTGGCCGCCCAGGCCGTC ACAAAGTCACATATCTGTTACATTTTGTGGCGGCAAAGGCCG CCACAAACAACAATAG	G/C	TACCGTTGGCAGTTCTGGCCGCCCAGGCCGCCACAAATGCC TAATATCCGTTGGTCATTGTGGCTGCTTTGGCCGCCACAAAG GATCAAGCATATCCTAT
Ca_LG_4:1044998	CGCCCAGGCCGTCACAAAGTCACATATCTGTTACATTTTGTG GCGGCAAAGGCCGCCACAAACAACAATAGTACCGTTGGCAGT TCTGGCCGCCCAGGCC	C/T	GCCACAAATGCCTAATATCCGTTGGTCATTGTGGCTGCTTTG GCCGCCACAAAGGATCAAGCATATCCTATATATATTACTTCA CTCCTCTTTCATTTTT
Ca_LG_4:1103746	CATCCTTCACGCAAGTCAAATAGCCTTTTAACCAAAGCTCTGA TAGGAAACAAACACAGAAACACAACAACAAGTAATACGCAGC GGATATAAAATTCCC	C/T	CCAACTTGCAAGAACTCGTGAGGAGCAACAACAAATATATAT GGCAGCAAATTAATTAAACTAAAAGAGCGTAACAACACTGAT ATTTTTAATGTGGAAA
Ca_LG_4:1178441	GCTTTTGCAGTAAATGGCAATGTGAATCCAGCTGCTGAAGCA AATGTAAGTATTTCCATTTTGTATTCAAATTATTTGTATAACCT CCATAAAAATATCA	A/G	TATCATTTGCTGCTCGATCTATCTGACTCTACAGTTGGATTC GATTCCAGATATTTGGTGATCCAGATGCTGCAGATGTTGTAT TCACAAGTGGTGCAGA
Ca_LG_4:1354494	ATGAGTTTTTGATAGCTTTGATGAAGTTCATGGATTGTATCAG GGTGGAATTAAGCTGCCAACAAACATACTTAGCAAGATTAGTT CATGGATATGTGTC	C/T	ACTGCTTAATTTTGTGTGTTTTTGCAAGCTTCATGTTCTGTTC TACTCTTTTGCAGCCAAGAAAGATCATACCTTGTGCTCATTG TAGAATGGGAACCAT
Ca_LG_4:1354532	ATCAGGGTGGAATTAAGCTGCCAACAAACATACTTAGCAAGA TTAGTTCATGGATATGTGTCACTGCTTAATTTTGTGTGTTTTTG CAAGCTTCATGTTC	C/A	TGTTCTACTCTTTTGCAGCCAAGAAAGATCATACCTTGTGCT CATTGTAGAATGGGAACCATTGGAAAATTGTTACATCAAAAG CAACATGGGAATGCAA
Ca_LG_4:1354847	GTTAAATCTGCAGTGGATATTCAAACATCGGGTGATCTAANNN NNNNNNNNNNNNNNNNNNNNNNNNNNNNNNNNNNNNNNNN NNNNNNGCTGCTGCTG	G/A	CAACAGGTACTTGCTTTATCTTTCTACGCTTGCATGTTATAAG ATTAGTAATTGAATAGTATTAAACTATATACTACCAACACTTT AGATTGAAGATGTA
Ca_LG_4:1354851	AATCTGCAGTGGATATTCAAACATCGGGTGATCTAANNNNNNN NNNNNNNNNNNNNNNNNNNNNNNNNNNNNNNNNNNNNNNN NNNGCTGCTGCTGCAAC	C/T	AGGTACTTGCTTTATCTTTCTACGCTTGCATGTTATAAGATTA GTAATTGAATAGTATTAAACTATATACTACCAACACTTTAGAT TGAAGATGTATTCG
Ca_LG_4:1354900	NNNNNNNNNNNNNNNNNNNNNNNNNNNNNNNNNNNNNNGCTG CTGCTGCAACAGGTACTTGCTTTATCTTTCTACGCTTGCATGT TATAAGATTAGTAATT	T/C	GAATAGTATTAAACTATATACTACCAACACTTTAGATTGAAGA TGTATTCGATGTTTGACATGTTTTAGTGTCCAACACATATTGT TAACATGTCTGTTT

Positions	-100 bp flanking sequences	SNPs within *desi* cultivar	+100 bp flanking sequences
Ca_LG_4:2874793	GTATAATAAATACTAATCAATATGCTGCATCAATGTGGTTGGCCACAACAGTTAGTGACATTCACAAAAGTTAGTTGCATTTAAAACTATGATTAGAACT	T/A	ATGGCTGCAAATTGAAACCGTGTTTTTCACATGATTTATATTTTCCGGCTTTATAATTCTCAATACATGCATTGGCAAACGAGAAATACATGTATCCACT
Ca_LG_4:2957801	NNNNNNNNNNNNNNNNNNNNNNNNNNNNNNNNNNNNNNNNNNNNNNNNNNNNNNNNNNNNNNNNNNNNNNNNNNNNNNNCTTGTATTGTTACTGTGAATGTA	A/G	AAATAGCAAAAGGAAGGAAGTTGTAACTAGATTGAGAGCTATGAAGATTTAATTGAAGGATAGTTTGGAGTTCATATAGTAAAGAGATAAGGCTCAAGAA
Ca_LG_4:2957825	NNNNNNNNNNNNNNNNNNNNNNNNNNNNNNNNNNNNNNNNNNNNNNNNNNNNNCTTGTATTGTTACTGTGAATGTAAAATAGCAAAAGGAAGGAAGTTGT	T/A	AACTAGATTGAGAGCTATGAAGATTTAATTGAAGGATAGTTTGGAGTTCATATAGTAAAGAGATAAGGCTCAAGAACAAGACATTGTTGAGTTGTGAATG
Ca_LG_4:2957831	NNNNNNNNNNNNNNNNNNNNNNNNNNNNNNNNNNNNNNNNNNNNNNCTTGTATTGTTACTGTGAATGTAAAATAGCAAAAGGAAGGAAGTTGTAACTAG	G/A	ATTGAGAGCTATGAAGATTTAATTGAAGGATAGTTTGGAGTTCATATAGTAAAGAGATAAGGCTCAAGAACAAGACATTGTTGAGTTGTGAATGAATAAT
Ca_LG_4:3258751	CATGTTCTTGATAAGGAGAAATTTCGATAGAATCGAGATGGGTTGTGGAAATTGTCTGCTTTCTCATCGCTCAACAATTTTTTCTTTTCTACACTAGTTT	T/C	GAATTTGGAGATTCCCCAAATCTGCTCCCTATCTTCTTTTAGTGTTGCTTTGGATAATTATTGAAACTTGAGAGGGAAGAATGGACATGCACAGGGTCGT
Ca_LG_4:3258759	TGATAAGGAGAAATTTCGATAGAATCGAGATGGGTTGTGGAAATTGTCTGCTTTCTCATCGCTCAACAATTTTTTCTTTTCTACACTAGTTTGAATTTGG	G/A	AGATTCCCCAAATCTGCTCCCTATCTTCTTTTAGTGTTGCTTTGGATAATTATTGAAACTTGAGAGGGAAGAATGGACATGCACAGGGTCGTATTGGGGG
Ca_LG_4:3258775	CGATAGAATCGAGATGGGTTGTGGAAATTGTCTGCTTTCTCATCGCTCAACAATTTTTTCTTTTCTACACTAGTTTGAATTTGGAGATTCCCCAAATCTG	G/A	CTCCCTATCTTCTTTTAGTGTTGCTTTGGATAATTATTGAAACTTGAGAGGGAAGAATGGACATGCACAGGGTCGTATTGGGGGAAGAAGACTTCAGATG
Ca_LG_4:3258779	AGAATCGAGATGGGTTGTGGAAATTGTCTGCTTTCTCATCGCTCAACAATTTTTTCTTTTCTACACTAGTTTGAATTTGGAGATTCCCCAAATCTGCTCC	C/T	CTATCTTCTTTTAGTGTTGCTTTGGATAATTATTGAAACTTGAGAGGGAAGAATGGACATGCACAGGGTCGTATTGGGGGAAGAAGACTTCAGATGGTTG
Ca_LG_4:3258785	GAGATGGGTTGTGGAAATTGTCTGCTTTCTCATCGCTCAACAATTTTTTCTTTTCTACACTAGTTTGAATTTGGAGATTCCCCAAATCTGCTCCCTATCT	T/C	TCTTTTAGTGTTGCTTTGGATAATTATTGAAACTTGAGAGGGAAGAATGGACATGCACAGGGTCGTATTGGGGGAAGAAGACTTCAGATGGTTGTTGCCT
Ca_LG_4:3914079	AGTAGATGTCTCAAGTAAATTCCAAGGTTTAGTGATACTTACCCACAAGACCAAACAAAACACAATACATAGCAGCAAAAATAGGGAATGGTATTGATGG	G/C	AAATAAAGCTCCAAATTTTCCTGAAATAAAGAAACAAATTATGTATTTTGAATGAAACTATGGAATTGTAATAAATTTACACATTATCATAACTTCATAG
Ca_LG_4:3914098	TTCCAAGGTTTAGTGATACTTACCCACAAGACCAAACAAAACACAATACATAGCAGCAAAAATAGGGAATGGTATTGATGGAAATAAAGCTCCAAATTTT	T/A	CCTGAAATAAAGAAACAAATTATGTATTTTGAATGAAACTATGGAATTGTAATAAATTTACACATTATCATAACTTCATAGTTTGCCTTGCCACTCACCT
Ca_LG_4:3914659	TGGTTTAATTTTTCCAATAATAAAAGAAATGAATCACTAGTCAACAATAATATTAGGACGTAAGAAGCATCTTTATGCAGTTGAAAATACCTCAACTAAT	T/C	GAGACTAAAACAGCAGCCATCATTCCAAAAGAATGACCAGCATCAAATGTAGGAACACCCCACTCAAGAGGGTATGGAATCTTTATCCTGCAGAAACATG

Positions	-100 bp flanking sequences	SNPs within *desi* cultivar	+100 bp flanking sequences
Ca_LG_4:4313715	CAAGGTAACTAGTACAACAATCAGCCAACACAATGAGAAAAC AAACATGAAATTGAGAAAAAAGTTCAGCAAAAGAAACCAGAAA AACTGCTGCTGTCCG	G/A	AGTTCAGTTTCTCCCACCTCAGACCTCTGATCGACGATCCAA ACGGTCAGAATGAAGTACCACGAGTCACAAACCTGCTGTCC AAATTTCAGCCCGATCC
Ca_LG_4:4313744	ACAATGAGAAAACAAACATGAAATTGAGAAAAAAGTTCAGCAA AAGAAACCAGAAAAACTGCTGCTGTCCGAGTTCAGTTTCTCC CACCTCAGACCTCTG	G/A	ATCGACGATCCAAACGGTCAGAATGAAGTACCACGAGTCAC AAACCTGCTGTCCAAATTTCAGCCCGATCCGACGGTGAACG AAGGAGAAATCGCGAATC
Ca_LG_4:4494641	TGCTATTTTGAGGATACATAATGTTGTTGGAAATGCTGTAACA AAAGACAACTACATAGTATTAGGAACAGGCTCTTCTCAACTCT ACCATGCTGCTTTA	A/G	TATGCACTTTCTCCTTCACAACCCCCTAATCATCCCATTAATG TTGTTGCTGCTGCTCCTTATTACTCGGTAATATTTCTCTCTCT TCAATTTTTTTTTT
Ca_LG_4:5673656	TACTAGTGTGTATTTTGGTAATTTTAATTAAGCTTTATTTTATAA ATTTGATTGGAATTGAATTATGTCACACCCATCATGCCATCTG TACTTTTTGTTG	G/A	TATAAATATTATTATTACATCTCTGTAACAGTGTTCATTGCCA TTTTAATTTGGAAATTAATTTTCAAATTTTATAAATTAATAATT ATATTTTTGAAAT
Ca_LG_4:5673679	TTAATTAAGCTTTATTTTATAAATTTGATTGGAATTGAATTATGT CACACCCATCATGCCATCTGTACTTTTTGTTGTATAAATATTAT TATTACATCTC	C/T	TGTAACAGTGTTCATTGCCATTTTAATTTGGAAATTAATTTTC AAATTTTATAAATTAATAATTATATTTTTGAAATTATAAACTAG TAATTAAAATAAT
Ca_LG_4:5673686	AGCTTTATTTTATAAATTTGATTGGAATTGAATTATGTCACACC CATCATGCCATCTGTACTTTTTGTTGTATAAATATTATTATTAC ATCTCTGTAACA	A/T	GTGTTCATTGCCATTTTAATTTGGAAATTAATTTTCAAATTTTA TAAATTAATAATTATATTTTTGAAATTATAAACTAGTAATTAAA ATAATTAATTTA
Ca_LG_4:5673689	TTTATTTTATAAATTTGATTGGAATTGAATTATGTCACACCCAT CATGCCATCTGTACTTTTTGTTGTATAAATATTATTATTACATC TCTGTAACAGTG	G/C	TTCATTGCCATTTTAATTTGGAAATTAATTTTCAAATTTTATAA ATTAATAATTATATTTTTGAAATTATAAACTAGTAATTAAAATA ATTAATTTAATT
Ca_LG_4:7797075	ATCAAGGTCATGACGATGCTTTACTTGGTCTCTCATCAGGTGA TGAAGTTCTCAGAGAAGTTGCAGATAAATCTACTGCAGCAGG GGTTTGGCTAAAGCG	G/T	GAAAGGTCTCTACATGACTAAATCTTTAACCAACGGACTAAA CTTAAAGAAGAGGCTGCATTAGTTAAAGATGGAAGAAGATTC ATCAATCAAAGAACAT
Ca_LG_4:8203223	GCTAGAAGTTCCAATACACAGCGTGGGGTTATCTCTTCAAGT GTCTCATCAATCTGTGCTTGTAAATCTGGTGCTAGGCTGCTTG CCCCTTCTTCCTACG	G/A	TGATCAAGTGAGTCATCAGAAAGCATGTTGGCATATTGCATC AGAACTGCAGCTTAAAAGTCTTCAAGCTATATGCTATACACA TTGTGTTACTACACAT
Ca_LG_4:8309403	GCATGTGTTATGGATTTCTTCCTTTAAATTATATAATATAATAA TAATTTTTAGTCACTTTTTTCACTACTATTTAGCTGCACCTTAT GCTTTATTTTTT	T/A	AAAAAATTAATAGTTCCATTGTTATAATTTAACAAACTGTTGC TTTCCTGAGTGAGTTATATCGTCTAAATCATCATGCCATTTAA AGTTGAGTTACAAG
Ca_LG_4:8319249	TTTTTGACTGACCTTTAGTTAAGCCCCACCCCAATTGTCTGAT TGTCAGGAGCATTTCCATCTGGCAGCGAAGTCAGTTCAGTAC ACAAGTTTCCATATC	C/A	ATTTATTGGAGCTTTTTTGCACAGCAGCATTTATTAAGCGATT CATTTAAGTTTAGGCAGAAGTTGTGTTTTATTTATTACAAATA GATTTCTCACTGAT
Ca_LG_4:8333069	GGAGCATGCTGTTTACAATAGAAGAATCGGGTTGGACCATGT CGACCATTTGAGGATGTGATATTGATGATTCTAACTGAGGTTG CTGCATAGGCCATGC	C/G	ACTACTAGATTGATTGAGTAGTGGATTTTGGTTTTGATTATGA TATGGAAATTGGTTTGTTAGGCTTTGAGAGTTAACAAGATTC GGTGCAGCCACTTTA

Positions	-100 bp flanking sequences	SNPs within *desi* cultivar	+100 bp flanking sequences
Ca_LG_4:8444095	CTCACTCTTCTTCATGCATGCTCTCACATAACAAACAAATTCTATCTTGAAATTGCAGCAACGACCAGGTGTGAGGGTTCGTCAACCTGCACCTGCCACT	T/C	GGCCAACCAGCTGCTGCTACAGGAACACAAGTTGAGGAACCCCCATCCCCTGCTTCTAGTACATCTTCAAGTCAGTTCCAGAGGGAAGTAGCAGAGGAAT
Ca_LG_4:8444120	ACATAACAAACAAATTCTATCTTGAAATTGCAGCAACGACCAGGTGTGAGGGTTCGTCAACCTGCACCTGCCACTGGCCAACCAGCTGCTGCTACAGGAA	A/G	CACAAGTTGAGGAACCCCCATCCCCTGCTTCTAGTACATCTTCAAGTCAGTTCCAGAGGGAAGTAGCAGAGGAATTGAGCTCTCCAAAAGTTCCTAAAGC
Ca_LG_4:8444194	TGGCCAACCAGCTGCTGCTACAGGAACACAAGTTGAGGAACCCCCATCCCCTGCTTCTAGTACATCTTCAAGTCAGTTCCAGAGGGAAGTAGCAGAGGAA	A/G	TTGAGCTCTCCAAAAGTTCCTAAAGCAGCCCCTCCTGTTGCAGGTTTAAATAACCAGATGTCTTCTGTAGCTGGTAACCAGAAGCCACCTGTAGCTGGTC
Ca_LG_4:8444488	CCCAAAGAAGATTCAAGCAGCAGCACCTTCATCCTCTTCAGCAAATGAAAATGGAAAATCTCCTACAAAGGAGACCATAATGGAAGAAAGCATCCGGGTT	T/C	ACACGAGGCAGACTAAGAAAAACCAGATCATCGGGAACCCAGTAGCACAATATTGTCAATTTTTAGCCTGAAATAGGGATAGTTGGACCATGCTGCTGGC
Ca_LG_4:8448897	AAAAAACGATATTTCTCTTTCAGTGCAGCAGCAGCAATTTACTCCAGCATTAAGACTGATAAATTGTTTTCATGATTTTAACTGGACAAAATAATTGGTT	T/G	AACTGCAATAAAAACACATCAGCTGACCTCTTGCAGATTTCATCGCTTAAATTAAATATCCTCACAATATCCATGGAATATGGTGCCTGTCCAAGATATG
Ca_LG_4:8449057	CTCACAATATCCATGGAATATGGTGCCTGTCCAAGATATGTGTTGCATACATAGCCAGTGCCTGTACACATTACATTAACTTTAGATTGTCAAACTTAAC	C/T	GCACAAATTAGCTGCATAACTATGAAAACTCACTATCATTCACTCGATCAGATGATTGTTAAGAATGGAAAGTGAATAATAAAAATGACAATTTTTTGTA
Ca_LG_4:8449061	CAATATCCATGGAATATGGTGCCTGTCCAAGATATGTGTTGCATACATAGCCAGTGCCTGTACACATTACATTAACTTTAGATTGTCAAACTTAACGCAC	C/G	AAATTAGCTGCATAACTATGAAAACTCACTATCATTCACTCGATCAGATGATTGTTAAGAATGGAAAGTGAATAATAAAAATGACAATTTTTTGTAGTTT
Ca_LG_4:8757470	CGATAGAGAGGAGAATGTGATTAACAGATCTGAAGAAAGTATATACGAGGCTGAAGTGTTTGTTTTACGGTTTTTCACACATATGCACTTAATTTCCAAT	T/A	TTTTTTGCTTTTAGCAGATATTTCGTCCATGGCTGCTGCCAGTAGCACCTAGGCTCCATGCAAATGGTGGTTGCCATAGCTCACCACTCACCGCCATAAC
Ca_LG_4:8820503	TGTTACCTGAGGATTTCACTATCCGCAGGTAAATTACCTGCGGCTAAATCCGCAGGAATATTGAATAAAATCCGCAGCAAAGTTTAAGAAAACTTTAGAA	A/C	ATTTTTTTACCACTTTTCATGCAGAAATATTTGCAGCACAGTTCCTACAAATATATTTCGTATAAAAATCTGCAGGTATATCCATTGCACCAACCATATG
Ca_LG_4:8820516	TTTCACTATCCGCAGGTAAATTACCTGCGGCTAAATCCGCAGGAATATTGAATAAAATCCGCAGCAAAGTTTAAGAAAACTTTAGAAATTTTTTTACCAC	C/T	TTTTCATGCAGAAATATTTGCAGCACAGTTCCTACAAATATATTTCGTATAAAAATCTGCAGGTATATCCATTGCACCAACCATATGTTTCGCATTAAAA
Ca_LG_4:8820527	GCAGGTAAATTACCTGCGGCTAAATCCGCAGGAATATTGAATAAAATCCGCAGCAAAGTTTAAGAAAACTTTAGAAATTTTTTTACCACTTTTCATGCAG	G/A	AAATATTTGCAGCACAGTTCCTACAAATATATTTCGTATAAAAATCTGCAGGTATATCCATTGCACCAACCATATGTTTCGCATTAAAATCCGCAGATAA
Ca_LG_4:8912425	AATTGCAGCCATTATAAGTTACAAATCTCTTCCCTTGAAGAAGGAAGTGAAGAAATTGATACACCTTATTCTCCACGCCATTGCATTAATACTTGGAATC	C/A	GTTGGAATTTCCGCCGCGTTCAAGAATCACAATGAAAGTGGGATTGCCAATTTGTACAGCTTGCATTCATGGCTTGGAATTGGAGTTATTAGCCTTTATG

Positions	-100 bp flanking sequences	SNPs within *desi* cultivar	+100 bp flanking sequences
Ca_LG_4:8949417	AGGAAGGAAAGATACATTGGGGTACAGAAGCTAGTATGTTGAATCAATCTTGGCAAGATTTATTTTTATTGCTGTAATTCGGCACCAGCAGCTCCAACCT	T/C	TCCAAGAAGCAAAAACCGTAACAAATTTTGCTGAGCAGGAAAGATAGGAGTAATATTAAGGGATTATGCTTCTGCCAGGAAAACTACATATGAAGGAAAC
Ca_LG_4:9774540	TTAATGATGGCTGCATCCCATTATTTCTTAGAGAATGCTTTTGATTTTGTGTTTCTCTTTTTTCTGGAGGCCTATTTAAAAAACATTTCAGTTTGCCACA	A/C	AAACTTAATCTTTCCCTCTTTACTGAAATGAATTTGTTGAACTGATTAGAAGAAACAGGTTACACCCAATATTTTCCAGTGCTGTCAAGTTACTGCTAAG
Ca_LG_4:9774574	ATGCTTTTGATTTTGTGTTTCTCTTTTTTCTGGAGGCCTATTTAAAAAACATTTCAGTTTGCCACAAAACTTAATCTTTCCCTCTTTACTGAAATGAATT	T/G	TGTTGAACTGATTAGAAGAAACAGGTTACACCCAATATTTTCCAGTGCTGTCAAGTTACTGCTAAGGCGGTGCCATGAAGGATTTTATTTCAGCTGCCAT
Ca_LG_4:9774620	AAACATTTCAGTTTGCCACAAAACTTAATCTTTCCCTCTTTACTGAAATGAATTTGTTGAACTGATTAGAAGAAACAGGTTACACCCAATATTTTCCAGT	T/C	GCTGTCAAGTTACTGCTAAGGCGGTGCCATGAAGGATTTTATTTCAGCTGCCATAGGCCGCAGCACCTATTATATCTGCCATAGGTCGTAACACCATGTT
Ca_LG_4:9774632	TTGCCACAAAACTTAATCTTTCCCTCTTTACTGAAATGAATTTGTTGAACTGATTAGAAGAAACAGGTTACACCCAATATTTTCCAGTGCTGTCAAGTTA	A/T	CTGCTAAGGCGGTGCCATGAAGGATTTTATTTCAGCTGCCATAGGCCGCAGCACCTATTATATCTGCCATAGGTCGTAACACCATGTTTATATGATGGAT
Ca_LG_4:10004378	ATGTTGAATACAGTCGAATGTTGTCGATATCATTAGGCATTCAATTCAGAATGCTTCTCATTTTGAAGTAGCTGCTCAACAGTTGAAACAACATGCCTTC	C/A	ATTGTAAGTAATATGTTCAACAATTGTCATTCTCAAATGTTTGGGAAGGAATAGTCTGTGTTTTATGCTGCACTTTCATATTTTTACAACTAGACCACAA
Ca_LG_4:10123762	GTTCAAAGGTTCGGTTTATCCGTAGGAACCATAAGTATAACGAAGCTGCGAGGACTACTGCTGCGCCTAGGGTTGGGTTTGGTTTGGCTTCGGAGTTGCG	G/A	GCGTGCTGGTGTTTTTGTGAAGGTTGTGGAGGATGGTGATAAGGTAAATGCTGCCGATTCGTCGTTGAAGAGGGAGATGGTGAATGGAGGGATTGATTGG
Ca_LG_4:10410130	AATGAGATTGAATTCCTCTTCATGATTGTTTGGATCTTTATTGGTGAAATTGTAAGCAGGTAGATAAGTAGGTGCAGCAGATGTACTAATACATATGTCA	A/T	GAGAGTTTAGCATCCAAACAAGGTGATTTCTCAAGCTGCATAAATTAATTCATTTGTTATACACAATTAATCATAATCGTTGACTTCAAATATCTTACAA
Ca_LG_4:10431489	ATTTGTTAATATGGGTGAGAAGATGAATCATTATTATTAAAAAATAAAAAATAAAAAAGTAATAAGAGCAGCATGTTGGCTTCTGTATTAAGTAAAAAAT	T/A	ATCATAAAGATGGAAAAAGTAATCCAACTGTTCACATAGTAGACCGTCCCCCACTCACATCTTACAAGCCTCTCAAAAACATTCCCTTTTTCTTCTTCTT
Ca_LG_4:10553679	AGAATAGCCAATAAGTAAATATAATTTACAGAATAATATTAAAACCCAGAAGACTTTGCAAGAAAAAAATTATACAGCTGCTCAGACCTGAAGTACCAAC	C/T	GCCCAGTTGTACAGTGCATCATAATCTTCAGGGTTCCTCTCAATAGCAGCAGCATACCTAGAAAATCCAAGTAAGAAAATTGTAAGTTTGAATGAAGATT
Ca_LG_4:10553868	GAATGAAGATTTCCGTCCCTGAGAAGCACAGGCAAAACAGCAATGCCCGCAATGATAGGGTTCCTAATATTTGGGAAAGATTACAAAACAGATTGCAGTG	G/A	TTGCAAGAATTGATTGCAGTACCTCCTGGCAGCAAATGTAAGAATCCGTTGTCGGGACCGTCCCTCCTCATCAACACCAACAACGCTATTTATCAATTCC
Ca_LG_4:10949166	GAAAGCGAAGCCGAAGAGATAGTAGGAGAGTCCACCGGCGGCAGCATCAAGGACGTTGGTGAGCATGATGTTCATCGTGTTTTTGGCTCGAACGGAGCCG	G/A	GCGCAGAGCATGGCGAAGCCGAGCTGCATGGCGAAGACTAAATAAGCTGAGAAAAGAAGGTACGTGTTGTTCACTGCATAGGTGGTGTCGCTGAGTTGAT

**Positions**	**-100 bp flanking sequences**	**SNPs within *desi* cultivar**	**+100 bp flanking sequences**
Ca_LG_4:11432192	AAGAGAAAAAACTTATGTATGATTTGAGAATTTTTTTGACACTTTTCTATTTCTAGTGTAAGTTTATGTCCCCAACTTTTGTTTTCTTTTTATTTTAATA	A/G	AACTTTTAGCTTAGAAAAACACGAGTTCAACTTTATAACCTCATGGAATAAAGCTCCTATCTCCTCGGCAACAGTTGTAAACTGGTTCACGACTTTAAAT
Ca_LG_4:11852996	TAAAAAGAAAAAGAAAATGGAAGGAAATAGTAATGGAAATGTAGAACAGGTTCGGTATAGAGGAATTCGTAGAAGACCATGGGGTAAATTTGCAGCAGAG	G/A	ATTCGTGACCCAACAAGAAAAGGGACAAGAATATGGCTTGGAACATTTGATACTGCCGAGGAAGCTGCACGTGCTTATGATGTTGCTGCTTTTCATTTTC
Ca_LG_4:11853005	AAAGAAAATGGAAGGAAATAGTAATGGAAATGTAGAACAGGTTCGGTATAGAGGAATTCGTAGAAGACCATGGGGTAAATTTGCAGCAGAGATTCGTGAC	C/T	CCAACAAGAAAAGGGACAAGAATATGGCTTGGAACATTTGATACTGCCGAGGAAGCTGCACGTGCTTATGATGTTGCTGCTTTTCATTTTCGTGGTCATA
Ca_LG_4:11853012	ATGGAAGGAAATAGTAATGGAAATGTAGAACAGGTTCGGTATAGAGGAATTCGTAGAAGACCATGGGGTAAATTTGCAGCAGAGATTCGTGACCCAACAA	A/C	GAAAAGGGACAAGAATATGGCTTGGAACATTTGATACTGCCGAGGAAGCTGCACGTGCTTATGATGTTGCTGCTTTTCATTTTCGTGGTCATAGAGCTAT
Ca_LG_4:11853021	AATAGTAATGGAAATGTAGAACAGGTTCGGTATAGAGGAATTCGTAGAAGACCATGGGGTAAATTTGCAGCAGAGATTCGTGACCCAACAAGAAAAGGGA	A/T	CAAGAATATGGCTTGGAACATTTGATACTGCCGAGGAAGCTGCACGTGCTTATGATGTTGCTGCTTTTCATTTTCGTGGTCATAGAGCTATTCTTAATTT
Ca_LG_4:11853024	AGTAATGGAAATGTAGAACAGGTTCGGTATAGAGGAATTCGTAGAAGACCATGGGGTAAATTTGCAGCAGAGATTCGTGACCCAACAAGAAAAGGGACAA	A/C	GAATATGGCTTGGAACATTTGATACTGCCGAGGAAGCTGCACGTGCTTATGATGTTGCTGCTTTTCATTTTCGTGGTCATAGAGCTATTCTTAATTTCCC
Ca_LG_4:12009712	ATTCTTTCCTTGAGGCTGACAAGGTCAAGGAGTTCCTAAAGGACCCAAGCAAGTTTTCTGTAGCTGCAGTTGCTGCCCCAACTGCTGATTCTGGTACTGC	C/T	CTCTGCTGCTGCTACCAAGGAGGAGGAGAAAAAGGATGAATTGATGGTATAGTCTATGTTATTGATCCTGGATTTGCTATGCAGAATGAGTTGTTTGAGG
Ca_LG_4:12415083	TGAGAAATAGCGGAAGCCGCATAACAATAATTATTTATAAAACAAAGCATGTTGCATACAAATAAAAGCTGTCTAAATAGAAATTTAAAACTCAAAATCG	G/A	AAATTCAAAATCTCAATAATTGAATTCAAAACAACTAATAGTGCATATTGCCTACTGCCTACCTGCAGCCTAAATAGAAATCCATAAAGTGATATAAAAC
Ca_LG_4:12415117	TTATAAAACAAAGCATGTTGCATACAAATAAAAGCTGTCTAAATAGAAATTTAAAACTCAAAATCGAAATTCAAAATCTCAATAATTGAATTCAAAACAA	A/C	CTAATAGTGCATATTGCCTACTGCCTACCTGCAGCCTAAATAGAAATCCATAAAGTGATATAAAACTCAAAATCTCAATTCAAAATAACTAATAATTTTT
Ca_LG_4:12451717	CTGCAGAAGCAACAGCCATGTCAGTCTTAGCCATCTTCTCATCATCCTTGTTGTTAGATGCAGCAGCTGTTGCTGCAGTGATGGCTGCAACTGCAGAGGA	A/C	AACAGCAGCTACACAAATAGCAGCATGCAGTTGAGCATTATGGTTTCTGTTTTCTTCTTTCTTTTTTTCTCTTCTGTCTTTTAGCCATCTTCCTACTGTT
Ca_LG_4:12477345	TAAGTATGCTTGAAGAGACTAAGAAAATTGCTGAAAGAAGAGAGCAGCAAGTAATTCTTTCTCTCATGTCATTTGTTAATCATGAACCAAAACTTTCTTG	G/A	TAGTCTATTTATTGTACTTGTAGCATTTTTGTTGTTGCTGTTAACCTTGAAATCTTTTCATTCCTATGAGTGCAGCTTGTTTTGGTCAATCCCGGAAGTG
Ca_LG_4:12952417	TAGTGAGTATGATGTTGTTATTGGTGCAATTCAACAGAGGAGATGTAATTGGAATCAAGTGCTGCTTATGCAGCAATACTATTCGGTTTCCGAGGTTTCG	G/C	TATGCGCTGCAGCAGGTGGCGTGGAGGCGGCAACAAAGGGTGGTAAAGCCGGTTGTTAAGGAGTTTAGGAAGGTTAGGCAATGGCAGAGGTTTGAGGGTG

Positions	-100 bp flanking sequences	SNPs within *desi* cultivar	+100 bp flanking sequences
Ca_LG_4:12963452	GGCAACAAACAGACTTCGTTGAACAGATGTTAAGTTGCTGCAATGAAGGCAGCAAAAAAATATATATATCTTAAATTACTGCAATGAAGGCAGCAAAAAA	A/G	AATCTTAAATTGTTGCAACGAAGGCAGCAAAAAGGGCCACAATGAAGGTGGCTAAGGTTTCAGAAAAAAGAAAACCGCAATGAAGGCGGCAAATTTGTGC
Ca_LG_4:12998156	TCTGGTTAATGCAGCCTCCTTAATTTTTGCTTCATCTACTGTTCTTCCATCAGCTTTTATACAATGAATATGTTCTGGTGGTTCTAGTAGTGGTGGTGCT	T/C	GTGGTGAATTGGTTAGCCTGTGTGCCACATGCCTCTCCAAGGTTGTTGATATGTTTAAGACACCATCACCATGTTGTTGATGCCATATACTGCTGCTCAG
Ca_LG_4:13001285	TGGGCGGCCGCAGGACAGTGGCTGGGGTGATGAAGCAGCACCCTTGTAACACAGGTTTTGGTTGAAGGGTAGGATGCCGTAAATTTTAATTAAAATGAGG	G/A	GTTTTCGTAGGGCTGCGTTTCGAGCATACAAGCCAAAAAGTGTCATGTCCTTCATCATCATCAAACCTAAAGATCCGAAGTTGAGAATCCTCTTCAAGAG
Ca_LG_4:13575562	GGTCCAGTTTTGACATGGGCATAGAGAATAAAAATTGATGTTGGGGATGCAAGAGGGCTTGAATACTTGCATGAGAAAGCTGATCCCCATATTATTCACC	C/G	AGGACATCAAGTCAAGCAATGTGCTGATCTTTGATGATAATGTTGCTAAAATTGCAGATTTTGATTTGTCAAATCAGGCTCCAGACATGGCTGCACGTCT
Ca_LG_4:13575601	GTTGGGGATGCAAGAGGGCTTGAATACTTGCATGAGAAAGCTGATCCCCATATTATTCACCAGGACATCAAGTCAAGCAATGTGCTGATCTTTGATGATA	A/G	ATGTTGCTAAAATTGCAGATTTTGATTTGTCAAATCAGGCTCCAGACATGGCTGCACGTCTTCATTCCACCCATGTCCTTGGAACCTTTGGTTACCATGC
Ca_LG_4:14208847	GACCGATGTTCGCAATATCTTCAAATGATAGCTTTATGCCTAAATGCGCTTGAATTGCAGCAACATCCTGAACAACTGAATTCAGAGAAGCCTGGAGAGA	A/T	TGTCAAGGCAGCTTCCATTCTAGCCTGTGACTCTTGCATTTTGGCATTGTGAGCCATCTGAACTTCCATAAATTCCAGCAGTTTTGCGATGTACTCAGGA
Ca_LG_4:14209005	TGAACTTCCATAAATTCCAGCAGTTTTGCGATGTACTCAGGAGGGGCTGCCACTAGTTCAGCAGCTTGTTGAGGATTAGGTTGAGCTGGTTCAACATTCG	G/A	GCAGTGGTCTTATCCAAATACCATGCACCCTTTTCAATTGCATCTGATTAACAATTGATTCACCAAAAATAAGGGTGGTCTTGGAAGATTCTTCATTAAC
Ca_LG_4:14209007	AACTTCCATAAATTCCAGCAGTTTTGCGATGTACTCAGGAGGGGCTGCCACTAGTTCAGCAGCTTGTTGAGGATTAGGTTGAGCTGGTTCAACATTCGGC	C/T	AGTGGTCTTATCCAAATACCATGCACCCTTTTCAATTGCATCTGATTAACAATTGATTCACCAAAAATAAGGGTGGTCTTGGAAGATTCTTCATTAACAA
Ca_LG_4:14300967	GGTTTCTTGTAATTCCTTGATCTTGTTAAGCTCGGAAAGGCCACTATCGGAAGTTCCTCTATGAAGTTGGAGACTCATTGCCTTTGAATCGCGTTCTTCG	G/A	GCAGCTAATAGATCAGGCTCAGATGATCGGCCAGATTTTTTGGTAACGATCCATTCGTAGGAACTTCCTAATTGGAACAAACCAGATACCATGGCGTTGA
Ca_LG_4:14920983	TCCTTTGAAGATTTCTCAAATTTGTCAGGATTGCCCTTTCATGGAAAGTCAATAGATGGCAGTTCTGATGCTGATGCTGCAGATGAAGGATGGGAAGCAT	T/G	CTTTTGACAGGCACACTGCTGTGAAGGACTTGATGGTCGAAGGATTTGTTGAAACTATTTCGCTGCCAATCGGTCAAATGAAGGTCCATCATCAGATGCT
Ca_LG_4:14920994	TTTCTCAAATTTGTCAGGATTGCCCTTTCATGGAAAGTCAATAGATGGCAGTTCTGATGCTGATGCTGCAGATGAAGGATGGGAAGCATCTTTTGACAGG	G/A	CACACTGCTGTGAAGGACTTGATGGTCGAAGGATTTGTTGAAACTATTTCGCTGCCAATCGGTCAAATGAAGGTCCATCATCAGATGCTTATGTATACCT
Ca_LG_4:14921033	AATAGATGGCAGTTCTGATGCTGATGCTGCAGATGAAGGATGGGAAGCATCTTTTGACAGGCACACTGCTGTGAAGGACTTGATGGTCGAAGGATTTGTT	T/C	GAAACTATTTCGCTGCCAATCGGTCAAATGAAGGTCCATCATCAGATGCTTATGTATACCTTAACCCGGATGCTAGTGCCATGATCCGGAAACCATGCCG

Positions	-100 bp flanking sequences	SNPs within *desi* cultivar	+100 bp flanking sequences
Ca_LG_4:15160508	CACATCTTCCTTAGACACCACTGCAGCAGTACTCTTCAACAATTCCAAGGCCTGCTTTGAACTGATACGAGATGACTCAAACTTAGCTTTCAAATCATCG	G/A	CGCTCTTGTTGTGCTGCAACAAGGCTACTATGTGTAGCATCCTTTTCGTTACTCAAAAGATCGACGCGGCTTCTGAGCTCCTTGTTTCTCTCATTCATCT
Ca_LG_4:16440728	CTGATAAAACTTTATTTCCCTACAAACTAGTTTAATAATTAGAAAGCATTCCAAGTTACACATGCAGCTATTGAGGGCTAAACTACCATTCATATATACG	G/A	AACCTTTATACAGAGTTATCATTGGGATCTAAAATGCAGCTAGCAATAGTGCAGACACCAGCTATAACAACTGTTTTTCTAAATAATTGAAGAAGAAACA
Ca_LG_4:16444752	ATTATCTTCTATGTAGCCAGAATTATCATGTTCCTTACTTTCATTCAAAAATTTCCTAGCAACATGCTGCCATATATTCCTTAGTTCTTCCTCTCGTACA	A/G	GGCTTAATAAAATAATCACAAGCTCCGTGTCTAATTCCCTTCATGACAGTACTCGTCCTATCATCAACAGACATCACTGCAGCATGCAGAACATAACCAA
Ca_LG_4:16499218	GACTTTTCAGTGACATGGTGACAATTTCAACTGTTGCACCAAGGTTTCCTTTCAGTAGTGCTGCTTGATTGAATGGGTTTCTGTTCTTTCTATACCTTGC	C/A	ATTAATGTTATAGTACTCTCATTTGATCATTCAGCTTATTATAAATTGTTAAGGTTATTGGAGCTGCGATGTTCTATGTAAAAGTAGGAGACGCAGAAAT
Ca_LG_4:16541980	GCCATAAGTTACTTGAACCACTTCCGAAGGAAATGCGGCAACAAAAGAATCATTCTTCCAAACATGCACTGGTTCATTCACATGTGGAGCTATTTTCTCA	A/G	CAGCTTTTTCCATCAACATCAGTTGCTATCTCTAAACCAACTTCTTCCAAGGCTGCCACCCATTCCCTTGCCGACCCGTCAAATATAGGAATCTGTGCTA
Ca_LG_4:16995927	ATTTTGAACTTGAAATTTGTCTACATGCTTATCACCCCTTATAATATTTTTGACATACTGGTGATCTTTTGCAGCTACTGCATCTCAACCACTTCTTGAG	G/A	GAGGGTGACTGATCTGTAGCTGATGAATCATCAAAATAGTACTCAGTACTATAGTAGAAATACAAGAAAGTTGTAAGATTTTGGTAATGACTCTGACTTT
Ca_LG_4:17065080	AAGGACCTCCAGGAAACATGGGTTCATCAGGCTGGTGCTCCTGACCATGTTCATCATCAAACTGTTGCTGCTGCTGGTCATATTCATCATAAAACACATA	A/G	ATCGTGCTGGGACTGCTCATCCTGTTCTGCATCAAACATAAGATTTTGCTGGGGCTGCTCGTCCTGGGGCGCCTGCCTTTCCTCATTTCTCTTTCTCATT
Ca_LG_4:17065092	GAAACATGGGTTCATCAGGCTGGTGCTCCTGACCATGTTCATCATCAAACTGTTGCTGCTGCTGGTCATATTCATCATAAAACACATAATCGTGCTGGGA	A/G	CTGCTCATCCTGTTCTGCATCAAACATAAGATTTTGCTGGGGCTGCTCGTCCTGGGGCGCCTGCCTTTCCTCATTTCTCTTTCTCATTTTTTCAACAGCA
Ca_LG_4:17065141	CTGTTGCTGCTGCTGGTCATATTCATCATAAAACACATAATCGTGCTGGGACTGCTCATCCTGTTCTGCATCAAACATAAGATTTTGCTGGGGCTGCTCG	G/A	TCCTGGGGCGCCTGCCTTTCCTCATTTCTCTTTCTCATTTTTTCAACAGCAGCTCTCCTATTTGATTGTGTAGGAGGCGAACTCGCGAATAATCACCACC
Ca_LG_4:17313865	AGAAAATACATCCAAAACAGGAGAGACAATGGTTGTTTCGGAAAACTTTTCCAACAGCAACGGCAGGCAGCAGCAAACGGCGGCAGCGGCAGTAGCAGAC	C/T	GGGGCAGCAGCGACGATTGTGGACGGAAGTGACAGCAGGAAACAGATCTGAAATTAATTCCAGCAAATATCCTATTGATATGCAGCCTTTATGGCAGATC
Ca_LG_4:17361117	GTACAAGGAGTAATTTTTAACAGAAAGATTGAAGATTGCTTGTGTTCATCAGCCTCTTAACAGGTTCATCAAAATGGCATTATGTTTGTGGAATTTCACC	C/T	TGTCATATGCTGCTTTTATGATGTTTAACTTACAAAACTTATATTACTTAAGAATTATATTCTTGATGACACTGCTGCAAAGTACCTGTATTTTAATTAT
Ca_LG_4:18177920	GCAATTACAGTTGCAGACCATTGTTATCATTGGCGTCCGGTGAATTATGGCTGCCAGGCAAAAACCTGCCGTATAAATATGGGGTTGTGGCTCATGTCCC	C/A	AAACATGCCAGATATAATAGGCGTTGCGTCCAATAGCATCCAGCACATAATCTGCAATGTCAGCAGATTGACAACAATATTGTAGACCGCAATTTAAAAC

Positions	-100 bp flanking sequences	SNPs within *desi* cultivar	+100 bp flanking sequences
Ca_LG_4:18178044	TGCGTCCAATAGCATCCAGCACATAATCTGCAATGTCAGCAGATTGACAACAATATTGTAGACCGCAATTTAAAACATTGCATAAAAGGGCAATAATATT	T/A	AACGGAAAAAGCAGCCTGTATCGGGTATAAAATCTCACAGAAATCACTAGGTTACATTTCCATCACCAAGGAAGTATATCCAACTTTNNNNNNNNNNNNNN
Ca_LG_4:18769377	CTGGGTGTCCTTGTGCAGTCACTGTTAATTGCTGCAGGGTTGCGGCCCACTCCTGCACAAACAACTGCTGTTAGCTGGGTAATTTGATACTAATTTCGCG	G/A	TCATGGCATTTGCTGCAATTTAAAGTTTGATTCGGTGGTAATTGTTTATTAAAACCTATGTACTTCATGATTAAGAACTTTAGCATGCATCTTTTCTAGA
Ca_LG_4:19118253	TTGTGATTTTGTGAGGGCTTAGGCAGCCACAAAATCCAGTTAAATGTTTAAAAATTTGTGAGGGTTTTTTCAGCCACAAAATATAAATTTTGTGAGGGGA	A/C	AATGCCTCCACAAAAGACTGAAGTGACATGTCCATTTGTGGCTGCATAGGCCGCCACAAAAGCAACTGAACGTTACTAATTATATTTTTAATATTTTTTA
Ca_LG_4:19118256	TGATTTTGTGAGGGCTTAGGCAGCCACAAAATCCAGTTAAATGTTTAAAAATTTGTGAGGGTTTTTTCAGCCACAAAATATAAATTTTGTGAGGGGAAAT	T/G	GCCTCCACAAAAGACTGAAGTGACATGTCCATTTGTGGCTGCATAGGCCGCCACAAAAGCAACTGAACGTTACTAATTATATTTTTAATATTTTTTAAAT
Ca_LG_4:19118290	AGTTAAATGTTTAAAAATTTGTGAGGGTTTTTTCAGCCACAAAATATAAATTTTGTGAGGGGAAATGCCTCCACAAAAGACTGAAGTGACATGTCCATTT	T/G	GTGGCTGCATAGGCCGCCACAAAAGCAACTGAACGTTACTAATTATATTTTTAATATTTTTTAAATTTAAACAAACAACAGACTAAATGTACTTGGAAAA
Ca_LG_4:19118304	AAATTTGTGAGGGTTTTTTCAGCCACAAAATATAAATTTTGTGAGGGGAAATGCCTCCACAAAAGACTGAAGTGACATGTCCATTTGTGGCTGCATAGGC	C/T	CGCCACAAAAGCAACTGAACGTTACTAATTATATTTTTAATATTTTTTAAATTTAAACAAACAACAGACTAAATGTACTTGGAAAAGAGAGCATGATGCC
Ca_LG_4:19118319	TTTTCAGCCACAAAATATAAATTTTGTGAGGGGAAATGCCTCCACAAAAGACTGAAGTGACATGTCCATTTGTGGCTGCATAGGCCGCCACAAAAGCAAC	C/G	TGAACGTTACTAATTATATTTTTAATATTTTTTAAATTTAAACAAACAACAGACTAAATGTACTTGGAAAAGAGAGCATGATGCCGCAGTTTTGGCTTCA
Ca_LG_4:19189914	AATCTTGTATCAGTATACCAAATATGAGAAGTCTTTTTAATAAAGGAAAATCATAGAAGGAGATAAAATGAAATGAAACCTTGCAGCCCTTCCAGATGAG	G/A	GTGAAGCAAATGATAACAGAAGCTTTAACCTTGATGGCCGCGCGAACCTGTATGTTTCGGCAAAAAGAAGTTTACTTTCAAATATATACTTCAATTAGTA
Ca_LG_4:19189926	GTATACCAAATATGAGAAGTCTTTTTAATAAAGGAAAATCATAGAAGGAGATAAAATGAAATGAAACCTTGCAGCCCTTCCAGATGAGGTGAAGCAAATG	G/T	ATAACAGAAGCTTTAACCTTGATGGCCGCGCGAACCTGTATGTTTCGGCAAAAAGAAGTTTACTTTCAAATATATACTTCAATTAGTATATAGTTCTTTG
Ca_LG_4:19189932	CAAATATGAGAAGTCTTTTTAATAAAGGAAAATCATAGAAGGAGATAAAATGAAATGAAACCTTGCAGCCCTTCCAGATGAGGTGAAGCAAATGATAACA	A/C	GAAGCTTTAACCTTGATGGCCGCGCGAACCTGTATGTTTCGGCAAAAAGAAGTTTACTTTCAAATATATACTTCAATTAGTATATAGTTCTTTGAGCAAA
Ca_LG_4:19439675	AGCAACTCTTCTTGATATTTTTGCTGCTAAGGAAATTTCTGTATGTAAAATGCTACAATTACGGCATGAAAGTTACCTTATAATAGTATTATTGTTTGCT	T/A	TAAAGTAAATTATAAAACTGATTAGGTAGCAGCTAACAATGATTATCCATTGATATTTTTCCCTAGATATATTAATATTCAATTATCTAGCCCCTGCATT
Ca_LG_4:19697024	AATGTAAAGTTTGACTTTTTGTATGGTAGTAATAATAATATTCAAGTTTTTCTTTATTTAATTTTTTGCTTTTGCAAATTGCAGCTATCTACTAGAGTTT	T/A	TTGGCAAAGGAAGGTTTTCTTGTTGTGGTGGTGCCTTACAATGTGACATTTGATCATTCTCAAGCTGCTAAACAAGTATATGAGAGATTTCATACTTGTT

**Positions**	**-100 bp flanking sequences**	**SNPs within *desi* cultivar**	**+100 bp flanking sequences**
Ca_LG_4:19697065	TCAAGTTTTTCTTTATTTAATTTTTTGCTTTTGCAAATTGCAGCT<br>ATCTACTAGAGTTTTTGGCAAAGGAAGGTTTTCTTGTTGTGGT<br>GGTGCCTTACAA	A/G	TGTGACATTTGATCATTCTCAAGCTGCTAAACAAGTATATGA<br>GAGATTTCATACTTGTTTGGATACAATCCTCACATATGGGTT<br>GCCTCAAGCCAATTTG
Ca_LG_4:19839586	CCCACTACCTGCACTAGTCCCATTTGGCCTAGTTTCAGGTTGC<br>AGCTGGTTGGTGAAGTTGTCATGGGTACAAGACAAAATGACA<br>AATATTTAATCCAAG	G/C	AAAAACAAAATGCTACACAATCTACCACTTATATTAATTCTCC<br>CTTAGATGTCAACTTGGGTTTTGAACCTAGGCCAAATGGGTT<br>AATGTTGGATTTGGA
Ca_LG_4:19839598	ACTAGTCCCATTTGGCCTAGTTTCAGGTTGCAGCTGGTTGGT<br>GAAGTTGTCATGGGTACAAGACAAAATGACAAATATTTAATCC<br>AAGAAAAACAAAATG	G/A	CTACACAATCTACCACTTATATTAATTCTCCCTTAGATGTCAA<br>CTTGGGTTTTGAACCTAGGCCAAATGGGTTAATGTTGGATTT<br>GGATTCAATTCACAC
Ca_LG_4:19839602	GTCCCATTTGGCCTAGTTTCAGGTTGCAGCTGGTTGGTGAAG<br>TTGTCATGGGTACAAGACAAAATGACAAATATTTAATCCAAGA<br>AAAACAAAATGCTAC	C/A	ACAATCTACCACTTATATTAATTCTCCCTTAGATGTCAACTTG<br>GGTTTTGAACCTAGGCCAAATGGGTTAATGTTGGATTTGGAT<br>TCAATTCACACCTAT
Ca_LG_4:19839612	GCCTAGTTTCAGGTTGCAGCTGGTTGGTGAAGTTGTCATGGG<br>TACAAGACAAAATGACAAATATTTAATCCAAGAAAAACAAAAT<br>GCTACACAATCTACC	C/G	ACTTATATTAATTCTCCCTTAGATGTCAACTTGGGTTTTGAAC<br>CTAGGCCAAATGGGTTAATGTTGGATTTGGATTCAATTCACA<br>CCTATGACAAAGATT
Ca_LG_4:20666584	AGAAAAAAAAATCATCACACATCATCCAAAAATACTAGTCACT<br>AAGGAAACAAGATCATTGTTATAGATGAACCTGCTGAAGCCAT<br>GAAACATGTGGAAC	C/T	GGATGAAGAAAAAAACAGAAACCTCTGACTTACCGGATGCC<br>GCAGCCTTTCATATCAGGCTGACACTTTGTGAAAACATCAAA<br>TTAGTAATCCAAAAGCC
Ca_LG_4:21216323	CATGCAACTATTTTACAACCCCTCTAATGTTGAAAAAAAAAATA<br>TTTAAAACCTCACTCATTTCTCTTTACAGCTAAATTATATATAC<br>ACAAAAATTGTC	C/T	AAGGCAGTGGGGCAATAAAATAGGATCTTTGTCCCTTGGAG<br>CAGCTTTTTCACTTGGCATTTCCACTTCTTCTTTTAGCATCAA<br>TTGATCCTGAATGCTT
Ca_LG_4:21216549	GTTTCCTTGTTGACGATGAGATCATACTCCTAATTCACATTCA<br>AAAAGTTATAGGAAATGATATAAAGTAAGTTAGTAGTTGATAA<br>TAATATGATCCTTT	T/G	AAAAGAACTTAAGCAACATGAAATGCAGCAATACCTGGTTTC<br>TAAGTTCTTTGCTTGTTTATAATGAGTTGATTTATTTGGTCTA<br>GACGCTAATACATTG
Ca_LG_4:21288798	CCCACCCCCACCTCCACCACCACCTCAACAAACCTCATCATC<br>TTCAATACCTCCTCCTCCTCTTCCTCCTCCAAGAAGTGTGAAC<br>AACTCTGCTGCTATG	G/C	TCCGGTCCTCCTCCACCACCTCCACCACCACCTCAACAAAC<br>CTCATCATCTTCAATACCTCCTCCTCCTCTTCCTCCTCCAAG<br>AAATGTGAACAACTCTG
Ca_LG_4:21876993	GCTTAAAACTGGTTTTATTTTCTATTGGATGCTTAGCTTCAGAT<br>TTTCCAATTTGTGCCAAGATTGCTTTTTTTGTTGTTGATAGGCT<br>TTAGGAAAGCAC	C/T	GCTTGCTTGCCAGCCTGTATGAATGGCTGCTGCCAGGCTGT<br>TCACGATACTTTTGATTTCCTATTAGTTGTTCACGATACTTTT<br>GCATTCCTATTAGCTA
Ca_LG_4:21928427	TTTTTTTTTAATACTGATTTATTGTACCATTTGTTAGAATAAATG<br>GGCAGCATCAAAATCAAATTCAAAATCAAATTCAAAATCAAAA<br>TCAAATTCAAAT	T/A	TCAAAATCCTGTTATAGGGACTGCTCCTGCACAACATGGTAA<br>CTTAATAAATCATGTGGATCATTCAATACCATCTACTTCTACT<br>GCAACAAATCCAAAT
Ca_LG_4:22125617	TTTTCCTTCCTTCGTCGCCCCTCCCCTCTTAGGTACAGCACCA<br>CCGCACCGCTGCTCCACTGCATAGCACCACCACACCGCTGCT<br>CCACTGCAATACTGT	T/C	CGTACTGCTGCAACACACCGCACCGCTACATCATCACTGCA<br>TCGTTACAGCACCGCCGCTCCACTGCAACATTACTACAGATC<br>TTTGTTCTCGTTGTCTC

Positions	-100 bp flanking sequences	SNPs within *desi* cultivar	+100 bp flanking sequences
Ca_LG_4:22254115	GACCCCTACTTGATAATCTAAAAACTTTCAAGAGAAACCAAGTGCTAATGGGTTTTCTAAAACAATATCAAAGAAGGGGCAAAAATACAAGAAAGGAAGA	A/C	AAATTTCTTCTTTTTATGCAAAAAAAAAAAAATAATTGATGGAGAAAATAACAAAAAGAGAAAAAAGAATCCACATTTTCACAAGCTGCCTAGTGTGTACA
Ca_LG_4:23575398	GGATTTTGTATCAGTTATTTTGAGACTGAAAAATAAATTGTATAAAATGCAGCATTTAGAAGATATGAAGCAGCAGGGTGGTTGAGAAAAATGGTTGGAG	G/C	TTGTTGCCGCTAAAGATTTACCAGCAGAGCCTTCTGAGGAAGAGTTTAGGCTTGGTTTGAGGAGTGGGATCATTCTTTGTAATGTTCTTAACAAGGTTCA
Ca_LG_4:23846047	CGAAAGTAATCCTTACTTGGCCTTACCGTTGAGGTGTTTGTTAACATGAGCTTGAAATTTGACTTGAGATCATGCTTCTTGTTAGTTCTAGCTGCAATTT	T/G	TCTTGATTTCATTGTTCTATCAATAATGGGATGTGATATGAGTACAGGAAATATAAGATATAAGTTATTTTATAATTTTATAATTTTATTTGAGAGTATA
Ca_LG_4:23846209	AGTTATTTTATAATTTTATAATTTTATTTGAGAGTATACTATTTTTTGTTAATTTTCTATCTAATATTTTTAATGTCTTTTTTATATACTTTGATTTAAA	A/T	GTTGCAGCTCTGTTTTTTTTTTATTATTTAAAATTTATTGTTGGTGTTTAATTTGAATTTTGCATAGAAGATACTTTCAGATACTTTTGTTTTCATGTGA
Ca_LG_4:23846243	TATACTATTTTTTGTTAATTTTCTATCTAATATTTTTAATGTCTTTTTTATATACTTTGATTTAAAGTTGCAGCTCTGTTTTTTTTTTATTATTTAAAAT	T/A	TTATTGTTGGTGTTTAATTTGAATTTTGCATAGAAGATACTTTCAGATACTTTTGTTTTCATGTGAGTATTTGATAATTGACACCAGTTCTATATTGTAT
Ca_LG_4:23846424	CACCAGTTCTATATTGTATTTCTTTGTGCTTTGAGTGATTGACTTGATAAACAGCCATGGTGTTCATTGTGCTTTCACCATATAAGCTTATTTTAGTTAG	G/A	TCATGGAGTCACCCACATCTCATGTTTGCTGCAGTGGAGAGGCAACTAGAGCTTGCTGCAGTGGAGAGGCAACTAGAGCAATTCCAAACCGATTGTGATG
Ca_LG_4:24393454	GTCCACATTTAAATGTCTTGTGAACAGCACCCAACTAGTTTCATCATCTAATAACTGCAGTTGGTGAACAAAGGTTGCATCTGCAGCATGTTGTGCAACA	A/G	TTTGCATTGCGAGTGGTAACAAGAATTCTGCTGCCCGTCGATGTATCGGGAATGGCCTCTTTCAAGGTATCCAAGTACACTTGAGAAGTTTCTTTTATGC
Ca_LG_4:24394311	GAACATCCAGAAATAGCGCATCCATCAATTCCTTTTCCCCTCTTATTGATTGGATCTTCTCTTTGAGCCCAGTGACAGGTTGATACAAAGAGGCAGCTGG	G/T	TTCCATCGCCTGTGCTGGTGCTGACGTAGCACCAGAACTGTCAACAATGATTTCACTCTCTTGTATGCTGCTAATTGGGCCTTCTAATAACGGTACAATT
Ca_LG_4:24394319	AGAAATAGCGCATCCATCAATTCCTTTTCCCCTCTTATTGATTGGATCTTCTCTTTGAGCCCAGTGACAGGTTGATACAAAGAGGCAGCTGGTTCCATCG	G/A	CCTGTGCTGGTGCTGACGTAGCACCAGAACTGTCAACAATGATTTCACTCTCTTGTATGCTGCTAATTGGGCCTTCTAATAACGGTACAATTAGCTCTTG
Ca_LG_4:24394321	AAATAGCGCATCCATCAATTCCTTTTCCCCTCTTATTGATTGGATCTTCTCTTTGAGCCCAGTGACAGGTTGATACAAAGAGGCAGCTGGTTCCATCGCC	C/T	TGTGCTGGTGCTGACGTAGCACCAGAACTGTCAACAATGATTTCACTCTCTTGTATGCTGCTAATTGGGCCTTCTAATAACGGTACAATTAGCTCTTGTT
Ca_LG_4:24763278	GTATATGTGGATTGCTTGTGTCAGGTTGCTCCTGCAGAACTCGAATCGGTGCTGCTAAGTCATCCCCTTATTGTTGATGCAGCAGTTATACCGTGCGTTA	A/G	CCCTCAGCGCAGTTTCACTCTTTTTTCATTATCTTTGACTTTCTCATGTGAAAAGATATGTTGTAGGGTTGAAGATGAAGAAACTGGGCAGATACCGATG
Ca_LG_4:24763287	GATTGCTTGTGTCAGGTTGCTCCTGCAGAACTCGAATCGGTGCTGCTAAGTCATCCCCTTATTGTTGATGCAGCAGTTATACCGTGCGTTACCCTCAGCG	G/A	CAGTTTCACTCTTTTTTCATTATCTTTGACTTTCTCATGTGAAAAGATATGTTGTAGGGTTGAAGATGAAGAAACTGGGCAGATACCGATGGCGTATGTG

Positions	-100 bp flanking sequences	SNPs within *desi* cultivar	+100 bp flanking sequences
Ca_LG_4:24763309	CTGCAGAACTCGAATCGGTGCTGCTAAGTCATCCCCTTATTGTTGATGCAGCAGTTATACCGTGCGTTACCCTCAGCGCAGTTTCACTCTTTTTTCATTA	A/C	TCTTTGACTTTCTCATGTGAAAAGATATGTTGTAGGGTTGAAGATGAAGAAACTGGGCAGATACCGATGGCGTATGTGGTGAGAACAGCTGGTTCTGAAC
Ca_LG_4:25231360	GAGTTAGGTTGTAGGAAGAAAAAAAGAATAATGAATTGACTTACTTTGGTTGTGCTTGGCAACAAGCTGCGTAACTTAGCAAGATGATTATTGATTCTCT	T/C	CTCTACGTCTCCTTTCAGCTTCACTATGACTCTTTGATGCAGCAAGAGCCTTAGCTTCCATGATTTCTTGAGCACTCATCTTTCCAATTTCACCTTGAAG
Ca_LG_4:25425715	ACCCATATACGACAACATGTATACAGCCCTAGAATACATTTTCCACGGACGACCCAAATATGTTTGAACGCTGCCCTTAACTGGGGCAAGATCTGGGGCG	G/A	GGTAGGATCCGACAATCATGAATAGATATACCCGTATTTTGATTCGGGTCTTTTCTATTTTGGCCGTGATAGTGTTCTTTTGTTGGTCCATGGGCTTTC
Ca_LG_4:25443745	ATTGAATTTGTGAGGGCCAAAAGCCCTCACAAAGGAGTGGAAGCAGTCAAAAAGGAGATATTTGTGGCAGCCAAAACGGCCACAAAATTTGAAAATTTCG	G/A	TTGCCATTTGTGAAGGCAAAGGCTGCCACAAAGTCCTTATTTGATGAGGGCTTAGGCCGCCACAAATGTCTCATGTTGTGAGGGTTTAGACCGCCACAAA
Ca_LG_4:25845032	CTGCAGGGGTTGAGATGTGAGACGGTGCAGCAATTTCAACAGAGTTTAGTGAACCCTTTCTCTAAGAGGTTTATGTGGGAAAACAGCTCTCCTCGAGGAT	T/A	TGACTTGGTCTTGCCAAGATGATGTGGTTTTAAAGGAGAAATACTGCGGTTTGCCATCAAAGGCTGGTTTGAAGCTCCTAGTCACTCAAAAGTTTGTGAT
Ca_LG_4:25845067	TCAACAGAGTTTAGTGAACCCTTTCTCTAAGAGGTTTATGTGGGAAAACAGCTCTCCTCGAGGATTGACTTGGTCTTGCCAAGATGATGTGGTTTTAAAG	G/A	GAGAAATACTGCGGTTTGCCATCAAAGGCTGGTTTGAAGCTCCTAGTCACTCAAAAGTTTGTGATAGCAAGCAGCAACTAGCTTGTTTAATCTTTTTTTT
Ca_LG_4:25845078	TAGTGAACCCTTTCTCTAAGAGGTTTATGTGGGAAAACAGCTCTCCTCGAGGATTGACTTGGTCTTGCCAAGATGATGTGGTTTTAAAGGAGAAATACTG	G/T	CGGTTTGCCATCAAAGGCTGGTTTGAAGCTCCTAGTCACTCAAAAGTTTGTGATAGCAAGCAGCAACTAGCTTGTTTAATCTTTTTTTTAGGCAATACTA
Ca_LG_4:25845079	AGTGAACCCTTTCTCTAAGAGGTTTATGTGGGAAAACAGCTCTCCTCGAGGATTGACTTGGTCTTGCCAAGATGATGTGGTTTTAAAGGAGAAATACTGC	C/T	GGTTTGCCATCAAAGGCTGGTTTGAAGCTCCTAGTCACTCAAAAGTTTGTGATAGCAAGCAGCAACTAGCTTGTTTAATCTTTTTTTTAGGCAATACTAG
Ca_LG_4:25845130	ATTGACTTGGTCTTGCCAAGATGATGTGGTTTTAAAGGAGAAATACTGCGGTTTGCCATCAAAGGCTGGTTTGAAGCTCCTAGTCACTCAAAAGTTTGTG	G/A	ATAGCAAGCAGCAACTAGCTTGTTTAATCTTTTTTTTAGGCAATACTAGCAAGTTTAATCTCTTAAACCAATTAGTCTTAATTTTCAGTTTGTAACTTGC
Ca_LG_4:26710224	TGCCAGAGCGCACGATGCTGCTGCGCTCTGCGTCAAAGGAAACTCCGCCATTCTTAATTTTCCTCACCTTGCTTCTTCTCTTCCGAAACCCGCTTCTTTG	G/A	ACACCTCGCGACGTTCAAGCCGCAGCGGCTAAAGCAGCTCAAATGGACAACAAAATTGAATTATCATCGTCCACATCATTATCATCGTTCTCATCATCGA
Ca_LG_4:26710248	GCTCTGCGTCAAAGGAAACTCCGCCATTCTTAATTTTCCTCACCTTGCTTCTTCTCTTCCGAAACCCGCTTCTTTGACACCTCGCGACGTTCAAGCCGCA	A/G	GCGGCTAAAGCAGCTCAAATGGACAACAAAATTGAATTATCATCGTCCACATCATTATCATCGTTCTCATCATCGACAACGTTGTCATCAGCATCATTGA
Ca_LG_4:27047159	AGCAGGAAATTACAAACATATCTGGATATATTTAGTGGCGCCGACGCTCGGTGCTCTTGCTGGTTCTGGAGTTTATACGCTTGTCAAGCTGCGTGACAAC	C/T	GAAGCTAATCCAGCGCAATCGGTTAGAAGCTTCCGTCGCTAGATCTGGCTGCGGCACTGCTCTCTAGTCTTTACCATACCCTACTGCTCATGTTTCAAAC

Positions	-100 bp flanking sequences	SNPs within *desi* cultivar	+100 bp flanking sequences
Ca_LG_4:27047161	CAGGAAATTACAAACATATCTGGATATATTTAGTGGCGCCGACGCTCGGTGCTCTTGCTGGTTCTGGAGTTTATACGCTTGTCAAGCTGCGTGACAACGA	A/G	AGCTAATCCAGCGCAATCGGTTAGAAGCTTCCGTCGCTAGATCTGGCTGCGGCACTGCTCTCTAGTCTTTACCATACCCTACTGCTCATGTTTCAAACTC
Ca_LG_4:27047166	AATTACAAACATATCTGGATATATTTAGTGGCGCCGACGCTCGGTGCTCTTGCTGGTTCTGGAGTTTATACGCTTGTCAAGCTGCGTGACAACGAAGCTA	A/G	ATCCAGCGCAATCGGTTAGAAGCTTCCGTCGCTAGATCTGGCTGCGGCACTGCTCTCTAGTCTTTACCATACCCTACTGCTCATGTTTCAAACTCCTTGT
Ca_LG_4:27060282	AATATTGCAGAGGGTTGCATTGCTGGAGGTGTTGCCGGTGTTGCTGTAGAAGCAGCTTTATACCCCATTGATACCATCAAAACTCGACTACAGGTGAATT	T/G	AAGTATATTGAGTGAATATTTTTAATTCTATTTTGTCTGTTTTTATACCCCTCGGGTGGGGATTGTCAGGTACTAATAATATATACCCCATTTGGATGCT
Ca_LG_4:27089264	TTTATTTTATATATATATATATATAATTACATAATTTATATCACATGTAAAGAGCTGATTCATTCTTTAGTCTGTAAAGCAGCAGCAGCGTTTTGGTCAATC	C/T	GTAACGCTGAAAATAATTGTTTCTAATTCCATATGAAAGTATTTTATGGCTTCTGCTATATTAGAAGTTCAAATGTAATTCCATTTCATTGGTTTTTATG
Ca_LG_4:27089341	CAGCAGCAGCGTTTTGGTCAATCGTAACGCTGAAAATAATTGTTTCTAATTCCATATGAAAGTATTTTATGGCTTCTGCTATATTAGAAGTTCAAATGTA	A/T	ATTCCATTTCATTGGTTTTTATGACATTATTTGTGCTTTTCCATAGGATCAAATTGCTGCATATGTTGAGTACTTGATTCAGTTTACATCCGAACAATTT
Ca_LG_4:27092161	TAGAAATTGGATGCACTTGCATTTCTTGCGTGCAATAGGGACTGCAATGTCCATGAGAGCTGGCATAGCTGCTGATGCTGCAGCTGCTTTGCTTTTCCGC	C/T	ATACTTTCACAGCCAGCCTTACTTTTCCCCCCTCTTAGGCAAGTTGATGGAGTTGAAGTTCAGCATGAACCTTTGGGTGGTTATATATCTTCCTACAGCA
Ca_LG_4:27092349	CTTCCTACAGCAAGCAGGTTGTTTTCATATTCATTTTCCATTTTATTTATATTTCATTCTTATTTTGTAAAGTGTTGAAGATCTAATATATTTGTTGTTG	G/C	ATCAGATAGAAGTTCCTGCTGCCGAAGCCTCTATTGACGCTACTGCCCAAGGCATTGCATCATTGCTGTGTGCTCATGGTCCAGAAGTTGAATGGAGAAT
Ca_LG_4:27092420	GTGTTGAAGATCTAATATATTTGTTGTTGATCAGATAGAAGTTCCTGCTGCCGAAGCCTCTATTGACGCTACTGCCCAAGGCATTGCATCATTGCTGTGT	T/C	GCTCATGGTCCAGAAGTTGAATGGAGAATTTGCACCATTTGGGAAGCTGCTTATGGCCTGATTCCTGCAAGTTCCTCAGCTGTTGATCTTCCAGAGATTA
Ca_LG_4:27118358	AGATAAAAGCCTATGTTTGTGCAGTTGCTTTATAGGCTGCTGTGAGATTGTTTGGGATTATTTACGAGGAAAAATGAGAGATCATTCATAATGTCTAAAG	G/T	GATGAATGATGTTTATTGGATTGGAGAGGATCAGATGTGTCGTAATTTTTTTTGATTGATTTGTTACTTGCTATTCTAGATTAAAGCAAGCTGCTAATAA
Ca_LG_4:27251145	TATCTGCAGTATGGGTTTTAATTTTATATCATCAAGCATAACCTAACGGTAGATGATAATGCCTCTAGGCTGAAACTAAGGTTTGCAAAAGATAATAATT	T/G	ATGCATGAATTTAATTTCAAATGCAACATTTAACAACTTAGCAGCTTATATGACATATCTGTATTTGATGATAGATAGAACAAAAATAAAGCTAATACAA
Ca_LG_4:27499435	GAATACATTAAGTCTATGAAGTTAGTAGCAGATAGTGTAGCGTAGCGGCCGACTCCCAAAACGCTATAGCTGGTAGCAGCATGGTATGGCCGCTACGTCG	G/A	AAGTCTAGAAAAATATTACAAGCACTTTCAACCATACACATATGATGGCTAAAAAATTAGATTAATGCACAAAACTAAACACATATTTATAACTAATCAC
Ca_LG_4:27502909	TTGGGAAGTATATATGCGGTAAGGTAACCAATTTGCAATGAGAATCAGTTACGTTAAGGAATTATTATAGATAGAAGATATGAGCATCCTAATTCTCAAC	C/G	AATTATAATCAGAGAAACCCTCAGAGTTGGCAGCCAAATAGAAAGCTGCCATCTGCATTGGATATAAAGCCTATATGATCAACAAACTTAAATCACTACA

Positions	-100 bp flanking sequences	SNPs within *desi* cultivar	+100 bp flanking sequences
Ca_LG_4:27628912	AAATTTATATTTATATCATTTTATGTAACCCAGCAGCCGCAACACACGAAATACAAACAGTGCTGCTGTCTTCTTCTCCTTCTTTTTTGAATGCGTGATA	A/T	TTGCCAGAGAGAGCAAAAAAACAGCTGCACAAGACAGCCAGCAACCAGCGATGGTGACAGAGGGAGACTGGGTTTTCGATTTTTTGCCGAAAAATGAATT
Ca_LG_4:27630244	TGTAAGAGAGCTGCTAAACGACAGTAAAGAAGCCAGCTTTACTTTATTTTGTATAATATCTGCAAGTTATTGAGTTGTGTAAAAATGTTGATATTTCGCG	G/A	CCGCGATAGCGGCAATATTTCTCACTATTGATAACATAGATTAGAAAACAAAACATTTAATGCATTGACTACAATAAATGCAGCCATTTTCGATAAATTT
Ca_LG_4:27859952	AGAGTCCAATCTCAGTAGCAGCAGTAGCACCAAAGAAATATCACAAAACTTTCCTCGGACCCCAAAGCAATTACTTTATGAATGTGCTAGTGCACTTTCG	G/A	GATGGAAATGAAGTAAAAGCCTCGGCGATGATAGATGATCTAAGGCAGATGGTTTCGATTCAAGGAGATCCTTCCGAAAGAATTGCAGCCTACATGGTGG
Ca_LG_4:27880564	TCCCAGGTTCATATGCTGGTTTCGCAGGAGACCTTATAGGCATATTCTGCTGTTGAAACTGCTGCGGTAGCTGGGACCGTTGCTGAGGCATTGCCTTTAA	A/G	GAGTTGCTGCTGCTGTTGATATTGCATTAGTCTCTGTTGTTGCAAAAGATTCATCTGTGCAGCAGTAGCCTGAGAGGTTTGCCTTGACATATGGAGAAGT
Ca_LG_4:27910434	GGAGGTGTTCTTGAGGGGAAGTCTTCATAATATAATGCTTGAGGCATGTAGGAGAAAATGATCTCTGGTTTTTGTTGTGGTGAAATGCTGCTTACATGAG	G/A	GAAGGATTGGACAGATTTTGTATAGTTTTAGGATACCTTTGTTTTTCTATTCTCTTTTTGTAGAAGCCCCCTTTTTTCATGAATGATCATTTTTACTCAT
Ca_LG_4:27947794	CTATGTGGCCCTCTTGTAATGTCATTAACTTCCATTGCCATTGGATTGAGAGGAAAAAAACTGCATACTGCCATTGTGCAGGTACTTATGTTGCATATTT	T/C	GTGGAATGAATTTAAATCTCTATAGACACATGCAGCACATGGAAATTATGTCTGATAATATATCAGAACAATGTGTGTATGTTTCTTCTGTAATAAATGT
Ca_LG_4:28020670	TCTGGAAAGCCAATCGCAGATCGATGTAGATTTGCAAATCTGTCTTCTTTGAAATCATAACTTACAACCTGATTCCCAGAATTAGCAAATCAGAATTAAA	A/C	TCATATGATGCAGCTGAAAAAACTATCAAAATCACCATTCAATTTATATTTGTATTGAAGCATGATACTAGGTTAGAAAATGATTTCCAAAACAGAGATC
Ca_LG_4:28021749	CCAATTGAAAGCACCAACACACCCCGAGTGCTTATGCTAAGCATGAATCTGTAACAACACATTCCTTCTACAAGCTGCCATAGAAACACCACCAGTCCGG	G/A	TCCTTAAAGACACAACCCCACGTTGTCAGCCCCTCTTATCGCACCCAACATCCACTTGTGCAGTAAACTCGGCAGAAACAGAATTGGACAACAATCGGTT
Ca_LG_4:28021771	CCCGAGTGCTTATGCTAAGCATGAATCTGTAACAACACATTCCTTCTACAAGCTGCCATAGAAACACCACCAGTCCGGTCCTTAAAGACACAACCCCACG	G/A	TTGTCAGCCCCTCTTATCGCACCCAACATCCACTTGTGCAGTAAACTCGGCAGAAACAGAATTGGACAACAATCGGTTATGCTGCAGAGTTTCAATCATT
Ca_LG_4:28021789	GCATGAATCTGTAACAACACATTCCTTCTACAAGCTGCCATAGAAACACCACCAGTCCGGTCCTTAAAGACACAACCCCACGTTGTCAGCCCCTCTTATC	C/G	GCACCCAACATCCACTTGTGCAGTAAACTCGGCAGAAACAGAATTGGACAACAATCGGTTATGCTGCAGAGTTTCAATCATTTGTGTCCTCATATTGGCG
Ca_LG_4:28021797	CTGTAACAACACATTCCTTCTACAAGCTGCCATAGAAACACCACCAGTCCGGTCCTTAAAGACACAACCCCACGTTGTCAGCCCCTCTTATCGCACCCAA	A/G	CATCCACTTGTGCAGTAAACTCGGCAGAAACAGAATTGGACAACAATCGGTTATGCTGCAGAGTTTCAATCATTTGTGTCCTCATATTGGCGTGGTTGAT
Ca_LG_4:28913588	CAGGATGTTTAGATTTTATCATAGCAGCAATTCCACTAACATGTGGACTTGACATTGAAGTACCAGAAAGTATATTGAATTTCACTCTTCTTTGATCTGT	T/C	TGGTAAACTTGATGGACCAGTGACACCACTCCAAGCAGCAAGAATGTTCACACCAGGAGCAACTATATCAGGTTTTAGAATTTCTGGTGTTAGATAATTA

Positions	-100 bp flanking sequences	SNPs within *desi* cultivar	+100 bp flanking sequences
Ca_LG_4:29821986	CACAAAGTTTGTAAAACACGTTGCCATTTGTGAGGGCCTTTGCAACCACAAAAGGCTGCCTTTGTGAGGGCCTTTGCAGCCACAAAAACTTGCATTTGTT	T/G	AGGGCACATGCCGCCACAAAGGACAGACTGATTTTAAAAAATTCAGCCTTTGTGAGGGCCTAGGCTGCCACAAAATCCCTCCTTTGTGAGGGTTTAGGCC
Ca_LG_4:29822021	GCCTTTGCAACCACAAAAGGCTGCCTTTGTGAGGGCCTTTGCAGCCACAAAAACTTGCATTTGTTAGGGCACATGCCGCCACAAAGGACAGACTGATTTT	T/C	AAAAAATTCAGCCTTTGTGAGGGCCTAGGCTGCCACAAAATCCCTCCTTTGTGAGGGTTTAGGCCGCCACAAAGTACCATCCAGATTAAAAATAATATTT
Ca_LG_4:30361309	TTGATTATTGACTGAAAAACTATGAATAATTGCATGACTCATAAAGGTGACATTCTGTACTTGCTAGATTGAAGAGATTATTGCTGCTTTGTGTATTAAG	G/C	TGGATGTAGTCAGCATTCTGAAAATTTATAAAGGAGAATGGCAGTTACATTTGCAGCAAATATGTGTTCCATCACAAGTTCTAAAACGAAGGAAGGGATC
Ca_LG_4:30361322	GAAAAACTATGAATAATTGCATGACTCATAAAGGTGACATTCTGTACTTGCTAGATTGAAGAGATTATTGCTGCTTTGTGTATTAAGTGGATGTAGTCAG	G/T	CATTCTGAAAATTTATAAAGGAGAATGGCAGTTACATTTGCAGCAAATATGTGTTCCATCACAAGTTCTAAAACGAAGGAAGGGATCAAAAGGTTTGACA
Ca_LG_4:30791507	CATATTATCTTGACCTTGACATTGATGATAATGCAAACTCTTTCATCAATGCCAAGCTGCAAAAGGCCACAGCAAGTGGCTTTGGAACACCAAGGAAGAG	G/C	TTATTGGACTGTTCTTAGAGAAGCTGCCAAAAAGGAAGCTGAGGCTAGAATCCGACTCGGCTTGGAGCCAGCTGAGCTGTTAATAGTTAATCCTAATAAA
Ca_LG_4:30842459	GCCAACCATAACTGGAGGTGATGGGGACTGGTTGGAGCTTGCAGTCTGCACTTGTGAGGTGGACGATTTTGGATTTGCTGCAAAAGATATTTGTGTGTGC	C/G	CCCTGCTGCTGGGAACGAACCTGCTGCTGTGGAGGATTTTTAATCGATGATGACGATGTTGAAGCCATAGTTGGAGGTGGTGACAGAGAAGTGTTGGTTG
Ca_LG_4:30843944	CACATGGGCTGGTATTGGAAAAGGGTAAGGATTATTCTGCAAAATAGCCAAGTACTGAGTTTCATTGCCAGGCATATTTGGATAGGTGAAACTCATTGTT	T/A	GGAGCAGCAGCAGCAGCTCCAGCACCAGACGTATTCGGTGGAGCTGAATTGGATGTAGAAGATGGCTGTCCATTGCTTGTTACTGGCAAAGACTTCACAG
Ca_LG_4:30843980	CTGCAAAATAGCCAAGTACTGAGTTTCATTGCCAGGCATATTTGGATAGGTGAAACTCATTGTTGGAGCAGCAGCAGCAGCTCCAGCACCAGACGTATTC	C/A	GGTGGAGCTGAATTGGATGTAGAAGATGGCTGTCCATTGCTTGTTACTGGCAAAGACTTCACAGATCCAGGTCGAACAGATGCTGCTGCTGCCACCGCTG
Ca_LG_4:30844004	TTCATTGCCAGGCATATTTGGATAGGTGAAACTCATTGTTGGAGCAGCAGCAGCAGCTCCAGCACCAGACGTATTCGGTGGAGCTGAATTGGATGTAGAA	A/G	GATGGCTGTCCATTGCTTGTTACTGGCAAAGACTTCACAGATCCAGGTCGAACAGATGCTGCTGCTGCCACCGCTGCTTGTTGCTGATTCATGGGGAAGA
Ca_LG_4:30845093	TCTTAACTTCAAAATTGAACTTACCATTTTTCTCTGAGCCTGTTTGATGTTGCTGCTGCAACTGATGCTGATGCCTCTGACCATTATGATGTTGCTTCTG	G/T	GTTAAGATTATTGCCATTGTCATTGCCATTTGTATCCAACCTATCTGATTTCTCTAACTGAAGCTGCAGGTCAATGCCTCTCTCTTTCTGCACAGTTATA
Ca_LG_4:31306006	TAAGAATCTCTCTTGCTAACACATAAATGATCATAAACACAAAAATTCTATAGCATTTGAAGAAGAGTCTTACTCTTACCTAATCATTTTGGCAGCGCCG	G/A	AATCCGAGACTGTCATTAAACAACTCTGTCATGTATTTCTTCTGTACAAGCAGCTGAACCTCAGGATTGTTATAGATTCCTGGAAGATATGCCTCACCAG
Ca_LG_4:31628069	TTTTTCAGCCACAAAATCTAAATTTTGTGAGGGTAAAGGCCGCCACAAATTCCTGAAGGGACGTTACACTTTGTGGCTGCAAAGGCCGCCACAAAAGCCC	C/T	GAACCTTTGCATTTTGTGGCTGCAAAGGCCGCCACAAAAGAATAAATTTGCGTGGCATTTTATGGCCGCCCAGGCCGCCACAAAGTTTTATACCTGTTAC

Positions	-100 bp flanking sequences	SNPs within *desi* cultivar	+100 bp flanking sequences
Ca_LG_4:31673469	TAATTAAAATGATTTGCATCAGTGCCATTTTAACTACAAGCTGTTATCAGCTTTACCACTTTCTGTAAGATATATATCAGGGCGCGATGCCGATGATATC	C/T	GGTTCTGATATTACTATTGTTTGTTTGTGCAGTGTGAAACAAATGATGGGTGAAGCACCACACACTCAACTTGATGCTGAGTTAGCTGCAGGGATGGGAA
Ca_LG_4:31673483	TGCATCAGTGCCATTTTAACTACAAGCTGTTATCAGCTTTACCACTTTCTGTAAGATATATATCAGGGCGCGATGCCGATGATATCGGTTCTGATATTAC	C/T	TATTGTTTGTTTGTGCAGTGTGAAACAAATGATGGGTGAAGCACCACACACTCAACTTGATGCTGAGTTAGCTGCAGGGATGGGAATGCCAGATAATGGA
Ca_LG_4:31673486	ATCAGTGCCATTTTAACTACAAGCTGTTATCAGCTTTACCACTTTCTGTAAGATATATATCAGGGCGCGATGCCGATGATATCGGTTCTGATATTACTAT	T/C	TGTTTGTTTGTGCAGTGTGAAACAAATGATGGGTGAAGCACCACACACTCAACTTGATGCTGAGTTAGCTGCAGGGATGGGAATGCCAGATAATGGACCA
Ca_LG_4:31673547	AGGGCGCGATGCCGATGATATCGGTTCTGATATTACTATTGTTTGTTTGTGCAGTGTGAAACAAATGATGGGTGAAGCACCACACACTCAACTTGATGCT	T/C	GAGTTAGCTGCAGGGATGGGAATGCCAGATAATGGACCAAAGCTTATGTCTATGTAACTTTTTCTTTTGTAGTGGTTGGGAGATTTTGGTAGAAACTGGT
Ca_LG_4:32132436	AAAGCAAGTGTTGGGGTTCAAAGTTGGTGTTAAAGATTATAAATTGACTTAATATACTCTCGACTATGAAACCAAAGATACTAATATCTTGGCAGCATTC	C/T	CGAGTAACTCCTCAACCTGGAGTCCCTCTTGAATAAGTAGATGTTGCGGTAGCCGAATCTTCCATTGGGACATGGACAACTGTGTGGACCGATGGGCTTA
Ca_LG_4:32132463	TGTTAAAGATTATAAATTGACTTAATATACTCTCGACTATGAAACCAAAGATACTAATATCTTGGCAGCATTCCGAGTAACTCCTCAACCTGGAGTCCCT	T/G	CTTGAATAAGTAGATGTTGCGGTAGCCGAATCTTCCATTGGGACATGGACAACTGTGTGGACCGATGGGCTTACCATTCTTGATCTTTATAAAGGAAGAT
Ca_LG_4:32132465	TTAAAGATTATAAATTGACTTAATATACTCTCGACTATGAAACCAAAGATACTAATATCTTGGCAGCATTCCGAGTAACTCCTCAACCTGGAGTCCCTCT	T/C	TGAATAAGTAGATGTTGCGGTAGCCGAATCTTCCATTGGGACATGGACAACTGTGTGGACCGATGGGCTTACCATTCTTGATCTTTATAAAGGAAGATGC
Ca_LG_4:32132470	GATTATAAATTGACTTAATATACTCTCGACTATGAAACCAAAGATACTAATATCTTGGCAGCATTCCGAGTAACTCCTCAACCTGGAGTCCCTCTTGAAT	T/G	AAGTAGATGTTGCGGTAGCCGAATCTTCCATTGGGACATGGACAACTGTGTGGACCGATGGGCTTACCATTCTTGATCTTTATAAAGGAAGATGCTACCA
Ca_LG_4:32648363	TCGAAAAAGGTATGGTTTATTCTGCTAAATTTCTTTAACACATGAAAATTAATATCATTTGTTGCATAAATTAGTTTGAGATTTATATTGGAATTTATTA	A/C	TGTTTTTTGGAGCAGCAAAGGGAGCACTATGAGCGTTTGTCACAGAGTCTTAAAGCAGCAGGGCAATGACTTTTTTTGTTTTTTAATAGTATGTATCATT
Ca_LG_4:32678083	TATCACATATTCTTTATGATCTTAAGGCTGCACCAAGCTCTGTATTCTTGACAGCTTTCCCACAAGAGAAGTCAATACCTAATATGATTAGTATAAATGG	G/A	CTTCTTTTTTCTCATGAAGAAGTTCAATTCTAAAAACAAAGCATGCTGTATACAGTCTGAACAGATTGTGGCAGCTATGCATTTGGTAGATTTTGCCATT
Ca_LG_4:32934124	TCGGTATGGTTTCCACGCCCCATTCATGTTTTAGTCTTTGCATAATACATGAAGTTGCATGTTATTTTGTTGCTGCTAGCTTAGGGAATATGATTATTCG	G/A	GTGATGTGTTGTCTCAGTGGATTCTTATGTGTCCAATGTTTCTGTGCTGCTTTCAGTATCGATTCTCCAATGTAGATTACAACATAACAAGCGGTAACCG
Ca_LG_4:33011268	TCATTTACTCAAATATGTAGTTGTTGGCTTTTTAGGTAATGGAAGAGTTGGACGCATTGTGATGGCTGCTGCTGCTAAGCACCTTACACCAGTTGTGCTT	T/G	GAGCTTGGAGGAAAATCACCAGTTGTTGTTGATTCAGATATCAACTTAAAGGTATACTCTTACTGTCTCTTTTTGTATTTAACAAATAATTGCCATCACC

**Positions**	**-100 bp flanking sequences**	**SNPs within *desi* cultivar**	**+100 bp flanking sequences**
Ca_LG_4:33011292	TGGCTTTTTAGGTAATGGAAGAGTTGGACGCATTGTGATGGCTGCTGCTGCTAAGCACCTTACACCAGTTGTGCTTGAGCTTGGAGGAAAATCACCAGTT	T/A	GTTGTTGATTCAGATATCAACTTAAAGGTATACTCTTACTGTCTCTTTTTGTATTTAACAAATAATTGCCATCACCCTAGCTACCTTCTACCTCATTAAG
Ca_LG_4:33041382	AGCATCAACAGATTCATTGAATCAGCTCATAGCTGTTTTGCGTCTTGGATCAAGAAATGCTAGATATAGTGCAGCAAGAGCACTTCATGAACTTTTTGAT	T/A	GCTGAATATATCAGAGAATCAGAATTAGCTAAACAGGCCATTCAGCCACTGGTTGACATGCTTAACACAACATCAGGGAGTGAGCAGGAAGCTGCTCTCA
Ca_LG_4:33050134	TACAAAGCGTGCCTTGTTGCCAAGGGGTTTGATCAAACCCATTTTCCTCTCTATAAGAAATAACGAGTTTTTGTCTTCCGTTGGAGCCGACCCCAGTAGA	A/T	TGGGGATAAGTACTGTCTTATCTCATTCCCACTACCAATTGAGCTGCCAATAAAGGTAAAGGCTGAGGTGTTACACCAAATAACTACTAGTTCAACATAT
Ca_LG_4:33094489	CTTGTTTATCACCTAGAGATAGTAGCTAGGTGACACTGTGATGTTCTGGGGACAACACTTTGGCACGGATGATAAAGCTGCACTATGGTATAATTATACC	C/A	TTGGCATTAGGAAAATCAGTGGAGAAACAAGGCTGGATAGGTGTGTTTATACTGAAATTGCAGCAGGCTGCAATATTGTGAAGATTTCACATGTATGATA
Ca_LG_4:33094497	TCACCTAGAGATAGTAGCTAGGTGACACTGTGATGTTCTGGGGACAACACTTTGGCACGGATGATAAAGCTGCACTATGGTATAATTATACCTTGGCATT	T/A	AGGAAAATCAGTGGAGAAACAAGGCTGGATAGGTGTGTTTATACTGAAATTGCAGCAGGCTGCAATATTGTGAAGATTTCACATGTATGATATGATCATT
Ca_LG_4:33094520	GACACTGTGATGTTCTGGGGACAACACTTTGGCACGGATGATAAAGCTGCACTATGGTATAATTATACCTTGGCATTAGGAAAATCAGTGGAGAAACAAG	G/A	GCTGGATAGGTGTGTTTATACTGAAATTGCAGCAGGCTGCAATATTGTGAAGATTTCACATGTATGATATGATCATTACAACTTAGTACACACAATGGCT
Ca_LG_4:33094526	GTGATGTTCTGGGGACAACACTTTGGCACGGATGATAAAGCTGCACTATGGTATAATTATACCTTGGCATTAGGAAAATCAGTGGAGAAACAAGGCTGGA	A/T	TAGGTGTGTTTATACTGAAATTGCAGCAGGCTGCAATATTGTGAAGATTTCACATGTATGATATGATCATTACAACTTAGTACACACAATGGCTGCATTA
Ca_LG_4:33135730	CGTAGGAGGTTTTAATTAGAATGAGTTAATGGTTCTTCATTTATGAACAAGTTGTTAGAAGCAGCTTATAGGTGTTTATGAACAAGTGAAACCAAAAAAT	T/A	TTTTAAAAAGTTGAGCTGCATCCGTGCCAAGCTAATTTTAAGTCCAACTAATACAAGATGAGATTGTTTTAAAACTCATGGCTGCTATAAGATTCTTTTA
Ca_LG_4:33414141	TTTGTTAAAGATAAGGGTTAGTGAGAAAAACATGGGTTGTGTCGCCTCTAAACTAGAGGAAGAAGAAGAAGTTGTAGCTATCTGTAGAGAGAGGAAACGC	C/T	CAGTTGAAACTAGCTGTGGAGAAAAGGTATGAACTTGCTGAAGCTCATTGCAAGTATTTTCATTCTTTGAATGCTGTAGCTGCTTCCATTAAGCTTTTTG
Ca_LG_4:33414169	AACATGGGTTGTGTCGCCTCTAAACTAGAGGAAGAAGAAGAAGTTGTAGCTATCTGTAGAGAGAGGAAACGCCAGTTGAAACTAGCTGTGGAGAAAAGGT	T/A	ATGAACTTGCTGAAGCTCATTGCAAGTATTTTCATTCTTTGAATGCTGTAGCTGCTTCCATTAAGCTTTTTGTTGCACGCCATTCTTCACCTTCTTCACC
Ca_LG_4:33416822	AGTCAAATACTTTACATGTCCTGCATATGGGAAATTTTGCAATCAATCTCGTGGGTTAGCAACTCTTCAGCTTGAAGCCGAGCTTCACCACTGGCGCGCG	G/A	TGCTTTAGAGAGTATACTGCAGCTCAAAAAGCATATGTACAAGCTCTTCACGGATGGCTAAGTAAGTTCATAGTCCCCGAAGTTGAATTCTACTCAAGAC
Ca_LG_4:33528038	CAAGACCAGACCACAAAACCATCACTGGCATAGAAATAGCAGCTTCAATTCTGACTTCTCCAGCTTTGTCTTGGAGACCCAAATCTAAAACTTCAAAAAA	A/C	AACTTCACTGCTCAAGCTTGGACCTAACTTCGATAATACTTGAAGAGAAAGGCATTTGGTTTTCCAAGGCAGCTGCTTATCAATAACAAGGAGCACATGA

Positions	-100 bp flanking sequences	SNPs within *desi* cultivar	+100 bp flanking sequences
Ca_LG_4:33561693	TTTATAACACAAAAATAAATGTCAACACAATATAAGTGGAAGACAATTTCAGTATAGTTGCATATGTAACATAAAAATGAAGCATCTTACAATCCATATG	G/A	TTGTCAACACCACCAAGAACAGCAGCAGCCAACTTGGAGCGGAAAGGGTTCCAAATTCTGTACTCAGTCTTAGTACTGTCCTCATTCTGTAATATGTATG
Ca_LG_4:33676618	ATGGAATGCATTCTATTACATTCCACTCAATCACATTCTGTTTTTAACAATCCAAACAATGGAACATAAACTTATTCCATTCTATTCCATCCCATTCCAA	A/T	CTCATACCACCAATCCATAGTCTAAGTCTTGGCTTATGCATTATTTTATTTTATTTTATTTTATTTCTGGTGTTATGTTTGTTTTGCTGCTGTACATTGT
Ca_LG_4:33782852	TGCATTTACAGAAAACCCTGAACAGATCACCTCTAAAGCTGATATTGTAGTTTCAGCTGCTGGAGTCCCTAATTTGGTTCGGGGGAACTGGATAAAGTCT	T/C	GGTGCAACTGTGATCGATGTTGGAACAAGTCCTGTTGAGGTGAGCTTGCAATAATATATATATATTTTTTCACATTTTGCTTGCTGCATATCAGTTAACC
Ca_LG_4:33843812	CTGCTGTGATGACAATTGTTCCCAAACTTGGGAAAAAGGATATATATTTAGCATACTTGCCAATGGCTCATATCCTTGAGCTAGCAGCTGAGGTATGCAA	A/G	GGGGATATATAACTTTTTGGAATAGATTCAATAAATGTTTCAGTTCAACATTGCATTCTGTTTCCATTTATTTTGTGTATTGATAAACTCGAATACATTT
Ca_LG_4:33895688	TATTCTTACCTAATCAAATTCAAGATTTAGTCCCACAAGAAAGTCATAGACCTTATCTTTTTCGGTAAGCTCCTTCAAGACTACTGAATCTACTGCACAA	A/G	TGAATCTTTATCACTCTATAGTGGTCCAATTCCATCCAAAGATACTTCAATTGCTTGGCATATTATGTTACCTTGCTTTGCATCTACTGTTTTCACCTTC
Ca_LG_4:33895692	CTTACCTAATCAAATTCAAGATTTAGTCCCACAAGAAAGTCATAGACCTTATCTTTTTCGGTAAGCTCCTTCAAGACTACTGAATCTACTGCACAATGAA	A/G	TCTTTATCACTCTATAGTGGTCCAATTCCATCCAAAGATACTTCAATTGCTTGGCATATTATGTTACCTTGCTTTGCATCTACTGTTTTCACCTTCACAT
Ca_LG_4:33895725	AGAAAGTCATAGACCTTATCTTTTTCGGTAAGCTCCTTCAAGACTACTGAATCTACTGCACAATGAATCTTTATCACTCTATAGTGGTCCAATTCCATCC	C/G	AAAGATACTTCAATTGCTTGGCATATTATGTTACCTTGCTTTGCATCTACTGTTTTCACCTTCACATCATACACTTTGGCAACATATTTAGCCTTTGAAT
Ca_LG_4:33895726	GAAAGTCATAGACCTTATCTTTTTCGGTAAGCTCCTTCAAGACTACTGAATCTACTGCACAATGAATCTTTATCACTCTATAGTGGTCCAATTCCATCCA	A/G	AAGATACTTCAATTGCTTGGCATATTATGTTACCTTGCTTTGCATCTACTGTTTTCACCTTCACATCATACACTTTGGCAACATATTTAGCCTTTGAATA
Ca_LG_4:33895731	TCATAGACCTTATCTTTTTCGGTAAGCTCCTTCAAGACTACTGAATCTACTGCACAATGAATCTTTATCACTCTATAGTGGTCCAATTCCATCCAAAGAT	T/G	ACTTCAATTGCTTGGCATATTATGTTACCTTGCTTTGCATCTACTGTTTTCACCTTCACATCATACACTTTGGCAACATATTTAGCCTTTGAATAGGTTT
Ca_LG_4:33895742	ATCTTTTTCGGTAAGCTCCTTCAAGACTACTGAATCTACTGCACAATGAATCTTTATCACTCTATAGTGGTCCAATTCCATCCAAAGATACTTCAATTGC	C/G	TTGGCATATTATGTTACCTTGCTTTGCATCTACTGTTTTCACCTTCACATCATACACTTTGGCAACATATTTAGCCTTTGAATAGGTTTGTTCCATTGCA
Ca_LG_4:34242774	GGAGGCTGATCATGGGCAGCAACTGATGAACCCAAGTGTTTGGTAACTCATTCCAGAACGAGTCCAAACTGAGTGATAGTGTTTTGAAAGTGAGAAAAAT	T/G	TAACCAACAACTTCTTTTGGTCTTTCCTAATGGTCTTTAGAAGCTTTAAAAACCTTTTGTTCTCAGTTTCTGCCGATTTCATGCCGGTGGTTGAATCATT
Ca_LG_4:34242781	GATCATGGGCAGCAACTGATGAACCCAAGTGTTTGGTAACTCATTCCAGAACGAGTCCAAACTGAGTGATAGTGTTTTGAAAGTGAGAAAAATTAACCAA	A/T	CAACTTCTTTTGGTCTTTCCTAATGGTCTTTAGAAGCTTTAAAAACCTTTTGTTCTCAGTTTCTGCCGATTTCATGCCGGTGGTTGAATCATTACGCGCT

Positions	-100 bp flanking sequences	SNPs within *desi* cultivar	+100 bp flanking sequences
Ca_LG_4:34243041	GGAGATTTGGATTTTTCTTGTAAATCCGAATCAGAATTAGATTGAGCAATATTTGGAGAGATAGGAGAAGAGTTCAGACTTACTTGAGATGGTATGTTCC	C/T	TATGAGGGCTTTTTGAAGAATGGTGATCATATGATACGTTCAGATCAAAGACTAAGTGTTCACCATCCTTAACTGAAGGAGCAGCATTATGCGTAGATGG
Ca_LG_4:34243049	GGATTTTTCTTGTAAATCCGAATCAGAATTAGATTGAGCAATATTTGGAGAGATAGGAGAAGAGTTCAGACTTACTTGAGATGGTATGTTCCTATGAGGG	G/A	CTTTTTGAAGAATGGTGATCATATGATACGTTCAGATCAAAGACTAAGTGTTCACCATCCTTAACTGAAGGAGCAGCATTATGCGTAGATGGATCCTTAC
Ca_LG_4:34243058	TTGTAAATCCGAATCAGAATTAGATTGAGCAATATTTGGAGAGATAGGAGAAGAGTTCAGACTTACTTGAGATGGTATGTTCCTATGAGGGCTTTTTGAA	A/G	GAATGGTGATCATATGATACGTTCAGATCAAAGACTAAGTGTTCACCATCCTTAACTGAAGGAGCAGCATTATGCGTAGATGGATCCTTACTGACTTGTG
Ca_LG_4:34243061	TAAATCCGAATCAGAATTAGATTGAGCAATATTTGGAGAGATAGGAGAAGAGTTCAGACTTACTTGAGATGGTATGTTCCTATGAGGGCTTTTTGAAGAA	A/G	TGGTGATCATATGATACGTTCAGATCAAAGACTAAGTGTTCACCATCCTTAACTGAAGGAGCAGCATTATGCGTAGATGGATCCTTACTGACTTGTGGAG
Ca_LG_4:34243730	GATATCTTGAAGAACTGAGGTTCGAGTGACCATATACTGGATATACCAAGTTTCAGCAAAACAGTGGGCTCCAGCATCTTGTATATCAAGAATTTTGCAT	T/C	AGTGAAGTGGGGTTTACGTCCAAGTGTACACCCTTAAGTACAAAACTGAACCCAAGGCTTCCTTTACAGCCATTGCATGCAGCATAAAATGCCTTTACCA
Ca_LG_4:34243736	TTGAAGAACTGAGGTTCGAGTGACCATATACTGGATATACCAAGTTTCAGCAAAACAGTGGGCTCCAGCATCTTGTATATCAAGAATTTTGCATAGTGAA	A/T	GTGGGGTTTACGTCCAAGTGTACACCCTTAAGTACAAAACTGAACCCAAGGCTTCCTTTACAGCCATTGCATGCAGCATAAAATGCCTTTACCAGTCTTG
Ca_LG_4:34243745	TGAGGTTCGAGTGACCATATACTGGATATACCAAGTTTCAGCAAAACAGTGGGCTCCAGCATCTTGTATATCAAGAATTTTGCATAGTGAAGTGGGGTTT	T/A	ACGTCCAAGTGTACACCCTTAAGTACAAAACTGAACCCAAGGCTTCCTTTACAGCCATTGCATGCAGCATAAAATGCCTTTACCAGTCTTGGGTATTGCG
Ca_LG_4:34243769	GATATACCAAGTTTCAGCAAAACAGTGGGCTCCAGCATCTTGTATATCAAGAATTTTGCATAGTGAAGTGGGGTTTACGTCCAAGTGTACACCCTTAAGT	T/C	ACAAAACTGAACCCAAGGCTTCCTTTACAGCCATTGCATGCAGCATAAAATGCCTTTACCAGTCTTGGGTATTGCGGTTCATTCACTCTAAAAAAGGAAG
Ca_LG_4:34243785	GCAAAACAGTGGGCTCCAGCATCTTGTATATCAAGAATTTTGCATAGTGAAGTGGGGTTTACGTCCAAGTGTACACCCTTAAGTACAAAACTGAACCCAA	A/G	GGCTTCCTTTACAGCCATTGCATGCAGCATAAAATGCCTTTACCAGTCTTGGGTATTGCGGTTCATTCACTCTAAAAAAGGAAGTCCATCCTAATTTATC
Ca_LG_4:34243790	ACAGTGGGCTCCAGCATCTTGTATATCAAGAATTTTGCATAGTGAAGTGGGGTTTACGTCCAAGTGTACACCCTTAAGTACAAAACTGAACCCAAGGCTT	T/G	CCTTTACAGCCATTGCATGCAGCATAAAATGCCTTTACCAGTCTTGGGTATTGCGGTTCATTCACTCTAAAAAAGGAAGTCCATCCTAATTTATCAAATA
Ca_LG_4:34430500	CTGTTTTTTCGATTTCGAAGCTGTTTCTATGGCGGATGCTCTAACGGTGATTCCGGCTGCCGTGCTCCGTAATCTCGCCGATAAGCTTTATGAGAAGCGC	C/A	AAAAATGCTGCTCTCGAGGTTTTCTTCTCTCTCTCATTGTGATTTTTTGTTAGTTATTGCAATTTGGTTGAATTTGAAGGATCTTTTGTGGAAATAGGTT
Ca_LG_4:34438641	TGAATTAGTGGTTTGATGTTTACATCTTGACTCTTAATTTTATACCACCATGCAGCAAAGTGCAGCCTTTAAGATACTAAAAACTCGTTTAAAAGCTGTG	G/A	CCGTCATATTCTTTCAATCGTGGACAACTGAATAGAGTGCCTTCTGGGGATCCCAACCAATTTCCTCATCAGATGCCTGATGGATATCAAACCAATGAGG

Positions	-100 bp flanking sequences	SNPs within *desi* cultivar	+100 bp flanking sequences
Ca_LG_4:34438809	TCAGATGCCTGATGGATATCAAACCAATGAGGATGGTGATGTAACTGAAGATGATGGGAGCCCATATAATGGAATAAACTTTGCTGCGAGGTTGCAACAA	A/G	TTTCAGCAAATGCAGAAGCAGCACCGGGTACACACTAAATCACGAACAAAGTCACGCAGTGTGTCCATTTCATTACCAAAGGTTGTTGGCCTCTTCATTC
Ca_LG_4:34596612	TCCTTGAAACCAAGTTCCCACAGCAACAGCCCAATCTGCATAATTGGCAAAACCAACCAATTTTTCGCAAGAAATGAGTGGAAATGTATTGAAACTGAGC	C/T	GCAAGAAAAAGCAGCAACAGTTACAGTGTCGGGGGAAGTTTGTGGAAAAATCACCAAAAGATGGTAAACACCTCGGAATGTGGCATAGGGGTTTCTGATG
Ca_LG_4:34596851	GTACGGGTTTTGTGGTGGCGTGTGGGGCCCACATGCAGAGAAGGTGGAGGCACGTGTGGTGGTAAAGCATAGTCGTGTTTGTCAGAGGATTGTGCAGCGA	A/G	TCTGTGGTGGTTTTAGCAGTGGGGGGCACAGGAAAAGGTGCAGCTGGTGTTGAGGAAAAAACGTTGATTATGGCCCTGAAAAATGCAGAGAAGACAATGG
Ca_LG_4:34906257	ACTTTCCACGATATGTGAAAAGAGAAAATTCTGAAAGTGATGTTATCGTTGGTGTAATCAATTCTGGATTAACAAAGCATGTATATATATTATCAGTCTA	A/C	ACTTACATCAGTGTCAAATGATACTATATACTTTAAAAATGTAATTTCCATTGGATCATTACATGCTATGCAACATGGAGTGTTGACAATATTGGTAGCT
Ca_LG_4:34906346	TTATCAGTCTAACTTACATCAGTGTCAAATGATACTATATACTTTAAAAATGTAATTTCCATTGGATCATTACATGCTATGCAACATGGAGTGTTGACAA	A/G	TATTGGTAGCTGGAAACACGGGTCCTTGTCCTTCAACCTTAGACAATTTTTCACCATGGGCAATTGTTGTGGCGGCTAGCAGATTAGATAGAATTTTTCT
Ca_LG_4:34906353	TCTAACTTACATCAGTGTCAAATGATACTATATACTTTAAAAATGTAATTTCCATTGGATCATTACATGCTATGCAACATGGAGTGTTGACAATATTGGT	T/C	AGCTGGAAACACGGGTCCTTGTCCTTCAACCTTAGACAATTTTTCACCATGGGCAATTGTTGTGGCGGCTAGCAGATTAGATAGAATTTTTCTCTCTGCC
Ca_LG_4:34906364	TCAGTGTCAAATGATACTATATACTTTAAAAATGTAATTTCCATTGGATCATTACATGCTATGCAACATGGAGTGTTGACAATATTGGTAGCTGGAAACA	A/T	CGGGTCCTTGTCCTTCAACCTTAGACAATTTTTCACCATGGGCAATTGTTGTGGCGGCTAGCAGATTAGATAGAATTTTTCTCTCTGCCTCTCATTTTTA
Ca_LG_4:34906373	AATGATACTATATACTTTAAAAATGTAATTTCCATTGGATCATTACATGCTATGCAACATGGAGTGTTGACAATATTGGTAGCTGGAAACACGGGTCCTT	T/A	GTCCTTCAACCTTAGACAATTTTTCACCATGGGCAATTGTTGTGGCGGCTAGCAGATTAGATAGAATTTTTCTCTCTGCCTCTCATTTTTAGGTTTGTTA
Ca_LG_4:34906389	TTAAAAATGTAATTTCCATTGGATCATTACATGCTATGCAACATGGAGTGTTGACAATATTGGTAGCTGGAAACACGGGTCCTTGTCCTTCAACCTTAGA	A/G	CAATTTTTCACCATGGGCAATTGTTGTGGCGGCTAGCAGATTAGATAGAATTTTTCTCTCTGCCTCTCATTTTTAGGTTTGTTATTTTTAATTAAACATT
Ca_LG_4:35013400	GACGACCAACCGAACAATGCCCCCTCTGCCCCGTTAACTATGGACTTACTTGCTGTTCTTTCGACCTCTCCATCCGCACCTGCTCAAAATGGCGGCAACG	G/A	CATCCATGACTAGTGCGGACCAGATGAGAGAACAGCAATTCACTTCAGTCGTTGGAGAAAGAAGCAGCGGCAGTTCTCAGTCTCCAAATGATGATTCAGA
Ca_LG_4:35108781	TTCCAGGTTGATTCCTTCTTTTTTTCTGAGGCAAAACAGTTGATGAAGAAGCAGCTGTGGAAGATTGATTTTGTGTAATCTGACTTTGATGAATTTCTTG	G/T	TCTGATTTCCAAGAAAGGTGTAGATGAAGAAGAAGCAGCCATATTTTTTTTTTTTTGCAAATCTGAAAAATTCTTAAATAAATTTGTTACAATCTATAATA
Ca_LG_4:35142837	AGGCAGCAGACGGCAGCGACAGATCTACTCGCATGAGAGATTAGCAGCACATGAGAGCGCGTGTGACGGCTTTTGGCGACGCGCTTTCAGGCATAACCTG	G/A	ATCTGGAAATGACTGATATTCGCAGGAAAATTTGCCGAAAATAGTGGCAGCGGCGGCAATAGCGAATTGAATCAAAAACGAGTGAAACGTGTGGAAATAT

Positions	-100 bp flanking sequences	SNPs within *desi* cultivar	+100 bp flanking sequences
Ca_LG_4:35329973	GAGTCTACAATCCATGAGTTAGATATTCCCTTGTCAACAAAAT AGACACATGAAGAATTATCTTTTTGGGCCAAGGCAGCAGTTG AGTGTGTACTAGTGT	T/C	TTTGAAGGGATTGTTGTAGCAATTTTTGCAACACCTCCATTT GTTCCTTGCTGAATGGACTTGGTTCAGGTGTTGGGGCTGCC AACACTTTACCTCTTTC
Ca_LG_4:35496143	ATTTTGGTTGTTTCGTCTTTTCCAAATGAGTAAATTAGTAGACG TTTTAAATTAGGCTCGCTTCGGGCAGCAGACGGTAAAGAGGG AAAATGGTTAATTT	T/C	GCGGCAGTAGCAGGAGTAAACGTTTCAGGCAAAGAGTTGTT GGTTTTACATTGCAGCTTATTAGAGGTCTGTGGTCATTGTTA GTAGTTGTTTGCAGGTT
Ca_LG_4:35496689	TATGATGGAATGAGAGTTTACTGTTTACCTTCATATGTTATCTG GTGAGATAGATTGCAGAACTTCAATTCAGAGGATAATGTATTT GGGCAGCTGCAAT	T/G	GGAAAATTTGGGAAAATCAGGTTATACTTATAGACACATTCA TGTACATAAATTTAGGAAAATCAGTTTCATTTTGAAAGAATAT GCAGAAAGTTTATCT
Ca_LG_4:35510109	GTAAGTGATTGATGCACAATATATGTAAGTGTTATAAATTTAAG GGTATTAAATTATATCTATACCAATAAAAAAACTAGATAGTAAT CGAATTCCACTG	G/A	CACGAAATGAGATATATAACAATTTTTTCGCAGCGACAATCA ATTTGCTAGAAACAACATGCATATGACCTCGACTTATTTAAGT CATTGTAATAAGATT
Ca_LG_4:35554744	GTTTGAAACATCATATCAATTCCTAACTCACATAAACGACAATT TTTTTAAGTGTGGTTTAGTTCTGTTGGGTTTTTCTGTTTTTTTTT CTTTTCTTAAC	C/G	TGTTCTATGCAGCACAAATCAAATATATTATATTTGAATCCTA CCCTATTTTGGTGAACAGGCTGTAAGGCCCATGACCCTTCT GCAAATCTGTCAAGCG
Ca_LG_4:35835436	ATTTAAACAAGATTCCAACTTGATTAAAGTGATAAATATTTTAT CACATTGATAAATTTTCCACCATATATTCAATTCTATTCTAAAT GATGCTTGTTCC	C/T	AATTTAAAAGATTTGTATTAAATTTCAATTGATTGAGTAAACA TCACGACATCGGTTGGAGCGGTGGGCGGCGGCTAGTCAGA GGCGGTAGTGTGAGAAC
Ca_LG_4:35835439	TAAACAAGATTCCAACTTGATTAAAGTGATAAATATTTTATCAC ATTGATAAATTTTCCACCATATATTCAATTCTATTCTAAATGAT GCTTGTTCCAAT	T/G	TTAAAAGATTTGTATTAAATTTCAATTGATTGAGTAAACATCA CGACATCGGTTGGAGCGGTGGGCGGCGGCTAGTCAGAGGC GGTAGTGTGAGAACGAC
Ca_LG_4:35835466	GATAAATATTTTATCACATTGATAAATTTTCCACCATATATTCA ATTCTATTCTAAATGATGCTTGTTCCAATTTAAAAGATTTGTAT TAAATTTCAATT	T/G	GATTGAGTAAACATCACGACATCGGTTGGAGCGGTGGGCG GCGGCTAGTCAGAGGCGGTAGTGTGAGAACGACGGCATCG ACGGTGTCGCAACTAAGAGA
Ca_LG_4:35835468	TAAATATTTTATCACATTGATAAATTTTCCACCATATATTCAATT CTATTCTAAATGATGCTTGTTCCAATTTAAAAGATTTGTATTAA ATTTCAATTGA	A/T	TTGAGTAAACATCACGACATCGGTTGGAGCGGTGGGCGGC GGCTAGTCAGAGGCGGTAGTGTGAGAACGACGGCATCGAC GGTGTCGCAACTAAGAGACG
Ca_LG_4:36157742	TAGGAATAAAGAGCAATTTGCTGAGCATTGGGCAGCTGATTC AAAAGGGGTTTCAGGTGATCATGAAGAACGATGCCTTAGAAA TGTATGATGGGCAGAA	A/G	GAAGATGATACTCAAGGCTCCTCTTTCAAAGAACATAACCTT CATCATCAACATACAAGCTGCTGATATTCAATGTTCGAACGC AACAAGTTCAATTGAT
Ca_LG_4:36157778	TGATTCAAAAGGGGTTTCAGGTGATCATGAAGAACGATGCCT TAGAAATGTATGATGGGCAGAAGAAGATGATACTCAAGGCTC CTCTTTCAAAGAACAT	T/G	AACCTTCATCATCAACATACAAGCTGCTGATATTCAATGTTC GAACGCAACAAGTTCAATTGATGAAAACTGGTTGTGGCATTC AAGATTTGATCATCTT
Ca_LG_4:36247317	CTTATTGGATGCAACTGGCTCCTTCTCACTGGTATTATTTGTT GTATTTTCACTGACTTTAATTAGTTTTTGGATTTGGTATTGTTG TTTTTTGTCGATC	C/T	GGTCGAATCTGCAGCAGATAGCAGGATTTCAGATACATATTG TCTTATGTAAATATACAGTGTTGACAGGATAAAACGGACCTT CTCTAAAAATAATCTT

Positions	-100 bp flanking sequences	SNPs within *desi* cultivar	+100 bp flanking sequences
Ca_LG_4:36790362	CCTGAGGACTACGACGGCGTGCCAAATGAGGAGAAGTTAAATGGTTAAGCCAGAGGATACAAGGGATGCAGCCTGTTCCGTCGTCGATTGCGAATCGGAT	T/G	GAATTTTCCAGGTTTGTGATCGCGTAGGGTTATGGTTCCTAGGGTTTCGGCGCGTGTGATTGGGATTGATTTGCGAGTGAAAGGGTGGTCGGGGGATGAA
Ca_LG_4:36807377	ATTGTAACGACAGAAGTCTTCTAACCCTCTTGACTCTGCAGCACGGATCTCTCTTCCCCTCTTTTCAAGATCCCGTTGAGTCTTGGCGGCAGTCTGTGCG	G/A	GCGACAGAAGCAGCCATGTTATTCATAGCCTCTGCCATTTGATCATTCCTATTAGCGTTGGCTCTCGAAGCGTCATTTCTTCTTGGCGGCATGGTTTCCT
Ca_LG_4:36807380	GTAACGACAGAAGTCTTCTAACCCTCTTGACTCTGCAGCACGGATCTCTCTTCCCCTCTTTTCAAGATCCCGTTGAGTCTTGGCGGCAGTCTGTGCGGCG	G/C	ACAGAAGCAGCCATGTTATTCATAGCCTCTGCCATTTGATCATTCCTATTAGCGTTGGCTCTCGAAGCGTCATTTCTTCTTGGCGGCATGGTTTCCTATA
Ca_LG_4:36822272	ACATGGGAGATGATCAAAAAAATATGAAGAAAAATAAAAGAAACATACTGAAATTGGAGCAGCACCACATTTCCACTTGTTTACAGGATTCTTCAAGTTG	G/A	GTTACAGTTGCCATATATCCATTCAAACCAGCAGCCAGTATATGGTAGCAGATGTGTCCAAGAACCTGCAATAATATCAACAAGTTATAACAAGTGAAAA
Ca_LG_4:36822287	AAAAAAATATGAAGAAAAATAAAAGAAACATACTGAAATTGGAGCAGCACCACATTTCCACTTGTTTACAGGATTCTTCAAGTTGGTTACAGTTGCCATA	A/G	TATCCATTCAAACCAGCAGCCAGTATATGGTAGCAGATGTGTCCAAGAACCTGCAATAATATCAACAAGTTATAACAAGTGAAAAATCGAACACTGATGC
Ca_LG_4:36824657	TTTCTGCAAGCTGTGCGGCATCTGTGTTAGATGTCACACCTGAAATAAAATACCGTTTCAACATTAAGAAACACCTTTTGAAGATGTGAATATAACCATA	A/C	CATGAAGATTCAGGAAGAAGTATACCTCCAATTATGACAAGTCCATCAAGTTTCAAATCATTGCATGCGGCGAGTGCAGCATTAACTTGTTCTGTGGTTC
Ca_LG_4:36869088	CAACTCCCTACCAATTTTCTTAACATGCATAAAATTACAATAACCGCCACGGTTACAACTATTCTCCTCATACTGACGACACGTGGCTTCACGAAAATCG	G/T	GTCACCGGCGAAAAATCAGCTAAAATCGGACGACCGGAGTAAAACCTCCCGTGCAGCGAAGCCAAAGCAGCCGCAGCGTGATCCTCCTCACGAAACAGAA
Ca_LG_4:37080038	TATGTGTCAAGCAAAAAAATATGGAGCATTAATCTTCTAAGTAACCAATTCAAATACTCCAAATATGGCTACATAGATGCAGCCTATGAAAGCAAGTTCG	G/A	GCTAACATGATGTGTGAATCAAAAATGATGTTAGCCAAATTGTTCGGCTAAGCAAGTTCGTCTAACTACAAAGATGCAGCCTATGAAAGCCAAATTGTGT
Ca_LG_4:37080073	TCTAAGTAACCAATTCAAATACTCCAAATATGGCTACATAGATGCAGCCTATGAAAGCAAGTTCGGCTAACATGATGTGTGAATCAAAAATGATGTTAGC	C/T	CAAATTGTTCGGCTAAGCAAGTTCGTCTAACTACAAAGATGCAGCCTATGAAAGCCAAATTGTGTTCCATTTCCATATCATTATCAAGAACAGTACCACG
Ca_LG_4:37134986	TCCAAAGTAAAACAGAGCTGAAAAACATTATGCAGAAATTCTAGACTTACAGCAGCAGCAGCAGCAACAAGTAGCTTATATACTCTAAGCTGTTTTTCAT	T/C	ATTGATGCAATCTTTTACTAGTTCGTTAAAAATATAAATGAAACAATTGATTCATAACCTATGAAAAAACCCTTATATTATTAGATGAGCACAGGGCAGC
Ca_LG_4:37323433	TCCTAGAAATTCTTATAAGATTGATGAACAACACCCTGGATCTGATCTTGCTGCTGAAACTTCAGCTGCTTTGGCTGCTGCTTCCATTGTTTTCAATTCC	C/T	GTGAATCCCAAATATGCATCCAAATTGTTAACTCACGCTACACAAGTATGATTTATTTTTCTTTTTTTTTTGTCATACCTCTATTATATGCAAATTATTT
Ca_LG_4:37323682	CTTAAAGAAATGCATCTAAATAGAAAATAGAAAGTTTTTTTTTGTTAGGTGAAAATTTTTTATAATATTTTTGTTGCAGCTGTTTGACTTTGCAAACAAC	C/T	TATCAAGGCTTATACCATAATAGTATCCCACCGGCAGCGAAAATATATTCCAGCAGTGGATATAAGGTAATCTACTTAATTGTATTTGAATTTACTAAAC

Positions	-100 bp flanking sequences	SNPs within *desi* cultivar	+100 bp flanking sequences
Ca_LG_4:37323692	TGCATCTAAATAGAAAATAGAAAGTTTTTTTTTGTTAGGTGAAAATTTTTTATAATATTTTTGTTGCAGCTGTTTGACTTTGCAAACAACTATCAAGGCT	T/A	TATACCATAATAGTATCCCACCGGCAGCGAAAATATATTCCAGCAGTGGATATAAGGTAATCTACTTAATTGTATTTGAATTTACTAAACAAAGAAAGTG
Ca_LG_4:37323714	AGTTTTTTTTTGTTAGGTGAAAATTTTTTATAATATTTTTGTTGCAGCTGTTTGACTTTGCAAACAACTATCAAGGCTTATACCATAATAGTATCCCACC	C/T	GGCAGCGAAAATATATTCCAGCAGTGGATATAAGGTAATCTACTTAATTGTATTTGAATTTACTAAACAAAGAAAGTGGAAAATAGAAAACATTTTGTGT
Ca_LG_4:37323814	GGCAGCGAAAATATATTCCAGCAGTGGATATAAGGTAATCTACTTAATTGTATTTGAATTTACTAAACAAAGAAAGTGGAAAATAGAAAACATTTTGTGT	T/C	AAGAATATTGAAATTATTTGTACAATTACAGGACGAGTTACTATGGGCAGCAGCATGGCTTTACCGTGCAACGAACATGAATAAATACCTTGATTACCTT
Ca_LG_4:37535970	ACACAAACCTTTTCACCCTCCTTCTCCTTTCATTCTCGGAAAACGGTTACGTTCTGAACAAGATGACGCCGTTTCCGTTGCTGCACCTACCGCTCCACCT	T/G	GCTCTTTGGGCGCTTCCGTCAAGGCCTGACTTCGCTCAACTTTGGAGCTTCGCCGCCGCCGCAGCACAACCTCCGTCGGAAGTTCTTTCCATTTCTCCGC
Ca_LG_4:37864970	CCCCAAGGAAGAAACGACATGACTGAAGCAATCCAAGCCATGAATACAATGGCCGCAGCAATGGCCCAACAAGCTGCGATCCAGGCTCAACGAGATGCAT	T/C	AGAGGGATAAGAGGGATGGAGCAGCCAGTGCAGCAAGAGCCTTGAATGAATTTTGTCAACAAGATTTGCCTAAATTCAAAGGGGAACATGACCCGACAAG
Ca_LG_4:38023251	GCTCTTCTTCGTCACACGTGCCTACTCTCACCCCTGATTCAAATTCAACTCCTGCCGCACCAAAACTTGTCATGCTCTCTCCCACCCCTGGTTCAGAGTC	C/G	TAGGCCAGCAGCTTCTGCACCTAATTCTGTCCCCATCAGAGTTCCTCCTCAGAATACACATTCCATGCAAACAAGAGCCAAGAATGGCATTATGAAGCCA
Ca_LG_4:38023378	TGTCCCCATCAGAGTTCCTCCTCAGAATACACATTCCATGCAAACAAGAGCCAAGAATGGCATTATGAAGCCAAAACTGCATCCCACTCTGCTACTTACA	A/G	CACCTAAAACCTACCACTGTCCAACAGGAACTTGCTGCCCCATAGTGGTTGGCAGTAATGAAAACTGAGTATGCTGCTCTTCTTCATAACAATACTTGGT
Ca_LG_4:38127899	ATAACAAGGCCACAAAAGCAAAACTACTGCTGCGATTAGACTGACATACCTAGAGAGATTTTACGAGATTCGTAAATTAATCATTCAACTTTTGAACACC	C/T	TTTTTGACACACTTACAAAACTTATAATAGACATTAACTGCAGCCTTTATGTTTCTCCAATTCAAATTCGGAATTCCATATATTGTGTGGTCAAACAGCA
Ca_LG_4:38127903	CAAGGCCACAAAAGCAAAACTACTGCTGCGATTAGACTGACATACCTAGAGAGATTTTACGAGATTCGTAAATTAATCATTCAACTTTTGAACACCTTTT	T/G	TGACACACTTACAAAACTTATAATAGACATTAACTGCAGCCTTTATGTTTCTCCAATTCAAATTCGGAATTCCATATATTGTGTGGTCAAACAGCAATTA
Ca_LG_4:38169648	GATGAATCACACTTGATTTCGAAGGGGGTGGTCCACTTGGATGGCTGAATGATGGGAGTGGAGGTCACTGTTGCCTTAAAGCAATTAAACGCCTTCTTGC	C/A	ATTTGTCATCAAAGGTTAAAGTGACATCTTTTTGCAGCAAATTTGACAGTGGAAAAGTTATCTTGTTGAAGTCTTTGATAAAGCGCCTGTAAAAACTTGC
Ca_LG_4:38169653	ATCACACTTGATTTCGAAGGGGGTGGTCCACTTGGATGGCTGAATGATGGGAGTGGAGGTCACTGTTGCCTTAAAGCAATTAAACGCCTTCTTGCATTTG	G/A	TCATCAAAGGTTAAAGTGACATCTTTTTGCAGCAAATTTGACAGTGGAAAAGTTATCTTGTTGAAGTCTTTGATAAAGCGCCTGTAAAAACTTGCTTGGC
Ca_LG_4:39255326	AACACATGGATGACAGGATGAAAGAAAGAACGCATGAAATGATGCTCCACATATCAGATATAGAACATGAAAATGGAAAGCTGTCAATGCGTGTTTCTGT	T/C	TTTAGAAGATGAAGTGAGAGATTTGACAAACGAACAAGAGTCCCAATTATCAGAGCTAGAGAATGATAGAAATCAAGCTGCAAGGCTCCGAGAGAAGATT

Positions	-100 bp flanking sequences	SNPs within *desi* cultivar	+100 bp flanking sequences
Ca_LG_4:39255573	AAAACTGAAGACGACACAATTTCATTGGTCAGAAGCACAAGAAGAATCTGAATAATCTTAGAGGAGAAAATCAGCAGCTACAAATTACCATTGAAAATCC	C/T	TGAAGACGAGTGCAATTCATTTGAGAATCTAAATGGATATTTGAGGCAGCAGAAGTTGGAGTTAGAGGACAATTGCTCCCTTATGGGAGCCAGGTTGAGG
Ca_LG_4:39255601	TCAGAAGCACAAGAAGAATCTGAATAATCTTAGAGGAGAAAATCAGCAGCTACAAATTACCATTGAAAATCCTGAAGACGAGTGCAATTCATTTGAGAAT	T/G	CTAAATGGATATTTGAGGCAGCAGAAGTTGGAGTTAGAGGACAATTGCTCCCTTATGGGAGCCAGGTTGAGGGAATCAAGTGAAAGGTTTGCTGATTGCT
Ca_LG_4:39255616	GAATCTGAATAATCTTAGAGGAGAAAATCAGCAGCTACAAATTACCATTGAAAATCCTGAAGACGAGTGCAATTCATTTGAGAATCTAAATGGATATTTG	G/A	AGGCAGCAGAAGTTGGAGTTAGAGGACAATTGCTCCCTTATGGGAGCCAGGTTGAGGGAATCAAGTGAAAGGTTTGCTGATTGCTGTGGAAGAGTGGGAT
Ca_LG_4:39940251	AATTTGTGTGTGAACTTTTGTTTTACCAAATTGGTCAAGATACCTTAAGAATTCAAGGACATGAGCTCCACTACAACGTGAGAGTGAGAGTGGTGGTCTA	A/G	TGGTTTCTTAGGTATTGTCCAAAAGTGTTCCAATCTCTTCTCTTTTGATTCTCATATCTACTTGGTGTTGTTGATGTTGAAGGTGGTGATGTTGTTGATG
Ca_LG_4:39940272	TTTACCAAATTGGTCAAGATACCTTAAGAATTCAAGGACATGAGCTCCACTACAACGTGAGAGTGAGAGTGGTGGTCTATGGTTTCTTAGGTATTGTCCA	A/G	AAAGTGTTCCAATCTCTTCTCTTTTGATTCTCATATCTACTTGGTGTTGTTGATGTTGAAGGTGGTGATGTTGTTGATGATGTAGTAGATGATGATGATA
Ca_LG_4:39940275	ACCAAATTGGTCAAGATACCTTAAGAATTCAAGGACATGAGCTCCACTACAACGTGAGAGTGAGAGTGGTGGTCTATGGTTTCTTAGGTATTGTCCAAAA	A/C	GTGTTCCAATCTCTTCTCTTTTGATTCTCATATCTACTTGGTGTTGTTGATGTTGAAGGTGGTGATGTTGTTGATGATGTAGTAGATGATGATGATATGG
Ca_LG_4:39940284	GTCAAGATACCTTAAGAATTCAAGGACATGAGCTCCACTACAACGTGAGAGTGAGAGTGGTGGTCTATGGTTTCTTAGGTATTGTCCAAAAGTGTTCCAA	A/G	TCTCTTCTCTTTTGATTCTCATATCTACTTGGTGTTGTTGATGTTGAAGGTGGTGATGTTGTTGATGATGTAGTAGATGATGATGATATGGTCATCATGG
Ca_LG_4:39940293	CCTTAAGAATTCAAGGACATGAGCTCCACTACAACGTGAGAGTGAGAGTGGTGGTCTATGGTTTCTTAGGTATTGTCCAAAAGTGTTCCAATCTCTTCTC	C/T	TTTTGATTCTCATATCTACTTGGTGTTGTTGATGTTGAAGGTGGTGATGTTGTTGATGATGTAGTAGATGATGATGATATGGTCATCATGGATGTTGATG
Ca_LG_4:39941694	AGAATGGAAGATGATGATCAATAATCATGCACAGTATTAGCAAGTTGTCAAATGCCGTGTATAGTTTTAGTGTAATATTTATGTATAGTTTTTGTCTTCT	T/A	ATATGTATCTTTTAATTAAATAATAAGAGTAGTTAATTTGCGATTAAAAATTAGACCAACTTTCTTAAATTCATATAATTTTGGTTTTTGTATGTTATTT
Ca_LG_4:39941711	TCAATAATCATGCACAGTATTAGCAAGTTGTCAAATGCCGTGTATAGTTTTAGTGTAATATTTATGTATAGTTTTTGTCTTCTATATGTATCTTTTAATT	T/C	AAATAATAAGAGTAGTTAATTTGCGATTAAAAATTAGACCAACTTTCTTAAATTCATATAATTTTGGTTTTTGTATGTTATTTATTTAATTATTTATATA
Ca_LG_4:40180408	CAGCCTTTTGTGGCTGCAAAAGCCCTCACAAATGCCAACGTGTTTTACAAACTTTGTGGCTGCAAAAGCCCTCACAAAAGTTTGTAACCAGCTTATTTGT	T/C	GGCTGCCTTAGGCCGCCACAAATAACCCCTATGTGGCAGCTATTTGCCCCTTTGTGAGGGTTTTTGGCCCTCAAAATGCCTTGTTTTCTTGTTGTGGATTA
Ca_LG_4:41056739	GTGTGTACTTTGTCTTCTGTTCTTGGTGGTAATCTGTATTGAATTTCATTTGCAGCCATTGTTCAGGTTTTACTAGATTATGACGCAAGCTTAAGCAAAA	A/G	CCATTGGTCCATCCAATGCCACTCCACTTATAACTGCAGCGACGAGAGGGCACGTCGAAGTGGTAAACGAACTGCTGTCAAAGGATGGAAGCTTGTTGGA

**Positions**	**-100 bp flanking sequences**	**SNPs within *desi* cultivar**	**+100 bp flanking sequences**
Ca_LG_4:42064694	CCTCACAAATGTTTAAAAATTTAGGTAGATTTTGTGGCTGCATAAGCCCTCACAAAATCACAACAAAATCACAACGTTGTGATTTTGTAAGGGCTTAGGA	A/C	AGCCACAAAATCCACCTAAATTTTTAAGATTTTTGTGAGGGCTTTTACAGCCACAAAATCTAAATTTTGTGAGGGTAAAGGCTGCCATAAATGCCTGAAG
Ca_LG_4:42064902	CACTTTGTGGTTGCAAATGCCGCCACAAAAGCCTGAGCCTTTGCATTTTGTTGCTGCAAAGGCCGCCACAAAAGAATAAAATTACGTGGCATTTTTTGGT	T/C	CGCCCAGGCCGCCACAAAGTTTTATACCTGTTACATTTTGTGGCTGCTTTGACCGCCACAAAGGATCAACCAAATCCTATATATATTACTCCACTCCTCT
Ca_LG_4:42065641	TTTGTGGCAGCCTATGCCCTCACAAAGGCAGCCTTTTGTGGCTGCAAAGACCCTCACAAATGGCAACGTGTTTTACAAACTTTGTGGTTGCAAAAGCCCT	T/C	CACAAAAGTTTGTGACCAGCTTATTTGTGGCTGCCTAATGCCGCCACAAATAGCCCATTTGTGGCTGCTATTTGCCCTTTGTGAGGGCTTTTGGCCCTCA
Ca_LG_4:42080626	ACCTCACAATTATGTAAGTTAATAGATCATATATTAATTCTCTTTCTCTAAAAATATAGAAAAAAGGGGTTTGTGCACCAATATCTGCCATAAAAGGTCT	T/C	CAGCCATAGTAGATAAGAAGCTATATGACTTAACTCCAACATAATTATTCGTATATAGCTGGCTGTTTTAGGTACTTGAATATTTCCCAATTGTTCTGGT
Ca_LG_4:42384000	TCTTTTGGACCTGAAAAGATCCCATCTATGTTGCATTTCAGTTTTCCTGTTGGTGGCTTGGTCCATAATTGACTGGCAGCACCAGCTTGCACTGCTGTTC	C/T	CAGAAATTCTCCTCCTGCTGGTGACTTCCTGCTGCGTGTCTAGCTCCATATTCCAATCTGCCCAGTCCACCCAAGCCCAATGAAGAATTTGGATAGGGTC
Ca_LG_4:43193173	ACTTGGCTATTACCTGAATGATGACTTTCCACTGTCTCCAAATGCTATAAGAAATTAAAATATGTCTATGGTCAATTAATTTGTTCTTTGTTAATGTCTA	A/G	TAAAAGATAAAACTAGTTTATAATTTACCATGTGATTGGAGCATGTGACATTAGCACCACTATTGTGTTTTCTTTGTCCCACCTTTAATTTTTTCTGCAT
Ca_LG_4:43193220	TAAGAAATTAAAATATGTCTATGGTCAATTAATTTGTTCTTTGTTAATGTCTATAAAAGATAAAACTAGTTTATAATTTACCATGTGATTGGAGCATGTG	G/T	ACATTAGCACCACTATTGTGTTTTCTTTGTCCCACCTTTAATTTTTTCTGCATATAAAACAACATGTCAGTATTCATCCGAGTACGTATAAATGATATAT
Ca_LG_4:43193229	AAAATATGTCTATGGTCAATTAATTTGTTCTTTGTTAATGTCTATAAAAGATAAAACTAGTTTATAATTTACCATGTGATTGGAGCATGTGACATTAGCA	A/T	CCACTATTGTGTTTTCTTTGTCCCACCTTTAATTTTTTCTGCATATAAAACAACATGTCAGTATTCATCCGAGTACGTATAAATGATATATTGTGCTTGA
Ca_LG_4:43215941	TATGTATATAGATATAAATAAAATAATACCAGGAGTTAGCTCTAATACAAGGAGTATCGCATAGGAGCCGCATTCCAATTTCAGCAAAATTAGGATTTGC	C/T	AATTCCATTAGCCGCATTCCAAATTCAATTGCAGCCGCATTCCAAATTCACTTCCAATTTCATCAGATTCCCATTTGGATCCAATTTCCAATTCCAATCA
Ca_LG_4:43215945	TATATAGATATAAATAAAATAATACCAGGAGTTAGCTCTAATACAAGGAGTATCGCATAGGAGCCGCATTCCAATTTCAGCAAAATTAGGATTTGCAATT	T/C	CCATTAGCCGCATTCCAAATTCAATTGCAGCCGCATTCCAAATTCACTTCCAATTTCATCAGATTCCCATTTGGATCCAATTTCCAATTCCAATCACAAT
Ca_LG_4:43215955	TAAATAAAATAATACCAGGAGTTAGCTCTAATACAAGGAGTATCGCATAGGAGCCGCATTCCAATTTCAGCAAAATTAGGATTTGCAATTCCATTAGCCG	G/A	CATTCCAAATTCAATTGCAGCCGCATTCCAAATTCACTTCCAATTTCATCAGATTCCCATTTGGATCCAATTTCCAATTCCAATCACAATTCCAATTCCA
Ca_LG_4:43467673	AATGACGCTCCAAGAGTCAATGTCAATAGGGACGACCAAATGGCGGAAGCTATGAATAATATGGCTGCTTCTTTTGCTGCACGGACTGCTGCAAAGACTT	T/C	TGCGGGATCTAGAAAAGAGGGAAAGAGAGATCCGTGCTGATGAGTCAAGAGGATTGGAAGATTTTCGCCGTTATAATCCTCTCAAGTTTAAGAGTGATGA

Positions	-100 bp flanking sequences	SNPs within *desi* cultivar	+100 bp flanking sequences
Ca_LG_4:43467674	ATGACGCTCCAAGAGTCAATGTCAATAGGGACGACCAAATGGCGGAAGCTATGAATAATATGGCTGCTTCTTTTGCTGCACGGACTGCTGCAAAGACTTT	T/A	GCGGGATCTAGAAAAGAGGGAAAGAGAGATCCGTGCTGATGAGTCAAGAGGATTGGAAGATTTTCGCCGTTATAATCCTCTCAAGTTTAAGAGTGATGAG
Ca_LG_4:43467675	TGACGCTCCAAGAGTCAATGTCAATAGGGACGACCAAATGGCGGAAGCTATGAATAATATGGCTGCTTCTTTTGCTGCACGGACTGCTGCAAAGACTTTG	G/A	CGGGATCTAGAAAAGAGGGAAAGAGAGATCCGTGCTGATGAGTCAAGAGGATTGGAAGATTTTCGCCGTTATAATCCTCTCAAGTTTAAGAGTGATGAGA
Ca_LG_4:43467691	AATGTCAATAGGGACGACCAAATGGCGGAAGCTATGAATAATATGGCTGCTTCTTTTGCTGCACGGACTGCTGCAAAGACTTTGCGGGATCTAGAAAAGA	A/G	GGGAAAGAGAGATCCGTGCTGATGAGTCAAGAGGATTGGAAGATTTTCGCCGTTATAATCCTCTCAAGTTTAAGAGTGATGAGAATTCAGAGAAAGCGGA
Ca_LG_4:43478844	CACTTGAGATGAAAGCAACTAAAACTGCTATGCTAGAAAGTGAGACAGCCAAAACCCTTGTTCTTCCAATTGCAGCAGATAAGCGGAAAAGGGATAGAGT	T/C	CATTTGATTCACACAGAAGAATGCCAATAGTTGACGGAAAAACCTGTAATAGAAATTTGTATATATAATTAAACAAAATTTACAACTCAAAAACCTCATG
Ca_LG_4:43497433	TGATGGTCTAACTACAGCAGCGGGAGGATTAGAAGGGATATATGATTGGAGCAGCAACAACAAATTTTAAAAGAATTGCGGATGCCATAAGAATAAGATA	A/G	AAACAAAAATTAATGGTTGGGTGGAGCTGCAGCTGCCATCACCTATCACTTACACATATGCACAAGATCCCTTAGAAAAGTTTTATCCCGATGATGGTGA
Ca_LG_4:44270158	GCATCCACGAAAATCCCGCATGGGGTGTGGATGGTTTCGATCCTTTTGTTCCAGGGGGGGATGGCCTCTCATCATATTGCAGCAGGGACATTGGGCATATA	A/T	AGCGGGCCTTTTCCATCTTAGTGTACGTCCACCTCAACGTTTATACAAAGGGTTGCGGATGGGAAATATTGAAACCGTCCTTTCCAGTAGTATCACGGTT
Ca_LG_4:44273946	AATCTTGTGCATCCACGTAAATCCCGCATGGGGTGTGGATGGTTTCGATCCTTTTGTTCCAGGGGGGGATGGCCTCTCATCATATTGCAGCAGGGACGTTA	A/G	GGCATATTAGCGGGCCTTTTCCATCTTAGTGTACGTCCACCTCAACGTTTATACAAAGGGTTGCGTATGGGAAATATTGAAACCGTCCTTTCCAGTAGTA
Ca_LG_4:44274053	TAGCGGGCCTTTTCCATCTTAGTGTACGTCCACCTCAACGTTTATACAAAGGGTTGCGTATGGGAAATATTGAAACCGTCCTTTCCAGTAGTATCGCGGT	T/C	TGTATTTTTTGCAGCTTTTGTTGTTGCTGGAACTATGTGGTATGTTTCAGCAACTACCCCGATTGAATTATTTGGTCCTACTCGTTATCAATGGGATCAG
Ca_LG_4:44274098	ACAAAGGGTTGCGTATGGGAAATATTGAAACCGTCCTTTCCAGTAGTATCGCGGTTGTATTTTTTGCAGCTTTTGTTGTTGCTGGAACTATGTGGTATGT	T/G	TTCAGCAACTACCCCGATTGAATTATTTGGTCCTACTCGTTATCAATGGGATCAGGGATACTTCCAACAAGAAATATATCGAAGAGTTGGTGCTGGGCTA
Ca_LG_4:44389251	TCTATGTTATGTGTTCTACCATGAAGCCTCAAGGAGTCACAGAAGACCACATTAAGCTGAGAGTCTTCCCATTTTCACTCCAAGATGCTGCCAAGGACTG	G/T	GTTATACTACCTTCAACCGGGTTTTGTTACAAGTTGGAATGATCTCAAGATATTGTTTCTAGAAAAATTCTTCCCTGCCTCAAGTGTTGCTTCAATCAGA
Ca_LG_4:44389271	ATGAAGCCTCAAGGAGTCACAGAAGACCACATTAAGCTGAGAGTCTTCCCATTTTCACTCCAAGATGCTGCCAAGGACTGGTTATACTACCTTCAACCGG	G/T	GTTTTGTTACAAGTTGGAATGATCTCAAGATATTGTTTCTAGAAAAATTCTTCCCTGCCTCAAGTGTTGCTTCAATCAGAAAAGAAATATGTGGCATCAG
Ca_LG_4:44389292	GAAGACCACATTAAGCTGAGAGTCTTCCCATTTTCACTCCAAGATGCTGCCAAGGACTGGTTATACTACCTTCAACCGGGTTTTGTTACAAGTTGGAATG	G/C	ATCTCAAGATATTGTTTCTAGAAAAATTCTTCCCTGCCTCAAGTGTTGCTTCAATCAGAAAAGAAATATGTGGCATCAGGCAGATTGACAGGGAATCATT

Positions	-100 bp flanking sequences	SNPs within *desi* cultivar	+100 bp flanking sequences
Ca_LG_4:44389293	AAGACCACATTAAGCTGAGAGTCTTCCCATTTTCACTCCAAGATGCTGCCAAGGACTGGTTATACTACCTTCAACCGGGTTTTGTTACAAGTTGGAATGA	A/G	TCTCAAGATATTGTTTCTAGAAAAATTCTTCCCTGCCTCAAGTGTTGCTTCAATCAGAAAAGAAATATGTGGCATCAGGCAGATTGACAGGGAATCATTA
Ca_LG_4:44389299	ACATTAAGCTGAGAGTCTTCCCATTTTCACTCCAAGATGCTGCCAAGGACTGGTTATACTACCTTCAACCGGGTTTTGTTACAAGTTGGAATGATCTCAA	A/T	GATATTGTTTCTAGAAAAATTCTTCCCTGCCTCAAGTGTTGCTTCAATCAGAAAAGAAATATGTGGCATCAGGCAGATTGACAGGGAATCATTACATGAG
Ca_LG_4:44881279	TGTAAGAGCTCAACGGGGTATGGGGTAGAGATTGTGTTCTTAGGGGGGAGGATTTTCAGCTGATCTACCTTATGGTTGGATAGATAAATGTTTTGATTTA	A/C	TGCAATTATTTTTTAACAAGGGTTATTGAATATAAAAAACTTATTACACGAAACCCTATTTTCTTAGAACAGGTTGAGGGAGTAGGTATTATTGGTAGAG
Ca_LG_4:44896437	TTGGCAACCTACAAGGCTGCAATGTTAATTAAAATAGCTGGAGCAGCTATGGCTGCAGAATCAGTAGAAGCTGGGGCCAAATACTTTCTTGATTGATGGA	A/T	TAGATCAAATTGCGGATGCTAATTATAACACCAAATATTTTATTACAGGAATGCTCACAGTAAATTTGCTGCTGTGTTTTCCCCCTTCTTACTGTAAACA
Ca_LG_4:45129743	GTTGCGTTGTATTGTATACAGATTAAGCCTAATTTTTTTGACACAACTAGTTCATCCACGGCTGCACTAGTACATTGCTCGGACCCAATATGCCCTATCA	A/G	CGGTTCAAGGTGCGCAAGTTAAATGCTCTCGTAAGGTTAAGCATAATCAGTGCAGCTACAACTTTCAGTTTCAAGATAAGAGTGCTACCTCTGGTGTCTA
Ca_LG_4:45681679	AATAAGGCAGCTTCTAGAACACCCTAATTTCTTGGGTCTCAAGTCCGCGCAGTGTAATAATAATAATATGCATCTAAGCAATGGTGACTTAAACAGTAGG	G/A	TGCAGCCACCGCATACCACATTTAACCCTTATTACGACCTAATAAATACTAGTATACCAAATATTGGTTTACCAAGCAACAAAGCGCGAAAATATTCTCC
Ca_LG_4:46585492	GCCAAGTTCCCATTCCCCATGGGCAAAATGGCACTTGTCACCAAATTTGCAGCCTTCAGCAGAATTAAACTTGTTACATATGCGGCTTTTAACAGCAGAC	C/A	GGTGCAGAACCATTGTTAGCGACTGGGGGCGGTGCGACAACATTTCTGGATAATTGAGGTGCAGCAGAAGATTTTAGATTCATCATCTGGGCAACAGCAT
Ca_LG_4:47315089	GGCATCCAAAGTGAAGACCAGCTGTTTGCAGCTGGATCTTTGATATCAAGGAGTGATTATGAAGATGTTGTAACCGAAAGGTCCATTACAGAAGTGTGCG	G/A	GTTATCCATTGTGCTGCAATGCTTTGCCATCTGAACGCCCTCGGAAGGGTCGATATCGAATTTCATTGAAGGAGCACAAGGTGTATGACCTGCACGAAAC
Ca_LG_4:47321057	GATAAATAATTTTATAACCAAGACAGACAGTAAAGAAGGATTTATAAATTGCAATGGTGGTTACAGCTATGCTGCAATCGTTGATATTGCAGGAAAATGC	C/T	AAACATATGTGTGACTAATACGGGCGTAATTGCAGTTGCAGAGGCCTAAAATCTTTTACGCTGCATACACAATTTCAGTTGTGGACTAAAATTTAAAACC
Ca_LG_4:47321058	ATAAATAATTTTATAACCAAGACAGACAGTAAAGAAGGATTTATAAATTGCAATGGTGGTTACAGCTATGCTGCAATCGTTGATATTGCAGGAAAATGCA	A/G	AACATATGTGTGACTAATACGGGCGTAATTGCAGTTGCAGAGGCCTAAAATCTTTTACGCTGCATACACAATTTCAGTTGTGGACTAAAATTTAAAACCA
Ca_LG_4:47911371	AATCCAAGAAAAGTTGGAAAAAAGCAGCATTCAGTTCACACCAGAATTTGTTATGGAGATATTGCAAATATGTGGTATGCATGGCTGCACTGTGCTAAAT	T/A	TTTTTTTCATGGGTAGGAAAGCAGCCCGGCTACAGACACACAGCAGAATCATACAACATTGCAATCAAAATTGCAGGCCAAGGGAAAGATTTCAAGCACA
Ca_LG_4:47911395	CAGCATTCAGTTCACACCAGAATTTGTTATGGAGATATTGCAAATATGTGGTATGCATGGCTGCACTGTGCTAAATTTTTTTTCATGGGTAGGAAAGCAG	G/A	CCCGGCTACAGACACACAGCAGAATCATACAACATTGCAATCAAAATTGCAGGCCAAGGGAAAGATTTCAAGCACATGCGCAGCCTATTCTATGAAATGA

Positions	-100 bp flanking sequences	SNPs within *desi* cultivar	+100 bp flanking sequences
Ca_LG_4:47977459	AGCATTGCATATAAGTGGTGAAAATAATTTTACCAGAGAGACCGTTGCTCAGCCTCTCCCAGATGCAGCATTTGGTATAGATGGTGAAAATAGCTTTACG	G/A	CGATATTCTGTTACTCAGCATCTTCCTAATGCAGCATTGAATCTAGGTGGTGAAAATAGTTTTACCACAGATGCTATTGCTCAGCCTCTCCCAAATGCAG
Ca_LG_4:47977597	GAATCTAGGTGGTGAAAATAGTTTTACCACAGATGCTATTGCTCAGCCTCTCCCAAATGCAGCGTTGACTCTAGGTGGTGAAAATAATTTTACCACACTG	G/A	TCCACACATACTATTACTCAGCCTCTCCATAATGCAGCATTGAATCTAAGTGTGGAAAATAATTTTACCAGAGATACTATTACTCAGCCTCTCAATAATG
Ca_LG_4:48000234	AAATATGTACAGCAGCAACAACCTTGCAGAACTGAACAAAATAATTATTTATTTGAGTTCAGATCAGAACACTTTGTTAACCGAGCTTGTGAAATTGTAC	C/T	GATACTCCTCAATGGCTTCCTTGTAAGCATGATCCTGTTGTTTTTCCATTATGACATATTCAACCTGCAGCAGCATAGTGAGCCTTTAAAAGTATATATA
Ca_LG_4:49261527	AACCACAAAATCAACTCTTTGTGAGGGTAAAAGCCGCCACAAAATCCCAAAGTGACGTTACCTTTTGTGGCTGCAAAGGCCGCCACAAAAGTCTGAGTCG	G/A	TTACCTTTTGTGGCTGCAAAGGCCGCCACAAACTCCAAAAGGGTTGTTGCCATTTGTGGCCGCCACAAACGCATGCCTTTCTGACCGTTTTGACCGCCAC
Ca_LG_4:49685210	TCCAACGGATTCGACCCGTTTTGCCACCCCTAATTTTAGTGCAGCATAGCCATAACCAGATAGGTGAAGAAAGTGAACTAGGAGCACACATAGTGATCGG	G/A	AAGTAAAATAAAGAACAAAATTTGGCTGCTTTAGAATTGGAGTAAATTGTCCCAAGTTTTGGAGAAACAATTTATTCACTTTACGGGTTTTTGTTCTTCC
Ca_LG_4:49685219	TTCGACCCGTTTTGCCACCCCTAATTTTAGTGCAGCATAGCCATAACCAGATAGGTGAAGAAAGTGAACTAGGAGCACACATAGTGATCGGAAGTAAAAT	T/G	AAAGAACAAAATTTGGCTGCTTTAGAATTGGAGTAAATTGTCCCAAGTTTTGGAGAAACAATTTATTCACTTTACGGGTTTTTGTTCTTCCTATAATTTG
Ca_LG_4:49701750	TTAGAGAGAAGGTAGAAGTGAAAAATGATAAGAGGAGTGGAGTGATATTTATAGAAAATTATTGATCTTTTGTGGCGGCTTTAACGGTCAGAAAATAAGG	G/A	AATTTGTGGCGGTCTGGGCGGCCACAAATTGCAACGGATCAATGGGCATTTATGGCGGCCTTTGCCCTCATAAAGTGCAACGGACCAATGATTTTGTGGC
Ca_LG_4:49707939	ACAAACCTTAATATGCAATGATGCTACTTCCCATCTTTGTCTTCGACGACGCTGCTTCCCACCTCTGTCTCCGACGCTGCTTCCCACCTCTGTCTCCGAC	C/A	GATGACGCTGCTTCTCGCCTATGTTTTTGGCATCAGCGCTGCTTCCCATCTTTATCTCCGGCGTCGGCGCTGCATTTAAAAACCCTATTGGAGGTGGTTA
Ca_LG_4:49707974	TTTGTCTTCGACGACGCTGCTTCCCACCTCTGTCTCCGACGCTGCTTCCCACCTCTGTCTCCGACGATGACGCTGCTTCTCGCCTATGTTTTTGGCATCA	A/G	GCGCTGCTTCCCATCTTTATCTCCGGCGTCGGCGCTGCATTTAAAAACCCTATTGGAGGTGGTTAGAACTGTCATAAACATTTGCAGTAGTTGGAACAAC
Ca_LG_4:49707991	TGCTTCCCACCTCTGTCTCCGACGCTGCTTCCCACCTCTGTCTCCGACGATGACGCTGCTTCTCGCCTATGTTTTTGGCATCAGCGCTGCTTCCCATCTT	T/C	TATCTCCGGCGTCGGCGCTGCATTTAAAAACCCTATTGGAGGTGGTTAGAACTGTCATAAACATTTGCAGTAGTTGGAACAACAATTTTTTTAACCGTCG
Ca_LG_4:51096414	AAACTTTGCAACGAATTTGCTGCGGAATTTGCTACGGAATTTGCTACGGAATTTGCTACGGACATTTACCGGAGATAAATCATATGGAAAAAATATGAAC	C/T	GTTGCTGCGGAAATATATTCGTATGTAAATCCGCAACAACAAAAAATGTTACCTGCCGTTTACCTGCGGAATATGGTACCGCAGGAAACGTCTTAAGATA
Ca_LG_4:51294972	CCGGCAGTTTGGCGTTTAAATAAGCCTGTGTTAGAGCATGGAAAGGAGATATTCTAGAAACATAACCAGATTGTCTCAATCTATAGAAAATCTTCTCTGT	T/C	ATTATGGACATCACCCCTCTTTGCATACTGCTCCATAATAGTCATATAAGTAGTAAATAATGGCTTCATCTTATTCTGCTGCATTGCCTTCTGCAAAACA

Positions	-100 bp flanking sequences	SNPs within *desi* cultivar	+100 bp flanking sequences
Ca_LG_4:51391473	CCGTCACCACCAAGATTGTTGCCACACATCTGGAGCAGGAACCTCCACCCCTTCGATTCCAGACTCCCAACTTTCACTCTATGTACTCAATAGCAGCCGT	T/C	TGTGGCCGCGATTGTGGCGATGGCAATGGCTCTGCGCTACACGAAACGGTGTTGCAGCACAGTTTAGGATTGTGGCTGTAGCGGCGTTCTTCGCGGCACA
Ca_LG_4:51629260	TCAAAAACCTAAATTGATACTAAAATTACAATAAAAAGGATAATGAAAGCTGATGAAAATGGTCATATACAAAAGATGATATAGGAGCTGCTATTAAAAT	T/G	TAATTATGCTTTTGATTTCTCGGAATATAGCATTCTTAGGTTCTAAGGTCCGAAAATTTTCACCATAGATTTGTCAAAATCACAGCAAAATTACATGAGT
Ca_LG_4:51629261	CAAAAACCTAAATTGATACTAAAATTACAATAAAAAGGATAATGAAAGCTGATGAAAATGGTCATATACAAAAGATGATATAGGAGCTGCTATTAAAATT	T/C	AATTATGCTTTTGATTTCTCGGAATATAGCATTCTTAGGTTCTAAGGTCCGAAAATTTTCACCATAGATTTGTCAAAATCACAGCAAAATTACATGAGTC
Ca_LG_4:51629281	AAAATTACAATAAAAAGGATAATGAAAGCTGATGAAAATGGTCATATACAAAAGATGATATAGGAGCTGCTATTAAAATTAATTATGCTTTTGATTTCTC	C/T	GGAATATAGCATTCTTAGGTTCTAAGGTCCGAAAATTTTCACCATAGATTTGTCAAAATCACAGCAAAATTACATGAGTCAAAATTTTCATGTATTACAT
Ca_LG_4:51697856	CCACTACAATTAAGTATATATAGTTCAACCTAATAAGACTCATTAATTAAAAACAGATAAAAATTAATTCATTATTTATGTTATTTTATAGCTGCCACAA	A/G	AAGAATCAATTAGCAATTGCAACTGAAAGCCAACAAGTTGAAGCTCTTCCTGCCAATGGTACATATCTATGTGTGCTTAGTTATTTTCCTGACTATTGTT
Ca_LG_4:52255582	GAAATCTGTGCAGCATTTGAGGAATTAGAGAAACAGAAAGCTGGTGGTATGACAGATGACACTGATGATTCCCATTTTGGTTCAGAGGCCCCTTCTTTTT	T/G	ATGGGGCAGCGAGCAGCATAAAGGATGCTACTGATGTTGTGGTATTAAATGCAGAAAAGGCTAAGACTATTATGGAAGATGTTGGCTCAAATTTGGATCA
Ca_LG_4:52302185	CGGGTTTATCGTATCATTGCTTTGGTTCCTTCCTTGAGAGATGTTTGCTTTGCTTTATTGGAGACAAGTGAAATAGCAGCAGTTAAGAAAATTGCAATGG	G/A	CTACTTGGAATGAAAGGAATAAGATGCAATGGGATGATGAGGTTGAAGAGTGTAGGATTCCAACTCAACGAGCTAATGGGACGTTTTACGCAGATCGGAA
Ca_LG_4:52302368	TTTTACGCAGATCGGAAAAATGCAGCAGCACAGACGCGTATGGTTCGGGGGCAGCAGATGGAAAATTCTGATGTTGGGGACGGTGCATTGGAGTACTCCA	A/C	GTGTAATATTGATGCAGCATTTTTTCAACAAAGAGCTACAACTATTGGTATGTGACTGTGTGATCAGCAAGAACGGACTACATTCCATTCCAAAGAGCTA
Ca_LG_4:52303691	ATTTTTGTTTATATATATTATACACATATATATGACATGTGAGAGGATATAAAAGGCAAAATACACCAGCTTGCGATTTTGTTAATACAAATGCTGCAGC	C/A	AAGTAGATAGGGATCAACAAGTCACTTCCAGTATTTGAGAAAACGATAGTATAAGTTGGGTTAACAATTGAAAAACTGCTAACATACCTTTGGACTCAAG
Ca_LG_4:52310252	TGGCTGGTTGCTAGAAAGGCAGCCGGAGTTGACAGCGATGGATGACTGTATGAAGCCAAAAATTGAAAGACTGAAAGGTGGTGCTTCTGGTGGCAAATGC	C/T	CCCCGCCGGCAGCGGTAGTGTGAAGCACAGAGACGCATAATAAGAATCTGTTCCGATACCATGTAAAAATGAGTAACTTATGCGTGTCTAATGAGACACC
Ca_LG_4:52311412	ATGCTCAATGTGGTTGCTGCTGCACCTTTGGGTGCAAACCCACCTTAATTTTGTGGGGGGTGTATGAATGTACTAGTTTACATAGTTTTACTTTACTCTA	A/T	GTTTCTGTTTAGCTCTAGAATACTATTCTGCCATGTGGCAGCTCTGTTAAAGAGTTAGTTACAGGGGTGTAGGCTAAGCCCCTTAGAGTGTAGGATAAGA
Ca_LG_4:52320309	TGTACCCTGGACATTGTCACACTCACCTAATTTGCATTGTCTAGAGCCTAATTTCATTTTCATGCAGCAAGGTCAAATGTTTTATCCACACCCTTCCCAT	T/C	ATAAAAAAAAATTAATTAACGAAATAAAAGAAGGTATGTTAAGATGCACTAAGCAGCAACTTTATTACCAGCAGAAGATTTAGAGAAGAATCCTCCAGGT

**Positions**	**-100 bp flanking sequences**	**SNPs within *desi* cultivar**	**+100 bp flanking sequences**
Ca_LG_4:52329402	TTTGCGGCTGAGGTGACTGTTCTATATTTGACAAAGCATTTGCAGCTTTCTGCATAAGAGCATCAAATAATAATGAATAATAACAATAACAACAACAATA	A/G	ATGATAACAGCAGCAGCAGCAACAACAACAATAATATTACAATAATAATAACAACAACAACAACAATAACAAAAAGGGTAAGCCACCATTTTAGTCCCTA
Ca_LG_4:53224488	CTTAGTCTTGCCCCAGCTGCCACTCCAACACACGGCTCGGCTCAGCTCCGTTTTTCTGGATACTCCCCTTAGAATTATTAGGGTAGAGGCATAATTATAT	T/A	TATATGGCTGCTTGTACATTTTCTTTTTTCTTTTTTTAGCTTTTTTGATAAATTTATTTCACTTGGTACACATTCAACAACTTCTCTTTACTTCTTTTCT
Ca_LG_4:53291168	GCAACAGTCATTTGGGCATTTGTGGCGGCCTTTGCCACCACAAAATGTAACGGATATGTGAATTTGTGACGGCCTGGGCGGCCAGAAAATCAGTTTCACC	C/A	TTTAATCCTTTGTGGCGGCTTTTGCAGCCACAAAAGGTAACGGTCATAGGCTTTTGTGGTGGCCTATATAGCCACAAATTGACATGCGTTTTCAGTCATT
Ca_LG_4:53376164	GTCTTTGATTTATGGCTAGTGTGATAAACTGTTCATGTTGACGAGTGCATTGGATGCAGCCTTTCGGAGCCTTGGACTAGCTCTAGCAACTTTTTTTATG	G/T	ATTTTGTACGACCACAGCTGCGAAAGGACCACAAGTTTGCATTTGTGAATAGTCATGATGAATAATTAACATGTTTATCTACCATTTTCACTTTCCTTGT
Ca_LG_4:53785677	AATTAGATAAACCTTGCGAATAAAACCCCATCGGAGCTCAGGTGATTCAATCATATATGGATAAAGGCTGCCATGTGCTTGGGGAAACCCTGCCTCAATA	A/G	TCACCCTTACCCATGGCGCCGCACGGAAACAAACAACCTCAAACACAATTAATTAGATGTTTAATTATGTCTCAATTCAATTTGTTTTTTTGTTATTGTT
Ca_LG_4:53785704	CCATCGGAGCTCAGGTGATTCAATCATATATGGATAAAGGCTGCCATGTGCTTGGGGAAACCCTGCCTCAATATCACCCTTACCCATGGCGCCGCACGGA	A/T	AACAAACAACCTCAAACACAATTAATTAGATGTTTAATTATGTCTCAATTCAATTTGTTTTTTTGTTATTGTTGTTGCAAATTGTAACTATTTGTCTTGG
Ca_LG_4:53802031	TGGTGTTTTAAGGCTGGTGTTACTGATGGATGCAGTCGAACTGTTGGAAACCTAATTTACACAGTAAGTTTCATTTTTGCATTATGCATGCAGCATTACG	G/C	CAGCTTAAAATGCTTCAATGTTATATATAATTGTACTTTTGTAGATAATTTTTTTATATTTTGATTGTTTTTTGTTTGTTAATTGCTGACATTTCTCTTT
Ca_LG_4:54016880	TAGGAACCCACACCCCAGACAAGGCAGAGTTAGTGAGCCTTGTAATAGGCCACCATTTCACACGGTTCGGGGGAACGTGAGTCAGCCGCCGGCGCCCGAT	T/C	TGCGTCTTGACCTCTATCCAATTTTTGGGCCAATTCCCCCTTCGTACTACCAAAAATTGAGTCCAAAAATTAAAACAATCGGATAATATAATTTGAGAGT
Ca_LG_4:54016893	CCCAGACAAGGCAGAGTTAGTGAGCCTTGTAATAGGCCACCATTTCACACGGTTCGGGGGAACGTGAGTCAGCCGCCGGCGCCCGATTGCGTCTTGACCT	T/C	CTATCCAATTTTTGGGCCAATTCCCCCTTCGTACTACCAAAAATTGAGTCCAAAAATTAAAACAATCGGATAATATAATTTGAGAGTGAGGTATTTTGGT
Ca_LG_4:54016937	TCACACGGTTCGGGGGAACGTGAGTCAGCCGCCGGCGCCCGATTGCGTCTTGACCTCTATCCAATTTTTGGGCCAATTCCCCCTTCGTACTACCAAAAAT	T/A	TGAGTCCAAAAATTAAAACAATCGGATAATATAATTTGAGAGTGAGGTATTTTGGTGTAATATATATATATATATATATATATAAAAGAATCCACACAAT
Ca_LG_4:54111380	TGAGGGATCTGTAACCATGTCAAAATGTTTCAAACTGCAAGCATCATCGTTGCAACTTGCAGGAGCATTTTCATTCTTCAAGCTGCACAGTGTCTCGGAG	G/T	GCTTCCACATCGGATCCTTCTGGAAGAAAGGCTTCCTCATTTTTACAACTCTTGCTGCCCGAATTGTCACCATCAATGGCAACACATTCCCGAGAACTGG
Ca_LG_4:54123542	GTAACGGTCTAATTGTTTGTGGTGGCCTGGGCGGCCAGAAATGCCAACGTCATTGTGCTTCCTTTGTGGCGGCGGCCTATGGAGCCATAAATGCCAACGT	T/C	ACACTTGCTTTTGTGGCGGCCTATGCAGCCACAAATGCACATCTCACTTCAGTCATTTGTGACGGCTTTGCCCCTCATAAGATTTATTTTTTGTGGCTGT

**Positions**	**-100 bp flanking sequences**	**SNPs within *desi* cultivar**	**+100 bp flanking sequences**
Ca_LG_4:54316004	TGTATGTGAAGCAGCCACCAGGATATGAGTAAAAGGCAAGGAAGACAGTGTATATAAGCTGAAGAAAACACTATATGGCATGAAGCAAGCTCCTAGAGCT	T/C	TAGAACAAGAGGATTGGCAAATTCCTTCTAGAGCAGCAATTTGAAAAATATGTCAATGAGCATGGAGTTTATGTTAAGAAGAGAGCAGCAACACCTGCAC
Ca_LG_4:54727814	AGGCAACAATAGCTTGTAAGCCACATGACCCACCCTAGCTAGCACTTGATAAGGACCATAGAATCTTGCTGCTAACTTAGCATAAGGTCTTGAGGCTAAT	T/G	GATTTTAATTTGTACGGCTGCAGCTTCAAATAAACCCAATCACCCTCTGAAAACTCTTCAAATCTCCTGTGTTTATTGGCTTGGGCTCGCATCTGGTCTT
Ca_LG_4:54727829	GTAAGCCACATGACCCACCCTAGCTAGCACTTGATAAGGACCATAGAATCTTGCTGCTAACTTAGCATAAGGTCTTGAGGCTAATGATTTTAATTTGTAC	C/T	GGCTGCAGCTTCAAATAAACCCAATCACCCTCTGAAAACTCTTCAAATCTCCTGTGTTTATTGGCTTGGGCTCGCATCTGGTCTTGAGCCCTAAGTAGAT
Ca_LG_4:54728337	GTACCAGCTGCTTTGAATAACTCCTTCCAGAAATGGCTTAAAAAAATAGGATCCCTATCAGAGACAATAGTTGAAGGGAAACCATGAAGTTTAACAACTC	C/T	CTGAAATGAAGACATCAGCAACTTCCTTAGCTAAAAATGGGTGGCCCAATGCAACAAAATGGGCGTACTTAGATAGCATGTCTACTACTACTAGAATAGT
Ca_LG_4:54839408	ATCTTTCACAAGTGTGTTGTCACCACTTGCTACCCATTTTCCAAGCTGCATGGACTCATTGATTGATGATTTCTCATATGCCTTTGCTGCTGATACTAGA	A/T	GGTTGAATGTCAATTTCAGCCTCACCCATAAAATCATCCGTTTTGAATGAATCCTTGTCATATACAATCTGCATTTAAATATCACATCAAATCTGATCAA
Ca_LG_4:54839420	TGTGTTGTCACCACTTGCTACCCATTTTCCAAGCTGCATGGACTCATTGATTGATGATTTCTCATATGCCTTTGCTGCTGATACTAGAGGTTGAATGTCA	A/T	ATTTCAGCCTCACCCATAAAATCATCCGTTTTGAATGAATCCTTGTCATATACAATCTGCATTTAAATATCACATCAAATCTGATCAAACATGAAAGGCA
Ca_LG_5:219198	TTATAAGTAATTAATTAATTTATTTAAATGTAATAATAATAACAACGGCAGCTGCTGCTGCAATAACAACAACAAGAACAACAACAACAATATCACCAGC	C/G	ATGTATCTAGTTTTTTGCAATGAGAATCTACGTGGACTGTATAAGTGTTATTGTTATTTTTTGAATGGACTATATCTTCAAACTTAAAAGTGAGTTATTA
Ca_LG_5:219230	ATAATAATAACAACGGCAGCTGCTGCTGCAATAACAACAACAAGAACAACAACAACAATATCACCAGCATGTATCTAGTTTTTTGCAATGAGAATCTACG	G/A	TGGACTGTATAAGTGTTATTGTTATTTTTTGAATGGACTATATCTTCAAACTTAAAAGTGAGTTATTATTAGCATGGTCTGACAGGTTTATGGATTACTT
Ca_LG_5:330349	AATCGCCCTTCACCAGCATTGAATCAACTGCTTCGTATGCTTCTTCCAGTGTCATGGCAACAGTAACTCCTTTTCCAGCAGCCAGTCCATCTGCTTTGAT	T/C	GACAATCGGTGCTCCTTCTTCTTGAATATATTGCTTGGCTGCAATCGGGTCTGTAAACGTTTTGTACTGCACTAATAAGTCCATTATAAGTAAACACAAC
Ca_LG_5:330356	CTTCACCAGCATTGAATCAACTGCTTCGTATGCTTCTTCCAGTGTCATGGCAACAGTAACTCCTTTTCCAGCAGCCAGTCCATCTGCTTTGATGACAATC	C/T	GGTGCTCCTTCTTCTTGAATATATTGCTTGGCTGCAATCGGGTCTGTAAACGTTTTGTACTGCACTAATAAGTCCATTATAAGTAAACACAACACAAAAA
Ca_LG_5:330386	TGCTTCTTCCAGTGTCATGGCAACAGTAACTCCTTTTCCAGCAGCCAGTCCATCTGCTTTGATGACAATCGGTGCTCCTTCTTCTTGAATATATTGCTTG	G/T	GCTGCAATCGGGTCTGTAAACGTTTTGTACTGCACTAATAAGTCCATTATAAGTAAACACAACACAAAAACTTGGAAGCAAAATAGACAAACAAAGAGAG
Ca_LG_5:330977	TGTCACACAAATGCTTCATGAAATTCTTAGAACCTTCAAGAGCAGCAGCCTCTGCAGATGGACCAAAAGTTGGAATTCCAGCCTTAACTAAATAATTTGA	A/C	AAGACCTGCAACAAGTGGGGCCTCAGGTCCAACAACAACTAGTCCCACCCCCCATTTCCGACAAAATGAAACTACTGCTGCTCCATCATAAACATCAAGG

Positions	-100 bp flanking sequences	SNPs within *desi* cultivar	+100 bp flanking sequences
Ca_LG_5:330987	ATGCTTCATGAAATTCTTAGAACCTTCAAGAGCAGCAGCCTCTGCAGATGGACCAAAAGTTGGAATTCCAGCCTTAACTAAATAATTTGAAAGACCTGCA	A/C	ACAAGTGGGGCCTCAGGTCCAACAACAACTAGTCCCACCCCCCATTTCCGACAAAATGAAACTACTGCTGCTCCATCATAAACATCAAGGTCTGAGATGC
Ca_LG_5:330996	GAAATTCTTAGAACCTTCAAGAGCAGCAGCCTCTGCAGATGGACCAAAAGTTGGAATTCCAGCCTTAACTAAATAATTTGAAAGACCTGCAACAAGTGGG	G/A	GCCTCAGGTCCAACAACAACTAGTCCCACCCCCCATTTCCGACAAAATGAAACTACTGCTGCTCCATCATAAACATCAAGGTCTGAGATGCACGTTGCAT
Ca_LG_5:331010	CTTCAAGAGCAGCAGCCTCTGCAGATGGACCAAAAGTTGGAATTCCAGCCTTAACTAAATAATTTGAAAGACCTGCAACAAGTGGGGCCTCAGGTCCAAC	C/T	AACAACTAGTCCCACCCCCCATTTCCGACAAAATGAAACTACTGCTGCTCCATCATAAACATCAAGGTCTGAGATGCACGTTGCATTTCCTGAGCTTGCA
Ca_LG_5:1897496	TTAAAAACTCTAAAAAGAAAATTCACCTTGGTAGTTGGAACTTACCTTTGTAGAAACTGGAATGATTTCTTCTTTGAATTTACCAGCTGCTGTTGCAGCT	T/C	GCGGCGCGCCTGTGAGACTCGACCTAATTACATATTAAAAAGAGTTAATTCACCCTGATAATCTCTTTATAGATTCTCATAGTAAATTAAATGCATATTT
Ca_LG_5:1897502	ACTCTAAAAAGAAAATTCACCTTGGTAGTTGGAACTTACCTTTGTAGAAACTGGAATGATTTCTTCTTTGAATTTACCAGCTGCTGTTGCAGCTGCGGCG	G/A	CGCCTGTGAGACTCGACCTAATTACATATTAAAAAGAGTTAATTCACCCTGATAATCTCTTTATAGATTCTCATAGTAAATTAAATGCATATTTCATGCT
Ca_LG_5:1897517	TTCACCTTGGTAGTTGGAACTTACCTTTGTAGAAACTGGAATGATTTCTTCTTTGAATTTACCAGCTGCTGTTGCAGCTGCGGCGCGCCTGTGAGACTCG	G/A	ACCTAATTACATATTAAAAAGAGTTAATTCACCCTGATAATCTCTTTATAGATTCTCATAGTAAATTAAATGCATATTTCATGCTTACAGCAGCCTGATC
Ca_LG_5:1897557	ATGATTTCTTCTTTGAATTTACCAGCTGCTGTTGCAGCTGCGGCGCGCCTGTGAGACTCGACCTAATTACATATTAAAAAGAGTTAATTCACCCTGATAA	A/T	TCTCTTTATAGATTCTCATAGTAAATTAAATGCATATTTCATGCTTACAGCAGCCTGATCTTGCTCCTGCCTTGTGACGCCATAACGCTCAGCAACGTTT
Ca_LG_5:1897564	CTTCTTTGAATTTACCAGCTGCTGTTGCAGCTGCGGCGCGCCTGTGAGACTCGACCTAATTACATATTAAAAAGAGTTAATTCACCCTGATAATCTCTTT	T/C	ATAGATTCTCATAGTAAATTAAATGCATATTTCATGCTTACAGCAGCCTGATCTTGCTCCTGCCTTGTGACGCCATAACGCTCAGCAACGTTTTCAGAAG
Ca_LG_5:1897577	ACCAGCTGCTGTTGCAGCTGCGGCGCGCCTGTGAGACTCGACCTAATTACATATTAAAAAGAGTTAATTCACCCTGATAATCTCTTTATAGATTCTCATA	A/T	GTAAATTAAATGCATATTTCATGCTTACAGCAGCCTGATCTTGCTCCTGCCTTGTGACGCCATAACGCTCAGCAACGTTTTCAGAAGTGATTCCCATAGG
Ca_LG_5:2079020	GCCCATATTCCTTTTTACTTGGATGTCAAGACACGCCCCGTATTTAGCAAGGCATGGCTGACTTATTTTAGGAGCAGCAGCGACTGATTTTCCCTGATCC	C/G	AGTTTTTTAGCTGCAACTATGGCAACTTTATTACCCTCCAGCTTTTGTAGTTTGCCAGCTTTATTACCCTCCAATTTTTGAGGTTTGCCAGCTTTATTTT
Ca_LG_5:2187260	TTAACTGCAACAACTTAAATATACGCTATTGGAGCTGGAATTACCGCGGCTGTTGGCACCAAACTTGCCCTCCAATGGATCCTCGTTAAAGGATTTAGAT	T/G	TGTACTCATTCCAATTACCAGACTCAATGAGCCCGGTATTGTTATTTATTGTCACTACCTCCCCGTGTTAGGATTGGGTAATTTGCGCGCCTGCTGCCTT
Ca_LG_5:2372833	CTTAAACCAACTAGCTTATGATTCAAACAAAGGAGAAACCTTTTTTATTAAGAGGCAAAAGAATTTATTTATAATCATAATTGAGCCACAAGATGTGGAA	A/G	GGAATGAAAAACAGTTTGTTACAAGAAACTGCAAAAATGCAGTTAACACCAGAACCAAACGATTGCTCCTATGCTTAACCAATAGCATTCACTGATATAA

Positions	-100 bp flanking sequences	SNPs within *desi* cultivar	+100 bp flanking sequences
Ca_LG_5:2383126	TAGAATTAGTCCAATATATTCAAAATAGTAAAAAAAAACAAAAGGATTTATAGCTAACAAAAGAAATGCACAACATTCTAAAACATACCACAATTTGTGT	T/C	CTAAAACATACAGCTTATCCAAAGTCAAGTGGTGCTCTACAGATCTTAGTGCTGCAATTGAAGAAATTATTTTGCATTACAACATAACGAGTCGCAGTGG
Ca_LG_5:2383137	CAATATATTCAAAATAGTAAAAAAAAACAAAAGGATTTATAGCTAACAAAAGAAATGCACAACATTCTAAAACATACCACAATTTGTGTCTAAAACATAC	C/G	AGCTTATCCAAAGTCAAGTGGTGCTCTACAGATCTTAGTGCTGCAATTGAAGAAATTATTTTGCATTACAACATAACGAGTCGCAGTGGAGAAAGTAACA
Ca_LG_5:2383150	ATAGTAAAAAAAAACAAAAGGATTTATAGCTAACAAAAGAAATGCACAACATTCTAAAACATACCACAATTTGTGTCTAAAACATACAGCTTATCCAAAG	G/T	TCAAGTGGTGCTCTACAGATCTTAGTGCTGCAATTGAAGAAATTATTTTGCATTACAACATAACGAGTCGCAGTGGAGAAAGTAACAGCAAACTAGCAAA
Ca_LG_5:2605987	GTCATGATGTTATGGTGTAGCAGATTCTTTGCCCAATTGCTAGAGGCTGCAACAGCTATTATATCAGCCACGTTTCCCCCATAGCAACACCATGTTTATG	G/A	TGGTGTATTTTTGGCTTGCTGCCATTGTCCTTCATCTGCCACTGATAACATTTGAGAGAAGTGATAAATTGAGATGTAGAGACAAGCTTTCAAAGCACAT
Ca_LG_5:2605992	GATGTTATGGTGTAGCAGATTCTTTGCCCAATTGCTAGAGGCTGCAACAGCTATTATATCAGCCACGTTTCCCCCATAGCAACACCATGTTTATGTGGTG	G/C	TATTTTTGGCTTGCTGCCATTGTCCTTCATCTGCCACTGATAACATTTGAGAGAAGTGATAAATTGAGATGTAGAGACAAGCTTTCAAAGCACATGAATT
Ca_LG_5:2691950	TGGCTGCAAATAATTTGGACAAAAACATGAAAAGAATTTGGCTGCAAATAGTTTGGACAAAAACACTCATTTGTCCAAATAATTTGGCTGCAATAACATT	T/G	TTATCTATACTTTGGCTGCAATAATATGGCTGCAATAATTTGGTTTCAAATAATTTGGCAGCAAAAACAAAAAATGGTAATTATGGATCTCAAATTGAAA
Ca_LG_5:3207789	TCCATGTTGTTTTTAAACAGGTTGCTACCTTAGCGCCTAATGTCCCTGCAATGTATGAGCTGCATTTTGCAGTACCAATGGCCGGAGCCATTCTTTGTAA	A/C	TCTTAATTCTCGCTTAGATGCAGCTATGGTATCAGTCCTGCTGGAGCATTCACAAGCTAAGATTCTTTTTGTAGACTATCAACTACTTGAAATTGCTCAG
Ca_LG_5:3229986	TTTTGGGCTCATGGTGTTATTCATGCAGTTTTTCTTATTAATAGGCTGCCATCTAAATTTTTAAATTTTTTTTCTCCTTATCACATATTGCATGATTCTA	A/G	CTCCTGACATTTCCAACTTGAAGGTTTTTGGCTGCCTATGTTTTTCTACAACTTTGCAATCGTATGTTATCAAGGTGATGTTTTTCGAATCTTAGATTTA
Ca_LG_5:3737739	CAATGGTTCAAAACCCATCTTTTTTTCTATTGTCAGGGAGATGGAGAATTTGTTAGCAGCAGATATTAAACTCTCTTAATACTTTCAACTCATATCACAG	G/T	TTATCAATATCTTTGGATCAATTAGTTGAAACAATGATGAAGCTAGAAAAGTCAACGCAAGAAGCCTATGTTTTTTTTGCATATTTTTCTAACATGCAAC
Ca_LG_5:3755755	ATGCTTTGACAACTGTTCTTATTCATAATTTATCCTGTGGCATATTAAAGTGAATAAATTATTTGTTTCATGTATTTGCAGAAACTGACCGGGGGAACTA	A/C	TTGCTGAGACTAGGGCAGCAACCACCAACTGGGGACACCAGCCAAGTAAACAAGAAGCTGAGAAAATGATGAGAGAATCCACCTTGGATAGACTTTCTTC
Ca_LG_5:4630636	CATGCGATTGGAGATATGATCATCATCGTAGGCTGTTTCCTCGCCATCTTCGTCAGCTTGGGTAGCATCATTGGAGGGGCCAGCAGTGTCCTCTTGAGCG	G/A	GCACCAGATGATTGACCAAGGTCACTTTTATGCACATACGAGTGAAGCTGCTCATTCCTGACGATCTTCATGCTAGGCAGGACGCTTAAATCCAACACGT
Ca_LG_5:4630640	CGATTGGAGATATGATCATCATCGTAGGCTGTTTCCTCGCCATCTTCGTCAGCTTGGGTAGCATCATTGGAGGGGCCAGCAGTGTCCTCTTGAGCGGCAC	C/G	CAGATGATTGACCAAGGTCACTTTTATGCACATACGAGTGAAGCTGCTCATTCCTGACGATCTTCATGCTAGGCAGGACGCTTAAATCCAACACGTTGTC

Positions	-100 bp flanking sequences	SNPs within *desi* cultivar	+100 bp flanking sequences
Ca_LG_5:4630641	GATTGGAGATATGATCATCATCGTAGGCTGTTTCCTCGCCATCTTCGTCAGCTTGGGTAGCATCATTGGAGGGGCCAGCAGTGTCCTCTTGAGCGGCACC	C/T	AGATGATTGACCAAGGTCACTTTTATGCACATACGAGTGAAGCTGCTCATTCCTGACGATCTTCATGCTAGGCAGGACGCTTAAATCCAACACGTTGTCC
Ca_LG_5:4630656	CATCATCGTAGGCTGTTTCCTCGCCATCTTCGTCAGCTTGGGTAGCATCATTGGAGGGGCCAGCAGTGTCCTCTTGAGCGGCACCAGATGATTGACCAAG	G/T	GTCACTTTTATGCACATACGAGTGAAGCTGCTCATTCCTGACGATCTTCATGCTAGGCAGGACGCTTAAATCCAACACGTTGTCCTTCGTGGGTGTATAT
Ca_LG_5:4703781	TTCTTTTGTGATCAGTCTTATTGAACAAAGACTTACCACGGTAAGGAAGAAAGGGGAACAAGCACACTTGGAGAGCGCATTATAATGGATAGTTGTATGT	T/C	TGCGTTTGGGAAGGATGAATCGCTCCCGAAAAGGAATCTATTGATTCTCTCCCAATTGGATGGGCCGTAGGTGCGATGATTTACTTCACGGGCGAGGTCT
Ca_LG_5:4703788	GTGATCAGTCTTATTGAACAAAGACTTACCACGGTAAGGAAGAAAGGGGAACAAGCACACTTGGAGAGCGCATTATAATGGATAGTTGTATGTTGCGTTT	T/C	GGGAAGGATGAATCGCTCCCGAAAAGGAATCTATTGATTCTCTCCCAATTGGATGGGCCGTAGGTGCGATGATTTACTTCACGGGCGAGGTCTTTGGTTC
Ca_LG_5:4703845	CACTTGGAGAGCGCATTATAATGGATAGTTGTATGTTGCGTTTGGGAAGGATGAATCGCTCCCGAAAAGGAATCTATTGATTCTCTCCCAATTGGATGGG	G/A	CCGTAGGTGCGATGATTTACTTCACGGGCGAGGTCTTTGGTTCAAATCTAGGATGGCCCAACTGCGCCAAGGAAAAGAATAAAAGACTGATTTGACTCCT
Ca_LG_5:4703882	GCGTTTGGGAAGGATGAATCGCTCCCGAAAAGGAATCTATTGATTCTCTCCCAATTGGATGGGCCGTAGGTGCGATGATTTACTTCACGGGCGAGGTCTT	T/C	TGGTTCAAATCTAGGATGGCCCAACTGCGCCAAGGAAAAGAATAAAAGACTGATTTGACTCCTTCATGCATGCTCCAACTTGGCTCGGGGGATGTAGCTC
Ca_LG_5:4703894	GATGAATCGCTCCCGAAAAGGAATCTATTGATTCTCTCCCAATTGGATGGGCCGTAGGTGCGATGATTTACTTCACGGGCGAGGTCTTTGGTTCAAATCT	T/C	AGGATGGCCCAACTGCGCCAAGGAAAAGAATAAAAGACTGATTTGACTCCTTCATGCATGCTCCAACTTGGCTCGGGGGATGTAGCTCAGTTGGTAGAGC
Ca_LG_5:4703906	CCGAAAAGGAATCTATTGATTCTCTCCCAATTGGATGGGCCGTAGGTGCGATGATTTACTTCACGGGCGAGGTCTTTGGTTCAAATCTAGGATGGCCCAA	A/G	CTGCGCCAAGGAAAAGAATAAAAGACTGATTTGACTCCTTCATGCATGCTCCAACTTGGCTCGGGGGATGTAGCTCAGTTGGTAGAGCTCCGCTCTTGCA
Ca_LG_5:4704115	TTGCGATTACGGGTTGGAGGTCTAATTGTCCAGGCGGTAATGATAGTATCTTGTACCTGAACCGACGACTCACTTTTTTAAGTAATAGGAAAGAGGACCA	A/G	AAACATGCCACTGAAAGACTCTACTGAGACAAAGATGGGCTGTCACGAACGTAGAGGAGGTAGGATGGACAGTTGGTCACATGGACAGTAGTTGGAGTTG
Ca_LG_5:4766789	GATCTTGTGATGCTCTAACCAACTGATTGAGCTAAAGGGTCGACCCTATTTGCTTTTTATATACCTAATGGCATCCCTTATAGCGGCGGCAACAACAACG	G/A	ATGGTTTGGGTGAAAAATCAGAAGTAGTCAACAACGCCGACAATATTGGTTTAGGCAGTGGCGGCCGAAATGGTTTTTCCAATTTTTTTTGGCCATGAGG
Ca_LG_5:4961875	ATTGCAAAGCAGAAAAAGTAAAAACATGAAAAGAAACAAACCAAAGATCATGACAAAAAATTCTATAGATAAAACCGGTTTTCCTATGCCAAATTTCTCA	A/C	ACTTAAGTGCTAATTGAGAAAGAGTCTTTATAGAAGACCCATCTTCTTTGATTGCATTATTAGCAGCTTCTTTCAATTCCTTCATTCGGAGAGAGGCACG
Ca_LG_5:4961880	AAAGCAGAAAAAGTAAAAACATGAAAAGAAACAAACCAAAGATCATGACAAAAAATTCTATAGATAAAACCGGTTTTCCTATGCCAAATTTCTCAACTTA	A/G	AGTGCTAATTGAGAAAGAGTCTTTATAGAAGACCCATCTTCTTTGATTGCATTATTAGCAGCTTCTTTCAATTCCTTCATTCGGAGAGAGGCACGAAGAA

Positions	-100 bp flanking sequences	SNPs within *desi* cultivar	+100 bp flanking sequences
Ca_LG_5:4961897	AACATGAAAAGAAACAAACCAAAGATCATGACAAAAAATTCTATAGATAAAACCGGTTTTCCTATGCCAAATTTCTCAACTTAAGTGCTAATTGAGAAAG	G/T	AGTCTTTATAGAAGACCCATCTTCTTTGATTGCATTATTAGCAGCTTCTTTCAATTCCTTCATTCGGAGAGAGGCACGAAGAATTATCGTCACCGGAGAG
Ca_LG_5:4961905	AAGAAACAAACCAAAGATCATGACAAAAAATTCTATAGATAAAACCGGTTTTCCTATGCCAAATTTCTCAACTTAAGTGCTAATTGAGAAAGAGTCTTTA	A/G	TAGAAGACCCATCTTCTTTGATTGCATTATTAGCAGCTTCTTTCAATTCCTTCATTCGGAGAGAGGCACGAAGAATTATCGTCACCGGAGAGATGTACGA
Ca_LG_5:4961937	CTATAGATAAAACCGGTTTTCCTATGCCAAATTTCTCAACTTAAGTGCTAATTGAGAAAGAGTCTTTATAGAAGACCCATCTTCTTTGATTGCATTATTA	A/G	GCAGCTTCTTTCAATTCCTTCATTCGGAGAGAGGCACGAAGAATTATCGTCACCGGAGAGATGTACGAAGCAACACCGTCACCGGAGAGAGAGGCAGGAA
Ca_LG_5:4961956	TCCTATGCCAAATTTCTCAACTTAAGTGCTAATTGAGAAAGAGTCTTTATAGAAGACCCATCTTCTTTGATTGCATTATTAGCAGCTTCTTTCAATTCCT	T/A	TCATTCGGAGAGAGGCACGAAGAATTATCGTCACCGGAGAGATGTACGAAGCAACACCGTCACCGGAGAGAGAGGCAGGAAGCAGCGTCGCAGCACGTCG
Ca_LG_5:4961962	GCCAAATTTCTCAACTTAAGTGCTAATTGAGAAAGAGTCTTTATAGAAGACCCATCTTCTTTGATTGCATTATTAGCAGCTTCTTTCAATTCCTTCATTC	C/T	GGAGAGAGGCACGAAGAATTATCGTCACCGGAGAGATGTACGAAGCAACACCGTCACCGGAGAGAGAGGCAGGAAGCAGCGTCGCAGCACGTCGAAGAAA
Ca_LG_5:4961983	GCTAATTGAGAAAGAGTCTTTATAGAAGACCCATCTTCTTTGATTGCATTATTAGCAGCTTCTTTCAATTCCTTCATTCGGAGAGAGGCACGAAGAATTA	A/G	TCGTCACCGGAGAGATGTACGAAGCAACACCGTCACCGGAGAGAGAGGCAGGAAGCAGCGTCGCAGCACGTCGAAGAAAGGTTTCAAGATTGTTCCTTCT
Ca_LG_5:4962003	TATAGAAGACCCATCTTCTTTGATTGCATTATTAGCAGCTTCTTTCAATTCCTTCATTCGGAGAGAGGCACGAAGAATTATCGTCACCGGAGAGATGTAC	C/G	GAAGCAACACCGTCACCGGAGAGAGAGGCAGGAAGCAGCGTCGCAGCACGTCGAAGAAAGGTTTCAAGATTGTTCCTTCTTTTGCTTTTAATGCTTGGCA
Ca_LG_5:5372200	GGCGGCCACCATTTAAACAAATAGGCAGCCATCAACAACAATTTAAATCAACCAATTTAGCCATCATTCTAGACAGGCAGCCACCATTTTAAATCAGCAA	A/G	CAAAAATCAGCAAATCAACAACAAAAATCAGCAAATCAACATAAACACCTTCTATGCAACAAAAATCAGCAAATCAACAATAAAAATAAGCACAAACATC
Ca_LG_5:5599639	AGCTGGTCCTGAAAAACACCAAGCAGTAGCAGTAAGAAACAACGCTGATCTCTCGACATTCTATCGATGCAGTTTCGAAGGTTATCAAGACACACTGTAC	C/T	GCGCACTCTTTAAGACAATTCTACAGAGAATGTGACATTTATGGCACAGTTGATTTCATATTCGGCAACGCTGCAGTAGTCTTCCAAAATTGCAACATAT
Ca_LG_5:5599641	CTGGTCCTGAAAAACACCAAGCAGTAGCAGTAAGAAACAACGCTGATCTCTCGACATTCTATCGATGCAGTTTCGAAGGTTATCAAGACACACTGTACGC	C/T	GCACTCTTTAAGACAATTCTACAGAGAATGTGACATTTATGGCACAGTTGATTTCATATTCGGCAACGCTGCAGTAGTCTTCCAAAATTGCAACATATTC
Ca_LG_5:5599642	TGGTCCTGAAAAACACCAAGCAGTAGCAGTAAGAAACAACGCTGATCTCTCGACATTCTATCGATGCAGTTTCGAAGGTTATCAAGACACACTGTACGCG	G/T	CACTCTTTAAGACAATTCTACAGAGAATGTGACATTTATGGCACAGTTGATTTCATATTCGGCAACGCTGCAGTAGTCTTCCAAAATTGCAACATATTCG
Ca_LG_5:5599648	TGAAAAACACCAAGCAGTAGCAGTAAGAAACAACGCTGATCTCTCGACATTCTATCGATGCAGTTTCGAAGGTTATCAAGACACACTGTACGCGCACTCT	T/C	TTAAGACAATTCTACAGAGAATGTGACATTTATGGCACAGTTGATTTCATATTCGGCAACGCTGCAGTAGTCTTCCAAAATTGCAACATATTCGCTAGAA

**Positions**	**-100 bp flanking sequences**	**SNPs within *desi* cultivar**	**+100 bp flanking sequences**
Ca_LG_5:5599649	GAAAAACACCAAGCAGTAGCAGTAAGAAACAACGCTGATCTCTCGACATTCTATCGATGCAGTTTCGAAGGTTATCAAGACACACTGTACGCGCACTCTT	T/G	TAAGACAATTCTACAGAGAATGTGACATTTATGGCACAGTTGATTTCATATTCGGCAACGCTGCAGTAGTCTTCCAAAATTGCAACATATTCGCTAGAAA
Ca_LG_5:5599650	AAAAACACCAAGCAGTAGCAGTAAGAAACAACGCTGATCTCTCGACATTCTATCGATGCAGTTTCGAAGGTTATCAAGACACACTGTACGCGCACTCTTT	T/A	AAGACAATTCTACAGAGAATGTGACATTTATGGCACAGTTGATTTCATATTCGGCAACGCTGCAGTAGTCTTCCAAAATTGCAACATATTCGCTAGAAAA
Ca_LG_5:5599660	AGCAGTAGCAGTAAGAAACAACGCTGATCTCTCGACATTCTATCGATGCAGTTTCGAAGGTTATCAAGACACACTGTACGCGCACTCTTTAAGACAATTC	C/T	TACAGAGAATGTGACATTTATGGCACAGTTGATTTCATATTCGGCAACGCTGCAGTAGTCTTCCAAAATTGCAACATATTCGCTAGAAAACCGTTGCCAA
Ca_LG_5:5599673	AGAAACAACGCTGATCTCTCGACATTCTATCGATGCAGTTTCGAAGGTTATCAAGACACACTGTACGCGCACTCTTTAAGACAATTCTACAGAGAATGTG	G/A	ACATTTATGGCACAGTTGATTTCATATTCGGCAACGCTGCAGTAGTCTTCCAAAATTGCAACATATTCGCTAGAAAACCGTTGCCAAATCAAAAAAACGC
Ca_LG_5:5599687	TCTCTCGACATTCTATCGATGCAGTTTCGAAGGTTATCAAGACACACTGTACGCGCACTCTTTAAGACAATTCTACAGAGAATGTGACATTTATGGCACA	A/G	GTTGATTTCATATTCGGCAACGCTGCAGTAGTCTTCCAAAATTGCAACATATTCGCTAGAAAACCGTTGCCAAATCAAAAAAACGCGGTAACAGCACAAG
Ca_LG_5:5847419	AAAAACCTATCGATATACTTGTTTTGATGTAGCGTAAATGCCTTATGTAGATGCTATCACTTATTCTGCCTCGGCCGGATGCTGCCTTAGTTGATGTGAT	T/A	AGAATCCCCCTTGCTTCCTAATCATTTACTATGGTCTTGCTCAGTTAATAAAAGAAATTCGCTACCCCTACCTTCTCTTTCTTCCCTCACCCCACCTTTA
Ca_LG_5:5850279	TAGTCGCACACCAACCAAAGACTAAACTAAGGCAGCTATTGAGGAAGAAGGAGAGGCTAACGAGATATGGGCGACAAAGCGAGGGGATTGAGCCTTTCTT	T/C	CTAGCGCAAGAGTTTTCGTCGCTGGATTAAGACGACTTAACTACTTGTCTGAAAGCGGAAAAGAAAGTTGACTAGCCTTCAAGCCTGAATAAGCAAAAAA
Ca_LG_5:5853523	ATCTTCTTGTTGTTCCAAAGGAGCTTCTCATTCAAAAGGAAAGCAGAGAATGAAATGTGGGAAGCGAGCTTGCTGCACCTATCTATGGTAATAGAATCCT	T/C	GGTGGTAGTAGTTCTTTTTCTCTTAAAAGAGGGAATGAATAAGCTAATTTCTCACCAGATGAATGCAAGGCTGGAATAACAGAACAACTAGATTTTACTT
Ca_LG_5:5853567	AGAGAATGAAATGTGGGAAGCGAGCTTGCTGCACCTATCTATGGTAATAGAATCCTGGTGGTAGTAGTTCTTTTTCTCTTAAAAGAGGGAATGAATAAGC	C/G	TAATTTCTCACCAGATGAATGCAAGGCTGGAATAACAGAACAACTAGATTTTACTTACTAATAGGAAAGAAAAATCTATTGTTGACTTGTAAAGACGGGA
Ca_LG_5:5855317	ATGTTCCCTTTCTAAGATACGGATATAAGTCCCACATACCCCCTTCTCGAGCTTATTCTTTTTAAGAGACTGATGCCAACTATGAATATACGATCGAGTA	A/G	CGCTCTTGTCTATTTTTAGACTTCTTACTTTTATTTTTGCATTTTCTCTATAAAGCCTTCCCCAACGAAACAAATGTTGTTGCTCCTTCTGTTTAGAGTT
Ca_LG_5:5855358	CCTTCTCGAGCTTATTCTTTTTAAGAGACTGATGCCAACTATGAATATACGATCGAGTACGCTCTTGTCTATTTTTAGACTTCTTACTTTTATTTTTGCA	A/C	TTTTCTCTATAAAGCCTTCCCCAACGAAACAAATGTTGTTGCTCCTTCTGTTTAGAGTTTGCATGACTTAGCTCTCGTTGTGCGTGGACTTCCTAATCGA
Ca_LG_5:5855369	TTATTCTTTTTAAGAGACTGATGCCAACTATGAATATACGATCGAGTACGCTCTTGTCTATTTTTAGACTTCTTACTTTTATTTTTGCATTTTCTCTATA	A/G	AAGCCTTCCCCAACGAAACAAATGTTGTTGCTCCTTCTGTTTAGAGTTTGCATGACTTAGCTCTCGTTGTGCGTGGACTTCCTAATCGAACTAAGATGGG

Positions	-100 bp flanking sequences	SNPs within *desi* cultivar	+100 bp flanking sequences
Ca_LG_5:5855385	ACTGATGCCAACTATGAATATACGATCGAGTACGCTCTTGTCT ATTTTTAGACTTCTTACTTTTATTTTTGCATTTTCTCTATAAAGC CTTCCCCAACGA	A/G	AACAAATGTTGTTGCTCCTTCTGTTTAGAGTTTGCATGACTTA GCTCTCGTTGTGCGTGGACTTCCTAATCGAACTAAGATGGG TCGGGATGTTGGCTCA
Ca_LG_5:5867759	AAGCTCGGGCAGGAGTAGGACATTCCATAAAAATATTTCTGAT CCTCTAGCTCTCACTCGTTGCCCTATTCGGTCAAGCAACTTCA CTCCTCTCAAATCT	T/C	TATTGTTTCATCGACAAGTAGGGTTAATTCTAAGGTTCCTTAT TCTGGTTGTCAAGCTGAAGAAGAGGGAGAAAGAATGGGAAC AGCCCCACCAGCAGCC
Ca_LG_5:5867764	CGGGCAGGAGTAGGACATTCCATAAAAATATTTCTGATCCTCT AGCTCTCACTCGTTGCCCTATTCGGTCAAGCAACTTCACTCCT CTCAAATCTTATTG	G/T	TTTCATCGACAAGTAGGGTTAATTCTAAGGTTCCTTATTCTG GTTGTCAAGCTGAAGAAGAGGGAGAAAGAATGGGAACAGCC CCACCAGCAGCCCATCG
Ca_LG_5:5867776	GGACATTCCATAAAAATATTTCTGATCCTCTAGCTCTCACTCG TTGCCCTATTCGGTCAAGCAACTTCACTCCTCTCAAATCTTAT TGTTTCATCGACAA	A/G	GTAGGGTTAATTCTAAGGTTCCTTATTCTGGTTGTCAAGCTG AAGAAGAGGGAGAAAGAATGGGAACAGCCCCACCAGCAGC CCATCGAAAGGAATGGAA
Ca_LG_5:5867805	CTAGCTCTCACTCGTTGCCCTATTCGGTCAAGCAACTTCACTC CTCTCAAATCTTATTGTTTCATCGACAAGTAGGGTTAATTCTAA GGTTCCTTATTCT	T/C	GGTTGTCAAGCTGAAGAAGAGGGAGAAAGAATGGGAACAG CCCCACCAGCAGCCCATCGAAAGGAATGGAAAATGAAATAA GAATTCGTGTTCACTATCT
Ca_LG_5:5867817	CGTTGCCCTATTCGGTCAAGCAACTTCACTCCTCTCAAATCTT ATTGTTTCATCGACAAGTAGGGTTAATTCTAAGGTTCCTTATTC TGGTTGTCAAGCT	T/G	GAAGAAGAGGGAGAAAGAATGGGAACAGCCCCACCAGCAG CCCATCGAAAGGAATGGAAAATGAAATAAGAATTCGTGTTCA CTATCTATGGAGCCAGTG
Ca_LG_5:6641476	GGTCTTGATATTGTAACTAAAATATATCCTTTTACCTGTTGCAC ACAAAGCAGCTTGAATCATAATTTGATCGTCGATCCACAAAAG AAAATCCACACCT	T/C	ACAAGCTTATGACCACAAGCAGCGGCATGATCAAAAGGTCC CATTTCGTGATAAAGAGACCTGACATATTCCCACCCCTAAGT AGAATGAAGAATTTTAA
Ca_LG_5:6826378	GGAGAATCCGCTGTTCACTCCTATCGTCTTCTTGCTAACAGCC AAAGAGCGGATTGGCACAGCGAATTCGAGAGCAGCAGATTCC AACAACTGAAATTGC	C/T	GATTCTTCTTGAACTGACCTTTAATGAGGGAACGATATTGAT CCTCATTCATCTATATGTAGCTGCCTTTGTCTCTATCTATGAT GCTATAGATCATCTT
Ca_LG_5:6869647	GTCGATCCTCATCCTCCTCCACCTGAAGAGGATGATGAAGAA TATGAGTTTGGTGAGGCTACCGGTGAGGCTGAGGCGCCTATT GTGGTTGAGCCTGCCT	T/G	GTGAGGGTGCTGGTGCTGATATTGCTGCTTGGTGAGGATGT GGGTGAGGCTGAGGCGCATGAGGCGCCTGCTGTTGTGGTT GAGCTTGCCGGTGAGGGTG
Ca_LG_5:6869733	GGTTGAGCCTGCCTGTGAGGGTGCTGGTGCTGATATTGCTGC TTGGTGAGGATGTGGGTGAGGCTGAGGCGCATGAGGCGCCT GCTGTTGTGGTTGAGCT	T/C	TGCCGGTGAGGGTGCTGGATATTGCTGCTGCTGGTGAGGAT GTGGGTGAAGGTGCCGGAAGGGTGGCAAGGGGTGGTCAGT GGGCAAGGGTTGAAAGGGT
Ca_LG_5:6869738	AGCCTGCCTGTGAGGGTGCTGGTGCTGATATTGCTGCTTGGT GAGGATGTGGGTGAGGCTGAGGCGCATGAGGCGCCTGCTGT TGTGGTTGAGCTTGCCG	G/A	GTGAGGGTGCTGGATATTGCTGCTGCTGGTGAGGATGTGG GTGAAGGTGCCGGAAGGGTGGCAAGGGGTGGTCAGTGGGC AAGGGTTGAAAGGGTGCAGA
Ca_LG_5:6869789	GGTGAGGCTGAGGCGCATGAGGCGCCTGCTGTTGTGGTTGA GCTTGCCGGTGAGGGTGCTGGATATTGCTGCTGCTGGTGAG GATGTGGGTGAAGGTGCC	C/T	GGAAGGGTGGCAAGGGGTGGTCAGTGGGCAAGGGTTGAAA GGGTGCAGAGGAATCGAATTCCTGTGCATCGCCTTTCTTCT CCGTATTTCCGCAGAGGAA

Positions	-100 bp flanking sequences	SNPs within *desi* cultivar	+100 bp flanking sequences
Ca_LG_5:6870827	CCAACTCACCAGAAACATCAGTACCAGCACCAGCACCATCACCAGCAGGCTCAACCACAACAGCAGGCGCCTTAGCCTCACCGGTAGCCTCGCCTTCACT	T/C	GGCAGCCTCGCCTTCAACGGTAGCCTCACCCTCACCCTCACCAAACTCATATTCTTCATCATCCTCTTGAGGTGGAGGAGGATGAGGATCGACAACCCCT
Ca_LG_5:6870896	CCTTAGCCTCACCGGTAGCCTCGCCTTCACTGGCAGCCTCGCCTTCAACGGTAGCCTCACCCTCACCCTCACCAAACTCATATTCTTCATCATCCTCTTG	G/C	AGGTGGAGGAGGATGAGGATCGACAACCCCTTGGCACCCCTTCCATCACCAGCACCTTCACCCACATCCTCAGCAACATCAGCACCAGCACCAGCACCCTC
Ca_LG_5:6870898	TTAGCCTCACCGGTAGCCTCGCCTTCACTGGCAGCCTCGCCTTCAACGGTAGCCTCACCCTCACCCTCACCAAACTCATATTCTTCATCATCCTCTTGAG	G/A	GTGGAGGAGGATGAGGATCGACAACCCCTTGGCACCCCTTCCATCACCAGCACCTTCACCCACATCCTCAGCAACATCAGCACCAGCACCAGCACCCTCAC
Ca_LG_5:7534156	CCCTAAGGTTTGTCATTTTTTCAGATTTCAATAAACTAACATTTAACCTCATTTTTGTAAATAAACCACCTGTACAGCCAAAAAGAGCCAGCAAACCTCC	C/A	AACATTGGAGGGTAATAGGGCTGCCAAACTACAAAGGTTGGAGGGTAATAAAGCTGCCAAAATTGCAGCAAGAAAACTGGATTGGGGAAAATCGGTCGCT
Ca_LG_5:7534159	TAAGGTTTGTCATTTTTTCAGATTTCAATAAACTAACATTTAACCTCATTTTTGTAAATAAACCACCTGTACAGCCAAAAAGAGCCAGCAAACCTCCAAC	C/A	ATTGGAGGGTAATAGGGCTGCCAAACTACAAAGGTTGGAGGGTAATAAAGCTGCCAAAATTGCAGCAAGAAAACTGGATTGGGGAAAATCGGTCGCTGTT
Ca_LG_5:7534168	TCATTTTTTCAGATTTCAATAAACTAACATTTAACCTCATTTTTGTAAATAAACCACCTGTACAGCCAAAAAGAGCCAGCAAACCTCCAACATTGGAGGG	G/T	TAATAGGGCTGCCAAACTACAAAGGTTGGAGGGTAATAAAGCTGCCAAAATTGCAGCAAGAAAACTGGATTGGGGAAAATCGGTCGCTGTTGCCCATGTT
Ca_LG_5:7534179	GATTTCAATAAACTAACATTTAACCTCATTTTTGTAAATAAACCACCTGTACAGCCAAAAAGAGCCAGCAAACCTCCAACATTGGAGGGTAATAGGGCTG	G/A	CCAAACTACAAAGGTTGGAGGGTAATAAAGCTGCCAAAATTGCAGCAAGAAAACTGGATTGGGGAAAATCGGTCGCTGTTGCCCATGTTCCTAAAATAAG
Ca_LG_5:8858516	GTAGAAGGAGCACTTCAGGTTATGTATTCAAATTCATGAAATCTTCAATAGCATGGAGCTTAAATAAGCAGCTTGTGATAGCATTTTTCATCATGCGAAGC	C/T	TGAGTACATTGCTGACAGCTATGCAACCTGCCAAACATTGTGGTTGGAGTCATTAACAACAGAGCTAAAGATGGAAATTTGCAGACCAATTCCATTGCTG
Ca_LG_5:8858518	AGAAGGAGCACTTCAGGTTATGTATTCAAATTCATGAAATCTTCAATAGCATGGAGCTTAAATAAGCAGCTTGTGATAGCATTTTCATCATGCGAAGCTG	G/A	AGTACATTGCTGACAGCTATGCAACCTGCCAAACATTGTGGTTGGAGTCATTAACAACAGAGCTAAAGATGGAAATTTGCAGACCAATTCCATTGCTGAT
Ca_LG_5:8858520	AAGGAGCACTTCAGGTTATGTATTCAAATTCATGAAATCTTCAATAGCATGGAGCTTAAATAAGCAGCTTGTGATAGCATTTTCATCATGCGAAGCTGAG	G/A	TACATTGCTGACAGCTATGCAACCTGCCAAACATTGTGGTTGGAGTCATTAACAACAGAGCTAAAGATGGAAATTTGCAGACCAATTCCATTGCTGATTG
Ca_LG_5:9982389	ATAGAAGATGTGTGATCTTGTTTGACACACAAGAAATACACAAATAACATATTTTACATGGTATCGAGCAGGTTCATCCTAGGGCTGCTCTGCCTTTTCC	C/A	AACTGTGCCATAACCCTTTTAAGTTTTTTTTTCCTCTTCAAAATTCTGTGTTCCAACTGTGCTTTTCCATTCCAGCTGTGTGTATACTATTCTAGTGCAG
Ca_LG_5:10019465	GAGTGAGAAAATGCATCCAAAACAGGAGAGACAATGGTTGTTTCGGAAAACTTTTCCAGCAGAGACGGCAGACAGCAGCAGCAGACGGCAGCGGCGGCAG	G/A	TAGCAGACGGGGCAGCAGCGACGACAGGCAGCAGACGGCAGCAGATCTACTCGCATGAGAGATTAGCAGTGTGTGAGAGCGCGTGTGATGACTTTTGGCG

Positions	-100 bp flanking sequences	SNPs within *desi* cultivar	+100 bp flanking sequences
Ca_LG_5:10019478	CATCCAAAACAGGAGAGACAATGGTTGTTTCGGAAAACTTTTCCAGCAGAGACGGCAGACAGCAGCAGCAGACGGCAGCGGCGGCAGTAGCAGACGGGGC	C/T	AGCAGCGACGACAGGCAGCAGACGGCAGCAGATCTACTCGCATGAGAGATTAGCAGTGTGTGAGAGCGCGTGTGATGACTTTTGGCGATGCGCTTTCAGG
Ca_LG_5:10033305	TTGTTGTAACTGAACTTCATCTAAGGATATAACCTGATAAGTAAAGGCAATATAAGAATGATTCATAATCGCAGTAGGTAGGGCAGGAGTACACTGCTTA	A/C	AATTAGCAAATTCTCGAAATAAAAATAAAGTACCAACCAAACCACTCAGTTCAGCAGTAACAGAATATTGAGCAGCATTGTCATCAGGCTTTTGCGATCT
Ca_LG_5:10254664	GATAACTTAGAAGCAAAGATGGCAAAATATTATCATCTGGTATGTTCCATATGTTGCTATTCAGAATTGAAATCCAATCCTTTGCATCTACCTTTGAACT	T/G	CAAAAGCCCTCCGAGTGTATTGACAGCAATTGGCAATCCTCCACACTTTCTAGCAACCTTCCTACCAATTGCTTCTATGTTTTGGTATTTATTTCCACCA
Ca_LG_5:10254671	TAGAAGCAAAGATGGCAAAATATTATCATCTGGTATGTTCCATATGTTGCTATTCAGAATTGAAATCCAATCCTTTGCATCTACCTTTGAACTCAAAAGC	C/T	CCTCCGAGTGTATTGACAGCAATTGGCAATCCTCCACACTTTCTAGCAACCTTCCTACCAATTGCTTCTATGTTTTGGTATTTATTTCCACCATAATTTT
Ca_LG_5:10386031	AGATGACGAGGCTTCTGTGCCAAATAGATCATATACTCATCCACGATATCTTCTATGCGGAAAGCTGCTTCCCTAACTTGTTTCACCCATGTTTTGATCC	C/T	CTTCACTGTTGTTGTCTCCATTTAATGCAGCTCTTTTATCAGCATCCTTAAGGAAGGCTTGCATGCTTTCCAGTTCATCTTTAATGTCTGAAAATTCCTT
Ca_LG_5:10386036	ACGAGGCTTCTGTGCCAAATAGATCATATACTCATCCACGATATCTTCTATGCGGAAAGCTGCTTCCCTAACTTGTTTCACCCATGTTTTGATCCCTTCA	A/C	CTGTTGTTGTCTCCATTTAATGCAGCTCTTTTATCAGCATCCTTAAGGAAGGCTTGCATGCTTTCCAGTTCATCTTTAATGTCTGAAAATTCCTTGTGAA
Ca_LG_5:11191045	TTTCATTGGTAGTGCTATTGCGATTAGTGAGAAAACATTTTTGGATAAATATGTAACCCGATTTCAAAAAGACGCCCCTCTGTTGTTGGATGCATATAAA	A/T	GGTCACAAGTTAGATTGATCATCTGGGTATACTCTAAACTTTGAAGCTTTTAATTGGTTATACTTGATTCCAGAAGTTGACTACTCATTTTTTTATGATT
Ca_LG_5:11191062	TTGCGATTAGTGAGAAAACATTTTTGGATAAATATGTAACCCGATTTCAAAAAGACGCCCCTCTGTTGTTGGATGCATATAAAGGTCACAAGTTAGATTG	G/A	ATCATCTGGGTATACTCTAAACTTTGAAGCTTTTAATTGGTTATACTTGATTCCAGAAGTTGACTACTCATTTTTTTATGATTTTCATATATGGCTTATG
Ca_LG_5:11191105	ATTTCAAAAAGACGCCCCTCTGTTGTTGGATGCATATAAAGGTCACAAGTTAGATTGATCATCTGGGTATACTCTAAACTTTGAAGCTTTTAATTGGTTA	A/T	TACTTGATTCCAGAAGTTGACTACTCATTTTTTTATGATTTTCATATATGGCTTATGATGGTTTTTGTTACTTTGTTTTAGCATTAAATCATAAATATTT
Ca_LG_5:11191111	AAAAGACGCCCCTCTGTTGTTGGATGCATATAAAGGTCACAAGTTAGATTGATCATCTGGGTATACTCTAAACTTTGAAGCTTTTAATTGGTTATACTTG	G/T	ATTCCAGAAGTTGACTACTCATTTTTTTATGATTTTCATATATGGCTTATGATGGTTTTTGTTACTTTGTTTTAGCATTAAATCATAAATATTTGGATTT
Ca_LG_5:11657961	GAAGTAGATAGGAATTTAGAAACAGAGGATGTTGCCAACATTTTACTTGGAAAAAGTCTTTTAGTGCGCTGTGATCGTGTGGTAAAAACTCTTCGCCCTG	G/A	GGAAGAAAAAACTATCTACTTGGCCTGCACATTTAGAAATTTTCCCTGTAAGTCATTTTCCTTGCTGCAATCATTGACTTGCTATGTGATTATTGTGTTA
Ca_LG_5:11860170	CTATCTCATAAGTGCGTTGAGACCATAGCATGTTAAACGAAAGCGCCGCTTTTCTTTGTGTTGAGCTCCTCTTCCTTCGCTGCTTTCGTACACCAGGGGT	T/G	ATAGAAGCCCACGGAGCGGTAGGCTAAGCTGGAAAGAGAGAGATCAGTTGGTTTGGTTCTGGGTTGTCTCTATATTTCTAATTATTCTGTTAATAGAATC

Positions	-100 bp flanking sequences	SNPs within *desi* cultivar	+100 bp flanking sequences
Ca_LG_5:11860200	TGTTAAACGAAAGCGCCGCTTTTCTTTGTGTTGAGCTCCTCTTCCTTCGCTGCTTTCGTACACCAGGGGTATAGAAGCCCACGGAGCGGTAGGCTAAGCT	T/C	GGAAAGAGAGAGATCAGTTGGTTTGGTTCTGGGTTGTCTCTATATTTCTAATTATTCTGTTAATAGAATCTGAATTGATGCTGGTTTCTCCGTCTTATGT
Ca_LG_5:11860214	GCCGCTTTTCTTTGTGTTGAGCTCCTCTTCCTTCGCTGCTTTCGTACACCAGGGGTATAGAAGCCCACGGAGCGGTAGGCTAAGCTGGAAAGAGAGAGAT	T/G	CAGTTGGTTTGGTTCTGGGTTGTCTCTATATTTCTAATTATTCTGTTAATAGAATCTGAATTGATGCTGGTTTCTCCGTCTTATGTTTCAAAAATTATAA
Ca_LG_5:12037840	TTTCTGGTATAGGTCTCAACAATTTTATCATTTTCTTGATTCATAGAAGTATTATAGCCATGAATTTCATTAACAAGGTTATGATCAACATATAACATAC	C/G	TGAGCGGTTCTCAGATTGTTGATTGTAGTAGACACGTAGCGCTGTTGCTCTTTAAATATTTTTTATTAGCTAAGAATCCTAAATAATTACAAATAAAAGA
Ca_LG_5:12037854	CTCAACAATTTTATCATTTTCTTGATTCATAGAAGTATTATAGCCATGAATTTCATTAACAAGGTTATGATCAACATATAACATACTGAGCGGTTCTCAG	G/A	ATTGTTGATTGTAGTAGACACGTAGCGCTGTTGCTCTTTAAATATTTTTTATTAGCTAAGAATCCTAAATAATTACAAATAAAAGACACTAAAATAGCAG
Ca_LG_5:12037870	TTTTCTTGATTCATAGAAGTATTATAGCCATGAATTTCATTAACAAGGTTATGATCAACATATAACATACTGAGCGGTTCTCAGATTGTTGATTGTAGTA	A/T	GACACGTAGCGCTGTTGCTCTTTAAATATTTTTTATTAGCTAAGAATCCTAAATAATTACAAATAAAAGACACTAAAATAGCAGGTGGATAGAACTTTAT
Ca_LG_5:12038452	GCGCTTGGGTGGTTATTCTCGTCAACCTCCACTCTCATCTTTACGTAATACTGCACTCAAAGCTGCGGAAAAGAGATCACAGTTGGGATCTCTTCTTCCA	A/C	TCTGGACCAAATCGTATTGGTGGCAATAGTGTTATAATGAAGTCACTTACTCCAGCACAAGCAGCTGTGATGGCTGCAGAAAGGAGATTACAGGATGACA
Ca_LG_5:12038459	GGTGGTTATTCTCGTCAACCTCCACTCTCATCTTTACGTAATACTGCACTCAAAGCTGCGGAAAAGAGATCACAGTTGGGATCTCTTCTTCCATCTGGAC	C/A	CAAATCGTATTGGTGGCAATAGTGTTATAATGAAGTCACTTACTCCAGCACAAGCAGCTGTGATGGCTGCAGAAAGGAGATTACAGGATGACATATGGTG
Ca_LG_5:12038466	ATTCTCGTCAACCTCCACTCTCATCTTTACGTAATACTGCACTCAAAGCTGCGGAAAAGAGATCACAGTTGGGATCTCTTCTTCCATCTGGACCAAATCG	G/A	TATTGGTGGCAATAGTGTTATAATGAAGTCACTTACTCCAGCACAAGCAGCTGTGATGGCTGCAGAAAGGAGATTACAGGATGACATATGGTGTGGTTCT
Ca_LG_5:12038481	CACTCTCATCTTTACGTAATACTGCACTCAAAGCTGCGGAAAAGAGATCACAGTTGGGATCTCTTCTTCCATCTGGACCAAATCGTATTGGTGGCAATAG	G/A	TGTTATAATGAAGTCACTTACTCCAGCACAAGCAGCTGTGATGGCTGCAGAAAGGAGATTACAGGATGACATATGGTGTGGTTCTCAATTTTGTGATAAA
Ca_LG_5:12391970	AAGTTAGAAGTGAAAAATGATAAGAGGAGTGGAGTAATATATATATATATGATTGAGTTTATCCTTTGTGGCGGCCAAAGCAGACACAATAAGCAACGAT	T/C	TATGTGTCATTTGTGGCGGCCAAAACGGTCAAAAAGGCATGGATTTGTGGCGGCCTGGACGGCCACAACTGGCAACGGTCTCTTTATTCTTGTGTGGTGG
Ca_LG_5:12391988	GATAAGAGGAGTGGAGTAATATATATATATATGATTGAGTTTATCCTTTGTGGCGGCCAAAGCAGACACAATAAGCAACGATTATGTGTCATTTGTGGCG	G/A	GCCAAAACGGTCAAAAAGGCATGGATTTGTGGCGGCCTGGACGGCCACAACTGGCAACGGTCTCTTTATTCTTGTGTGGTGGCCTTTGCAGCCACAAAAT
Ca_LG_5:12392028	TTATCCTTTGTGGCGGCCAAAGCAGACACAATAAGCAACGATTATGTGTCATTTGTGGCGGCCAAAACGGTCAAAAAGGCATGGATTTGTGGCGGCCTGG	G/A	ACGGCCACAACTGGCAACGGTCTCTTTATTCTTGTGTGGTGGCCTTTGCAGCCACAAAATGCAACTGGCCATGCTTTTGTGGCGGCCTTTGCAGTCACAA

Positions	-100 bp flanking sequences	SNPs within *desi* cultivar	+100 bp flanking sequences
Ca_LG_5:12392042	GGCCAAAGCAGACACAATAAGCAACGATTATGTGTCATTTGTGGCGGCCAAAACGGTCAAAAAGGCATGGATTTGTGGCGGCCTGGACGGCCACAACTGG	G/A	CAACGGTCTCTTTATTCTTGTGTGGTGGCCTTTGCAGCCACAAAATGCAACTGGCCATGCTTTTGTGGCGGCCTTTGCAGTCACAAACTGCAACGTCACT
Ca_LG_5:12974344	AGGGTTAGGGCCTCAGAGGCAGTGTATCAATTGCAGCTTGCAGAAAATAGCAGTTTGTTCAAATTCCGTTACAATACTATAACCGCTATTTAACAAAACA	A/T	TTGTACTCAATTGTGTATTATTTAACGATAGTAATTTATTAAAATTCCGCTATAGCTGCAAATTTTTAGTGCTGCACAGGGGTATTGATGATCTATTGCT
Ca_LG_5:12974358	AGAGGCAGTGTATCAATTGCAGCTTGCAGAAAATAGCAGTTTGTTCAAATTCCGTTACAATACTATAACCGCTATTTAACAAAACATTGTACTCAATTGT	T/C	GTATTATTTAACGATAGTAATTTATTAAAATTCCGCTATAGCTGCAAATTTTTAGTGCTGCACAGGGGTATTGATGATCTATTGCTTTGCCTGTGAAAGA
Ca_LG_5:12974364	AGTGTATCAATTGCAGCTTGCAGAAAATAGCAGTTTGTTCAAATTCCGTTACAATACTATAACCGCTATTTAACAAAACATTGTACTCAATTGTGTATTA	A/G	TTTAACGATAGTAATTTATTAAAATTCCGCTATAGCTGCAAATTTTTAGTGCTGCACAGGGGTATTGATGATCTATTGCTTTGCCTGTGAAAGAGGCTGG
Ca_LG_5:12974372	AATTGCAGCTTGCAGAAAATAGCAGTTTGTTCAAATTCCGTTACAATACTATAACCGCTATTTAACAAAACATTGTACTCAATTGTGTATTATTTAACGA	A/G	TAGTAATTTATTAAAATTCCGCTATAGCTGCAAATTTTTAGTGCTGCACAGGGGTATTGATGATCTATTGCTTTGCCTGTGAAAGAGGCTGGTTTCAAGT
Ca_LG_5:13046477	TATTTATTAATTGAGTCTATAAAATTATTAACAGAAGACACAATCAAAGACATGTGCGAAACATCAAAAAGTGAGGATACAAAGAAGTCTGAAACATTGA	A/G	AAAGTGAGGATGCAAAGAAGTTAGAAACATTGAAAATTGAGGAAGAAAAGAAAGCTGAAACATTGAAAAGTGAAACACCAATATTAACTGCAGCAAGATA
Ca_LG_5:13046488	TGAGTCTATAAAATTATTAACAGAAGACACAATCAAAGACATGTGCGAAACATCAAAAAGTGAGGATACAAAGAAGTCTGAAACATTGAAAAGTGAGGAT	T/A	GCAAAGAAGTTAGAAACATTGAAAATTGAGGAAGAAAAGAAAGCTGAAACATTGAAAAGTGAAACACCAATATTAACTGCAGCAAGATATGGTATAGTTG
Ca_LG_5:13046490	AGTCTATAAAATTATTAACAGAAGACACAATCAAAGACATGTGCGAAACATCAAAAAGTGAGGATACAAAGAAGTCTGAAACATTGAAAAGTGAGGATGC	C/A	AAAGAAGTTAGAAACATTGAAAATTGAGGAAGAAAAGAAAGCTGAAACATTGAAAAGTGAAACACCAATATTAACTGCAGCAAGATATGGTATAGTTGAA
Ca_LG_5:13046498	AAATTATTAACAGAAGACACAATCAAAGACATGTGCGAAACATCAAAAAGTGAGGATACAAAGAAGTCTGAAACATTGAAAAGTGAGGATGCAAAGAAGT	T/A	TAGAAACATTGAAAATTGAGGAAGAAAAGAAAGCTGAAACATTGAAAAGTGAAACACCAATATTAACTGCAGCAAGATATGGTATAGTTGAAATGGTGAA
Ca_LG_5:13046499	AATTATTAACAGAAGACACAATCAAAGACATGTGCGAAACATCAAAAAGTGAGGATACAAAGAAGTCTGAAACATTGAAAAGTGAGGATGCAAAGAAGTT	T/A	AGAAACATTGAAAATTGAGGAAGAAAAGAAAGCTGAAACATTGAAAAGTGAAACACCAATATTAACTGCAGCAAGATATGGTATAGTTGAAATGGTGAAT
Ca_LG_5:13046505	TAACAGAAGACACAATCAAAGACATGTGCGAAACATCAAAAAGTGAGGATACAAAGAAGTCTGAAACATTGAAAAGTGAGGATGCAAAGAAGTTAGAAAC	C/T	ATTGAAAATTGAGGAAGAAAAGAAAGCTGAAACATTGAAAAGTGAAACACCAATATTAACTGCAGCAAGATATGGTATAGTTGAAATGGTGAATGTGCTT
Ca_LG_5:13142507	TACTCCTTCTCCGCCGTTGACTGATCTGATGACCTTAAATGCCTAGGGCAGCTCCTTCAACCATGGACTTGAAGTTCCGTGCTTGAAGTGAAGCTGGAAC	C/T	GAGTTCTTTCATAGCCCTATCCCGAGGATAGAAGGTTGAATTCTACTCTGCTGCTGACTTAGCTCGTCCTATCTCATCCCTTTCCTTTCTGTGGTTGTTA

Positions	-100 bp flanking sequences	SNPs within *desi* cultivar	+100 bp flanking sequences
Ca_LG_5:13152290	TGGATCGGAGAATTTATGGAGGAAATCGGCTTTTAAATTTCCAGAAACCACACGATTATATAGCCAAAGGGAATACGCCGCGCCTAAAATCATCCCAAGC	C/G	GCTGCTAATGTGGCTACAAAGCTATTTCTTTGGAAAGCTCCTACTGAGATGGGAAATTCCCCGATAAAGCTGCTAGTACCAGGTGAACTCATATTGGCCA
Ca_LG_5:13152351	AGCCAAAGGGAATACGCCGCGCCTAAAATCATCCCAAGCGCTGCTAATGTGGCTACAAAGCTATTTCTTTGGAAAGCTCCTACTGAGATGGGAAATTCCC	C/T	CGATAAAGCTGCTAGTACCAGGTGAACTCATATTGGCCAAAGTGGAAGAGAAGGAAATGGTAGAGAGATTCGGCATGGTGCTCACTAACCCTCCGTAATA
Ca_LG_5:13161179	GACATAAAATATGAATAATGAAGATAAATGAAATATATATCTAATCTAAGAATTTTGAGGCTGTACACCTAATTTTTTTTCCTGGGATTGTAGTTCAATC	C/T	GGTCAGAGCACCGCCCTGTCAAGGCGGAAGCTGCGGGTTCGAGCCCCGTCAGTCCCGACCTAGGATCCGATGAATCGATCAATTCATCTCTGTAGGGGGG
Ca_LG_5:13667522	ACTAACATTTAACCTCATTTTTGTAGATCCAATTAAATAAACCACCCGTACAGCCAAAGAAAGCCAGCAAACCTCAAAAATTGGAGGGTAATAGAGCTGG	G/C	AAAACTACAAAGGCTGGAGGGTAATAAAGCTGCCAAAGTTGCAGCAAGAAAACTGGATCGGGGAAAATCGGTCGTTGCTGCTGCTGCTCCTAAAATAAGT
Ca_LG_5:13685133	TGGATATGCTCTTCTTCGATGCTCTTTCTGAGAAAAGGATTCATAATTCACTACAATTTTTTACGTCCGTTGTCCTCAAAAAGATACTTATAGCGAGCAT	T/G	CACTACTAAAAATGACCGTTCGATAGGTTCCCATTTGGGGGCTTCAACTAGATTTCGCCCTATGCATTGGGAATTGGATCAAGATCAACTGCTTTTTAAT
Ca_LG_5:13685182	ACTACAATTTTTTACGTCCGTTGTCCTCAAAAAGATACTTATAGCGAGCATCACTACTAAAAATGACCGTTCGATAGGTTCCCATTTGGGGGCTTCAACT	T/C	AGATTTCGCCCTATGCATTGGGAATTGGATCAAGATCAACTGCTTTTTAATTAAACTTTTGATAAGTGAAACCATTTTCTAGACACAAGCTTACATCATA
Ca_LG_5:13685201	GTTGTCCTCAAAAAGATACTTATAGCGAGCATCACTACTAAAAATGACCGTTCGATAGGTTCCCATTTGGGGGCTTCAACTAGATTTCGCCCTATGCATT	T/C	GGGAATTGGATCAAGATCAACTGCTTTTTAATTAAACTTTTGATAAGTGAAACCATTTTCTAGACACAAGCTTACATCATATTTTTCTAAAATAAAGAAA
Ca_LG_5:14305618	CTTTGGTTGTGTTATAGTACATGAAGTTCTCTCCCATTTCATTGAGCATGGTGTTGAGTAGCCATGACATCACTAGGTTGTTTTCTAGTTGCAATATTTT	T/C	GAGTTTGGGATCTCATTTTTCTGGATTTTTGAAGTCTCTTATGATATACCTTTCTTTCCCTTTTTCTTGAAGGTAGATTCTAATTGGATGCACTTATTGA
Ca_LG_5:14305650	CCCATTTCATTGAGCATGGTGTTGAGTAGCCATGACATCACTAGGTTGTTTTCTAGTTGCAATATTTTGAGTTTGGGATCTCATTTTTCTGGATTTTTGA	A/G	AGTCTCTTATGATATACCTTTCTTTCCCTTTTTCTTGAAGGTAGATTCTAATTGGATGCACTTATTGATTGTAATTCTAGCCATTCATATTGTAATTGTT
Ca_LG_5:14305659	TTGAGCATGGTGTTGAGTAGCCATGACATCACTAGGTTGTTTTCTAGTTGCAATATTTTGAGTTTGGGATCTCATTTTTCTGGATTTTTGAAGTCTCTTA	A/T	TGATATACCTTTCTTTCCCTTTTTCTTGAAGGTAGATTCTAATTGGATGCACTTATTGATTGTAATTCTAGCCATTCATATTGTAATTGTTGTATTATTC
Ca_LG_5:14305661	GAGCATGGTGTTGAGTAGCCATGACATCACTAGGTTGTTTTCTAGTTGCAATATTTTGAGTTTGGGATCTCATTTTTCTGGATTTTTGAAGTCTCTTATG	G/A	ATATACCTTTCTTTCCCTTTTTCTTGAAGGTAGATTCTAATTGGATGCACTTATTGATTGTAATTCTAGCCATTCATATTGTAATTGTTGTATTATTCTA
Ca_LG_5:14305668	GTGTTGAGTAGCCATGACATCACTAGGTTGTTTTCTAGTTGCAATATTTTGAGTTTGGGATCTCATTTTTCTGGATTTTTGAAGTCTCTTATGATATACC	C/T	TTTCTTTCCCTTTTTCTTGAAGGTAGATTCTAATTGGATGCACTTATTGATTGTAATTCTAGCCATTCATATTGTAATTGTTGTATTATTCTATTTACAT

Positions	-100 bp flanking sequences	SNPs within *desi* cultivar	+100 bp flanking sequences
Ca_LG_5:14585128	AATTTATGATAAACTTTGATTTTTGACTAACCATGCACCAACTCTTCGATATATTTCTTGTTTGATGGTTCTATGAGTATCCAAAATACAAACGAGATGA	A/G	ATGTTTATTGTCCCAACTATTTTTGTTAGTCTCGATACCGATAAGAAATGAAATATTTTTTTTACAAAATTTTCTCCTGGGATGAAGAATAAAGCATAT
Ca_LG_5:14585146	ATTTTTGACTAACCATGCACCAACTCTTCGATATATTTCTTGTTTGATGGTTCTATGAGTATCCAAAATACAAACGAGATGAATGTTTATTGTCCCAACT	T/C	ATTTTTGTTAGTCTCGATACCGATAAGAAATGAAATATTTTTTTTACAAAATTTTCTCCTGGGATGAAGAATAAAGCATATCTTAGTTTCAATTAATGC
Ca_LG_5:16045497	TTGGAGCAAGTGGTGGCTATGGTATTGAATTCATCTCCTGATCAGCATCCTCATGTAAGGTGGGCAGCTATTAATGCAATTGGTGGGCAATTGTCTACTG	G/A	ATTTGGGTCTATATTTGCAAGTTCAATATCATCGGGGGGTGTTTCCAGCCCTAGCTGCTGCGATGGATGATTTTCAGAATCCTCGTGTAGAGACTCATGC
Ca_LG_5:16045507	TGGTGGCTATGGTATTGAATTCATCTCCTGATCAGCATCCTCATGTAAGGTGGGCAGCTATTAATGCAATTGGTGGGCAATTGTCTACTGATTTGGGTCT	T/C	ATATTTGCAAGTTCAATATCATCGGGGGGTGTTTCCAGCCCTAGCTGCTGCGATGGATGATTTTCAGAATCCTCGTGTAGAGACTCATGCCACTTCAGCT
Ca_LG_5:16045512	GCTATGGTATTGAATTCATCTCCTGATCAGCATCCTCATGTAAGGTGGGCAGCTATTAATGCAATTGGTGGGCAATTGTCTACTGATTTGGGTCTATATT	T/A	TGCAAGTTCAATATCATCGGGGGGTGTTTCCAGCCCTAGCTGCTGCGATGGATGATTTTCAGAATCCTCGTGTAGAGACTCATGCCACTTCAGCTCTGCT
Ca_LG_5:16045545	CCTCATGTAAGGTGGGCAGCTATTAATGCAATTGGTGGGCAATTGTCTACTGATTTGGGTCTATATTTGCAAGTTCAATATCATCGGGGGGTGTTTCCAG	G/T	CCCTAGCTGCTGCGATGGATGATTTTCAGAATCCTCGTGTAGAGACTCATGCCACTTCAGCTCTGCTCAACTTCAGCGAGAATTGCACACATGATATTTT
Ca_LG_5:16142733	AAGTTCGTATATGTGCTCCTTTTCAAGAAATTATTCATACTCATCTCCGAATATAGCTTTATTCATGGTTATGATGCGTGAATCGTCGTTGCTGCCCATG	G/A	TTCCTTTTTACTTGGATGTCAAGACATGCCCCGTATTTAGCAAGGCATGGGTGACTTATTTTAGGAGCAACAGCGACTGATTTTCCTCGATTCAGTTTTT
Ca_LG_5:16142736	TTCGTATATGTGCTCCTTTTCAAGAAATTATTCATACTCATCTCCGAATATAGCTTTATTCATGGTTATGATGCGTGAATCGTCGTTGCTGCCCATGTTC	C/G	CTTTTTACTTGGATGTCAAGACATGCCCCGTATTTAGCAAGGCATGGGTGACTTATTTTAGGAGCAACAGCGACTGATTTTCCTCGATTCAGTTTTTTAG
Ca_LG_5:16142773	TCATCTCCGAATATAGCTTTATTCATGGTTATGATGCGTGAATCGTCGTTGCTGCCCATGTTCCTTTTTACTTGGATGTCAAGACATGCCCCGTATTTAG	G/C	CAAGGCATGGGTGACTTATTTTAGGAGCAACAGCGACTGATTTTCCTCGATTCAGTTTTTTAGCTGCAACTTTGGCAGCTTTATTACCCTCCAGCTTTTG
Ca_LG_5:16142783	ATATAGCTTTATTCATGGTTATGATGCGTGAATCGTCGTTGCTGCCCATGTTCCTTTTTACTTGGATGTCAAGACATGCCCCGTATTTAGCAAGGCATGG	G/A	GTGACTTATTTTAGGAGCAACAGCGACTGATTTTCCTCGATTCAGTTTTTTAGCTGCAACTTTGGCAGCTTTATTACCCTCCAGCTTTTGTAGTTTGCCA
Ca_LG_5:16142784	TATAGCTTTATTCATGGTTATGATGCGTGAATCGTCGTTGCTGCCCATGTTCCTTTTTACTTGGATGTCAAGACATGCCCCGTATTTAGCAAGGCATGGG	G/T	TGACTTATTTTAGGAGCAACAGCGACTGATTTTCCTCGATTCAGTTTTTTAGCTGCAACTTTGGCAGCTTTATTACCCTCCAGCTTTTGTAGTTTGCCAG
Ca_LG_5:16544262	TATGATGTCAGGAAAACAATGACTCGATCCTTTGTTTCCTAATTCCTATATTCGCTGCATTTTATAAGCAACCTAGAGGGCACTACCTCTACTTCAAATA	A/C	TTTATATCATGTTCCTATACAATATTTTTTTTTATTCTTAGTTATTTGAGTTATGACTTGTTTTAAAGGATTGAGATTTATTAGCAACTATCATTTCCTC

Positions	-100 bp flanking sequences	SNPs within *desi* cultivar	+100 bp flanking sequences
Ca_LG_5:16544286	CGATCCTTTGTTTCCTAATTCCTATATTCGCTGCATTTTATAAGCAACCTAGAGGGCACTACCTCTACTTCAAATATTTATATCATGTTCCTATACAATA	A/G	TTTTTTTTTATTCTTAGTTATTTGAGTTATGACTTGTTTTAAAGGATTGAGATTTATTAGCAACTATCATTTCCTCTTTTTTATTTTTCCAATGCTTGTA
Ca_LG_5:16544287	GATCCTTTGTTTCCTAATTCCTATATTCGCTGCATTTTATAAGCAACCTAGAGGGCACTACCTCTACTTCAAATATTTATATCATGTTCCTATACAATAT	T/A	TTTTTTTTATTCTTAGTTATTTGAGTTATGACTTGTTTTAAAGGATTGAGATTTATTAGCAACTATCATTTCCTCTTTTTTATTTTTCCAATGCTTGTAG
Ca_LG_5:16544319	CATTTTATAAGCAACCTAGAGGGCACTACCTCTACTTCAAATATTTATATCATGTTCCTATACAATATTTTTTTTTATTCTTAGTTATTTGAGTTATGAC	C/A	TTGTTTTAAAGGATTGAGATTTATTAGCAACTATCATTTCCTCTTTTTTATTTTTCCAATGCTTGTAGCAGCATATTGTTCCTTTTCTTTTGTTTTATTT
Ca_LG_5:16580335	ACTGGCGTGAACCACAATGTTTTAGAGAGGACAAAGTGGTCAAACTCATCGTGTATGACAGTGCATATACCCACCTAGTTGCGCTGCAACGAAAAGGAAG	G/A	CTTCAATGTTGCATTTGACTTGACCTTGCACTGGTGCAATCCAATGTGTTAGCAGCTTGGTTGTCTCATTGCTGATGCAACAACTTTTGCATTTGTTAGT
Ca_LG_5:16580337	TGGCGTGAACCACAATGTTTTAGAGAGGACAAAGTGGTCAAACTCATCGTGTATGACAGTGCATATACCCACCTAGTTGCGCTGCAACGAAAAGGAAGCT	T/A	TCAATGTTGCATTTGACTTGACCTTGCACTGGTGCAATCCAATGTGTTAGCAGCTTGGTTGTCTCATTGCTGATGCAACAACTTTTGCATTTGTTAGTCC
Ca_LG_5:16580360	AGAGGACAAAGTGGTCAAACTCATCGTGTATGACAGTGCATATACCCACCTAGTTGCGCTGCAACGAAAAGGAAGCTTCAATGTTGCATTTGACTTGACC	C/T	TTGCACTGGTGCAATCCAATGTGTTAGCAGCTTGGTTGTCTCATTGCTGATGCAACAACTTTTGCATTTGTTAGTCCATGCACAACTCTCTTGTGCGTTC
Ca_LG_5:16580363	GGACAAAGTGGTCAAACTCATCGTGTATGACAGTGCATATACCCACCTAGTTGCGCTGCAACGAAAAGGAAGCTTCAATGTTGCATTTGACTTGACCTTG	G/A	CACTGGTGCAATCCAATGTGTTAGCAGCTTGGTTGTCTCATTGCTGATGCAACAACTTTTGCATTTGTTAGTCCATGCACAACTCTCTTGTGCGTTCATG
Ca_LG_5:16580365	ACAAAGTGGTCAAACTCATCGTGTATGACAGTGCATATACCCACCTAGTTGCGCTGCAACGAAAAGGAAGCTTCAATGTTGCATTTGACTTGACCTTGCA	A/T	CTGGTGCAATCCAATGTGTTAGCAGCTTGGTTGTCTCATTGCTGATGCAACAACTTTTGCATTTGTTAGTCCATGCACAACTCTCTTGTGCGTTCATGAA
Ca_LG_5:16580377	AACTCATCGTGTATGACAGTGCATATACCCACCTAGTTGCGCTGCAACGAAAAGGAAGCTTCAATGTTGCATTTGACTTGACCTTGCACTGGTGCAATCC	C/T	AATGTGTTAGCAGCTTGGTTGTCTCATTGCTGATGCAACAACTTTTGCATTTGTTAGTCCATGCACAACTCTCTTGTGCGTTCATGAACCATAAAAACAT
Ca_LG_5:16580382	ATCGTGTATGACAGTGCATATACCCACCTAGTTGCGCTGCAACGAAAAGGAAGCTTCAATGTTGCATTTGACTTGACCTTGCACTGGTGCAATCCAATGT	T/C	GTTAGCAGCTTGGTTGTCTCATTGCTGATGCAACAACTTTTGCATTTGTTAGTCCATGCACAACTCTCTTGTGCGTTCATGAACCATAAAAACATCTTCT
Ca_LG_5:16580404	CCCACCTAGTTGCGCTGCAACGAAAAGGAAGCTTCAATGTTGCATTTGACTTGACCTTGCACTGGTGCAATCCAATGTGTTAGCAGCTTGGTTGTCTCAT	T/C	TGCTGATGCAACAACTTTTGCATTTGTTAGTCCATGCACAACTCTCTTGTGCGTTCATGAACCATAAAAACATCTTCTACTATGTCATCCCAAATATGAT
Ca_LG_5:16580406	CACCTAGTTGCGCTGCAACGAAAAGGAAGCTTCAATGTTGCATTTGACTTGACCTTGCACTGGTGCAATCCAATGTGTTAGCAGCTTGGTTGTCTCATTG	G/A	CTGATGCAACAACTTTTGCATTTGTTAGTCCATGCACAACTCTCTTGTGCGTTCATGAACCATAAAAACATCTTCTACTATGTCATCCCAAATATGATTG

Positions	-100 bp flanking sequences	SNPs within *desi* cultivar	+100 bp flanking sequences
Ca_LG_5:17127789	NNNNNNNNNNNNNNNNNNNNNNNNNNNNNNNNNNNNNNNNN NNNNNNNNNNNNNNNNNNNNNNNNNNNNNNNNNNNNNNNNN NNNNNNCAGGGAATTTTT	T/C	AGACTTTAAAGTTGAACTGGAAAGCATCCAATTCTTCCTTAA GGATGCTGATAAAAGAGTTGCAGCTGATGGAGACAACAACA ATGAATGAATCAAAATA
Ca_LG_5:17159396	TTTCATCCATTAATATACAAGTTGAGAACATAAGTTTCACAGCT ACAAGCGAGAAGGTTGAGAGTTAACAGGGAAATAGGAAACAA CAATAGGAGCTGTA	A/T	GATCGCTGAGGATCCCCTTTGGGTTTTCTTGACAAAGAACCT GCTTCCACCACCACAGGAGTAGTACTCTTTTCTTCTTTGGAA TTAAAGCAGCAGAATA
Ca_LG_5:17500932	TAATTCCACCGCTTTACCTGATATTTCTTTTGCTTCTTCAAGGT CCTTCTTTGGAAGTACTGAGGCTGCTAAGTCTGATCTGCAAGT CAAACTGATGCAG	G/A	GTAAAATCTCAGTCATCTCCATGATAAATTTTGTACGACAAC CCAGTTAAATAACAACTGGTGCAATTATTGGCATGGCAGCAA TGTGAATCATGGAACA
Ca_LG_5:17500974	GTCCTTCTTTGGAAGTACTGAGGCTGCTAAGTCTGATCTGCAA GTCAAACTGATGCAGGTAAAATCTCAGTCATCTCCATGATAAA TTTTGTACGACAAC	C/T	CCAGTTAAATAACAACTGGTGCAATTATTGGCATGGCAGCAA TGTGAATCATGGAACAGCACTTTGCCCCTTTTCTGGAAACGT ATTAGGTTGATTGTAG
Ca_LG_5:18375842	AATAAATACATAAGAGAGGTGTTGAGAGATCTTGGAACAGAA CATCAGTGAATCACCTCCACCTCCATATGTTGTTGAGATCAGA TTTTCAAAAACAGTA	A/T	ACTACATACGATCATAGAATTACAAAGACTGGCTTATCTCAG CAAAATGCAGCTTCACTTTATTATTTAAAATGAGGTGCATTTC ATGAAGCTAGGAATG
Ca_LG_5:18375851	ATAAGAGAGGTGTTGAGAGATCTTGGAACAGAACATCAGTGA ATCACCTCCACCTCCATATGTTGTTGAGATCAGATTTTCAAAA ACAGTAACTACATAC	C/T	GATCATAGAATTACAAAGACTGGCTTATCTCAGCAAAATGCA GCTTCACTTTATTATTTAAAATGAGGTGCATTTCATGAAGCTA GGAATGATTTCTTAC
Ca_LG_5:18375871	TCTTGGAACAGAACATCAGTGAATCACCTCCACCTCCATATGT TGTTGAGATCAGATTTTCAAAAACAGTAACTACATACGATCAT AGAATTACAAAGAC	C/T	TGGCTTATCTCAGCAAAATGCAGCTTCACTTTATTATTTAAAA TGAGGTGCATTTCATGAAGCTAGGAATGATTTCTTACTTGGA ATCTCTTACATTTTG
Ca_LG_5:18375874	TGGAACAGAACATCAGTGAATCACCTCCACCTCCATATGTTGT TGAGATCAGATTTTCAAAAACAGTAACTACATACGATCATAGA ATTACAAAGACTGG	G/A	CTTATCTCAGCAAAATGCAGCTTCACTTTATTATTTAAAATGA GGTGCATTTCATGAAGCTAGGAATGATTTCTTACTTGGAATC TCTTACATTTTGGTG
Ca_LG_5:18653777	CTTGTAGCTGGATTCTCCAGAACCACAAGAATCCTTAGTTTGA ATTTGAATAGGATTTCAACTCAGCACCTTTTGATATTTTGAGAA GAGTTGCTCTTTA	A/G	GAGAGCACAATACGATGAAAGTTGTAAGCTGCGTTCGGAGG GTTATTGTCTACCATTGGCCTCTATGGTACAATCAGTCGGGG CCTAAGAGACGGTGGTT
Ca_LG_5:18653787	GATTCTCCAGAACCACAAGAATCCTTAGTTTGAATTTGAATAG GATTTCAACTCAGCACCTTTTGATATTTTGAGAAGAGTTGCTC TTTAGAGAGCACAA	A/G	TACGATGAAAGTTGTAAGCTGCGTTCGGAGGGTTATTGTCTA CCATTGGCCTCTATGGTACAATCAGTCGGGGCCTAAGAGAC GGTGGTTTACCCTGTGG
Ca_LG_5:18654368	ATCTTAGTAGCCAGAGGAAAAGAAAGCAAAAGCGATTACCGT AGTAGCGGCAAGAAAAATGGGAACAACCTAAACCGTGAAAAC GGGGTTGTGGGAGAGA	A/C	AATACAAGTGTCGTGTTGCTAGGCAAAGCAATCGAATGTTGC ACCCTAGATGGTGGAAGTCCTGTAGCCGAAAGCATCACTAG CTTATGCTCTAACCCGA
Ca_LG_5:18654377	GCCAGAGGAAAAGAAAGCAAAAGCGATTACCGTAGTAGCGG CAAGAAAAATGGGAACAACCTAAACCGTGAAAACGGGGTTGT GGGAGAGAAATACAAGT	T/C	GTCGTGTTGCTAGGCAAAGCAATCGAATGTTGCACCCTAGA TGGTGGAAGTCCTGTAGCCGAAAGCATCACTAGCTTATGCT CTAACCCGAGTAGCATGG

Positions	-100 bp flanking sequences	SNPs within *desi* cultivar	+100 bp flanking sequences
Ca_LG_5:18654424	AAATGGGAACAACCTAAACCGTGAAAACGGGGTTGTGGGAGAGAAATACAAGTGTCGTGTTGCTAGGCAAAGCAATCGAATGTTGCACCCTAGATGGTGG	G/A	AAGTCCTGTAGCCGAAAGCATCACTAGCTTATGCTCTAACCCGAGTAGCATGGGGCACGTGGAATCCCGTGTGAATCAGCAAGGACCACCTTGCAAGGCT
Ca_LG_5:18654431	AACAACCTAAACCGTGAAAACGGGGTTGTGGGAGAGAAATACAAGTGTCGTGTTGCTAGGCAAAGCAATCGAATGTTGCACCCTAGATGGTGGAAGTCCT	T/A	GTAGCCGAAAGCATCACTAGCTTATGCTCTAACCCGAGTAGCATGGGGCACGTGGAATCCCGTGTGAATCAGCAAGGACCACCTTGCAAGGCTAATACTA
Ca_LG_5:19728236	AGCAATTTAAGATTTTTTTTTGCTGCCTTCATTGCAGCAATTTAAGATTTTTTTTTTTTGCTGCCTTCATTGCAGCAACTTAAGATCCCTTTTTTGCTGC	C/T	CTTCATTGCAGCTACTTAACATCTGTTAAANNNNNNNNNNNNNNNNNNNNNNNNNNNNNNNNNNNNNNNNNNNNNNNNNNNNNNNNNNNNNNNNNNNNNNN
Ca_LG_5:19770007	ATGAAATCCAGGATTTTGATGTAACATTTGGCATCTATGGAAGTGTCATAGGCTGCACAACTGGATGCATCTATGAAATAATATAGTTAATGTAAGTTAT	T/G	ATTTTTTTCACATGTTTACTACCTATACTGATGCATAAACGGATTGAAACTAAAAGCTTACGTTATTTATTGTTGAAGAAATAAAAATGTACATTTACAA
Ca_LG_5:19770036	GGCATCTATGGAAGTGTCATAGGCTGCACAACTGGATGCATCTATGAAATAATATAGTTAATGTAAGTTATATTTTTTTCACATGTTTACTACCTATACT	T/A	GATGCATAAACGGATTGAAACTAAAAGCTTACGTTATTTATTGTTGAAGAAATAAAAATGTACATTTACAAACTTATATGTTTAGATGTACTAATGAATT
Ca_LG_5:19770058	GCTGCACAACTGGATGCATCTATGAAATAATATAGTTAATGTAAGTTATATTTTTTTCACATGTTTACTACCTATACTGATGCATAAACGGATTGAAACT	T/C	AAAAGCTTACGTTATTTATTGTTGAAGAAATAAAAATGTACATTTACAAACTTATATGTTTAGATGTACTAATGAATTTGTGTAGATTTACATGACACTT
Ca_LG_5:20631627	TATCCTGTTCTGACTCATCTAGTCTATGCAACTCCATTTTGTGCTCCCTGAGCTTTCCAAACAATGTTGCGATGGTCATTTTTGCCAGATCTTTTGACTC	C/T	AGCAATAGCAGTTATTTTGGGCTGCCATTCTCTTGTTAAACACCTCATGATTTTTTCAATCTGCTGTTCATTTTCCACATGTTTTCCCAGGGCATGAAGG
Ca_LG_5:20631635	TCTGACTCATCTAGTCTATGCAACTCCATTTTGTGCTCCCTGAGCTTTCCAAACAATGTTGCGATGGTCATTTTTGCCAGATCTTTTGACTCAGCAATAG	G/T	CAGTTATTTTGGGCTGCCATTCTCTTGTTAAACACCTCATGATTTTTTCAATCTGCTGTTCATTTTCCACATGTTTTCCCAGGGCATGAAGGTTGTTTAC
Ca_LG_5:20631637	TGACTCATCTAGTCTATGCAACTCCATTTTGTGCTCCCTGAGCTTTCCAAACAATGTTGCGATGGTCATTTTTGCCAGATCTTTTGACTCAGCAATAGCA	A/T	GTTATTTTGGGCTGCCATTCTCTTGTTAAACACCTCATGATTTTTTCAATCTGCTGTTCATTTTCCACATGTTTTCCCAGGGCATGAAGGTTGTTTACGA
Ca_LG_5:20737748	TATAATCATAGTTTCTCTTTTCCAACATTCCGACACGTTTCACTCGATTTCCATTCAATTTGTTACTGTCGCCGTTGCCACTATTTCCGGCAAATTTTCT	T/C	ATCGAACAACAGTCATCTTCAGATCAAGTCATGACCAAAAGCGCGTCGCCAAAAACCGTCACACGCGCTGCAAATCTCTCATGCGAGTAGATATGTCGCT
Ca_LG_5:20737863	TCTTCAGATCAAGTCATGACCAAAAGCGCGTCGCCAAAAACCGTCACACGCGCTGCAAATCTCTCATGCGAGTAGATATGTCGCTGCTGCCGCTACTGCC	C/T	TGTCGTTGCTGCTGTCACGTCTCCTACTGCTGCCATCTGTGGTTACTGCCTGTCGTTGCTGCTGTCACGTCTCCTACTGCTGCCATCTGTGGTTGCTGCA
Ca_LG_5:22170319	CATCACCATATTTTTAAACAATTGCTCATCCAATCATTTTATGAAGGTATGCTACCTATGGATAGAAGCATTTTGGATGCTGCTAGTGGGGGAGCACTTG	G/A	TCGATAAGACCCCAGAAGCGGCAAAAGCTTTGATTGAGAACATGTCCATTAATTCTCAGTAGTTTACAACTAGAAGCAATTCAGTAGTGTTGATAAGGGG

**Positions**	**-100 bp flanking sequences**	**SNPs within *desi* cultivar**	**+100 bp flanking sequences**
Ca_LG_5:22170327	TATTTTTAAACAATTGCTCATCCAATCATTTTATGAAGGTATGCTACCTATGGATAGAAGCATTTTGGATGCTGCTAGTGGGGGAGCACTTGTCGATAAG	G/A	ACCCCAGAAGCGGCAAAAGCTTTGATTGAGAACATGTCCATTAATTCTCAGTAGTTTACAACTAGAAGCAATTCAGTAGTGTTGATAAGGGGTATGAATG
Ca_LG_5:22170334	AAACAATTGCTCATCCAATCATTTTATGAAGGTATGCTACCTATGGATAGAAGCATTTTGGATGCTGCTAGTGGGGGAGCACTTGTCGATAAGACCCCAG	G/A	AAGCGGCAAAAGCTTTGATTGAGAACATGTCCATTAATTCTCAGTAGTTTACAACTAGAAGCAATTCAGTAGTGTTGATAAGGGGTATGAATGAAATTCA
Ca_LG_5:22170374	CTATGGATAGAAGCATTTTGGATGCTGCTAGTGGGGGAGCACTTGTCGATAAGACCCCAGAAGCGGCAAAAGCTTTGATTGAGAACATGTCCATTAATTC	C/T	TCAGTAGTTTACAACTAGAAGCAATTCAGTAGTGTTGATAAGGGGTATGAATGAAATTCAAGCTTCTTTCCACAAGAATTTAGAAAGCAGACTTGATGAG
Ca_LG_5:22170379	GATAGAAGCATTTTGGATGCTGCTAGTGGGGGAGCACTTGTCGATAAGACCCCAGAAGCGGCAAAAGCTTTGATTGAGAACATGTCCATTAATTCTCAGT	T/A	AGTTTACAACTAGAAGCAATTCAGTAGTGTTGATAAGGGGTATGAATGAAATTCAAGCTTCTTTCCACAAGAATTTAGAAAGCAGACTTGATGAGCTTAC
Ca_LG_5:24175662	TGGATGTTCGTGTTAAAAATAAAGCCGGCAAAGTTGCAGCTCAAAAACTGGATCGGGGAAATAAAGCTGCCAAAGTTGCAGCTCAAAAACTAGATCGGGA	A/G	AAAATCAGTTGCTGCTCCTGCTCCTAAAATAAGTTAGCCATGTCTTGCTCGATATGGGTCGTGTCTTGACATAAAAGTAAAAACGAATTAGGGCAACAAC
Ca_LG_5:24179245	GATTTCTTGCAGCCAAAGATCAGCCTTGTCGGGGTCATGTTCCCCTTTAAATTTAGGCGAATCTTGTCGACGAAATTCATTCAATGTTCTTGCTGCACTG	G/A	GCTGCTTCATCCCTCTAATCTCTTTGTGCATCTCGTTGAGCCTAGACCGCAGCTTGTTGGGCCATTGCTGCGGCCATTGCATTCATTGCTTGGATTGCTT
Ca_LG_5:24179265	CAGCCTTGTCGGGGTCATGTTCCCCTTTAAATTTAGGCGAATCTTGTCGACGAAATTCATTCAATGTTCTTGCTGCACTGGCTGCTTCATCCCTCTAATC	C/A	TCTTTGTGCATCTCGTTGAGCCTAGACCGCAGCTTGTTGGGCCATTGCTGCGGCCATTGCATTCATTGCTTGGATTGCTTCAATCATGTCGTTTCTTCCT
Ca_LG_5:24179266	AGCCTTGTCGGGGTCATGTTCCCCTTTAAATTTAGGCGAATCTTGTCGACGAAATTCATTCAATGTTCTTGCTGCACTGGCTGCTTCATCCCTCTAATCT	T/C	CTTTGTGCATCTCGTTGAGCCTAGACCGCAGCTTGTTGGGCCATTGCTGCGGCCATTGCATTCATTGCTTGGATTGCTTCAATCATGTCGTTTCTTCCTT
Ca_LG_5:24179269	CTTGTCGGGGTCATGTTCCCCTTTAAATTTAGGCGAATCTTGTCGACGAAATTCATTCAATGTTCTTGCTGCACTGGCTGCTTCATCCCTCTAATCTCTT	T/A	TGTGCATCTCGTTGAGCCTAGACCGCAGCTTGTTGGGCCATTGCTGCGGCCATTGCATTCATTGCTTGGATTGCTTCAATCATGTCGTTTCTTCCTTGGG
Ca_LG_5:24179293	AAATTTAGGCGAATCTTGTCGACGAAATTCATTCAATGTTCTTGCTGCACTGGCTGCTTCATCCCTCTAATCTCTTTGTGCATCTCGTTGAGCCTAGACC	C/T	GCAGCTTGTTGGGCCATTGCTGCGGCCATTGCATTCATTGCTTGGATTGCTTCAATCATGTCGTTTCTTCCTTGGGGAACCGATTGGTTTGTGCGACGAG
Ca_LG_5:24179297	TTAGGCGAATCTTGTCGACGAAATTCATTCAATGTTCTTGCTGCACTGGCTGCTTCATCCCTCTAATCTCTTTGTGCATCTCGTTGAGCCTAGACCGCAG	G/T	CTTGTTGGGCCATTGCTGCGGCCATTGCATTCATTGCTTGGATTGCTTCAATCATGTCGTTTCTTCCTTGGGGAACCGATTGGTTTGTGCGACGAGTCAT
Ca_LG_5:25167090	GCAGAACCAAAACAAGCAGAACCAGAACAAGCAGCACATCAAAATGCAGAACCAGAACAAGCAGAACCAGAACAAGCAGCACAGAATAATGCAGAACCAA	A/G	AACAAGCAGAACCAGAACAAGCAGCACATCAAAATGCAGAACCAGAACAAGCAGCACATAATAATGCAGAACCAGAACAAGCACATCAAAATGTAGAACA

Positions	-100 bp flanking sequences	SNPs within *desi* cultivar	+100 bp flanking sequences
Ca_LG_5:25167184	AACCAAAACAAGCAGAACCAGAACAAGCAGCACATCAAAATGCAGAACCAGAACAAGCAGCACATAATAATGCAGAACCAGAACAAGCACATCAAAATGT	T/C	AGAACAAGCACATCAAAATGCAGAACAATGCAGAACATCACAATGTAGAACAAGCAGCACATCAAAATGCAGAATAATGCAGAACAAGCAGCACATCAAA
Ca_LG_5:25167230	ACCAGAACAAGCAGCACATAATAATGCAGAACCAGAACAAGCACATCAAAATGTAGAACAAGCACATCAAAATGCAGAACAATGCAGAACATCACAATGT	T/C	AGAACAAGCAGCACATCAAAATGCAGAATAATGCAGAACAAGCAGCACATCAAAATCCAGAATTGAACTAGCAGAATAAAACCAGAACAAGCACAAGAAT
Ca_LG_5:26747814	TATGGAATCAGCAAATATAGATACTTCAGTGAAAAAAGGGAATATAATATCAGTTGATAAGGATGCAGCTCCAGGGCAAGCTTCTGATTCTTGTGTCGAC	C/T	GCCGAGTTGGCATTTAAAGAGAGTGAACAATCTAAGGAGCAGCAAGCATCCATGTTAAAAGAAACCAGTAATTCAAAATATGTTTCTTGGGATGGCGACA
Ca_LG_5:27299558	GAGAAATCATTTCTCTCTTTTTCTCTCTTCTCTCTCTTTCTCTCTTCTCCATCTTCTCTCTTCGTTTCTCTATCTTGGTCGTTCTCGCTTGCTGCATTCG	G/A	ATTATGCCGATCTTCTCGCTTGCTACATTTGATTATGCCGATCTTCTCGCTTGCTGCATCTGTGCTGACATTTTGCCTGTATTTTTCCTTCTTTCTTTCT
Ca_LG_5:27299562	AATCATTTCTCTCTTTTTCTCTCTTCTCTCTCTTTCTCTCTTCTCCATCTTCTCTCTTCGTTTCTCTATCTTGGTCGTTCTCGCTTGCTGCATTCGATTA	A/C	TGCCGATCTTCTCGCTTGCTACATTTGATTATGCCGATCTTCTCGCTTGCTGCATCTGTGCTGACATTTTGCCTGTATTTTTCCTTCTTTCTTTCTTTCT
Ca_LG_5:27299565	CATTTCTCTCTTTTTCTCTCTTCTCTCTCTTTCTCTCTTCTCCATCTTCTCTCTTCGTTTCTCTATCTTGGTCGTTCTCGCTTGCTGCATTCGATTATGC	C/T	CGATCTTCTCGCTTGCTACATTTGATTATGCCGATCTTCTCGCTTGCTGCATCTGTGCTGACATTTTGCCTGTATTTTTCCTTCTTTCTTTCTTTCTGGT
Ca_LG_5:27299583	TCTTCTCTCTCTTTCTCTCTTCTCCATCTTCTCTCTTCGTTTCTCTATCTTGGTCGTTCTCGCTTGCTGCATTCGATTATGCCGATCTTCTCGCTTGCTA	A/G	CATTTGATTATGCCGATCTTCTCGCTTGCTGCATCTGTGCTGACATTTTGCCTGTATTTTTCCTTCTTTCTTTCTTTCTGGTTAAGTTGTCATTTTATTT
Ca_LG_5:27299606	CCATCTTCTCTCTTCGTTTCTCTATCTTGGTCGTTCTCGCTTGCTGCATTCGATTATGCCGATCTTCTCGCTTGCTACATTTGATTATGCCGATCTTCTC	C/T	GCTTGCTGCATCTGTGCTGACATTTTGCCTGTATTTTTCCTTCTTTCTTTCTTTCTGGTTAAGTTGTCATTTTATTTTAAGTCATTAACCATATTTATAT
Ca_LG_5:27657951	ACAAGCCACAGGCATAAATGTAATCAAAGTCTGCTTTTGGCATATGCTGTTGCAGCTGCTGCAGCATTGAGTTTGTCAAATCCTTTTGTAATGGAATACA	A/G	AAGGAAATGATAGCAGCAGAGATTCAGAGATGACTGAGAAATCTAGCATGGGAGGCAATGATAAGGACATTGATAAAAAAGAAAAGACAAGGAGAAAGAA
Ca_LG_5:28037026	AAGGAAGGCGGCCACATTACCTAAGGGAGGCGGCCACAAAGCTCAGACTCAGTGGCTAGGCAACAAACAGAAGGAATGAGAACGAAGAAAGACGAAGACA	A/G	GCGATGGCTAGGATGACATCACCGACGACAACGACAATACGGTGGCAGCACGACAATGTCAAAATGCATGGTTGCCGTTGACAGCGACGACAGTGACCTT
Ca_LG_5:29178061	AAATAGAAACCGCAATGAAGGTGGGTGGTAGGTTTATGCGGCAAATTTATGCGGAAGCAAATAGGTATAAAAAAATCTCAAGTTGCTGCAATGAAGGCAA	A/G	CAAAAAGGCAAAAAGAAATCTCACGTTGCTGCAATGAAGGCAGTAAAAAGGGCCATTCCATTCTCGAGCATGTTCAATCGTCCTCCCCAAGTGCTTGTTC
Ca_LG_5:31834738	TGAGTGAAAAGTATCATGTAGTGAGGAATGTCTCGAATCTCATGTTAGACAACTAAATGCATCTCGAGGTGATGTAGAGGAACTGGGGGACCTTCTCGAA	A/G	CACATGATAGGCAATTGAGAGACATTTGTTTTCTCTGACACATATTGTTTAGTTGCACTTAATAGGTCGACTTTGTCTCTAGCGGCCAACCACTCATAAT

**Positions**	**-100 bp flanking sequences**	**SNPs within *desi* cultivar**	**+100 bp flanking sequences**
Ca_LG_5:31834743	GAAAAGTATCATGTAGTGAGGAATGTCTCGAATCTCATGTTAGACAACTAAATGCATCTCGAGGTGATGTAGAGGAACTGGGGGACCTTCTCGAACACAT	T/G	GATAGGCAATTGAGAGACATTTGTTTTCTCTGACACATATTGTTTAGTTGCACTTAATAGGTCGACTTTGTCTCTAGCGGCCAACCACTCATAATTGGTC
Ca_LG_5:32180340	CTAATACCCCAAGAGATGATCCATGATCTACATACAGAACCTCGTAATCACACTGATGATTCGGGTCATCACAGTGATGATTTTGAATTGCAGCGCATAC	C/T	GGGATCCGTACATTTCACAATGTTATTATGCGGTTTATAAAGTTGTTCTACAGGCTGCAAACAAACCAAGTTCAATTCAAAATTACCATAACATCATTCC
Ca_LG_5:32180391	ACTGATGATTCGGGTCATCACAGTGATGATTTTGAATTGCAGCGCATACGGGATCCGTACATTTCACAATGTTATTATGCGGTTTATAAAGTTGTTCTAC	C/T	AGGCTGCAAACAAACCAAGTTCAATTCAAAATTACCATAACATCATTCCTTAGTAGTAACAAAATATTGTAAGGTAAAACATACCTTGGTGCAACCTGTA
Ca_LG_5:32187420	GAACTTATTGTCATTTGTAAATAAATATGCTGCTAGTGGCTTTGTTCCGGAGTTTATCACATCGAAGCTTTCTTCCAGCTTATTCACCTGTTCCAGGCAA	A/C	AAAGCAAGATTATAATTTGATACAAGCTGATGAAGGGTATCGATGAATATTCGAAGTATAAATACATGTTTCCAAGTTATTAGTTAAGCAATTAGCTAAA
Ca_LG_5:32187428	TGTCATTTGTAAATAAATATGCTGCTAGTGGCTTTGTTCCGGAGTTTATCACATCGAAGCTTTCTTCCAGCTTATTCACCTGTTCCAGGCAAAAAGCAAG	G/A	ATTATAATTTGATACAAGCTGATGAAGGGTATCGATGAATATTCGAAGTATAAATACATGTTTCCAAGTTATTAGTTAAGCAATTAGCTAAAACGTTTTG
Ca_LG_5:33219485	GATTACTTCAGAAGCAACATAAAATAGATAAGATTTTGTCTTCGATTTTTGATGTTTTCTATCCTCGTGATTTTTTATTTGTGCAAGCATTGGATTGTCT	T/C	GATGTTGGATCTATCACACAATCATTCTCAGCAGCCTCCCAAACACCATTAGCATCAAGATAAGCCTCAATTCTGGTTCCTCATATGTGATAGTTCCCAC
Ca_LG_5:33219489	ACTTCAGAAGCAACATAAAATAGATAAGATTTTGTCTTCGATTTTTGATGTTTTCTATCCTCGTGATTTTTTATTTGTGCAAGCATTGGATTGTCTGATG	G/T	TTGGATCTATCACACAATCATTCTCAGCAGCCTCCCAAACACCATTAGCATCAAGATAAGCCTCAATTCTGGTTCCTCATATGTGATAGTTCCCACCTTA
Ca_LG_5:33219497	AGCAACATAAAATAGATAAGATTTTGTCTTCGATTTTTGATGTTTTCTATCCTCGTGATTTTTTATTTGTGCAAGCATTGGATTGTCTGATGTTGGATCT	T/G	ATCACACAATCATTCTCAGCAGCCTCCCAAACACCATTAGCATCAAGATAAGCCTCAATTCTGGTTCCTCATATGTGATAGTTCCCACCTTAAAAAAATTG
Ca_LG_5:33405843	AAGGGTGGAAAACGTGATTGGAGGCCATTCAAATCTGTGGATGAAGAAGGAAGAGGGAAGAAAAATGAGAGCAGCAATGGAATTAGAGTAGAAAATGAAC	C/T	ACGAAATTGGTCATCCCTTGGTTCACTCACCTAGCAATGAAGAACATAAATATATACCAGTATCCCATTGGAAAGCTGAGGATGCAGCCAGTTGGCATAA
Ca_LG_5:33760675	NNNNNNNNNNNNNNNNNNNNNNNNNNNNNNNNNNNGCATTTGTGGCGGCCAAAGCAGCCACAATGCCAACGGATACTATCCATTTGTGACGGCCTGAGCGGT	T/C	CAGAAATGCCAGCTGTATTGGGCCTCCTTTGTGGCGGCATTTGCAGCCACAAATTCCACGTGCGCATTTCTTTGTGGCGACCTTTGCAGCCACAAATGCC
Ca_LG_5:35336065	AATTTACCTTGTGTCTTGATGTGGCAGGCGCATGAAAAAATCTAGTGTTCAAATCTTCTTCTTTCAACCAGAATGATTTAGCTCGTTGTCGCAAAAAAAT	T/C	GTTTTCTTTGACTACCAGTGATGTCATTTGCTTTGCAAATAAGTGAATCTTTCCGCATTTGTGTTGCTGCCCGATTGATGCGCATTTTCTGGTGCTTTCT
Ca_LG_5:35336068	TTACCTTGTGTCTTGATGTGGCAGGCGCATGAAAAAATCTAGTGTTCAAATCTTCTTCTTTCAACCAGAATGATTTAGCTCGTTGTCGCAAAAAAATGTT	T/C	TTCTTTGACTACCAGTGATGTCATTTGCTTTGCAAATAAGTGAATCTTTCCGCATTTGTGTTGCTGCCCGATTGATGCGCATTTTCTGGTGCTTTCTTAC

Positions	-100 bp flanking sequences	SNPs within *desi* cultivar	+100 bp flanking sequences
Ca_LG_5:35336080	TTGATGTGGCAGGCGCATGAAAAAATCTAGTGTTCAAATCTTCTTCTTTCAACCAGAATGATTTAGCTCGTTGTCGCAAAAAAATGTTTTCTTTGACTAC	C/G	CAGTGATGTCATTTGCTTTGCAAATAAGTGAATCTTTCCGCATTTGTGTTGCTGCCCGATTGATGCGCATTTTCTGGTGCTTTCTTACATTGTATAATTT
Ca_LG_5:35336086	TGGCAGGCGCATGAAAAAATCTAGTGTTCAAATCTTCTTCTTTCAACCAGAATGATTTAGCTCGTTGTCGCAAAAAAATGTTTTCTTTGACTACCAGTGA	A/G	TGTCATTTGCTTTGCAAATAAGTGAATCTTTCCGCATTTGTGTTGCTGCCCGATTGATGCGCATTTTCTGGTGCTTTCTTACATTGTATAATTTCTTAAC
Ca_LG_5:35336087	GGCAGGCGCATGAAAAAATCTAGTGTTCAAATCTTCTTCTTTCAACCAGAATGATTTAGCTCGTTGTCGCAAAAAAATGTTTTCTTTGACTACCAGTGAT	T/A	GTCATTTGCTTTGCAAATAAGTGAATCTTTCCGCATTTGTGTTGCTGCCCGATTGATGCGCATTTTCTGGTGCTTTCTTACATTGTATAATTTCTTAACG
Ca_LG_5:35490898	ATTTAAGATGGTATCAGAGCTCCTGATCCTTGGGAGCCTCTGACCGTGCCTGTAAACCCTAAGTTGCAGCCTTCATTGTTGCGCCACCTTTAAGCATTGC	C/T	TGCAGCCTTCATTGCAGCGCAACCCTCCGTGCCTGTAAACCCTAAGTTGCAGCCTTCATTGCTGCGCTACCTTTAAGCATTGTTGCAGCCTTCATTGCAG
Ca_LG_5:35490926	CTTGGGAGCCTCTGACCGTGCCTGTAAACCCTAAGTTGCAGCCTTCATTGTTGCGCCACCTTTAAGCATTGCTGCAGCCTTCATTGCAGCGCAACCCTCC	C/A	GTGCCTGTAAACCCTAAGTTGCAGCCTTCATTGCTGCGCTACCTTTAAGCATTGTTGCAGCCTTCATTGCAGCACAACCCTCTCTTCCTTGATCATGGCT
Ca_LG_5:36109771	CGAGGTTAAGGTTCTACATTTCAGTGAGGCACACACCCTTGGTGCTTTTGACATGCTTTTAGTGCCCCTTATTGATAACTGTAATTAAAAATCCCGTAGA	A/C	AGCTCAGCCACATAATGTAATATGAATAATTATAAGGGAAGTAAAATAGTCTGAATGTTGTTAAAAAGTTATCTGATCCGTGGGACTATGTTTTGCAACA
Ca_LG_5:37312384	ATGTAACAGGTATAAAACTTTGTGGCGCCCTGGGCGGCCAAAAAATGCCATGTAACTTTATTCTTTTGTGGCGGCATTTGCAACCACAAAATGCAAAGGC	C/A	TCAGGCTTTTGTGGCGGCCTTTGCAGCCACAAAATGTAATGTCCCTTCAGACATTTGTGGCGGCCTTTACCCTCACAAAATTTAGATTTTGTGGCTGTAA
Ca_LG_5:37524302	ATTGTGGAAGAAGTTGGGGAAGAATGTTGAACAGACTGTGACCGACAATGCAGCAAATTACAACGTTGCTGGTGCAATGTTAATGGAAAAAAGAAAGAAA	A/G	CTTTATTGGACACCATGTGCAGCACTTGTATTGATCTTATGATAAAAGAGGATTTTGAGAAAAAAACTAAAAGTTCATCGTGAAACAATTTCAAAGAGTA
Ca_LG_5:37524303	TTGTGGAAGAAGTTGGGGAAGAATGTTGAACAGACTGTGACCGACAATGCAGCAAATTACAACGTTGCTGGTGCAATGTTAATGGAAAAAAGAAAGAAAC	C/T	TTTATTGGACACCATGTGCAGCACTTGTATTGATCTTATGATAAAAGAGGATTTTGAGAAAAAAACTAAAAGTTCATCGTGAAACAATTTCAAAGAGTAA
Ca_LG_5:37524308	GAAGAAGTTGGGGAAGAATGTTGAACAGACTGTGACCGACAATGCAGCAAATTACAACGTTGCTGGTGCAATGTTAATGGAAAAAAGAAAGAAACTTTAT	T/C	TGGACACCATGTGCAGCACTTGTATTGATCTTATGATAAAAGAGGATTTTGAGAAAAAAACTAAAAGTTCATCGTGAAACAATTTCAAAGAGTAAGAAAA
Ca_LG_5:37524312	AAGTTGGGGAAGAATGTTGAACAGACTGTGACCGACAATGCAGCAAATTACAACGTTGCTGGTGCAATGTTAATGGAAAAAAGAAAGAAACTTTATTGGA	A/G	CACCATGTGCAGCACTTGTATTGATCTTATGATAAAAGAGGATTTTGAGAAAAAAACTAAAAGTTCATCGTGAAACAATTTCAAAGAGTAAGAAAATTAT
Ca_LG_5:37838689	GAACGTCGTTCCGTGGAATATATGACTCATGCTCCTTTAGGTTCTTTAAATCTCGTTGGCATAGCTACAGAGATTAATGCAGTCAATTATGTCTCTCCTG	G/A	GAAGTTGGTTAGCTACTTCTCATTTTGTTCTAGAATTCTTCCTATTTGTAGGTCATTTATGGAACACGAGAAGAGCTCACACAGCTGCCACAGAATTTGA

Positions	-100 bp flanking sequences	SNPs within *desi* cultivar	+100 bp flanking sequences
Ca_LG_5:37838723	CTTTAGGTTCTTTAAATCTCGTTGGCATAGCTACAGAGATTAATGCAGTCAATTATGTCTCTCCTGGAAGTTGGTTAGCTACTTCTCATTTTGTTCTAGA	A/G	ATTCTTCCTATTTGTAGGTCATTTATGGAACACGAGAAGAGCTCACACAGCTGCCACAGAATTTGAAAACGGAATTGATCGAGATTTTGAGACTGTCCTT
Ca_LG_5:37838736	AAATCTCGTTGGCATAGCTACAGAGATTAATGCAGTCAATTATGTCTCTCCTGGAAGTTGGTTAGCTACTTCTCATTTTGTTCTAGAATTCTTCCTATTT	T/C	GTAGGTCATTTATGGAACACGAGAAGAGCTCACACAGCTGCCACAGAATTTGAAAACGGAATTGATCGAGATTTTGAGACTGTCCTTTCCATGACTCCTC
Ca_LG_5:37838752	GCTACAGAGATTAATGCAGTCAATTATGTCTCTCCTGGAAGTTGGTTAGCTACTTCTCATTTTGTTCTAGAATTCTTCCTATTTGTAGGTCATTTATGGA	A/C	ACACGAGAAGAGCTCACACAGCTGCCACAGAATTTGAAAACGGAATTGATCGAGATTTTGAGACTGTCCTTTCCATGACTCCTCTTAATTGAGACATGAA
Ca_LG_5:37838755	ACAGAGATTAATGCAGTCAATTATGTCTCTCCTGGAAGTTGGTTAGCTACTTCTCATTTTGTTCTAGAATTCTTCCTATTTGTAGGTCATTTATGGAACA	A/G	CGAGAAGAGCTCACACAGCTGCCACAGAATTTGAAAACGGAATTGATCGAGATTTTGAGACTGTCCTTTCCATGACTCCTCTTAATTGAGACATGAAATC
Ca_LG_5:37838758	GAGATTAATGCAGTCAATTATGTCTCTCCTGGAAGTTGGTTAGCTACTTCTCATTTTGTTCTAGAATTCTTCCTATTTGTAGGTCATTTATGGAACACGA	A/G	GAAGAGCTCACACAGCTGCCACAGAATTTGAAAACGGAATTGATCGAGATTTTGAGACTGTCCTTTCCATGACTCCTCTTAATTGAGACATGAAATCCAA
Ca_LG_5:40489652	ATGGATATTAAACAAAGGGAATAATATTAGAGTTTGCATTTGGTTATGGTGACACAACAAAAATCAAACCTTAAATCATTGCAGCAACATACTACGTTCT	T/C	AAAAACCAAAATCCTAATAACAGAACTTATCTAGGAAGGTTTATATAAAATTACCTAATGCAGCAACCAATTTAGCAGGGAACTTTTGACCATGAGATAT
Ca_LG_5:40995033	TTTAAGTGGTGTTTTCAGTGGCAGACGAGTGAGTAACACGTAAGAACCTGCCCATATGAGGGGGACAACAGCTGGAAACAATTGCTAATACCCCATAGGT	T/C	TGAGGAGCAAAAGGAGGAATCTGCCCACGGAGGGGCTCACGTTTGATTAGCTAGTTGATGAGGTAATAGCTTACCAAGGCGATGATCAGTAGCTAGTTCG
Ca_LG_5:40995055	AGACGAGTGAGTAACACGTAAGAACCTGCCCATATGAGGGGGACAACAGCTGGAAACAATTGCTAATACCCCATAGGTTGAGGAGCAAAAGGAGGAATCT	T/C	GCCCACGGAGGGGCTCACGTTTGATTAGCTAGTTGATGAGGTAATAGCTTACCAAGGCGATGATCAGTAGCTAGTTCGAGAGGGTGATCAACCACACTGG
Ca_LG_5:40995072	GTAAGAACCTGCCCATATGAGGGGGACAACAGCTGGAAACAATTGCTAATACCCCATAGGTTGAGGAGCAAAAGGAGGAATCTGCCCACGGAGGGGCTCA	A/G	CGTTTGATTAGCTAGTTGATGAGGTAATAGCTTACCAAGGCGATGATCAGTAGCTAGTTCGAGAGGGTGATCAACCACACTGGGACGAGAGGATAATCAA
Ca_LG_5:40995076	GAACCTGCCCATATGAGGGGGACAACAGCTGGAAACAATTGCTAATACCCCATAGGTTGAGGAGCAAAAGGAGGAATCTGCCCACGGAGGGGCTCACGTT	T/C	TGATTAGCTAGTTGATGAGGTAATAGCTTACCAAGGCGATGATCAGTAGCTAGTTCGAGAGGGTGATCAACCACACTGGGACGAGAGGATAATCAATAGT
Ca_LG_5:40995091	AGGGGGACAACAGCTGGAAACAATTGCTAATACCCCATAGGTTGAGGAGCAAAAGGAGGAATCTGCCCACGGAGGGGCTCACGTTTGATTAGCTAGTTGA	A/G	TGAGGTAATAGCTTACCAAGGCGATGATCAGTAGCTAGTTCGAGAGGGTGATCAACCACACTGGGACGAGAGGATAATCAATAGTTCGCTTCTTTACTGA
Ca_LG_5:40995128	TAGGTTGAGGAGCAAAAGGAGGAATCTGCCCACGGAGGGGCTCACGTTTGATTAGCTAGTTGATGAGGTAATAGCTTACCAAGGCGATGATCAGTAGCTA	A/G	GTTCGAGAGGGTGATCAACCACACTGGGACGAGAGGATAATCAATAGTTCGCTTCTTTACTGAGGATGATCGATAATGGGAAAAAGCCTGACAGAGCAAT

**Positions**	**-100 bp flanking sequences**	**SNPs within *desi* cultivar**	**+100 bp flanking sequences**
Ca_LG_5:40995131	GTTGAGGAGCAAAAGGAGGAATCTGCCCACGGAGGGGCTCACGTTTGATTAGCTAGTTGATGAGGTAATAGCTTACCAAGGCGATGATCAGTAGCTAGTT	T/C	CGAGAGGGTGATCAACCACACTGGGACGAGAGGATAATCAATAGTTCGCTTCTTTACTGAGGATGATCGATAATGGGAAAAAGCCTGACAGAGCAATGTC
Ca_LG_5:40995139	GCAAAAGGAGGAATCTGCCCACGGAGGGGCTCACGTTTGATTAGCTAGTTGATGAGGTAATAGCTTACCAAGGCGATGATCAGTAGCTAGTTCGAGAGGG	G/A	TGATCAACCACACTGGGACGAGAGGATAATCAATAGTTCGCTTCTTTACTGAGGATGATCGATAATGGGAAAAAGCCTGACAGAGCAATGTCGCGTGGAG
Ca_LG_5:40995221	GTAGCTAGTTCGAGAGGGTGATCAACCACACTGGGACGAGAGGATAATCAATAGTTCGCTTCTTTACTGAGGATGATCGATAATGGGAAAAAGCCTGACA	A/G	GAGCAATGTCGCGTGGAGGAAGAAGGCATACGGGTCGTCAACTTCTTTCCCCGGAGAAGAAACAATGACGATATATAGGAAATAAGCAATGACTAACTCT
Ca_LG_5:40995230	TCGAGAGGGTGATCAACCACACTGGGACGAGAGGATAATCAATAGTTCGCTTCTTTACTGAGGATGATCGATAATGGGAAAAAGCCTGACAGAGCAATGT	T/C	CGCGTGGAGGAAGAAGGCATACGGGTCGTCAACTTCTTTCCCCGGAGAAGAAACAATGACGATATATAGGAAATAAGCAATGACTAACTCTATGCAAGCA
Ca_LG_5:40995249	CACTGGGACGAGAGGATAATCAATAGTTCGCTTCTTTACTGAGGATGATCGATAATGGGAAAAAGCCTGACAGAGCAATGTCGCGTGGAGGAAGAAGGCA	A/C	TACGGGTCGTCAACTTCTTTCCCCGGAGAAGAAACAATGACGATATATAGGAAATAAGCAATGACTAACTCTATGCAAGCAGTCGCGATAAGATAGAGGA
Ca_LG_5:40995270	AATAGTTCGCTTCTTTACTGAGGATGATCGATAATGGGAAAAAGCCTGACAGAGCAATGTCGCGTGGAGGAAGAAGGCATACGGGTCGTCAACTTCTTTC	C/T	CCCGGAGAAGAAACAATGACGATATATAGGAAATAAGCAATGACTAACTCTATGCAAGCAGTCGCGATAAGATAGAGGATGCAAACATTATCCATAAATA
Ca_LG_5:40995273	AGTTCGCTTCTTTACTGAGGATGATCGATAATGGGAAAAAGCCTGACAGAGCAATGTCGCGTGGAGGAAGAAGGCATACGGGTCGTCAACTTCTTTCCCC	C/T	GGAGAAGAAACAATGACGATATATAGGAAATAAGCAATGACTAACTCTATGCAAGCAGTCGCGATAAGATAGAGGATGCAAACATTATCCATAAATAATT
Ca_LG_5:40995292	GATGATCGATAATGGGAAAAAGCCTGACAGAGCAATGTCGCGTGGAGGAAGAAGGCATACGGGTCGTCAACTTCTTTCCCCGGAGAAGAAACAATGACGA	A/G	TATATAGGAAATAAGCAATGACTAACTCTATGCAAGCAGTCGCGATAAGATAGAGGATGCAAACATTATCCATAAATAATTGGGCGTAAAGCGTTTGTAG
Ca_LG_5:40995296	ATCGATAATGGGAAAAAGCCTGACAGAGCAATGTCGCGTGGAGGAAGAAGGCATACGGGTCGTCAACTTCTTTCCCCGGAGAAGAAACAATGACGATATA	A/C	TAGGAAATAAGCAATGACTAACTCTATGCAAGCAGTCGCGATAAGATAGAGGATGCAAACATTATCCATAAATAATTGGGCGTAAAGCGTTTGTAGGTTG
Ca_LG_5:40995298	CGATAATGGGAAAAAGCCTGACAGAGCAATGTCGCGTGGAGGAAGAAGGCATACGGGTCGTCAACTTCTTTCCCCGGAGAAGAAACAATGACGATATATA	A/G	GGAAATAAGCAATGACTAACTCTATGCAAGCAGTCGCGATAAGATAGAGGATGCAAACATTATCCATAAATAATTGGGCGTAAAGCGTTTGTAGGTTGTC
Ca_LG_5:40995301	TAATGGGAAAAAGCCTGACAGAGCAATGTCGCGTGGAGGAAGAAGGCATACGGGTCGTCAACTTCTTTCCCCGGAGAAGAAACAATGACGATATATAGGA	A/G	AATAAGCAATGACTAACTCTATGCAAGCAGTCGCGATAAGATAGAGGATGCAAACATTATCCATAAATAATTGGGCGTAAAGCGTTTGTAGGTTGTCTTT
Ca_LG_5:41083113	ATGACACTAGGATGACAATGGGCTTCCATCAAGTGGAATGGTTTACTAATGCTGCTGAACAACTACTGATCTTGAATGAGTTGGTTGTTGCACTAGCATG	G/T	CACACTGTTGCCTTGGCTTCCCATGGCTCAAATCTTAAAGGCACACTCGGTTTATAGGGCAGCAAATTTGATTGTACAGACTTTGATCACTTGGGATGTT

Positions	-100 bp flanking sequences	SNPs within *desi* cultivar	+100 bp flanking sequences
Ca_LG_5:41083122	GGATGACAATGGGCTTCCATCAAGTGGAATGGTTTACTAATGCTGCTGAACAACTACTGATCTTGAATGAGTTGGTTGTTGCACTAGCATGCACACTGTT	T/A	GCCTTGGCTTCCCATGGCTCAAATCTTAAAGGCACACTCGGTTTATAGGGCAGCAAATTTGATTGTACAGACTTTGATCACTTGGGATGTTGAAGTAGAT
Ca_LG_5:42374509	TTAGCAGCAAGTGAAAGAGATGCAACAGCAGCAAGGTGAGTCATCCATGGCTTCTCATTCTGAAAACGAAAACTGAACAGAAACCTATCAAGGTAGTTAA	A/G	CAGCAAGAACAGCTGTTAGAGCAGAAAAAGAGTAATAAACTTTCACTTTCATCATCCATTCAATCGATTCTCTTCGATCACTTTCCAAAACCGAGTTTGT
Ca_LG_5:42552273	TTTGGGGCATTCTTTTCCTGATTTGGTATGTTTTTGGGGAAGTTTTTGGTGTGGTTGGCTGCGTTCCTCCCTCCTTGGTTACAAATGCTTGCTGAATGCG	G/A	CCAAGTGTCCAATGTCTCTTCACACTTTGCTGCTGCTTTCTCTTTTTTCGCTTTTCAATTTCAACACACACCACTTAGGACCACTTAACATACATAACAC
Ca_LG_5:43909988	TTTCTTTTTCAACTTTCAACCTCTTGTTACTTCTTTCAATAAACTGACTTTTACAAGTGTTTTCTGATCGTAAGTTCTCATTCTCTTGGGTGACTTTTTC	C/A	TAACTTTGCTTGCAGCTCTTTTTTCTCCTCTTCAGATTTTAGCAATGATGCTTTGAGTTCTTCTATCTCTTCATTTGCAACAAGAATTGGTTCAGACATT
Ca_LG_5:44874037	CTACCCTTGGGAGAGGGACAACAGTTGGAAACGGTTGCTAATACCCATAGGCTGAGGAGCAAAAGGAGGAACCTGCCCAAGGAGGGTTTGCGTCTGATTT	T/A	GCTAGTTGGTGAGGTAATAGCTTACCAAGGCGATGGTCAATAGCTGGTCCGAGAGGATGATCAGCCACACCGGGACTAGGACAAGGTCCAAATTCCTACG
Ca_LG_5:44874073	GCTAATACCCATAGGCTGAGGAGCAAAAGGAGGAACCTGCCCAAGGAGGGTTTGCGTCTGATTTGCTAGTTGGTGAGGTAATAGCTTACCAAGGCGATGG	G/A	TCAATAGCTGGTCCGAGAGGATGATCAGCCACACCGGGACTAGGACAAGGTCCAAATTCCTACGGGAGGAATCCAAATGATACTCTGTTTTGTTTGTTTT
Ca_LG_5:44874077	ATACCCATAGGCTGAGGAGCAAAAGGAGGAACCTGCCCAAGGAGGGTTTGCGTCTGATTTGCTAGTTGGTGAGGTAATAGCTTACCAAGGCGATGGTCAA	A/G	TAGCTGGTCCGAGAGGATGATCAGCCACACCGGGACTAGGACAAGGTCCAAATTCCTACGGGAGGAATCCAAATGATACTCTGTTTTGTTTGTTTTGATT
Ca_LG_5:44874108	CCTGCCCAAGGAGGGTTTGCGTCTGATTTGCTAGTTGGTGAGGTAATAGCTTACCAAGGCGATGGTCAATAGCTGGTCCGAGAGGATGATCAGCCACACC	C/T	GGGACTAGGACAAGGTCCAAATTCCTACGGGAGGAATCCAAATGATACTCTGTTTTGTTTGTTTTGATTGAGTATTATCTTATCATAGGTTACTTAAGTA
Ca_LG_5:44874115	AAGGAGGGTTTGCGTCTGATTTGCTAGTTGGTGAGGTAATAGCTTACCAAGGCGATGGTCAATAGCTGGTCCGAGAGGATGATCAGCCACACCGGGACTA	A/G	GGACAAGGTCCAAATTCCTACGGGAGGAATCCAAATGATACTCTGTTTTGTTTGTTTTGATTGAGTATTATCTTATCATAGGTTACTTAAGTATCTTCTG
Ca_LG_5:44874116	AGGAGGGTTTGCGTCTGATTTGCTAGTTGGTGAGGTAATAGCTTACCAAGGCGATGGTCAATAGCTGGTCCGAGAGGATGATCAGCCACACCGGGACTAG	G/A	GACAAGGTCCAAATTCCTACGGGAGGAATCCAAATGATACTCTGTTTTGTTTGTTTTGATTGAGTATTATCTTATCATAGGTTACTTAAGTATCTTCTGC
Ca_LG_5:44874124	TTGCGTCTGATTTGCTAGTTGGTGAGGTAATAGCTTACCAAGGCGATGGTCAATAGCTGGTCCGAGAGGATGATCAGCCACACCGGGACTAGGACAAGGT	T/C	CCAAATTCCTACGGGAGGAATCCAAATGATACTCTGTTTTGTTTGTTTTGATTGAGTATTATCTTATCATAGGTTACTTAAGTATCTTCTGCCTGTTGGA
Ca_LG_5:44874128	GTCTGATTTGCTAGTTGGTGAGGTAATAGCTTACCAAGGCGATGGTCAATAGCTGGTCCGAGAGGATGATCAGCCACACCGGGACTAGGACAAGGTCCAA	A/G	ATTCCTACGGGAGGAATCCAAATGATACTCTGTTTTGTTTGTTTTGATTGAGTATTATCTTATCATAGGTTACTTAAGTATCTTCTGCCTGTTGGAGACT

Positions	-100 bp flanking sequences	SNPs within *desi* cultivar	+100 bp flanking sequences
Ca_LG_5:44874130	CTGATTTGCTAGTTGGTGAGGTAATAGCTTACCAAGGCGATGGTCAATAGCTGGTCCGAGAGGATGATCAGCCACACCGGGACTAGGACAAGGTCCAAAT	T/C	TCCTACGGGAGGAATCCAAATGATACTCTGTTTTGTTTGTTTTGATTGAGTATTATCTTATCATAGGTTACTTAAGTATCTTCTGCCTGTTGGAGACTAA
Ca_LG_5:45109641	AAACATCAAGAGATTGTTCTACCCAAACACCGTTGACGAAAGACAAGCGCGGTCCTCCACCAGGTGAGAGTATAAGGAGTTAAGATTAGCGGTGGATTTT	T/G	GATCGGAGGAAGGAGAGAAGCTGTTGTTGTGTGGGACCCTCAGAACCAGCGGTGATGATGCTTAGCACAGTTTGGAGCGACAATGGCGAAAACACAATAT
Ca_LG_5:45109645	ATCAAGAGATTGTTCTACCCAAACACCGTTGACGAAAGACAAGCGCGGTCCTCCACCAGGTGAGAGTATAAGGAGTTAAGATTAGCGGTGGATTTTGATC	C/T	GGAGGAAGGAGAGAAGCTGTTGTTGTGTGGGACCCTCAGAACCAGCGGTGATGATGCTTAGCACAGTTTGGAGCGACAATGGCGAAAACACAATATTCTG
Ca_LG_5:45109665	AAACACCGTTGACGAAAGACAAGCGCGGTCCTCCACCAGGTGAGAGTATAAGGAGTTAAGATTAGCGGTGGATTTTGATCGGAGGAAGGAGAGAAGCTGT	T/A	TGTTGTGTGGGACCCTCAGAACCAGCGGTGATGATGCTTAGCACAGTTTGGAGCGACAATGGCGAAAACACAATATTCTGTCTTTGAAATTTTTCTTTGG
Ca_LG_5:45342576	TATGGTTGATTTATTTCTGCAGTACTTAATGGAGTTCAATAGCAGCCCTGGCGATCTGATGGAGCTTTCCCCTCTTTTTTCTGATGATAGCCGTGTTGCT	T/C	GAGGCTGCTTCAATTGCACAAAAATTGCGTAAGAATTAAGATGTCTTTTAAATGTTTTATAATGTTTTGGCCTAATTATGTCAGTGCTGTTCCAATTCTG
Ca_LG_5:45346468	GACTGAAGCAGCTAATTTGAGGGAAAGATTTGTGCTGGGTACAAGTGTTAGCCGTAGGGTTGCTGCTGCGGCCGCATCTGCTGTAATTAAATGCTTTTTT	T/C	CGCATGCCGTTGTTTAAGTATTGTCTAGTATGAAATTAAAGGACATTTAACCAAACATAATGAAGATTTACTTGTTGATATGCAGGCGGAGGCCGCGGCT
Ca_LG_5:45389039	CGAGGAGAGATTGTTACACTTGCCCAGGTACCGTGGTTTTATTGTATATGTTTGGATCATGTCATTCATTGAAAAGGTTTTCCTGTCTTTGCAAATTACA	A/C	AATAGAAACATTTTATATCTTACACTTAAAAAATAACAAAAAACACATGCCTTATTGCCCTTCTAACATTATAGTGCTGCTTTGACTAAAATATATAGCT
Ca_LG_5:45389093	GATCATGTCATTCATTGAAAAGGTTTTCCTGTCTTTGCAAATTACAAATAGAAACATTTTATATCTTACACTTAAAAAATAACAAAAAACACATGCCTTA	A/T	TTGCCCTTCTAACATTATAGTGCTGCTTTGACTAAAATATATAGCTTGAAAGTTGAATTTGTCTTTGCTGTATAAAGTTTGAAATGGACTTTTTTCAGTA
Ca_LG_5:45389096	CATGTCATTCATTGAAAAGGTTTTCCTGTCTTTGCAAATTACAAATAGAAACATTTTATATCTTACACTTAAAAAATAACAAAAAACACATGCCTTATTG	G/T	CCCTTCTAACATTATAGTGCTGCTTTGACTAAAATATATAGCTTGAAAGTTGAATTTGTCTTTGCTGTATAAAGTTTGAAATGGACTTTTTTCAGTATAT
Ca_LG_5:45569112	TAAATCACATTCACATAACTCTAACAACCAAATTTCATGACTCTTTTTTTGAAAAATTGAAGCAACAAAAGTCCAACGGCCAACCCAGCAACAATAACAA	A/G	AAGCAACATAAATGATTGGATTCATGATACATTTCTCTTATTCCAAATAAAATAAAGAAAATAAATAGTTAATAATATAATCAACCAAGAAATTATGACT
Ca_LG_5:45569118	ACATTCACATAACTCTAACAACCAAATTTCATGACTCTTTTTTTGAAAAATTGAAGCAACAAAAGTCCAACGGCCAACCCAGCAACAATAACAAAAGCAA	A/G	CATAAATGATTGGATTCATGATACATTTCTCTTATTCCAAATAAAATAAAGAAAATAAATAGTTAATAATATAATCAACCAAGAAATTATGACTTAAATT
Ca_LG_5:45569121	TTCACATAACTCTAACAACCAAATTTCATGACTCTTTTTTTGAAAAATTGAAGCAACAAAAGTCCAACGGCCAACCCAGCAACAATAACAAAAGCAACAT	T/G	AAATGATTGGATTCATGATACATTTCTCTTATTCCAAATAAAATAAAGAAAATAAATAGTTAATAATATAATCAACCAAGAAATTATGACTTAAATTATT

Positions	-100 bp flanking sequences	SNPs within *desi* cultivar	+100 bp flanking sequences
Ca_LG_5:45569142	AATTTCATGACTCTTTTTTTGAAAAATTGAAGCAACAAAAGTCCAACGGCCAACCCAGCAACAATAACAAAAGCAACATAAATGATTGGATTCATGATAC	C/A	ATTTCTCTTATTCCAAATAAAATAAAGAAAATAAATAGTTAATAATATAATCAACCAAGAAATTATGACTTAAATTATTTCATTCTTCATATTTATCCGT
Ca_LG_5:45569143	ATTTCATGACTCTTTTTTTGAAAAATTGAAGCAACAAAAGTCCAACGGCCAACCCAGCAACAATAACAAAAGCAACATAAATGATTGGATTCATGATACA	A/G	TTTCTCTTATTCCAAATAAAATAAAGAAAATAAATAGTTAATAATATAATCAACCAAGAAATTATGACTTAAATTATTTCATTCTTCATATTTATCCGTT
Ca_LG_5:45569150	GACTCTTTTTTTGAAAAATTGAAGCAACAAAAGTCCAACGGCCAACCCAGCAACAATAACAAAAGCAACATAAATGATTGGATTCATGATACATTTCTCT	T/C	TATTCCAAATAAAATAAAGAAAATAAATAGTTAATAATATAATCAACCAAGAAATTATGACTTAAATTATTTCATTCTTCATATTTATCCGTTTTTTTTT
Ca_LG_5:45569174	CAACAAAAGTCCAACGGCCAACCCAGCAACAATAACAAAAGCAACATAAATGATTGGATTCATGATACATTTCTCTTATTCCAAATAAAATAAAGAAAAT	T/G	AAATAGTTAATAATATAATCAACCAAGAAATTATGACTTAAATTATTTCATTCTTCATATTTATCCGTTTTTTTTTCCAGACATCACATGACACAAGAAA
Ca_LG_5:45818915	TCTCTTTCCTCTATTTTTTGTTTATATTTTCTTTTTCTTTTTTGGTTCCACTCTGTGCTAGGTGACCTTCAGACTTACTCTGCTTATGGTTTGGCCTTAA	A/G	AAACGAGCTTACCAGATGTCGCTCTATGGTGTGATGTGCAATTAGGGGTGGTAATAGGTCAGACTTTGAAAGGTCTGAGTCTGGCATACGGATTTCAGTC
Ca_LG_5:45818929	TTTTTGTTTATATTTTCTTTTTCTTTTTTGGTTCCACTCTGTGCTAGGTGACCTTCAGACTTACTCTGCTTATGGTTTGGCCTTAAAAACGAGCTTACCA	A/G	GATGTCGCTCTATGGTGTGATGTGCAATTAGGGGTGGTAATAGGTCAGACTTTGAAAGGTCTGAGTCTGGCATACGGATTTCAGTCGATTTTTGTCGGGT
Ca_LG_5:45818930	TTTTGTTTATATTTTCTTTTTCTTTTTTGGTTCCACTCTGTGCTAGGTGACCTTCAGACTTACTCTGCTTATGGTTTGGCCTTAAAAACGAGCTTACCAG	G/A	ATGTCGCTCTATGGTGTGATGTGCAATTAGGGGTGGTAATAGGTCAGACTTTGAAAGGTCTGAGTCTGGCATACGGATTTCAGTCGATTTTTGTCGGGTG
Ca_LG_6:515885	TATACACCTATTGTAGGATGAGAATGAAGTGTCTAAAATTAACATCAGAATTTTCTCACTCAGCGTCGTGCTGCAACCTGGTGCTTACACACAGGGCAGA	A/C	AATTTTTGTGTGCAACCCATTGTTGTATACATTGAAAGTGATACAAGTGATCACAGTTAAGCCTCCCAAGTTTATCATCCACTTCATACTCTTCCTGTAC
Ca_LG_6:657055	TTATATATATAATAAAATATTGTTTAAATATGGCTTAATTTATGTTTAGTTATTGTAAATGAGAAAGTCAAATCCGTCTTGCAGCTGCAGCTACACCCATCC	C/A	GACATAAACACAATTACTTAAATACTCTTCTATATCTTAAAATAATAATAAAACCTTAATTTTGTCAATCTTTCTTCTTTTTCCTTGCTGCCACCGTCCA
Ca_LG_6:657056	TATATATAATAAAATATTGTTTAAATATGGCTTAATTTATGTTTAGTTATTGTAAATGAGAAAGTCAAATCCGTCTTGCAGCTGCAGCTACACCCATCCG	G/A	ACATAAACACAATTACTTAAATACTCTTCTATATCTTAAAATAATAATAAAACCTTAATTTTGTCAATCTTTCTTCTTTTTCCTTGCTGCCACCGTCCAC
Ca_LG_6:1187030	TTTTTGCCATTGAACTTCCCAATCAAACTCCTCGTAACACTCATAATTATCAACTTTACGTAATACAAAACTTCTCTATAGTTTAGAATGAAGTTCTTCC	C/A	CGAAGTTTGAGACAAAGTAATACAAAACTTCTCTATAGTTTAGAGCTTGGGTACCGGTAAGAAAGGAATCCATAAAAACGTGATCCAACACCATGTCCCA
Ca_LG_6:1187038	ATTGAACTTCCCAATCAAACTCCTCGTAACACTCATAATTATCAACTTTACGTAATACAAAACTTCTCTATAGTTTAGAATGAAGTTCTTCCCGAAGTTT	T/C	GAGACAAAGTAATACAAAACTTCTCTATAGTTTAGAGCTTGGGTACCGGTAAGAAAGGAATCCATAAAAACGTGATCCAACACCATGTCCCAAGATTTGA

Positions	-100 bp flanking sequences	SNPs within *desi* cultivar	+100 bp flanking sequences
Ca_LG_6:2677753	TAAGCATGTGTGTGCTTTGAATATTCAATTTAAGCCATATTCTATGCATACAACTATACTTTCTTGTATTTCTTTTTTTCATTCATTCAGGGAACAGCCC	C/T	GGGATTATGAGCGAGCTGCTGAGGCTTACATGCATGCTAAAGCGCAATCTAATGCACAAGCCATGTTCAATCTTGGATACATGCATGAGCATGGCCAAGG
Ca_LG_6:3060633	GGTGATATTTGATATATTTTGTTTGGATGTTTCGCATTTGCTGCTATACTATATCGTGAAAGTACATAACTCAATTTATGATATGTTTTGTATTTGTAGA	A/G	AGTTTGTGCGATGTGTGGCAAGCAAATTCTTGACACCAAGTTATATAAACAACGCATTGTGTAGTGCATTTGTCCTGCATTGTGGTTTTTCAGGGACCTC
Ca_LG_6:3060659	ATGTTTCGCATTTGCTGCTATACTATATCGTGAAAGTACATAACTCAATTTATGATATGTTTTGTATTTGTAGAAGTTTGTGCGATGTGTGGCAAGCAAA	A/G	TTCTTGACACCAAGTTATATAAACAACGCATTGTGTAGTGCATTTGTCCTGCATTGTGGTTTTTCAGGGACCTCTGAAAACAAAGGAGGTGGGATGGGAA
Ca_LG_6:3060667	CATTTGCTGCTATACTATATCGTGAAAGTACATAACTCAATTTATGATATGTTTTGTATTTGTAGAAGTTTGTGCGATGTGTGGCAAGCAAATTCTTGAC	C/T	ACCAAGTTATATAAACAACGCATTGTGTAGTGCATTTGTCCTGCATTGTGGTTTTTCAGGGACCTCTGAAAACAAAGGAGGTGGGATGGGAAATTTCCAA
Ca_LG_6:3060676	CTATACTATATCGTGAAAGTACATAACTCAATTTATGATATGTTTTGTATTTGTAGAAGTTTGTGCGATGTGTGGCAAGCAAATTCTTGACACCAAGTTA	A/T	TATAAACAACGCATTGTGTAGTGCATTTGTCCTGCATTGTGGTTTTTCAGGGACCTCTGAAAACAAAGGAGGTGGGATGGGAAATTTCCAACCATTTTTT
Ca_LG_6:3060686	TCGTGAAAGTACATAACTCAATTTATGATATGTTTTGTATTTGTAGAAGTTTGTGCGATGTGTGGCAAGCAAATTCTTGACACCAAGTTATATAAACAAC	C/A	GCATTGTGTAGTGCATTTGTCCTGCATTGTGGTTTTTCAGGGACCTCTGAAAACAAAGGAGGTGGGATGGGAAATTTCCAACCATTTTTTGTGCTCAACC
Ca_LG_6:3060690	GAAAGTACATAACTCAATTTATGATATGTTTTGTATTTGTAGAAGTTTGTGCGATGTGTGGCAAGCAAATTCTTGACACCAAGTTATATAAACAACGCAT	T/A	TGTGTAGTGCATTTGTCCTGCATTGTGGTTTTTCAGGGACCTCTGAAAACAAAGGAGGTGGGATGGGAAATTTCCAACCATTTTTTGTGCTCAACCTAGA
Ca_LG_6:3491941	CAGAGAAGGAACTTACTAGTCCACCTTTGGATATGTTCCCGCCAAATAAGAGCAGCATGGAATCAGATATGGCCGTTGAATCCCTGAGGAATACTGTATT	T/G	CACTTTAACCTTTTCATTAAACACTAACCATGGGTATGGAATTTTGGGTACCGCAGCATTTACTGAGTTCTACACGTGCAGCACCAAAATTTTAGAACTT
Ca_LG_6:3558824	ATCTTCACCAGCTGTCAACACCCCAAATACTTATTTCCTCATTAGGTCTGGATAGGCCCACCTCGAATCAAAGACTATATTTGCTGCCAATAAGAAATAG	G/T	GAGTAATTTATTTTATACAGTTTGCTCTGTGCCATATCGGGTTATGTATCTTTGGGCTTTCCCTTTTTGGGTTTAGTTCGATTCTTCACCTTCATTGCCA
Ca_LG_6:3558868	GGTCTGGATAGGCCCACCTCGAATCAAAGACTATATTTGCTGCCAATAAGAAATAGGAGTAATTTATTTTATACAGTTTGCTCTGTGCCATATCGGGTTA	A/G	TGTATCTTTGGGCTTTCCCTTTTTGGGTTTAGTTCGATTCTTCACCTTCATTGCCAATGCTCTCTCTCGAATATTATACAGTTCGGAATAAGCCGCAAAA
Ca_LG_6:3567354	CCCCGTAACACATGCTTGGTATTATACCAGAGAGTCCCACATTGTTAGTTCACAAATACAACTTAGCAGGTGTCAGATATAAAAGCAGCAGCGAGTCCAT	T/A	CTTACAATTCATACCTTTCTCGGCCTTTTGGCTAAGATCAAGTGTAGTATCTGTTCTTATCAGTTTAATATCTGATATGTGGTCCAATGGGCCACACGAT
Ca_LG_6:4971056	GTAATTTGGGAATATTAATTTTTTAAAACATTTGAGGGGTGTGGGTAGTAAATAAGGGGGTGTTGGTAGCAATATTGTAAGTCTTTTCTTTTCTCATCAC	C/A	GAAAAGTCTTTCTCAAAGATTCTAAGATTCTATAGACATGGATATATTATATATCAAAGTATATGATATATGAAATTGAGATAGTTACCTTTCAGAAAAT

Positions	-100 bp flanking sequences	SNPs within *desi* cultivar	+100 bp flanking sequences
Ca_LG_6:4971063	GGGAATATTAATTTTTTAAAACATTTGAGGGGTGTGGGTAGTAAATAAGGGGGTGTTGGTAGCAATATTGTAAGTCTTTTCTTTTCTCATCACGAAAAGT	T/C	CTTTCTCAAAGATTCTAAGATTCTATAGACATGGATATATTATATATCAAAGTATATGATATATGAAATTGAGATAGTTACCTTTCAGAAAATTCCAATA
Ca_LG_6:5093190	GTGAGCAAATTCAATTGTAACCTGAAGAGTTAAATAAAAGCAATGAAGATAAAGCAGCATTCCGCAAATTTATATCACTCACTGGAACACAAGTAATCAA	A/G	CTGAAACTCATTCTGGCAAAGTGGGCAGAGATTAGTGATGGTTGCCCAGTTGTCAATGCAGGCAAAGCAGAACCTGTGAAACAAATGAACCATGCTAAAA
Ca_LG_6:5841972	AAGAGGAGTGATATTTATAGAAAAATCTTGACCTTTGTGGCGGCCAAGCAGCCACAAAATCCAACGGTAACTAAAGCATTTGTTGCTGCCTAAGCAGCCA	A/C	CAACGGGCAACGTGTAATTCGGCATTTGTGGCAGCCTTTGCAGCCACAAAGTGTAACGGTCCAGTGTTTTTGTGGCAGCTTGGGCGGTCATAAATGGTTT
Ca_LG_6:5841978	AGTGATATTTATAGAAAAATCTTGACCTTTGTGGCGGCCAAGCAGCCACAAAATCCAACGGTAACTAAAGCATTTGTTGCTGCCTAAGCAGCCACAACGG	G/C	GCAACGTGTAATTCGGCATTTGTGGCAGCCTTTGCAGCCACAAAGTGTAACGGTCCAGTGTTTTTGTGGCAGCTTGGGCGGTCATAAATGGTTTGCCTTT
Ca_LG_6:7118099	TGTGGTTGCCAAATTTCATACAGTTCAAATTTGATAGCTTACATTACATGTATATGGATTGCAGGCTCAACAGTTGAACAAGGACATTTTTGAGACTATA	A/T	TACTATCATGCTCTGAAAACTTCTTGTGAATTGGCTGCAAAGGAAGGACCCTATGAAACATATAGTGGTAGTCCTGTAAGCAAGGTATTTTCATCTGAGA
Ca_LG_6:7118123	TCAAATTTGATAGCTTACATTACATGTATATGGATTGCAGGCTCAACAGTTGAACAAGGACATTTTTGAGACTATATACTATCATGCTCTGAAAACTTCT	T/A	TGTGAATTGGCTGCAAAGGAAGGACCCTATGAAACATATAGTGGTAGTCCTGTAAGCAAGGTATTTTCATCTGAGAGATATTTTTTTTCTGTCACTTCTT
Ca_LG_6:7118129	TTGATAGCTTACATTACATGTATATGGATTGCAGGCTCAACAGTTGAACAAGGACATTTTTGAGACTATATACTATCATGCTCTGAAAACTTCTTGTGAA	A/G	TTGGCTGCAAAGGAAGGACCCTATGAAACATATAGTGGTAGTCCTGTAAGCAAGGTATTTTCATCTGAGAGATATTTTTTTTCTGTCACTTCTTCTATGT
Ca_LG_6:7124530	AAGAAAATACAGAGGAGTGAGACAGAGACCATGGGGAAAATATGCTGCAGAGATAAGAGATCCTTTTAAAGCAACAAGAGTTTGGTTAGGAACATTTGAA	A/T	ACAGCTGAAGATGCAGCTATAGCTTATGATCAAGCTTCATTGCGTTTTAGAGGAAACAAAGCAAAACTCAATTTTCCTGAAAATGTTAGCCTCAAGCAAC
Ca_LG_6:7124532	GAAAATACAGAGGAGTGAGACAGAGACCATGGGGAAAATATGCTGCAGAGATAAGAGATCCTTTTAAAGCAACAAGAGTTTGGTTAGGAACATTTGAAAC	C/A	AGCTGAAGATGCAGCTATAGCTTATGATCAAGCTTCATTGCGTTTTAGAGGAAACAAAGCAAAACTCAATTTTCCTGAAAATGTTAGCCTCAAGCAACAA
Ca_LG_6:7124533	AAAATACAGAGGAGTGAGACAGAGACCATGGGGAAAATATGCTGCAGAGATAAGAGATCCTTTTAAAGCAACAAGAGTTTGGTTAGGAACATTTGAAACA	A/T	GCTGAAGATGCAGCTATAGCTTATGATCAAGCTTCATTGCGTTTTAGAGGAAACAAAGCAAAACTCAATTTTCCTGAAAATGTTAGCCTCAAGCAACAAC
Ca_LG_6:7170960	TCTTTATATTCTTTAGAGCATTAGGATGGCCTTCAGTTTTGGGAGATATTCTCTTGCTAGAGAGAAAAACTGGAGATATAGATGCATTCATGCTCCGTTC	C/T	GCTAGTAAATATTGATGATGATTTGCTGCTGCTTCCACTATTTCCCGTTTCTGATCCTAGCATGTTTTGTCCTATACTCTTCTCCTGTCAAAACCAAAGG
Ca_LG_6:8076698	ATCTCAAAACTTTATCTTCATGTTCTTTAATCACGTATCCCATTGGCAGCTCAACATAGACCTCTTCTTCAAGATAGCCATTTAGAAATGTCGATTTTAC	C/T	ATCAAGTTGATAAAGTTTCCACTTATTTTGAGCTTCTATAGAGATTAGCAAATGAATAGTGTCCATGCGATCAACTGGTGCAAAAACTTCTTCATAGTCA

Positions	-100 bp flanking sequences	SNPs within *desi* cultivar	+100 bp flanking sequences
Ca_LG_6:8076700	CTCAAAACTTTATCTTCATGTTCTTTAATCACGTATCCCATTGGCAGCTCAACATAGACCTCTTCTTCAAGATAGCCATTTAGAAATGTCGATTTTACAT	T/G	CAAGTTGATAAAGTTTCCACTTATTTTGAGCTTCTATAGAGATTAGCAAATGAATAGTGTCCATGCGATCAACTGGTGCAAAAACTTCTTCATAGTCAAC
Ca_LG_6:8076713	CTTCATGTTCTTTAATCACGTATCCCATTGGCAGCTCAACATAGACCTCTTCTTCAAGATAGCCATTTAGAAATGTCGATTTTACATCAAGTTGATAAAG	G/T	TTTCCACTTATTTTGAGCTTCTATAGAGATTAGCAAATGAATAGTGTCCATGCGATCAACTGGTGCAAAAACTTCTTCATAGTCAACTCCATGTTTTTGT
Ca_LG_6:8086321	TATTGCTTTTGATATTCTTTTTCATTTTCAGCAATGTGTAGGAATAGGATTAGTTGCTTGAACTTTGAATATGATGAAAATATTTAGGTTGTAATATAGT	T/C	TTTAAATTGTTGTCTGCAGCAGTTATTGCGGATTTTGAAGTATCTCGTTGGTATTGCGACCACAATTTAGCCCAACTATATTTTCCCGCAATATAAAGGT
Ca_LG_6:8147968	TATCTCAACCTCAATATTTTGGTGCTTTAGGAAGTTTTAGAGATAGCCAGCGAGGTGAAATAGGATGCAGTTACACGGATAATATTTTTTAGCACCCTCA	A/T	TCTGGAGCCATACTCATCTACTTGTCTTGAATTTGAATTGCTGCTTTTAGCCGTTTACCGTCTCTTGCTACCAAAACCATCTCCATTCCAAGATTTTTAT
Ca_LG_6:8147975	ACCTCAATATTTTGGTGCTTTAGGAAGTTTTAGAGATAGCCAGCGAGGTGAAATAGGATGCAGTTACACGGATAATATTTTTTAGCACCCTCATCTGGAG	G/C	CCATACTCATCTACTTGTCTTGAATTTGAATTGCTGCTTTTAGCCGTTTACCGTCTCTTGCTACCAAAACCATCTCCATTCCAAGATTTTTATTATTATA
Ca_LG_6:8153146	GATGGAAGGCAGCAGTAGACGGCGGATCTTGGAGGCATTAGAGGCACATGAGCGCGTGCGGTGCAACTTTTAACCTAAAAAAATATGGGTTGTGTAGATT	T/C	GGGAGATTCCACACATAATGAAAGTGGTTGTGTCGGAAATTTTTTCCAGCAGCAACGGCAGGCAGCAGTAGCAGCAGACGGGGCAGCAGCGGTGATTGTG
Ca_LG_6:8153186	GAGGCACATGAGCGCGTGCGGTGCAACTTTTAACCTAAAAAAATATGGGTTGTGTAGATTGGGAGATTCCACACATAATGAAAGTGGTTGTGTCGGAAAT	T/A	TTTTTCCAGCAGCAACGGCAGGCAGCAGTAGCAGCAGACGGGGCAGCAGCGGTGATTGTGGGTGAAAGTGACAACAATCACAAAAAGGAATGATCATGCT
Ca_LG_6:8241302	TGTTCACGATCTTTCACAGAGAAGAACGAAGACTAGCTTGCAGAAATGGGCAGCTGAGATTGCTGCAACTGGAACATTTTCAGCTCCTTTGGGATCTGGT	T/G	GGCCCTGGTGGCCTTCCTGTTCCGTATATTGTTATTGGTAACAAAGTTGATATTGCTGCAAAAGAGGGTACAAGAGGAAGCAGTGGGAATCTTGTTGACG
Ca_LG_6:8241326	AACGAAGACTAGCTTGCAGAAATGGGCAGCTGAGATTGCTGCAACTGGAACATTTTCAGCTCCTTTGGGATCTGGTGGCCCTGGTGGCCTTCCTGTTCCG	G/A	TATATTGTTATTGGTAACAAAGTTGATATTGCTGCAAAAGAGGGTACAAGAGGAAGCAGTGGGAATCTTGTTGACGTTGCACGCCAGTGGGTTGAGAAGC
Ca_LG_6:8274170	AGTATCCGTACCTGGTTTGAAAAAGAGGGGGAAATTAACCAAAATATAAGTAAAGATATCATACACAAGTTCTCTGCTAGAATTGTCAAATAGCAGCAAC	C/T	ACAAAGCTATTGCATAGCGGAATTTTACTAAAATGTTATTATATAGAGCTATAGCATAGCCAAATTTTAATAAAATTTTAGTAGAAAGTGTCATCAAATA
Ca_LG_6:8274198	GGGAAATTAACCAAAATATAAGTAAAGATATCATACACAAGTTCTCTGCTAGAATTGTCAAATAGCAGCAACACAAAGCTATTGCATAGCGGAATTTTAC	C/A	TAAAATGTTATTATATAGAGCTATAGCATAGCCAAATTTTAATAAAATTTTAGTAGAAAGTGTCATCAAATAGTGGTTATAGNNNNNNNNNNNNNNNNNN
Ca_LG_6:8503370	AGGGTATAAAGAGATTTCAAAATGCATTGATCCAACTCCCAATTCTCCGTACAAAGCTAGAAACATGTGGAAAAATGCATTGATCAAACACCCAAATCAA	A/C	AAAGTGCTTTTCCATAATAGATTCCACGAATGGAACATGGAATAGTAAAACTACTAGGGTCTTGCATTTTTGTAAGGAGCTTTTGGTGTAACATTATATG

**Positions**	**-100 bp flanking sequences**	**SNPs within *desi* cultivar**	**+100 bp flanking sequences**
Ca_LG_6:8503381	AGATTTCAAAATGCATTGATCCAACTCCCAATTCTCCGTACAAAGCTAGAAACATGTGGAAAAATGCATTGATCAAACACCCAAATCAAAAAGTGCTTTT	T/C	CCATAATAGATTCCACGAATGGAACATGGAATAGTAAAACTACTAGGGTCTTGCATTTTTGTAAGGAGCTTTTGGTGTAACATTATATGCATTCCTTAGG
Ca_LG_6:8503385	TTCAAAATGCATTGATCCAACTCCCAATTCTCCGTACAAAGCTAGAAACATGTGGAAAAATGCATTGATCAAACACCCAAATCAAAAAGTGCTTTTCCAT	T/C	AATAGATTCCACGAATGGAACATGGAATAGTAAAACTACTAGGGTCTTGCATTTTTGTAAGGAGCTTTTGGTGTAACATTATATGCATTCCTTAGGGTTA
Ca_LG_6:8503393	GCATTGATCCAACTCCCAATTCTCCGTACAAAGCTAGAAACATGTGGAAAAATGCATTGATCAAACACCCAAATCAAAAAGTGCTTTTCCATAATAGATT	T/A	CCACGAATGGAACATGGAATAGTAAAACTACTAGGGTCTTGCATTTTTGTAAGGAGCTTTTGGTGTAACATTATATGCATTCCTTAGGGTTACAATTTCA
Ca_LG_6:8503398	GATCCAACTCCCAATTCTCCGTACAAAGCTAGAAACATGTGGAAAAATGCATTGATCAAACACCCAAATCAAAAAGTGCTTTTCCATAATAGATTCCACG	G/C	AATGGAACATGGAATAGTAAAACTACTAGGGTCTTGCATTTTTGTAAGGAGCTTTTGGTGTAACATTATATGCATTCCTTAGGGTTACAATTTCAAACTC
Ca_LG_6:8503410	AATTCTCCGTACAAAGCTAGAAACATGTGGAAAAATGCATTGATCAAACACCCAAATCAAAAAGTGCTTTTCCATAATAGATTCCACGAATGGAACATGG	G/A	AATAGTAAAACTACTAGGGTCTTGCATTTTTGTAAGGAGCTTTTGGTGTAACATTATATGCATTCCTTAGGGTTACAATTTCAAACTCCCGAATTCTTAT
Ca_LG_6:8503417	CGTACAAAGCTAGAAACATGTGGAAAAATGCATTGATCAAACACCCAAATCAAAAAGTGCTTTTCCATAATAGATTCCACGAATGGAACATGGAATAGTA	A/G	AAACTACTAGGGTCTTGCATTTTTGTAAGGAGCTTTTGGTGTAACATTATATGCATTCCTTAGGGTTACAATTTCAAACTCCCGAATTCTTATTCTTTTG
Ca_LG_6:8503425	GCTAGAAACATGTGGAAAAATGCATTGATCAAACACCCAAATCAAAAAGTGCTTTTCCATAATAGATTCCACGAATGGAACATGGAATAGTAAAACTACT	T/C	AGGGTCTTGCATTTTTGTAAGGAGCTTTTGGTGTAACATTATATGCATTCCTTAGGGTTACAATTTCAAACTCCCGAATTCTTATTCTTTTGGTGATTAT
Ca_LG_6:8503445	TGCATTGATCAAACACCCAAATCAAAAAGTGCTTTTCCATAATAGATTCCACGAATGGAACATGGAATAGTAAAACTACTAGGGTCTTGCATTTTTGTAA	A/G	GGAGCTTTTGGTGTAACATTATATGCATTCCTTAGGGTTACAATTTCAAACTCCCGAATTCTTATTCTTTTGGTGATTATATCCTTTAAAAACTTTGCAT
Ca_LG_6:8819393	TTGGTTTTGGATGCGCACTAAAAAAGGGGCAACTAGTGTTGGCACAGCTGTAACGGAGTAAAAAAAGGACAGAACAGTGGCAGCTGGTGTAAAGCATAGC	C/T	TGTGGTTGAAAGGAAAAAAAAGTCTTTGGAAAAACACAAGTGCTTATGAGGCAGTTGTAGCGTCGTGGCTGATTGCTAAAAAGGAAGTCGGAGTTGACGG
Ca_LG_6:8819398	TTTGGATGCGCACTAAAAAAGGGGCAACTAGTGTTGGCACAGCTGTAACGGAGTAAAAAAAGGACAGAACAGTGGCAGCTGGTGTAAAGCATAGCTGTGG	G/T	TTGAAAGGAAAAAAAAGTCTTTGGAAAAACACAAGTGCTTATGAGGCAGTTGTAGCGTCGTGGCTGATTGCTAAAAAGGAAGTCGGAGTTGACGGCGACA
Ca_LG_6:8887721	CTCATCTGGAAATATTTCTGGGGCTGCATCAAAATACTCAGAAGCATTGGCATTGTGTCCTATGAGATCCAAGAAGGAGAGAGTTGTTCTCTATAGTAAC	C/T	CGTGCTCAATGCTATCTTTTGCTGCAACAACCTTTGGCTGCCATAAGTGATGCTACCTGTGCACTTTGTCTCCATAGACCTGTGAATCGTCATGCCAAAA
Ca_LG_6:8939884	GATAGCTTTAAATGGGGGCTTATTTTCTTTGTTAATTTTCATTTCATTTCAACATTAACATTTTTTCTCTCCATGTTGCTCGGCTGCTTACAAAATTTTT	T/C	GGTCATATAATTTCAGGCTACTATTGGTATCGATTTTTTGTCAAAAACAATGTACCTTGAAGATAGAACTGTTCGTCTGCAGCTTTGGTAGGATAAGTTC

Positions	-100 bp flanking sequences	SNPs within *desi* cultivar	+100 bp flanking sequences
Ca_LG_6:8958366	ATCAATTAAAATCCATGTAACCAACTTCACGAAATGAGATACAGCTTTATTGTTAGTTATTCATAGCTAAACAAGCAGCAGCATAATTCAGCTAAGCACT	T/A	TGATTCAAAATGAAAATATGTCTGTTACATTTTCCCAACCACTCAAGAAACCAAAAGCTGATGTCACAATATAAAAGCCACATACCTGCAGCATTTGTCA
Ca_LG_6:9002833	TCAACTCGATTGCCAGCTGCATAAGAAAACCAGATGAACTAAGAAAAGCAGTTGAAGGGAAAGTGATTAGTTCTAAAGCAATGTCATGAACTTTAACTAC	C/A	TGAAATTTGAGTAGCATACCATCAATAAATTGCAATGCTGCCTTCTGAACATAGAGAGCAATACGAATTTTTTCCTGATCGAGTAAATCAAGTTAATATA
Ca_LG_6:9033205	ATTCCCAGATCAACTGTTCTTTAAAAAGTCTTCTATTTGAAGTTTCTCTTTTGTAGTTTGCTGCTAGCCTAAGTAGTTTGGGTTAGCTACAGTTACTTGC	C/T	TTTCCTTAAGTAGTGTGCTTTTCTAGCTCATTATGAAGAGCTTTGACAAAATGACCTGCATTGTGCATGCAGCCGCCTCCGCCTGTTAATGACACCAACC
Ca_LG_6:9689078	GCTAATATTCAAAATGGTTAATGACTTTTGGCACTATCTACACTCCCTGATGTGATCTCTGTAGATGTAGCTTAAGGGAATTGCTCCTCAATCCAGCATT	T/C	CTTGCCACATATTGGGCAAACTTTCATCGTTGGTTTCCGCCACCGCCGCCACTCCATTGAAACCTTTCCATACAGTTTTTGATCAAGTTCCTGTAAGATC
Ca_LG_6:9689117	ACACTCCCTGATGTGATCTCTGTAGATGTAGCTTAAGGGAATTGCTCCTCAATCCAGCATTCTTGCCACATATTGGGCAAACTTTCATCGTTGGTTTCCG	G/A	CCACCGCCGCCACTCCATTGAAACCTTTCCATACAGTTTTTGATCAAGTTCCTGTAAGATCTTAGCAAAGCCATCATTCGGCTGCGCTCGATGGTGAACT
Ca_LG_6:9826416	AGGATCCTCTGATTCTGTTGAAATTGAAACTGATAGGGAAAATGCTGCATCACTCGCCAAGGCACTTTGTACAGCTTTTGTAATTCCTATGACACTCTGT	T/C	GTTATCATCTACTCATTACTTTACTGCACGTACCCGAGAGACAGAGAGCGAGCGAGAATGGTTGCATTAGAGGAATCAGAAATGCAGCAACTAGATGTGG
Ca_LG_6:9826466	CACTCGCCAAGGCACTTTGTACAGCTTTTGTAATTCCTATGACACTCTGTGTTATCATCTACTCATTACTTTACTGCACGTACCCGAGAGACAGAGAGCG	G/A	AGCGAGAATGGTTGCATTAGAGGAATCAGAAATGCAGCAACTAGATGTGGAAGATTGCACAAGAGAAGAATATTGTGAAATTCATGTTTTAGAATCAAAT
Ca_LG_6:10242531	TCGGGTTACTGCAATGGTGGTGGCGGAATCAGATTTCTGAGAGTACCACAAAGGAGTTGCAATTCTGCCCCTTCCGATTCTGAAAATAACCCATTATCAT	T/G	AAACCTTCTCGTCATTCTTCCTCTTATGAGCGTCATTCTTCCTCTTATCGCTCATAATGTGATTGTTGCCCATTCCTTGTTTGTTCTCCACAAGGACCTT
Ca_LG_6:10719710	TATCTTCTTCTCCTTCTATATGGATTTTTATATCTTCATTGTTCTCAAGAAGATCCATTCTGGATAGTGAACATGGCTGCTATTTTCCGACGTGACATCG	G/A	GTGATTTTCCTTCCTCCAACCATTTCAAGCAGCAGCATCCCATAACTATAAACATCAGACTTATATGATACATTGCCGAAGTTCCTAGAAAATACCTCTG
Ca_LG_6:10770645	TCCCCGTATCCACTTCATGCTTTCGTCCTACGCACCTGTGATCTCTGCTGCCAAGGCATACCATGAGCAGCTCTCTGTGCCTGAAATCACCAATGCTGTA	A/G	TTTGAGCCTGCTAGCATGATGGCCAAGTGTGATCCAAGGCACGGGAAATACATGGCTTGCTGCTTGATGTATCGTGGAGATGTTGTACCCAAGGATGTAA
Ca_LG_6:10782487	AACTTCAAAAATAAAATAAAAAACTAGTTAAACAGAAGCACATCTAACCTGTACACGAGGATTCTGAAAATCATCCATCGCAGCAGCTAGTGCTGGCAAC	C/G	ACCCCTTGATGGTATTGAACTTGCAAATCTGGACCCAAATCAGTGGACAGTTGCCCGATTGCATTAATAGCTGCCCACCTTACACGAGGATGTTGATCAG
Ca_LG_6:11104497	CTTCCTAGAAGATTGGCTGCTATATTAATCCATCTGCTGGTAATGCTTCTATAGTGTTGCTATAGCAAGTAGGATCTATGTATAATATCTACAGGATCAT	T/G	AGAAAGCAAAAAATTAAGTCACATAATGACACTCTGAAGACTTCAGCAGCATAAGACGGCGTAGCAAACAATCAAATGAATATGCTAAGGAAATGAGAAA

Positions	-100 bp flanking sequences	SNPs within *desi* cultivar	+100 bp flanking sequences
Ca_LG_6:12035671	ACAAAATCCAGCTATGGGGGGAAATGGAACACTGATCTCTCAAACAAACGACACAACAGCTTCTGCAGCAGGAGCTAATGATTCTATGCAAAACCTGAAT	T/C	CAGGTCAGCAACTCCATTCAGAAAACCTTGGGCCTTATCCACCAGCTTTACCTTACAGTGTCTACCTTCAACGCTGCCTTTCAAATGCCACTCCTCCAAC
Ca_LG_6:12036343	AACTTAACATTTCTGTTTGGGGCAGAATTTTCGCAAGCATCTTTTGGAGGAATTGGAGCAGAACTTCCCTGATGAGGTTGAAAGTTTTAGAGAGAGTCGC	C/T	GCTGCTTCCGCTGCTGTAAGTGTTACTTATTTGGTCTTTTATTTCAATGTATCCATAACTATTGAATAACCTTCCTACATCATTGGCTGTTCCCAATTTT
Ca_LG_6:12277946	AACTGCAGCTAAATGTTTTGGAAATGAACTTTGTTCTATATATTATTTAATTTAATATTCATATACATACAAAATTCAGAATTAGCAGTCATCCCGCAAC	C/T	GCTCCATCTTGCTTTAGTGTTTTGAGGTGGCTGCTACACACTGCTATCCAGGATCAATAACTGCCAATTATACTTGGTTTCTGTTTTATATAGTTGGGTT
Ca_LG_6:12279324	AGCTATGAATTCTTCACTTGCTTCATCCTCCCTCCCTTTGATGATGCAGCAAGCTGTTGCCATGCAACAAATGCAATTCCAGCAAGCGCTGCTTATGCAG	G/A	CAAACTATGACTGCACAGCAGGCTGCTAACCGAGCTGCAACCATGAAATCTGCCACAGATTTAGCAGCAGCACGAGCTGCAGAAATTAGTAAGAAATTGA
Ca_LG_6:12315924	ACTAGTATATTTATAGTTGAAGAGAGACACAGTGAAAAGCAAAAAATATGATGATAAAAGTATAATATTTTACGGTGGTACTTAACGAAAAAAAGATTCA	A/G	TTAAAGCAGGTTTTGAAAAGCTCACTAACCAAACAGTACCCACACTTCATTTCTCTCTTTCTCTCTCTCTCTGCGCACACACCCGCAGCCATGGCGTC
Ca_LG_6:12319010	CCTAGGCAGAGGTGCAGGGAGGCTGCATTGGTATCAGTGGCTGAGGGAGCTCTGGTGGGAGAGAGTGGAAGGAAGGTTGCTGCAGTACCTTTATGTTAGA	A/C	AAGGGATTGCAAAAATAGAACCCTAGGGGTCAGAGGTTTCATTTTTCAGAAGTCAAGCCCAACCTGGTGTGAGAATTGCTCATATCCAGGAGATCCCAAA
Ca_LG_6:12330400	GATGGGATCCACGTGTCCACCTCCAATATCAAATGGGTCTGCTTCCTTGTGTGTGGGACCTTCTTCAGATATTAGGCTTCCATCAGTTCCACTTTGGGAG	G/T	GCTGCACTTTTCAATGTTACAATTAGTTAGTTTTGATAATGTGTTTGGATCATATTTTGATCTCGAATATAATTGATTTTGTAAAACTAATTTGATTATA
Ca_LG_6:12433128	AGCATCAAAGTATATTCCAAATGGTGGCTAATGGCTATGCTCAATTTCTGAAAAAGATGGACTAAGAGGAAAGAGATTGGGGGTAGTAATATTAAACAAT	T/G	ATTGGAAATTACACTTTTTGGCATGAAACTTTCAAGCTGCACCTGAAAATGTTAAGGTATCAAAAGTTATTATGTGACTGAATTCTAATACTTGTAAGGA
Ca_LG_6:12851337	ATTGATGGAATTGGAAAAAGTGTACTTGGTCTTTGAAATCTTTATATGGTTTTGGTTAATGAAACAAGGCTGGAGGCTACAAGGCAGCAACCAATCCTTT	T/C	TTTTCTGTTCTTTTTGGGCATTAGTAGAATATACGATGTGCAGCACAACAACAACCACTGATCCTTCTTTTCTCTTCTTTTTGGGAATTAGTGGGATGTA
Ca_LG_6:12851342	TGGAATTGGAAAAAGTGTACTTGGTCTTTGAAATCTTTATATGGTTTTGGTTAATGAAACAAGGCTGGAGGCTACAAGGCAGCAACCAATCCTTTTTTTC	C/A	TGTTCTTTTTGGGCATTAGTAGAATATACGATGTGCAGCACAACAACAACCACTGATCCTTCTTTTCTCTTCTTTTTGGGAATTAGTGGGATGTACAATG
Ca_LG_6:12851380	ATATGGTTTTGGTTAATGAAACAAGGCTGGAGGCTACAAGGCAGCAACCAATCCTTTTTTTCTGTTCTTTTTGGGCATTAGTAGAATATACGATGTGCAG	G/A	CACAACAACAACCACTGATCCTTCTTTTCTCTTCTTTTTGGGAATTAGTGGGATGTACAATGTGCACGACAATTTTTTTTTGGAATAGAAGATACAGTAA
Ca_LG_6:13401545	ATCTTTTTAATTATGTTTTATCTAAATAAGTTACTTTTATATAGATTTAATTTTTTTTTTTTGCGTTTTTTGTAGTTTCCTTGCTTAGTGTGGCGTTGTG	G/T	TTTATTCGTCGTTTTGGTGCTGCTTTTTTGACTTTCTATTTTGTTGCGGTGTTCTTTGGTTCATATTGATAAATTAACTTTGATTAAGTGCAAATTCAAC

Positions	-100 bp flanking sequences	SNPs within *desi* cultivar	+100 bp flanking sequences
Ca_LG_6:14696295	TTGAGTATGTGCAGCATTGCTTCGAAACAGAAACCAGTATGTTTCTAGTGTAAAATAAAAGCAAATCACAAACATAACCAAAACTAGAGGGTTCTATATA	A/G	TAACTCAAAAGGGGTAGAAAAGGGTTTTCTTACCCCATAAAAGATAAAAGGCCTCGAGTGAGAAACACAACCTAATAGAACACCTGCAGCAGCCTAAGGCT
Ca_LG_6:14925559	TTATCTTGGAATCGAATTCCCTAGCTGCCTTAAATCTTATCAAGAATGGGACTGACATCTAGCTATCATCCTCATGTTCTTCTTATCAGTCACATTCCTC	C/T	GTCTCTTGCAGCAGCCTTTGTTTTTGGTTCTGAAGCACACCTTAAAGAGAAGAAAATGAAGGTCCTCAAATGATCTGCTCAAGATTTGAAGTTTTTGCTC
Ca_LG_6:14987662	TTTATATAGTCCCCCGGCTTATCACCGACATAAGCCGACAGTCTATTCAGGGTTCCAAACTCAATGTTGGCAACTAAACACAAAGGTTGCGCTCGTTGCA	A/G	GGACTTAACCCAACACCTTACGGCACGAGCTGACGACAGCCATGCACCACCTGTCCGCGTTCCCGAAGGCATCCCTCTCTTTCAAGAGGATTCGCGACAT
Ca_LG_6:14987681	TATCACCGACATAAGCCGACAGTCTATTCAGGGTTCCAAACTCAATGTTGGCAACTAAACACAAAGGTTGCGCTCGTTGCAGGACTTAACCCAACACCTT	T/C	ACGGCACGAGCTGACGACAGCCATGCACCACCTGTCCGCGTTCCCGAAGGCATCCCTCTCTTTCAAGAGGATTCGCGACATGTCAAGCCTTGGTAAGATT
Ca_LG_6:16721241	CTAGCTTGTAAGGGGGTAGGGATGCCCCTTTCTAAAACTTAGATTTAATATAAGGAAGAATAAGGCCCTTTTTTGTTTGGAGTTTTCCCCAACCTATACA	A/C	TAAGGGCTTCCGTTTTCAGCAGCATCGCACTGCCGCATAAACAATGGATCCGCTGGGCTGGATGGGCTTCTCTGGAAAGGAATAAGGAATAGTGAAAAGC
Ca_LG_6:16826987	AAAACGCCATTCATCGCGAAAAAAGTTTGTCACGCTCGTATCAGAATTGAAGAATAATAACTGCAGCATGATCTCTTTTGAAAGTCAAGATCTTTGTTAT	T/C	TACTTCATGATAGCTTTATCCGTAACAAATAGCACAGCTCGTATAAAAAAGTCTAACACGCCTTCTATCAAAATTAAATCCTTACTAAAATAAATAAATA
Ca_LG_6:16826993	CCATTCATCGCGAAAAAAGTTTGTCACGCTCGTATCAGAATTGAAGAATAATAACTGCAGCATGATCTCTTTTGAAAGTCAAGATCTTTGTTATTACTTC	C/G	ATGATAGCTTTATCCGTAACAAATAGCACAGCTCGTATAAAAAAGTCTAACACGCCTTCTATCAAAATTAAATCCTTACTAAAATAAATAAATAGATAAA
Ca_LG_6:16826996	TTCATCGCGAAAAAAGTTTGTCACGCTCGTATCAGAATTGAAGAATAATAACTGCAGCATGATCTCTTTTGAAAGTCAAGATCTTTGTTATTACTTCATG	G/C	ATAGCTTTATCCGTAACAAATAGCACAGCTCGTATAAAAAAGTCTAACACGCCTTCTATCAAAATTAAATCCTTACTAAAATAAATAAATAGATAAAAAC
Ca_LG_6:16827011	GTTTGTCACGCTCGTATCAGAATTGAAGAATAATAACTGCAGCATGATCTCTTTTGAAAGTCAAGATCTTTGTTATTACTTCATGATAGCTTTATCCGTA	A/C	ACAAATAGCACAGCTCGTATAAAAAAGTCTAACACGCCTTCTATCAAAATTAAATCCTTACTAAAATAAATAAATAGATAAAAACAATAAACAACTCAAA
Ca_LG_6:16939749	TCAAACTAATAGTAATTGTTCTGCCTAGGCCTGATTGTAAAATCCTTTCAACTTTAAATTAACGAATACTAATTATTAAGGCTAAGCTGCCTATGCCTGC	C/T	GGAACTAACTTTTCAAGACATCCTAGCCACACGTGTTTTCATTAGCTATATGTCCAAGCAGCTAGCTGTATTCCCAGCCAATCATGATTTCAAACCCATT
Ca_LG_6:16939750	CAAACTAATAGTAATTGTTCTGCCTAGGCCTGATTGTAAAATCCTTTCAACTTTAAATTAACGAATACTAATTATTAAGGCTAAGCTGCCTATGCCTGCG	G/A	GAACTAACTTTTCAAGACATCCTAGCCACACGTGTTTTCATTAGCTATATGTCCAAGCAGCTAGCTGTATTCCCAGCCAATCATGATTTCAAACCCATTC
Ca_LG_6:16939780	TGATTGTAAAATCCTTTCAACTTTAAATTAACGAATACTAATTATTAAGGCTAAGCTGCCTATGCCTGCGGAACTAACTTTTCAAGACATCCTAGCCACA	A/G	CGTGTTTTCATTAGCTATATGTCCAAGCAGCTAGCTGTATTCCCAGCCAATCATGATTTCAAACCCATTCATTTCAAAAACAAAACAAACCAGCCTTAGC

Positions	-100 bp flanking sequences	SNPs within *desi* cultivar	+100 bp flanking sequences
Ca_LG_6:16939792	CCTTTCAACTTTAAATTAACGAATACTAATTATTAAGGCTAAGCTGCCTATGCCTGCGGAACTAACTTTTCAAGACATCCTAGCCACACGTGTTTTCATT	T/C	AGCTATATGTCCAAGCAGCTAGCTGTATTCCCAGCCAATCATGATTTCAAACCCATTCATTTCAAAAACAAAACAAACCAGCCTTAGCCTCATTTCAAAA
Ca_LG_6:17125227	CTCCTCTTGAGTTGAATGTGAAATATGCTTCTTCAGATGTTGTTCCTTTACCATATCTCACTTTGTATTGTACTTTGATTGACAGCTTGATGTATCTTAC	C/T	GATTACCAAACCTGATATTGCTTATGTTGTTCATGTTGTCAGTCAGTTTGTTGTGTCTTCCACTACAATACATTGTGATGTAGCTAAATATCACTAGAGG
Ca_LG_6:17125266	TTGTTCCTTTACCATATCTCACTTTGTATTGTACTTTGATTGACAGCTTGATGTATCTTACGATTACCAAACCTGATATTGCTTATGTTGTTCATGTTGT	T/A	CAGTCAGTTTGTTGTGTCTTCCACTACAATACATTGTGATGTAGCTAAATATCACTAGAGGGGGGGGGGGGGTTGAATATGGATTTTGCTGTTTAAAAATA
Ca_LG_6:17125282	TCTCACTTTGTATTGTACTTTGATTGACAGCTTGATGTATCTTACGATTACCAAACCTGATATTGCTTATGTTGTTCATGTTGTCAGTCAGTTTGTTGTG	G/C	TCTTCCACTACAATACATTGTGATGTAGCTAAATATCACTAGAGGGGGGGGGGGGTTGAATATGGATTTTGCTGTTTAAAAATAATTTTCAAGAGATTGT
Ca_LG_6:17125284	TCACTTTGTATTGTACTTTGATTGACAGCTTGATGTATCTTACGATTACCAAACCTGATATTGCTTATGTTGTTCATGTTGTCAGTCAGTTTGTTGTGTC	C/A	TTCCACTACAATACATTGTGATGTAGCTAAATATCACTAGAGGGGGGGGGGGTTGAATATGGATTTTGCTGTTTAAAAATAATTTTCAAGAGATTGTAA
Ca_LG_6:18395040	ATATTGTTGTTACTAAAGAAATAAAGGAGGCTATTTGGATGGGAGGAATGGTTAAGAGCATGGAGACAAAACAAGTTGTTACCAAGGTGTTCTGTGATAA	A/G	TCAAAGTAGTATTCACTTGGCAAGAAATCAAGTATACCATGATGAGAGGACAAAACACATAGATGACAAGCTACATTTTGTGAGAGAAGTCATTGCAAAA
Ca_LG_6:18395048	GTTACTAAAGAAATAAAGGAGGCTATTTGGATGGGAGGAATGGTTAAGAGCATGGAGACAAAACAAGTTGTTACCAAGGTGTTCTGTGATAATCAAAGTA	A/G	GTATTCACTTGGCAAGAAATCAAGTATACCATGATGAGAGGACAAAACACATAGATGACAAGCTACATTTTGTGAGAGAAGTCATTGCAAAAGGAAAGAT
Ca_LG_6:19288654	CTTTTATGATCTGCAGAAAAATGCATGGGTACCAGGAAGTACCAGGAATCCTGAATCATATATTTTGAAAGATCAGGTATTATGGCATGAAATGCAGTCC	C/T	GATAGATATTTTCAGTGCTTTAAGTCTGCTGCATACTATCTTATACCTCCAAACAAATCAGAGTGGGTGATTAATAGGGGATACTATCTTATACCTTTTG
Ca_LG_6:19731770	TCCAAGAAGGGATGACACAACAAGAGTCAACGACAATGGGGATGATCAAATGGCCGAAGCTATGAATAACATGGTTACTTCTGTTGCTGCATAGACTCTG	G/A	CGAGATCTGGAGAAGAGGGAAATAGAGATATGTGCTGCTGCGTCAAGGGGATTAGAAAATTTTCGTCGTTACCATCCTCCAAAGTTCAAGGGTGATGAGA
Ca_LG_6:19731781	ATGACACAACAAGAGTCAACGACAATGGGGATGATCAAATGGCCGAAGCTATGAATAACATGGTTACTTCTGTTGCTGCATAGACTCTGCGAGATCTGGA	A/G	GAAGAGGGAAATAGAGATATGTGCTGCTGCGTCAAGGGGATTAGAAAATTTTCGTCGTTACCATCCTCCAAAGTTCAAGGGTGATGAGAACTCAGAAAAC
Ca_LG_6:19731805	ATGGGGATGATCAAATGGCCGAAGCTATGAATAACATGGTTACTTCTGTTGCTGCATAGACTCTGCGAGATCTGGAGAAGAGGGAAATAGAGATATGTGC	C/T	TGCTGCGTCAAGGGGATTAGAAAATTTTCGTCGTTACCATCCTCCAAAGTTCAAGGGTGATGAGAACTCAGAAAACGCTGATCAGTGAATTTCCGAAGTG
Ca_LG_6:21242042	TCGCGGCAGTGAGAACGAGGAAAATTGAGAGTTGGTGAATGAGGGTAAAAGAGAAACAAATCAAGAGGGTTTTCTGGTGAGTGAGGGAAAACGAGGGCGA	A/G	CACAATACGAAGAAGGAAGGAAAGAGGCGAAGGCGGCGCCTGCAAGGCTGCCGCCGTGGGATTAGGGTGAAAAAGATAGAAAGAACAAAAGTAAAGGAAA

Positions	-100 bp flanking sequences	SNPs within *desi* cultivar	+100 bp flanking sequences
Ca_LG_6:21242047	GCAGTGAGAACGAGGAAAATTGAGAGTTGGTGAATGAGGGTAAAAGAGAAACAAATCAAGAGGGTTTTCTGGTGAGTGAGGGAAAACGAGGGCGACACAA	A/G	TACGAAGAAGGAAGGAAAGAGGCGAAGGCGGCGCCTGCAAGGCTGCCGCCGTGGGATTAGGGTGAAAAAGATAGAAAGAACAAAAGTAAAGGAAAAAAAG
Ca_LG_6:21242065	ATTGAGAGTTGGTGAATGAGGGTAAAAGAGAAACAAATCAAGAGGGTTTTCTGGTGAGTGAGGGAAAACGAGGGCGACACAATACGAAGAAGGAAGGAAA	A/G	GAGGCGAAGGCGGCGCCTGCAAGGCTGCCGCCGTGGGATTAGGGTGAAAAAGATAGAAAGAACAAAAGTAAAGGAAAAAAAGAGAGTGAGGGAATAACAG
Ca_LG_6:21242070	GAGTTGGTGAATGAGGGTAAAAGAGAAACAAATCAAGAGGGTTTTCTGGTGAGTGAGGGAAAACGAGGGCGACACAATACGAAGAAGGAAGGAAAGAGGC	C/A	GAAGGCGGCGCCTGCAAGGCTGCCGCCGTGGGATTAGGGTGAAAAAGATAGAAAGAACAAAAGTAAAGGAAAAAAAGAGAGTGAGGGAATAACAGAGGTT
Ca_LG_6:23710050	TTAATAAAATAAAAGTTTAGCCATCATTTCAAAAACAACTCATGTAGTAATAAAGCAAAGCAGCTCCCAGACATCACAGTGACCTAAGTAATTAAATTTC	C/T	AAGCATATCACGTTCTGCGCACAACATCAGCAAATCGCAAGATTTCAATCAAAGCATAAACCACACGATTTCAATTCAAGGTTTCTTAAAATACTAAACA
Ca_LG_6:23710055	AAAATAAAAGTTTAGCCATCATTTCAAAAACAACTCATGTAGTAATAAAGCAAAGCAGCTCCCAGACATCACAGTGACCTAAGTAATTAAATTTCAAGCA	A/G	TATCACGTTCTGCGCACAACATCAGCAAATCGCAAGATTTCAATCAAAGCATAAACCACACGATTTCAATTCAAGGTTTCTTAAAATACTAAACAAGGCA
Ca_LG_6:23853490	TTGCTGTCGAAGCGCTAAACTTAGCTAAAGATTTCAACATGTTGTCCTATATATAATTCTCTTCTGCGGCTACTACACTTTCTTTTAGCTTCTATTTTCT	T/C	TAAGTTGGATGAGGAAACATCATGTGAGTACAGAGATCTACCGGAGCCTATCAAAAAACCAGGTTGCATACCACTCCATATGGCAGCGATCTTCTTACAC
Ca_LG_6:23853533	GTCCTATATATAATTCTCTTCTGCGGCTACTACACTTTCTTTTAGCTTCTATTTTCTTAAGTTGGATGAGGAAACATCATGTGAGTACAGAGATCTACCG	G/A	GAGCCTATCAAAAAACCAGGTTGCATACCACTCCATATGGCAGCGATCTTCTTACACCGGCTCAAGATAGATGGAGTCAAGCTTACAAACACTTCCTCCA
Ca_LG_6:23853547	TCTCTTCTGCGGCTACTACACTTTCTTTTAGCTTCTATTTTCTTAAGTTGGATGAGGAAACATCATGTGAGTACAGAGATCTACCGGAGCCTATCAAAAA	A/T	ACCAGGTTGCATACCACTCCATATGGCAGCGATCTTCTTACACCGGCTCAAGATAGATGGAGTCAAGCTTACAAACACTTCCTCCAACATTCTAAAACCA
Ca_LG_6:23858611	AATTTGTGGTTGCCTAAGTCCTCACAAAATCACAACGTTGTGATTTTGTTAGGGCTTAAGCAGCCACAAAATCCACTTAAATTTTTAAGATTTTTGTGAA	A/G	GGCTTTTTCAGCCACAAAATCTAAATTTTGTGAGGGTCAAGGTCGCCACAAAATCCTGAAGTGACATTACAATTTGTGGCTGCAAAGGCCGCCACAAAAG
Ca_LG_6:23858669	AGCAGCCACAAAATCCACTTAAATTTTTAAGATTTTTGTGAAGGCTTTTTCAGCCACAAAATCTAAATTTTGTGAGGGTCAAGGTCGCCACAAAATCCTG	G/A	AAGTGACATTACAATTTGTGGCTGCAAAGGCCGCCACAAAAGCCTGAGTCGTTATATTTTGTGGCTGCAAAGGCCGCCACAAAGGAACCAAAGTAACGTT
Ca_LG_6:23858693	TTTTAAGATTTTTGTGAAGGCTTTTTCAGCCACAAAATCTAAATTTTGTGAGGGTCAAGGTCGCCACAAAATCCTGAAGTGACATTACAATTTGTGGCTG	G/A	CAAAGGCCGCCACAAAAGCCTGAGTCGTTATATTTTGTGGCTGCAAAGGCCGCCACAAAGGAACCAAAGTAACGTTGCATTTTTTGCTGTTCAGGCCGCC
Ca_LG_6:23858700	ATTTTTGTGAAGGCTTTTTCAGCCACAAAATCTAAATTTTGTGAGGGTCAAGGTCGCCACAAAATCCTGAAGTGACATTACAATTTGTGGCTGCAAAGGC	C/T	CGCCACAAAAGCCTGAGTCGTTATATTTTGTGGCTGCAAAGGCCGCCACAAAGGAACCAAAGTAACGTTGCATTTTTTGCTGTTCAGGCCGCCACAAAGT

Positions	-100 bp flanking sequences	SNPs within *desi* cultivar	+100 bp flanking sequences
Ca_LG_6:23858712	GCTTTTTCAGCCACAAAATCTAAATTTTGTGAGGGTCAAGGTCGCCACAAAATCCTGAAGTGACATTACAATTTGTGGCTGCAAAGGCCGCCACAAAAGC	C/T	CTGAGTCGTTATATTTTGTGGCTGCAAAGGCCGCCACAAAGGAACCAAAGTAACGTTGCATTTTTTGCTGTTCAGGCCGCCACAAAGTTTTAAACCGTTA
Ca_LG_6:23858718	TCAGCCACAAAATCTAAATTTTGTGAGGGTCAAGGTCGCCACAAAATCCTGAAGTGACATTACAATTTGTGGCTGCAAAGGCCGCCACAAAAGCCTGAGT	T/C	CGTTATATTTTGTGGCTGCAAAGGCCGCCACAAAGGAACCAAAGTAACGTTGCATTTTTTGCTGTTCAGGCCGCCACAAAGTTTTAAACCGTTACCTTTT
Ca_LG_6:23858724	ACAAAATCTAAATTTTGTGAGGGTCAAGGTCGCCACAAAATCCTGAAGTGACATTACAATTTGTGGCTGCAAAGGCCGCCACAAAAGCCTGAGTCGTTAT	T/C	ATTTTGTGGCTGCAAAGGCCGCCACAAAGGAACCAAAGTAACGTTGCATTTTTTGCTGTTCAGGCCGCCACAAAGTTTTAAACCGTTACCTTTTGTGGCT
Ca_LG_6:23858730	TCTAAATTTTGTGAGGGTCAAGGTCGCCACAAAATCCTGAAGTGACATTACAATTTGTGGCTGCAAAGGCCGCCACAAAAGCCTGAGTCGTTATATTTTG	G/T	TGGCTGCAAAGGCCGCCACAAAGGAACCAAAGTAACGTTGCATTTTTTGCTGTTCAGGCCGCCACAAAGTTTTAAACCGTTACCTTTTGTGGCTGCAAAG
Ca_LG_6:23947676	ATTTCGATCCTCCTTTAACGATTCTGCCTCTATTCCGCTGCTGAGTTCCCAACAGTTTGGGAATATAATCATAGTTTCTCTTTTCTAGCATGATCATTCC	C/A	TTTTTCTGACTGTTGTCGCTTCCGCCTACAATCGTCGCTGCTGCCTCGTCTGCTATTGTCGCTGCCTCCGTCTGCTGCTGTCCCGTTTGCTATTGCCGCC
Ca_LG_6:23965648	TATTATATTTTTGTAATTTGTCAGGAGTGAAGAGACCAAAATATTGTTCAGTTGCTGCACCAGGCTTCTGATTTTCATCAAACATAGCAAACAAATAAGC	C/T	CTCAATAGGTTGACCAGGCCGCATAGGAGTTCCAATCCCACTATTGACATGATTAATCAAATTAGTATAATATGCAGCAGCATTTTCAGTTGTGGCCCCT
Ca_LG_6:23965669	CAGGAGTGAAGAGACCAAAATATTGTTCAGTTGCTGCACCAGGCTTCTGATTTTCATCAAACATAGCAAACAAATAAGCCTCAATAGGTTGACCAGGCCG	G/T	CATAGGAGTTCCAATCCCACTATTGACATGATTAATCAAATTAGTATAATATGCAGCAGCATTTTCAGTTGTGGCCCCTTCCCCACCTGCAGATGGCCAT
Ca_LG_6:23965683	CCAAAATATTGTTCAGTTGCTGCACCAGGCTTCTGATTTTCATCAAACATAGCAAACAAATAAGCCTCAATAGGTTGACCAGGCCGCATAGGAGTTCCAA	A/T	TCCCACTATTGACATGATTAATCAAATTAGTATAATATGCAGCAGCATTTTCAGTTGTGGCCCCTTCCCCACCTGCAGATGGCCATCCACTCTCAGACAC
Ca_LG_6:24222466	TGAATTTTGTACAAACCTGAAAGCAAGCACCACAAGCAGCTCCATCATAGAAAAGTGAAGAAACACCAGCTGCAAGTTGTCCACCACTAAAGTCCAATGC	C/G	TAAAGAACCATACCCACATGCCCCAGCTGCATAAAAATAAATATAAAAATTAAACAATGAAAAAATATTTGTTAACAAAAGAACTCTATATGAAATGAAA
Ca_LG_6:24222480	ACCTGAAAGCAAGCACCACAAGCAGCTCCATCATAGAAAAGTGAAGAAACACCAGCTGCAAGTTGTCCACCACTAAAGTCCAATGCTAAAGAACCATACC	C/T	CACATGCCCCAGCTGCATAAAAATAAATATAAAAATTAAACAATGAAAAAATATTTGTTAACAAAAGAACTCTATATGAAATGAAAATTGTTAATTACAT
Ca_LG_6:24222487	AGCAAGCACCACAAGCAGCTCCATCATAGAAAAGTGAAGAAACACCAGCTGCAAGTTGTCCACCACTAAAGTCCAATGCTAAAGAACCATACCCACATGC	C/T	CCCAGCTGCATAAAAATAAATATAAAAATTAAACAATGAAAAAATATTTGTTAACAAAAGAACTCTATATGAAATGAAAATTGTTAATTACATGAGAGAG
Ca_LG_6:25357840	CCCTACTCCAATTACCACCCACACCTTTGACAAGTCCTCCTGTTTTACAACCACCTCTGGTGAATCCAATGCCTTAGGCGAGCTCAGTGCTGCCTGCATT	T/C	AACCCTATCAACCTCTCTGCAAATTTGCAAAAACTCTCAGTGACAATAACTTTCTCTGGTGGAAGCAGCAAAGTAAGCTAGTTTTGAAGGGTCATCGCCA

**Positions**	**-100 bp flanking sequences**	**SNPs within *desi* cultivar**	**+100 bp flanking sequences**
Ca_LG_6:25407317	TGTTAAAAAGGCTATACCAGTGCTATAGCCCTTTAGTGTAGCGGATTTTGGACAAAACGCTATTTTCCATGATGCAACATTGGCTGCCGCTTGCTACAAC	C/T	TCAAACATTTCGAATTACTGGAATTGTTGTTGATGCATGAACAATAGGTTACTCGATCAGTGGAATTTGTTTCTAGTAAATGCAAGGTAACACTGCCTAG
Ca_LG_6:25407328	CTATACCAGTGCTATAGCCCTTTAGTGTAGCGGATTTTGGACAAAACGCTATTTTCCATGATGCAACATTGGCTGCCGCTTGCTACAACTCAAACATTTC	C/T	GAATTACTGGAATTGTTGTTGATGCATGAACAATAGGTTACTCGATCAGTGGAATTTGTTTCTAGTAAATGCAAGGTAACACTGCCTAGTACCTACAATA
Ca_LG_6:25407394	CATTGGCTGCCGCTTGCTACAACTCAAACATTTCGAATTACTGGAATTGTTGTTGATGCATGAACAATAGGTTACTCGATCAGTGGAATTTGTTTCTAGT	T/C	AAATGCAAGGTAACACTGCCTAGTACCTACAATATATTCACAATGAGTTTGACCCTTCACTTGACCCGATTACGGCAGCAGCTCTATAGTATCATGTTTC
Ca_LG_6:25599940	NNNNNNNNNNNNNNNNNNNNNNNNNNNNNNNNNNNNNNNNNNNNNTTCTAAGTTTGCTGCCCCATTTTTAAAAAGGGGGAAAAAATTCAAATTTTTAAAA	A/T	TGTTAGAAAAATCTGGTATTCTAGTTTTTTGAAAAAGTGTATAATCTTAAGGGATAAAATCCGGTAAATTCTAAGTTTGCTGCCCTTTTTTTGAAAATCT
Ca_LG_6:25600066	TTTTGAAAAAGTGTATAATCTTAAGGGATAAAATCCGGTAAATTCTAAGTTTGCTGCCCTTTTTTTGAAAATCTGGTATTCTGTTTCAGATTTTTAAGGT	T/G	GTAAAAAATTTGGAACATCTTTATAATCTTAACTCTTATTTTAAAATTTTTAAAGTTTTTAAATGAATTTGAGTTTTAATTTTAATATTATAAGTCTATT
Ca_LG_6:25772731	GTTGGTTGGGTTTAGATCTTATGTTGCTTGTCGGCTTATATCTGCAATATCTTCAAATGACATCTTAAGGCCTAGGTGTTCTTGTATTGCAGCCACATCC	C/A	TGAGCGACGGAATTGAGTGATGCCTGGAGAGTCTTGAGAGATGCTTCTACTCTTGCATTATGAGCAGCTTGATTCTCCATAAATTCCAGCGGCTTCGTTA
Ca_LG_6:26811992	GATTATTCTTTTATATGTAATGTAGAAACATTAAAACAACTTATGAAAATGGTTTCGAATTTCTTAACCTTACTAATGTTGTTCCTTGCTGCTCTTATCT	T/C	TTGTTCCACAAGGACTTGCTGATGTAAGCCCACAAATCTCCAAGACACCAATTCCTAAACCACCTAATTACGACAAAAAAGGAGAACTCGACATTATTAG
Ca_LG_6:26863178	TAGCTTTTACAGTCTTGGCAAATTAGGTGACAGAATAAAACCAAGTGTTACTACCTGATATACAGTTTTTGCAGCCCGTTTTACTGTCACTTGTAACTGT	T/C	ATCAATGCATCTTTAACAAGCATACCACGAACCAAAGCAGCAACCAAGTTGACCTTCTTTGGACTCTAAAATAAGGTTTAAAAAATGAACATACATTAAG
Ca_LG_6:27812911	GGCAATACACCTCAAGTAGAAGGAACCTTGAGATGGTCTCCCTTCCCATCTCCCTTTCTCTTTCAACCTCTCTATGTCTATCTCTTATCTCAGACTCTCA	A/C	TAACCAACACTCCAAACAGCAGCAGCGAGCCCCTACTACATTGCTTGTCCCTACATGAGTCAAGGAAACCAATCCAAACTGAACAATTGTGATTGCGCTG
Ca_LG_6:27812925	AGTAGAAGGAACCTTGAGATGGTCTCCCTTCCCATCTCCCTTTCTCTTTCAACCTCTCTATGTCTATCTCTTATCTCAGACTCTCATAACCAACACTCCA	A/G	AACAGCAGCAGCGAGCCCCTACTACATTGCTTGTCCCTACATGAGTCAAGGAAACCAATCCAAACTGAACAATTGTGATTGCGCTGGAAATGATATATCA
Ca_LG_6:29978947	CTTGGATCCATTGATCCGCCTTCTCTGAATTCTCATCCCCCTTAAACTTGGGAGGATTGTAACGACGAAAGTCTTCTAATCCTCTTGACTCTGCAGCACG	G/A	GATCTCTCTTTCCCTCTTTTCAAGATCACGTCGAGTCTTGGCAGCAGTCTGTGCAGCAACAAAAGCAACCATGTTATTCATAGCTTCCGCCATTCGATCG
Ca_LG_6:29978955	CATTGATCCGCCTTCTCTGAATTCTCATCCCCCTTAAACTTGGGAGGATTGTAACGACGAAAGTCTTCTAATCCTCTTGACTCTGCAGCACGGATCTCTC	C/T	TTTCCCTCTTTTCAAGATCACGTCGAGTCTTGGCAGCAGTCTGTGCAGCAACAAAAGCAACCATGTTATTCATAGCTTCCGCCATTCGATCGTCCCTATT

**Positions**	**-100 bp flanking sequences**	**SNPs within *desi* cultivar**	**+100 bp flanking sequences**
Ca_LG_6:29978961	TCCGCCTTCTCTGAATTCTCATCCCCCTTAAACTTGGGAGGATTGTAACGACGAAAGTCTTCTAATCCTCTTGACTCTGCAGCACGGATCTCTCTTTCCC	C/A	TCTTTTCAAGATCACGTCGAGTCTTGGCAGCAGTCTGTGCAGCAACAAAAGCAACCATGTTATTCATAGCTTCCGCCATTCGATCGTCCCTATTAGCATT
Ca_LG_6:29978996	GGGAGGATTGTAACGACGAAAGTCTTCTAATCCTCTTGACTCTGCAGCACGGATCTCTCTTTCCCTCTTTTCAAGATCACGTCGAGTCTTGGCAGCAGTC	C/A	TGTGCAGCAACAAAAGCAACCATGTTATTCATAGCTTCCGCCATTCGATCGTCCCTATTAGCATTGGCTCTTGGAACGTCATTCCTTCTTGGCGGCATGG
Ca_LG_6:29979009	CGACGAAAGTCTTCTAATCCTCTTGACTCTGCAGCACGGATCTCTCTTTCCCTCTTTTCAAGATCACGTCGAGTCTTGGCAGCAGTCTGTGCAGCAACAA	A/G	AAGCAACCATGTTATTCATAGCTTCCGCCATTCGATCGTCCCTATTAGCATTGGCTCTTGGAACGTCATTCCTTCTTGGCGGCATGGTTTTCCTATAAAA
Ca_LG_6:30417319	ACGGTATTTGAAGAAGGCACCAAGACAAAGCTTATTTTTCCAATGGACTCAGAACTATAACTTAGTGGTTTTAGTGGTGCTGGCTGGGGAACATGCAAGA	A/G	ACACTAGAAGATCATATTTTGGAAGTAGTATCTTCTTAGGAAACTCATTAATAAGTTGGAAGACTAATAAGCAAACAATAGTAGCAAAATCATCATCAGT
Ca_LG_6:30417334	GGCACCAAGACAAAGCTTATTTTTCCAATGGACTCAGAACTATAACTTAGTGGTTTTAGTGGTGCTGGCTGGGGAACATGCAAGAACACTAGAAGATCAT	T/A	ATTTTGGAAGTAGTATCTTCTTAGGAAACTCATTAATAAGTTGGAAGACTAATAAGCAAACAATAGTAGCAAAATCATCATCAGTGGCTGAGTATAGGGC
Ca_LG_6:30417335	GCACCAAGACAAAGCTTATTTTTCCAATGGACTCAGAACTATAACTTAGTGGTTTTAGTGGTGCTGGCTGGGGAACATGCAAGAACACTAGAAGATCATA	A/T	TTTTGGAAGTAGTATCTTCTTAGGAAACTCATTAATAAGTTGGAAGACTAATAAGCAAACAATAGTAGCAAAATCATCATCAGTGGCTGAGTATAGGGCA
Ca_LG_6:30417338	CCAAGACAAAGCTTATTTTTCCAATGGACTCAGAACTATAACTTAGTGGTTTTAGTGGTGCTGGCTGGGGAACATGCAAGAACACTAGAAGATCATATTT	T/C	TGGAAGTAGTATCTTCTTAGGAAACTCATTAATAAGTTGGAAGACTAATAAGCAAACAATAGTAGCAAAATCATCATCAGTGGCTGAGTATAGGGCACTT
Ca_LG_6:30417340	AAGACAAAGCTTATTTTTCCAATGGACTCAGAACTATAACTTAGTGGTTTTAGTGGTGCTGGCTGGGGAACATGCAAGAACACTAGAAGATCATATTTTG	G/A	GAAGTAGTATCTTCTTAGGAAACTCATTAATAAGTTGGAAGACTAATAAGCAAACAATAGTAGCAAAATCATCATCAGTGGCTGAGTATAGGGCACTTGC
Ca_LG_6:30417346	AAGCTTATTTTTCCAATGGACTCAGAACTATAACTTAGTGGTTTTAGTGGTGCTGGCTGGGGAACATGCAAGAACACTAGAAGATCATATTTTGGAAGTA	A/T	GTATCTTCTTAGGAAACTCATTAATAAGTTGGAAGACTAATAAGCAAACAATAGTAGCAAAATCATCATCAGTGGCTGAGTATAGGGCACTTGCAACTGC
Ca_LG_6:30417349	CTTATTTTTCCAATGGACTCAGAACTATAACTTAGTGGTTTTAGTGGTGCTGGCTGGGGAACATGCAAGAACACTAGAAGATCATATTTTGGAAGTAGTA	A/G	TCTTCTTAGGAAACTCATTAATAAGTTGGAAGACTAATAAGCAAACAATAGTAGCAAAATCATCATCAGTGGCTGAGTATAGGGCACTTGCAACTGCCAC
Ca_LG_6:30417351	TATTTTTCCAATGGACTCAGAACTATAACTTAGTGGTTTTAGTGGTGCTGGCTGGGGAACATGCAAGAACACTAGAAGATCATATTTTGGAAGTAGTATC	C/T	TTCTTAGGAAACTCATTAATAAGTTGGAAGACTAATAAGCAAACAATAGTAGCAAAATCATCATCAGTGGCTGAGTATAGGGCACTTGCAACTGCCACTA
Ca_LG_6:30417365	ACTCAGAACTATAACTTAGTGGTTTTAGTGGTGCTGGCTGGGGAACATGCAAGAACACTAGAAGATCATATTTTGGAAGTAGTATCTTCTTAGGAAACTC	C/T	ATTAATAAGTTGGAAGACTAATAAGCAAACAATAGTAGCAAAATCATCATCAGTGGCTGAGTATAGGGCACTTGCAACTGCCACTAGTGAACTTCAATGA

Positions	-100 bp flanking sequences	SNPs within *desi* cultivar	+100 bp flanking sequences
Ca_LG_6:30564125	ATAAATAACATGGACAACCTTACCAACTCTTTGTGCAAGGACTGCTCCCACTACATAGTTAGATGCATCACACATAATTTCAAAAGGGAGGATCCAATTG	G/T	GGTGGCTGAATTATGGGAGTAGAGGTCAGTCATGCCTTTAAGAAATCAAACCTCTTCTTGCATTTGTCGTCAAAGGTGAAATTGACATCGTTTTGCAGCA
Ca_LG_6:31061055	CGTTACTGGAAAATATTATTCTTTCATTCAACCACTTCAGTAATGTAGAAGAATTCACAATCATGTCTTCCTCCAAATTAAACATCATTGATTTAAGCAA	A/G	CAATGATCTATCAGGGTCTTTTCCAACATCTATCTTACAGCTCGGTTCACTTTCTGTCCTCGATGTTTCCTCTAACAGGTTGAATGGGTCGCTGCAGCTA
Ca_LG_6:31061072	ATTCTTTCATTCAACCACTTCAGTAATGTAGAAGAATTCACAATCATGTCTTCCTCCAAATTAAACATCATTGATTTAAGCAACAATGATCTATCAGGGT	T/C	CTTTTCCAACATCTATCTTACAGCTCGGTTCACTTTCTGTCCTCGATGTTTCCTCTAACAGGTTGAATGGGTCGCTGCAGCTAGATAAGTTTTTGGAGCT
Ca_LG_6:31061092	CAGTAATGTAGAAGAATTCACAATCATGTCTTCCTCCAAATTAAACATCATTGATTTAAGCAACAATGATCTATCAGGGTCTTTTCCAACATCTATCTTA	A/C	CAGCTCGGTTCACTTTCTGTCCTCGATGTTTCCTCTAACAGGTTGAATGGGTCGCTGCAGCTAGATAAGTTTTTGGAGCTTAGAAATTTAACGTATCTAG
Ca_LG_6:31061099	GTAGAAGAATTCACAATCATGTCTTCCTCCAAATTAAACATCATTGATTTAAGCAACAATGATCTATCAGGGTCTTTTCCAACATCTATCTTACAGCTCG	G/A	GTTCACTTTCTGTCCTCGATGTTTCCTCTAACAGGTTGAATGGGTCGCTGCAGCTAGATAAGTTTTTGGAGCTTAGAAATTTAACGTATCTAGACCTTTC
Ca_LG_6:31061111	ACAATCATGTCTTCCTCCAAATTAAACATCATTGATTTAAGCAACAATGATCTATCAGGGTCTTTTCCAACATCTATCTTACAGCTCGGTTCACTTTCTG	G/A	TCCTCGATGTTTCCTCTAACAGGTTGAATGGGTCGCTGCAGCTAGATAAGTTTTTGGAGCTTAGAAATTTAACGTATCTAGACCTTTCATTCAATAACTT
Ca_LG_6:31061120	TCTTCCTCCAAATTAAACATCATTGATTTAAGCAACAATGATCTATCAGGGTCTTTTCCAACATCTATCTTACAGCTCGGTTCACTTTCTGTCCTCGATG	G/C	TTTCCTCTAACAGGTTGAATGGGTCGCTGCAGCTAGATAAGTTTTTGGAGCTTAGAAATTTAACGTATCTAGACCTTTCATTCAATAACTTCTCAATCAA
Ca_LG_6:31061125	CTCCAAATTAAACATCATTGATTTAAGCAACAATGATCTATCAGGGTCTTTTCCAACATCTATCTTACAGCTCGGTTCACTTTCTGTCCTCGATGTTTCC	C/G	TCTAACAGGTTGAATGGGTCGCTGCAGCTAGATAAGTTTTTGGAGCTTAGAAATTTAACGTATCTAGACCTTTCATTCAATAACTTCTCAATCAATGTGA
Ca_LG_6:32225588	TAAGCCAAGGAGTTCCTTCATGCAGAGATTAGGGATTCAATCAATGTTTATGAAGATAAAGCAACACATATCTTAAGGCTTGTTGGGTCTGCAGCTCAAC	C/T	AAGTAATGTATTAATTGAGATTCAACATGATTCTTTACTCTCAAAATGGTTACCCGACAAGGAATAAACACAAAACATCAAGCACAAAACTCTTCATAGT
Ca_LG_6:32225590	AGCCAAGGAGTTCCTTCATGCAGAGATTAGGGATTCAATCAATGTTTATGAAGATAAAGCAACACATATCTTAAGGCTTGTTGGGTCTGCAGCTCAACAA	A/G	GTAATGTATTAATTGAGATTCAACATGATTCTTTACTCTCAAAATGGTTACCCGACAAGGAATAAACACAAAACATCAAGCACAAAACTCTTCATAGTTA
Ca_LG_6:32317480	AGATTTGATGTCAACAAGTTAATCATGGTGATTAGGTGAGCTGGCAGCTGCAGTACTTGTGTCAATTTCCAAAGGAGTAGCAGAACAAGATGCAGGGGAA	A/G	AGGGGAGGGGATGCAATATTATGAAAATCATCAAATACAAGACCATTGTTATTAGGGAGAGGATTTGGTTGCTGCAAAGTAGAATTAACTATAGAATAAG
Ca_LG_6:32317500	AATCATGGTGATTAGGTGAGCTGGCAGCTGCAGTACTTGTGTCAATTTCCAAAGGAGTAGCAGAACAAGATGCAGGGGAAAGGGGAGGGGATGCAATATT	T/C	ATGAAAATCATCAAATACAAGACCATTGTTATTAGGGAGAGGATTTGGTTGCTGCAAAGTAGAATTAACTATAGAATAAGGAAATATAAACTAAAAAAAA

Positions	-100 bp flanking sequences	SNPs within *desi* cultivar	+100 bp flanking sequences
Ca_LG_6:32317501	ATCATGGTGATTAGGTGAGCTGGCAGCTGCAGTACTTGTGTCAATTTCCAAAGGAGTAGCAGAACAAGATGCAGGGGAAAGGGGAGGGGATGCAATATTA	A/G	TGAAAATCATCAAATACAAGACCATTGTTATTAGGGAGAGGATTTGGTTGCTGCAAAGTAGAATTAACTATAGAATAAGGAAATATAAACTAAAAAAAAA
Ca_LG_6:32722745	ATTTTCCCTTTCTGATATCACTCATACTGGCGAAATATGTTCAAATGATCTTGATCAACTGCAGCCTGAATTAAGTAATCCTAGCTTTCCTTCTCCTGAG	G/C	GGCGAGTATCAGCTGATGTCTCTGGATGAAAGGCTGCTGCTTGAGTTGCAGAGCATTGGTTTATATCCAGAAATATTGGTAGGGTTTTATTTCATATATG
Ca_LG_6:33379159	TTGGTGAGAGTTATGATCGTTTAAATAGTTCATCAAACTGAAGAAGTGATGGATGAATGTGAGAATTGCAGTGGCGGCGGCATCTAGCGTGGAAGAAGAT	T/C	TGCATCTGGAAGAAGGTTGCGATGCAGATTTTTAATTTGTGCAGAAGTGGTGGAGCTAGCAATTATCTAGGTGCCCAAAGTAGCAGATTTAAAATACCTA
Ca_LG_6:33379166	GAGTTATGATCGTTTAAATAGTTCATCAAACTGAAGAAGTGATGGATGAATGTGAGAATTGCAGTGGCGGCGGCATCTAGCGTGGAAGAAGATTGCATCT	T/G	GGAAGAAGGTTGCGATGCAGATTTTTAATTTGTGCAGAAGTGGTGGAGCTAGCAATTATCTAGGTGCCCAAAGTAGCAGATTTAAAATACCTAAAACAGA
Ca_LG_6:33379175	TCGTTTAAATAGTTCATCAAACTGAAGAAGTGATGGATGAATGTGAGAATTGCAGTGGCGGCGGCATCTAGCGTGGAAGAAGATTGCATCTGGAAGAAGG	G/A	TTGCGATGCAGATTTTTAATTTGTGCAGAAGTGGTGGAGCTAGCAATTATCTAGGTGCCCAAAGTAGCAGATTTAAAATACCTAAAACAGAGTGGGTCAA
Ca_LG_6:33379176	CGTTTAAATAGTTCATCAAACTGAAGAAGTGATGGATGAATGTGAGAATTGCAGTGGCGGCGGCATCTAGCGTGGAAGAAGATTGCATCTGGAAGAAGGT	T/C	TGCGATGCAGATTTTTAATTTGTGCAGAAGTGGTGGAGCTAGCAATTATCTAGGTGCCCAAAGTAGCAGATTTAAAATACCTAAAACAGAGTGGGTCAAA
Ca_LG_6:33667098	CAGGGATGTTTGGATAGATGTCAAGGTAGCTTCCATCCTTGCATTTGACTCTAATATTTTGGCGTTGTGTTCCATTTGAGCTGCCATAAATTCCATCAGC	C/T	TTTGAAACAAATGCAGGAGCCTCAGCTGCTGATTCAGATGCTTGTGGAGGAGTTGGTTCAACATTTGGCATTGGTCTAATCCAAACATTATCTACTTTCT
Ca_LG_6:33667124	TAGCTTCCATCCTTGCATTTGACTCTAATATTTTGGCGTTGTGTTCCATTTGAGCTGCCATAAATTCCATCAGCTTTGAAACAAATGCAGGAGCCTCAGC	C/T	TGCTGATTCAGATGCTTGTGGAGGAGTTGGTTCAACATTTGGCATTGGTCTAATCCAAACATTATCTACTTTCTTCAGTTTCATCTGATTCACAATCGCT
Ca_LG_6:33837911	TGATTGTCGATCACAATTTTTTGGTGTTGCGATTGTGCTTTTGGGTTGTTTCTCGCTGTTGCTCGTTACTGCGTTTCTGGGTCATTCTCGCTGCTTGTTG	G/A	ATCGGTTTCTGCTCAGCCGTTGTTGCTTGCGTGATCGCGTTTGGTTGTCTCGTGGAAATTTTTTTGTTACAGACCTCTTGTTTAAGAACACATTTGATTC
Ca_LG_6:35297705	GGATCTTGCTTAGGACAGAGGGGACACAACCACCCAAAAAAAAAAGTTTGGTAAAATGACCAAAATGCTCTTATTAAAATTACAAAATTACAATAATACT	T/C	TAAACCCTAAATCATATCATCTCTCTTCCTTTCTCTCCTGTGCGCTGCTCTCTCTTCCTCTCTCACTCTCCTGTTGTGTTGCGTCGCGTCGCTGTCGTGC
Ca_LG_6:35297710	TTGCTTAGGACAGAGGGGACACAACCACCCAAAAAAAAAAGTTTGGTAAAATGACCAAAATGCTCTTATTAAAATTACAAAATTACAATAATACTTAAAC	C/T	CCTAAATCATATCATCTCTCTTCCTTTCTCTCCTGTGCGCTGCTCTCTCTTCCTCTCTCACTCTCCTGTTGTGTTGCGTCGCGTCGCTGTCGTGCCTCCC
Ca_LG_6:35297711	TGCTTAGGACAGAGGGGACACAACCACCCAAAAAAAAAAGTTTGGTAAAATGACCAAAATGCTCTTATTAAAATTACAAAATTACAATAATACTTAAACC	C/T	CTAAATCATATCATCTCTCTTCCTTTCTCTCCTGTGCGCTGCTCTCTCTTCCTCTCTCACTCTCCTGTTGTGTTGCGTCGCGTCGCTGTCGTGCCTCCCT

Positions	-100 bp flanking sequences	SNPs within *desi* cultivar	+100 bp flanking sequences
Ca_LG_6:35297746	AAAAGTTTGGTAAAATGACCAAAATGCTCTTATTAAAATTACAAAATTACAATAATACTTAAACCCTAAATCATATCATCTCTCTTCCTTTCTCTCCTGT	T/C	GCGCTGCTCTCTCTTCCTCTCTCACTCTCCTGTTGTGTTGCGTCGCGTCGCTGTCGTGCCTCCCTCTTGCCGTCTTCACCGCTGCTACCTCGCCTCGGTC
Ca_LG_6:35297751	TTTGGTAAAATGACCAAAATGCTCTTATTAAAATTACAAAATTACAATAATACTTAAACCCTAAATCATATCATCTCTCTTCCTTTCTCTCCTGTGCGCT	T/G	GCTCTCTCTTCCTCTCTCACTCTCCTGTTGTGTTGCGTCGCGTCGCTGTCGTGCCTCCCTCTTGCCGTCTTCACCGCTGCTACCTCGCCTCGGTCGTTTG
Ca_LG_6:35297779	TAAAATTACAAAATTACAATAATACTTAAACCCTAAATCATATCATCTCTCTTCCTTTCTCTCCTGTGCGCTGCTCTCTCTTCCTCTCTCACTCTCCTGT	T/C	TGTGTTGCGTCGCGTCGCTGTCGTGCCTCCCTCTTGCCGTCTTCACCGCTGCTACCTCGCCTCGGTCGTTTGTCGTCAAAACTCCAAAGGTTTGGATTTT
Ca_LG_6:35791158	GTACTGTCAACCACAGAGCTGTTGAATTAAGAGTGATAAAATAACTTAAATTTTCTCGTACTTAATTCAATGGCTTTATAAGTGCTGCAGAAGATCCAGA	A/G	CACAAAAATGATAGGTTTAAATATAGGAGATAGAGAGTAAAAGTATGACAGAGAGAGAGAGAGAATGTAGATAGCGCAGCGAAAAATAGAATGCAAATTATC
Ca_LG_6:36295595	AGACATGGTAGTTGAAGTATAGTTAATTGGTGCTATGTTTGGGTTGGTGACGTGGACGGTTAATAGAACTTCGGCGTCTACGACAGGTATGTTTAGTTTG	G/T	AAGGAAGTGAAGTTGATGGAGATAAGATGGAAGGTTGGGTCTTTTGGTTTTGCTGAGAGAATTGAGGTTGCTGCTACTGCTGAGGCTGCTCCCACTATTG
Ca_LG_6:36295605	GTTGAAGTATAGTTAATTGGTGCTATGTTTGGGTTGGTGACGTGGACGGTTAATAGAACTTCGGCGTCTACGACAGGTATGTTTAGTTTGAAGGAAGTGA	A/T	AGTTGATGGAGATAAGATGGAAGGTTGGGTCTTTTGGTTTTGCTGAGAGAATTGAGGTTGCTGCTACTGCTGAGGCTGCTCCCACTATTGCTGATGTCCA
Ca_LG_6:37115683	CTCCCCAAGAAGGAACGACGCTGCAAAGGTCAACATCAATAGGAATGATCAAAAGGTTGAAGCAATGAATAACGCTTATGTTGATGCACAGACAGCTCCC	C/G	AGGACTCTACAAGATCTGGAGAAGAGGGAAAGAGAGATATGTGCTGCTGTGTTAGGGGGATTGGAGGATTTTCGTTGTTACCATCCTCCAAAGTTCAAGG
Ca_LG_6:37115694	GGAACGACGCTGCAAAGGTCAACATCAATAGGAATGATCAAAAGGTTGAAGCAATGAATAACGCTTATGTTGATGCACAGACAGCTCCCAGGACTCTACA	A/G	AGATCTGGAGAAGAGGGAAAGAGAGATATGTGCTGCTGTGTTAGGGGGATTGGAGGATTTTCGTTGTTACCATCCTCCAAAGTTCAAGGGTGGTGATAAC
Ca_LG_6:37224650	CGACAACAATATCCTGCGTTTTTGTTTTGACATAGCAGCAACTATCATCTCCCAACATTTTCATGCACTGCCTCCCAACATCCTACCCCTTACCAAATCA	A/G	GACTCCAAACACATTTGGTGTGGCGACTTTATCCCTCAACATAGACTTGTGATAAAATATGTCCTTAAATGAAACTGAGGCAAACCCTTGGATTTGTATC
Ca_LG_6:37224656	CAATATCCTGCGTTTTTGTTTTGACATAGCAGCAACTATCATCTCCCAACATTTTCATGCACTGCCTCCCAACATCCTACCCCTTACCAAATCAGACTCC	C/T	AAACACATTTGGTGTGGCGACTTTATCCCTCAACATAGACTTGTGATAAAATATGTCCTTAAATGAAACTGAGGCAAACCCTTGGATTTGTATCCGATGA
Ca_LG_6:37224668	TTTTTGTTTTGACATAGCAGCAACTATCATCTCCCAACATTTTCATGCACTGCCTCCCAACATCCTACCCCTTACCAAATCAGACTCCAAACACATTTGG	G/T	TGTGGCGACTTTATCCCTCAACATAGACTTGTGATAAAATATGTCCTTAAATGAAACTGAGGCAAACCCTTGGATTTGTATCCGATGATTAATGGACTAC
Ca_LG_6:37224671	TTGTTTTGACATAGCAGCAACTATCATCTCCCAACATTTTCATGCACTGCCTCCCAACATCCTACCCCTTACCAAATCAGACTCCAAACACATTTGGTGT	T/C	GGCGACTTTATCCCTCAACATAGACTTGTGATAAAATATGTCCTTAAATGAAACTGAGGCAAACCCTTGGATTTGTATCCGATGATTAATGGACTACGGC

Positions	-100 bp flanking sequences	SNPs within *desi* cultivar	+100 bp flanking sequences
Ca_LG_6:37224694	TCATCTCCCAACATTTTCATGCACTGCCTCCCAACATCCTACCCCTTACCAAATCAGACTCCAAACACATTTGGTGTGGCGACTTTATCCCTCAACATAG	G/A	ACTTGTGATAAAATATGTCCTTAAATGAAACTGAGGCAAACCCTTGGATTTGTATCCGATGATTAATGGACTACGGCAATCCCTTCACTTTGCTATTGAG
Ca_LG_6:37224695	CATCTCCCAACATTTTCATGCACTGCCTCCCAACATCCTACCCCTTACCAAATCAGACTCCAAACACATTTGGTGTGGCGACTTTATCCCTCAACATAGA	A/T	CTTGTGATAAAATATGTCCTTAAATGAAACTGAGGCAAACCCTTGGATTTGTATCCGATGATTAATGGACTACGGCAATCCCTTCACTTTGCTATTGAGC
Ca_LG_6:37225042	TTTTATAGCCCTCACTTCTTGACCTAATGGGCCCTTCTTTGGAAGTCCAATCCCATTAAGATAAATCTCTAAGATCCACACCACTTGTCCTACGAGTATT	T/A	ACAGCGCATGAAGCTGGCTGCCCCTTCTCCATGCAGTTTTTCCTTACAGGGGGCATGGAGCTGGAGCATGCGTAGCTCATGTTACTCGTCTGTGTAGGGA
Ca_LG_6:37371714	TTATATAAGCATATTTGATGAACAGTAGCTAGTGTGTTAAGTCATGATTCTTATCTAAGGCTGATAATTGGGCTTATAATGCCTTTGTTTATGGACCTCA	A/G	AGACCTTTCATTTCCTACTTCGGTCTATCATCTTATGTTTTTTTATCCAATGGAAGATTCTACATGGGAAACAGCTTCTTCCAATCACAATCAGTATACA
Ca_LG_6:37371715	TATATAAGCATATTTGATGAACAGTAGCTAGTGTGTTAAGTCATGATTCTTATCTAAGGCTGATAATTGGGCTTATAATGCCTTTGTTTATGGACCTCAA	A/G	GACCTTTCATTTCCTACTTCGGTCTATCATCTTATGTTTTTTTATCCAATGGAAGATTCTACATGGGAAACAGCTTCTTCCAATCACAATCAGTATACAA
Ca_LG_6:37410867	AAGCAAGCCATTTGTAGCCGCCCAGGCCGCTACAAAAGCATTGATCCATTGCACTTTGTGGCTGCAAAGGCCGCCACAAATTCCCATAGTTCCGTTGCAC	C/A	TTTGTGGCCGCCCAGGCCGCCACAAAAGGCTTCATTTCTAACTGTTAAAGCCGCAACAAATTCCCATTGTACCTTTGGAATTTGTGGCTGCTTTGGTCGC
Ca_LG_6:38477318	TGACATCTTGGTTTATAGTAGAGATGAGGCTGCACATGAAGATCATTTGCGGAAAGTGCTGGAAGCATTAAGAGGAAATTCCTTACTGGTCAACAAAAAA	A/T	AAATGTAGTTTTGTTCAGCCAAGCCTAGAGTATTTGGGTCATGTTATCTCTGAGGCAGGAGTGGCAGCCGACCAGACAAAGGTAGAGGCTGTAAAATCTT
Ca_LG_6:38706508	ATCATTCACATTGATTGAAATGTTATTGAATGAAAGGTCTATTGCAGTTAAATTTATAAGCTTCAAAAGCTCATCTAGCTGCAGTGAACCATTCAACCTA	A/G	TTAGAGGAAAGATCCAAGAAAGAAAGTGAACTGAGCTGGAAGATAGATATTGGAAAAGGCCCTGATAGACTATTGCTACTTAAATCAAGAGTGTTCACTA
Ca_LG_6:38706514	CACATTGATTGAAATGTTATTGAATGAAAGGTCTATTGCAGTTAAATTTATAAGCTTCAAAAGCTCATCTAGCTGCAGTGAACCATTCAACCTATTAGAG	G/C	GAAAGATCCAAGAAAGAAAGTGAACTGAGCTGGAAGATAGATATTGGAAAAGGCCCTGATAGACTATTGCTACTTAAATCAAGAGTGTTCACTACAGATG
Ca_LG_6:38706523	TGAAATGTTATTGAATGAAAGGTCTATTGCAGTTAAATTTATAAGCTTCAAAAGCTCATCTAGCTGCAGTGAACCATTCAACCTATTAGAGGAAAGATCC	C/G	AAGAAAGAAAGTGAACTGAGCTGGAAGATAGATATTGGAAAAGGCCCTGATAGACTATTGCTACTTAAATCAAGAGTGTTCACTACAGATGAAGACAAAA
Ca_LG_6:38706525	AAATGTTATTGAATGAAAGGTCTATTGCAGTTAAATTTATAAGCTTCAAAAGCTCATCTAGCTGCAGTGAACCATTCAACCTATTAGAGGAAAGATCCAA	A/G	GAAAGAAAGTGAACTGAGCTGGAAGATAGATATTGGAAAAGGCCCTGATAGACTATTGCTACTTAAATCAAGAGTGTTCACTACAGATGAAGACAAAATT
Ca_LG_6:38706528	TGTTATTGAATGAAAGGTCTATTGCAGTTAAATTTATAAGCTTCAAAAGCTCATCTAGCTGCAGTGAACCATTCAACCTATTAGAGGAAAGATCCAAGAA	A/T	AGAAAGTGAACTGAGCTGGAAGATAGATATTGGAAAAGGCCCTGATAGACTATTGCTACTTAAATCAAGAGTGTTCACTACAGATGAAGACAAAATTATG

**Positions**	**-100 bp flanking sequences**	**SNPs within *desi* cultivar**	**+100 bp flanking sequences**
Ca_LG_6:38706557	AAATTTATAAGCTTCAAAAGCTCATCTAGCTGCAGTGAACCATTCAACCTATTAGAGGAAAGATCCAAGAAAGAAAGTGAACTGAGCTGGAAGATAGATA	A/G	TTGGAAAAGGCCCTGATAGACTATTGCTACTTAAATCAAGAGTGTTCACTACAGATGAAGACAAAATTATGAACTTGTCAAATTTACTAAACTGGTTGAA
Ca_LG_6:38706563	ATAAGCTTCAAAAGCTCATCTAGCTGCAGTGAACCATTCAACCTATTAGAGGAAAGATCCAAGAAAGAAAGTGAACTGAGCTGGAAGATAGATATTGGAA	A/G	AAGGCCCTGATAGACTATTGCTACTTAAATCAAGAGTGTTCACTACAGATGAAGACAAAATTATGAACTTGTCAAATTTACTAAACTGGTTGAATGAAAG
Ca_LG_6:39811116	TGTAACTAATGAAAAAAAATTACAAGAAAAATAATACAAGTCGTAATGACATCGGTTAAACAAGAAAGTTGATGTAGAGAGTGGTAGCAGCCGATGTTCG	G/A	TCAATCTCTCGTCGAACTCTTCTGTCTACTTTGAGCTAACTTGAAAATCTTTTAGTCACTACTAGAAAAATCCATTTTAGCTGCCATATTTAGCATCGGC
Ca_LG_6:39811133	AATTACAAGAAAAATAATACAAGTCGTAATGACATCGGTTAAACAAGAAAGTTGATGTAGAGAGTGGTAGCAGCCGATGTTCGTCAATCTCTCGTCGAAC	C/G	TCTTCTGTCTACTTTGAGCTAACTTGAAAATCTTTTAGTCACTACTAGAAAAATCCATTTTAGCTGCCATATTTAGCATCGGCCCCCGACTCCGACGCTA
Ca_LG_6:39811189	GTAGAGAGTGGTAGCAGCCGATGTTCGTCAATCTCTCGTCGAACTCTTCTGTCTACTTTGAGCTAACTTGAAAATCTTTTAGTCACTACTAGAAAAATCC	C/T	ATTTTAGCTGCCATATTTAGCATCGGCCCCCGACTCCGACGCTAGTTAGCGTCGGTGTAGCGTCAGTCAAGCAAACGCTAAAGTGTCAAATTTTAGTTCT
Ca_LG_6:42594667	TCAGTTTTTACCGCTCCTAACTAGAAAAGTCGAATGCAGCCGCGAAGACATCATCTCTCATGGCGCTATACTATGATAGGCCCTTCGGAATTATGGGGTG	G/T	CCCGAGTCCTACATCGAGTAGTGCAGGATGCTCAGTGAAGTGTTTAAGTGACATGGTTGTGCCTCATGATAGCTAGTTGTTAAGGAAGGGTTTTCCCGAG
Ca_LG_6:42594670	GTTTTTACCGCTCCTAACTAGAAAAGTCGAATGCAGCCGCGAAGACATCATCTCTCATGGCGCTATACTATGATAGGCCCTTCGGAATTATGGGGTGCCC	C/T	GAGTCCTACATCGAGTAGTGCAGGATGCTCAGTGAAGTGTTTAAGTGACATGGTTGTGCCTCATGATAGCTAGTTGTTAAGGAAGGGTTTTCCCGAGTAT
Ca_LG_6:42594682	CCTAACTAGAAAAGTCGAATGCAGCCGCGAAGACATCATCTCTCATGGCGCTATACTATGATAGGCCCTTCGGAATTATGGGGTGCCCGAGTCCTACATC	C/T	GAGTAGTGCAGGATGCTCAGTGAAGTGTTTAAGTGACATGGTTGTGCCTCATGATAGCTAGTTGTTAAGGAAGGGTTTTCCCGAGTATTGCATAATGCAT
Ca_LG_6:42999386	TTGTCATTAGACACAAGAAACTCAGAAATTTCTCATATTTTTACATGGTATCAGAGCAGGTTCTGATTTTGACATATCTCTAGGTTTCACTACAGCCGCT	T/C	GGCAGCATCATAACCACGGTTTTTCCTAGTTTTTCCACATTTATGTCCTTTTTTATACTGTAACAGCTGTGCCAACACCAGCCCCCCTTTTCCAGTGCGA
Ca_LG_6:45576037	CTTAGGAGCAAAAGGGCTTCTCCCTATGAAAATGAAAGATGGAAATGGTAGCACAGATTCTTATTGTGTTGCAAAATATGGTCAGAAATGGATCAGAACA	A/T	AGAACACTTTTAGACACTTTTAGTCCTAAATGGAATGAACAATACACATGGGAGGTTTATGATCCTTGCACTGTCATAACATTAGGTGTTTTTGACAATT
Ca_LG_6:45576044	GCAAAAGGGCTTCTCCCTATGAAAATGAAAGATGGAAATGGTAGCACAGATTCTTATTGTGTTGCAAAATATGGTCAGAAATGGATCAGAACAAGAACAC	C/A	TTTTAGACACTTTTAGTCCTAAATGGAATGAACAATACACATGGGAGGTTTATGATCCTTGCACTGTCATAACATTAGGTGTTTTTGACAATTGCCATTT
Ca_LG_6:45576047	AAAGGGCTTCTCCCTATGAAAATGAAAGATGGAAATGGTAGCACAGATTCTTATTGTGTTGCAAAATATGGTCAGAAATGGATCAGAACAAGAACACTTT	T/C	TAGACACTTTTAGTCCTAAATGGAATGAACAATACACATGGGAGGTTTATGATCCTTGCACTGTCATAACATTAGGTGTTTTTGACAATTGCCATTTAGG

Positions	-100 bp flanking sequences	SNPs within *desi* cultivar	+100 bp flanking sequences
Ca_LG_6:45576049	AGGGCTTCTCCCTATGAAAATGAAAGATGGAAATGGTAGCACAGATTCTTATTGTGTTGCAAAATATGGTCAGAAATGGATCAGAACAAGAACACTTTTA	A/T	GACACTTTTAGTCCTAAATGGAATGAACAATACACATGGGAGGTTTATGATCCTTGCACTGTCATAACATTAGGTGTTTTTGACAATTGCCATTTAGGTG
Ca_LG_6:45576076	TGGAAATGGTAGCACAGATTCTTATTGTGTTGCAAAATATGGTCAGAAATGGATCAGAACAAGAACACTTTTAGACACTTTTAGTCCTAAATGGAATGAA	A/G	CAATACACATGGGAGGTTTATGATCCTTGCACTGTCATAACATTAGGTGTTTTTGACAATTGCCATTTAGGTGGAGGAGATAAAAAAGCTTCGAATGGAA
Ca_LG_6:45764396	ACTGAAATCCGTAGGCCAGGCTCAGACCTTTCAAAGTCTGACCTATTACCACCCCTAATTGCACATCACACCATAGAGCGACATGTGGTAAGCTCGTTTT	T/C	TAAGGCCAAACCATAAGCAGCACCTCTCGAATCAGGAAATATCCTCGGAAAACCACCATGTGCTATGACCAACGGCAGATTACCTGCAAAACAATCAACA
Ca_LG_6:45905256	CCTCCCAAGGGCAGGTTCTTACGCGTTACTCACCCGTCCGCCACTGGAAACACCACTTCCCGTCCGACTTGCATGTGTAAAGCATGCCGCCAGCGTTCAT	T/A	CCTGAGCCAGGATCAAACTCTCCATGAGATTCCTAGTTGCATTACTTATAGCTTCCTTGCTCGTAGACAAAGCTGATTCGGAATTTTATTTCATTTCAAG
Ca_LG_6:45905257	CTCCCAAGGGCAGGTTCTTACGCGTTACTCACCCGTCCGCCACTGGAAACACCACTTCCCGTCCGACTTGCATGTGTAAAGCATGCCGCCAGCGTTCATC	C/T	CTGAGCCAGGATCAAACTCTCCATGAGATTCCTAGTTGCATTACTTATAGCTTCCTTGCTCGTAGACAAAGCTGATTCGGAATTTTATTTCATTTCAAGG
Ca_LG_6:46091122	GAAGATGGGTGACAGAGATCAAAGACTCTATACAGAAAATTAGGCTCTGGCTCGGTACATTTGATACAGCTGAAGATGCAGCTCGTGCTTATGACACTGC	C/T	TGCATGAGCTTTAAGAGGTGCTAATGCATGAACAAACTTTGATTTGCCACAAGCTGCAACCAGTGGTGTTGGTGGTGGTGCCAAACGTGGTGCTAGTTCA
Ca_LG_6:46544703	ATACTCCCATGTTTAATTTTGTTAAGTATTAACTTCAGAGTTGTATGCAATATTTTTGTTCACTACATTATTTTACTGTTCTGATTATTTTCCACCCAAT	T/C	AAAAAGTATCTCTCTACCAACACAAGGCATTGAAGTACTTCAAACCTGTTGCGGGAATGGGAATTCATTTATACGTAGCACATTAATTAATTTATTGATA
Ca_LG_6:46544744	TGTATGCAATATTTTTGTTCACTACATTATTTTACTGTTCTGATTATTTTCCACCCAATAAAAAGTATCTCTCTACCAACACAAGGCATTGAAGTACTTC	C/T	AAACCTGTTGCGGGAATGGGAATTCATTTATACGTAGCACATTAATTAATTTATTGATACAAAAACAAAAAAAAAAGACAACATGCAGCATATTGCTAGT
Ca_LG_6:46647588	CAGTCAAACTAAATTGATCACCATGAGTTGTAGCTTGATTGTTTGAATCTTGATCATCATGAGTTGCAGCTTGATTGTTTAAAGCATGATCATCATGAGT	T/C	TGCAGCTTGATTGTTTGAAGCTTGATCATTATTTGCAGCAGCTTGATGATCATGAGTTGAAGATTGATCATCATGAATTGCAGTTTGATCATGAGTTGCA
Ca_LG_6:46647651	TTGCAGCTTGATTGTTTAAAGCATGATCATCATGAGTTGCAGCTTGATTGTTTGAAGCTTGATCATTATTTGCAGCAGCTTGATGATCATGAGTTGAAGA	A/C	TTGATCATCATGAATTGCAGTTTGATCATGAGTTGCAGCTTGATCATCATGAATAGTTGGCAAAAATCCAGATGTTGGTGAAGGGCAATTAGTATGTCCT
Ca_LG_6:46647662	TTGTTTAAAGCATGATCATCATGAGTTGCAGCTTGATTGTTTGAAGCTTGATCATTATTTGCAGCAGCTTGATGATCATGAGTTGAAGATTGATCATCAT	T/C	GAATTGCAGTTTGATCATGAGTTGCAGCTTGATCATCATGAATAGTTGGCAAAAATCCAGATGTTGGTGAAGGGCAATTAGTATGTCCTTCTACAAATAG
Ca_LG_6:46979685	AAGAAATATGGAGAAGTCGGTAACTGATTTTCTGGAATCGGGGCATGCTGCTCGGTCACTATCACTAAAAGTTGAAATGTTGAAGAAGAAGAGAAGAATG	G/A	AACCTTTTCCGGGGCAGCCCTTAATATAATGAAGAATACGAGTAGCAACATGAAAATGTGAAGAAATAGGATTTGATACAAATTGACTAAGTTTGTGAGT

Positions	-100 bp flanking sequences	SNPs within *desi* cultivar	+100 bp flanking sequences
Ca_LG_6:46979688	AAATATGGAGAAGTCGGTAACTGATTTTCTGGAATCGGGGCATGCTGCTCGGTCACTATCACTAAAAGTTGAAATGTTGAAGAAGAAGAGAAGAATGAAC	C/T	CTTTTCCGGGGCAGCCCTTAATATAATGAAGAATACGAGTAGCAACATGAAAATGTGAAGAAATAGGATTTGATACAAATTGACTAAGTTTGTGAGTAGA
Ca_LG_6:46979693	TGGAGAAGTCGGTAACTGATTTTCTGGAATCGGGGCATGCTGCTCGGTCACTATCACTAAAAGTTGAAATGTTGAAGAAGAAGAGAAGAATGAACCTTTT	T/C	CCGGGGCAGCCCTTAATATAATGAAGAATACGAGTAGCAACATGAAAATGTGAAGAAATAGGATTTGATACAAATTGACTAAGTTTGTGAGTAGAAAAGG
Ca_LG_6:46979694	GGAGAAGTCGGTAACTGATTTTCTGGAATCGGGGCATGCTGCTCGGTCACTATCACTAAAAGTTGAAATGTTGAAGAAGAAGAGAAGAATGAACCTTTTC	C/A	CGGGGCAGCCCTTAATATAATGAAGAATACGAGTAGCAACATGAAAATGTGAAGAAATAGGATTTGATACAAATTGACTAAGTTTGTGAGTAGAAAAGGA
Ca_LG_6:46979695	GAGAAGTCGGTAACTGATTTTCTGGAATCGGGGCATGCTGCTCGGTCACTATCACTAAAAGTTGAAATGTTGAAGAAGAAGAGAAGAATGAACCTTTTCC	C/T	GGGGCAGCCCTTAATATAATGAAGAATACGAGTAGCAACATGAAAATGTGAAGAAATAGGATTTGATACAAATTGACTAAGTTTGTGAGTAGAAAAGGAG
Ca_LG_6:46979700	GTCGGTAACTGATTTTCTGGAATCGGGGCATGCTGCTCGGTCACTATCACTAAAAGTTGAAATGTTGAAGAAGAAGAGAAGAATGAACCTTTTCCGGGGC	C/T	AGCCCTTAATATAATGAAGAATACGAGTAGCAACATGAAAATGTGAAGAAATAGGATTTGATACAAATTGACTAAGTTTGTGAGTAGAAAAGGAGAGATT
Ca_LG_6:48646958	GTGGCGGCCAAAGCAGCCACAATGAGCAACTGATATACGACGTTTGTGGCGGCCAAAACGGTCAGAAATGCAGGGATTTGTGGGGGCCTGGGCGGCCACT	T/A	ACTGGCAACGGTCCTTTGGGTGTTTGTGGCGGCCTTTGCAGCCACAAAAGGTAACAGATATAAAACTTTGTGGCGGCCTGGGCGGCCAAAAAAAGCAACG
Ca_LG_6:48647035	TTGTGGGGGCCTGGGCGGCCACTACTGGCAACGGTCCTTTGGGTGTTTGTGGCGGCCTTTGCAGCCACAAAAGGTAACAGATATAAAACTTTGTGGCGGC	C/T	CTGGGCGGCCAAAAAAAGCAACGTCACTTTATTCGTTTGTGGCGGCCTTTGCAGCCATAAAAGGTAACGACTCAGGCTTTTGTGGCGGCCTTTGCAGCCA
Ca_LG_6:48647044	CCTGGGCGGCCACTACTGGCAACGGTCCTTTGGGTGTTTGTGGCGGCCTTTGCAGCCACAAAAGGTAACAGATATAAAACTTTGTGGCGGCCTGGGCGGC	C/A	CAAAAAAAGCAACGTCACTTTATTCGTTTGTGGCGGCCTTTGCAGCCATAAAAGGTAACGACTCAGGCTTTTGTGGCGGCCTTTGCAGCCACAAAGGGTA
Ca_LG_6:48647052	GCCACTACTGGCAACGGTCCTTTGGGTGTTTGTGGCGGCCTTTGCAGCCACAAAAGGTAACAGATATAAAACTTTGTGGCGGCCTGGGCGGCCAAAAAAA	A/G	GCAACGTCACTTTATTCGTTTGTGGCGGCCTTTGCAGCCATAAAAGGTAACGACTCAGGCTTTTGTGGCGGCCTTTGCAGCCACAAAGGGTAACATCACT
Ca_LG_6:48647057	TACTGGCAACGGTCCTTTGGGTGTTTGTGGCGGCCTTTGCAGCCACAAAAGGTAACAGATATAAAACTTTGTGGCGGCCTGGGCGGCCAAAAAAAGCAAC	C/T	GTCACTTTATTCGTTTGTGGCGGCCTTTGCAGCCATAAAAGGTAACGACTCAGGCTTTTGTGGCGGCCTTTGCAGCCACAAAGGGTAACATCACTTTAGG
Ca_LG_6:48711123	GGTTTTGTGTTGGGCTGCAGAGTGGATGAAGATGCGCAAAATCTATTTTTTGATTGCCCCATGGCTGCTGGATGTGGGGGGATGTTTTGAAGTGGTGGAG	G/A	TTTTTAGTGTGCTCTTCCAATTAGTGTGTTGGGTCATGCAATTCAATGTAGTGAATTAGGGAGGCAGCGAAGGAATAGCCTACAAGTCTTGAATCTTATT
Ca_LG_6:48711144	GTGGATGAAGATGCGCAAAATCTATTTTTTGATTGCCCCATGGCTGCTGGATGTGGGGGGATGTTTTGAAGTGGTGGAGTTTTTAGTGTGCTCTTCCAAT	T/A	TAGTGTGTTGGGTCATGCAATTCAATGTAGTGAATTAGGGAGGCAGCGAAGGAATAGCCTACAAGTCTTGAATCTTATTTGGTTAGCAATTGTTTGGTCC

Positions	-100 bp flanking sequences	SNPs within *desi* cultivar	+100 bp flanking sequences
Ca_LG_6:49096275	CCACCAGCTTCACTCCTAGCTTTTCACAAAGGTAATTCAATCTCATTTCATCACCGCCCCTTACTTCTCGTAGATCAAGAACCTGTTGTCTTGACAGCAG	G/A	AGTGTAAACATGAAGCTGCTGCTGCTGATTAAAATGTGACTGAAGCTGCTGAAAAAGTAATTTGTTTGAGTGTTGTTGTTGGTCTTGTTTGGCAACTTCG
Ca_LG_6:49097971	CTTGAAGTCAAGGAAATTAAAATTGGAGAACTGAGGTTGGTGGTTTCTGTGGTGAAAATTGTTATGAACAGTATCAATAGCAGTAGAATTAATTAATTAT	T/G	CTACTCAATGGTCAAAATGTACTGTAGAAGAGAATGGCAGCCTTGGACTTATTGATGAGCTACTTCATACTCAAGTGCAGCCAGAGTAGAGACAGTGACC
Ca_LG_6:49099323	CCTATTCCCTACCTCCATTTTCTTAGTATAAACCCGTCCACCAGCCCGTTTCCTCCCTTCCAACACCGTCACTTTAAACCCAAACCGCATCAACTGCCTC	C/T	GCAGCAGCCAACCCAGCAAGGCCAGCACCAATTATAATCACACCAGGCTTACTAGGTTCGGTCGGAATTTTATCCTTAATCGGCGAAGCAACACCAAAAT
Ca_LG_6:49353378	ATCAGCATCAATGCTGTTGGAATTATCTGCAGCAGCCCGATGTTCGTCGTATTTGCCATTATATTGGTAGATTTCCAACTCACCCCAAGGTGTCGGTGTA	A/C	ACTTCTTCGGTTAAGTCAAACCATCTTGGTGTGAATTTATGCCCTTTTTCCTCCCGGGCTCTCTTTTCAGCACGCTGCCTCTCCTCCAAACTGAAAAGAA
Ca_LG_6:50237470	ATAGATAACAAAATATAATAAAAAAAGTATAGATAACAGAATAAAAATGAGTGTTTTTGCTGCCAAATTATTTGAAGCCAAATTATTGCAGCCAAAGTAT	T/A	AGATAACAAGTTATTGCAGTCAAATTATTTGAACAAATGCGTGTTTTTGTTCAAACTATTTGCAGCCAAATTCTTTTCATGTTTTTGTCCAAATTATATG
Ca_LG_6:50237477	ACAAAATATAATAAAAAAAGTATAGATAACAGAATAAAAATGAGTGTTTTTGCTGCCAAATTATTTGAAGCCAAATTATTGCAGCCAAAGTATAGATAAC	C/T	AAGTTATTGCAGTCAAATTATTTGAACAAATGCGTGTTTTTGTTCAAACTATTTGCAGCCAAATTCTTTTCATGTTTTTGTCCAAATTATATGCAGCCAA
Ca_LG_6:50237514	AAATGAGTGTTTTTGCTGCCAAATTATTTGAAGCCAAATTATTGCAGCCAAAGTATAGATAACAAGTTATTGCAGTCAAATTATTTGAACAAATGCGTGT	T/C	TTTTGTTCAAACTATTTGCAGCCAAATTCTTTTCATGTTTTTGTCCAAATTATATGCAGCCAAAGTATAGATAACAAGTTATTTGCAGCCAAATAATTTG
Ca_LG_6:50237521	TGTTTTTGCTGCCAAATTATTTGAAGCCAAATTATTGCAGCCAAAGTATAGATAACAAGTTATTGCAGTCAAATTATTTGAACAAATGCGTGTTTTTGTT	T/C	CAAACTATTTGCAGCCAAATTCTTTTCATGTTTTTGTCCAAATTATATGCAGCCAAAGTATAGATAACAAGTTATTTGCAGCCAAATAATTTGGACAAAT
Ca_LG_6:50237609	CGTGTTTTTGTTCAAACTATTTGCAGCCAAATTCTTTTCATGTTTTTGTCCAAATTATATGCAGCCAAAGTATAGATAACAAGTTATTTGCAGCCAAATA	A/T	ATTTGGACAAATTAGTGTTTTTGTCCAAATTATTTGCAGCCAAATTATTTGGAGAAATTATTGCAGCCAAAGTATAGATAACAAGTTATTGCAGCCAAAT
Ca_LG_6:50237614	TTTTGTTCAAACTATTTGCAGCCAAATTCTTTTCATGTTTTTGTCCAAATTATATGCAGCCAAAGTATAGATAACAAGTTATTTGCAGCCAAATAATTTG	G/T	GACAAATTAGTGTTTTTGTCCAAATTATTTGCAGCCAAATTATTTGGAGAAATTATTGCAGCCAAAGTATAGATAACAAGTTATTGCAGCCAAATTATTT
Ca_LG_6:50237632	CAGCCAAATTCTTTTCATGTTTTTGTCCAAATTATATGCAGCCAAAGTATAGATAACAAGTTATTTGCAGCCAAATAATTTGGACAAATTAGTGTTTTTG	G/A	TCCAAATTATTTGCAGCCAAATTATTTGGAGAAATTATTGCAGCCAAAGTATAGATAACAAGTTATTGCAGCCAAATTATTTGGACAAATGAGTGTTTTT
Ca_LG_6:50237663	TTATATGCAGCCAAAGTATAGATAACAAGTTATTTGCAGCCAAATAATTTGGACAAATTAGTGTTTTTGTCCAAATTATTTGCAGCCAAATTATTTGGAG	G/C	AAATTATTGCAGCCAAAGTATAGATAACAAGTTATTGCAGCCAAATTATTTGGACAAATGAGTGTTTTTGTCCAAATTATTTGCAGTCAAATTCTTTTCA

Positions	-100 bp flanking sequences	SNPs within *desi* cultivar	+100 bp flanking sequences
Ca_LG_6:50237686	AACAAGTTATTTGCAGCCAAATAATTTGGACAAATTAGTGTTTTTGTCCAAATTATTTGCAGCCAAATTATTTGGAGAAATTATTGCAGCCAAAGTATAG	G/T	ATAACAAGTTATTGCAGCCAAATTATTTGGACAAATGAGTGTTTTTGTCCAAATTATTTGCAGTCAAATTCTTTTCATATTTTTGTCCAAATTATTAAGC
Ca_LG_6:50240831	TTGTGTTGATAAAATTGGATTTGAAGAGAAAGTTGGAGCGTCGTAATATGATTTGAATATGGCGGAAAACAGCATTGAAACCATTCCGGCGAAGATGATT	T/A	CCTACACCTTGCATGGCGAAAACTGCTGCTATGAAAGCGCCACGTGTCCTTTTGTTGGCGTATTCTGACATGATTGTTGCAGATAAAGGGTAGTCTCCTC
Ca_LG_6:50356695	TTGTCATGTAACAATTAAAGTATTAATTACGGAAAATGGTTTTGAGATTTACCTTATTATGAGGAGCAGCACAAAAACCACAAGTAGGTGAAGAAGCCTG	G/T	TAAACACCTCCTACAATTCCATCTATCATTGTGAGGTCTCAAAACCATAGGTAAGTGAATTTGAGTCTCATGAAAAACAACAGTCAAAAGAGTCAAAGAG
Ca_LG_6:50356706	CAATTAAAGTATTAATTACGGAAAATGGTTTTGAGATTTACCTTATTATGAGGAGCAGCACAAAAACCACAAGTAGGTGAAGAAGCCTGTAAACACCTCC	C/A	TACAATTCCATCTATCATTGTGAGGTCTCAAAACCATAGGTAAGTGAATTTGAGTCTCATGAAAAACAACAGTCAAAAGAGTCAAAGAGAGAAAAACACC
Ca_LG_6:50817846	AAAAAAGGAAGCAAACCTAAAAAATTGGAGGTTAATAGAGCTACCAAATCACAAAGGTCGGAGGGTAATAAAGCTGCCAAACTTGCAGCAAGAAAAAAAG	G/C	TGGATCGGGGAAAATCGATCGCTGCTGCTGCTGCTTCTAATAAAAGTCAGCCACGCCTTGGTAAATACGGGGCGTGTCTTGAAATCCAAATGAAAAGGAA
Ca_LG_6:50864939	TATATAAAATAGATACAAGTAACTCTTTTAATGGAATCACTGCCTCCAAGGTGAGCATGGCATTACAAGTTATTCTTCTAATGGAATCCATAAGCAGCCG	G/A	TTATCTGATTGCAATTTGGCCTGTATCGTGGCGCTACAGCCGCTGCTCAAAACCGTAACACAATGGGATTCTGTGTAGCCCCTAGGGGAAGCTATTGACT
Ca_LG_6:50965456	TGATACATGAGTTGGTTTCAAAATCTCAATCAGATTGAGAAACAGAAAAATCTGAAGTTCTGATCTAAATTAAGTAAATACAATTCACATGAATATATTG	G/C	ATACCTAAGACATTGCAGCAAACTCTCAGAAGCAGCAGCCTCTTGTATAGCCTCAGTTGCTGCCATTTGTGCTGCATCTCTGTGCTTCATTACTTCCTGT
Ca_LG_6:50965474	CAAAATCTCAATCAGATTGAGAAACAGAAAAATCTGAAGTTCTGATCTAAATTAAGTAAATACAATTCACATGAATATATTGATACCTAAGACATTGCAG	G/A	CAAACTCTCAGAAGCAGCAGCCTCTTGTATAGCCTCAGTTGCTGCCATTTGTGCTGCATCTCTGTGCTTCATTACTTCCTGTCAATATGTAAAAAAAATA
Ca_LG_6:51038916	TAGTGGTTATTGCAATTTTCACTTTCATGCCCTATTATGATTCATATATTGTAATACATGCCATGAACTCACACATGAGCTTGGCATTTGCTGCAAGATA	A/T	CCTCCCCCATTTAAAAACCCGTATACTCAATTACATCTAGAGGCGCTGCCTATTTAAATTGATTATATATTTCTTGCTGTTGATATTATTTTACATGCGA
Ca_LG_6:51038936	ACTTTCATGCCCTATTATGATTCATATATTGTAATACATGCCATGAACTCACACATGAGCTTGGCATTTGCTGCAAGATACCTCCCCCATTTAAAAACCC	C/T	GTATACTCAATTACATCTAGAGGCGCTGCCTATTTAAATTGATTATATATTTCTTGCTGTTGATATTATTTTACATGCGATTCATTCTTTTGTTCATCAT
Ca_LG_6:51038945	CCCTATTATGATTCATATATTGTAATACATGCCATGAACTCACACATGAGCTTGGCATTTGCTGCAAGATACCTCCCCCATTTAAAAACCCGTATACTCA	A/G	ATTACATCTAGAGGCGCTGCCTATTTAAATTGATTATATATTTCTTGCTGTTGATATTATTTTACATGCGATTCATTCTTTTGTTCATCATAGTTCAGAA
Ca_LG_6:51086481	TCTATATTTCCTGATATCAAACACCCCCTAAAGTAAAATTGAAATTTGGTGGTTTTATGTTTTTGATAGGCGAATGCCATCCTTCTTCCACTTGAATCCG	G/A	TGTTGTCTAAGGACGCAGCTGCTATGGCTGATGCTATAGCTAGGGAAAGCGAGACCAAGAAGGAAATATCACACATCCATGGACAAGCTATATATCAATC

**Positions**	**-100 bp flanking sequences**	**SNPs within *desi* cultivar**	**+100 bp flanking sequences**
Ca_LG_6:51288474	CTTGGCTACCAAGGGCCTCCAGTTTTTCAAGCTTATCGTAGTCCAACAAACTATCAAAACCCATCCTAGGAGAATCAAAGTTCGCAGCCACACAATGTCA	A/G	GCCATCCTCTGTTATGGGAGTGTGAAATCGGGATTCAGAACTCATGCAACTCACGGCAGCCAAATCCACTAGATTCAGCCATGGGGAATGAAGTTCACGG
Ca_LG_6:51316827	GTTTTTAACAAAACTGCAATGTCGTATTCGAATACAGCATCCACAACAAAAAACATGTATTTTGTGAGGGCCAAAAGCCCTCATAAAGGGGAAAAAACAG	G/A	CCACAAAGGGGGCATTTGTGGCGGCCTTAGGCAGCCACAAATAAGGTGGTCACAAACCTTTGTGGCGGCCAAAACGGCCACCAAGTGTGAAATTTACGTT
Ca_LG_6:51316854	TCGAATACAGCATCCACAACAAAAAACATGTATTTTGTGAGGGCCAAAAGCCCTCATAAAGGGGAAAAAACAGCCACAAAGGGGGCATTTGTGGCGGCCT	T/A	TAGGCAGCCACAAATAAGGTGGTCACAAACCTTTGTGGCGGCCAAAACGGCCACCAAGTGTGAAATTTACGTTGTCCTTTGTGAGGACAAATGCTGTCAC
Ca_LG_6:51462417	ACAATAAAGCCACAATTAGTACAAAGTATTGTCAAATAGCAGCAGTATAGCCTTGTAGCATAGCAGAATTTGAACAAATTGCTATATTTTGCAATCCAAT	T/A	TTTCTAGCAGCCTGTCTGCCCACTTCCATAAAATAACATTGTGGCAATGTAGACCTTAGTGGTCATGTATATTGGATATTTGGGAGCCATAACAAGTGTG
Ca_LG_6:51541228	ACATGTTGCATGGAAGGTCGGGTGCAACTTCCATTACTAAAATTTCCACCAACTTTGCTCTATCACTTGCTTGATTATGATGGAGATAGTGCAGCAAAAC	C/A	ATTTTCGTCGTAACATTAGAATTTATAATGCGGCTTTTGCTTTTACATTTATGGGTGGAAAAATTGACTATGAAATCAACAAACAGGGCGACCCTTATGT
Ca_LG_6:51541233	TTGCATGGAAGGTCGGGTGCAACTTCCATTACTAAAATTTCCACCAACTTTGCTCTATCACTTGCTTGATTATGATGGAGATAGTGCAGCAAAACATTTT	T/C	CGTCGTAACATTAGAATTTATAATGCGGCTTTTGCTTTTACATTTATGGGTGGAAAAATTGACTATGAAATCAACAAACAGGGCGACCCTTATGTCTTAA
Ca_LG_6:51541277	CAACTTTGCTCTATCACTTGCTTGATTATGATGGAGATAGTGCAGCAAAACATTTTCGTCGTAACATTAGAATTTATAATGCGGCTTTTGCTTTTACATT	T/C	TATGGGTGGAAAAATTGACTATGAAATCAACAAACAGGGCGACCCTTATGTCTTAAGAATCAATGGACAGACATACCACCTACTTGGGTCTTTATGTCCT
Ca_LG_6:51638153	CCAAGTCATGCTACATCCAAAAATCTCGCATAACAATGGACCTTTGTCTTTTGTCCTTAAAAATGACAGGTATAAACATGCAGCAAATCCACAAACACAA	A/G	TCAGAGATATTTTAATCCATATAATTCCCAGAAAGAAAAAAAAAAAAACAGACCAAGCGTGCCATCACATTCAGTTCACTCGGTATTAGATTCAGTTCCA
Ca_LG_6:51946358	TCACTTTCCGCCGGTAATCGCCGCTAACCACCGTCAACCGCGCACTGCCACTGCCTTCTTTTTTTCGGTGTGTTTTTGGACATATAGCTGATGAAAACAT	T/C	GCGTGGCTAGAATGTCTTGAAAAGTTCCGCAGGCATAGGCAACTTAGCCTTAATAGTTAGTATTTGCTAGTTTAAAGCTGAAAAGATTTTGCAATCAGGC
Ca_LG_6:51946369	CGGTAATCGCCGCTAACCACCGTCAACCGCGCACTGCCACTGCCTTCTTTTTTTCGGTGTGTTTTTGGACATATAGCTGATGAAAACATGCGTGGCTAGA	A/G	ATGTCTTGAAAAGTTCCGCAGGCATAGGCAACTTAGCCTTAATAGTTAGTATTTGCTAGTTTAAAGCTGAAAAGATTTTGCAATCAGGCCTAGGCAGAAC
Ca_LG_6:51982868	CATGGTTTTCCGCAAATATACATACTTAGGACGAAGAACATCTATATTGTATCTGAGCAGCATTGGGAAATCAGCGATCATTTCACCCAGTTGCCGAAGG	G/A	CGTATGCCAAGAGACAAATAATATTTTAAATTTACTTCGAGCTTATGTACTATGCTGCATCCCAAGAGCTCAGGTCCAAGAGCTACGACCTTTCCAATAT
Ca_LG_6:52270780	TGAAAGTTAGACATTCGTCTTTGACTGAATTCATTTCGAACAAAGTAGCTTCTCTGAAAGTGGAGCATTTTGCTTGAAGATTATTCATTAATTGCATTCT	T/C	TACAAAGATAATTCGGTAAAACGTGGAGTAGTGACTTCAGATAATTCTTTGTGTATTTTTGGGTGTGGTGGCAATAAGACCATGCAACATGTATTCTTTG

Positions	-100 bp flanking sequences	SNPs within *desi* cultivar	+100 bp flanking sequences
Ca_LG_6:52270795	CGTCTTTGACTGAATTCATTTCGAACAAAGTAGCTTCTCTGAAAGTGGAGCATTTTGCTTGAAGATTATTCATTAATTGCATTCTTACAAAGATAATTCG	G/A	GTAAAACGTGGAGTAGTGACTTCAGATAATTCTTTGTGTATTTTTGGGTGTGGTGGCAATAAGACCATGCAACATGTATTCTTTGATTGCTTATTTTTTT
Ca_LG_6:52329312	CAAAGTTGTGAAGAAAGCCTCCGATCCGAAATGCTGTGGCGGACTCGGGTTGCAGCTTCCTCTCGTTGTTCGGTTTCCTGATGTTTTAAAGAATCGTCTC	C/G	GAGTCTCTTCACGCTGCCTTCGACGGTGCGATTCAATCTCAAGGGTATGAATCTCATTACCAAGGTGTTTACCCTGTGAAGTGTAACCAGGACCGGTTCG
Ca_LG_6:52368621	TTTAAGATTTTTGTGAGGGCTTTTTCAGCCACAAAATACAAATTTTGTGAGGGTAAAGGCAGCCACAAATACTTGAAGTGACGTTGCAGTTTGTGGCTGT	T/C	AAAGGCCGCCACAAAAGCCTGACATGTTGCATTTTGTGGCTGCAAAGGCTGCCAGAAAGGAATAAAGAGACGTTGGCTTTTTTGGCTGTCCAGGCCGCCA
Ca_LG_6:52368625	AGATTTTTGTGAGGGCTTTTTCAGCCACAAAATACAAATTTTGTGAGGGTAAAGGCAGCCACAAATACTTGAAGTGACGTTGCAGTTTGTGGCTGTAAAG	G/T	GCCGCCACAAAAGCCTGACATGTTGCATTTTGTGGCTGCAAAGGCTGCCAGAAAGGAATAAAGAGACGTTGGCTTTTTTGGCTGTCCAGGCCGCCACAAA
Ca_LG_6:52368645	TCAGCCACAAAATACAAATTTTGTGAGGGTAAAGGCAGCCACAAATACTTGAAGTGACGTTGCAGTTTGTGGCTGTAAAGGCCGCCACAAAAGCCTGACA	A/C	TGTTGCATTTTGTGGCTGCAAAGGCTGCCAGAAAGGAATAAAGAGACGTTGGCTTTTTTGGCTGTCCAGGCCGCCACAAATCCATGCCTTTTTGACCGTT
Ca_LG_6:52368660	AAATTTTGTGAGGGTAAAGGCAGCCACAAATACTTGAAGTGACGTTGCAGTTTGTGGCTGTAAAGGCCGCCACAAAAGCCTGACATGTTGCATTTTGTGG	G/C	CTGCAAAGGCTGCCAGAAAGGAATAAAGAGACGTTGGCTTTTTTGGCTGTCCAGGCCGCCACAAATCCATGCCTTTTTGACCGTTTTGGCCGCCACAAAG
Ca_LG_6:52590090	CCAACGGCTATAATGCCATTTGTGGCGGCCTTTGCCGCCAGAAAATCCAACGGTACAATCCATTTGTGACGGCCTAAGCGGCCACAAACGCCAGCTGTAT	T/A	TGAGCCTCCTTTGTGGCGGCCTTTGCAGCCACAAATGCCACGTGCGCATTCCTTTGTGGCAGCCTTTGCAGCCACAAATGCCACTGGTCCCGTCTGCCTT
Ca_LG_6:52590099	ATAATGCCATTTGTGGCGGCCTTTGCCGCCAGAAAATCCAACGGTACAATCCATTTGTGACGGCCTAAGCGGCCACAAACGCCAGCTGTATTGAGCCTCC	C/A	TTTGTGGCGGCCTTTGCAGCCACAAATGCCACGTGCGCATTCCTTTGTGGCAGCCTTTGCAGCCACAAATGCCACTGGTCCCGTCTGCCTTTGTGACGGA
Ca_LG_6:53602784	AATGTGAATTTCCTCTCAAATAGGTAAAGGAGTGGCTCGCAGACTAGGAGGCTGAAGCATTGAGATGCCAGAAGCTGCTGGTGGAGGAGGAGGAAGCTGT	T/C	ACAGACAAACACCACATGGACCACTCCTAAGCTGAATTTATTGTTGTCAATAAGCTGAATTCACGTGGAGAAGGGCATTGAGCATCGACCGAAGCTTCTT
Ca_LG_6:53602828	TAGGAGGCTGAAGCATTGAGATGCCAGAAGCTGCTGGTGGAGGAGGAGGAAGCTGTACAGACAAACACCACATGGACCACTCCTAAGCTGAATTTATTGT	T/C	TGTCAATAAGCTGAATTCACGTGGAGAAGGGCATTGAGCATCGACCGAAGCTTCTTAGCTGAGATTTCAACTCTTGGAAATACCAGGCACAGGAACATTG
Ca_LG_6:53602863	GGTGGAGGAGGAGGAAGCTGTACAGACAAACACCACATGGACCACTCCTAAGCTGAATTTATTGTTGTCAATAAGCTGAATTCACGTGGAGAAGGGCATT	T/C	GAGCATCGACCGAAGCTTCTTAGCTGAGATTTCAACTCTTGGAAATACCAGGCACAGGAACATTGTGAAGCTACATGAATTCTTTGAAAATAATTTTGAT
Ca_LG_6:53602904	CCACTCCTAAGCTGAATTTATTGTTGTCAATAAGCTGAATTCACGTGGAGAAGGGCATTGAGCATCGACCGAAGCTTCTTAGCTGAGATTTCAACTCTTG	G/A	GAAATACCAGGCACAGGAACATTGTGAAGCTACATGAATTCTTTGAAAATAATTTTGATGCGAAATTACCTGCGGAATTTGATTCAAAAAGTATTTTAGT

Positions	-100 bp flanking sequences	SNPs within *desi* cultivar	+100 bp flanking sequences
Ca_LG_6:53776640	TTGATGGCCAAGTACTATTCCAGACTTTGATACAATTCAACACAAAAGTCTATACTCTATAGAGATTGTTTCAGATAGACTTTGACAATCTTTCAACTCA	A/G	GTTTCCTGCAGCTAAATCGATATTAATCACGTGAAGCATCAGAAAGGTTAAGGATATCAAACATGTAAACTTCACTTAGCCTTAATTCTACACACACACA
Ca_LG_6:53804707	TATAAAAGATTTGTATTCGGAGGTATCGAAACTTGAGGTAAAAGAAAAGTCTTCAGAAAGTAGTGGTAGCATAAACCATTGTCTAAAATCAGACGCACGG	G/A	CCGATATACAAACACCTGACAGAGATTCAAAATGAAATAACAGTCTGGTTGGAGAAAAGTGCCTTGCTGAAAGAAGAGCTACAACATAGATTCTCGTCTT
Ca_LG_6:53810516	GGTGTACAACATAATAACCATGGTCTGTGATTTTTTTGTAATTCTGTATGAATGTTTAGGACAAGGTTACTAAAATGTAAGTGGCATTTGGAATAATAAC	C/G	TGGCTGGATGCAGCAGTACATAAAAACGGATTAGAAGCCAAGTGCAGCTGGTTTAAATTGAAATGATGCATATAATGAAACTGTTTGGCAGGTACGAATG
Ca_LG_6:53810606	GAATAATAACTGGCTGGATGCAGCAGTACATAAAAACGGATTAGAAGCCAAGTGCAGCTGGTTTAAATTGAAATGATGCATATAATGAAACTGTTTGGCA	A/T	GGTACGAATGGGGTGCCAGTTTTTTTGTACAATATGCTTAGAGTTATGACAGTACACTGATGAATTATTCTGCATCATGGCTAAGTTGTGATCAGTTTTA
Ca_LG_6:53850382	TGCTGCTGGAAATTTGGAGGCGGTTGATGTTGTTGTATTGAGAGTATTTTTGCTGCTGTTGGAACATTGGTTGTTGCTACAAGTATTGAGGCCTTGATTG	G/T	TTGTTGTTGGTGGGGCGGGAACTGCTAATGAAACGCATGAGGAAATGAAAATTATTGCTATTGAAACATCTGAAACTGATGTTGCTACTCTTGAAACTTT
Ca_LG_6:53850395	TTGGAGGCGGTTGATGTTGTTGTATTGAGAGTATTTTTGCTGCTGTTGGAACATTGGTTGTTGCTACAAGTATTGAGGCCTTGATTGTTGTTGTTGGTGG	G/A	GGCGGGAACTGCTAATGAAACGCATGAGGAAATGAAAATTATTGCTATTGAAACATCTGAAACTGATGTTGCTACTCTTGAAACTTTTGTGTATATTCTT
Ca_LG_6:53850406	TGATGTTGTTGTATTGAGAGTATTTTTGCTGCTGTTGGAACATTGGTTGTTGCTACAAGTATTGAGGCCTTGATTGTTGTTGTTGGTGGGGCGGGAACTG	G/A	CTAATGAAACGCATGAGGAAATGAAAATTATTGCTATTGAAACATCTGAAACTGATGTTGCTACTCTTGAAACTTTTGTGTATATTCTTTATTTACTTTA
Ca_LG_6:53850409	TGTTGTTGTATTGAGAGTATTTTTGCTGCTGTTGGAACATTGGTTGTTGCTACAAGTATTGAGGCCTTGATTGTTGTTGTTGGTGGGGCGGGAACTGCTA	A/G	ATGAAACGCATGAGGAAATGAAAATTATTGCTATTGAAACATCTGAAACTGATGTTGCTACTCTTGAAACTTTTGTGTATATTCTTTATTTACTTTATCC
Ca_LG_6:53850420	TGAGAGTATTTTTGCTGCTGTTGGAACATTGGTTGTTGCTACAAGTATTGAGGCCTTGATTGTTGTTGTTGGTGGGGCGGGAACTGCTAATGAAACGCAT	T/G	GAGGAAATGAAAATTATTGCTATTGAAACATCTGAAACTGATGTTGCTACTCTTGAAACTTTTGTGTATATTCTTTATTTACTTTATCCATTGCTGCACG
Ca_LG_6:54107084	AGACTTTTCATTTTTACGAAGGACAACTAGGATAGATGAACGACAATATTTATATTATAAATATGTTATCCTCATGGTGCTGCTAAGTGTAAGATCGTGG	G/A	TTGAATAGTTGGGTAGATTTGAAAACGCGTCTTGTTTTGGACCAATTAATTAACCAAACTAAATATAACAACCACACACATACCGTGCTGCTCTATAGTT
Ca_LG_6:54107089	TTTCATTTTTACGAAGGACAACTAGGATAGATGAACGACAATATTTATATTATAAATATGTTATCCTCATGGTGCTGCTAAGTGTAAGATCGTGGTTGAA	A/T	TAGTTGGGTAGATTTGAAAACGCGTCTTGTTTTGGACCAATTAATTAACCAAACTAAATATAACAACCACACACATACCGTGCTGCTCTATAGTTTCATT
Ca_LG_6:54107093	ATTTTTACGAAGGACAACTAGGATAGATGAACGACAATATTTATATTATAAATATGTTATCCTCATGGTGCTGCTAAGTGTAAGATCGTGGTTGAATAGT	T/C	TGGGTAGATTTGAAAACGCGTCTTGTTTTGGACCAATTAATTAACCAAACTAAATATAACAACCACACACATACCGTGCTGCTCTATAGTTTCATTAAAA

Positions	-100 bp flanking sequences	SNPs within *desi* cultivar	+100 bp flanking sequences
Ca_LG_6:54107096	TTTACGAAGGACAACTAGGATAGATGAACGACAATATTTATATTATAAATATGTTATCCTCATGGTGCTGCTAAGTGTAAGATCGTGGTTGAATAGTTGG	G/A	GTAGATTTGAAAACGCGTCTTGTTTTGGACCAATTAATTAACCAAACTAAATATAACAACCACACACATACCGTGCTGCTCTATAGTTTCATTAAAATCA
Ca_LG_6:54107103	AGGACAACTAGGATAGATGAACGACAATATTTATATTATAAATATGTTATCCTCATGGTGCTGCTAAGTGTAAGATCGTGGTTGAATAGTTGGGTAGATT	T/C	TGAAAACGCGTCTTGTTTTGGACCAATTAATTAACCAAACTAAATATAACAACCACACACATACCGTGCTGCTCTATAGTTTCATTAAAATCAAATATAG
Ca_LG_6:54107104	GGACAACTAGGATAGATGAACGACAATATTTATATTATAAATATGTTATCCTCATGGTGCTGCTAAGTGTAAGATCGTGGTTGAATAGTTGGGTAGATTT	T/A	GAAAACGCGTCTTGTTTTGGACCAATTAATTAACCAAACTAAATATAACAACCACACACATACCGTGCTGCTCTATAGTTTCATTAAAATCAAATATAGA
Ca_LG_6:54107108	AACTAGGATAGATGAACGACAATATTTATATTATAAATATGTTATCCTCATGGTGCTGCTAAGTGTAAGATCGTGGTTGAATAGTTGGGTAGATTTGAAA	A/C	ACGCGTCTTGTTTTGGACCAATTAATTAACCAAACTAAATATAACAACCACACACATACCGTGCTGCTCTATAGTTTCATTAAAATCAAATATAGATAAA
Ca_LG_6:54107127	CAATATTTATATTATAAATATGTTATCCTCATGGTGCTGCTAAGTGTAAGATCGTGGTTGAATAGTTGGGTAGATTTGAAAACGCGTCTTGTTTTGGACC	C/A	AATTAATTAACCAAACTAAATATAACAACCACACACATACCGTGCTGCTCTATAGTTTCATTAAAATCAAATATAGATAAAGAGACAAGAGTATCACACG
Ca_LG_6:54107135	ATATTATAAATATGTTATCCTCATGGTGCTGCTAAGTGTAAGATCGTGGTTGAATAGTTGGGTAGATTTGAAAACGCGTCTTGTTTTGGACCAATTAATT	T/A	AACCAAACTAAATATAACAACCACACACATACCGTGCTGCTCTATAGTTTCATTAAAATCAAATATAGATAAAGAGACAAGAGTATCACACGAATAATGT
Ca_LG_6:54267854	TTGTGTAACCTGATTTGAAAGGACCATTGCAGCCAATACATGCAACATCAGGACATATTTTTGAGCCACATTCAATAGGAATGTAGGATGCTGAATTGTA	A/G	GTGAGTGTCACAATCATACCAAAGATTTTCACCAGCTAGATCAATGGCTAGATTGAAGATTTGTGACATGCAACAAACCTCACTGATGATGCATTATCAT
Ca_LG_6:54267861	ACCTGATTTGAAAGGACCATTGCAGCCAATACATGCAACATCAGGACATATTTTTGAGCCACATTCAATAGGAATGTAGGATGCTGAATTGTAGTGAGTG	G/C	TCACAATCATACCAAAGATTTTCACCAGCTAGATCAATGGCTAGATTGAAGATTTGTGACATGCAACAAACCTCACTGATGATGCATTATCATGAACAGC
Ca_LG_6:54267901	TCAGGACATATTTTTGAGCCACATTCAATAGGAATGTAGGATGCTGAATTGTAGTGAGTGTCACAATCATACCAAAGATTTTCACCAGCTAGATCAATGG	G/A	CTAGATTGAAGATTTGTGACATGCAACAAACCTCACTGATGATGCATTATCATGAACAGCAGCATGGGTCAACCATCAATTTCATCAATCTTAAATTCTC
Ca_LG_6:54267903	AGGACATATTTTTGAGCCACATTCAATAGGAATGTAGGATGCTGAATTGTAGTGAGTGTCACAATCATACCAAAGATTTTCACCAGCTAGATCAATGGCT	T/C	AGATTGAAGATTTGTGACATGCAACAAACCTCACTGATGATGCATTATCATGAACAGCAGCATGGGTCAACCATCAATTTCATCAATCTTAAATTCTCTC
Ca_LG_6:54267927	AATAGGAATGTAGGATGCTGAATTGTAGTGAGTGTCACAATCATACCAAAGATTTTCACCAGCTAGATCAATGGCTAGATTGAAGATTTGTGACATGCAA	A/G	CAAACCTCACTGATGATGCATTATCATGAACAGCAGCATGGGTCAACCATCAATTTCATCAATCTTAAATTCTCTCTTGAGTTTAATACTTGGTAAAACA
Ca_LG_6:54267939	GGATGCTGAATTGTAGTGAGTGTCACAATCATACCAAAGATTTTCACCAGCTAGATCAATGGCTAGATTGAAGATTTGTGACATGCAACAAACCTCACTG	G/A	ATGATGCATTATCATGAACAGCAGCATGGGTCAACCATCAATTTCATCAATCTTAAATTCTCTCTTGAGTTTAATACTTGGTAAAACAATAAGCTATATG

Positions	-100 bp flanking sequences	SNPs within *desi* cultivar	+100 bp flanking sequences
Ca_LG_6:54554915	ATGAACATTTAGGTCATTTTAGTCCTCCCTAAGCTTATTCTTGTTCAATTAGGGTTTTTAACCAATCTTGTGCATCCAATAAGTTCATTTGTGTCATTGG	G/C	ATGAACATTTAGGTCATTTTCGTCCTCCCTATGCTTATTCTTGTCCAATTAGGGTTTCTACCCACTCTTGTGCATCCAATAAGTTCATTTGTGTAATTGG
Ca_LG_6:54695492	ATCAACTCTGAAACTTTTTGTTCAATTTTAGTAATTAAATATAATTCGATAGTAAAATGCAATCCTTGAGTTTGCACTCGGTACGTTTCTAGGACCGACT	T/C	AACGCCTCACTTACACCGGTACCTATGTAGTGTAGTGTCGGTCGCACATTCACATAGCGTTCAGACACACGTGCCTTCCATAACCTAGGGTATTCAAGTC
Ca_LG_6:54695493	TCAACTCTGAAACTTTTTGTTCAATTTTAGTAATTAAATATAATTCGATAGTAAAATGCAATCCTTGAGTTTGCACTCGGTACGTTTCTAGGACCGACTA	A/G	ACGCCTCACTTACACCGGTACCTATGTAGTGTAGTGTCGGTCGCACATTCACATAGCGTTCAGACACACGTGCCTTCCATAACCTAGGGTATTCAAGTCA
Ca_LG_6:54695503	AACTTTTTGTTCAATTTTAGTAATTAAATATAATTCGATAGTAAAATGCAATCCTTGAGTTTGCACTCGGTACGTTTCTAGGACCGACTAACGCCTCACT	T/C	TACACCGGTACCTATGTAGTGTAGTGTCGGTCGCACATTCACATAGCGTTCAGACACACGTGCCTTCCATAACCTAGGGTATTCAAGTCAGCTGCATATG
Ca_LG_6:54695505	CTTTTTGTTCAATTTTAGTAATTAAATATAATTCGATAGTAAAATGCAATCCTTGAGTTTGCACTCGGTACGTTTCTAGGACCGACTAACGCCTCACTTA	A/G	CACCGGTACCTATGTAGTGTAGTGTCGGTCGCACATTCACATAGCGTTCAGACACACGTGCCTTCCATAACCTAGGGTATTCAAGTCAGCTGCATATGAT
Ca_LG_6:54695509	TTGTTCAATTTTAGTAATTAAATATAATTCGATAGTAAAATGCAATCCTTGAGTTTGCACTCGGTACGTTTCTAGGACCGACTAACGCCTCACTTACACC	C/A	GGTACCTATGTAGTGTAGTGTCGGTCGCACATTCACATAGCGTTCAGACACACGTGCCTTCCATAACCTAGGGTATTCAAGTCAGCTGCATATGATTAGG
Ca_LG_6:54695518	TTTAGTAATTAAATATAATTCGATAGTAAAATGCAATCCTTGAGTTTGCACTCGGTACGTTTCTAGGACCGACTAACGCCTCACTTACACCGGTACCTAT	T/C	GTAGTGTAGTGTCGGTCGCACATTCACATAGCGTTCAGACACACGTGCCTTCCATAACCTAGGGTATTCAAGTCAGCTGCATATGATTAGGTAGCTTTGC
Ca_LG_6:54695555	CCTTGAGTTTGCACTCGGTACGTTTCTAGGACCGACTAACGCCTCACTTACACCGGTACCTATGTAGTGTAGTGTCGGTCGCACATTCACATAGCGTTCA	A/G	GACACACGTGCCTTCCATAACCTAGGGTATTCAAGTCAGCTGCATATGATTAGGTAGCTTTGCAGCTGCAAAAGGATTAGACATCTTCCCATGATATGGT
Ca_LG_6:54695559	GAGTTTGCACTCGGTACGTTTCTAGGACCGACTAACGCCTCACTTACACCGGTACCTATGTAGTGTAGTGTCGGTCGCACATTCACATAGCGTTCAGACA	A/T	CACGTGCCTTCCATAACCTAGGGTATTCAAGTCAGCTGCATATGATTAGGTAGCTTTGCAGCTGCAAAAGGATTAGACATCTTCCCATGATATGGTTCCT
Ca_LG_6:54695562	TTTGCACTCGGTACGTTTCTAGGACCGACTAACGCCTCACTTACACCGGTACCTATGTAGTGTAGTGTCGGTCGCACATTCACATAGCGTTCAGACACAC	C/T	GTGCCTTCCATAACCTAGGGTATTCAAGTCAGCTGCATATGATTAGGTAGCTTTGCAGCTGCAAAAGGATTAGACATCTTCCCATGATATGGTTCCTTTT
Ca_LG_6:54695573	TACGTTTCTAGGACCGACTAACGCCTCACTTACACCGGTACCTATGTAGTGTAGTGTCGGTCGCACATTCACATAGCGTTCAGACACACGTGCCTTCCAT	T/G	AACCTAGGGTATTCAAGTCAGCTGCATATGATTAGGTAGCTTTGCAGCTGCAAAAGGATTAGACATCTTCCCATGATATGGTTCCTTTTGGTCCGAACCT
Ca_LG_6:54695579	TCTAGGACCGACTAACGCCTCACTTACACCGGTACCTATGTAGTGTAGTGTCGGTCGCACATTCACATAGCGTTCAGACACACGTGCCTTCCATAACCTA	A/G	GGGTATTCAAGTCAGCTGCATATGATTAGGTAGCTTTGCAGCTGCAAAAGGATTAGACATCTTCCCATGATATGGTTCCTTTTGGTCCGAACCTGGTGCT

Positions	-100 bp flanking sequences	SNPs within *desi* cultivar	+100 bp flanking sequences
Ca_LG_6:54695588	GACTAACGCCTCACTTACACCGGTACCTATGTAGTGTAGTGTCGGTCGCACATTCACATAGCGTTCAGACACACGTGCCTTCCATAACCTAGGGTATTCA	A/G	AGTCAGCTGCATATGATTAGGTAGCTTTGCAGCTGCAAAAGGATTAGACATCTTCCCATGATATGGTTCCTTTTGGTCCGAACCTGGTGCTACATTAGGT
Ca_LG_6:54695591	TAACGCCTCACTTACACCGGTACCTATGTAGTGTAGTGTCGGTCGCACATTCACATAGCGTTCAGACACACGTGCCTTCCATAACCTAGGGTATTCAAGT	T/C	CAGCTGCATATGATTAGGTAGCTTTGCAGCTGCAAAAGGATTAGACATCTTCCCATGATATGGTTCCTTTTGGTCCGAACCTGGTGCTACATTAGGTTAG
Ca_LG_7:190132	ACTAAAAGAATTCTGGGAAATTGGGGTGAAAACATTTGATGCATGCAAAAAAGAGTCATTCCAAATGCGTGTTGCTATCATGTGGACTATCAATGATTTT	T/C	CCGGCATATGCAAATTTGTCAGGCTGGAGCACTAAAGGTCGATACGCTTGTCCATGTTGTGGTTTAAAAACTGCGTCTCATTGGTTGCGTCATAGTTGGA
Ca_LG_7:190135	AAAAGAATTCTGGGAAATTGGGGTGAAAACATTTGATGCATGCAAAAAAGAGTCATTCCAAATGCGTGTTGCTATCATGTGGACTATCAATGATTTTCCG	G/A	GCATATGCAAATTTGTCAGGCTGGAGCACTAAAGGTCGATACGCTTGTCCATGTTGTGGTTTAAAAACTGCGTCTCATTGGTTGCGTCATAGTTGGAAGT
Ca_LG_7:190177	CAAAAAAGAGTCATTCCAAATGCGTGTTGCTATCATGTGGACTATCAATGATTTTCCGGCATATGCAAATTTGTCAGGCTGGAGCACTAAAGGTCGATAC	C/T	GCTTGTCCATGTTGTGGTTTAAAAACTGCGTCTCATTGGTTGCGTCATAGTTGGAAGTTTTGCTACGTGTGTCATCGTTGTTGGTTAGAGCCTAATAGTA
Ca_LG_7:190195	AATGCGTGTTGCTATCATGTGGACTATCAATGATTTTCCGGCATATGCAAATTTGTCAGGCTGGAGCACTAAAGGTCGATACGCTTGTCCATGTTGTGGT	T/A	TTAAAAACTGCGTCTCATTGGTTGCGTCATAGTTGGAAGTTTTGCTACGTGTGTCATCGTTGTTGGTTAGAGCCTAATAGTAAGTGGAGATACAATAGAA
Ca_LG_7:794077	AATCAAAAAATAAAAATAAACAGAATAATATTAAATAAAAGAGAATCAAATCTGATTTTCCTTGACTCTTCAACACTATTGATAGTTATAATTTACAACT	T/G	GCAATTTCTTGCTTTTCAGGAGTTGATTGCTGCTCTTCTTGTATGCTCATTTTTATGTCTATTCCCAGTCCATGACAGATATGAGAAACAACTTCAACCA
Ca_LG_7:794079	TCAAAAAATAAAAATAAACAGAATAATATTAAATAAAAGAGAATCAAATCTGATTTTCCTTGACTCTTCAACACTATTGATAGTTATAATTTACAACTGC	C/A	AATTTCTTGCTTTTCAGGAGTTGATTGCTGCTCTTCTTGTATGCTCATTTTTATGTCTATTCCCAGTCCATGACAGATATGAGAAACAACTTCAACCAGT
Ca_LG_7:803446	TCCATGCTTGAAGACCTTGAATTTTGTTTTGGTTCATCATATCCTGCAGGCTCAAAGACCTTTCATAGCATACTACACATTTGGCTTGGACGCATTGCAC	C/A	AACCTAGACAAGGTAACATGACATTTCCTTCACACAGTGGGAGAATCTAAACATGTGATCAATGGCATTGCTGATAAGCATCTGCATCGAAAAGAGTTAT
Ca_LG_7:803479	TCATCATATCCTGCAGGCTCAAAGACCTTTCATAGCATACTACACATTTGGCTTGGACGCATTGCACAACCTAGACAAGGTAACATGACATTTCCTTCAC	C/T	ACAGTGGGAGAATCTAAACATGTGATCAATGGCATTGCTGATAAGCATCTGCATCGAAAAGAGTTATATATTTTTCTTATTTCACGTTTGAATATTGATC
Ca_LG_7:803527	TGGCTTGGACGCATTGCACAACCTAGACAAGGTAACATGACATTTCCTTCACACAGTGGGAGAATCTAAACATGTGATCAATGGCATTGCTGATAAGCAT	T/G	CTGCATCGAAAAGAGTTATATATTTTTCTTATTTCACGTTTGAATATTGATCTTTTTTTTTCTTCCCTAAGATCTTTTTTAAAGAGTTGTACCTATCCAGC
Ca_LG_7:860001	ATAGTAGCATGATATATGCATAAAGACAAACACACACAATAGCAAGGACAGAGAATGTAGGCGGACACAACAGACAGCAAGGAGGGCACAGCAGCAGCGG	G/A	CATACACAAACGCAGCAGCGCACATACAACCAAACACACACACTAGAGGGCACAACAGATTTGTCAGTACAATTTTTTTGTCTTACTTCATATGAAATTT

Positions	-100 bp flanking sequences	SNPs within *desi* cultivar	+100 bp flanking sequences
Ca_LG_7:1021578	TAAGAATGCGAACTGCATTGCCTTTTTTTATTCCTTTATTCAACTTGGTCAGTAAGCTTAGAAGAAAAAGATTGATAGAGTTAAAAAACGTTTTCCTAGG	G/A	ATGATTTCATCACCATAAGGTTGTTATGGCTTGCTGCATCCTCTTGATGAAAAGTTAGAGTATCAAAAGAGTCCTTTAGTTGAAATCAACGTGTATTCCG
Ca_LG_7:1022258	ACAGTTGCATAAAATTAAAACAAAAAGCAAGCATGCTATAGTTTATCAGCTGCAATTCAAGTTTACTTTATGTGTATCCACGAATCTGGAACAAATTATG	G/C	TTTGACTTACATCGGAGTGCGAGTGAAAGGGTAAAAAGTGGCTGCGGAAAAAAGGATGAGTCTGAGGTTGTAGAACCAAGAGCTGCAGATCCCTGAAACA
Ca_LG_7:1101149	CCAATGATGTCTTTGCAGCTGATATTGGGAATAACAGCCTCAGCAGCAGGCACAGCAGGGGGTGTAGCATATTTGGGTTTGAAAGGAAACAACCATGTGG	G/A	ATTGGCATAAAATTTGCAATATTTATGACAAGTTCTGTAGGCATATTGGCAGCTCTGTAGCAGTGGGAGTATTTGGAAGCATTGTGCTTGTCTTGCTCAT
Ca_LG_7:1101150	CAATGATGTCTTTGCAGCTGATATTGGGAATAACAGCCTCAGCAGCAGGCACAGCAGGGGGTGTAGCATATTTGGGTTTGAAAGGAAACAACCATGTGGA	A/G	TTGGCATAAAATTTGCAATATTTATGACAAGTTCTGTAGGCATATTGGCAGCTCTGTAGCAGTGGGAGTATTTGGAAGCATTGTGCTTGTCTTGCTCATT
Ca_LG_7:1103940	GTCTTCAAAACCAAACCTTGCAGCATGAATGGTTTCAAAAGCTTTCAGATAGTCCCCAACCTCAAGTGATTGAAGATGCATAGAGGCTTTCAAAAAAGTG	G/C	TCAACAGAGGCTGCTCTAAGTCCCAACTCGGTGAGCCTCTCAAAGTACAATATTTTCCATGTCTGCAAATGAATGAGCACATAAAACACATGAAGAGAAA
Ca_LG_7:1173747	CCAACCAAGTATCGAACACCCTTCACTACTAAGGATTTCAAAACATGTCATATTACAGCTAAAATAAATCACTTGCAGCTTATGAAATGCAATTATTTTG	G/C	TTATATTCAATTACAAATAATGTTTCAGGCTATCAGTGCCAAAATACAGTCAAAGATTGCCGTTTGACGAACCACAATAATTGTAAATTATGCTTTAGTT
Ca_LG_7:1395485	GCTTACCTGTGCTTTGCCATTTGCAGGTTGCCTGCGGATAACATGCGGCCTCTCGTTAGGTGTTCCGGGTGCATATCCGGCCCCGCCTTGTTTCTCTTCT	T/C	CTCACTTTGTTGAAAATGTGAGTATAGCTGTCAGCTGATGCAGGGTTACTCACATCCCACTCCCCGAACTTTGGAACAGCAGCACCTTTATCAGGCTGAT
Ca_LG_7:1395487	TTACCTGTGCTTTGCCATTTGCAGGTTGCCTGCGGATAACATGCGGCCTCTCGTTAGGTGTTCCGGGTGCATATCCGGCCCCGCCTTGTTTCTCTTCTCT	T/G	CACTTTGTTGAAAATGTGAGTATAGCTGTCAGCTGATGCAGGGTTACTCACATCCCACTCCCCGAACTTTGGAACAGCAGCACCTTTATCAGGCTGATGT
Ca_LG_7:1395514	GCCTGCGGATAACATGCGGCCTCTCGTTAGGTGTTCCGGGTGCATATCCGGCCCCGCCTTGTTTCTCTTCTCTCACTTTGTTGAAAATGTGAGTATAGCT	T/C	GTCAGCTGATGCAGGGTTACTCACATCCCACTCCCCGAACTTTGGAACAGCAGCACCTTTATCAGGCTGATGTATCAGAATTCTAAAGTCAGAACATATT
Ca_LG_7:1395515	CCTGCGGATAACATGCGGCCTCTCGTTAGGTGTTCCGGGTGCATATCCGGCCCCGCCTTGTTTCTCTTCTCTCACTTTGTTGAAAATGTGAGTATAGCTG	G/A	TCAGCTGATGCAGGGTTACTCACATCCCACTCCCCGAACTTTGGAACAGCAGCACCTTTATCAGGCTGATGTATCAGAATTCTAAAGTCAGAACATATTT
Ca_LG_7:1395526	CATGCGGCCTCTCGTTAGGTGTTCCGGGTGCATATCCGGCCCCGCCTTGTTTCTCTTCTCTCACTTTGTTGAAAATGTGAGTATAGCTGTCAGCTGATGC	C/T	AGGGTTACTCACATCCCACTCCCCGAACTTTGGAACAGCAGCACCTTTATCAGGCTGATGTATCAGAATTCTAAAGTCAGAACATATTTGCAACAGAAAA
Ca_LG_7:1395532	GCCTCTCGTTAGGTGTTCCGGGTGCATATCCGGCCCCGCCTTGTTTCTCTTCTCTCACTTTGTTGAAAATGTGAGTATAGCTGTCAGCTGATGCAGGGTT	T/C	ACTCACATCCCACTCCCCGAACTTTGGAACAGCAGCACCTTTATCAGGCTGATGTATCAGAATTCTAAAGTCAGAACATATTTGCAACAGAAAATCTCAA

Positions	-100 bp flanking sequences	SNPs within *desi* cultivar	+100 bp flanking sequences
Ca_LG_7:1395537	TCGTTAGGTGTTCCGGGTGCATATCCGGCCCCGCCTTGTTTCTCTTCTCTCACTTTGTTGAAAATGTGAGTATAGCTGTCAGCTGATGCAGGGTTACTCA	A/T	CATCCCACTCCCCGAACTTTGGAACAGCAGCACCTTTATCAGGCTGATGTATCAGAATTCTAAAGTCAGAACATATTTGCAACAGAAAATCTCAAAATGA
Ca_LG_7:1757610	ATGCACAACTTTTTCATTTTCATCAATGTGTTTCATTACACATTTTCTCATGCGCTTAATAGTAGGCAAAAACTCAATGGCTGCCCAAGTGAGGTGGTAG	G/C	TCGTTATCCTCCTGTGTGTGTCGATCATTCCACCAGAACACATTCGACTTTGCATTTGTGGTCACCTTCCAAACATCCATGAAACTAGTCGATGTCCAAC
Ca_LG_7:1757615	CAACTTTTTCATTTTCATCAATGTGTTTCATTACACATTTTCTCATGCGCTTAATAGTAGGCAAAAACTCAATGGCTGCCCAAGTGAGGTGGTAGTCGTT	T/C	ATCCTCCTGTGTGTGTCGATCATTCCACCAGAACACATTCGACTTTGCATTTGTGGTCACCTTCCAAACATCCATGAAACTAGTCGATGTCCAACTCCAA
Ca_LG_7:1757622	TTCATTTTCATCAATGTGTTTCATTACACATTTTCTCATGCGCTTAATAGTAGGCAAAAACTCAATGGCTGCCCAAGTGAGGTGGTAGTCGTTATCCTCC	C/T	TGTGTGTGTCGATCATTCCACCAGAACACATTCGACTTTGCATTTGTGGTCACCTTCCAAACATCCATGAAACTAGTCGATGTCCAACTCCAACGACTAC
Ca_LG_7:1757631	ATCAATGTGTTTCATTACACATTTTCTCATGCGCTTAATAGTAGGCAAAAACTCAATGGCTGCCCAAGTGAGGTGGTAGTCGTTATCCTCCTGTGTGTGT	T/C	CGATCATTCCACCAGAACACATTCGACTTTGCATTTGTGGTCACCTTCCAAACATCCATGAAACTAGTCGATGTCCAACTCCAACGACTACTCGTCGATA
Ca_LG_7:2021560	CTATGGCGATTACAACAATTAATGATATCAATTTAGGTACAAAATTTGATACAACCCCGACACCAATGAGGACAGTAACTCAGCAAAAGACAGTATCATG	G/C	CTGATAAAGTTCAAGGGATGTTGATGTTCCCTTCCCTACTCCAGTAGTCAGGAATTGTTGCCAGCTGCATTTTTAGTTGAGCCTTGAATAGTCTCCAAAA
Ca_LG_7:2021569	TTACAACAATTAATGATATCAATTTAGGTACAAAATTTGATACAACCCCGACACCAATGAGGACAGTAACTCAGCAAAAGACAGTATCATGCTGATAAAG	G/A	TTCAAGGGATGTTGATGTTCCCTTCCCTACTCCAGTAGTCAGGAATTGTTGCCAGCTGCATTTTTAGTTGAGCCTTGAATAGTCTCCAAAAGATGTTCAA
Ca_LG_7:2282369	TTGTTGTAAACAAATTAGATACGTGATTTATTGCTTAAGTGCATTTATGCAGCTATTAATTTTGTACATTTTACTCTGTTGTTCAATCTGTTATCAAACC	C/T	TGGCATTCAAGTTTAGTTTGTTTATCACTTGCCCATGCTGCTCCATGCCGGCAGACATTAGATGTTAAAAGTTCTAAGAATCTGAGCCTCTGTATGAAGT
Ca_LG_7:2282391	GTGATTTATTGCTTAAGTGCATTTATGCAGCTATTAATTTTGTACATTTTACTCTGTTGTTCAATCTGTTATCAAACCTGGCATTCAAGTTTAGTTTGTT	T/G	TATCACTTGCCCATGCTGCTCCATGCCGGCAGACATTAGATGTTAAAAGTTCTAAGAATCTGAGCCTCTGTATGAAGTAAATTATGTCAATTACTGACAC
Ca_LG_7:2320730	TGGAATAAACGACTCTCCGGCTTTGGTTGCTGCTGATGTTGGCATGGCAATTGGTGCTGGAACTGACGTAGCTATAGAAGCAGCTGATATAGTTCTTGTC	C/A	AAAAGCAACTTGGAAGATGTAATTACAGCCATAGATCTATCTAGAAAGACCATGTCTCGTATCAGGCTGAACTACATTTGGGCTCTTGGCTACAACATTT
Ca_LG_7:2320865	TCTATCTAGAAAGACCATGTCTCGTATCAGGCTGAACTACATTTGGGCTCTTGGCTACAACATTTTGGGCATGCCAGTTGCTGCTGGTGTTTTGTACCCA	A/T	TTTACTGGAATCAGATTGCCACCATGGCTTGCTGGTGCTTGCATGGCTGCATCTTCCCTTAGTGTGGTTTCTTCGTCTCTTTTGCTGCAGTTCTATAAAA
Ca_LG_7:2320869	TCTAGAAAGACCATGTCTCGTATCAGGCTGAACTACATTTGGGCTCTTGGCTACAACATTTTGGGCATGCCAGTTGCTGCTGGTGTTTTGTACCCATTTA	A/T	CTGGAATCAGATTGCCACCATGGCTTGCTGGTGCTTGCATGGCTGCATCTTCCCTTAGTGTGGTTTCTTCGTCTCTTTTGCTGCAGTTCTATAAAAAACC

Positions	-100 bp flanking sequences	SNPs within *desi* cultivar	+100 bp flanking sequences
Ca_LG_7:2320870	CTAGAAAGACCATGTCTCGTATCAGGCTGAACTACATTTGGGCTCTTGGCTACAACATTTTGGGCATGCCAGTTGCTGCTGGTGTTTTGTACCCATTTAC	C/T	TGGAATCAGATTGCCACCATGGCTTGCTGGTGCTTGCATGGCTGCATCTTCCCTTAGTGTGGTTTCTTCGTCTCTTTTGCTGCAGTTCTATAAAAAACCT
Ca_LG_7:2320880	CATGTCTCGTATCAGGCTGAACTACATTTGGGCTCTTGGCTACAACATTTTGGGCATGCCAGTTGCTGCTGGTGTTTTGTACCCATTTACTGGAATCAGA	A/G	TTGCCACCATGGCTTGCTGGTGCTTGCATGGCTGCATCTTCCCTTAGTGTGGTTTCTTCGTCTCTTTTGCTGCAGTTCTATAAAAAACCTTTGCATGTAG
Ca_LG_7:2320886	TCGTATCAGGCTGAACTACATTTGGGCTCTTGGCTACAACATTTTGGGCATGCCAGTTGCTGCTGGTGTTTTGTACCCATTTACTGGAATCAGATTGCCA	A/T	CCATGGCTTGCTGGTGCTTGCATGGCTGCATCTTCCCTTAGTGTGGTTTCTTCGTCTCTTTTGCTGCAGTTCTATAAAAAACCTTTGCATGTAGAATCCA
Ca_LG_7:2364063	ATGCAATTTGAAGTTAATGTAATCAAAATGGATCGGCATTGTAATATCCAAACGTTGCCACCTAGTCAACAGAGGTTCTGAAGGCTGCAACTCCTTGACG	G/A	TACTGCTTACTCTTCTTCTACAATGAAAAGTGGATTATTGATGATATTAAATGCTTCATGCTGCCTTTCAACTCTAAATCACATCTTTAAACTAAGTGCC
Ca_LG_7:2685767	GTAATTTGATGTGCAGCCTCTTCCAACTAAACTTGTTCTAAGAAAAAAGAGGGCGAAAGAGGGAAGATCTGGTGATGAGGTTGAGCAATTTCCAATTCCG	G/T	GCTAGAGTGACTGTGAGGAGAAGGTCAAGTGTTGCTGCAATTGAGCGGAAGGATTCTGAGGTATGGCAACCACGTTATCTTACAAGCTTTCAATTTTATT
Ca_LG_7:2739241	GCAGAGACACTATTTTGGTACAACAAAAACGACGACGTTTCATCCTATAGAGGATTAGAACTATGGAACCAACAGCAGCAACAACAGCAGCAGGAAGATG	G/T	TAATAGCAGGAGGAACGCAACAAGCACGGCCGTTTTTCTCGCGAGATCTCTATGGTTTAGGTGTGGGACCCAGCAGGGTTTCATCAGATGATCATCAGAC
Ca_LG_7:3052289	NNNNNNNNNNNNNNNNNNNNNNNNNNNNNNNNNNNNNNNNNNNNNNNNNNNNNNNNNGGGTAATTTGATTGTATGAGGTTGCACAACATTGCTGCTTTTTC	C/G	TGTTTTCAGCTCTAAAACATGAATGGCATTATAATATGATATCTTCTTTTGCAGCATCGAGAACCCAAAGTTTTCTGCCAAGGCCTCTGGATTTTTCCAC
Ca_LG_7:3052317	NNNNNNNNNNNNNNNNNNNNNNNNNNNNNGGGTAATTTGATTGTATGAGGTTGCACAACATTGCTGCTTTTTCTGTTTTCAGCTCTAAAACATGAATGGCA	A/G	TTATAATATGATATCTTCTTTTGCAGCATCGAGAACCCAAAGTTTTCTGCCAAGGCCTCTGGATTTTTCCACCTTTTAGGTTTGTGATATAGTTGTATCT
Ca_LG_7:3052675	TATACCTGTACAAACTTTGGGCGGCACACGCAGGGTAATGGCATATTATTTATCAAAAACTGAAAGTGCCTGGCAGCACCATATTTGATGGCCAATTTTG	G/T	CATTATTGTTTACCTATTCTTTGCTAGTTGTCTGCCATTGTTCTAAAACGTGTTTGTTAATGTTTTGCTGAAATTGCTTGGCTAGCACTTTGTTGAAAAG
Ca_LG_7:3052682	GTACAAACTTTGGGCGGCACACGCAGGGTAATGGCATATTATTTATCAAAAACTGAAAGTGCCTGGCAGCACCATATTTGATGGCCAATTTTGCATTATT	T/A	GTTTACCTATTCTTTGCTAGTTGTCTGCCATTGTTCTAAAACGTGTTTGTTAATGTTTTGCTGAAATTGCTTGGCTAGCACTTTGTTGAAAAGGAAAAAA
Ca_LG_7:3168420	TCGCCGTCAAGTTCAACACCATACTTAAGCCACTGCCAATTCTGAACCCTGCTAATCTCGGCCGTTGCAGCATCCTCCATGAGATTGTAAAGAGGCACAG	G/A	AACCAGCTCCAGTGAGCCATGCTGCCACATACTGAATTCCAACCCGTGTATTCAACCGTAGACCTTCCATCGTTCGGACTCCTCTAGGTATCTGCAAGAG
Ca_LG_7:4501736	GAAAATAGAAATATACATTGAGGTGTATACCTGGATTATCAGCTACGAAACGGACTGCTGCCCATCCACCGACCGGAACTCCAATTGTATTCATGTACGC	C/G	GGGATCCACTAAATTGAATTGTGCAGCTAGTGGATCATAGTTTCCTGTGCCATATCCTACAACATAAAAGCTGTAGCCATGGATGTGAATTGGGTGGTTT

Positions	-100 bp flanking sequences	SNPs within *desi* cultivar	+100 bp flanking sequences
Ca_LG_7:4612116	ATCCCCACCGCCAGAACCAATGCTGCTATTCTCCGGCAACGCAGCACTCTTAACCATCATACGAGCCGCTTCCGCCGCTGCAGCCTGAATATCCCTTGCT	T/C	GAAGACGGTGGAGGAAGCAACGGTAAACGGTGCACCTCGTCGGGGAAATTGAGCTGCGCTTTTCGACCTTTCAAACAGTACGCCGCAACGTCGTAGGCTT
Ca_LG_7:5320476	TGCCTTCTCCATCTCTGGCGTCAACTGCATGTACCCAATAAGAGAATATCAATATACTGCAGGACAATTTCTACGAAGTAAATGCCAATTCAGAAATAAT	T/C	TATTACCGCAGGGGGCCTAAGAGGCTGATTAGCAGCACCCACCAGAACAGAAACCACAGATTGTGGTCCCAGAGGACCAGAATGCTGTACTAGATTTTCT
Ca_LG_7:6312451	AGACGGGCAATCATCACCTTTATCCAGTATTGCAAAAAGAATGAGGCCTGCTTCCACAGGTGTTGATGCAATGCAGCAGCAGCAAATAGGCTCACACGTC	C/T	GACGCCCTTCAAGGACCGGATATTAATTGGCAGAATACACTATTTCAACAACAAGCAATGGCCAGAGGTATTCAGTATTCCAGTGGTGGCATTCAGAAGT
Ca_LG_7:6312484	AAAAAGAATGAGGCCTGCTTCCACAGGTGTTGATGCAATGCAGCAGCAGCAAATAGGCTCACACGTCGACGCCCTTCAAGGACCGGATATTAATTGGCAG	G/A	AATACACTATTTCAACAACAAGCAATGGCCAGAGGTATTCAGTATTCCAGTGGTGGCATTCAGAAGTTTCCCCCACAGGTTTTTGAAGGGGGTTTAAATC
Ca_LG_7:6316384	ACACCAACAATCCCAACAGCAGCAACACCACCAACAGCAGTTGCAATTACAACAACAGCATTTACATCAGCAGTTGCAGCAGCAATTACAGCAGCAGCAG	G/A	CAACAACAACAACAGGAAACAACTTCGCAATTGCAGGCAGTTGTTTCTCCCCCACAAGTGGGATCACCGTCGACAATGGGAGTTTCATCATTAAGCCAAC
Ca_LG_7:6496052	TGTTTAACAAATAACACTGCTATTTTGAAAATAACTATGCTTATGTTACATAAGCTTATGTAATAACCATAATAATAGAATCCATTTGGGAGAAACCATC	C/A	TAAAACTTTTCCTCACCGTAAATGTATGCTGTTGTTTAGCAGCACCATAGCTTTCTTTCACTATGCGGCCTGCAACAATCCTTTTGCCGCACGGTGGACC
Ca_LG_7:6639740	GAGGTGAGTGCGGTGGAAGAAGCGGCACCGTGAACGGCTGCCAGACGCGCCGTCACGCGCGGTGGCTATTGGCGGCGCGTGGATCTCACGCGCGTGAGGG	G/T	GGTTGCTGATGACGTAGCTGAAGGGTTTGAGTGATCTATGGTGTACGGCGCTGCTGGAGTGGTCGTTCGTTGAAAAAAATCATCAGAAACGGTGACGACA
Ca_LG_7:7866464	TTCTCCGAATCTCAACCTAGGGTTTTCAAAGCTGAAAAGGTTTCTTTGATCCCCAAGAGGCATTGGTAAGCCTGGTCTTGCAGTTGACCTACCGAGTCCC	C/T	GCAGCTTGAAGAGTGGCTAGACTTTGTGCTGGGAGCTGACCAGCAGCTGCAAGAGTTTGAAGATCAAGACCACTAATAGATGAAATTGTCCCGAATGATG
Ca_LG_7:8133197	AGTTAGAGGCAATGACACTATCATTTGCTACAACTACAACTAGGCTTATGCACAATTAAGGCATTGAAATACCTGAGCAGCAAATAATGACAAAATACCG	G/A	CTGCCAGCACCCACATCAACTACTACGCGGCCAGTAAAATCAGCACGGTTCTCCATAACCGCGGCATAATAGGTTCCTAAAACAATATCACGAATGAAAT
Ca_LG_7:8133200	TAGAGGCAATGACACTATCATTTGCTACAACTACAACTAGGCTTATGCACAATTAAGGCATTGAAATACCTGAGCAGCAAATAATGACAAAATACCGCTG	G/A	CCAGCACCCACATCAACTACTACGCGGCCAGTAAAATCAGCACGGTTCTCCATAACCGCGGCATAATAGGTTCCTAAAACAATATCACGAATGAAATCAT
Ca_LG_7:8133209	TGACACTATCATTTGCTACAACTACAACTAGGCTTATGCACAATTAAGGCATTGAAATACCTGAGCAGCAAATAATGACAAAATACCGCTGCCAGCACCC	C/A	ACATCAACTACTACGCGGCCAGTAAAATCAGCACGGTTCTCCATAACCGCGGCATAATAGGTTCCTAAAACAATATCACGAATGAAATCATTAGGTAAGA
Ca_LG_7:8133218	CATTTGCTACAACTACAACTAGGCTTATGCACAATTAAGGCATTGAAATACCTGAGCAGCAAATAATGACAAAATACCGCTGCCAGCACCCACATCAACT	T/C	ACTACGCGGCCAGTAAAATCAGCACGGTTCTCCATAACCGCGGCATAATAGGTTCCTAAAACAATATCACGAATGAAATCATTAGGTAAGAGTAAAATAA

Positions	-100 bp flanking sequences	SNPs within *desi* cultivar	+100 bp flanking sequences
Ca_LG_7:8133224	CTACAACTACAACTAGGCTTATGCACAATTAAGGCATTGAAATACCTGAGCAGCAAATAATGACAAAATACCGCTGCCAGCACCCACATCAACTACTACG	G/A	CGGCCAGTAAAATCAGCACGGTTCTCCATAACCGCGGCATAATAGGTTCCTAAAACAATATCACGAATGAAATCATTAGGTAAGAGTAAAATAAATATAT
Ca_LG_7:8415484	GGTGATGTAGCGGTGCAGTGTGTTGTAGCAGTATGACAATATTGCAGTGGAGCAGCAGCGGTGTGGTGGTGCTATGCAGTGGAGCAGCGGTGTGGTGGTG	G/A	CTATGCAGTGGAGCAGCAGTGCGGTGGTGTTGTACCTGCGATGGGAGGGGCGACGAAGGAAGGAAAAGGGGTTTTATGTTTCTGATGTGATTTTATGTTT
Ca_LG_7:8421007	GCTTTGCCATGATGTTAGAAGATGTTTCAGATTGTGCAGGTGGTTGGGCACTTGCTTGGGCTTGATCAGCACTGGGTTGTGCCTGATCAGCAGTAGATGA	A/G	AGCTCTTGTCCATACTCCATTTATGCGTCGCAGTTGCATTTGGTTCACGATTGATTCACCAAATGTCAGTGTGGATTTGAGAGATTCTTCATTGGCTAGA
Ca_LG_7:8519553	AATTGTAGATAACTTTTTAATATTGAATGCAATTAACCCTACTAACATATTTGCATTGCAGTCTAAACTCATATGTACCATTGCTAACTCAAAGTGAGAG	G/C	AATGGCACAGTGGACTTAATCTTCTTAGTTTACTATTCATGACATGAATTCTACAAATCCCAAATTTTAGCTTAACCGAAATAAACCAGAAACAAATGGT
Ca_LG_7:8519563	AACTTTTTAATATTGAATGCAATTAACCCTACTAACATATTTGCATTGCAGTCTAAACTCATATGTACCATTGCTAACTCAAAGTGAGAGAATGGCACAG	G/C	TGGACTTAATCTTCTTAGTTTACTATTCATGACATGAATTCTACAAATCCCAAATTTTAGCTTAACCGAAATAAACCAGAAACAAATGGTACTTATTATA
Ca_LG_7:8519578	AATGCAATTAACCCTACTAACATATTTGCATTGCAGTCTAAACTCATATGTACCATTGCTAACTCAAAGTGAGAGAATGGCACAGTGGACTTAATCTTCT	T/C	TAGTTTACTATTCATGACATGAATTCTACAAATCCCAAATTTTAGCTTAACCGAAATAAACCAGAAACAAATGGTACTTATTATATAATTGTTTAATATT
Ca_LG_7:8519630	CCATTGCTAACTCAAAGTGAGAGAATGGCACAGTGGACTTAATCTTCTTAGTTTACTATTCATGACATGAATTCTACAAATCCCAAATTTTAGCTTAACC	C/T	GAAATAAACCAGAAACAAATGGTACTTATTATATAATTGTTTAATATTCAACCAATGAACTTTTTATCACAATTTTTTTACCGTTAAAATATTATTCTTA
Ca_LG_7:9520523	AGACCACGGTCTCAATTTGCAAGCATGACAAAGCCTTTGAACTTTCCAAATTATCTTTAGTTTTTGGATCATAGGTAGGTTCAGGTGGCTGCACTGCAGC	C/A	CAAGGAAGATGTCTCCACAACAGGATCTGGGTGTGGAGGTCCAATAGATACTTTTGGTGGTCTCTGCATATGGAGACAATTTAATACCACAATAGTGTGC
Ca_LG_7:9520544	AGCATGACAAAGCCTTTGAACTTTCCAAATTATCTTTAGTTTTTGGATCATAGGTAGGTTCAGGTGGCTGCACTGCAGCCAAGGAAGATGTCTCCACAAC	C/T	AGGATCTGGGTGTGGAGGTCCAATAGATACTTTTGGTGGTCTCTGCATATGGAGACAATTTAATACCACAATAGTGTGCTTGCGTATTTTGAAATAACAA
Ca_LG_7:9520545	GCATGACAAAGCCTTTGAACTTTCCAAATTATCTTTAGTTTTTGGATCATAGGTAGGTTCAGGTGGCTGCACTGCAGCCAAGGAAGATGTCTCCACAACA	A/T	GGATCTGGGTGTGGAGGTCCAATAGATACTTTTGGTGGTCTCTGCATATGGAGACAATTTAATACCACAATAGTGTGCTTGCGTATTTTGAAATAACAAA
Ca_LG_7:9520557	CTTTGAACTTTCCAAATTATCTTTAGTTTTTGGATCATAGGTAGGTTCAGGTGGCTGCACTGCAGCCAAGGAAGATGTCTCCACAACAGGATCTGGGTGT	T/C	GGAGGTCCAATAGATACTTTTGGTGGTCTCTGCATATGGAGACAATTTAATACCACAATAGTGTGCTTGCGTATTTTGAAATAACAAACTTCTAGCATAC
Ca_LG_7:9881086	AATGATAGTAGGGTTGAGATATCCTCGCCCTCCGAACCCAAACGTGAAGGTCTCCCCTCATCTGAATCTTTGCAGGGGAATCTCCACTCATTGCTTCCCT	T/C	TAATATCCTCCACCACATCATGGTGGTTTACAGGAGATCCCAGAGGCTCGCTCAGAAAGGCTGTTACCATACATTTTGGCACAACTTTACTCAAGTAGCC

Positions	-100 bp flanking sequences	SNPs within *desi* cultivar	+100 bp flanking sequences
Ca_LG_7:9881110	TCGCCCTCCGAACCCAAACGTGAAGGTCTCCCCTCATCTGAA TCTTTGCAGGGGAATCTCCACTCATTGCTTCCCTTAATATCCT CCACCACATCATGGT	T/G	GGTTTACAGGAGATCCCAGAGGCTCGCTCAGAAAGGCTGTT ACCATACATTTTGGCACAACTTTACTCAAGTAGCCACTAGAC TCACTATAGTAGTTCGT
Ca_LG_7:9881140	CCCTCATCTGAATCTTTGCAGGGGAATCTCCACTCATTGCTTC CCTTAATATCCTCCACCACATCATGGTGGTTTACAGGAGATCC CAGAGGCTCGCTCA	A/G	GAAAGGCTGTTACCATACATTTTGGCACAACTTTACTCAAGT AGCCACTAGACTCACTATAGTAGTTCGTTCGTCTGTCCTTGT TGAAATCAATGCTTTT
Ca_LG_7:9881375	CAAACCATGTAGTTAGGTAGAGACAATCAATACACTTATACGC AATGAAGGGAATCTGGGATCCCTATCGATATAAAAAACGAATA ATATGTGTATAGAT	T/G	AAGCTACCTTTCTCACACTCAATAGATATATCTGGTACAGTTA AGTACAATACGAGACAATGGAATGCTATGGGATGGATGGTA TAGGGTTGTTTGTGCC
Ca_LG_7:9881390	GGTAGAGACAATCAATACACTTATACGCAATGAAGGGAATCT GGGATCCCTATCGATATAAAAAACGAATAATATGTGTATAGAT AAGCTACCTTTCTCA	A/T	CACTCAATAGATATATCTGGTACAGTTAAGTACAATACGAGA CAATGGAATGCTATGGGATGGATGGTATAGGGTTGTTTGTG CCCAAAAGTGACGATTC
Ca_LG_7:9881403	AATACACTTATACGCAATGAAGGGAATCTGGGATCCCTATCGA TATAAAAAACGAATAATATGTGTATAGATAAGCTACCTTTCTCA CACTCAATAGATA	A/G	TATCTGGTACAGTTAAGTACAATACGAGACAATGGAATGCTA TGGGATGGATGGTATAGGGTTGTTTGTGCCCAAAAGTGACG ATTCTCTTGCCCCCTTG
Ca_LG_7:9881414	ACGCAATGAAGGGAATCTGGGATCCCTATCGATATAAAAAAC GAATAATATGTGTATAGATAAGCTACCTTTCTCACACTCAATA GATATATCTGGTACA	A/G	GTTAAGTACAATACGAGACAATGGAATGCTATGGGATGGAT GGTATAGGGTTGTTTGTGCCCAAAAGTGACGATTCTCTTGCC CCCTTGTCCATAAGGAC
Ca_LG_7:9881417	CAATGAAGGGAATCTGGGATCCCTATCGATATAAAAAACGAAT AATATGTGTATAGATAAGCTACCTTTCTCACACTCAATAGATAT ATCTGGTACAGTT	T/A	AAGTACAATACGAGACAATGGAATGCTATGGGATGGATGGT ATAGGGTTGTTTGTGCCCAAAAGTGACGATTCTCTTGCCCCC TTGTCCATAAGGACCTT
Ca_LG_7:9881418	AATGAAGGGAATCTGGGATCCCTATCGATATAAAAAACGAATA ATATGTGTATAGATAAGCTACCTTTCTCACACTCAATAGATATA TCTGGTACAGTTA	A/C	AGTACAATACGAGACAATGGAATGCTATGGGATGGATGGTA TAGGGTTGTTTGTGCCCAAAAGTGACGATTCTCTTGCCCCCT TGTCCATAAGGACCTTG
Ca_LG_7:9881434	GATCCCTATCGATATAAAAAACGAATAATATGTGTATAGATAA GCTACCTTTCTCACACTCAATAGATATATCTGGTACAGTTAAG TACAATACGAGACA	A/G	ATGGAATGCTATGGGATGGATGGTATAGGGTTGTTTGTGCC CAAAAGTGACGATTCTCTTGCCCCCTTGTCCATAAGGACCTT GTGGCATACATGTGAAA
Ca_LG_7:10260505	CCTATATTCGTTACATTCTAAAGGACAATAGATACACACATTAT TTCAAGTTAATTTCATTGAACCTTATCTGGCTCAACCCACAGG TCAGATTTCAAAA	A/C	AAACCTATCGATATACTTGTTTTGATGTAGCGTAAATGCCTG ATGTAGATGCTATCACTTATTCTGCCTCGGTCGGATGTTGCC TTAGTTGATGTGATAG
Ca_LG_7:10260548	TTTCAAGTTAATTTCATTGAACCTTATCTGGCTCAACCCACAG GTCAGATTTCAAAAAAACCTATCGATATACTTGTTTTGATGTAG CGTAAATGCCTGA	A/C	TGTAGATGCTATCACTTATTCTGCCTCGGTCGGATGTTGCCT TAGTTGATGTGATAGAATCCCCCTTGCTTCCTAATCATTTACT ATGGTCTTGCTCAGT
Ca_LG_7:10264601	ATATTCAATTCCATTCCTCCATAACCATGCTTTCCAACTCGATC CAGCTCTCAAACTATACGATCCTCGTCACCAGTCAGCAGCTA ATACTGATAGCTCA	A/C	TGCGGACCCTTGCCTTCCATCTTCCTTCCTAGGCTCGGATGT TACATCCGAATGTTGCAGCCTAGTCAAATCGGTATTTCCCCG CCTACCATTAAATCAA

Positions	-100 bp flanking sequences	SNPs within *desi* cultivar	+100 bp flanking sequences
Ca_LG_7:10264620	CATAACCATGCTTTCCAACTCGATCCAGCTCTCAAACTATACGATCCTCGTCACCAGTCAGCAGCTAATACTGATAGCTCATGCGGACCCTTGCCTTCCA	A/C	TCTTCCTTCCTAGGCTCGGATGTTACATCCGAATGTTGCAGCCTAGTCAAATCGGTATTTCCCCGCCTACCATTAAATCAAAATAAAGAAGTAGGGGCTT
Ca_LG_7:10266275	CCAGAAGGGATTGGCTTCACGAATGCTTAGCTCAGAACCTAAACTACTGGCTTTCTCGCATTGAAGGTATCTGTTGAGGGAATCTATCCCAAAGGTAGGT	T/C	CCAAGGCTGCTTAATGAGCACCCCTTGAAAGAACAAGAGCTGCATTCATCCCACCTGCGGAAGTCTCTTAACCACCTATAAATGTCCCTTTTTGGCTAGT
Ca_LG_7:10266295	GAATGCTTAGCTCAGAACCTAAACTACTGGCTTTCTCGCATTGAAGGTATCTGTTGAGGGAATCTATCCCAAAGGTAGGTCCAAGGCTGCTTAATGAGCA	A/G	CCCCTTGAAAGAACAAGAGCTGCATTCATCCCACCTGCGGAAGTCTCTTAACCACCTATAAATGTCCCTTTTTGGCTAGTATTATCCAAAATCTAGAAAG
Ca_LG_7:10267731	GAAAGTTACCTAGACTAATAAGGCGAGACTTAGGGACCTAGCTAGATTCACTAGCACCGTCTACTCTCACAGCGGATAGGGATTCCGTTCCCTTTCTAAT	T/C	TGTAAGAGTTGCTACGGATATAAGTCCCACAGACCCCCCTTCTCGAGCTTATTCTTTTTAAGAGACTGATGCCAGCTATGAATATACGATCGAATGCGCT
Ca_LG_7:10267765	GACCTAGCTAGATTCACTAGCACCGTCTACTCTCACAGCGGATAGGGATTCCGTTCCCTTTCTAATTGTAAGAGTTGCTACGGATATAAGTCCCACAGAC	C/T	CCCCCTTCTCGAGCTTATTCTTTTTAAGAGACTGATGCCAGCTATGAATATACGATCGAATGCGCTCTTGTCTATTTTTAGGTTTCTTACTTTTATTTTT
Ca_LG_7:10267806	ATAGGGATTCCGTTCCCTTTCTAATTGTAAGAGTTGCTACGGATATAAGTCCCACAGACCCCCCTTCTCGAGCTTATTCTTTTTAAGAGACTGATGCCAG	G/A	CTATGAATATACGATCGAATGCGCTCTTGTCTATTTTTAGGTTTCTTACTTTTATTTTTGCATTTTCTCTTTAAAGCCTTCCCCAACGAAACAAATGATG
Ca_LG_7:10267847	GATATAAGTCCCACAGACCCCCCTTCTCGAGCTTATTCTTTTTAAGAGACTGATGCCAGCTATGAATATACGATCGAATGCGCTCTTGTCTATTTTTAGG	G/A	TTTCTTACTTTTATTTTTGCATTTTCTCTTTAAAGCCTTCCCCAACGAAACAAATGATGTTGCTCCTTCTGTTTAGAGTTCACATGACTTAGCTCTCGTT
Ca_LG_7:10267868	CCTTCTCGAGCTTATTCTTTTTAAGAGACTGATGCCAGCTATGAATATACGATCGAATGCGCTCTTGTCTATTTTTAGGTTTCTTACTTTTATTTTTGCA	A/C	TTTTCTCTTTAAAGCCTTCCCCAACGAAACAAATGATGTTGCTCCTTCTGTTTAGAGTTCACATGACTTAGCTCTCGTTGTGCGTGGACTTCCTAATCGA
Ca_LG_7:10267879	TTATTCTTTTTAAGAGACTGATGCCAGCTATGAATATACGATCGAATGCGCTCTTGTCTATTTTTAGGTTTCTTACTTTTATTTTTGCATTTTCTCTTTA	A/G	AAGCCTTCCCCAACGAAACAAATGATGTTGCTCCTTCTGTTTAGAGTTCACATGACTTAGCTCTCGTTGTGCGTGGACTTCCTAATCGAACTAAGATGGA
Ca_LG_7:10267895	ACTGATGCCAGCTATGAATATACGATCGAATGCGCTCTTGTCTATTTTTAGGTTTCTTACTTTTATTTTTGCATTTTCTCTTTAAAGCCTTCCCCAACGA	A/G	AACAAATGATGTTGCTCCTTCTGTTTAGAGTTCACATGACTTAGCTCTCGTTGTGCGTGGACTTCCTAATCGAACTAAGATGGATCGGGTTGTTGGCTCA
Ca_LG_7:10282318	CGGGCAGGAGTGGGACATTCCATAAAAATATTTCTGATCCTCTAGCTCTCACTCCTTGCCCTATTCGGTCAAGCAGCTTCACTCCTCTCAAATCCTATTA	A/T	TTTCATCGACAAGTAGGGTTAATTATAAGGTTCCTTATTCTGGTTGTCAAGCTGAAGAAGAGGGAGAAAGAATGGGCACAACCCCACCAGCAGCCCGTGT
Ca_LG_7:10282371	CCTTGCCCTATTCGGTCAAGCAGCTTCACTCCTCTCAAATCCTATTATTTCATCGACAAGTAGGGTTAATTATAAGGTTCCTTATTCTGGTTGTCAAGCT	T/G	GAAGAAGAGGGAGAAAGAATGGGCACAACCCCACCAGCAGCCCGTGTTTTCGACCTGAAAGGAATGGAAAATGAAATAAGAATTCGTGTTCACTATCTAT

Positions	-100 bp flanking sequences	SNPs within *desi* cultivar	+100 bp flanking sequences
Ca_LG_7:10282399	CTCCTCTCAAATCCTATTATTTCATCGACAAGTAGGGTTAATTATAAGGTTCCTTATTCTGGTTGTCAAGCTGAAGAAGAGGGAGAAAGAATGGGCACAA	A/G	CCCCACCAGCAGCCCGTGTTTTCGACCTGAAAGGAATGGAAAATGAAATAAGAATTCGTGTTCACTATCTATGGAGCGGAGTGTCTATCCTTAATCTCCT
Ca_LG_7:10341134	CCTTCCCGGTCCTCTCGTACTAGGGAAAGGTCCTCTCAATGCTCTAACGCCCACACCGGATATGGACCGAACTGTCTCACGACGTTCTGAACCCAGCTCA	A/G	CGTACCGCTTTAATGGGCGAACAGCCCAACCCTTGGAACCTACTACAGCTCCAGGTGGCGAAGAGCCGACATCGAGGTGCCAAACCTTCCCGTCGATGTG
Ca_LG_7:10503482	TTTGTGGTGGCCTTTGCAGCCACAAAAGGTAACAGTCTCAGGCGTTTGTGGCGGCCTGTGCAGCCACAAATGGACATATCCCTTCAGGCTTTTGTGGCGG	G/A	ATTTGACCCTCACAAATTTTAGATTTTGTGGCTGAAAAAGCCCTCACAAAAATTTTAAAAATTTAAGTGGATTTTGTGGCTGCTTAAGCCCTCACAAAAT
Ca_LG_7:10503536	CCTGTGCAGCCACAAATGGACATATCCCTTCAGGCTTTTGTGGCGGATTTGACCCTCACAAATTTTAGATTTTGTGGCTGAAAAAGCCCTCACAAAAATT	T/C	TTAAAAATTTAAGTGGATTTTGTGGCTGCTTAAGCCCTCACAAAATCACAGCGTTGTGATTTTGTGAGGGCTTAGGCAGCCAAAAATTCCACCTAAATTT
Ca_LG_7:10510335	ATGTCGTCTTTGATTTGCGCTCCTCACTTTGTCGGTCGTCGCGTCACAATCGATGCAGCTCACTCCTTCACATCACAATTTGTGTTCCTAATCGGTGCTA	A/G	CTCTGTCACGTCACGATCGGTGCTGCTCACTCCGCCGCGTCACGATATGTGCTCGCCTCTACGATTCCAACCTATTTTCATCTCGTCCCTCTATGTTCGT
Ca_LG_7:11185996	CAACCCTGCATTCCAGTATCCTATGATGCCAGCATGTAGAGCTACAACGGCTTCCCCTATCTTTGTTGCAGCTCCCGCCGAGAAAGGAAACTTTGTTGTC	C/T	GACTTTCTCATGGGTGGAGTTTCAGCTGCTGTGTCTAAAACTGCTGCTGCTCCAATTGAACGAATTAAGCTTTTGATCCAGAACCAAGATGAGATGATTA
Ca_LG_7:11292502	TTCATTTGGTTGAAAAGAAGATGGAGAAGTCGAAAGAAGAGATGTAACGGATGGTGGTGTTGAGCAGAAATATCCTGCTAAGGACTGTGCTTCGAGTGAA	A/C	AAAAACTTTTGTTGCTGCCGTGCTAGCTGCATTCGCATTTCACGATTCTCCTTTTGTAGCTCAAGTACTAGCTTGGCCTGGTATGTTCCTGTTTCAGTTA
Ca_LG_7:11292507	TTGGTTGAAAAGAAGATGGAGAAGTCGAAAGAAGAGATGTAACGGATGGTGGTGTTGAGCAGAAATATCCTGCTAAGGACTGTGCTTCGAGTGAAAAAAA	A/G	CTTTTGTTGCTGCCGTGCTAGCTGCATTCGCATTTCACGATTCTCCTTTTGTAGCTCAAGTACTAGCTTGGCCTGGTATGTTCCTGTTTCAGTTACTGGA
Ca_LG_7:11292544	TGTAACGGATGGTGGTGTTGAGCAGAAATATCCTGCTAAGGACTGTGCTTCGAGTGAAAAAAACTTTTGTTGCTGCCGTGCTAGCTGCATTCGCATTTCA	A/C	CGATTCTCCTTTTGTAGCTCAAGTACTAGCTTGGCCTGGTATGTTCCTGTTTCAGTTACTGGAAATAGATCCTCATTTGCGTCATTTGCCTGCACCCATG
Ca_LG_7:11293446	TGAAGATGTTTCATTTGCACGAGTTGGTTCTGTAGTTCTGTTATGATTTCCTTGCTGAAGCAATATCATCACCTGAAGCAGCAAAAGCAAATTAAAACTC	C/T	AAAAAATTCATTCAGCAATCTTCCAATATACCAGAATTGTTCAAAATATTACTTCATCAGTAGAGTAAACTCTGTATTGTGTAAGACCTACTGCCACAAT
Ca_LG_7:11293484	TGTTATGATTTCCTTGCTGAAGCAATATCATCACCTGAAGCAGCAAAAGCAAATTAAAACTCAAAAAATTCATTCAGCAATCTTCCAATATACCAGAATT	T/G	GTTCAAAATATTACTTCATCAGTAGAGTAAACTCTGTATTGTGTAAGACCTACTGCCACAATCCCCTAAAATATAGTTAGACAAGGTACGATCAGATTGT
Ca_LG_7:11293545	CAAAAAATTCATTCAGCAATCTTCCAATATACCAGAATTGTTCAAAATATTACTTCATCAGTAGAGTAAACTCTGTATTGTGTAAGACCTACTGCCACAA	A/T	TCCCCTAAAATATAGTTAGACAAGGTACGATCAGATTGTGATTTAACGGGATACAACAAAAACAAGAACTCTTCACAACAAACAAAAATTCACAGGTACC

Positions	-100 bp flanking sequences	SNPs within *desi* cultivar	+100 bp flanking sequences
Ca_LG_7:11333084	AGAATGTTTTTGCTGTCGCAGTTGCAACAGAATATTCTTTCCCTGAGGATGACAAGGTCAAGGAGTTCCTAAAGGACCCAAGCAAGTTTGCTGTAGCTGT	T/C	AGTTGCTTCCCCAGCTGCTGATTCTGGTACTGCCCCTTCTGCTGCTGCCAAGGAGGAGGAGAAGAAGGATGAATTGATGGTATAGTCTATGTTACTGATC
Ca_LG_7:11333092	TTTGCTGTCGCAGTTGCAACAGAATATTCTTTCCCTGAGGATGACAAGGTCAAGGAGTTCCTAAAGGACCCAAGCAAGTTTGCTGTAGCTGTAGTTGCTT	T/G	CCCCAGCTGCTGATTCTGGTACTGCCCCTTCTGCTGCTGCCAAGGAGGAGGAGAAGAAGGATGAATTGATGGTATAGTCTATGTTACTGATCCTGGATTT
Ca_LG_7:11882749	CTATTTTTACAAAAGATATGAAGGCTGTGAGGGTGTTCTTGAACACTGAATCCCTGTATTTCATACCCATCGGGAACTTGCCCAGGCTGCGCAACCTCTA	A/G	GTTGAGGTGTGGGCTTTCCTCTGGGATGTTCTAAGTCCCTAAAGATAGACTCCCCACTTATGCCCAAGGAATTTCTTTTGAGCGCACCATGAAGCCGTTG
Ca_LG_7:11882765	TATGAAGGCTGTGAGGGTGTTCTTGAACACTGAATCCCTGTATTTCATACCCATCGGGAACTTGCCCAGGCTGCGCAACCTCTAGTTGAGGTGTGGGCTT	T/C	TCCTCTGGGATGTTCTAAGTCCCTAAAGATAGACTCCCCACTTATGCCCAAGGAATTTCTTTTGAGCGCACCATGAAGCCGTTGACTAATTCCCTAAGAA
Ca_LG_7:11882775	GTGAGGGTGTTCTTGAACACTGAATCCCTGTATTTCATACCCATCGGGAACTTGCCCAGGCTGCGCAACCTCTAGTTGAGGTGTGGGCTTTCCTCTGGGA	A/C	TGTTCTAAGTCCCTAAAGATAGACTCCCCACTTATGCCCAAGGAATTTCTTTTGAGCGCACCATGAAGCCGTTGACTAATTCCCTAAGAAGGCTGTGAAT
Ca_LG_7:11882781	GTGTTCTTGAACACTGAATCCCTGTATTTCATACCCATCGGGAACTTGCCCAGGCTGCGCAACCTCTAGTTGAGGTGTGGGCTTTCCTCTGGGATGTTCT	T/C	AAGTCCCTAAAGATAGACTCCCCACTTATGCCCAAGGAATTTCTTTTGAGCGCACCATGAAGCCGTTGACTAATTCCCTAAGAAGGCTGTGAATTTATTT
Ca_LG_7:11882802	CTGTATTTCATACCCATCGGGAACTTGCCCAGGCTGCGCAACCTCTAGTTGAGGTGTGGGCTTTCCTCTGGGATGTTCTAAGTCCCTAAAGATAGACTCC	C/G	CCACTTATGCCCAAGGAATTTCTTTTGAGCGCACCATGAAGCCGTTGACTAATTCCCTAAGAAGGCTGTGAATTTATTTATGAAAACAACTCTGTTTAAA
Ca_LG_7:11882805	TATTTCATACCCATCGGGAACTTGCCCAGGCTGCGCAACCTCTAGTTGAGGTGTGGGCTTTCCTCTGGGATGTTCTAAGTCCCTAAAGATAGACTCCCCA	A/G	CTTATGCCCAAGGAATTTCTTTTGAGCGCACCATGAAGCCGTTGACTAATTCCCTAAGAAGGCTGTGAATTTATTTATGAAAACAACTCTGTTTAAAACC
Ca_LG_7:11882816	CATCGGGAACTTGCCCAGGCTGCGCAACCTCTAGTTGAGGTGTGGGCTTTCCTCTGGGATGTTCTAAGTCCCTAAAGATAGACTCCCCACTTATGCCCAA	A/G	GGAATTTCTTTTGAGCGCACCATGAAGCCGTTGACTAATTCCCTAAGAAGGCTGTGAATTTATTTATGAAAACAACTCTGTTTAAAACCACAACAACACA
Ca_LG_7:11882828	GCCCAGGCTGCGCAACCTCTAGTTGAGGTGTGGGCTTTCCTCTGGGATGTTCTAAGTCCCTAAAGATAGACTCCCCACTTATGCCCAAGGAATTTCTTTT	T/C	GAGCGCACCATGAAGCCGTTGACTAATTCCCTAAGAAGGCTGTGAATTTATTTATGAAAACAACTCTGTTTAAAACCACAACAACACATGTGAGAAATTT
Ca_LG_7:11882839	GCAACCTCTAGTTGAGGTGTGGGCTTTCCTCTGGGATGTTCTAAGTCCCTAAAGATAGACTCCCCACTTATGCCCAAGGAATTTCTTTTGAGCGCACCAT	T/G	GAAGCCGTTGACTAATTCCCTAAGAAGGCTGTGAATTTATTTATGAAAACAACTCTGTTTAAAACCACAACAACACATGTGAGAAATTTCCAAAATATTG
Ca_LG_7:12552938	TGTAAGCTTTTATGTGCCCAAAAAGAACCAAGCAAATCCACCACCTTCAAAGGGTCTTCAAGTTCAAAAATGGAAAAATGTGTATGCAGTATTTAGACAG	G/A	TTTGCTGGATATGTGAATGACAAAAATGTTGCAGAACAAGCTGCAACATTGAAGGATAGTATCAATGGTACTAAATGGCTATCTGCTATTGAAAAAAGTC

Positions	-100 bp flanking sequences	SNPs within *desi* cultivar	+100 bp flanking sequences
Ca_LG_7:12552943	GCTTTTATGTGCCCAAAAAGAACCAAGCAAATCCACCACCTTCAAAGGGTCTTCAAGTTCAAAAATGGAAAAATGTGTATGCAGTATTTAGACAGTTTGC	C/G	TGGATATGTGAATGACAAAAATGTTGCAGAACAAGCTGCAACATTGAAGGATAGTATCAATGGTACTAAATGGCTATCTGCTATTGAAAAAAGTCATAAA
Ca_LG_7:12552970	CAAATCCACCACCTTCAAAGGGTCTTCAAGTTCAAAAATGGAAAAATGTGTATGCAGTATTTAGACAGTTTGCTGGATATGTGAATGACAAAAATGTTGC	C/T	AGAACAAGCTGCAACATTGAAGGATAGTATCAATGGTACTAAATGGCTATCTGCTATTGAAAAAAGTCATAAAGCTCCTAATTTCTATGTGGCTCGGTAT
Ca_LG_7:12580076	TAGTGATGGCTCCTTTTTTAAATATTCATATATTGTAAGCTTTTATGTGCCTAAAAAGAACCAAGCAAATCCACCACCTGCAAAGGGTCTTCAAGTTCAA	A/C	AGATGGAAAAATGTGTATGCAGCAGTTAGACAGTTTAGTGGATTTGTGAATGACAAAAATGTTGCAGAACAAGCTGCGGCATTGAAGGATAGTATCAATG
Ca_LG_7:12580113	AGCTTTTATGTGCCTAAAAAGAACCAAGCAAATCCACCACCTGCAAAGGGTCTTCAAGTTCAAAGATGGAAAAATGTGTATGCAGCAGTTAGACAGTTTA	A/G	GTGGATTTGTGAATGACAAAAATGTTGCAGAACAAGCTGCGGCATTGAAGGATAGTATCAATGGTACTAAATGGCTATCTGCTATTGAAAAAAGTCATAA
Ca_LG_7:13714054	ATTATTTCACCTATTATTCCACCCAATGATCAAAGACAACTGCGGATTCACGATGGTTGAACAAAAGAGAGACAGGGCATGCAATTGATCCATACCAGCT	T/C	GGTACCCACCGCTAGACGAGGATCGACCCACCAGTATGGTAATTAGGTATCGACTTTTGACAAAGATACTGACTACATGATAGATAGAGATGCAAGGTAA
Ca_LG_7:13714058	TTTCACCTATTATTCCACCCAATGATCAAAGACAACTGCGGATTCACGATGGTTGAACAAAAGAGAGACAGGGCATGCAATTGATCCATACCAGCTGGTA	A/T	CCCACCGCTAGACGAGGATCGACCCACCAGTATGGTAATTAGGTATCGACTTTTGACAAAGATACTGACTACATGATAGATAGAGATGCAAGGTAATACA
Ca_LG_7:13714080	TGATCAAAGACAACTGCGGATTCACGATGGTTGAACAAAAGAGAGACAGGGCATGCAATTGATCCATACCAGCTGGTACCCACCGCTAGACGAGGATCGA	A/G	CCCACCAGTATGGTAATTAGGTATCGACTTTTGACAAAGATACTGACTACATGATAGATAGAGATGCAAGGTAATACACTACTTAAATGCATTATTGTAA
Ca_LG_7:13714089	ACAACTGCGGATTCACGATGGTTGAACAAAAGAGAGACAGGGCATGCAATTGATCCATACCAGCTGGTACCCACCGCTAGACGAGGATCGACCCACCAGT	T/C	ATGGTAATTAGGTATCGACTTTTGACAAAGATACTGACTACATGATAGATAGAGATGCAAGGTAATACACTACTTAAATGCATTATTGTAAACCATAGTT
Ca_LG_7:14030720	ATAAAATATTCATTGATTACTGGTTAACGTGATAAACAGAATAACAGTGAAGTATCAAAAGAAAAATATGGATGGCAGGCTACGCTCACGTCCTTATACA	A/G	AAAACCAACAAAATAATAATAGGGATAACAAATAGGGATATTAGCTAGTAGTGATAGCATGCAGTAAAATCTTTATAAAATAATAAATAAAAAAACAAAG
Ca_LG_7:14525832	CGATGCCTGGAGAGTCTTGAGAGATGCCTCTACTCTTGCATTATGAGCAGCTTGAATCTCCATGAATTCCAGTAGCTTCGTCAAGACCACAGAATGTGCT	T/G	TCGACTGGCTCAGCTTGTTCTGCAGTTGGCTGCACTTGTTCAGTAGACGGCTGAGCTCTAGTCCATATGCCGTTGATGCGCTTCAGTTGCATTTGGTTAA
Ca_LG_7:14571742	CGGTGTTTGTTGGTTTTGGATGCGCACTAGAGAGGAGGCAGCTGGTGTTGACACATCTGTAACAGAGTAAAAAAAAAATAGACAGAATAGTGACAGCTGA	A/G	TGTAAAGCACAGTTGTGTTTGTAAGAAAAAAAAAGTCACTGGAAAAACGCATAAGGAAGGCAGCGCTTTTAAGGCAGCTGTAGCGTTGTGGCTGGTTGCT
Ca_LG_7:14571783	CTGGTGTTGACACATCTGTAACAGAGTAAAAAAAAAATAGACAGAATAGTGACAGCTGATGTAAAGCACAGTTGTGTTTGTAAGAAAAAAAAAGTCACTG	G/T	GAAAAACGCATAAGGAAGGCAGCGCTTTTAAGGCAGCTGTAGCGTTGTGGCTGGTTGCTAGAAAGGCAGGCGGAGTTGACAGTGACAGATGATTGTATGA

**Positions**	**-100 bp flanking sequences**	**SNPs within *desi* cultivar**	**+100 bp flanking sequences**
Ca_LG_7:14694780	GCTTTTTAGCCACAAAATTTAAAATTTGTGAGGGTCAAATCCGTCACAAAAGCCTGAACTGACATGTCCATTTGTGGCTGCATAGGCCGCCACAAACGCA	A/T	TGAGACCGTTACCTTTTGTGGCTGCAAATGCCGCCACAAAGGAATAAAGTGCTACGTGTTTTTCTGGCCGTCCAGGCTACCACAAATATTTATATCCATT
Ca_LG_7:14694809	GAGGGTCAAATCCGTCACAAAAGCCTGAACTGACATGTCCATTTGTGGCTGCATAGGCCGCCACAAACGCATGAGACCGTTACCTTTTGTGGCTGCAAAT	T/G	GCCGCCACAAAGGAATAAAGTGCTACGTGTTTTTCTGGCCGTCCAGGCTACCACAAATATTTATATCCATTACATCTTGTGGCGGCAAAGGCCGCCACAA
Ca_LG_7:14694833	CTGAACTGACATGTCCATTTGTGGCTGCATAGGCCGCCACAAACGCATGAGACCGTTACCTTTTGTGGCTGCAAATGCCGCCACAAAGGAATAAAGTGCT	T/A	ACGTGTTTTTCTGGCCGTCCAGGCTACCACAAATATTTATATCCATTACATCTTGTGGCGGCAAAGGCCGCCACAAACGCCCAAAGGACCGTTGCCAGTT
Ca_LG_7:14694835	GAACTGACATGTCCATTTGTGGCTGCATAGGCCGCCACAAACGCATGAGACCGTTACCTTTTGTGGCTGCAAATGCCGCCACAAAGGAATAAAGTGCTAC	C/T	GTGTTTTTCTGGCCGTCCAGGCTACCACAAATATTTATATCCATTACATCTTGTGGCGGCAAAGGCCGCCACAAACGCCCAAAGGACCGTTGCCAGTTGT
Ca_LG_7:15388269	ACATCTTGCAGCATTGTGCTTTGATCAACAAAATTCGTCTGTTTTTTGGTGTTTTGTTGTTACCTATGAAGTCTATCTATTATGACTGTGGGTAGTAGAC	C/T	ATAGATGTTTAACAAGGTATGGTGTTTGCAGAGTTGGTTGAGGTAGCATTAAATTCGATTGGAAAACATTATGTTTTGGAAATGAGTGCTGCCCAATTTC
Ca_LG_7:15422373	TAGCAATCAATCACTTGTTGGCAACTCACAGTTTATTTGCTACATACAGGTGTTATCAACCCTCACACTAGCAGCAGCTTCATCAACAGCTAGTGTTGTC	C/A	GATCTATTGCTTAATTCCCATGGATCTTTTTGCCCTATAAAGCTTTGCTGCAGGTACAGGATATCTGCAGTTATGGCCTTCGTGTCATGGATTCTATCCT
Ca_LG_7:15584199	TTCCAACAACTCCGTCAGCATCGGTAACGGATTGGAGGTGAAAACAGCAAGCTTCAAGGGCAGCATTACAAGCAGAAACATCAGGTATCATGAAATCAAA	A/G	CTTCTCATCGGAAGCAACCCTGCAATTATGGATGAAAAATCTGTTACGGAAGAGAGAGGTGAGAAGTGAAAAAGCAGGGGGAGCAGTGTTGGCAGATTTC
Ca_LG_7:17466638	GCCAAAAGCCCTCACAAAGGGTAAAAAAACAACCACAAAGGGGCTATTTGTGGCAGCCTTAGGCAGCCACAAATAAGTTGGTCACAAACATTTGTGAGGG	G/A	CCTTTGCAGCCACAAAGTTTGAAAAATACGTTGTCATTTTTTAGGGCCTTTGCAGCCACAAAATGTATGCCTTTGTGATGGCCTTTGCAGCCACAAAATA
Ca_LG_7:17466639	CCAAAAGCCCTCACAAAGGGTAAAAAAACAACCACAAAGGGGCTATTTGTGGCAGCCTTAGGCAGCCACAAATAAGTTGGTCACAAACATTTGTGAGGGC	C/T	CTTTGCAGCCACAAAGTTTGAAAAATACGTTGTCATTTTTTAGGGCCTTTGCAGCCACAAAATGTATGCCTTTGTGATGGCCTTTGCAGCCACAAAATAC
Ca_LG_7:17601974	GAGCCCCAATCGTTGTCTTCTCCTTCTCGCCGTTCTCACTCAAAACTGTCGTTCTCACCGTTCTAGCCGTCAATCAATCAGCTGCACTCGCCGTCGAACC	C/T	AGTCGCGCACTCACCGTCGAACCGGAGCTGCACTCGCCGTCGAACCGGAGCTGCACTCGTCGTCGAACCAGTCGCGCCGTCGAACCCGAGCTGCACTCAC
Ca_LG_7:17602002	GCCGTTCTCACTCAAAACTGTCGTTCTCACCGTTCTAGCCGTCAATCAATCAGCTGCACTCGCCGTCGAACCAGTCGCGCACTCACCGTCGAACCGGAGC	C/T	TGCACTCGCCGTCGAACCGGAGCTGCACTCGTCGTCGAACCAGTCGCGCCGTCGAACCCGAGCTGCACTCACCGTCGTTCTCACCGTTCTCGCACCACTG
Ca_LG_7:17602025	TTCTCACCGTTCTAGCCGTCAATCAATCAGCTGCACTCGCCGTCGAACCAGTCGCGCACTCACCGTCGAACCGGAGCTGCACTCGCCGTCGAACCGGAGC	C/T	TGCACTCGTCGTCGAACCAGTCGCGCCGTCGAACCCGAGCTGCACTCACCGTCGTTCTCACCGTTCTCGCACCACTGGTAATTTCTTGAACCACCATTAT

Positions	-100 bp flanking sequences	SNPs within *desi* cultivar	+100 bp flanking sequences
Ca_LG_7:17602034	TTCTAGCCGTCAATCAATCAGCTGCACTCGCCGTCGAACCAGTCGCGCACTCACCGTCGAACCGGAGCTGCACTCGCCGTCGAACCGGAGCTGCACTCGT	T/C	CGTCGAACCAGTCGCGCCGTCGAACCCGAGCTGCACTCACCGTCGTTCTCACCGTTCTCGCACCACTGGTAATTTCTTGAACCACCATTATGTTCTCGCA
Ca_LG_7:19362097	TGCCTCCAAGAAAGAACGTCAGTGGGGATGATCAAATGGTTGAAGCTACGAATAACATGGATGTTTCTGTTGTTACACAGACATCTGCAAAGACTCTGCG	G/A	AGATCTAGAGAAGAGGGAAAGAGAGATTTGTGCTACTTAGTCAAGAGGATTGAAAGATTTTCGTCATTACCATCCTCCGAAGTTCAAGGGCGATGAAAAC
Ca_LG_7:20334840	GGGCACAGGTCTCTCAACTTTCCAATAGTCAAAAAGATACCTAAAAAGTGAGAAAAACGTGTTTTTATACATTCACATTGCGCTATACGCCCCAGGCGCG	G/A	GGAATTGGGCTTCGGGCGCGCCTGGACAGTGTCAAAAGGGTGGAAATGCACCCTAGGTTCAGCTGTTGCGCTTTAGGCACACAACTTCTTCTGTTTGACA
Ca_LG_7:20334844	ACAGGTCTCTCAACTTTCCAATAGTCAAAAAGATACCTAAAAAGTGAGAAAAACGTGTTTTTATACATTCACATTGCGCTATACGCCCCAGGCGCGGGAA	A/T	TTGGGCTTCGGGCGCGCCTGGACAGTGTCAAAAGGGTGGAAATGCACCCTAGGTTCAGCTGTTGCGCTTTAGGCACACAACTTCTTCTGTTTGACATTTT
Ca_LG_7:20334858	TTTCCAATAGTCAAAAAGATACCTAAAAAGTGAGAAAAACGTGTTTTTATACATTCACATTGCGCTATACGCCCCAGGCGCGGGAATTGGGCTTCGGGCG	G/C	CGCCTGGACAGTGTCAAAAGGGTGGAAATGCACCCTAGGTTCAGCTGTTGCGCTTTAGGCACACAACTTCTTCTGTTTGACATTTTCTGCATTTGTCTCA
Ca_LG_7:20334871	AAAAGATACCTAAAAAGTGAGAAAAACGTGTTTTTATACATTCACATTGCGCTATACGCCCCAGGCGCGGGAATTGGGCTTCGGGCGCGCCTGGACAGTG	G/C	TCAAAAGGGTGGAAATGCACCCTAGGTTCAGCTGTTGCGCTTTAGGCACACAACTTCTTCTGTTTGACATTTTCTGCATTTGTCTCATGTTTGGAGTCTG
Ca_LG_7:20919524	ATATGGGCTCAAACAATCGTCACAAACATATCCAAGGGGGCAAGTCCATATAGATCTCTTCTTCATGATCTCCATGGAGAAAAACATTTTTTACATCAAA	A/T	TTGATGTAAGTTCCAACTATAATTTGCTGCTAGAGATAATATTACCTCGTCTGCTTTCATTTTTGCAACCAGAGCAAATGTCTCCGAGTAATCCACCCCA
Ca_LG_7:20919528	GGGCTCAAACAATCGTCACAAACATATCCAAGGGGGCAAGTCCATATAGATCTCTTCTTCATGATCTCCATGGAGAAAAACATTTTTTACATCAAATTGA	A/G	TGTAAGTTCCAACTATAATTTGCTGCTAGAGATAATATTACCTCGTCTGCTTTCATTTTTGCAACCAGAGCAAATGTCTCCGAGTAATCCACCCCATATG
Ca_LG_7:20919531	CTCAAACAATCGTCACAAACATATCCAAGGGGGCAAGTCCATATAGATCTCTTCTTCATGATCTCCATGGAGAAAAACATTTTTTACATCAAATTGATGT	T/C	AAGTTCCAACTATAATTTGCTGCTAGAGATAATATTACCTCGTCTGCTTTCATTTTTGCAACCAGAGCAAATGTCTCCGAGTAATCCACCCCATATGTTT
Ca_LG_7:21182360	TTACCCTCACAAAGGGTTGATTTTGTGGCTGCAAAAGCCCTCACAAAAATCTTAAAAATTTAACTTGATTTTGTGGCTGCCTAAGCCCTCACAAAATTAA	A/T	AACGTTGTGATTTTGTGAGGGCTTAGGCAGCCACAAAATCCAGCTAAATTTTTAAGCTTTTGTGAGGGCTTTTTCAGCCACAAATAAACACCAATTTGAA
Ca_LG_7:21941032	GGATGGCCAAATGGCTGGAGCAATGAATAACATGGCTCTTCTGTTGCTGCACAGACAGCTGCAAAGACTCTGCGGGATCTAGAGAAGATGGAAAGAGAAG	G/A	TTCGTGCTGCTGAGTCAAGGGGATTGGAAGATTTTCATCGTCACAATCCTCAAAAGTTCAGGGGTGATGAGAGTCCAGAAAAAGCCGACCATTAGATTCA
Ca_LG_7:22470471	ATTGGAACTCACTAATTTTCACTTCATCATTGCCAGTTAGCAATAAATCATCAACATAAAGGCATATCAGCAGTGCAGGTGCTGCTGCTCCCTTCATGAT	T/C	ATAAACTCCATGCTCATTCGCACATTTTTCAAATTGCTGCTCTATGAGGAATCTGTCAATCCTCTTGTTCCAAGCTCTAGGAGCTTGCTTCAGGCCGTAT

Positions	-100 bp flanking sequences	SNPs within *desi* cultivar	+100 bp flanking sequences
Ca_LG_7:22470490	CACTTCATCATTGCCAGTTAGCAATAAATCATCAACATAAAGGCATATCAGCAGTGCAGGTGCTGCTGCTCCCTTCATGATATAAACTCCATGCTCATTC	C/T	GCACATTTTTCAAATTGCTGCTCTATGAGGAATCTGTCAATCCTCTTGTTCCAAGCTCTAGGAGCTTGCTTCAGGCCGTATAAGGTTTTCTTCAATTTGT
Ca_LG_7:22785096	ACCAGATGATTTTAACCGAATATTTAAAATAAATTTGGATCTGATTTTGAATTTGGGCCTTATAACCGGATTTTTTGCACAACCCTAGCTAGCAGTGTCA	A/G	GGCTTTGCTCGCAATTTGAGTGGAACAAAAGACCTGAATTAAAGTTCAGAGTTCTCTTGTGCTGCTATCTAAAGAATGCAGAGTAAGTAAGACCCTAACG
Ca_LG_7:22785097	CCAGATGATTTTAACCGAATATTTAAAATAAATTTGGATCTGATTTTGAATTTGGGCCTTATAACCGGATTTTTTGCACAACCCTAGCTAGCAGTGTCAG	G/A	GCTTTGCTCGCAATTTGAGTGGAACAAAAGACCTGAATTAAAGTTCAGAGTTCTCTTGTGCTGCTATCTAAAGAATGCAGAGTAAGTAAGACCCTAACGA
Ca_LG_7:22785109	AACCGAATATTTAAAATAAATTTGGATCTGATTTTGAATTTGGGCCTTATAACCGGATTTTTTGCACAACCCTAGCTAGCAGTGTCAGGCTTTGCTCGCA	A/G	ATTTGAGTGGAACAAAAGACCTGAATTAAAGTTCAGAGTTCTCTTGTGCTGCTATCTAAAGAATGCAGAGTAAGTAAGACCCTAACGAGATAAAGATTAA
Ca_LG_7:22785113	GAATATTTAAAATAAATTTGGATCTGATTTTGAATTTGGGCCTTATAACCGGATTTTTTGCACAACCCTAGCTAGCAGTGTCAGGCTTTGCTCGCAATTT	T/C	GAGTGGAACAAAAGACCTGAATTAAAGTTCAGAGTTCTCTTGTGCTGCTATCTAAAGAATGCAGAGTAAGTAAGACCCTAACGAGATAAAGATTAAAAAA
Ca_LG_7:22785116	TATTTAAAATAAATTTGGATCTGATTTTGAATTTGGGCCTTATAACCGGATTTTTTGCACAACCCTAGCTAGCAGTGTCAGGCTTTGCTCGCAATTTGAG	G/C	TGGAACAAAAGACCTGAATTAAAGTTCAGAGTTCTCTTGTGCTGCTATCTAAAGAATGCAGAGTAAGTAAGACCCTAACGAGATAAAGATTAAAAAACCT
Ca_LG_7:22785123	AATAAATTTGGATCTGATTTTGAATTTGGGCCTTATAACCGGATTTTTTGCACAACCCTAGCTAGCAGTGTCAGGCTTTGCTCGCAATTTGAGTGGAACA	A/T	AAAGACCTGAATTAAAGTTCAGAGTTCTCTTGTGCTGCTATCTAAAGAATGCAGAGTAAGTAAGACCCTAACGAGATAAAGATTAAAAAACCTACTTCTA
Ca_LG_7:22785143	TGAATTTGGGCCTTATAACCGGATTTTTTGCACAACCCTAGCTAGCAGTGTCAGGCTTTGCTCGCAATTTGAGTGGAACAAAAGACCTGAATTAAAGTTC	C/A	AGAGTTCTCTTGTGCTGCTATCTAAAGAATGCAGAGTAAGTAAGACCCTAACGAGATAAAGATTAAAAAACCTACTTCTAAACAAGGGCGAGTGAAGTAA
Ca_LG_7:22851018	GTTTATTTTGTGGTTGAAAAAGCCCTCACAAAAATCTTAAATATTTAACTTGATTTTGTAGCTGCTTAAGCCCTCACAAAATTACAACGTTGTGATTTTG	G/C	TGAGGGCTTAGGCAGTCACAAAATCCAGCTAAATTTTTAAACTTTTGTGAGGGCTTTTTCAGCCACAAATAAAGACTAATTTGAATTTTTGTATTTGTGA
Ca_LG_7:22884294	CTTACAACCAAATGAAGGTTTCAACATCATAATTTCAGTGGCGAATATGACCCTTGGATGCAGCAATATGTAAAGGAGTTTCGAAAAATTGTATCTTACC	C/T	GATACGCTCCAAAATAAATGGATCATCCTGAATTACTTTGTAGAGTTGATCTATGCTACCTACTTCAGCTGCTTCATTCAGTTGTTGGTCAACTTTATTT
Ca_LG_7:24035480	TCTGATTTTGCTGCCATATTCATATAGATATCATATTAAGGATCACAAGAAGTATAAACAGATTTATCAGAGGATGCTACGCCTTACCGATGCAAGGAGG	G/A	TAAATTTTTAAAATGACAATGCAGCTTGTAACTGGAAATGGCAATGCAGCTTGTAACTGGAAATGGCAATGCACTATGTAATGTACGTCCACTAATTAAT
Ca_LG_7:24035496	TATTCATATAGATATCATATTAAGGATCACAAGAAGTATAAACAGATTTATCAGAGGATGCTACGCCTTACCGATGCAAGGAGGTAAATTTTTAAAATGA	A/G	CAATGCAGCTTGTAACTGGAAATGGCAATGCAGCTTGTAACTGGAAATGGCAATGCACTATGTAATGTACGTCCACTAATTAATCCAAATTTATGCTAAT

Positions	-100 bp flanking sequences	SNPs within *desi* cultivar	+100 bp flanking sequences
Ca_LG_7:24035537	ACAGATTTATCAGAGGATGCTACGCCTTACCGATGCAAGGAGGTAAATTTTTAAAATGACAATGCAGCTTGTAACTGGAAATGGCAATGCAGCTTGTAAC	C/A	TGGAAATGGCAATGCACTATGTAATGTACGTCCACTAATTAATCCAAATTTATGCTAATAATAGTATACTCAAATTCAAATTCACATCTTCTATATGCTT
Ca_LG_7:24342399	GAAGTGTGAAGTCGGTTAAATGACAAGATTTTAAATTTTCAGTATGTGTTTGCTGCTGCTGGAAGTGAGACTGCAGCAATTATCATTAATTGGGCAATGA	A/G	CAGAGATGATAAAAGATCCAAGAGTTCTGAAGATAGCACAAGCTGAAGTGAGAGATAAAATTAACAGGAGAGGAATAATTGATGAAACCACACTTGGTGA
Ca_LG_7:24342401	AGTGTGAAGTCGGTTAAATGACAAGATTTTAAATTTTCAGTATGTGTTTGCTGCTGCTGGAAGTGAGACTGCAGCAATTATCATTAATTGGGCAATGACA	A/T	GAGATGATAAAAGATCCAAGAGTTCTGAAGATAGCACAAGCTGAAGTGAGAGATAAAATTAACAGGAGAGGAATAATTGATGAAACCACACTTGGTGAGT
Ca_LG_7:24342414	TTAAATGACAAGATTTTAAATTTTCAGTATGTGTTTGCTGCTGCTGGAAGTGAGACTGCAGCAATTATCATTAATTGGGCAATGACAGAGATGATAAAAG	G/A	ATCCAAGAGTTCTGAAGATAGCACAAGCTGAAGTGAGAGATAAAATTAACAGGAGAGGAATAATTGATGAAACCACACTTGGTGAGTTAAAATATTTGAA
Ca_LG_7:24666413	CGTTACCTTTTGTGGCTGCAAAGGCCGCCACAAAAGCCTGAGCCGTTACCTTTTGTGGCTGCAAAGGCCGCCACAAAATCCCAAAGTGACGTTGCCATTT	T/A	GTGGCCGCCCAGGCCGCCACAAATGCTTATAGTCGTTACCTTTTGTGTCTGCAAAGGCCGCCACAAACTCCAAAAGGACCGTTGCCATTTGTGGCCGCCC
Ca_LG_7:24666429	TGCAAAGGCCGCCACAAAAGCCTGAGCCGTTACCTTTTGTGGCTGCAAAGGCCGCCACAAAATCCCAAAGTGACGTTGCCATTTGTGGCCGCCCAGGCCG	G/T	CCACAAATGCTTATAGTCGTTACCTTTTGTGTCTGCAAAGGCCGCCACAAACTCCAAAAGGACCGTTGCCATTTGTGGCCGCCCAAGCCGCCACAAACAC
Ca_LG_7:24666439	GCCACAAAAGCCTGAGCCGTTACCTTTTGTGGCTGCAAAGGCCGCCACAAAATCCCAAAGTGACGTTGCCATTTGTGGCCGCCCAGGCCGCCACAAATGC	C/G	TTATAGTCGTTACCTTTTGTGTCTGCAAAGGCCGCCACAAACTCCAAAAGGACCGTTGCCATTTGTGGCCGCCCAAGCCGCCACAAACACATGCCTTTCT
Ca_LG_7:24666446	AAGCCTGAGCCGTTACCTTTTGTGGCTGCAAAGGCCGCCACAAAATCCCAAAGTGACGTTGCCATTTGTGGCCGCCCAGGCCGCCACAAATGCTTATAGT	T/C	CGTTACCTTTTGTGTCTGCAAAGGCCGCCACAAACTCCAAAAGGACCGTTGCCATTTGTGGCCGCCCAAGCCGCCACAAACACATGCCTTTCTAACCGTT
Ca_LG_7:24824943	TTTCGAGAAATTCATCATACTCATCTCCAAATATAGCTTTATTCATGGGTATGATGTGCGAATCGTCGTTGCTGCCCTTGTTCCGTTTTACTTGGATGTC	C/T	AAGACACGCCCCGTATTTAGCAAGGCATGGCTGACTTATTTTAGGAGCAGCAGCAGCAGCGACCGATTTTCCCCGATCCAGTGTTCTTGCAGCAACTTTG
Ca_LG_7:24824952	ATTCATCATACTCATCTCCAAATATAGCTTTATTCATGGGTATGATGTGCGAATCGTCGTTGCTGCCCTTGTTCCGTTTTACTTGGATGTCAAGACACGC	C/T	CCCGTATTTAGCAAGGCATGGCTGACTTATTTTAGGAGCAGCAGCAGCAGCGACCGATTTTCCCCGATCCAGTGTTCTTGCAGCAACTTTGGCAGCTTTA
Ca_LG_7:24824953	TTCATCATACTCATCTCCAAATATAGCTTTATTCATGGGTATGATGTGCGAATCGTCGTTGCTGCCCTTGTTCCGTTTTACTTGGATGTCAAGACACGCC	C/T	CCGTATTTAGCAAGGCATGGCTGACTTATTTTAGGAGCAGCAGCAGCAGCGACCGATTTTCCCCGATCCAGTGTTCTTGCAGCAACTTTGGCAGCTTTAT
Ca_LG_7:24825018	CCTTGTTCCGTTTTACTTGGATGTCAAGACACGCCCCGTATTTAGCAAGGCATGGCTGACTTATTTTAGGAGCAGCAGCAGCAGCGACCGATTTTCCCCG	G/A	ATCCAGTGTTCTTGCAGCAACTTTGGCAGCTTTATTACCCTCCAGCCTTTGTAGTTTGCCAGCTCTATTACCCTCCAATTTTTGAGGTTTGCTGGCTTTT

Positions	-100 bp flanking sequences	SNPs within *desi* cultivar	+100 bp flanking sequences
Ca_LG_7:24825034	TTGGATGTCAAGACACGCCCCGTATTTAGCAAGGCATGGCTGACTTATTTTAGGAGCAGCAGCAGCAGCGACCGATTTTCCCCGATCCAGTGTTCTTGCA	A/T	GCAACTTTGGCAGCTTTATTACCCTCCAGCCTTTGTAGTTTGCCAGCTCTATTACCCTCCAATTTTTGAGGTTTGCTGGCTTTTTTTGGCTGTACGGGTG
Ca_LG_7:24911538	GAGAATCACTTTCATTACTAACATGCTTTGCCCCAACATCATCACCAACATTACCAAAAAGAAGAACAATAATTCCAACAAGTGCAGCAACAAAAGCAGA	A/C	TCCAAAAGAAGTTCTAATTTGTCCAGGAACAACTGAAGCAACAGTGAAACCCAAACACAAACCAGCAGCTCTAATCCAACCATACCAAACACATAAAACA
Ca_LG_7:25526821	TTTGATTTCACGGTAATTTGTATGAATCGTATGTTGCTTTATCGAAACTAATTACTTACTTCAGTGAGTTATCCTCCAACAATTCCCTTTAGAAATCAAT	T/A	TGCATCTAATGGCTTAGCTACTAACTTATTTTCTGGAAGTTTGTATAACAACAATTCTTATATAGCATAATCCGTTTGTTGACAGAAACAAAGCAAGGCT
Ca_LG_7:25727402	ACTTTTCTCGGTATGCCTTTTAGGTGATCCTTCCGAGCTGCCCACTTCATCGTCCCTGACATTTTCACCGCCGACGATCACGCACTAAATAGCAGCTGCC	C/T	TTCTTTTCATTTTTTTGAGAATTGGAACACGTCTGTCCAGATTTCCAATCAAAACTTTTATGGATTTTGGTCTTTTAACGTCCAAGCCCAACCAAAGCGC
Ca_LG_7:26397306	TTCCGCGTGACGTTCCTCGGGTGCCGAACAATATAGATTGGGAGTGGCAGTCTACTATGAGATCATTTGTAGCTGCCTTTCAGAGCCTATATCCTGATGC	C/T	GACTTTTCATAGAGAGATCACTGCAGACTACTATGCATGGTACTTAAATGTATCACATCCTCTGATCCTCCCTCTACCTACTGCTGCACCTTCGAGTCCA
Ca_LG_7:27314623	AATTGTATTTAACTTGTTACTATCTCGTCAATTAATAATAATGAATTATTCGTCTGTTTTCGTTCTTTTTGTGGAAGGAAGCAGCCATTTGTTATATAAA	A/T	GGAACAAACTCTACCTTCTTTGTGCGGAGATACTGTTAATGTTAATAATTAAATTAAATAAATAGATGTAATTATAATTCGTGCAGCTACCATTGATAAC
Ca_LG_7:27314624	ATTGTATTTAACTTGTTACTATCTCGTCAATTAATAATAATGAATTATTCGTCTGTTTTCGTTCTTTTTGTGGAAGGAAGCAGCCATTTGTTATATAAAG	G/T	GAACAAACTCTACCTTCTTTGTGCGGAGATACTGTTAATGTTAATAATTAAATTAAATAAATAGATGTAATTATAATTCGTGCAGCTACCATTGATAACA
Ca_LG_7:27314670	ATTCGTCTGTTTTCGTTCTTTTTGTGGAAGGAAGCAGCCATTTGTTATATAAAGGAACAAACTCTACCTTCTTTGTGCGGAGATACTGTTAATGTTAATA	A/G	ATTAAATTAAATAAATAGATGTAATTATAATTCGTGCAGCTACCATTGATAACAAAGTTGTAGGATTCCGTGAAGATTCATAAAACTTTGTTCTTTACGC
Ca_LG_7:27327551	AATATACCTGTGTTCGCAAGTCATCAGGGCCAAGTTTTGCCCTCAGAATTTGCAAAGTTGTTTGTTCATGCTGCACACTCAATGGATATGCTTCCATCAA	A/G	CGAGAGTGCTATTGCTATTGCGTGGTAACTTGCAGCTGTCTGCAACGAATCAGCATCCAAGAAAGATGCAAATGTTATAACACAAATGAGAAGCTTTATT
Ca_LG_7:27327573	ATCAGGGCCAAGTTTTGCCCTCAGAATTTGCAAAGTTGTTTGTTCATGCTGCACACTCAATGGATATGCTTCCATCAACGAGAGTGCTATTGCTATTGCG	G/A	TGGTAACTTGCAGCTGTCTGCAACGAATCAGCATCCAAGAAAGATGCAAATGTTATAACACAAATGAGAAGCTTTATTGCATAAGTAATACAAGCCTATT
Ca_LG_7:27671648	TTGAAGATCCCTTGAGCGTACAAGATATTGAATCTCCAAGAGATTCAACTGGCCATAGGACACATACAACTTCAACTAGGAGGGATACCGATTAAGAAGA	A/G	CAAGCATGGTAGGCCTTGCACGAGGTACAACAAGAGGTGGAGCTCCATCTGCACGTATTGTTGTCTACAAATGACAGTATGTATTTTAGAGGTGCAATTT
Ca_LG_7:27671671	GATATTGAATCTCCAAGAGATTCAACTGGCCATAGGACACATACAACTTCAACTAGGAGGGATACCGATTAAGAAGACAAGCATGGTAGGCCTTGCACGA	A/G	GGTACAACAAGAGGTGGAGCTCCATCTGCACGTATTGTTGTCTACAAATGACAGTATGTATTTTAGAGGTGCAATTTCCATCGGATCATTCCATGCTATG

Positions	-100 bp flanking sequences	SNPs within *desi* cultivar	+100 bp flanking sequences
Ca_LG_7:27671678	AATCTCCAAGAGATTCAACTGGCCATAGGACACATACAACTTC AACTAGGAGGGATACCGATTAAGAAGACAAGCATGGTAGGCC TTGCACGAGGTACAA	A/G	CAAGAGGTGGAGCTCCATCTGCACGTATTGTTGTCTACAAAT GACAGTATGTATTTTAGAGGTGCAATTTCCATCGGATCATTC CATGCTATGCAACATG
Ca_LG_7:27671709	ACATACAACTTCAACTAGGAGGGATACCGATTAAGAAGACAA GCATGGTAGGCCTTGCACGAGGTACAACAAGAGGTGGAGCT CCATCTGCACGTATTGT	T/C	TGTCTACAAATGACAGTATGTATTTTAGAGGTGCAATTTCCAT CGGATCATTCCATGCTATGCAACATGGAGTGTTGACAGTATT GGCAGCTGGAAATCG
Ca_LG_7:27671713	ACAACTTCAACTAGGAGGGATACCGATTAAGAAGACAAGCAT GGTAGGCCTTGCACGAGGTACAACAAGAGGTGGAGCTCCAT CTGCACGTATTGTTGTC	C/G	TACAAATGACAGTATGTATTTTAGAGGTGCAATTTCCATCGG ATCATTCCATGCTATGCAACATGGAGTGTTGACAGTATTGGC AGCTGGAAATCGTGTC
Ca_LG_7:27671792	CCATCTGCACGTATTGTTGTCTACAAATGACAGTATGTATTTTA GAGGTGCAATTTCCATCGGATCATTCCATGCTATGCAACATG GAGTGTTGACAGTA	A/G	TTGGCAGCTGGAAATCGTGTCCTCGTCCTTCAACCTTAGGC AATTGTTGTGGCGGCTTGCACATTAGATAGAATGTTTGTGAC CAAGGTCAAATTAGGAG
Ca_LG_7:27841262	TTTCCTAATATTAAGTATGTTCAAGGGATTATGACAGGTGCTA TGGAACCTTATTTGAAAAAGCTGAGGCATTATGCAAGAGATGT TCCTTTGGTGACTA	A/T	CTGAGTATGGAGCTTCTGAAGGTTGGATTGCATCAAATGTCC ATCCTGAAATTCCTCCTGAATTGGCCTCTTATGTTGTTCTTCC TCAAATTGCTTACTT
Ca_LG_7:27841267	TAATATTAAGTATGTTCAAGGGATTATGACAGGTGCTATGGAA CCTTATTTGAAAAAGCTGAGGCATTATGCAAGAGATGTTCCTT TGGTGACTACTGAG	G/C	TATGGAGCTTCTGAAGGTTGGATTGCATCAAATGTCCATCCT GAAATTCCTCCTGAATTGGCCTCTTATGTTGTTCTTCCTCAAA TTGCTTACTTTGAAT
Ca_LG_7:27841285	AGGGATTATGACAGGTGCTATGGAACCTTATTTGAAAAAGCT GAGGCATTATGCAAGAGATGTTCCTTTGGTGACTACTGAGTAT GGAGCTTCTGAAGGT	T/A	TGGATTGCATCAAATGTCCATCCTGAAATTCCTCCTGAATTG GCCTCTTATGTTGTTCTTCCTCAAATTGCTTACTTTGAATTTA TTCCTATGGCACAAC
Ca_LG_7:27841289	ATTATGACAGGTGCTATGGAACCTTATTTGAAAAAGCTGAGGC ATTATGCAAGAGATGTTCCTTTGGTGACTACTGAGTATGGAGC TTCTGAAGGTTGGA	A/G	TTGCATCAAATGTCCATCCTGAAATTCCTCCTGAATTGGCCT CTTATGTTGTTCTTCCTCAAATTGCTTACTTTGAATTTATTCCT ATGGCACAACTGGA
Ca_LG_7:28006530	TGAAATGTAAGTGCCAAGCTGCATTGAGCTGACTACTTCCCTT TGTCCCTTTAGAGCCTTCTCATAGCCCTTAAAAAGTGAGTTAC ACCATTTTCCGACC	C/T	GACCCTATTGGAACAACCAACGCTGCAGCCAAAGCCGTTAT AACAGGCGGTGCAGCAATAGAAGCAGCTACTACTGAGAAAA TCAACACAGAAACAAACG
Ca_LG_7:28344103	GTTTCTCGCTGCTGCTCGTTACTGCGTTTCTGGGTCTTTCTCG CTGCCGATCGTTACTGCGTTTCTGGGTCATTCTCGCTGCTGC TCATTACATTCTCGT	T/C	TGCTGCTCATTACATTCTCGCTGCTGCTCGTTACTGCGTTTC TGGGTCATTTTCGCTGCTTGTTGATTGGTTTCCGCTCAGCCG CCGCTGCTTGCGTGAT
Ca_LG_7:28592202	AGATCAATTAACAAAGTTTTTGGCCGATACAACAAGAATTGCT TACTTAAATGATAATAGATTAAGAATCTACTTATGTAATAAAGT GGATCCCCTAAGA	A/G	TTTTGAGCAGCGTTGTAGTATCATATTCCAAAGATAGCAAGT CTTTTCTTTTCTCATTAAGAAAAGTCTTTCTCAAAGATTCTATA GACATGAATATATA
Ca_LG_7:28592226	CGATACAACAAGAATTGCTTACTTAAATGATAATAGATTAAGA ATCTACTTATGTAATAAAGTGGATCCCCTAAGATTTTGAGCAG CGTTGTAGTATCAT	T/G	ATTCCAAAGATAGCAAGTCTTTTCTTTTCTCATTAAGAAAAGT CTTTCTCAAAGATTCTATAGACATGAATATATAAAACATATAT TTTGCATATACAAA

Positions	-100 bp flanking sequences	SNPs within *desi* cultivar	+100 bp flanking sequences
Ca_LG_7:28592229	TACAACAAGAATTGCTTACTTAAATGATAATAGATTAAGAATCTACTTATGTAATAAAGTGGATCCCCTAAGATTTTGAGCAGCGTTGTAGTATCATATT	T/C	CCAAAGATAGCAAGTCTTTTCTTTTCTCATTAAGAAAAGTCTTTCTCAAAGATTCTATAGACATGAATATATAAAACATATATTTTGCATATACAAAAGT
Ca_LG_7:28592240	TTGCTTACTTAAATGATAATAGATTAAGAATCTACTTATGTAATAAAGTGGATCCCCTAAGATTTTGAGCAGCGTTGTAGTATCATATTCCAAAGATAGC	C/T	AAGTCTTTTCTTTTCTCATTAAGAAAAGTCTTTCTCAAAGATTCTATAGACATGAATATATAAAACATATATTTTGCATATACAAAAGTAAAACTTTGAT
Ca_LG_7:28592260	AGATTAAGAATCTACTTATGTAATAAAGTGGATCCCCTAAGATTTTGAGCAGCGTTGTAGTATCATATTCCAAAGATAGCAAGTCTTTTCTTTTCTCATT	T/C	AAGAAAAGTCTTTCTCAAAGATTCTATAGACATGAATATATAAAACATATATTTTGCATATACAAAAGTAAAACTTTGATTAGCTAGAACTGTACACCCT
Ca_LG_7:28592269	ATCTACTTATGTAATAAAGTGGATCCCCTAAGATTTTGAGCAGCGTTGTAGTATCATATTCCAAAGATAGCAAGTCTTTTCTTTTCTCATTAAGAAAAGT	T/C	CTTTCTCAAAGATTCTATAGACATGAATATATAAAACATATATTTTGCATATACAAAAGTAAAACTTTGATTAGCTAGAACTGTACACCCTACAAAATGC
Ca_LG_7:28592295	CCTAAGATTTTGAGCAGCGTTGTAGTATCATATTCCAAAGATAGCAAGTCTTTTCTTTTCTCATTAAGAAAAGTCTTTCTCAAAGATTCTATAGACATGA	A/G	ATATATAAAACATATATTTTGCATATACAAAAGTAAAACTTTGATTAGCTAGAACTGTACACCCTACAAAATGCAGTAGGTATGCTTTCGTTGCATAGTC
Ca_LG_7:29777819	AATCATATTATTTTTCAAGTGGCTATGCCAGATGTTGTAGAAGCTTGAGGTTATAGTATGCCGGATTAATATGTTTATAGATTATAGTGAAGCAGCAGCG	G/A	CAATTTTAAGTTGTATCACTGTGGTGAAAGAACTAATACTAGTTAAACACTTGCTAGCTTGAGAAACACATTTTCTGCAATTAGTTGTTGATTTCATATT
Ca_LG_7:30019324	ACTTCTTGAATCCACTGATCAGCTTTTTCTGAGTTCTCATCACCCTTGAACTTTGGAGGATGGTAACGACGAAAATCTTCCAATCCCCTTGACGCAGCAT	T/A	CACATATCTCTCTTTCCCTCTTCTCCAGATCATGCAGAGTCCTTGCAGCAGTCTGTGCAGCAACAAAAGCAACCATGTTGTTCATAACTTAAGTCACTTG
Ca_LG_7:30085012	ATAGATATTAGCACCCTTTATTTGCTGCCACATTTTCAACACGCACACTCAACATGAAGGAACCTGACAATACACCTCAATGAGAAGGAACCTGGAAATA	A/G	GTATCCCTCCCCATTAATAGTCCCTTTTCTCACTCAAGCTCTCTCTATCTCTACTCTCAGCCTCTATCCTAACAGACGCTCCAACCAGCAGCAGCGAGCC
Ca_LG_7:30085070	GGAACCTGACAATACACCTCAATGAGAAGGAACCTGGAAATAGTATCCCTCCCCATTAATAGTCCCTTTTCTCACTCAAGCTCTCTCTATCTCTACTCTC	C/A	AGCCTCTATCCTAACAGACGCTCCAACCAGCAGCAGCGAGCCCCCACCCCATTGCTTGTCCACCACCACCAATGCCTTGTTTCACCCGTTGGCCCTCTCT
Ca_LG_7:30085080	AATACACCTCAATGAGAAGGAACCTGGAAATAGTATCCCTCCCCATTAATAGTCCCTTTTCTCACTCAAGCTCTCTCTATCTCTACTCTCAGCCTCTATC	C/A	CTAACAGACGCTCCAACCAGCAGCAGCGAGCCCCCACCCCATTGCTTGTCCACCACCACCAATGCCTTGTTTCACCCGTTGGCCCTCTCTCTCGGCATGA
Ca_LG_7:30604011	TGCAACTAATTTAATTGGTTTCAAGACCTGCATCTATCCCTATGGTGATTTGAGATATGTTTTCATATTATCGTATAGCTGCGTTTGTTGCTCGAGGCCG	G/A	ACTGTTCATCATCGTAAGTCTCTGAAACCTTCTTTCGACTATTTATCGTTTTGTGCGTGTATTTGTTGTCTTTTTGAATCTCACTGAACCCTTAATCCAT
Ca_LG_7:30604013	CAACTAATTTAATTGGTTTCAAGACCTGCATCTATCCCTATGGTGATTTGAGATATGTTTTCATATTATCGTATAGCTGCGTTTGTTGCTCGAGGCCGAC	C/T	TGTTCATCATCGTAAGTCTCTGAAACCTTCTTTCGACTATTTATCGTTTTGTGCGTGTATTTGTTGTCTTTTTGAATCTCACTGAACCCTTAATCCATTT

Positions	-100 bp flanking sequences	SNPs within *desi* cultivar	+100 bp flanking sequences
Ca_LG_7:30604020	TTTAATTGGTTTCAAGACCTGCATCTATCCCTATGGTGATTTGAGATATGTTTTCATATTATCGTATAGCTGCGTTTGTTGCTCGAGGCCGACTGTTCAT	T/C	CATCGTAAGTCTCTGAAACCTTCTTTCGACTATTTATCGTTTTGTGCGTGTATTTGTTGTCTTTTTGAATCTCACTGAACCCTTAATCCATTTTACTAAG
Ca_LG_7:30615886	AACCAAATGCAACTGAAGCGCATCAACGACATATGGACTAGAGCTCAGCCAACTACTGAACAAGTGCAGCCAACTGCAGAGCAAGCTGAGCCAGTTGAAT	T/G	CACATTTTGTGGTCTTGACGAAGCTGCTGGAATTCATGGAGATTCAAGCTGCTCATAACGCAAGAGTAGAAGCATCTCTCAAGACTCTCCAGGCATCACT
Ca_LG_7:30707315	TGAAAGCAAATGACATCGTAAAAAAAAAGAAAAGAATGTCCTGAGAGAAGAAAGGAAGTCAGGGAATGAGCTGGTGCTATAGCCAGAATCGCTTGTTACG	G/A	GAATAGGTTGCATATAGTATACTACCAGTGGAGTCATAAGTTGTAGATCTTCAATGGCTTAATCCGTTGTTCAAATAGCAGCTGCTTGGGCTTACCTTAT
Ca_LG_7:30739580	CCCAGATCGTACGCTAAGGTCCCTAAGCAATCACTTAGTGGAAAAGGAAGTGATCGAGCGATGACAACCAGGAGGTGGGCTTGGAAGCAGCCATCCTTTG	G/A	AAGAAAGCGTAATAGCTCACTGGTCTAGCTCCATGGCACCGAAAATGTATCAGGGCTCAAGTGATTCACCGAAGCGACGAGACCTTGAAAGTTGTTCAAG
Ca_LG_7:30762270	TTTCCATCCCGACAATTACTACTGACTTTCCATTTTTGGGATTTCACTTAAAAGACTGGACTTCAAGCAGCAATGAGAGCAAAGGCAAGGAATTGATGCG	G/A	CCAATAATCGAAGAATGGGGCAAGGCTAATTTCCAGACAATGGCAAGTGCTTGAGAAGGAGCTGTGAAAGAAGGGGCTGCTAGAGAATAGGGAGCTCTTG
Ca_LG_7:32450356	TCTAATAATTAATTAAATTAAAAATGATAGCAAATCCAAGATAAATAAATAACAAGCACATTTTAAAGTGGAAATTACCTGCTGCAGGTACTCCTCGACG	G/A	CTTGTGGCCTCGACAGGAAAAACATCTTCAAAAGTTGTCTTTATACAACAACAGATAGATAGAGGGCACAGCATTAGCAAAATACATGAGTTGAGGAAAT
Ca_LG_7:32507631	CATATCTAAGTTTGATGGACATGCTCACCAATCTTACAATTGGTAGTTGTGATGGTGTGGAGTCCTTCCCCAATAAGGGTTTTGCGTTGCTGCCTCCCTC	C/T	CCTTACCTCTCTAACGCTAAGCAGTATGTCAAGTTTGCACACGTTGGAATGCACGGAGCTTCTCCACCTCACGTCCCTCCAACACTTAGCAATTTTGACT
Ca_LG_7:32724929	CTTATAGGAAAACTATGCTGCCAAGAAGGAATGATGCTCCAAGAGTAAATGTCAATAAGGATGACCAAATGGCGGAAGCTATGAATAACATGGCTGCTTC	C/A	TGTTGCTACCCAAACTATGGCAAAGACTCGACGGGATCAAGAAAAGAGGGAAAAAGAGATCCTTGTTGCTGAGTCAAGAGGATTAGAATATTTTCATCAT
Ca_LG_7:32724934	AGGAAAACTATGCTGCCAAGAAGGAATGATGCTCCAAGAGTAAATGTCAATAAGGATGACCAAATGGCGGAAGCTATGAATAACATGGCTGCTTCTGTTG	G/T	CTACCCAAACTATGGCAAAGACTCGACGGGATCAAGAAAAGAGGGAAAAAGAGATCCTTGTTGCTGAGTCAAGAGGATTAGAATATTTTCATCATTACAA
Ca_LG_7:33857933	ATACCAAAATACCCCAATAACATTTCAAATACTATTCGCATTTTCTGCACCCTGCATCCTGCGAACATGACTGCAACCTTGATTTGCACTTCACTGGACC	C/T	GTGCCGGCGCAGCCAGGTATTGCGCTGCACCGCTCTGGTACGATCTCACCGGGAAACACTGCGATTAAACTACCTAGATTGATGTTAATCTTAGAGTAAT
Ca_LG_7:35422143	GAAATTGCTGCAAACGTGAGGATGGAAATTGCTGCAAACGTGAGGATGAAAATTGCTGCAAACGTGAGGATGGAAATTGCTGCAAACGTGAGGAGGATGA	A/G	GCTGCAAGTTGAAAAACTCCAGATTTGGACAATCTGATCTCTCACCAGAACAAATGTGTTTATTCAAATGCATAACAATGTTAAACATGAATAAACAAAG
Ca_LG_7:35890994	ATACCACACATGCTGTTACGACCAAATGTCAATGTTCCAGCAGGCGATTGCACGGGCATTTTTCCGTCAGCACCCCATTCAACAAACGGCGTTGCTTCAT	T/C	TTATGTCATATCCACTTGTCCATGTAACTGTCATCTGCACAAGACAATAACAACAATTCCTGAAATCACTTGCATAGCTCTAATAGACATAGTGGTTCCA

Positions	-100 bp flanking sequences	SNPs within *desi* cultivar	+100 bp flanking sequences
Ca_LG_7:35891017	AAATGTCAATGTTCCAGCAGGCGATTGCACGGGCATTTTTCC GTCAGCACCCCATTCAACAAACGGCGTTGCTTCATTTATGTCA TATCCACTTGTCCAT	T/G	GTAACTGTCATCTGCACAAGACAATAACAACAATTCCTGAAA TCACTTGCATAGCTCTAATAGACATAGTGGTTCCAATGACAT CAATTATTATTCCTAT
Ca_LG_7:35929680	GTTAATGGAGTAAATGTATTTGGTTATGGTAACGGAACAGCAA GTGGTGGATCTCCGAAAGCACGAGTTGCAGCCTATAAAGTAT GTTGGGATGGATGTT	T/C	ATGACGCTGATATCTTGGCTGGTTTTGAAGCTGCAATACATG ATGGTGTTGATGTGATTTCAATGTCTTTGGGTGGTGATCCTC AAGAATATTTTCAAAA
Ca_LG_7:37423848	GGCATTCACATTGCTCGATGTATAATTTTAGGTTGAGGACGAA GCAGTGCTAGTCGAAGATGTGCCAACAATGAAGTCAAAAAGA AGACTTGTCTTGACA	A/T	ACACAGCTTATGCAGCAACTACTTAACCCTCCGCCAACATCA GTTCTCTGTGCAGATGTCAAGTTGCATCATGAAAGTGTGGTT TACTCTGTTGCTAGAT
Ca_LG_7:37423851	ATTCACATTGCTCGATGTATAATTTTAGGTTGAGGACGAAGCA GTGCTAGTCGAAGATGTGCCAACAATGAAGTCAAAAAGAAGA CTTGTCTTGACAACA	A/G	CAGCTTATGCAGCAACTACTTAACCCTCCGCCAACATCAGTT CTCTGTGCAGATGTCAAGTTGCATCATGAAAGTGTGGTTTAC TCTGTTGCTAGATTAG
Ca_LG_7:37423855	ACATTGCTCGATGTATAATTTTAGGTTGAGGACGAAGCAGTGC TAGTCGAAGATGTGCCAACAATGAAGTCAAAAAGAAGACTTG TCTTGACAACACAGC	C/G	TTATGCAGCAACTACTTAACCCTCCGCCAACATCAGTTCTCT GTGCAGATGTCAAGTTGCATCATGAAAGTGTGGTTTACTCTG TTGCTAGATTAGTGTT
Ca_LG_7:37696118	GATAGTTATGTGGTTATAATGTGAGGCAGAGTTCTTATTCTTT CGCCGTCCTGCCCCAACTGGAACATTCCTCGTGGCGCCACCT GCAGTCCAGTATCTT	T/C	TGACAGGCTCTGCAGAAATAACGTGGCTGGTTGACATTGTA GTTGTTGTAGTAACAGAATTTAGTGTCCATGCTATTGCATCG AGGACATGGTAGCAATT
Ca_LG_7:37696124	TATGTGGTTATAATGTGAGGCAGAGTTCTTATTCTTTCGCCGT CCTGCCCCAACTGGAACATTCCTCGTGGCGCCACCTGCAGTC CAGTATCTTTGACAG	G/C	GCTCTGCAGAAATAACGTGGCTGGTTGACATTGTAGTTGTTG TAGTAACAGAATTTAGTGTCCATGCTATTGCATCGAGGACAT GGTAGCAATTTATCGG
Ca_LG_7:37696127	GTGGTTATAATGTGAGGCAGAGTTCTTATTCTTTCGCCGTCCT GCCCCAACTGGAACATTCCTCGTGGCGCCACCTGCAGTCCAG TATCTTTGACAGGCT	T/C	CTGCAGAAATAACGTGGCTGGTTGACATTGTAGTTGTTGTAG TAACAGAATTTAGTGTCCATGCTATTGCATCGAGGACATGGT AGCAATTTATCGGGCT
Ca_LG_7:37696128	TGGTTATAATGTGAGGCAGAGTTCTTATTCTTTCGCCGTCCTG CCCCAACTGGAACATTCCTCGTGGCGCCACCTGCAGTCCAGT ATCTTTGACAGGCTC	C/T	TGCAGAAATAACGTGGCTGGTTGACATTGTAGTTGTTGTAGT AACAGAATTTAGTGTCCATGCTATTGCATCGAGGACATGGTA GCAATTTATCGGGCTT
Ca_LG_7:37696139	TGAGGCAGAGTTCTTATTCTTTCGCCGTCCTGCCCCAACTGG AACATTCCTCGTGGCGCCACCTGCAGTCCAGTATCTTTGACA GGCTCTGCAGAAATAA	A/G	CGTGGCTGGTTGACATTGTAGTTGTTGTAGTAACAGAATTTA GTGTCCATGCTATTGCATCGAGGACATGGTAGCAATTTATCG GGCTTCTTCAGTGTTT
Ca_LG_7:37696148	GTTCTTATTCTTTCGCCGTCCTGCCCCAACTGGAACATTCCTC GTGGCGCCACCTGCAGTCCAGTATCTTTGACAGGCTCTGCAG AAATAACGTGGCTGG	G/A	TTGACATTGTAGTTGTTGTAGTAACAGAATTTAGTGTCCATG CTATTGCATCGAGGACATGGTAGCAATTTATCGGGCTTCTTC AGTGTTTTCTCTTGTG
Ca_LG_7:38157474	GTTTGTGGCGGCCTGAGCGGCCACAAATGACAACGTCACTTT GGAATTTTGTGGCGGCCTTTGCAGCCACAAAAGGTAATGACT CAGGCTTTTGTGGCGA	A/G	CCTTTGCAGCCACAAAAGGTAACGTCACTTTGGGATTTTGTG GCGGCCTTTACCCTCACAAATGGTTGTATTTTGTGGCTGAAA AAACCTCACAAAAATC

Positions	-100 bp flanking sequences	SNPs within *desi* cultivar	+100 bp flanking sequences
Ca_LG_7:38970890	ATTACATATCTATATTGAGCATCATTTATGTCAATTTATAAGCAGATAAGCTAGACCAGTTAGAATCCTTATATATCCACGTGGCTATGTACTTTTTTCC	C/G	CATGCTGCTGCTCCTTATTCAAAATCCTTCATCTTTGGAATCAAATCAAACAATATATAGTTGACTTATATGCATGCATGTCTTCCTAAACATTGATGTT
Ca_LG_7:39561953	AAAGAGAACATAAAGAAGTTTCAAGTGAAAGCTTCGATGAAAGAGAAAGTTGTAACAGGAGTTACAGCAGCTGCATTAACAGCTTCAATGATGGCTCCCA	A/G	ATGTGGCTGAAGCTGCTTCTGTTTCACCTTCTCTCAAGAATTTCTTGCTCAGCATAGGTGCTGGTGGACTTGTTGCTGTTGCCATTGTTGGTGCTGTTAT
Ca_LG_7:40072316	AATTTGCTGACACATGATGTTAATCAATTGGAAAGTGTTGACGGAAACTAGGAATGAGTTGATTTCCCATCTAGGGGCTGCAAAACTGAACCATGATTAC	C/T	GATCAATCCTTCTTGGTGCAGTGTGCATCTGTGCCATGTTGGGTGCAATCATGATAACCCACACTGCGGAGGAAATCTCGAATTTCTTTAGCGCTGCTAT
Ca_LG_7:40551975	AGTTAAATGCGAAAAGAAACAAATTTTTTATTTTTATTAATTACAAAAGGGTATTAGAACTTGATCCTTTGACCTTGGTTGATAAAATTTGCAGCGCGTA	A/T	TCACCTGAAACTTCAAAGAAAATGTGACTTCAAATTCCACGTATTTGAATGCCTAAGGTAACTTGTTTTTCGTGTCATATTACTCCTTATTCTTTTGTTG
Ca_LG_7:40808909	CAGGACATAGACATCAATGGAAGAATGTTTGTCAAGTAGTGAATGTTATTAATCCCCTACAACAATTCATGGCAGCCGACTCCAAAGACGCTATAGCAGG	G/A	ATAGCGGATAACAGGAAGGCTGCTATATGAATTTGTTTTGCAGTTTAAAAATGATGAGAATTACTCAAAATTCACAAGATTCATAATTAACAATAAAAAG
Ca_LG_7:40846059	AACAAGTTCTGCATCAGCTCCTTCAAGTGTATTCCCAGCTTTAGCTAATAGAAGAGTAATATTTCCACTATAAGGCCTGCTACCATGATCTGCTGCGTCG	G/C	GAAACATGTTCAGCCTTCTGTGACTGATCAGTTTCCGACCTAGTTGCTGCTCCATCTTCAGTTTCATTTACACTAGTACAAATCAAATCAAAACAATGTT
Ca_LG_7:42143183	ACTCTGTTACAGCTTGGTTCCGTACCTTCTTCAGATCTTACTCAGTCAAGCAGCAGCAGGAGGCAGAGCTGCAAAACAACCGAAGATGATCCATCAAGTA	A/G	CTTCAAGAGACATTACTTCTTTCAAGCACAAGTCAGACCTCCAATATACGCCTCACAACTCAACATCCCCTGCTTCCCACCAAAAAGCAGTAAACGGCAT
Ca_LG_7:42169378	AGTGAGGTTGATGTTATTAATTTCAATTTGAACGGAAAATCAAAGATGCAGCGATAATTGTAGTGGAGAAATTAAGGGATTGTTTCTCTCTCTCTCTCTC	C/A	TCTATATGTGTGTGTGTGTAAGGCAGGCAGGCAGGCAGGCAGCCATGTTTTGTCCTCACCACTATCTGACACACTGTTCCCCTCTTTCTCTCTTTCTCTC
Ca_LG_7:42169380	TGAGGTTGATGTTATTAATTTCAATTTGAACGGAAAATCAAAGATGCAGCGATAATTGTAGTGGAGAAATTAAGGGATTGTTTCTCTCTCTCTCTCTCTC	C/A	TATATGTGTGTGTGTGTAAGGCAGGCAGGCAGGCAGGCAGCCATGTTTTGTCCTCACCACTATCTGACACACTGTTCCCCTCTTTCTCTCTTTCTCTCTC
Ca_LG_7:42169384	GTTGATGTTATTAATTTCAATTTGAACGGAAAATCAAAGATGCAGCGATAATTGTAGTGGAGAAATTAAGGGATTGTTTCTCTCTCTCTCTCTCTCTATA	A/G	TGTGTGTGTGTGTAAGGCAGGCAGGCAGGCAGGCAGCCATGTTTTGTCCTCACCACTATCTGACACACTGTTCCCCTCTTTCTCTCTTTCTCTCTCACTC
Ca_LG_7:42170111	GATTCATCATCATAATCAACTACACAATGTTATAACATCTTCCACAGTGGCAGTGGCAGTGGCAGTAGCAGCACCTCATAACAACCCTTGTTCAAATGGG	G/C	ACAACAGTTGGAGAGGTAGATGAGTCAAGCTACAAGAAAGTGGATGTGGTAGTTGTAAGGTACAGAGAATGCCTAAAGAACCATGCTGCTGCAATGGGAG
Ca_LG_7:42192196	TACAACAGCTCGACGATGCTGCTGGTGATGTTTCTGGACTTGCTGTCAAATGGTACTACATCTTCTATCTCTCTCATTAATATTTTTTAATCTATATCAT	T/A	TAACTTTTTTAATCAAATTTTGCAGCCTTGCTCCATTAGTTAGGAAGATGAGTGAGTCAAGGGTTGTGGAAATGAGCAGTCAACTCTGTGACAAAATACT

Positions	-100 bp flanking sequences	SNPs within *desi* cultivar	+100 bp flanking sequences
Ca_LG_7:42347522	TCTTGTGCCTCGAGTTTTCGATTATATATATATATACGGAAATATATTAGCTGATGTAATTAGTAGTTGTTGTTCTTGTTTCGTTACGTACCCTTAGTAG	G/A	TTCCTATACCGGTTATGTTCCTTGGATTATTTACAAGCGGTATTCAAGCTCTTTATTTTTGCTTACTTTAGCTGCAGCTTATATAGGTGAATCCATGGGA
Ca_LG_7:42411688	TAATAGTAGTGCAGATAACAGATGTAGGTATTCCGGCACCTAATGCAGCCTGTCTAGCAGGAGCCTGCCCTAAATTTGCACTAAGAACATTTCCAAAAAA	A/G	CACCTCTTGCACAAGTGATGGATCAACATTGGCTCTCTTGAGAGCAGCTGCAGATTTATATATCAACTTAAAACACAAGAGAAGCAGAAATTTCAAAAGT
Ca_LG_7:42845017	AAACACAAATAATAGAAAATATTATAATTTTAAGATGCAGCAGCTATAAAGATTAATAAACTTTGAGACATAAAATATGTATCTCTCAAATTCAAATTGA	A/G	TGTCTCGCAAAGCTGCTATAAAGAATGTAAATACTATTTTTTCGGGGCAAGTTTACAACTATTAAGCATTGTGTATTATTAAAATATTGAATATCACTAT
Ca_LG_7:42879661	GGTTATCTATTTGGCCTTCCAATTGGCATTGTTGCTGACTCTGTTGGTGCAACCATTGGTGCTGTGGCTGCATTTCTTCTTGGTAGAACAGTAAGTTTTA	A/G	CATTAATTTATATTTGTGAAAGTAAAAAAGTGAAAACTAGTCCACTAGTTTCTGTTGGATATACATTAATTGAGGTTTCTCTTAATTAATACCAAATAAT
Ca_LG_7:42883120	ACATTTGAAGTCTCTCATGGGAAAAGTGCAACATTTTGGTGTTCCATCTCTTGGCGGTTGCTTCGATAGCTGCCGCGACCATACACAAGGCTCGGGATTC	C/T	GGGACGAGGATATGGAACCTCAGTGATAAGCCAGTGGAGCTGCAAATAAGGGTAGGATCAATACTAAAGAAGATTTATACATTGAAGCCAGGGTCTTCAA
Ca_LG_7:43536774	AGTAGATACAATAGACTTGGTTGATCTGAAAGATGTAAAGTTTGTCCACTTATAAGATGTTTGTTCATTTAAAAAACTCAAATTTAAATTATGTTATTGG	G/C	TGTTGGGTTAGCCGTTGGACTTGCTTCTATTGGACCTTTAATTGGTCAAAACACAACTGCAGGATAAGTTGTAGATTACCATTCATAAAATTTAATATTT
Ca_LG_7:43536777	AGATACAATAGACTTGGTTGATCTGAAAGATGTAAAGTTTGTCCACTTATAAGATGTTTGTTCATTTAAAAAACTCAAATTTAAATTATGTTATTGGTGT	T/C	TGGGTTAGCCGTTGGACTTGCTTCTATTGGACCTTTAATTGGTCAAAACACAACTGCAGGATAAGTTGTAGATTACCATTCATAAAATTTAATATTTACT
Ca_LG_7:43536784	ATAGACTTGGTTGATCTGAAAGATGTAAAGTTTGTCCACTTATAAGATGTTTGTTCATTTAAAAAACTCAAATTTAAATTATGTTATTGGTGTTGGGTTA	A/G	GCCGTTGGACTTGCTTCTATTGGACCTTTAATTGGTCAAAACACAACTGCAGGATAAGTTGTAGATTACCATTCATAAAATTTAATATTTACTTTATAAA
Ca_LG_7:43536812	AGTTTGTCCACTTATAAGATGTTTGTTCATTTAAAAAACTCAAATTTAAATTATGTTATTGGTGTTGGGTTAGCCGTTGGACTTGCTTCTATTGGACCTT	T/G	TAATTGGTCAAAACACAACTGCAGGATAAGTTGTAGATTACCATTCATAAAATTTAATATTTACTTTATAAAATTTATCGTCATTAAAATATATATAAAA
Ca_LG_7:43536813	GTTTGTCCACTTATAAGATGTTTGTTCATTTAAAAAACTCAAATTTAAATTATGTTATTGGTGTTGGGTTAGCCGTTGGACTTGCTTCTATTGGACCTTT	T/G	AATTGGTCAAAACACAACTGCAGGATAAGTTGTAGATTACCATTCATAAAATTTAATATTTACTTTATAAAATTTATCGTCATTAAAATATATATAAAAG
Ca_LG_7:43540170	CTTTTTTGTGTAATCTTATTTTTTTTAAAAGTTGTATAGCTGCTTTTATGGTGCATTCATGTCCGTATTTGATTATATAACCTATTTTCAAGTATTGTTT	T/C	TTTTGACATTGTTGCTAAATCTGGTGCTTTTCTAAAATTTTACACTATCTCAGGTTTTGGTCACACATTGTAGCTGCTTATGCATTTACCTTCTGGACAT
Ca_LG_7:43540275	ACATTGTTGCTAAATCTGGTGCTTTTCTAAAATTTTACACTATCTCAGGTTTTGGTCACACATTGTAGCTGCTTATGCATTTACCTTCTGGACATGCTAT	T/A	ATTTTGATGAGAGAGTATGAAAAAGTTGCCTCAATGAGATTACAATTTGTTGCAGCAGAAAAGCGTCGTCCTGATCAATTTTCGGTAATATTTTGTTCTC

Positions	-100 bp flanking sequences	SNPs within *desi* cultivar	+100 bp flanking sequences
Ca_LG_7:43541296	TACATTATTAACTAGATATATTACAATAGATATAATTTTGCCCATTTATTTAGGAAGCGAAAAAACAGTATGTGACAGCATATTTAATGTGACACTTACG	G/A	CAGGTTGTTTATAATGCAAACAAATTAGCAAAGTTGGTTAAAAAGAAGAGCAAGATGCAGAATTGGCTCGTCTATTATCAAAATAAATTTGCCAGAACTT
Ca_LG_7:43541337	CCATTTATTTAGGAAGCGAAAAAACAGTATGTGACAGCATATTTAATGTGACACTTACGCAGGTTGTTTATAATGCAAACAAATTAGCAAAGTTGGTTAA	A/C	AAAGAAGAGCAAGATGCAGAATTGGCTCGTCTATTATCAAAATAAATTTGCCAGAACTTCAAAAAAACCTGAAGTGAAGGTATTAGTTTTAAACTGATGT
Ca_LG_7:43615860	TGTAACTGTATGGTAATAATATTGATGGAGAGGCAGGGAGATAGTCAAAAACTGTAAGCAACTAAGAATAGACTCACATTTGTACCACGAAGCATATGAG	G/C	CAGAAGTAGAGACACCAGCTGCAACAGACAGGACCAATGGCAAGCCAGAAGAGTAAAGTAGCCAAAATAAATATGTGGAGAAGGCAAGAGAGCAAGACAA
Ca_LG_7:43615877	AATATTGATGGAGAGGCAGGGAGATAGTCAAAAACTGTAAGCAACTAAGAATAGACTCACATTTGTACCACGAAGCATATGAGCAGAAGTAGAGACACCA	A/G	GCTGCAACAGACAGGACCAATGGCAAGCCAGAAGAGTAAAGTAGCCAAAATAAATATGTGGAGAAGGCAAGAGAGCAAGACAAGAACAGGTATGGTACCA
Ca_LG_7:43615878	ATATTGATGGAGAGGCAGGGAGATAGTCAAAAACTGTAAGCAACTAAGAATAGACTCACATTTGTACCACGAAGCATATGAGCAGAAGTAGAGACACCAG	G/A	CTGCAACAGACAGGACCAATGGCAAGCCAGAAGAGTAAAGTAGCCAAAATAAATATGTGGAGAAGGCAAGAGAGCAAGACAAGAACAGGTATGGTACCAT
Ca_LG_7:43615906	AAAAACTGTAAGCAACTAAGAATAGACTCACATTTGTACCACGAAGCATATGAGCAGAAGTAGAGACACCAGCTGCAACAGACAGGACCAATGGCAAGCC	C/T	AGAAGAGTAAAGTAGCCAAAATAAATATGTGGAGAAGGCAAGAGAGCAAGACAAGAACAGGTATGGTACCATACCAGCTGCTGTATTGAGTAGGTTGGAG
Ca_LG_7:43615910	ACTGTAAGCAACTAAGAATAGACTCACATTTGTACCACGAAGCATATGAGCAGAAGTAGAGACACCAGCTGCAACAGACAGGACCAATGGCAAGCCAGAA	A/T	GAGTAAAGTAGCCAAAATAAATATGTGGAGAAGGCAAGAGAGCAAGACAAGAACAGGTATGGTACCATACCAGCTGCTGTATTGAGTAGGTTGGAGGATC
Ca_LG_7:43617496	AAAGAAGTTACTATAGAGAGGTTTTAACCACATGATCTCCTTCCCCTTGAGATTCATTGGAACAAAGGGATGAAGTCATAATGACTGAGAAATTTAAACT	T/A	GACCGGTACTGGATTCCAATGGCAGCATTAGAGTATTATGTTCTTATCAAACAAATATCAAGTAGTCAGGGAAATGAAAACAAACCTTGTTGCCAATTGC
Ca_LG_7:43617812	AAAGATAGAAATCCAGAGTACATTATAGGGAGAAAGAACTACAAAGCCTTTTCATAAAGAAAAAAATAGAAGTGGGAAGTCTATCACACTAATTGGTTTG	G/A	CACTCACTCACCAAAGAGATATAAGCAAGCCAGCAAGCTGCCTAGAACCCATACGCTCAAAAGCTTTTCCTTCTTCCAAGGCCTTCCCTATTGTATCCAG
Ca_LG_7:43617821	AATCCAGAGTACATTATAGGGAGAAAGAACTACAAAGCCTTTTCATAAAGAAAAAAATAGAAGTGGGAAGTCTATCACACTAATTGGTTTGCACTCACTC	C/T	ACCAAAGAGATATAAGCAAGCCAGCAAGCTGCCTAGAACCCATACGCTCAAAAGCTTTTCCTTCTTCCAAGGCCTTCCCTATTGTATCCAGCCACCACTG
Ca_LG_7:43617843	GAAAGAACTACAAAGCCTTTTCATAAAGAAAAAAATAGAAGTGGGAAGTCTATCACACTAATTGGTTTGCACTCACTCACCAAAGAGATATAAGCAAGCC	C/G	AGCAAGCTGCCTAGAACCCATACGCTCAAAAGCTTTTCCTTCTTCCAAGGCCTTCCCTATTGTATCCAGCCACCACTGCCCCATTGCACTTCCTTCTAGA
Ca_LG_7:43643955	TTGCTTCTTATCTTCTTCCCCATGTACCCCAGCAGGTTCTCGCAGCACTTAGTGTTGAAATTCTCAAAGCTGCTGGTGAACGCAGTGGTGGGTCTCTTGA	A/T	TGGTGTAGCATTTCTCCTCGAATCTGACTTCTTAGGTGATCCTGCAGCTACTTATGCTGTGGCAGACATTATTGCTAAATTGGAGGATGAGGCTGTTGCT

Positions	-100 bp flanking sequences	SNPs within *desi* cultivar	+100 bp flanking sequences
Ca_LG_7:44642083	GCCTAGAGTGTGCACCTTTACTGTCGCTGCCATATTTACACAATCCATAAGAATGATTTAGAGGTGGATGTACAACGTAGACTAGTAGCACCAACTACCG	G/T	GCAATAAAAGTCCATACTTAGTTAAATCATCCCAAATTCACTGATCAAAGTGTAAAACTTCACTGCAGCCAATAGCAAGAGGCTATACTATTCATTTGAA
Ca_LG_7:44646522	ATAAAGCTAGCCAACTTAGTGGTTACTAAACGGGGGTAGATTAAACTCGTTGATTTTGGTGCAGCAACAAACCTGAAGATCGAAAAGAATTATATTCCCG	G/A	ACCGCACCCTTTTGGATCCCAACTGTTGCCCTCTAGAACTATATGTACTCCCGGAAGAAACACCAAGTCCTCCACCGGAGCCTATTGCTGCTATCTTTTC
Ca_LG_7:44646543	GTTACTAAACGGGGGTAGATTAAACTCGTTGATTTTGGTGCAGCAACAAACCTGAAGATCGAAAAGAATTATATTCCCGACCGCACCCTTTTGGATCCCA	A/G	ACTGTTGCCCTCTAGAACTATATGTACTCCCGGAAGAAACACCAAGTCCTCCACCGGAGCCTATTGCTGCTATCTTTTCACCAATCCTATGGCAGGTTTA
Ca_LG_7:44646547	CTAAACGGGGGTAGATTAAACTCGTTGATTTTGGTGCAGCAACAAACCTGAAGATCGAAAAGAATTATATTCCCGACCGCACCCTTTTGGATCCCAACTG	G/A	TTGCCCTCTAGAACTATATGTACTCCCGGAAGAAACACCAAGTCCTCCACCGGAGCCTATTGCTGCTATCTTTTCACCAATCCTATGGCAGGTTTAATTA
Ca_LG_7:44646556	GGTAGATTAAACTCGTTGATTTTGGTGCAGCAACAAACCTGAAGATCGAAAAGAATTATATTCCCGACCGCACCCTTTTGGATCCCAACTGTTGCCCTCT	T/C	AGAACTATATGTACTCCCGGAAGAAACACCAAGTCCTCCACCGGAGCCTATTGCTGCTATCTTTTCACCAATCCTATGGCAGGTTTAATTACTTATATCT
Ca_LG_7:44652201	CCATACAAACAAAAAACAGGAAAATAAACTAGGGGGCAACAGCCCCAGGAAAATCAGCACATGGAAGCTCTACTAATAATGGTGATGCCAAAGTCTAGCG	G/A	ACACATAGTTAAACTTAACCACCCCAGTCCTTATGCAGCCAAGCTTAAGTATGCAAGAATATATTGATATAGCTATGGTTTACTTTTTTTTCTCTTTAGC
Ca_LG_7:44655530	ATTTTAAGCACCAATGTGAGCATTTACCCTTTTAAAATTAAATTTGGCCTAGAGTGTGCACATTTACTGTCGCTGCCATATTTACACCATCACAAGAATG	G/T	ATTCAGAGGTGGATGTACAGCGTAGACTAGTAGCAGCAACTACCTGCAATAAAAGTTCCTACATAGTTAAATCATCCCAAATTCACCGCTCAAGTGTACA
Ca_LG_7:44655784	TGATTATAAGGCAAGTGATTCTTAAATATGTCATGACTAAAAAAATAACAGGAAAACAAAAGAGGACAAATTTCAAGAACTTGTATACCTCTTCACCAAT	T/C	AGTCTCTTGTTAAAAAACATAGAGCAGCACCATAATCTTGAGCACTAGCAGCAGAACTCAAAAACTAGTTGAATTATGTCACTTGTACCTGAAACGGAAC
Ca_LG_7:44887529	CTAACTAATATTATGTGTTTTTGCAGCTGTTGTTGGTGATGGATTTATTGCACAAGATATCACATTTAGGAACACTGCTGGTGCAAACAACCATCAAGCG	G/T	GTTGCATTGCGTTCTGGATCAGACTTATCTGTGTTTTACAGATGTAGCTTTGAAGGTTATCAAGACACACTATATGTGCATTCAGATAGACAATTCTATA
Ca_LG_7:44887538	ATTATGTGTTTTTGCAGCTGTTGTTGGTGATGGATTTATTGCACAAGATATCACATTTAGGAACACTGCTGGTGCAAACAACCATCAAGCGGTTGCATTG	G/C	CGTTCTGGATCAGACTTATCTGTGTTTTACAGATGTAGCTTTGAAGGTTATCAAGACACACTATATGTGCATTCAGATAGACAATTCTATAAGGAATGTA
Ca_LG_7:44887547	TTTTGCAGCTGTTGTTGGTGATGGATTTATTGCACAAGATATCACATTTAGGAACACTGCTGGTGCAAACAACCATCAAGCGGTTGCATTGCGTTCTGGA	A/C	TCAGACTTATCTGTGTTTTACAGATGTAGCTTTGAAGGTTATCAAGACACACTATATGTGCATTCAGATAGACAATTCTATAAGGAATGTAACATTTATG
Ca_LG_7:44887553	AGCTGTTGTTGGTGATGGATTTATTGCACAAGATATCACATTTAGGAACACTGCTGGTGCAAACAACCATCAAGCGGTTGCATTGCGTTCTGGATCAGAC	C/T	TTATCTGTGTTTTACAGATGTAGCTTTGAAGGTTATCAAGACACACTATATGTGCATTCAGATAGACAATTCTATAAGGAATGTAACATTTATGGCACAG

**Positions**	**-100 bp flanking sequences**	**SNPs within *desi* cultivar**	**+100 bp flanking sequences**
Ca_LG_7:44887554	GCTGTTGTTGGTGATGGATTTATTGCACAAGATATCACATTTAGGAACACTGCTGGTGCAAACAACCATCAAGCGGTTGCATTGCGTTCTGGATCAGACT	T/C	TATCTGTGTTTTACAGATGTAGCTTTGAAGGTTATCAAGACACACTATATGTGCATTCAGATAGACAATTCTATAAGGAATGTAACATTTATGGCACAGT
Ca_LG_7:44887559	TGTTGGTGATGGATTTATTGCACAAGATATCACATTTAGGAACACTGCTGGTGCAAACAACCATCAAGCGGTTGCATTGCGTTCTGGATCAGACTTATCT	T/A	GTGTTTTACAGATGTAGCTTTGAAGGTTATCAAGACACACTATATGTGCATTCAGATAGACAATTCTATAAGGAATGTAACATTTATGGCACAGTTGACT
Ca_LG_7:44887562	TGGTGATGGATTTATTGCACAAGATATCACATTTAGGAACACTGCTGGTGCAAACAACCATCAAGCGGTTGCATTGCGTTCTGGATCAGACTTATCTGTG	G/A	TTTTACAGATGTAGCTTTGAAGGTTATCAAGACACACTATATGTGCATTCAGATAGACAATTCTATAAGGAATGTAACATTTATGGCACAGTTGACTTCA
Ca_LG_7:44887570	GATTTATTGCACAAGATATCACATTTAGGAACACTGCTGGTGCAAACAACCATCAAGCGGTTGCATTGCGTTCTGGATCAGACTTATCTGTGTTTTACAG	G/A	ATGTAGCTTTGAAGGTTATCAAGACACACTATATGTGCATTCAGATAGACAATTCTATAAGGAATGTAACATTTATGGCACAGTTGACTTCATATTTGGT
Ca_LG_7:44941905	GTTCACAAAAAACAGAAAAACTAATTTTGATAACTATTATGTTCATCCAACTCTTTTATGTGAAAGCTGAGAAGCAGCTTAAGAGTTAGTTGGTAGAAGT	T/A	GATTATAATTGGTTGGAATGAATAATGTCTTATTCAAAGGAAAAACATATATAGAAACGTAATTATTTCATCTCTATATATACGTAGAAAATATGAATTA
Ca_LG_7:44956677	CATAGGAGAATAGACATCAACAAATTTAACATCGTAATCGGGAAGGGTGGCTTTAAGGTTAGAAACACCACTAGCTAACATGCCATTGAATTGTTGTGCT	T/A	AACCCATTCTCGTAATCTACAATTACCCTTAAAGGTCCTCCCAATATTGATCTAACTATAGGTAAACACCCCAATGGTAGTGTACTTAGAACCCACACAT
Ca_LG_7:44956681	GGAGAATAGACATCAACAAATTTAACATCGTAATCGGGAAGGGTGGCTTTAAGGTTAGAAACACCACTAGCTAACATGCCATTGAATTGTTGTGCTAACC	C/A	CATTCTCGTAATCTACAATTACCCTTAAAGGTCCTCCCAATATTGATCTAACTATAGGTAAACACCCCAATGGTAGTGTACTTAGAACCCACACATGTCG
Ca_LG_8:21450	AATAGTTATTGCTAAATATAACCGAAGCAATCCTAAAATTACTTAAAAGATCAATGCAGTCCTGAATTATTGGATCAACAGCTGCAATGCACCGTTTTCC	C/G	TTGCAAACATCCCACCACCACCAGTTTGTCTGATTGAATTTCTACGCTTTGGATGTTTTATTGTGCTGCCAGTTGGAGACATCAGCGCACCCGGAGAGCT
Ca_LG_8:106826	CAATTTGATACCGTAAATGATCTATACCGTTGATTCAAAATCAAACGATTCAGATTACACGATGAATTTTATCCGAATAACTGAGCTGCAATATTGTGTC	C/A	TAATTCAAAGCCTGTATATTTTCATTATCAACTCGAAGGAGGAGGATAGCCACTGGTATCTGACCATGTTGAAGGTGGTAAACCCGACCCACCACCAACA
Ca_LG_8:107418	TTTTGCTCTGCTACCTCTGAATTTCAAAGCAGCTTCATCGTAAGCTCTTGCTGCAGCTTCAGCAGTTTCGAATGTACCAAGCCATACTCTTGCTGCTTTA	A/G	TGTGGATCTCTAATCTCTGCTGCCCATTTTCCCCATGGTCTCTGTCTCACTCCTCTGTATCTTCTTCTTCTCTCTCCACTTTCTTCTTCATTTGACTCTC
Ca_LG_8:293105	TGAAAAAAGGAGAAAGAGAAATGAGGAAATGCAGGCGCCCCAAGATGAGCAGCCCCAGCAAAATCCTATGTTTGATGCAGAACAGGATGACCAGCCCCAC	C/G	CAAGATCATGTGTTTGATGATGAACATGATCAGCAGCAGCAACAGTTTGATGATGAACATGGTCAACAACACCAACCTGAAGAACCTATGTTTCCTGGAG
Ca_LG_8:337799	AGGTTAGTTGCGCTATCGATGCACACCCTATTTCAAAACTCATCCAAACATTCAAAGATGCCGAAATCAGTATCGTTGAGTCAAAACTCAACACTGCTAG	G/A	TGATACCGTCTTCCACACATTCATAATCAAGTCTCAAGGTTCCGAGCAGCTGACAAAGGAGAAATTGATTGCAGCATTTTCCAGAGAATCCAACTCTTTA

Positions	-100 bp flanking sequences	SNPs within *desi* cultivar	+100 bp flanking sequences
Ca_LG_8:337800	GGTTAGTTGCGCTATCGATGCACACCCTATTTCAAAACTCATCCAAACATTCAAAGATGCCGAAATCAGTATCGTTGAGTCAAAACTCAACACTGCTAGT	T/C	GATACCGTCTTCCACACATTCATAATCAAGTCTCAAGGTTCCGAGCAGCTGACAAAGGAGAAATTGATTGCAGCATTTTCCAGAGAATCCAACTCTTTAT
Ca_LG_8:531135	TATTAGCAGCAGAAAAATCGCCGTGGCCTAACTATAGAAGGAAAAACAATGTGTCGACAGTCAAACTGAAATGAAAACCAAAAAATGGACAGCTTGTGCA	A/T	AACAAACAGATAGATAATATAGACGACTTAATATGATAAAATCATAAAAATATTCATTCTACAATGTATTAGTGCTGTAAGAGAACTGTTGGTAAATGGA
Ca_LG_8:1083880	TCAGAGTCCAGATGGTTGCACAGTGGCATCAGCAGCGGCCGATGAAACACTGAGGTTTTGGAATGCTTTTGGCACTCCAGAAGTAGTAACCAAAGCTGCT	T/A	CCAAAAGCTAGAGCTGAGCCATTTTCACATCTTAGTCGCATTCGATAAGATGCCACGTGTGAAATTGTTTGTTTGTTGTCACCAAGTTATATTTTCTACT
Ca_LG_8:1433753	ACAAAAAGACCATCCGAAAACCAGAACTCGCCTTCATTTTTCTTCTTAGGTGTTAAATACAACTCATTGTTCAACGCAGCTTCGATTTTCGTCATTCTAC	C/G	TATCATTATTCTTCTTCATACATTTAGGATACACAGTATTCCATTGCTGCTCTAAAGCTATAGTAGTACTAGCAGCACTTCCAACACTTCCAACACATGA
Ca_LG_8:1518484	AATTCTCAAAGGCCTAGGCAAAACCAAGGCCTAAGAGGGGGTTTGGAAATAGTTTTCAACCAGCAATAGACGCGCCTCGGTTAAAACCTCACTAGCTGCG	G/A	TCATTGCCCCAAGCGCAAAGATGCTTTTGAAAAATCTTGTGACAACTCTATTAATTGAAGCTGCCAATAATTTTGTATATTGTTTTGGTCGATGTGGTAC
Ca_LG_8:1525590	ATTGTTTCGAAGGGAATTGACATTTGAGCAAACTCTTTGCCTTTGGGAAGTAATGTGGGCAGATCAAGCTGCAATCAGAGCAGGTATCGGGATTTCTGCA	A/G	TGGAGTAAAGTTAGGCAGCGTGCACCGCCAACAGATGATCTGTTGCTCTACGCAATTGCAGCTTCGGTATTACAGAGGAGGAAATTGATCATTGAAAAGT
Ca_LG_8:1683247	ATAATCAAACAACTTTGCTATCCCTTCAGTGCCACACTTGTTTGCAGTGATAGGAAATAAAAAATTCATGCATTGCTTACAGAAGCAGCAAACATATTTC	C/A	AATTCTATTAGTAACTCAGCTAGTATAACACATAGAGATAGTTTAATTTCGCTTGAATGCGATGACAATTGCAGAGCAGTTATCTTTGCAGCGACGCTCA
Ca_LG_8:3229548	CTCTCATTCCTCACTGTGTAAGTCATGATTATGTCTCTGACAATGACGCGCTACTCATCCACCATCTTCTAAACTGCAAACGTTTGAACTTGCCTCATGT	T/G	GATTCTTCAACACATGATCTGTGCAGCAACCAAAGACTACAAGAAGAATATTGTACCTTATGGAATGGTATTGACTAAAGTCTTCAGGTATTTTGGAGTC
Ca_LG_8:3229567	AAGTCATGATTATGTCTCTGACAATGACGCGCTACTCATCCACCATCTTCTAAACTGCAAACGTTTGAACTTGCCTCATGTGATTCTTCAACACATGATC	C/A	TGTGCAGCAACCAAAGACTACAAGAAGAATATTGTACCTTATGGAATGGTATTGACTAAAGTCTTCAGGTATTTTGGAGTCTCCCTTTCTTCTGAAAAAT
Ca_LG_8:4385393	TTTTAGGAAGTTGGTTGCACTGGAAAAGGTGCAGCTGGTGTTGGCACAACAATAACAATGTAAATAAGCACAGAACTACAGAAAAAACATTGGTTACGAC	C/T	GCAGCGGAATAATGCAGAGAAGACGATGGAAGGGACGCCAGAGTTGACGATGATGGACAACTGTTTATGGCAGAAAATTAAAAGATTGAGAGGTGGTGTT
Ca_LG_8:4961561	TTGGTGGGACACCCCAACCAGTGCATTGAAGTCCACCAGTGCAATCACCTGTTTGGCAGCGGCCACGACCGCTGCCATCGAAATTGCAACCAGTACGACT	T/C	CCAAATACGAGCCATTGAAGTGCCAGCGTTAACCCAAAGATTCCATGTTTGACCGCGGTCAAGGCGACGACCACCGCCCGGACTAGCAGCTGCCCAAACG
Ca_LG_8:5588533	CAGAGAGGAGATGATAGAGGTATTAGGGGAGTAGTAATAGTTGGAGTAGCCTTGCTGCAACCTCTACCAAGAGGTGAAGAGATACCTTTCCCTTGAAGCG	G/A	CACTTTGCTTAACTGTTGAATCAGATGTTCTTGCAGCTGCAGATCTCAATTAGCAAGAGTTAAACAATCACATTAACTAATCAAACTAGCAAAAAAGCTA

Positions	-100 bp flanking sequences	SNPs within *desi* cultivar	+100 bp flanking sequences
Ca_LG_8:5612684	ACACGGATACCGGCACGACAATAAATTACAAACATAGAAATAGAAAGATAAATCAAGTACCTGTTGCAGCAGCAGCAGTAAGAGTCATTAGATCACCCGG	G/A	TGTTTGAATATCGACTGCAGATTTAACAGTAGAAGCAACACGGTTATAATCAGCACCTGCTAACTCTGCCATTTCAACACAGTAAGATGCTAAGAGTTGT
Ca_LG_8:6984350	TCTTCCCATGCTCGAAGGAATGATGTTTCGTCTACTGGAAACCCTGCAAATCAAGCTGCAAAAGTAACAAGGCCTTCACCTGCTGCTGCTGCTTCACCTA	A/G	CATATGATGAACAGGTATTATGTTTTTCCATCTTATAATGAAGAAATGTTGTCGAGTGAATTGTTAATGGTTGAAGGTTTGTCATAGATTTAACAATGAT
Ca_LG_8:7172479	GGTACATTTGGGTTTGTACACTCTTCTCTGTCTTTTATTTGTCCTATTCCTGCATAAAAGGACTGTATATATGACTTATGAGTTGTCAAGCCAATAATTT	T/C	ATACTTATTCTTATTTTGGCAGCTACGTTGCTCCTGAATATGCAAGTACTGGAATGTTGAACGAACGAAGTGATGTATATAGTTTTGGAATTCTTATTAT
Ca_LG_8:7345832	TTTTTTTAAGGCATTACCGGATTGTTAGCAGCCACACCACCATCTACGAGATTGTATTCTTTGATGACTTTCCCTTGTTCATCTTTTTTTTCGAAATAGT	T/A	GAGCTGGCAAATATGTTGGAGCTGCCGACGTGGCTATGCAAATATCTGACAGTGGTACATCGAAACATGGATCACATCCTATCTGCATAACATCGTTGAA
Ca_LG_8:7533984	TGGGGCAGGAAGCCGACCAATGCTATCGACTTTATCCTTTTGCTTTGGAGGTGGTCTAGCTGCTTTAGGCAACAATCTTCCTTTCTTCTCGAACCACTCA	A/G	GGCTTCAACAGTGCCCGGAAGCCCAATTTATTGTAATACACCTTTCTGACAGACCCACCAGCTGCTTCAACTGCTTCCTTGGCCCTAACAGTGACCCTTG
Ca_LG_8:7534011	GACTTTATCCTTTTGCTTTGGAGGTGGTCTAGCTGCTTTAGGCAACAATCTTCCTTTCTTCTCGAACCACTCAGGCTTCAACAGTGCCCGGAAGCCCAAT	T/C	TTATTGTAATACACCTTTCTGACAGACCCACCAGCTGCTTCAACTGCTTCCTTGGCCCTAACAGTGACCCTTGATACCTGTAAGCTCAATATATTAAAAA
Ca_LG_8:8145459	GTGGATGATACCGTTGCATTTTGCATTTGCGTTACAATTTCCTCTCAATAGGTTTCTACAGAGCCAGCTTAAAACCGCTGCTATAGCTTGGGTGTCTTTC	C/G	GTGGCACTTTTGGTTCATGTTTTTGTTAGTTGGTTGTTTGTGTTTAAGTTTCAGTTTGGTGTTATTGGAACCGCTGCTACTTTGAATTTCTCTTGGTGGG
Ca_LG_8:8175288	TTTTATACATACCAATATAATGTTTTGAAATGTCTTTGAAATTACATAAATCATGGGTATTTATTTATTTATTTTTTATTGTATGCAGCTAGTAGTACAG	G/A	GTCGGACTCAAGCTTGTGTTAAACATTCCAGACCAAACATAGTTGGGATGCAACCCTTACCATATAACATTGGAAAAGGAAAACTTCGGACTGCAGCTTT
Ca_LG_8:8904923	CTTGAACACATCCTTTACATATCTCATTTGGCACTTGAATTTCTGGAATTCCTATCACCATATCACTACTACGTAGCATTTGAAGACTTCTAAAATTCAT	T/A	ATGTCCAAACCTCTGGTGCCATAGCCAATCTTTCCTCCTGATTTCTGCAGCAAGACACTAATTTTCATTGATCTGGATTTCAATCTTGAAGGTTCTGTTC
Ca_LG_8:8904933	TCCTTTACATATCTCATTTGGCACTTGAATTTCTGGAATTCCTATCACCATATCACTACTACGTAGCATTTGAAGACTTCTAAAATTCATATGTCCAAAC	C/G	CTCTGGTGCCATAGCCAATCTTTCCTCCTGATTTCTGCAGCAAGACACTAATTTTCATTGATCTGGATTTCAATCTTGAAGGTTCTGTTCTTAGACAATG
Ca_LG_8:8904956	CTTGAATTTCTGGAATTCCTATCACCATATCACTACTACGTAGCATTTGAAGACTTCTAAAATTCATATGTCCAAACCTCTGGTGCCATAGCCAATCTTT	T/C	CCTCCTGATTTCTGCAGCAAGACACTAATTTTCATTGATCTGGATTTCAATCTTGAAGGTTCTGTTCTTAGACAATGGTGCCTTCAAGATAAGACTCTTA
Ca_LG_8:8904963	TTCTGGAATTCCTATCACCATATCACTACTACGTAGCATTTGAAGACTTCTAAAATTCATATGTCCAAACCTCTGGTGCCATAGCCAATCTTTCCTCCTG	G/A	ATTTCTGCAGCAAGACACTAATTTTCATTGATCTGGATTTCAATCTTGAAGGTTCTGTTCTTAGACAATGGTGCCTTCAAGATAAGACTCTTAGCACTAT

**Positions**	**-100 bp flanking sequences**	**SNPs within *desi* cultivar**	**+100 bp flanking sequences**
Ca_LG_8:9386225	TCTTGAGATCAAAACAAAGTAAAACAACATGGTTCCAACAATTTGTTCCAACAATGAATGTGGTTCCTACGATGCTGCTGGTATCAAGAACATTTGTGGG	G/A	AAGTGTTTTAGAGAATGGTTCGACGGCACCACTAAATCAAATAATGAAGAACATGTTTTGGAAAAACCCGTATTTGCACCGTTTTGTTCGAATGAAAAAT
Ca_LG_8:11277186	TATTTTGACAGCTTGAATAGGTGTGTGTGCGCACGTGTGACACTGACAGATAATATAGATATACCTCCGAAGGTGCAGCCCCCCATATGAAGACACGCAA	A/G	GCGAAACTGAGGAAGTCCATAAGCTCCAGCAGCCATAATTCCCAAGCGACTTTGATAGTTCATTTGAACAAGACGACCCAATGCATATCTTCCAAGAAAT
Ca_LG_8:11724247	GGATTTGGGCTATTGATAAGCCAAACATTGCACAGCCTCCAGATGGATGGCAACGGCTGCTACGAATCAGAGGTGAAGGAAGCAGCAAATTTGCAGATAT	T/C	GTATGGATTCATTATTGGTCATTCTTTTTATCTGTTATTATTATAGAAATAGCCTTATACAGTGATACGATACCACTGCTCCCCATTCTTTACGAATTTT
Ca_LG_8:12879270	ACATTCCACGTCACCTTCACCACCTCCTCCAAGCCCTCACTCTTTTCTGCAATACAACCATCGCAGCCAACACTAAAACGAAACACGCCAGGGGGATCCG	G/A	CCACCATTTCATCCGCGGTTCTCGATGCAGCGGCTACAACGCTAGAGTCTCGCTTGGAAGAAATCGAAACCGCAAAGATGATATCGCCGAGGGATAGGAT
Ca_LG_8:13504269	AATCATATGACATTTGAATCTTTTTTTCTCAACTATGTTTGTGCAGAAATAACCAAACTATGACAAGTAATAATAACACAACAGCAGCAATAACTTCAAC	C/G	TCGATCCTCTTCAAGCAAACAAGTTTACTCATCAATCTCTAGTGCTCTTCTTTTAACCTTTCTCCTCTTTCTTTTCGATCTCGAACGAGCTTAACAAGCT
Ca_LG_8:13532638	AAAAGTAAATTATACAAACATTTTCAAAAAAAAAAAAAATAATAACATATTGGATAATCAAAATAATATAGTGATTATTTTCATGCATCTGGATGGATACT	T/A	TTTGAATTGAGTCTTTGGTTCTCTCATTCCATGTTTCAACTTTCAAGGTGACAGCAGCCTTTTCTTGTGTTTCTTTTTCTTTTGTTTTTTCATCTTTGAT
Ca_LG_8:13532647	TTATACAAACATTTTCAAAAAAAAAAAAAATAATAACATATTGGATAATCAAAATAATATAGTGATTATTTTCATGCATCTGGATGGATACTTTTGAATTG	G/T	AGTCTTTGGTTCTCTCATTCCATGTTTCAACTTTCAAGGTGACAGCAGCCTTTTCTTGTGTTTCTTTTTCTTTTGTTTTTTCATCTTTGATTTGATTTTG
Ca_LG_8:13796464	CCGTGGTATGCGCCAATAAGACCACCAAAAGCCTTTGTGGCACTAGTGGTACACAGAAGTCATGGGTGATCATTGGTCCGATGCTTCGGGCGAAACCAAT	T/C	TCCCAGGGTGTGACGGGCGGTGTGTACAGGGCCCGGGTACATATTCACCGCGGCATGCTGATCCGCGATTACTAGCGATTCCAACTTCATGTTCCCGAGT
Ca_LG_8:13796470	TATGCGCCAATAAGACCACCAAAAGCCTTTGTGGCACTAGTGGTACACAGAAGTCATGGGTGATCATTGGTCCGATGCTTCGGGCGAAACCAATTCCCAG	G/T	GGTGTGACGGGCGGTGTGTACAGGGCCCGGGTACATATTCACCGCGGCATGCTGATCCGCGATTACTAGCGATTCCAACTTCATGTTCCCGAGTTGCAGA
Ca_LG_8:13796493	AGCCTTTGTGGCACTAGTGGTACACAGAAGTCATGGGTGATCATTGGTCCGATGCTTCGGGCGAAACCAATTCCCAGGGTGTGACGGGCGGTGTGTACAG	G/A	GGCCCGGGTACATATTCACCGCGGCATGCTGATCCGCGATTACTAGCGATTCCAACTTCATGTTCCCGAGTTGCAGAGAACAATCCGAACTGAGGCAATC
Ca_LG_8:13796502	GGCACTAGTGGTACACAGAAGTCATGGGTGATCATTGGTCCGATGCTTCGGGCGAAACCAATTCCCAGGGTGTGACGGGCGGTGTGTACAGGGCCCGGGT	T/A	ACATATTCACCGCGGCATGCTGATCCGCGATTACTAGCGATTCCAACTTCATGTTCCCGAGTTGCAGAGAACAATCCGAACTGAGGCAATCTTTCCGGAT
Ca_LG_8:13796505	ACTAGTGGTACACAGAAGTCATGGGTGATCATTGGTCCGATGCTTCGGGCGAAACCAATTCCCAGGGTGTGACGGGCGGTGTGTACAGGGCCCGGGTACA	A/G	TATTCACCGCGGCATGCTGATCCGCGATTACTAGCGATTCCAACTTCATGTTCCCGAGTTGCAGAGAACAATCCGAACTGAGGCAATCTTTCCGGATTCG

Positions	-100 bp flanking sequences	SNPs within *desi* cultivar	+100 bp flanking sequences
Ca_LG_8:13796528	GGTGATCATTGGTCCGATGCTTCGGGCGAAACCAATTCCCAGGGTGTGACGGGCGGTGTGTACAGGGCCCGGGTACATATTCACCGCGGCATGCTGATCC	C/T	GCGATTACTAGCGATTCCAACTTCATGTTCCCGAGTTGCAGAGAACAATCCGAACTGAGGCAATCTTTCCGGATTCGCTCCGCCTTACAGCCTTGCTTCC
Ca_LG_8:13796658	CCGAGTTGCAGAGAACAATCCGAACTGAGGCAATCTTTCCGGATTCGCTCCGCCTTACAGCCTTGCTTCCCATTGTAATTGCCATTGTAGCACGTGTGTG	G/A	GCCCAGCCCATAAGGGCCATGCGGACTTGACGTCATCCCCACCTTCCTCCAGTATATCACTGGCAGTCCCTCGTGAGTGCGGCATGCACCTTTTAGTTTG
Ca_LG_8:13796668	GAGAACAATCCGAACTGAGGCAATCTTTCCGGATTCGCTCCGCCTTACAGCCTTGCTTCCCATTGTAATTGCCATTGTAGCACGTGTGTGGCCCAGCCCA	A/G	TAAGGGCCATGCGGACTTGACGTCATCCCCACCTTCCTCCAGTATATCACTGGCAGTCCCTCGTGAGTGCGGCATGCACCTTTTAGTTTGTTTCGGAGCC
Ca_LG_8:13796680	AACTGAGGCAATCTTTCCGGATTCGCTCCGCCTTACAGCCTTGCTTCCCATTGTAATTGCCATTGTAGCACGTGTGTGGCCCAGCCCATAAGGGCCATGC	C/A	GGACTTGACGTCATCCCCACCTTCCTCCAGTATATCACTGGCAGTCCCTCGTGAGTGCGGCATGCACCTTTTAGTTTGTTTCGGAGCCGTTTGTTTTGGC
Ca_LG_8:13797322	GACACGAGCTGACGACAGCCATGCAGCACCTGTATGAAAGTCAGTACCATCCCGTTAAGGACAGGTTTTCTTGTTCATATGTCAAGGGCTGGTAAGGTTT	T/C	TGCGCGTTGTATCGAATTAAACCACATGCTCCACCGCTTGTGCAGGCCCCCGTCAATTCCTTTGAGTTTCGGTCTTGCGACCGTACTCCCCAGGCGGAGT
Ca_LG_8:13797332	GACGACAGCCATGCAGCACCTGTATGAAAGTCAGTACCATCCCGTTAAGGACAGGTTTTCTTGTTCATATGTCAAGGGCTGGTAAGGTTTTGCGCGTTGT	T/C	ATCGAATTAAACCACATGCTCCACCGCTTGTGCAGGCCCCCGTCAATTCCTTTGAGTTTCGGTCTTGCGACCGTACTCCCCAGGCGGAGTGTTTCACGCG
Ca_LG_8:13797366	TACCATCCCGTTAAGGACAGGTTTTCTTGTTCATATGTCAAGGGCTGGTAAGGTTTTGCGCGTTGTATCGAATTAAACCACATGCTCCACCGCTTGTGCA	A/G	GGCCCCCGTCAATTCCTTTGAGTTTCGGTCTTGCGACCGTACTCCCCAGGCGGAGTGTTTCACGCGTTAGCTGGGCCCCTGATCCACGTAGACCAAGGGC
Ca_LG_8:13797392	TTGTTCATATGTCAAGGGCTGGTAAGGTTTTGCGCGTTGTATCGAATTAAACCACATGCTCCACCGCTTGTGCAGGCCCCCGTCAATTCCTTTGAGTTTC	C/T	GGTCTTGCGACCGTACTCCCCAGGCGGAGTGTTTCACGCGTTAGCTGGGCCCCTGATCCACGTAGACCAAGGGCGAACACTCATCGTTTACGGCATGGAC
Ca_LG_8:13797393	TGTTCATATGTCAAGGGCTGGTAAGGTTTTGCGCGTTGTATCGAATTAAACCACATGCTCCACCGCTTGTGCAGGCCCCCGTCAATTCCTTTGAGTTTCG	G/A	GTCTTGCGACCGTACTCCCCAGGCGGAGTGTTTCACGCGTTAGCTGGGCCCCTGATCCACGTAGACCAAGGGCGAACACTCATCGTTTACGGCATGGACT
Ca_LG_8:13797394	GTTCATATGTCAAGGGCTGGTAAGGTTTTGCGCGTTGTATCGAATTAAACCACATGCTCCACCGCTTGTGCAGGCCCCCGTCAATTCCTTTGAGTTTCGG	G/A	TCTTGCGACCGTACTCCCCAGGCGGAGTGTTTCACGCGTTAGCTGGGCCCCTGATCCACGTAGACCAAGGGCGAACACTCATCGTTTACGGCATGGACTA
Ca_LG_8:13797483	TTGAGTTTCGGTCTTGCGACCGTACTCCCCAGGCGGAGTGTTTCACGCGTTAGCTGGGCCCCTGATCCACGTAGACCAAGGGCGAACACTCATCGTTTAC	C/G	GGCATGGACTACCAGGGTATCTAATCCCGTTCGCTCCCCATGCTTTCGCACTCCAGCGTCGGTAGGGACCCAGAGAGCTGCCTTCGCTTTTGGCGTTCCT
Ca_LG_8:13797487	GTTTCGGTCTTGCGACCGTACTCCCCAGGCGGAGTGTTTCACGCGTTAGCTGGGCCCCTGATCCACGTAGACCAAGGGCGAACACTCATCGTTTACGGCA	A/G	TGGACTACCAGGGTATCTAATCCCGTTCGCTCCCCATGCTTTCGCACTCCAGCGTCGGTAGGGACCCAGAGAGCTGCCTTCGCTTTTGGCGTTCCTTCGT

Positions	-100 bp flanking sequences	SNPs within *desi* cultivar	+100 bp flanking sequences
Ca_LG_8:13797511	CCAGGCGGAGTGTTTCACGCGTTAGCTGGGCCCCTGATCCACGTAGACCAAGGGCGAACACTCATCGTTTACGGCATGGACTACCAGGGTATCTAATCCC	C/T	GTTCGCTCCCCATGCTTTCGCACTCCAGCGTCGGTAGGGACCCAGAGAGCTGCCTTCGCTTTTGGCGTTCCTTCGTAGATCTGCGGATTTCACCCCTACA
Ca_LG_8:13797515	GCGGAGTGTTTCACGCGTTAGCTGGGCCCCTGATCCACGTAGACCAAGGGCGAACACTCATCGTTTACGGCATGGACTACCAGGGTATCTAATCCCGTTC	C/T	GCTCCCCATGCTTTCGCACTCCAGCGTCGGTAGGGACCCAGAGAGCTGCCTTCGCTTTTGGCGTTCCTTCGTAGATCTGCGGATTTCACCCCTACACACG
Ca_LG_8:13797524	TTCACGCGTTAGCTGGGCCCCTGATCCACGTAGACCAAGGGCGAACACTCATCGTTTACGGCATGGACTACCAGGGTATCTAATCCCGTTCGCTCCCCAT	T/C	GCTTTCGCACTCCAGCGTCGGTAGGGACCCAGAGAGCTGCCTTCGCTTTTGGCGTTCCTTCGTAGATCTGCGGATTTCACCCCTACACACGAAATTCCAC
Ca_LG_8:13797885	GCCGGGGCTTCTTCCTCGAGTCCTGTCATGATCGCGCACTCGACGAAAGAGCTTTACAAGCGGCATTGCCCTTCTTCACTCACGCGATATTGCTGGATCG	G/A	GGCTTTCGCCCATTGTCCAAGATTCCCCACTGCTGCCCCCCGTGGGAGTCCGGGCCGTGTCTCAGTCCCAGTGTGGCTGATCATCCGAAAAGACCAGCTA
Ca_LG_8:13797923	CTCGACGAAAGAGCTTTACAAGCGGCATTGCCCTTCTTCACTCACGCGATATTGCTGGATCGGGCTTTCGCCCATTGTCCAAGATTCCCCACTGCTGCCC	C/T	CCCGTGGGAGTCCGGGCCGTGTCTCAGTCCCAGTGTGGCTGATCATCCGAAAAGACCAGCTAAGCATCATTGGCTTGGTCAGCCTTTACCTTACCAACTA
Ca_LG_8:13797929	GAAAGAGCTTTACAAGCGGCATTGCCCTTCTTCACTCACGCGATATTGCTGGATCGGGCTTTCGCCCATTGTCCAAGATTCCCCACTGCTGCCCCCCGTG	G/A	GGAGTCCGGGCCGTGTCTCAGTCCCAGTGTGGCTGATCATCCGAAAAGACCAGCTAAGCATCATTGGCTTGGTCAGCCTTTACCTTACCAACTACCTAAT
Ca_LG_8:13797936	CTTTACAAGCGGCATTGCCCTTCTTCACTCACGCGATATTGCTGGATCGGGCTTTCGCCCATTGTCCAAGATTCCCCACTGCTGCCCCCCGTGGGAGTCC	C/T	GGGCCGTGTCTCAGTCCCAGTGTGGCTGATCATCCGAAAAGACCAGCTAAGCATCATTGGCTTGGTCAGCCTTTACCTTACCAACTACCTAATACTACGC
Ca_LG_8:13797942	AAGCGGCATTGCCCTTCTTCACTCACGCGATATTGCTGGATCGGGCTTTCGCCCATTGTCCAAGATTCCCCACTGCTGCCCCCCGTGGGAGTCCGGGCCG	G/T	TGTCTCAGTCCCAGTGTGGCTGATCATCCGAAAAGACCAGCTAAGCATCATTGGCTTGGTCAGCCTTTACCTTACCAACTACCTAATACTACGCAGGCTC
Ca_LG_8:14508234	TCCTAGAAAGTCTATTAATTGTCTCTGGTTATATGTGTGTTGGTTGCGAAATTACACAAACCATGGCTGCTTAACTTTTAGGCAGTGAGCAAGCATATAA	A/G	TTATAAATGTTTTCAGTACATGACTATAATATCCAAGATCTATCGAACCAAAAATATACCTTGCCAATTGCATTGCTTAATTTGGAAAATGCTTCCACCT
Ca_LG_8:15270292	ACCACAAAAAATTAGACCGTTACATTTTGTGGCGGCAAAGGCCGCCACAAACAACAATAGTACCGTTGGCACGTTCTGGCCGCCCAGGCCGCCACAAATG	G/A	CCTAATATCCGTTGGTCATTGTGGTTGTTTTGGCCGCCACAAAAGATCTATCATACCCTATATATATTACTCCACTCCTCTTTCATTTTTTCACTTCTAA
Ca_LG_8:15577061	GCCATTTTTTGGCGGTACGTTAGCAGCACGTCTATCGATTGTGGCTATGCTTGTGAAATACAATTTGAATTTTAAATTGACAAATTGTTTGTTGTCTACT	T/A	TTAGCAGCATGGCTAAAATTGAGCTGCCATATTTTGCAACTGAGATTACAACTCCATCAAGCCCCTTTGCACTATCTCTAGCAAAGTATTTGCATTCCAT
Ca_LG_8:17305796	GAGCTTGGTTTTGTTACTTGGGATACAAGAAACAACAATCATGAGTCTCAATTGCCTTACCTGCAGCCAAGTTCTACAAAGGACAGATTCATTTAGAGAA	A/G	TTATTCAAAGAAAATGAATATCAAGAAAGTCGCAAAAACGTCGACAGAACTTGGTCGGGAATATGTCGGCACCGCTGCATAAAGAGATACCAAAAGGCA

**Positions**	**-100 bp flanking sequences**	**SNPs within *desi* cultivar**	**+100 bp flanking sequences**
Ca_LG_8:17316273	GAATATAAGGAAGAGCCCTTTACATCAACCCAATTTCGGCAACAATGCTGCTAGACAACAGCCTCAGCCTCAGGTTTACAATATAAGCAAGAACGATTTT	T/C	CGGGATATTGTTCAGCAGCTTACTGGATCACCTTCACAGGATCCTCCACCTCGGCCTCCACATAATCCGCCAAAACCGCAGAGCATGCGTTTGCAGAAGA
Ca_LG_8:17354052	ATGTAAAATGCAGCAAGCCATGCTGTTTTCTGCCATCAATAAAAACATTATAAAGCCGAAACTCTGCATTCAATGTTAGAGATTTTTTTTTATTGAATTA	A/C	TGAAGGGGAGTAGAGAAATGAGGCATACATACCTGTGCAACAGGGGCATTGTCATCTATAAATGGTGCTGCAAGACTTATGAAGGAAGCCTCACCAACAC

Positions	-100 bp flanking sequences	SNPs within *kabuli* cultivar	+100 bp flanking sequences
Ca_LG_1:230358	CCTGAAGCCGACAAGGTCAAGGAGTTCCTTAAGGTAATTGCTTGGAAATTTTAATCATCTCACTATTTGAGAACAATCTGTTAATTCTGTATTTCTTATA	A/T	CATCAATATTGTTCAATGCAGGACCCAAGCAAGTTTGCTGTAGCTGCAGTTGCTACCCCAGCTGCTGATTCTGGTGCTGCCCCTGCTGCTGCTGCCAAGG
Ca_LG_1:799854	CCTAAATTGTGTCTGCAATGCCGTTGTGTAGCCTTCAAAATCCGCAATATTGCGGCTGCAATTGTGGTTGCAGACCGCAATTTACAATTGTGTTGCAGTT	T/C	GAGTTCCACTTTGCACTGCGATTCCGCGCTACAGCGGCACGATTGACATCGTAGCTTAATTTCATACGTCGTTGCTCGTTTTCTGTGCTCTAAATTTTGA
Ca_LG_1:799879	GTGTAGCCTTCAAAATCCGCAATATTGCGGCTGCAATTGTGGTTGCAGACCGCAATTTACAATTGTGTTGCAGTTGAGTTCCACTTTGCACTGCGATTCC	C/T	GCGCTACAGCGGCACGATTGACATCGTAGCTTAATTTCATACGTCGTTGCTCGTTTTCTGTGCTCTAAATTTTGATTTCACATCCTTAGGTATCTATAGT
Ca_LG_1:912192	TTAGTTCCTTTTGCGCCTCTCGTGCCTCGTGTTCTGCCGTTTTTGCTCGCTCTTCAAATGCTGCTATCTGTGCAACTATCTTGTTAGAGTGCTGAGATAA	A/G	GACATTGGCGCAACCTCTAGATGGACGAGAATCTGGGACTATACTTGCGACACATGGTCTATAAAGCGAGGATCTATCTCCAACACCATAGATCCTCTCT
Ca_LG_1:912201	TTTGCGCCTCTCGTGCCTCGTGTTCTGCCGTTTTTGCTCGCTCTTCAAATGCTGCTATCTGTGCAACTATCTTGTTAGAGTGCTGAGATAAGACATTGGC	C/A	GCAACCTCTAGATGGACGAGAATCTGGGACTATACTTGCGACACATGGTCTATAAAGCGAGGATCTATCTCCAACACCATAGATCCTCTCTTTATTCTTC
Ca_LG_1:912209	TCTCGTGCCTCGTGTTCTGCCGTTTTTGCTCGCTCTTCAAATGCTGCTATCTGTGCAACTATCTTGTTAGAGTGCTGAGATAAGACATTGGCGCAACCTC	C/T	TAGATGGACGAGAATCTGGGACTATACTTGCGACACATGGTCTATAAAGCGAGGATCTATCTCCAACACCATAGATCCTCTCTTTATTCTTCCCCCAACA
Ca_LG_1:1153230	ACCTCAAGCCATCTAGAAAAGCCAAAGGCAGCTTTTGGGATATAATCCGGTGCTTCTCCATCATTCTTGACAAAAGACTGGCATTTGATTTTGTATACAA	A/G	ACTTATCCTTTGTACGACATTGCATTTGATTTTCTCATTTCATGTCATATAATGACTGAAAAATTTAAACAGACTGGTACTTGATTCAAATGGCAGCATT
Ca_LG_1:1153271	ATAATCCGGTGCTTCTCCATCATTCTTGACAAAAGACTGGCATTTGATTTTGTATACAAACTTATCCTTTGTACGACATTGCATTTGATTTTCTCATTTC	C/G	ATGTCATATAATGACTGAAAAATTTAAACAGACTGGTACTTGATTCAAATGGCAGCATTAGAGTATTATGTTCTTATCAAACAACGTTATTTGTAATTAT
Ca_LG_1:1187336	TATATATCACAAATACCACTATTTGTTGCAAGTTTAAGAGAGAGTTAATACAACTCCCAAGAACACAAAGTAGATGTTGGCTGCTTCCTAAATAGTAAGG	G/A	CATGATGGAGTCTCCTCCATCAGTGGGCAATCAAAGATCCACGTAAATGTTGGCAGCTTCCTAAATGGTAAGACATGATGGAGAGTAATAGCAAGGGACT
Ca_LG_1:1564405	AACTTTTTGGCCAAATCATAAATTTTTAACACACAATACCATTTTGTAATAGCAAGATACCTCGTGTGCAGCGTAAATCTCAGTGTTAAAACCAATCTCG	G/T	TCGCCGTGTTCATTAGTACCAAAGCCTCTAGGTTTTCCAAAATAGATACCTGAATAACAAACAAAGCAGCAGCAAGACTGAGTTAGGTGAATTAGCTTCC
Ca_LG_1:1564411	TTGGCCAAATCATAAATTTTTAACACACAATACCATTTTGTAATAGCAAGATACCTCGTGTGCAGCGTAAATCTCAGTGTTAAAACCAATCTCGTCGCCG	G/A	TGTTCATTAGTACCAAAGCCTCTAGGTTTTCCAAAATAGATACCTGAATAACAAACAAAGCAGCAGCAAGACTGAGTTAGGTGAATTAGCTTCCAATATT
Ca_LG_1:1564415	CCAAATCATAAATTTTTAACACACAATACCATTTTGTAATAGCAAGATACCTCGTGTGCAGCGTAAATCTCAGTGTTAAAACCAATCTCGTCGCCGTGTT	T/A	CATTAGTACCAAAGCCTCTAGGTTTTCCAAAATAGATACCTGAATAACAAACAAAGCAGCAGCAAGACTGAGTTAGGTGAATTAGCTTCCAATATTAACA

**Positions**	**-100 bp flanking sequences**	**SNPs within *kabuli* cultivar**	**+100 bp flanking sequences**
Ca_LG_1:1785547	CTCTTTCCCAACTGGATCAAGCCAGCAGATTCAGAGCCACCACCTCTTTTGGTTTACAAATGGTGTCAAGGTATAAACAATTTGCAGGGTATATGGGACA	A/C	CCAGTGATGGTCAGTGTGTTGTGATGCTTCAGACGAAGTTTGAGAAATTTTTCGAGAAGATTGATTTAACAATGTTAAATAGGTATGCAATTGTAAACCA
Ca_LG_1:1968008	ACTGTGTTTGAAAGAAAAAAAAAAGTCACTGAAAAACACATAGTGAAGGTTGTGCTTTTGAGGCCAGCTGTAGCGTCGTGGCTGGTTGCTAGAAAGACAG	G/A	CCGGAGTTGATGGTGACAGATGACTGTATGAAGCCAAAAAATTGAAAGACAGAAAAAGGTGGTGCTGTTGGCGGCATTTGCTGCCGCCGGCAGCGTTAGT
Ca_LG_1:1968018	AAAGAAAAAAAAAAGTCACTGAAAAACACATAGTGAAGGTTGTGCTTTTGAGGCCAGCTGTAGCGTCGTGGCTGGTTGCTAGAAAGACAGCCGGAGTTGA	A/G	TGGTGACAGATGACTGTATGAAGCCAAAAAATTGAAAGACAGAAAAAGGTGGTGCTGTTGGCGGCATTTGCTGCCGCCGGCAGCGTTAGTGAAGCACAAA
Ca_LG_1:1968019	AAGAAAAAAAAAAGTCACTGAAAAACACATAGTGAAGGTTGTGCTTTTGAGGCCAGCTGTAGCGTCGTGGCTGGTTGCTAGAAAGACAGCCGGAGTTGAT	T/C	GGTGACAGATGACTGTATGAAGCCAAAAAATTGAAAGACAGAAAAAGGTGGTGCTGTTGGCGGCATTTGCTGCCGCCGGCAGCGTTAGTGAAGCACAAAG
Ca_LG_1:1968021	GAAAAAAAAAAGTCACTGAAAAACACATAGTGAAGGTTGTGCTTTTGAGGCCAGCTGTAGCGTCGTGGCTGGTTGCTAGAAAGACAGCCGGAGTTGATGG	G/T	TGACAGATGACTGTATGAAGCCAAAAAATTGAAAGACAGAAAAAGGTGGTGCTGTTGGCGGCATTTGCTGCCGCCGGCAGCGTTAGTGAAGCACAAAGAC
Ca_LG_1:1968036	CTGAAAAACACATAGTGAAGGTTGTGCTTTTGAGGCCAGCTGTAGCGTCGTGGCTGGTTGCTAGAAAGACAGCCGGAGTTGATGGTGACAGATGACTGTA	A/G	TGAAGCCAAAAAATTGAAAGACAGAAAAAGGTGGTGCTGTTGGCGGCATTTGCTGCCGCCGGCAGCGTTAGTGAAGCACAAAGACGGTCACAAAATCAAA
Ca_LG_1:1968059	GTGCTTTTGAGGCCAGCTGTAGCGTCGTGGCTGGTTGCTAGAAAGACAGCCGGAGTTGATGGTGACAGATGACTGTATGAAGCCAAAAAATTGAAAGACA	A/T	GAAAAAGGTGGTGCTGTTGGCGGCATTTGCTGCCGCCGGCAGCGTTAGTGAAGCACAAAGACGGTCACAAAATCAAAACCTGCTCTGATACCATGTAGAA
Ca_LG_1:3462003	CAAGTAATCATAACATAAAACAAACGGGTGCATGTGATAGAATGCATACAGTTCATTTGCAGCATACAGTTGTGCTTCATCTTTAGGCATGCTGCAATAA	A/C	AAGATCAAATTATAATTACTACTATTAGTATTATAAATGCAAGCATATGTGAAAATGAATATGATTGGTTTTCAAAAAAGCGCACCTGTAAAATATGTAA
Ca_LG_1:3462525	CAAGCCAAGCAAACCAAATGGATCAGGAGTAGATTGTGCAGTTTGCATAGACTTCATGCCATGATCCCTAAAGGACTGATTTGCAGCTGACATTTGCTGC	C/T	AGGCGAAACTGGGACTGGTTCTGGTGTTGTTGATACTGCTGGATGAGCTGGTCATATGAACCCGTACCAGAAACTGTATTTGGAGAATTTAGGGGCCTCA
Ca_LG_1:3473190	CAATCCTAATGCTATTTTGAATGGACTAGAGATCATGAAAATGAACAATTCCATAGGTAGTCTTAGTGCTGCAGCAGCGTCTGGTACTGGGGGTGATTCC	C/T	TCTGGTTCGAGTTCTAGTAAGGTTGGCGTGATAGCAGGTGTGAGTGTAGGGATAGTGAGTGCATTGGTCTTGGCCGGTGTTTTCTGTGTAATGTGCAGGA
Ca_LG_1:3923732	TCCTTTGTGAGGGCTTTTCAGCCAAAAAATCAGCCATATATGAGGGTAAAGGCTGCCACAAAGGCAATCCATTTGTGACCGCCACAAAATCAGTATACCG	G/A	TTGCACTTTGTGGCTGCAAAGGCCACCACAAATACTCATTGTACCGTTGCCCGTTGTGGCTGCTTAGGCCGCTACAAATTCCTTCATTTCTGACCGTAAA
Ca_LG_1:4151327	TGATTTTTTCTTCCTGTTAATCTGGTTTTTGCAGCGAGGAGATAATTAAAAGCAAATTCAATTCTATGATTGGTCTTGTTGACCGTGAACGTGTTGATGC	C/G	AGCACTGTATGATGATTTTGAAACCGTAAAGGCTCGCGAGGTGTGTTCAATTTTACTTTGCAGCATTGGTTGCTGTACTACTGAGATATACTAGTTAGTA

Positions	-100 bp flanking sequences	SNPs within *kabuli* cultivar	+100 bp flanking sequences
Ca_LG_1:4210820	GCAGAATCTTGATTGAGCCCGAGGTTCAGCCTTCTGACTTGGCTACCAGAATCTCAATCGAACCTGAGGATCAGCCTTCTGACTCGGCTAGCAGCATCTC	C/G	GATTGAACTCAAGGATCAGCCTCTTGACTTGGATGGAGATGATGCAACTAATAATTTGGCAGCATTTGAATATACTGATGAACTATACAATTTCTACAAG
Ca_LG_1:4359791	TTCTTTCCAATTCAATACAAACAAAAACCATAACTGACCTTTAATGACCCATCTACCTAATCTTCTCCTTTCCACTGATCCTATTAGCAGCTTCCCTAAT	T/C	TTTTTCAATATAACCCTCAATAGGAGGAGTAAGAGTAGCCATAAAACGATTCGCAGGAAAAGGTGAGAAATCAGGCACCGAAGCAGCTTCTTTCAAACCC
Ca_LG_1:4359834	ATGACCCATCTACCTAATCTTCTCCTTTCCACTGATCCTATTAGCAGCTTCCCTAATTTTTTCAATATAACCCTCAATAGGAGGAGTAAGAGTAGCCATA	A/G	AAACGATTCGCAGGAAAAGGTGAGAAATCAGGCACCGAAGCAGCTTCTTTCAAACCCTCAGGCAAAGCTTCAATGGCTTCCTTTTTCATCCTCAAAAGCG
Ca_LG_1:4359844	TACCTAATCTTCTCCTTTCCACTGATCCTATTAGCAGCTTCCCTAATTTTTTCAATATAACCCTCAATAGGAGGAGTAAGAGTAGCCATAAAACGATTCG	G/A	CAGGAAAAGGTGAGAAATCAGGCACCGAAGCAGCTTCTTTCAAACCCTCAGGCAAAGCTTCAATGGCTTCCTTTTTCATCCTCAAAAGCGTCGTTTCCGC
Ca_LG_1:4359845	ACCTAATCTTCTCCTTTCCACTGATCCTATTAGCAGCTTCCCTAATTTTTTCAATATAACCCTCAATAGGAGGAGTAAGAGTAGCCATAAAACGATTCGC	C/T	AGGAAAAGGTGAGAAATCAGGCACCGAAGCAGCTTCTTTCAAACCCTCAGGCAAAGCTTCAATGGCTTCCTTTTTCATCCTCAAAAGCGTCGTTTCCGCA
Ca_LG_1:4359858	CTTTCCACTGATCCTATTAGCAGCTTCCCTAATTTTTTCAATATAACCCTCAATAGGAGGAGTAAGAGTAGCCATAAAACGATTCGCAGGAAAAGGTGAG	G/C	AAATCAGGCACCGAAGCAGCTTCTTTCAAACCCTCAGGCAAAGCTTCAATGGCTTCCTTTTTCATCCTCAAAAGCGTCGTTTCCGCAGCTTGTCTCGCTC
Ca_LG_1:4359871	CTATTAGCAGCTTCCCTAATTTTTTCAATATAACCCTCAATAGGAGGAGTAAGAGTAGCCATAAAACGATTCGCAGGAAAAGGTGAGAAATCAGGCACCG	G/A	AAGCAGCTTCTTTCAAACCCTCAGGCAAAGCTTCAATGGCTTCCTTTTTCATCCTCAAAAGCGTCGTTTCCGCAGCTTGTCTCGCTCTATGCTTCCTCAT
Ca_LG_1:4359909	AATAGGAGGAGTAAGAGTAGCCATAAAACGATTCGCAGGAAAAGGTGAGAAATCAGGCACCGAAGCAGCTTCTTTCAAACCCTCAGGCAAAGCTTCAATG	G/A	GCTTCCTTTTTCATCCTCAAAAGCGTCGTTTCCGCAGCTTGTCTCGCTCTATGCTTCCTCATCAAAACCCTACTATACTCCTTCGCCAATCTCCTCCCAT
Ca_LG_1:4359922	AGAGTAGCCATAAAACGATTCGCAGGAAAAGGTGAGAAATCAGGCACCGAAGCAGCTTCTTTCAAACCCTCAGGCAAAGCTTCAATGGCTTCCTTTTTCA	A/G	TCCTCAAAAGCGTCGTTTCCGCAGCTTGTCTCGCTCTATGCTTCCTCATCAAAACCCTACTATACTCCTTCGCCAATCTCCTCCCATCTTCAGCCGTTAA
Ca_LG_1:4359923	GAGTAGCCATAAAACGATTCGCAGGAAAAGGTGAGAAATCAGGCACCGAAGCAGCTTCTTTCAAACCCTCAGGCAAAGCTTCAATGGCTTCCTTTTTCAT	T/A	CCTCAAAAGCGTCGTTTCCGCAGCTTGTCTCGCTCTATGCTTCCTCATCAAAACCCTACTATACTCCTTCGCCAATCTCCTCCCATCTTCAGCCGTTAAT
Ca_LG_1:4359933	AAAACGATTCGCAGGAAAAGGTGAGAAATCAGGCACCGAAGCAGCTTCTTTCAAACCCTCAGGCAAAGCTTCAATGGCTTCCTTTTTCATCCTCAAAAGC	C/T	GTCGTTTCCGCAGCTTGTCTCGCTCTATGCTTCCTCATCAAAACCCTACTATACTCCTTCGCCAATCTCCTCCCATCTTCAGCCGTTAATTCATACTTGG
Ca_LG_1:4359938	GATTCGCAGGAAAAGGTGAGAAATCAGGCACCGAAGCAGCTTCTTTCAAACCCTCAGGCAAAGCTTCAATGGCTTCCTTTTTCATCCTCAAAAGCGTCGT	T/A	TTCCGCAGCTTGTCTCGCTCTATGCTTCCTCATCAAAACCCTACTATACTCCTTCGCCAATCTCCTCCCATCTTCAGCCGTTAATTCATACTTGGGCAAT

Positions	-100 bp flanking sequences	SNPs within *kabuli* cultivar	+100 bp flanking sequences
Ca_LG_1:4950745	AACAAGCCAATCAAATCATGTCGTAAAGCTAAAAACAGAAAGCAGAAGCTGCATTGCTTTTTGCTGCCTGAAAATTGTATTTCAAGTAAATTGTTGATAT	T/C	CTTTGTCCTACGAACAATAAAGTTGTAAAAATACGATTTTCAATCAAACTCAGTTTTGAGCAATCTATTGACGGCATATTTCACAAGTTGCTACTTTCCT
Ca_LG_1:4950756	CAAATCATGTCGTAAAGCTAAAAACAGAAAGCAGAAGCTGCATTGCTTTTTGCTGCCTGAAAATTGTATTTCAAGTAAATTGTTGATATCTTTGTCCTAC	C/A	GAACAATAAAGTTGTAAAAATACGATTTTCAATCAAACTCAGTTTTGAGCAATCTATTGACGGCATATTTCACAAGTTGCTACTTTCCTGCACTTTTTTT
Ca_LG_1:4950763	TGTCGTAAAGCTAAAAACAGAAAGCAGAAGCTGCATTGCTTTTTGCTGCCTGAAAATTGTATTTCAAGTAAATTGTTGATATCTTTGTCCTACGAACAAT	T/C	AAAGTTGTAAAAATACGATTTTCAATCAAACTCAGTTTTGAGCAATCTATTGACGGCATATTTCACAAGTTGCTACTTTCCTGCACTTTTTTTCTTCCAC
Ca_LG_1:4950774	TAAAAACAGAAAGCAGAAGCTGCATTGCTTTTTGCTGCCTGAAAATTGTATTTCAAGTAAATTGTTGATATCTTTGTCCTACGAACAATAAAGTTGTAAA	A/T	AATACGATTTTCAATCAAACTCAGTTTTGAGCAATCTATTGACGGCATATTTCACAAGTTGCTACTTTCCTGCACTTTTTTTCTTCCACTAACAGAAATT
Ca_LG_1:4950805	TTGCTGCCTGAAAATTGTATTTCAAGTAAATTGTTGATATCTTTGTCCTACGAACAATAAAGTTGTAAAAATACGATTTTCAATCAAACTCAGTTTTGAG	G/A	CAATCTATTGACGGCATATTTCACAAGTTGCTACTTTCCTGCACTTTTTTTCTTCCACTAACAGAAATTTTCTAGAAACATATTTTTTATATAACATCAA
Ca_LG_1:5103778	CAGTACTAATTGAGAGCAAACTAATTTGTGTTTGTATAGTTCTCAAGTAACTGTCATAAATCAATGCTTTCGGTGTTGACGGCGCCGCCGTAACTGGTGG	G/T	CATCTCCACCGGAGTTGTCTCATATTCCATTGCCTTGTGGAGCGGGTTGCTGCCTTTACCATTTGGGACTGGCCATGAGACATACATATATAACACGAAT
Ca_LG_1:5103786	ATTGAGAGCAAACTAATTTGTGTTTGTATAGTTCTCAAGTAACTGTCATAAATCAATGCTTTCGGTGTTGACGGCGCCGCCGTAACTGGTGGCATCTCCA	A/G	CCGGAGTTGTCTCATATTCCATTGCCTTGTGGAGCGGGTTGCTGCCTTTACCATTTGGGACTGGCCATGAGACATACATATATAACACGAATTATGATAT
Ca_LG_1:5212532	GAGAACAAGTGTCTATCTAATCAAGAAGAAGAGTGAAGTGTTGGCTGTGTGTAGAGGGTTCAAGCTAATGGCAGAAAAACAAAGTGGCTGCAAGATTGTA	A/G	TCATTCATTAATGATTTTACAAAGAAAAATTGGGTCTATCTAATCAAGAAGAAGAGTGAAGTGTTGGCTGTGTGTAGAGGGTTCAAGCTAATGGCAAAAA
Ca_LG_1:5212680	AAGAAGAGTGAAGTGTTGGCTGTGTGTAGAGGGTTCAAGCTAATGGCAAAAAAACAAAGTGGCTGCAATCTTAAAGGGATTAGAACATATGGAGGTGGTG	G/A	AGTATCAATCTTATGAATTTTATGACAAAGAAAATTATTCATGAAGTAACTGCACCATATATATCAGGACATAATAGAACTGCATAAAGGAGAAATAGAA
Ca_LG_1:5858788	CTCAACCGAAGAGCAGCCCATGCCAATGCGCAACATGTTCGTTCAAGCAGTGAATATCTTGTTTCACAACTGGTGAACTTTTTTCTCAAATAATAAATAA	A/G	CATGTTCTTTCCTACCAGATTCATCATGCTTCCCCAGTACACAACCCATTGACTCGTCAAGTATTGTTAAGTACATAATAAGTGATTTCCCAGGAACATG
Ca_LG_1:6309152	ATGAATCAAAATATAAAAACAGACATACTGAACAATATAAGAATTAAAATAAGTTTTGATATTCCAGGATTGATTAACCTGAGCGGAATCAGAAATCAAC	C/A	AGCAACACAAGATCACTGCTCGATACAGTTTCCCATATATCACCTAGAGTCCCATTTTCTTCAGAAAACCCAGCAGCTCTAGCTTCAGCAAAGGAACTAG
Ca_LG_1:6309158	CAAAATATAAAAACAGACATACTGAACAATATAAGAATTAAAATAAGTTTTGATATTCCAGGATTGATTAACCTGAGCGGAATCAGAAATCAACAGCAAC	C/T	ACAAGATCACTGCTCGATACAGTTTCCCATATATCACCTAGAGTCCCATTTTCTTCAGAAAACCCAGCAGCTCTAGCTTCAGCAAAGGAACTAGAACCTT

Positions	-100 bp flanking sequences	SNPs within *kabuli* cultivar	+100 bp flanking sequences
Ca_LG_1:6436343	ATCTAAGGATCTAGCAAAGATGACGACAGCAACATTATTTGGA<br>AAATTAAGGGAACATGAAATGGAATTACAACGACTGGATGAAT<br>CAGAAATGGAAAGT	T/C	AGGAAAAAGAAAGGTTTATCTCTGAAGGTTCAAGCAAAGCAA<br>TCTAAACCTGAATCCGATAGCTACTCAAATGAATCCAGCAGT<br>GAAAATGAAGAGACTG
Ca_LG_1:6462414	NNNNNNNNNNNNNNNNNNNNNNNNNNNNNNNNNNNNNNNNN<br>NNNNNNNNNNNNNNNNNNNNNNNNCGTATGTGGCGGCCTTT<br>GCCGCCACAAAATGCAAT	T/C	GGATATAAAAATTTGTGGCGGCCTGGACGGCCAGAAAAGCC<br>AACGTCTCTTTATTCCTTTGTGGCGGCCTTTGCAGCCACAAA<br>CTGCAACGTCACTTCAA
Ca_LG_1:6462446	NNNNNNNNNNNNNNNNNNNNNNNNNNNNNNNNNCGTATGTG<br>GCGGCCTTTGCCGCCACAAAATGCAATGGATATAAAAATTTGT<br>GGCGGCCTGGACGGCC	C/T	AGAAAAGCCAACGTCTCTTTATTCCTTTGTGGCGGCCTTTGC<br>AGCCACAAACTGCAACGTCACTTCAAGTATTTGTGGCTGCAT<br>TTACCCTCACAAAATT
Ca_LG_1:6800039	TGTCTAAAACGCATGCAGCCTCTTCTCTCTTATTTTTGCAACC<br>CACCGCTGCACTGGGGTAAAAGGGGCATATCACATCTAAGTC<br>CAAGTAGCAGCTGCT	T/G	TCAGACCGATTTTGGTGCAATTTGCAGCTGCGGTAGCTACTC<br>CTGCAAAACGCAACTCATTGGACTGTTGCGCCACAATAGAG<br>CTCACCACCGCACAACT
Ca_LG_1:6800040	GTCTAAAACGCATGCAGCCTCTTCTCTCTTATTTTTGCAACCC<br>ACCGCTGCACTGGGGTAAAAGGGGCATATCACATCTAAGTCC<br>AAGTAGCAGCTGCTT	T/A	CAGACCGATTTTGGTGCAATTTGCAGCTGCGGTAGCTACTC<br>CTGCAAAACGCAACTCATTGGACTGTTGCGCCACAATAGAG<br>CTCACCACCGCACAACTG
Ca_LG_1:6822949	CAACAGCTATGCCAAGTTTCTCCCGGATAGAGGACACTCGTT<br>GTTCTGAATGTTCGAGTTCATTTGCTTTCTCACGCAATTCAGT<br>ACGTGCAGCAGTAAT	T/G	AGCTTCCTCAGCTTGATGTAAGCTTTCCCTTAGAACAAATAT<br>TTCATTTTCATTCTCAAGACGCAGCATATCTTGACGATGCAT<br>CTTTTCCTTCAGTTGA
Ca_LG_1:6824151	GCAAGTTCGATGTTGGATGCTTCCAAATCTGAACAATGCTGCT<br>TCAATCCTTCAAACTGGTCCTTGAGATCACTGTACTTTGCATC<br>CTCAATTTTCCTAC	C/T	CGTCTCGTTCCCCCTTGAACAATACAGCTGCCCTCTGAACAT<br>CCAGCTTCCAATTTAAAAGCAATTTTCTCAAGTTTCCTACTTG<br>CTCTTCAGTCAACAT
Ca_LG_1:6824153	AAGTTCGATGTTGGATGCTTCCAAATCTGAACAATGCTGCTTC<br>AATCCTTCAAACTGGTCCTTGAGATCACTGTACTTTGCATCCT<br>CAATTTTCCTACCG	G/A	TCTCGTTCCCCCTTGAACAATACAGCTGCCCTCTGAACATCC<br>AGCTTCCAATTTAAAAGCAATTTTCTCAAGTTTCCTACTTGCT<br>CTTCAGTCAACATAA
Ca_LG_1:6824162	GTTGGATGCTTCCAAATCTGAACAATGCTGCTTCAATCCTTCA<br>AACTGGTCCTTGAGATCACTGTACTTTGCATCCTCAATTTTCC<br>TACCGTCTCGTTCC	C/T	CCCTTGAACAATACAGCTGCCCTCTGAACATCCAGCTTCCAA<br>TTTAAAAGCAATTTTCTCAAGTTTCCTACTTGCTCTTCAGTCA<br>ACATAATCAATGAGT
Ca_LG_1:6826878	CAAATTAGTTTCCTCTCCTGTATCATTTTCCCTGACCGCTGAC<br>GACGAATCAACAGATGGTTCAACATTATCAGGAGCTGCTGAG<br>CTTGCCAAAGACTGT	T/C	GACTCTAATGATTCGGTGATAACCACATTGGGGTCACTGTTG<br>GTTTCAAGATTTCCATCAGTAACCTGTGATGATCTTGTTGAA<br>ATAGAGGTAACACTAG
Ca_LG_1:6826986	TGATTCGGTGATAACCACATTGGGGTCACTGTTGGTTTCAAGA<br>TTTCCATCAGTAACCTGTGATGATCTTGTTGAAATAGAGGTAA<br>CACTAGCAGCATCA	A/G	GAATCAGCATCGGACTCGGGTATCTGATGCTTGGAAGATTTT<br>TTTGATGATTTCCCACGGCTACTACCACCTTTATTGTCCTTGT<br>CCTTCTTTTGACGAT
Ca_LG_1:7226629	AGAATATAATGAGCAGCAGCGAGAATGTAATGAGCAGCAGCG<br>AGAATGTAATGAGCAGCAGCGAGAATGTAATGAGCAGCAGCG<br>AGAATGTAATGAGCAA	A/G	CAGCGAGAATGACCCAGAAACGCAGTAACGATCGGCAGCG<br>AGAAAGACCCAGAAACGCAGTAACGAGCAGCAGCGAGAAA<br>CAACCCAAAAGCACAATCGC

Positions	-100 bp flanking sequences	SNPs within *kabuli* cultivar	+100 bp flanking sequences
Ca_LG_1:7406703	TGAACCTGTCATTGATGAATTGGAGTGATTCGCAGCTGGAGAATCGTTTGAAGATTCCTCGGAGAATCTCGGAACCGAAACATTGGGAAAGTTGAGGCGG	G/C	GCACAAGAACCATACATTGCCCTGGCTGCTTCATCGTATGCAAGAGCAGCACCAATGGCAGTCAAAAAAGTACCTAGCCATAACCTTTTCCCTCTGTTCG
Ca_LG_1:8617451	GTTGGAATTGAGGCAGAAGATGAGAAGATTGAGCTTCTCCTAACTGAAGTTAAGGGAAAAGATTTTGCCGAGCTTATTGCTAGTGGAAGAGAAAAGTTGT	T/G	CTTCAGTTCCTTCTGGTGGTGGTGCTGTTGCTGTTTCTGCTGCACCTGCCGGAGGTGCTGCAGCTGCCGCACCTGCTGCTGAAGCAAAGGAGGAGAAGAA
Ca_LG_1:8617453	TGGAATTGAGGCAGAAGATGAGAAGATTGAGCTTCTCCTAACTGAAGTTAAGGGAAAAGATTTTGCCGAGCTTATTGCTAGTGGAAGAGAAAAGTTGTCT	T/A	TCAGTTCCTTCTGGTGGTGGTGCTGTTGCTGTTTCTGCTGCACCTGCCGGAGGTGCTGCAGCTGCCGCACCTGCTGCTGAAGCAAAGGAGGAGAAGAAGG
Ca_LG_1:8617459	TGAGGCAGAAGATGAGAAGATTGAGCTTCTCCTAACTGAAGTTAAGGGAAAAGATTTTGCCGAGCTTATTGCTAGTGGAAGAGAAAAGTTGTCTTCAGTT	T/G	CCTTCTGGTGGTGGTGCTGTTGCTGTTTCTGCTGCACCTGCCGGAGGTGCTGCAGCTGCCGCACCTGCTGCTGAAGCAAAGGAGGAGAAGAAGGTCGAAG
Ca_LG_1:8617462	GGCAGAAGATGAGAAGATTGAGCTTCTCCTAACTGAAGTTAAGGGAAAAGATTTTGCCGAGCTTATTGCTAGTGGAAGAGAAAAGTTGTCTTCAGTTCCT	T/C	TCTGGTGGTGGTGCTGTTGCTGTTTCTGCTGCACCTGCCGGAGGTGCTGCAGCTGCCGCACCTGCTGCTGAAGCAAAGGAGGAGAAGAAGGTCGAAGAAA
Ca_LG_1:8617489	CCTAACTGAAGTTAAGGGAAAAGATTTTGCCGAGCTTATTGCTAGTGGAAGAGAAAAGTTGTCTTCAGTTCCTTCTGGTGGTGGTGCTGTTGCTGTTTCT	T/C	GCTGCACCTGCCGGAGGTGCTGCAGCTGCCGCACCTGCTGCTGAAGCAAAGGAGGAGAAGAAGGTCGAAGAAAAGGAAGAATCCGATGATGTAAGTTTTG
Ca_LG_1:8658689	TATCTTAATATGCTTGACCAATAGTCTCAATTTCAGATATATGTGCCTCAACCTCTTTAACTTTAACAGCTTCACTCAGCTCCAACTTGTTTCTTTCAAT	T/A	TGCATCCAGCTTGGACTTTAAGTCCTCTATTTCAGCTTATGTGGCTTTAAGCATTTGTTCACAAGTAGCTTCAGCTTCATTAGCAGCTTTTATTCTCAAC
Ca_LG_1:8658704	GACCAATAGTCTCAATTTCAGATATATGTGCCTCAACCTCTTTAACTTTAACAGCTTCACTCAGCTCCAACTTGTTTCTTTCAATTGCATCCAGCTTGGA	A/C	CTTTAAGTCCTCTATTTCAGCTTATGTGGCTTTAAGCATTTGTTCACAAGTAGCTTCAGCTTCATTAGCAGCTTTTATTCTCAACTTTAGACCATGTTCA
Ca_LG_1:8658706	CCAATAGTCTCAATTTCAGATATATGTGCCTCAACCTCTTTAACTTTAACAGCTTCACTCAGCTCCAACTTGTTTCTTTCAATTGCATCCAGCTTGGACT	T/C	TTAAGTCCTCTATTTCAGCTTATGTGGCTTTAAGCATTTGTTCACAAGTAGCTTCAGCTTCATTAGCAGCTTTTATTCTCAACTTTAGACCATGTTCATC
Ca_LG_1:8658724	GATATATGTGCCTCAACCTCTTTAACTTTAACAGCTTCACTCAGCTCCAACTTGTTTCTTTCAATTGCATCCAGCTTGGACTTTAAGTCCTCTATTTCAG	G/A	CTTATGTGGCTTTAAGCATTTGTTCACAAGTAGCTTCAGCTTCATTAGCAGCTTTTATTCTCAACTTTAGACCATGTTCATCTAAAGCATTTTTCAGTAT
Ca_LG_1:8658742	TCTTTAACTTTAACAGCTTCACTCAGCTCCAACTTGTTTCTTTCAATTGCATCCAGCTTGGACTTTAAGTCCTCTATTTCAGCTTATGTGGCTTTAAGCA	A/G	TTTGTTCACAAGTAGCTTCAGCTTCATTAGCAGCTTTTATTCTCAACTTTAGACCATGTTCATCTAAAGCATTTTTCAGTATTTCAGCTTGAAAGTGAGG
Ca_LG_1:8847140	CTGAAAAATGTCTTTACATTGGATGGTTTTGGGTTTTGATGATCCCTACCTTATTGACCGCAACTTTTGTATTTATTATCACTTTCATTCTTGCCCCTCT	T/C	AGTAGATAGTGACGGTATTCATGAGCCTATTTTCTGCATCTCTACTTTACAAAAACAATATTATTTTTGGTGCCATTATTCCTACTTCTGCGGCTATTGG

Positions	-100 bp flanking sequences	SNPs within *kabuli* cultivar	+100 bp flanking sequences
Ca_LG_1:8847161	GATGGTTTTGGGTTTTGATGATCCCTACCTTATTGACCGCAACTTTTGTATTTATTATCACTTTCATTCTTGCCCCTCTAGTAGATAGTGACGGTATTCA	A/G	TGAGCCTATTTTCTGCATCTCTACTTTACAAAAACAATATTATTTTTGGTGCCATTATTCCTACTTCTGCGGCTATTGGTTTTAACTTTTACCCAATATG
Ca_LG_1:8847177	GATGATCCCTACCTTATTGACCGCAACTTTTGTATTTATTATCACTTTCATTCTTGCCCCTCTAGTAGATAGTGACGGTATTCATGAGCCTATTTTCTGC	C/G	ATCTCTACTTTACAAAAACAATATTATTTTTGGTGCCATTATTCCTACTTCTGCGGCTATTGGTTTTAACTTTTACCCAATATGGGAAGTTGTATCAAGT
Ca_LG_1:8847190	TTATTGACCGCAACTTTTGTATTTATTATCACTTTCATTCTTGCCCCTCTAGTAGATAGTGACGGTATTCATGAGCCTATTTTCTGCATCTCTACTTTAC	C/T	AAAAACAATATTATTTTTGGTGCCATTATTCCTACTTCTGCGGCTATTGGTTTTAACTTTTACCCAATATGGGAAGTTGTATCAAGTTGATGAATGGTTA
Ca_LG_1:8847191	TATTGACCGCAACTTTTGTATTTATTATCACTTTCATTCTTGCCCCTCTAGTAGATAGTGACGGTATTCATGAGCCTATTTTCTGCATCTCTACTTTACA	A/G	AAAACAATATTATTTTTGGTGCCATTATTCCTACTTCTGCGGCTATTGGTTTTAACTTTTACCCAATATGGGAAGTTGTATCAAGTTGATGAATGGTTAT
Ca_LG_1:8847192	ATTGACCGCAACTTTTGTATTTATTATCACTTTCATTCTTGCCCCTCTAGTAGATAGTGACGGTATTCATGAGCCTATTTTCTGCATCTCTACTTTACAA	A/G	AAACAATATTATTTTTGGTGCCATTATTCCTACTTCTGCGGCTATTGGTTTTAACTTTTACCCAATATGGGAAGTTGTATCAAGTTGATGAATGGTTATA
Ca_LG_1:8847207	TGTATTTATTATCACTTTCATTCTTGCCCCTCTAGTAGATAGTGACGGTATTCATGAGCCTATTTTCTGCATCTCTACTTTACAAAAACAATATTATTTT	T/C	TGGTGCCATTATTCCTACTTCTGCGGCTATTGGTTTTAACTTTTACCCAATATGGGAAGTTGTATCAAGTTGATGAATGGTTATACAATGGCGATCTGTA
Ca_LG_1:8847244	GATAGTGACGGTATTCATGAGCCTATTTTCTGCATCTCTACTTTACAAAAACAATATTATTTTTGGTGCCATTATTCCTACTTCTGCGGCTATTGGTTTT	T/G	AACTTTTACCCAATATGGGAAGTTGTATCAAGTTGATGAATGGTTATACAATGGCGATCTGTATGAACTAATTGTTCTACACTTCTTACTTGGTGTAGCT
Ca_LG_1:8847245	ATAGTGACGGTATTCATGAGCCTATTTTCTGCATCTCTACTTTACAAAAACAATATTATTTTTGGTGCCATTATTCCTACTTCTGCGGCTATTGGTTTTA	A/C	ACTTTTACCCAATATGGGAAGTTGTATCAAGTTGATGAATGGTTATACAATGGCGATCTGTATGAACTAATTGTTCTACACTTCTTACTTGGTGTAGCTT
Ca_LG_1:8847253	GGTATTCATGAGCCTATTTTCTGCATCTCTACTTTACAAAAACAATATTATTTTTGGTGCCATTATTCCTACTTCTGCGGCTATTGGTTTTAACTTTTAC	C/T	CCAATATGGGAAGTTGTATCAAGTTGATGAATGGTTATACAATGGCGATCTGTATGAACTAATTGTTCTACACTTCTTACTTGGTGTAGCTTGTTACATG
Ca_LG_1:8847300	TTATTTTTGGTGCCATTATTCCTACTTCTGCGGCTATTGGTTTTAACTTTTACCCAATATGGGAAGTTGTATCAAGTTGATGAATGGTTATACAATGGCG	G/A	ATCTGTATGAACTAATTGTTCTACACTTCTTACTTGGTGTAGCTTGTTACATGGTTCGTGAATGGGAACTTAGTTTTCGTCTGGGTATGTGCCCTTGGAT
Ca_LG_1:8847301	TATTTTTGGTGCCATTATTCCTACTTCTGCGGCTATTGGTTTTAACTTTTACCCAATATGGGAAGTTGTATCAAGTTGATGAATGGTTATACAATGGCGA	A/G	TCTGTATGAACTAATTGTTCTACACTTCTTACTTGGTGTAGCTTGTTACATGGTTCGTGAATGGGAACTTAGTTTTCGTCTGGGTATGTGCCCTTGGATT
Ca_LG_1:8847304	TTTTGGTGCCATTATTCCTACTTCTGCGGCTATTGGTTTTAACTTTTACCCAATATGGGAAGTTGTATCAAGTTGATGAATGGTTATACAATGGCGATCT	T/C	GTATGAACTAATTGTTCTACACTTCTTACTTGGTGTAGCTTGTTACATGGTTCGTGAATGGGAACTTAGTTTTCGTCTGGGTATGTGCCCTTGGATTGTT

Positions	-100 bp flanking sequences	SNPs within *kabuli* cultivar	+100 bp flanking sequences
Ca_LG_1:8847305	TTTGGTGCCATTATTCCTACTTCTGCGGCTATTGGTTTTAACTTTTACCCAATATGGGAAGTTGTATCAAGTTGATGAATGGTTATACAATGGCGATCTG	G/A	TATGAACTAATTGTTCTACACTTCTTACTTGGTGTAGCTTGTTACATGGTTCGTGAATGGGAACTTAGTTTTCGTCTGGGTATGTGCCCTTGGATTGTTG
Ca_LG_1:8847355	AATATGGGAAGTTGTATCAAGTTGATGAATGGTTATACAATGGCGATCTGTATGAACTAATTGTTCTACACTTCTTACTTGGTGTAGCTTGTTACATGGT	T/G	TCGTGAATGGGAACTTAGTTTTCGTCTGGGTATGTGCCCTTGGATTGTTGTTGCATATTCAACTCCTATTGCAATTGCTACTGTTTTCTTGATCTACCCA
Ca_LG_1:8847362	GAAGTTGTATCAAGTTGATGAATGGTTATACAATGGCGATCTGTATGAACTAATTGTTCTACACTTCTTACTTGGTGTAGCTTGTTACATGGTTCGTGAA	A/G	TGGGAACTTAGTTTTCGTCTGGGTATGTGCCCTTGGATTGTTGTTGCATATTCAACTCCTATTGCAATTGCTACTGTTTTCTTGATCTACCCAATAGGTC
Ca_LG_1:8847390	TACAATGGCGATCTGTATGAACTAATTGTTCTACACTTCTTACTTGGTGTAGCTTGTTACATGGTTCGTGAATGGGAACTTAGTTTTCGTCTGGGTATGT	T/C	GCCCTTGGATTGTTGTTGCATATTCAACTCCTATTGCAATTGCTACTGTTTTCTTGATCTACCCAATAGGTCAAGGAAGCTTTTTAGATGGTATTCCTCT
Ca_LG_1:8847403	TGTATGAACTAATTGTTCTACACTTCTTACTTGGTGTAGCTTGTTACATGGTTCGTGAATGGGAACTTAGTTTTCGTCTGGGTATGTGCCCTTGGATTGT	T/C	TGTTGCATATTCAACTCCTATTGCAATTGCTACTGTTTTCTTGATCTACCCAATAGGTCAAGGAAGCTTTTTAGATGGTATTCCTCTAGGAATTTATGGT
Ca_LG_1:8847417	GTTCTACACTTCTTACTTGGTGTAGCTTGTTACATGGTTCGTGAATGGGAACTTAGTTTTCGTCTGGGTATGTGCCCTTGGATTGTTGTTGCATATTCAA	A/G	CTCCTATTGCAATTGCTACTGTTTTCTTGATCTACCCAATAGGTCAAGGAAGCTTTTTAGATGGTATTCCTCTAGGAATTTATGGTACTTTAAACTTTAT
Ca_LG_1:8847423	CACTTCTTACTTGGTGTAGCTTGTTACATGGTTCGTGAATGGGAACTTAGTTTTCGTCTGGGTATGTGCCCTTGGATTGTTGTTGCATATTCAACTCCTA	A/G	TTGCAATTGCTACTGTTTTCTTGATCTACCCAATAGGTCAAGGAAGCTTTTTAGATGGTATTCCTCTAGGAATTTATGGTACTTTAAACTTTATGATTGT
Ca_LG_1:8847470	TAGTTTTCGTCTGGGTATGTGCCCTTGGATTGTTGTTGCATATTCAACTCCTATTGCAATTGCTACTGTTTTCTTGATCTACCCAATAGGTCAAGGAAGC	C/T	TTTTTAGATGGTATTCCTCTAGGAATTTATGGTACTTTAAACTTTATGATTGTATTTCAAGCTAAGCATAATATTCTTATGCACCCATTTCACATGTTAG
Ca_LG_1:8847475	TTCGTCTGGGTATGTGCCCTTGGATTGTTGTTGCATATTCAACTCCTATTGCAATTGCTACTGTTTTCTTGATCTACCCAATAGGTCAAGGAAGCTTTTT	T/C	AGATGGTATTCCTCTAGGAATTTATGGTACTTTAAACTTTATGATTGTATTTCAAGCTAAGCATAATATTCTTATGCACCCATTTCACATGTTAGGTGTA
Ca_LG_1:8847485	TATGTGCCCTTGGATTGTTGTTGCATATTCAACTCCTATTGCAATTGCTACTGTTTTCTTGATCTACCCAATAGGTCAAGGAAGCTTTTTAGATGGTATT	T/G	CCTCTAGGAATTTATGGTACTTTAAACTTTATGATTGTATTTCAAGCTAAGCATAATATTCTTATGCACCCATTTCACATGTTAGGTGTAGTTTGTGTAA
Ca_LG_1:8847499	TTGTTGTTGCATATTCAACTCCTATTGCAATTGCTACTGTTTTCTTGATCTACCCAATAGGTCAAGGAAGCTTTTTAGATGGTATTCCTCTAGGAATTTA	A/C	TGGTACTTTAAACTTTATGATTGTATTTCAAGCTAAGCATAATATTCTTATGCACCCATTTCACATGTTAGGTGTAGTTTGTGTAATCGGCGGCTCTTTA
Ca_LG_1:8847509	ATATTCAACTCCTATTGCAATTGCTACTGTTTTCTTGATCTACCCAATAGGTCAAGGAAGCTTTTTAGATGGTATTCCTCTAGGAATTTATGGTACTTTA	A/C	AACTTTATGATTGTATTTCAAGCTAAGCATAATATTCTTATGCACCCATTTCACATGTTAGGTGTAGTTTGTGTAATCGGCGGCTCTTTATTTAGTGTTA

Positions	-100 bp flanking sequences	SNPs within *kabuli* cultivar	+100 bp flanking sequences
Ca_LG_1:8847682	TAATCGGCGGCTCTTTATTTAGTGTTATGCACAGTTCCTTGGTAACTTCTAGTTTGATCAGGGAAACAGCAAAAAATGAATCTACTAATGAAGGTTACAA	A/G	ATTTAGTCAAGAGGAAGAAACCTATAATATTGTAGCTGCTCATGGTTATTTTGGCCGATTGATCTTCCAATATGTGAGCTTCAACATTTCTCGTCTTTAC
Ca_LG_1:8847686	CGGCGGCTCTTTATTTAGTGTTATGCACAGTTCCTTGGTAACTTCTAGTTTGATCAGGGAAACAGCAAAAAATGAATCTACTAATGAAGGTTACAAATTT	T/C	AGTCAAGAGGAAGAAACCTATAATATTGTAGCTGCTCATGGTTATTTTGGCCGATTGATCTTCCAATATGTGAGCTTCAACATTTCTCGTCTTTACATTT
Ca_LG_1:8847687	GGCGGCTCTTTATTTAGTGTTATGCACAGTTCCTTGGTAACTTCTAGTTTGATCAGGGAAACAGCAAAAAATGAATCTACTAATGAAGGTTACAAATTTA	A/G	GTCAAGAGGAAGAAACCTATAATATTGTAGCTGCTCATGGTTATTTTGGCCGATTGATCTTCCAATATGTGAGCTTCAACATTTCTCGTCTTTACATTTC
Ca_LG_1:8847757	ATGAATCTACTAATGAAGGTTACAAATTTAGTCAAGAGGAAGAAACCTATAATATTGTAGCTGCTCATGGTTATTTTGGCCGATTGATCTTCCAATATGT	T/C	GAGCTTCAACATTTCTCGTCTTTACATTTCTTTCTAGCTACTTGGCCTGTAGTAGGTATTTGGTTTACCGCCTTAGGTATCAACGCTATGAATTTCAATT
Ca_LG_1:8847761	ATCTACTAATGAAGGTTACAAATTTAGTCAAGAGGAAGAAACCTATAATATTGTAGCTGCTCATGGTTATTTTGGCCGATTGATCTTCCAATATGTGAGC	C/T	TTCAACATTTCTCGTCTTTACATTTCTTTCTAGCTACTTGGCCTGTAGTAGGTATTTGGTTTACCGCCTTAGGTATCAACGCTATGAATTTCAATTTAAA
Ca_LG_1:8847769	ATGAAGGTTACAAATTTAGTCAAGAGGAAGAAACCTATAATATTGTAGCTGCTCATGGTTATTTTGGCCGATTGATCTTCCAATATGTGAGCTTCAACAT	T/A	TTCTCGTCTTTACATTTCTTTCTAGCTACTTGGCCTGTAGTAGGTATTTGGTTTACCGCCTTAGGTATCAACGCTATGAATTTCAATTTAAATGGTTTCA
Ca_LG_1:8847797	AGAAACCTATAATATTGTAGCTGCTCATGGTTATTTTGGCCGATTGATCTTCCAATATGTGAGCTTCAACATTTCTCGTCTTTACATTTCTTTCTAGCTA	A/G	CTTGGCCTGTAGTAGGTATTTGGTTTACCGCCTTAGGTATCAACGCTATGAATTTCAATTTAAATGGTTTCAGTTTTAACCAATCCATAGTTGACAGTCA
Ca_LG_1:8847817	CTGCTCATGGTTATTTTGGCCGATTGATCTTCCAATATGTGAGCTTCAACATTTCTCGTCTTTACATTTCTTTCTAGCTACTTGGCCTGTAGTAGGTATT	T/C	TGGTTTACCGCCTTAGGTATCAACGCTATGAATTTCAATTTAAATGGTTTCAGTTTTAACCAATCCATAGTTGACAGTCAAGGTCGTGTAATTAATACAT
Ca_LG_1:8847829	ATTTTGGCCGATTGATCTTCCAATATGTGAGCTTCAACATTTCTCGTCTTTACATTTCTTTCTAGCTACTTGGCCTGTAGTAGGTATTTGGTTTACCGCC	C/G	TTAGGTATCAACGCTATGAATTTCAATTTAAATGGTTTCAGTTTTAACCAATCCATAGTTGACAGTCAAGGTCGTGTAATTAATACATGGATTGCTATTA
Ca_LG_1:8847840	TTGATCTTCCAATATGTGAGCTTCAACATTTCTCGTCTTTACATTTCTTTCTAGCTACTTGGCCTGTAGTAGGTATTTGGTTTACCGCCTTAGGTATCAA	A/G	CGCTATGAATTTCAATTTAAATGGTTTCAGTTTTAACCAATCCATAGTTGACAGTCAAGGTCGTGTAATTAATACATGGATTGCTATTATTAATCGTGTT
Ca_LG_1:8847842	GATCTTCCAATATGTGAGCTTCAACATTTCTCGTCTTTACATTTCTTTCTAGCTACTTGGCCTGTAGTAGGTATTTGGTTTACCGCCTTAGGTATCAACG	G/A	CTATGAATTTCAATTTAAATGGTTTCAGTTTTAACCAATCCATAGTTGACAGTCAAGGTCGTGTAATTAATACATGGATTGCTATTATTAATCGTGTTAA
Ca_LG_1:8847848	CCAATATGTGAGCTTCAACATTTCTCGTCTTTACATTTCTTTCTAGCTACTTGGCCTGTAGTAGGTATTTGGTTTACCGCCTTAGGTATCAACGCTATGA	A/G	ATTTCAATTTAAATGGTTTCAGTTTTAACCAATCCATAGTTGACAGTCAAGGTCGTGTAATTAATACATGGATTGCTATTATTAATCGTGTTAACCTTGG

Positions	-100 bp flanking sequences	SNPs within *kabuli* cultivar	+100 bp flanking sequences
Ca_LG_1:8847849	CAATATGTGAGCTTCAACATTTCTCGTCTTTACATTTCTTTCTAGCTACTTGGCCTGTAGTAGGTATTTGGTTTACCGCCTTAGGTATCAACGCTATGAA	A/C	TTTCAATTTAAATGGTTTCAGTTTTAACCAATCCATAGTTGACAGTCAAGGTCGTGTAATTAATACATGGATTGCTATTATTAATCGTGTTAACCTTGGT
Ca_LG_1:8847870	TCTCGTCTTTACATTTCTTTCTAGCTACTTGGCCTGTAGTAGGTATTTGGTTTACCGCCTTAGGTATCAACGCTATGAATTTCAATTTAAATGGTTTCAG	G/A	TTTTAACCAATCCATAGTTGACAGTCAAGGTCGTGTAATTAATACATGGATTGCTATTATTAATCGTGTTAACCTTGGTATGGAAGTTATGCATGAATGT
Ca_LG_1:8847884	TTCTTTCTAGCTACTTGGCCTGTAGTAGGTATTTGGTTTACCGCCTTAGGTATCAACGCTATGAATTTCAATTTAAATGGTTTCAGTTTTAACCAATCCA	A/G	TAGTTGACAGTCAAGGTCGTGTAATTAATACATGGATTGCTATTATTAATCGTGTTAACCTTGGTATGGAAGTTATGCATGAATGTAATGCTCATAATTT
Ca_LG_1:8847892	AGCTACTTGGCCTGTAGTAGGTATTTGGTTTACCGCCTTAGGTATCAACGCTATGAATTTCAATTTAAATGGTTTCAGTTTTAACCAATCCATAGTTGAC	C/T	AGTCAAGGTCGTGTAATTAATACATGGATTGCTATTATTAATCGTGTTAACCTTGGTATGGAAGTTATGCATGAATGTAATGCTCATAATTTCCCTATAG
Ca_LG_1:8847916	TTGGTTTACCGCCTTAGGTATCAACGCTATGAATTTCAATTTAAATGGTTTCAGTTTTAACCAATCCATAGTTGACAGTCAAGGTCGTGTAATTAATACA	A/C	TGGATTGCTATTATTAATCGTGTTAACCTTGGTATGGAAGTTATGCATGAATGTAATGCTCATAATTTCCCTATAGACCTAACTACGGTCGAGGTTCCAT
Ca_LG_1:8847920	TTTACCGCCTTAGGTATCAACGCTATGAATTTCAATTTAAATGGTTTCAGTTTTAACCAATCCATAGTTGACAGTCAAGGTCGTGTAATTAATACATGGA	A/G	TTGCTATTATTAATCGTGTTAACCTTGGTATGGAAGTTATGCATGAATGTAATGCTCATAATTTCCCTATAGACCTAACTACGGTCGAGGTTCCATCTAT
Ca_LG_1:8847921	TTACCGCCTTAGGTATCAACGCTATGAATTTCAATTTAAATGGTTTCAGTTTTAACCAATCCATAGTTGACAGTCAAGGTCGTGTAATTAATACATGGAT	T/C	TGCTATTATTAATCGTGTTAACCTTGGTATGGAAGTTATGCATGAATGTAATGCTCATAATTTCCCTATAGACCTAACTACGGTCGAGGTTCCATCTATA
Ca_LG_1:8847924	CCGCCTTAGGTATCAACGCTATGAATTTCAATTTAAATGGTTTCAGTTTTAACCAATCCATAGTTGACAGTCAAGGTCGTGTAATTAATACATGGATTGC	C/A	TATTATTAATCGTGTTAACCTTGGTATGGAAGTTATGCATGAATGTAATGCTCATAATTTCCCTATAGACCTAACTACGGTCGAGGTTCCATCTATAAAT
Ca_LG_1:8847934	TATCAACGCTATGAATTTCAATTTAAATGGTTTCAGTTTTAACCAATCCATAGTTGACAGTCAAGGTCGTGTAATTAATACATGGATTGCTATTATTAAT	T/C	CGTGTTAACCTTGGTATGGAAGTTATGCATGAATGTAATGCTCATAATTTCCCTATAGACCTAACTACGGTCGAGGTTCCATCTATAAATGGATAATATT
Ca_LG_1:8847939	ACGCTATGAATTTCAATTTAAATGGTTTCAGTTTTAACCAATCCATAGTTGACAGTCAAGGTCGTGTAATTAATACATGGATTGCTATTATTAATCGTGT	T/C	TAACCTTGGTATGGAAGTTATGCATGAATGTAATGCTCATAATTTCCCTATAGACCTAACTACGGTCGAGGTTCCATCTATAAATGGATAATATTTTAGT
Ca_LG_1:8847968	AGTTTTAACCAATCCATAGTTGACAGTCAAGGTCGTGTAATTAATACATGGATTGCTATTATTAATCGTGTTAACCTTGGTATGGAAGTTATGCATGAAT	T/C	GTAATGCTCATAATTTCCCTATAGACCTAACTACGGTCGAGGTTCCATCTATAAATGGATAATATTTTAGTTTTTAAGAAGGATACAAGTTTTTAAAAGT
Ca_LG_1:8847989	GACAGTCAAGGTCGTGTAATTAATACATGGATTGCTATTATTAATCGTGTTAACCTTGGTATGGAAGTTATGCATGAATGTAATGCTCATAATTTCCCTA	A/C	TAGACCTAACTACGGTCGAGGTTCCATCTATAAATGGATAATATTTTAGTTTTTAAGAAGGATACAAGTTTTTAAAAGTAAAGGGCGGGTTGATTTAAAA

Positions	-100 bp flanking sequences	SNPs within *kabuli* cultivar	+100 bp flanking sequences
Ca_LG_1:8848011	ATACATGGATTGCTATTATTAATCGTGTTAACCTTGGTATGGAAGTTATGCATGAATGTAATGCTCATAATTTCCCTATAGACCTAACTACGGTCGAGGT	T/C	TCCATCTATAAATGGATAATATTTTAGTTTTTAAGAAGGATACAAGTTTTTAAAAGTAAAGGGCGGGTTGATTTAAAAGTAAAATGCTCATAATCTCAAC
Ca_LG_1:8848055	GTTATGCATGAATGTAATGCTCATAATTTCCCTATAGACCTAACTACGGTCGAGGTTCCATCTATAAATGGATAATATTTTAGTTTTTAAGAAGGATACA	A/G	AGTTTTTAAAAGTAAAGGGCGGGTTGATTTAAAAGTAAAATGCTCATAATCTCAACAATGTTGTATATTAGTTCAAATGCCACTATCGGGTTGATTTACA
Ca_LG_1:9430870	GTAGGATCTCGAAGATCTTCTCGATTTCTTGCAGCCAAAGATCAACCTTGTCGGGGTCATGTTCCCCTTTGAATTTAGGCGGATCTTGTCGACGAAATTC	C/A	ATTCAATGCTCTTGCTGCACTGGCTACTTCATCCTTCTGATCCCTCTATGCATCTCGTTGAGCCTGGATCGCAGCTTGTTGGGCCATTGTTGCGGCCATT
Ca_LG_1:9430893	ATTTCTTGCAGCCAAAGATCAACCTTGTCGGGGTCATGTTCCCCTTTGAATTTAGGCGGATCTTGTCGACGAAATTCATTCAATGCTCTTGCTGCACTGG	G/C	CTACTTCATCCTTCTGATCCCTCTATGCATCTCGTTGAGCCTGGATCGCAGCTTGTTGGGCCATTGTTGCGGCCATTGCATTCATGGCTTGGATTGCTTC
Ca_LG_1:9430907	AAGATCAACCTTGTCGGGGTCATGTTCCCCTTTGAATTTAGGCGGATCTTGTCGACGAAATTCATTCAATGCTCTTGCTGCACTGGCTACTTCATCCTTC	C/T	TGATCCCTCTATGCATCTCGTTGAGCCTGGATCGCAGCTTGTTGGGCCATTGTTGCGGCCATTGCATTCATGGCTTGGATTGCTTCAGTCATGTCGTTTC
Ca_LG_1:9756498	GACTTGGGAACTCTTTGTTATTTCTTGGGGATTAAAGTTGCCTACTCTCCTAATGGTTGCCTTATTTCTAAATCGAACTACATTGCCAACTTTCTTAACT	T/C	AGACTCGTCTTTCTGATACTAGAGCAGCAAATACTCCTCTTGAGTTGAATGTGAAATATGCTTCCTCGGATGGTGTTCCTTTACCAGATTCCACTTTGTA
Ca_LG_1:10251478	TTCATCCCATTTTTTTGGGGATAGGCGGCGAATACAAATCTAATAAGTGAAGTAGTCGTCGTCTGACCAATTGGCTCGGACACCAGACCGCTCGTGCCCA	A/G	CCCGTTCTGTCTCGCCCTAAATGGAATGACTCTCTTAGTTACGATGCGCCCCGGTTTGAGTCCCCACTTCCACTCTTCTCTGCGCGCAACCCAAGAAGCT
Ca_LG_1:10251482	TCCCATTTTTTTGGGGATAGGCGGCGAATACAAATCTAATAAGTGAAGTAGTCGTCGTCTGACCAATTGGCTCGGACACCAGACCGCTCGTGCCCACCCG	G/A	TTCTGTCTCGCCCTAAATGGAATGACTCTCTTAGTTACGATGCGCCCCGGTTTGAGTCCCCACTTCCACTCTTCTCTGCGCGCAACCCAAGAAGCTGGCT
Ca_LG_1:10251507	GAATACAAATCTAATAAGTGAAGTAGTCGTCGTCTGACCAATTGGCTCGGACACCAGACCGCTCGTGCCCACCCGTTCTGTCTCGCCCTAAATGGAATGA	A/G	CTCTCTTAGTTACGATGCGCCCCGGTTTGAGTCCCCACTTCCACTCTTCTCTGCGCGCAACCCAAGAAGCTGGCTTTGCCAACACAACATTAGGGCCGTC
Ca_LG_1:10251522	AAGTGAAGTAGTCGTCGTCTGACCAATTGGCTCGGACACCAGACCGCTCGTGCCCACCCGTTCTGTCTCGCCCTAAATGGAATGACTCTCTTAGTTACGA	A/C	TGCGCCCCGGTTTGAGTCCCCACTTCCACTCTTCTCTGCGCGCAACCCAAGAAGCTGGCTTTGCCAACACAACATTAGGGCCGTCCCCTTCATTCTATGC
Ca_LG_1:10251534	CGTCGTCTGACCAATTGGCTCGGACACCAGACCGCTCGTGCCCACCCGTTCTGTCTCGCCCTAAATGGAATGACTCTCTTAGTTACGATGCGCCCCGGTT	T/C	TGAGTCCCCACTTCCACTCTTCTCTGCGCGCAACCCAAGAAGCTGGCTTTGCCAACACAACATTAGGGCCGTCCCCTTCATTCTATGCCGACCCCGGCCC
Ca_LG_1:10251546	AATTGGCTCGGACACCAGACCGCTCGTGCCCACCCGTTCTGTCTCGCCCTAAATGGAATGACTCTCTTAGTTACGATGCGCCCCGGTTTGAGTCCCCACT	T/G	TCCACTCTTCTCTGCGCGCAACCCAAGAAGCTGGCTTTGCCAACACAACATTAGGGCCGTCCCCTTCATTCTATGCCGACCCCGGCCCCGGGGCTGGCTTT

Positions	-100 bp flanking sequences	SNPs within *kabuli* cultivar	+100 bp flanking sequences
Ca_LG_1:10251550	GGCTCGGACACCAGACCGCTCGTGCCCACCCGTTCTGTCTCGCCCTAAATGGAATGACTCTCTTAGTTACGATGCGCCCCGGTTTGAGTCCCCACTTCCA	A/G	CTCTTCTCTGCGCGCAACCCAAGAAGCTGGCTTTGCCAACACAACATTAGGGCCGTCCCCTTCATTCTATGCCGACCCCGGCCCGGGGCTGGCTTTTTGG
Ca_LG_1:10251559	ACCAGACCGCTCGTGCCCACCCGTTCTGTCTCGCCCTAAATGGAATGACTCTCTTAGTTACGATGCGCCCCGGTTTGAGTCCCCACTTCCACTCTTCTCT	T/C	GCGCGCAACCCAAGAAGCTGGCTTTGCCAACACAACATTAGGGCCGTCCCCTTCATTCTATGCCGACCCCGGCCCGGGGCTGGCTTTTTGGGAAGCCCGT
Ca_LG_1:10251562	AGACCGCTCGTGCCCACCCGTTCTGTCTCGCCCTAAATGGAATGACTCTCTTAGTTACGATGCGCCCCGGTTTGAGTCCCCACTTCCACTCTTCTCTGCG	G/C	CGCAACCCAAGAAGCTGGCTTTGCCAACACAACATTAGGGCCGTCCCCTTCATTCTATGCCGACCCCGGCCCGGGGCTGGCTTTTTGGGAAGCCCGTTCC
Ca_LG_1:10251577	ACCCGTTCTGTCTCGCCCTAAATGGAATGACTCTCTTAGTTACGATGCGCCCCGGTTTGAGTCCCCACTTCCACTCTTCTCTGCGCGCAACCCAAGAAGC	C/T	TGGCTTTGCCAACACAACATTAGGGCCGTCCCCTTCATTCTATGCCGACCCCGGCCCGGGGCTGGCTTTTTGGGAAGCCCGTTCCCACCGCGCTCACGGC
Ca_LG_1:10761209	TTCACGTGAACCTTCTGATGCAAAGTTGCTGGTGAGTGATGCTGATGAAGAAAGGAATGCAGCTAAGACAACCCAATCTTCAATCCAGAATACTAATAGC	C/A	AGTGGTATAGATGTTGAAAAGTCCTGCAATGCTGCTACCACTGAAGCAGGCTTTAAAAGTTCATCAATAGAGAATAATTCAGGCAAAGTACATGATGAAT
Ca_LG_1:11501517	CAAGAATGGTCTGAAGAGAAAAGACAAGAATGGCTTTTGTCTGAGTTGAGTGGGAAAAGGCCATTGTTTGGTCCCGACCTTCCTCAAACCGAAGAAATTC	C/A	AAGATGTCTTGGAGACATTTCGTGTCATAGCAGAATTACCATCAGACAACTTTGGAGCCTATATCATATCTATGGCAACTGCACCATCTGATGTGCTTGC
Ca_LG_1:11501518	AAGAATGGTCTGAAGAGAAAAGACAAGAATGGCTTTTGTCTGAGTTGAGTGGGAAAAGGCCATTGTTTGGTCCCGACCTTCCTCAAACCGAAGAAATTCA	A/G	AGATGTCTTGGAGACATTTCGTGTCATAGCAGAATTACCATCAGACAACTTTGGAGCCTATATCATATCTATGGCAACTGCACCATCTGATGTGCTTGCG
Ca_LG_1:11501525	GTCTGAAGAGAAAAGACAAGAATGGCTTTTGTCTGAGTTGAGTGGGAAAAGGCCATTGTTTGGTCCCGACCTTCCTCAAACCGAAGAAATTCAAGATGTC	C/T	TTGGAGACATTTCGTGTCATAGCAGAATTACCATCAGACAACTTTGGAGCCTATATCATATCTATGGCAACTGCACCATCTGATGTGCTTGCGGTCGAAC
Ca_LG_1:11501531	AGAGAAAAGACAAGAATGGCTTTTGTCTGAGTTGAGTGGGAAAAGGCCATTGTTTGGTCCCGACCTTCCTCAAACCGAAGAAATTCAAGATGTCTTGGAG	G/C	ACATTTCGTGTCATAGCAGAATTACCATCAGACAACTTTGGAGCCTATATCATATCTATGGCAACTGCACCATCTGATGTGCTTGCGGTCGAACTTCTTC
Ca_LG_1:11501534	GAAAAGACAAGAATGGCTTTTGTCTGAGTTGAGTGGGAAAAGGCCATTGTTTGGTCCCGACCTTCCTCAAACCGAAGAAATTCAAGATGTCTTGGAGACA	A/G	TTTCGTGTCATAGCAGAATTACCATCAGACAACTTTGGAGCCTATATCATATCTATGGCAACTGCACCATCTGATGTGCTTGCGGTCGAACTTCTTCAAC
Ca_LG_1:11501539	GACAAGAATGGCTTTTGTCTGAGTTGAGTGGGAAAAGGCCATTGTTTGGTCCCGACCTTCCTCAAACCGAAGAAATTCAAGATGTCTTGGAGACATTTCG	G/A	TGTCATAGCAGAATTACCATCAGACAACTTTGGAGCCTATATCATATCTATGGCAACTGCACCATCTGATGTGCTTGCGGTCGAACTTCTTCAACGCGAA
Ca_LG_1:11501553	TTGTCTGAGTTGAGTGGGAAAAGGCCATTGTTTGGTCCCGACCTTCCTCAAACCGAAGAAATTCAAGATGTCTTGGAGACATTTCGTGTCATAGCAGAAT	T/C	TACCATCAGACAACTTTGGAGCCTATATCATATCTATGGCAACTGCACCATCTGATGTGCTTGCGGTCGAACTTCTTCAACGCGAATGCCACATCAAGAA

Positions	-100 bp flanking sequences	SNPs within *kabuli* cultivar	+100 bp flanking sequences
Ca_LG_1:11501576	GCCATTGTTTGGTCCCGACCTTCCTCAAACCGAAGAAATTCAAGATGTCTTGGAGACATTTCGTGTCATAGCAGAATTACCATCAGACAACTTTGGAGCC	C/A	TATATCATATCTATGGCAACTGCACCATCTGATGTGCTTGCGGTCGAACTTCTTCAACGCGAATGCCACATCAAGAATCCGTTAAGAGTTGTACCGTTGT
Ca_LG_1:11501750	GAATCCGTTAAGAGTTGTACCGTTGTTTGAGAAGCTTGATGATCTTGAGGCTGCTCCTGCTGCGTTGGCTCGGCTGTTCTCAGTAGAATGGTACAGAAAC	C/T	CGGATCGATGGTAAGCAAGAAGTCATGATTGGATATTCGGATTCAGGAAAAGACGCCGGAAGGTTTTCTGCAGCATGGCAGCTATATAAGGCTCAAGAGG
Ca_LG_1:11501757	TTAAGAGTTGTACCGTTGTTTGAGAAGCTTGATGATCTTGAGGCTGCTCCTGCTGCGTTGGCTCGGCTGTTCTCAGTAGAATGGTACAGAAACCGGATCG	G/A	ATGGTAAGCAAGAAGTCATGATTGGATATTCGGATTCAGGAAAAGACGCCGGAAGGTTTTCTGCAGCATGGCAGCTATATAAGGCTCAAGAGGAACTCAG
Ca_LG_1:11501759	AAGAGTTGTACCGTTGTTTGAGAAGCTTGATGATCTTGAGGCTGCTCCTGCTGCGTTGGCTCGGCTGTTCTCAGTAGAATGGTACAGAAACCGGATCGAT	T/C	GGTAAGCAAGAAGTCATGATTGGATATTCGGATTCAGGAAAAGACGCCGGAAGGTTTTCTGCAGCATGGCAGCTATATAAGGCTCAAGAGGAACTCAGAA
Ca_LG_1:11501762	AGTTGTACCGTTGTTTGAGAAGCTTGATGATCTTGAGGCTGCTCCTGCTGCGTTGGCTCGGCTGTTCTCAGTAGAATGGTACAGAAACCGGATCGATGGT	T/G	AAGCAAGAAGTCATGATTGGATATTCGGATTCAGGAAAAGACGCCGGAAGGTTTTCTGCAGCATGGCAGCTATATAAGGCTCAAGAGGAACTCAGAAATG
Ca_LG_1:11501783	GCTTGATGATCTTGAGGCTGCTCCTGCTGCGTTGGCTCGGCTGTTCTCAGTAGAATGGTACAGAAACCGGATCGATGGTAAGCAAGAAGTCATGATTGGA	A/G	TATTCGGATTCAGGAAAAGACGCCGGAAGGTTTTCTGCAGCATGGCAGCTATATAAGGCTCAAGAGGAACTCAGAAATGTTGCTCAGAAATTCGGTATTA
Ca_LG_1:11501789	TGATCTTGAGGCTGCTCCTGCTGCGTTGGCTCGGCTGTTCTCAGTAGAATGGTACAGAAACCGGATCGATGGTAAGCAAGAAGTCATGATTGGATATTCG	G/T	GATTCAGGAAAAGACGCCGGAAGGTTTTCTGCAGCATGGCAGCTATATAAGGCTCAAGAGGAACTCAGAAATGTTGCTCAGAAATTCGGTATTAAGCTAA
Ca_LG_1:11501798	GGCTGCTCCTGCTGCGTTGGCTCGGCTGTTCTCAGTAGAATGGTACAGAAACCGGATCGATGGTAAGCAAGAAGTCATGATTGGATATTCGGATTCAGGA	A/T	AAAGACGCCGGAAGGTTTTCTGCAGCATGGCAGCTATATAAGGCTCAAGAGGAACTCAGAAATGTTGCTCAGAAATTCGGTATTAAGCTAACCATGTTCC
Ca_LG_1:11502496	CGTGCGATACCGTGGATCTTTGCATGGACACAAACAAGGTTTCATCTTCCAGTATGGCTAGGATTTGGAGCAGCATTTAAACAAGTTATTGAGAAGGATG	G/A	TTAAGAATCTTCATATGCTGCAAGAGATGTACAATAAATGGCCTTTCTTTAGGGTCACAATTGATTTAGTTGAAATGGTGTTTGCCAAAGGAGACCCTGG
Ca_LG_1:11502500	CGATACCGTGGATCTTTGCATGGACACAAACAAGGTTTCATCTTCCAGTATGGCTAGGATTTGGAGCAGCATTTAAACAAGTTATTGAGAAGGATGTTAA	A/G	GAATCTTCATATGCTGCAAGAGATGTACAATAAATGGCCTTTCTTTAGGGTCACAATTGATTTAGTTGAAATGGTGTTTGCCAAAGGAGACCCTGGAATT
Ca_LG_1:11502501	GATACCGTGGATCTTTGCATGGACACAAACAAGGTTTCATCTTCCAGTATGGCTAGGATTTGGAGCAGCATTTAAACAAGTTATTGAGAAGGATGTTAAG	G/A	AATCTTCATATGCTGCAAGAGATGTACAATAAATGGCCTTTCTTTAGGGTCACAATTGATTTAGTTGAAATGGTGTTTGCCAAAGGAGACCCTGGAATTG
Ca_LG_1:11502510	GATCTTTGCATGGACACAAACAAGGTTTCATCTTCCAGTATGGCTAGGATTTGGAGCAGCATTTAAACAAGTTATTGAGAAGGATGTTAAGAATCTTCAT	T/A	ATGCTGCAAGAGATGTACAATAAATGGCCTTTCTTTAGGGTCACAATTGATTTAGTTGAAATGGTGTTTGCCAAAGGAGACCCTGGAATTGCAGCTTTGA

Positions	-100 bp flanking sequences	SNPs within *kabuli* cultivar	+100 bp flanking sequences
Ca_LG_1:11502516	TGCATGGACACAAACAAGGTTTCATCTTCCAGTATGGCTAGG ATTTGGAGCAGCATTTAAACAAGTTATTGAGAAGGATGTTAAG AATCTTCATATGCTG	G/A	CAAGAGATGTACAATAAATGGCCTTTCTTTAGGGTCACAATT GATTTAGTTGAAATGGTGTTTGCCAAAGGAGACCCTGGAATT GCAGCTTTGAATGATA
Ca_LG_1:11502532	AGGTTTCATCTTCCAGTATGGCTAGGATTTGGAGCAGCATTTA AACAAGTTATTGAGAAGGATGTTAAGAATCTTCATATGCTGCA AGAGATGTACAATA	A/C	AATGGCCTTTCTTTAGGGTCACAATTGATTTAGTTGAAATGG TGTTTGCCAAAGGAGACCCTGGAATTGCAGCTTTGAATGATA GGCTCCTAGTTTCACA
Ca_LG_1:11502555	AGGATTTGGAGCAGCATTTAAACAAGTTATTGAGAAGGATGTT AAGAATCTTCATATGCTGCAAGAGATGTACAATAAATGGCCTT TCTTTAGGGTCACA	A/T	ATTGATTTAGTTGAAATGGTGTTTGCCAAAGGAGACCCTGGA ATTGCAGCTTTGAATGATAGGCTCCTAGTTTCACAGGATCTG TGGCCATTTGGTGAGC
Ca_LG_1:11502567	AGCATTTAAACAAGTTATTGAGAAGGATGTTAAGAATCTTCAT ATGCTGCAAGAGATGTACAATAAATGGCCTTTCTTTAGGGTCA CAATTGATTTAGTT	T/G	GAAATGGTGTTTGCCAAAGGAGACCCTGGAATTGCAGCTTT GAATGATAGGCTCCTAGTTTCACAGGATCTGTGGCCATTTG GTGAGCAGTTGAGGAACA
Ca_LG_1:11502585	TGAGAAGGATGTTAAGAATCTTCATATGCTGCAAGAGATGTAC AATAAATGGCCTTTCTTTAGGGTCACAATTGATTTAGTTGAAAT GGTGTTTGCCAAA	A/G	GGAGACCCTGGAATTGCAGCTTTGAATGATAGGCTCCTAGT TTCACAGGATCTGTGGCCATTTGGTGAGCAGTTGAGGAACA AATATGAAGAAACTAAGA
Ca_LG_1:11503094	AAGGATCCGCGATCCAAACTACAGCGTGACGGTGCGCCCTCA CATTTCAAGAGAGTCCGTGGAAACAAGTAAACCAGCCGATGA ACTTGTAACACTGAAT	T/C	CCAACAAGTGAATTTGGTCCTGGATTGGAAGACACACTCATT CTTACCATGAAGGGTATTGCTGCTGGAATGCAGAACACTGG TTAAATTTTTGGTTACT
Ca_LG_1:11503106	TCCAAACTACAGCGTGACGGTGCGCCCTCACATTTCAAGAGA GTCCGTGGAAACAAGTAAACCAGCCGATGAACTTGTAACACT GAATCCAACAAGTGAA	A/G	TTTGGTCCTGGATTGGAAGACACACTCATTCTTACCATGAAG GGTATTGCTGCTGGAATGCAGAACACTGGTTAAATTTTTGGT TACTTTGTATTTGTTT
Ca_LG_1:11503108	CAAACTACAGCGTGACGGTGCGCCCTCACATTTCAAGAGAGT CCGTGGAAACAAGTAAACCAGCCGATGAACTTGTAACACTGA ATCCAACAAGTGAATT	T/A	TGGTCCTGGATTGGAAGACACACTCATTCTTACCATGAAGG GTATTGCTGCTGGAATGCAGAACACTGGTTAAATTTTTGGTT ACTTTGTATTTGTTTCT
Ca_LG_1:11503111	ACTACAGCGTGACGGTGCGCCCTCACATTTCAAGAGAGTCCG TGGAAACAAGTAAACCAGCCGATGAACTTGTAACACTGAATC CAACAAGTGAATTTGG	G/C	TCCTGGATTGGAAGACACACTCATTCTTACCATGAAGGGTAT TGCTGCTGGAATGCAGAACACTGGTTAAATTTTTGGTTACTT TGTATTTGTTTCTTAA
Ca_LG_1:11503112	CTACAGCGTGACGGTGCGCCCTCACATTTCAAGAGAGTCCGT GGAAACAAGTAAACCAGCCGATGAACTTGTAACACTGAATCC AACAAGTGAATTTGGT	T/A	CCTGGATTGGAAGACACACTCATTCTTACCATGAAGGGTATT GCTGCTGGAATGCAGAACACTGGTTAAATTTTTGGTTACTTT GTATTTGTTTCTTAAT
Ca_LG_1:11503118	CGTGACGGTGCGCCCTCACATTTCAAGAGAGTCCGTGGAAAC AAGTAAACCAGCCGATGAACTTGTAACACTGAATCCAACAAGT GAATTTGGTCCTGGA	A/T	TTGGAAGACACACTCATTCTTACCATGAAGGGTATTGCTGCT GGAATGCAGAACACTGGTTAAATTTTTGGTTACTTTGTATTTG TTTCTTAATGTAAGT
Ca_LG_1:11503127	GCGCCCTCACATTTCAAGAGAGTCCGTGGAAACAAGTAAACC AGCCGATGAACTTGTAACACTGAATCCAACAAGTGAATTTGGT CCTGGATTGGAAGAC	C/T	ACACTCATTCTTACCATGAAGGGTATTGCTGCTGGAATGCAG AACACTGGTTAAATTTTTGGTTACTTTGTATTTGTTTCTTAAT GTAAGTGTGCTAAGA

Positions	-100 bp flanking sequences	SNPs within *kabuli* cultivar	+100 bp flanking sequences
Ca_LG_1:11503130	CCCTCACATTTCAAGAGAGTCCGTGGAAACAAGTAAACCAGCCGATGAACTTGTAACACTGAATCCAACAAGTGAATTTGGTCCTGGATTGGAAGACACA	A/T	CTCATTCTTACCATGAAGGGTATTGCTGCTGGAATGCAGAACACTGGTTAAATTTTTGGTTACTTTGTATTTGTTTCTTAATGTAAGTGTGCTAAGATAT
Ca_LG_1:11503139	TTCAAGAGAGTCCGTGGAAACAAGTAAACCAGCCGATGAACTTGTAACACTGAATCCAACAAGTGAATTTGGTCCTGGATTGGAAGACACACTCATTCTT	T/C	ACCATGAAGGGTATTGCTGCTGGAATGCAGAACACTGGTTAAATTTTTGGTTACTTTGTATTTGTTTCTTAATGTAAGTGTGCTAAGATATCATAAATAC
Ca_LG_1:11808658	AAAGCAACAATTATCAATTCTATTATATTTATTGATTCGCATTATCAAACAAGAACCTCGCAATAAGAACAACTTGCAGCAGTGACAATAAAAATTACCC	C/T	GCAACAATTGCTGAAAATATTGCTCTTTAAATAAAAACATCATAATTAGACAAACTGCAGCAATCACAATATGACCAAATTAAAGCAGACACTTTAAACA
Ca_LG_1:11989386	GATTGCTGTTAAACAGCTCTCTTCGAAATCAAAGCAAGGGAACCGCGAATTCGTTAACGAAATAGGCATGATATCTGCTTTGCAACATCCAAATCTTGTC	C/T	AAGCTTTATGGCTGTTGCATTGAAGGAAACCAATTGCTGCTTGTATATGAATACATGGAGAACAATAGTCTTGCTCGCGCTCTTTTCGGTAAATGCTTCA
Ca_LG_1:12145611	ATTCGAAGCAACTCGTACAAATGAAGGGTTGAGGGATTTTGGAGCTATGTATTCGAAGCATCTCGTAAAAATTGAGGGAAATTGAGGGTTTGGAGATTTG	G/T	GCAGCGGGAATGAAAAGTTTTGGGGTGGGACTATGAAAACTTTTATTTTGGAGAATTTGTTTTGGGGCGGGCTGATCTATACTAACTATTTAACTATGTC
Ca_LG_1:12145635	AGGGTTGAGGGATTTTGGAGCTATGTATTCGAAGCATCTCGTAAAAATTGAGGGAAATTGAGGGTTTGGAGATTTGGCAGCGGGAATGAAAAGTTTTGGG	G/A	GTGGGACTATGAAAACTTTTATTTTGGAGAATTTGTTTTGGGGCGGGCTGATCTATACTAACTATTTAACTATGTCACTAAATACTTATCTCTAAATACC
Ca_LG_1:12145650	TGGAGCTATGTATTCGAAGCATCTCGTAAAAATTGAGGGAAATTGAGGGTTTGGAGATTTGGCAGCGGGAATGAAAAGTTTTGGGGTGGGACTATGAAAA	A/T	CTTTTATTTTGGAGAATTTGTTTTGGGGCGGGCTGATCTATACTAACTATTTAACTATGTCACTAAATACTTATCTCTAAATACCTATCTATACATAAAA
Ca_LG_1:12145651	GGAGCTATGTATTCGAAGCATCTCGTAAAAATTGAGGGAAATTGAGGGTTTGGAGATTTGGCAGCGGGAATGAAAAGTTTTGGGGTGGGACTATGAAAAC	C/T	TTTTATTTTGGAGAATTTGTTTTGGGGCGGGCTGATCTATACTAACTATTTAACTATGTCACTAAATACTTATCTCTAAATACCTATCTATACATAAAAC
Ca_LG_1:12145662	TTCGAAGCATCTCGTAAAAATTGAGGGAAATTGAGGGTTTGGAGATTTGGCAGCGGGAATGAAAAGTTTTGGGGTGGGACTATGAAAACTTTTATTTTGG	G/A	AGAATTTGTTTTGGGGCGGGCTGATCTATACTAACTATTTAACTATGTCACTAAATACTTATCTCTAAATACCTATCTATACATAAAACTTCTTATGTAG
Ca_LG_1:12145663	TCGAAGCATCTCGTAAAAATTGAGGGAAATTGAGGGTTTGGAGATTTGGCAGCGGGAATGAAAAGTTTTGGGGTGGGACTATGAAAACTTTTATTTTGGA	A/G	GAATTTGTTTTGGGGCGGGCTGATCTATACTAACTATTTAACTATGTCACTAAATACTTATCTCTAAATACCTATCTATACATAAAACTTCTTATGTAGT
Ca_LG_1:12145664	CGAAGCATCTCGTAAAAATTGAGGGAAATTGAGGGTTTGGAGATTTGGCAGCGGGAATGAAAAGTTTTGGGGTGGGACTATGAAAACTTTTATTTTGGAG	G/A	AATTTGTTTTGGGGCGGGCTGATCTATACTAACTATTTAACTATGTCACTAAATACTTATCTCTAAATACCTATCTATACATAAAACTTCTTATGTAGTG
Ca_LG_1:12557788	ATTCCTTTAGTTTCTGGCAGTATGAAGTATACAAAGATTGTCATAGTCACAAGCGGCAAAGAATATGAACAATCCAAACTTCATATGGCAGAGCATGGTT	T/C	AAGAAACATTGTGCCACAAGAAACTTGTGCTCTATGCTTCCTTTCCCGCAATTTTCTCAAAGGCTGTTCAACTCTCGTTGAAGCTATACTCGCTGCCACA

Positions	-100 bp flanking sequences	SNPs within *kabuli* cultivar	+100 bp flanking sequences
Ca_LG_1:13171929	TGGGAATGAGTATCTCATTAATCTCATTGATTCACCTGGACACGTTGACTTCTCATCTGAGGTTACTGCTGCACTCCGTATTACTGATGGAGCACTAGTG	G/T	GTGGTGGATTGTGTTGAGGGTGTTTGTGTCCAAACTGAAACTGTTCTGCGACAGGCTCTTGGTGAAAGGATCAGGCCTGTTCTGACTGTTAACAAGATGG
Ca_LG_1:13270277	TTAACTCTAACAGTGGCACTAATGAAAAACAACATATAATAGGAGACAAGTTGAAGTTGTAAATGGCTGCCAGTGCAACTGCAGAAAATATGACAACTAG	G/A	GGTCGATCGAGAGCTTCTATATTCCTCTCTCCCATCAAACTGGAATTAATTCATATGCTGCCCCCACATGCTCTTTAATTATTGCCTTTCGTCAACAGTT
Ca_LG_1:14432048	GTGCAGTGCTGCTATGATAAACCAAATAACGAAGGTTGCTGCTAGCTACAACAGTGAAGTCAAGACAGTCTATCAACAAAGGAAACAAAAAAAAAAACTTC	C/A	ATCAATAAATAATAAATGATTTAATTAGCAACAAAGTAGTAGAAAAAAATAGAGAAAGAGTTGAAATTGAATAACAAAAAAAAATTACCGTTTCCAACACC
Ca_LG_1:14432057	TGCTATGATAAACCAAATAACGAAGGTTGCTGCTAGCTACAACAGTGAAGTCAAGACAGTCTATCAACAAAGGAAACAAAAAAAAAACTTCATCAATAAA	A/G	TAATAAATGATTTAATTAGCAACAAAGTAGTAGAAAAAAATAGAGAAAGAGTTGAAATTGAATAACAAAAAAAAATTACCGTTTCCAACACCAAAAATGGA
Ca_LG_1:14432122	AACAAAGGAAACAAAAAAAAAAACTTCATCAATAAATAATAAATGATTTAATTAGCAACAAAGTAGTAGAAAAAAATAGAGAAAGAGTTGAAATTGAATAA	A/C	CAAAAAAAAATTACCGTTTCCAACACCAAAAATGGAAAAATGCAGTACATTGGTCAACACAGTAGTACAACAATGCAACAAAAATGCAGAAAATGCACCAA
Ca_LG_1:14432126	AAGGAAACAAAAAAAAAACTTCATCAATAAATAATAAATGATTTAATTAGCAACAAAGTAGTAGAAAAAAATAGAGAAAGAGTTGAAATTGAATAACAAA	A/G	AAAAATTACCGTTTCCAACACCAAAAATGGAAAAATGCAGTACATTGGTCAACACAGTAGTACAACAATGCAACAAAAATGCAGAAAATGCACCAAAAAT
Ca_LG_1:14648077	GGAATGAAGAAACCAGACTCAATGGCAACTCAATTCTTGGCCCAAAAATCATTTGCGCAAACGCAATTAGCAGCAACAAAATTCGAAGAGGAATACTTTG	G/A	ACATCTTGGCCCAACTTCTCAAAAGCAAGGTCCAATCCTCAAAACCTTTCAAAGGGCAGCGCTTCGGAAGACACCATTAATTTGGAAAATGACAACGAAG
Ca_LG_1:14648083	AAGAAACCAGACTCAATGGCAACTCAATTCTTGGCCCAAAAATCATTTGCGCAAACGCAATTAGCAGCAACAAAATTCGAAGAGGAATACTTTGACATCT	T/A	TGGCCCAACTTCTCAAAAGCAAGGTCCAATCCTCAAAACCTTTCAAAGGGCAGCGCTTCGGAAGACACCATTAATTTGGAAAATGACAACGAAGACGATT
Ca_LG_1:14705085	AAAAGGCAAGAGAGTACTACACAAGAATATGAGCAGAGAGTATAGAGTGCAACTGCTCCACCTTCTCCCACTGATTTGACCCTTCTTCCATGTGGTCCCC	C/A	TCTTATCCCCAGAATTCACGCAATTTTATATTAAATTGGGGCACTGGCGTGGTGATAATAATTCATGAGGGCCCCAAAATTAGTAGAGGAAAACGTGTTA
Ca_LG_1:14705092	AAGAGAGTACTACACAAGAATATGAGCAGAGAGTATAGAGTGCAACTGCTCCACCTTCTCCCACTGATTTGACCCTTCTTCCATGTGGTCCCCTCTTATC	C/T	CCCAGAATTCACGCAATTTTATATTAAATTGGGGCACTGGCGTGGTGATAATAATTCATGAGGGCCCCAAAATTAGTAGAGGAAAACGTGTTATGTTTCT
Ca_LG_1:14705095	AGAGTACTACACAAGAATATGAGCAGAGAGTATAGAGTGCAACTGCTCCACCTTCTCCCACTGATTTGACCCTTCTTCCATGTGGTCCCCTCTTATCCCC	C/G	AGAATTCACGCAATTTTATATTAAATTGGGGCACTGGCGTGGTGATAATAATTCATGAGGGCCCCAAAATTAGTAGAGGAAAACGTGTTATGTTTCTAGT
Ca_LG_1:14705139	GCTCCACCTTCTCCCACTGATTTGACCCTTCTTCCATGTGGTCCCCTCTTATCCCCAGAATTCACGCAATTTTATATTAAATTGGGGCACTGGCGTGGTG	G/T	ATAATAATTCATGAGGGCCCCAAAATTAGTAGAGGAAAACGTGTTATGTTTCTAGTGAGAAGCCCCTTCACGAACGAATCTGTCAGTGTTCTTTCTCTCT

Positions	-100 bp flanking sequences	SNPs within *kabuli* cultivar	+100 bp flanking sequences
Ca_LG_1:14705149	CTCCCACTGATTTGACCCTTCTTCCATGTGGTCCCCTCTTATCCCCAGAATTCACGCAATTTTATATTAAATTGGGGCACTGGCGTGGTGATAATAATTC	C/T	ATGAGGGCCCCAAAATTAGTAGAGGAAAACGTGTTATGTTTCTAGTGAGAAGCCCCTTCACGAACGAATCTGTCAGTGTTCTTTCTCTCTCCTACATTAT
Ca_LG_1:14705161	TGACCCTTCTTCCATGTGGTCCCCTCTTATCCCCAGAATTCACGCAATTTTATATTAAATTGGGGCACTGGCGTGGTGATAATAATTCATGAGGGCCCCA	A/G	AAATTAGTAGAGGAAAACGTGTTATGTTTCTAGTGAGAAGCCCCTTCACGAACGAATCTGTCAGTGTTCTTTCTCTCTCCTACATTATCCATGACTATAA
Ca_LG_1:14705162	GACCCTTCTTCCATGTGGTCCCCTCTTATCCCCAGAATTCACGCAATTTTATATTAAATTGGGGCACTGGCGTGGTGATAATAATTCATGAGGGCCCCAA	A/C	AATTAGTAGAGGAAAACGTGTTATGTTTCTAGTGAGAAGCCCCTTCACGAACGAATCTGTCAGTGTTCTTTCTCTCTCCTACATTATCCATGACTATAAT
Ca_LG_1:14705183	CCTCTTATCCCCAGAATTCACGCAATTTTATATTAAATTGGGGCACTGGCGTGGTGATAATAATTCATGAGGGCCCCAAAATTAGTAGAGGAAAACGTGT	T/G	TATGTTTCTAGTGAGAAGCCCCTTCACGAACGAATCTGTCAGTGTTCTTTCTCTCTCCTACATTATCCATGACTATAATAATTTTAAAAGTTTTATTTTC
Ca_LG_1:14705184	CTCTTATCCCCAGAATTCACGCAATTTTATATTAAATTGGGGCACTGGCGTGGTGATAATAATTCATGAGGGCCCCAAAATTAGTAGAGGAAAACGTGTT	T/C	ATGTTTCTAGTGAGAAGCCCCTTCACGAACGAATCTGTCAGTGTTCTTTCTCTCTCCTACATTATCCATGACTATAATAATTTTAAAAGTTTTATTTTCA
Ca_LG_1:14705185	TCTTATCCCCAGAATTCACGCAATTTTATATTAAATTGGGGCACTGGCGTGGTGATAATAATTCATGAGGGCCCCAAAATTAGTAGAGGAAAACGTGTTA	A/G	TGTTTCTAGTGAGAAGCCCCTTCACGAACGAATCTGTCAGTGTTCTTTCTCTCTCCTACATTATCCATGACTATAATAATTTTAAAAGTTTTATTTTCAA
Ca_LG_1:14705193	CCAGAATTCACGCAATTTTATATTAAATTGGGGCACTGGCGTGGTGATAATAATTCATGAGGGCCCCAAAATTAGTAGAGGAAAACGTGTTATGTTTCTA	A/G	GTGAGAAGCCCCTTCACGAACGAATCTGTCAGTGTTCTTTCTCTCTCCTACATTATCCATGACTATAATAATTTTAAAAGTTTTATTTTCAAAACTATAT
Ca_LG_1:14705206	AATTTTATATTAAATTGGGGCACTGGCGTGGTGATAATAATTCATGAGGGCCCCAAAATTAGTAGAGGAAAACGTGTTATGTTTCTAGTGAGAAGCCCCT	T/A	TCACGAACGAATCTGTCAGTGTTCTTTCTCTCTCCTACATTATCCATGACTATAATAATTTTAAAAGTTTTATTTTCAAAACTATATATTATTTTCAATC
Ca_LG_1:15365789	TAGAGAGAAGTTAGAAGTGAAAAATGATAAGAGGAGTGGAGTAATATATATAGAATTTGGTTGATCCTTTGTGGCGGCTAAAGCAGCCACAATGAGCAAT	T/C	GGATATATGGCGTTTGTGGCGGCCAAAACGGTCAGAAATGCAGGGATTTGTGGCGGCCTGGGCGGCCACAACTGACAACAGTCATTTGGGTGTTTGTGGC
Ca_LG_1:15365791	GAGAGAAGTTAGAAGTGAAAAATGATAAGAGGAGTGGAGTAATATATATAGAATTTGGTTGATCCTTTGTGGCGGCTAAAGCAGCCACAATGAGCAATGG	G/A	ATATATGGCGTTTGTGGCGGCCAAAACGGTCAGAAATGCAGGGATTTGTGGCGGCCTGGGCGGCCACAACTGACAACAGTCATTTGGGTGTTTGTGGCGG
Ca_LG_1:15561297	CCTCTGCCACTATCTCCTCCAGCTCAATCTCGATAGGCGCCACTGCTCCAGCTGCTGGTGCATTCCACCATTCTAAGAATAGCGGAAGTATCGCCCAACG	G/A	CAGCACTGCTGCAACTACCAGCACAACGACGAAAATGAAGATGTAAATAAGCCACAGCGGAGGTCGATGGAAGTAGCTGTAGTCAGGATTCTTGAATAGC
Ca_LG_1:15582344	ACAAGTGGCATTTGTGGCTGCAAAGGCCGCCACAAAGGAGGCCCAATACAGCTGGTGTTTGTGGCCGCTTAGGCCGTCACAACTGATTGTACCGTTGGAT	T/A	TTTCTGGCGGCAAAGGCCGCCACAAATGGCATTATAGCCGTTGGCTTTTCTGGCCGCTCAGGCCGCCACAAACGTCATGTATCCGTTGGCATTGTGACTG

Positions	-100 bp flanking sequences	SNPs within *kabuli* cultivar	+100 bp flanking sequences
Ca_LG_1:15582347	AGTGGCATTTGTGGCTGCAAAGGCCGCCACAAAGGAGGCCC AATACAGCTGGTGTTTGTGGCCGCTTAGGCCGTCACAACTGA TTGTACCGTTGGATTTT	T/A	CTGGCGGCAAAGGCCGCCACAAATGGCATTATAGCCGTTGG CTTTTCTGGCCGCTCAGGCCGCCACAAACGTCATGTATCCG TTGGCATTGTGACTGTTT
Ca_LG_1:16011338	CTTGGGCCCTCCAATCGGGTCTTGGGCGGTTCCCGTAAAGCC CACAAAAATCTAACCCCAACCCGTTCAAGCCCAATAGCAATCT GCTCTACCTGTTTTG	G/A	CTTCGAGACTTCCCATACTACCAAAGCAAAGAAAAACCACAG ACGCAAGAGGCTGCATATCCAACCACTTCATGATGTAATCAT ATTGAGCCGGGTTCGG
Ca_LG_1:16011355	GGTCTTGGGCGGTTCCCGTAAAGCCCACAAAAATCTAACCCC AACCCGTTCAAGCCCAATAGCAATCTGCTCTACCTGTTTTGCT TCGAGACTTCCCATA	A/G	CTACCAAAGCAAAGAAAAACCACAGACGCAAGAGGCTGCAT ATCCAACCACTTCATGATGTAATCATATTGAGCCGGGTTCGG GTCCCATTGAACCGGTC
Ca_LG_1:16125628	GACTAGAGCTCATCCGTCTACTGAACAAGTGCAGCCAACTGC AGAGCAAGCTGAGTCAGTCGAAGCACATTATGTGGTCTTAAT GAAGCTGCTGGAATTC	C/T	ATGGAGATTCAAGTTGCTCATAATGCAAGAGTATAAGCATCT CTCAAGACTCTCCAGGCATCGCTTGATTCCGTCGTTCAGGAT GTGGCTGCAATACAAG
Ca_LG_1:16125652	ACAAGTGCAGCCAACTGCAGAGCAAGCTGAGTCAGTCGAAG CACATTATGTGGTCTTAATGAAGCTGCTGGAATTCATGGAGAT TCAAGTTGCTCATAAT	T/C	GCAAGAGTATAAGCATCTCTCAAGACTCTCCAGGCATCGCTT GATTCCGTCGTTCAGGATGTGGCTGCAATACAAGAACATCTA GGCCTTAAGATGTCAT
Ca_LG_1:16153461	AAATATAAGAAAAAAATATAAAATCAAATCAACTTTTAATTCTAG AGTGGAAAAAAAAATAGAGAGAGAGGTGGGGGCGGCTGTATA GAAACAGAGTTGCTG	G/C	GAAAAAGAAAGAAAAGAGAGGAAGAAGAAAAAAGAAAGGAA AGGAAAAGCATAGAAAAGAAATAGAGGACTTGCAGTGCGGA TGTTGATCGCGCAATGGC
Ca_LG_1:16153474	AATATAAAATCAAATCAACTTTTAATTCTAGAGTGGAAAAAAAA TAGAGAGAGAGGTGGGGGCGGCTGTATAGAAACAGAGTTGC TGGAAAAAGAAAGAA	A/G	AAGAGAGGAAGAAGAAAAAAGAAAGGAAAGGAAAAGCATAG AAAAGAAATAGAGGACTTGCAGTGCGGATGTTGATCGCGCA ATGGCGGCAGGAGGGATG
Ca_LG_1:16153477	ATAAAATCAAATCAACTTTTAATTCTAGAGTGGAAAAAAAAATAG AGAGAGAGGTGGGGGCGGCTGTATAGAAACAGAGTTGCTGG AAAAAGAAAGAAAAG	G/T	AGAGGAAGAAGAAAAAAGAAAGGAAAGGAAAAGCATAGAAA AGAAATAGAGGACTTGCAGTGCGGATGTTGATCGCGCAATG GCGGCAGGAGGGATGCTC
Ca_LG_1:16153479	AAAATCAAATCAACTTTTAATTCTAGAGTGGAAAAAAAAATAGA GAGAGAGGTGGGGGCGGCTGTATAGAAACAGAGTTGCTGGA AAAAGAAAGAAAAGAG	G/A	AGGAAGAAGAAAAAAGAAAGGAAAGGAAAAGCATAGAAAAG AAATAGAGGACTTGCAGTGCGGATGTTGATCGCGCAATGGC GGCAGGAGGGATGCTCTT
Ca_LG_1:16265674	NNNNNNNNNNNNNNNNNNNNNNNNNNNNNNNNNNNNNNNNN NNNNNNNNNNNNNNNNNNNNNNNNNNNNNNNNNNNNNNNNN AGCCACAATGAGCAACGG	G/T	ATATATGGCTTTTGTGGCGGCCAAAACGGTCAGAAAGGCAT GCGTTTGTGGCGGCTTAGGCGGCCACAAATGGCAGCGGTC CTTTTGGAGTTTGGGGCGC
Ca_LG_1:16265888	AAAGGTAACGACTATAAGCGTTTGTGGCGGCCTGGGCGGCC ACAAATGGCAACGTCACTTTGGGATTTTGTGGCGGCTTTTCCA GCCACAAAAGGTAACG	G/A	ACTCAGGCTTTTGTGGCGGCCTTTGCAGCCACAAAAGGTAA CGTCACTTTTGGATTTTGTGGCGGCCTTTACCCTCACAAAGG GTTGATTTTGTGGCTGA
Ca_LG_1:16759735	TCTAGATGTTGCGCCAATTTCTTGTCTCAACCATCTAACGAGA TAGCTGCACAGATAGTAGCATTTGAGGAGCGAGCAAAAGTGG CAGAAAACGAGGCAC	C/T	GAGAGGCACAAGAGGAGCTAAGGAAAGTAGAGCAACGTCG ACAAGAGGAAGTTCAACAAACAGAGCAACACACTACAGAGC TATAGATGCAGCTAGCAAC

**Positions**	**-100 bp flanking sequences**	**SNPs within *kabuli* cultivar**	**+100 bp flanking sequences**
Ca_LG_1:16759738	AGATGTTGCGCCAATTTCTTGTCTCAACCATCTAACGAGATAG CTGCACAGATAGTAGCATTTGAGGAGCGAGCAAAAGTGGCAG AAAACGAGGCACGAG	G/A	AGGCACAAGAGGAGCTAAGGAAAGTAGAGCAACGTCGACAA GAGGAAGTTCAACAAACAGAGCAACACACTACAGAGCTATA GATGCAGCTAGCAACATT
Ca_LG_1:16759763	AACCATCTAACGAGATAGCTGCACAGATAGTAGCATTTGAGG AGCGAGCAAAAGTGGCAGAAAACGAGGCACGAGAGGCACAA GAGGAGCTAAGGAAAGT	T/A	AGAGCAACGTCGACAAGAGGAAGTTCAACAAACAGAGCAAC ACACTACAGAGCTATAGATGCAGCTAGCAACATTGGCAAAA CGTGTTGCTTCCATACAG
Ca_LG_1:16759803	GGAGCGAGCAAAAGTGGCAGAAAACGAGGCACGAGAGGCAC AAGAGGAGCTAAGGAAAGTAGAGCAACGTCGACAAGAGGAA GTTCAACAAACAGAGCAA	A/G	CACACTACAGAGCTATAGATGCAGCTAGCAACATTGGCAAA ACGTGTTGCTTCCATACAGGCCGAGTCATCTCGTCGTCGTC GTCATCCAGATTATGACG
Ca_LG_1:16759805	AGCGAGCAAAAGTGGCAGAAAACGAGGCACGAGAGGCACAA GAGGAGCTAAGGAAAGTAGAGCAACGTCGACAAGAGGAAGT TCAACAAACAGAGCAACA	A/G	CACTACAGAGCTATAGATGCAGCTAGCAACATTGGCAAAAC GTGTTGCTTCCATACAGGCCGAGTCATCTCGTCGTCGTCGT CATCCAGATTATGACGAG
Ca_LG_1:17262877	AGATACTATTATTGTGCCACAGAAAAAGAAAGACAGATAGGCA TGCAGCTATATCCAATGCTTAAGCATATAATAGCAATATTTGA AAGGGAAAAAATAG	G/A	TTGGGATTATATTTTGGGAAGGACAAAGCACTCACAGTTGAA AGTTCAGCAGATAATTGCTCTTCGTTGGCTGCAGCAGCATCT TTTGCTGATATTAATT
Ca_LG_1:18568388	ATATAAGAGGATCAAATTATAATTAACTAAAAGATTAAAAATAA AATAAGGTTAAGTTAAGTTGCAGCCAGTAGTGACAGTGAAGT CCACACAATTCAGT	T/A	TTCAACAACAATGCTGTTGTCTCTCTCAGTTTCAGTTTCAAAG TTTTTAACCCTAACAAGGTATGCTTTCTCTCTTTCTTCCATTC CCAATTTTCTTCCA
Ca_LG_1:18568393	AGAGGATCAAATTATAATTAACTAAAAGATTAAAAATAAAATAA GGTTAAGTTAAGTTGCAGCCAGTAGTGACAGTGAAGTCCACA CAATTCAGTTTCAA	A/G	CAACAATGCTGTTGTCTCTCTCAGTTTCAGTTTCAAAGTTTTT AACCCTAACAAGGTATGCTTTCTCTCTTTCTTCCATTCCCAAT TTTCTTCCAATTTC
Ca_LG_1:18568402	AATTATAATTAACTAAAAGATTAAAAATAAAATAAGGTTAAGTT AAGTTGCAGCCAGTAGTGACAGTGAAGTCCACACAATTCAGT TTCAACAACAATGC	C/T	TGTTGTCTCTCTCAGTTTCAGTTTCAAAGTTTTTAACCCTAAC AAGGTATGCTTTCTCTCTTTCTTCCATTCCCAATTTTCTTCCA ATTTCTTCTTCAAA
Ca_LG_1:18568407	TAATTAACTAAAAGATTAAAAATAAAATAAGGTTAAGTTAAGTT GCAGCCAGTAGTGACAGTGAAGTCCACACAATTCAGTTTCAA CAACAATGCTGTTG	G/T	TCTCTCTCAGTTTCAGTTTCAAAGTTTTTAACCCTAACAAGGT ATGCTTTCTCTCTTTCTTCCATTCCCAATTTTCTTCCAATTTCT TCTTCAAACACTT
Ca_LG_1:18568411	TAACTAAAAGATTAAAAATAAAATAAGGTTAAGTTAAGTTGCA GCCAGTAGTGACAGTGAAGTCCACACAATTCAGTTTCAACAA CAATGCTGTTGTCTC	C/T	TCTCAGTTTCAGTTTCAAAGTTTTTAACCCTAACAAGGTATGC TTTCTCTCTTTCTTCCATTCCCAATTTTCTTCCAATTTCTTCTT CAAACACTTTCCT
Ca_LG_1:18568434	TAAGGTTAAGTTAAGTTGCAGCCAGTAGTGACAGTGAAGTCC ACACAATTCAGTTTCAACAACAATGCTGTTGTCTCTCTCAGTTT CAGTTTCAAAGTTT	T/A	TTAACCCTAACAAGGTATGCTTTCTCTCTTTCTTCCATTCCCA ATTTTCTTCCAATTTCTTCTTCAAACACTTTCCTTCTTTCATTC TCTTCTCATTCTC
Ca_LG_1:18689505	GATATAACCACCATTGACTAAATTGCTACTGTGGGATTCTTAA AGTATGATTCCTCGTTCTCATGTCATTCCTCCTGTAGCCTGTA TTATCACTCCATCG	G/A	TTTACAAAGTAAAAACCTTAAAACACATAAACTGTTCAAATAT AGACCACAAGGACTGCCCTAAAAATCAACTAATTTAATATTA ATCAAATAAATATAT

Positions	-100 bp flanking sequences	SNPs within *kabuli* cultivar	+100 bp flanking sequences
Ca_LG_1:18689527	TTGCTACTGTGGGATTCTTAAAGTATGATTCCTCGTTCTCATGTCATTCCTCCTGTAGCCTGTATTATCACTCCATCGTTTACAAAGTAAAAACCTTAAA	A/C	ACACATAAACTGTTCAAATATAGACCACAAGGACTGCCCTAAAAATCAACTAATTTAATATTAATCAAATAAATATATATAATTATATGTTACAAATTTT
Ca_LG_1:18691570	ATGCGACCACATTAAGGGAGGCGGCCAGGCAGTTCAGAACAAACTCAATGGCTAGGCAACGAACAAAAAGAAGCGAAAACGAAGAAGGACGAAGAAACGA	A/G	CGAGCAGTGACAACGGCGCGCAGTGACGACGGCGAGCAGTGGCGACGGCGAGCAGCGACCACGACGAGCAGCGACCACGGAGAGCAAGTGACCAGGACGA
Ca_LG_1:18898367	GTCGCTCCTGTAGCTGCTCTGGAGTCTGGTTTTGAGAAAATACACCATTTGGCTAGCCACTGGTGCAGACTTCATCCCATTTTGTTGCTGCTCTTGAGTG	G/C	TGGTAGTGAGAAGAATTCGCCATTTCGCTAGCACCTGGTAGAGATTCCATCACATGTTGTTGCTGCTGTTGAGGCTCTTGAGTTGGGTTTTGAGAAGAAT
Ca_LG_1:18898372	TCCTGTAGCTGCTCTGGAGTCTGGTTTTGAGAAAATACACCATTTGGCTAGCCACTGGTGCAGACTTCATCCCATTTTGTTGCTGCTCTTGAGTGTGGTA	A/G	GTGAGAAGAATTCGCCATTTCGCTAGCACCTGGTAGAGATTCCATCACATGTTGTTGCTGCTGTTGAGGCTCTTGAGTTGGGTTTTGAGAAGAATACACC
Ca_LG_1:18898393	TGGTTTTGAGAAAATACACCATTTGGCTAGCCACTGGTGCAGACTTCATCCCATTTTGTTGCTGCTCTTGAGTGTGGTAGTGAGAAGAATTCGCCATTTC	C/G	GCTAGCACCTGGTAGAGATTCCATCACATGTTGTTGCTGCTGTTGAGGCTCTTGAGTTGGGTTTTGAGAAGAATACACCATTTGGCTAACACCTGGTGCA
Ca_LG_1:18898399	TGAGAAAATACACCATTTGGCTAGCCACTGGTGCAGACTTCATCCCATTTTGTTGCTGCTCTTGAGTGTGGTAGTGAGAAGAATTCGCCATTTCGCTAGC	C/T	ACCTGGTAGAGATTCCATCACATGTTGTTGCTGCTGTTGAGGCTCTTGAGTTGGGTTTTGAGAAGAATACACCATTTGGCTAACACCTGGTGCAAATTTC
Ca_LG_1:18898414	TTTGGCTAGCCACTGGTGCAGACTTCATCCCATTTTGTTGCTGCTCTTGAGTGTGGTAGTGAGAAGAATTCGCCATTTCGCTAGCACCTGGTAGAGATTC	C/T	CATCACATGTTGTTGCTGCTGTTGAGGCTCTTGAGTTGGGTTTTGAGAAGAATACACCATTTGGCTAACACCTGGTGCAAATTTCATCCCATTTTGTTGC
Ca_LG_1:18898482	TTCGCCATTTCGCTAGCACCTGGTAGAGATTCCATCACATGTTGTTGCTGCTGTTGAGGCTCTTGAGTTGGGTTTTGAGAAGAATACACCATTTGGCTAA	A/G	CACCTGGTGCAAATTTCATCCCATTTTGTTGCTGCAGTGGCTGCTCTTGAGTCTGGTAGTGAGAAGAATTCACCATTTGGCTAGCACTTGGTACAGATTC
Ca_LG_1:18898485	GCCATTTCGCTAGCACCTGGTAGAGATTCCATCACATGTTGTTGCTGCTGTTGAGGCTCTTGAGTTGGGTTTTGAGAAGAATACACCATTTGGCTAACAC	C/A	CTGGTGCAAATTTCATCCCATTTTGTTGCTGCAGTGGCTGCTCTTGAGTCTGGTAGTGAGAAGAATTCACCATTTGGCTAGCACTTGGTACAGATTCCAT
Ca_LG_1:18898492	CGCTAGCACCTGGTAGAGATTCCATCACATGTTGTTGCTGCTGTTGAGGCTCTTGAGTTGGGTTTTGAGAAGAATACACCATTTGGCTAACACCTGGTGC	C/T	AAATTTCATCCCATTTTGTTGCTGCAGTGGCTGCTCTTGAGTCTGGTAGTGAGAAGAATTCACCATTTGGCTAGCACTTGGTACAGATTCCATCACATGT
Ca_LG_1:18898513	CCATCACATGTTGTTGCTGCTGTTGAGGCTCTTGAGTTGGGTTTTGAGAAGAATACACCATTTGGCTAACACCTGGTGCAAATTTCATCCCATTTTGTTG	G/T	CTGCAGTGGCTGCTCTTGAGTCTGGTAGTGAGAAGAATTCACCATTTGGCTAGCACTTGGTACAGATTCCATCACATGTTGCTGCTCTTGAGTTGGGTGG
Ca_LG_1:18898521	TGTTGTTGCTGCTGTTGAGGCTCTTGAGTTGGGTTTTGAGAAGAATACACCATTTGGCTAACACCTGGTGCAAATTTCATCCCATTTTGTTGCTGCAGTG	G/T	GCTGCTCTTGAGTCTGGTAGTGAGAAGAATTCACCATTTGGCTAGCACTTGGTACAGATTCCATCACATGTTGCTGCTCTTGAGTTGGGTGGTGAGAAGA

Positions	-100 bp flanking sequences	SNPs within *kabuli* cultivar	+100 bp flanking sequences
Ca_LG_1:18898845	TGTTGCTGCTCTTGAGTTGGGTGGTGAGAAGAATACGCCGTTTTGCCAACACCTGCTGCAAATTTCATCACATTTTGTTGCTGCAGTTGCATCTCTTGAG	G/A	TCTGGTAGTGAGAAGAATTTACCATTTGGCTAGCACCTGGTACAGATTCCATCACATGCTTCTCTTGAGTATGGTAGTGAGAAGAATTCACCATTTGGCT
Ca_LG_1:18898866	TGGTGAGAAGAATACGCCGTTTTGCCAACACCTGCTGCAAATTTCATCACATTTTGTTGCTGCAGTTGCATCTCTTGAGTCTGGTAGTGAGAAGAATTTA	A/G	CCATTTGGCTAGCACCTGGTACAGATTCCATCACATGCTTCTCTTGAGTATGGTAGTGAGAAGAATTCACCATTTGGCTAGCACTGGGTACAGATTCCGT
Ca_LG_1:18898888	TGCCAACACCTGCTGCAAATTTCATCACATTTTGTTGCTGCAGTTGCATCTCTTGAGTCTGGTAGTGAGAAGAATTTACCATTTGGCTAGCACCTGGTAC	C/A	AGATTCCATCACATGCTTCTCTTGAGTATGGTAGTGAGAAGAATTCACCATTTGGCTAGCACTGGGTACAGATTCCGTCACATTTTGTTGCTGTTCTTGA
Ca_LG_1:18996048	GGCAGGCAAGAGACAACCTGGCGAACTGAAACATCTTAGTAGCCAGAGGAAAAGAAAGCAAAAGCGATTCCCGTAGTAGCGGCGAGCGAAATGGGAACAA	A/G	CCTAAACCGTGAAAACGGGAATGTGGGAGAGCAATACAAGCGTCGTGCTGCTAGGCGAAGCAATCGAATGTTGCACCCTAGATGGTGAAAGTCCAGTAGC
Ca_LG_1:18996068	GCGAACTGAAACATCTTAGTAGCCAGAGGAAAAGAAAGCAAAAGCGATTCCCGTAGTAGCGGCGAGCGAAATGGGAACAACCTAAACCGTGAAAACGGGA	A/G	ATGTGGGAGAGCAATACAAGCGTCGTGCTGCTAGGCGAAGCAATCGAATGTTGCACCCTAGATGGTGAAAGTCCAGTAGCCGAAAGCATCACTAGCTTAT
Ca_LG_1:18996069	CGAACTGAAACATCTTAGTAGCCAGAGGAAAAGAAAGCAAAAGCGATTCCCGTAGTAGCGGCGAGCGAAATGGGAACAACCTAAACCGTGAAAACGGGAA	A/T	TGTGGGAGAGCAATACAAGCGTCGTGCTGCTAGGCGAAGCAATCGAATGTTGCACCCTAGATGGTGAAAGTCCAGTAGCCGAAAGCATCACTAGCTTATG
Ca_LG_1:18996157	TGAAAACGGGAATGTGGGAGAGCAATACAAGCGTCGTGCTGCTAGGCGAAGCAATCGAATGTTGCACCCTAGATGGTGAAAGTCCAGTAGCCGAAAGCAT	T/G	CACTAGCTTATGCTCTAACCCGAGTAGCAAGGACCACCTTGCAAGGCTAAATACTCCTGGGTGACCGATAGTGAAGTAGTACTGTTAGGGAAAGGTGAAA
Ca_LG_1:18996779	GAGGCAAACTCTGAATACTAGATATGACCCCAAAATAAGAAGGGTCAAGGTCGGCCAGTGAGACGATGGGGGATAAGCTTCATCGTCGAGAGGGAAACAA	A/G	CCCAGATATCCAGCTAAGGCCCATAAATGATCGCTAAGTGATAAAGGAGGTAGGAGTGCAGAGACAACCAGGAGGTTTGCCTAGAAGCAGCCACCCTTCA
Ca_LG_1:18996787	CTCTGAATACTAGATATGACCCCAAAATAAGAAGGGTCAAGGTCGGCCAGTGAGACGATGGGGGATAAGCTTCATCGTCGAGAGGGAAACAACCCAGATA	A/C	TCCAGCTAAGGCCCATAAATGATCGCTAAGTGATAAAGGAGGTAGGAGTGCAGAGACAACCAGGAGGTTTGCCTAGAAGCAGCCACCCTTCAAAGAGTGC
Ca_LG_1:18996788	TCTGAATACTAGATATGACCCCAAAATAAGAAGGGTCAAGGTCGGCCAGTGAGACGATGGGGGATAAGCTTCATCGTCGAGAGGGAAACAACCCAGATAT	T/A	CCAGCTAAGGCCCATAAATGATCGCTAAGTGATAAAGGAGGTAGGAGTGCAGAGACAACCAGGAGGTTTGCCTAGAAGCAGCCACCCTTCAAAGAGTGCG
Ca_LG_1:18996802	ATGACCCCAAAATAAGAAGGGTCAAGGTCGGCCAGTGAGACGATGGGGGATAAGCTTCATCGTCGAGAGGGAAACAACCCAGATATCCAGCTAAGGCCCA	A/C	TAAATGATCGCTAAGTGATAAAGGAGGTAGGAGTGCAGAGACAACCAGGAGGTTTGCCTAGAAGCAGCCACCCTTCAAAGAGTGCGTAATAGCTCACTGA
Ca_LG_1:18996846	GGGGGATAAGCTTCATCGTCGAGAGGGAAACAACCCAGATATCCAGCTAAGGCCCATAAATGATCGCTAAGTGATAAAGGAGGTAGGAGTGCAGAGACAA	A/G	CCAGGAGGTTTGCCTAGAAGCAGCCACCCTTCAAAGAGTGCGTAATAGCTCACTGATCGAGCGCTCTTGCGCCGAAGATGAACGGGGCTAAGCGATCTGC

**Positions**	**-100 bp flanking sequences**	**SNPs within *kabuli* cultivar**	**+100 bp flanking sequences**
Ca_LG_1:19592161	CCTTACCATTACTAAAGTTATATTTAGAATTATGCTTCACAAACTCTTGTCTCACTCATTTCACACCTGCTGTGCGAGCACGGTTTCTCCCTCTCTGAAC	C/A	GTCGTTTCACCGATCCGACCGTTGAAAACGTGGATCTGAATGTTGAGAACTTGCTGTCCAAAAATCAACACGATCCAACGGTTAACGAATGCGCAATCGA
Ca_LG_1:19592206	CTTGTCTCACTCATTTCACACCTGCTGTGCGAGCACGGTTTCTCCCTCTCTGAACGTCGTTTCACCGATCCGACCGTTGAAAACGTGGATCTGAATGTTG	G/T	AGAACTTGCTGTCCAAAAATCAACACGATCCAACGGTTAACGAATGCGCAATCGATGTTTTTCTAATACTGATTTAACAAATTCGGGAATGAGGTTACTC
Ca_LG_1:19744738	CTGCAACAAGTCCTCTGCATCACTTTTTGGGTCATGTGCAGCCTCCACATCATTAAATTTATCTTAACCATCATGTTCTGGTTTAACCAGCAGCACATCC	C/T	TCTCCCTCACTTTCTGTTGTTTTTCCATTTCTTCTTTTGATCACTATGGTGCAAGCTCCCTCAGCTTCCAAAGTTCTATCATCAGCAAGCCTGGTCTTGC
Ca_LG_1:20192575	ATTTTCAAAGTGTCAAAAATGCATAAACAATGTTTAAAAATAATATTTTTGGCAAAATACAATGTTGTATTCGAATACAGCATGTTGTAGCCGAATACAC	C/G	GTGATAAGTTAGTAGCTAAAATTAAACATATGTATTCGACTACGAGATGGTGCAGCTGAATACAAGTGCTAAGTTAGTAGCAAAAACTGAACATCTGTAT
Ca_LG_1:20192585	TGTCAAAAATGCATAAACAATGTTTAAAAATAATATTTTTGGCAAAATACAATGTTGTATTCGAATACAGCATGTTGTAGCCGAATACACGTGATAAGTT	T/C	AGTAGCTAAAATTAAACATATGTATTCGACTACGAGATGGTGCAGCTGAATACAAGTGCTAAGTTAGTAGCAAAAACTGAACATCTGTATTTGACTACGA
Ca_LG_1:20192602	CAATGTTTAAAAATAATATTTTTGGCAAAATACAATGTTGTATTCGAATACAGCATGTTGTAGCCGAATACACGTGATAAGTTAGTAGCTAAAATTAAAC	C/T	ATATGTATTCGACTACGAGATGGTGCAGCTGAATACAAGTGCTAAGTTAGTAGCAAAAACTGAACATCTGTATTTGACTACGAGATGGTGTAGTCGAATA
Ca_LG_1:20254544	GCGTGAATGCTTGTTGCTTCCCCAATTTATTTTTACCTGTATGTCAAGACACGACCCATATCGAGCAAGGCATGGCTGACTTATTTTAGGAGCAGCAACT	T/A	GATTTTCCCCGATCCAGTTTTTGAGCTGCAACTTTGGCAACTTTGTGCGGGTGGTTTATTGGATTGAATCTGAAAAAATGAGGTTAATTAGTTTTTTAGT
Ca_LG_1:20380006	TTTCCTTGTGTGTTTTTTTCAATGACTTTTCTTTTGTTCTTGATATGTCCATTCATCAATCACCTGTTTGAAGTTAGTGAATGCAGCGTCATCTTCAGCG	G/A	GTAAACGGGATGCCAACAGATGATTTCATATGCTCCAACAAATCCTGCTTGGCAGCAAGAAGCTCATGGCACTGCTCTTTCAAGGTTTAGGTCACACCAA
Ca_LG_1:20481355	ATAGGCTGCATTCGAAACAGTTCATATTCTATAGGCTATAATATATTCTATAGGCTGCATTCGAAACAGTTCATATTCGAAACAGTTCATCTTGAGAAAT	T/A	TTTTTTCCACAAGTCCATAATATATTCTATAGGCTGCATTCGAAACAGTTCATATTCTTGAGATAATGTATTTAGTTTAGAACATTTTACCTCAATAATT
Ca_LG_1:21007926	ATTACAGCTGCCACTGATTCACTCACTAGACTAGGTATAAATACTGAAATTTGTGTTGTGCTTAGTTTTTCAGTTAATGGAAATCAGTTCACATTTTCTC	C/T	CGATTTTATCCCAGTTCTCAATGACTTCGTTTTCTTAAAGCTGCCTTCTTCATGGTGTTTTGTGCTAGGAATTTTGACTCCCTTTACTGGTGAACTGCTC
Ca_LG_1:21469407	TTTTTCTTCGTGGTAATTTGTTATGGTGATTTTCCTTTGTGTACCAAAATCGGCCATCTAAAATTGCAGCGAATCAAGCAACGAAATCACGCACCATCTC	C/T	CACTCAACTGAGAATTTTCGATTGAAATAATTGCGGTTTGCGAGAACAGAGATTTAGGCTGCATACCCTTACGTTGATCCAAAAAAACAAATACAGAATC
Ca_LG_1:21705993	CACAAACTGATTATTTTATGTCATGTCATCCTTTGTGGTGGCCTAAACCCTCACAAAAGACAACTTTTGTGGCGGCTTTGGCCCTCACAAACGCTAACTG	G/A	TTTTAAACTTCTTAGTCTTTTGTGGCGGACTTTTCTGTCACAGTTTTGTGAAGGCTTAAGACGTCACAAATGGTAACAAAAAAAAACTGAATTTTGTGGT

Positions	-100 bp flanking sequences	SNPs within *kabuli* cultivar	+100 bp flanking sequences
Ca_LG_1:21706004	TATTTTATGTCATGTCATCCTTTGTGGTGGCCTAAACCCTCACAAAAGACAACTTTTGTGGCGGCTTTGGCCCTCACAAACGCTAACTGTTTTAAACTTC	C/T	TTAGTCTTTTGTGGCGGACTTTTCTGTCACAGTTTTGTGAAGGCTTAAGACGTCACAAATGGTAACAAAAAAAAACTGAATTTTGTGGTTGTTTTGGCCG
Ca_LG_1:22441075	GCCACTTACAATACCCCGTCGCGTAATTAAGTCGTCTGCAAAAGATTCTACCTGCCGCTCAATAGGAATTGCGTTTCAAGGTGTCCCGCAAGGCTCATCT	T/C	GCCTTACGAGGTCCACCGACGACACGTGCCTCTGGGGGGCCAAGGCCCCCTACTGCTGACTAGCGATGCGTGTGCCAGTCATTCGCTTGTTGACCACGTC
Ca_LG_1:22441093	TCGCGTAATTAAGTCGTCTGCAAAAGATTCTACCTGCCGCTCAATAGGAATTGCGTTTCAAGGTGTCCCGCAAGGCTCATCTGCCTTACGAGGTCCACCG	G/A	ACGACACGTGCCTCTGGGGGGCCAAGGCCCCCTACTGCTGACTAGCGATGCGTGTGCCAGTCATTCGCTTGTTGACCACGTCTACGACTAAGACGTGGAC
Ca_LG_1:24118226	CCATCACAAATACAAAAATTGAAATTGGTCTTTATTTGTAGCTGAAAAAGCTCTCACAAATTATTAAAAATTTAGGTGGAATTTATGGCTGGCTAAGCCT	T/C	TCACAAAATCACAACGTTGTGATTTTGTAAGGGCTTAAGCAGCCACAAATTCCGCCTAATTTTTTAAGATTTTTGTGAGGGTTTTTGCAGCTACAAAGTC
Ca_LG_1:24118255	CTTTATTTGTAGCTGAAAAAGCTCTCACAAATTATTAAAAATTTAGGTGGAATTTATGGCTGGCTAAGCCTTCACAAAATCACAACGTTGTGATTTTGTA	A/G	AGGGCTTAAGCAGCCACAAATTCCGCCTAATTTTTTAAGATTTTTGTGAGGGTTTTTGCAGCTACAAAGTCTAAAATTTGTGAGGGTCAAGTCCGTCACA
Ca_LG_1:24118273	AAGCTCTCACAAATTATTAAAAATTTAGGTGGAATTTATGGCTGGCTAAGCCTTCACAAAATCACAACGTTGTGATTTTGTAAGGGCTTAAGCAGCCACA	A/T	AATTCCGCCTAATTTTTTAAGATTTTTGTGAGGGTTTTTGCAGCTACAAAGTCTAAAATTTGTGAGGGTCAAGTCCGTCACAAAAGCCTGAAATGACATG
Ca_LG_1:24118280	CACAAATTATTAAAAATTTAGGTGGAATTTATGGCTGGCTAAGCCTTCACAAAATCACAACGTTGTGATTTTGTAAGGGCTTAAGCAGCCACAAATTCCG	G/A	CCTAATTTTTTAAGATTTTTGTGAGGGTTTTTGCAGCTACAAAGTCTAAAATTTGTGAGGGTCAAGTCCGTCACAAAAGCCTGAAATGACATGTCAATTT
Ca_LG_1:24118285	ATTATTAAAAATTTAGGTGGAATTTATGGCTGGCTAAGCCTTCACAAAATCACAACGTTGTGATTTTGTAAGGGCTTAAGCAGCCACAAATTCCGCCTAA	A/C	TTTTTTAAGATTTTTGTGAGGGTTTTTGCAGCTACAAAGTCTAAAATTTGTGAGGGTCAAGTCCGTCACAAAAGCCTGAAATGACATGTCAATTTGTGGC
Ca_LG_1:24464785	CATATATTTGGTCAATATGGGATTTTAACACATCCCTTCAAGTCCAGCACAATGAAGGTGCCGAGGCCAAAACGAAGGCAGCTGCAATGCAGGACGATAC	C/G	GGTTTCCAACAACAGTTTTAAAAAGAAAAAAGTAAACATAGGAAAAATCTCACCGTCAAATTTTCGAAAGCACTTTTGAAGCGCTGCAGAAGAAGTTGCA
Ca_LG_1:24728837	ATTTCCCTCTATAGACTTGGTTAGAACAAATAGAGTTTTACAACTAGTTCACATGGACCTTTTTGGACCTGCCCAAGTTAAGAGTTTAGGTGGAAACCTG	G/T	TATGGATATGTGCTGGTTGATGATTACTCAAGGTTCACATGGACTTATTTTCTGGCTCATAAGAGTGATACATTCTCAATTTTTAAAAAATTTGCTGCTT
Ca_LG_1:24728861	AACAAATAGAGTTTTACAACTAGTTCACATGGACCTTTTTGGACCTGCCCAAGTTAAGAGTTTAGGTGGAAACCTGTATGGATATGTGCTGGTTGATGAT	T/C	TACTCAAGGTTCACATGGACTTATTTTCTGGCTCATAAGAGTGATACATTCTCAATTTTTAAAAAATTTGCTGCTTTAGTTCAAAATGAGAATGATCAGA
Ca_LG_1:24838360	ATTGATCACTTCTGCCATGCTCGTTGGCCTTCGGTTTACATACACGGTAAAACCGGGCGTTTCTTCCTTAGCTTCTTGTTTACCAATTGCCATTTCTCTG	G/A	TGGCTTGATTCAATATGACCTGCAGCTTGAGATTTTGATACTTCAAAACTAGCACTGCCATGCACTTGCTGTGAATCTTTGGCATCGTGCTCGACTTCGG

Positions	-100 bp flanking sequences	SNPs within *kabuli* cultivar	+100 bp flanking sequences
Ca_LG_1:25584206	CTCAGGAGAATGATCCTTTTTCATTCTTCCTTAATAGTCAAATTACTTTCATGTAAGGAAAAAAATAGAGGAAATATTGTTGGCTGCAGCATAACTTAAG	G/A	TATTGGAAGAAAACACTCACCGAGATTTGGACCGTTCTTGAGACATATTTCATGGATCCTCAATCGTTCTCTTTGAACACCACATAGCCCCTGAAGAAGA
Ca_LG_1:26421258	GTGATTTTGTGAGGGCTTAGGCAACCACGAAATCCAGTTAAATTTTTAAGAATTTTGTGAGGGCTTTTACAGCCACAAAAAGTAAATTTTGTGAGGGGCA	A/T	GATCCGTCACAAATGACTGAAATGACATGGCAATTTGTGGCTACATAGGCCGCCACAAAAGCCTATGACCGTTACCTTTTGTGGCTGCAAAAGCCGCCAC
Ca_LG_1:26421264	TTGTGAGGGCTTAGGCAACCACGAAATCCAGTTAAATTTTTAAGAATTTTGTGAGGGCTTTTACAGCCACAAAAAGTAAATTTTGTGAGGGGCAGATCCG	G/A	TCACAAATGACTGAAATGACATGGCAATTTGTGGCTACATAGGCCGCCACAAAAGCCTATGACCGTTACCTTTTGTGGCTGCAAAAGCCGCCACAAAGGA
Ca_LG_1:26421300	TTTTTAAGAATTTTGTGAGGGCTTTTACAGCCACAAAAAGTAAATTTTGTGAGGGGCAGATCCGTCACAAATGACTGAAATGACATGGCAATTTGTGGCT	T/C	ACATAGGCCGCCACAAAAGCCTATGACCGTTACCTTTTGTGGCTGCAAAAGCCGCCACAAAGGATTAAATATGTAACTGATTTTCTGGCCGCCCAGGCCG
Ca_LG_1:26421301	TTTTAAGAATTTTGTGAGGGCTTTTACAGCCACAAAAAGTAAATTTTGTGAGGGGCAGATCCGTCACAAATGACTGAAATGACATGGCAATTTGTGGCTA	A/G	CATAGGCCGCCACAAAAGCCTATGACCGTTACCTTTTGTGGCTGCAAAAGCCGCCACAAAGGATTAAATATGTAACTGATTTTCTGGCCGCCCAGGCCGT
Ca_LG_1:26421302	TTTAAGAATTTTGTGAGGGCTTTTACAGCCACAAAAAGTAAATTTTGTGAGGGGCAGATCCGTCACAAATGACTGAAATGACATGGCAATTTGTGGCTAC	C/T	ATAGGCCGCCACAAAAGCCTATGACCGTTACCTTTTGTGGCTGCAAAAGCCGCCACAAAGGATTAAATATGTAACTGATTTTCTGGCCGCCCAGGCCGTC
Ca_LG_1:26862043	AGAGGGCTTTTTTAAAAACAAATGACATACATCTTTTGGGGTATCTTTCAAGTAGAGTCGGTCGCGGTATTTTTAGAGCAGCTTCATCGAAGCTGCCCTA	A/G	AGCATAGGTTTCTCCAATAGATGCAATAGGCAACAATAGAGGGAGAGTTCACAACACCCTTTTATTTTAAGTTGAAGTCATATATAAATATCCTTTATTT
Ca_LG_1:26862061	CAAATGACATACATCTTTTGGGGTATCTTTCAAGTAGAGTCGGTCGCGGTATTTTTAGAGCAGCTTCATCGAAGCTGCCCTAAGCATAGGTTTCTCCAAT	T/C	AGATGCAATAGGCAACAATAGAGGGAGAGTTCACAACACCCTTTTATTTTAAGTTGAAGTCATATATAAATATCCTTTATTTTGTTTGATTGTTTGACAA
Ca_LG_1:26862076	TTTTGGGGTATCTTTCAAGTAGAGTCGGTCGCGGTATTTTTAGAGCAGCTTCATCGAAGCTGCCCTAAGCATAGGTTTCTCCAATAGATGCAATAGGCAA	A/G	CAATAGAGGGAGAGTTCACAACACCCTTTTATTTTAAGTTGAAGTCATATATAAATATCCTTTATTTTGTTTGATTGTTTGACAATATTTTCATTAATTG
Ca_LG_1:28120286	GATTAAACAAAAGGATTGGCTAATTAAAGTGCTAATGCTACAACCAGCCCATAAATAGTTAAAGCTTCCATAAAAGCCAGACTAAGTAATAAAGTACCCT	T/C	TTATTTTGTCCTCTGCTTCCGGTTTTCGCACAATCCCTTCCATATTCGGTAGCCATTTCATATTTAGTAGTCGTTCCATATTCGGTAGCCATGCAATTAG
Ca_LG_1:28120311	AAAGTGCTAATGCTACAACCAGCCCATAAATAGTTAAAGCTTCCATAAAAGCCAGACTAAGTAATAAAGTACCCTTTATTTTGTCCTCTGCTTCCGGTTT	T/G	TCGCACAATCCCTTCCATATTCGGTAGCCATTTCATATTTAGTAGTCGTTCCATATTCGGTAGCCATGCAATTAGTCAATAACTGAGTAGTCCTTCCATA
Ca_LG_1:28120315	TGCTAATGCTACAACCAGCCCATAAATAGTTAAAGCTTCCATAAAAGCCAGACTAAGTAATAAAGTACCCTTTATTTTGTCCTCTGCTTCCGGTTTTCGC	C/T	ACAATCCCTTCCATATTCGGTAGCCATTTCATATTTAGTAGTCGTTCCATATTCGGTAGCCATGCAATTAGTCAATAACTGAGTAGTCCTTCCATATTTG

Positions	-100 bp flanking sequences	SNPs within *kabuli* cultivar	+100 bp flanking sequences
Ca_LG_1:28120316	GCTAATGCTACAACCAGCCCATAAATAGTTAAAGCTTCCATAAAAGCCAGACTAAGTAATAAAGTACCCTTTATTTTGTCCTCTGCTTCCGGTTTTCGCA	A/G	CAATCCCTTCCATATTCGGTAGCCATTTCATATTTAGTAGTCGTTCCATATTCGGTAGCCATGCAATTAGTCAATAACTGAGTAGTCCTTCCATATTTGT
Ca_LG_1:29061769	GACAGAGTACATAAAAAAAATGCATTAATACTTGACAGAATCAAAATTTGAATCACCCTTTTCATCTATGCTAATTTTCTAATGGTCTAGACAATCAAAA	A/T	TTTAATTTTTGTATAGAGTATTCTTTGAAAAGATATCGATGATCCATATGCGGTTGAAAATAATGTAATATAAACCTGAAACGCGATAGAACAAGTTGAA
Ca_LG_1:29061783	AAAAAATGCATTAATACTTGACAGAATCAAAATTTGAATCACCCTTTTCATCTATGCTAATTTTCTAATGGTCTAGACAATCAAAATTTAATTTTTGTAT	T/A	AGAGTATTCTTTGAAAAGATATCGATGATCCATATGCGGTTGAAAATAATGTAATATAAACCTGAAACGCGATAGAACAAGTTGAAAGATTCCATGGTTC
Ca_LG_1:29442116	CGTCAAAGATAGAATTGATGGTTGCACTTTTTCCAACTCCTGTCTTTCCAAGAACCATTATTGTACAAGAGAAATCAAGTGGTTCTTGACCCACTGATTC	C/G	AAGTTGCTCGGCCATTGCACTAGCACGATCAAAGCTAAAAGCGCCAACACGGCTGCCATTCCTCCCTCTGAGTTGTTCAGCCAGTCCTAATCTATACAGA
Ca_LG_1:29442138	TGCACTTTTTCCAACTCCTGTCTTTCCAAGAACCATTATTGTACAAGAGAAATCAAGTGGTTCTTGACCCACTGATTCAAGTTGCTCGGCCATTGCACTA	A/T	GCACGATCAAAGCTAAAAGCGCCAACACGGCTGCCATTCCTCCCTCTGAGTTGTTCAGCCAGTCCTAATCTATACAGAACTTGTGCTACAACAACATTGT
Ca_LG_1:29442164	CAAGAACCATTATTGTACAAGAGAAATCAAGTGGTTCTTGACCCACTGATTCAAGTTGCTCGGCCATTGCACTAGCACGATCAAAGCTAAAAGCGCCAAC	C/A	ACGGCTGCCATTCCTCCCTCTGAGTTGTTCAGCCAGTCCTAATCTATACAGAACTTGTGCTACAACAACATTGTGGGGTGTCTGCCCAAGCCTATTAGCT
Ca_LG_1:29442282	TCTGAGTTGTTCAGCCAGTCCTAATCTATACAGAACTTGTGCTACAACAACATTGTGGGGTGTCTGCCCAAGCCTATTAGCTAGCCGCAAAAACTTCACC	C/A	CTAATCATTTGAAGTTTCTCACGAGTCTCGTCATACTCCTCAGCCTCCCCAGATGAGGACTCGTCCAATTTTTGGGACTGCAAATTAGATACAGTATTAT
Ca_LG_1:29442376	TTCACCCTAATCATTTGAAGTTTCTCACGAGTCTCGTCATACTCCTCAGCCTCCCCAGATGAGGACTCGTCCAATTTTTGGGACTGCAAATTAGATACAG	G/C	TATTATTGACCCGCGGCTGCTGCACTACCCGTGGAGCAGGTTTCAATAATGGAGCTGCAGGTCCAAGGCCAGCAGGAGGAACAAGAGTGGTGGTAGTGGC
Ca_LG_1:29649191	ATTAAAATTAAGACCTTTTTTAATTAAGAGAGAGAGAGAGAGAAACCATGTTTCAGTTTTCATCAGCGCCTAAACCTGTAACGAGACCTAGCAGCACCTC	C/T	AACCCACCGCAGCGCTGACTTCACTACACACTGTTGTGTACAACGTTGCCTGTACTACCCACTGTTCTGCCGCAGCACCGCGTTCTCTATTTTCACGTTA
Ca_LG_1:30253632	GTGTAGATGATGTTGTCCTTGTTGGTGGCTCTTCTAGGATTCCCAAAGTGAAGGAGTTGTGCGAGAGCATTAATCCTGATGAGGCTGTGGCTTATGGGGG	G/C	AGCTGTTCAGGCTGCATTGTTGAGTGAAGGCTTTAACAATATTCCAAAGCTGGTGTTGCAGGATGTCACACCTTTGTCTCTTGGTAGGTCAGTATTAGGA
Ca_LG_1:30284273	GGCGATCTTCTTGTTAATCCTGTTCAGGTTAACATTGGAAATGTAGACGAGCTTGCTGCAAATAAATCCATCACACAGGTATTCAATTAATGCCCACTTA	A/G	TTTAAAATCTCAAAGTTGCTGCTAAATGGACAGTTCTTTTTTTTATCTTGCTTCGTCAATGTTTTTGAAATCTCAAAGTTGTCATTGTGGTCAACTTGTA
Ca_LG_1:30285027	TGGGGCAGCTGCAATTCATGGAGACAAGTCTCAAGGTGAGAGAGACTGGGTTTTAGGTCAGTTCCGGACTGGGAAGTCTCCAATTTTGGTTGCCACTGAT	T/C	GTTGCTGCCCGTGGTCTTGATATTAAGGATATAAGGTTAGCCTCTTTGTATTTAAAGTGCTTATTCTCAATTTTATATATGAAGCAGTCATTTGTCGTTT

Positions	-100 bp flanking sequences	SNPs within *kabuli* cultivar	+100 bp flanking sequences
Ca_LG_1:30295802	ACAACTTGACCCAAAAGTATTACAACAGTAAAAAGGAGACACTCCTGCTAAAAGGAGACAACTTTTCTGTATGCTTTTTGATTCGTTGGATGGTTCAGTA	A/C	AAGTTGGATCAATGCCTGTTGATTGTATCATGCAGCAGGGAAGCTGTTTCTTTTTTTAATCAAATTATGTGGCTAATGCTTTTGATACAAGGCCCTTCCT
Ca_LG_1:30310231	ATGTCAGATTTCCTAAACTAAACGTCGTTACTTTCAATAAAGAAGGTCTACAACACATGTTCTTCCAAAGGAAGGCTTTGCTGCAGCACCTACAAGGGTC	C/T	CATATTCCACTCTTGTAAGTCACAGGGATATTTTGAAGGCTGCTATTGATAGTCTGCTCAGAAAAACACATTAAAATCCTTGTGAACAAACTGAGAAGAG
Ca_LG_1:30942802	TGCTCGAATTAGTAATAGAAATGGTGGAACAAGTGCAAAACCAAGAGGTTCTTTGTTGACAGTGGCTTTTCAAATATTGATGTCTAATGCAGAAGTAGTG	G/C	AATTTGGAACATGCTGCAGCTGCTGTTAACTCACTCATCAATACAACTGTTGATAACATAAGGGATGCTCTGTTCAAAATCTAGGAAGTTTTTTTATCAC
Ca_LG_1:31172867	TAAATGGATTGTGGAGAACAGTAAAGAGTTGGATGTTACTAAGGGTAACCTTGTTGCAGCAGCAAAGGAAATTGAACAATACAAGGAGCAAATGCATAAA	A/G	TTACATCAAAATCTTGAACAAAGAATGAATGAGCTTAGAGAAATCGATGAAGAAAGAAGAGAACATTGTGCTGTTACAAAAAAGCAACAAGATGCATTGA
Ca_LG_1:31172868	AAATGGATTGTGGAGAACAGTAAAGAGTTGGATGTTACTAAGGGTAACCTTGTTGCAGCAGCAAAGGAAATTGAACAATACAAGGAGCAAATGCATAAAT	T/G	TACATCAAAATCTTGAACAAAGAATGAATGAGCTTAGAGAAATCGATGAAGAAAGAAGAGAACATTGTGCTGTTACAAAAAAGCAACAAGATGCATTGAA
Ca_LG_1:31926195	ACGCATGGCAGCAACGTGAAGCACTGTGTACCCTCGAGAATTCCGAAGATTGACATTAGCCATACCCAACCCAAGTACTTCGGAAACGACCTTGGGGTCG	G/A	CAGTAGGCTGCTGCGTAATGGAGAGCACCGGCTTCGTCTAAAGTAATGTCCGACTCATTTAAAAGAAGTTTAACAAGCTCGACATCATCCGAGTCCAATG
Ca_LG_1:32113130	TTTTGAGTACTTTCAGTGCATCGTAGCGCAAGTTTTATGAACAATCTTCTCCTTTCTTCAATCTGCAGTAGCTAGCATCAAGAGAATAAGGTAGTGATCG	G/A	ATGCAAATTCAAGTTCTGCAGCAAACAAAAGCAGCAGAGACCGTAACACTATTTCCTTTTTTTTTTTTTTTTGTTTCTTACTACTCATGTGTCTTGCACCA
Ca_LG_1:32434343	TGAATTTCATTCGGTTGGCAATCAAGACCAACAGCTGCACCTGCAGGCTGCAGCTATTATATGATTCTGAATCATTTGCCTGCTCATGAAATGAACTTAC	C/T	TGGTTCCTGAATTGTGACAATACTGCGATATAAACTTTGTTTATAGGGACATGCTTGGATTAGTAGAGCATCAAGAAGTCGAAATTTAGTCTGGAAAGTA
Ca_LG_1:32434370	CCAACAGCTGCACCTGCAGGCTGCAGCTATTATATGATTCTGAATCATTTGCCTGCTCATGAAATGAACTTACTGGTTCCTGAATTGTGACAATACTGCG	G/C	ATATAAACTTTGTTTATAGGGACATGCTTGGATTAGTAGAGCATCAAGAAGTCGAAATTTAGTCTGGAAAGTAAGCGAAAAACAACCAGGTAAGCTGCTT
Ca_LG_1:32452598	CAATACTGGCTTCTTCTGTTTCGTTGCATAAGATGGCAGCATGATCAGGAGAATCTAATGTAGGCAACTCAGGTGTCTCTGTCTTTCTAACTTGCAGTCC	C/T	GGTAGAGCGTGCAAGGTCAATCCATTGCTGCAGCAATTTACAGAAAAACCAAATTTGAAAAATCCTAGTCAAATTTAGAAACTATATGTAGATGAATGAG
Ca_LG_1:32924483	GACAACACCTATCCTACTTGCTACTTTGCTGCATTCTTTGTACTATTTGGCCTAATTTTTTAATTTCTGTTATTATGGTTTTAACTTCTATATTGTTATA	A/G	TTTGTCTAGGTTCTGCTATTACTGGTCCTATTGGTAAGGAATGTGCTGATCTATGGCCAAGGATTGCAAGTGCAGCCAATGCAATTGTATGAGTGGGGCG
Ca_LG_1:33038368	AAGTTTTGGTTGAGACAATTTGCGATGTTCTTGATGAGTTATACACAATTTCTTTAGGGATTCAAACATGTTCCCGTTTGCAAGGAAAGCTGCTGAGTTG	G/C	TTTGCAACAGGACACTGAAGCACATGGAACAAGACGTTGTGCTGTCCTAGTATGTACATTATTCAGCTCCTTGGGAATAATGTTCCTTCTATGCTGCACA

Positions	-100 bp flanking sequences	SNPs within *kabuli* cultivar	+100 bp flanking sequences
Ca_LG_1:33038427	ATTCAAACATGTTCCCGTTTGCAAGGAAAGCTGCTGAGTTGTTTGCAACAGGACACTGAAGCACATGGAACAAGACGTTGTGCTGTCCTAGTATGTACAT	T/A	TATTCAGCTCCTTGGGAATAATGTTCCTTCTATGCTGCACAATTTTCATTATTAGGTTAGCCATACACTTCCAAGTTTCTGCATTTGGCAAGCTATTGGG
Ca_LG_1:33043165	TCACAGAATTCAAACGCAACGATTTTGCTGCACTACCTGAAATTTTTCAAGAACATATTCAAACTAATAATAATCTAGATGTCAAATATTGCAAAATAAG	G/A	CATTTCTCTTTACCCAAATAACTACTCACCAAAATCACCAACTGATGCATCTTCCTTTGTTTTCAACGCAGCTGCAATCTGATGATGATTACTAGCAGTA
Ca_LG_1:33043233	AATAATCTAGATGTCAAATATTGCAAAATAAGCATTTCTCTTTACCCAAATAACTACTCACCAAAATCACCAACTGATGCATCTTCCTTTGTTTTCAACG	G/T	CAGCTGCAATCTGATGATGATTACTAGCAGTATCAACCACTGAACCAGTAAGGTTTCTTGCAAGATGTCTAATTTGATTTAGACGCAGCCTTGGTAAATT
Ca_LG_1:33043281	AATAACTACTCACCAAAATCACCAACTGATGCATCTTCCTTTGTTTTCAACGCAGCTGCAATCTGATGATGATTACTAGCAGTATCAACCACTGAACCAG	G/A	TAAGGTTTCTTGCAAGATGTCTAATTTGATTTAGACGCAGCCTTGGTAAATTATTTAGCTTACAAGCTAGAGTTTCCATGTTAATTGATGATCCTATTGT
Ca_LG_1:33057870	AATACTACACAAGAAATGTGAATCTATACCTCATCACAAATATCATAAAGTCATTAAATCGCGTGTTTGCTGCAGTGAGAAATCACAATAATTGATAGGC	C/T	ATACATCTAGTATCTACAAATGTCAATCACTCCACCAATGCAAAAAAACTTATTGTGGCCAAACCAATGGTGTCAATCGCGAAATGTGGATAATAGTTTG
Ca_LG_1:33076269	CTATAACCCTACTTAATTGATACTTTGATATTGGAACATAATCATTCACCATATATAAGGCATAGTATTTATACTAGTATCATGATTGAGGGGTGTCACA	A/C	ATTTGCAGCTGCTTATTAAGTTTGTTGTTGCTTGCTGGCACTGAGTGACCACAGCAATTATTAGAAAATGGGTATGGTAGTTATGATCTTACCCAACAAC
Ca_LG_1:33106822	TTGCTCCTGCTGCATCAAGTTTTGGTTCAGAACAACCAAAATTTTCCTCCATCGCCAACTTTTCCTCGCTGCCATCTCCTCCAGTGTTGTCCACATCATT	T/C	GCCATTGTTCTCAGTTCCCTCATCACTGCTACTACCAGAGTTACGATCCCTGTCTTTATCAATGGCATGGAGTGGTTTGATTTCATCGTCTACATTGTCA
Ca_LG_1:33256282	GTGGGTAAGAAAATTAACAGTGGTGTAACTTGTATACATTCTTTCAGGATATGATTATGGGGAGAAAAAGCGAATTCCTGACTGGTGTGATAGAATAATG	G/A	TATCGTGATACTCGGGCAGCTGCCGTGTCTGACTGCAATTTAGACTGTCCTGCTGTGTCTTCAATTTTACAGTATGTTTCATGGTCAAAATTGTTTTCTA
Ca_LG_1:33289544	GGCGGTGTTTGTTGATTTTGGAGCGCACTAGAAAAGAGGCAGCACAGCTGTAACAGAGTAAAAAAAAAACAAAACAGTGACAGCTGGTGTAAAGCACAGC	C/T	TGTGTTTGAAAGAAAAAAAAATCACTGGAAAAACACACAGTGAAGGCGGTGCTTTTGAGGCTAGCTGTAGTGTCGTGGCTGATTGCTAGAAAGGCAGCCG
Ca_LG_1:33289576	AAAGAGGCAGCACAGCTGTAACAGAGTAAAAAAAAAACAAAACAGTGACAGCTGGTGTAAAGCACAGCTGTGTTTGAAAGAAAAAAAAATCACTGGAAAA	A/C	ACACACAGTGAAGGCGGTGCTTTTGAGGCTAGCTGTAGTGTCGTGGCTGATTGCTAGAAAGGCAGCCGGAGTTGTTGGTGACAGATGGCTGTATGAAGCC
Ca_LG_1:34019011	TCAGTACCCTAAAGTACAATATTTATTTAAGATAAACAAATGTGTAACACATTATCAATTACTTATTCCACGGCCACGAATAGGCATCCCTATTTGTTAC	C/T	CCTAATTGGTCCTTTTTCGCTGATAACCCAATTACATTCAGAACATGGATCTCTAAGACCTACATATATGTCAAACCAGTGAAATTCTCCTTCCCACTTA
Ca_LG_1:34019037	TTAAGATAAACAAATGTGTAACACATTATCAATTACTTATTCCACGGCCACGAATAGGCATCCCTATTTGTTACCCTAATTGGTCCTTTTTCGCTGATAA	A/T	CCCAATTACATTCAGAACATGGATCTCTAAGACCTACATATATGTCAAACCAGTGAAATTCTCCTTCCCACTTAAAGGAACAATAGAATAGTGTTGTGCC

Positions	-100 bp flanking sequences	SNPs within *kabuli* cultivar	+100 bp flanking sequences
Ca_LG_1:35152413	TTTTCAGTGACGACATGGATTAGTACAGAACCAGTTTTCTCAGAACACTTAAGTTCTTCAAGAGTTGCAACAAGTTCATCAATGTTATGACCATCAACAG	G/A	GGCCAATATAATTAAGTCCAAGCTCATCAAAAAGTGTTGATCCAGAACCACCAATCATACTGCGAGCATATTCGTCAACTTTTTCAGCCAATTCATGCAT
Ca_LG_1:35152415	TTCAGTGACGACATGGATTAGTACAGAACCAGTTTTCTCAGAACACTTAAGTTCTTCAAGAGTTGCAACAAGTTCATCAATGTTATGACCATCAACAGGG	G/A	CCAATATAATTAAGTCCAAGCTCATCAAAAAGTGTTGATCCAGAACCACCAATCATACTGCGAGCATATTCGTCAACTTTTTCAGCCAATTCATGCATTG
Ca_LG_1:35152427	ATGGATTAGTACAGAACCAGTTTTCTCAGAACACTTAAGTTCTTCAAGAGTTGCAACAAGTTCATCAATGTTATGACCATCAACAGGGCCAATATAATTA	A/C	AGTCCAAGCTCATCAAAAAGTGTTGATCCAGAACCACCAATCATACTGCGAGCATATTCGTCAACTTTTTCAGCCAATTCATGCATTGAGCCATAAGCTA
Ca_LG_1:35152432	TTAGTACAGAACCAGTTTTCTCAGAACACTTAAGTTCTTCAAGAGTTGCAACAAGTTCATCAATGTTATGACCATCAACAGGGCCAATATAATTAAGTCC	C/G	AAGCTCATCAAAAAGTGTTGATCCAGAACCACCAATCATACTGCGAGCATATTCGTCAACTTTTTCAGCCAATTCATGCATTGAGCCATAAGCTAATTGT
Ca_LG_1:35152436	TACAGAACCAGTTTTCTCAGAACACTTAAGTTCTTCAAGAGTTGCAACAAGTTCATCAATGTTATGACCATCAACAGGGCCAATATAATTAAGTCCAAGC	C/T	TCATCAAAAAGTGTTGATCCAGAACCACCAATCATACTGCGAGCATATTCGTCAACTTTTTCAGCCAATTCATGCATTGAGCCATAAGCTAATTGTGTTA
Ca_LG_1:35152445	AGTTTTCTCAGAACACTTAAGTTCTTCAAGAGTTGCAACAAGTTCATCAATGTTATGACCATCAACAGGGCCAATATAATTAAGTCCAAGCTCATCAAAA	A/G	AGTGTTGATCCAGAACCACCAATCATACTGCGAGCATATTCGTCAACTTTTTCAGCCAATTCATGCATTGAGCCATAAGCTAATTGTGTTAGGCATAAGA
Ca_LG_1:35327500	TTGTAATTTGTAAACCTGTTTTAGCATTGTCTATCTATAGCTCTATATTCCTGCAACAACATTCTGTTATTGGCTTTAGCTCTATCTTTCTGTAACATCG	G/A	CTTTAATCTCATGTAAATCTATATAATAATGTAGCAATTATTATGGTTCAGGCAAATTGCTATGGTTATGCTGCTTCACCTTAGAAGTCCCAAGTGCTGT
Ca_LG_1:35327638	TATTATGGTTCAGGCAAATTGCTATGGTTATGCTGCTTCACCTTAGAAGTCCCAAGTGCTGTGCTTTCAAAGATTCATCCCACAATGCCCACAACATCAC	C/T	TTCACCTTCAAAAAACCTTGTCCAAAACATGTCGTCTGGAAGCATTGCAGTGTCAAAATGATGTGCAGTATTTCTACATTAGTTGTTTGCTGCCTATGTT
Ca_LG_1:35327670	CTGCTTCACCTTAGAAGTCCCAAGTGCTGTGCTTTCAAAGATTCATCCCACAATGCCCACAACATCACTTCACCTTCAAAAAACCTTGTCCAAAACATGT	T/A	CGTCTGGAAGCATTGCAGTGTCAAAATGATGTGCAGTATTTCTACATTAGTTGTTTGCTGCCTATGTTATGAATTCTGTTTTGGGGTGATAGGCTCTTAT
Ca_LG_1:35327671	TGCTTCACCTTAGAAGTCCCAAGTGCTGTGCTTTCAAAGATTCATCCCACAATGCCCACAACATCACTTCACCTTCAAAAAACCTTGTCCAAAACATGTC	C/G	GTCTGGAAGCATTGCAGTGTCAAAATGATGTGCAGTATTTCTACATTAGTTGTTTGCTGCCTATGTTATGAATTCTGTTTTGGGGTGATAGGCTCTTATG
Ca_LG_1:35327680	TTAGAAGTCCCAAGTGCTGTGCTTTCAAAGATTCATCCCACAATGCCCACAACATCACTTCACCTTCAAAAAACCTTGTCCAAAACATGTCGTCTGGAAG	G/A	CATTGCAGTGTCAAAATGATGTGCAGTATTTCTACATTAGTTGTTTGCTGCCTATGTTATGAATTCTGTTTTGGGGTGATAGGCTCTTATGCATGAGAAT
Ca_LG_1:35327688	CCCAAGTGCTGTGCTTTCAAAGATTCATCCCACAATGCCCACAACATCACTTCACCTTCAAAAAACCTTGTCCAAAACATGTCGTCTGGAAGCATTGCAG	G/A	TGTCAAAATGATGTGCAGTATTTCTACATTAGTTGTTTGCTGCCTATGTTATGAATTCTGTTTTGGGGTGATAGGCTCTTATGCATGAGAATACTGAAGT

**Positions**	**-100 bp flanking sequences**	**SNPs within *kabuli* cultivar**	**+100 bp flanking sequences**
Ca_LG_1:35327708	AGATTCATCCCACAATGCCCACAACATCACTTCACCTTCAAAAAACCTTGTCCAAAACATGTCGTCTGGAAGCATTGCAGTGTCAAAATGATGTGCAGTA	A/G	TTTCTACATTAGTTGTTTGCTGCCTATGTTATGAATTCTGTTTTGGGGTGATAGGCTCTTATGCATGAGAATACTGAAGTAGGGAAATGATGGGTGCTGG
Ca_LG_1:35590217	ATACCAAATGTTCTAAGGTTTTAGATTATATGTGAATCAAAATTTCTTAGTGATCTTGAAGCATTTTGATAATTGCTGCTAACGCTGTCCAAATTCTCCC	C/T	GACAAATTTTAATTCCGAGGATACAAGTAAAAAATTGCTTATACAAAATTCACCAACATAGCAGCATAATTGACTTATATTTATCAAAACAAACAGGTTA
Ca_LG_1:35590238	TAGATTATATGTGAATCAAAATTTCTTAGTGATCTTGAAGCATTTTGATAATTGCTGCTAACGCTGTCCAAATTCTCCCGACAAATTTTAATTCCGAGGA	A/G	TACAAGTAAAAAATTGCTTATACAAAATTCACCAACATAGCAGCATAATTGACTTATATTTATCAAAACAAACAGGTTATCCCATATTTTTTTATTGGTA
Ca_LG_1:36808803	ATTGGTTTTTGCAGCCTTAATGCTTTTTGCTGGTTGGTTTCATTATCCCAAAGCTGCTCCAAAATTGGCTTCGTTCCAAGATGTAGAATCCATGTTGAAC	C/T	CACCATTTGGCAGGGCTACTAGGACTTGGGTCTCTTTCTTGGGCGGGGAATCAAGTACATGTATCTTTACCAATTAACCAATTTCTAAATGTTGGAGTAG
Ca_LG_1:37653172	CAATTTTAGGTGTTGGTGCGGGATTTGTTGTGGGGAAAGAAGGACCAATGGTGCATACAGGCGCTTGCATTGCTAATCTACTAGGACAAGGTGGTTCTAC	C/G	AAAATATGGACTGACCTGGAAATGGCTGAGATACTTCAAAAATGATAGAGATAGAAGGGATTTGATCACGTGCGGAGCAGCTGCAGGTGTTGCTGCTGCC
Ca_LG_1:37653200	TGTGGGGAAAGAAGGACCAATGGTGCATACAGGCGCTTGCATTGCTAATCTACTAGGACAAGGTGGTTCTACAAAATATGGACTGACCTGGAAATGGCTG	G/C	AGATACTTCAAAAATGATAGAGATAGAAGGGATTTGATCACGTGCGGAGCAGCTGCAGGTGTTGCTGCTGCCTTTCGTGCCCCTGTTGGTGGTGTCCTTT
Ca_LG_1:37653212	AGGACCAATGGTGCATACAGGCGCTTGCATTGCTAATCTACTAGGACAAGGTGGTTCTACAAAATATGGACTGACCTGGAAATGGCTGAGATACTTCAAA	A/T	AATGATAGAGATAGAAGGGATTTGATCACGTGCGGAGCAGCTGCAGGTGTTGCTGCTGCCTTTCGTGCCCCTGTTGGTGGTGTCCTTTTCGCACTGGAAG
Ca_LG_1:37653227	TACAGGCGCTTGCATTGCTAATCTACTAGGACAAGGTGGTTCTACAAAATATGGACTGACCTGGAAATGGCTGAGATACTTCAAAAATGATAGAGATAGA	A/G	AGGGATTTGATCACGTGCGGAGCAGCTGCAGGTGTTGCTGCTGCCTTTCGTGCCCCTGTTGGTGGTGTCCTTTTCGCACTGGAAGAAGCAACTTCATGGT
Ca_LG_1:37653246	AATCTACTAGGACAAGGTGGTTCTACAAAATATGGACTGACCTGGAAATGGCTGAGATACTTCAAAAATGATAGAGATAGAAGGGATTTGATCACGTGCG	G/A	GAGCAGCTGCAGGTGTTGCTGCTGCCTTTCGTGCCCCTGTTGGTGGTGTCCTTTTCGCACTGGAAGAAGCAACTTCATGGTGGAGGAGTGCTCTTCTATG
Ca_LG_1:37735245	TGATGGACATGCTCACCGATCTTAGAATTGGAAAAGTATGTGATGGTGTGGAGTCTTTCCCCAATAAGGATTTTGCGTTGCTGCCTCCCTCCCTTACCTC	C/A	TCTATCGCTAAGCAATATGCCAAGTTTGCACACGTTGGACTGCACGGAGCTTCTCCACCTCACGTCCCTCCAACAATTAGAAATTGACAGTTGTTCAAAT
Ca_LG_1:37912214	TATAGCGTCAATATAGCCGCTATTTGAAAACATTTTTGTACTAAATAATGTATCGCACAACAATAGTGATTTGTTCAAATTCTGCTATGTTATAGCTGCG	G/A	ATTAGACAACACTAAATTGAACCAAAAAAATAAAAACACCCAACCTTGTAAATTAGGATTCTTAATAGTCCAAGGGCTCCAGCAAGATTGCAATCTGTCC
Ca_LG_1:38256174	CGTGCGCGTTCCTTTGTGGCGGCCTTTGCAGCCACAAATGCCACGTGCGCGTTCCTTTGTGGCGGCCTTTGCAGCCACAAATGTCCACTTGTCCCGTCCG	G/T	CCTTTGTGACGGATTTGGCCCTCACAAAATTGATTATTTGTGGCTGTAAAAGCCCTCACAAAATCCTTAAATATTTAACTGGATTTTGTGGCTGCCCAAG

Positions	-100 bp flanking sequences	SNPs within *kabuli* cultivar	+100 bp flanking sequences
Ca_LG_1:38409310	CGTTGATAGCGGGGGATAAGTTAAGGTCAAGCGGATAAGATTCATCGGCGGCGGCGTCACGGTCGCGGCTGGAGGATTCAACGGTGCTGCTTTGGCTAGT	T/G	ACTTTGGATCTTTATATTTTCGGAAGGGTAAGGGAAGTTAGTTTTAGCCTTAGAACCACGGAAATCACGTGCGGCGGTGTCGTAGGCTCGTGCAGCCTCT
Ca_LG_1:38524971	AGACAATGCTTTTGATACAGAGGTTGAAACTTTGGGCAAGATTAGGCACAAGAACATTGTGAAACTATGGTGTTGTTGTACCACTAGGGATTGCAAGCTA	A/T	TTGGTTTATGAGTATATGCCAAATGGTAGTCTTGGTGATTTGCTGCATAGCAATAAAGGAGGGTTGTTGGATTGGCCAACAAGGTATAAGATAGCTCTTG
Ca_LG_1:38525016	GCACAAGAACATTGTGAAACTATGGTGTTGTTGTACCACTAGGGATTGCAAGCTATTGGTTTATGAGTATATGCCAAATGGTAGTCTTGGTGATTTGCTG	G/T	CATAGCAATAAAGGAGGGTTGTTGGATTGGCCAACAAGGTATAAGATAGCTCTTGATGCTGCAGAAGGGCTCTCTTATCTGCATCATGACTGTGTCCCTC
Ca_LG_1:38525022	GAACATTGTGAAACTATGGTGTTGTTGTACCACTAGGGATTGCAAGCTATTGGTTTATGAGTATATGCCAAATGGTAGTCTTGGTGATTTGCTGCATAGC	C/T	AATAAAGGAGGGTTGTTGGATTGGCCAACAAGGTATAAGATAGCTCTTGATGCTGCAGAAGGGCTCTCTTATCTGCATCATGACTGTGTCCCTCCTATTG
Ca_LG_1:38525024	ACATTGTGAAACTATGGTGTTGTTGTACCACTAGGGATTGCAAGCTATTGGTTTATGAGTATATGCCAAATGGTAGTCTTGGTGATTTGCTGCATAGCAA	A/C	TAAAGGAGGGTTGTTGGATTGGCCAACAAGGTATAAGATAGCTCTTGATGCTGCAGAAGGGCTCTCTTATCTGCATCATGACTGTGTCCCTCCTATTGTT
Ca_LG_1:38525055	TAGGGATTGCAAGCTATTGGTTTATGAGTATATGCCAAATGGTAGTCTTGGTGATTTGCTGCATAGCAATAAAGGAGGGTTGTTGGATTGGCCAACAAGG	G/A	TATAAGATAGCTCTTGATGCTGCAGAAGGGCTCTCTTATCTGCATCATGACTGTGTCCCTCCTATTGTTCATAGAGATGTGAAATCTAATAACATCTTGT
Ca_LG_1:38525058	GGATTGCAAGCTATTGGTTTATGAGTATATGCCAAATGGTAGTCTTGGTGATTTGCTGCATAGCAATAAAGGAGGGTTGTTGGATTGGCCAACAAGGTAT	T/C	AAGATAGCTCTTGATGCTGCAGAAGGGCTCTCTTATCTGCATCATGACTGTGTCCCTCCTATTGTTCATAGAGATGTGAAATCTAATAACATCTTGTTGG
Ca_LG_1:38525068	CTATTGGTTTATGAGTATATGCCAAATGGTAGTCTTGGTGATTTGCTGCATAGCAATAAAGGAGGGTTGTTGGATTGGCCAACAAGGTATAAGATAGCTC	C/G	TTGATGCTGCAGAAGGGCTCTCTTATCTGCATCATGACTGTGTCCCTCCTATTGTTCATAGAGATGTGAAATCTAATAACATCTTGTTGGACGAGGACTT
Ca_LG_1:38563993	GGAATCAATTTTTTTAACTGTCATGGAAACATTGCAGCTATTCAACCCCACTCTGTAGCATATGAATAATCAAAACGTTTTGATTGGATGTAATAAAGTA	A/T	GAAAACATATACAGATAATATTTCTCGTACCTTCCACAACATGTCGCCAAAAGAAGAATTTGGCTGCTCCCTACATCATTTGCATTGCTTGTTACATTAA
Ca_LG_1:38564038	CCCCACTCTGTAGCATATGAATAATCAAAACGTTTTGATTGGATGTAATAAAGTAGAAAACATATACAGATAATATTTCTCGTACCTTCCACAACATGTC	C/T	GCCAAAAGAAGAATTTGGCTGCTCCCTACATCATTTGCATTGCTTGTTACATTAAATGTAGAAGACAATGGCAGTGATTCCCATGTTTGACTTTCTGGAA
Ca_LG_1:38564461	AATTACAATCTCTCCTTCAACCACAAGAGCCAAACCCACCTGTATTATTCGGAAATCTCTTGGAGAATATTAGTGTGTAGTGTCAGAATTCAATCTAATA	A/G	TAATTGTAATGTCACACAAGTTAGCTCAGCTGGCAGCTGCTAATACAGTTGCAATGGACAATGATAGTTAGTATCTAAAAGCTTCTAATAACAGCTGCGT
Ca_LG_1:38564469	TCTCTCCTTCAACCACAAGAGCCAAACCCACCTGTATTATTCGGAAATCTCTTGGAGAATATTAGTGTGTAGTGTCAGAATTCAATCTAATATAATTGTA	A/T	ATGTCACACAAGTTAGCTCAGCTGGCAGCTGCTAATACAGTTGCAATGGACAATGATAGTTAGTATCTAAAAGCTTCTAATAACAGCTGCGTCAGTAGTA

Positions	-100 bp flanking sequences	SNPs within *kabuli* cultivar	+100 bp flanking sequences
Ca_LG_1:38564525	GAATATTAGTGTGTAGTGTCAGAATTCAATCTAATATAATTGTAATGTCACACAAGTTAGCTCAGCTGGCAGCTGCTAATACAGTTGCAATGGACAATGA	A/G	TAGTTAGTATCTAAAAGCTTCTAATAACAGCTGCGTCAGTAGTAGGAGAGTTGGTTAGAATAACAGTTGTAGAATGTTTAGCATGTGTTTGTATAAAAAA
Ca_LG_1:38923448	AACAGATTTACAAATTACAAATTCAAAATCAATAGCAAAAAGATAAGGCTACAACAGATTTACAAATTCAAAATTAATATAAAAAAGATACAATAGCAGC	C/T	AGCTATACTTTGTGAGAACTCCAATAAAAAAGGCGATGGCTGCAAATCTGTGAAGCATCATTTTGAGAGGGCATAGGAGCCTGCTGATACCTATCGAAAA
Ca_LG_1:39657931	TGGATGAATTTGTCTCAGTAGTCTTTCATTCTTAAACTCTAAGTTCACAACACTACATTGATCATCATCAACTCCAAACACTACACTGATGCGTCTACTG	G/C	CATCTTGATTCTCCACAATGACGGAATCACAATCACCGTTAGTTTATTATCTTCCATTTAATCATCTTTCTAATTTACCAAATTCATTTTCGGATCTCTA
Ca_LG_1:39657941	TGTCTCAGTAGTCTTTCATTCTTAAACTCTAAGTTCACAACACTACATTGATCATCATCAACTCCAAACACTACACTGATGCGTCTACTGCATCTTGATT	T/G	CTCCACAATGACGGAATCACAATCACCGTTAGTTTATTATCTTCCATTTAATCATCTTTCTAATTTACCAAATTCATTTTCGGATCTCTAATTTCTTTGC
Ca_LG_1:39657948	GTAGTCTTTCATTCTTAAACTCTAAGTTCACAACACTACATTGATCATCATCAACTCCAAACACTACACTGATGCGTCTACTGCATCTTGATTCTCCACA	A/G	ATGACGGAATCACAATCACCGTTAGTTTATTATCTTCCATTTAATCATCTTTCTAATTTACCAAATTCATTTTCGGATCTCTAATTTCTTTGCTTTTTTT
Ca_LG_1:39657960	TCTTAAACTCTAAGTTCACAACACTACATTGATCATCATCAACTCCAAACACTACACTGATGCGTCTACTGCATCTTGATTCTCCACAATGACGGAATCA	A/G	CAATCACCGTTAGTTTATTATCTTCCATTTAATCATCTTTCTAATTTACCAAATTCATTTTCGGATCTCTAATTTCTTTGCTTTTTTTTTTGTAGGCGGA
Ca_LG_1:39658091	ATCATCTTTCTAATTTACCAAATTCATTTTCGGATCTCTAATTTCTTTGCTTTTTTTTTTGTAGGCGGAGAAGATTAACACTATCTTGAAGGCTGCTGGG	G/C	GTCACTGTCGAATCATACTGGCCAAGCTTATTCGCCAAACTTGCTCAGAATAAGAGCATTGACGATCTCGTTTTGAACGATGGCGCTGCCGGTGGTGCCG
Ca_LG_1:39658171	CTATCTTGAAGGCTGCTGGGGTCACTGTCGAATCATACTGGCCAAGCTTATTCGCCAAACTTGCTCAGAATAAGAGCATTGACGATCTCGTTTTGAACGA	A/C	TGGCGCTGCCGGTGGTGCCGCTGTTGCCGTATCTGCTCCTGCCGCTGCTGCTGGTGGTGGAGCCGCTGCAGCTGCCGCACCTGCTGTCGAGGCCAAGAAG
Ca_LG_1:39658193	CACTGTCGAATCATACTGGCCAAGCTTATTCGCCAAACTTGCTCAGAATAAGAGCATTGACGATCTCGTTTTGAACGATGGCGCTGCCGGTGGTGCCGCT	T/C	GTTGCCGTATCTGCTCCTGCCGCTGCTGCTGGTGGTGGAGCCGCTGCAGCTGCCGCACCTGCTGTCGAGGCCAAGAAGGTACTGTTAGTAGGACCATTTA
Ca_LG_1:39658203	TCATACTGGCCAAGCTTATTCGCCAAACTTGCTCAGAATAAGAGCATTGACGATCTCGTTTTGAACGATGGCGCTGCCGGTGGTGCCGCTGTTGCCGTAT	T/G	CTGCTCCTGCCGCTGCTGCTGGTGGTGGAGCCGCTGCAGCTGCCGCACCTGCTGTCGAGGCCAAGAAGGTACTGTTAGTAGGACCATTTAGACTTAAATA
Ca_LG_1:39658218	TTATTCGCCAAACTTGCTCAGAATAAGAGCATTGACGATCTCGTTTTGAACGATGGCGCTGCCGGTGGTGCCGCTGTTGCCGTATCTGCTCCTGCCGCTG	G/T	CTGCTGGTGGTGGAGCCGCTGCAGCTGCCGCACCTGCTGTCGAGGCCAAGAAGGTACTGTTAGTAGGACCATTTAGACTTAAATATCATATCTTGCATCA
Ca_LG_2:674638	TTAAAATAAATATACCATTAACTAGATCTAGATGTGATATAAATAAATAAAGCAACTGGGAGATTGAACTAGCTGAAGACTTTAACACATTACATTCATA	A/T	TCTTGAACTTTCCTTTGATAATCTCTTACTGCATTAGCAGCAGCACCACCTGCAAGGACGGCCTCTTCTAGCTCCCTTACAGTTTGGGTGAGCTTTTCAA

**Positions**	**-100 bp flanking sequences**	**SNPs within *kabuli* cultivar**	**+100 bp flanking sequences**
Ca_LG_2:674698	AGATTGAACTAGCTGAAGACTTTAACACATTACATTCATATCTTGAACTTTCCTTTGATAATCTCTTACTGCATTAGCAGCAGCACCACCTGCAAGGACG	G/T	GCCTCTTCTAGCTCCCTTACAGTTTGGGTGAGCTTTTCAACCTCTGCCACCTTTTGTCTATGCATTTTGTCCAAAATTTTATTTTCCTCCTGAGAGAAAT
Ca_LG_2:674700	ATTGAACTAGCTGAAGACTTTAACACATTACATTCATATCTTGAACTTTCCTTTGATAATCTCTTACTGCATTAGCAGCAGCACCACCTGCAAGGACGGC	C/T	CTCTTCTAGCTCCCTTACAGTTTGGGTGAGCTTTTCAACCTCTGCCACCTTTTGTCTATGCATTTTGTCCAAAATTTTATTTTCCTCCTGAGAGAAATGG
Ca_LG_2:674716	ACTTTAACACATTACATTCATATCTTGAACTTTCCTTTGATAATCTCTTACTGCATTAGCAGCAGCACCACCTGCAAGGACGGCCTCTTCTAGCTCCCTT	T/C	ACAGTTTGGGTGAGCTTTTCAACCTCTGCCACCTTTTGTCTATGCATTTTGTCCAAAATTTTATTTTCCTCCTGAGAGAAATGGTGATGAAACAAGAAGT
Ca_LG_2:733624	TGGCCCATGAGTTGTTGGAAGAAGGGATTTAATCCCTAAATCTCTATATATAAACAGATACTGCTGCAGCAGCAACATAAGCAGAGAGATCATTGCAACA	A/C	TAGAAGAAGAAAATTGGGTTCCGGCGGTGGATGATTGGTCAGTAATCTTGTTAATTTTCATATGCTGTTCTTATCGCGACTTTGATTATTGATATATTTT
Ca_LG_2:733683	ACTGCTGCAGCAGCAACATAAGCAGAGAGATCATTGCAACATAGAAGAAGAAAATTGGGTTCCGGCGGTGGATGATTGGTCAGTAATCTTGTTAATTTTC	C/A	ATATGCTGTTCTTATCGCGACTTTGATTATTGATATATTTTATTAGTAATTTTGTATTCTCTTTTGAATCGTGATTCAAAGAGAATACAAAACTACGATA
Ca_LG_2:765882	AACCAAAATCTGAAAGCTTAGCATTGTAATCCTGAAAAAAATAAAATGAGTGTGTTCATGTCACTTTATGCTGCCTCTATTTGATCAACATATATTAATT	T/C	GAGTGCATAATGAATGTGTACTTACAGCATCTAATAGAATGTTTGAGGCTTTAATATCTCTATAGATAACTGGTTTCTCTTCTTCATGAAGAAAAGCTAG
Ca_LG_2:765883	ACCAAAATCTGAAAGCTTAGCATTGTAATCCTGAAAAAAATAAAATGAGTGTGTTCATGTCACTTTATGCTGCCTCTATTTGATCAACATATATTAATTG	G/T	AGTGCATAATGAATGTGTACTTACAGCATCTAATAGAATGTTTGAGGCTTTAATATCTCTATAGATAACTGGTTTCTCTTCTTCATGAAGAAAAGCTAGT
Ca_LG_2:813151	CGGCTATGGCTGCAGCACAAGGATTGAGCAACTGCTCAGCCCACCTACCATCCTTGTTATTTCCACTGTTACAGTTACTTCCATTGGCCTTAGCTGCACC	C/T	CTTCTTGTTTCCACCTGGTTTTCTAACCACTGTCCCTTCAAGAGCTGCATCTCCATTATCAGCTTCATTCATCGAACGACTCTTGATTCTCTTGTTCTTT
Ca_LG_2:1099766	CTCATCCCCCTTAAACTTGGGAGGATTGTAACGACGGAAGTCTTCTAATCCTCTTGACTCTGCAGCACGGATCTCTCTTCCCCTCTTTTCAAGATCGCGT	T/C	TGAGTCTTGGCAGCAGTCTGTGCAGCAACAAAAGCAGCCATGTTATTCATAGCCTCCGCCATTTGATCATCCCTATTAGCATTGGCTCTCGGAGCGTCAT
Ca_LG_2:1110794	GTTGAATACCAAGTTAGAGATGCTGCAATGAATATTATTAACAATGTGGGAAAGCTCTCGTTGGTTGATCTTGCAGGGTCAGAGAGAGCTCTTGCCACAG	G/A	ATCAAAGAACACTTAGATCTCTTGAGGGTGCCAACATTAACCGATCTCTTCTTGCACTAAGCTGCTGCATTAATGCTCTTGTAGATGGAAAGAAGCATAT
Ca_LG_2:1110798	AATACCAAGTTAGAGATGCTGCAATGAATATTATTAACAATGTGGGAAAGCTCTCGTTGGTTGATCTTGCAGGGTCAGAGAGAGCTCTTGCCACAGATCA	A/G	AAGAACACTTAGATCTCTTGAGGGTGCCAACATTAACCGATCTCTTCTTGCACTAAGCTGCTGCATTAATGCTCTTGTAGATGGAAAGAAGCATATACCA
Ca_LG_2:2148375	AGATGAGGTGGTGTCGAGCAACCCATGGCCTTGCAAAGTTTGTTGGGGAAGTGTAAACAATTAGTGGATGGGTTACTACTATCCACAAAGATGTTTTTGT	T/C	TGGCTGATGATAATGATAAAGAGTTAGGACAACAGCTAAAAGATGAATTGAATAGGGAATTGCAAGAGCATTTGGAATGCTACACTGCAAGGGCTACCTC

Positions	-100 bp flanking sequences	SNPs within *kabuli* cultivar	+100 bp flanking sequences
Ca_LG_2:2285962	TTATAACATACTGAAGAATATTTAGATGATACTTAAAGATAGAATGCCCAACCTCCGTATCTAGCATTAAGAACAAAGGGCTGTAATGTTCTGGCAGCTA	A/G	CAACGGTAAAATTCCTAGAATTTGTCATCTGGCACAAATCCCCTTTGCAGCATTTCATCATGGACACGGAATGCCTCTTTTAGGTTGTCGTTCGAGACAT
Ca_LG_2:2285980	TATTTAGATGATACTTAAAGATAGAATGCCCAACCTCCGTATCTAGCATTAAGAACAAAGGGCTGTAATGTTCTGGCAGCTACAACGGTAAAATTCCTAG	G/A	AATTTGTCATCTGGCACAAATCCCCTTTGCAGCATTTCATCATGGACACGGAATGCCTCTTTTAGGTTGTCGTTCGAGACATACCCATTTATTAGTAATG
Ca_LG_2:2727167	TCCACCGTTGGTCATAGCACATGGTGGTTTTTCGGGGATATTTCCCGATTCAAGTGGTGCTGCTTATAGTTTGGCCTTACAAACGAGCTTACCGGATGTT	T/C	GCTCTATGGTGTGATGTGCAATTGACAAAAGATGCAGCTGGGATTTGCCTTCCAAATGTCAACCTTTTAAATTCTACTTACGTTTCAATTGCTATCCCAA
Ca_LG_2:2730020	GACCTATAAATGCTCTGTTTCAAATTTAATTTTCTCTTATTTTCCATTTAATAATATTATGTTGTGTTCTTCTCTTATCAGAATGCAGCCTATCTTGCAG	G/A	AAAACCAAGGATTTAGTGTGACCGATAAGGTCATTGATGCTTTGCGCAAAGCAGGTTATGATAAACCAGGGAGCCAAAAGGTTTATATTCAATCCACTAA
Ca_LG_2:2730179	GATAAACCAGGGAGCCAAAAGGTTTATATTCAATCCACTAATAGCTCTGTACTTCTGAAATTCAAGGAGAAAACCAATTACGAGCTTGTCTACAAGATTG	G/T	ACGAGACTGTCAGTGATGCTGCCAACGCAGCTGTTGAGGATATAAAAACGTTTGCGGGTTCTGTGGTTATCAACAAGGACTCGGTATTTCCTCAGAATAG
Ca_LG_2:2989447	TCATGTATTTACCTTTTTACTCATGAAAAAACTTGATTGATTTTCAGAAAAGTTGGTTTTGCTGCATACATAGTTCTCCAGCAGAATCGTAGCAATGATC	C/T	GGTCCTATGTATATGGTTGAATGGAAGTCGAGTCATGTGTAACTAGCTCCTAATGTTTTCTCAAGTATATGAGCTGTTAAGCATAATTAACTCTTCTTGG
Ca_LG_2:3001582	CAGCAAAGAAGGCAGTGGAGAGTGAGCTTAGAAGGTGGCGTGAGAGAGAGCAGAAGAAAGCAGCAGAAACCGCGTCTCGAATATTGGCCGAAACACAAAC	C/T	GTCGCCATCATCATCATTGTCATCACATTTATCTCCTAAGCACTACAGGTTTCAAAAGCAGAATTCAGCTCCAAAAACCATTGAGGTGAGGAAGTTTAAA
Ca_LG_2:3001822	GTATTAGTGCCATCTTTCATAAGAAAAAGAGGCTGCATGTTGAGAAAGGATATCCTTCTTACCTTCCAGGTGAAAATCCTTTGTGAAATTTTGTTTTAGC	C/T	TTTTGTGCAGCTCTTGCATGTGAGAGATTAAACATATTGGTTTATGTGAAAGTTGAGGCCTATGTGATCAGATTTTTGTCATAATCATAGCTTGAGAGAA
Ca_LG_2:3081515	TGGATGAATCTTTTAGGTGTTTACCCTCTTGATAAAAGTCAGTGTTGTTAAATGGTGGTCATGGCGGTTCTATAGGCTGCAATGGCATGTTTATACGGCG	G/A	GATTTTTGTCCTTCCGCCACCGGCAACACTGATCAAAGATTACTTATTTTGTTCATTATTGTAATCTGTTGTGTAACCCGCTTGAGCTGCCACTGTGTCA
Ca_LG_2:3134323	TAGGAGAGCTGCTGTTGAAAAAAGGAGAAAGAGAAATGAGGAAAAGCAGGCGCCCCAAGATGAGTAGCCTCAGCAAAATCCTATGTTTGATGCAGAACAT	T/G	GATGAGCAGCCCCATGATGAGCAGCCCCAGCAAGATCATGTGTTTGATGATGAACATGATCAGCAGCAGCAACAGTTTGATGATGAACATGGTCAGTAGC
Ca_LG_2:3153586	CAGATTTGGTTACCTTACCCTTGTTTTTGTGAGGGTAGTGTATGATTTATTCTGGGCCAAATGATTGGGCCTGTGGACCTTATCTCAATGCGGATCTGAC	C/G	CTCAAATTAAAATTTGTGAAGCTGCTTTTTGCCCCTCTCTTTTCTTAGAACCCTTTTTCGGCATGTAATACTTAATTTTAAAATAATCCTTTTAAAATAT
Ca_LG_2:3158619	TACTTTCGTACAATATATGAATATCATGTCACCACACACTACTGATCCCGTGCCAAGTGTCAAATTCATTGTAATGCAGCTTAAACTCAAATTTTTCTCA	A/G	TGTCTAGCTACCCCGTGAATCAGTAGTAGTGATGTAGTTCCGCTGCATAGTGAAGCACAACTTACCCCAAAATGATAAAATAGAATGACATAGGCCAAGT

Positions	-100 bp flanking sequences	SNPs within *kabuli* cultivar	+100 bp flanking sequences
Ca_LG_2:3158634	TATGAATATCATGTCACCACACACTACTGATCCCGTGCCAAGTGTCAAATTCATTGTAATGCAGCTTAAACTCAAATTTTTCTCATGTCTAGCTACCCCG	G/A	TGAATCAGTAGTAGTGATGTAGTTCCGCTGCATAGTGAAGCACAACTTACCCCAAAATGATAAAATAGAATGACATAGGCCAAGTTTTGGTTAACTTTTG
Ca_LG_2:3229911	CATTGGTTGCATTGTCTTCAGTATTGTCAATTGCGAAAAATAGTTGTTTGTTTGAATTCTGCTATGCTATAGGGCCGCTATAGCTCCTATTAAATAACAT	T/C	TTTGTACTAAATAAGATATTACGGAACAATAACAATTTGTTTAAATTATGCTATACTATCGTGCTAAAATACCGCTGTTTGACAACACTAATTGTCATTC
Ca_LG_2:3229932	TATTGTCAATTGCGAAAAATAGTTGTTTGTTTGAATTCTGCTATGCTATAGGGCCGCTATAGCTCCTATTAAATAACATTTTGTACTAAATAAGATATTA	A/G	CGGAACAATAACAATTTGTTTAAATTATGCTATACTATCGTGCTAAAATACCGCTGTTTGACAACACTAATTGTCATTCACTTAGTTATCAAATTGATTT
Ca_LG_2:3229988	CTATAGCTCCTATTAAATAACATTTTGTACTAAATAAGATATTACGGAACAATAACAATTTGTTTAAATTATGCTATACTATCGTGCTAAAATACCGCTG	G/A	TTTGACAACACTAATTGTCATTCACTTAGTTATCAAATTGATTTGCATAGCAGCCATGTTTTTGGCAAAAGCATTCGGGCGAAGATTCTTTGCTGCAGCT
Ca_LG_2:3406492	TTTCTCTTACATGTGAAACTTTATCCCACTTCCCAGCTGCATCATACACGTTAGAAAGTACAGTGTAACACCCAGTACGAGCATCTGAATCCTCCTCAAC	C/T	CAAACTGTGGGCTGCATATTCACCTATGTCCAAGTTTCCATGGTTCCCAGCACCACCAAGCAAACTCATCCATATGACTTTGTTGGGTCTCACCGGCACC
Ca_LG_2:3413832	GGAATACTTTTTTTAAAATCTCGCGTTTTTGTGTTGTGTTATGAATGATTATGATGATTATTATTATTATTGTTACATTTTGAAGTTTGTTAGTTTTATG	G/A	TTGTTGATATCACATGAAAGCAGCATAGTTAGTTATGTGTTGGATGATGACACTGTTCTTTTCAAGTTTAAATATGTTTGGTGATTCCTGAAGTTGTTCT
Ca_LG_2:3418267	CAATGTGGTGTATGTAAATCAAATTTGCTGCTGCAGGCTAATGAAGTCACAAACTATGGGTGATGCCAACAGCTTTGAATTCATGAGAAGCAGAAGGGTT	T/G	TTTGAGATCAATCCTGACCACCATATTATCAAGAACTTGGATGTATGAATCTTTTTTTGTCATGTATAGTAAGTCTAATTATATAATAAATTGCAAGTTG
Ca_LG_2:3437724	CAAGCATAAGCCTCGATTACCTTGGACGGCAATCAATGCATCTGTGGGGGTATTTGTTATAACCTTGCTTCTGGGACATATATTTTATGCAGCGATAAAT	T/C	CGTATAGCCAAAGTGGAGGAAGACTGTCGTAAAATGGAAGAGCTGAAAGTTCGTGCCGAAGCTGCAGATGTCGCAAAATCTCAGGTTCTCTTGTAAATTC
Ca_LG_2:3442527	GAACCAACCTTGTTATTTTCCAAACACATTGAAAGACCATGCTTCTTATGCCTTCAACAGTTACTTTCAGAAGTTGAAGCATGATGGTGGTTCTTGTTTT	T/C	TTCAGAGGTGCTGCCATTACAACTGAAGTTGATCCTAGTAAGTATTATTCAACAGATTCTTGATAACCACACCCCTCCTTTTTGTGCTTTGGTTTAAACT
Ca_LG_2:3460854	AGGTATCGATGATTCAACATCGGCTTATGCCCGTGGAGGCGGACTACATATAGTCGAAAAGAGGTTATCAAAACTTGCTCAGCATTTCAAAGTGCCATTT	T/C	GAATTTCATGCTGCAGCCATCTCAGGTTGTGATGTTCAACTGCATAACCTTGGAGTTCGACCGGGGGAAGCTCTAGCTGTAAATTTTGCATTTATGCTAC
Ca_LG_2:3836655	GACAGATTCGTACTAGTAGTATTAATTAGACAAGTACAGAACACCATTCCATACTAATTAGTTTCTTTATATCTTCATTTAATCCTCCCTGTTTCAGGTC	C/T	AAGTTTTCACTGACGTAGTTGGCAGCCCATACTATGTTGCTCCCGAGGTTCTCCTCAAGCATTATGGGCCTGAAGCAGATGTTTGGACCGCCGGTGTCAT
Ca_LG_2:4705113	TGATAAAATTTTAGTATGAACTTATAAATTGTTTAGAAAGACATACATTACAGTCAAATGCAAATCTCCTTGCAAGCATGATGGCTGCATTCCTTTCTCG	G/T	CTCTGATTCAAAAGCTAATACAAAAGAATGTCCTTGTTTCGGCTGCCAAAATAGTGCCTGCGCCGCAGCATTTCCACCGCCTCGAACTCCACAAAGCTAA

Positions	-100 bp flanking sequences	SNPs within *kabuli* cultivar	+100 bp flanking sequences
Ca_LG_2:4705138	AAATTGTTTAGAAAGACATACATTACAGTCAAATGCAAATCTCCTTGCAAGCATGATGGCTGCATTCCTTTCTCGCTCTGATTCAAAAGCTAATACAAAA	A/T	GAATGTCCTTGTTTCGGCTGCCAAAATAGTGCCTGCGCCGCAGCATTTCCACCGCCTCGAACTCCACAAAGCTAAAGAACACATTACCAAAGCACATTGG
Ca_LG_2:4705141	TTGTTTAGAAAGACATACATTACAGTCAAATGCAAATCTCCTTGCAAGCATGATGGCTGCATTCCTTTCTCGCTCTGATTCAAAAGCTAATACAAAAGAA	A/G	TGTCCTTGTTTCGGCTGCCAAAATAGTGCCTGCGCCGCAGCATTTCCACCGCCTCGAACTCCACAAAGCTAAAGAACACATTACCAAAGCACATTGGTTT
Ca_LG_2:4705142	TGTTTAGAAAGACATACATTACAGTCAAATGCAAATCTCCTTGCAAGCATGATGGCTGCATTCCTTTCTCGCTCTGATTCAAAAGCTAATACAAAAGAAT	T/A	GTCCTTGTTTCGGCTGCCAAAATAGTGCCTGCGCCGCAGCATTTCCACCGCCTCGAACTCCACAAAGCTAAAGAACACATTACCAAAGCACATTGGTTTC
Ca_LG_2:4705153	ACATACATTACAGTCAAATGCAAATCTCCTTGCAAGCATGATGGCTGCATTCCTTTCTCGCTCTGATTCAAAAGCTAATACAAAAGAATGTCCTTGTTTC	C/T	GGCTGCCAAAATAGTGCCTGCGCCGCAGCATTTCCACCGCCTCGAACTCCACAAAGCTAAAGAACACATTACCAAAGCACATTGGTTTCCCAAATTCAAG
Ca_LG_2:4705156	TACATTACAGTCAAATGCAAATCTCCTTGCAAGCATGATGGCTGCATTCCTTTCTCGCTCTGATTCAAAAGCTAATACAAAAGAATGTCCTTGTTTCGGC	C/T	TGCCAAAATAGTGCCTGCGCCGCAGCATTTCCACCGCCTCGAACTCCACAAAGCTAAAGAACACATTACCAAAGCACATTGGTTTCCCAAATTCAAGTAG
Ca_LG_2:4765828	TGCAAGGGCACACAAAAAATAAGGAAAAGACAGAAAAAGAACATTGCAGTAATCTGCATAGTAAAAGAGAACATAAATTGAATCCTAATTTCGGTTAGAG	G/T	AAATAAAAATACATTAGACATAAAACACAGGATCTGAAAAAGGGATGAAATAGCATGCGGAAAAAGATAACCTTGTAATCATAAAGATCGCTACCAGCAT
Ca_LG_2:4765841	AAAAAATAAGGAAAAGACAGAAAAAGAACATTGCAGTAATCTGCATAGTAAAAGAGAACATAAATTGAATCCTAATTTCGGTTAGAGAAATAAAAATACA	A/G	TTAGACATAAAACACAGGATCTGAAAAAGGGATGAAATAGCATGCGGAAAAAGATAACCTTGTAATCATAAAGATCGCTACCAGCATAACCACTACTTTC
Ca_LG_2:4765859	AGAAAAAGAACATTGCAGTAATCTGCATAGTAAAAGAGAACATAAATTGAATCCTAATTTCGGTTAGAGAAATAAAAATACATTAGACATAAAACACAGG	G/A	ATCTGAAAAAGGGATGAAATAGCATGCGGAAAAAGATAACCTTGTAATCATAAAGATCGCTACCAGCATAACCACTACTTTCACTTAATTTTCCGCTGTG
Ca_LG_2:4765863	AAAGAACATTGCAGTAATCTGCATAGTAAAAGAGAACATAAATTGAATCCTAATTTCGGTTAGAGAAATAAAAATACATTAGACATAAAACACAGGATCT	T/C	GAAAAAGGGATGAAATAGCATGCGGAAAAAGATAACCTTGTAATCATAAAGATCGCTACCAGCATAACCACTACTTTCACTTAATTTTCCGCTGTGAACA
Ca_LG_2:4765864	AAGAACATTGCAGTAATCTGCATAGTAAAAGAGAACATAAATTGAATCCTAATTTCGGTTAGAGAAATAAAAATACATTAGACATAAAACACAGGATCTG	G/A	AAAAAGGGATGAAATAGCATGCGGAAAAAGATAACCTTGTAATCATAAAGATCGCTACCAGCATAACCACTACTTTCACTTAATTTTCCGCTGTGAACAC
Ca_LG_2:4765873	GCAGTAATCTGCATAGTAAAAGAGAACATAAATTGAATCCTAATTTCGGTTAGAGAAATAAAAATACATTAGACATAAAACACAGGATCTGAAAAAGGGA	A/T	TGAAATAGCATGCGGAAAAAGATAACCTTGTAATCATAAAGATCGCTACCAGCATAACCACTACTTTCACTTAATTTTCCGCTGTGAACACGTTCAACAT
Ca_LG_2:4765970	GGATGAAATAGCATGCGGAAAAAGATAACCTTGTAATCATAAAGATCGCTACCAGCATAACCACTACTTTCACTTAATTTTCCGCTGTGAACACGTTCAA	A/G	CATCTTCATCTGTCTCTTGATCAACTTCATTCTCTGCATCATGGTAACCATTTCCTTTCACATCTATATTGTCGTCATCGGTCGAATCTTCGGTCCCGCT

**Positions**	**-100 bp flanking sequences**	**SNPs within *kabuli* cultivar**	**+100 bp flanking sequences**
Ca_LG_2:4766417	AATAATTGCTTGAGCGTTATTTGCGGCATTAGCGGCGGCAAGAAGCTGAGCTGAACTATCACCACTACCATCAAATTCATCCTCCAGTTCACATTCTCCA	A/G	GCTAAGAGAGGTCGTAAAAGCAAGGGAGCCATGCAAGCAGCAACAGCAGAAGCAGTCATCCTATTCTCGTTACAATGAGAAGCAACCGTGTGCATCATCT
Ca_LG_2:4766429	AGCGTTATTTGCGGCATTAGCGGCGGCAAGAAGCTGAGCTGAACTATCACCACTACCATCAAATTCATCCTCCAGTTCACATTCTCCAGCTAAGAGAGGT	T/C	CGTAAAAGCAAGGGAGCCATGCAAGCAGCAACAGCAGAAGCAGTCATCCTATTCTCGTTACAATGAGAAGCAACCGTGTGCATCATCTTCAAAATTCTGT
Ca_LG_2:4766430	GCGTTATTTGCGGCATTAGCGGCGGCAAGAAGCTGAGCTGAACTATCACCACTACCATCAAATTCATCCTCCAGTTCACATTCTCCAGCTAAGAGAGGTC	C/T	GTAAAAGCAAGGGAGCCATGCAAGCAGCAACAGCAGAAGCAGTCATCCTATTCTCGTTACAATGAGAAGCAACCGTGTGCATCATCTTCAAAATTCTGTA
Ca_LG_2:5498748	CTTATATTATCACAAGAATTGCAGCAATTAGAAAATTTTACATCAGCTTTCAAATCAGATGTTAAACCTTTATGTCACTACTCTACTTCAAAAGAACTTT	T/G	TACTTTCAAATTATGTAGTTTTAAATTGGGGTCCGCAATTGTAGAGATACCAACTCTATTTGTTTGTAATTTTTGTGATATCAAAGTTTGCTACATAACT
Ca_LG_2:5498863	TAGTTTTAAATTGGGGTCCGCAATTGTAGAGATACCAACTCTATTTGTTTGTAATTTTTGTGATATCAAAGTTTGCTACATAACTGCGACAGCAATCACT	T/C	GAAAGCTTATATAATTCAATGACATTGTGAAAATTTGTATATTTTGCAGGGTGTAGTTTATTCTTTGTATAGCGAAGAACATGGCGTCACTTGGTGATAT
Ca_LG_2:5646437	ATGCAATCCAGATAGAGGAGAATAAGGTAGAAAGTATCAAGAGGGACAATAGAAAAACATCAAAATGAACTTGCAGCACAAAAAAGTATTACACTAATGC	C/A	TTTGGCTGCTGCTAAGGAGGCTAAAGCATTAGCAGAGGCTTAAGCAAATAATTAAGCAAGAATTGATCTAGAGATTCGTCTAAGAGAAGTCGAGGAACAT
Ca_LG_2:5831254	TTCTTCCCTTGAATTAGATTGTGTTGAACCTGTTGCTAGCCCTCTTCCTAAAATGATCATTGTAGCGTCCGTCGCTGCTGGTGTTCAGTTTGGATGGGCG	G/A	CTACAGCTCTCATTGTTGACCCCTTACGTGCAGACTCTTGGCGTTCCGCACATATGGGCATCTTTTATTTGGCTCTGTGGTCCAATGTCGGGTCTTCTTG
Ca_LG_2:5831260	CCTTGAATTAGATTGTGTTGAACCTGTTGCTAGCCCTCTTCCTAAAATGATCATTGTAGCGTCCGTCGCTGCTGGTGTTCAGTTTGGATGGGCGCTACAG	G/A	CTCTCATTGTTGACCCCTTACGTGCAGACTCTTGGCGTTCCGCACATATGGGCATCTTTTATTTGGCTCTGTGGTCCAATGTCGGGTCTTCTTGTTCAAC
Ca_LG_2:5831356	ACAGCTCTCATTGTTGACCCCTTACGTGCAGACTCTTGGCGTTCCGCACATATGGGCATCTTTTATTTGGCTCTGTGGTCCAATGTCGGGTCTTCTTGTT	T/G	CAACCCGTTGTTGGTTATTATAGTGATCGAAGTACGTCACGCTTCGGCCGCCGTCGTCCATTTATCTTCTTTGGTACCCTCTCTGTCGCCGTTGCTGTCT
Ca_LG_2:5831363	TCATTGTTGACCCCTTACGTGCAGACTCTTGGCGTTCCGCACATATGGGCATCTTTTATTTGGCTCTGTGGTCCAATGTCGGGTCTTCTTGTTCAACCCG	G/A	TTGTTGGTTATTATAGTGATCGAAGTACGTCACGCTTCGGCCGCCGTCGTCCATTTATCTTCTTTGGTACCCTCTCTGTCGCCGTTGCTGTCTTTCTTAT
Ca_LG_2:5831367	TGTTGACCCCTTACGTGCAGACTCTTGGCGTTCCGCACATATGGGCATCTTTTATTTGGCTCTGTGGTCCAATGTCGGGTCTTCTTGTTCAACCCGTTGT	T/C	TGGTTATTATAGTGATCGAAGTACGTCACGCTTCGGCCGCCGTCGTCCATTTATCTTCTTTGGTACCCTCTCTGTCGCCGTTGCTGTCTTTCTTATTGGT
Ca_LG_2:5831389	TCTTGGCGTTCCGCACATATGGGCATCTTTTATTTGGCTCTGTGGTCCAATGTCGGGTCTTCTTGTTCAACCCGTTGTTGGTTATTATAGTGATCGAAGT	T/C	ACGTCACGCTTCGGCCGCCGTCGTCCATTTATCTTCTTTGGTACCCTCTCTGTCGCCGTTGCTGTCTTTCTTATTGGTTACGCCGCTGACCTCGGTCACT

Positions	-100 bp flanking sequences	SNPs within *kabuli* cultivar	+100 bp flanking sequences
Ca_LG_2:5831392	TGGCGTTCCGCACATATGGGCATCTTTTATTTGGCTCTGTGGTCCAATGTCGGGTCTTCTTGTTCAACCCGTTGTTGGTTATTATAGTGATCGAAGTACG	G/C	TCACGCTTCGGCCGCCGTCGTCCATTTATCTTCTTTGGTACCCTCTCTGTCGCCGTTGCTGTCTTTCTTATTGGTTACGCCGCTGACCTCGGTCACTCTT
Ca_LG_2:5831624	CGAGCTGTTGCTTTCTTTGTCCTCGGATTCTGGATCCTTGACGTAGCAAACAACATGCTCCAGGGACCATGCCGTGCTTTCCTCGCCGACTTAGCAGCCA	A/G	GCGATGAAAAAATGACAAGAACAGGTAACGCGTTCTTCTCGTTCTTCATGGCTGTCGGAAACATTTTGGGATACGCAGCTGGTTCATACAGCAGCCTATA
Ca_LG_2:5873610	TGTAAAGAAGATTTTTGCAATCCTCCAAACTAAGACTTGATAAGTATTTCATATTCTTTCCAAACTTTGGAAGCTTTTTTACTTTTTTGCAGCCTGAAAA	A/G	AATCAATTCCTCTAGAGAATCCATTTCCAATTTTGTTGGAAGGGTTTGAAGATTTATGCAATCTTTCAAGTTCAGTTTGACAAGCTTCTTGTGTTTTCCA
Ca_LG_2:5873617	AAGATTTTTGCAATCCTCCAAACTAAGACTTGATAAGTATTTCATATTCTTTCCAAACTTTGGAAGCTTTTTTACTTTTTTGCAGCCTGAAAAAATCAAT	T/C	TCCTCTAGAGAATCCATTTCCAATTTTGTTGGAAGGGTTTGAAGATTTATGCAATCTTTCAAGTTCAGTTTGACAAGCTTCTTGTGTTTTCCAACAGATT
Ca_LG_2:5873622	TTTTGCAATCCTCCAAACTAAGACTTGATAAGTATTTCATATTCTTTCCAAACTTTGGAAGCTTTTTTACTTTTTTGCAGCCTGAAAAAATCAATTCCTC	C/T	TAGAGAATCCATTTCCAATTTTGTTGGAAGGGTTTGAAGATTTATGCAATCTTTCAAGTTCAGTTTGACAAGCTTCTTGTGTTTTCCAACAGATTTGTGA
Ca_LG_2:5873638	ACTAAGACTTGATAAGTATTTCATATTCTTTCCAAACTTTGGAAGCTTTTTTACTTTTTTGCAGCCTGAAAAAATCAATTCCTCTAGAGAATCCATTTCC	C/A	AATTTTGTTGGAAGGGTTTGAAGATTTATGCAATCTTTCAAGTTCAGTTTGACAAGCTTCTTGTGTTTTCCAACAGATTTGTGAACCTTAACAAGGCTTG
Ca_LG_2:5882661	AAAATACTATATTTTATAGTTATATAACTATTATAATAATAAAATACAATAATACCGATAAAGAAGGTCTCCTTTTAGGTTATCAAATTGGCAGCATGAA	A/T	GTGGAGAATGTCATGTGGAGTATAGATAATTTATAATTTATGGACATCTTTTGGGTGTTTTTTGTCTTTGAAACTTGTTGCCATTAGCTATTGCAGCAGG
Ca_LG_2:5892888	TGCGTGAGACCGCGAGTTCGTGTTCGTCTCCCCCGTGCGCCGTGCTGCTGCTGCGTCCGCCGTCCAGCAGTCTCTGTTCTGCGTGAGTATTCTTCACTGC	C/T	CATTTTGCACTTGATTTGTTGTTGAATTTTCTGTTATCATTATTCATTGCTGCACTTGATTTTCTGTTGGGAGTTGAATTGTTATATCATTGAATTGAAT
Ca_LG_2:5893006	TTGTTGAATTTTCTGTTATCATTATTCATTGCTGCACTTGATTTTCTGTTGGGAGTTGAATTGTTATATCATTGAATTGAATTGAATTGTTTTGAACATG	G/C	TTATCATTATTCATTGCTGCTCTTGATTTTCTGTTGTTGCGCTAAACTTGTTCTCTTATCATTGATGCAAATGAATTGTTGCGAGCTGAACTGAGTTTTG
Ca_LG_2:5901802	AACTGTATAATATTTTATATAGAAGAATGTACCTGTTGTCATGATGGCTGATTTAATAGCTGCTGGACTCCATTCAGGATAAAGTGTCTTGAGAAGACCT	T/A	ACAATACCCGCAACATGAGGACAAGACATAGAAGTTCCTTGTTGCACGTTATAAGGAATTCGTCGTGTATCCTCTGCTAAATTAGATGGACCAACAGCTA
Ca_LG_2:5901811	ATATTTTATATAGAAGAATGTACCTGTTGTCATGATGGCTGATTTAATAGCTGCTGGACTCCATTCAGGATAAAGTGTCTTGAGAAGACCTACAATACCC	C/A	GCAACATGAGGACAAGACATAGAAGTTCCTTGTTGCACGTTATAAGGAATTCGTCGTGTATCCTCTGCTAAATTAGATGGACCAACAGCTAGTGAATAAG
Ca_LG_2:5901850	TGATTTAATAGCTGCTGGACTCCATTCAGGATAAAGTGTCTTGAGAAGACCTACAATACCCGCAACATGAGGACAAGACATAGAAGTTCCTTGTTGCACG	G/A	TTATAAGGAATTCGTCGTGTATCCTCTGCTAAATTAGATGGACCAACAGCTAGTGAATAAGCTGCTAATATGTTCACTCCAGGAGCAGTTATGTCAGGCT

Positions	-100 bp flanking sequences	SNPs within *kabuli* cultivar	+100 bp flanking sequences
Ca_LG_2:5901853	TTTAATAGCTGCTGGACTCCATTCAGGATAAAGTGTCTTGAGAAGACCTACAATACCCGCAACATGAGGACAAGACATAGAAGTTCCTTGTTGCACGTTA	A/G	TAAGGAATTCGTCGTGTATCCTCTGCTAAATTAGATGGACCAACAGCTAGTGAATAAGCTGCTAATATGTTCACTCCAGGAGCAGTTATGTCAGGCTGAA
Ca_LG_2:5901882	AAAGTGTCTTGAGAAGACCTACAATACCCGCAACATGAGGACAAGACATAGAAGTTCCTTGTTGCACGTTATAAGGAATTCGTCGTGTATCCTCTGCTAA	A/G	ATTAGATGGACCAACAGCTAGTGAATAAGCTGCTAATATGTTCACTCCAGGAGCAGTTATGTCAGGCTGAAAGAGTAAACAAAATATTCGTAACCACAAA
Ca_LG_2:5901901	TACAATACCCGCAACATGAGGACAAGACATAGAAGTTCCTTGTTGCACGTTATAAGGAATTCGTCGTGTATCCTCTGCTAAATTAGATGGACCAACAGCT	T/C	AGTGAATAAGCTGCTAATATGTTCACTCCAGGAGCAGTTATGTCAGGCTGAAAGAGTAAACAAAATATTCGTAACCACAAAATGAGTGAAGGAAGGTACT
Ca_LG_2:6282000	CAAAGTTGAACGAAACCGAGCAGCTTCGTGCATCGTAATCCATAGCAAAATGGTTAAAGGTCGACAAGGTGAACGTGTTAGGTTTGTGATCTCACTCTTC	C/T	CAGATTTTTTGTTCTTTAGCTTTCGATCTGTGTGCTGTAAGGGTTTTGGTTAACAATGCTGCATATAAATGTAAATGATAAAACAAAACTTAGCTTTGTT
Ca_LG_2:6314936	ACTAATTGTTGGAAACAGATGTGAAACAGTGAAAGATATAATTTTATTTTACAATGCAAAAAATGTGAGTTTTGATCTTAGCCTAATTAATGAGGTGAAA	A/T	TTAATTTAGTGAAGTACAAAAGCAGCAAGTTGTGACAAGGAATTAGAAAATTTCAAATTGAAAAAGATAAGCTATTTGAATCATTCTGGTATGTTTTGAT
Ca_LG_2:6779817	ACTCCACTCCTAACTCCGCCACCTCAGCCACCAAGCCAACTTTATATCCCCACCTCTTCCTATCAGTGTTGTTAAATGGCAGCCACCGCAGAATGGCGCT	T/G	GTGGATTTCTTTGACAAGGGTCATAGGCAATTTATGAAGGGATGCCTGCCGTGACTTAAAATAAATTGTACATCTGCCTCATCTTTTTTTTAACCCTCTA
Ca_LG_2:6779831	TCCGCCACCTCAGCCACCAAGCCAACTTTATATCCCCACCTCTTCCTATCAGTGTTGTTAAATGGCAGCCACCGCAGAATGGCGCTGTGGATTTCTTTGA	A/G	CAAGGGTCATAGGCAATTTATGAAGGGATGCCTGCCGTGACTTAAAATAAATTGTACATCTGCCTCATCTTTTTTTTAACCCTCTAGCTCCCAGAGTAAT
Ca_LG_2:6779862	ATCCCCACCTCTTCCTATCAGTGTTGTTAAATGGCAGCCACCGCAGAATGGCGCTGTGGATTTCTTTGACAAGGGTCATAGGCAATTTATGAAGGGATGC	C/T	CTGCCGTGACTTAAAATAAATTGTACATCTGCCTCATCTTTTTTTTAACCCTCTAGCTCCCAGAGTAATCCAAAGTTCAAATGGTAGGTAAGTAAAACCT
Ca_LG_2:6936604	TCCACTAGCAACATCCAAGATTAAGGGGTTAAGTCGCATTTGGGGTTGCCTAACTGATTTTACCCCATCCTCCAATAGTATCCTATGCATAATATGGACG	G/T	GACTAATACCAGGAATATCAGCCAATGTCCATCCGATTGCTTTCCTGTGTTTTTTCAAAATTTGCAGCAACTTATCTTCCTGTTCATCTTCAAGTTTGGC
Ca_LG_2:6936630	GGTTAAGTCGCATTTGGGGTTGCCTAACTGATTTTACCCCATCCTCCAATAGTATCCTATGCATAATATGGACGGACTAATACCAGGAATATCAGCCAAT	T/G	GTCCATCCGATTGCTTTCCTGTGTTTTTTCAAAATTTGCAGCAACTTATCTTCCTGTTCATCTTCAAGTTTGGCTGAAATAATCACATGTAATTTTCCAC
Ca_LG_2:7216363	CAGAACAGATATCACTTAGCACTCATACTATTAATAAAATGTTGGGAGTGAGGGTAGAAGGCTAGTCAGGAGTAATAGGAAAATAAACCCAAAATAGCTA	A/G	CAATTCAAACAACCACCTCACCTTAGTACTGTGAGCTAGGCATACGCTATGCCAGTGAAAGAGGAATGAACTGATTTTGGGGCTAAGCTGAGACACGGCG
Ca_LG_2:7216393	TTAATAAAATGTTGGGAGTGAGGGTAGAAGGCTAGTCAGGAGTAATAGGAAAATAAACCCAAAATAGCTACAATTCAAACAACCACCTCACCTTAGTACT	T/C	GTGAGCTAGGCATACGCTATGCCAGTGAAAGAGGAATGAACTGATTTTGGGGCTAAGCTGAGACACGGCGACAATAAATAAATTATGCAAGAAAAACGTG

Positions	-100 bp flanking sequences	SNPs within *kabuli* cultivar	+100 bp flanking sequences
Ca_LG_2:7573082	AATTATTTTCAAATAATGAAGTAAGATTTGATGTGTATATACCATTAGCATCTTTGAGACATTGCTGGCACCAAAAATTTTGTGTACAGAAGCAAACTTC	C/T	TGAGGCTCATGAGGAGAGAAATATGGAGAAAAAGGGCATTCTTGTGCACACCTTCTTCTCAAAAGCTTACATGCTGCACAAGGTGTTGTTGTATTTAATG
Ca_LG_2:7573094	ATAATGAAGTAAGATTTGATGTGTATATACCATTAGCATCTTTGAGACATTGCTGGCACCAAAAATTTTGTGTACAGAAGCAAACTTCTGAGGCTCATGA	A/T	GGAGAGAAATATGGAGAAAAAGGGCATTCTTGTGCACACCTTCTTCTCAAAAGCTTACATGCTGCACAAGGTGTTGTTGTATTTAATGTTCCAGAACCTC
Ca_LG_2:7573097	ATGAAGTAAGATTTGATGTGTATATACCATTAGCATCTTTGAGACATTGCTGGCACCAAAAATTTTGTGTACAGAAGCAAACTTCTGAGGCTCATGAGGA	A/T	GAGAAATATGGAGAAAAAGGGCATTCTTGTGCACACCTTCTTCTCAAAAGCTTACATGCTGCACAAGGTGTTGTTGTATTTAATGTTCCAGAACCTCCTA
Ca_LG_2:7573100	AAGTAAGATTTGATGTGTATATACCATTAGCATCTTTGAGACATTGCTGGCACCAAAAATTTTGTGTACAGAAGCAAACTTCTGAGGCTCATGAGGAGAG	G/A	AAATATGGAGAAAAAGGGCATTCTTGTGCACACCTTCTTCTCAAAAGCTTACATGCTGCACAAGGTGTTGTTGTATTTAATGTTCCAGAACCTCCTAACA
Ca_LG_2:7573106	GATTTGATGTGTATATACCATTAGCATCTTTGAGACATTGCTGGCACCAAAAATTTTGTGTACAGAAGCAAACTTCTGAGGCTCATGAGGAGAGAAATAT	T/A	GGAGAAAAAGGGCATTCTTGTGCACACCTTCTTCTCAAAAGCTTACATGCTGCACAAGGTGTTGTTGTATTTAATGTTCCAGAACCTCCTAACATTCTTC
Ca_LG_2:7573118	ATATACCATTAGCATCTTTGAGACATTGCTGGCACCAAAAATTTTGTGTACAGAAGCAAACTTCTGAGGCTCATGAGGAGAGAAATATGGAGAAAAAGGG	G/A	CATTCTTGTGCACACCTTCTTCTCAAAAGCTTACATGCTGCACAAGGTGTTGTTGTATTTAATGTTCCAGAACCTCCTAACATTCTTCCCTTTTTAAAAC
Ca_LG_2:7573124	CATTAGCATCTTTGAGACATTGCTGGCACCAAAAATTTTGTGTACAGAAGCAAACTTCTGAGGCTCATGAGGAGAGAAATATGGAGAAAAAGGGCATTCT	T/C	TGTGCACACCTTCTTCTCAAAAGCTTACATGCTGCACAAGGTGTTGTTGTATTTAATGTTCCAGAACCTCCTAACATTCTTCCCTTTTTAAAACAACCAT
Ca_LG_2:7573126	TTAGCATCTTTGAGACATTGCTGGCACCAAAAATTTTGTGTACAGAAGCAAACTTCTGAGGCTCATGAGGAGAGAAATATGGAGAAAAAGGGCATTCTTG	G/C	TGCACACCTTCTTCTCAAAAGCTTACATGCTGCACAAGGTGTTGTTGTATTTAATGTTCCAGAACCTCCTAACATTCTTCCCTTTTTAAAACAACCATCT
Ca_LG_2:7573127	TAGCATCTTTGAGACATTGCTGGCACCAAAAATTTTGTGTACAGAAGCAAACTTCTGAGGCTCATGAGGAGAGAAATATGGAGAAAAAGGGCATTCTTGT	T/A	GCACACCTTCTTCTCAAAAGCTTACATGCTGCACAAGGTGTTGTTGTATTTAATGTTCCAGAACCTCCTAACATTCTTCCCTTTTTAAAACAACCATCTC
Ca_LG_2:8024030	ACCAAGATGTTTTCCTTCTGTGTTTCAATGTAAAAGCTTTTGAATTTTCCTTGCATGTCAGACATCAGTGCAACTCTTAATTGAAAATTTTGCCTCTTTA	A/G	AGGCATCATATGTTACTACACCATCATTGCACAATTGTTGCAACTCATAAGCGGCTGTAAATACACATCAATTTTACCTTTTGGATTGTACCTGGAATAT
Ca_LG_2:8024032	CAAGATGTTTTCCTTCTGTGTTTCAATGTAAAAGCTTTTGAATTTTCCTTGCATGTCAGACATCAGTGCAACTCTTAATTGAAAATTTTGCCTCTTTAAG	G/A	GCATCATATGTTACTACACCATCATTGCACAATTGTTGCAACTCATAAGCGGCTGTAAATACACATCAATTTTACCTTTTGGATTGTACCTGGAATATTC
Ca_LG_2:8024059	GTAAAAGCTTTTGAATTTTCCTTGCATGTCAGACATCAGTGCAACTCTTAATTGAAAATTTTGCCTCTTTAAGGCATCATATGTTACTACACCATCATTG	G/C	CACAATTGTTGCAACTCATAAGCGGCTGTAAATACACATCAATTTTACCTTTTGGATTGTACCTGGAATATTCAATGACAAGAACATATAAGGCATCATC

Positions	-100 bp flanking sequences	SNPs within *kabuli* cultivar	+100 bp flanking sequences
Ca_LG_2:8185828	GTATTCTTTCTCTGAAGATGAAGCAGCAGACTTGAGCAACTTAAACTCCTTCTCAGCATCAGCCAATTCCCACCTGGCAAACTCTAAGGCAGCTGCAACA	A/G	CGCTTTTCATCTTGAGTACATTTAGCGGCTTCTGATAGAATACATTCGATCTGCTAGGTTCAAATAATTATTTACAACAGTGAGCAGCAGCAGTGTTGTC
Ca_LG_2:8185921	TGCAACACGCTTTTCATCTTGAGTACATTTAGCGGCTTCTGATAGAATACATTCGATCTGCTAGGTTCAAATAATTATTTACAACAGTGAGCAGCAGCAG	G/A	TGTTGTCCAGCGAAACATCAAAATAAAATATATAAGTGGGGGGATAAGCAGCAGGGGAAACAGGACCTGTTCTTCACTACTTGCAATCCTCGTCTTTGAT
Ca_LG_2:8185937	TCTTGAGTACATTTAGCGGCTTCTGATAGAATACATTCGATCTGCTAGGTTCAAATAATTATTTACAACAGTGAGCAGCAGCAGTGTTGTCCAGCGAAAC	C/T	ATCAAAATAAAATATATAAGTGGGGGGATAAGCAGCAGGGGAAACAGGACCTGTTCTTCACTACTTGCAATCCTCGTCTTTGATTTTTCTATCAAAGAAT
Ca_LG_2:8185979	TGCTAGGTTCAAATAATTATTTACAACAGTGAGCAGCAGCAGTGTTGTCCAGCGAAACATCAAAATAAAATATATAAGTGGGGGGATAAGCAGCAGGGGA	A/T	AACAGGACCTGTTCTTCACTACTTGCAATCCTCGTCTTTGATTTTTCTATCAAAGAATTAAGCTGTTGAAGTTGATCAGCTAAGGTTTGCTTTTCAGACA
Ca_LG_2:8185991	ATAATTATTTACAACAGTGAGCAGCAGCAGTGTTGTCCAGCGAAACATCAAAATAAAATATATAAGTGGGGGGATAAGCAGCAGGGGAAACAGGACCTGT	T/C	TCTTCACTACTTGCAATCCTCGTCTTTGATTTTTCTATCAAAGAATTAAGCTGTTGAAGTTGATCAGCTAAGGTTTGCTTTTCAGACAGTAAAGCGTTAT
Ca_LG_2:8314607	CATAAGCAACAACATCACTAGCCAAACACCAGGCGAAATCTTTAGAAGCAAACACGAAAATCTTATAAGTAAAAGCAGCGAGTGGCTAAAAGACACATCT	T/C	GAATCATGCTCCGTCGTGGCAACGCTCGTTGCTGGTGTTTCCTTCGCCACAACGAATTCTGTCCCCGGCGGAACAACAGATGAAGGCACACTAACACTAG
Ca_LG_2:8314629	CAAACACCAGGCGAAATCTTTAGAAGCAAACACGAAAATCTTATAAGTAAAAGCAGCGAGTGGCTAAAAGACACATCTGAATCATGCTCCGTCGTGGCAA	A/G	CGCTCGTTGCTGGTGTTTCCTTCGCCACAACGAATTCTGTCCCCGGCGGAACAACAGATGAAGGCACACTAACACTAGAAGGCAAACCCGCATTTGATAT
Ca_LG_2:8345045	AGATCCAAAAGAAATGTTTGACACGTGTCATGACCGAAATATTCTTCACATTGCTGCAAACTATGAAAAATATGAATTGGTACAAAATATATTACAAAGT	T/C	CAAATTCGTGAACATCATACGATGATAAATCAAAAGGATAAAGAAGGAAATACACCTTTGCATTTGGCCGCAAGATCATGTCATCCCACAAGTGTTTACT
Ca_LG_2:8345050	CAAAAGAAATGTTTGACACGTGTCATGACCGAAATATTCTTCACATTGCTGCAAACTATGAAAAATATGAATTGGTACAAAATATATTACAAAGTCAAAT	T/C	TCGTGAACATCATACGATGATAAATCAAAAGGATAAAGAAGGAAATACACCTTTGCATTTGGCCGCAAGATCATGTCATCCCACAAGTGTTTACTACTTA
Ca_LG_2:8345053	AAGAAATGTTTGACACGTGTCATGACCGAAATATTCTTCACATTGCTGCAAACTATGAAAAATATGAATTGGTACAAAATATATTACAAAGTCAAATTCG	G/C	TGAACATCATACGATGATAAATCAAAAGGATAAAGAAGGAAATACACCTTTGCATTTGGCCGCAAGATCATGTCATCCCACAAGTGTTTACTACTTAATT
Ca_LG_2:8345059	TGTTTGACACGTGTCATGACCGAAATATTCTTCACATTGCTGCAAACTATGAAAAATATGAATTGGTACAAAATATATTACAAAGTCAAATTCGTGAACA	A/T	TCATACGATGATAAATCAAAAGGATAAAGAAGGAAATACACCTTTGCATTTGGCCGCAAGATCATGTCATCCCACAAGTGTTTACTACTTAATTTGGAAT
Ca_LG_2:8345065	ACACGTGTCATGACCGAAATATTCTTCACATTGCTGCAAACTATGAAAAATATGAATTGGTACAAAATATATTACAAAGTCAAATTCGTGAACATCATAC	C/A	GATGATAAATCAAAAGGATAAAGAAGGAAATACACCTTTGCATTTGGCCGCAAGATCATGTCATCCCACAAGTGTTTACTACTTAATTTGGAATGAAAGA

**Positions**	**-100 bp flanking sequences**	**SNPs within *kabuli* cultivar**	**+100 bp flanking sequences**
Ca_LG_2:8348680	AAACACGTTGGCATTTGTGAGAGTTTTTGCAGCCACAAAAGGCTGCCTTTGTGAGGGCCTTGCAGCCACAAAAGTTTGCCTTTGTGAGGGCATAGGCCGT	T/C	CACAAAGGACAAACTGATTTTAAAAAATTCAGCCTTTGTGAGGGCCCAAGTTGCCACAAAATCCCTTCTTTGTGAGGGTTTAGGCCGCCACAAAATACCT
Ca_LG_2:8348682	ACACGTTGGCATTTGTGAGAGTTTTTGCAGCCACAAAAGGCTGCCTTTGTGAGGGCCTTGCAGCCACAAAAGTTTGCCTTTGTGAGGGCATAGGCCGTCA	A/G	CAAAGGACAAACTGATTTTAAAAAATTCAGCCTTTGTGAGGGCCCAAGTTGCCACAAAATCCCTTCTTTGTGAGGGTTTAGGCCGCCACAAAATACCTCC
Ca_LG_2:8348692	ATTTGTGAGAGTTTTTGCAGCCACAAAAGGCTGCCTTTGTGAGGGCCTTGCAGCCACAAAAGTTTGCCTTTGTGAGGGCATAGGCCGTCACAAAGGACAA	A/G	ACTGATTTTAAAAAATTCAGCCTTTGTGAGGGCCCAAGTTGCCACAAAATCCCTTCTTTGTGAGGGTTTAGGCCGCCACAAAATACCTCCAGATTAAAAA
Ca_LG_2:8348700	GAGTTTTTGCAGCCACAAAAGGCTGCCTTTGTGAGGGCCTTGCAGCCACAAAAGTTTGCCTTTGTGAGGGCATAGGCCGTCACAAAGGACAAACTGATTT	T/A	TAAAAAATTCAGCCTTTGTGAGGGCCCAAGTTGCCACAAAATCCCTTCTTTGTGAGGGTTTAGGCCGCCACAAAATACCTCCAGATTAAAAAATAAATAT
Ca_LG_2:8567932	ACATCCAGCAACTAGACATGAACAATACATTAATTATGGTTTGTTAGAGGACGAAGTTTACATGCACCGGCAGCTGGGTTTTGGGGCAGCAGATAAAACT	T/G	TCGTTTATGAGAGACATTAAGATGTCAACAATTCACATCTTGGCGTAAAAAAAAAATATCCCCAAGAAAAGGTCTTATCCATTAAGACAAGGTTTCTATC
Ca_LG_2:8568028	AACTTCGTTTATGAGAGACATTAAGATGTCAACAATTCACATCTTGGCGTAAAAAAAAAATATCCCCAAGAAAAGGTCTTATCCATTAAGACAAGGTTTC	C/T	TATCAGTGTTGTCAGATAGCATCTATAGCGGCGCTATAGTGCTATAACATAGCAGAAATTGAAACAAAACGCTATTGTTCCGCGGTATGCTATTTAGTAC
Ca_LG_2:9337072	ATTTATATCTAAGTCCTCCACATATAGTTCCTCTAATTATATTTTCTAAACGAATGAGAGCAATCCAAATTGATCTTGTAAGAGATTTGTATCGGAACGG	G/A	ATACATTTATTTTTCAAATGATTCATATCATCAAGTGTACCCATTCCAAATTTCATTCCAATCAAATGATCTGCCGCTATCAATATATCTCGCGGCAACA
Ca_LG_2:9337077	TATCTAAGTCCTCCACATATAGTTCCTCTAATTATATTTTCTAAACGAATGAGAGCAATCCAAATTGATCTTGTAAGAGATTTGTATCGGAACGGATACA	A/G	TTTATTTTTCAAATGATTCATATCATCAAGTGTACCCATTCCAAATTTCATTCCAATCAAATGATCTGCCGCTATCAATATATCTCGCGGCAACAAAAAT
Ca_LG_2:9444110	CTTAAAACTAAATATCTGTTGTAAATTTGCAGTGAAGGAATCAAAAGAATACCCAGCATTGAAAGAATAGCAGCACGAGCTGCCAAAAGCTTTGCATCCT	T/C	TTTTGCTTCTAGCCACATCTCCGGTGTAACTTGAACCAATAAAATCCACAGTACATACAAAAACTGGAACCACTTCTTGGAGCTGAACAGTGTTGTATGT
Ca_LG_2:9444132	AAATTTGCAGTGAAGGAATCAAAAGAATACCCAGCATTGAAAGAATAGCAGCACGAGCTGCCAAAAGCTTTGCATCCTTTTTGCTTCTAGCCACATCTCC	C/T	GGTGTAACTTGAACCAATAAAATCCACAGTACATACAAAAACTGGAACCACTTCTTGGAGCTGAACAGTGTTGTATGTAGGTAGTTCCTTATTCAACTTT
Ca_LG_2:9444140	AGTGAAGGAATCAAAAGAATACCCAGCATTGAAAGAATAGCAGCACGAGCTGCCAAAAGCTTTGCATCCTTTTTGCTTCTAGCCACATCTCCGGTGTAAC	C/T	TTGAACCAATAAAATCCACAGTACATACAAAAACTGGAACCACTTCTTGGAGCTGAACAGTGTTGTATGTAGGTAGTTCCTTATTCAACTTTGCAGCATA
Ca_LG_2:9444151	CAAAAGAATACCCAGCATTGAAAGAATAGCAGCACGAGCTGCCAAAAGCTTTGCATCCTTTTTGCTTCTAGCCACATCTCCGGTGTAACTTGAACCAATA	A/G	AAATCCACAGTACATACAAAAACTGGAACCACTTCTTGGAGCTGAACAGTGTTGTATGTAGGTAGTTCCTTATTCAACTTTGCAGCATACTCGTTCAAGA

Positions	-100 bp flanking sequences	SNPs within *kabuli* cultivar	+100 bp flanking sequences
Ca_LG_2:9444161	CCCAGCATTGAAAGAATAGCAGCACGAGCTGCCAAAAGCTTTGCATCCTTTTTGCTTCTAGCCACATCTCCGGTGTAACTTGAACCAATAAAATCCACAG	G/A	TACATACAAAAACTGGAACCACTTCTTGGAGCTGAACAGTGTTGTATGTAGGTAGTTCCTTATTCAACTTTGCAGCATACTCGTTCAAGACCTGCTTGCA
Ca_LG_2:9444163	CAGCATTGAAAGAATAGCAGCACGAGCTGCCAAAAGCTTTGCATCCTTTTTGCTTCTAGCCACATCTCCGGTGTAACTTGAACCAATAAAATCCACAGTA	A/G	CATACAAAAACTGGAACCACTTCTTGGAGCTGAACAGTGTTGTATGTAGGTAGTTCCTTATTCAACTTTGCAGCATACTCGTTCAAGACCTGCTTGCAAA
Ca_LG_2:9885743	GTACTTCAGATGTCTTTTGCATTTTTCTTTGTGAAGATCTAGTTTTCGCTATAGCTGCATTTGGTTTCAGTGATTCAATTTTTGCTTCCTCTTTTGGCTG	G/T	GGAGTCCGGAGTTGTAGGTACTTCAGACATCTTTTCCATTTTTCTCTGTGAAGATCTAGTTTTCGCTATAGCTGCCTTTGGTTTCAGTGATTCAATTTTT
Ca_LG_2:9885752	ATGTCTTTTGCATTTTTCTTTGTGAAGATCTAGTTTTCGCTATAGCTGCATTTGGTTTCAGTGATTCAATTTTTGCTTCCTCTTTTGGCTGGGAGTCCGG	G/A	AGTTGTAGGTACTTCAGACATCTTTTCCATTTTTCTCTGTGAAGATCTAGTTTTCGCTATAGCTGCCTTTGGTTTCAGTGATTCAATTTTTGCTTCCTCT
Ca_LG_2:9885771	TTGTGAAGATCTAGTTTTCGCTATAGCTGCATTTGGTTTCAGTGATTCAATTTTTGCTTCCTCTTTTGGCTGGGAGTCCGGAGTTGTAGGTACTTCAGAC	C/T	ATCTTTTCCATTTTTCTCTGTGAAGATCTAGTTTTCGCTATAGCTGCCTTTGGTTTCAGTGATTCAATTTTTGCTTCCTCTTTTGGCTCAGAGTCTGGAG
Ca_LG_2:9885779	ATCTAGTTTTCGCTATAGCTGCATTTGGTTTCAGTGATTCAATTTTTGCTTCCTCTTTTGGCTGGGAGTCCGGAGTTGTAGGTACTTCAGACATCTTTTC	C/G	CATTTTTCTCTGTGAAGATCTAGTTTTCGCTATAGCTGCCTTTGGTTTCAGTGATTCAATTTTTGCTTCCTCTTTTGGCTCAGAGTCTGGAGTTGTAGGT
Ca_LG_2:10142007	AATATACATTGTTTTCTGAATCTTTTCCAATTGAGACAGACAATATATCGACCCCATCAGCAATAGCATCGTCAAATGCAGCAAGAATATTTGCATCATT	T/C	GCAACCACTAGACCAACATGCTTTGTAGACAGCAATGCGAGCTGAGGGAGCACCACCTCTTGCTGTTCCTTGTGCAAAGCCTAGCATGCTTGCCTGGCTA
Ca_LG_2:10142008	ATATACATTGTTTTCTGAATCTTTTCCAATTGAGACAGACAATATATCGACCCCATCAGCAATAGCATCGTCAAATGCAGCAAGAATATTTGCATCATTG	G/A	CAACCACTAGACCAACATGCTTTGTAGACAGCAATGCGAGCTGAGGGAGCACCACCTCTTGCTGTTCCTTGTGCAAAGCCTAGCATGCTTGCCTGGCTAA
Ca_LG_2:10142011	TACATTGTTTTCTGAATCTTTTCCAATTGAGACAGACAATATATCGACCCCATCAGCAATAGCATCGTCAAATGCAGCAAGAATATTTGCATCATTGCAA	A/C	CCACTAGACCAACATGCTTTGTAGACAGCAATGCGAGCTGAGGGAGCACCACCTCTTGCTGTTCCTTGTGCAAAGCCTAGCATGCTTGCCTGGCTAACCG
Ca_LG_2:10142017	GTTTTCTGAATCTTTTCCAATTGAGACAGACAATATATCGACCCCATCAGCAATAGCATCGTCAAATGCAGCAAGAATATTTGCATCATTGCAACCACTA	A/G	GACCAACATGCTTTGTAGACAGCAATGCGAGCTGAGGGAGCACCACCTCTTGCTGTTCCTTGTGCAAAGCCTAGCATGCTTGCCTGGCTAACCGGGTTCC
Ca_LG_2:10142018	TTTTCTGAATCTTTTCCAATTGAGACAGACAATATATCGACCCCATCAGCAATAGCATCGTCAAATGCAGCAAGAATATTTGCATCATTGCAACCACTAG	G/A	ACCAACATGCTTTGTAGACAGCAATGCGAGCTGAGGGAGCACCACCTCTTGCTGTTCCTTGTGCAAAGCCTAGCATGCTTGCCTGGCTAACCGGGTTCCC
Ca_LG_2:10142027	TCTTTTCCAATTGAGACAGACAATATATCGACCCCATCAGCAATAGCATCGTCAAATGCAGCAAGAATATTTGCATCATTGCAACCACTAGACCAACATG	G/A	CTTTGTAGACAGCAATGCGAGCTGAGGGAGCACCACCTCTTGCTGTTCCTTGTGCAAAGCCTAGCATGCTTGCCTGGCTAACCGGGTTCCCTGCTGCAGT

Positions	-100 bp flanking sequences	SNPs within *kabuli* cultivar	+100 bp flanking sequences
Ca_LG_2:11196986	ATAATCGACAATCGAAGTGAGAAAATGCATCCAAAACAGGAGAGACAGTGGTTGTTTCGGAAAACTTTTCTAGCAGCGATGGCAGGCAGCAGCAGCAAAA	A/C	GGCAGCAAACGGCGGCAACGGCAGTAGCAGACGGGGCAGCAGCGACGATTGTGGACGGAAGTGATAGTAGTCAGAAAAAGGAATGATCATGCTAGAAAAG
Ca_LG_2:11852495	ACAATGTAAAAATTGACCAACTTCGCTAAATTGAGGTGTCTTATCCTCTTTATTCCTTTGTGGCGGCCTTTGCAGCCACAAAATGCAACAGACCAGGCTT	T/C	TTGTGGCGGCCTTTGCAGCCACAAACTGCAATGTCACTTCAAGTATTTGTAGCTGCCTTTACCCTCACAAAATTTGTATTTTGTGGCTGAAAAAGCCCTC
Ca_LG_2:11852506	ATTGACCAACTTCGCTAAATTGAGGTGTCTTATCCTCTTTATTCCTTTGTGGCGGCCTTTGCAGCCACAAAATGCAACAGACCAGGCTTTTGTGGCGGCC	C/A	TTTGCAGCCACAAACTGCAATGTCACTTCAAGTATTTGTAGCTGCCTTTACCCTCACAAAATTTGTATTTTGTGGCTGAAAAAGCCCTCACAAAAATCTT
Ca_LG_2:13061998	GCAACAACCATCAGAAAATTGCTTCAAAATAATTAAGTGGCAGCAGTTCTGCTGAACAGCTTTAGATATTCAGTTGGGATTTCTAACTTTTCATGTAGTG	G/A	ACATTTTTAATATAAAGTAATTAAGTTAAGCTCTCATAATTGTGATGAAGGCAATAATATCATTGCCCTCGCTGTTTGCAGCTGATACCGTATATTAAGT
Ca_LG_2:13593546	AAATCACCAACACTAAGCTTTGGTTTTGCGAAACAGAGGCAGGGAAGAAGAAACAGTTCTCGCGAGAAGGAGGAAATGTTGTTCTTTCACTAACACTTGG	G/A	TTTTCTTGAAGCCAAAGAAAGAAAAAGAAAGGAGAAGAAGAAAGGATTCGCGAGCAGGGAGGAAACGTTTCTGCTTTCTTCCTTTTCTTATCCTTGCTTC
Ca_LG_2:13593566	TGGTTTTGCGAAACAGAGGCAGGGAAGAAGAAACAGTTCTCGCGAGAAGGAGGAAATGTTGTTCTTTCACTAACACTTGGTTTTCTTGAAGCCAAAGAAA	A/G	GAAAAAGAAAGGAGAAGAAGAAAGGATTCGCGAGCAGGGAGGAAACGTTTCTGCTTTCTTCCTTTTCTTATCCTTGCTTCTGATATAACTATGTTTATAC
Ca_LG_2:13593589	GAAGAAGAAACAGTTCTCGCGAGAAGGAGGAAATGTTGTTCTTTCACTAACACTTGGTTTTCTTGAAGCCAAAGAAAGAAAAAGAAAGGAGAAGAAGAAA	A/G	GGATTCGCGAGCAGGGAGGAAACGTTTCTGCTTTCTTCCTTTTCTTATCCTTGCTTCTGATATAACTATGTTTATACATTACTTACTTACTAAGTAATAT
Ca_LG_2:13593592	GAAGAAACAGTTCTCGCGAGAAGGAGGAAATGTTGTTCTTTCACTAACACTTGGTTTTCTTGAAGCCAAAGAAAGAAAAAGAAAGGAGAAGAAGAAAGGA	A/T	TTCGCGAGCAGGGAGGAAACGTTTCTGCTTTCTTCCTTTTCTTATCCTTGCTTCTGATATAACTATGTTTATACATTACTTACTTACTAAGTAATATATA
Ca_LG_2:13744687	TCTCACTCATCTCGGATGTATCTTTGATGATGACCTCCATTGCCTTGAACTCCATCTCGTCCTCTTTTAGTTGTGCCAGTTTTTCTAAGGCTGCTACTTC	C/T	TTTATTCATTGTATCTTGCACAACATTATTAATAAGAGGTGTTTCAACCGCCTTTGCCTTGCCCATTCTTTTTGTTTCCTTTTGTCCCATTAGACGCTCC
Ca_LG_2:13744713	ATGATGACCTCCATTGCCTTGAACTCCATCTCGTCCTCTTTTAGTTGTGCCAGTTTTTCTAAGGCTGCTACTTCTTTATTCATTGTATCTTGCACAACAT	T/G	TATTAATAAGAGGTGTTTCAACCGCCTTTGCCTTGCCCATTCTTTTTGTTTCCTTTTGTCCCATTAGACGCTCCATTGGTGATGTTGAGTCAATTTCATA
Ca_LG_2:14097659	TGGATAGAATCTTCATTTCATTTTTAATCCACCATTGTCACTCATGAATAACCTATGTAGGATTGATTAATGAAGCCAAACATGTGCTGCATATATTTCG	G/A	TTTCTGCTTACTCATAGGGTGTTTATGCTCTTCACCTGTCATGATGCAGGTCTCTATGTTCTTGCTGCTGAATCTGTGCAAATTCCTTGGTTTGATTTTG
Ca_LG_2:15037375	CTGTAAAGTCCATTGAAAAAGATATAGAAACACAATAATATCCAATAGGAAAGATTTTTAAAATGAAAATAAAAAAGTATTCTAGACACCTAAAGTCGAG	G/A	AATTGAGAGATCTTTCAACTGCAGCCCAAGCTTCAGCTCTTTTGGCATACATATCAGGTGAATTCTAGTCTAAAATAGCAATTCTCGGACAATAATGATC

**Positions**	**-100 bp flanking sequences**	**SNPs within *kabuli* cultivar**	**+100 bp flanking sequences**
Ca_LG_2:15620896	GAAAGAAGAACAAGCACACTTGGAGAGCGCATTACAATGGATAGTCGTATGTTGCGTTCGAAAGGATAAATCATTCCTGAAAAGGAAACTATTGATTCTC	C/T	TCCCAATTGGATGGACCGTAGGTGTGATGATTTACATCATGGGTGAGGTCTTAGTTTTAAATTCAGGATGGCCCAACTGCGCCAAGGAAAAGAATAAAAG
Ca_LG_2:15620921	AGCGCATTACAATGGATAGTCGTATGTTGCGTTCGAAAGGATAAATCATTCCTGAAAAGGAAACTATTGATTCTCTCCCAATTGGATGGACCGTAGGTGT	T/C	GATGATTTACATCATGGGTGAGGTCTTAGTTTTAAATTCAGGATGGCCCAACTGCGCCAAGGAAAAGAATAAAAGACTAATTTGACTTTTTCATGCATGC
Ca_LG_2:15620932	ATGGATAGTCGTATGTTGCGTTCGAAAGGATAAATCATTCCTGAAAAGGAAACTATTGATTCTCTCCCAATTGGATGGACCGTAGGTGTGATGATTTACA	A/T	TCATGGGTGAGGTCTTAGTTTTAAATTCAGGATGGCCCAACTGCGCCAAGGAAAAGAATAAAAGACTAATTTGACTTTTTCATGCATGCTCCAACTTTGC
Ca_LG_2:15620936	ATAGTCGTATGTTGCGTTCGAAAGGATAAATCATTCCTGAAAAGGAAACTATTGATTCTCTCCCAATTGGATGGACCGTAGGTGTGATGATTTACATCAT	T/C	GGGTGAGGTCTTAGTTTTAAATTCAGGATGGCCCAACTGCGCCAAGGAAAAGAATAAAAGACTAATTTGACTTTTTCATGCATGCTCCAACTTTGCTCGA
Ca_LG_2:15631359	AGTGGCATCGTTTAAAGTGTCAATAAGTGGCATCATATAATCCAAGTAGTTATTATTGTTTAATATTCTGCTGCAGGTTAAGGTTGGAATCCAAGGGGCT	T/C	GAAGAGTTTGGTGAGTTTGAGGAATGGTTCAATTACAGGCTATCAAGAGCACGAGAGCAGCACCTGAAACATGTGCTGATTTCCTTGGAAGTTTCATAGC
Ca_LG_2:15631404	GTAGTTATTATTGTTTAATATTCTGCTGCAGGTTAAGGTTGGAATCCAAGGGGCTGAAGAGTTTGGTGAGTTTGAGGAATGGTTCAATTACAGGCTATCA	A/T	AGAGCACGAGAGCAGCACCTGAAACATGTGCTGATTTCCTTGGAAGTTTCATAGCTGATAAAACTAACTCACAATTCATAAAAGGTGGAAAATGGCTTGT
Ca_LG_2:16592886	AGAGATAACTGGAGGTTGGTATCATGTTGATAAGATTTATGGCCGGCAGCAGAGAGTGAAGATTGCAGCGGTGACGACCGGTGAGGTTTCTCGTTGAAGA	A/G	AAGGTTGTTAGATGAAGATTGATGCGGTAGCGGCGGCGGCGAGGTTGCTCGTTAAAGGAAGATTGCTCATTGAAGATTGCAGCGGCGGCGGCGGCGAGGT
Ca_LG_2:16592918	AGATTTATGGCCGGCAGCAGAGAGTGAAGATTGCAGCGGTGACGACCGGTGAGGTTTCTCGTTGAAGAAAGGTTGTTAGATGAAGATTGATGCGGTAGCG	G/A	GCGGCGGCGAGGTTGCTCGTTAAAGGAAGATTGCTCATTGAAGATTGCAGCGGCGGCGGCGGCGAGGTTGCAAATGGAGGTGTTGGGTGGACAGGAGGGT
Ca_LG_2:17538199	ACTCCAGCAGCTTCGTCATAATGTCAGAAGATGCTTCAACTGGTGCAGATGGTTGGGCACTTGCTTGGGCTTGATCAGCACTGGGTTGTGCATGATCAGT	T/C	AGTAGATGGAGTTCTTGTCCATACTCCATTAATGCGTCGCAGCTGCATTTGGTTCACAATTGATTCACCAAATGTCAGTGTGGTTTTGAGAGATTCTTCA
Ca_LG_2:17629707	CTCAAAGGCAGCAGCCCAAGTTTCATAATTTGCAGCACCTACCAATTTTTTCAAAAAGAATCAAAGAGTAAAGAAAGTGAAAGCGAAAGCGAAAGATGAA	A/G	GAAGCAGCAAATCCGGTGTCGGACATGAAGTTCTATGTGAGATGAAGAAGTTTGTTGTAAAATCATCAAAAGATCAAAAACACCTCCTAATGTGGCATAG
Ca_LG_2:17629794	AGCGAAAGATGAAGAAGCAGCAAATCCGGTGTCGGACATGAAGTTCTATGTGAGATGAAGAAGTTTGTTGTAAAATCATCAAAAGATCAAAAACACCTCC	C/T	TAATGTGGCATAGAGGGTTCTGCAGTGGTCAGCACAAGAAGCTGCTTGCTGGAGTGGTGCTGCGTATGTTTCTGGTGGTGGCGCATGGATCCCATGCACA
Ca_LG_2:18051569	TTCTGGGGTCTTATCGCCAAGTGTTCCCCCACTAGTAGCATCCAAAATACTTTTATCCATAGGCAGCAAGCCTTCATAAAAATACTAGATGAGCAATTGT	T/C	TCAGAAATTTGGTGATGAGGGCAACTAGACACTAATTTCTTGAATATCTCTCAATACTCATGTAACGATTCCCTATCAATCTGCCTAATGCCACATATTT

Positions	-100 bp flanking sequences	SNPs within *kabuli* cultivar	+100 bp flanking sequences
Ca_LG_2:18051573	GGGGTCTTATCGCCAAGTGTTCCCCCACTAGTAGCATCCAAAATACTTTTATCCATAGGCAGCAAGCCTTCATAAAAATACTAGATGAGCAATTGTTCAG	G/A	AAATTTGGTGATGAGGGCAACTAGACACTAATTTCTTGAATATCTCTCAATACTCATGTAACGATTCCCTATCAATCTGCCTAATGCCACATATTTATTT
Ca_LG_2:18051575	GGTCTTATCGCCAAGTGTTCCCCCACTAGTAGCATCCAAAATACTTTTATCCATAGGCAGCAAGCCTTCATAAAAATACTAGATGAGCAATTGTTCAGAA	A/G	ATTTGGTGATGAGGGCAACTAGACACTAATTTCTTGAATATCTCTCAATACTCATGTAACGATTCCCTATCAATCTGCCTAATGCCACATATTTATTTTC
Ca_LG_2:20693112	TTATAAATTGAAAATCGTTCATTCTGGAAACAACCAGAATACCAAGCAGAAGTTCAACCAGAAGACTAAACAAAAGTTCAATAAATTAAACCAGAATACT	T/C	GATTATTGATCCTAGCAACAAACTTTATATGAATTACAAAGCATTTATACAACATCATTGAAGAAAAATACATGTTAAGAAAAATTACATGATACTATTT
Ca_LG_2:20693115	TAAATTGAAAATCGTTCATTCTGGAAACAACCAGAATACCAAGCAGAAGTTCAACCAGAAGACTAAACAAAAGTTCAATAAATTAAACCAGAATACTGAT	T/C	TATTGATCCTAGCAACAAACTTTATATGAATTACAAAGCATTTATACAACATCATTGAAGAAAAATACATGTTAAGAAAAATTACATGATACTATTTCAA
Ca_LG_2:21224300	AGAGAATAAACTCTCAATTTTTTACTCAATCAAATTGTAATTTTATTCAATCACAATTCATATACAAGTAGCATGCCATAACTTTTTGTCTTCCTAAACA	A/G	TAAGACTTAAAACGCCAATTGAATGTCTAATTGATCACTTTAATGGCTACACTTCAATTGGAAATTAACTAGTGCCATAAAGTGTAAGTTACATTTCTCC
Ca_LG_2:21224326	CAATCAAATTGTAATTTTATTCAATCACAATTCATATACAAGTAGCATGCCATAACTTTTTGTCTTCCTAAACATAAGACTTAAAACGCCAATTGAATGT	T/C	CTAATTGATCACTTTAATGGCTACACTTCAATTGGAAATTAACTAGTGCCATAAAGTGTAAGTTACATTTCTCCAACAATCATATCTTGCGTAAAATAAA
Ca_LG_2:21224364	CAAGTAGCATGCCATAACTTTTTGTCTTCCTAAACATAAGACTTAAAACGCCAATTGAATGTCTAATTGATCACTTTAATGGCTACACTTCAATTGGAAA	A/G	TTAACTAGTGCCATAAAGTGTAAGTTACATTTCTCCAACAATCATATCTTGCGTAAAATAAACATACAACGGCTAGTATTATACCTTTCATCTAAAAAAC
Ca_LG_2:21224392	CCTAAACATAAGACTTAAAACGCCAATTGAATGTCTAATTGATCACTTTAATGGCTACACTTCAATTGGAAATTAACTAGTGCCATAAAGTGTAAGTTAC	C/G	ATTTCTCCAACAATCATATCTTGCGTAAAATAAACATACAACGGCTAGTATTATACCTTTCATCTAAAAAACTTAAAATGCTACTAATGAATAAAAAAAG
Ca_LG_2:21224394	TAAACATAAGACTTAAAACGCCAATTGAATGTCTAATTGATCACTTTAATGGCTACACTTCAATTGGAAATTAACTAGTGCCATAAAGTGTAAGTTACAT	T/C	TTCTCCAACAATCATATCTTGCGTAAAATAAACATACAACGGCTAGTATTATACCTTTCATCTAAAAAACTTAAAATGCTACTAATGAATAAAAAAAGGA
Ca_LG_2:22661597	TGTAGCCCGAAACTAGAATACGAGGGCAGATTTGAAATCGCGAGGTTGAGGCGTGATTGACTGACCAAGCAGCAACTGAGAGGCGTCAAAATGCGCCAAA	A/C	ATGTTGGAAAACAGAAACTATAATTATATTCCCTAATTGTTGGGCTCTGATACCATGTTGAAGTTGTGGGATTTTAGAGAAAATGAGAAGAGAAAATAAT
Ca_LG_2:22661603	CCGAAACTAGAATACGAGGGCAGATTTGAAATCGCGAGGTTGAGGCGTGATTGACTGACCAAGCAGCAACTGAGAGGCGTCAAAATGCGCCAAAATGTTG	G/A	GAAAACAGAAACTATAATTATATTCCCTAATTGTTGGGCTCTGATACCATGTTGAAGTTGTGGGATTTTAGAGAAAATGAGAAGAGAAAATAATAAGGGT
Ca_LG_2:22661609	CTAGAATACGAGGGCAGATTTGAAATCGCGAGGTTGAGGCGTGATTGACTGACCAAGCAGCAACTGAGAGGCGTCAAAATGCGCCAAAATGTTGGAAAAC	C/G	AGAAACTATAATTATATTCCCTAATTGTTGGGCTCTGATACCATGTTGAAGTTGTGGGATTTTAGAGAAAATGAGAAGAGAAAATAATAAGGGTTTGAAT

Positions	-100 bp flanking sequences	SNPs within *kabuli* cultivar	+100 bp flanking sequences
Ca_LG_2:25186109	CTTTCTTGCATTTATAGTTTTGAACTTTGACAGGTAAGTCAAACAAAAGCTGATAAAATTACAATAGCACTCACATTGTACCACCTGCAGCAGGACCAAT	T/C	AGCTTTGAAAAAAGACATAGCTGTCATAGAAATTCCATTAGCTGCCCCTCTTTGGTGTTGTTCCTGTTTTCCAAATCAACATGTCATATACATATGTCAC
Ca_LG_2:25186117	CATTTATAGTTTTGAACTTTGACAGGTAAGTCAAACAAAAGCTGATAAAATTACAATAGCACTCACATTGTACCACCTGCAGCAGGACCAATAGCTTTGA	A/C	AAAAAGACATAGCTGTCATAGAAATTCCATTAGCTGCCCCTCTTTGGTGTTGTTCCTGTTTTCCAAATCAACATGTCATATACATATGTCACAATTCCAA
Ca_LG_2:25204180	AGTGTAGTCGGGAGTTAGTTTTGGAATTATTCAAAGTGGTGTTAACATTCTTCTTGATGATGATTTTATAGGAAAACCATGCCGCCAAGAAGGAATGACG	G/C	CTCCAAGAGTCAGTGTCAATAGGGACGATCAAATGGCGGATGCTATGAATAACATGGCTGCTTCTTTTGCTGCCCAAACTATTGCAAAGACTCGACGGGA
Ca_LG_2:25204221	TTAACATTCTTCTTGATGATGATTTTATAGGAAAACCATGCCGCCAAGAAGGAATGACGCTCCAAGAGTCAGTGTCAATAGGGACGATCAAATGGCGGAT	T/A	GCTATGAATAACATGGCTGCTTCTTTTGCTGCCCAAACTATTGCAAAGACTCGACGGGATCTAGAAAAGAGGGAAAGAGAGATCCGTGCTGCTGAGTCAA
Ca_LG_2:25204235	GATGATGATTTTATAGGAAAACCATGCCGCCAAGAAGGAATGACGCTCCAAGAGTCAGTGTCAATAGGGACGATCAAATGGCGGATGCTATGAATAACAT	T/C	GGCTGCTTCTTTTGCTGCCCAAACTATTGCAAAGACTCGACGGGATCTAGAAAAGAGGGAAAGAGAGATCCGTGCTGCTGAGTCAAGATGATTAGAAGAT
Ca_LG_2:25204246	TATAGGAAAACCATGCCGCCAAGAAGGAATGACGCTCCAAGAGTCAGTGTCAATAGGGACGATCAAATGGCGGATGCTATGAATAACATGGCTGCTTCTT	T/G	TTGCTGCCCAAACTATTGCAAAGACTCGACGGGATCTAGAAAAGAGGGAAAGAGAGATCCGTGCTGCTGAGTCAAGATGATTAGAAGATTTTCGTTGTTA
Ca_LG_2:25617708	AAAGAGGTGCACCAAAAAGCAACAAATATAAAGTCAAATGAAAAATAAGTTGATTCAAATATGAGAAGAGATAAAAACTAATATAATGCATATATACAAT	T/C	AGATAGGAAAAATATAAAATATGCAGCTACAATAAGTAATAACACAAGCAATAAATTCCAACAAATTATCTGGTTAACATAGTGACAACATACAACAATT
Ca_LG_2:25693443	GATAATTTTGAATTAGTACTATAATATATCGGTGAAGAAGAGATAAGTTGTGCTTCAACATTTAAGCACCCTTGTTCAAAAGGAGCTTAGCCTATTTCTT	T/C	GGTTTCGATCAACAATTTAACAATCTTGTTAGCTTGCTCCCAACAATCAAGTCTACTCTTGAAGATGCTGAGGAGAAACAATTCTCAAACAGATCTATCA
Ca_LG_2:25693466	ATATATCGGTGAAGAAGAGATAAGTTGTGCTTCAACATTTAAGCACCCTTGTTCAAAAGGAGCTTAGCCTATTTCTTGGTTTCGATCAACAATTTAACAA	A/G	TCTTGTTAGCTTGCTCCCAACAATCAAGTCTACTCTTGAAGATGCTGAGGAGAAACAATTCTCAAACAGATCTATCAAGGATTGGTTCCTTAGGCTCAAA
Ca_LG_2:25693468	ATATCGGTGAAGAAGAGATAAGTTGTGCTTCAACATTTAAGCACCCTTGTTCAAAAGGAGCTTAGCCTATTTCTTGGTTTCGATCAACAATTTAACAATC	C/T	TTGTTAGCTTGCTCCCAACAATCAAGTCTACTCTTGAAGATGCTGAGGAGAAACAATTCTCAAACAGATCTATCAAGGATTGGTTCCTTAGGCTCAAAGA
Ca_LG_2:25693472	CGGTGAAGAAGAGATAAGTTGTGCTTCAACATTTAAGCACCCTTGTTCAAAAGGAGCTTAGCCTATTTCTTGGTTTCGATCAACAATTTAACAATCTTGT	T/C	TAGCTTGCTCCCAACAATCAAGTCTACTCTTGAAGATGCTGAGGAGAAACAATTCTCAAACAGATCTATCAAGGATTGGTTCCTTAGGCTCAAAGATGTT
Ca_LG_2:26761243	TATTCTTTTCATTACCAAAACCAAGTTGTTTATCATGGAAACTGTGTTGAAGATGTTCATGAAGTTTGTTATGTTATTTGTTGTTGTTGCAGCTGCTATA	A/G	GTTGGTAACGTACAAGGTCTCCCACCTTGTTGCCCAGCAGATAAATCATTAGAATGTCAACCAATTCACTGTCCTAAGCTCCCATGTTGCATCCCTCCAA

Positions	-100 bp flanking sequences	SNPs within *kabuli* cultivar	+100 bp flanking sequences
Ca_LG_2:26761252	CATTACCAAAACCAAGTTGTTTATCATGGAAACTGTGTTGAAGATGTTCATGAAGTTTGTTATGTTATTTGTTGTTGTTGCAGCTGCTATAGTTGGTAAC	C/T	GTACAAGGTCTCCCACCTTGTTGCCCAGCAGATAAATCATTAGAATGTCAACCAATTCACTGTCCTAAGCTCCCATGTTGCATCCCTCCAACCAGTGACG
Ca_LG_2:26761268	TTGTTTATCATGGAAACTGTGTTGAAGATGTTCATGAAGTTTGTTATGTTATTTGTTGTTGTTGCAGCTGCTATAGTTGGTAACGTACAAGGTCTCCCAC	C/T	CTTGTTGCCCAGCAGATAAATCATTAGAATGTCAACCAATTCACTGTCCTAAGCTCCCATGTTGCATCCCTCCAACCAGTGACGTGAATCAGTAAAATTC
Ca_LG_2:29019383	ATTCCAAATCAAAATTAACTAACAAAAATCAGCACTAACACAATAGAAATTCGAATCTTTTGAACTAAGCAGCGACGCGATCTGGAGACAGATGAAGTTG	G/C	CAGCTAACAGAGAGGCGAGAGCGGCCATGGAGGCAGTCAGGCAGAGGCGGCCGCAGAGTGGAAGCCGAGATGGGACAAGATCGGAGGGAGGAGTGCTGTG
Ca_LG_2:29019385	TCCAAATCAAAATTAACTAACAAAAATCAGCACTAACACAATAGAAATTCGAATCTTTTGAACTAAGCAGCGACGCGATCTGGAGACAGATGAAGTTGCA	A/G	GCTAACAGAGAGGCGAGAGCGGCCATGGAGGCAGTCAGGCAGAGGCGGCCGCAGAGTGGAAGCCGAGATGGGACAAGATCGGAGGGAGGAGTGCTGTGTG
Ca_LG_2:29951000	GTTTTAATAAGGACTTTGGCCTACTCCATTTTAAGTATTTTAATTTGCTACTTTTAGCACCTAGGTAATTGCTAGCTGCACCCCCCACCACAAATTAATG	G/A	ACCAGCATCGCAGTCTTTTTCCTGAGGCAATCTTCTTCCACTCTAAACGATGCCGCCATTGAAATTCTCACACATCTCTCTCCTCTTCATTTCGATGAAC
Ca_LG_2:29951011	GACTTTGGCCTACTCCATTTTAAGTATTTTAATTTGCTACTTTTAGCACCTAGGTAATTGCTAGCTGCACCCCCCACCACAAATTAATGACCAGCATCGC	C/T	AGTCTTTTTCCTGAGGCAATCTTCTTCCACTCTAAACGATGCCGCCATTGAAATTCTCACACATCTCTCTCCTCTTCATTTCGATGAACTATTTAAAGGA
Ca_LG_2:29951036	ATTTTAATTTGCTACTTTTAGCACCTAGGTAATTGCTAGCTGCACCCCCCACCACAAATTAATGACCAGCATCGCAGTCTTTTTCCTGAGGCAATCTTCT	T/C	TCCACTCTAAACGATGCCGCCATTGAAATTCTCACACATCTCTCTCCTCTTCATTTCGATGAACTATTTAAAGGAACATAACCCTCACCAATATCCCCTT
Ca_LG_2:30430402	ACATCATCACTAACCTTAGTTCCAACCTCAAAATTACCAATACCCTTTTCGAATTGCGAATCCTCGTCAGTCGCAGCACCACCATCCTCGCCGGCGGTGG	G/A	AAAAGGGTCTTGTGGGGAGGGTTCTATCACCGGAAAGGTTTAAACTTTGTGAGAAACTAGTGATACAACGAGGGTTCAACAAATGGGTCGAAAAGGGACG
Ca_LG_2:30603876	AGTTTTAAAATTGCGGCCACAATATATAATTTATGCAATATCTACGACGGCAATTGTAGCTGCATTATTCTCATTTTGTAAATGTTCTGCAGCGTAGCTG	G/A	CAACTACAATTTAAAATTATTGATAGATGAAATGGTAGAATTTACGAATTGAAATTCTCACCTGTGCCTTGTGATGATGTTGATGATGAAGCAGGCATAG
Ca_LG_2:31287895	ACAAACGCGCGGCCGCCTCTGCTCCTCTGCTCGCTGCGGGCCTGTGGCCGCCTCTGTCTGACTGCCTCCATGGCCGTTCATCGCTCTCGCCTCTCTGTTT	T/C	GCCGCAGATCGCGTTGCTGCTTTGTTCAAAAGGTTCGGATTTTTATTTTGTTAGTGCTGATTTTTCTTAGTTAGTGCTGATTTTTGTTAGTTTTGATTTT
Ca_LG_2:31336725	TAGCCTAATGGTATGTCTTTTCTATTCTTGTATTTTGGTTAAAAATGGCTCAAGCACAAGATTTTATGTGATGGGCATATATGCAGCACATAATGATTTA	A/C	ACTAAATAACAGGTAGAGAAAACCAAAAGAAAGGTGGAGTAACACCTTTTTAGCCCGCTCAAACCTGACTTCTATCAATAATTTTTTTATGAATTTAATC
Ca_LG_2:31336739	GTCTTTTCTATTCTTGTATTTTGGTTAAAAATGGCTCAAGCACAAGATTTTATGTGATGGGCATATATGCAGCACATAATGATTTAACTAAATAACAGGT	T/C	AGAGAAAACCAAAAGAAAGGTGGAGTAACACCTTTTTAGCCCGCTCAAACCTGACTTCTATCAATAATTTTTTTATGAATTTAATCGTCCATTAACCTTC

Positions	-100 bp flanking sequences	SNPs within *kabuli* cultivar	+100 bp flanking sequences
Ca_LG_2:31336858	GTGGAGTAACACCTTTTTAGCCCGCTCAAACCTGACTTCTATCAATAATTTTTTTATGAATTTAATCGTCCATTAACCTTCATCTCTAGTTCTTAATATT	T/C	ACTGCTGCCAGGCGTTGGTGTTTTCCTTCTGTTGAAGTTGTGGGATTTTAGAGAAAAGGAGGAGAGAAAATAATAAGAGTTTAAATATTATTGATAATAG
Ca_LG_2:31337744	TCTAAGATATCTCTATGATTATAAATTGATCATAGGAAATAACTCAAAATACTCTAACACCTTCTTTCTTATTGAATCAACTTTGCTAAATGCTATATAG	G/A	TGAATTTTTACTTCTTGGTTGGCAAGTTGTTACAACTAAATTTGTCTCACAATCTACCTAGGAGAGTAAATACAAAAAAATTGGGTATGGAAGAAAATGG
Ca_LG_2:31337761	ATTATAAATTGATCATAGGAAATAACTCAAAATACTCTAACACCTTCTTTCTTATTGAATCAACTTTGCTAAATGCTATATAGTGAATTTTTACTTCTTG	G/C	GTTGGCAAGTTGTTACAACTAAATTTGTCTCACAATCTACCTAGGAGAGTAAATACAAAAAAATTGGGTATGGAAGAAAATGGGTCTTTTAATGTGTGGA
Ca_LG_2:31337882	AATTTGTCTCACAATCTACCTAGGAGAGTAAATACAAAAAAATTGGGTATGGAAGAAAATGGGTCTTTTAATGTGTGGAAGAAATGAACATTTGTTAGAA	A/G	TAAGGATTAATGCAGCTGCCTGTATTTATGGATACCTTCATTGAATTTATGGATACCTGTAGTTATGTATTTATGGATACTGTATTTTATAAGAAAGGGA
Ca_LG_2:31821808	AATTGACATGACATCGATGAGTCCGTTGGCTCTTAGTTCCTCATCTTTGCCTATGTCAACTGTTTCAACACCTCCCATGTCTCCCTTGGCAGCTTCATCG	G/A	TCTCCCAAGAGTGGAAACTTGTGGCAGAACAAACTAAACTTCACTCCACCATCATTGCAGCTCTCAGGTAGCCGCTTGAAGAGTGCTTTGAGTGCCAGGG
Ca_LG_2:32056168	TTTTGGCCAAATCATAAATTTTTAACCCAATAATACCATCTTGTAATAGCAAGATACCTCGTGTGCAGTGTAAATCTCAGTGTTAAAACCAATCTCTTCC	C/G	CCATGTCCATTAGTACCAAAGCCCCTAGGTTTTCCAAAATAGATACCTGAATAACAAACAAAGCAGCAGCAAGACTGAGTTAGGTGAACTAGCTTCCAAT
Ca_LG_2:32056175	CAAATCATAAATTTTTAACCCAATAATACCATCTTGTAATAGCAAGATACCTCGTGTGCAGTGTAAATCTCAGTGTTAAAACCAATCTCTTCCCCATGTC	C/A	CATTAGTACCAAAGCCCCTAGGTTTTCCAAAATAGATACCTGAATAACAAACAAAGCAGCAGCAAGACTGAGTTAGGTGAACTAGCTTCCAATATTATCA
Ca_LG_2:32056193	CCCAATAATACCATCTTGTAATAGCAAGATACCTCGTGTGCAGTGTAAATCTCAGTGTTAAAACCAATCTCTTCCCCATGTCCATTAGTACCAAAGCCCC	C/T	TAGGTTTTCCAAAATAGATACCTGAATAACAAACAAAGCAGCAGCAAGACTGAGTTAGGTGAACTAGCTTCCAATATTATCAATCAAATGAAATGAATGA
Ca_LG_2:32056215	AGCAAGATACCTCGTGTGCAGTGTAAATCTCAGTGTTAAAACCAATCTCTTCCCCATGTCCATTAGTACCAAAGCCCCTAGGTTTTCCAAAATAGATACC	C/A	TGAATAACAAACAAAGCAGCAGCAAGACTGAGTTAGGTGAACTAGCTTCCAATATTATCAATCAAATGAAATGAATGAAGAGTTAATATTCAAAATTTTA
Ca_LG_2:32724076	ACACGAACTCCCTTCCTTGGGTCGCGGATCTCAGCTGCCCATTTTCCCCATGGACGTTGGCGGATACCCCTATATTGATTCTTCCTCTTTCTCTTACATT	T/G	CCTTTTCAGCTTGCTCATTTGATTTCGGTGACACGGATTTTGCAGCAGTTGATCCTACAACACAATTCAAAACAAGTATAAAAAGCGTGTGGTTCGAACA
Ca_LG_2:32808956	AAACAGGGAGAGATAGAGAGAGAAAAAAGTACCTGTGCAGAAGACCCGAAAGCGTTTCTTATTATGTGCAGCCAGTTTAAGAACTTCAAAGACAACTCTA	A/G	GAAAAACCATGAACCAAAATGGTACAGCCATCAAATATAAAATCTTGACTAAGCATTGCAATAATCTTGCGAGCCTGCAAAAAATATAGGAATAATCAAT
Ca_LG_2:32863801	AAGGCGAAGGGAAATGAGCCGAGAAGTCAATGCACCAGATCCCGGTCCTTTAGCCACCTAAGAAAATGGAGTTGAAGGAAAGCAGAATCATACCGAACAA	A/G	CCAAAGCCATTAAATTCTGTTTGCTTTGACAGCAGCTTAACCAAAAGGGGAGGTCTCGCTCTTTTTGCTCTTCTATAGGATAGGATCTTGGGGGAGACTT

Positions	-100 bp flanking sequences	SNPs within *kabuli* cultivar	+100 bp flanking sequences
Ca_LG_2:32863813	AATGAGCCGAGAAGTCAATGCACCAGATCCCGGTCCTTTAGCCACCTAAGAAAATGGAGTTGAAGGAAAGCAGAATCATACCGAACAACCAAAGCCATTA	A/C	AATTCTGTTTGCTTTGACAGCAGCTTAACCAAAAGGGGAGGTCTCGCTCTTTTTGCTCTTCTATAGGATAGGATCTTGGGGGAGACTTTCTAATGGCTTT
Ca_LG_2:32863861	AGAAAATGGAGTTGAAGGAAAGCAGAATCATACCGAACAACCAAAGCCATTAAATTCTGTTTGCTTTGACAGCAGCTTAACCAAAAGGGGAGGTCTCGCT	T/C	CTTTTTGCTCTTCTATAGGATAGGATCTTGGGGGAGACTTTCTAATGGCTTTGATAGTTTAGAGTTGGACTTAGACTTTCCGAATCTTCTTTGGAAGATA
Ca_LG_2:33232165	TGATAATTTGCTCTAAACTATGAATTGGCAGCATCATGATTTTGCATATGTTACATGAGTGTTACTTCTAATTGGAAGCATCATGATAGTATGATACTTA	A/G	TAATTGTGCTGAATTTATGCTATATGTTACATGAGTGTTACTTCTGCATTATGAGAATTTATTGATTAATTAGCTCTTCTAATTTCTTATCTCTTCTAAT
Ca_LG_2:33232178	TAAACTATGAATTGGCAGCATCATGATTTTGCATATGTTACATGAGTGTTACTTCTAATTGGAAGCATCATGATAGTATGATACTTATAATTGTGCTGAA	A/T	TTTATGCTATATGTTACATGAGTGTTACTTCTGCATTATGAGAATTTATTGATTAATTAGCTCTTCTAATTTCTTATCTCTTCTAATTTGCTCTAAACTA
Ca_LG_2:33232658	TATATACTTGATGCATTTAGGACTGCTGCTATATACTTGTTATTAGGACTGCTTCTATTTGGTCTGCTTGTTATTTGGACTGCTGCTATATTTTGGACTG	G/A	CTATATTTTGGACAACTGCTATATTTACTTGTTAGGACTGCTGCTATATACTTGATGCATTTAGGACTGCTGCTATATACTTGTTATTTAGGACTACTAT
Ca_LG_2:33232664	CTTGATGCATTTAGGACTGCTGCTATATACTTGTTATTAGGACTGCTTCTATTTGGTCTGCTTGTTATTTGGACTGCTGCTATATTTTGGACTGCTATAT	T/C	TTTGGACAACTGCTATATTTACTTGTTAGGACTGCTGCTATATACTTGATGCATTTAGGACTGCTGCTATATACTTGTTATTTAGGACTACTATATACTT
Ca_LG_2:33232887	TTACTTGTTAGGACTGCTACTATATACTTGATGCATTTAGGACTGCTGCTATATACTTGTTATTTGGACTGCTGCTATTTGGTCTGCTTGTTATTTGGAT	T/C	TGCTGCTATATTTATGACTGTTTTTTCTTTGGTTTGCTGCTATATTTAGGACTGCTATATTTTCCTGCAATCAAACAGAAGATGCAGTCCAAAACTTTTA
Ca_LG_2:33232905	ACTATATACTTGATGCATTTAGGACTGCTGCTATATACTTGTTATTTGGACTGCTGCTATTTGGTCTGCTTGTTATTTGGATTGCTGCTATATTTATGAC	C/T	TGTTTTTTCTTTGGTTTGCTGCTATATTTAGGACTGCTATATTTTCCTGCAATCAAACAGAAGATGCAGTCCAAAACTTTTAGGACTGTTGTTTCTTAGT
Ca_LG_2:33232921	ATTTAGGACTGCTGCTATATACTTGTTATTTGGACTGCTGCTATTTGGTCTGCTTGTTATTTGGATTGCTGCTATATTTATGACTGTTTTTTCTTTGGTT	T/C	TGCTGCTATATTTAGGACTGCTATATTTTCCTGCAATCAAACAGAAGATGCAGTCCAAAACTTTTAGGACTGTTGTTTCTTAGTTACTTATTTGGACTGC
Ca_LG_3:23775	TTCTATACAATGTGGTACTTTAGTATTTATAGAGTTGAAAAATACTCAAAATGTGAGATCTTTTCAATGTGGGACTTCAATGCGCTACTTCACTTCCTCC	C/T	GAAATCACCTTCTCAAGGTATCTATCTCTTCCGATCTGATCTGTTTTCTCTCTCTCTCTAGTATTATGCACTTGTAATGTAGTTAGGTTACTCAATGTTA
Ca_LG_3:23776	TCTATACAATGTGGTACTTTAGTATTTATAGAGTTGAAAAATACTCAAAATGTGAGATCTTTTCAATGTGGGACTTCAATGCGCTACTTCACTTCCTCCG	G/A	AAATCACCTTCTCAAGGTATCTATCTCTTCCGATCTGATCTGTTTTCTCTCTCTCTCTAGTATTATGCACTTGTAATGTAGTTAGGTTACTCAATGTTAA
Ca_LG_3:1107617	GATACACCCCATGTTTAGCAAGGCGTGGCTGACTTATTTTGGGAACATGGGCAGTAGCGACCGATTTTTCCCGATCCAGTTTTCTTGCTGCAATTTTGGA	A/C	AACTTTATTACCCTCCAGCCTTTGTAGTTTGGCAGCCCTATTTACCCTCTAATGTTGGAGGTTTGCTAGCTCTTTTTGGCTGTACGGGTGGTTTATTTAA

Positions	-100 bp flanking sequences	SNPs within *kabuli* cultivar	+100 bp flanking sequences
Ca_LG_3:1107638	GGCGTGGCTGACTTATTTTGGGAACATGGGCAGTAGCGACCGATTTTTCCCGATCCAGTTTTCTTGCTGCAATTTTGGAAACTTTATTACCCTCCAGCCT	T/G	TTGTAGTTTGGCAGCCCTATTTACCCTCTAATGTTGGAGGTTTGCTAGCTCTTTTTGGCTGTACGGGTGGTTTATTTAATTGGATCTACAAAAATGAGGT
Ca_LG_3:1112996	TATAAATATATATATCTATATATAGATAGATAGATAGATATAGAAGCAAATCCTGAAATGTCTGGTTTCAAGGAAAAGTGGCAGCGCAAAACGCCACAAC	C/G	TGTTGCTTGATTATCTATATTCTTCCAATTGAAGTCTGCAATTTCAGAAGTCCCATTCCATAGTAGAAGATGATATAACCGTTCATCGGAACAAGGCGAT
Ca_LG_3:1113006	TATATCTATATATAGATAGATAGATAGATATAGAAGCAAATCCTGAAATGTCTGGTTTCAAGGAAAAGTGGCAGCGCAAAACGCCACAACTGTTGCTTGA	A/G	TTATCTATATTCTTCCAATTGAAGTCTGCAATTTCAGAAGTCCCATTCCATAGTAGAAGATGATATAACCGTTCATCGGAACAAGGCGATTGCGATATCA
Ca_LG_3:2634611	ATGAAAGCATGCTTGGTGGGCTGCCAATCCTATTGCCACGAGCAGCCATCTCAGCCATAGCGCGTATACTTGTGATGGGCGATGACCCTCCATTATTAAA	A/G	CACATCATGCAGGCTGGAATCAAGAGCATGTCGCATATTCTCTGGCTTCACAGCTGCACTGGCTTGCTCAGCGGGATAGCAAGATGGATTCTGGGGTACT
Ca_LG_3:3375551	CCCTTCCCATCCTACGGTTGAGAAATGATTTTGTGTTGCTTCGACGCTTTTAAGAGACATAGCTTTGGAAAAAACGTATTAGTAGCTTATATGCCGTGGA	A/G	AAGGTTACAATTCTGAAGATGCTGTACTTAGTGAGCGTTTGGCCTATAAAGATATTTATACTTTTTTCACATAACGAAATATGAATTACTAATAAAATTC
Ca_LG_3:3735155	TGGTTGTTACTTTGAGGTTCTTCAATTACTTGATAAAATGAAGCAGAAAAATGTTAAGATGAATAAGGTAGCGATCGTGAATGCTCTTTTGGCAGCTTCG	G/A	GAGATGAGAGACCTCGAGAAAGGGAAGGAAATTCATGATTGTGCTTTGCAGCTGGGGATAATGTCAGATATTGTTGTTGCTACTCCTGTAGTGAGCATGT
Ca_LG_3:4133484	GAACACAAAATGAAGGATAGTAGTAGGTTTTGGTACAAAAGATGCTTTAGTGATATTTGATGGTCTGAACAAGGAACTCTGTGTGGGGATCCATCTTGTA	A/T	GTTATTCGCATTGTTCACCTTGGTTGAAATCTTGTTGGTTGTTATGCACTTTATATTTTAAGCAAGAAGCTTCATCCTTGATGATGGCCTATGGTGGTGA
Ca_LG_3:4133517	TACAAAAGATGCTTTAGTGATATTTGATGGTCTGAACAAGGAACTCTGTGTGGGGATCCATCTTGTAGTTATTCGCATTGTTCACCTTGGTTGAAATCTT	T/G	GTTGGTTGTTATGCACTTTATATTTTAAGCAAGAAGCTTCATCCTTGATGATGGCCTATGGTGGTGATGAATTGGGAATGGTTTTTCACCCTCCATTCAA
Ca_LG_3:4133519	CAAAAGATGCTTTAGTGATATTTGATGGTCTGAACAAGGAACTCTGTGTGGGGATCCATCTTGTAGTTATTCGCATTGTTCACCTTGGTTGAAATCTTGT	T/G	TGGTTGTTATGCACTTTATATTTTAAGCAAGAAGCTTCATCCTTGATGATGGCCTATGGTGGTGATGAATTGGGAATGGTTTTTCACCCTCCATTCAAAA
Ca_LG_3:4133528	CTTTAGTGATATTTGATGGTCTGAACAAGGAACTCTGTGTGGGGATCCATCTTGTAGTTATTCGCATTGTTCACCTTGGTTGAAATCTTGTTGGTTGTTA	A/C	TGCACTTTATATTTTAAGCAAGAAGCTTCATCCTTGATGATGGCCTATGGTGGTGATGAATTGGGAATGGTTTTTCACCCTCCATTCAAAATGTTTTGTT
Ca_LG_3:4133532	AGTGATATTTGATGGTCTGAACAAGGAACTCTGTGTGGGGATCCATCTTGTAGTTATTCGCATTGTTCACCTTGGTTGAAATCTTGTTGGTTGTTATGCA	A/G	CTTTATATTTTAAGCAAGAAGCTTCATCCTTGATGATGGCCTATGGTGGTGATGAATTGGGAATGGTTTTTCACCCTCCATTCAAAATGTTTTGTTTGTT
Ca_LG_3:4598590	TAACATTATAACTGGTTTGGTGTGATAAAGACAGCTATAGAAATTTCAAAGGGGGGAGAGGACCGTCTAGCTGCTATGGTCCTAACTCCTAAGCCTGCAC	C/T	ACAAGTCACCCTCCTCCTCTATTTGATGCTAAAAATACGCAGCCTTTGCCCTTGACCCTTTTCAACTCATACATTTTTCATTCAGTGTTAACTATGGTGA

Positions	-100 bp flanking sequences	SNPs within *kabuli* cultivar	+100 bp flanking sequences
Ca_LG_3:5081278	ATAAATAGTCAGACTAGTTGGTGGGCCTGTGAGACTCTACCTTTATCAGCCCTCCACCGTTCTGTCCATACAGTAGCATCTACTAACGATGAAGAAGAAG	G/A	CTGGACCCGACTCAGAGCTTCCTCTGCCAGAATCACTGCCAGAATCTCATTTGTATTGTGTATGATTCTTAATAAAATAGCTAGTTGTATTGTAGAAGAA
Ca_LG_3:5081280	AAATAGTCAGACTAGTTGGTGGGCCTGTGAGACTCTACCTTTATCAGCCCTCCACCGTTCTGTCCATACAGTAGCATCTACTAACGATGAAGAAGAAGCT	T/C	GGACCCGACTCAGAGCTTCCTCTGCCAGAATCACTGCCAGAATCTCATTTGTATTGTGTATGATTCTTAATAAAATAGCTAGTTGTATTGTAGAAGAAAT
Ca_LG_3:5318863	CGTGAAAGAGCTGAATTCAGAGAGAGAGGCAAAGAGAAAAGTGAGAGAGGAAGAGCAAACCCTATTTTCGCATAAACAGAGCAGCTGCACTAGATTCGCG	G/C	ATTTCTCCTTTGTTCACCGTCGGATCGGGCTGAAATTTGAACAGCAGGTTCGTGACTCGTGGTACTTCATTCTGACCGTTTGGATCGTCGATCAGAGGTC
Ca_LG_3:5848239	GTGAGATCAATAAATCCAGCTCCCTTAAACTCTGCTGTCCCCATCAATGCGGAAGAAGCATCTTGTTCAACACAAGAAGCAGCTCTGTCTGACCTAGACG	G/A	ACTTAGATAAACAATTTGTTGAGAACATTTTTGGTCTAATGCAGCAAGAGCAAACATTTTCTGGCCAGGAGAAATTGATGGAGTGGATATTGACGATACA
Ca_LG_3:5862667	GTTCTTTTTGAAACAATTGATTAGTGAATGCTCCACCACCACTAACATTAGTCATGTTAGATAAATCAAAGTGTTCATAAGAGGGCATGTTATGCTGCAC	C/T	CATGATGGAACTAGAGTATGGTGGTCTAGCAGAAGCTGAAACATGATCAGGACCAACCATGCTACTAACAAAGCTTTTTTGCATCATGGAAGAGTTACCA
Ca_LG_3:5895714	AATTTTTATACAAAACCTTTTAAGTAGAATAAAACAAAAGAGGAGCTGTAAAATACAAACCCAAGGCTGCCAAAGCTTGAACTTCCATCAAAGCTTTTCT	T/G	CCTTCAATGACAAAATATCATATTTAGTGGCTAAACACAAGAAGAGAAGAAACATCCATCACTATGAAAATGGTACCTTTCAGTTTCCTGGTGCAACTGT
Ca_LG_3:5949266	CCCTGTTTGATAGTAAAGAATTACTATAATGCTATGTGTATTTAGTTGTACCATTGGAATTAATCTCAGCTGCCATTCTCTGTTAACATGATTGCAGCTA	A/G	TGCATTCTCGTTGCTGGAGCACTTGGCGGGAGATTTGATCATGAGATTGGAAATATCAATGTGCTGTGTCGATTCTCCAACACACGCATTATCCTTCTAT
Ca_LG_3:6000013	AGGTGAAAAAAATAAACAAAAAAAAATTATGAACAATTGAATGAATTTTTAAACTTTTGAATGAATATATAGTGCATTACCAATACGTAGAGGCAATTCT	T/C	AGCAGCTTTAGCTGTGAAAGAAACATAGCTGATGCTATAGAGTTGCTTGTTAATTGGGATAGCTGCGATTTTCCATTTTTTTGATGTATTGATTCTTTCT
Ca_LG_3:6000021	AAAATAAACAAAAAAAAATTATGAACAATTGAATGAATTTTTAAACTTTTGAATGAATATATAGTGCATTACCAATACGTAGAGGCAATTCTAGCAGCTT	T/C	TAGCTGTGAAAGAAACATAGCTGATGCTATAGAGTTGCTTGTTAATTGGGATAGCTGCGATTTTCCATTTTTTTGATGTATTGATTCTTTCTAACTCTTG
Ca_LG_3:6000033	AAAAAATTATGAACAATTGAATGAATTTTTAAACTTTTGAATGAATATATAGTGCATTACCAATACGTAGAGGCAATTCTAGCAGCTTTAGCTGTGAAAG	G/C	AAACATAGCTGATGCTATAGAGTTGCTTGTTAATTGGGATAGCTGCGATTTTCCATTTTTTTGATGTATTGATTCTTTCTAACTCTTGACCAAACTGATC
Ca_LG_3:6454994	CAGATGCAACCAGATAACCCTAAGCCCTTCTTCTTGTCTGAACCCGGTGCCGCATTAGGTTAGGGAGCTGGGCTTTTTTCGCTCCTCTCACATTCATTCA	A/G	TTCGAGCTGCTTCGCTCCTCCGCATCCCAAAGCGCGCAGCCGTCCTACGCGCGAGACACTAAGTAATCCAAGCCAACCCACATTACTGACTAACGGTGGA
Ca_LG_3:7068872	CACAATTTGAAAAGTTGTGGAGCTATCCAAGCCAGTAGATATTTCATCCATTAATAGAACTTTTGCTGCTCCAACCAACATCTCTCCTGTTGTAACTCTT	T/C	TTCCTTTGTCCGCCCGAGATACCTCGCCGCATTCCATCCCCCACAATAGTATCACTACATACATCCAACCCCAAGATCTGCAAAATTTTATTTTATTGTA

Positions	-100 bp flanking sequences	SNPs within *kabuli* cultivar	+100 bp flanking sequences
Ca_LG_3:7069058	TTTATTTTATTGTAGCTAAATTTTTCGTCAATAATTCAACTTTTTTTTGGCACTAAAGAACCTTATTCTCTAGTATTACCTTCAGCATATAATCTATTAC	C/T	CAGACTTTCCTCCTGGCCTCCAGCTGCAGCTGCCTGGTATATCAAAAGTCAATGAGATGCACACATACGCCCTCCGGTCATTACAGTAAACGAAAATAAC
Ca_LG_3:7069070	TAGCTAAATTTTTCGTCAATAATTCAACTTTTTTTTGGCACTAAAGAACCTTATTCTCTAGTATTACCTTCAGCATATAATCTATTACCAGACTTTCCTC	C/A	CTGGCCTCCAGCTGCAGCTGCCTGGTATATCAAAAGTCAATGAGATGCACACATACGCCCTCCGGTCATTACAGTAAACGAAAATAACTACTTTTTCTCA
Ca_LG_3:7371113	ACCTTTGGTGAAGAAATCTGCAATCTGATCAGTGCTGGAGACAAGGAGTAGCTTCATGGTTCCATTGTTGGCTTTCGCTATAACAATATGACAATCAATG	G/A	TCAAGATGTTTGGTTCTCTCATGAAAAACTGGATTGGCTGCTATATGAAGGGTGCTTCCATTGTCACAATAAATAGTTGGTGTCTTGATATAAGTAATCT
Ca_LG_3:7371116	TTTGGTGAAGAAATCTGCAATCTGATCAGTGCTGGAGACAAGGAGTAGCTTCATGGTTCCATTGTTGGCTTTCGCTATAACAATATGACAATCAATGTCA	A/T	AGATGTTTGGTTCTCTCATGAAAAACTGGATTGGCTGCTATATGAAGGGTGCTTCCATTGTCACAATAAATAGTTGGTGTCTTGATATAAGTAATCTTTA
Ca_LG_3:7371125	GAAATCTGCAATCTGATCAGTGCTGGAGACAAGGAGTAGCTTCATGGTTCCATTGTTGGCTTTCGCTATAACAATATGACAATCAATGTCAAGATGTTTG	G/T	GTTCTCTCATGAAAAACTGGATTGGCTGCTATATGAAGGGTGCTTCCATTGTCACAATAAATAGTTGGTGTCTTGATATAAGTAATCTTTAAGTCCTCAA
Ca_LG_3:7901135	TCCACTCAAATGTTATTATTGCAAGCAGCCAGGCCACAAAGAGGCTAGCTGCTGGAAGAAAGAGGCAGATGAACAAAAGGGTGATCAAAAAATCCAATTTC	C/T	GTGGAGCATGAGCAAAAATTGTTCTTAGCACAAAGTGCAGCTAGCAATGATGCAGGAGGTGGCATGTGGTATGTTGACAGTGGATGCTCCAACCATATGT
Ca_LG_3:8166260	GTGTAAACAAGTTGAGCCGAGCTGAACACTTAAATTTTGTGCCTGTGGACTTGTCATATCCCAATCCACAAATAGTATCAGCTTATATGCATATATAATA	A/G	TAATGTTTTGGTAAAAAAATGTATATAATGCAAAAATTCAAGCTATGTAGCAAATGACAGAATGTTAGCCACCAAATCACTTTAGTAGCTTTACCTATTT
Ca_LG_3:8166266	ACAAGTTGAGCCGAGCTGAACACTTAAATTTTGTGCCTGTGGACTTGTCATATCCCAATCCACAAATAGTATCAGCTTATATGCATATATAATATAATGT	T/C	TTTGGTAAAAAAATGTATATAATGCAAAAATTCAAGCTATGTAGCAAATGACAGAATGTTAGCCACCAAATCACTTTAGTAGCTTTACCTATTTTATCAT
Ca_LG_3:8166303	TGTGGACTTGTCATATCCCAATCCACAAATAGTATCAGCTTATATGCATATATAATATAATGTTTTGGTAAAAAAATGTATATAATGCAAAAATTCAAGC	C/T	TATGTAGCAAATGACAGAATGTTAGCCACCAAATCACTTTAGTAGCTTTACCTATTTTATCATTTCCCCATACATTCTTTTTCATGTGTTGACCTACATG
Ca_LG_3:8484990	TGATAACGACGAAAATCTTCCAATCCCCTTGACTTTGCAACAGGAATCTATCTTTCCCTCTTCTCCTGATCCTACAGAGTCTTTGCAGCAGTTTGTGCAG	G/A	CAACAAAAGCAACCATGTTATTCATAGCTTCAACAATTTGATCATCCCCATTGACGTTTACTCTTGCAGCGTCATCCCTTCTTGGAGGCATTGTTTCCTC
Ca_LG_3:8485014	CCCCTTGACTTTGCAACAGGAATCTATCTTTCCCTCTTCTCCTGATCCTACAGAGTCTTTGCAGCAGTTTGTGCAGCAACAAAAGCAACCATGTTATTCA	A/C	TAGCTTCAACAATTTGATCATCCCCATTGACGTTTACTCTTGCAGCGTCATCCCTTCTTGGAGGCATTGTTTCCTCAAAACCAACATCGAGAAGAAATCT
Ca_LG_3:8485025	TGCAACAGGAATCTATCTTTCCCTCTTCTCCTGATCCTACAGAGTCTTTGCAGCAGTTTGTGCAGCAACAAAAGCAACCATGTTATTCATAGCTTCAACA	A/C	ATTTGATCATCCCCATTGACGTTTACTCTTGCAGCGTCATCCCTTCTTGGAGGCATTGTTTCCTCAAAACCAACATCGAGAAGAAATCTCAACACCCGTT

Positions	-100 bp flanking sequences	SNPs within *kabuli* cultivar	+100 bp flanking sequences
Ca_LG_3:8656523	AACATTGACTTTGACTTAAGACGATGGAGCATAGCTGCATACTACGACAGTGAGGCAGCGAGATAACAAGATAGCGTGACACAACAGAGAGGTGACGCAA	A/C	CAAAGAGGCAGCAACAAGATATATATATATAGAGAGAGATAGCGCGATGCAGTTAAAAAAATGTGGGTCGACAAAAAAGTAAAAAATTTCTTTTGTTTAGAA
Ca_LG_3:8860693	TGCAGCCACTTAAGTAACTCAACTGTGTCCCTGTACCTGAGAAACAGAAGTTGCTTTTTTTCACCATCTGTAGTACACAACATGATGAATAAATTGCAAC	C/T	CATAATACCATATCTACATTAAGTAGGTAGTTAATCCCAGATGGTGGTGCGTCGCAACCAACCTCAAAAAATGGGATAGCAGGATATCCTGGTTTGCCAC
Ca_LG_3:9101461	GAAATGACCTTCTGCATATCGATCTACATTTGAGGGAGCAGGACTTCCAGTTTTCTCTTTCTCCGCTTCTTGAGGTAGTCTCCGGAAGAAATCCACAGTG	G/A	AGATACGAAGATTCCATATCTACAAGCCGGAGAGTTGTCTTCCTACCTTCTTCACGAAACCTTTCTAAAGCCTCATTAGCCGCTGCAGCTAGTTCAGCTT
Ca_LG_3:9101482	ATCTACATTTGAGGGAGCAGGACTTCCAGTTTTCTCTTTCTCCGCTTCTTGAGGTAGTCTCCGGAAGAAATCCACAGTGAGATACGAAGATTCCATATCT	T/C	ACAAGCCGGAGAGTTGTCTTCCTACCTTCTTCACGAAACCTTTCTAAAGCCTCATTAGCCGCTGCAGCTAGTTCAGCTTGAAAAGTTGGGAATCGCTTCA
Ca_LG_3:9101491	TGAGGGAGCAGGACTTCCAGTTTTCTCTTTCTCCGCTTCTTGAGGTAGTCTCCGGAAGAAATCCACAGTGAGATACGAAGATTCCATATCTACAAGCCGG	G/A	AGAGTTGTCTTCCTACCTTCTTCACGAAACCTTTCTAAAGCCTCATTAGCCGCTGCAGCTAGTTCAGCTTGAAAAGTTGGGAATCGCTTCAGTTCCTGCT
Ca_LG_3:9101493	AGGGAGCAGGACTTCCAGTTTTCTCTTTCTCCGCTTCTTGAGGTAGTCTCCGGAAGAAATCCACAGTGAGATACGAAGATTCCATATCTACAAGCCGGAG	G/T	AGTTGTCTTCCTACCTTCTTCACGAAACCTTTCTAAAGCCTCATTAGCCGCTGCAGCTAGTTCAGCTTGAAAAGTTGGGAATCGCTTCAGTTCCTGCTCA
Ca_LG_3:9461188	ATAGCTTTCCAGAGTAGGCATGACAATAAAGTTAACGGACTTGGTATCGCTGCTGGTGGCATGGCATCAGCTAAAGCTGCAGATATACAGGATGTGGACA	A/C	AAACTGAAGGATCCTCCAAAGGAGGTGGAAAGAAGAAGGGCAAGAAGGGGAAAAAGGTTAGCCCTTTAGTTTTGGGATTTAATGTTGTGAGTAACCGGAT
Ca_LG_3:9461435	TTAAATTTTATGCATGAGGTTGTTGCGGTAGATGTACATACATTTTGGTCGCACATTGTAAATGCTTTTGTTCGATTTAGGATATTTATTTCCTACACAT	T/G	ACTGTAGCAGCATTTAGATCAAACTTAACCAATTTTGTTCTCTAAATTTAGTTGCTTTGACTTGTTATATTGGTTTAAATTTAATCGATCTAATAGATTA
Ca_LG_3:9723383	CAAATTCAGCAACAGCGTGTGAGGTCTTTGATGTTATAAATTTGGTTGTTTTACTTAATGGATGCAAACTTGTTGATTTAGTTTTGGGTGTTTAAAAAAT	T/C	CAGTTTAGTATCTTGGAGTTATTACAGACAATTGGTTGTAAGAACTTTGTTGTAGTTGGCTAATCTTTGCACTACTTGTCCATGGACTAGTGGTATTAAT
Ca_LG_3:9723395	CAGCGTGTGAGGTCTTTGATGTTATAAATTTGGTTGTTTTACTTAATGGATGCAAACTTGTTGATTTAGTTTTGGGTGTTTAAAAAATCAGTTTAGTATC	C/T	TTGGAGTTATTACAGACAATTGGTTGTAAGAACTTTGTTGTAGTTGGCTAATCTTTGCACTACTTGTCCATGGACTAGTGGTATTAATTCACGTTTCTTG
Ca_LG_3:9723418	ATAAATTTGGTTGTTTTACTTAATGGATGCAAACTTGTTGATTTAGTTTTGGGTGTTTAAAAAATCAGTTTAGTATCTTGGAGTTATTACAGACAATTGG	G/A	TTGTAAGAACTTTGTTGTAGTTGGCTAATCTTTGCACTACTTGTCCATGGACTAGTGGTATTAATTCACGTTTCTTGTGTTTCATAGTGGTATTAATTCA
Ca_LG_3:9723435	ACTTAATGGATGCAAACTTGTTGATTTAGTTTTGGGTGTTTAAAAAATCAGTTTAGTATCTTGGAGTTATTACAGACAATTGGTTGTAAGAACTTTGTTG	G/A	TAGTTGGCTAATCTTTGCACTACTTGTCCATGGACTAGTGGTATTAATTCACGTTTCTTGTGTTTCATAGTGGTATTAATTCAGAAAAATTGTTAACTTG

Positions	-100 bp flanking sequences	SNPs within *kabuli* cultivar	+100 bp flanking sequences
Ca_LG_3:9723439	AATGGATGCAAACTTGTTGATTTAGTTTTGGGTGTTTAAAAAATCAGTTTAGTATCTTGGAGTTATTACAGACAATTGGTTGTAAGAACTTTGTTGTAGT	T/C	TGGCTAATCTTTGCACTACTTGTCCATGGACTAGTGGTATTAATTCACGTTTCTTGTGTTTCATAGTGGTATTAATTCAGAAAAATTGTTAACTTGTGTT
Ca_LG_3:9986264	CGCTCTCGCCGCGGGAGCAGCTCAAGCAGTCCACCAACAGCCGACGGGTTCGGAACTGGGACCCCCGTGCCCAGCCCTCAGAGCCAATCCTTTTCCCGAG	G/A	GTTACGGATCCATTTTGCCGACTTCCCTTGCCTACATTGTTCCATCGACCAGAGGCTGTTCACCTTGGAGACCTGATGCGGTTATGAGTACGACCGGGCA
Ca_LG_3:9986275	CGGGAGCAGCTCAAGCAGTCCACCAACAGCCGACGGGTTCGGAACTGGGACCCCCGTGCCCAGCCCTCAGAGCCAATCCTTTTCCCGAGGTTACGGATCC	C/T	ATTTTGCCGACTTCCCTTGCCTACATTGTTCCATCGACCAGAGGCTGTTCACCTTGGAGACCTGATGCGGTTATGAGTACGACCGGGCATGGAAGGCACT
Ca_LG_3:9986294	CCACCAACAGCCGACGGGTTCGGAACTGGGACCCCCGTGCCCAGCCCTCAGAGCCAATCCTTTTCCCGAGGTTACGGATCCATTTTGCCGACTTCCCTTG	G/A	CCTACATTGTTCCATCGACCAGAGGCTGTTCACCTTGGAGACCTGATGCGGTTATGAGTACGACCGGGCATGGAAGGCACTCGGTCCTCCGGATTTTCAA
Ca_LG_3:9986295	CACCAACAGCCGACGGGTTCGGAACTGGGACCCCCGTGCCCAGCCCTCAGAGCCAATCCTTTTCCCGAGGTTACGGATCCATTTTGCCGACTTCCCTTGC	C/T	CTACATTGTTCCATCGACCAGAGGCTGTTCACCTTGGAGACCTGATGCGGTTATGAGTACGACCGGGCATGGAAGGCACTCGGTCCTCCGGATTTTCAAG
Ca_LG_3:9986307	ACGGGTTCGGAACTGGGACCCCCGTGCCCAGCCCTCAGAGCCAATCCTTTTCCCGAGGTTACGGATCCATTTTGCCGACTTCCCTTGCCTACATTGTTCC	C/T	ATCGACCAGAGGCTGTTCACCTTGGAGACCTGATGCGGTTATGAGTACGACCGGGCATGGAAGGCACTCGGTCCTCCGGATTTTCAAGGGCCGCCAGGGG
Ca_LG_3:9986311	GTTCGGAACTGGGACCCCCGTGCCCAGCCCTCAGAGCCAATCCTTTTCCCGAGGTTACGGATCCATTTTGCCGACTTCCCTTGCCTACATTGTTCCATCG	G/A	ACCAGAGGCTGTTCACCTTGGAGACCTGATGCGGTTATGAGTACGACCGGGCATGGAAGGCACTCGGTCCTCCGGATTTTCAAGGGCCGCCAGGGGCGCA
Ca_LG_3:10753824	AACAAATAGAATATATAATAATCCTTAATTGTTTTGATTTTTCAATGAGATTGATTGCTTTTGTTTTTTGTTTCAGCCTGATGTAACTGCGCCAGGTGTA	A/G	AACATACTTGCTGCCTATTCATTGTTTGCAAGTGCATCTAATTTACTAACAGATACTCGTCGAGGTTTTCCATTCAATGTAATGCAGGGAACTTCTATGT
Ca_LG_3:10753870	GAGATTGATTGCTTTTGTTTTTTGTTTCAGCCTGATGTAACTGCGCCAGGTGTAAACATACTTGCTGCCTATTCATTGTTTGCAAGTGCATCTAATTTAC	C/T	TAACAGATACTCGTCGAGGTTTTCCATTCAATGTAATGCAGGGAACTTCTATGTCTTGCCCTCATGTCGCTGGCATTGCAGGACTTATCAAAACACTTCA
Ca_LG_3:10753911	TGCGCCAGGTGTAAACATACTTGCTGCCTATTCATTGTTTGCAAGTGCATCTAATTTACTAACAGATACTCGTCGAGGTTTTCCATTCAATGTAATGCAG	G/A	GGAACTTCTATGTCTTGCCCTCATGTCGCTGGCATTGCAGGACTTATCAAAACACTTCATCCTAATTGGAGTCCAGCTGCTATTAAATCAGCTATCATGA
Ca_LG_3:10753929	ACTTGCTGCCTATTCATTGTTTGCAAGTGCATCTAATTTACTAACAGATACTCGTCGAGGTTTTCCATTCAATGTAATGCAGGGAACTTCTATGTCTTGC	C/T	CCTCATGTCGCTGGCATTGCAGGACTTATCAAAACACTTCATCCTAATTGGAGTCCAGCTGCTATTAAATCAGCTATCATGACCACGGGTATTGTCTCTC
Ca_LG_3:10753940	ATTCATTGTTTGCAAGTGCATCTAATTTACTAACAGATACTCGTCGAGGTTTTCCATTCAATGTAATGCAGGGAACTTCTATGTCTTGCCCTCATGTCGC	C/T	TGGCATTGCAGGACTTATCAAAACACTTCATCCTAATTGGAGTCCAGCTGCTATTAAATCAGCTATCATGACCACGGGTATTGTCTCTCATCACTCAC

Positions	-100 bp flanking sequences	SNPs within *kabuli* cultivar	+100 bp flanking sequences
Ca_LG_3:10753950	TGCAAGTGCATCTAATTTACTAACAGATACTCGTCGAGGTTTTCCATTCAATGTAATGCAGGGAACTTCTATGTCTTGCCCTCATGTCGCTGGCATTGCA	A/T	GGACTTATCAAAACACTTCATCCTAATTGGAGTCCAGCTGCTATTAAATCAGCTATCATGACCACGGGTATTGTCTCTCTCATCACTCACACAGACACTT
Ca_LG_3:11242017	CTCTGATAATTAAAGTTTGGATCCAATGTTTCTCTCAGTAGTATCTTCAACGGCCCTAATCACGTGACCCAAAATCTTCTTCCGATTCGCAGCGTACACA	A/G	TGTCCATCCCATCCACGCTTCACCAAATCAACGGCCAAACCAACCGCAGCCTTAACCGTCTCCTCAACGCTATCATGTGCCGAGTCGATTATCCCCACCC
Ca_LG_3:11242029	AAGTTTGGATCCAATGTTTCTCTCAGTAGTATCTTCAACGGCCCTAATCACGTGACCCAAAATCTTCTTCCGATTCGCAGCGTACACATGTCCATCCCAT	T/G	CCACGCTTCACCAAATCAACGGCCAAACCAACCGCAGCCTTAACCGTCTCCTCAACGCTATCATGTGCCGAGTCGATTATCCCCACCCGCACCGCCTCCT
Ca_LG_3:11242062	TTCAACGGCCCTAATCACGTGACCCAAAATCTTCTTCCGATTCGCAGCGTACACATGTCCATCCCATCCACGCTTCACCAAATCAACGGCCAAACCAACC	C/A	GCAGCCTTAACCGTCTCCTCAACGCTATCATGTGCCGAGTCGATTATCCCCACCCGCACCGCCTCCTCAGCAGTCATCTTCTCCGCCTGCATCAGGATCC
Ca_LG_3:11242084	CCCAAAATCTTCTTCCGATTCGCAGCGTACACATGTCCATCCCATCCACGCTTCACCAAATCAACGGCCAAACCAACCGCAGCCTTAACCGTCTCCTCAA	A/G	CGCTATCATGTGCCGAGTCGATTATCCCCACCCGCACCGCCTCCTCAGCAGTCATCTTCTCCGCCTGCATCAGGATCCGACGGCGTACCGCAGGTGCTCC
Ca_LG_3:11242095	CTTCCGATTCGCAGCGTACACATGTCCATCCCATCCACGCTTCACCAAATCAACGGCCAAACCAACCGCAGCCTTAACCGTCTCCTCAACGCTATCATGT	T/C	GCCGAGTCGATTATCCCCACCCGCACCGCCTCCTCAGCAGTCATCTTCTCCGCCTGCATCAGGATCCGACGGCGTACCGCAGGTGCTCCGACCTTAGCAT
Ca_LG_3:11242107	AGCGTACACATGTCCATCCCATCCACGCTTCACCAAATCAACGGCCAAACCAACCGCAGCCTTAACCGTCTCCTCAACGCTATCATGTGCCGAGTCGATT	T/A	ATCCCCACCCGCACCGCCTCCTCAGCAGTCATCTTCTCCGCCTGCATCAGGATCCGACGGCGTACCGCAGGTGCTCCGACCTTAGCATCAACGACGGCGA
Ca_LG_3:11689279	GGCCACAAAATAGTGTTTTTCTTGTAGTGGTATAACCATTCATCAATGGATGCAGCTTCCCATATCGAGTATAAGTGCAAACCAATAGTCGCAAAAGTAT	T/G	AAATAATGGCACCATAAATAATATTGTTTCCGTAAAGTAGAGATCTAGAAACATGCTCACGAATACCATCAATATCTACAGGAGGGGCAACAACGAAACC
Ca_LG_3:11689280	GCCACAAAATAGTGTTTTTCTTGTAGTGGTATAACCATTCATCAATGGATGCAGCTTCCCATATCGAGTATAAGTGCAAACCAATAGTCGCAAAAGTATA	A/G	AATAATGGCACCATAAATAATATTGTTTCCGTAAAGTAGAGATCTAGAAACATGCTCACGAATACCATCAATATCTACAGGAGGGGCAACAACGAAACCA
Ca_LG_3:11689294	TTTTTCTTGTAGTGGTATAACCATTCATCAATGGATGCAGCTTCCCATATCGAGTATAAGTGCAAACCAATAGTCGCAAAAGTATAAATAATGGCACCAT	T/G	AAATAATATTGTTTCCGTAAAGTAGAGATCTAGAAACATGCTCACGAATACCATCAATATCTACAGGAGGGGCAACAACGAAACCATAATAAATACATAA
Ca_LG_3:11689311	TAACCATTCATCAATGGATGCAGCTTCCCATATCGAGTATAAGTGCAAACCAATAGTCGCAAAAGTATAAATAATGGCACCATAAATAATATTGTTTCCG	G/A	TAAAGTAGAGATCTAGAAACATGCTCACGAATACCATCAATATCTACAGGAGGGGCAACAACGAAACCATAATAAATACATAAGTTGTTGTCAATAAGGT
Ca_LG_3:11689325	TGGATGCAGCTTCCCATATCGAGTATAAGTGCAAACCAATAGTCGCAAAAGTATAAATAATGGCACCATAAATAATATTGTTTCCGTAAAGTAGAGATCT	T/C	AGAAACATGCTCACGAATACCATCAATATCTACAGGAGGGGCAACAACGAAACCATAATAAATACATAAGTTGTTGTCAATAAGGTAGCATGTATATAAA

Positions	-100 bp flanking sequences	SNPs within *kabuli* cultivar	+100 bp flanking sequences
Ca_LG_3:11689333	GCTTCCCATATCGAGTATAAGTGCAAACCAATAGTCGCAAAAGTATAAATAATGGCACCATAAATAATATTGTTTCCGTAAAGTAGAGATCTAGAAACAT	T/G	GCTCACGAATACCATCAATATCTACAGGAGGGGCAACAACGAAACCATAATAAATACATAAGTTGTTGTCAATAAGGTAGCATGTATATAAAATTGCATA
Ca_LG_3:11689359	ACCAATAGTCGCAAAAGTATAAATAATGGCACCATAAATAATATTGTTTCCGTAAAGTAGAGATCTAGAAACATGCTCACGAATACCATCAATATCTACA	A/T	GGAGGGGCAACAACGAAACCATAATAAATACATAAGTTGTTGTCAATAAGGTAGCATGTATATAAAATTGCATAGTTATGATAAAATCTCAATTAATTAA
Ca_LG_3:11737480	CAATGACACAATTGACCTTATTGTATACAAAAGATTGGTTAAAAAGCAAATTGGAAAAATTATGAGATGTCTAACCAGAGAATGGCAGCCCAAAATAACA	A/T	GCCATAGTTGAATCTAAAGATCTAGCAAAGATGACAACTGCAACATTGTTTGGAAAACTAAGGGAACATGAAATGGAACTGCATAGGCTGGACGAATCAG
Ca_LG_3:11740509	ACAAGCCTCTGAATCTGCAACTGAGCCTCCTGCATATGTTGCAAAATTGATGGAATTTATGGCAGCTCAAATGGCACACAATGCCAAACTGCTAGAGTCC	C/T	AATGCAAGGATGGAAGCTACTTTAACATCTATCCAAACATCCCTGAAGTCAGTTGTTCAGGATGTTGCTGCAATTCAAGCGCACCTAGGCCTCAAGCTAT
Ca_LG_3:11740533	GCCTCCTGCATATGTTGCAAAATTGATGGAATTTATGGCAGCTCAAATGGCACACAATGCCAAACTGCTAGAGTCCAATGCAAGGATGGAAGCTACTTTA	A/G	ACATCTATCCAAACATCCCTGAAGTCAGTTGTTCAGGATGTTGCTGCAATTCAAGCGCACCTAGGCCTCAAGCTATCATTCGAAGACATAGCAGATATTG
Ca_LG_3:12179531	TTTACTTTTAAAGCCAAAGTGTCCATTTGAATAACTGATAGCCGCTACCAGCTATCAGTTATGAGAAGGTTATATCCGCTACCAGCTATCAGCTATGAGA	A/G	AGGCGATAGCCTCTACCAGCTATCAGCTATGAGAAGGCATACCAGCTAGCTAGCTGCTTTATGATAGCCAATAGCTGATGGCCGACAGAGGATATCACCT
Ca_LG_3:12179532	TTACTTTTAAAGCCAAAGTGTCCATTTGAATAACTGATAGCCGCTACCAGCTATCAGTTATGAGAAGGTTATATCCGCTACCAGCTATCAGCTATGAGAA	A/C	GGCGATAGCCTCTACCAGCTATCAGCTATGAGAAGGCATACCAGCTAGCTAGCTGCTTTATGATAGCCAATAGCTGATGGCCGACAGAGGATATCACCTA
Ca_LG_3:12179555	ATTTGAATAACTGATAGCCGCTACCAGCTATCAGTTATGAGAAGGTTATATCCGCTACCAGCTATCAGCTATGAGAAGGCGATAGCCTCTACCAGCTATC	C/G	AGCTATGAGAAGGCATACCAGCTAGCTAGCTGCTTTATGATAGCCAATAGCTGATGGCCGACAGAGGATATCACCTATCAGATATCAAGATATCAGATTT
Ca_LG_3:12812902	CACACTCTTTGTCTCACCACAAAAAGTTTTTGAACTTCCCAGAACCTGTTGCCCCCTACAGAAAGTTGTCTGCCCCACTTGCATCTCTCCCATCCATGTC	C/T	CACATCTGTTGGCACTCTTCCATTTACTTTTGGGTCTCCCAATACAATTGTTGCCACTACTACTCAGCAAACAACTCATCTTCGCCCTCCTCCTCACCAG
Ca_LG_3:13612324	AATAGAAAATAAAAAAGATAAAATTAAGGACCTTGCAGCAAAAAGATCCACAACCAAAAGGTGAATCCAAGCAGAGGCTAAAGTCAACTCACTAGAGAAC	C/T	ATTTTACCTATGCTAGTAAGCTGCATGGAGCATAAAAATATAAACAGTTATTTATGAGTTTGATAAATAGAACTCTTTATGGAAGGAAAAACTTAACTTC
Ca_LG_3:13738851	TCTATGTCAAACTTCATAGCGCTTGAATAAGACATAGCAGCTGCACGCAGACTGTAAACTGTGCACCAGGTAAGTTCCCATGAAAGAGAGCCTCCCTAAC	C/G	CAGCACTTTCACAGTATCACCTTTCCCCTAACAATACCAAGGATAATTCTTTTCTGATGTGGAAGATCAGACAAAGCTGCAACCACAGAGCATACTAAGA
Ca_LG_3:13991853	TGTACAACGGTGTCTTGATTTCATTAAATGCATTTTGATGAGAGTACAATATTTTTTTAAAATTAGTTGAGTTTTGTTATGAAAGTTCATCAAAGTCAAA	A/G	TATCATTTAGCTACACATTTCAGTTTAAATTAACATTATAATGCAGCAGTGTCGAACTTTTAAAAAGAATGTACTATTGAATTCCATATAATTTGGGATT

Positions	-100 bp flanking sequences	SNPs within *kabuli* cultivar	+100 bp flanking sequences
Ca_LG_3:13991859	ACGGTGTCTTGATTTCATTAAATGCATTTTGATGAGAGTACAA TATTTTTTTAAAATTAGTTGAGTTTTGTTATGAAAGTTCATCAAA GTCAAATATCAT	T/A	TTAGCTACACATTTCAGTTTAAATTAACATTATAATGCAGCAG TGTCGAACTTTTAAAAAGAATGTACTATTGAATTCCATATAAT TTGGGATTAATCTC
Ca_LG_3:14395637	CAGCCAAGTTGTATGACATAACATGTATTGCCTTAACCGACGA GCAGCCCATGCCAATGTGCAACATGTTCGTTCAAGCAGTGAA TACCTTGTTTCACAA	A/T	TTGGTGAACTTTTTGCTCAAATAATAAATAGCATGTTCTTTCC TACCAGATTCATCATGATGACTCGTCAAGTACTGTGAAGTAC ATAATAAGTGGTTTC
Ca_LG_3:15400516	GCGACTCTATTGAATTCGATGAAAACTCAAAATTTTAGACCGC GACACGAAGGTTACAGAGTTGTTCAGCTTGAGGCAAACACTA CACACACCTATTGAA	A/T	TGTGGCTGCAATGGTCCTTTCAAACCAGGTTTCACAAACAAC ACATGTCCTGCTAACACAATCAACACATTAGCCAATTTAATTT TTGGTGGTAGACTTG
Ca_LG_3:15424931	TTACTAATCATGATTTAAAGTTTGTTTGTTTGTATGTTATAAATT TAATGCAAGCCTTGAATGCAATGGCCACTGCAATGGCCCAAC AGGCTCAGCAGGC	C/T	TGCAAACCAGGTTTAACTTAATGCACAAAAGGACCAGAGGG AGGAAGCAGCGATTGCTATGATAGGACTGACTAAATTCTGTC GACAAGATCCGCCAAAA
Ca_LG_3:15424937	ATCATGATTTAAAGTTTGTTTGTTTGTATGTTATAAATTTAATG CAAGCCTTGAATGCAATGGCCACTGCAATGGCCCAACAGGCT CAGCAGGCTGCAAA	A/T	CCAGGTTTAACTTAATGCACAAAAGGACCAGAGGGAGGAAG CAGCGATTGCTATGATAGGACTGACTAAATTCTGTCGACAAG ATCCGCCAAAATTTAAA
Ca_LG_3:15424943	ATTTAAAGTTTGTTTGTTTGTATGTTATAAATTTAATGCAAGCC TTGAATGCAATGGCCACTGCAATGGCCCAACAGGCTCAGCAG GCTGCAAACCAGGT	T/C	TTAACTTAATGCACAAAAGGACCAGAGGGAGGAAGCAGCGA TTGCTATGATAGGACTGACTAAATTCTGTCGACAAGATCCGC CAAAATTTAAAGGGGAG
Ca_LG_3:15424957	TGTTTGTATGTTATAAATTTAATGCAAGCCTTGAATGCAATGG CCACTGCAATGGCCCAACAGGCTCAGCAGGCTGCAAACCAG GTTTAACTTAATGCAC	C/A	AAAAGGACCAGAGGGAGGAAGCAGCGATTGCTATGATAGGA CTGACTAAATTCTGTCGACAAGATCCGCCAAAATTTAAAGGG GAGCATGACCCTGACAA
Ca_LG_3:15424961	TGTATGTTATAAATTTAATGCAAGCCTTGAATGCAATGGCCAC TGCAATGGCCCAACAGGCTCAGCAGGCTGCAAACCAGGTTTA ACTTAATGCACAAAA	A/T	GGACCAGAGGGAGGAAGCAGCGATTGCTATGATAGGACTG ACTAAATTCTGTCGACAAGATCCGCCAAAATTTAAAGGGGAG CATGACCCTGACAAGGCT
Ca_LG_3:15424984	GCCTTGAATGCAATGGCCACTGCAATGGCCCAACAGGCTCAG CAGGCTGCAAACCAGGTTTAACTTAATGCACAAAAGGACCAG AGGGAGGAAGCAGCGA	A/G	TTGCTATGATAGGACTGACTAAATTCTGTCGACAAGATCCGC CAAAATTTAAAGGGGAGCATGACCCTGACAAGGCTGATCTC TGGCAGCAGGAGATTGA
Ca_LG_3:15424991	ATGCAATGGCCACTGCAATGGCCCAACAGGCTCAGCAGGCT GCAAACCAGGTTTAACTTAATGCACAAAAGGACCAGAGGGAG GAAGCAGCGATTGCTAT	T/C	GATAGGACTGACTAAATTCTGTCGACAAGATCCGCCAAAATT TAAAGGGGAGCATGACCCTGACAAGGCTGATCTCTGGCAGC AGGAGATTGAGAAGATA
Ca_LG_3:15776921	TCCTCTGATGTTCCTGTTCCATCTCCTTCATCCTCGTTTGAAG AGTCATCCTCCTCCGAGTAACTTTGTGTTTCCTTCTTTTTGCTG CTGCCAAATTCAG	G/A	GGGGAGATAGATCATGATTATCTAGTAGTTGTCTTTGTGTTG AACTAGCTGTTGAGTTGATTTGTTTTGTTTTGTTGCCTATGTA TCTTGTTTGATGTTT
Ca_LG_3:15776941	ATCTCCTTCATCCTCGTTTGAAGAGTCATCCTCCTCCGAGTAA CTTTGTGTTTCCTTCTTTTTGCTGCTGCCAAATTCAGGGGGAG ATAGATCATGATTA	A/T	TCTAGTAGTTGTCTTTGTGTTGAACTAGCTGTTGAGTTGATTT GTTTTGTTTTGTTGCCTATGTATCTTGTTTGATGTTTATCTTG TGTTGAACAAACTT

Positions	-100 bp flanking sequences	SNPs within *kabuli* cultivar	+100 bp flanking sequences
Ca_LG_3:16108747	TATCTTCCTCTTTTGGTGAAGCAACTTTAGGTTCATTGACCAATACAGGTGCAGAATTTGAAGAATTTTCAATAGTAGCAGCCTAAAAGATAAAAATCAA	A/G	AGTAAATGGTTATTGTTAATTTATATTACAATTACAATAAATCGTAGCAGTATTAGGGATGATGAAAATTAATCATTCAAAGTAAACTCGACGAGAACTA
Ca_LG_3:16372227	ACAAAAAAGTTAAAAGTTAGTAACCCTAAAAATAATTGCACACTTGAAGGCACAACAAAGCGGCTGTGTTTGCAGAAAGCACAAGTGCAGCAGCAGCTTA	A/G	ACGGACGCGGCAACGGCTGGGTGTTTTGGTGACACGGCGGACATCCTTAGCGGCTTGGATCCTCAAAACTTGATCATCCAGCTTTAACCGCTCTGAGGCT
Ca_LG_3:16372234	AGTTAAAAGTTAGTAACCCTAAAAATAATTGCACACTTGAAGGCACAACAAAGCGGCTGTGTTTGCAGAAAGCACAAGTGCAGCAGCAGCTTAACGGACG	G/C	CGGCAACGGCTGGGTGTTTTGGTGACACGGCGGACATCCTTAGCGGCTTGGATCCTCAAAACTTGATCATCCAGCTTTAACCGCTCTGAGGCTTTCCATA
Ca_LG_3:16372238	AAAAGTTAGTAACCCTAAAAATAATTGCACACTTGAAGGCACAACAAAGCGGCTGTGTTTGCAGAAAGCACAAGTGCAGCAGCAGCTTAACGGACGCGGC	C/A	AACGGCTGGGTGTTTTGGTGACACGGCGGACATCCTTAGCGGCTTGGATCCTCAAAACTTGATCATCCAGCTTTAACCGCTCTGAGGCTTTCCATACGGC
Ca_LG_3:16372239	AAAGTTAGTAACCCTAAAAATAATTGCACACTTGAAGGCACAACAAAGCGGCTGTGTTTGCAGAAAGCACAAGTGCAGCAGCAGCTTAACGGACGCGGCA	A/T	ACGGCTGGGTGTTTTGGTGACACGGCGGACATCCTTAGCGGCTTGGATCCTCAAAACTTGATCATCCAGCTTTAACCGCTCTGAGGCTTTCCATACGGCC
Ca_LG_3:16372247	TAACCCTAAAAATAATTGCACACTTGAAGGCACAACAAAGCGGCTGTGTTTGCAGAAAGCACAAGTGCAGCAGCAGCTTAACGGACGCGGCAACGGCTGG	G/T	GTGTTTTGGTGACACGGCGGACATCCTTAGCGGCTTGGATCCTCAAAACTTGATCATCCAGCTTTAACCGCTCTGAGGCTTTCCATACGGCCGCCATCTT
Ca_LG_3:16372260	AATTGCACACTTGAAGGCACAACAAAGCGGCTGTGTTTGCAGAAAGCACAAGTGCAGCAGCAGCTTAACGGACGCGGCAACGGCTGGGTGTTTTGGTGAC	C/T	ACGGCGGACATCCTTAGCGGCTTGGATCCTCAAAACTTGATCATCCAGCTTTAACCGCTCTGAGGCTTTCCATACGGCCGCCATCTTTATCATTGTTCTC
Ca_LG_3:16372261	ATTGCACACTTGAAGGCACAACAAAGCGGCTGTGTTTGCAGAAAGCACAAGTGCAGCAGCAGCTTAACGGACGCGGCAACGGCTGGGTGTTTTGGTGACA	A/G	CGGCGGACATCCTTAGCGGCTTGGATCCTCAAAACTTGATCATCCAGCTTTAACCGCTCTGAGGCTTTCCATACGGCCGCCATCTTTATCATTGTTCTCC
Ca_LG_3:16372262	TTGCACACTTGAAGGCACAACAAAGCGGCTGTGTTTGCAGAAAGCACAAGTGCAGCAGCAGCTTAACGGACGCGGCAACGGCTGGGTGTTTTGGTGACAC	C/T	GGCGGACATCCTTAGCGGCTTGGATCCTCAAAACTTGATCATCCAGCTTTAACCGCTCTGAGGCTTTCCATACGGCCGCCATCTTTATCATTGTTCTCCA
Ca_LG_3:16372266	ACACTTGAAGGCACAACAAAGCGGCTGTGTTTGCAGAAAGCACAAGTGCAGCAGCAGCTTAACGGACGCGGCAACGGCTGGGTGTTTTGGTGACACGGCG	G/A	GACATCCTTAGCGGCTTGGATCCTCAAAACTTGATCATCCAGCTTTAACCGCTCTGAGGCTTTCCATACGGCCGCCATCTTTATCATTGTTCTCCAAGGT
Ca_LG_3:16372289	GCTGTGTTTGCAGAAAGCACAAGTGCAGCAGCAGCTTAACGGACGCGGCAACGGCTGGGTGTTTTGGTGACACGGCGGACATCCTTAGCGGCTTGGATCC	C/T	TCAAAACTTGATCATCCAGCTTTAACCGCTCTGAGGCTTTCCATACGGCCGCCATCTTTATCATTGTTCTCCAAGGTTGTAAGAGAAAATCTCCATTGCT
Ca_LG_3:16372308	CAAGTGCAGCAGCAGCTTAACGGACGCGGCAACGGCTGGGTGTTTTGGTGACACGGCGGACATCCTTAGCGGCTTGGATCCTCAAAACTTGATCATCCAG	G/A	CTTTAACCGCTCTGAGGCTTTCCATACGGCCGCCATCTTTATCATTGTTCTCCAAGGTTGTAAGAGAAAATCTCCATTGCTCGAATTTGAGAGAGAGAGA

Positions	-100 bp flanking sequences	SNPs within *kabuli* cultivar	+100 bp flanking sequences
Ca_LG_3:16697127	AATTGAAGAAAGTGACAATGTTTGAATATGTGCGTATTTAATATTGCTACACTGTAATTTTATTAATGAACATCTAAATCACCTAATTCCATAGCAGAAT	T/C	CCGACCTTGGAATTTAATGTGTTAAGCATATGATTTTTCCGTCTTATCCAAAGTGGTCAGAAATGTGAGACAGCTTTTTTGTACTCTATATTTTTTGTAT
Ca_LG_3:16697142	CAATGTTTGAATATGTGCGTATTTAATATTGCTACACTGTAATTTTATTAATGAACATCTAAATCACCTAATTCCATAGCAGAATCCGACCTTGGAATTT	T/G	AATGTGTTAAGCATATGATTTTTCCGTCTTATCCAAAGTGGTCAGAAATGTGAGACAGCTTTTTTGTACTCTATATTTTTTGTATGAATTTCTGGTTCTG
Ca_LG_3:16854692	TTTTTCTTTGTCTTTAATGTTAGGAAAAAATAGCTAAGCAAGCTATATAACATACCAAAGCCTGAACTCCGCCAGCTGCTGCAATGGCCTCTCGATTTCT	T/C	TTCGTCAAATGACAAATGACAAATTCCAAAATGAACCAGCAGCTTCTTGCCTATAGAAATACAACAATGTCAAATCAACACTGTAATGAATGTAATATTT
Ca_LG_3:16854754	TGAACTCCGCCAGCTGCTGCAATGGCCTCTCGATTTCTTTCGTCAAATGACAAATGACAAATTCCAAAATGAACCAGCAGCTTCTTGCCTATAGAAATAC	C/T	AACAATGTCAAATCAACACTGTAATGAATGTAATATTTTTCTAGGTACAAGAGAATAGAACAAAGAGAAGCACTATCTCAAAAGCAGGAGCTATTAGGTG
Ca_LG_3:16854774	AATGGCCTCTCGATTTCTTTCGTCAAATGACAAATGACAAATTCCAAAATGAACCAGCAGCTTCTTGCCTATAGAAATACAACAATGTCAAATCAACACT	T/A	GTAATGAATGTAATATTTTTCTAGGTACAAGAGAATAGAACAAAGAGAAGCACTATCTCAAAAGCAGGAGCTATTAGGTGAAAGAACGTGAATGATTTTG
Ca_LG_3:17621916	GTTTAATATCTAAATCAAACAGGTCACATATTAGTTTTAAAGTTGCATGTGACAAGAAATAGGTTTAATGCATCGCGAAATTCAGCTGCTGCAGCAGTGG	G/T	GAATATCGCGTCAGCAGCGTTTTGCGCAGCAGCAGGAGCCGCTGAAAAGATAGCAGTCGCAAAATGACAGTGCTGCCAACCTCTGACTTCCGTAGATTAG
Ca_LG_3:18077530	TTTTTTAGACATCTAGAACAGATTAGTCTGAAGAGCCTCAATCACTGACAATCTTTCGCAGCTTGCATTTACAGGTTCAACTGATACTGGAAAAGCCGTT	T/C	CTTCAACTGGCAGCAAAAAGCAATCTTAAACCTGTAACTTTGGAGCTTGGTGGAAAATCTCCTTTTATTGTATGCGAAGATGCTGATGTAGATCAAGCTG
Ca_LG_3:18314841	ACAAACTTTCTACCATTAGCACATTTACATGGAAAATAGTATCAATCCAATATCTTGTGCTATTTGTTCTACCACATTCAAATAAAATGGCAAGAGCAGT	T/C	AATGATCATGCTTTGACCATCATATGATTCCAACGTCATTTTCTTCAATCTCACTTCCATAAGATTTGGAGTTACTATTATCATTTACACTTGGTGTAGA
Ca_LG_3:18314872	GAAAATAGTATCAATCCAATATCTTGTGCTATTTGTTCTACCACATTCAAATAAAATGGCAAGAGCAGTAATGATCATGCTTTGACCATCATATGATTCC	C/A	AACGTCATTTTCTTCAATCTCACTTCCATAAGATTTGGAGTTACTATTATCATTTACACTTGGTGTAGAGCTAGCAGTGCTGCTTGTGGCATGCGGTGAA
Ca_LG_3:18314875	AATAGTATCAATCCAATATCTTGTGCTATTTGTTCTACCACATTCAAATAAAATGGCAAGAGCAGTAATGATCATGCTTTGACCATCATATGATTCCAAC	C/A	GTCATTTTCTTCAATCTCACTTCCATAAGATTTGGAGTTACTATTATCATTTACACTTGGTGTAGAGCTAGCAGTGCTGCTTGTGGCATGCGGTGAATCA
Ca_LG_3:19643788	TTGGTGAGGGTTATGATCCTTTAAATAGTTCATCAAAATGAAGAGGAGAGATATGTGTGAGAATTTCAATGGCGGCAATGTCTAGAGTGGAAGAAGACTA	A/G	CATCAGGAAGAAGACTGTGATGCTGATTTTTAATTTGTGGTGGGGGTGCAGCTAGCGATTACCTAGGTGCCAAAAGTAGCAGATTAAAAGACTTAAAATG
Ca_LG_3:19643792	TGAGGGTTATGATCCTTTAAATAGTTCATCAAAATGAAGAGGAGAGATATGTGTGAGAATTTCAATGGCGGCAATGTCTAGAGTGGAAGAAGACTACATC	C/T	AGGAAGAAGACTGTGATGCTGATTTTTAATTTGTGGTGGGGGTGCAGCTAGCGATTACCTAGGTGCCAAAAGTAGCAGATTAAAAGACTTAAAATGGAGT

Positions	-100 bp flanking sequences	SNPs within *kabuli* cultivar	+100 bp flanking sequences
Ca_LG_3:19643806	CTTTAAATAGTTCATCAAAATGAAGAGGAGAGATATGTGTGAGAATTTCAATGGCGGCAATGTCTAGAGTGGAAGAAGACTACATCAGGAAGAAGACTGT	T/C	GATGCTGATTTTTAATTTGTGGTGGGGGTGCAGCTAGCGATTACCTAGGTGCCAAAAGTAGCAGATTAAAAGACTTAAAATGGAGTGGGCCAAAGTCCAT
Ca_LG_3:20244569	TCATATTGTGTCAACACATGTGTCACCACCAGTCCCCTGGAGAATTCGGGCTGCTGATCCTGCTCCTCAACATCATGAATATCATCAGCATGTGCAGGGA	A/G	CATTAGCAGCATCATCAACATCATGAATATCATCATCAACAGCATGTGCATCACACTACTCCTCAGCCTGCTCCATTTGTTGATGCTCATGGACATGCTC
Ca_LG_3:20663037	TCCACGATTAAATAATATTTCATTCTGGTTGTTGCCGCCAAGTCTCTTGCTCCTATTAAGCTCAGCCTTAGTAGAGGTGGGTAGCGGCACTGGGTGGACA	A/G	GTCTATCCGCCCTTAAGTGGTATTACCAGCCATTCTGGAGGAGCAGTTGATTCAGCAATTTCTAGTCTTCATCTATCTGGTGTTTCATCCATTTTAGGTT
Ca_LG_3:20663045	TAAATAATATTTCATTCTGGTTGTTGCCGCCAAGTCTCTTGCTCCTATTAAGCTCAGCCTTAGTAGAGGTGGGTAGCGGCACTGGGTGGACAGTCTATCC	C/T	GCCCTTAAGTGGTATTACCAGCCATTCTGGAGGAGCAGTTGATTCAGCAATTTCTAGTCTTCATCTATCTGGTGTTTCATCCATTTTAGGTTCTATCAAT
Ca_LG_3:20917708	ACTTTGAAAAATTTCTTGGTCACTTTTTATTTTAAATTTTAGATTTTATTAATTATTTCTTTACCAAAATACTCCTAATTAATTTACATCTTTTTAACTT	T/G	CCCAAATCTTCTTTTGCTTCGTTATTGGCTTCTCTTTGTAGCTTATACCCAACCTGCAGCACTTGTCACTGGGCGAGAAAGAATTGAAGTTGATTTTGCA
Ca_LG_3:21245355	ATCCCTTAACAGAGTTATGAATTGTTTTAAAGGATTGAGATTTATTAGCAACTATTATTTCCTCTTTTTTATTTTTCCAATGCTTGTAGCAGCATATTGA	A/T	TCCTTTTCTATTTTTTATTTCCTTCATTTTCTGTCATAGCACTGAAACAAGTCTCATGATAAGTTTAATGTTGCTGTTTGGTCTGAACTAAGAGTGGTTG
Ca_LG_3:21245410	TATTTCCTCTTTTTTATTTTTCCAATGCTTGTAGCAGCATATTGATCCTTTTCTATTTTTTATTTCCTTCATTTTCTGTCATAGCACTGAAACAAGTCTC	C/T	ATGATAAGTTTAATGTTGCTGTTTGGTCTGAACTAAGAGTGGTTGCAAATTGAAAGTTTACTTTGTAGGATTGTTATGACATGTAGAATTTAACACAAGT
Ca_LG_3:21773230	AAAATCAGATGATGACTGCATAGAGTAGTGGTATCTTGACATAGGCATCTCTAATCTTAAAGTGCTTAGGAGAGATGGAGGTGGTGATTATCAATTCCAT	T/C	GAGTTCCATGAATTATGTGACATAGAGGGAATGATTCATGAAGTGACTACACCATATATACCACAACATAATGGAGCTGTAAAAACAGGAAGATAACCAT
Ca_LG_3:21773263	TCTTGACATAGGCATCTCTAATCTTAAAGTGCTTAGGAGAGATGGAGGTGGTGATTATCAATTCCATGAGTTCCATGAATTATGTGACATAGAGGGAATG	G/A	ATTCATGAAGTGACTACACCATATATACCACAACATAATGGAGCTGTAAAAACAGGAAGATAACCATTCTCAATATGGCAAGAAGCATGTTGAAGGGAGA
Ca_LG_3:21786287	CTCTTGCACTTTCAACTTATGTTGATGCTAATTGGGGCAGCATTGTGGATACTAGAAGATCCACTACTGGTTTTTGCCTCTTCATTGGTGATGCTTTAGC	C/T	TTCTTGGAAGGCAAAAAAAAAAAAACAACTTACAGTCTCTAAATCTTCTGTTGCAGCTAAGTATAGGGCATTGTCTTCTACTGCTAGTGAATTTGTTTGG
Ca_LG_3:21786338	CTAGAAGATCCACTACTGGTTTTTGCCTCTTCATTGGTGATGCTTTAGCTTCTTGGAAGGCAAAAAAAAAAAAACAACTTACAGTCTCTAAATCTTCTGT	T/C	TGCAGCTAAGTATAGGGCATTGTCTTCTACTGCTAGTGAATTTGTTTGGTTGCGACATTTACTTGTTCACTTTGAAGTCTCTCCAACTTCAGTTATGGTT
Ca_LG_3:21787965	GAATATCAGCTTAAAACAACTTATAGACATGTCAAAAACTGCAACAAAGTTTGCATTTAAATTCCCTACTTTTCACACATTTCAGCAGCAAAATCAAGAT	T/A	AAACAAAAAGAAAAAACATGAAATTCCATTTTATGTTCCAAACTTTATCTCTCTCTCTAGAGCAAGAAAGTAAAGAATCCGAAGAAAACCCATGAAAGCA

Positions	-100 bp flanking sequences	SNPs within *kabuli* cultivar	+100 bp flanking sequences
Ca_LG_3:22567722	GTTTCCACCTCTAACCCTAAGTCATTATTAGAATTGGAGCATCCCTGAATCCATGGTTTTGAATTAGCAATAGAAGTAGGGGCACCATCAACAACAACAA	A/G	CATGTTCCTTAAGAGCTTTGATGGAATGATTCTGCCTGTTAGATGCTGAAAAAGTTGGAGCACATTTTGATGACTTCTTTGAAAATATTACAGATTTTGA
Ca_LG_3:22567727	CACCTCTAACCCTAAGTCATTATTAGAATTGGAGCATCCCTGAATCCATGGTTTTGAATTAGCAATAGAAGTAGGGGCACCATCAACAACAACAACATGT	T/C	TCCTTAAGAGCTTTGATGGAATGATTCTGCCTGTTAGATGCTGAAAAAGTTGGAGCACATTTTGATGACTTCTTTGAAAATATTACAGATTTTGAAGGAG
Ca_LG_3:22672155	AATAGGAGCTGGATCAGACGTATTTCCTGATAAGCAACTCAATCCTGAAGCAGCATACAAAGAAATGGACACCATGAAACTTAATTTGATATAATAATCT	T/C	CTTGCTTAACTTTATGATATAATAAAAATAATAAAATTTCAGCTATGTTTTGTTCTCTTATTCTTGCACTTGAATGGACAGTGCAGGCAATCAAATAAAT
Ca_LG_3:22672159	GGAGCTGGATCAGACGTATTTCCTGATAAGCAACTCAATCCTGAAGCAGCATACAAAGAAATGGACACCATGAAACTTAATTTGATATAATAATCTCTTG	G/A	CTTAACTTTATGATATAATAAAAATAATAAAATTTCAGCTATGTTTTGTTCTCTTATTCTTGCACTTGAATGGACAGTGCAGGCAATCAAATAAATAATC
Ca_LG_3:22672188	GCAACTCAATCCTGAAGCAGCATACAAAGAAATGGACACCATGAAACTTAATTTGATATAATAATCTCTTGCTTAACTTTATGATATAATAAAAATAATA	A/T	AAATTTCAGCTATGTTTTGTTCTCTTATTCTTGCACTTGAATGGACAGTGCAGGCAATCAAATAAATAATCTCAGGAGGACAAATAGAAAAGGGGCAATT
Ca_LG_3:22718591	ACGATATGAATGCTTGACTCGTGATTCTTCCATTGCTGCTAATGCTATTTTTGTACTATGCTGGACTTGACATTGCTTCTTATCTTTGGTGTTCTTTGGG	G/A	GTTACAATAGTTCAATGAGAGATGCTGCTTGTCTTGATCTTGTTGATTGGCGGGTGAAGAGACCCGAATGGGGATTCAGGAATGGGAAGGGTCATTTTCC
Ca_LG_3:23135840	ATCGTAGACTAGCAGCCATGCCTTGAATTATCTTCTCCAATTTACTCTTTCTTCATCAATGGACTTCATTGCGCTAGTTATCACACATTGCAAGTATCAA	A/T	CTCTGCAGCTCCTTTCCAATGCACATCGACTTTGTTCTCATTTTCCTTCCTTATCGCAACACCACTCCGTTTCTTCTCCGAGTTAAATGTTTCAACATGA
Ca_LG_3:23233656	AATTACCAGTGGTGCGAGAACGGTGAGAACGACGGTGAGTGCAGCTCGGGTTCGACGGCGCGACTGGTTCGACGGCGAGTGCAGCTCCGGTTCGACGGCG	G/A	AGTGCGCGACTGGTTTGACGGCGCGACTGGTTCGACGGTGAGTGCAGCTGGTTGATAGACGACGAGAATGGTGAGAACGACCGTTTTAAGTGAGAACGGC
Ca_LG_3:23888880	AAAATTAAGTAATTAACTTACATGCTTGATATGCTGTCATTTTAGTTGTCATTTTTCTGCAGTTCAGATCCCTGTGATTTGAACATTTGCTGCTGTGATG	G/T	ATGAGGATCGGAGAACCAAAAACAACTGCACTTATCTTTGCATCTGGCAAAATGGTATCTTTGCATTTGGCGTTTTATTGCAATAGCTAAATAGTTTAGT
Ca_LG_3:23888890	AATTAACTTACATGCTTGATATGCTGTCATTTTAGTTGTCATTTTTCTGCAGTTCAGATCCCTGTGATTTGAACATTTGCTGCTGTGATGATGAGGATCG	G/A	GAGAACCAAAAACAACTGCACTTATCTTTGCATCTGGCAAAATGGTATCTTTGCATTTGGCGTTTTATTGCAATAGCTAAATAGTTTAGTCCTTTTACTG
Ca_LG_3:23888913	CTGTCATTTTAGTTGTCATTTTTCTGCAGTTCAGATCCCTGTGATTTGAACATTTGCTGCTGTGATGATGAGGATCGGAGAACCAAAAACAACTGCACTT	T/C	ATCTTTGCATCTGGCAAAATGGTATCTTTGCATTTGGCGTTTTATTGCAATAGCTAAATAGTTTAGTCCTTTTACTGTTGTTGGCTACCTTGGTTTTAGT
Ca_LG_3:23888922	TAGTTGTCATTTTTCTGCAGTTCAGATCCCTGTGATTTGAACATTTGCTGCTGTGATGATGAGGATCGGAGAACCAAAAACAACTGCACTTATCTTTGCA	A/G	TCTGGCAAAATGGTATCTTTGCATTTGGCGTTTTATTGCAATAGCTAAATAGTTTAGTCCTTTTACTGTTGTTGGCTACCTTGGTTTTAGTTGGGTGCCT

Positions	-100 bp flanking sequences	SNPs within *kabuli* cultivar	+100 bp flanking sequences
Ca_LG_3:23888931	TTTTTCTGCAGTTCAGATCCCTGTGATTTGAACATTTGCTGCTGTGATGATGAGGATCGGAGAACCAAAAACAACTGCACTTATCTTTGCATCTGGCAAA	A/G	ATGGTATCTTTGCATTTGGCGTTTTATTGCAATAGCTAAATAGTTTAGTCCTTTTACTGTTGTTGGCTACCTTGGTTTTAGTTGGGTGCCTTAAATTCCA
Ca_LG_3:24020120	GCCTTTTGAAGGAATGTCTAATTTACAAGCCGCTTATGCTGCTGCCTTCAAGGTATTCAATCCGAAATTACCAAGTGTTTGTTTATTCATCAATCAGCCT	T/G	GTTTACTTTCCATGCTTCCGGTTTTCATTGTTTTTCTGCCCGGAACAAAGGAAAGTTTGCTTTTCTTCGCCAATTGTTTTCTTTGAAAAGGTTAAAAAAC
Ca_LG_3:24020135	GTCTAATTTACAAGCCGCTTATGCTGCTGCCTTCAAGGTATTCAATCCGAAATTACCAAGTGTTTGTTTATTCATCAATCAGCCTGTTTACTTTCCATGC	C/T	TTCCGGTTTTCATTGTTTTTCTGCCCGGAACAAAGGAAAGTTTGCTTTTCTTCGCCAATTGTTTTCTTTGAAAAGGTTAAAAAACTAACAGTAAGTGCTA
Ca_LG_3:24042167	ACCATGTGGTCAAGTGCAACTATATGTAGCATCATGCGTTGGTTATCTAAGGAGCCCAGGTCCATCTGTACCAGAACCATGCTGCAATGGGATTAGAACT	T/A	GTCAATAGTCAATCAAAAACAACTGCTGATCGTCAAAGTGTATGTAATTGTCTCAAATCAACTGCACTGAGCTTCCCTGGACTCAATCCTCAAGCACTTG
Ca_LG_3:24042170	ATGTGGTCAAGTGCAACTATATGTAGCATCATGCGTTGGTTATCTAAGGAGCCCAGGTCCATCTGTACCAGAACCATGCTGCAATGGGATTAGAACTGTC	C/A	AATAGTCAATCAAAAACAACTGCTGATCGTCAAAGTGTATGTAATTGTCTCAAATCAACTGCACTGAGCTTCCCTGGACTCAATCCTCAAGCACTTGCTG
Ca_LG_3:24042173	TGGTCAAGTGCAACTATATGTAGCATCATGCGTTGGTTATCTAAGGAGCCCAGGTCCATCTGTACCAGAACCATGCTGCAATGGGATTAGAACTGTCAAT	T/C	AGTCAATCAAAAACAACTGCTGATCGTCAAAGTGTATGTAATTGTCTCAAATCAACTGCACTGAGCTTCCCTGGACTCAATCCTCAAGCACTTGCTGCTC
Ca_LG_3:24042192	GTAGCATCATGCGTTGGTTATCTAAGGAGCCCAGGTCCATCTGTACCAGAACCATGCTGCAATGGGATTAGAACTGTCAATAGTCAATCAAAAACAACTG	G/T	CTGATCGTCAAAGTGTATGTAATTGTCTCAAATCAACTGCACTGAGCTTCCCTGGACTCAATCCTCAAGCACTTGCTGCTCTTCCTGCTAATTGTGGTGT
Ca_LG_3:24042215	AAGGAGCCCAGGTCCATCTGTACCAGAACCATGCTGCAATGGGATTAGAACTGTCAATAGTCAATCAAAAACAACTGCTGATCGTCAAAGTGTATGTAAT	T/G	TGTCTCAAATCAACTGCACTGAGCTTCCCTGGACTCAATCCTCAAGCACTTGCTGCTCTTCCTGCTAATTGTGGTGTTAACTTGCCATACAAAATTTCCA
Ca_LG_3:24042225	GGTCCATCTGTACCAGAACCATGCTGCAATGGGATTAGAACTGTCAATAGTCAATCAAAAACAACTGCTGATCGTCAAAGTGTATGTAATTGTCTCAAAT	T/A	CAACTGCACTGAGCTTCCCTGGACTCAATCCTCAAGCACTTGCTGCTCTTCCTGCTAATTGTGGTGTTAACTTGCCATACAAAATTTCCACCTCCATTGA
Ca_LG_3:24042231	TCTGTACCAGAACCATGCTGCAATGGGATTAGAACTGTCAATAGTCAATCAAAAACAACTGCTGATCGTCAAAGTGTATGTAATTGTCTCAAATCAACTG	G/T	CACTGAGCTTCCCTGGACTCAATCCTCAAGCACTTGCTGCTCTTCCTGCTAATTGTGGTGTTAACTTGCCATACAAAATTTCCACCTCCATTGACTGCAA
Ca_LG_3:24333072	GATCAAACGAAGCAAGTTTGCTACACACATGTTACCACCACCGGAACCAAACCCTTATTTTTCTTCTCTGGCAGTCGTTCTCTTTGTTCTTCTACATTCA	A/G	GATGTAACATCCGAGCCTAGGAAGGAAGAGGGAAGGCAAGGGTCCCAGGAGCTATCAGTATTAGCTGCTGACTGGTGACGAGAGGATCATATAGTTTGAG
Ca_LG_3:24942309	CTAGAAGAAACTAATCTTCAATGTTTTCATTAACAGCAATGGATCAAATGTTCGTGGTTACTTCATTTGGGCAGCATTTGATACATTTGAATTTCAAGCG	G/A	GGATATTCAGGACATTGGGGACTTTATCATGTTGATTTTAATGATAATCTCAAGCGTATACCAAAGGACTCTGCTGAATGGTATAGAAAGTTTCTGATAC

**Positions**	**-100 bp flanking sequences**	**SNPs within *kabuli* cultivar**	**+100 bp flanking sequences**
Ca_LG_3:24942312	GAAGAAACTAATCTTCAATGTTTTCATTAACAGCAATGGATCAAATGTTCGTGGTTACTTCATTTGGGCAGCATTTGATACATTTGAATTTCAAGCGGGA	A/C	TATTCAGGACATTGGGGACTTTATCATGTTGATTTTAATGATAATCTCAAGCGTATACCAAAGGACTCTGCTGAATGGTATAGAAAGTTTCTGATACCAG
Ca_LG_3:24942318	ACTAATCTTCAATGTTTTCATTAACAGCAATGGATCAAATGTTCGTGGTTACTTCATTTGGGCAGCATTTGATACATTTGAATTTCAAGCGGGATATTCA	A/T	GGACATTGGGGACTTTATCATGTTGATTTTAATGATAATCTCAAGCGTATACCAAAGGACTCTGCTGAATGGTATAGAAAGTTTCTGATACCAGAGTGTG
Ca_LG_3:24942322	ATCTTCAATGTTTTCATTAACAGCAATGGATCAAATGTTCGTGGTTACTTCATTTGGGCAGCATTTGATACATTTGAATTTCAAGCGGGATATTCAGGAC	C/A	ATTGGGGACTTTATCATGTTGATTTTAATGATAATCTCAAGCGTATACCAAAGGACTCTGCTGAATGGTATAGAAAGTTTCTGATACCAGAGTGTGGACA
Ca_LG_3:25396854	TCCCCCATCGTCTCACTGGCCGACCTTGACCCTTCTTATTTTGGGGTCATATCTAGTATTCAGAGTTTGCCTCGATTTGGTACCGCTCTCGCGGCCCGCA	A/G	CTGAAACAGTGCTTTACCCCTAGATGTCCAGTCAACTGCTGCGCCTCAACGCATTTCGGGGAGAACCAGCTAGCTCTGGGTTCGAGTGGCATTTCACCGC
Ca_LG_3:25396856	CCCCATCGTCTCACTGGCCGACCTTGACCCTTCTTATTTTGGGGTCATATCTAGTATTCAGAGTTTGCCTCGATTTGGTACCGCTCTCGCGGCCCGCACT	T/C	GAAACAGTGCTTTACCCCTAGATGTCCAGTCAACTGCTGCGCCTCAACGCATTTCGGGGAGAACCAGCTAGCTCTGGGTTCGAGTGGCATTTCACCGCTA
Ca_LG_3:25419260	CAACGACAATGGGGATGATCAAATGGCTGAAGCAATGAATAACATGGTTGCTTCTGTTGCTGCACAGACTACTGCATAGACTCTGCGAGATCTGGAGAAA	A/G	AGGGAAATAGAGATATGTGCTGTTGCGTCAAGGGGATTAGAAGATTTTAGTCGTTATCATCCTCCAAAGTTCAAGGGTGATGAGAACTCAGAAAACGCGG
Ca_LG_3:25654293	GAAAAGAAAGCAAAAGCAATTTTTGTAGTAGTGGCGAACCAAATGAGAACAACCTAAAGTGTGGGGTTGTGGGAGACCAATACAAGTATCGTGCTGCTAT	T/G	GCGAAGTAATCAAATGTTGCACCCTAGATGGTGAAAGTCCAGTATTCGAAAGTATCACTAGCTTATGCTCTAACTCAAGTAGCATAGGGCACGTGGAATC
Ca_LG_3:25654300	AAGCAAAAGCAATTTTTGTAGTAGTGGCGAACCAAATGAGAACAACCTAAAGTGTGGGGTTGTGGGAGACCAATACAAGTATCGTGCTGCTATGCGAAGT	T/C	AATCAAATGTTGCACCCTAGATGGTGAAAGTCCAGTATTCGAAAGTATCACTAGCTTATGCTCTAACTCAAGTAGCATAGGGCACGTGGAATCCCGTGGG
Ca_LG_3:25796940	CTTTGTCAGGTGCCAACATATACGGGGTCCGCCCCCTTTCATCTTTGATGCATGGATCCAAGCCTTGTTCCAGGAGTTCCATAACCTTTTCAGAGTCACC	C/T	AGATTGTGCTGCTTCATGTAAAGGAGTTGACTTAGCAGGCAGCTCATTTTCATTTTCGCCATTACTCAAAATAAGAGCTTCATCATTTTGGTTACTTGAA
Ca_LG_3:26268417	AAAATGTCAATACACTCACAATTTTCAGCTCCAATATGTTTAAGAACACCTTTCACCCAAACACAATGACAAGCACATGAGGTTGCTGCCACATATTCAA	A/G	CTTTTGTGGAGGATAATGTAACTATGGCCTGCTTCTTTGAGCTCCAACACACAGCTGCCTCACTTAGCAGAAAGACATAGCCAGAAGTACTCTTTCTGTC
Ca_LG_3:27394533	AGGCGTCGATGGAGCCAGTCCATCTACTGCTATCAGAACAGTAGGTCTCGAAAGAGTTCAAATGATGACGATACATCTTGCATCTGTTGGAGAAACCTAT	T/C	GCTCAGGCCAGCTTCGATGAAGCTGCTTTAAAGATATCGCGACCGACTCTACTGAAAGATACCCCAAAAGATGTATGAAATTTCTTTTAAAAAAGCCCTC
Ca_LG_3:27394549	AGTCCATCTACTGCTATCAGAACAGTAGGTCTCGAAAGAGTTCAAATGATGACGATACATCTTGCATCTGTTGGAGAAACCTATGCTCAGGCCAGCTTCG	G/C	ATGAAGCTGCTTTAAAGATATCGCGACCGACTCTACTGAAAGATACCCCAAAAGATGTATGAAATTTCTTTTAAAAAAGCCCTCTAATTATTGTTGATTT

Positions	-100 bp flanking sequences	SNPs within *kabuli* cultivar	+100 bp flanking sequences
Ca_LG_3:27397559	AATGAATACATAAGAGAGGTGTTGAGAGACCTTGGAACAGAACATCAGTGAATCACCTCCACCTCCATATGCTGTTTCGGTCAGATTTTAAAAAACAGTA	A/T	ACTGCATATGATCATAGAATTATAAAGATTAGCTTATCTCAGCAAAATGCAACTTCACTTTATCATTTAAAATGAGGTGCATTTTATGATGCTAGGAACG
Ca_LG_3:27397590	TTGGAACAGAACATCAGTGAATCACCTCCACCTCCATATGCTGTTTCGGTCAGATTTTAAAAAACAGTAACTGCATATGATCATAGAATTATAAAGATTA	A/G	GCTTATCTCAGCAAAATGCAACTTCACTTTATCATTTAAAATGAGGTGCATTTTATGATGCTAGGAACGATTTCTTAGTTAGAACCTCTTACATTTTGGT
Ca_LG_3:27397591	TGGAACAGAACATCAGTGAATCACCTCCACCTCCATATGCTGTTTCGGTCAGATTTTAAAAAACAGTAACTGCATATGATCATAGAATTATAAAGATTAG	G/A	CTTATCTCAGCAAAATGCAACTTCACTTTATCATTTAAAATGAGGTGCATTTTATGATGCTAGGAACGATTTCTTAGTTAGAACCTCTTACATTTTGGTG
Ca_LG_3:27613844	AAATGCTGAACCTGTTCAGCCTTCTACACAACAAGCTGCTGAGCCAGTGGCAGCCACTCCTGAGTTCTTCGCAAAGATGCTAGAGTTCATGGAAGCTCAA	A/G	GCGGCTCACAACGCTAAGATGCAAGCGTCAAATGATCGAATGGAAGCTGCTTTGAAGACACTCCAGACATCTCTGAACTCAGTTGTTCAGAATGTTGATG
Ca_LG_3:27875755	ATCGATAAGAGCAACAACATCAGTGGTGATTATGTCCACATATTTATTTATGAAAATCCCAAGACCTCTTATTAGGTAGAAGTCCAATCCTATAGCTTCC	C/T	TCAATGCTAGGACGTTGCACCTTGACAGCCACGAGTTTACCCGAATACTTCAACCGAGCTTTATAAACTTGACCTAAACTAGCTGCAGCCATAGGTGATG
Ca_LG_3:27875758	GATAAGAGCAACAACATCAGTGGTGATTATGTCCACATATTTATTTATGAAAATCCCAAGACCTCTTATTAGGTAGAAGTCCAATCCTATAGCTTCCTCA	A/G	ATGCTAGGACGTTGCACCTTGACAGCCACGAGTTTACCCGAATACTTCAACCGAGCTTTATAAACTTGACCTAAACTAGCTGCAGCCATAGGTGATGGTG
Ca_LG_3:27875763	GAGCAACAACATCAGTGGTGATTATGTCCACATATTTATTTATGAAAATCCCAAGACCTCTTATTAGGTAGAAGTCCAATCCTATAGCTTCCTCAATGCT	T/C	AGGACGTTGCACCTTGACAGCCACGAGTTTACCCGAATACTTCAACCGAGCTTTATAAACTTGACCTAAACTAGCTGCAGCCATAGGTGATGGTGTTATA
Ca_LG_3:27875767	AACAACATCAGTGGTGATTATGTCCACATATTTATTTATGAAAATCCCAAGACCTCTTATTAGGTAGAAGTCCAATCCTATAGCTTCCTCAATGCTAGGA	A/G	CGTTGCACCTTGACAGCCACGAGTTTACCCGAATACTTCAACCGAGCTTTATAAACTTGACCTAAACTAGCTGCAGCCATAGGTGATGGTGTTATAGAAG
Ca_LG_3:27875785	TATGTCCACATATTTATTTATGAAAATCCCAAGACCTCTTATTAGGTAGAAGTCCAATCCTATAGCTTCCTCAATGCTAGGACGTTGCACCTTGACAGCC	C/A	ACGAGTTTACCCGAATACTTCAACCGAGCTTTATAAACTTGACCTAAACTAGCTGCAGCCATAGGTGATGGTGTTATAGAAGAAAAAATGGAGTCAAGGG
Ca_LG_3:27875788	GTCCACATATTTATTTATGAAAATCCCAAGACCTCTTATTAGGTAGAAGTCCAATCCTATAGCTTCCTCAATGCTAGGACGTTGCACCTTGACAGCCACG	G/A	AGTTTACCCGAATACTTCAACCGAGCTTTATAAACTTGACCTAAACTAGCTGCAGCCATAGGTGATGGTGTTATAGAAGAAAAAATGGAGTCAAGGGATA
Ca_LG_3:27875794	ATATTTATTTATGAAAATCCCAAGACCTCTTATTAGGTAGAAGTCCAATCCTATAGCTTCCTCAATGCTAGGACGTTGCACCTTGACAGCCACGAGTTTA	A/C	CCCGAATACTTCAACCGAGCTTTATAAACTTGACCTAAACTAGCTGCAGCCATAGGTGATGGTGTTATAGAAGAAAAAATGGAGTCAAGGGATAATCCTA
Ca_LG_3:27875797	TTTATTTATGAAAATCCCAAGACCTCTTATTAGGTAGAAGTCCAATCCTATAGCTTCCTCAATGCTAGGACGTTGCACCTTGACAGCCACGAGTTTACCC	C/A	GAATACTTCAACCGAGCTTTATAAACTTGACCTAAACTAGCTGCAGCCATAGGTGATGGTGTTATAGAAGAAAAAATGGAGTCAAGGGATAATCCTAATT

Positions	-100 bp flanking sequences	SNPs within *kabuli* cultivar	+100 bp flanking sequences
Ca_LG_3:27875800	ATTTATGAAAATCCCAAGACCTCTTATTAGGTAGAAGTCCAATCCTATAGCTTCCTCAATGCTAGGACGTTGCACCTTGACAGCCACGAGTTTACCCGAA	A/G	TACTTCAACCGAGCTTTATAAACTTGACCTAAACTAGCTGCAGCCATAGGTGATGGTGTTATAGAAGAAAAAATGGAGTCAAGGGATAATCCTAATTCCC
Ca_LG_3:27875802	TTATGAAAATCCCAAGACCTCTTATTAGGTAGAAGTCCAATCCTATAGCTTCCTCAATGCTAGGACGTTGCACCTTGACAGCCACGAGTTTACCCGAATA	A/T	CTTCAACCGAGCTTTATAAACTTGACCTAAACTAGCTGCAGCCATAGGTGATGGTGTTATAGAAGAAAAAATGGAGTCAAGGGATAATCCTAATTCCCTT
Ca_LG_3:27875824	TATTAGGTAGAAGTCCAATCCTATAGCTTCCTCAATGCTAGGACGTTGCACCTTGACAGCCACGAGTTTACCCGAATACTTCAACCGAGCTTTATAAACT	T/C	TGACCTAAACTAGCTGCAGCCATAGGTGATGGTGTTATAGAAGAAAAAATGGAGTCAAGGGATAATCCTAATTCCCTTTCAATGCATGCAAAGGCCTCCT
Ca_LG_3:28434842	CTGCTCTCATGGTTCTTCAAATACAAATTGAAGGAGAGAGCATTAACTTTCATTACTTTGATTCACAAGGTTCAATATTGTATCTTCCATCAGTTCTGCA	A/T	AGAATAGCGTCGCCCATTTCAAAACCAACTGTTTCAGCATCGAATCGAAGGTCCATCCATGTTCCATTTTTTGCCAAGTCTTTCTTAACAATTTGTTCCA
Ca_LG_3:28434848	TCATGGTTCTTCAAATACAAATTGAAGGAGAGAGCATTAACTTTCATTACTTTGATTCACAAGGTTCAATATTGTATCTTCCATCAGTTCTGCAAGAATA	A/G	GCGTCGCCCATTTCAAAACCAACTGTTTCAGCATCGAATCGAAGGTCCATCCATGTTCCATTTTTTGCCAAGTCTTTCTTAACAATTTGTTCCAAAGTAT
Ca_LG_3:28434849	CATGGTTCTTCAAATACAAATTGAAGGAGAGAGCATTAACTTTCATTACTTTGATTCACAAGGTTCAATATTGTATCTTCCATCAGTTCTGCAAGAATAG	G/A	CGTCGCCCATTTCAAAACCAACTGTTTCAGCATCGAATCGAAGGTCCATCCATGTTCCATTTTTTGCCAAGTCTTTCTTAACAATTTGTTCCAAAGTATG
Ca_LG_3:28434851	TGGTTCTTCAAATACAAATTGAAGGAGAGAGCATTAACTTTCATTACTTTGATTCACAAGGTTCAATATTGTATCTTCCATCAGTTCTGCAAGAATAGCG	G/A	TCGCCCATTTCAAAACCAACTGTTTCAGCATCGAATCGAAGGTCCATCCATGTTCCATTTTTTGCCAAGTCTTTCTTAACAATTTGTTCCAAAGTATGCG
Ca_LG_3:28434857	TTCAAATACAAATTGAAGGAGAGAGCATTAACTTTCATTACTTTGATTCACAAGGTTCAATATTGTATCTTCCATCAGTTCTGCAAGAATAGCGTCGCCC	C/T	ATTTCAAAACCAACTGTTTCAGCATCGAATCGAAGGTCCATCCATGTTCCATTTTTTGCCAAGTCTTTCTTAACAATTTGTTCCAAAGTATGCGGTGGAG
Ca_LG_3:28434878	AGAGCATTAACTTTCATTACTTTGATTCACAAGGTTCAATATTGTATCTTCCATCAGTTCTGCAAGAATAGCGTCGCCCATTTCAAAACCAACTGTTTCA	A/G	GCATCGAATCGAAGGTCCATCCATGTTCCATTTTTTGCCAAGTCTTTCTTAACAATTTGTTCCAAAGTATGCGGTGGAGGCAAAGGAAGGACATGCCAGT
Ca_LG_3:28434881	GCATTAACTTTCATTACTTTGATTCACAAGGTTCAATATTGTATCTTCCATCAGTTCTGCAAGAATAGCGTCGCCCATTTCAAAACCAACTGTTTCAGCA	A/T	TCGAATCGAAGGTCCATCCATGTTCCATTTTTTGCCAAGTCTTTCTTAACAATTTGTTCCAAAGTATGCGGTGGAGGCAAAGGAAGGACATGCCAGTGCA
Ca_LG_3:28966924	AACAATTTTTTTGAGGTACAATCTAGTACGAAAGGTGTTACACAATGAAAACTTGCTTATATAATGAAAATAAATCACCTCAGCAGCCTCTGAAGTTGAT	T/A	GAAAGATACTGATAATAATAATGCCGCAACGCATCAGGTACGGATGCAGGTGTGTTGTGCCAAATGTCCAAGTTCTGGTCAGTAACCTGCAGCAATAGTT
Ca_LG_3:28966954	AAAGGTGTTACACAATGAAAACTTGCTTATATAATGAAAATAAATCACCTCAGCAGCCTCTGAAGTTGATGAAAGATACTGATAATAATAATGCCGCAAC	C/G	GCATCAGGTACGGATGCAGGTGTGTTGTGCCAAATGTCCAAGTTCTGGTCAGTAACCTGCAGCAATAGTTATAGTATAGAATAAGAAATCACTAAAATTA

Positions	-100 bp flanking sequences	SNPs within *kabuli* cultivar	+100 bp flanking sequences
Ca_LG_3:28966964	CACAATGAAAACTTGCTTATATAATGAAAATAAATCACCTCAG CAGCCTCTGAAGTTGATGAAAGATACTGATAATAATAATGCCG CAACGCATCAGGTA	A/C	CGGATGCAGGTGTGTTGTGCCAAATGTCCAAGTTCTGGTCA GTAACCTGCAGCAATAGTTATAGTATAGAATAAGAAATCACT AAAATTAAGCACCAAAT
Ca_LG_3:28966966	CAATGAAAACTTGCTTATATAATGAAAATAAATCACCTCAGCA GCCTCTGAAGTTGATGAAAGATACTGATAATAATAATGCCGCA ACGCATCAGGTACG	G/A	GATGCAGGTGTGTTGTGCCAAATGTCCAAGTTCTGGTCAGT AACCTGCAGCAATAGTTATAGTATAGAATAAGAAATCACTAA AATTAAGCACCAAATAG
Ca_LG_3:28966967	AATGAAAACTTGCTTATATAATGAAAATAAATCACCTCAGCAG CCTCTGAAGTTGATGAAAGATACTGATAATAATAATGCCGCAA CGCATCAGGTACGG	G/A	ATGCAGGTGTGTTGTGCCAAATGTCCAAGTTCTGGTCAGTAA CCTGCAGCAATAGTTATAGTATAGAATAAGAAATCACTAAAA TTAAGCACCAAATAGG
Ca_LG_3:28966970	GAAAACTTGCTTATATAATGAAAATAAATCACCTCAGCAGCCT CTGAAGTTGATGAAAGATACTGATAATAATAATGCCGCAACGC ATCAGGTACGGATG	G/T	CAGGTGTGTTGTGCCAAATGTCCAAGTTCTGGTCAGTAACCT GCAGCAATAGTTATAGTATAGAATAAGAAATCACTAAAATTAA GCACCAAATAGGAAG
Ca_LG_3:28966973	AACTTGCTTATATAATGAAAATAAATCACCTCAGCAGCCTCTG AAGTTGATGAAAGATACTGATAATAATAATGCCGCAACGCATC AGGTACGGATGCAG	G/A	GTGTGTTGTGCCAAATGTCCAAGTTCTGGTCAGTAACCTGCA GCAATAGTTATAGTATAGAATAAGAAATCACTAAAATTAAGCA CCAAATAGGAAGGGC
Ca_LG_3:29822260	TATTTTCATATTGTTACTACATATAATTGTCTTGTTACCGTAAG ATTTTGAACGAAGTCTCTAGATTGATTAAGAAATAATTTTCTTC GCCATTAAGCAA	A/G	CATCCTTTAAGTGAGTAGGGGTGCGTCGGACTTCCGACGAA TAATAGATACCCCCACACCCCAGGCAGCATTAGTGAGCCGT GAAATAGACCATTCTTCA
Ca_LG_3:29822286	TTGTCTTGTTACCGTAAGATTTTGAACGAAGTCTCTAGATTGA TTAAGAAATAATTTTCTTCGCCATTAAGCAACATCCTTTAAGTG AGTAGGGGTGCGT	T/C	CGGACTTCCGACGAATAATAGATACCCCCACACCCCAGGCA GCATTAGTGAGCCGTGAAATAGACCATTCTTCACCTAAATGA CTTAATTCACTATCAAT
Ca_LG_3:29822292	TGTTACCGTAAGATTTTGAACGAAGTCTCTAGATTGATTAAGA AATAATTTTCTTCGCCATTAAGCAACATCCTTTAAGTGAGTAG GGGTGCGTCGGACT	T/C	TCCGACGAATAATAGATACCCCCACACCCCAGGCAGCATTA GTGAGCCGTGAAATAGACCATTCTTCACCTAAATGACTTAAT TCACTATCAATGAATGA
Ca_LG_3:29822293	GTTACCGTAAGATTTTGAACGAAGTCTCTAGATTGATTAAGAA ATAATTTTCTTCGCCATTAAGCAACATCCTTTAAGTGAGTAGG GGTGCGTCGGACTT	T/G	CCGACGAATAATAGATACCCCCACACCCCAGGCAGCATTAG TGAGCCGTGAAATAGACCATTCTTCACCTAAATGACTTAATT CACTATCAATGAATGAC
Ca_LG_3:29822308	TGAACGAAGTCTCTAGATTGATTAAGAAATAATTTTCTTCGCC ATTAAGCAACATCCTTTAAGTGAGTAGGGGTGCGTCGGACTT CCGACGAATAATAGA	A/G	TACCCCCACACCCCAGGCAGCATTAGTGAGCCGTGAAATAG ACCATTCTTCACCTAAATGACTTAATTCACTATCAATGAATGA CATACACATAAGGAAT
Ca_LG_3:29822329	TTAAGAAATAATTTTCTTCGCCATTAAGCAACATCCTTTAAGTG AGTAGGGGTGCGTCGGACTTCCGACGAATAATAGATACCCCC ACACCCCAGGCAGC	C/A	ATTAGTGAGCCGTGAAATAGACCATTCTTCACCTAAATGACT TAATTCACTATCAATGAATGACATACACATAAGGAATACAAG GTTGGTTTGGTGGTTG
Ca_LG_3:29822330	TAAGAAATAATTTTCTTCGCCATTAAGCAACATCCTTTAAGTGA GTAGGGGTGCGTCGGACTTCCGACGAATAATAGATACCCCCA CACCCCAGGCAGCA	A/G	TTAGTGAGCCGTGAAATAGACCATTCTTCACCTAAATGACTT AATTCACTATCAATGAATGACATACACATAAGGAATACAAGG TTGGTTTGGTGGTTGA

Positions	-100 bp flanking sequences	SNPs within *kabuli* cultivar	+100 bp flanking sequences
Ca_LG_3:29822344	CTTCGCCATTAAGCAACATCCTTTAAGTGAGTAGGGGTGCGTCGGACTTCCGACGAATAATAGATACCCCCACACCCCAGGCAGCATTAGTGAGCCGTGA	A/T	AATAGACCATTCTTCACCTAAATGACTTAATTCACTATCAATGAATGACATACACATAAGGAATACAAGGTTGGTTTGGTGGTTGATAAGCAACAAACTT
Ca_LG_3:32243548	TTGTGAGGGCCAAATCCGTCACAAAGGCAGACGGGACTGGCATTTGTGGCTGCAAAGGCCGCCACAAAGGAATGCGCACGTGGCATTTGTGGCTGCAAAG	G/T	GCCGCCACAAAGGAGACCCAATACAGCTGGCGTTTGTGGCCGCTTAGGCCGTCACAAACGTGATGTAACCGTTGGCATTGTGGCTGCTTTGGCCGCCACA
Ca_LG_3:32243564	CGTCACAAAGGCAGACGGGACTGGCATTTGTGGCTGCAAAGGCCGCCACAAAGGAATGCGCACGTGGCATTTGTGGCTGCAAAGGCCGCCACAAAGGAGA	A/G	CCCAATACAGCTGGCGTTTGTGGCCGCTTAGGCCGTCACAAACGTGATGTAACCGTTGGCATTGTGGCTGCTTTGGCCGCCACAAATGCTCAACGTGACC
Ca_LG_3:32243580	GGGACTGGCATTTGTGGCTGCAAAGGCCGCCACAAAGGAATGCGCACGTGGCATTTGTGGCTGCAAAGGCCGCCACAAAGGAGACCCAATACAGCTGGCG	G/A	TTTGTGGCCGCTTAGGCCGTCACAAACGTGATGTAACCGTTGGCATTGTGGCTGCTTTGGCCGCCACAAATGCTCAACGTGACCCTATATATATTTTTCT
Ca_LG_3:32243619	ATGCGCACGTGGCATTTGTGGCTGCAAAGGCCGCCACAAAGGAGACCCAATACAGCTGGCGTTTGTGGCCGCTTAGGCCGTCACAAACGTGATGTAACCG	G/A	TTGGCATTGTGGCTGCTTTGGCCGCCACAAATGCTCAACGTGACCCTATATATATTTTTCTCCCCTCCTCCTTCATTTTTTCACATCCAATTTCTCTCTA
Ca_LG_3:33034813	ACGATGTGTGATACAATAGGCTTTCACTTAAGATTTTATTTTGGCACTCACCCTATAAATTTGTTAGTATTTGGTTTGGTTTTACCCAGTGTTTTTTTCG	G/A	TATCGCTGCAGCGTTCAGCAATAATGATTATAGACGCCATATATATAAACAGCTAAAGCTTAAAAAGTGAGTACTGTGGTATATATAAAAATTATTATTA
Ca_LG_3:33066259	AGTAGATTTAAATGACAGAACCAACTGATGTGATAGACAAATGCTGCCCATGTCAATAAGAACTTGATGAGACTCAGATCTCATCAGACACATTGCTTCG	G/T	GTCATATTTGCTGCATCTGGAGTTACATGGTACCAAAATTGATTGTGGAACTGTGTTTATTCTCCCCTTGTGAGTAAGGCATGATGCTGGGTATTTTGGG
Ca_LG_3:33209811	TTGTTTGCGATTTTTATCAAATTGAAACCCTTAGATTAGGCTTTTTTTAGCTGCCAATTTTGATTTATGTATTAAGATGCTGACGATTTTATTTCAGAGG	G/T	GATTTTGTTCATTTGATGCTCCGAAAGTGTTGATATTGGTTTTTGAATGGTTTACAATTGCAGCTTGTGCGCAGCAACAGTAAGAAGTTTACTCCGTCGC
Ca_LG_3:33213264	AAAATTGGATGTGGTCACAGTAAAAAAGTGCATTACAAACCCTAACCATTTAGTAGCAGCTAAAGGGCTATTTTTCCGTGATTTGTGTCCGCTACTGCAA	A/G	AACACAGATAGCCAGTGTTTAGCGTCGCACCATGGGCCGTTCCCGCTTCGCTATTCCTCTATAGCTGCTATTTGCAACTTAGGCATATATAATCCATCAG
Ca_LG_3:33213303	CCCTAACCATTTAGTAGCAGCTAAAGGGCTATTTTTCCGTGATTTGTGTCCGCTACTGCAAAACACAGATAGCCAGTGTTTAGCGTCGCACCATGGGCCG	G/A	TTCCCGCTTCGCTATTCCTCTATAGCTGCTATTTGCAACTTAGGCATATATAATCCATCAGTTTGGGTGGTCGTAGTTTGTTTATTCATAATAAGATTGT
Ca_LG_3:33252426	ATAAGAAGAAGGTGCAGTGAGTAGACTTGGATAAGGAGCTGCAGCATACGGTGCAGCAGTTTGGGGGTACGTATAACCGACTGCGGTGCCAGAGTTTACA	A/G	CGAATTTTTCCAGGTTGCATGTATCCTGGGGTTATTGGTAAGGCTGCCATTGGAACTACTCCTGCAGGAAAAGGCATTTGACCAGATTTACCCTTGTGGG
Ca_LG_3:33255448	CCAATACTGTGCAGACTGCAATACACAGCAGCAATCACATAATCTGACACTCCATCTGCCTAAACAACTTTGCATCCTTCCTCATATGCCAAAGCCAGTG	G/A	CCAACCTCACTGGACCCGTGCGGCGTCAAACACCACCATGCACTGGCTGTTCTCTGTAACCATCAGAAGCCATTTGCTGCACCGCGTTATCACTATTGTT

Positions	-100 bp flanking sequences	SNPs within *kabuli* cultivar	+100 bp flanking sequences
Ca_LG_3:33631943	GCCAAGCTTGATGAGAAGAAAAATATTAGGGATTTGATTTCTAGTGCTGCTGGCGGTGCAGCTCCAGCCGCTGCCGCAGCCTCAGCCGCTGCCGCAGCCC	C/T	CAGCCGCTGCCGCCAGCCGCTGCCGCTGCTCCAGCAGCTGAGGAGAAGAAGAAGGTAATCCCTTAGTGAAATTTATTTTCCTTTTCTTTTCAACTTTGAC
Ca_LG_3:33847655	TCCTTGGATTGGTATGACTGGCTTTTGTATCTACCTTTGGCAGCGTTCGCTTGCTACAGACCTTCAGAATCTGTCAATGGACCTCCGGAGAAAGCAGTCA	A/T	GCATATTTAAAACGTTTGCAGCAGCAACGAGAGGTTGCAACTTTTCATGTCTAGCCAATTTTTCAATTCCAAATTCTTTCATATTATCCTCCGACATATT
Ca_LG_3:34134919	CACTATTCGTTGGTTTGAACAAAGGATAGGCTAGGCCTCAAACATGATTTTAAGATCCTAGCTACGCCATACGCGTTCCATACATAATCTGGAAAGCTGT	T/C	AGGGATGACAAAAAGAGGGCGCTTTCATGTGTTGCACAGAACACAGATAAAAGAAAGAAACCTTACTAAATAATAGAGAAGATGGATCATATCTCCTTTA
Ca_LG_3:34134946	AGGCTAGGCCTCAAACATGATTTTAAGATCCTAGCTACGCCATACGCGTTCCATACATAATCTGGAAAGCTGTAGGGATGACAAAAAGAGGGCGCTTTCA	A/C	TGTGTTGCACAGAACACAGATAAAAGAAAGAAACCTTACTAAATAATAGAGAAGATGGATCATATCTCCTTTATCGAACACTAAATGGTAAATCCTACCT
Ca_LG_3:34647021	GAGCTTGGCTCGACAAGCTTTGTGGGCAAGTGGTGCGGCAGCCTGTGATCCCTCCACTGTTAGGAGCCGAAGGTTTTCGCTGCTCTGTGAGTCCAGCCTT	T/C	ACGCTATACCCTCCAGTCTAGGAGCCAAGAGACCCGAGCGTTCGCCCAAGTCCCGTGACTCAACTCTCAAATTGGAGCGGGCAGGCTTGCACAGTGCAGC
Ca_LG_3:34647061	GCCTGTGATCCCTCCACTGTTAGGAGCCGAAGGTTTTCGCTGCTCTGTGAGTCCAGCCTTACGCTATACCCTCCAGTCTAGGAGCCAAGAGACCCGAGCG	G/C	TTCGCCCAAGTCCCGTGACTCAACTCTCAAATTGGAGCGGGCAGGCTTGCACAGTGCAGCCACCAACCCAACCAAACAAGCCAAAGCTATGTTCCATCAC
Ca_LG_3:34647092	GGTTTTCGCTGCTCTGTGAGTCCAGCCTTACGCTATACCCTCCAGTCTAGGAGCCAAGAGACCCGAGCGTTCGCCCAAGTCCCGTGACTCAACTCTCAAA	A/C	TTGGAGCGGGCAGGCTTGCACAGTGCAGCCACCAACCCAACCAAACAAGCCAAAGCTATGTTCCATCACCATTTTGCCCCCGCTTGGGTCACAAGTTGGA
Ca_LG_3:34904395	CACCGGTTGTACTCACCCTGATCTGCAAGAGGATTTGATCACGACTGTTTTGAATCTCTCAATTCACGACGATAATAAGAAAGTGTTTGCAGAGGATCCG	G/A	GCCCTCATTTCCCTTCTCATTGATGCCTTGAAATGTGGAACCATCCAAACAAGAAGCAATGCTGCAGCAGCTATTTTCACACTATCAGCTCTTGATTCCA
Ca_LG_3:35384180	ATCATATCCTATCTCTTCCTCTCTCACCTGTGCAGCAACTCTCTATTCCTCTCTCACCTGCGCACGGTGTCGCCCTCTTGCGCCGCTGATGCCTCGCCTT	T/C	GCTTCAGCTCGCCTCAGTCGTTCGTCGTCAAAACTCCAAAGGTTTGGAATTTGCTGATTTGTTGAACTAGGTGTTTGAACAAGATGTTTGTGTTGATTTT
Ca_LG_3:35384196	TCCTCTCTCACCTGTGCAGCAACTCTCTATTCCTCTCTCACCTGCGCACGGTGTCGCCCTCTTGCGCCGCTGATGCCTCGCCTTGCTTCAGCTCGCCTCA	A/G	GTCGTTCGTCGTCAAAACTCCAAAGGTTTGGAATTTGCTGATTTGTTGAACTAGGTGTTTGAACAAGATGTTTGTGTTGATTTTTGTTGCATACAAGGTG
Ca_LG_3:35543341	CAACGTTAGTTGCAGCGCAGTGTGTGGAGGCTGCTGAAGCAATGGGAGCGGAGCGCGACCACCTTGCTTCTGTGGTCAGCTCAGCTGTGAATGTCCGTTT	T/C	TCACGATGATATCACTACTCTTACTGCTGCAGCTGCCACAGGTATCACTTTTTGCATTTTATATCACGGTGGCATTATTATTTTTTATATAATAGAGAAT
Ca_LG_3:35916630	TGTGATGGTAGGAGCACTCCCAGCTGCATTGACTTACTACTGGCGGATGAAGATGCCCGAAACTGCTCGTTACACCGCTCTAGTTGCTAAGAACACGGCG	G/A	CAGGCTGCAGCAGATATGTCTAAGGTTCTGCAAGTCGAGATCCAAGCTGAACCAAAGACAAATGAGCAGAAAAAGTCTTATGGTTTATTCTCAAAGGAAT

Positions	-100 bp flanking sequences	SNPs within *kabuli* cultivar	+100 bp flanking sequences
Ca_LG_3:35929901	GATTTAGGCATGGGCTTGGAAGTGCTTTCTCCGTTATGCCCACATCTTTTTCTTGAAATGGCAGGCCTAGGAAATTTTGCAAAGGTTTATATTTTTATCA	A/G	AATTCTCTTAAAAGTAATTTCCTCTTATGGCATCTGCTGCATTTTAAAGTTTGATTCAGTGGTAATTGTTTATTAAAACCTATGTACTTCATGATTAAGA
Ca_LG_3:35929902	ATTTAGGCATGGGCTTGGAAGTGCTTTCTCCGTTATGCCCACATCTTTTTCTTGAAATGGCAGGCCTAGGAAATTTTGCAAAGGTTTATATTTTTATCAA	A/G	ATTCTCTTAAAAGTAATTTCCTCTTATGGCATCTGCTGCATTTTAAAGTTTGATTCAGTGGTAATTGTTTATTAAAACCTATGTACTTCATGATTAAGAA
Ca_LG_3:35929912	GGGCTTGGAAGTGCTTTCTCCGTTATGCCCACATCTTTTTCTTGAAATGGCAGGCCTAGGAAATTTTGCAAAGGTTTATATTTTTATCAAATTCTCTTAA	A/T	AAGTAATTTCCTCTTATGGCATCTGCTGCATTTTAAAGTTTGATTCAGTGGTAATTGTTTATTAAAACCTATGTACTTCATGATTAAGAACTTTAGCAAG
Ca_LG_3:35969418	TTGGTTTCAGCATGACCGTCCCCCTTAGTTGCATTGTCATCCACTTCAAGAGTGTTTGACTTTTTGGGAAGTTTTCGATCAAGACTCTGAGCAGCTTCCG	G/A	ACTCTTCAGATTCATCACCTTCACATCTTTCATTGGGGTTTTGAACAGCTTCGGCATCATGCTTGGCAGTGTCAGCATTTTGTGTTAGTTGTGCCCCTGA
Ca_LG_3:35969543	TCTTTCATTGGGGTTTTGAACAGCTTCGGCATCATGCTTGGCAGTGTCAGCATTTTGTGTTAGTTGTGCCCCTGAACTTTGAGAAGCTTCCAACTCTTCG	G/A	TACTTAGAAACCAACTGCTGCAGCTCATCATTGAGATATAAAGCCTCAAAAAGTGTCTCTTCATCATTTGTGGTGCTTTCCACAATCTCCTTGATAACAG
Ca_LG_3:35989008	TGGAAGTTCTTCATCATCAGCTGTTTCAACCATCACCACCAACACACCAAACAGTAGCAGCCGCTATGAGAATCAGAAACGCCGTGATTGGAACACTTTT	T/C	GGTCAGTATCTTAAGAATCATCGACCTCCTCTTTCGCTCTCGCGGTGCAGCGGTGCACATGTTCTTGAATTTCTCAGGTAAATATGTAAATATAATCAAC
Ca_LG_3:36173029	GAGTCAACAAACACATTCCCTGTGGTCATAGAAATCATGGGTTGTTCGTTTATAATCACATGAGATAGGTAACAGACTAAATCACAATTTTGTAAAGCCC	C/T	GAATGCAGCAATTAATAATATAGAGAAGAAAAAAATGTGTTGGAAATCCATGTGTGAGATAAAATCTGACAGTGGGAAGAAATTAGCAAATTCAAGACAG
Ca_LG_3:36299589	GATAATATTTAAATTAATTAGGACAAAAAAATAGGCTATCTCTATGTGTGTTTGGGATAGCAGCTGTCTCATTATGAAATCAATGTGTTTTCTTGTGAAG	G/C	ATTATCCTGTATGTTTAGAGATGCTACTTGCCTCCCCAAGGCTGCTTTTATCTGTGCTGTTGAATATTTCTGAAACTTTGCTACCTAGACTTCTTTTCCT
Ca_LG_3:36299605	ATTAGGACAAAAAAATAGGCTATCTCTATGTGTGTTTGGGATAGCAGCTGTCTCATTATGAAATCAATGTGTTTTCTTGTGAAGATTATCCTGTATGTTT	T/A	AGAGATGCTACTTGCCTCCCCAAGGCTGCTTTTATCTGTGCTGTTGAATATTTCTGAAACTTTGCTACCTAGACTTCTTTTCCTACTCGTGTATGATTTT
Ca_LG_3:36501498	ATTTGCAATTCCAAATGGTGATTTCAATGTCCGCTAGGCCACTCAGAAATACAGATAACAGCTGCCATACCATCAAATTGCGTGCAAAGGGAGAGAACCG	G/A	TGGTCTGCAGAAAGGCAGCCATTAATGTCCCCAATCTGTACATAATTAAGTCGAGCCACTTTCATCCTAATTGTGCTTTAACTTTTATGTTTTATTCAAA
Ca_LG_3:36595777	ATCCCCATTCGGGTCTCTTCACTAGCCAATCAACAAGATCAAGTGAAGCAGCATCTCTCATTGAAATATTGTAACCCCAAAGATAACGAGCAATGTCAAA	A/G	TCCAGCATAAAAGGGTACAAAAATAGCATTAGCAATGGAAGAATCATGAGTCAAGCATTCATACTGTTTCATCCTGTTGGCGAAGATGACATCAACGGCA
Ca_LG_3:38115345	AAGAACTTATGTGACTATATCTAACATAACTAACCTCTTAACACCATTAATCCTAGCAGCCTCAATCATGTTGAAGCTAATCATTGTGTTGTTATACATT	T/A	ATAACCGAGTGGTTGGATTGGATAAAACCCATTCCACCCATATCAGCAGCAAGATTGAAAACATGATCCACATCCTTGGTAACTTTGAGACAGTTATCCA

Positions	-100 bp flanking sequences	SNPs within *kabuli* cultivar	+100 bp flanking sequences
Ca_LG_3:38451306	TAGATAGGCCAGTGTGGCTTTTTTTTTATAATAGTAGCTTTCGTTCTGTTGTTCTTTTATTGTTATTCATTTTTGTTTGTAAATGCAGCACTGTTTTTCA	A/G	AACTGAGCTTGATTTTCTTTCAGTTTGGCAGCACAATGTGCCTCCCTTTGGTTGGATACTTCCTCTGTTAATGCTGCTATTGTAAATGCCTATAAAGCTG
Ca_LG_3:38451394	CACTGTTTTTCAAACTGAGCTTGATTTTCTTTCAGTTTGGCAGCACAATGTGCCTCCCTTTGGTTGGATACTTCCTCTGTTAATGCTGCTATTGTAAATG	G/A	CCTATAAAGCTGCCTGCGACAGATATTACCAGAAATACGAGAGTAAACACAAAACTAGGAACAAAGGTTTTCATGGATCAATTGAGAACTTGGTTGTACC
Ca_LG_3:38452060	TTTTTTTACCCCCTGTTGACTACTCAATAAATATTTGATAAGAATGTTGAATTTATGTAGAAGAGTCCACATAGAAGCTTGCTTTCAGCATATATATAAC	C/T	TGTGCTATCTTATTGTTTGCAGGGATGCTGCTGCCGTTGCTCAGTTCTGGGATTGGCTAGAGACGGAAATTACTAACAAAAGGATATTAACCGAAGTAGA
Ca_LG_3:38467528	TTCTGTACTTGTAGTCATTGAGGTCATTCTGACAACGTTTTCCTTTCCTTGTAAGGAAGAGCTGCATAAGGTCCTTCTATTGGATGGTGGAGGCAAGGAG	G/A	TTATTAATTCTTCTCAGTGGGGGTCTTCCCAAAGGAACCAGTGGTGTCTTCTTAATGGTTTTCTCGGTTGCCGCATGTGTTGATGATGACACTGTTGAAG
Ca_LG_3:39509275	ACAAATGAATCAAATTCATTCCCTCGTAATCACCAATGGCTACCTCCTTCACCAACCGAATACTTCAAACCATAAAAAATGGATGCCTGACACTTCTCTC	C/A	CACTGCCCCCATTTTGCAGCCCATGGACGAAAGATTCAAGAGGGTGTGATAAAAGATATAGCGTGGACAGACTATAATAGAATCCTGGTCCTACTTGATC
Ca_LG_3:39636362	ACAAGAGCCTACAGCTCCAATTATACATCAACTAGATTATTATTATTGTTATTATTATCAGCTTATCAAGACAATTAACTATATTAACCGAATAATACAA	A/G	TGTCTTAAACTTGGAGAACAAAATATAGTACCTTGAAATTTGGATATAGCAGCAGAGGGCAAGAGGCATATCTCGTTAAAGGAATCATATCCAACCAAAA
Ca_LG_3:39665887	CTAATCAGTATTGTCTGTTGTGAATGCTTGCCTGCCTTGCAGTGTGCTGTCGGATATTGAATAGAAACTTGAGATTACGGTCGTTAGGTTCCTGGTCTCC	C/G	GAGCAATATAACGTTATGTCTTGGTCAGCAGCTGGGCAGTCAGCATAACGTTTGGGCGACATAACGTTTGGGGACAGGTCTTTTCTGGAACCAAGTCTTC
Ca_LG_3:39713198	AATACAGAAATTAATTTTTTGTATTTATATTACAATTAAGTTGGACATGTGGCTAAAGATCATCTGTTTATTGCTGCTAGAGATACCCTCATAATAATTC	C/T	ATAAAAGACAAAAGACAACACAACTTTATCTATCATGTATATATAAGAAAACATCCAGAACAACAATTGCCTTTTTAAGTGCTTCAAGAAAAAATGGCTGC
Ca_LG_3:39727327	AGTATTTAGTATGTTATTGCGCCAATTGTTTAGAAAATTTCCAAGCCACGGCCGCCGTCAGCGAGCAAATATTCGTCACATCTTGGAGCAGCCTCTTAGT	T/C	CGCATCGTTCCTGCATCTTCTTCGTCACAAACTTTCTGTGGGGTACCAGCTGCTCCAAGATGTACTAGAGATGAGTACCCAGCGCAATAAAAAAGGTATG
Ca_LG_3:39801715	CTATGGAGATTGCAACAATTACTGATATCAGCTTAGGTACAAAATTTGATACAACCACGACACTGATGAAAACAGTAACTCAATGTCGGCAACAGACTTG	G/C	TGATGAATAACATCTGCGTGGAAACAGTATCATCTTGATAAAATTCAAGGGATGTTGACGTTCAGCAGTCAGGAATTGTTGCCAGCTGCATTTTTAGTTG
Ca_LG_3:39823622	TTTGTTTTTTTTGTGATTATAATAGAGATCCGTGAGAACCAAAGAAAGAGGATCAAGCGTGCGTTGTTGCAGCCATGGTGAAATCCCTCTCTCTACTCTA	A/T	ACTTTTTTGATTTTGAAAATGTGAAATTTTGCATTGCTGAATGGTTAATTTGTTTTATATTTCAGGCGGATGTAGACCCAGAAGTTGCAGCACAAGGAGT
Ca_LG_3:39837598	CTTTCTTCTTTTTCATTTTTATTTTTATTTTTATTTTTCCGATGCGATTCCAGCGCGATTTGCTGCTGCAAAAGAGAATTTCGTCGCAGCAGAGCAAAGT	T/C	TCCACATCGCACTGTGATTCTGCGATTGCGCGCTATAGCTGCGCCATTGAAAACATAGCTTTTCTGGCTATTTATAGTAATTGATTTTCCCATTACATGT

Positions	-100 bp flanking sequences	SNPs within *kabuli* cultivar	+100 bp flanking sequences
Ca_LG_3:39842881	AGTTGAAAAACATTTAGACACCTGCTGCATAAATCTGTTATTTCTGTAATTTGAATGTAGATATAACCTATTCAACATCTACACTTTGAATTGAAGGCAC	C/T	GTTCAACACGTGGCAGCGCTTGACATTGTCTCCTAGGACAATGTCGCATGTAATTACACTCAATCACTTTTAGTGTTTAATTTAATTTTTTTACTGATGT
Ca_LG_3:39887505	TCAATTATCGAGACTCGGCCCAGGTTCTGCTACCTCCTCGAGGCTGGCTGCAATTAAGGGACTCTCTTTAGATGATGGAGCCAATAATGAAAAGTGCACG	G/A	GCAATCACTAATGCCGAGGCATTAAATCCAAAGTACGAGTTTCCGAACATGAAAACAGCTGCTATCATATCAGACCAGAAGACACTGAAGGTTCGAATCA
Ca_LG_3:39940995	TTTGCTGCGGATTTTCCTGCGGAGTTTGCATGGAATTTGCTGCGGAAATTTGCCGCAGATAAATCCTATGGAAAAAGATGAACTTTGCTGCGGAAATATA	A/G	GTCGTATGTAAATCCGCAGCAAAAGAAAACGTTACCTGCCGTTTTACCTGCGGATAGTGATACCGTAGGAAAAGTCTTAAGATAATTGTGACCCGCAGGA
Ca_LG_3:39941002	CGGATTTTCCTGCGGAGTTTGCATGGAATTTGCTGCGGAAATTTGCCGCAGATAAATCCTATGGAAAAAGATGAACTTTGCTGCGGAAATATAGTCGTAT	T/C	GTAAATCCGCAGCAAAAGAAAACGTTACCTGCCGTTTTACCTGCGGATAGTGATACCGTAGGAAAAGTCTTAAGATAATTGTGACCCGCAGGAAAATCCT
Ca_LG_3:40487434	AAAAGTAGAAAAGCAAATAGGATAAATCAACACAACGTCAAAATAATCCATGTTATAGTGAAACAAGAGGAGCTCAGCACTGGATACATCAATTTTCAAG	G/A	CATATAAAATGGAGAAGCCCAAAGCAAATAAGTCTCGGTAGCCTTTTCTTATTTTAGGAAACATGACAAACAGAATTCTACAACTTACATTTGCTTCAGC
Ca_LG_3:40927037	ACAGTAAAGCTAACCTGTGACAAGTGAGAGCATGCAGCCTCAACTGCATTGGCAGACCATGACTTCCCAAGGGATTTTCTGACAATAAACCTCAAAGTCT	T/A	TGACTGGTAACAATCCTAACGATGCTATTAAGTAGCTAGCAGCAGCAGCTAGAATTTGTGATCTGCAATCGCATTAACCATTAGTCATTTAGAATAACAT
Ca_LG_3:41410475	GCACCATATCTAACACTAAATTTGAGCTGCCATTAGCTTAGAGCACCCAGGTTTAATGGAGTCACACGTGGTCTGTGCGATAGATGGCTACTTTTCCAAA	A/G	TTTCTTGAGCAGCCAACAAGTTTTGTAACTGGCTCAAACCTTTTTATTATTTATTTATTTTTTTTTTGATGTTGGCAAAGTAGAATNNNNNNNNNNNNNN
Ca_LG_3:41410495	TTTGAGCTGCCATTAGCTTAGAGCACCCAGGTTTAATGGAGTCACACGTGGTCTGTGCGATAGATGGCTACTTTTCCAAATTTCTTGAGCAGCCAACAAG	G/C	TTTTGTAACTGGCTCAAACCTTTTTATTATTTATTTATTTTTTTTTTGATGTTGGCAAAGTAGAATNNNNNNNNNNNNNNNNNNNNNNNNNNNNNNNNNN
Ca_LG_3:41426169	CGTAGAGTAACTCCCGATATAGCAGTAAAAGCAAGACGCGTAAGCGGATCTACTCATCAAGTTCCGACTGAAATAGGATCCACACAAGGAAAAGCACTTG	G/T	CTATTCGTTGGTTATTAGGGGCAGCCCGAAAACGTCCGGGTCGAAATATGGCTTTCAAATTAAGTTCTGAATTAGTGGATGCTGCCAAAGGAAGTGGCGA
Ca_LG_3:41429764	GATTGACGACTTACCAATCCAATGACGTTCGCACACAACATTTCAAAAAAGGGGTACTCTATTCTCGTAATTCCATAATAGATGCCTGTTGGCATGCCAA	A/G	CCTTCCTTCTCCTTTCATGACCTATCCGAAAACAAAGAAAATTCTTTACTTAATTAGTGATGAATATCGGAATAGGACAAACCATCCCGTGGATCTCTTT
Ca_LG_3:41429771	GACTTACCAATCCAATGACGTTCGCACACAACATTTCAAAAAAGGGGTACTCTATTCTCGTAATTCCATAATAGATGCCTGTTGGCATGCCAACCTTCCT	T/C	TCTCCTTTCATGACCTATCCGAAAACAAAGAAAATTCTTTACTTAATTAGTGATGAATATCGGAATAGGACAAACCATCCCGTGGATCTCTTTGTTTGCT
Ca_LG_3:41429774	TTACCAATCCAATGACGTTCGCACACAACATTTCAAAAAAGGGGTACTCTATTCTCGTAATTCCATAATAGATGCCTGTTGGCATGCCAACCTTCCTTCT	T/C	CCTTTCATGACCTATCCGAAAACAAAGAAAATTCTTTACTTAATTAGTGATGAATATCGGAATAGGACAAACCATCCCGTGGATCTCTTTGTTTGCTTCA

Positions	-100 bp flanking sequences	SNPs within *kabuli* cultivar	+100 bp flanking sequences
Ca_LG_3:41755896	TAGTTTACAATTCGTTGGGCACTAAGCAATATATTGAGAGCAGTGGTTTCAAGAGAGATGTGCCAAATTTGGAAGAAGTTGTTATTCAAGAAGGTGTTGT	T/C	TCACTTCAACAACCAAGAAGCTGCTGAGGATATCACCAATCACATTTATGAATTTATCAAGAAGTTCTGATTTTGTCACTTGTTTTAGTTAAAAATCATC
Ca_LG_3:41755903	CAATTCGTTGGGCACTAAGCAATATATTGAGAGCAGTGGTTTCAAGAGAGATGTGCCAAATTTGGAAGAAGTTGTTATTCAAGAAGGTGTTGTTCACTTC	C/T	AACAACCAAGAAGCTGCTGAGGATATCACCAATCACATTTATGAATTTATCAAGAAGTTCTGATTTTGTCACTTGTTTTAGTTAAAAATCATCTTCTAAT
Ca_LG_3:41925198	AATGAATGAAAAACATGTTAGTGGTCACGAAACAGATAAGGCAAGCCACGAAAATTTCTTCCCTGACACAGTTCGCTTCCCACCCCACCCATACCATACT	T/C	ATCATTCCGTGCAAGAAATTCAAAAGCATTACATGAATTTGTGATAATAAAATTAAAATATATTATCGAATAAGTTGAGTTGAGTGATTCAGCATGGCCA
Ca_LG_4:94968	TTCTATTAGCAGCAGCAGCAAGCACATCAATGTGCATATTATCAGCAAAAAGGATTGACGATGGATGTGATGAGTGTTGACTATCAGCATACCTGACCCC	C/T	AACTAGCAATTGTGATTTCTCATCCCTTGTAGCAATTGACAATTCATTTTTTTAGTACAAAGAAAGTTGCAAAAGTCTTAACCTAATGGAATGATTTATC
Ca_LG_4:197030	AACAGAGTAACCGATGGTTGAGGTAAACCAGAAGCTAGTAATCTCGCTCAAACAACTGAACAGACTCCAAGTCCTCTCCTTTCACTCAATTCACTGACAG	G/C	AATGTTGTGGTGGTGGTGATGATGATGATGATCATAATAATGATGATGATACATCTAAATGCAGCGGCATCATAAGAAAAAATGTAAGGGAAAATCACCC
Ca_LG_4:197033	AGAGTAACCGATGGTTGAGGTAAACCAGAAGCTAGTAATCTCGCTCAAACAACTGAACAGACTCCAAGTCCTCTCCTTTCACTCAATTCACTGACAGAAT	T/C	GTTGTGGTGGTGGTGATGATGATGATGATCATAATAATGATGATGATACATCTAAATGCAGCGGCATCATAAGAAAAAATGTAAGGGAAAATCACCCTTG
Ca_LG_4:197244	CAAAAATTCTGGAAGGAAGAATATCGAAAAGAAGATCATAAGAAAAAAAACCAACAAAAACATTAAATGTTGAATTATAGAGGTAAATTTTCCAGTAAGT	T/C	CATTCAAGCTGATAATGATGACAAATCATTTCAAAGCTACTGAGATGCTTGAGTTCTTCAGTGCTTTAATTTTAAACTTTCTCTATTGATGAAAGCTTAC
Ca_LG_4:197270	AAAAGAAGATCATAAGAAAAAAAACCAACAAAAACATTAAATGTTGAATTATAGAGGTAAATTTTCCAGTAAGTCATTCAAGCTGATAATGATGACAAAT	T/C	CATTTCAAAGCTACTGAGATGCTTGAGTTCTTCAGTGCTTTAATTTTAAACTTTCTCTATTGATGAAAGCTTACATGCCAAAATTTTGCAAACATTCAAA
Ca_LG_4:284435	TCCGAAGCGACAAAAAGCTATTGCAGACAAATGCACCTCATATTTTTGATGTTGTAGTTGCAGCTGATGGTACTGGAGATTTTACTACAGTGATGGAGGT	T/C	GGTGCATGCAGCCCCAAGCTCTAGTGAGAAGCGTTATGTCATATTTATAAAGAGTAAAGTTTATATGGAGAATTTTGAGGTTGACAATGATTGGAGAGGG
Ca_LG_4:476652	CAGGGATGTTTGGATAGATGTCAAGGTAGCTTCCATCCTTGCATTTGACTCTAATATTTTGGCGTTGTGTTCCATTTGAGCTGCCATAAATTCCATCAGC	C/T	TTTGAAACATATGCAGGAGCCTCAGCTGCTGATTCAGATGCTTGTGGAGGAGTTGGTTCAACATTTGGCATTGGTCTAATCCAAACATTATCTACTTTCT
Ca_LG_4:488531	TAGCTTGAGGCCGAGGTGTGCCTGAATTGCAGCAACATCCTGAACAACTGATTTTAGGGATGTTTGGATAGATGTCAAAGTAGCTTCCATCCTTGCATTT	T/A	GACTCTAGTATTTTGGCGTTGTGTTCCATTTGAGCTGCCATAAATTCCATCAGTTTTGCAACAAATGCAGGAGTCTCAGCTGCTGATTCAGAGGCTTGTG
Ca_LG_4:488592	GTTTGGATAGATGTCAAAGTAGCTTCCATCCTTGCATTTGACTCTAGTATTTTGGCGTTGTGTTCCATTTGAGCTGCCATAAATTCCATCAGTTTTGCAA	A/G	CAAATGCAGGAGTCTCAGCTGCTGATTCAGAGGCTTGTGGAGGAGTTGGTTCAACATTTGGCATTGGTCAAATCCAAACATTATCTACTTTCTTCAGTTT

Positions	-100 bp flanking sequences	SNPs within *kabuli* cultivar	+100 bp flanking sequences
Ca_LG_4:488605	TCAAAGTAGCTTCCATCCTTGCATTTGACTCTAGTATTTTGGCGTTGTGTTCCATTTGAGCTGCCATAAATTCCATCAGTTTTGCAACAAATGCAGGAGT	T/C	CTCAGCTGCTGATTCAGAGGCTTGTGGAGGAGTTGGTTCAACATTTGGCATTGGTCAAATCCAAACATTATCTACTTTCTTCAGTTTCATCTGATTCACA
Ca_LG_4:488611	TAGCTTCCATCCTTGCATTTGACTCTAGTATTTTGGCGTTGTGTTCCATTTGAGCTGCCATAAATTCCATCAGTTTTGCAACAAATGCAGGAGTCTCAGC	C/T	TGCTGATTCAGAGGCTTGTGGAGGAGTTGGTTCAACATTTGGCATTGGTCAAATCCAAACATTATCTACTTTCTTCAGTTTCATCTGATTCACAATTGCT
Ca_LG_4:1011492	TATAATCAACAAGTTGTTGCATGAAAAATAATACATACATGTCAGCCCGTTCCACACTGTATGACAGGTTTCTAACATATATTGTCTTCGATGCAGCATG	G/A	TTTTTCGTTCAGAGCGAGTGGGGTTTCATGCTACAAAAAATCCAGGAATAACATCATATAACTGATTTGATGATTATTACATTGCAGGAAGAAAAGATAT
Ca_LG_4:1011657	TTTGATGATTATTACATTGCAGGAAGAAAAGATATCGGTCAGTTACGACTTTTGTTTCAATGCGCAGCATCAAACATTTCGACATCCTTAACCTCTTTCA	A/T	CGTCTCCTATGCCTCAATAAACACAAAAAATGAAGTTTTCAGCAGCCATGGGAAAATGTATTATGAAATGCTTACCATTTCCTGAGGTGTTTTGGGGACT
Ca_LG_4:1011793	TTTCAGCAGCCATGGGAAAATGTATTATGAAATGCTTACCATTTCCTGAGGTGTTTTGGGGACTTTTTCCTCTATTTCTCCCGCATTGTACTCTGGACTC	C/T	TCCAAATCATCGGGCTGCAATGAAAACAAAGGCACCAAGATAGTAAAAACACTGCTAAAAAACAAGTATATGCAAAACGTTCAAGATACTTATTTTGTAA
Ca_LG_4:1354494	ATGAGTTTTTGATAGCTTTGATGAAGTTCATGGATTGTATCAGGGTGGAATTAAGCTGCCAACAAACATACTTAGCAAGATTAGTTCATGGATATGTGTC	C/T	ACTGCTTAATTTTGTGTGTTTTTGCAAGCTTCATGTTCTGTTCTACTCTTTTGCAGCCAAGAAAGATCATACCTTGTGCTCATTGTAGAATGGGAACCAT
Ca_LG_4:1354532	ATCAGGGTGGAATTAAGCTGCCAACAAACATACTTAGCAAGATTAGTTCATGGATATGTGTCACTGCTTAATTTTGTGTGTTTTTGCAAGCTTCATGTTC	C/A	TGTTCTACTCTTTTGCAGCCAAGAAAGATCATACCTTGTGCTCATTGTAGAATGGGAACCATTGGAAAATTGTTACATCAAAAGCAACATGGGAATGCAA
Ca_LG_4:1354847	GTTAAATCTGCAGTGGATATTCAAACATCGGGTGATCTAANNNNNNNNNNNNNNNNNNNNNNNNNNNNNNNNNNNNNNNNNNNNNNNNNNNGCTGCTGCTG	G/A	CAACAGGTACTTGCTTTATCTTTCTACGCTTGCATGTTATAAGATTAGTAATTGAATAGTATTAAACTATATACTACCAACACTTTAGATTGAAGATGTA
Ca_LG_4:1354851	AATCTGCAGTGGATATTCAAACATCGGGTGATCTAANNNNNNNNNNNNNNNNNNNNNNNNNNNNNNNNNNNNNNNNNNNNNNNNNNGCTGCTGCTGCAAC	C/T	AGGTACTTGCTTTATCTTTCTACGCTTGCATGTTATAAGATTAGTAATTGAATAGTATTAAACTATATACTACCAACACTTTAGATTGAAGATGTATTCG
Ca_LG_4:1354900	NNNNNNNNNNNNNNNNNNNNNNNNNNNNNNNNNNNNNGCTGCTGCTGCAACAGGTACTTGCTTTATCTTTCTACGCTTGCATGTTATAAGATTAGTAATT	T/C	GAATAGTATTAAACTATATACTACCAACACTTTAGATTGAAGATGTATTCGATGTTTGACATGTTTTAGTGTCCAACACATATTGTTAACATGTCTGTTT
Ca_LG_4:2857950	CCCCTGCATGTTGCACAAACGGAGCGAAATCAGAATCAACTCTACCGAGCCTCTGAATCTGCAATCCAACAAAACAAAAGCTGCTAAGTAATGAGAATAC	C/T	GATAACAAATGTGGTGAAAATGAAAACTAGAAATGCAAACAAATACTTAGCCAGTAATAATAAAAGCATTACATTATTTCCTCCAGCGGCAGCAGCCCAT
Ca_LG_4:2873956	TGAAGGCCAAATTAAACTCCAGGAAGTTGATAACCACTTCCACTCTCATGTTAGCAACAAGCAAGCTGTTGCAAACATGCTGCTGCAACTACAAGCAATC	C/G	AGTGTCATGCCATTCCAACCATTTGCCAACAAGGAGCCACACTTCAAACACTCTACTGCATGTAGCAAACAAATGATTTATGGTTTTAATTTCATTATAG

Positions	-100 bp flanking sequences	SNPs within *kabuli* cultivar	+100 bp flanking sequences
Ca_LG_4:2873959	AGGCCAAATTAAACTCCAGGAAGTTGATAACCACTTCCACTCT<br>CATGTTAGCAACAAGCAAGCTGTTGCAAACATGCTGCTGCAA<br>CTACAAGCAATCAGT	T/G	GTCATGCCATTCCAACCATTTGCCAACAAGGAGCCACACTTC<br>AAACACTCTACTGCATGTAGCAAACAAATGATTTATGGTTTTA<br>ATTTCATTATAGCAC
Ca_LG_4:2873960	GGCCAAATTAAACTCCAGGAAGTTGATAACCACTTCCACTCTC<br>ATGTTAGCAACAAGCAAGCTGTTGCAAACATGCTGCTGCAAC<br>TACAAGCAATCAGTG	G/A	TCATGCCATTCCAACCATTTGCCAACAAGGAGCCACACTTCA<br>AACACTCTACTGCATGTAGCAAACAAATGATTTATGGTTTTAA<br>TTTCATTATAGCACC
Ca_LG_4:2873964	AAATTAAACTCCAGGAAGTTGATAACCACTTCCACTCTCATGT<br>TAGCAACAAGCAAGCTGTTGCAAACATGCTGCTGCAACTACA<br>AGCAATCAGTGTCAT	T/G	GCCATTCCAACCATTTGCCAACAAGGAGCCACACTTCAAACA<br>CTCTACTGCATGTAGCAAACAAATGATTTATGGTTTTAATTTC<br>ATTATAGCACCAAAC
Ca_LG_4:2957801	NNNNNNNNNNNNNNNNNNNNNNNNNNNNNNNNNNNNNNNNNNN<br>NNNNNNNNNNNNNNNNNNNNNNNNNNNNNNNNNNNNNNNCTTGT<br>ATTGTTACTGTGAATGTA	A/G	AAATAGCAAAAGGAAGGAAGTTGTAACTAGATTGAGAGCTAT<br>GAAGATTTAATTGAAGGATAGTTTGGAGTTCATATAGTAAAG<br>AGATAAGGCTCAAGAA
Ca_LG_4:2957823	NNNNNNNNNNNNNNNNNNNNNNNNNNNNNNNNNNNNNNNNNNN<br>NNNNNNNNNNNNNNNCTTGTATTGTTACTGTGAATGTAAAATAG<br>CAAAAGGAAGGAAGTT	T/C	GTAACTAGATTGAGAGCTATGAAGATTTAATTGAAGGATAGT<br>TTGGAGTTCATATAGTAAAGAGATAAGGCTCAAGAACAAGAC<br>ATTGTTGAGTTGTGAA
Ca_LG_4:2957825	NNNNNNNNNNNNNNNNNNNNNNNNNNNNNNNNNNNNNNNNNNN<br>NNNNNNNNNNNNNCTTGTATTGTTACTGTGAATGTAAAATAGCA<br>AAAGGAAGGAAGTTGT	T/A	AACTAGATTGAGAGCTATGAAGATTTAATTGAAGGATAGTTT<br>GGAGTTCATATAGTAAAGAGATAAGGCTCAAGAACAAGACAT<br>TGTTGAGTTGTGAATG
Ca_LG_4:2957831	NNNNNNNNNNNNNNNNNNNNNNNNNNNNNNNNNNNNNNNNNNN<br>NNNNNNCTTGTATTGTTACTGTGAATGTAAAATAGCAAAAGGA<br>AGGAAGTTGTAACTAG	G/A	ATTGAGAGCTATGAAGATTTAATTGAAGGATAGTTTGGAGTT<br>CATATAGTAAAGAGATAAGGCTCAAGAACAAGACATTGTTGA<br>GTTGTGAATGAATAAT
Ca_LG_4:2957915	AGGAAGTTGTAACTAGATTGAGAGCTATGAAGATTTAATTGAA<br>GGATAGTTTGGAGTTCATATAGTAAAGAGATAAGGCTCAAGAA<br>CAAGACATTGTTGA	A/T	GTTGTGAATGAATAATTAAAGTCATGCTGCGAAGAATGCATA<br>CCACTAAGCAAAAGCTACTTTCAAAAAGTGATTCGATAATTG<br>ACGGTGGTATTGAAAA
Ca_LG_4:2980905	CAGCTTTTGAAGTTTGCCAACTTTATTATCCTCCAATTTTTGAA<br>GTTTGTCGACTTTATTTTTACCACGAACATCCAGTTGTGGTGC<br>GGCAACTGGTTTA	A/G	TTCTGGCAGCTGGTTTGCTGTGCGGGGTGGTTTATTGGATTT<br>AATCTGAAAAAATGAGGTTAATTAGTTTTTTAGTTTATTGAAT<br>CTGAAAAAATGACAA
Ca_LG_4:3078718	TATTTTTTAATCTTGAGGTCATTTGTGGCGGCCAAAGCCCTCA<br>CAAAGGAGGGATTTTGTGGCGGCCTGGGCCCTCACAAAGGC<br>TGAATTTTTTAAAATC	C/T	AGTCTGTCCTTTGTGGCGGCCTATACCCTCACAAAGGCAGA<br>CTTTTGTGGCTGCAAAGGCCCTCACAAAGGCAGCCTTTTGT<br>GGCTGCAAAAGTCCTCAC
Ca_LG_4:3078732	GAGGTCATTTGTGGCGGCCAAAGCCCTCACAAAGGAGGGATT<br>TTGTGGCGGCCTGGGCCCTCACAAAGGCTGAATTTTTTAAAA<br>TCAGTCTGTCCTTTGT	T/C	GGCGGCCTATACCCTCACAAAGGCAGACTTTTGTGGCTGCA<br>AAGGCCCTCACAAAGGCAGCCTTTTGTGGCTGCAAAAGTCC<br>TCACAAATGCCAACGTGT
Ca_LG_4:3078743	TGGCGGCCAAAGCCCTCACAAAGGAGGGATTTTGTGGCGGC<br>CTGGGCCCTCACAAAGGCTGAATTTTTTAAAATCAGTCTGTCC<br>TTTGTGGCGGCCTATA	A/G	CCCTCACAAAGGCAGACTTTTGTGGCTGCAAAGGCCCTCAC<br>AAAGGCAGCCTTTTGTGGCTGCAAAAGTCCTCACAAATGCC<br>AACGTGTTTTCCACACTT

**Positions**	**-100 bp flanking sequences**	**SNPs within *kabuli* cultivar**	**+100 bp flanking sequences**
Ca_LG_4:3078758	TCACAAAGGAGGGATTTTGTGGCGGCCTGGGCCCTCACAAAGGCTGAATTTTTTAAAATCAGTCTGTCCTTTGTGGCGGCCTATACCCTCACAAAGGCAG	G/A	ACTTTTGTGGCTGCAAAGGCCCTCACAAAGGCAGCCTTTTGTGGCTGCAAAAGTCCTCACAAATGCCAACGTGTTTTCCACACTTTGTGGCTGCAAAAGC
Ca_LG_4:3914254	TTTACACATTATCATAACTTCATAGTTTGCCTTGCCACTCACCTAAAATTGAGAAAAATATCATGAAGCCAGCAGAAACTTGAATGACCCTTCGGCTGCT	T/C	GATTCGGTTGCTTCCTAGAAGTCCTATATTCTCTCTGTCATGATGCTAAAAGAATCAATTTGTGTCCCACACAATAAATGAAAACTATGAATGAGAAAAA
Ca_LG_4:4494641	TGCTATTTTGAGGATACATAATGTTGTTGGAAATGCTGTAACAAAAGACAACTACATAGTATTAGGAACAGGCTCTTCTCAACTCTACCATGCTGCTTTA	A/G	TATGCACTTTCTCCTTCACAACCCCCTAATCATCCCATTAATGTTGTTGCTGCTGCTCCTTATTACTCGGTAATATTTCTCTCTCTTCAATTTTTTTTTT
Ca_LG_4:5673644	TTTGTTAACTAATACTAGTGTGTATTTTGGTAATTTTAATTAAGCTTTATTTTATAAATTTGATTGGAATTGAATTATGTCACACCCATCATGCCATCTG	G/C	TACTTTTTGTTGTATAAATATTATTATTACATCTCTGTAACAGTGTTCATTGCCATTTTAATTTGGAAATTAATTTTCAAATTTTATAAATTAATAATTA
Ca_LG_4:5673656	TACTAGTGTGTATTTTGGTAATTTTAATTAAGCTTTATTTTATAAATTTGATTGGAATTGAATTATGTCACACCCATCATGCCATCTGTACTTTTTGTTG	G/A	TATAAATATTATTATTACATCTCTGTAACAGTGTTCATTGCCATTTTAATTTGGAAATTAATTTTCAAATTTTATAAATTAATAATTATATTTTTGAAAT
Ca_LG_4:5673679	TTAATTAAGCTTTATTTTATAAATTTGATTGGAATTGAATTATGTCACACCCATCATGCCATCTGTACTTTTTGTTGTATAAATATTATTATTACATCTC	C/T	TGTAACAGTGTTCATTGCCATTTTAATTTGGAAATTAATTTTCAAATTTTATAAATTAATAATTATATTTTTGAAATTATAAACTAGTAATTAAAATAAT
Ca_LG_4:5673686	AGCTTTATTTTATAAATTTGATTGGAATTGAATTATGTCACACCCATCATGCCATCTGTACTTTTTGTTGTATAAATATTATTATTACATCTCTGTAACA	A/T	GTGTTCATTGCCATTTTAATTTGGAAATTAATTTTCAAATTTTATAAATTAATAATTATATTTTTGAAATTATAAACTAGTAATTAAAATAATTAATTTA
Ca_LG_4:5673689	TTTATTTTATAAATTTGATTGGAATTGAATTATGTCACACCCATCATGCCATCTGTACTTTTTGTTGTATAAATATTATTATTACATCTCTGTAACAGTG	G/C	TTCATTGCCATTTTAATTTGGAAATTAATTTTCAAATTTTATAAATTAATAATTATATTTTTGAAATTATAAACTAGTAATTAAAATAATTAATTTAATT
Ca_LG_4:6561298	AAGGGAGTAATAGCAGCCACCATACCTGAGATTTGAAAGCTTAATCAACGATCAATACCGATAAGACAGTTGATTTTGTAGAAATTATACAAAGCACAAC	C/T	TGCAGTAATTTACCAGGCAAGTGAGCTGCACCACCAGCACCAGCAATAATAACTTGAACGCCTCGTTTGTGAGCAGACGAGGCATAAGAGTACATCAGTT
Ca_LG_4:6561377	AGAAATTATACAAAGCACAACTGCAGTAATTTACCAGGCAAGTGAGCTGCACCACCAGCACCAGCAATAATAACTTGAACGCCTCGTTTGTGAGCAGACG	G/A	AGGCATAAGAGTACATCAGTTCCGGAGTTCGGTGTGCTGAAACTATTCTTATCTCATGAGGAACGCCAAACATCTCCAAGATTTCAGCAGCACTTTTCAT
Ca_LG_4:6701524	TTCAGGTATATATGATATATACCATGCTGCTATAATGATTTCTACAACACTATACGAATAATCTAATCAATTGAACAGAAAAACAATAAAATGAATTTCC	C/G	TGCCTTTAATTTGCTGCAACCATCCAAGAGACAAGGATCGAGGTCTGAGGTGCAGGAAAAGGAGCTAGAGAACAACAGTGCATAATAACTACTTTTTGTA
Ca_LG_4:7686911	CATTGACCAACCAATATTTAAGGTCAAATGGTGTATGGAAAAACAGAAGTTATCTTACATCAATCGAGATGCTCCAAAGCATTCAGCAAATGACATCAAG	G/A	GCAGGTATATAGTCGATGTCAAAACCAGCAGCAACAGCGCGTGCAAATGCCATGCCTTGCTCTTTCTGCAGCACTGATTTGCGCGTCTCTAGGACTTTCA

Positions	-100 bp flanking sequences	SNPs within *kabuli* cultivar	+100 bp flanking sequences
Ca_LG_4:7687107	TTCAAAAGCTGAACTCTGAGAAAATGTGGTATTATTGAATATAAAACATGAACAATCTGTTTCACATGCTGAAAAGAAGTTTTATGAATAATGAACAAAA	A/T	TAAGAAAAGAGCAGCATATTTATCTTATCATATTAATGTGCAATTGTGCGAAACAAGCAACTCTGTCCATCAGAGCTTTAGTAATTTATAAGAGCGCAGA
Ca_LG_4:7797075	ATCAAGGTCATGACGATGCTTTACTTGGTCTCTCATCAGGTGATGAAGTTCTCAGAGAAGTTGCAGATAAATCTACTGCAGCAGGGGTTTGGCTAAAGCG	G/T	GAAAGGTCTCTACATGACTAAATCTTTAACCAACGGACTAAACTTAAAGAAGAGGCTGCATTAGTTAAAGATGGAAGAAGATTCATCAATCAAAGAACAT
Ca_LG_4:7806322	CATATTATTATAATCATTTCACGATCACAAAACAACTTAACTTTACATAGAAATTGACGCAGCTTTTGTGAGTATATGCTGCAGTTACACTCAATTATTG	G/T	AGTAAATTACTCCATGCATGATTCAATTTGGTACTAACTAAATAATATTTAATTGCATTATAAATTTGTATATAGTACGGTGGTCCAAAATGTACTGCCA
Ca_LG_4:7871755	ACAACAAGCTCGCGACTCTAATAGAAACAAGCTTGCTCTTTTTCCTTCTCTCACTCTACTTTATTTGACAGGTGCGGAGAATATCGATACGCTAAAACAG	G/C	ACTGCTATTGGCTGCATTGCCTATCTATTCGATTATGTCAGAATCTAACTTTGATTAAAAAAAATATACTTTATCATTTTCTTTCAACTTTCATCTCTTC
Ca_LG_4:8203223	GCTAGAAGTTCCAATACACAGCGTGGGGTTATCTCTTCAAGTGTCTCATCAATCTGTGCTTGTAAATCTGGTGCTAGGCTGCTTGCCCCTTCTTCCTACG	G/A	TGATCAAGTGAGTCATCAGAAAGCATGTTGGCATATTGCATCAGAACTGCAGCTTAAAAGTCTTCAAGCTATATGCTATACACATTGTGTTACTACACAT
Ca_LG_4:8233006	CAAATTCGTTACTCACAGGCATGGGAGGCAGTTACAAATTCATTAACAACAAAATAAAGCAGCAAAGGATGAGAAGACCATCTTGACAAATTTTACAAGC	C/G	GAATTGAAAATGTCTAGAACATGCTAAAGAACTGAGCTAAAATATTTGCAGCCCTTAATTGAGAGCCCAAAAAAATTGTTTAATTTCACTCACACCAAAA
Ca_LG_4:8318211	CGTTTTGTGTGATTTAACCCATGTGCTCCCACCCTTGTGTACCTGCGCAGAAGCTGCTGTAACCAGCGTCAGCTGGTCGTATCTTCTGTCCTGTTCCCAA	A/G	TCATTATAAAAAGTAAAGTGCAGCAATAGGGTGTACCCTTTGCATATGTACACCTGGTGCATACCTGTACTCGGCCATTTTTGGCGTACCAATGCTATAG
Ca_LG_4:8333069	GGAGCATGCTGTTTACAATAGAAGAATCGGGTTGGACCATGTCGACCATTTGAGGATGTGATATTGATGATTCTAACTGAGGTTGCTGCATAGGCCATGC	C/G	ACTACTAGATTGATTGAGTAGTGGATTTTGGTTTTGATTATGATATGGAAATTGGTTTGTTAGGCTTTGAGAGTTAACAAGATTCGGTGCAGCCACTTTA
Ca_LG_4:8444120	ACATAACAAACAAATTCTATCTTGAAATTGCAGCAACGACCAGGTGTGAGGGTTCGTCAACCTGCACCTGCCACTGGCCAACCAGCTGCTGCTACAGGAA	A/G	CACAAGTTGAGGAACCCCCATCCCCTGCTTCTAGTACATCTTCAAGTCAGTTCCAGAGGGAAGTAGCAGAGGAATTGAGCTCTCCAAAAGTTCCTAAAGC
Ca_LG_4:8444194	TGGCCAACCAGCTGCTGCTACAGGAACACAAGTTGAGGAACCCCCATCCCCTGCTTCTAGTACATCTTCAAGTCAGTTCCAGAGGGAAGTAGCAGAGGAA	A/G	TTGAGCTCTCCAAAAGTTCCTAAAGCAGCCCCTCCTGTTGCAGGTTTAAATAACCAGATGTCTTCTGTAGCTGGTAACCAGAAGCCACCTGTAGCTGGTC
Ca_LG_4:8444488	CCCAAAGAAGATTCAAGCAGCAGCACCTTCATCCTCTTCAGCAAATGAAAATGGAAAATCTCCTACAAAGGAGACCATAATGGAAGAAAGCATCCGGGTT	T/C	ACACGAGGCAGACTAAGAAAAACCAGATCATCGGGAACCCAGTAGCACAATATTGTCAATTTTTAGCCTGAAATAGGGATAGTTGGACCATGCTGCTGGC
Ca_LG_4:8757470	CGATAGAGAGGAGAATGTGATTAACAGATCTGAAGAAAGTATATACGAGGCTGAAGTGTTTGTTTTACGGTTTTTCACACATATGCACTTAATTTCCAAT	T/A	TTTTTTGCTTTTAGCAGATATTTCGTCCATGGCTGCTGCCAGTAGCACCTAGGCTCCATGCAAATGGTGGTTGCCATAGCTCACCACTCACCGCCATAAC

Positions	-100 bp flanking sequences	SNPs within *kabuli* cultivar	+100 bp flanking sequences
Ca_LG_4:8762033	TTGCAGTAAGAGGTTTTGTTAATGCAGCTGCCCATCACAAGCACCATCCACTTCATCATCATTGTGGCTGCCTCTACAAGTAACACAACCTCTGCCTCCT	T/C	CATCACATTGCTACGAACTACATTATTAATAATGCAGCTCCATCCATTTCTGGTAATTCAACAACTCCAAGCTCTGAAATAAGTGTTGAACAAAAGCCTC
Ca_LG_4:8762415	CAAGTAACATAACCCCTGCCTCCCCATCACATTGCTACAAACTACATTATTAATAATGCAGCTACATCCACTTCTGGTAATTCAACAACTCCAAGCTCAG	G/C	CCTGAGGCTCTTCTTCATCACACATGCCTACTTCCACCCCTGATTCAAATTCAAATCCTGCAGCACCCAGACCTATAATGGGCAGATGTATTAACAAAAC
Ca_LG_4:8820503	TGTTACCTGAGGATTTCACTATCCGCAGGTAAATTACCTGCGGCTAAATCCGCAGGAATATTGAATAAAATCCGCAGCAAAGTTTAAGAAAACTTTAGAA	A/C	ATTTTTTTACCACTTTTCATGCAGAAATATTTGCAGCACAGTTCCTACAAATATATTTCGTATAAAAATCTGCAGGTATATCCATTGCACCAACCATATG
Ca_LG_4:8820516	TTTCACTATCCGCAGGTAAATTACCTGCGGCTAAATCCGCAGGAATATTGAATAAAATCCGCAGCAAAGTTTAAGAAAACTTTAGAAATTTTTTTACCAC	C/T	TTTTCATGCAGAAATATTTGCAGCACAGTTCCTACAAATATATTTCGTATAAAAATCTGCAGGTATATCCATTGCACCAACCATATGTTTCGCATTAAAA
Ca_LG_4:8820527	GCAGGTAAATTACCTGCGGCTAAATCCGCAGGAATATTGAATAAAATCCGCAGCAAAGTTTAAGAAAACTTTAGAAATTTTTTTACCACTTTTCATGCAG	G/A	AAATATTTGCAGCACAGTTCCTACAAATATATTTCGTATAAAAATCTGCAGGTATATCCATTGCACCAACCATATGTTTCGCATTAAAATCCGCAGATAA
Ca_LG_4:8877775	AAGGGATGACTTTGGACAAAGCATTATATAAAGCCAATCAAAAATTAGTTTCTATGCAACCATACAAGCTTATAGGAAAGGCTCCTAGTCCAATAAGCAA	A/T	AAACGTTATAGGGCTTGCTGCTGCATCTCCTTCTGTTCTTAAAGCTGCAGCTCCAGCCGAATGGTCTAGTCAAGATGTCACCGAAGTCATGAAAGGAAGG
Ca_LG_4:8912374	TCAATGTCATTGGATTTTATTGACCTACAAATTGTTTCACAATTGTAAAAAAATTGCAGCCATTATAAGTTACAAATCTCTTCCCTTGAAGAAGGAAGTG	G/A	AAGAAATTGATACACCTTATTCTCCACGCCATTGCATTAATACTTGGAATCGTTGGAATTTCCGCCGCGTTCAAGAATCACAATGAAAGTGGGATTGCCA
Ca_LG_4:8912685	TCGGGATATGATTATGGTTTCTTATTGTCATTGATGTTGCTGTTTACACTGAATGTATCAGTGTTGTCAAATTGACGCCATGACGTTGAGACATGGTGGT	T/A	TTCTATGTCAGCTGCAATGTCAATGCCTATTATATCCGCTATGTTTTTGCTATAGGCTGCAAACACCATATTTATAAAGAGGATTTTAGGTTGGCTGACA
Ca_LG_4:8921971	GTCATCTTCAGTATATTTTAACCTCCTTCACAAACTTTCTGGAAAATGTATTACAAGTGATCACATTTTAGCTTGCTGCAACTGCAAGAATTTAAAAAAT	T/C	AAAAACTACCAAGGTTGAACTGTTGTACCTTTTTATACTTCTAAAGCATGCTGCAACACATTTACTAAGCAGCTTATGAGTGAAATTTAATGAACCTTTA
Ca_LG_4:8961518	CGGTATCGCCGCAATCGAAGCGATGAAAATGCTAGGTATTCGATCCGTTGGTCTTTCACTTTCACTGTATAAGGGTTTACCTTTAGGGAGTGGGTTGGGA	A/G	TCCAGTGCGGCAAGCGCAGCTGCAGCCGCAGTTGCAGTCAACGAGTTATTCGGTAAAAGATTAACCGTTGACGACCTCGTTTTAGCTTCGTTGAAATCAG
Ca_LG_4:8961521	TATCGCCGCAATCGAAGCGATGAAAATGCTAGGTATTCGATCCGTTGGTCTTTCACTTTCACTGTATAAGGGTTTACCTTTAGGGAGTGGGTTGGGATCC	C/T	AGTGCGGCAAGCGCAGCTGCAGCCGCAGTTGCAGTCAACGAGTTATTCGGTAAAAGATTAACCGTTGACGACCTCGTTTTAGCTTCGTTGAAATCAGAAG
Ca_LG_4:8961626	GGCAAGCGCAGCTGCAGCCGCAGTTGCAGTCAACGAGTTATTCGGTAAAAGATTAACCGTTGACGACCTCGTTTTAGCTTCGTTGAAATCAGAAGAGAAA	A/G	GTTTCAGGTTACCATGCTGACAATGTTGCACCATCGATCATGGGTGGTTTCGTTCTCATAAGCAATTATGAACCGTTGGAATTGTTGAGGCTGAAATTCC

**Positions**	**-100 bp flanking sequences**	**SNPs within *kabuli* cultivar**	**+100 bp flanking sequences**
Ca_LG_4:10123723	AGAAAATGCTGAACCGATTGAGATCGATTAAATTGAACCGTTC<br>AAAGGTTCGGTTTATCCGTAGGAACCATAAGTATAACGAAGCT<br>GCGAGGACTACTGC	C/T	TGCGCCTAGGGTTGGGTTTGGTTTGGCTTCGGAGTTGCGGC<br>GTGCTGGTGTTTTTGTGAAGGTTGTGGAGGATGGTGATAAG<br>GTAAATGCTGCCGATTCG
Ca_LG_4:10410177	AATTGTAAGCAGGTAGATAAGTAGGTGCAGCAGATGTACTAA<br>TACATATGTCAGAGAGTTTAGCATCCAAACAAGGTGATTTCTC<br>AAGCTGCATAAATTA	A/C	ATTCATTTGTTATACACAATTAATCATAATCGTTGACTTCAAA<br>TATCTTACAATTTTAATTACCTGATAAGATGAGAAAATAATTG<br>GCTGCAAAGACTTG
Ca_LG_4:10553679	AGAATAGCCAATAAGTAAATATAATTTACAGAATAATATTAAAA<br>CCCAGAAGACTTTGCAAGAAAAAAATTATACAGCTGCTCAGAC<br>CTGAAGTACCAAC	C/T	GCCCAGTTGTACAGTGCATCATAATCTTCAGGGTTCCTCTCA<br>ATAGCAGCAGCATACCTAGAAAATCCAAGTAAGAAAATTGTA<br>AGTTTGAATGAAGATT
Ca_LG_4:10949142	GCCGTTGGAGGGTGCACCGAAGGCGAAAGCGAAGCCGAAGA<br>GATAGTAGGAGAGTCCACCGGCGGCAGCATCAAGGACGTTG<br>GTGAGCATGATGTTCATC	C/G	GTGTTTTTGGCTCGAACGGAGCCGGCGCAGAGCATGGCGA<br>AGCCGAGCTGCATGGCGAAGACTAAATAAGCTGAGAAAAGA<br>AGGTACGTGTTGTTCACTG
Ca_LG_4:10949166	GAAAGCGAAGCCGAAGAGATAGTAGGAGAGTCCACCGGCGG<br>CAGCATCAAGGACGTTGGTGAGCATGATGTTCATCGTGTTTTT<br>GGCTCGAACGGAGCCG	G/A	GCGCAGAGCATGGCGAAGCCGAGCTGCATGGCGAAGACTA<br>AATAAGCTGAGAAAAGAAGGTACGTGTTGTTCACTGCATAG<br>GTGGTGTCGCTGAGTTGAT
Ca_LG_4:10949264	CGGCGCAGAGCATGGCGAAGCCGAGCTGCATGGCGAAGACT<br>AAATAAGCTGAGAAAAGAAGGTACGTGTTGTTCACTGCATAG<br>GTGGTGTCGCTGAGTTG	G/T	ATTTGAAATGGCATTGAATTGGGAGCAGAGGTAGTTAGCGG<br>CGGCGGTTGCGTTTGCTGTGGCGGTGATGAATGGAGCGAG<br>GTCGGTGGGTGAGCAAGAG
Ca_LG_4:11432192	AAGAGAAAAAACTTATGTATGATTTGAGAATTTTTTTGACACTT<br>TTCTATTTCTAGTGTAAGTTTATGTCCCCAACTTTTGTTTTCTTT<br>TTATTTTAATA	A/G	AACTTTTAGCTTAGAAAAACACGAGTTCAACTTTATAACCTCA<br>TGGAATAAAGCTCCTATCTCCTCGGCAACAGTTGTAAACTGG<br>TTCACGACTTTAAAT
Ca_LG_4:11757687	TAGCACCAAATGGATTTGTTTCAGGCTTCCCTCCATTTTCCTC<br>AAAAGCTGCCCTTAAACGACCTATAAGTGCATCAAGGCTACC<br>CCAAGCTTGTCTTAG	G/A	TGGACATGGACATGGTGCTGGAGGATTTGGATGTCCATAGA<br>ATGGACATATTGGTGTATGCACTTTTGTCTTCCCAAATTGGT<br>CCAAATACCTACATACA
Ca_LG_4:11757709	AGGCTTCCCTCCATTTTCCTCAAAAGCTGCCCTTAAACGACCT<br>ATAAGTGCATCAAGGCTACCCCAAGCTTGTCTTAGTGGACAT<br>GGACATGGTGCTGGA	A/T	GGATTTGGATGTCCATAGAATGGACATATTGGTGTATGCACT<br>TTTGTCTTCCCAAATTGGTCCAAATACCTACATACATCATCAT<br>AACAACTTTTCAACA
Ca_LG_4:11852996	TAAAAAGAAAAAGAAAATGGAAGGAAATAGTAATGGAAATGTA<br>GAACAGGTTCGGTATAGAGGAATTCGTAGAAGACCATGGGGT<br>AAATTTGCAGCAGAG	G/A	ATTCGTGACCCAACAAGAAAAGGGACAAGAATATGGCTTGG<br>AACATTTGATACTGCCGAGGAAGCTGCACGTGCTTATGATGT<br>TGCTGCTTTTCATTTTC
Ca_LG_4:11853005	AAAGAAAATGGAAGGAAATAGTAATGGAAATGTAGAACAGGT<br>TCGGTATAGAGGAATTCGTAGAAGACCATGGGGTAAATTTGC<br>AGCAGAGATTCGTGAC	C/T	CCAACAAGAAAAGGGACAAGAATATGGCTTGGAACATTTGA<br>TACTGCCGAGGAAGCTGCACGTGCTTATGATGTTGCTGCTTT<br>TCATTTTCGTGGTCATA
Ca_LG_4:11853012	ATGGAAGGAAATAGTAATGGAAATGTAGAACAGGTTCGGTAT<br>AGAGGAATTCGTAGAAGACCATGGGGTAAATTTGCAGCAGAG<br>ATTCGTGACCCAACAA	A/C	GAAAAGGGACAAGAATATGGCTTGGAACATTTGATACTGCC<br>GAGGAAGCTGCACGTGCTTATGATGTTGCTGCTTTTCATTTT<br>CGTGGTCATAGAGCTAT

Positions	-100 bp flanking sequences	SNPs within *kabuli* cultivar	+100 bp flanking sequences
Ca_LG_4:11853021	AATAGTAATGGAAATGTAGAACAGGTTCGGTATAGAGGAATTCGTAGAAGACCATGGGGTAAATTTGCAGCAGAGATTCGTGACCCAACAAGAAAAGGGA	A/T	CAAGAATATGGCTTGGAACATTTGATACTGCCGAGGAAGCTGCACGTGCTTATGATGTTGCTGCTTTTCATTTTCGTGGTCATAGAGCTATTCTTAATTT
Ca_LG_4:11853024	AGTAATGGAAATGTAGAACAGGTTCGGTATAGAGGAATTCGTAGAAGACCATGGGGTAAATTTGCAGCAGAGATTCGTGACCCAACAAGAAAAGGGACAA	A/C	GAATATGGCTTGGAACATTTGATACTGCCGAGGAAGCTGCACGTGCTTATGATGTTGCTGCTTTTCATTTTCGTGGTCATAGAGCTATTCTTAATTTCCC
Ca_LG_4:12405300	ATATATATATGTTTGTACTATTTCAGTTTCAATAATAATAAGAAATTTTCATTCCCTCTCTTGTTTTAACCTAGCTGCACCCTAAAACACATGGTATCAG	G/A	AGCTTCGGCTCTGATCATGTGGCAGCTCACCATGTCCTCACAAGAAACAGAAAAAAAATGATTCATCCACTACTACAGTACCAGAAAAAAATTCTGGTGA
Ca_LG_4:12405304	ATATATGTTTGTACTATTTCAGTTTCAATAATAATAAGAAATTTTCATTCCCTCTCTTGTTTTAACCTAGCTGCACCCTAAAACACATGGTATCAGAGCT	T/C	TCGGCTCTGATCATGTGGCAGCTCACCATGTCCTCACAAGAAACAGAAAAAAAATGATTCATCCACTACTACAGTACCAGAAAAAAATTCTGGTGACCCT
Ca_LG_4:12405317	CTATTTCAGTTTCAATAATAATAAGAAATTTTCATTCCCTCTCTTGTTTTAACCTAGCTGCACCCTAAAACACATGGTATCAGAGCTTCGGCTCTGATCA	A/C	TGTGGCAGCTCACCATGTCCTCACAAGAAACAGAAAAAAAATGATTCATCCACTACTACAGTACCAGAAAAAAATTCTGGTGACCCTAAAACACATGTCA
Ca_LG_4:12415083	TGAGAAATAGCGGAAGCCGCATAACAATAATTATTTATAAAACAAAGCATGTTGCATACAAATAAAAGCTGTCTAAATAGAAATTTAAAACTCAAAATCG	G/A	AAATTCAAAATCTCAATAATTGAATTCAAAACAACTAATAGTGCATATTGCCTACTGCCTACCTGCAGCCTAAATAGAAATCCATAAAGTGATATAAAAC
Ca_LG_4:12415117	TTATAAAACAAAGCATGTTGCATACAAATAAAAGCTGTCTAAATAGAAATTTAAAACTCAAAATCGAAATTCAAAATCTCAATAATTGAATTCAAAACAA	A/C	CTAATAGTGCATATTGCCTACTGCCTACCTGCAGCCTAAATAGAAATCCATAAAGTGATATAAAACTCAAAATCTCAATTCAAAATAACTAATAATTTTT
Ca_LG_4:12415137	CATACAAATAAAAGCTGTCTAAATAGAAATTTAAAACTCAAAATCGAAATTCAAAATCTCAATAATTGAATTCAAAACAACTAATAGTGCATATTGCCTA	A/G	CTGCCTACCTGCAGCCTAAATAGAAATCCATAAAGTGATATAAAACTCAAAATCTCAATTCAAAATAACTAATAATTTTTTATTCCTAAGTCCTAACTAG
Ca_LG_4:12425564	GGCAGCCGGAGTTGATGGTGACAAATGACTGTATGAAGCCAAAAAATTGAAACACTGAAAAAAGTGGTGCTGCCGGCGGCATTTGCCGCCGCCGACAGCG	G/A	GTAGTGAAGCACAAAGACAGATCACAAAATCAGAACCTGCTCTGATACCATGTCAAGAATTAGAATAAAATTATATTTATTGAAACTTGAAATATATTGG
Ca_LG_4:12451717	CTGCAGAAGCAACAGCCATGTCAGTCTTAGCCATCTTCTCATCATCCTTGTTGTTAGATGCAGCAGCTGTTGCTGCAGTGATGGCTGCAACTGCAGAGGA	A/C	AACAGCAGCTACACAAATAGCAGCATGCAGTTGAGCATTATGGTTTCTGTTTTCTTCTTTCTTTTTTTCTCTTCTGTCTTTTAGCCATCTTCCTACTGTT
Ca_LG_4:12477345	TAAGTATGCTTGAAGAGACTAAGAAAATTGCTGAAAGAAGAGAGCAGCAAGTAATTCTTTCTCTCATGTCATTTGTTAATCATGAACCAAAACTTTCTTG	G/A	TAGTCTATTTATTGTACTTGTAGCATTTTTGTTGTTGCTGTTAACCTTGAAATCTTTTCATTCCTATGAGTGCAGCTTGTTTTGGTCAATCCCGGAAGTG
Ca_LG_4:12479697	TAGGGCAAATAAAATCAAATTATATCCAAAAAAAGGAAATAATAGTTGGCTGCAAGGTGAAAAAGGAGGAAAGTAATTCCGCTTTGTCACAAAATCATCA	A/G	ACCAATGACATGTCATTAACATACAAGGAAGCAGCAGCCTACATTACTACAAAGTTCCCTAGCATTGAAACACAAAAAATATGGGGGAAAATAGTTTCCG

Positions	-100 bp flanking sequences	SNPs within *kabuli* cultivar	+100 bp flanking sequences
Ca_LG_4:12976726	AACTTCCAAGAAATGTTCAATAAGTTGTCCAACATGTTGAACAGAAGGCTACAATGAAAGATGCAATTCAAGCACTAGACAAGAATGGAACATGGAAATT	T/A	TGTGGAGCTGCCAAAAGGAAAAAAATCAGTTGGGTATAAATGGGTCTTCTCAATAAAGCACAATGTTGATGAGAGTGTAAATAGGTGTAAAGCCCGCTTG
Ca_LG_4:12987378	TGTTAATGGTTTTCCATATTTACTATAATATGCTGCTTTTATATGTTGCATGTCAATCTCAGTCCTTGTCACAATTATCCTTATTAATCTACTGTCATTA	A/T	GTTCCAATACCCTTCATTGCTTTCCGTAAAAGCTGCAAATATATATGCATAAAATACATCATTAACTATAAACCAACTACTCCCTTCAGGCTTATTGAAA
Ca_LG_4:13006125	TTGTCAACAGAAAACAAGACTGATGCAAAAAAGGAAAATACAAATAAAATATTTACCACAGCGTGGTCAAGAGAATCAGCCAATGCTTTTGACGATGAAA	A/G	CATCCAAGTTCAGGACAACTGCAGCATCAACACGTAGCAAGGGTTCAAGCATCTCTCTGGTTGGAGTTGGATGACGCCTACGCAAGAACCAAAAAACAAT
Ca_LG_4:14208847	GACCGATGTTCGCAATATCTTCAAATGATAGCTTTATGCCTAAATGCGCTTGAATTGCAGCAACATCCTGAACAACTGAATTCAGAGAAGCCTGGAGAGA	A/T	TGTCAAGGCAGCTTCCATTCTAGCCTGTGACTCTTGCATTTTGGCATTGTGAGCCATCTGAACTTCCATAAATTCCAGCAGTTTTGCGATGTACTCAGGA
Ca_LG_4:14444844	AATACAAAATGGTGGTTCGAGAAATTACCAGTGGTGCGAGAACGGTGAGAACGACAGTGAGTGCAGCTCGGGTTCGACGGCGCGACTGGTTCGACGACGT	T/C	GACTGGTTCGACGACGAGTGTAGCTAGTTGATTGACGGCGAGAAGGAGAAGACAACGATTAGGATTCGTGACTCAGAGGTTAGGGGTCCGATTGAGGTTC
Ca_LG_4:14921013	TTGCCCTTTCATGGAAAGTCAATAGATGGCAGTTCTGATGCTGATGCTGCAGATGAAGGATGGGAAGCATCTTTTGACAGGCACACTGCTGTGAAGGACT	T/C	TGATGGTCGAAGGATTTGTTGAAACTATTTCGCTGCCAATCGGTCAAATGAAGGTCCATCATCAGATGCTTATGTATACCTTAACCCGGATGCTAGTGCC
Ca_LG_4:14921033	AATAGATGGCAGTTCTGATGCTGATGCTGCAGATGAAGGATGGGAAGCATCTTTTGACAGGCACACTGCTGTGAAGGACTTGATGGTCGAAGGATTTGTT	T/C	GAAACTATTTCGCTGCCAATCGGTCAAATGAAGGTCCATCATCAGATGCTTATGTATACCTTAACCCGGATGCTAGTGCCATGATCCGGAAACCATGCCG
Ca_LG_4:15244395	AATTTGGCGACGACTTCGAATATAATAGGGGATGCCAAGTAACAAGTAAGTAGTAGTAGTAGCAGCATATTTATTACCATCTTTACCGCTGCTGTGTTTC	C/T	CTACGTGCACGCACGTTTCAAAATCTATGTTACCACTAATTAATTTTCCCATACATACACAATAATTAATATTCATAGATTCGCAACATAACTAAGTGAG
Ca_LG_4:16499218	GACTTTTCAGTGACATGGTGACAATTTCAACTGTTGCACCAAGGTTTCCTTTCAGTAGTGCTGCTTGATTGAATGGGTTTCTGTTCTTTCTATACCTTGC	C/A	ATTAATGTTATAGTACTCTCATTTGATCATTCAGCTTATTATAAATTGTTAAGGTTATTGGAGCTGCGATGTTCTATGTAAAAGTAGGAGACGCAGAAAT
Ca_LG_4:16499256	CCAAGGTTTCCTTTCAGTAGTGCTGCTTGATTGAATGGGTTTCTGTTCTTTCTATACCTTGCATTAATGTTATAGTACTCTCATTTGATCATTCAGCTTA	A/G	TTATAAATTGTTAAGGTTATTGGAGCTGCGATGTTCTATGTAAAAGTAGGAGACGCAGAAATGGTTTATACAGGAGACTACAATATGACACCAGATAGAC
Ca_LG_4:16812351	AAAGGGTGAATGGTATCTGGATAAGGCCCCTACCAAATGCTGAACCAGTTCAGCCTAATACACAACAAGCTGCTGAGCCAGTAGCAGCCCCTCTTGAGTC	C/T	CTTAGCAAAGCTGCTAGAGTTTATGGAAGCTCAGACGACTCACAACGCTAAGATGCAAGCGTCAAATGCTCGAATGGAAGCTGCTTTGAAGACACTCCAG
Ca_LG_4:17065092	GAAACATGGGTTCATCAGGCTGGTGCTCCTGACCATGTTCATCATCAAACTGTTGCTGCTGCTGGTCATATTCATCATAAAACACATAATCGTGCTGGGA	A/G	CTGCTCATCCTGTTCTGCATCAAACATAAGATTTTGCTGGGGCTGCTCGTCCTGGGGCGCCTGCCTTTCCTCATTTCTCTTTCTCATTTTTTCAACAGCA

Positions	-100 bp flanking sequences	SNPs within *kabuli* cultivar	+100 bp flanking sequences
Ca_LG_4:17313865	AGAAAATACATCCAAAACAGGAGAGACAATGGTTGTTTCGGAAAACTTTTCCAACAGCAACGGCAGGCAGCAGCAAACGGCGGCAGCGGCAGTAGCAGAC	C/T	GGGGCAGCAGCGACGATTGTGGACGGAAGTGACAGCAGGAAACAGATCTGAAATTAATTCCAGCAAATATCCTATTGATATGCAGCCTTTATGGCAGATC
Ca_LG_4:17668344	AATCCTCCCCCTTCAGACACGGACGTCGAAGCTGCTCAAAAGGGATGTTGCAAGAGTTGCTTAAAGCCTGTGAGCAAATGTCTTTCTCTATGGCGTGAGA	A/G	GCAGAAGATGAGGGAGAGGGGGACAACGCATGATGAGCAGCTTGCGATGTAGAAGGGGTGGAATCATCAAGGAACCCCCAAGGACAGATTGGCAATGGCA
Ca_LG_4:19001574	TCAATTGATGAACTTGACATCTTCATTGTTGCAGCAATAGCACAATTATGAAGATCGGCCAAGGAAAAGAAATTCCGCTGCAAAGAAGACGATAAAGAAG	G/C	ATACTCTATTTCGCGGATGAAGTGGTGGAAGCGAAGGTGAAGGTGACAGAGAGTTTGAACTGGAAAAAGAATCTTCACTTGTAATACTAGAGCTGCTATC
Ca_LG_4:19118319	TTTTCAGCCACAAAATATAAATTTTGTGAGGGGAAATGCCTCCACAAAAGACTGAAGTGACATGTCCATTTGTGGCTGCATAGGCCGCCACAAAAGCAAC	C/G	TGAACGTTACTAATTATATTTTTAATATTTTTTAAATTTAAACAAACAACAGACTAAATGTACTTGGAAAAGAGAGCATGATGCCGCAGTTTTGGCTTCA
Ca_LG_4:19189914	AATCTTGTATCAGTATACCAAATATGAGAAGTCTTTTTAATAAAGGAAAATCATAGAAGGAGATAAAATGAAATGAAACCTTGCAGCCCTTCCAGATGAG	G/A	GTGAAGCAAATGATAACAGAAGCTTTAACCTTGATGGCCGCGCGAACCTGTATGTTTCGGCAAAAAGAAGTTTACTTTCAAATATATACTTCAATTAGTA
Ca_LG_4:19189926	GTATACCAAATATGAGAAGTCTTTTTAATAAAGGAAAATCATAGAAGGAGATAAAATGAAATGAAACCTTGCAGCCCTTCCAGATGAGGTGAAGCAAATG	G/T	ATAACAGAAGCTTTAACCTTGATGGCCGCGCGAACCTGTATGTTTCGGCAAAAAGAAGTTTACTTTCAAATATATACTTCAATTAGTATATAGTTCTTTG
Ca_LG_4:19189932	CAAATATGAGAAGTCTTTTTAATAAAGGAAAATCATAGAAGGAGATAAAATGAAATGAAACCTTGCAGCCCTTCCAGATGAGGTGAAGCAAATGATAACA	A/C	GAAGCTTTAACCTTGATGGCCGCGCGAACCTGTATGTTTCGGCAAAAAGAAGTTTACTTTCAAATATATACTTCAATTAGTATATAGTTCTTTGAGCAAA
Ca_LG_4:19439625	TCTCTAATTATTGTTTATGCTTTCAAGATGATGACCCAAATGATTGATAGAGCAACTCTTCTTGATATTTTTGCTGCTAAGGAAATTTCTGTATGTAAAA	A/G	TGCTACAATTACGGCATGAAAGTTACCTTATAATAGTATTATTGTTTGCTTAAAGTAAATTATAAAACTGATTAGGTAGCAGCTAACAATGATTATCCAT
Ca_LG_4:19439675	AGCAACTCTTCTTGATATTTTTGCTGCTAAGGAAATTTCTGTATGTAAAATGCTACAATTACGGCATGAAAGTTACCTTATAATAGTATTATTGTTTGCT	T/A	TAAAGTAAATTATAAAACTGATTAGGTAGCAGCTAACAATGATTATCCATTGATATTTTTCCCTAGATATATTAATATTCAATTATCTAGCCCCTGCATT
Ca_LG_4:19697024	AATGTAAAGTTTGACTTTTTGTATGGTAGTAATAATAATATTCAAGTTTTTCTTTATTTAATTTTTTGCTTTTGCAAATTGCAGCTATCTACTAGAGTTT	T/A	TTGGCAAAGGAAGGTTTTCTTGTTGTGGTGGTGCCTTACAATGTGACATTTGATCATTCTCAAGCTGCTAAACAAGTATATGAGAGATTTCATACTTGTT
Ca_LG_4:19697065	TCAAGTTTTTCTTTATTTAATTTTTTGCTTTTGCAAATTGCAGCTATCTACTAGAGTTTTTGGCAAAGGAAGGTTTTCTTGTTGTGGTGGTGCCTTACAA	A/G	TGTGACATTTGATCATTCTCAAGCTGCTAAACAAGTATATGAGAGATTTCATACTTGTTTGGATACAATCCTCACATATGGGTTGCCTCAAGCCAATTTG
Ca_LG_4:19839598	ACTAGTCCCATTTGGCCTAGTTTCAGGTTGCAGCTGGTTGGTGAAGTTGTCATGGGTACAAGACAAAATGACAAATATTTAATCCAAGAAAAACAAAATG	G/A	CTACACAATCTACCACTTATATTAATTCTCCCTTAGATGTCAACTTGGGTTTTGAACCTAGGCCAAATGGGTTAATGTTGGATTTGGATTCAATTCACAC

Positions	-100 bp flanking sequences	SNPs within *kabuli* cultivar	+100 bp flanking sequences
Ca_LG_4:19839602	GTCCCATTTGGCCTAGTTTCAGGTTGCAGCTGGTTGGTGAAGTTGTCATGGGTACAAGACAAAATGACAAATATTTAATCCAAGAAAAACAAAATGCTAC	C/A	ACAATCTACCACTTATATTAATTCTCCCTTAGATGTCAACTTGGGTTTTGAACCTAGGCCAAATGGGTTAATGTTGGATTTGGATTCAATTCACACCTAT
Ca_LG_4:19839612	GCCTAGTTTCAGGTTGCAGCTGGTTGGTGAAGTTGTCATGGGTACAAGACAAAATGACAAATATTTAATCCAAGAAAAACAAAATGCTACACAATCTACC	C/G	ACTTATATTAATTCTCCCTTAGATGTCAACTTGGGTTTTGAACCTAGGCCAAATGGGTTAATGTTGGATTTGGATTCAATTCACACCTATGACAAAGATT
Ca_LG_4:19989626	GCATTTACATAATAATTGATCTGAAATAGTCAGATTACACTCTTGTATTTCATAGTTAGTGGTGATTGTAATGGGCCACGGGGTGGTTCCAGGTGTTGAA	A/T	GATCCATCTTATAGTTACGAGGATTATTGAGTAGTGTTAGTCAGTGTTGAGGTTGCATCGTAGTAAATCGTGCTAGGATGTATTGAGATTGCACCTTGGT
Ca_LG_4:19989638	TAATTGATCTGAAATAGTCAGATTACACTCTTGTATTTCATAGTTAGTGGTGATTGTAATGGGCCACGGGGTGGTTCCAGGTGTTGAAGATCCATCTTAT	T/G	AGTTACGAGGATTATTGAGTAGTGTTAGTCAGTGTTGAGGTTGCATCGTAGTAAATCGTGCTAGGATGTATTGAGATTGCACCTTGGTAACTATAACTAG
Ca_LG_4:20556142	ATCCATGTACACCTTCATTGCAGCATCTTCTAAAGAATCCCTTTGAAGGGGAAGTTTTAACCTGTCCTCGTACATCTTTGTTGCTATTGCATTTGCAGTG	G/A	CTAGGGTTTCCAACTAACCCGTTCGTACCACCACCATTCAAGAGATGAGCCCTATCCCTACCCTGCTGTTGCTGCTGCTGCTGCTGTTGCTGCTGCTGCT
Ca_LG_4:21184381	ATTAAGGCACCTATGAATTTCAATTTTTTCTTTGGAAGGTTAAATGTAGATTTCATTTACTATAATTTGATAGATCTATATAAGCAGCAGCTAAAGTAGT	T/C	AAAAATAAGAGCTTGAATACTGCTTGTAAATAATCCAAGGAACATTACAGGTATAGGAATAGGAACTACTAATGGTACTAATGAAACAAGAACAACTACT
Ca_LG_4:21216019	TAATGCTGCAGAGAACAACCCTTTTGTTCTCTTTACAACTAATAAATACACTCGAAGCACAAAAACATCATTATCAAACCCTTTGTGTAACAAAAAAAAA	A/C	CCCTTTGTCTTTTTCTTTTCTTGTGTCCTCTAATTTGGATCTTCTACATACTACTGTTGCATGGTGCAGCGAGATATAAAGCCATTTCATTTAGAGAGAA
Ca_LG_4:21216323	CATGCAACTATTTTACAACCCCTCTAATGTTGAAAAAAAAAAATATTTAAAACCTCACTCATTTCTCTTTACAGCTAAATTATATATACACAAAAATTGTC	C/T	AAGGCAGTGGGGCAATAAAATAGGATCTTTGTCCCTTGGAGCAGCTTTTTCACTTGGCATTTCCACTTCTTCTTTTAGCATCAATTGATCCTGAATGCTT
Ca_LG_4:21288798	CCCACCCCCACCTCCACCACCACCTCAACAAACCTCATCATCTTCAATACCTCCTCCTCCTCTTCCTCCTCCAAGAAGTGTGAACAACTCTGCTGCTATG	G/C	TCCGGTCCTCCTCCACCACCTCCACCACCACCTCAACAAACCTCATCATCTTCAATACCTCCTCCTCCTCTTCCTCCTCCAAGAAATGTGAACAACTCTG
Ca_LG_4:21876826	AACCACCGTCTCTGTCTTCATCGGTATCTGAAAAGGCATGTATCTTCTCATTATCTTCATGTTTGCTGCTTAGATTGGAAGCTAAAATTTAAATTTAAAA	A/G	GATGAAATTTTATTTTGTTTGTGTGGATGTTGATTTTGGTTGAGATCATTTTAGTTGTGATAGTAAAGCTTAAAACTGGTTTTATTTTCTATTGGATGCT
Ca_LG_4:21876993	GCTTAAAACTGGTTTTATTTTCTATTGGATGCTTAGCTTCAGATTTTCCAATTTGTGCCAAGATTGCTTTTTTTGTTGTTGATAGGCTTTAGGAAAGCAC	C/T	GCTTGCTTGCCAGCCTGTATGAATGGCTGCTGCCAGGCTGTTCACGATACTTTTGATTTCCTATTAGTTGTTCACGATACTTTTGCATTCCTATTAGCTA
Ca_LG_4:22125617	TTTTCCTTCCTTCGTCGCCCCTCCCCTCTTAGGTACAGCACCACCGCACCGCTGCTCCACTGCATAGCACCACCACACCGCTGCTCCACTGCAATACTGT	T/C	CGTACTGCTGCAACACACCGCACCGCTACATCATCACTGCATCGTTACAGCACCGCCGCTCCACTGCAACATTACTACAGATCTTTGTTCTCGTTGTCTC

**Positions**	**-100 bp flanking sequences**	**SNPs within *kabuli* cultivar**	**+100 bp flanking sequences**
Ca_LG_4:22293200	ATTTGGGTTATGCTTGAACAACTAGGAGCATCAGTGTCCTTTATAATTGATCTGGTCTTTGCGAGTGTAATAGGTTATGGAAGCAGCTCAAGCTTCAACG	G/A	TCATTACCAGGAACCATTCGTGGAGGCTACTCATTGCAAAATGTCAATCATATTGCACATCTTTCTGGTGCTCTTGTTGGTGTTCTCTTGGTTTGGCTTC
Ca_LG_4:22399619	TGGAAGGTATGTGGGACTGATCCTAGCTAATGTGTTTGATTGTGTTGGGGACTAGTCAGTAGTCGCTTCTATTCTTTGAATTGTTGAATATCTTATGGTG	G/A	TGTTCTTTTCTTGAATTGTTGCAGTTAAAAACATATTTAACAAAAAGGGATAGTCAAAAGCTTGCTGCTGCTGTAAAACTTTATTTATACAAGAAAGGAG
Ca_LG_4:22399637	GATCCTAGCTAATGTGTTTGATTGTGTTGGGGACTAGTCAGTAGTCGCTTCTATTCTTTGAATTGTTGAATATCTTATGGTGTGTTCTTTTCTTGAATTG	G/A	TTGCAGTTAAAAACATATTTAACAAAAAGGGATAGTCAAAAGCTTGCTGCTGCTGTAAAACTTTATTTATACAAGAAAGGAGTCAGTGAGGAATGCCATG
Ca_LG_4:22716800	GTTGATGTAATTGTTAATAAATTATGACTTGAATATAACAATTTCTTCTATCTTCAGTTGTTCGAAGAGCTGCAAAAACCGGTTTAGAGGATGTCATTGC	C/T	GCCAAGAGTCAATGCAGAAGTAGACAATGTCCTTGCTTTGCTGCTGGACGTGAATGTGATCCAGATGTTTGTCGAAATTGTTGGGTTAGGTAATGAACCA
Ca_LG_4:23453802	TACAAGTAGGAGTTTATCATCTAATCTTCTAACCTCTTTGACCAAGCCATTAAGTAGAGTCAAAGCTTGTGAATATTCCTTAGTTTCCATCAAAAGAGTA	A/G	GCAAGCCTTGCCTCAACTCGCTGCCTAAGAAAGGTACGCTTTTCATCGCGAGTCCATTGCACCATTTCTTTGCAGAGTGAAATTTGTAAATCAGATGTCC
Ca_LG_4:23575384	TGAAAAAGATATTTGGATTTTGTATCAGTTATTTTGAGACTGAAAAATAAATTGTATAAAATGCAGCATTTAGAAGATATGAAGCAGCAGGGTGGTTGAG	G/A	AAAAATGGTTGGAGTTGTTGCCGCTAAAGATTTACCAGCAGAGCCTTCTGAGGAAGAGTTTAGGCTTGGTTTGAGGAGTGGGATCATTCTTTGTAATGTT
Ca_LG_4:23575398	GGATTTTGTATCAGTTATTTTGAGACTGAAAAATAAATTGTATAAAATGCAGCATTTAGAAGATATGAAGCAGCAGGGTGGTTGAGAAAAATGGTTGGAG	G/C	TTGTTGCCGCTAAAGATTTACCAGCAGAGCCTTCTGAGGAAGAGTTTAGGCTTGGTTTGAGGAGTGGGATCATTCTTTGTAATGTTCTTAACAAGGTTCA
Ca_LG_4:23846243	TATACTATTTTTTGTTAATTTTCTATCTAATATTTTTAATGTCTTTTTTATATACTTTGATTTAAAGTTGCAGCTCTGTTTTTTTTTTATTATTTAAAAT	T/A	TTATTGTTGGTGTTTAATTTGAATTTTGCATAGAAGATACTTTCAGATACTTTTGTTTTCATGTGAGTATTTGATAATTGACACCAGTTCTATATTGTAT
Ca_LG_4:23846282	TGTCTTTTTTATATACTTTGATTTAAAGTTGCAGCTCTGTTTTTTTTTTATTATTTAAAATTTATTGTTGGTGTTTAATTTGAATTTTGCATAGAAGATA	A/T	CTTTCAGATACTTTTGTTTTCATGTGAGTATTTGATAATTGACACCAGTTCTATATTGTATTTCTTTGTGCTTTGAGTGATTGACTTGATAAACAGCCAT
Ca_LG_4:23846289	TTTATATACTTTGATTTAAAGTTGCAGCTCTGTTTTTTTTTTATTATTTAAAATTTATTGTTGGTGTTTAATTTGAATTTTGCATAGAAGATACTTTCAG	G/A	ATACTTTTGTTTTCATGTGAGTATTTGATAATTGACACCAGTTCTATATTGTATTTCTTTGTGCTTTGAGTGATTGACTTGATAAACAGCCATGGTGTTC
Ca_LG_4:24384221	CACAGGAAGACCATTTATGGCATTGATGCTGCAAATATTCAACAACAAAAGCTAGTACCAGCAGCAGCACCACCACCACCACCGGTTGCCTCCTCCTCCG	G/A	CGTCCTCGTATCGGCGTGTCACAGGGCTCAAACACAAGGTCCTATCAATCAGAGGGGAAAAGGAATTGATGGATGCACTTTTTGTGGATGCTGGTGAGAT
Ca_LG_4:24393454	GTCCACATTTAAATGTCTTGTGAACAGCACCCAACTAGTTTCATCATCTAATAACTGCAGTTGGTGAACAAAGGTTGCATCTGCAGCATGTTGTGCAACA	A/G	TTTGCATTGCGAGTGGTAACAAGAATTCTGCTGCCCGTCGATGTATCGGGAATGGCCTCTTTCAAGGTATCCAAGTACACTTGAGAAGTTTCTTTTATGC

Positions	-100 bp flanking sequences	SNPs within *kabuli* cultivar	+100 bp flanking sequences
Ca_LG_4:24394311	GAACATCCAGAAATAGCGCATCCATCAATTCCTTTTCCCCTCTTATTGATTGGATCTTCTCTTTGAGCCCAGTGACAGGTTGATACAAAGAGGCAGCTGG	G/T	TTCCATCGCCTGTGCTGGTGCTGACGTAGCACCAGAACTGTCAACAATGATTTCACTCTCTTGTATGCTGCTAATTGGGCCTTCTAATAACGGTACAATT
Ca_LG_4:24394319	AGAAATAGCGCATCCATCAATTCCTTTTCCCCTCTTATTGATTGGATCTTCTCTTTGAGCCCAGTGACAGGTTGATACAAAGAGGCAGCTGGTTCCATCG	G/A	CCTGTGCTGGTGCTGACGTAGCACCAGAACTGTCAACAATGATTTCACTCTCTTGTATGCTGCTAATTGGGCCTTCTAATAACGGTACAATTAGCTCTTG
Ca_LG_4:24394321	AAATAGCGCATCCATCAATTCCTTTTCCCCTCTTATTGATTGGATCTTCTCTTTGAGCCCAGTGACAGGTTGATACAAAGAGGCAGCTGGTTCCATCGCC	C/T	TGTGCTGGTGCTGACGTAGCACCAGAACTGTCAACAATGATTTCACTCTCTTGTATGCTGCTAATTGGGCCTTCTAATAACGGTACAATTAGCTCTTGTT
Ca_LG_4:24762269	GATCCCAACTGGGGTTCCTACAATTCTCACCTCAAGTCCATCTGATGGAAAATTTCTATCAGTTGAAGAGTTAATACAAGGCTGCTATGATTCAATACAG	G/C	TTGCCACATGTTCCTGTTATGCAATCAGACACTGCTGCTGTACTTTACTCTTCAGGGACTACAGGAGTAAGTAAAGGCGTTGTTTTGACACACTCAAACC
Ca_LG_4:24763278	GTATATGTGGATTGCTTGTGTCAGGTTGCTCCTGCAGAACTCGAATCGGTGCTGCTAAGTCATCCCCTTATTGTTGATGCAGCAGTTATACCGTGCGTTA	A/G	CCCTCAGCGCAGTTTCACTCTTTTTTCATTATCTTTGACTTTCTCATGTGAAAAGATATGTTGTAGGGTTGAAGATGAAGAAACTGGGCAGATACCGATG
Ca_LG_4:24763287	GATTGCTTGTGTCAGGTTGCTCCTGCAGAACTCGAATCGGTGCTGCTAAGTCATCCCCTTATTGTTGATGCAGCAGTTATACCGTGCGTTACCCTCAGCG	G/A	CAGTTTCACTCTTTTTTCATTATCTTTGACTTTCTCATGTGAAAAGATATGTTGTAGGGTTGAAGATGAAGAAACTGGGCAGATACCGATGGCGTATGTG
Ca_LG_4:24763309	CTGCAGAACTCGAATCGGTGCTGCTAAGTCATCCCCTTATTGTTGATGCAGCAGTTATACCGTGCGTTACCCTCAGCGCAGTTTCACTCTTTTTTCATTA	A/C	TCTTTGACTTTCTCATGTGAAAAGATATGTTGTAGGGTTGAAGATGAAGAAACTGGGCAGATACCGATGGCGTATGTGGTGAGAACAGCTGGTTCTGAAC
Ca_LG_4:24771099	AAATCCCGTGACTCACTATAAAGAGTCCAACATTCAGCAGCATCTGAGCATACGATATACCTGAACATCAGATAAATGCCGACCATTACGACTTCCACCA	A/G	ACAATGTACAACTTTTCATCAACTACTGCTGCTGCATGCTACAAGGAAAGGACAATAAATGGAGTAAGAATCTGGCCCAAGGCTAAGGAATGTTTTCAGC
Ca_LG_4:25101186	ATTGATCCTTAGGTTTTAGATTGAATTCATTAAAATTTGGTAATTTGACATGAGTTTGGAGAAACTGAGAGGATAAGAGAGCCTTACAAAATATGAAGTA	A/G	GGTGCAAAACCAAATCCACCAAAGAATCCAAGAAGATCACCAAAGAAAGGAAAAGTGACACCAAAGAATAATGTTAAAGCTGAAAATATTTCAACAAACT
Ca_LG_4:25101189	GATCCTTAGGTTTTAGATTGAATTCATTAAAATTTGGTAATTTGACATGAGTTTGGAGAAACTGAGAGGATAAGAGAGCCTTACAAAATATGAAGTAGGT	T/A	GCAAAACCAAATCCACCAAAGAATCCAAGAAGATCACCAAAGAAAGGAAAAGTGACACCAAAGAATAATGTTAAAGCTGAAAATATTTCAACAAACTTTA
Ca_LG_4:25101213	CATTAAAATTTGGTAATTTGACATGAGTTTGGAGAAACTGAGAGGATAAGAGAGCCTTACAAAATATGAAGTAGGTGCAAAACCAAATCCACCAAAGAAT	T/C	CCAAGAAGATCACCAAAGAAAGGAAAAGTGACACCAAAGAATAATGTTAAAGCTGAAAATATTTCAACAAACTTTATTAGTAACATAAAGAGTTCAAGAC
Ca_LG_4:25213894	GACTATGGCAGCCACTGCCTTTAAAAAATCAGGGAAAAATACTGATAAAAAGGCAGCCTTATTATTAATCATTGGATTCACTGGTCAACTAAAAGGATGG	G/A	TGGGATAATATAGTTACTGAAGAAGAAAAAACTATGATATTAACAGCAGTTAAAGTAGATAATAATGGAATACCAATTCAGTAAATACCCTTTTATATGC

Positions	-100 bp flanking sequences	SNPs within *kabuli* cultivar	+100 bp flanking sequences
Ca_LG_4:25396413	AGTAAACCAACTGCCAGAATAAACCAGCTGTCGCACCACAACTGGATGTTCGTGGTAAAAATAAAGCCGACAAACTTCAAAAATTGGAGGGTAATAAAGC	C/T	TGGCAAACTTCAAAAGTTGGAGGGTAATAAAGCTGCCAAAGTTGTTGCTCAAAAACTGGATCGGGAAAAATCAGTTGCTGCTCTTGCTCCTAAAATAAGT
Ca_LG_4:25396417	AACCAACTGCCAGAATAAACCAGCTGTCGCACCACAACTGGATGTTCGTGGTAAAAATAAAGCCGACAAACTTCAAAAATTGGAGGGTAATAAAGCTGGC	C/A	AAACTTCAAAAGTTGGAGGGTAATAAAGCTGCCAAAGTTGTTGCTCAAAAACTGGATCGGGAAAAATCAGTTGCTGCTCTTGCTCCTAAAATAAGTCAGC
Ca_LG_4:25845067	TCAACAGAGTTTAGTGAACCCTTTCTCTAAGAGGTTTATGTGGGAAAACAGCTCTCCTCGAGGATTGACTTGGTCTTGCCAAGATGATGTGGTTTTAAAG	G/A	GAGAAATACTGCGGTTTGCCATCAAAGGCTGGTTTGAAGCTCCTAGTCACTCAAAAGTTTGTGATAGCAAGCAGCAACTAGCTTGTTTAATCTTTTTTTT
Ca_LG_4:25845078	TAGTGAACCCTTTCTCTAAGAGGTTTATGTGGGAAAACAGCTCTCCTCGAGGATTGACTTGGTCTTGCCAAGATGATGTGGTTTTAAAGGAGAAATACTG	G/T	CGGTTTGCCATCAAAGGCTGGTTTGAAGCTCCTAGTCACTCAAAAGTTTGTGATAGCAAGCAGCAACTAGCTTGTTTAATCTTTTTTTTAGGCAATACTA
Ca_LG_4:25845079	AGTGAACCCTTTCTCTAAGAGGTTTATGTGGGAAAACAGCTCTCCTCGAGGATTGACTTGGTCTTGCCAAGATGATGTGGTTTTAAAGGAGAAATACTGC	C/T	GGTTTGCCATCAAAGGCTGGTTTGAAGCTCCTAGTCACTCAAAAGTTTGTGATAGCAAGCAGCAACTAGCTTGTTTAATCTTTTTTTTAGGCAATACTAG
Ca_LG_4:26741231	AGGAATTTGGATGAAATTGTCAAGCATGGGTGCAGAGACTATTGGACTGGGTGTAGAAACTGTTGGACTGGATGTAGAGGTTGTTGGATTATGTGCAGAC	C/G	GTTGCTGGACTGGGTTCTACAATAAGGGCAGGAAAGAGTGAGGGACTTGGAATTGTTGGATTAAGAATGCAGCATGTGCCAACAGTGTGATGAGGAATAA
Ca_LG_4:26741236	TTTGGATGAAATTGTCAAGCATGGGTGCAGAGACTATTGGACTGGGTGTAGAAACTGTTGGACTGGATGTAGAGGTTGTTGGATTATGTGCAGACGTTGC	C/T	TGGACTGGGTTCTACAATAAGGGCAGGAAAGAGTGAGGGACTTGGAATTGTTGGATTAAGAATGCAGCATGTGCCAACAGTGTGATGAGGAATAAAGCTA
Ca_LG_4:26741254	GCATGGGTGCAGAGACTATTGGACTGGGTGTAGAAACTGTTGGACTGGATGTAGAGGTTGTTGGATTATGTGCAGACGTTGCTGGACTGGGTTCTACAAT	T/C	AAGGGCAGGAAAGAGTGAGGGACTTGGAATTGTTGGATTAAGAATGCAGCATGTGCCAACAGTGTGATGAGGAATAAAGCTAGTGGCAACAACAGTATGA
Ca_LG_4:26741262	GCAGAGACTATTGGACTGGGTGTAGAAACTGTTGGACTGGATGTAGAGGTTGTTGGATTATGTGCAGACGTTGCTGGACTGGGTTCTACAATAAGGGCAG	G/A	GAAAGAGTGAGGGACTTGGAATTGTTGGATTAAGAATGCAGCATGTGCCAACAGTGTGATGAGGAATAAAGCTAGTGGCAACAACAGTATGATGAGGAGA
Ca_LG_4:27089264	TTTATTTTATATATATATATATATAATTACATAATTTATATCACATGTAAAGAGCTGATTCATTCTTTAGTCTGTAAAGCAGCAGCAGCGTTTTGGTCAATC	C/T	GTAACGCTGAAAATAATTGTTTCTAATTCCATATGAAAGTATTTTATGGCTTCTGCTATATTAGAAGTTCAAATGTAATTCCATTTCATTGGTTTTTATG
Ca_LG_4:27577829	GTCCTTCATATTGAGATCATTTGATAGCATATCATCCCTTGGAGACCTTTGCACATCGTCTTCATCTCTATGATGGGAATTGTTTTCAGCAGCCGGTGTA	A/G	TCTGTCTTCTCTCCCTTGATATTTCCAATAACCAAAATGTCAGTATGACTATTACTTATAGACACAACTGGAGTAACATTATCACTCCGCTCATATCCCA
Ca_LG_4:27630244	TGTAAGAGAGCTGCTAAACGACAGTAAAGAAGCCAGCTTTACTTTATTTTGTATAATATCTGCAAGTTATTGAGTTGTGTAAAAATGTTGATATTTCGCG	G/A	CCGCGATAGCGGCAATATTTCTCACTATTGATAACATAGATTAGAAAACAAAACATTTAATGCATTGACTACAATAAATGCAGCCATTTTCGATAAATTT

Positions	-100 bp flanking sequences	SNPs within *kabuli* cultivar	+100 bp flanking sequences
Ca_LG_4:27910434	GGAGGTGTTCTTGAGGGGAAGTCTTCATAATATAATGCTTGAGGCATGTAGGAGAAAATGATCTCTGGTTTTTGTTGTGGTGAAATGCTGCTTACATGAG	G/A	GAAGGATTGGACAGATTTTGTATAGTTTTAGGATACCTTTGTTTTTCTATTCTCTTTTTGTAGAAGCCCCCTTTTTTCATGAATGATCATTTTTACTCAT
Ca_LG_4:27958812	TGTGGTTTCATGTTACGAGCACGTGCATCAGCACGCTCTTGCAGCCTTCTAATCCTAGGCGCTAACCCACACACATAATCCTGCGCCCTTTTCCCCTCAG	G/C	TCGTCAATCCCTCCAGCTTCTCTAATCTCCACCGTTGGACCAAAAACTCCAAAATATCAGCGTAATCATTTGCCGTGTAGACGCCCGTACGCTGCGCCAC
Ca_LG_4:28020781	AGCTGAAAAAACTATCAAAATCACCATTCAATTTATATTTGTATTGAAGCATGATACTAGGTTAGAAAATGATTTCCAAAACAGAGATCACTACAAAAAG	G/T	CTGCATGCACATTAATAACATTGTTCACCAATTAATTCAGAGTATTTCTCAGCAGCCACTACCAAAATTTGAGTATTTGATTTGTTATGGAACAGATGTA
Ca_LG_4:28021749	CCAATTGAAAGCACCAACACACCCCGAGTGCTTATGCTAAGCATGAATCTGTAACAACACATTCCTTCTACAAGCTGCCATAGAAACACCACCAGTCCGG	G/A	TCCTTAAAGACACAACCCCACGTTGTCAGCCCCTCTTATCGCACCCAACATCCACTTGTGCAGTAAACTCGGCAGAAACAGAATTGGACAACAATCGGTT
Ca_LG_4:28021771	CCCGAGTGCTTATGCTAAGCATGAATCTGTAACAACACATTCCTTCTACAAGCTGCCATAGAAACACCACCAGTCCGGTCCTTAAAGACACAACCCCACG	G/A	TTGTCAGCCCCTCTTATCGCACCCAACATCCACTTGTGCAGTAAACTCGGCAGAAACAGAATTGGACAACAATCGGTTATGCTGCAGAGTTTCAATCATT
Ca_LG_4:28021789	GCATGAATCTGTAACAACACATTCCTTCTACAAGCTGCCATAGAAACACCACCAGTCCGGTCCTTAAAGACACAACCCCACGTTGTCAGCCCCTCTTATC	C/G	GCACCCAACATCCACTTGTGCAGTAAACTCGGCAGAAACAGAATTGGACAACAATCGGTTATGCTGCAGAGTTTCAATCATTTGTGTCCTCATATTGGCG
Ca_LG_4:28021797	CTGTAACAACACATTCCTTCTACAAGCTGCCATAGAAACACCACCAGTCCGGTCCTTAAAGACACAACCCCACGTTGTCAGCCCCTCTTATCGCACCCAA	A/G	CATCCACTTGTGCAGTAAACTCGGCAGAAACAGAATTGGACAACAATCGGTTATGCTGCAGAGTTTCAATCATTTGTGTCCTCATATTGGCGTGGTTGAT
Ca_LG_4:28021840	CCAGTCCGGTCCTTAAAGACACAACCCCACGTTGTCAGCCCCTCTTATCGCACCCAACATCCACTTGTGCAGTAAACTCGGCAGAAACAGAATTGGACAA	A/G	CAATCGGTTATGCTGCAGAGTTTCAATCATTTGTGTCCTCATATTGGCGTGGTTGATCTCTGAAATAAAGTTGCTTGCACTCCTGGCTACCTCCATCGAG
Ca_LG_4:30847709	CTATGCTATTCTATTCTACGCTTCTCTCTTCGCTTCTTATCTTTTTTCTCTTCTCTTCACGCTGCTGCTGCTGCACCAGTCCTCTTTCTCTCTCTAATTT	T/C	TCTCTCTCTTCCCCTTTCTTCGCGACCTCTTTTTGTGCTCCAATCTCATCTCCTTTCCGCTATTTCACTCTCTGCCAAACCCACTCCAAATACTACTGGA
Ca_LG_4:31848829	CAGGTTATGTGTTCAAATTCAGGAAATATCCAATAGCATGGAGCTCAAAAAAGCAGCATGTGATGTCACTTTCATCATGTGAGGTTGAGTATAGTGCTGG	G/A	CAGCTATGCAGCCTGCCAAACATTGTGGTTGGAGTCATTAACACCAGAGCTAAATATGGGAATTTGTAGGCCAATTCAATTGCTGATTGACAATATTTCA
Ca_LG_4:32132436	AAAGCAAGTGTTGGGGTTCAAAGTTGGTGTTAAAGATTATAAATTGACTTAATATACTCTCGACTATGAAACCAAAGATACTAATATCTTGGCAGCATTC	C/T	CGAGTAACTCCTCAACCTGGAGTCCCTCTTGAATAAGTAGATGTTGCGGTAGCCGAATCTTCCATTGGGACATGGACAACTGTGTGGACCGATGGGCTTA
Ca_LG_4:32132463	TGTTAAAGATTATAAATTGACTTAATATACTCTCGACTATGAAACCAAAGATACTAATATCTTGGCAGCATTCCGAGTAACTCCTCAACCTGGAGTCCCT	T/G	CTTGAATAAGTAGATGTTGCGGTAGCCGAATCTTCCATTGGGACATGGACAACTGTGTGGACCGATGGGCTTACCATTCTTGATCTTTATAAAGGAAGAT

Positions	-100 bp flanking sequences	SNPs within *kabuli* cultivar	+100 bp flanking sequences
Ca_LG_4:32617421	AATAGAATTCACAGGTCCATGATGATAAATGAATTCTAGCATTTTTAAATTTTTCAAATGAATTCTAGCATTATTCAGAGTTGTTTTCAAGTAGCTGCGA	A/T	TATCAAAATTAAAATGTCTGCACTATCTGTTATGACAATTTGGTTTGTCATGACAATTTGGTTGTTCTAACGTATAATAGATGCATCTAATTAGTCAGCA
Ca_LG_4:32648363	TCGAAAAAGGTATGGTTTATTCTGCTAAATTTCTTTAACACATGAAAATTAATATCATTTGTTGCATAAATTAGTTTGAGATTTATATTGGAATTTATTA	A/C	TGTTTTTTGGAGCAGCAAAGGGAGCACTATGAGCGTTTGTCACAGAGTCTTAAAGCAGCAGGGCAATGACTTTTTTTGTTTTTTAATAGTATGTATCATT
Ca_LG_4:32934124	TCGGTATGGTTTCCACGCCCCATTCATGTTTTAGTCTTTGCATAATACATGAAGTTGCATGTTATTTTGTTGCTGCTAGCTTAGGGAATATGATTATTCG	G/A	GTGATGTGTTGTCTCAGTGGATTCTTATGTGTCCAATGTTTCTGTGCTGCTTTCAGTATCGATTCTCCAATGTAGATTACAACATAACAAGCGGTAACCG
Ca_LG_4:33094489	CTTGTTTATCACCTAGAGATAGTAGCTAGGTGACACTGTGATGTTCTGGGGACAACACTTTGGCACGGATGATAAAGCTGCACTATGGTATAATTATACC	C/A	TTGGCATTAGGAAAATCAGTGGAGAAACAAGGCTGGATAGGTGTGTTTATACTGAAATTGCAGCAGGCTGCAATATTGTGAAGATTTCACATGTATGATA
Ca_LG_4:33094497	TCACCTAGAGATAGTAGCTAGGTGACACTGTGATGTTCTGGGGACAACACTTTGGCACGGATGATAAAGCTGCACTATGGTATAATTATACCTTGGCATT	T/A	AGGAAAATCAGTGGAGAAACAAGGCTGGATAGGTGTGTTTATACTGAAATTGCAGCAGGCTGCAATATTGTGAAGATTTCACATGTATGATATGATCATT
Ca_LG_4:33094520	GACACTGTGATGTTCTGGGGACAACACTTTGGCACGGATGATAAAGCTGCACTATGGTATAATTATACCTTGGCATTAGGAAAATCAGTGGAGAAACAAG	G/A	GCTGGATAGGTGTGTTTATACTGAAATTGCAGCAGGCTGCAATATTGTGAAGATTTCACATGTATGATATGATCATTACAACTTAGTACACACAATGGCT
Ca_LG_4:33094526	GTGATGTTCTGGGGACAACACTTTGGCACGGATGATAAAGCTGCACTATGGTATAATTATACCTTGGCATTAGGAAAATCAGTGGAGAAACAAGGCTGGA	A/T	TAGGTGTGTTTATACTGAAATTGCAGCAGGCTGCAATATTGTGAAGATTTCACATGTATGATATGATCATTACAACTTAGTACACACAATGGCTGCATTA
Ca_LG_4:33094541	CAACACTTTGGCACGGATGATAAAGCTGCACTATGGTATAATTATACCTTGGCATTAGGAAAATCAGTGGAGAAACAAGGCTGGATAGGTGTGTTTATAC	C/T	TGAAATTGCAGCAGGCTGCAATATTGTGAAGATTTCACATGTATGATATGATCATTACAACTTAGTACACACAATGGCTGCATTAGCCCAAATCAACTTG
Ca_LG_4:33094544	CACTTTGGCACGGATGATAAAGCTGCACTATGGTATAATTATACCTTGGCATTAGGAAAATCAGTGGAGAAACAAGGCTGGATAGGTGTGTTTATACTGA	A/G	AATTGCAGCAGGCTGCAATATTGTGAAGATTTCACATGTATGATATGATCATTACAACTTAGTACACACAATGGCTGCATTAGCCCAAATCAACTTGTAT
Ca_LG_4:33328604	GGACAAACAAAAAGGAAAATGGAGCTGCTAGCCACCAATGAGCAGTATCTCACGACTCAGTAACCATTAGAAACAACATGTCCATTTCAATAAATGTATT	T/A	CTCTATTTTGAACATTGGAATGACTCACCAGGCCATGCAGCGATTCCAATATGTTGAAGAACTGGTTGAGTTGGAATTGTCCCATCAACATCAAGGACTA
Ca_LG_4:33414132	AAACCAAACTTTGTTAAAGATAAGGGTTAGTGAGAAAAACATGGGTTGTGTCGCCTCTAAACTAGAGGAAGAAGAAGAAGTTGTAGCTATCTGTAGAGAG	G/A	AGGAAACGCCAGTTGAAACTAGCTGTGGAGAAAAGGTATGAACTTGCTGAAGCTCATTGCAAGTATTTTCATTCTTTGAATGCTGTAGCTGCTTCCATTA
Ca_LG_4:33414141	TTTGTTAAAGATAAGGGTTAGTGAGAAAAACATGGGTTGTGTCGCCTCTAAACTAGAGGAAGAAGAAGAAGTTGTAGCTATCTGTAGAGAGAGGAAACGC	C/T	CAGTTGAAACTAGCTGTGGAGAAAAGGTATGAACTTGCTGAAGCTCATTGCAAGTATTTTCATTCTTTGAATGCTGTAGCTGCTTCCATTAAGCTTTTTG

**Positions**	**-100 bp flanking sequences**	**SNPs within *kabuli* cultivar**	**+100 bp flanking sequences**
Ca_LG_4:33414169	AACATGGGTTGTGTCGCCTCTAAACTAGAGGAAGAAGAAGAAGTTGTAGCTATCTGTAGAGAGAGGAAACGCCAGTTGAAACTAGCTGTGGAGAAAAGGT	T/A	ATGAACTTGCTGAAGCTCATTGCAAGTATTTTCATTCTTTGAATGCTGTAGCTGCTTCCATTAAGCTTTTTGTTGCACGCCATTCTTCACCTTCTTCACC
Ca_LG_4:33483504	TGTTTGCTCAAGTGTAGGTAAGTAAGGCAGTGAAGATATGTATATATCTTGCATTGAGGGAATTTAATCCATTTTTGAATGTTCTTATTTGTTACCAACA	A/T	CAAGCAACTTATTTGATAGAGTTGTTTATCTTACAAACTATACTTTTGCAGCCCTACATTCTCACAAGCCGGAATTGTTTTACTTTTCAATAGTCTTGCA
Ca_LG_4:33517058	TGCCTTTTTTGCTTATACGTTCGACCAAATTTTTGACAAATTTAATTTGTTCCATTCTGACAGCAATATGAAGAGCTGTGTCTTTATCCATAGTTAAGGA	A/G	AGTGCATATCCAATCAGGATGAGTTTTATCGTACGATGAAGCTGTTTTCCACTCCCCTGTTGCTGCTGCCGTACAAATACGCTTTTTATATGCTCCTGTG
Ca_LG_4:33517062	TTTTTTGCTTATACGTTCGACCAAATTTTTGACAAATTTAATTTGTTCCATTCTGACAGCAATATGAAGAGCTGTGTCTTTATCCATAGTTAAGGAAGTG	G/A	CATATCCAATCAGGATGAGTTTTATCGTACGATGAAGCTGTTTTCCACTCCCCTGTTGCTGCTGCCGTACAAATACGCTTTTTATATGCTCCTGTGCACA
Ca_LG_4:33561629	AGACACGTGAGAGACAGTTGTTCCTGAAGCAGCTCCTAGGTAAAGGACTTTAGCACCAGGTTTCTTTATAACACAAAAATAAATGTCAACACAATATAAG	G/T	TGGAAGACAATTTCAGTATAGTTGCATATGTAACATAAAAATGAAGCATCTTACAATCCATATGTTGTCAACACCACCAAGAACAGCAGCAGCCAACTTG
Ca_LG_4:33687949	TTACCTTGCCATGATATCTTTGTTCTCTCTTCAAGTGAATAGAAACAGCAGGAATGTCCTTAAGTAAAAGGCGTTCTGCAAGTAGCTTCCCAATCTTTGA	A/C	AGCTGCAGCTACATCCCGAGTAGTTTCCAAGTTTGATCTCAAGGCTTTCTCTTGTGAGCTTGCAGAAGAGGCTACAGTAGCAGTTGGGGTGTGGATCACT
Ca_LG_4:33718331	TCTACTCTCCTTAGGATAATTCTTTTCCCTATCCCTATCCCTCGTATCCCTATGCTCATAATCAACTCTCATATCACCATCATCTACACCACTCCTGTCC	C/A	GATGCAACCGGAAAAGCAGCAGCCGGAGGGGGCACGCCAATTCTCTCCGACCGCGCTTTCTGAGCCAAAGCCCTCAGCTCCTGTTCCTTCCTTTCCTTCT
Ca_LG_4:33824284	ACAAAACTAGCCACACCCATTTTGTATAAAATCCAGAAAAAAAATATTGCATATATTATCATGTTTCATTGTTGCTGCAATCAAATCTATTTAACAGCAC	C/G	ACAAACTTTCTTAGCTTCAATACAGAATGCTTTCTAGCTAACAGAGTTTTGGGGCGATCTCAAAGTAGCCCAACTGTGAACCACATTCATTGTCCTCTGG
Ca_LG_4:33895688	TATTCTTACCTAATCAAATTCAAGATTTAGTCCCACAAGAAAGTCATAGACCTTATCTTTTTCGGTAAGCTCCTTCAAGACTACTGAATCTACTGCACAA	A/G	TGAATCTTTATCACTCTATAGTGGTCCAATTCCATCCAAAGATACTTCAATTGCTTGGCATATTATGTTACCTTGCTTTGCATCTACTGTTTTCACCTTC
Ca_LG_4:33895692	CTTACCTAATCAAATTCAAGATTTAGTCCCACAAGAAAGTCATAGACCTTATCTTTTTCGGTAAGCTCCTTCAAGACTACTGAATCTACTGCACAATGAA	A/G	TCTTTATCACTCTATAGTGGTCCAATTCCATCCAAAGATACTTCAATTGCTTGGCATATTATGTTACCTTGCTTTGCATCTACTGTTTTCACCTTCACAT
Ca_LG_4:33895725	AGAAAGTCATAGACCTTATCTTTTTCGGTAAGCTCCTTCAAGACTACTGAATCTACTGCACAATGAATCTTTATCACTCTATAGTGGTCCAATTCCATCC	C/G	AAAGATACTTCAATTGCTTGGCATATTATGTTACCTTGCTTTGCATCTACTGTTTTCACCTTCACATCATACACTTTGGCAACATATTTAGCCTTTGAAT
Ca_LG_4:33895726	GAAAGTCATAGACCTTATCTTTTTCGGTAAGCTCCTTCAAGACTACTGAATCTACTGCACAATGAATCTTTATCACTCTATAGTGGTCCAATTCCATCCA	A/G	AAGATACTTCAATTGCTTGGCATATTATGTTACCTTGCTTTGCATCTACTGTTTTCACCTTCACATCATACACTTTGGCAACATATTTAGCCTTTGAATA

**Positions**	**-100 bp flanking sequences**	**SNPs within *kabuli* cultivar**	**+100 bp flanking sequences**
Ca_LG_4:33895731	TCATAGACCTTATCTTTTTCGGTAAGCTCCTTCAAGACTACTGAATCTACTGCACAATGAATCTTTATCACTCTATAGTGGTCCAATTCCATCCAAAGAT	T/G	ACTTCAATTGCTTGGCATATTATGTTACCTTGCTTTGCATCTACTGTTTTCACCTTCACATCATACACTTTGGCAACATATTTAGCCTTTGAATAGGTTT
Ca_LG_4:33895742	ATCTTTTTCGGTAAGCTCCTTCAAGACTACTGAATCTACTGCACAATGAATCTTTATCACTCTATAGTGGTCCAATTCCATCCAAAGATACTTCAATTGC	C/G	TTGGCATATTATGTTACCTTGCTTTGCATCTACTGTTTTCACCTTCACATCATACACTTTGGCAACATATTTAGCCTTTGAATAGGTTTGTTCCATTGCA
Ca_LG_4:34028631	TGGGCGACTTGCTGAGATTGTATTCCATGGTAAAAAAAAATATGTTGGATGAGTGGAGCGCTACTTGAGTGCACAATGGTTTGAGGGGGTGACTCACATG	G/A	GCTGGTGCAAGGTCTCAATGGCAGCCCTCGTCTATCGGGTTTATAAAATGTTGATGATTCTTTCTCGTCAGTGTATAATAAGGCGAGAAATTGACCTTTG
Ca_LG_4:34074437	AGCCCCAATAAATCCATGAGATATTATTTGCAAAATGACCCCGTTGAGCCCAGTATCACTTATAGAACCAATTCCTATAATTATGAAACCCATATGAGAG	G/T	ACCGAGGAATAAGCTATTCTTTTTTTTAAATTGCGTTGACCAAAAGCTGTTAAAGCGGCATAGATTATTTGAATAGAACCTACTATCATTAACCAGGGGC
Ca_LG_4:34074440	CCCAATAAATCCATGAGATATTATTTGCAAAATGACCCCGTTGAGCCCAGTATCACTTATAGAACCAATTCCTATAATTATGAAACCCATATGAGAGACC	C/A	GAGGAATAAGCTATTCTTTTTTTTAAATTGCGTTGACCAAAAGCTGTTAAAGCGGCATAGATTATTTGAATAGAACCTACTATCATTAACCAGGGGCAAA
Ca_LG_4:34074449	TCCATGAGATATTATTTGCAAAATGACCCCGTTGAGCCCAGTATCACTTATAGAACCAATTCCTATAATTATGAAACCCATATGAGAGACCGAGGAATAA	A/G	GCTATTCTTTTTTTTAAATTGCGTTGACCAAAAGCTGTTAAAGCGGCATAGATTATTTGAATAGAACCTACTATCATTAACCAGGGGCAAAATATGGAAT
Ca_LG_4:34243730	GATATCTTGAAGAACTGAGGTTCGAGTGACCATATACTGGATATACCAAGTTTCAGCAAAACAGTGGGCTCCAGCATCTTGTATATCAAGAATTTTGCAT	T/C	AGTGAAGTGGGGTTTACGTCCAAGTGTACACCCTTAAGTACAAAACTGAACCCAAGGCTTCCTTTACAGCCATTGCATGCAGCATAAAATGCCTTTACCA
Ca_LG_4:34243736	TTGAAGAACTGAGGTTCGAGTGACCATATACTGGATATACCAAGTTTCAGCAAAACAGTGGGCTCCAGCATCTTGTATATCAAGAATTTTGCATAGTGAA	A/T	GTGGGGTTTACGTCCAAGTGTACACCCTTAAGTACAAAACTGAACCCAAGGCTTCCTTTACAGCCATTGCATGCAGCATAAAATGCCTTTACCAGTCTTG
Ca_LG_4:34243745	TGAGGTTCGAGTGACCATATACTGGATATACCAAGTTTCAGCAAAACAGTGGGCTCCAGCATCTTGTATATCAAGAATTTTGCATAGTGAAGTGGGGTTT	T/A	ACGTCCAAGTGTACACCCTTAAGTACAAAACTGAACCCAAGGCTTCCTTTACAGCCATTGCATGCAGCATAAAATGCCTTTACCAGTCTTGGGTATTGCG
Ca_LG_4:34596600	AATGGTACCTTGTCCTTGAAACCAAGTTCCCACAGCAACAGCCCAATCTGCATAATTGGCAAAACCAACCAATTTTTCGCAAGAAATGAGTGGAAATGTA	A/T	TTGAAACTGAGCGCAAGAAAAAGCAGCAACAGTTACAGTGTCGGGGGAAGTTTGTGGAAAAATCACCAAAAGATGGTAAACACCTCGGAATGTGGCATAG
Ca_LG_4:34785904	CGCTATCGCGTCTCTCTAAAATTGCAGTCATGGTAAAATCTCGGTTTATTTAAATTTAATTATCAGGGCAATAAGGGGCATGATGACTTGACGTCATCCT	T/C	CACCTTCCTCCGGCTTATCACCGGCAGTCTGTTCAGGGTTCCAAACTCAATGTTGGCAACTAAACACGAGGGTTTGACAGATGCGATAGTTCTCTTGTTA
Ca_LG_4:34906257	ACTTTCCACGATATGTGAAAAGAGAAAATTCTGAAAGTGATGTTATCGTTGGTGTAATCAATTCTGGATTAACAAAGCATGTATATATATTATCAGTCTA	A/C	ACTTACATCAGTGTCAAATGATACTATATACTTTAAAAATGTAATTTCCATTGGATCATTACATGCTATGCAACATGGAGTGTTGACAATATTGGTAGCT

Positions	-100 bp flanking sequences	SNPs within *kabuli* cultivar	+100 bp flanking sequences
Ca_LG_4:34906364	TCAGTGTCAAATGATACTATATACTTTAAAAATGTAATTTCCATTGGATCATTACATGCTATGCAACATGGAGTGTTGACAATATTGGTAGCTGGAAACA	A/T	CGGGTCCTTGTCCTTCAACCTTAGACAATTTTTCACCATGGGCAATTGTTGTGGCGGCTAGCAGATTAGATAGAATTTTTCTCTCTGCCTCTCATTTTTA
Ca_LG_4:34906373	AATGATACTATATACTTTAAAAATGTAATTTCCATTGGATCATTACATGCTATGCAACATGGAGTGTTGACAATATTGGTAGCTGGAAACACGGGTCCTT	T/A	GTCCTTCAACCTTAGACAATTTTTCACCATGGGCAATTGTTGTGGCGGCTAGCAGATTAGATAGAATTTTTCTCTCTGCCTCTCATTTTTAGGTTTGTTA
Ca_LG_4:34906389	TTAAAAATGTAATTTCCATTGGATCATTACATGCTATGCAACATGGAGTGTTGACAATATTGGTAGCTGGAAACACGGGTCCTTGTCCTTCAACCTTAGA	A/G	CAATTTTTCACCATGGGCAATTGTTGTGGCGGCTAGCAGATTAGATAGAATTTTTCTCTCTGCCTCTCATTTTTAGGTTTGTTATTTTTAATTAAACATT
Ca_LG_4:35013400	GACGACCAACCGAACAATGCCCCCTCTGCCCCGTTAACTATGGACTTACTTGCTGTTCTTTCGACCTCTCCATCCGCACCTGCTCAAAATGGCGGCAACG	G/A	CATCCATGACTAGTGCGGACCAGATGAGAGAACAGCAATTCACTTCAGTCGTTGGAGAAAGAAGCAGCGGCAGTTCTCAGTCTCCAAATGATGATTCAGA
Ca_LG_4:35080336	CTTAGATGATAAATGATGAGGGATGGTAATATTGTTACTTTCATGGTGATGATGTGCAGCCTCAAGTTGAAGTTAAGAGCTTTCCTGCTGAACCTATTAA	A/T	GCCCCTTGCTGCTAATCATTTGGGCACTTATCTAGCTGGTGGAGCTTTGTCAGGTGACATATACTTGTGGGAGGTGAGTTTTCAATTTTCAGATTCATGC
Ca_LG_4:35491044	TTCACATTACAAAAACCATCAATTAAGAAAAGAACCATCACTACAAGAAAACAAGGATTTTGTGAGGGCCAAAAGCCCTCACAAAGGGTAGAAAGCAGCT	T/C	ACAAAGGGGGTATTTGTGGCGGCCTTAGGCAGCCACAAATACGCTGGTCACAAACGTTTGTGGCGGCCAACAATGAAGACATTGACGATGTTGCATATAG
Ca_LG_4:35496143	ATTTTGGTTGTTTCGTCTTTTCCAAATGAGTAAATTAGTAGACGTTTTAAATTAGGCTCGCTTCGGGCAGCAGACGGTAAAGAGGGAAAATGGTTAATTT	T/C	GCGGCAGTAGCAGGAGTAAACGTTTCAGGCAAAGAGTTGTTGGTTTTACATTGCAGCTTATTAGAGGTCTGTGGTCATTGTTAGTAGTTGTTTGCAGGTT
Ca_LG_4:35496689	TATGATGGAATGAGAGTTTACTGTTTACCTTCATATGTTATCTGGTGAGATAGATTGCAGAACTTCAATTCAGAGGATAATGTATTTGGGCAGCTGCAAT	T/G	GGAAAATTTGGGAAAATCAGGTTATACTTATAGACACATTCATGTACATAAATTTAGGAAAATCAGTTTCATTTTGAAAGAATATGCAGAAAGTTTATCT
Ca_LG_4:35586575	CAATAAAACCTAAATTGCAGCTGCAACTTAACTGTTTTCATTTACCCTTAAAAGGGTTAACGTTTAAAAATTACAAGTGTCTCCAATCATTGATTTTGTC	C/G	TTCAAACAAATGAGAAAATGATTGATGAAATTATGCTTTGTTGTTGGTTTGTAATTTGAGCAGTGCAAAATCTTCCATCGCAAGCCACGGTTTATTAGCT
Ca_LG_4:35767834	TCTACCCATGACTGATAGGGCCAGTTACCCCAAAAGGGGTTATATCTGGCCACCAATGACAATGGCATTGGCCATTGTTTTGATTTCTACTACTGTGGTT	T/C	TCTGCTGCTGCAGATAGTTACCTTTACTCTTCTCCTCCACCTCCTTATGAGTATAAGTCACCTCCACCTCCATCTCCTTCACCACCACCTCCTTATGTGT
Ca_LG_4:35835436	ATTTAAACAAGATTCCAACTTGATTAAAGTGATAAATATTTTATCACATTGATAAATTTTCCACCATATATTCAATTCTATTCTAAATGATGCTTGTTCC	C/T	AATTTAAAAGATTTGTATTAAATTTCAATTGATTGAGTAAACATCACGACATCGGTTGGAGCGGTGGGCGGCGGCTAGTCAGAGGCGGTAGTGTGAGAAC
Ca_LG_4:35835439	TAAACAAGATTCCAACTTGATTAAAGTGATAAATATTTTATCACATTGATAAATTTTCCACCATATATTCAATTCTATTCTAAATGATGCTTGTTCCAAT	T/G	TTAAAAGATTTGTATTAAATTTCAATTGATTGAGTAAACATCACGACATCGGTTGGAGCGGTGGGCGGCGGCTAGTCAGAGGCGGTAGTGTGAGAACGAC

Positions	-100 bp flanking sequences	SNPs within *kabuli* cultivar	+100 bp flanking sequences
Ca_LG_4:35835466	GATAAATATTTTATCACATTGATAAATTTTCCACCATATATTCAATTCTATTCTAAATGATGCTTGTTCCAATTTAAAAGATTTGTATTAAATTTCAATT	T/G	GATTGAGTAAACATCACGACATCGGTTGGAGCGGTGGGCGGCGGCTAGTCAGAGGCGGTAGTGTGAGAACGACGGCATCGACGGTGTCGCAACTAAGAGA
Ca_LG_4:35835468	TAAATATTTTATCACATTGATAAATTTTCCACCATATATTCAATTCTATTCTAAATGATGCTTGTTCCAATTTAAAAGATTTGTATTAAATTTCAATTGA	A/T	TTGAGTAAACATCACGACATCGGTTGGAGCGGTGGGCGGCGGCTAGTCAGAGGCGGTAGTGTGAGAACGACGGCATCGACGGTGTCGCAACTAAGAGACG
Ca_LG_4:36145339	GTTCACTTTGCATGCTGTTGTGGTGTTATTCTTGGGTGTTGGATTGTTTGGTGTGTTGGCTAGTAACCGCAATTTGAGAGGCAGCGAGGCGAAGGGACCG	G/T	TTGCTGGCTGAAATTGAATCTACTGATATGAGGATATGTGATCTTTCTGAGCTGCAGATGGGGTGAAATGTGATTGGAAACAAAGAAGGAAAGTTTGCAA
Ca_LG_4:36247123	CACCATCACCATCCTATAGCATAAATGAATTAAGTTAGAGAGGCATAGGACAGATATTGTTTGCATTTTGTATCATATTGCAACACCACAAATTTGCACT	T/C	TGATATTATTATTAAATAAACTCACATGAGCTGTTGGTTCTACTTTGCTAACAAGCATGTCAGGATTTGGACGATACTTTGATACATCCTCACTCTTATT
Ca_LG_4:36822272	ACATGGGAGATGATCAAAAAAATATGAAGAAAAATAAAAGAAACATACTGAAATTGGAGCAGCACCACATTTCCACTTGTTTACAGGATTCTTCAAGTTG	G/A	GTTACAGTTGCCATATATCCATTCAAACCAGCAGCCAGTATATGGTAGCAGATGTGTCCAAGAACCTGCAATAATATCAACAAGTTATAACAAGTGAAAA
Ca_LG_4:36822287	AAAAAAATATGAAGAAAAATAAAAGAAACATACTGAAATTGGAGCAGCACCACATTTCCACTTGTTTACAGGATTCTTCAAGTTGGTTACAGTTGCCATA	A/G	TATCCATTCAAACCAGCAGCCAGTATATGGTAGCAGATGTGTCCAAGAACCTGCAATAATATCAACAAGTTATAACAAGTGAAAAATCGAACACTGATGC
Ca_LG_4:36824657	TTTCTGCAAGCTGTGCGGCATCTGTGTTAGATGTCACACCTGAAATAAAATACCGTTTCAACATTAAGAAACACCTTTTGAAGATGTGAATATAACCATA	A/C	CATGAAGATTCAGGAAGAAGTATACCTCCAATTATGACAAGTCCATCAAGTTTCAAATCATTGCATGCGGCGAGTGCAGCATTAACTTGTTCTGTGGTTC
Ca_LG_4:36824673	GGCATCTGTGTTAGATGTCACACCTGAAATAAAATACCGTTTCAACATTAAGAAACACCTTTTGAAGATGTGAATATAACCATACATGAAGATTCAGGAA	A/T	GAAGTATACCTCCAATTATGACAAGTCCATCAAGTTTCAAATCATTGCATGCGGCGAGTGCAGCATTAACTTGTTCTGTGGTTCTAATTTGATCTTTCGT
Ca_LG_4:37080038	TATGTGTCAAGCAAAAAAATATGGAGCATTAATCTTCTAAGTAACCAATTCAAATACTCCAAATATGGCTACATAGATGCAGCCTATGAAAGCAAGTTCG	G/A	GCTAACATGATGTGTGAATCAAAAATGATGTTAGCCAAATTGTTCGGCTAAGCAAGTTCGTCTAACTACAAAGATGCAGCCTATGAAAGCCAAATTGTGT
Ca_LG_4:37080098	AAATATGGCTACATAGATGCAGCCTATGAAAGCAAGTTCGGCTAACATGATGTGTGAATCAAAAATGATGTTAGCCAAATTGTTCGGCTAAGCAAGTTCG	G/A	TCTAACTACAAAGATGCAGCCTATGAAAGCCAAATTGTGTTCCATTTCCATATCATTATCAAGAACAGTACCACGCTAGTACCACTTACCTCATTCTAGA
Ca_LG_4:37323427	GACAACTCCTAGAAATTCTTATAAGATTGATGAACAACACCCTGGATCTGATCTTGCTGCTGAAACTTCAGCTGCTTTGGCTGCTGCTTCCATTGTTTTC	C/T	AATTCCGTGAATCCCAAATATGCATCCAAATTGTTAACTCACGCTACACAAGTATGATTTATTTTTCTTTTTTTTTTGTCATACCTCTATTATATGCAAA
Ca_LG_4:37323430	AACTCCTAGAAATTCTTATAAGATTGATGAACAACACCCTGGATCTGATCTTGCTGCTGAAACTTCAGCTGCTTTGGCTGCTGCTTCCATTGTTTTCAAT	T/C	TCCGTGAATCCCAAATATGCATCCAAATTGTTAACTCACGCTACACAAGTATGATTTATTTTTCTTTTTTTTTTGTCATACCTCTATTATATGCAAATTA

Positions	-100 bp flanking sequences	SNPs within *kabuli* cultivar	+100 bp flanking sequences
Ca_LG_4:37323682	CTTAAAGAAATGCATCTAAATAGAAAATAGAAAGTTTTTTTTTG TTAGGTGAAAATTTTTTATAATATTTTTGTTGCAGCTGTTTGAC TTTGCAAACAAC	C/T	TATCAAGGCTTATACCATAATAGTATCCCACCGGCAGCGAAA ATATATTCCAGCAGTGGATATAAGGTAATCTACTTAATTGTAT TTGAATTTACTAAAC
Ca_LG_4:37323692	TGCATCTAAATAGAAAATAGAAAGTTTTTTTTTGTTAGGTGAAA ATTTTTTATAATATTTTTGTTGCAGCTGTTTGACTTTGCAAACA ACTATCAAGGCT	T/A	TATACCATAATAGTATCCCACCGGCAGCGAAAATATATTCCA GCAGTGGATATAAGGTAATCTACTTAATTGTATTTGAATTTAC TAAACAAAGAAAGTG
Ca_LG_4:37520959	TAAACCACCCGCACAACAAAATAAAGCCGACAAACCTAAAAAT TGGAGGGTAATAAAGCTGGCAAACTACAAAAACTGGAGGGTA ATAAAACTGCCAAAA	A/C	TTGCAGCTAAAAAACTGGATCGGGGAAAATCAATCGCTGTT GCTCCTAAAATAAGTCAGCCATGCCTTGCTAAATACGGGGC GTGTCTTGACATCCAAGT
Ca_LG_4:37535970	ACACAAACCTTTTCACCCTCCTTCTCCTTTCATTCTCGGAAAA CGGTTACGTTCTGAACAAGATGACGCCGTTTCCGTTGCTGCA CCTACCGCTCCACCT	T/G	GCTCTTTGGGCGCTTCCGTCAAGGCCTGACTTCGCTCAACT TTGGAGCTTCGCCGCCGCCGCAGCACAACCTCCGTCGGAA GTTCTTTCCATTTCTCCGC
Ca_LG_4:37825237	AGAAAAACTCACACCTTGGACGATGTCATTACGTCATTACGTC ATCACCGTCGTTGCCTTGCTGCCACCGTCGCCACCGTGTTGT CGCTGTCATCACCCG	G/A	AGGTCATCCTAGACGTCGTCGTTGTCGCCTATGTCTCTACCA TTGTCGTCTTCATTCTTTGTTCGTTTATCTTGTTTGCTGCCTG GCCGCCTCGTTACCA
Ca_LG_4:37825261	GTCATTACGTCATTACGTCATCACCGTCGTTGCCTTGCTGCCA CCGTCGCCACCGTGTTGTCGCTGTCATCACCCGAGGTCATCC TAGACGTCGTCGTTG	G/A	TCGCCTATGTCTCTACCATTGTCGTCTTCATTCTTTGTTCGTT TATCTTGTTTGCTGCCTGGCCGCCTCGTTACCATGTTGGCC GCCTCCTTTAATGTTG
Ca_LG_4:37825281	TCACCGTCGTTGCCTTGCTGCCACCGTCGCCACCGTGTTGTC GCTGTCATCACCCGAGGTCATCCTAGACGTCGTCGTTGTCGC CTATGTCTCTACCATT	T/C	GTCGTCTTCATTCTTTGTTCGTTTATCTTGTTTGCTGCCTGGC CGCCTCGTTACCATGTTGGCCGCCTCCTTTAATGTTGGCCG CCTCCTTTCATTGTTG
Ca_LG_4:37825282	CACCGTCGTTGCCTTGCTGCCACCGTCGCCACCGTGTTGTCG CTGTCATCACCCGAGGTCATCCTAGACGTCGTCGTTGTCGCC TATGTCTCTACCATTG	G/A	TCGTCTTCATTCTTTGTTCGTTTATCTTGTTTGCTGCCTGGCC GCCTCGTTACCATGTTGGCCGCCTCCTTTAATGTTGGCCGC CTCCTTTCATTGTTGG
Ca_LG_4:38127899	ATAACAAGGCCACAAAAGCAAAACTACTGCTGCGATTAGACT GACATACCTAGAGAGATTTTACGAGATTCGTAAATTAATCATT CAACTTTTGAACACC	C/T	TTTTTGACACACTTACAAAACTTATAATAGACATTAACTGCAG CCTTTATGTTTCTCCAATTCAAATTCGGAATTCCATATATTGT GTGGTCAAACAGCA
Ca_LG_4:38127903	CAAGGCCACAAAAGCAAAACTACTGCTGCGATTAGACTGACA TACCTAGAGAGATTTTACGAGATTCGTAAATTAATCATTCAACT TTTGAACACCTTTT	T/G	TGACACACTTACAAAACTTATAATAGACATTAACTGCAGCCTT TATGTTTCTCCAATTCAAATTCGGAATTCCATATATTGTGTGG TCAAACAGCAATTA
Ca_LG_4:38169653	ATCACACTTGATTTCGAAGGGGGTGGTCCACTTGGATGGCTG AATGATGGGAGTGGAGGTCACTGTTGCCTTAAAGCAATTAAA CGCCTTCTTGCATTTG	G/A	TCATCAAAGGTTAAAGTGACATCTTTTTGCAGCAAATTTGAC AGTGGAAAAGTTATCTTGTTGAAGTCTTTGATAAAGCGCCTG TAAAAACTTGCTTGGC
Ca_LG_4:38288915	TCGCTATTTCACCCTAGCCTCACAAGAATGGGTCATTTAAACT AATGTACTGTACTTCAATAAGATTAGCTTACCTCTTCAAAAGG AGACTGAATTCCAG	G/A	GAGGCGGTTTAGAATGTTCTTCAGTGTACACTGCAGCTTGG CCTAATGCAGCTAAAAGAGTTTGCTCAGCATATCCAATTCGC AACTTGGTCTAGAAAAC

Positions	-100 bp flanking sequences	SNPs within *kabuli* cultivar	+100 bp flanking sequences
Ca_LG_4:39255601	TCAGAAGCACAAGAAGAATCTGAATAATCTTAGAGGAGAAAATCAGCAGCTACAAATTACCATTGAAAATCCTGAAGACGAGTGCAATTCATTTGAGAAT	T/G	CTAAATGGATATTTGAGGCAGCAGAAGTTGGAGTTAGAGGACAATTGCTCCCTTATGGGAGCCAGGTTGAGGGAATCAAGTGAAAGGTTTGCTGATTGCT
Ca_LG_4:39255616	GAATCTGAATAATCTTAGAGGAGAAAATCAGCAGCTACAAATTACCATTGAAAATCCTGAAGACGAGTGCAATTCATTTGAGAATCTAAATGGATATTTG	G/A	AGGCAGCAGAAGTTGGAGTTAGAGGACAATTGCTCCCTTATGGGAGCCAGGTTGAGGGAATCAAGTGAAAGGTTTGCTGATTGCTGTGGAAGAGTGGGAT
Ca_LG_4:39322609	CGGGGTGCTTACAGTTGCTGGGTTCCTTAATCTGGGAAAGGATGAAGTACTGCATCTTGCCGGTGAGCTTTGCGAAGAACTTCAAGCGCTTGGCAAACCC	C/T	GGTGAAGCTGCCAAAATTGCCCTGGAGTACTGTGGAGATGTTAACTCTGGTGTGAATTTATTAATTTCTGCACGGGATTGGGAAGAAGCTTTGAGGGTTG
Ca_LG_4:39336421	GATGGATTTTTTCAATCTAACAATCGTAGTTTTTGAAAGTAACTATTATAAGGGATTTGATCACAAGGAAGCCATCCATCCAATTCATCCATGTCTTTAT	T/C	TGTTTCAAAGGAAGTATCAGAAATTTGCAGCAACTTGTCGTGGTGTGATTGTGTTGAGCGAAAAAGTCCCACATCGCCCAAAACTATAAATAAATTGGCA
Ca_LG_4:39336462	ACTATTATAAGGGATTTGATCACAAGGAAGCCATCCATCCAATTCATCCATGTCTTTATTGTTTCAAAGGAAGTATCAGAAATTTGCAGCAACTTGTCGT	T/C	GGTGTGATTGTGTTGAGCGAAAAAGTCCCACATCGCCCAAAACTATAAATAAATTGGCAGAAACTCTTATTTAAGGTAGTGTCAGAAATTGAGAAAAGCA
Ca_LG_4:39941711	TCAATAATCATGCACAGTATTAGCAAGTTGTCAAATGCCGTGTATAGTTTTAGTGTAATATTTATGTATAGTTTTTGTCTTCTATATGTATCTTTTAATT	T/C	AAATAATAAGAGTAGTTAATTTGCGATTAAAAATTAGACCAACTTTCTTAAATTCATATAATTTTGGTTTTTGTATGTTATTTATTTAATTATTTATATA
Ca_LG_4:40124290	ATTACAATTTAAAACAATAAAGAAAACCCCTTTATGCTGTCTTGCCAGCTGTTACATGCCTTGTTTGTTTTCCTTTTGCTGCACTACCTTGCTTTTGCTC	C/T	TAGCTGCCTGCCTTATTTGATCTTTCTGTTGCTGCACTGCATTGAAGCACTATCATTGTGCCTGTTTTGTAACTAAAAAAAGATAGTCTTATAAGTCAGA
Ca_LG_4:40124294	CAATTTAAAACAATAAAGAAAACCCCTTTATGCTGTCTTGCCAGCTGTTACATGCCTTGTTTGTTTTCCTTTTGCTGCACTACCTTGCTTTTGCTCTAGC	C/A	TGCCTGCCTTATTTGATCTTTCTGTTGCTGCACTGCATTGAAGCACTATCATTGTGCCTGTTTTGTAACTAAAAAAAGATAGTCTTATAAGTCAGAGAGA
Ca_LG_4:40124297	TTTAAAACAATAAAGAAAACCCCTTTATGCTGTCTTGCCAGCTGTTACATGCCTTGTTTGTTTTCCTTTTGCTGCACTACCTTGCTTTTGCTCTAGCTGC	C/T	CTGCCTTATTTGATCTTTCTGTTGCTGCACTGCATTGAAGCACTATCATTGTGCCTGTTTTGTAACTAAAAAAAGATAGTCTTATAAGTCAGAGAGATGA
Ca_LG_4:40363080	GGTCTCAACCAGGAACGATTAATATAAACCAATTATCCCAACATTCATTTCAGCTTTTAGGCTATTTTTCAAATGTGCGGCTAAATCGTTCAGTGGTACA	A/G	GAGTCACATGCTGCAAAATACATTTCTAATCAAAATTGTTAGAAAAAAACTAGATATAATAGTTTCAATTATTCCTCTAATTAGATCATTGGCTAAAGGG
Ca_LG_4:40612505	ACAACGTATTAACGCCCAATTAAGGCCTTCAAAGAATGGATGTTGTTTAATCTCCGCAGCGCCTTTCAATGTACCCAAACGGTTTTCAGGCTCTTTAACT	T/C	AACAATCCTTTAATTAGATCCCTCGCTTGGAAACTAACAAATGGGCTATCAGGGAATCTGAGATTTTGCAACACAACGTTGGCTAACGTTTCTTCATTGT
Ca_LG_4:40612511	TATTAACGCCCAATTAAGGCCTTCAAAGAATGGATGTTGTTTAATCTCCGCAGCGCCTTTCAATGTACCCAAACGGTTTTCAGGCTCTTTAACTAACAAT	T/C	CCTTTAATTAGATCCCTCGCTTGGAAACTAACAAATGGGCTATCAGGGAATCTGAGATTTTGCAACACAACGTTGGCTAACGTTTCTTCATTGTTAGAAC

Positions	-100 bp flanking sequences	SNPs within *kabuli* cultivar	+100 bp flanking sequences
Ca_LG_4:40612515	AACGCCCAATTAAGGCCTTCAAAGAATGGATGTTGTTTAATCTCCGCAGCGCCTTTCAATGTACCCAAACGGTTTTCAGGCTCTTTAACTAACAATCCTT	T/C	TAATTAGATCCCTCGCTTGGAAACTAACAAATGGGCTATCAGGGAATCTGAGATTTTGCAACACAACGTTGGCTAACGTTTCTTCATTGTTAGAACCTTT
Ca_LG_4:40612520	CCAATTAAGGCCTTCAAAGAATGGATGTTGTTTAATCTCCGCAGCGCCTTTCAATGTACCCAAACGGTTTTCAGGCTCTTTAACTAACAATCCTTTAATT	T/C	AGATCCCTCGCTTGGAAACTAACAAATGGGCTATCAGGGAATCTGAGATTTTGCAACACAACGTTGGCTAACGTTTCTTCATTGTTAGAACCTTTGAAGG
Ca_LG_4:40612529	GCCTTCAAAGAATGGATGTTGTTTAATCTCCGCAGCGCCTTTCAATGTACCCAAACGGTTTTCAGGCTCTTTAACTAACAATCCTTTAATTAGATCCCTC	C/A	GCTTGGAAACTAACAAATGGGCTATCAGGGAATCTGAGATTTTGCAACACAACGTTGGCTAACGTTTCTTCATTGTTAGAACCTTTGAAGGGTGTTCTAC
Ca_LG_4:40612534	CAAAGAATGGATGTTGTTTAATCTCCGCAGCGCCTTTCAATGTACCCAAACGGTTTTCAGGCTCTTTAACTAACAATCCTTTAATTAGATCCCTCGCTTG	G/C	GAAACTAACAAATGGGCTATCAGGGAATCTGAGATTTTGCAACACAACGTTGGCTAACGTTTCTTCATTGTTAGAACCTTTGAAGGGTGTTCTACCATAT
Ca_LG_4:40612551	TTAATCTCCGCAGCGCCTTTCAATGTACCCAAACGGTTTTCAGGCTCTTTAACTAACAATCCTTTAATTAGATCCCTCGCTTGGAAACTAACAAATGGGC	C/T	TATCAGGGAATCTGAGATTTTGCAACACAACGTTGGCTAACGTTTCTTCATTGTTAGAACCTTTGAAGGGTGTTCTACCATATAAAGCTCATAAAGGAA
Ca_LG_4:40612552	TAATCTCCGCAGCGCCTTTCAATGTACCCAAACGGTTTTCAGGCTCTTTAACTAACAATCCTTTAATTAGATCCCTCGCTTGGAAACTAACAAATGGGCT	T/C	ATCAGGGAATCTGAGATTTTGCAACACAACGTTGGCTAACGTTTCTTCATTGTTAGAACCTTTGAAGGGTGTTCTACCATATAAAAGCTCATAAAGGAAA
Ca_LG_4:41204559	GTTACTCGCTGCTGCGGTTGAGATCGTGATTCTTGATTTGTTTTTGGGTTGTCCTTGCTTGAGCGTGTTACTCGTTGCTGCTGCTTGGTCGATTGTCATC	C/T	GTGAGCGTCTTACTTGTTTCTGCGTTGCTGCTCCTGCTCTGTTTCTGCGTTGCTGCTCCTGCTCTGTTTTTGTGTTGCTGCTCCTGCTCTGTTTCTGTAC
Ca_LG_4:42064694	CCTCACAAATGTTTAAAAATTTAGGTAGATTTTGTGGCTGCATAAGCCCTCACAAAATCACAACAAAATCACAACGTTGTGATTTTGTAAGGGCTTAGGA	A/C	AGCCACAAAATCCACCTAAATTTTTAAGATTTTTGTGAGGGCTTTTACAGCCACAAAATCTAAATTTTGTGAGGGTAAAGGCTGCCATAAATGCCTGAAG
Ca_LG_4:42064902	CACTTTGTGGTTGCAAATGCCGCCACAAAAGCCTGAGCCTTTGCATTTTGTTGCTGCAAAGGCCGCCACAAAAGAATAAAATTACGTGGCATTTTTTGGT	T/C	CGCCCAGGCCGCCACAAAGTTTTATACCTGTTACATTTTGTGGCTGCTTTGACCGCCACAAAGGATCAACCAAATCCTATATATATTACTCCACTCCTCT
Ca_LG_4:42080626	ACCTCACAATTATGTAAGTTAATAGATCATATATTAATTCTCTTTCTCTAAAAATATAGAAAAAAGGGGTTTGTGCACCAATATCTGCCATAAAAGGTCT	T/C	CAGCCATAGTAGATAAGAAGCTATATGACTTAACTCCAACATAATTATTCGTATATAGCTGGCTGTTTTAGGTACTTGAATATTTCCCAATTGTTCTGGT
Ca_LG_4:43193173	ACTTGGCTATTACCTGAATGATGACTTTCCACTGTCTCCAAATGCTATAAGAAATTAAAATATGTCTATGGTCAATTAATTTGTTCTTTGTTAATGTCTA	A/G	TAAAAGATAAAACTAGTTTATAATTTACCATGTGATTGGAGCATGTGACATTAGCACCACTATTGTGTTTTCTTTGTCCCACCTTTAATTTTTTCTGCAT
Ca_LG_4:43193220	TAAGAAATTAAAATATGTCTATGGTCAATTAATTTGTTCTTTGTTAATGTCTATAAAAGATAAAACTAGTTTATAATTTACCATGTGATTGGAGCATGTG	G/T	ACATTAGCACCACTATTGTGTTTTCTTTGTCCCACCTTTAATTTTTTCTGCATATAAAACAACATGTCAGTATTCATCCGAGTACGTATAAATGATATAT

**Positions**	**-100 bp flanking sequences**	**SNPs within *kabuli* cultivar**	**+100 bp flanking sequences**
Ca_LG_4:43193229	AAAATATGTCTATGGTCAATTAATTTGTTCTTTGTTAATGTCTATAAAAGATAAAACTAGTTTATAATTTACCATGTGATTGGAGCATGTGACATTAGCA	A/T	CCACTATTGTGTTTTCTTTGTCCCACCTTTAATTTTTTCTGCATATAAAACAACATGTCAGTATTCATCCGAGTACGTATAAATGATATATTGTGCTTGA
Ca_LG_4:43215941	TATGTATATAGATATAAATAAAATAATACCAGGAGTTAGCTCTAATACAAGGAGTATCGCATAGGAGCCGCATTCCAATTTCAGCAAAATTAGGATTTGC	C/T	AATTCCATTAGCCGCATTCCAAATTCAATTGCAGCCGCATTCCAAATTCACTTCCAATTTCATCAGATTCCCATTTGGATCCAATTTCCAATTCCAATCA
Ca_LG_4:43215945	TATATAGATATAAATAAAATAATACCAGGAGTTAGCTCTAATACAAGGAGTATCGCATAGGAGCCGCATTCCAATTTCAGCAAAATTAGGATTTGCAATT	T/C	CCATTAGCCGCATTCCAAATTCAATTGCAGCCGCATTCCAAATTCACTTCCAATTTCATCAGATTCCCATTTGGATCCAATTTCCAATTCCAATCACAAT
Ca_LG_4:43215955	TAAATAAAATAATACCAGGAGTTAGCTCTAATACAAGGAGTATCGCATAGGAGCCGCATTCCAATTTCAGCAAAATTAGGATTTGCAATTCCATTAGCCG	G/A	CATTCCAAATTCAATTGCAGCCGCATTCCAAATTCACTTCCAATTTCATCAGATTCCCATTTGGATCCAATTTCCAATTCCAATCACAATTCCAATTCCA
Ca_LG_4:43384229	GAAAATATGAAGAGATGCAGCCCAATACAAATCAATGCCAAACACTCAAAATTGATGATCTTCCCAATACCTTTAATGGCCTTAAGACTGATTGATCCTC	C/G	GTCAGGAGCAAATCAAAGGTGTGGTAGCTGCAAAGACTCCAACGATCACGCTAGTTATGGATTCAAAATAGTTTTAGTAACGAAGAGAACATACCTTAGA
Ca_LG_4:43467673	AATGACGCTCCAAGAGTCAATGTCAATAGGGACGACCAAATGGCGGAAGCTATGAATAATATGGCTGCTTCTTTTGCTGCACGGACTGCTGCAAAGACTT	T/C	TGCGGGATCTAGAAAAGAGGGAAAGAGAGATCCGTGCTGATGAGTCAAGAGGATTGGAAGATTTTCGCCGTTATAATCCTCTCAAGTTTAAGAGTGATGA
Ca_LG_4:43467674	ATGACGCTCCAAGAGTCAATGTCAATAGGGACGACCAAATGGCGGAAGCTATGAATAATATGGCTGCTTCTTTTGCTGCACGGACTGCTGCAAAGACTTT	T/A	GCGGGATCTAGAAAAGAGGGAAAGAGAGATCCGTGCTGATGAGTCAAGAGGATTGGAAGATTTTCGCCGTTATAATCCTCTCAAGTTTAAGAGTGATGAG
Ca_LG_4:43467675	TGACGCTCCAAGAGTCAATGTCAATAGGGACGACCAAATGGCGGAAGCTATGAATAATATGGCTGCTTCTTTTGCTGCACGGACTGCTGCAAAGACTTTG	G/A	CGGGATCTAGAAAAGAGGGAAAGAGAGATCCGTGCTGATGAGTCAAGAGGATTGGAAGATTTTCGCCGTTATAATCCTCTCAAGTTTAAGAGTGATGAGA
Ca_LG_4:43467691	AATGTCAATAGGGACGACCAAATGGCGGAAGCTATGAATAATATGGCTGCTTCTTTTGCTGCACGGACTGCTGCAAAGACTTTGCGGGATCTAGAAAAGA	A/G	GGGAAAGAGAGATCCGTGCTGATGAGTCAAGAGGATTGGAAGATTTTCGCCGTTATAATCCTCTCAAGTTTAAGAGTGATGAGAATTCAGAGAAAGCGGA
Ca_LG_4:43478844	CACTTGAGATGAAAGCAACTAAAACTGCTATGCTAGAAAGTGAGACAGCCAAAACCCTTGTTCTTCCAATTGCAGCAGATAAGCGGAAAAGGGATAGAGT	T/C	CATTTGATTCACACAGAAGAATGCCAATAGTTGACGGAAAAACCTGTAATAGAAATTTGTATATATAATTAAACAAAATTTACAACTCAAAAACCTCATG
Ca_LG_4:43497433	TGATGGTCTAACTACAGCAGCGGGAGGATTAGAAGGGATATATGATTGGAGCAGCAACAACAAATTTTAAAAGAATTGCGGATGCCATAAGAATAAGATA	A/G	AAACAAAAATTAATGGTTGGGTGGAGCTGCAGCTGCCATCACCTATCACTTACACATATGCACAAGATCCCTTAGAAAAGTTTTATCCCGATGATGGTGA
Ca_LG_4:43522145	GAGCAAGGCATGTCTGACTTCTTTTAGGAGCAGGAGCAGCAACTGATTTTCCCCGATCCAGTTTTTGAGCAGCAACTTTGGCAGCTTTATTACCCTCCAT	T/G	CTTTTGAAGTTTTCCAGCTTTATTACCCTCCAATTTTTGAAGTTTTCCGACTTTATTTTTACCACGAACATCCAGTTGTGGTGCGGCAGTTGGTTTATTC

**Positions**	**-100 bp flanking sequences**	**SNPs within *kabuli* cultivar**	**+100 bp flanking sequences**
Ca_LG_4:44270158	GCATCCACGAAAATCCCGCATGGGGTGTGGATGGTTTCGATCCTTTTGTTCCAGGGGGGATGGCCTCTCATCATATTGCAGCAGGGACATTGGGCATATA	A/T	AGCGGGCCTTTTCCATCTTAGTGTACGTCCACCTCAACGTTTATACAAAGGGTTGCGGATGGGAAATATTGAAACCGTCCTTTCCAGTAGTATCACGGTT
Ca_LG_4:44273943	ACCAATCTTGTGCATCCACGTAAATCCCGCATGGGGTGTGGATGGTTTCGATCCTTTTGTTCCAGGGGGGATGGCCTCTCATCATATTGCAGCAGGGACG	G/A	TTAGGCATATTAGCGGGCCTTTTCCATCTTAGTGTACGTCCACCTCAACGTTTATACAAAGGGTTGCGTATGGGAAATATTGAAACCGTCCTTTCCAGTA
Ca_LG_4:44274053	TAGCGGGCCTTTTCCATCTTAGTGTACGTCCACCTCAACGTTTATACAAAGGGTTGCGTATGGGAAATATTGAAACCGTCCTTTCCAGTAGTATCGCGGT	T/C	TGTATTTTTTGCAGCTTTTGTTGTTGCTGGAACTATGTGGTATGTTTCAGCAACTACCCCGATTGAATTATTTGGTCCTACTCGTTATCAATGGGATCAG
Ca_LG_4:44389271	ATGAAGCCTCAAGGAGTCACAGAAGACCACATTAAGCTGAGAGTCTTCCCATTTTCACTCCAAGATGCTGCCAAGGACTGGTTATACTACCTTCAACCGG	G/T	GTTTTGTTACAAGTTGGAATGATCTCAAGATATTGTTTCTAGAAAAATTCTTCCCTGCCTCAAGTGTTGCTTCAATCAGAAAAGAAATATGTGGCATCAG
Ca_LG_4:44389292	GAAGACCACATTAAGCTGAGAGTCTTCCCATTTTCACTCCAAGATGCTGCCAAGGACTGGTTATACTACCTTCAACCGGGTTTTGTTACAAGTTGGAATG	G/C	ATCTCAAGATATTGTTTCTAGAAAAATTCTTCCCTGCCTCAAGTGTTGCTTCAATCAGAAAAGAAATATGTGGCATCAGGCAGATTGACAGGGAATCATT
Ca_LG_4:44389293	AAGACCACATTAAGCTGAGAGTCTTCCCATTTTCACTCCAAGATGCTGCCAAGGACTGGTTATACTACCTTCAACCGGGTTTTGTTACAAGTTGGAATGA	A/G	TCTCAAGATATTGTTTCTAGAAAAATTCTTCCCTGCCTCAAGTGTTGCTTCAATCAGAAAAGAAATATGTGGCATCAGGCAGATTGACAGGGAATCATTA
Ca_LG_4:44389299	ACATTAAGCTGAGAGTCTTCCCATTTTCACTCCAAGATGCTGCCAAGGACTGGTTATACTACCTTCAACCGGGTTTTGTTACAAGTTGGAATGATCTCAA	A/T	GATATTGTTTCTAGAAAAATTCTTCCCTGCCTCAAGTGTTGCTTCAATCAGAAAAGAAATATGTGGCATCAGGCAGATTGACAGGGAATCATTACATGAG
Ca_LG_4:44389329	TCCAAGATGCTGCCAAGGACTGGTTATACTACCTTCAACCGGGTTTTGTTACAAGTTGGAATGATCTCAAGATATTGTTTCTAGAAAAATTCTTCCCTGC	C/T	CTCAAGTGTTGCTTCAATCAGAAAAGAAATATGTGGCATCAGGCAGATTGACAGGGAATCATTACATGAGTATTGAGAGAGATTCAAGAAATTAGTGTCA
Ca_LG_4:44881279	TGTAAGAGCTCAACGGGGTATGGGGTAGAGATTGTGTTCTTAGGGGGGAGGATTTTCAGCTGATCTACCTTATGGTTGGATAGATAAATGTTTTGATTTA	A/C	TGCAATTATTTTTTAACAAGGGTTATTGAATATAAAAAACTTATTACACGAAACCCTATTTTCTTAGAACAGGTTGAGGGAGTAGGTATTATTGGTAGAG
Ca_LG_4:44881313	TGTTCTTAGGGGGGAGGATTTTCAGCTGATCTACCTTATGGTTGGATAGATAAATGTTTTGATTTATGCAATTATTTTTTAACAAGGGTTATTGAATATA	A/C	AAAAACTTATTACACGAAACCCTATTTTCTTAGAACAGGTTGAGGGAGTAGGTATTATTGGTAGAGATGAAGTAATAAATTGGGGCTTATCAGGATCGAT
Ca_LG_4:44881327	AGGATTTTCAGCTGATCTACCTTATGGTTGGATAGATAAATGTTTTGATTTATGCAATTATTTTTTAACAAGGGTTATTGAATATAAAAAACTTATTACA	A/G	CGAAACCCTATTTTCTTAGAACAGGTTGAGGGAGTAGGTATTATTGGTAGAGATGAAGTAATAAATTGGGGCTTATCAGGATCGATGCTTCGAGCTTCTG
Ca_LG_4:45153049	GTGATGACATTAAAGAGATATGCTTTGTATTGGATGGAAGTAATGAATAATTTTTTGCACCATTTCTTAGTTGATAACTAATCCTTAAGATATTTAAGTT	T/A	AATTTTTTATTTTATGTGGATTTACATGGGATTTTATTTTTTGCTGCAAATGTCTAAGGAATTACATGGAGGTAATATTCTGCAGGAAAAATCCGCAACA

Positions	-100 bp flanking sequences	SNPs within *kabuli* cultivar	+100 bp flanking sequences
Ca_LG_4:45153056	CATTAAAGAGATATGCTTTGTATTGGATGGAAGTAATGAATAATTTTTTGCACCATTTCTTAGTTGATAACTAATCCTTAAGATATTTAAGTTAATTTTT	T/G	TATTTTATGTGGATTTACATGGGATTTTATTTTTTGCTGCAAATGTCTAAGGAATTACATGGAGGTAATATTCTGCAGGAAAAATCCGCAACAAAGGTTA
Ca_LG_4:45153078	TTGGATGGAAGTAATGAATAATTTTTTGCACCATTTCTTAGTTGATAACTAATCCTTAAGATATTTAAGTTAATTTTTTATTTTATGTGGATTTACATGG	G/C	GATTTTATTTTTTGCTGCAAATGTCTAAGGAATTACATGGAGGTAATATTCTGCAGGAAAAATCCGCAACAAAGGTTATGTTTGTCCGATCTTTGGGAAA
Ca_LG_4:45741886	TCCACCTTCGGCACTCAACTGCGGTGCTGGCTGGCGGGTACTATGTCATTATTTCACAGTACATGTATAAGTGCATTTATTTCATGAAAATTAGAATCAT	T/C	GTGTTGAAGTTTGATGATTTCAGGGTATTGGGACTCTTTTAGATGGCTCTTTTGGCACGACAACTGGATCCACTGCATCAGTATACGTTTTGCTGCACTT
Ca_LG_4:45741928	ATGTCATTATTTCACAGTACATGTATAAGTGCATTTATTTCATGAAAATTAGAATCATGTGTTGAAGTTTGATGATTTCAGGGTATTGGGACTCTTTTAG	G/T	ATGGCTCTTTTGGCACGACAACTGGATCCACTGCATCAGTATACGTTTTGCTGCACTTTTTATTGTCATTAAATTTGTTTGACCTTGGAGAATTTTATCA
Ca_LG_4:45741958	GCATTTATTTCATGAAAATTAGAATCATGTGTTGAAGTTTGATGATTTCAGGGTATTGGGACTCTTTTAGATGGCTCTTTTGGCACGACAACTGGATCCA	A/G	CTGCATCAGTATACGTTTTGCTGCACTTTTTATTGTCATTAAATTTGTTTGACCTTGGAGAATTTTATCATTTATTATCTCATGTTGAGTATATTAAATA
Ca_LG_4:46585492	GCCAAGTTCCCATTCCCCATGGGCAAAATGGCACTTGTCACCAAATTTGCAGCCTTCAGCAGAATTAAACTTGTTACATATGCGGCTTTTAACAGCAGAC	C/A	GGTGCAGAACCATTGTTAGCGACTGGGGGCGGTGCGACAACATTTCTGGATAATTGAGGTGCAGCAGAAGATTTTAGATTCATCATCTGGGCAACAGCAT
Ca_LG_4:47085638	TTTTGAATCTTTATTTATCAACTATCAATCTATGTGCATTGTTATCTTTCAGAATTTTGTTGGATAAATCCGGGGCAACTATTGTGGTTGTTTTGTCAAG	G/A	GGGATGCATGCTCAAACTAATTTTCTCTAACCTTGAATATTTTGAATCTTGGATGTGCTTAATTGTGTTGTAGGACCTAATAACAACCCAACATAAAGCA
Ca_LG_4:47085643	AATCTTTATTTATCAACTATCAATCTATGTGCATTGTTATCTTTCAGAATTTTGTTGGATAAATCCGGGGCAACTATTGTGGTTGTTTTGTCAAGGGGAT	T/A	GCATGCTCAAACTAATTTTCTCTAACCTTGAATATTTTGAATCTTGGATGTGCTTAATTGTGTTGTAGGACCTAATAACAACCCAACATAAAGCAAAACA
Ca_LG_4:47085692	TTTTGTTGGATAAATCCGGGGCAACTATTGTGGTTGTTTTGTCAAGGGGATGCATGCTCAAACTAATTTTCTCTAACCTTGAATATTTTGAATCTTGGAT	T/C	GTGCTTAATTGTGTTGTAGGACCTAATAACAACCCAACATAAAGCAAAACACACTAAGTCACAAATACATCTTCCTCCTAATCACACGATACATTTTTAT
Ca_LG_4:47085703	AAATCCGGGGCAACTATTGTGGTTGTTTTGTCAAGGGGATGCATGCTCAAACTAATTTTCTCTAACCTTGAATATTTTGAATCTTGGATGTGCTTAATTG	G/T	TGTTGTAGGACCTAATAACAACCCAACATAAAGCAAAACACACTAAGTCACAAATACATCTTCCTCCTAATCACACGATACATTTTTATTCAAGACCATG
Ca_LG_4:47085705	ATCCGGGGCAACTATTGTGGTTGTTTTGTCAAGGGGATGCATGCTCAAACTAATTTTCTCTAACCTTGAATATTTTGAATCTTGGATGTGCTTAATTGTG	G/T	TTGTAGGACCTAATAACAACCCAACATAAAGCAAAACACACTAAGTCACAAATACATCTTCCTCCTAATCACACGATACATTTTTATTCAAGACCATGGC
Ca_LG_4:47186557	TGGACGAATCGTCGTTGCTGCCCATATTCCTCTTTACTTGGATGTCAAGACACGCCCCGTATTTAACAAGGCATGGCTGACTTATTTTAGGAGCAGTAGC	C/A	GACTGATTTTCCCCGATCCAGTTTTTTAGCTGCAACTTTCGCAGCTTTATTACCCTCCAGCTTTTGTAGTTTGCCAGCTTTATTACTCTCCAATTTTTCA

**Positions**	**-100 bp flanking sequences**	**SNPs within *kabuli* cultivar**	**+100 bp flanking sequences**
Ca_LG_4:47212634	AATTGATCCGTTGCACTTTGTGGCGGCAAAAGCCGCCACAAATTCCCATAGTTCCGTTGCACGTTGTGGCCGCCCAGGCCGCCACAAAGGACTTAATTTT	T/G	TGACCGTTAAAGCCGCCACAAAGGATTTTGTACCGTTGGCTTTTGTGGCTGCTTTGGCCGCCATAAAGAATCAACCATTTTCTATAAATATCCCTCCACT
Ca_LG_4:47315089	GGCATCCAAAGTGAAGACCAGCTGTTTGCAGCTGGATCTTTGATATCAAGGAGTGATTATGAAGATGTTGTAACCGAAAGGTCCATTACAGAAGTGTGCG	G/A	GTTATCCATTGTGCTGCAATGCTTTGCCATCTGAACGCCCTCGGAAGGGTCGATATCGAATTTCATTGAAGGAGCACAAGGTGTATGACCTGCACGAAAC
Ca_LG_4:47875466	TCATGACCCTTAAACTTGGGAGGATTGTAACGACGAAAATCTTCTAATCCTCTTAACTCTGCAGCACAGATCTGTCTTTCCCTCTTTTCTAGATCCCGTC	C/A	GAGTCTTTGCAGCAGTCTGGGTAGTAACAGAAGCAGCCATGTTATTCATAGCTTCTGCCATTTGGTCATCCCTATTGACATTGACTCTTGGAGCGGCATT
Ca_LG_4:47911395	CAGCATTCAGTTCACACCAGAATTTGTTATGGAGATATTGCAAATATGTGGTATGCATGGCTGCACTGTGCTAAATTTTTTTTCATGGGTAGGAAAGCAG	G/A	CCCGGCTACAGACACACAGCAGAATCATACAACATTGCAATCAAAATTGCAGGCCAAGGGAAAGATTTCAAGCACATGCGCAGCCTATTCTATGAAATGA
Ca_LG_4:48000234	AAATATGTACAGCAGCAACAACCTTGCAGAACTGAACAAAATAATTATTTATTTGAGTTCAGATCAGAACACTTTGTTAACCGAGCTTGTGAAATTGTAC	C/T	GATACTCCTCAATGGCTTCCTTGTAAGCATGATCCTGTTGTTTTTCCATTATGACATATTCAACCTGCAGCAGCATAGTGAGCCTTTAAAAGTATATATA
Ca_LG_4:48474897	CCTCAATACTTGCATCAACAACCACTATTCCAGCAGCTACAAAATTACTCTCAATACTCCCAGCAGCAGCAACCGCCTCTAAATTTCCAACAGCAACACC	C/G	AGGATCCTCCGAACTCTGTCTTCCAGCAACAGCAGTAGCCACCAATGTTTCAGCAACAACAATATTTTCATAATTATCAGTATGTGTTGTCCTCTTCACA
Ca_LG_4:49261571	TCCCAAAGTGACGTTACCTTTTGTGGCTGCAAAGGCCGCCACAAAAGTCTGAGTCGTTACCTTTTGTGGCTGCAAAGGCCGCCACAAACTCCAAAAGGGT	T/C	TGTTGCCATTTGTGGCCGCCACAAACGCATGCCTTTCTGACCGTTTTGACCGCCACAAAAGCCATTTATTTGTTGCTCATTGTGGCTGTTTTGGCCGCCA
Ca_LG_4:49261572	CCCAAAGTGACGTTACCTTTTGTGGCTGCAAAGGCCGCCACAAAAGTCTGAGTCGTTACCTTTTGTGGCTGCAAAGGCCGCCACAAACTCCAAAAGGGTT	T/C	GTTGCCATTTGTGGCCGCCACAAACGCATGCCTTTCTGACCGTTTTGACCGCCACAAAAGCCATTTATTTGTTGCTCATTGTGGCTGTTTTGGCCGCCAC
Ca_LG_4:49707991	TGCTTCCCACCTCTGTCTCCGACGCTGCTTCCCACCTCTGTCTCCGACGATGACGCTGCTTCTCGCCTATGTTTTTGGCATCAGCGCTGCTTCCCATCTT	T/C	TATCTCCGGCGTCGGCGCTGCATTTAAAAACCCTATTGGAGGTGGTTAGAACTGTCATAAACATTTGCAGTAGTTGGAACAACAATTTTTTTAACCGTCG
Ca_LG_4:49708002	TCTGTCTCCGACGCTGCTTCCCACCTCTGTCTCCGACGATGACGCTGCTTCTCGCCTATGTTTTTGGCATCAGCGCTGCTTCCCATCTTTATCTCCGGCG	G/A	TCGGCGCTGCATTTAAAAACCCTATTGGAGGTGGTTAGAACTGTCATAAACATTTGCAGTAGTTGGAACAACAATTTTTTTAACCGTCGCAAAATAGTCT
Ca_LG_4:49708009	CCGACGCTGCTTCCCACCTCTGTCTCCGACGATGACGCTGCTTCTCGCCTATGTTTTTGGCATCAGCGCTGCTTCCCATCTTTATCTCCGGCGTCGGCGC	C/T	TGCATTTAAAAACCCTATTGGAGGTGGTTAGAACTGTCATAAACATTTGCAGTAGTTGGAACAACAATTTTTTTAACCGTCGCAAAATAGTCTTGCGATG
Ca_LG_4:49732213	AGCAGCTATAACAGCAGCTTCAGCAGCTGCTGCTGTGGCTTCTGCAACAGCAATTGCATGATTTCTCTCTTCTGATATCAAAGGTGTTGATGTTGTGCTC	C/T	TCCTCATTTGTCACATCATCTGGTGAACTCTCTGCAGGAAAATGCTCAAATGACACCTCCTCTGCCACTTCATGCTGCAATTTCTCTCTGTTTTCCTTCT

Positions	-100 bp flanking sequences	SNPs within *kabuli* cultivar	+100 bp flanking sequences
Ca_LG_4:50462907	TCAGATTTCATCTTCGTTACCAACTTCATGTCCTCGTCCGACAATCCATCCAGCGGCTGCAAATCAGTCTTGTTGCGAGTCGAACGGACAACAACTCTTG	G/A	CGCTACTGCTAGGAGCTGCAACATGAGTCCTAGCTGCATCTACTAGCTTTCCTTGGACCTCACTCTTACCATCATCATTTACATCATCCAACCCAATTAA
Ca_LG_4:50770236	GGTCTGGAGCAGGTAGTGGCTGAAGCAAGCCTCCCAGTGCTGCAGCTATATAATTTTGGCACTGGCAAGTATCACACTCCTGGAAAAATTTCTGCACATA	A/C	TTTCTTTATCACAATCCAATAAACATTGGCTGCTAACACCTATATGTTTGGTGAAAACCTGAATGATCTCCTTCAGGAGTGGTGTGGAATTCTTTCAACA
Ca_LG_4:51162330	GGCAGAGGAGGACAAAACTAAGGCAAATGCAGCAGGTGCACTTAGCAATCTGGTTCGTAACTCTGACAAACTTTGTGAAGACATTGTGTCGAAAGGAGCG	G/T	GTTCAGGTATGCTGCATTTTATTTTAATATTGTGATATTTTTCAGTTTCAATTTAGTATTTACACAGTAAAAAATGCAATTACATCAATTTTCATATCTA
Ca_LG_4:51294972	CCGGCAGTTTGGCGTTTAAATAAGCCTGTGTTAGAGCATGGAAAGGAGATATTCTAGAAACATAACCAGATTGTCTCAATCTATAGAAAATCTTCTCTGT	T/C	ATTATGGACATCACCCCTCTTTGCATACTGCTCCATAATAGTCATATAAGTAGTAAATAATGGCTTCATCTTATTCTGCTGCATTGCCTTCTGCAAAACA
Ca_LG_4:51294999	GTGTTAGAGCATGGAAAGGAGATATTCTAGAAACATAACCAGATTGTCTCAATCTATAGAAAATCTTCTCTGTATTATGGACATCACCCCTCTTTGCATA	A/G	CTGCTCCATAATAGTCATATAAGTAGTAAATAATGGCTTCATCTTATTCTGCTGCATTGCCTTCTGCAAAACAGAGTCTGCCTTCTCAATTTCCCCTGCA
Ca_LG_4:51295019	GATATTCTAGAAACATAACCAGATTGTCTCAATCTATAGAAAATCTTCTCTGTATTATGGACATCACCCCTCTTTGCATACTGCTCCATAATAGTCATAT	T/A	AAGTAGTAAATAATGGCTTCATCTTATTCTGCTGCATTGCCTTCTGCAAAACAGAGTCTGCCTTCTCAATTTCCCCTGCATGAATATAAAGTGACACAAG
Ca_LG_4:51295025	CTAGAAACATAACCAGATTGTCTCAATCTATAGAAAATCTTCTCTGTATTATGGACATCACCCCTCTTTGCATACTGCTCCATAATAGTCATATAAGTAG	G/T	TAAATAATGGCTTCATCTTATTCTGCTGCATTGCCTTCTGCAAAACAGAGTCTGCCTTCTCAATTTCCCCTGCATGAATATAAAGTGACACAAGAGCATC
Ca_LG_4:51295030	AACATAACCAGATTGTCTCAATCTATAGAAAATCTTCTCTGTATTATGGACATCACCCCTCTTTGCATACTGCTCCATAATAGTCATATAAGTAGTAAAT	T/C	AATGGCTTCATCTTATTCTGCTGCATTGCCTTCTGCAAAACAGAGTCTGCCTTCTCAATTTCCCCTGCATGAATATAAAGTGACACAAGAGCATCCCAGG
Ca_LG_4:51295300	ATGCCTTGCATAAATGTTGAGGAGTATTGAGTAGTTCTTAGCGGAAAGCTTCCATTTATTTGACATAATCTCAAAAACTGCCTCTGCTTCTTCAATTTTG	G/C	TTTAACTTTCCCCAAGCTTCAACAGCAGCAAGGCAATCCTCTATCCGAGGAGGCCTTGACTCACAAACCTTCCAAATTCTCTCCACTTCGTCGACCCTTC
Ca_LG_4:51295303	CCTTGCATAAATGTTGAGGAGTATTGAGTAGTTCTTAGCGGAAAGCTTCCATTTATTTGACATAATCTCAAAAACTGCCTCTGCTTCTTCAATTTTGTTT	T/C	AACTTTCCCCAAGCTTCAACAGCAGCAAGGCAATCCTCTATCCGAGGAGGCCTTGACTCACAAACCTTCCAAATTCTCTCCACTTCGTCGACCCTTCCCA
Ca_LG_4:51465978	ACCTAGTGAAAATAGTAATATTAATTGATTTAAATAATAGCATAAAATGAAAAAATAAATGGAAGATTCAAATTATGTTTTGTTAGTTGTTTACATACCA	A/T	ACTGAGAATAGAAGAACAAAGATCACGGCTGCCATCATAAACTTCTGTGCCTGAGCCATTGCCTTTTTCTCTTCAGTTATTCTCTATATTTGATCAATAA
Ca_LG_4:51629260	TCAAAAACCTAAATTGATACTAAAATTACAATAAAAAGGATAATGAAAGCTGATGAAAATGGTCATATACAAAAGATGATATAGGAGCTGCTATTAAAAT	T/G	TAATTATGCTTTTGATTTCTCGGAATATAGCATTCTTAGGTTCTAAGGTCCGAAAATTTTCACCATAGATTTGTCAAAATCACAGCAAAATTACATGAGT

**Positions**	**-100 bp flanking sequences**	**SNPs within *kabuli* cultivar**	**+100 bp flanking sequences**
Ca_LG_4:51629261	CAAAAACCTAAATTGATACTAAAATTACAATAAAAAGGATAATGAAAGCTGATGAAAATGGTCATATACAAAAGATGATATAGGAGCTGCTATTAAAATT	T/C	AATTATGCTTTTGATTTCTCGGAATATAGCATTCTTAGGTTCTAAGGTCCGAAAATTTTCACCATAGATTTGTCAAAATCACAGCAAAATTACATGAGTC
Ca_LG_4:51629281	AAAATTACAATAAAAAGGATAATGAAAGCTGATGAAAATGGTCATATACAAAAGATGATATAGGAGCTGCTATTAAAATTAATTATGCTTTTGATTTCTC	C/T	GGAATATAGCATTCTTAGGTTCTAAGGTCCGAAAATTTTCACCATAGATTTGTCAAAATCACAGCAAAATTACATGAGTCAAAATTTTCATGTATTACAT
Ca_LG_4:51697856	CCACTACAATTAAGTATATATAGTTCAACCTAATAAGACTCATTAATTAAAAACAGATAAAAATTAATTCATTATTTATGTTATTTTATAGCTGCCACAA	A/G	AAGAATCAATTAGCAATTGCAACTGAAAGCCAACAAGTTGAAGCTCTTCCTGCCAATGGTACATATCTATGTGTGCTTAGTTATTTTCCTGACTATTGTT
Ca_LG_4:52237259	CAAGAACTAGTACATTCAAAGAATAGTTGAAATATTCTCCTTTAGCTTCAAGATTGAAAGCAGGCTTGTTTGCACCATCAAGATTGAAAATTTGACTTTG	G/A	ATGATCAGTAACAATCGTCAATCTGCCAAGCAATTGGGCTGCAATTGATTCTTGTTAGTCAAACCAAGCCTTAGAAGTAGACTTCAGAATTTGATTTGTC
Ca_LG_4:52237273	TTCAAAGAATAGTTGAAATATTCTCCTTTAGCTTCAAGATTGAAAGCAGGCTTGTTTGCACCATCAAGATTGAAAATTTGACTTTGATGATCAGTAACAA	A/T	TCGTCAATCTGCCAAGCAATTGGGCTGCAATTGATTCTTGTTAGTCAAACCAAGCCTTAGAAGTAGACTTCAGAATTTGATTTGTCACAATTTCTCAAAT
Ca_LG_4:52237275	CAAAGAATAGTTGAAATATTCTCCTTTAGCTTCAAGATTGAAAGCAGGCTTGTTTGCACCATCAAGATTGAAAATTTGACTTTGATGATCAGTAACAATC	C/G	GTCAATCTGCCAAGCAATTGGGCTGCAATTGATTCTTGTTAGTCAAACCAAGCCTTAGAAGTAGACTTCAGAATTTGATTTGTCACAATTTCTCAAATTT
Ca_LG_4:52237284	GTTGAAATATTCTCCTTTAGCTTCAAGATTGAAAGCAGGCTTGTTTGCACCATCAAGATTGAAAATTTGACTTTGATGATCAGTAACAATCGTCAATCTG	G/A	CCAAGCAATTGGGCTGCAATTGATTCTTGTTAGTCAAACCAAGCCTTAGAAGTAGACTTCAGAATTTGATTTGTCACAATTTCTCAAATTTCAAATTTTG
Ca_LG_4:52237292	ATTCTCCTTTAGCTTCAAGATTGAAAGCAGGCTTGTTTGCACCATCAAGATTGAAAATTTGACTTTGATGATCAGTAACAATCGTCAATCTGCCAAGCAA	A/C	TTGGGCTGCAATTGATTCTTGTTAGTCAAACCAAGCCTTAGAAGTAGACTTCAGAATTTGATTTGTCACAATTTCTCAAATTTCAAATTTTGTTCTTCAT
Ca_LG_4:52255582	GAAATCTGTGCAGCATTTGAGGAATTAGAGAAACAGAAAGCTGGTGGTATGACAGATGACACTGATGATTCCCATTTTGGTTCAGAGGCCCCTTCTTTTT	T/G	ATGGGGCAGCGAGCAGCATAAAGGATGCTACTGATGTTGTGGTATTAAATGCAGAAAAGGCTAAGACTATTATGGAAGATGTTGGCTCAAATTTGGATCA
Ca_LG_4:52302368	TTTTACGCAGATCGGAAAAATGCAGCAGCACAGACGCGTATGGTTCGGGGGCAGCAGATGGAAAATTCTGATGTTGGGGACGGTGCATTGGAGTACTCCA	A/C	GTGTAATATTGATGCAGCATTTTTTCAACAAAGAGCTACAACTATTGGTATGTGACTGTGTGATCAGCAAGAACGGACTACATTCCATTCCAAAGAGCTA
Ca_LG_4:52666975	CAATTGTCGTATGTGTTCCTACAGTAATGTATACAGCAATATTTTGAACCTTTGAATTAAAGAAATGACTTAAAGATTTTAAATGATAAAAAGCAGTTCA	A/T	ATATGAACGAAGGGAAGTAGGGCAGCGCCAAAAAATTGCTAAAATGAGATGTTGTAACAAGTTTTGCAGCACAAAAAGCTTGCTGTGGAAGCAGATTTGA
Ca_LG_4:52666989	GTTCCTACAGTAATGTATACAGCAATATTTTGAACCTTTGAATTAAAGAAATGACTTAAAGATTTTAAATGATAAAAAGCAGTTCAATATGAACGAAGGG	G/C	AAGTAGGGCAGCGCCAAAAAATTGCTAAAATGAGATGTTGTAACAAGTTTTGCAGCACAAAAAGCTTGCTGTGGAAGCAGATTTGAAGTAGATAAATAAA

Positions	-100 bp flanking sequences	SNPs within *kabuli* cultivar	+100 bp flanking sequences
Ca_LG_4:52666993	CTACAGTAATGTATACAGCAATATTTTGAACCTTTGAATTAAAGAAATGACTTAAAGATTTTAAATGATAAAAAGCAGTTCAATATGAACGAAGGGAAGT	T/A	AGGGCAGCGCCAAAAAATTGCTAAAATGAGATGTTGTAACAAGTTTTGCAGCACAAAAAGCTTGCTGTGGAAGCAGATTTGAAGTAGATAAATAAACCCC
Ca_LG_4:53291168	GCAACAGTCATTTGGGCATTTGTGGCGGCCTTTGCCACCACAAAATGTAACGGATATGTGAATTTGTGACGGCCTGGGCGGCCAGAAAATCAGTTTCACC	C/A	TTTAATCCTTTGTGGCGGCTTTTGCAGCCACAAAAGGTAACGGTCATAGGCTTTTGTGGTGGCCTATATAGCCACAAATTGACATGCGTTTTCAGTCATT
Ca_LG_4:53540195	CAAAGGTTTGTATGAATTCTTTGGTAGCTCAACACATGTACTCTTTACCTTCTTATGCATTTATAGTGCAAGATTTTACTACTCAAGTTGTGTTATATAT	T/C	TCATCATCAATACATTGCTGGATTTATTATGAAGGGTCTTTTGCTCATGGAGCGATATTTTTTTATTAGAGATTACAATCCCGAACAGAATGAAGATAAT
Ca_LG_4:53540217	GGTAGCTCAACACATGTACTCTTTACCTTCTTATGCATTTATAGTGCAAGATTTTACTACTCAAGTTGTGTTATATATTCATCATCAATACATTGCTGGA	A/G	TTTATTATGAAGGGTCTTTTGCTCATGGAGCGATATTTTTTTATTAGAGATTACAATCCCGAACAGAATGAAGATAATGTATTCGCAAGAATGTTAGATC
Ca_LG_4:53540232	GTACTCTTTACCTTCTTATGCATTTATAGTGCAAGATTTTACTACTCAAGTTGTGTTATATATTCATCATCAATACATTGCTGGATTTATTATGAAGGGT	T/G	CTTTTGCTCATGGAGCGATATTTTTTTATTAGAGATTACAATCCCGAACAGAATGAAGATAATGTATTCGCAAGAATGTTAGATCACAAAGAAGCTATCT
Ca_LG_4:53785677	AATTAGATAAACCTTGCGAATAAAACCCCATCGGAGCTCAGGTGATTCAATCATATATGGATAAAGGCTGCCATGTGCTTGGGGAAACCCTGCCTCAATA	A/G	TCACCCTTACCCATGGCGCCGCACGGAAACAAACAACCTCAAACACAATTAATTAGATGTTTAATTATGTCTCAATTCAATTTGTTTTTTTGTTATTGTT
Ca_LG_4:53785704	CCATCGGAGCTCAGGTGATTCAATCATATATGGATAAAGGCTGCCATGTGCTTGGGGAAACCCTGCCTCAATATCACCCTTACCCATGGCGCCGCACGGA	A/T	AACAAACAACCTCAAACACAATTAATTAGATGTTTAATTATGTCTCAATTCAATTTGTTTTTTTGTTATTGTTGTTGCAAATTGTAACTATTTGTCTTGG
Ca_LG_4:54016880	TAGGAACCCACACCCCAGACAAGGCAGAGTTAGTGAGCCTTGTAATAGGCCACCATTTCACACGGTTCGGGGGAACGTGAGTCAGCCGCCGGCGCCCGAT	T/C	TGCGTCTTGACCTCTATCCAATTTTTGGGCCAATTCCCCCTTCGTACTACCAAAAATTGAGTCCAAAAATTAAAACAATCGGATAATATAATTTGAGAGT
Ca_LG_4:54016893	CCCAGACAAGGCAGAGTTAGTGAGCCTTGTAATAGGCCACCATTTCACACGGTTCGGGGGAACGTGAGTCAGCCGCCGGCGCCCGATTGCGTCTTGACCT	T/C	CTATCCAATTTTTGGGCCAATTCCCCCTTCGTACTACCAAAAATTGAGTCCAAAAATTAAAACAATCGGATAATATAATTTGAGAGTGAGGTATTTTGGT
Ca_LG_4:54111380	TGAGGGATCTGTAACCATGTCAAAATGTTTCAAACTGCAAGCATCATCGTTGCAACTTGCAGGAGCATTTTCATTCTTCAAGCTGCACAGTGTCTCGGAG	G/T	GCTTCCACATCGGATCCTTCTGGAAGAAAGGCTTCCTCATTTTTACAACTCTTGCTGCCCGAATTGTCACCATCAATGGCAACACATTCCCGAGAACTGG
Ca_LG_4:54728337	GTACCAGCTGCTTTGAATAACTCCTTCCAGAAATGGCTTAAAAAAATAGGATCCCTATCAGAGACAATAGTTGAAGGGAAACCATGAAGTTTAACAACTC	C/T	CTGAAATGAAGACATCAGCAACTTCCTTAGCTAAAAATGGGTGGCCCAATGCAACAAAATGGGCGTACTTAGATAGCATGTCTACTACTACTAGAATAGT
Ca_LG_4:54839408	ATCTTTCACAAGTGTGTTGTCACCACTTGCTACCCATTTTCCAAGCTGCATGGACTCATTGATTGATGATTTCTCATATGCCTTTGCTGCTGATACTAGA	A/T	GGTTGAATGTCAATTTCAGCCTCACCCATAAAATCATCCGTTTTGAATGAATCCTTGTCATATACAATCTGCATTTAAATATCACATCAAATCTGATCAA

Positions	-100 bp flanking sequences	SNPs within *kabuli* cultivar	+100 bp flanking sequences
Ca_LG_4:54839420	TGTGTTGTCACCACTTGCTACCCATTTTCCAAGCTGCATGGACTCATTGATTGATGATTTCTCATATGCCTTTGCTGCTGATACTAGAGGTTGAATGTCA	A/T	ATTTCAGCCTCACCCATAAAATCATCCGTTTTGAATGAATCCTTGTCATATACAATCTGCATTTAAATATCACATCAAATCTGATCAAACATGAAAGGCA
Ca_LG_5:219198	TTATAAGTAATTAATTAATTTATTTAAATGTAATAATAATAACAACGGCAGCTGCTGCTGCAATAACAACAACAAGAACAACAACAACAATATCACCAGC	C/G	ATGTATCTAGTTTTTTGCAATGAGAATCTACGTGGACTGTATAAGTGTTATTGTTATTTTTTGAATGGACTATATCTTCAAACTTAAAAGTGAGTTATTA
Ca_LG_5:330349	AATCGCCCTTCACCAGCATTGAATCAACTGCTTCGTATGCTTCTTCCAGTGTCATGGCAACAGTAACTCCTTTTCCAGCAGCCAGTCCATCTGCTTTGAT	T/C	GACAATCGGTGCTCCTTCTTCTTGAATATATTGCTTGGCTGCAATCGGGTCTGTAAACGTTTTGTACTGCACTAATAAGTCCATTATAAGTAAACACAAC
Ca_LG_5:330356	CTTCACCAGCATTGAATCAACTGCTTCGTATGCTTCTTCCAGTGTCATGGCAACAGTAACTCCTTTTCCAGCAGCCAGTCCATCTGCTTTGATGACAATC	C/T	GGTGCTCCTTCTTCTTGAATATATTGCTTGGCTGCAATCGGGTCTGTAAACGTTTTGTACTGCACTAATAAGTCCATTATAAGTAAACACAACACAAAAA
Ca_LG_5:330977	TGTCACACAAATGCTTCATGAAATTCTTAGAACCTTCAAGAGCAGCAGCCTCTGCAGATGGACCAAAAGTTGGAATTCCAGCCTTAACTAAATAATTTGA	A/C	AAGACCTGCAACAAGTGGGGCCTCAGGTCCAACAACAACTAGTCCCACCCCCCATTTCCGACAAAATGAAACTACTGCTGCTCCATCATAAACATCAAGG
Ca_LG_5:330987	ATGCTTCATGAAATTCTTAGAACCTTCAAGAGCAGCAGCCTCTGCAGATGGACCAAAAGTTGGAATTCCAGCCTTAACTAAATAATTTGAAAGACCTGCA	A/C	ACAAGTGGGGCCTCAGGTCCAACAACAACTAGTCCCACCCCCCATTTCCGACAAAATGAAACTACTGCTGCTCCATCATAAACATCAAGGTCTGAGATGC
Ca_LG_5:330996	GAAATTCTTAGAACCTTCAAGAGCAGCAGCCTCTGCAGATGGACCAAAAGTTGGAATTCCAGCCTTAACTAAATAATTTGAAAGACCTGCAACAAGTGGG	G/A	GCCTCAGGTCCAACAACAACTAGTCCCACCCCCCATTTCCGACAAAATGAAACTACTGCTGCTCCATCATAAACATCAAGGTCTGAGATGCACGTTGCAT
Ca_LG_5:331010	CTTCAAGAGCAGCAGCCTCTGCAGATGGACCAAAAGTTGGAATTCCAGCCTTAACTAAATAATTTGAAAGACCTGCAACAAGTGGGGCCTCAGGTCCAAC	C/T	AACAACTAGTCCCACCCCCCATTTCCGACAAAATGAAACTACTGCTGCTCCATCATAAACATCAAGGTCTGAGATGCACGTTGCATTTCCTGAGCTTGCA
Ca_LG_5:779392	CTACTTGGCTACCTCATACATTGCAATAAGCATTTTTGTGAGTTTCAGTTTTGCTATGGTATTGTGGTGGCTGCCCTTAAGATGTTGAGCACCGTTGCTG	G/A	CTGGATTAGCTATTGATGTCTGGGGGCCCATCAGTGACGATGCAGGAGGATTTGCTGAGTGAACTGATGCCCTTGATGCTGCTGACAACACTACTGCCGC
Ca_LG_5:980103	TTATGTGCTGATGTGGTCGAAAGTTATTCTTAGAATTATTGAAGTAGTGTTAAGACTCTACTTGATGATGGTTTTATAGGGAAACCATGCTGCCAAGGAC	C/G	GAATGACGTTCCAAGAGCCAATGTCAATAGGGATGACCGAATGGCGGAGGCTATGAATAACCTGGCTGCTTCTGTTGCTGCACAGACTGCTGCCAAGACT
Ca_LG_5:980140	ATTGAAGTAGTGTTAAGACTCTACTTGATGATGGTTTTATAGGGAAACCATGCTGCCAAGGACGAATGACGTTCCAAGAGCCAATGTCAATAGGGATGAC	C/T	CGAATGGCGGAGGCTATGAATAACCTGGCTGCTTCTGTTGCTGCACAGACTGCTGCCAAGACTCAACGTGATCTCGAAAAGAGGGGAAGAGAGATCCGTG
Ca_LG_5:1183176	GATGGATATTAGCTCAAACATCAGATATGGATGTACCCGAGACGTAACGCAGGAGGATATTGAGTGGGCTGCGAAGCAAGCCTATGCTCATGACTTTATC	C/T	TCAGCACTTCCTAATGGCTATGAAACACTTGTGGATGATGATCTTTTAAGTGGGGGACAAAAGCAACGAATTGCTATTGCTAGGGCCATTCTTAGGGACC

Positions	-100 bp flanking sequences	SNPs within *kabuli* cultivar	+100 bp flanking sequences
Ca_LG_5:1183179	GGATATTAGCTCAAACATCAGATATGGATGTACCCGAGACGTAACGCAGGAGGATATTGAGTGGGCTGCGAAGCAAGCCTATGCTCATGACTTTATCTCA	A/T	GCACTTCCTAATGGCTATGAAACACTTGTGGATGATGATCTTTTAAGTGGGGGACAAAAGCAACGAATTGCTATTGCTAGGGCCATTCTTAGGGACCCGA
Ca_LG_5:1183180	GATATTAGCTCAAACATCAGATATGGATGTACCCGAGACGTAACGCAGGAGGATATTGAGTGGGCTGCGAAGCAAGCCTATGCTCATGACTTTATCTCAG	G/T	CACTTCCTAATGGCTATGAAACACTTGTGGATGATGATCTTTTAAGTGGGGGACAAAAGCAACGAATTGCTATTGCTAGGGCCATTCTTAGGGACCCGAA
Ca_LG_5:1183188	CTCAAACATCAGATATGGATGTACCCGAGACGTAACGCAGGAGGATATTGAGTGGGCTGCGAAGCAAGCCTATGCTCATGACTTTATCTCAGCACTTCCT	T/A	AATGGCTATGAAACACTTGTGGATGATGATCTTTTAAGTGGGGGACAAAAGCAACGAATTGCTATTGCTAGGGCCATTCTTAGGGACCCGAAAATATTGA
Ca_LG_5:1183203	TGGATGTACCCGAGACGTAACGCAGGAGGATATTGAGTGGGCTGCGAAGCAAGCCTATGCTCATGACTTTATCTCAGCACTTCCTAATGGCTATGAAACA	A/C	CTTGTGGATGATGATCTTTTAAGTGGGGGACAAAAGCAACGAATTGCTATTGCTAGGGCCATTCTTAGGGACCCGAAAATATTGATCCTTGATGAAGCCA
Ca_LG_5:1698093	CGTGTAATGTTGCATAAATTGTATTTCTTTTTTATATAACATAATTCTATAATATAATGTTTGTACCTCTGTTGCTGCAGATGTTCAAAGTACAGGAACG	G/A	TTCCATAACAGTAATGGACCAAGGATTATAGTTGGTATCATAAATGATAACAAGCAAAACATAGATGTCTTAAATAGTAGCTTTCGCGTTGTGGCTTCAG
Ca_LG_5:1787249	GTGGCCTGTTCGCTGCCTTTATAGAGGCGGCCGAGCTAAGTTAAGCCAAGGATGGTATGAATTTACATTAGAGAACAATTTAGGCGAAGGTGATGTTTGC	C/T	GTTTTCGAGCTGCTTAGAACCAGAGACGTGGTGCTGCAAGTTACTTTATTCCGCGTAGCTGAGGATGAATCAGGGTTATTCAACCCTTCGATGCAGCCGA
Ca_LG_5:1787255	TGTTCGCTGCCTTTATAGAGGCGGCCGAGCTAAGTTAAGCCAAGGATGGTATGAATTTACATTAGAGAACAATTTAGGCGAAGGTGATGTTTGCGTTTTC	C/T	GAGCTGCTTAGAACCAGAGACGTGGTGCTGCAAGTTACTTTATTCCGCGTAGCTGAGGATGAATCAGGGTTATTCAACCCTTCGATGCAGCCGAACCAAA
Ca_LG_5:1897496	TTAAAAACTCTAAAAAGAAAATTCACCTTGGTAGTTGGAACTTACCTTTGTAGAAACTGGAATGATTTCTTCTTTGAATTTACCAGCTGCTGTTGCAGCT	T/C	GCGGCGCGCCTGTGAGACTCGACCTAATTACATATTAAAAAGAGTTAATTCACCCTGATAATCTCTTTATAGATTCTCATAGTAAATTAAATGCATATTT
Ca_LG_5:1897502	ACTCTAAAAAGAAAATTCACCTTGGTAGTTGGAACTTACCTTTGTAGAAACTGGAATGATTTCTTCTTTGAATTTACCAGCTGCTGTTGCAGCTGCGGCG	G/A	CGCCTGTGAGACTCGACCTAATTACATATTAAAAAGAGTTAATTCACCCTGATAATCTCTTTATAGATTCTCATAGTAAATTAAATGCATATTTCATGCT
Ca_LG_5:1897517	TTCACCTTGGTAGTTGGAACTTACCTTTGTAGAAACTGGAATGATTTCTTCTTTGAATTTACCAGCTGCTGTTGCAGCTGCGGCGCGCCTGTGAGACTCG	G/A	ACCTAATTACATATTAAAAAGAGTTAATTCACCCTGATAATCTCTTTATAGATTCTCATAGTAAATTAAATGCATATTTCATGCTTACAGCAGCCTGATC
Ca_LG_5:1897557	ATGATTTCTTCTTTGAATTTACCAGCTGCTGTTGCAGCTGCGGCGCGCCTGTGAGACTCGACCTAATTACATATTAAAAAGAGTTAATTCACCCTGATAA	A/T	TCTCTTTATAGATTCTCATAGTAAATTAAATGCATATTTCATGCTTACAGCAGCCTGATCTTGCTCCTGCCTTGTGACGCCATAACGCTCAGCAACGTTT
Ca_LG_5:1897564	CTTCTTTGAATTTACCAGCTGCTGTTGCAGCTGCGGCGCGCCTGTGAGACTCGACCTAATTACATATTAAAAAGAGTTAATTCACCCTGATAATCTCTTT	T/C	ATAGATTCTCATAGTAAATTAAATGCATATTTCATGCTTACAGCAGCCTGATCTTGCTCCTGCCTTGTGACGCCATAACGCTCAGCAACGTTTTCAGAAG

Positions	-100 bp flanking sequences	SNPs within *kabuli* cultivar	+100 bp flanking sequences
Ca_LG_5:1897577	ACCAGCTGCTGTTGCAGCTGCGGCGCCTGTGAGACTCGACCTAATTACATATTAAAAAGAGTTAATTCACCCTGATAATCTCTTTATAGATTCTCATA	A/T	GTAAATTAAATGCATATTTCATGCTTACAGCAGCCTGATCTTGCTCCTGCCTTGTGACGCCATAACGCTCAGCAACGTTTTCAGAAGTGATTCCCATAGG
Ca_LG_5:1945041	AAAATGTAACGGTCTAATTGTTTGTGGCAGCCTAGGCGGCCAGAAATGCCAATGTCATTGTGTTTCCTTTGTGGCGGCCTATGCAGCCATAAATGCCAAT	T/C	GTTCACTTGCTTTTGTGGTGGCCTATGCAGCCACAAATGGACATCTTACTTCAATAATTTGTGACGGCTTTGCCCCTCACAAAATTTATTTTTTGTGGCT
Ca_LG_5:2383097	GTCCATTGTCCGCTGTGAAATAATAATTTTAGAATTAGTCCAATATATTCAAAATAGTAAAAAAAAACAAAAGGATTTATAGCTAACAAAAGAAATGCAC	C/T	AACATTCTAAAACATACCACAATTTGTGTCTAAAACATACAGCTTATCCAAAGTCAAGTGGTGCTCTACAGATCTTAGTGCTGCAATTGAAGAAATTATT
Ca_LG_5:2383126	TAGAATTAGTCCAATATATTCAAAATAGTAAAAAAAAACAAAAGGATTTATAGCTAACAAAAGAAATGCACAACATTCTAAAACATACCACAATTTGTGT	T/C	CTAAAACATACAGCTTATCCAAAGTCAAGTGGTGCTCTACAGATCTTAGTGCTGCAATTGAAGAAATTATTTTGCATTACAACATAACGAGTCGCAGTGG
Ca_LG_5:2383137	CAATATATTCAAAATAGTAAAAAAAAACAAAAGGATTTATAGCTAACAAAAGAAATGCACAACATTCTAAAACATACCACAATTTGTGTCTAAAACATAC	C/G	AGCTTATCCAAAGTCAAGTGGTGCTCTACAGATCTTAGTGCTGCAATTGAAGAAATTATTTTGCATTACAACATAACGAGTCGCAGTGGAGAAAGTAACA
Ca_LG_5:2383150	ATAGTAAAAAAAAACAAAAGGATTTATAGCTAACAAAAGAAATGCACAACATTCTAAAACATACCACAATTTGTGTCTAAAACATACAGCTTATCCAAAG	G/T	TCAAGTGGTGCTCTACAGATCTTAGTGCTGCAATTGAAGAAATTATTTTGCATTACAACATAACGAGTCGCAGTGGAGAAAGTAACAGCAAACTAGCAAA
Ca_LG_5:2383198	ACATTCTAAAACATACCACAATTTGTGTCTAAAACATACAGCTTATCCAAAGTCAAGTGGTGCTCTACAGATCTTAGTGCTGCAATTGAAGAAATTATTT	T/C	TGCATTACAACATAACGAGTCGCAGTGGAGAAAGTAACAGCAAACTAGCAAAGAATATATCTTTACAGTAGTCATACCTTGCACCTGGCAACCGACGCCA
Ca_LG_5:2605987	GTCATGATGTTATGGTGTAGCAGATTCTTTGCCCAATTGCTAGAGGCTGCAACAGCTATTATATCAGCCACGTTTCCCCCATAGCAACACCATGTTTATG	G/A	TGGTGTATTTTTGGCTTGCTGCCATTGTCCTTCATCTGCCACTGATAACATTTGAGAGAAGTGATAAATTGAGATGTAGAGACAAGCTTTCAAAGCACAT
Ca_LG_5:2605992	GATGTTATGGTGTAGCAGATTCTTTGCCCAATTGCTAGAGGCTGCAACAGCTATTATATCAGCCACGTTTCCCCCATAGCAACACCATGTTTATGTGGTG	G/C	TATTTTTGGCTTGCTGCCATTGTCCTTCATCTGCCACTGATAACATTTGAGAGAAGTGATAAATTGAGATGTAGAGACAAGCTTTCAAAGCACATGAATT
Ca_LG_5:2691950	TGGCTGCAAATAATTTGGACAAAAACATGAAAAGAATTTGGCTGCAAATAGTTTGGACAAAAACACTCATTTGTCCAAATAATTTGGCTGCAATAACATT	T/G	TTATCTATACTTTGGCTGCAATAATATGGCTGCAATAATTTGGTTTCAAATAATTTGGCAGCAAAAACAAAAAATGGTAATTATGGATCTCAAATTGAAA
Ca_LG_5:3207789	TCCATGTTGTTTTTAAACAGGTTGCTACCTTAGCGCCTAATGTCCCTGCAATGTATGAGCTGCATTTTGCAGTACCAATGGCCGGAGCCATTCTTTGTAA	A/C	TCTTAATTCTCGCTTAGATGCAGCTATGGTATCAGTCCTGCTGGAGCATTCACAAGCTAAGATTCTTTTTGTAGACTATCAACTACTTGAAATTGCTCAG
Ca_LG_5:3207803	AAACAGGTTGCTACCTTAGCGCCTAATGTCCCTGCAATGTATGAGCTGCATTTTGCAGTACCAATGGCCGGAGCCATTCTTTGTAATCTTAATTCTCGCT	T/C	TAGATGCAGCTATGGTATCAGTCCTGCTGGAGCATTCACAAGCTAAGATTCTTTTTGTAGACTATCAACTACTTGAAATTGCTCAGGGAGCATTGAACTT

Positions	-100 bp flanking sequences	SNPs within *kabuli* cultivar	+100 bp flanking sequences
Ca_LG_5:3229986	TTTTGGGCTCATGGTGTTATTCATGCAGTTTTTCTTATTAATAGGCTGCCATCTAAATTTTTAAATTTTTTTTCTCCTTATCACATATTGCATGATTCTA	A/G	CTCCTGACATTTCCAACTTGAAGGTTTTTGGCTGCCTATGTTTTTCTACAACTTTGCAATCGTATGTTATCAAGGTGATGTTTTTCGAATCTTAGATTTA
Ca_LG_5:3500868	AGTGACCCCAGAAGCCATATTGGATAGCAGAGAGGACAAGCAGGGTTACTTAGAGGTGCTAGTTAAATGGAAAGATCTTCCTCAGCATGAGTGTACCTGG	G/A	GAATTGGCANNNNNNNNNNNNNNNNNNNNNNNNNNNNNNNNNNNNNNNNNNNNNNNNNNNNNNNNNNNNNNNNNNNNNNNNNNNNNNNNNNNNNN
Ca_LG_5:3737739	CAATGGTTCAAAACCCATCTTTTTTTCTATTGTCAGGGAGATGGAGAATTTGTTAGCAGCAGATATTAAACTCTCTTAATACTTTCAACTCATATCACAG	G/T	TTATCAATATCTTTGGATCAATTAGTTGAAACAATGATGAAGCTAGAAAAGTCAACGCAAGAAGCCTATGTTTTTTTTGCATATTTTTCTAACATGCAAC
Ca_LG_5:4563026	TCATAATTCAAATAACATAACCTAATTTCTTAATACACTTCCCAAAAAAAATAGACTTTCTAGATTCTAAAATAGCTGCAATTTACATCAGACTCAACAG	G/A	ACGTTACAAAAAATGGTTCTCGATCAAAGAGGCCTACTAAAAAGAAATATGTTGAGACTTGAATCTAATAACAAATTTCTACTCAACTGCCTGCGAATCT
Ca_LG_5:4563039	AACATAACCTAATTTCTTAATACACTTCCCAAAAAAAATAGACTTTCTAGATTCTAAAATAGCTGCAATTTACATCAGACTCAACAGACGTTACAAAAAA	A/T	TGGTTCTCGATCAAAGAGGCCTACTAAAAAGAAATATGTTGAGACTTGAATCTAATAACAAATTTCTACTCAACTGCCTGCGAATCTGAAAAAATAAACA
Ca_LG_5:4563041	CATAACCTAATTTCTTAATACACTTCCCAAAAAAAATAGACTTTCTAGATTCTAAAATAGCTGCAATTTACATCAGACTCAACAGACGTTACAAAAAATG	G/A	GTTCTCGATCAAAGAGGCCTACTAAAAAGAAATATGTTGAGACTTGAATCTAATAACAAATTTCTACTCAACTGCCTGCGAATCTGAAAAAATAAACAAC
Ca_LG_5:4630640	CGATTGGAGATATGATCATCATCGTAGGCTGTTTCCTCGCCATCTTCGTCAGCTTGGGTAGCATCATTGGAGGGGCCAGCAGTGTCCTCTTGAGCGGCAC	C/G	CAGATGATTGACCAAGGTCACTTTTATGCACATACGAGTGAAGCTGCTCATTCCTGACGATCTTCATGCTAGGCAGGACGCTTAAATCCAACACGTTGTC
Ca_LG_5:4630656	CATCATCGTAGGCTGTTTCCTCGCCATCTTCGTCAGCTTGGGTAGCATCATTGGAGGGGCCAGCAGTGTCCTCTTGAGCGGCACCAGATGATTGACCAAG	G/T	GTCACTTTTATGCACATACGAGTGAAGCTGCTCATTCCTGACGATCTTCATGCTAGGCAGGACGCTTAAATCCAACACGTTGTCCTTCGTGGGTGTATAT
Ca_LG_5:4630669	TGTTTCCTCGCCATCTTCGTCAGCTTGGGTAGCATCATTGGAGGGGCCAGCAGTGTCCTCTTGAGCGGCACCAGATGATTGACCAAGGTCACTTTTATGC	C/T	ACATACGAGTGAAGCTGCTCATTCCTGACGATCTTCATGCTAGGCAGGACGCTTAAATCCAACACGTTGTCCTTCGTGGGTGTATATATTACTTGTTCAG
Ca_LG_5:4703781	TTCTTTTGTGATCAGTCTTATTGAACAAAGACTTACCACGGTAAGGAAGAAAGGGGAACAAGCACACTTGGAGAGCGCATTATAATGGATAGTTGTATGT	T/C	TGCGTTTGGGAAGGATGAATCGCTCCCGAAAAGGAATCTATTGATTCTCTCCCAATTGGATGGGCCGTAGGTGCGATGATTTACTTCACGGGCGAGGTCT
Ca_LG_5:4703788	GTGATCAGTCTTATTGAACAAAGACTTACCACGGTAAGGAAGAAAGGGGAACAAGCACACTTGGAGAGCGCATTATAATGGATAGTTGTATGTTGCGTTT	T/C	GGGAAGGATGAATCGCTCCCGAAAAGGAATCTATTGATTCTCTCCCAATTGGATGGGCCGTAGGTGCGATGATTTACTTCACGGGCGAGGTCTTTGGTTC
Ca_LG_5:4703845	CACTTGGAGAGCGCATTATAATGGATAGTTGTATGTTGCGTTTGGGAAGGATGAATCGCTCCCGAAAAGGAATCTATTGATTCTCTCCCAATTGGATGGG	G/A	CCGTAGGTGCGATGATTTACTTCACGGGCGAGGTCTTTGGTTCAAATCTAGGATGGCCCAACTGCGCCAAGGAAAAGAATAAAAGACTGATTTGACTCCT

**Positions**	**-100 bp flanking sequences**	**SNPs within *kabuli* cultivar**	**+100 bp flanking sequences**
Ca_LG_5:4703882	GCGTTTGGGAAGGATGAATCGCTCCCGAAAAGGAATCTATTGATTCTCTCCCAATTGGATGGGCCGTAGGTGCGATGATTTACTTCACGGGCGAGGTCTT	T/C	TGGTTCAAATCTAGGATGGCCCAACTGCGCCAAGGAAAAGAATAAAAGACTGATTTGACTCCTTCATGCATGCTCCAACTTGGCTCGGGGGATGTAGCTC
Ca_LG_5:4703894	GATGAATCGCTCCCGAAAAGGAATCTATTGATTCTCTCCCAATTGGATGGGCCGTAGGTGCGATGATTTACTTCACGGGCGAGGTCTTTGGTTCAAATCT	T/C	AGGATGGCCCAACTGCGCCAAGGAAAAGAATAAAAGACTGATTTGACTCCTTCATGCATGCTCCAACTTGGCTCGGGGGATGTAGCTCAGTTGGTAGAGC
Ca_LG_5:4703906	CCGAAAAGGAATCTATTGATTCTCTCCCAATTGGATGGGCCGTAGGTGCGATGATTTACTTCACGGGCGAGGTCTTTGGTTCAAATCTAGGATGGCCCAA	A/G	CTGCGCCAAGGAAAAGAATAAAAGACTGATTTGACTCCTTCATGCATGCTCCAACTTGGCTCGGGGGATGTAGCTCAGTTGGTAGAGCTCCGCTCTTGCA
Ca_LG_5:4766778	GTTAAACCTCCGATCTTGTGATGCTCTAACCAACTGATTGAGCTAAAGGGTCGACCCTATTTGCTTTTTATATACCTAATGGCATCCCTTATAGCGGCGG	G/A	CAACAACAACGATGGTTTGGGTGAAAAATCAGAAGTAGTCAACAACGCCGACAATATTGGTTTAGGCAGTGGCGGCCGAAATGGTTTTTCCAATTTTTTT
Ca_LG_5:4766789	GATCTTGTGATGCTCTAACCAACTGATTGAGCTAAAGGGTCGACCCTATTTGCTTTTTATATACCTAATGGCATCCCTTATAGCGGCGGCAACAACAACG	G/A	ATGGTTTGGGTGAAAAATCAGAAGTAGTCAACAACGCCGACAATATTGGTTTAGGCAGTGGCGGCCGAAATGGTTTTTCCAATTTTTTTTGGCCATGAGG
Ca_LG_5:4766825	GGGTCGACCCTATTTGCTTTTTATATACCTAATGGCATCCCTTATAGCGGCGGCAACAACAACGATGGTTTGGGTGAAAAATCAGAAGTAGTCAACAACG	G/A	CCGACAATATTGGTTTAGGCAGTGGCGGCCGAAATGGTTTTTCCAATTTTTTTTGGCCATGAGGGAACTTCTAATGCCGTCAATGTTGAAATCTAGAAAC
Ca_LG_5:4961875	ATTGCAAAGCAGAAAAAGTAAAAACATGAAAAGAAACAAACCAAAGATCATGACAAAAAATTCTATAGATAAAACCGGTTTTCCTATGCCAAATTTCTCA	A/C	ACTTAAGTGCTAATTGAGAAAGAGTCTTTATAGAAGACCCATCTTCTTTGATTGCATTATTAGCAGCTTCTTTCAATTCCTTCATTCGGAGAGAGGCACG
Ca_LG_5:4961880	AAAGCAGAAAAAGTAAAAACATGAAAAGAAACAAACCAAAGATCATGACAAAAAATTCTATAGATAAAACCGGTTTTCCTATGCCAAATTTCTCAACTTA	A/G	AGTGCTAATTGAGAAAGAGTCTTTATAGAAGACCCATCTTCTTTGATTGCATTATTAGCAGCTTCTTTCAATTCCTTCATTCGGAGAGAGGCACGAAGAA
Ca_LG_5:4961897	AACATGAAAAGAAACAAACCAAAGATCATGACAAAAAATTCTATAGATAAAACCGGTTTTCCTATGCCAAATTTCTCAACTTAAGTGCTAATTGAGAAAG	G/T	AGTCTTTATAGAAGACCCATCTTCTTTGATTGCATTATTAGCAGCTTCTTTCAATTCCTTCATTCGGAGAGAGGCACGAAGAATTATCGTCACCGGAGAG
Ca_LG_5:4961905	AAGAAACAAACCAAAGATCATGACAAAAAATTCTATAGATAAAACCGGTTTTCCTATGCCAAATTTCTCAACTTAAGTGCTAATTGAGAAAGAGTCTTTA	A/G	TAGAAGACCCATCTTCTTTGATTGCATTATTAGCAGCTTCTTTCAATTCCTTCATTCGGAGAGAGGCACGAAGAATTATCGTCACCGGAGAGATGTACGA
Ca_LG_5:4961927	ACAAAAAATTCTATAGATAAAACCGGTTTTCCTATGCCAAATTTCTCAACTTAAGTGCTAATTGAGAAAGAGTCTTTATAGAAGACCCATCTTCTTTGAT	T/G	TGCATTATTAGCAGCTTCTTTCAATTCCTTCATTCGGAGAGAGGCACGAAGAATTATCGTCACCGGAGAGATGTACGAAGCAACACCGTCACCGGAGAGA
Ca_LG_5:4961932	AAATTCTATAGATAAAACCGGTTTTCCTATGCCAAATTTCTCAACTTAAGTGCTAATTGAGAAAGAGTCTTTATAGAAGACCCATCTTCTTTGATTGCAT	T/C	TATTAGCAGCTTCTTTCAATTCCTTCATTCGGAGAGAGGCACGAAGAATTATCGTCACCGGAGAGATGTACGAAGCAACACCGTCACCGGAGAGAGAGGC

Positions	-100 bp flanking sequences	SNPs within *kabuli* cultivar	+100 bp flanking sequences
Ca_LG_5:4961937	CTATAGATAAAACCGGTTTTCCTATGCCAAATTTCTCAACTTAAGTGCTAATTGAGAAAGAGTCTTTATAGAAGACCCATCTTCTTTGATTGCATTATTA	A/G	GCAGCTTCTTTCAATTCCTTCATTCGGAGAGAGGCACGAAGAATTATCGTCACCGGAGAGATGTACGAAGCAACACCGTCACCGGAGAGAGAGGCAGGAA
Ca_LG_5:4961956	TCCTATGCCAAATTTCTCAACTTAAGTGCTAATTGAGAAAGAGTCTTTATAGAAGACCCATCTTCTTTGATTGCATTATTAGCAGCTTCTTTCAATTCCT	T/A	TCATTCGGAGAGAGGCACGAAGAATTATCGTCACCGGAGAGATGTACGAAGCAACACCGTCACCGGAGAGAGAGGCAGGAAGCAGCGTCGCAGCACGTCG
Ca_LG_5:4961962	GCCAAATTTCTCAACTTAAGTGCTAATTGAGAAAGAGTCTTTATAGAAGACCCATCTTCTTTGATTGCATTATTAGCAGCTTCTTTCAATTCCTTCATTC	C/T	GGAGAGAGGCACGAAGAATTATCGTCACCGGAGAGATGTACGAAGCAACACCGTCACCGGAGAGAGAGGCAGGAAGCAGCGTCGCAGCACGTCGAAGAAA
Ca_LG_5:4961983	GCTAATTGAGAAAGAGTCTTTATAGAAGACCCATCTTCTTTGATTGCATTATTAGCAGCTTCTTTCAATTCCTTCATTCGGAGAGAGGCACGAAGAATTA	A/G	TCGTCACCGGAGAGATGTACGAAGCAACACCGTCACCGGAGAGAGAGGCAGGAAGCAGCGTCGCAGCACGTCGAAGAAAGGTTTCAAGATTGTTCCTTCT
Ca_LG_5:4962003	TATAGAAGACCCATCTTCTTTGATTGCATTATTAGCAGCTTCTTTCAATTCCTTCATTCGGAGAGAGGCACGAAGAATTATCGTCACCGGAGAGATGTAC	C/G	GAAGCAACACCGTCACCGGAGAGAGAGGCAGGAAGCAGCGTCGCAGCACGTCGAAGAAAGGTTTCAAGATTGTTCCTTCTTTTGCTTTTAATGCTTGGCA
Ca_LG_5:4962010	GACCCATCTTCTTTGATTGCATTATTAGCAGCTTCTTTCAATTCCTTCATTCGGAGAGAGGCACGAAGAATTATCGTCACCGGAGAGATGTACGAAGCAA	A/G	CACCGTCACCGGAGAGAGAGGCAGGAAGCAGCGTCGCAGCACGTCGAAGAAAGGTTTCAAGATTGTTCCTTCTTTTGCTTTTAATGCTTGGCAACAATCG
Ca_LG_5:5599639	AGCTGGTCCTGAAAAACACCAAGCAGTAGCAGTAAGAAACAACGCTGATCTCTCGACATTCTATCGATGCAGTTTCGAAGGTTATCAAGACACACTGTAC	C/T	GCGCACTCTTTAAGACAATTCTACAGAGAATGTGACATTTATGGCACAGTTGATTTCATATTCGGCAACGCTGCAGTAGTCTTCCAAAATTGCAACATAT
Ca_LG_5:5599641	CTGGTCCTGAAAAACACCAAGCAGTAGCAGTAAGAAACAACGCTGATCTCTCGACATTCTATCGATGCAGTTTCGAAGGTTATCAAGACACACTGTACGC	C/T	GCACTCTTTAAGACAATTCTACAGAGAATGTGACATTTATGGCACAGTTGATTTCATATTCGGCAACGCTGCAGTAGTCTTCCAAAATTGCAACATATTC
Ca_LG_5:5599642	TGGTCCTGAAAAACACCAAGCAGTAGCAGTAAGAAACAACGCTGATCTCTCGACATTCTATCGATGCAGTTTCGAAGGTTATCAAGACACACTGTACGCG	G/T	CACTCTTTAAGACAATTCTACAGAGAATGTGACATTTATGGCACAGTTGATTTCATATTCGGCAACGCTGCAGTAGTCTTCCAAAATTGCAACATATTCG
Ca_LG_5:5599648	TGAAAAACACCAAGCAGTAGCAGTAAGAAACAACGCTGATCTCTCGACATTCTATCGATGCAGTTTCGAAGGTTATCAAGACACACTGTACGCGCACTCT	T/C	TTAAGACAATTCTACAGAGAATGTGACATTTATGGCACAGTTGATTTCATATTCGGCAACGCTGCAGTAGTCTTCCAAAATTGCAACATATTCGCTAGAA
Ca_LG_5:5599649	GAAAAACACCAAGCAGTAGCAGTAAGAAACAACGCTGATCTCTCGACATTCTATCGATGCAGTTTCGAAGGTTATCAAGACACACTGTACGCGCACTCTT	T/G	TAAGACAATTCTACAGAGAATGTGACATTTATGGCACAGTTGATTTCATATTCGGCAACGCTGCAGTAGTCTTCCAAAATTGCAACATATTCGCTAGAAA
Ca_LG_5:5599650	AAAAACACCAAGCAGTAGCAGTAAGAAACAACGCTGATCTCTCGACATTCTATCGATGCAGTTTCGAAGGTTATCAAGACACACTGTACGCGCACTCTTT	T/A	AAGACAATTCTACAGAGAATGTGACATTTATGGCACAGTTGATTTCATATTCGGCAACGCTGCAGTAGTCTTCCAAAATTGCAACATATTCGCTAGAAAA

**Positions**	**-100 bp flanking sequences**	**SNPs within *kabuli* cultivar**	**+100 bp flanking sequences**
Ca_LG_5:5599660	AGCAGTAGCAGTAAGAAACAACGCTGATCTCTCGACATTCTATCGATGCAGTTTCGAAGGTTATCAAGACACACTGTACGCGCACTCTTTAAGACAATTC	C/T	TACAGAGAATGTGACATTTATGGCACAGTTGATTTCATATTCGGCAACGCTGCAGTAGTCTTCCAAAATTGCAACATATTCGCTAGAAAACCGTTGCCAA
Ca_LG_5:5599673	AGAAACAACGCTGATCTCTCGACATTCTATCGATGCAGTTTCGAAGGTTATCAAGACACACTGTACGCGCACTCTTTAAGACAATTCTACAGAGAATGTG	G/A	ACATTTATGGCACAGTTGATTTCATATTCGGCAACGCTGCAGTAGTCTTCCAAAATTGCAACATATTCGCTAGAAAACCGTTGCCAAATCAAAAAAACGC
Ca_LG_5:5599687	TCTCTCGACATTCTATCGATGCAGTTTCGAAGGTTATCAAGACACACTGTACGCGCACTCTTTAAGACAATTCTACAGAGAATGTGACATTTATGGCACA	A/G	GTTGATTTCATATTCGGCAACGCTGCAGTAGTCTTCCAAAATTGCAACATATTCGCTAGAAAACCGTTGCCAAATCAAAAAAACGCGGTAACAGCACAAG
Ca_LG_5:5847419	AAAAACCTATCGATATACTTGTTTTGATGTAGCGTAAATGCCTTATGTAGATGCTATCACTTATTCTGCCTCGGCCGGATGCTGCCTTAGTTGATGTGAT	T/A	AGAATCCCCCTTGCTTCCTAATCATTTACTATGGTCTTGCTCAGTTAATAAAAGAAATTCGCTACCCCTACCTTCTCTTTCTTCCCTCACCCCACCTTTA
Ca_LG_5:5847440	TTTTGATGTAGCGTAAATGCCTTATGTAGATGCTATCACTTATTCTGCCTCGGCCGGATGCTGCCTTAGTTGATGTGATAGAATCCCCCTTGCTTCCTAA	A/C	TCATTTACTATGGTCTTGCTCAGTTAATAAAAGAAATTCGCTACCCCTACCTTCTCTTTCTTCCCTCACCCCACCTTTAGCCCTTGCTTTCGTTTTGCGC
Ca_LG_5:5850247	CGGATAGGAAATCGTATAGGCCCAGAAGCTCATAGTCGCACACCAACCAAAGACTAAACTAAGGCAGCTATTGAGGAAGAAGGAGAGGCTAACGAGATAT	T/G	GGGCGACAAAGCGAGGGGATTGAGCCTTTCTTCTAGCGCAAGAGTTTTCGTCGCTGGATTAAGACGACTTAACTACTTGTCTGAAAGCGGAAAAGAAAGT
Ca_LG_5:5850279	TAGTCGCACACCAACCAAAGACTAAACTAAGGCAGCTATTGAGGAAGAAGGAGAGGCTAACGAGATATGGGCGACAAAGCGAGGGGATTGAGCCTTTCTT	T/C	CTAGCGCAAGAGTTTTCGTCGCTGGATTAAGACGACTTAACTACTTGTCTGAAAGCGGAAAAGAAAGTTGACTAGCCTTCAAGCCTGAATAAGCAAAAAA
Ca_LG_5:5855315	TTATGTTCCCTTTCTAAGATACGGATATAAGTCCCACATACCCCCTTCTCGAGCTTATTCTTTTTAAGAGACTGATGCCAACTATGAATATACGATCGAG	G/A	TACGCTCTTGTCTATTTTTAGACTTCTTACTTTTATTTTTGCATTTTCTCTATAAAGCCTTCCCCAACGAAACAAATGTTGTTGCTCCTTCTGTTTAGAG
Ca_LG_5:5855358	CCTTCTCGAGCTTATTCTTTTTAAGAGACTGATGCCAACTATGAATATACGATCGAGTACGCTCTTGTCTATTTTTAGACTTCTTACTTTTATTTTTGCA	A/C	TTTTCTCTATAAAGCCTTCCCCAACGAAACAAATGTTGTTGCTCCTTCTGTTTAGAGTTTGCATGACTTAGCTCTCGTTGTGCGTGGACTTCCTAATCGA
Ca_LG_5:5855367	GCTTATTCTTTTTAAGAGACTGATGCCAACTATGAATATACGATCGAGTACGCTCTTGTCTATTTTTAGACTTCTTACTTTTATTTTTGCATTTTCTCTA	A/T	TAAAGCCTTCCCCAACGAAACAAATGTTGTTGCTCCTTCTGTTTAGAGTTTGCATGACTTAGCTCTCGTTGTGCGTGGACTTCCTAATCGAACTAAGATG
Ca_LG_5:5855369	TTATTCTTTTTAAGAGACTGATGCCAACTATGAATATACGATCGAGTACGCTCTTGTCTATTTTTAGACTTCTTACTTTTATTTTTGCATTTTCTCTATA	A/G	AAGCCTTCCCCAACGAAACAAATGTTGTTGCTCCTTCTGTTTAGAGTTTGCATGACTTAGCTCTCGTTGTGCGTGGACTTCCTAATCGAACTAAGATGGG
Ca_LG_5:5855385	ACTGATGCCAACTATGAATATACGATCGAGTACGCTCTTGTCTATTTTTAGACTTCTTACTTTTATTTTTGCATTTTCTCTATAAAGCCTTCCCCAACGA	A/G	AACAAATGTTGTTGCTCCTTCTGTTTAGAGTTTGCATGACTTAGCTCTCGTTGTGCGTGGACTTCCTAATCGAACTAAGATGGGTCGGGATGTTGGCTCA

Positions	-100 bp flanking sequences	SNPs within *kabuli* cultivar	+100 bp flanking sequences
Ca_LG_5:5855411	CGAGTACGCTCTTGTCTATTTTTAGACTTCTTACTTTTATTTTTGCATTTTCTCTATAAAGCCTTCCCCAACGAAACAAATGTTGTTGCTCCTTCTGTTT	T/C	AGAGTTTGCATGACTTAGCTCTCGTTGTGCGTGGACTTCCTAATCGAACTAAGATGGGTCGGGATGTTGGCTCAAATAAGGTTTTCTCAGGGAAGGAATA
Ca_LG_5:5867759	AAGCTCGGGCAGGAGTAGGACATTCCATAAAAATATTTCTGATCCTCTAGCTCTCACTCGTTGCCCTATTCGGTCAAGCAACTTCACTCCTCTCAAATCT	T/C	TATTGTTTCATCGACAAGTAGGGTTAATTCTAAGGTTCCTTATTCTGGTTGTCAAGCTGAAGAAGAGGGAGAAAGAATGGGAACAGCCCCACCAGCAGCC
Ca_LG_5:5867764	CGGGCAGGAGTAGGACATTCCATAAAAATATTTCTGATCCTCTAGCTCTCACTCGTTGCCCTATTCGGTCAAGCAACTTCACTCCTCTCAAATCTTATTG	G/T	TTTCATCGACAAGTAGGGTTAATTCTAAGGTTCCTTATTCTGGTTGTCAAGCTGAAGAAGAGGGAGAAAGAATGGGAACAGCCCCACCAGCAGCCCATCG
Ca_LG_5:5867776	GGACATTCCATAAAAATATTTCTGATCCTCTAGCTCTCACTCGTTGCCCTATTCGGTCAAGCAACTTCACTCCTCTCAAATCTTATTGTTTCATCGACAA	A/G	GTAGGGTTAATTCTAAGGTTCCTTATTCTGGTTGTCAAGCTGAAGAAGAGGGAGAAAGAATGGGAACAGCCCCACCAGCAGCCCATCGAAAGGAATGGAA
Ca_LG_5:5867805	CTAGCTCTCACTCGTTGCCCTATTCGGTCAAGCAACTTCACTCCTCTCAAATCTTATTGTTTCATCGACAAGTAGGGTTAATTCTAAGGTTCCTTATTCT	T/C	GGTTGTCAAGCTGAAGAAGAGGGAGAAAGAATGGGAACAGCCCCACCAGCAGCCCATCGAAAGGAATGGAAAATGAAATAAGAATTCGTGTTCACTATCT
Ca_LG_5:5867817	CGTTGCCCTATTCGGTCAAGCAACTTCACTCCTCTCAAATCTTATTGTTTCATCGACAAGTAGGGTTAATTCTAAGGTTCCTTATTCTGGTTGTCAAGCT	T/G	GAAGAAGAGGGAGAAAGAATGGGAACAGCCCCACCAGCAGCCCATCGAAAGGAATGGAAAATGAAATAAGAATTCGTGTTCACTATCTATGGAGCCAGTG
Ca_LG_5:6576980	TTGTAGTTATTGGTTTCCTCTGATTTCTAGTTGTATTTGGTTCTAGTTGCCTCTGATTTTGGTTTGGGCTCAACTCAGCAAGCTATAGGAGTACACTCCC	C/A	TTCCATCTTTTCAACCTCCTTGCTTTTCACCTTTCTTACGGCTACAATGTTCCTATGTATTCATATAATTAAAAAATACATCCTCTCTTCAACCATTTTT
Ca_LG_5:6577004	TTCTAGTTGTATTTGGTTCTAGTTGCCTCTGATTTTGGTTTGGGCTCAACTCAGCAAGCTATAGGAGTACACTCCCTTCCATCTTTTCAACCTCCTTGCT	T/A	TTTCACCTTTCTTACGGCTACAATGTTCCTATGTATTCATATAATTAAAAAATACATCCTCTCTTCAACCATTTTTTTAGCCACTTTTGTTTTTTTTTTC
Ca_LG_5:6641476	GGTCTTGATATTGTAACTAAAATATATCCTTTTACCTGTTGCACACAAAGCAGCTTGAATCATAATTTGATCGTCGATCCACAAAAGAAAATCCACACCT	T/C	ACAAGCTTATGACCACAAGCAGCGGCATGATCAAAAGGTCCCATTTCGTGATAAAGAGACCTGACATATTCCCACCCCTAAGTAGAATGAAGAATTTTAA
Ca_LG_5:6758586	TAAATAATATTAATCAATTTTGCATGGAAAAACAATCTTACCTCAACATATATAGATTCTAGCAAGAAGATTGATCAAATGCAGCTGATCCTACAAGTTT	T/G	GAGAATTGTTTCTATCTACAAATTGAGGAACATCAATAAAAGTATGTCAGTTGATTCAAACCATGATAGATTATTGCTTCTTTGGAAAAAAAGGCCACAA
Ca_LG_5:6869647	GTCGATCCTCATCCTCCTCCACCTGAAGAGGATGATGAAGAATATGAGTTTGGTGAGGCTACCGGTGAGGCTGAGGCGCCTATTGTGGTTGAGCCTGCCT	T/G	GTGAGGGTGCTGGTGCTGATATTGCTGCTTGGTGAGGATGTGGGTGAGGCTGAGGCGCATGAGGCGCCTGCTGTTGTGGTTGAGCTTGCCGGTGAGGGTG
Ca_LG_5:6869697	TGGTGAGGCTACCGGTGAGGCTGAGGCGCCTATTGTGGTTGAGCCTGCCTGTGAGGGTGCTGGTGCTGATATTGCTGCTTGGTGAGGATGTGGGTGAGGC	C/T	TGAGGCGCATGAGGCGCCTGCTGTTGTGGTTGAGCTTGCCGGTGAGGGTGCTGGATATTGCTGCTGCTGGTGAGGATGTGGGTGAAGGTGCCGGAAGGGT

**Positions**	**-100 bp flanking sequences**	**SNPs within *kabuli* cultivar**	**+100 bp flanking sequences**
Ca_LG_5:6869703	GGCTACCGGTGAGGCTGAGGCGCCTATTGTGGTTGAGCCTGCCTGTGAGGGTGCTGGTGCTGATATTGCTGCTTGGTGAGGATGTGGGTGAGGCTGAGGC	C/T	GCATGAGGCGCCTGCTGTTGTGGTTGAGCTTGCCGGTGAGGGTGCTGGATATTGCTGCTGCTGGTGAGGATGTGGGTGAAGGTGCCGGAAGGGTGGCAAG
Ca_LG_5:6869706	TACCGGTGAGGCTGAGGCGCCTATTGTGGTTGAGCCTGCCTGTGAGGGTGCTGGTGCTGATATTGCTGCTTGGTGAGGATGTGGGTGAGGCTGAGGCGCA	A/C	TGAGGCGCCTGCTGTTGTGGTTGAGCTTGCCGGTGAGGGTGCTGGATATTGCTGCTGCTGGTGAGGATGTGGGTGAAGGTGCCGGAAGGGTGGCAAGGGG
Ca_LG_5:6869733	GGTTGAGCCTGCCTGTGAGGGTGCTGGTGCTGATATTGCTGCTTGGTGAGGATGTGGGTGAGGCTGAGGCGCATGAGGCGCCTGCTGTTGTGGTTGAGCT	T/C	TGCCGGTGAGGGTGCTGGATATTGCTGCTGCTGGTGAGGATGTGGGTGAAGGTGCCGGAAGGGTGGCAAGGGGTGGTCAGTGGGCAAGGGTTGAAAGGGT
Ca_LG_5:6869738	AGCCTGCCTGTGAGGGTGCTGGTGCTGATATTGCTGCTTGGTGAGGATGTGGGTGAGGCTGAGGCGCATGAGGCGCCTGCTGTTGTGGTTGAGCTTGCCG	G/A	GTGAGGGTGCTGGATATTGCTGCTGCTGGTGAGGATGTGGGTGAAGGTGCCGGAAGGGTGGCAAGGGGTGGTCAGTGGGCAAGGGTTGAAAGGGTGCAGA
Ca_LG_5:6870827	CCAACTCACCAGAAACATCAGTACCAGCACCAGCACCATCACCAGCAGGCTCAACCACAACAGCAGGCGCCTTAGCCTCACCGGTAGCCTCGCCTTCACT	T/C	GGCAGCCTCGCCTTCAACGGTAGCCTCACCCTCACCCTCACCAAACTCATATTCTTCATCATCCTCTTGAGGTGGAGGAGGATGAGGATCGACAACCCCT
Ca_LG_5:6870846	AGTACCAGCACCAGCACCATCACCAGCAGGCTCAACCACAACAGCAGGCGCCTTAGCCTCACCGGTAGCCTCGCCTTCACTGGCAGCCTCGCCTTCAACG	G/A	GTAGCCTCACCCTCACCCTCACCAAACTCATATTCTTCATCATCCTCTTGAGGTGGAGGAGGATGAGGATCGACAACCCCTTGGCACCCCTTCCATCACCA
Ca_LG_5:6870896	CCTTAGCCTCACCGGTAGCCTCGCCTTCACTGGCAGCCTCGCCTTCAACGGTAGCCTCACCCTCACCCTCACCAAACTCATATTCTTCATCATCCTCTTG	G/C	AGGTGGAGGAGGATGAGGATCGACAACCCCTTGGCACCCCTTCCATCACCAGCACCTTCACCCACATCCTCAGCAACATCAGCACCAGCACCAGCACCCTC
Ca_LG_5:6870898	TTAGCCTCACCGGTAGCCTCGCCTTCACTGGCAGCCTCGCCTTCAACGGTAGCCTCACCCTCACCCTCACCAAACTCATATTCTTCATCATCCTCTTGAG	G/A	GTGGAGGAGGATGAGGATCGACAACCCCTTGGCACCCCTTCCATCACCAGCACCTTCACCCACATCCTCAGCAACATCAGCACCAGCACCAGCACCCTCAC
Ca_LG_5:6985476	TGATAATCTGAAAGAGTTCTAACTCTTAAAGGTAGAATCACTTTTGACTCTTGTACCACATTAAAATGTACATATCAATTACACATGAAGCAAGACTATA	A/G	TGCGCAGATTGTCCACATTAGATTATTGGAAAAGTAACCAAAGAAGAGAAAAGAAAATTTGAACAAAATGTCACACAAATAGTTATTTGTTTGTTTTTAT
Ca_LG_5:6985489	GAGTTCTAACTCTTAAAGGTAGAATCACTTTTGACTCTTGTACCACATTAAAATGTACATATCAATTACACATGAAGCAAGACTATATGCGCAGATTGTC	C/T	CACATTAGATTATTGGAAAAGTAACCAAAGAAGAGAAAAGAAAATTTGAACAAAATGTCACACAAATAGTTATTTGTTTGTTTTTATAGATACCGTGATG
Ca_LG_5:8077964	AAATCACAATCTGATCGTCCCTTGTCTAACTATATTTTAGGGGATTGTGGCAGCTGGTCTTACACAATACAGAGTTTACTCTATTGATGAAGTAATACTT	T/C	TGAACCATTCTGGTATATTGGAAGACTGCTGTATGAATTTTTTGAGTTTTTATTTGCTTTTGCTGCTTCAGGTGATGACATTGCTTCAGCAAGGAAATCG
Ca_LG_5:8078850	TTCATCTTCTGTCAAATATATCACTCCGTTGATTTTGAATAATGAAACTGAAATCTGTTAGACATGGATGCAGGCAAATGATGCAAATGAAGATCTATTT	T/A	CCAGTAACTGAAACAGGAACAGACCAGGCCAAGCTGGTACTTGAGCTGCAGAAGGAGAATCGTGAAATGCGAATGCAGCTAGCACGGCAACAACAAAAGC

Positions	-100 bp flanking sequences	SNPs within *kabuli* cultivar	+100 bp flanking sequences
Ca_LG_5:8078865	ATATATCACTCCGTTGATTTTGAATAATGAAACTGAAATCTGTTAGACATGGATGCAGGCAAATGATGCAAATGAAGATCTATTTCCAGTAACTGAAACA	A/T	GGAACAGACCAGGCCAAGCTGGTACTTGAGCTGCAGAAGGAGAATCGTGAAATGCGAATGCAGCTAGCACGGCAACAACAAAAGCTTTTGACACTCGAAG
Ca_LG_5:8827358	GATTTACTACTTTTGCTGCTATGTTTTCTTCCTCAGCCTCTTTTACTCCCTTGTTGATGTTGATTTCTGGTTCTTGACTACCAAATGTTTTAATCAGATA	A/C	GGTTAAAAAAGATTTGGATTTGGATTTTCTTGATCCCAAGAACAAAACCAGAGCAATATCCAAGCATCTAGGCTCGTGAACGCCCACTGCACTTTATCAA
Ca_LG_5:8858516	GTAGAAGGAGCACTTCAGGTTATGTATTCAAATTCATGAAATCTTCAATAGCATGGAGCTTAAATAAGCAGCTTGTGATAGCATTTTCATCATGCGAAGC	C/T	TGAGTACATTGCTGACAGCTATGCAACCTGCCAAACATTGTGGTTGGAGTCATTAACAACAGAGCTAAAGATGGAAATTTGCAGACCAATTCCATTGCTG
Ca_LG_5:8858518	AGAAGGAGCACTTCAGGTTATGTATTCAAATTCATGAAATCTTCAATAGCATGGAGCTTAAATAAGCAGCTTGTGATAGCATTTTCATCATGCGAAGCTG	G/A	AGTACATTGCTGACAGCTATGCAACCTGCCAAACATTGTGGTTGGAGTCATTAACAACAGAGCTAAAGATGGAAATTTGCAGACCAATTCCATTGCTGAT
Ca_LG_5:8858520	AAGGAGCACTTCAGGTTATGTATTCAAATTCATGAAATCTTCAATAGCATGGAGCTTAAATAAGCAGCTTGTGATAGCATTTTCATCATGCGAAGCTGAG	G/A	TACATTGCTGACAGCTATGCAACCTGCCAAACATTGTGGTTGGAGTCATTAACAACAGAGCTAAAGATGGAAATTTGCAGACCAATTCCATTGCTGATTG
Ca_LG_5:9031733	ACAAGCAGCTATTGCTGCAGATGAGCTTCCTCCTCTTCCTATTGGTAGTAAAAAAAGAAAACCCAATGCTAATGGTCCTAGGAGAAGCTCTAAGGTTTGG	G/A	CTGAACTTTAACATTCTACCCGATGGGCCTATCCCAACTGCTGCTTGTAAACATTGTCACCAAAGATACCTTTGCCATTCTAAAACACATGGAACCTCGA
Ca_LG_5:9042477	AGAGAAGTTAGAAGTGAAAAATGATAAGAGGAGTGGAGTAATATATATAGGATTGGGTTGATCCTTTGTGGCAGCCAAAGCAGCCACAATAAGCAACGGT	T/C	TATGTGTCATTTGTGGCGGCCAAAACGGACAAAAAGACATGGATTTGTGGCGGCCTGGACGACCAGAAAAGCCAACGTCTCTTTATTCTTTTGTGGCGGC
Ca_LG_5:9982511	GTTTTTTTTTCCTCTTCAAAATTCTGTGTTCCAACTGTGCTTTTCCATTCCAGCTGTGTGTATACTATTCTAGTGCAGCCCACCACCACAGACCGCCGCA	A/G	CAATCCTCCAGCGAATACGGTGACATTTTTTTTTGCGCTGCCAAACTCCAGCAAACACGGAGACACATTTGGCCACCAATTTTTTCAAAGAAAATCGTCG
Ca_LG_5:9982523	TCTTCAAAATTCTGTGTTCCAACTGTGCTTTTCCATTCCAGCTGTGTGTATACTATTCTAGTGCAGCCCACCACCACAGACCGCCGCACAATCCTCCAGC	C/T	GAATACGGTGACATTTTTTTTTGCGCTGCCAAACTCCAGCAAACACGGAGACACATTTGGCCACCAATTTTTTCAAAGAAAATCGTCGCTCAAGGCCTTA
Ca_LG_5:10033305	TTGTTGTAACTGAACTTCATCTAAGGATATAACCTGATAAGTAAAGGCAATATAAGAATGATTCATAATCGCAGTAGGTAGGGCAGGAGTACACTGCTTA	A/C	AATTAGCAAATTCTCGAAATAAAAATAAAGTACCAACCAAACCACTCAGTTCAGCAGTAACAGAATATTGAGCAGCATTGTCATCAGGCTTTTGCGATCT
Ca_LG_5:10254664	GATAACTTAGAAGCAAAGATGGCAAAATATTATCATCTGGTATGTTCCATATGTTGCTATTCAGAATTGAAATCCAATCCTTTGCATCTACCTTTGAACT	T/G	CAAAAGCCCTCCGAGTGTATTGACAGCAATTGGCAATCCTCCACACTTTCTAGCAACCTTCCTACCAATTGCTTCTATGTTTTGGTATTTATTTCCACCA
Ca_LG_5:10254671	TAGAAGCAAAGATGGCAAAATATTATCATCTGGTATGTTCCATATGTTGCTATTCAGAATTGAAATCCAATCCTTTGCATCTACCTTTGAACTCAAAAGC	C/T	CCTCCGAGTGTATTGACAGCAATTGGCAATCCTCCACACTTTCTAGCAACCTTCCTACCAATTGCTTCTATGTTTTGGTATTTATTTCCACCATAATTTT

Positions	-100 bp flanking sequences	SNPs within *kabuli* cultivar	+100 bp flanking sequences
Ca_LG_5:10386031	AGATGACGAGGCTTCTGTGCCAAATAGATCATATACTCATCCACGATATCTTCTATGCGGAAAGCTGCTTCCCTAACTTGTTTCACCCATGTTTTGATCC	C/T	CTTCACTGTTGTTGTCTCCATTTAATGCAGCTCTTTTATCAGCATCCTTAAGGAAGGCTTGCATGCTTTCCAGTTCATCTTTAATGTCTGAAAATTCCTT
Ca_LG_5:10386036	ACGAGGCTTCTGTGCCAAATAGATCATATACTCATCCACGATATCTTCTATGCGGAAAGCTGCTTCCCTAACTTGTTTCACCCATGTTTTGATCCCTTCA	A/C	CTGTTGTTGTCTCCATTTAATGCAGCTCTTTTATCAGCATCCTTAAGGAAGGCTTGCATGCTTTCCAGTTCATCTTTAATGTCTGAAAATTCCTTGTGAA
Ca_LG_5:10386093	AGCTGCTTCCCTAACTTGTTTCACCCATGTTTTGATCCCTTCACTGTTGTTGTCTCCATTTAATGCAGCTCTTTTATCAGCATCCTTAAGGAAGGCTTGC	C/G	ATGCTTTCCAGTTCATCTTTAATGTCTGAAAATTCCTTGTGAACACCTTTGATCAGTTCGGCTTCTTCTGTTAATAGCGGAAGCAGTTGGTCAATAGCAA
Ca_LG_5:11632797	TCTTCTTTTCTGATAGTATGCTTTGTACCAGATTCATGACTGTTTGATTCCTCCTTTCCACTACTCCGTTTTGTTGAGGAGTATAGGCAGCCGTTAGTTG	G/T	CCTTTTGATGTCGTGTTCTTTGCAAAATTCGATAAATTCATCTGAGTTGAATTCTCCTCCTATGTCCGTTCTTAGGCAGCAAACCGGAAATTCAACTTCT
Ca_LG_5:11632859	ACTCCGTTTTGTTGAGGAGTATAGGCAGCCGTTAGTTGCCTTTTGATGTCGTGTTCTTTGCAAAATTCGATAAATTCATCTGAGTTGAATTCTCCTCCTA	A/C	TGTCCGTTCTTAGGCAGCAAACCGGAAATTCAACTTCTTTCTCAACACTTGCTTTGAATACTTTGAAGGAGTTGAAGGCTTCATATTTTTTTGCAATGAG
Ca_LG_5:11657961	GAAGTAGATAGGAATTTAGAAACAGAGGATGTTGCCAACATTTTACTTGGAAAAAGTCTTTTAGTGCGCTGTGATCGTGTGGTAAAAACTCTTCGCCCTG	G/A	GGAAGAAAAAACTATCTACTTGGCCTGCACATTTAGAAATTTTCCCTGTAAGTCATTTTCCTTGCTGCAATCATTGACTTGCTATGTGATTATTGTGTTA
Ca_LG_5:11658126	TGCAATCATTGACTTGCTATGTGATTATTGTGTTAGAAGGTTGAACATATATGAAGTTGCATGTGGATCTGTGATCTGTCAATATTTCAGTATATTTCTG	G/A	GTGCTGCAGTACCTGTTTAATATTATTTTTTCTCATTTTATCCTCATAAGCTGTAATAACACCCCTTTCTTGTAATTAAGAAGTTGAATTTCAATTTCAA
Ca_LG_5:11674988	TCGGTGGCAACGTGTTAGTGTCATGTTCGTGTTTTTGCTAGCTCAATGTTTTGCTTAGATGAGCTTGGTATTTGGTTCGCTTTGAAGGTTCTTTGTTGCG	G/A	GTGACTTTATTTATGGCTTTATTTTATGTTTGCATAATTGACATAACTGTTTTTTCTTTTTACATGATAGGCTGCATAAAGCTTATCTTTGATTGAATTT
Ca_LG_5:11675004	AGTGTCATGTTCGTGTTTTTGCTAGCTCAATGTTTTGCTTAGATGAGCTTGGTATTTGGTTCGCTTTGAAGGTTCTTTGTTGCGGTGACTTTATTTATGG	G/T	CTTTATTTTATGTTTGCATAATTGACATAACTGTTTTTTCTTTTTACATGATAGGCTGCATAAAGCTTATCTTTGATTGAATTTAGCCCTCCAGCTATTG
Ca_LG_5:11860170	CTATCTCATAAGTGCGTTGAGACCATAGCATGTTAAACGAAAGCGCCGCTTTTCTTTGTGTTGAGCTCCTCTTCCTTCGCTGCTTTCGTACACCAGGGGT	T/G	ATAGAAGCCCACGGAGCGGTAGGCTAAGCTGGAAAGAGAGAGATCAGTTGGTTTGGTTCTGGGTTGTCTCTATATTTCTAATTATTCTGTTAATAGAATC
Ca_LG_5:11860200	TGTTAAACGAAAGCGCCGCTTTTCTTTGTGTTGAGCTCCTCTTCCTTCGCTGCTTTCGTACACCAGGGGTATAGAAGCCCACGGAGCGGTAGGCTAAGCT	T/C	GGAAAGAGAGAGATCAGTTGGTTTGGTTCTGGGTTGTCTCTATATTTCTAATTATTCTGTTAATAGAATCTGAATTGATGCTGGTTTCTCCGTCTTATGT
Ca_LG_5:11860214	GCCGCTTTTCTTTGTGTTGAGCTCCTCTTCCTTCGCTGCTTTCGTACACCAGGGGTATAGAAGCCCACGGAGCGGTAGGCTAAGCTGGAAAGAGAGAGAT	T/G	CAGTTGGTTTGGTTCTGGGTTGTCTCTATATTTCTAATTATTCTGTTAATAGAATCTGAATTGATGCTGGTTTCTCCGTCTTATGTTTCAAAAATTATAA

Positions	-100 bp flanking sequences	SNPs within *kabuli* cultivar	+100 bp flanking sequences
Ca_LG_5:12037840	TTTCTGGTATAGGTCTCAACAATTTTATCATTTTCTTGATTCATAGAAGTATTATAGCCATGAATTTCATTAACAAGGTTATGATCAACATATAACATAC	C/G	TGAGCGGTTCTCAGATTGTTGATTGTAGTAGACACGTAGCGCTGTTGCTCTTTAAATATTTTTTATTAGCTAAGAATCCTAAATAATTACAAATAAAAGA
Ca_LG_5:12037854	CTCAACAATTTTATCATTTTCTTGATTCATAGAAGTATTATAGCCATGAATTTCATTAACAAGGTTATGATCAACATATAACATACTGAGCGGTTCTCAG	G/A	ATTGTTGATTGTAGTAGACACGTAGCGCTGTTGCTCTTTAAATATTTTTTATTAGCTAAGAATCCTAAATAATTACAAATAAAAGACACTAAAATAGCAG
Ca_LG_5:12037870	TTTTCTTGATTCATAGAAGTATTATAGCCATGAATTTCATTAACAAGGTTATGATCAACATATAACATACTGAGCGGTTCTCAGATTGTTGATTGTAGTA	A/T	GACACGTAGCGCTGTTGCTCTTTAAATATTTTTTATTAGCTAAGAATCCTAAATAATTACAAATAAAAGACACTAAAATAGCAGGTGGATAGAACTTTAT
Ca_LG_5:12038452	GCGCTTGGGTGGTTATTCTCGTCAACCTCCACTCTCATCTTTACGTAATACTGCACTCAAAGCTGCGGAAAAGAGATCACAGTTGGGATCTCTTCTTCCA	A/C	TCTGGACCAAATCGTATTGGTGGCAATAGTGTTATAATGAAGTCACTTACTCCAGCACAAGCAGCTGTGATGGCTGCAGAAAGGAGATTACAGGATGACA
Ca_LG_5:12038454	GCTTGGGTGGTTATTCTCGTCAACCTCCACTCTCATCTTTACGTAATACTGCACTCAAAGCTGCGGAAAAGAGATCACAGTTGGGATCTCTTCTTCCATC	C/A	TGGACCAAATCGTATTGGTGGCAATAGTGTTATAATGAAGTCACTTACTCCAGCACAAGCAGCTGTGATGGCTGCAGAAAGGAGATTACAGGATGACATA
Ca_LG_5:12038459	GGTGGTTATTCTCGTCAACCTCCACTCTCATCTTTACGTAATACTGCACTCAAAGCTGCGGAAAAGAGATCACAGTTGGGATCTCTTCTTCCATCTGGAC	C/A	CAAATCGTATTGGTGGCAATAGTGTTATAATGAAGTCACTTACTCCAGCACAAGCAGCTGTGATGGCTGCAGAAAGGAGATTACAGGATGACATATGGTG
Ca_LG_5:12038466	ATTCTCGTCAACCTCCACTCTCATCTTTACGTAATACTGCACTCAAAGCTGCGGAAAAGAGATCACAGTTGGGATCTCTTCTTCCATCTGGACCAAATCG	G/A	TATTGGTGGCAATAGTGTTATAATGAAGTCACTTACTCCAGCACAAGCAGCTGTGATGGCTGCAGAAAGGAGATTACAGGATGACATATGGTGTGGTTCT
Ca_LG_5:12038481	CACTCTCATCTTTACGTAATACTGCACTCAAAGCTGCGGAAAAGAGATCACAGTTGGGATCTCTTCTTCCATCTGGACCAAATCGTATTGGTGGCAATAG	G/A	TGTTATAATGAAGTCACTTACTCCAGCACAAGCAGCTGTGATGGCTGCAGAAAGGAGATTACAGGATGACATATGGTGTGGTTCTCAATTTTGTGATAAA
Ca_LG_5:12365614	ACAGTTTTCATTTCCTCATGCTTTTCATCATCAGTTCCCGCCTCACCAACAACAACAATCAAGGCATCAATACTTGCAGCAACAACCACTGTACCAACAG	G/A	CAGCAACCGCCTCCAAATTTTCAGCAGCAACAACAGGATCATCCAAACTCCGCCTTCCAGCAACAACATCAACCAATGTTTCAGCAACAATAATATTTTA
Ca_LG_5:12378702	AAAACATGGTGTTGACTATGAAGAAGTTTTTGCACCAGTTTCTCGCGCGGACACTATTCGTTTGCTAATCTCTGTAGCAGCTCAAAATAAGTGGAAATTT	T/G	TATCAACTTGATGTAAAATCGGCATTTCTAAATGGCTATCTTGAAGAAGAGGTCTAAGTTGAGCAGCCAATGGGGTACATGATTAAAGGACATGAAGATA
Ca_LG_5:12391970	AAGTTAGAAGTGAAAAATGATAAGAGGAGTGGAGTAATATATATATATATGATTGAGTTTATCCTTTGTGGCGGCCAAAGCAGACACAATAAGCAACGAT	T/C	TATGTGTCATTTGTGGCGGCCAAAACGGTCAAAAAGGCATGGATTTGTGGCGGCCTGGACGGCCACAACTGGCAACGGTCTCTTTATTCTTGTGTGGTGG
Ca_LG_5:12392028	TTATCCTTTGTGGCGGCCAAAGCAGACACAATAAGCAACGATTATGTGTCATTTGTGGCGGCCAAAACGGTCAAAAAGGCATGGATTTGTGGCGGCCTGG	G/A	ACGGCCACAACTGGCAACGGTCTCTTTATTCTTGTGTGGTGGGCCTTTGCAGCCACAAAATGCAACTGGCCATGCTTTTGTGGCGGCCTTTGCAGTCACAA

**Positions**	**-100 bp flanking sequences**	**SNPs within *kabuli* cultivar**	**+100 bp flanking sequences**
Ca_LG_5:12392042	GGCCAAAGCAGACACAATAAGCAACGATTATGTGTCATTTGTGGCGGCCAAAACGGTCAAAAAGGCATGGATTTGTGGCGGCCTGGACGGCCACAACTGG	G/A	CAACGGTCTCTTTATTCTTGTGTGGTGGCCTTTGCAGCCACAAAATGCAACTGGCCATGCTTTTGTGGCGGCCTTTGCAGTCACAAACTGCAACGTCACT
Ca_LG_5:12974344	AGGGTTAGGGCCTCAGAGGCAGTGTATCAATTGCAGCTTGCAGAAAATAGCAGTTTGTTCAAATTCCGTTACAATACTATAACCGCTATTTAACAAAACA	A/T	TTGTACTCAATTGTGTATTATTTAACGATAGTAATTTATTAAAATTCCGCTATAGCTGCAAATTTTTAGTGCTGCACAGGGGTATTGATGATCTATTGCT
Ca_LG_5:12974358	AGAGGCAGTGTATCAATTGCAGCTTGCAGAAAATAGCAGTTTGTTCAAATTCCGTTACAATACTATAACCGCTATTTAACAAAACATTGTACTCAATTGT	T/C	GTATTATTTAACGATAGTAATTTATTAAAATTCCGCTATAGCTGCAAATTTTTAGTGCTGCACAGGGGTATTGATGATCTATTGCTTTGCCTGTGAAAGA
Ca_LG_5:12974364	AGTGTATCAATTGCAGCTTGCAGAAAATAGCAGTTTGTTCAAATTCCGTTACAATACTATAACCGCTATTTAACAAAACATTGTACTCAATTGTGTATTA	A/G	TTTAACGATAGTAATTTATTAAAATTCCGCTATAGCTGCAAATTTTTAGTGCTGCACAGGGGTATTGATGATCTATTGCTTTGCCTGTGAAAGAGGCTGG
Ca_LG_5:12974372	AATTGCAGCTTGCAGAAAATAGCAGTTTGTTCAAATTCCGTTACAATACTATAACCGCTATTTAACAAAACATTGTACTCAATTGTGTATTATTTAACGA	A/G	TAGTAATTTATTAAAATTCCGCTATAGCTGCAAATTTTTAGTGCTGCACAGGGGTATTGATGATCTATTGCTTTGCCTGTGAAAGAGGCTGGTTTCAAGT
Ca_LG_5:13046477	TATTTATTAATTGAGTCTATAAAATTATTAACAGAAGACACAATCAAAGACATGTGCGAAACATCAAAAAGTGAGGATACAAAGAAGTCTGAAACATTGA	A/G	AAAGTGAGGATGCAAAGAAGTTAGAAACATTGAAAATTGAGGAAGAAAAGAAAGCTGAAACATTGAAAAGTGAAACACCAATATTAACTGCAGCAAGATA
Ca_LG_5:13046488	TGAGTCTATAAAATTATTAACAGAAGACACAATCAAAGACATGTGCGAAACATCAAAAAGTGAGGATACAAAGAAGTCTGAAACATTGAAAAGTGAGGAT	T/A	GCAAAGAAGTTAGAAACATTGAAAATTGAGGAAGAAAAGAAAGCTGAAACATTGAAAAGTGAAACACCAATATTAACTGCAGCAAGATATGGTATAGTTG
Ca_LG_5:13046490	AGTCTATAAAATTATTAACAGAAGACACAATCAAAGACATGTGCGAAACATCAAAAAGTGAGGATACAAAGAAGTCTGAAACATTGAAAAGTGAGGATGC	C/A	AAAGAAGTTAGAAACATTGAAAATTGAGGAAGAAAAGAAAGCTGAAACATTGAAAAGTGAAACACCAATATTAACTGCAGCAAGATATGGTATAGTTGAA
Ca_LG_5:13046498	AAATTATTAACAGAAGACACAATCAAAGACATGTGCGAAACATCAAAAAGTGAGGATACAAAGAAGTCTGAAACATTGAAAAGTGAGGATGCAAAGAAGT	T/A	TAGAAACATTGAAAATTGAGGAAGAAAAGAAAGCTGAAACATTGAAAAGTGAAACACCAATATTAACTGCAGCAAGATATGGTATAGTTGAAATGGTGAA
Ca_LG_5:13046499	AATTATTAACAGAAGACACAATCAAAGACATGTGCGAAACATCAAAAAGTGAGGATACAAAGAAGTCTGAAACATTGAAAAGTGAGGATGCAAAGAAGTT	T/A	AGAAACATTGAAAATTGAGGAAGAAAAGAAAGCTGAAACATTGAAAAGTGAAACACCAATATTAACTGCAGCAAGATATGGTATAGTTGAAATGGTGAAT
Ca_LG_5:13046505	TAACAGAAGACACAATCAAAGACATGTGCGAAACATCAAAAAGTGAGGATACAAAGAAGTCTGAAACATTGAAAAGTGAGGATGCAAAGAAGTTAGAAAC	C/T	ATTGAAAATTGAGGAAGAAAAGAAAGCTGAAACATTGAAAAGTGAAACACCAATATTAACTGCAGCAAGATATGGTATAGTTGAAATGGTGAATGTGCTT
Ca_LG_5:13117846	GTGTCTTGGTTGTCTCCCATAATAGGTACAATTAAATGCAATATTGACGCAATACTATTCAAAGATATAGGTCGCATCAAAGTGGGTTTTTGTTTACATA	A/G	ACGAATGGGGAAGAGTGTTGGGTGCTCAAATAGAGTGGCACCCATGGAACATGATTGCGCTAGAGGTGGAAGCTTGGGGACTGTTTCAAGTGATCAAGTG

Positions	-100 bp flanking sequences	SNPs within *kabuli* cultivar	+100 bp flanking sequences
Ca_LG_5:13117859	CTCCCATAATAGGTACAATTAAATGCAATATTGACGCAATACTATTCAAAGATATAGGTCGCATCAAAGTGGGTTTTTGTTTACATAACGAATGGGGAAG	G/T	AGTGTTGGGTGCTCAAATAGAGTGGCACCCATGGAACATGATTGCGCTAGAGGTGGAAGCTTGGGGACTGTTTCAAGTGATCAAGTGGATAACATCTTAT
Ca_LG_5:14305618	CTTTGGTTGTGTTATAGTACATGAAGTTCTCTCCCATTTCATTGAGCATGGTGTTGAGTAGCCATGACATCACTAGGTTGTTTTCTAGTTGCAATATTTT	T/C	GAGTTTGGGATCTCATTTTTCTGGATTTTTGAAGTCTCTTATGATATACCTTTCTTTCCCTTTTTCTTGAAGGTAGATTCTAATTGGATGCACTTATTGA
Ca_LG_5:14305650	CCCATTTCATTGAGCATGGTGTTGAGTAGCCATGACATCACTAGGTTGTTTTCTAGTTGCAATATTTTGAGTTTGGGATCTCATTTTTCTGGATTTTTGA	A/G	AGTCTCTTATGATATACCTTTCTTTCCCTTTTTCTTGAAGGTAGATTCTAATTGGATGCACTTATTGATTGTAATTCTAGCCATTCATATTGTAATTGTT
Ca_LG_5:14585128	AATTTATGATAAACTTTGATTTTTGACTAACCATGCACCAACTCTTCGATATATTTCTTGTTTGATGGTTCTATGAGTATCCAAAATACAAACGAGATGA	A/G	ATGTTTATTGTCCCAACTATTTTTGTTAGTCTCGATACCGATAAGAAATGAAATATTTTTTTTTACAAAATTTTCTCCTGGGATGAAGAATAAAGCATAT
Ca_LG_5:14585146	ATTTTTGACTAACCATGCACCAACTCTTCGATATATTTCTTGTTTGATGGTTCTATGAGTATCCAAAATACAAACGAGATGAATGTTTATTGTCCCAACT	T/C	ATTTTTGTTAGTCTCGATACCGATAAGAAATGAAATATTTTTTTTTACAAAATTTTCTCCTGGGATGAAGAATAAAGCATATCTTAGTTTCAATTAATGC
Ca_LG_5:14892166	GGAAAATTGGAATTTGTGGCGGCCTTTGCAGCCACAAAGTGCAACGGATCAATGCTTTTGTTGCGGCCTGGGCGGCCACAAACGACTTGCCTTTGTGGCG	G/A	GCCTTTACCCTCACAAAAGGTTGATTTTGTGGCTGAAAAAATCCTCACAAAAGACTTAAATTTTAGCTGGATTTTGTGGCGGCCTAAGCCCTCACAAAAT
Ca_LG_5:14892174	GGAATTTGTGGCGGCCTTTGCAGCCACAAAGTGCAACGGATCAATGCTTTTGTTGCGGCCTGGGCGGCCACAAACGACTTGCCTTTGTGGCGGCCTTTAC	C/A	CCTCACAAAAGGTTGATTTTGTGGCTGAAAAAATCCTCACAAAAGACTTAAATTTTAGCTGGATTTTGTGGCGGCCTAAGCCCTCACAAAATAACAATGT
Ca_LG_5:15182998	CATACTTAGTCCAAAAAATTAGCACCAAAATTTTAGTTCATCTCATATCTCAAACGATGTAACCTATACTCATAGAACTCTAACTGCTCCAAATTGATCAT	T/C	GATTATATTTTCTTTCGAATTTATCCTCACTTAAAGATTACAACAAACTAAAGCAAAGATTTCCAAGTAGCAGATGAGATAAATAATTATTTACTGAAAG
Ca_LG_5:15183014	ATTAGCACCAAAATTTTAGTTCATCTCATATCTCAAACGATGTAACCTATACTCATAGAACTCTAACTGCTCCAAATTGATCATGATTATATTTTCTTTC	C/T	GAATTTATCCTCACTTAAAGATTACAACAAACTAAAGCAAAGATTTCCAAGTAGCAGATGAGATAAATAATTATTTACTGAAAGATATAACACACAACCA
Ca_LG_5:15183035	CATCTCATATCTCAAACGATGTAACCTATACTCATAGAACTCTAACTGCTCCAAATTGATCATGATTATATTTTCTTTCGAATTTATCCTCACTTAAAGA	A/G	TTACAACAAACTAAAGCAAAGATTTCCAAGTAGCAGATGAGATAAATAATTATTTACTGAAAGATATAACACACAACCAAACCAAATTGAATCAGGTAGT
Ca_LG_5:15183047	CAAACGATGTAACCTATACTCATAGAACTCTAACTGCTCCAAATTGATCATGATTATATTTTCTTTCGAATTTATCCTCACTTAAAGATTACAACAAACT	T/C	AAAGCAAAGATTTCCAAGTAGCAGATGAGATAAATAATTATTTACTGAAAGATATAACACACAACCAAACCAAATTGAATCAGGTAGTACACAAACCACT
Ca_LG_5:15183052	GATGTAACCTATACTCATAGAACTCTAACTGCTCCAAATTGATCATGATTATATTTTCTTTCGAATTTATCCTCACTTAAAGATTACAACAAACTAAAGC	C/A	AAAGATTTCCAAGTAGCAGATGAGATAAATAATTATTTACTGAAAGATATAACACACAACCAAACCAAATTGAATCAGGTAGTACACAAACCACTTTAAA

**Positions**	**-100 bp flanking sequences**	**SNPs within *kabuli* cultivar**	**+100 bp flanking sequences**
Ca_LG_5:16045497	TTGGAGCAAGTGGTGGCTATGGTATTGAATTCATCTCCTGATCAGCATCCTCATGTAAGGTGGGCAGCTATTAATGCAATTGGTGGGCAATTGTCTACTG	G/A	ATTTGGGTCTATATTTGCAAGTTCAATATCATCGGGGGGTGTTTCCAGCCCTAGCTGCTGCGATGGATGATTTTCAGAATCCTCGTGTAGAGACTCATGC
Ca_LG_5:16045512	GCTATGGTATTGAATTCATCTCCTGATCAGCATCCTCATGTAAGGTGGGCAGCTATTAATGCAATTGGTGGGCAATTGTCTACTGATTTGGGTCTATATT	T/A	TGCAAGTTCAATATCATCGGGGGGTGTTTCCAGCCCTAGCTGCTGCGATGGATGATTTTCAGAATCCTCGTGTAGAGACTCATGCCACTTCAGCTCTGCT
Ca_LG_5:16045531	CTCCTGATCAGCATCCTCATGTAAGGTGGGCAGCTATTAATGCAATTGGTGGGCAATTGTCTACTGATTTGGGTCTATATTTGCAAGTTCAATATCATCG	G/A	GGGGGTGTTTCCAGCCCTAGCTGCTGCGATGGATGATTTTCAGAATCCTCGTGTAGAGACTCATGCCACTTCAGCTCTGCTCAACTTCAGCGAGAATTGC
Ca_LG_5:16045545	CCTCATGTAAGGTGGGCAGCTATTAATGCAATTGGTGGGCAATTGTCTACTGATTTGGGTCTATATTTGCAAGTTCAATATCATCGGGGGGTGTTTCCAG	G/T	CCCTAGCTGCTGCGATGGATGATTTTCAGAATCCTCGTGTAGAGACTCATGCCACTTCAGCTCTGCTCAACTTCAGCGAGAATTGCACACATGATATTTT
Ca_LG_5:16142733	AAGTTCGTATATGTGCTCCTTTTCAAGAAATTATTCATACTCATCTCCGAATATAGCTTTATTCATGGTTATGATGCGTGAATCGTCGTTGCTGCCCATG	G/A	TTCCTTTTTACTTGGATGTCAAGACATGCCCCGTATTTAGCAAGGCATGGGTGACTTATTTTAGGAGCAACAGCGACTGATTTTCCTCGATTCAGTTTTT
Ca_LG_5:16142736	TTCGTATATGTGCTCCTTTTCAAGAAATTATTCATACTCATCTCCGAATATAGCTTTATTCATGGTTATGATGCGTGAATCGTCGTTGCTGCCCATGTTC	C/G	CTTTTTACTTGGATGTCAAGACATGCCCCGTATTTAGCAAGGCATGGGTGACTTATTTTAGGAGCAACAGCGACTGATTTTCCTCGATTCAGTTTTTTAG
Ca_LG_5:16142773	TCATCTCCGAATATAGCTTTATTCATGGTTATGATGCGTGAATCGTCGTTGCTGCCCATGTTCCTTTTTACTTGGATGTCAAGACATGCCCCGTATTTAG	G/C	CAAGGCATGGGTGACTTATTTTAGGAGCAACAGCGACTGATTTTCCTCGATTCAGTTTTTTAGCTGCAACTTTGGCAGCTTTATTACCCTCCAGCTTTTG
Ca_LG_5:16142783	ATATAGCTTTATTCATGGTTATGATGCGTGAATCGTCGTTGCTGCCCATGTTCCTTTTTACTTGGATGTCAAGACATGCCCCGTATTTAGCAAGGCATGG	G/A	GTGACTTATTTTAGGAGCAACAGCGACTGATTTTCCTCGATTCAGTTTTTTAGCTGCAACTTTGGCAGCTTTATTACCCTCCAGCTTTTGTAGTTTGCCA
Ca_LG_5:16142784	TATAGCTTTATTCATGGTTATGATGCGTGAATCGTCGTTGCTGCCCATGTTCCTTTTTACTTGGATGTCAAGACATGCCCCGTATTTAGCAAGGCATGGG	G/T	TGACTTATTTTAGGAGCAACAGCGACTGATTTTCCTCGATTCAGTTTTTTAGCTGCAACTTTGGCAGCTTTATTACCCTCCAGCTTTTGTAGTTTGCCAG
Ca_LG_5:16580335	ACTGGCGTGAACCACAATGTTTTAGAGAGGACAAAGTGGTCAAACTCATCGTGTATGACAGTGCATATACCCACCTAGTTGCGCTGCAACGAAAAGGAAG	G/A	CTTCAATGTTGCATTTGACTTGACCTTGCACTGGTGCAATCCAATGTGTTAGCAGCTTGGTTGTCTCATTGCTGATGCAACAACTTTTGCATTTGTTAGT
Ca_LG_5:16580337	TGGCGTGAACCACAATGTTTTAGAGAGGACAAAGTGGTCAAACTCATCGTGTATGACAGTGCATATACCCACCTAGTTGCGCTGCAACGAAAAGGAAGCT	T/A	TCAATGTTGCATTTGACTTGACCTTGCACTGGTGCAATCCAATGTGTTAGCAGCTTGGTTGTCTCATTGCTGATGCAACAACTTTTGCATTTGTTAGTCC
Ca_LG_5:16580360	AGAGGACAAAGTGGTCAAACTCATCGTGTATGACAGTGCATATACCCACCTAGTTGCGCTGCAACGAAAAGGAAGCTTCAATGTTGCATTTGACTTGACC	C/T	TTGCACTGGTGCAATCCAATGTGTTAGCAGCTTGGTTGTCTCATTGCTGATGCAACAACTTTTGCATTTGTTAGTCCATGCACAACTCTCTTGTGCGTTC

Positions	-100 bp flanking sequences	SNPs within *kabuli* cultivar	+100 bp flanking sequences
Ca_LG_5:16580363	GGACAAAGTGGTCAAACTCATCGTGTATGACAGTGCATATACCCACCTAGTTGCGCTGCAACGAAAAGGAAGCTTCAATGTTGCATTTGACTTGACCTTG	G/A	CACTGGTGCAATCCAATGTGTTAGCAGCTTGGTTGTCTCATTGCTGATGCAACAACTTTTGCATTTGTTAGTCCATGCACAACTCTCTTGTGCGTTCATG
Ca_LG_5:16580365	ACAAAGTGGTCAAACTCATCGTGTATGACAGTGCATATACCCACCTAGTTGCGCTGCAACGAAAAGGAAGCTTCAATGTTGCATTTGACTTGACCTTGCA	A/T	CTGGTGCAATCCAATGTGTTAGCAGCTTGGTTGTCTCATTGCTGATGCAACAACTTTTGCATTTGTTAGTCCATGCACAACTCTCTTGTGCGTTCATGAA
Ca_LG_5:16580377	AACTCATCGTGTATGACAGTGCATATACCCACCTAGTTGCGCTGCAACGAAAAGGAAGCTTCAATGTTGCATTTGACTTGACCTTGCACTGGTGCAATCC	C/T	AATGTGTTAGCAGCTTGGTTGTCTCATTGCTGATGCAACAACTTTTGCATTTGTTAGTCCATGCACAACTCTCTTGTGCGTTCATGAACCATAAAAACAT
Ca_LG_5:16580382	ATCGTGTATGACAGTGCATATACCCACCTAGTTGCGCTGCAACGAAAAGGAAGCTTCAATGTTGCATTTGACTTGACCTTGCACTGGTGCAATCCAATGT	T/C	GTTAGCAGCTTGGTTGTCTCATTGCTGATGCAACAACTTTTGCATTTGTTAGTCCATGCACAACTCTCTTGTGCGTTCATGAACCATAAAAACATCTTCT
Ca_LG_5:16580404	CCCACCTAGTTGCGCTGCAACGAAAAGGAAGCTTCAATGTTGCATTTGACTTGACCTTGCACTGGTGCAATCCAATGTGTTAGCAGCTTGGTTGTCTCAT	T/C	TGCTGATGCAACAACTTTTGCATTTGTTAGTCCATGCACAACTCTCTTGTGCGTTCATGAACCATAAAAACATCTTCTACTATGTCATCCCAAATATGAT
Ca_LG_5:16580406	CACCTAGTTGCGCTGCAACGAAAAGGAAGCTTCAATGTTGCATTTGACTTGACCTTGCACTGGTGCAATCCAATGTGTTAGCAGCTTGGTTGTCTCATTG	G/A	CTGATGCAACAACTTTTGCATTTGTTAGTCCATGCACAACTCTCTTGTGCGTTCATGAACCATAAAAACATCTTCTACTATGTCATCCCAAATATGATTG
Ca_LG_5:17127789	NNNNNNNNNNNNNNNNNNNNNNNNNNNNNNNNNNNNNNNNNNNNNNNNNNNNNNNNNNNNNNNNNNNNNNNNNNNNNNNNNNNNNNNCAGGGAATTTTT	T/C	AGACTTTAAAGTTGAACTGGAAAGCATCCAATTCTTCCTTAAGGATGCTGATAAAAGAGTTGCAGCTGATGGAGACAACAACAATGAATGAATCAAAATA
Ca_LG_5:17159396	TTTCATCCATTAATATACAAGTTGAGAACATAAGTTTCACAGCTACAAGCGAGAAGGTTGAGAGTTAACAGGGAAATAGGAAACAACAATAGGAGCTGTA	A/T	GATCGCTGAGGATCCCCTTTGGGTTTTCTTGACAAAGAACCTGCTTCCACCACCACAGGAGTAGTACTCTTTTCTTCTTTGGAATTAAAGCAGCAGAATA
Ca_LG_5:19770007	ATGAAATCCAGGATTTTGATGTAACATTTGGCATCTATGGAAGTGTCATAGGCTGCACAACTGGATGCATCTATGAAATAATATAGTTAATGTAAGTTAT	T/G	ATTTTTTTCACATGTTTACTACCTATACTGATGCATAAACGGATTGAAACTAAAAGCTTACGTTATTTATTGTTGAAGAAATAAAAATGTACATTTACAA
Ca_LG_5:19770036	GGCATCTATGGAAGTGTCATAGGCTGCACAACTGGATGCATCTATGAAATAATATAGTTAATGTAAGTTATATTTTTTTCACATGTTTACTACCTATACT	T/A	GATGCATAAACGGATTGAAACTAAAAGCTTACGTTATTTATTGTTGAAGAAATAAAAATGTACATTTACAAACTTATATGTTTAGATGTACTAATGAATT
Ca_LG_5:19770058	GCTGCACAACTGGATGCATCTATGAAATAATATAGTTAATGTAAGTTATATTTTTTTCACATGTTTACTACCTATACTGATGCATAAACGGATTGAAACT	T/C	AAAAGCTTACGTTATTTATTGTTGAAGAAATAAAAATGTACATTTACAAACTTATATGTTTAGATGTACTAATGAATTTGTGTAGATTTACATGACACTT
Ca_LG_5:19879470	CTCAAGGAGTCACAAAAGACCACATTAAGCTGAAAGCCTTCCCATCTTCACTCTAAGATGCTGCCAAAGACTGGTTATACTACCTTCAATCAGGTTCTGT	T/C	TACAAGTTGGAATGATCTCAAGATATTGCTTCTAGAAAAATTCTTCCCTACCTCAAGAGCTGCTTCAATCAGAAAAGTAATATATGGCAGCAGGCAGATT

**Positions**	**-100 bp flanking sequences**	**SNPs within *kabuli* cultivar**	**+100 bp flanking sequences**
Ca_LG_5:19879484	AAAGACCACATTAAGCTGAAAGCCTTCCCATCTTCACTCTAAGATGCTGCCAAAGACTGGTTATACTACCTTCAATCAGGTTCTGTTACAAGTTGGAATG	G/A	ATCTCAAGATATTGCTTCTAGAAAAATTCTTCCCTACCTCAAGAGCTGCTTCAATCAGAAAAGTAATATATGGCAGCAGGCAGATTGACATGGAATCATT
Ca_LG_5:20737748	TATAATCATAGTTTCTCTTTTCCAACATTCCGACACGTTTCACTCGATTTCCATTCAATTTGTTACTGTCGCCGTTGCCACTATTTCCGGCAAATTTTCT	T/C	ATCGAACAACAGTCATCTTCAGATCAAGTCATGACCAAAAGCGCGTCGCCAAAAACCGTCACACGCGCTGCAAATCTCTCATGCGAGTAGATATGTCGCT
Ca_LG_5:20737822	TTGCCACTATTTCCGGCAAATTTTCTATCGAACAACAGTCATCTTCAGATCAAGTCATGACCAAAAGCGCGTCGCCAAAAACCGTCACACGCGCTGCAAA	A/G	TCTCTCATGCGAGTAGATATGTCGCTGCTGCCGCTACTGCCTGTCGTTGCTGCTGTCACGTCTCCTACTGCTGCCATCTGTGGTTACTGCCTGTCGTTGC
Ca_LG_5:20737850	CGAACAACAGTCATCTTCAGATCAAGTCATGACCAAAAGCGCGTCGCCAAAAACCGTCACACGCGCTGCAAATCTCTCATGCGAGTAGATATGTCGCTGC	C/T	TGCCGCTACTGCCTGTCGTTGCTGCTGTCACGTCTCCTACTGCTGCCATCTGTGGTTACTGCCTGTCGTTGCTGCTGTCACGTCTCCTACTGCTGCCATC
Ca_LG_5:20737854	CAACAGTCATCTTCAGATCAAGTCATGACCAAAAGCGCGTCGCCAAAAACCGTCACACGCGCTGCAAATCTCTCATGCGAGTAGATATGTCGCTGCTGCC	C/T	GCTACTGCCTGTCGTTGCTGCTGTCACGTCTCCTACTGCTGCCATCTGTGGTTACTGCCTGTCGTTGCTGCTGTCACGTCTCCTACTGCTGCCATCTGTG
Ca_LG_5:20737863	TCTTCAGATCAAGTCATGACCAAAAGCGCGTCGCCAAAAACCGTCACACGCGCTGCAAATCTCTCATGCGAGTAGATATGTCGCTGCTGCCGCTACTGCC	C/T	TGTCGTTGCTGCTGTCACGTCTCCTACTGCTGCCATCTGTGGTTACTGCCTGTCGTTGCTGCTGTCACGTCTCCTACTGCTGCCATCTGTGGTTGCTGCA
Ca_LG_5:20737866	TCAGATCAAGTCATGACCAAAAGCGCGTCGCCAAAAACCGTCACACGCGCTGCAAATCTCTCATGCGAGTAGATATGTCGCTGCTGCCGCTACTGCCTGT	T/C	CGTTGCTGCTGTCACGTCTCCTACTGCTGCCATCTGTGGTTACTGCCTGTCGTTGCTGCTGTCACGTCTCCTACTGCTGCCATCTGTGGTTGCTGCATGT
Ca_LG_5:21032521	ACTTAATTCAAAAGTTAGCACCAAAACTTTAGTTCATCTCATATCTGAAACGATGTAACCTATACTCCAAATTGATCACGATTATATTTTCTTTTGAATA	A/T	TATTCTCACTTAAAGGTTACAAAAAACTAAATCATATATTTCCAAGTAGCGGACAAGATAAACTCGTTTGACAAATTAAAATAATTTTTCTAGGTTAATA
Ca_LG_5:21032525	AATTCAAAAGTTAGCACCAAAACTTTAGTTCATCTCATATCTGAAACGATGTAACCTATACTCCAAATTGATCACGATTATATTTTCTTTTGAATATATT	T/C	CTCACTTAAAGGTTACAAAAAACTAAATCATATATTTCCAAGTAGCGGACAAGATAAACTCGTTTGACAAATTAAAATAATTTTTCTAGGTTAATACCAT
Ca_LG_5:22170319	CATCACCATATTTTTAAACAATTGCTCATCCAATCATTTTATGAAGGTATGCTACCTATGGATAGAAGCATTTTGGATGCTGCTAGTGGGGGAGCACTTG	G/A	TCGATAAGACCCCAGAAGCGGCAAAAGCTTTGATTGAGAACATGTCCATTAATTCTCAGTAGTTTACAACTAGAAGCAATTCAGTAGTGTTGATAAGGGG
Ca_LG_5:22170327	TATTTTTAAACAATTGCTCATCCAATCATTTTATGAAGGTATGCTACCTATGGATAGAAGCATTTTGGATGCTGCTAGTGGGGGAGCACTTGTCGATAAG	G/A	ACCCCAGAAGCGGCAAAAGCTTTGATTGAGAACATGTCCATTAATTCTCAGTAGTTTACAACTAGAAGCAATTCAGTAGTGTTGATAAGGGGTATGAATG
Ca_LG_5:22170374	CTATGGATAGAAGCATTTTGGATGCTGCTAGTGGGGGAGCACTTGTCGATAAGACCCCAGAAGCGGCAAAAGCTTTGATTGAGAACATGTCCATTAATTC	C/T	TCAGTAGTTTACAACTAGAAGCAATTCAGTAGTGTTGATAAGGGGTATGAATGAAATTCAAGCTTCTTTCCACAAGAATTTAGAAAGCAGACTTGATGAG

Positions	-100 bp flanking sequences	SNPs within *kabuli* cultivar	+100 bp flanking sequences
Ca_LG_5:24179245	GATTTCTTGCAGCCAAAGATCAGCCTTGTCGGGGTCATGTTCCCCTTTAAATTTAGGCGAATCTTGTCGACGAAATTCATTCAATGTTCTTGCTGCACTG	G/A	GCTGCTTCATCCCTCTAATCTCTTTGTGCATCTCGTTGAGCCTAGACCGCAGCTTGTTGGGCCATTGCTGCGGCCATTGCATTCATTGCTTGGATTGCTT
Ca_LG_5:24179265	CAGCCTTGTCGGGGTCATGTTCCCCTTTAAATTTAGGCGAATCTTGTCGACGAAATTCATTCAATGTTCTTGCTGCACTGGCTGCTTCATCCCTCTAATC	C/A	TCTTTGTGCATCTCGTTGAGCCTAGACCGCAGCTTGTTGGGCCATTGCTGCGGCCATTGCATTCATTGCTTGGATTGCTTCAATCATGTCGTTTCTTCCT
Ca_LG_5:24179266	AGCCTTGTCGGGGTCATGTTCCCCTTTAAATTTAGGCGAATCTTGTCGACGAAATTCATTCAATGTTCTTGCTGCACTGGCTGCTTCATCCCTCTAATCT	T/C	CTTTGTGCATCTCGTTGAGCCTAGACCGCAGCTTGTTGGGCCATTGCTGCGGCCATTGCATTCATTGCTTGGATTGCTTCAATCATGTCGTTTCTTCCTT
Ca_LG_5:24179269	CTTGTCGGGGTCATGTTCCCCTTTAAATTTAGGCGAATCTTGTCGACGAAATTCATTCAATGTTCTTGCTGCACTGGCTGCTTCATCCCTCTAATCTCTT	T/A	TGTGCATCTCGTTGAGCCTAGACCGCAGCTTGTTGGGCCATTGCTGCGGCCATTGCATTCATTGCTTGGATTGCTTCAATCATGTCGTTTCTTCCTTGGG
Ca_LG_5:24179293	AAATTTAGGCGAATCTTGTCGACGAAATTCATTCAATGTTCTTGCTGCACTGGCTGCTTCATCCCTCTAATCTCTTTGTGCATCTCGTTGAGCCTAGACC	C/T	GCAGCTTGTTGGGCCATTGCTGCGGCCATTGCATTCATTGCTTGGATTGCTTCAATCATGTCGTTTCTTCCTTGGGGAACCGATTGGTTTGTGCGACGAG
Ca_LG_5:24179297	TTAGGCGAATCTTGTCGACGAAATTCATTCAATGTTCTTGCTGCACTGGCTGCTTCATCCCTCTAATCTCTTTGTGCATCTCGTTGAGCCTAGACCGCAG	G/T	CTTGTTGGGCCATTGCTGCGGCCATTGCATTCATTGCTTGGATTGCTTCAATCATGTCGTTTCTTCCTTGGGGAACCGATTGGTTTGTGCGACGAGTCAT
Ca_LG_5:24906908	TATGATGATACTTTCATTTTTTGGAGCTTGATATGTTTTCAATATGATTTAAAATTGTGGTCTACAATTACAATTGCAATTGCAGCTGCATTGGCTGCAA	A/T	ATGTCAATATTTTACTGTAATGTAAAAGTTCGAAGCATAGCTGCAATCACAATTTAAAATATGATTTGCATTTTGCTACGTGTAGAAATGAATGAAGGAC
Ca_LG_5:24961066	AATCCGTGCTCTTCATACACAGATAAGGGATTTGAAACTTCAGATCTTCTTGTCTCAACCATTCTATTGTGCTGCTCAGAAACGCAATTGAAATTCCTCA	A/C	TATTTGCAGGAGTATCAAAAGAAAATTCCACATGAGATGCTGGTGGTGGATTTAATCTTGCTTGCTCTTGATCCTTCTCTTGAGCAGAGACAGCTGCAAA
Ca_LG_5:24961088	ATAAGGGATTTGAAACTTCAGATCTTCTTGTCTCAACCATTCTATTGTGCTGCTCAGAAACGCAATTGAAATTCCTCATATTTGCAGGAGTATCAAAAGA	A/T	AAATTCCACATGAGATGCTGGTGGTGGATTTAATCTTGCTTGCTCTTGATCCTTCTCTTGAGCAGAGACAGCTGCAAACTTTGATGCTAGCTTTTCACGC
Ca_LG_5:25167090	GCAGAACCAAAACAAGCAGAACCAGAACAAGCAGCACATCAAAATGCAGAACCAGAACAAGCAGAACCAGAACAAGCAGCACAGAATAATGCAGAACCAA	A/G	AACAAGCAGAACCAGAACAAGCAGCACATCAAAATGCAGAACCAGAACAAGCAGCACATAATAATGCAGAACCAGAACAAGCACATCAAAATGTAGAACA
Ca_LG_5:25167184	AACCAAAACAAGCAGAACCAGAACAAGCAGCACATCAAAATGCAGAACCAGAACAAGCAGCACATAATAATGCAGAACCAGAACAAGCACATCAAAATGT	T/C	AGAACAAGCACATCAAAATGCAGAACAATGCAGAACATCACAATGTAGAACAAGCAGCACATCAAAATGCAGAATAATGCAGAACAAGCAGCACATCAAA
Ca_LG_5:25167186	CCAAAACAAGCAGAACCAGAACAAGCAGCACATCAAAATGCAGAACCAGAACAAGCAGCACATAATAATGCAGAACCAGAACAAGCACATCAAAATGTAG	G/A	AACAAGCACATCAAAATGCAGAACAATGCAGAACATCACAATGTAGAACAAGCAGCACATCAAAATGCAGAATAATGCAGAACAAGCAGCACATCAAAAT

Positions	-100 bp flanking sequences	SNPs within *kabuli* cultivar	+100 bp flanking sequences
Ca_LG_5:25167230	ACCAGAACAAGCAGCACATAATAATGCAGAACCAGAACAAGCACATCAAAATGTAGAACAAGCACATCAAAATGCAGAACAATGCAGAACATCACAATGT	T/C	AGAACAAGCAGCACATCAAAATGCAGAATAATGCAGAACAAGCAGCACATCAAAATCCAGAATTGAACTAGCAGAATAAAACCAGAACAAGCACAAGAAT
Ca_LG_5:25415579	TTCTGGTTTTATTTTGATGTGCTGCTGGTTTTATTCTGATGGTTCTGTGCTGGTTCTGCTGGTTTTATTCTGGTTTTATTTTGATGTGCTGCTGGTTTTA	A/T	TTCTGTTGGTTCTGTGCTGGTTCTGCTGGTTTTGATGTGCTGCTGGTTTTGTGCTGGTTCTGCTGGTTTTGTTGTGGTTTAGTTTTTTTGACATGTTTTT
Ca_LG_5:25415585	TTTTATTTTGATGTGCTGCTGGTTTTATTCTGATGGTTCTGTGCTGGTTCTGCTGGTTTTATTCTGGTTTTATTTTGATGTGCTGCTGGTTTTATTCTGT	T/C	TGGTTCTGTGCTGGTTCTGCTGGTTTTGATGTGCTGCTGGTTTTGTGCTGGTTCTGCTGGTTTTGTTGTGGTTTAGTTTTTTTGACATGTTTTTGGTTCT
Ca_LG_5:26747814	TATGGAATCAGCAAATATAGATACTTCAGTGAAAAAAGGGAATATAATATCAGTTGATAAGGATGCAGCTCCAGGGCAAGCTTCTGATTCTTGTGTCGAC	C/T	GCCGAGTTGGCATTTAAAGAGAGTGAACAATCTAAGGAGCAGCAAGCATCCATGTTAAAAGAAACCAGTAATTCAAAATATGTTTCTTGGGATGGCGACA
Ca_LG_5:27299558	GAGAAATCATTTCTCTCTTTTTCTCTCTTCTCTCTCTTTCTCTCTTCTCCATCTTCTCTCTTCGTTTCTCTATCTTGGTCGTTCTCGCTTGCTGCATTCG	G/A	ATTATGCCGATCTTCTCGCTTGCTACATTTGATTATGCCGATCTTCTCGCTTGCTGCATCTGTGCTGACATTTTGCCTGTATTTTTCCTTCTTTCTTTCT
Ca_LG_5:27299562	AATCATTTCTCTCTTTTTCTCTCTTCTCTCTCTTTCTCTCTTCTCCATCTTCTCTCTTCGTTTCTCTATCTTGGTCGTTCTCGCTTGCTGCATTCGATTA	A/C	TGCCGATCTTCTCGCTTGCTACATTTGATTATGCCGATCTTCTCGCTTGCTGCATCTGTGCTGACATTTTGCCTGTATTTTTCCTTCTTTCTTTCTTTCT
Ca_LG_5:27299565	CATTTCTCTCTTTTTCTCTCTTCTCTCTCTTTCTCTCTTCTCCATCTTCTCTCTTCGTTTCTCTATCTTGGTCGTTCTCGCTTGCTGCATTCGATTATGC	C/T	CGATCTTCTCGCTTGCTACATTTGATTATGCCGATCTTCTCGCTTGCTGCATCTGTGCTGACATTTTGCCTGTATTTTTCCTTCTTTCTTTCTTTCTGGT
Ca_LG_5:27299583	TCTTCTCTCTCTTTCTCTCTTCTCCATCTTCTCTCTTCGTTTCTCTATCTTGGTCGTTCTCGCTTGCTGCATTCGATTATGCCGATCTTCTCGCTTGCTA	A/G	CATTTGATTATGCCGATCTTCTCGCTTGCTGCATCTGTGCTGACATTTTGCCTGTATTTTTCCTTCTTTCTTTCTTTCTGGTTAAGTTGTCATTTTATTT
Ca_LG_5:27299606	CCATCTTCTCTCTTCGTTTCTCTATCTTGGTCGTTCTCGCTTGCTGCATTCGATTATGCCGATCTTCTCGCTTGCTACATTTGATTATGCCGATCTTCTC	C/T	GCTTGCTGCATCTGTGCTGACATTTTGCCTGTATTTTTCCTTCTTTCTTTCTTTCTGGTTAAGTTGTCATTTTATTTTAAGTCATTAACCATATTTATAT
Ca_LG_5:27412391	AAACATTCAAGATGACGAAGTAAAAGCAAGCACGAAAAAGTATACCTCCATGATCTGGAATTTTTTTCAAAATGGCATCGGCTGCTTTGAGTTCCTTTTT	T/C	GTATTGTTTAGTTTCAAAGTATACATCTACATTGTTTTCTGTTACTGATGCATGTAAGCATCAGATCTCTCATTGTAACAAGTATTTGAAAAATCACAAA
Ca_LG_5:27657951	ACAAGCCACAGGCATAAATGTAATCAAAGTCTGCTTTTGGCATATGCTGTTGCAGCTGCTGCAGCATTGAGTTTGTCAAATCCTTTTGTAATGGAATACA	A/G	AAGGAAATGATAGCAGCAGAGATTCAGAGATGACTGAGAAATCTAGCATGGGAGGCAATGATAAGGACATTGATAAAAAAGAAAAGACAAGGAGAAAGAA
Ca_LG_5:28037026	AAGGAAGGCGGCCACATTACCTAAGGGAGGCGGCCACAAAGCTCAGACTCAGTGGCTAGGCAACAAACAGAAGGAATGAGAACGAAGAAAGACGAAGACA	A/G	GCGATGGCTAGGATGACATCACCGACGACAACGACAATACGGTGGCAGCACGACAATGTCAAAATGCATGGTTGCCGTTGACAGCGACGACAGTGACCTT

Positions	-100 bp flanking sequences	SNPs within *kabuli* cultivar	+100 bp flanking sequences
Ca_LG_5:28037048	AAGGGAGGCGGCCACAAAGCTCAGACTCAGTGGCTAGGCAA<br>CAAACAGAAGGAATGAGAACGAAGAAAGACGAAGACAGCGAT<br>GGCTAGGATGACATCAC	C/A	CGACGACAACGACAATACGGTGGCAGCACGACAATGTCAAA<br>ATGCATGGTTGCCGTTGACAGCGACGACAGTGACCTTCTCT<br>TCTTATTGCTCATTTGCT
Ca_LG_5:28244603	AATATTTGCATGTACTGTTGGACAAAAACCAAGCAAAGCTAGA<br>TACTACTTGGATGATAGTGTGAAAGTGATGATGTTACTTCAAG<br>GTCTTGGTGCTGCA	A/T	GCTGCTGAGGCTGCTGCAAAAGTTTCTGCTGAAACCTCTTCT<br>GAAGTTTGTTCTGAAGATGTTGTTTGAAAATGTGCACTATTTT<br>TGCTGTCTTGACAAC
Ca_LG_5:29178061	AAATAGAAACCGCAATGAAGGTGGGTGGTAGGTTTATGCGGC<br>AAATTTATGCGGAAGCAAATAGGTATAAAAAAATCTCAAGTTG<br>CTGCAATGAAGGCAA	A/G	CAAAAAGGCAAAAAGAAATCTCACGTTGCTGCAATGAAGGC<br>AGTAAAAAGGGCCATTCCATTCTCGAGCATGTTCAATCGTCC<br>TCCCCAAGTGCTTGTTC
Ca_LG_5:30436997	TCACTGGTTCACATGAGCAGAGATGAATCAACTAAATGATGTA<br>AGCTAATCTCTAACTGATTTAAAAAGGCCTATAGTCAAGTAAT<br>TTTCATCTATATTC	C/T	TCCTATTTTTGGCACCTCTAACTTTAATCTGGGATATCTATTA<br>AAACATTGATCAATAGGTAATGCTACTAAACAATATCAACTTG<br>TTTCAATTGGATAT
Ca_LG_5:30437044	AATCTCTAACTGATTTAAAAAGGCCTATAGTCAAGTAATTTTCA<br>TCTATATTCTCCTATTTTTGGCACCTCTAACTTTAATCTGGGAT<br>ATCTATTAAAAC	C/T	ATTGATCAATAGGTAATGCTACTAAACAATATCAACTTGTTTC<br>AATTGGATATGATCTTGATTTCTAAGTCACCCTCCATTCCGA<br>AATTTTCCAACAGCT
Ca_LG_5:31834738	TGAGTGAAAAGTATCATGTAGTGAGGAATGTCTCGAATCTCAT<br>GTTAGACAACTAAATGCATCTCGAGGTGATGTAGAGGAACTG<br>GGGGACCTTCTCGAA	A/G	CACATGATAGGCAATTGAGAGACATTTGTTTTCTCTGACACA<br>TATTGTTTAGTTGCACTTAATAGGTCGACTTTGTCTCTAGCG<br>GCCAACCACTCATAAT
Ca_LG_5:31834743	GAAAAGTATCATGTAGTGAGGAATGTCTCGAATCTCATGTTAG<br>ACAACTAAATGCATCTCGAGGTGATGTAGAGGAACTGGGGGA<br>CCTTCTCGAACACAT	T/G	GATAGGCAATTGAGAGACATTTGTTTTCTCTGACACATATTG<br>TTTAGTTGCACTTAATAGGTCGACTTTGTCTCTAGCGGCCAA<br>CCACTCATAATTGGTC
Ca_LG_5:32180340	CTAATACCCCAAGAGATGATCCATGATCTACATACAGAACCTC<br>GTAATCACACTGATGATTCGGGTCATCACAGTGATGATTTTGA<br>ATTGCAGCGCATAC	C/T	GGGATCCGTACATTTCACAATGTTATTATGCGGTTTATAAAG<br>TTGTTCTACAGGCTGCAAACAAACCAAGTTCAATTCAAAATT<br>ACCATAACATCATTCC
Ca_LG_5:32180391	ACTGATGATTCGGGTCATCACAGTGATGATTTTGAATTGCAGC<br>GCATACGGGATCCGTACATTTCACAATGTTATTATGCGGTTTA<br>TAAAGTTGTTCTAC	C/T	AGGCTGCAAACAAACCAAGTTCAATTCAAAATTACCATAACA<br>TCATTCCTTAGTAGTAACAAAATATTGTAAGGTAAAACATACC<br>TTGGTGCAACCTGTA
Ca_LG_5:32187420	GAACTTATTGTCATTTGTAAATAAATATGCTGCTAGTGGCTTT<br>GTTCCGGAGTTTATCACATCGAAGCTTTCTTCCAGCTTATTCA<br>CCTGTTCCAGGCAA	A/C	AAAGCAAGATTATAATTTGATACAAGCTGATGAAGGGTATCG<br>ATGAATATTCGAAGTATAAATACATGTTTCCAAGTTATTAGTT<br>AAGCAATTAGCTAAA
Ca_LG_5:33219423	TACTCTTGCTTGAGAAAATCCCAAATTTCTATGGCTAACTTTAG<br>AGCCATAATTTTTGTAAAGATTACTTCAGAAGCAACATAAAATA<br>GATAAGATTTTG	G/T	TCTTCGATTTTTGATGTTTTCTATCCTCGTGATTTTTTATTTGT<br>GCAAGCATTGGATTGTCTGATGTTGGATCTATCACACAATCA<br>TTCTCAGCAGCCTC
Ca_LG_5:33219428	TTGCTTGAGAAAATCCCAAATTTCTATGGCTAACTTTAGAGCC<br>ATAATTTTTGTAAAGATTACTTCAGAAGCAACATAAAATAGATA<br>AGATTTTGTCTTC	C/T	GATTTTTGATGTTTTCTATCCTCGTGATTTTTTATTTGTGCAA<br>GCATTGGATTGTCTGATGTTGGATCTATCACACAATCATTCT<br>CAGCAGCCTCCCAAA

Positions	-100 bp flanking sequences	SNPs within *kabuli* cultivar	+100 bp flanking sequences
Ca_LG_5:33219445	AAATTTCTATGGCTAACTTTAGAGCCATAATTTTTGTAAAGATTACTTCAGAAGCAACATAAAATAGATAAGATTTTGTCTTCGATTTTTGATGTTTTCT	T/C	ATCCTCGTGATTTTTTATTTGTGCAAGCATTGGATTGTCTGATGTTGGATCTATCACACAATCATTCTCAGCAGCCTCCCAAACACCATTAGCATCAAGA
Ca_LG_5:33219466	GAGCCATAATTTTTGTAAAGATTACTTCAGAAGCAACATAAAATAGATAAGATTTTGTCTTCGATTTTTGATGTTTTCTATCCTCGTGATTTTTTATTTG	G/C	TGCAAGCATTGGATTGTCTGATGTTGGATCTATCACACAATCATTCTCAGCAGCCTCCCAAACACCATTAGCATCAAGATAAGCCTCAATTCTGGTTCCT
Ca_LG_5:33219485	GATTACTTCAGAAGCAACATAAAATAGATAAGATTTTGTCTTCGATTTTTGATGTTTTCTATCCTCGTGATTTTTTATTTGTGCAAGCATTGGATTGTCT	T/C	GATGTTGGATCTATCACACAATCATTCTCAGCAGCCTCCCAAACACCATTAGCATCAAGATAAGCCTCAATTCTGGTTCCTCATATGTGATAGTTCCCAC
Ca_LG_5:33219489	ACTTCAGAAGCAACATAAAATAGATAAGATTTTGTCTTCGATTTTTGATGTTTTCTATCCTCGTGATTTTTTATTTGTGCAAGCATTGGATTGTCTGATG	G/T	TTGGATCTATCACACAATCATTCTCAGCAGCCTCCCAAACACCATTAGCATCAAGATAAGCCTCAATTCTGGTTCCTCATATGTGATAGTTCCCACCTTA
Ca_LG_5:33219497	AGCAACATAAAATAGATAAGATTTTGTCTTCGATTTTTGATGTTTTCTATCCTCGTGATTTTTTATTTGTGCAAGCATTGGATTGTCTGATGTTGGATCT	T/G	ATCACACAATCATTCTCAGCAGCCTCCCAAACACCATTAGCATCAAGATAAGCCTCAATTCTGGTTCCTCATATGTGATAGTTCCCACCTTAAAAAAATTG
Ca_LG_5:33405843	AAGGGTGGAAAACGTGATTGGAGGCCATTCAAATCTGTGGATGAAGAAGGAAGAGGGAAGAAAAATGAGAGCAGCAATGGAATTAGAGTAGAAAATGAAC	C/T	ACGAAATTGGTCATCCCTTGGTTCACTCACCTAGCAATGAAGAACATAAATATATACCAGTATCCCATTGGAAAGCTGAGGATGCAGCCAGTTGGCATAA
Ca_LG_5:33412168	GCAAAGACCTGAAGACATGAGTTATGTTAGACCAGTTTCGGTTTGTGATGGTTCTGCTACCGATTTAGCTGGTGAAATTGTGGCAGCATTATCTGCGGCG	G/A	TCGATGGTATTCAGAGAAGACCAAGATTACTCAGGGAAACTAGTTCAAGCAGCAGAAAGCCTATATGAGGCAGTTACAAAGGAAGATCCTAAAAAGCAAG
Ca_LG_5:33760675	NNNNNNNNNNNNNNNNNNNNNNNNNNNNNNNNNNNGCATTTGTGGCGGCCAAAGCAGCCACAATGCCAACGGATACTATCCATTTGTGACGGCCTGAGCGGT	T/C	CAGAAATGCCAGCTGTATTGGGCCTCCTTTGTGGCGGCATTTGCAGCCACAAATTCCACGTGCGCATTTCTTTGTGGCGACCTTTGCAGCCACAAATGCC
Ca_LG_5:35490898	ATTTAAGATGGTATCAGAGCTCCTGATCCTTGGGAGCCTCTGACCGTGCCTGTAAACCCTAAGTTGCAGCCTTCATTGTTGCGCCACCTTTAAGCATTGC	C/T	TGCAGCCTTCATTGCAGCGCAACCCTCCGTGCCTGTAAACCCTAAGTTGCAGCCTTCATTGCTGCGCTACCTTTAAGCATTGTTGCAGCCTTCATTGCAG
Ca_LG_5:37312384	ATGTAACAGGTATAAAACTTTGTGGCGCCCTGGGCGGCCAAAAAATGCCATGTAACTTTATTCTTTTGTGGCGGCATTTGCAACCACAAAATGCAAAGGC	C/A	TCAGGCTTTTGTGGCGGCCTTTGCAGCCACAAAATGTAATGTCCCTTCAGACATTTGTGGCGGCCTTTACCCTCACAAAATTTAGATTTTGTGGCTGTAA
Ca_LG_5:37838689	GAACGTCGTTCCGTGGAATATATGACTCATGCTCCTTTAGGTTCTTTAAATCTCGTTGGCATAGCTACAGAGATTAATGCAGTCAATTATGTCTCTCCTG	G/A	GAAGTTGGTTAGCTACTTCTCATTTTGTTCTAGAATTCTTCCTATTTGTAGGTCATTTATGGAACACGAGAAGAGCTCACACAGCTGCCACAGAATTTGA
Ca_LG_5:37838723	CTTTAGGTTCTTTAAATCTCGTTGGCATAGCTACAGAGATTAATGCAGTCAATTATGTCTCTCCTGGAAGTTGGTTAGCTACTTCTCATTTTGTTCTAGA	A/G	ATTCTTCCTATTTGTAGGTCATTTATGGAACACGAGAAGAGCTCACACAGCTGCCACAGAATTTGAAAACGGAATTGATCGAGATTTTGAGACTGTCCTT

Positions	-100 bp flanking sequences	SNPs within *kabuli* cultivar	+100 bp flanking sequences
Ca_LG_5:37838736	AAATCTCGTTGGCATAGCTACAGAGATTAATGCAGTCAATTATGTCTCTCCTGGAAGTTGGTTAGCTACTTCTCATTTTGTTCTAGAATTCTTCCTATTT	T/C	GTAGGTCATTTATGGAACACGAGAAGAGCTCACACAGCTGCCACAGAATTTGAAAACGGAATTGATCGAGATTTTGAGACTGTCCTTTCCATGACTCCTC
Ca_LG_5:38476695	GGAAAGCAGCACCGAAAAGTGATGTCATTCCAATGACACAAAACAACCACGCATAGATGCTAGGCCCATCCATACTTTGCCTTTTCCTCTGCTGCATTCT	T/C	TCTTCTTCGATAACTTCAACTGAATCTCATTCTTTTACGAGATCTCATTTCTCTCTTTTATAATAAAATACTGAAGTTGACTAACATATTAGAATTTAAC
Ca_LG_5:38569638	AAATCATCATTATATTGCTACCTAAGGAGATTTAATTTGTTAATGTCTCTTACTTACAGTGAACAGAGTATGCAGCATTTTTATTGGCACTTGGCATTAT	T/C	ATATCTATAAGATCTTTATGATAACAGGATGTGCATTATAGTTTTCTTGAAAAATTAATGTGCTGCAAACCAGGATCTCACTTATTGCTTGAATTTGAAT
Ca_LG_5:39945759	GTTGTGTCAAGTGTTCAAAATTTAAACTTGTTGTTAAAATTGAATTTTATAAGAGAAAGAAGGTAGATCACTTATTCAATTTTTAGGGGTTAGTTAACCT	T/A	GCCAAACCTAAAGACACCATGCAAAAAGGCTTCAGTGTGGTAACTTCTTTACGGCGGGCCATGGCAGCTGCGGCATCCATGGTGGAAAAACTGAAAAACA
Ca_LG_5:40018134	TATTCCCTCACTCTCTTTTTTTCCTTTACTTTTGTTCTCTCTCTCTCTCTTTTTCACCCTAACCCCACGACAGCAGCCTTGCAGGCGCCTCCTTCCTTCT	T/C	TTCTTCGTATTGTGCCGCCCTCGTTTTCCCTCACTCACCAGAAAACTCTCTTGATTTCCCTCTCTTTCCCCCTCATTCACTAGCTCTCAATTTTCCTTGT
Ca_LG_5:40018146	CTCTTTTTTTCCTTTACTTTTGTTCTCTCTCTCTCTCTTTTTCACCCTAACCCCACGACAGCAGCCTTGCAGGCGCCTCCTTCCTTCTTTCTTCGTATTG	G/T	TGCCGCCCTCGTTTTCCCTCACTCACCAGAAAACTCTCTTGATTTCCCTCTCTTTCCCCCTCATTCACTAGCTCTCAATTTTCCTTGTTCTCACTGCCGC
Ca_LG_5:40018181	TCTTTTTCACCCTAACCCCACGACAGCAGCCTTGCAGGCGCCTCCTTCCTTCTTTCTTCGTATTGTGCCGCCCTCGTTTTCCCTCACTCACCAGAAAACT	T/C	CTCTTGATTTCCCTCTCTTTCCCCCTCATTCACTAGCTCTCAATTTTCCTTGTTCTCACTGCCGCGAACCCAGGCGTTGCCTTCGCCCAAAGTCGCGAAC
Ca_LG_5:40026045	TGCTCTGAATCGAACCGCTTTATCGTGCAATCGCGGTGCGGAGTGGAGATCTTCTGCGATGCAGGTCTCTCGATCGGCTATCGCGGCCGCAAATCGCGCT	T/G	GGAATCGCATCCTCGCGCGCGGTACAGGACAGAGGAAAGAAATCCGAAAATGAAGAATGAAAAAAGGGAAACCTGGATTACTTTGGACACTGTTTCTGGC
Ca_LG_5:40995033	TTTAAGTGGTGTTTTCAGTGGCAGACGAGTGAGTAACACGTAAGAACCTGCCCATATGAGGGGGACAACAGCTGGAAACAATTGCTAATACCCCATAGGT	T/C	TGAGGAGCAAAAGGAGGAATCTGCCCACGGAGGGGCTCACGTTTGATTAGCTAGTTGATGAGGTAATAGCTTACCAAGGCGATGATCAGTAGCTAGTTCG
Ca_LG_5:40995055	AGACGAGTGAGTAACACGTAAGAACCTGCCCATATGAGGGGGACAACAGCTGGAAACAATTGCTAATACCCCATAGGTTGAGGAGCAAAAGGAGGAATCT	T/C	GCCCACGGAGGGGCTCACGTTTGATTAGCTAGTTGATGAGGTAATAGCTTACCAAGGCGATGATCAGTAGCTAGTTCGAGAGGGTGATCAACCACACTGG
Ca_LG_5:40995061	GTGAGTAACACGTAAGAACCTGCCCATATGAGGGGGACAACAGCTGGAAACAATTGCTAATACCCCATAGGTTGAGGAGCAAAAGGAGGAATCTGCCCAC	C/A	GGAGGGGCTCACGTTTGATTAGCTAGTTGATGAGGTAATAGCTTACCAAGGCGATGATCAGTAGCTAGTTCGAGAGGGTGATCAACCACACTGGGACGAG
Ca_LG_5:40995072	GTAAGAACCTGCCCATATGAGGGGGACAACAGCTGGAAACAATTGCTAATACCCCATAGGTTGAGGAGCAAAAGGAGGAATCTGCCCACGGAGGGGCTCA	A/G	CGTTTGATTAGCTAGTTGATGAGGTAATAGCTTACCAAGGCGATGATCAGTAGCTAGTTCGAGAGGGTGATCAACCACACTGGGACGAGAGGATAATCAA

Positions	-100 bp flanking sequences	SNPs within *kabuli* cultivar	+100 bp flanking sequences
Ca_LG_5:40995076	GAACCTGCCCATATGAGGGGGACAACAGCTGGAAACAATTGCTAATACCCCATAGGTTGAGGAGCAAAAGGAGGAATCTGCCCACGGAGGGGCTCACGTT	T/C	TGATTAGCTAGTTGATGAGGTAATAGCTTACCAAGGCGATGATCAGTAGCTAGTTCGAGAGGGTGATCAACCACACTGGGACGAGAGGATAATCAATAGT
Ca_LG_5:40995091	AGGGGGACAACAGCTGGAAACAATTGCTAATACCCCATAGGTTGAGGAGCAAAAGGAGGAATCTGCCCACGGAGGGGCTCACGTTTGATTAGCTAGTTGA	A/G	TGAGGTAATAGCTTACCAAGGCGATGATCAGTAGCTAGTTCGAGAGGGTGATCAACCACACTGGGACGAGAGGATAATCAATAGTTCGCTTCTTTACTGA
Ca_LG_5:40995209	AGGCGATGATCAGTAGCTAGTTCGAGAGGGTGATCAACCACACTGGGACGAGAGGATAATCAATAGTTCGCTTCTTTACTGAGGATGATCGATAATGGGA	A/C	AAAAGCCTGACAGAGCAATGTCGCGTGGAGGAAGAAGGCATACGGGTCGTCAACTTCTTTCCCCGGAGAAGAAACAATGACGATATATAGGAAATAAGCA
Ca_LG_5:40995221	GTAGCTAGTTCGAGAGGGTGATCAACCACACTGGGACGAGAGGATAATCAATAGTTCGCTTCTTTACTGAGGATGATCGATAATGGGAAAAAGCCTGACA	A/G	GAGCAATGTCGCGTGGAGGAAGAAGGCATACGGGTCGTCAACTTCTTTCCCCGGAGAAGAAACAATGACGATATATAGGAAATAAGCAATGACTAACTCT
Ca_LG_5:40995230	TCGAGAGGGTGATCAACCACACTGGGACGAGAGGATAATCAATAGTTCGCTTCTTTACTGAGGATGATCGATAATGGGAAAAAGCCTGACAGAGCAATGT	T/C	CGCGTGGAGGAAGAAGGCATACGGGTCGTCAACTTCTTTCCCCGGAGAAGAAACAATGACGATATATAGGAAATAAGCAATGACTAACTCTATGCAAGCA
Ca_LG_5:40995249	CACTGGGACGAGAGGATAATCAATAGTTCGCTTCTTTACTGAGGATGATCGATAATGGGAAAAAGCCTGACAGAGCAATGTCGCGTGGAGGAAGAAGGCA	A/C	TACGGGTCGTCAACTTCTTTCCCCGGAGAAGAAACAATGACGATATATAGGAAATAAGCAATGACTAACTCTATGCAAGCAGTCGCGATAAGATAGAGGA
Ca_LG_5:40995270	AATAGTTCGCTTCTTTACTGAGGATGATCGATAATGGGAAAAAGCCTGACAGAGCAATGTCGCGTGGAGGAAGAAGGCATACGGGTCGTCAACTTCTTTC	C/T	CCCGGAGAAGAAACAATGACGATATATAGGAAATAAGCAATGACTAACTCTATGCAAGCAGTCGCGATAAGATAGAGGATGCAAACATTATCCATAAATA
Ca_LG_5:40995273	AGTTCGCTTCTTTACTGAGGATGATCGATAATGGGAAAAAGCCTGACAGAGCAATGTCGCGTGGAGGAAGAAGGCATACGGGTCGTCAACTTCTTTCCCC	C/T	GGAGAAGAAACAATGACGATATATAGGAAATAAGCAATGACTAACTCTATGCAAGCAGTCGCGATAAGATAGAGGATGCAAACATTATCCATAAATAATT
Ca_LG_5:40995292	GATGATCGATAATGGGAAAAAGCCTGACAGAGCAATGTCGCGTGGAGGAAGAAGGCATACGGGTCGTCAACTTCTTTCCCCGGAGAAGAAACAATGACGA	A/G	TATATAGGAAATAAGCAATGACTAACTCTATGCAAGCAGTCGCGATAAGATAGAGGATGCAAACATTATCCATAAATAATTGGGCGTAAAGCGTTTGTAG
Ca_LG_5:40995296	ATCGATAATGGGAAAAAGCCTGACAGAGCAATGTCGCGTGGAGGAAGAAGGCATACGGGTCGTCAACTTCTTTCCCCGGAGAAGAAACAATGACGATATA	A/C	TAGGAAATAAGCAATGACTAACTCTATGCAAGCAGTCGCGATAAGATAGAGGATGCAAACATTATCCATAAATAATTGGGCGTAAAGCGTTTGTAGGTTG
Ca_LG_5:40995298	CGATAATGGGAAAAAGCCTGACAGAGCAATGTCGCGTGGAGGAAGAAGGCATACGGGTCGTCAACTTCTTTCCCCGGAGAAGAAACAATGACGATATATA	A/G	GGAAATAAGCAATGACTAACTCTATGCAAGCAGTCGCGATAAGATAGAGGATGCAAACATTATCCATAAATAATTGGGCGTAAAGCGTTTGTAGGTTGTC
Ca_LG_5:40995301	TAATGGGAAAAAGCCTGACAGAGCAATGTCGCGTGGAGGAAGAAGGCATACGGGTCGTCAACTTCTTTCCCCGGAGAAGAAACAATGACGATATATAGGA	A/G	AATAAGCAATGACTAACTCTATGCAAGCAGTCGCGATAAGATAGAGGATGCAAACATTATCCATAAATAATTGGGCGTAAAGCGTTTGTAGGTTGTCTTT

**Positions**	**-100 bp flanking sequences**	**SNPs within *kabuli* cultivar**	**+100 bp flanking sequences**
Ca_LG_5:41083113	ATGACACTAGGATGACAATGGGCTTCCATCAAGTGGAATGGTTTACTAATGCTGCTGAACAACTACTGATCTTGAATGAGTTGGTTGTTGCACTAGCATG	G/T	CACACTGTTGCCTTGGCTTCCCATGGCTCAAATCTTAAAGGCACACTCGGTTTATAGGGCAGCAAATTTGATTGTACAGACTTTGATCACTTGGGATGTT
Ca_LG_5:41083122	GGATGACAATGGGCTTCCATCAAGTGGAATGGTTTACTAATGCTGCTGAACAACTACTGATCTTGAATGAGTTGGTTGTTGCACTAGCATGCACACTGTT	T/A	GCCTTGGCTTCCCATGGCTCAAATCTTAAAGGCACACTCGGTTTATAGGGCAGCAAATTTGATTGTACAGACTTTGATCACTTGGGATGTTGAAGTAGAT
Ca_LG_5:41896370	GCTCACTAGTAAAACATGATATTTTTTTAATGAAAATAAAGGAAGAACCGTATAGATTCCATACCAACCACACTGATCCGTGGATAAATAAGGAATACAT	T/G	AAACAAAAACAGCAATTGGACCGGAGAATGCAATTGCATTATAAGAGCGCAATTGAACATAGAGCAAGTTCAAATTCAGTAACATAAAATCTATTAATCA
Ca_LG_5:43910046	GTTTTCTGATCGTAAGTTCTCATTCTCTTGGGTGACTTTTTCTAACTTTGCTTGCAGCTCTTTTTTCTCCTCTTCAGATTTTAGCAATGATGCTTTGAGT	T/C	TCTTCTATCTCTTCATTTGCAACAAGAATTGGTTCAGACATTTCCTGATCATTTGAGGGGGTACTGTGGAAAGGCAACTTAATTTCTTGGACTCTATTTT
Ca_LG_5:43910103	CTCTTTTTTCTCCTCTTCAGATTTTAGCAATGATGCTTTGAGTTCTTCTATCTCTTCATTTGCAACAAGAATTGGTTCAGACATTTCCTGATCATTTGAG	G/T	GGGGTACTGTGGAAAGGCAACTTAATTTCTTGGACTCTATTTTTTACCCATTGTTGATAGGGTTCTTTTGTGCTGCAACTTCTGCATCCAAGTGCTTTTC
Ca_LG_5:43910106	TTTTTTCTCCTCTTCAGATTTTAGCAATGATGCTTTGAGTTCTTCTATCTCTTCATTTGCAACAAGAATTGGTTCAGACATTTCCTGATCATTTGAGGGG	G/T	GTACTGTGGAAAGGCAACTTAATTTCTTGGACTCTATTTTTTACCCATTGTTGATAGGGTTCTTTTGTGCTGCAACTTCTGCATCCAAGTGCTTTTCCTT
Ca_LG_5:43910123	ATTTTAGCAATGATGCTTTGAGTTCTTCTATCTCTTCATTTGCAACAAGAATTGGTTCAGACATTTCCTGATCATTTGAGGGGGTACTGTGGAAAGGCAA	A/G	CTTAATTTCTTGGACTCTATTTTTTACCCATTGTTGATAGGGTTCTTTTGTGCTGCAACTTCTGCATCCAAGTGCTTTTCCTTTTCTGATGATCTTCTCC
Ca_LG_5:44874011	GGCGAATGGATGGGTAATACAAGAACCTACCCTTGGGAGAGGGACAACAGTTGGAAACGGTTGCTAATACCCATAGGCTGAGGAGCAAAAGGAGGAACCT	T/C	GCCCAAGGAGGGTTTGCGTCTGATTTGCTAGTTGGTGAGGTAATAGCTTACCAAGGCGATGGTCAATAGCTGGTCCGAGAGGATGATCAGCCACACCGGG
Ca_LG_5:44874037	CTACCCTTGGGAGAGGGACAACAGTTGGAAACGGTTGCTAATACCCATAGGCTGAGGAGCAAAAGGAGGAACCTGCCCAAGGAGGGTTTGCGTCTGATTT	T/A	GCTAGTTGGTGAGGTAATAGCTTACCAAGGCGATGGTCAATAGCTGGTCCGAGAGGATGATCAGCCACACCGGGACTAGGACAAGGTCCAAATTCCTACG
Ca_LG_5:44874073	GCTAATACCCATAGGCTGAGGAGCAAAAGGAGGAACCTGCCCAAGGAGGGTTTGCGTCTGATTTGCTAGTTGGTGAGGTAATAGCTTACCAAGGCGATGG	G/A	TCAATAGCTGGTCCGAGAGGATGATCAGCCACACCGGGACTAGGACAAGGTCCAAATTCCTACGGGAGGAATCCAAATGATACTCTGTTTTGTTTGTTTT
Ca_LG_5:44874077	ATACCCATAGGCTGAGGAGCAAAAGGAGGAACCTGCCCAAGGAGGGTTTGCGTCTGATTTGCTAGTTGGTGAGGTAATAGCTTACCAAGGCGATGGTCAA	A/G	TAGCTGGTCCGAGAGGATGATCAGCCACACCGGGACTAGGACAAGGTCCAAATTCCTACGGGAGGAATCCAAATGATACTCTGTTTTGTTTGTTTTGATT
Ca_LG_5:44874108	CCTGCCCAAGGAGGGTTTGCGTCTGATTTGCTAGTTGGTGAGGTAATAGCTTACCAAGGCGATGGTCAATAGCTGGTCCGAGAGGATGATCAGCCACACC	C/T	GGGACTAGGACAAGGTCCAAATTCCTACGGGAGGAATCCAAATGATACTCTGTTTTGTTTGTTTTGATTGAGTATTATCTTATCATAGGTTACTTAAGTA

Positions	-100 bp flanking sequences	SNPs within *kabuli* cultivar	+100 bp flanking sequences
Ca_LG_5:44874115	AAGGAGGGTTTGCGTCTGATTTGCTAGTTGGTGAGGTAATAGCTTACCAAGGCGATGGTCAATAGCTGGTCCGAGAGGATGATCAGCCACACCGGGACTA	A/G	GGACAAGGTCCAAATTCCTACGGGAGGAATCCAAATGATACTCTGTTTTGTTTGTTTTGATTGAGTATTATCTTATCATAGGTTACTTAAGTATCTTCTG
Ca_LG_5:44874116	AGGAGGGTTTGCGTCTGATTTGCTAGTTGGTGAGGTAATAGCTTACCAAGGCGATGGTCAATAGCTGGTCCGAGAGGATGATCAGCCACACCGGGACTAG	G/A	GACAAGGTCCAAATTCCTACGGGAGGAATCCAAATGATACTCTGTTTTGTTTGTTTTGATTGAGTATTATCTTATCATAGGTTACTTAAGTATCTTCTGC
Ca_LG_5:44874124	TTGCGTCTGATTTGCTAGTTGGTGAGGTAATAGCTTACCAAGGCGATGGTCAATAGCTGGTCCGAGAGGATGATCAGCCACACCGGGACTAGGACAAGGT	T/C	CCAAATTCCTACGGGAGGAATCCAAATGATACTCTGTTTTGTTTGTTTTGATTGAGTATTATCTTATCATAGGTTACTTAAGTATCTTCTGCCTGTTGGA
Ca_LG_5:44874128	GTCTGATTTGCTAGTTGGTGAGGTAATAGCTTACCAAGGCGATGGTCAATAGCTGGTCCGAGAGGATGATCAGCCACACCGGGACTAGGACAAGGTCCAA	A/G	ATTCCTACGGGAGGAATCCAAATGATACTCTGTTTTGTTTGTTTTGATTGAGTATTATCTTATCATAGGTTACTTAAGTATCTTCTGCCTGTTGGAGACT
Ca_LG_5:44874130	CTGATTTGCTAGTTGGTGAGGTAATAGCTTACCAAGGCGATGGTCAATAGCTGGTCCGAGAGGATGATCAGCCACACCGGGACTAGGACAAGGTCCAAAT	T/C	TCCTACGGGAGGAATCCAAATGATACTCTGTTTTGTTTGTTTTGATTGAGTATTATCTTATCATAGGTTACTTAAGTATCTTCTGCCTGTTGGAGACTAA
Ca_LG_5:45108199	CACAGCAGAGAGTGAGGCAAAGAGAAAAGTGAGAGAGGAAGAGCAAAACCTATTTTCGCATAAAACAGAGCAGCTGCACTAGATTCACGATTTCTCCTTC	C/T	GTTCACCGTCGGATCGGACCGAAATTTAAACAACAGGTTCGTGACTCATGGTACTTCAATCTGACCGGTTGGATTGTCGATCAGAGGTATGAGGTGGGAG
Ca_LG_5:45109641	AAACATCAAGAGATTGTTCTACCCAAACACCGTTGACGAAAGACAAGCGCGGTCCTCCACCAGGTGAGAGTATAAGGAGTTAAGATTAGCGGTGGATTTT	T/G	GATCGGAGGAAGGAGAGAAGCTGTTGTTGTGTGGGACCCTCAGAACCAGCGGTGATGATGCTTAGCACAGTTTGGAGCGACAATGGCGAAAACACAATAT
Ca_LG_5:45109645	ATCAAGAGATTGTTCTACCCAAACACCGTTGACGAAAGACAAGCGCGGTCCTCCACCAGGTGAGAGTATAAGGAGTTAAGATTAGCGGTGGATTTTGATC	C/T	GGAGGAAGGAGAGAAGCTGTTGTTGTGTGGGACCCTCAGAACCAGCGGTGATGATGCTTAGCACAGTTTGGAGCGACAATGGCGAAAACACAATATTCTG
Ca_LG_5:45109665	AAACACCGTTGACGAAAGACAAGCGCGGTCCTCCACCAGGTGAGAGTATAAGGAGTTAAGATTAGCGGTGGATTTTGATCGGAGGAAGGAGAGAAGCTGT	T/A	TGTTGTGTGGGACCCTCAGAACCAGCGGTGATGATGCTTAGCACAGTTTGGAGCGACAATGGCGAAAACACAATATTCTGTCTTTGAAATTTTTCTTTGG
Ca_LG_5:45346468	GACTGAAGCAGCTAATTTGAGGGAAAGATTTGTGCTGGGTACAAGTGTTAGCCGTAGGGTTGCTGCTGCGGCCGCATCTGCTGTAATTAAATGCTTTTTT	T/C	CGCATGCCGTTGTTTAAGTATTGTCTAGTATGAAATTAAAGGACATTTAACCAAACATAATGAAGATTTACTTGTTGATATGCAGGCGGAGGCCGCGGCT
Ca_LG_5:45346480	TAATTTGAGGGAAAGATTTGTGCTGGGTACAAGTGTTAGCCGTAGGGTTGCTGCTGCGGCCGCATCTGCTGTAATTAAATGCTTTTTTCGCATGCCGTTG	G/A	TTTAAGTATTGTCTAGTATGAAATTAAAGGACATTTAACCAAACATAATGAAGATTTACTTGTTGATATGCAGGCGGAGGCCGCGGCTGCTGCTGGTGAA
Ca_LG_5:45389039	CGAGGAGAGATTGTTACACTTGCCCAGGTACCGTGGTTTTATTGTATATGTTTGGATCATGTCATTCATTGAAAAGGTTTTCCTGTCTTTGCAAATTACA	A/C	AATAGAAACATTTTATATCTTACACTTAAAAAATAACAAAAACACATGCCTTATTGCCCTTCTAACATTATAGTGCTGCTTTGACTAAAATATATAGCT

Positions	-100 bp flanking sequences	SNPs within *kabuli* cultivar	+100 bp flanking sequences
Ca_LG_5:45389093	GATCATGTCATTCATTGAAAAGGTTTTCCTGTCTTTGCAAATTACAAATAGAAACATTTTATATCTTACACTTAAAAAATAACAAAAAACACATGCCTTA	A/T	TTGCCCTTCTAACATTATAGTGCTGCTTTGACTAAAATATATAGCTTGAAAGTTGAATTTGTCTTTGCTGTATAAAGTTTGAAATGGACTTTTTTCAGTA
Ca_LG_5:45389096	CATGTCATTCATTGAAAAGGTTTTCCTGTCTTTGCAAATTACAAATAGAAACATTTTATATCTTACACTTAAAAAATAACAAAAAACACATGCCTTATTG	G/T	CCCTTCTAACATTATAGTGCTGCTTTGACTAAAATATATAGCTTGAAAGTTGAATTTGTCTTTGCTGTATAAAGTTTGAAATGGACTTTTTTCAGTATAT
Ca_LG_5:45569112	TAAATCACATTCACATAACTCTAACAACCAAATTTCATGACTCTTTTTTTGAAAAATTGAAGCAACAAAAGTCCAACGGCCAACCCAGCAACAATAACAA	A/G	AAGCAACATAAATGATTGGATTCATGATACATTTCTCTTATTCCAAATAAAATAAAGAAAATAAATAGTTAATAATATAATCAACCAAGAAATTATGACT
Ca_LG_5:45569142	AATTTCATGACTCTTTTTTTGAAAAATTGAAGCAACAAAAGTCCAACGGCCAACCCAGCAACAATAACAAAAGCAACATAAATGATTGGATTCATGATAC	C/A	ATTTCTCTTATTCCAAATAAAATAAAGAAAATAAATAGTTAATAATATAATCAACCAAGAAATTATGACTTAAATTATTTCATTCTTCATATTTATCCGT
Ca_LG_5:45569143	ATTTCATGACTCTTTTTTTGAAAAATTGAAGCAACAAAAGTCCAACGGCCAACCCAGCAACAATAACAAAAGCAACATAAATGATTGGATTCATGATACA	A/G	TTTCTCTTATTCCAAATAAAATAAAGAAAATAAATAGTTAATAATATAATCAACCAAGAAATTATGACTTAAATTATTTCATTCTTCATATTTATCCGTT
Ca_LG_5:45569150	GACTCTTTTTTTGAAAAATTGAAGCAACAAAAGTCCAACGGCCAACCCAGCAACAATAACAAAAGCAACATAAATGATTGGATTCATGATACATTTCTCT	T/C	TATTCCAAATAAAATAAAGAAAATAAATAGTTAATAATATAATCAACCAAGAAATTATGACTTAAATTATTTCATTCTTCATATTTATCCGTTTTTTTTT
Ca_LG_5:45569174	CAACAAAAGTCCAACGGCCAACCCAGCAACAATAACAAAAGCAACATAAATGATTGGATTCATGATACATTTCTCTTATTCCAAATAAAATAAAGAAAAT	T/G	AAATAGTTAATAATATAATCAACCAAGAAATTATGACTTAAATTATTTCATTCTTCATATTTATCCGTTTTTTTTTCCAGACATCACATGACACAAGAAA
Ca_LG_6:2681518	TTGATGAAGTTAAAACATGATCTGACCCCATCTGCACCAACTACAGCAGCATGGATTCTAGCTGTTGGATCCTCTAGGGTCAGTTTCATCCTATACACCC	C/A	CCCCAGCAGGAAAGCACAGCTTTTCAGCTTGGTATGGCATTGCTGCTACCACCCGAACGACACAGTTGAATCTGGCTGTCACCTTCAAAACACAGTATAT
Ca_LG_6:3060633	GGTGATATTTGATATATTTTGTTTGGATGTTTCGCATTTGCTGCTATACTATATCGTGAAAGTACATAACTCAATTTATGATATGTTTTGTATTTGTAGA	A/G	AGTTTGTGCGATGTGTGGCAAGCAAATTCTTGACACCAAGTTATATAAACAACGCATTGTGTAGTGCATTTGTCCTGCATTGTGGTTTTTCAGGGACCTC
Ca_LG_6:3060659	ATGTTTCGCATTTGCTGCTATACTATATCGTGAAAGTACATAACTCAATTTATGATATGTTTTGTATTTGTAGAAGTTTGTGCGATGTGTGGCAAGCAAA	A/G	TTCTTGACACCAAGTTATATAAACAACGCATTGTGTAGTGCATTTGTCCTGCATTGTGGTTTTTCAGGGACCTCTGAAAACAAAGGAGGTGGGATGGGAA
Ca_LG_6:3060667	CATTTGCTGCTATACTATATCGTGAAAGTACATAACTCAATTTATGATATGTTTTGTATTTGTAGAAGTTTGTGCGATGTGTGGCAAGCAAATTCTTGAC	C/T	ACCAAGTTATATAAACAACGCATTGTGTAGTGCATTTGTCCTGCATTGTGGTTTTTCAGGGACCTCTGAAAACAAAGGAGGTGGGATGGGAAATTTCCAA
Ca_LG_6:3060676	CTATACTATATCGTGAAAGTACATAACTCAATTTATGATATGTTTTGTATTTGTAGAAGTTTGTGCGATGTGTGGCAAGCAAATTCTTGACACCAAGTTA	A/T	TATAAACAACGCATTGTGTAGTGCATTTGTCCTGCATTGTGGTTTTTCAGGGACCTCTGAAAACAAAGGAGGTGGGATGGGAAATTTCCAACCATTTTTT

**Positions**	**-100 bp flanking sequences**	**SNPs within *kabuli* cultivar**	**+100 bp flanking sequences**
Ca_LG_6:3060686	TCGTGAAAGTACATAACTCAATTTATGATATGTTTTGTATTTGTAGAAGTTTGTGCGATGTGTGGCAAGCAAATTCTTGACACCAAGTTATATAAACAAC	C/A	GCATTGTGTAGTGCATTTGTCCTGCATTGTGGTTTTTCAGGGACCTCTGAAAACAAAGGAGGTGGGATGGGAAATTTCCAACCATTTTTTGTGCTCAACC
Ca_LG_6:3060690	GAAAGTACATAACTCAATTTATGATATGTTTTGTATTTGTAGAAGTTTGTGCGATGTGTGGCAAGCAAATTCTTGACACCAAGTTATATAAACAACGCAT	T/A	TGTGTAGTGCATTTGTCCTGCATTGTGGTTTTTCAGGGACCTCTGAAAACAAAGGAGGTGGGATGGGAAATTTCCAACCATTTTTTGTGCTCAACCTAGA
Ca_LG_6:3491941	CAGAGAAGGAACTTACTAGTCCACCTTTGGATATGTTCCCGCCAAATAAGAGCAGCATGGAATCAGATATGGCCGTTGAATCCCTGAGGAATACTGTATT	T/G	CACTTTAACCTTTTCATTAAACACTAACCATGGGTATGGAATTTTGGGTACCGCAGCATTTACTGAGTTCTACACGTGCAGCACCAAAATTTTAGAACTT
Ca_LG_6:4971056	GTAATTTGGGAATATTAATTTTTTAAAACATTTGAGGGGTGTGGGTAGTAAATAAGGGGGTGTTGGTAGCAATATTGTAAGTCTTTTCTTTTCTCATCAC	C/A	GAAAAGTCTTTCTCAAAGATTCTAAGATTCTATAGACATGGATATATTATATATCAAAGTATATGATATATGAAATTGAGATAGTTACCTTTCAGAAAAT
Ca_LG_6:4971063	GGGAATATTAATTTTTTAAAACATTTGAGGGGTGTGGGTAGTAAATAAGGGGGTGTTGGTAGCAATATTGTAAGTCTTTTCTTTTCTCATCACGAAAAGT	T/C	CTTTCTCAAAGATTCTAAGATTCTATAGACATGGATATATTATATATCAAAGTATATGATATATGAAATTGAGATAGTTACCTTTCAGAAAATTCCAATA
Ca_LG_6:5380454	TTCCAGGGTTGGCTGCAACGGGCTGCTATCTGGGATTAACTACCTAGCTTGAAACCAAGTTCTCACAACAGCAACCCAATTTGCGTAATTGGAAAAACCG	G/A	ACCAATTTTTCGCAAGGAATGAGCAGAAGAGTAGTGAAACTGAAAGCGCAACGAAAAGCAGCAACAGATACAGTAACGAACATGCGATCCACGCGAAGGG
Ca_LG_6:5841978	AGTGATATTTATAGAAAAATCTTGACCTTTGTGGCGGCCAAGCAGCCACAAAATCCAACGGTAACTAAAGCATTTGTTGCTGCCTAAGCAGCCACAACGG	G/C	GCAACGTGTAATTCGGCATTTGTGGCAGCCTTTGCAGCCACAAAGTGTAACGGTCCAGTGTTTTTGTGGCAGCTTGGGCGGTCATAAATGGTTTGCCTTT
Ca_LG_6:5842033	CAACGGTAACTAAAGCATTTGTTGCTGCCTAAGCAGCCACAACGGGCAACGTGTAATTCGGCATTTGTGGCAGCCTTTGCAGCCACAAAGTGTAACGGTC	C/T	CAGTGTTTTTGTGGCAGCTTGGGCGGTCATAAATGGTTTGCCTTTGTGGCGGCCTTTACTCTTATAAATGGGTGATTTTGTGGTTGAGAAAGCTCTCACA
Ca_LG_6:5842049	ATTTGTTGCTGCCTAAGCAGCCACAACGGGCAACGTGTAATTCGGCATTTGTGGCAGCCTTTGCAGCCACAAAGTGTAACGGTCCAGTGTTTTTGTGGCA	A/G	GCTTGGGCGGTCATAAATGGTTTGCCTTTGTGGCGGCCTTTACTCTTATAAATGGGTGATTTTGTGGTTGAGAAAGCTCTCACAAAGGACTACAATTTCA
Ca_LG_6:5842063	AAGCAGCCACAACGGGCAACGTGTAATTCGGCATTTGTGGCAGCCTTTGCAGCCACAAAGTGTAACGGTCCAGTGTTTTTGTGGCAGCTTGGGCGGTCAT	T/C	AAATGGTTTGCCTTTGTGGCGGCCTTTACTCTTATAAATGGGTGATTTTGTGGTTGAGAAAGCTCTCACAAAGGACTACAATTTCAACTGGATTTTGTGG
Ca_LG_6:6389358	GATGGAGGATGTTTTGGAGGAAGAAATCAAAGTGATCAATGATACAGTGGGACCTGAGGAGCATGTTATTTGTGCCTTATCTGGTGGTGTTGATTCCACT	T/C	GTTGCTGCTACCATTGTTCATAAAGCCATTGGGAATAGGCTTCATTGTGTTTTTGTTGACAATGGATTGCTAAGGTTACTATAAGTCTGTTTTTTTCCAT
Ca_LG_6:7116485	TTCTTGGTGTGCATGAAAGATGATAGTATTGAGGGGATTTATGATACTTTGAAGGAGTGTGCTGTTATTAGTAAATCTGCAGGAGGAATTGGTGTTTCTG	G/A	TTCATAACATACGTGCACATGGTAGTTACATTCGAGGAACAAATGGGACATCTAATGGTATTGTTCCAATGCTCCGTGTGTTTAATGACACTGCCCGCTA

Positions	-100 bp flanking sequences	SNPs within *kabuli* cultivar	+100 bp flanking sequences
Ca_LG_6:7116502	AGATGATAGTATTGAGGGGATTTATGATACTTTGAAGGAGTGTGCTGTTATTAGTAAATCTGCAGGAGGAATTGGTGTTTCTGTTCATAACATACGTGCA	A/C	CATGGTAGTTACATTCGAGGAACAAATGGGACATCTAATGGTATTGTTCCAATGCTCCGTGTGTTTAATGACACTGCCCGCTATGTTGATCAGGGTGGAG
Ca_LG_6:7116520	GATTTATGATACTTTGAAGGAGTGTGCTGTTATTAGTAAATCTGCAGGAGGAATTGGTGTTTCTGTTCATAACATACGTGCACATGGTAGTTACATTCGA	A/T	GGAACAAATGGGACATCTAATGGTATTGTTCCAATGCTCCGTGTGTTTAATGACACTGCCCGCTATGTTGATCAGGGTGGAGGCAAGAGGAAAGGTATAA
Ca_LG_6:7116535	GAAGGAGTGTGCTGTTATTAGTAAATCTGCAGGAGGAATTGGTGTTTCTGTTCATAACATACGTGCACATGGTAGTTACATTCGAGGAACAAATGGGACA	A/G	TCTAATGGTATTGTTCCAATGCTCCGTGTGTTTAATGACACTGCCCGCTATGTTGATCAGGGTGGAGGCAAGAGGAAAGGTATAAAGAAACCATTTTGAG
Ca_LG_6:7118057	ACTAGGAATGTCATTTGATTCACCAGAGGTAAATATATTTTATGTGGTTGCCAAATTTCATACAGTTCAAATTTGATAGCTTACATTACATGTATATGGA	A/G	TTGCAGGCTCAACAGTTGAACAAGGACATTTTTGAGACTATATACTATCATGCTCTGAAAACTTCTTGTGAATTGGCTGCAAAGGAAGGACCCTATGAAA
Ca_LG_6:7118058	CTAGGAATGTCATTTGATTCACCAGAGGTAAATATATTTTATGTGGTTGCCAAATTTCATACAGTTCAAATTTGATAGCTTACATTACATGTATATGGAT	T/G	TGCAGGCTCAACAGTTGAACAAGGACATTTTTGAGACTATATACTATCATGCTCTGAAAACTTCTTGTGAATTGGCTGCAAAGGAAGGACCCTATGAAAC
Ca_LG_6:7854519	TACGCCTTACCATCAAAGTCTGTAATTAATCTATCCATTAAGCATCAAAAAAGGCTCTAGATGAACTAAGTTTCAATTCTTACTGTAAACAGATGAACGC	C/A	ATGTCTCAAATTATCTTTTGAAAGAAATGTACATGTAATTTTCATACACCAAGCTGCACATAATTAAGTTAACGATTCATTAGTACGCCTTACCATCAAA
Ca_LG_6:7854524	CTTACCATCAAAGTCTGTAATTAATCTATCCATTAAGCATCAAAAAAGGCTCTAGATGAACTAAGTTTCAATTCTTACTGTAAACAGATGAACGCATGTC	C/T	TCAAATTATCTTTTGAAAGAAATGTACATGTAATTTTCATACACCAAGCTGCACATAATTAAGTTAACGATTCATTAGTACGCCTTACCATCAAAGTCTG
Ca_LG_6:8076698	ATCTCAAAACTTTATCTTCATGTTCTTTAATCACGTATCCCATTGGCAGCTCAACATAGACCTCTTCTTCAAGATAGCCATTTAGAAATGTCGATTTTAC	C/T	ATCAAGTTGATAAAGTTTCCACTTATTTTGAGCTTCTATAGAGATTAGCAAATGAATAGTGTCCATGCGATCAACTGGTGCAAAAACTTCTTCATAGTCA
Ca_LG_6:8076700	CTCAAAACTTTATCTTCATGTTCTTTAATCACGTATCCCATTGGCAGCTCAACATAGACCTCTTCTTCAAGATAGCCATTTAGAAATGTCGATTTTACAT	T/G	CAAGTTGATAAAGTTTCCACTTATTTTGAGCTTCTATAGAGATTAGCAAATGAATAGTGTCCATGCGATCAACTGGTGCAAAAACTTCTTCATAGTCAAC
Ca_LG_6:8076713	CTTCATGTTCTTTAATCACGTATCCCATTGGCAGCTCAACATAGACCTCTTCTTCAAGATAGCCATTTAGAAATGTCGATTTTACATCAAGTTGATAAAG	G/T	TTTCCACTTATTTTGAGCTTCTATAGAGATTAGCAAATGAATAGTGTCCATGCGATCAACTGGTGCAAAAACTTCTTCATAGTCAACTCCATGTTTTTGT
Ca_LG_6:8086128	ACGAAGAACCCTAACCCAAAAAATTCCTAAATCAAATCTTTCTTTTAGCAAATTAAAAACCCTAAACCGCTGCTTCTTCTGCTGAAATCGTGATTCTCGC	C/T	TTCATTCGGCTGCGATTCTCCTTACAGGTTAGAATTGGAAAAGTTTCTGATTGTTGATGATGCTACTTTGTTTTCACTGAGCTACAAATTTTTTATTGCT
Ca_LG_6:8147968	TATCTCAACCTCAATATTTTGGTGCTTTAGGAAGTTTTAGAGATAGCCAGCGAGGTGAAATAGGATGCAGTTACACGGATAATATTTTTTAGCACCCTCA	A/T	TCTGGAGCCATACTCATCTACTTGTCTTGAATTTGAATTGCTGCTTTTAGCCGTTTACCGTCTCTTGCTACCAAAACCATCTCCATTCCAAGATTTTTAT

**Positions**	**-100 bp flanking sequences**	**SNPs within *kabuli* cultivar**	**+100 bp flanking sequences**
Ca_LG_6:8147975	ACCTCAATATTTTGGTGCTTTAGGAAGTTTTAGAGATAGCCAGCGAGGTGAAATAGGATGCAGTTACACGGATAATATTTTTTAGCACCCTCATCTGGAG	G/C	CCATACTCATCTACTTGTCTTGAATTTGAATTGCTGCTTTTAGCCGTTTACCGTCTCTTGCTACCAAAACCATCTCCATTCCAAGATTTTTATTATTATA
Ca_LG_6:8153021	AGTCACTAAACTAAATTGCATGAGAAACACCAAAAAGGAAGCAGCGCGACTCTAATGAAACGAAACAGGAGAGACAATGGTTGTTTCGGAAAACTTGCTG	G/A	GAAAACTTTTCCAGCAGCGACAACAGATGGAAGGCAGCAGTAGACGGCGGATCTTGGAGGCATTAGAGGCACATGAGCGCGTGCGGTGCAACTTTTAACC
Ca_LG_6:8153022	GTCACTAAACTAAATTGCATGAGAAACACCAAAAAGGAAGCAGCGCGACTCTAATGAAACGAAACAGGAGAGACAATGGTTGTTTCGGAAAACTTGCTGG	G/A	AAAACTTTTCCAGCAGCGACAACAGATGGAAGGCAGCAGTAGACGGCGGATCTTGGAGGCATTAGAGGCACATGAGCGCGTGCGGTGCAACTTTTAACCT
Ca_LG_6:8153028	AAACTAAATTGCATGAGAAACACCAAAAAGGAAGCAGCGCGACTCTAATGAAACGAAACAGGAGAGACAATGGTTGTTTCGGAAAACTTGCTGGAAAACT	T/G	TTTCCAGCAGCGACAACAGATGGAAGGCAGCAGTAGACGGCGGATCTTGGAGGCATTAGAGGCACATGAGCGCGTGCGGTGCAACTTTTAACCTAAAAAA
Ca_LG_6:8153035	ATTGCATGAGAAACACCAAAAAGGAAGCAGCGCGACTCTAATGAAACGAAACAGGAGAGACAATGGTTGTTTCGGAAAACTTGCTGGAAAACTTTTCCAG	G/A	CAGCGACAACAGATGGAAGGCAGCAGTAGACGGCGGATCTTGGAGGCATTAGAGGCACATGAGCGCGTGCGGTGCAACTTTTAACCTAAAAAAATATGGG
Ca_LG_6:8274170	AGTATCCGTACCTGGTTTGAAAAAGAGGGGGAAATTAACCAAAATATAAGTAAAGATATCATACACAAGTTCTCTGCTAGAATTGTCAAATAGCAGCAAC	C/T	ACAAAGCTATTGCATAGCGGAATTTTACTAAAATGTTATTATATAGAGCTATAGCATAGCCAAATTTTAATAAAATTTTAGTAGAAAGTGTCATCAAATA
Ca_LG_6:8274198	GGGAAATTAACCAAAATATAAGTAAAGATATCATACACAAGTTCTCTGCTAGAATTGTCAAATAGCAGCAACACAAAGCTATTGCATAGCGGAATTTTAC	C/A	TAAAATGTTATTATATAGAGCTATAGCATAGCCAAATTTTAATAAAATTTTAGTAGAAAGTGTCATCAAATAGTGGTTATAGNNNNNNNNNNNNNNNNNN
Ca_LG_6:8503370	AGGGTATAAAGAGATTTCAAAATGCATTGATCCAACTCCCAATTCTCCGTACAAAGCTAGAAACATGTGGAAAAATGCATTGATCAAACACCCAAATCAA	A/C	AAAGTGCTTTTCCATAATAGATTCCACGAATGGAACATGGAATAGTAAAACTACTAGGGTCTTGCATTTTTGTAAGGAGCTTTTGGTGTAACATTATATG
Ca_LG_6:8503381	AGATTTCAAAATGCATTGATCCAACTCCCAATTCTCCGTACAAAGCTAGAAACATGTGGAAAAATGCATTGATCAAACACCCAAATCAAAAAGTGCTTTT	T/C	CCATAATAGATTCCACGAATGGAACATGGAATAGTAAAACTACTAGGGTCTTGCATTTTTGTAAGGAGCTTTTGGTGTAACATTATATGCATTCCTTAGG
Ca_LG_6:8503385	TTCAAAATGCATTGATCCAACTCCCAATTCTCCGTACAAAGCTAGAAACATGTGGAAAAATGCATTGATCAAACACCCAAATCAAAAAGTGCTTTTCCAT	T/C	AATAGATTCCACGAATGGAACATGGAATAGTAAAACTACTAGGGTCTTGCATTTTTGTAAGGAGCTTTTGGTGTAACATTATATGCATTCCTTAGGGTTA
Ca_LG_6:8503393	GCATTGATCCAACTCCCAATTCTCCGTACAAAGCTAGAAACATGTGGAAAAATGCATTGATCAAACACCCAAATCAAAAAGTGCTTTTCCATAATAGATT	T/A	CCACGAATGGAACATGGAATAGTAAAACTACTAGGGTCTTGCATTTTTGTAAGGAGCTTTTGGTGTAACATTATATGCATTCCTTAGGGTTACAATTTCA
Ca_LG_6:8503398	GATCCAACTCCCAATTCTCCGTACAAAGCTAGAAACATGTGGAAAAATGCATTGATCAAACACCCAAATCAAAAAGTGCTTTTCCATAATAGATTCCACG	G/C	AATGGAACATGGAATAGTAAAACTACTAGGGTCTTGCATTTTTGTAAGGAGCTTTTGGTGTAACATTATATGCATTCCTTAGGGTTACAATTTCAAACTC

Positions	-100 bp flanking sequences	SNPs within *kabuli* cultivar	+100 bp flanking sequences
Ca_LG_6:8503410	AATTCTCCGTACAAAGCTAGAAACATGTGGAAAAATGCATTGATCAAACACCCAAATCAAAAAGTGCTTTTCCATAATAGATTCCACGAATGGAACATGG	G/A	AATAGTAAAACTACTAGGGTCTTGCATTTTTGTAAGGAGCTTTTGGTGTAACATTATATGCATTCCTTAGGGTTACAATTTCAAACTCCCGAATTCTTAT
Ca_LG_6:8503417	CGTACAAAGCTAGAAACATGTGGAAAAATGCATTGATCAAACACCCAAATCAAAAAGTGCTTTTCCATAATAGATTCCACGAATGGAACATGGAATAGTA	A/G	AAACTACTAGGGTCTTGCATTTTTGTAAGGAGCTTTTGGTGTAACATTATATGCATTCCTTAGGGTTACAATTTCAAACTCCCGAATTCTTATTCTTTTG
Ca_LG_6:8503425	GCTAGAAACATGTGGAAAAATGCATTGATCAAACACCCAAATCAAAAAGTGCTTTTCCATAATAGATTCCACGAATGGAACATGGAATAGTAAAACTACT	T/C	AGGGTCTTGCATTTTTGTAAGGAGCTTTTGGTGTAACATTATATGCATTCCTTAGGGTTACAATTTCAAACTCCCGAATTCTTATTCTTTTGGTGATTAT
Ca_LG_6:8503445	TGCATTGATCAAACACCCAAATCAAAAAGTGCTTTTCCATAATAGATTCCACGAATGGAACATGGAATAGTAAAACTACTAGGGTCTTGCATTTTTGTAA	A/G	GGAGCTTTTGGTGTAACATTATATGCATTCCTTAGGGTTACAATTTCAAACTCCCGAATTCTTATTCTTTTGGTGATTATATCCTTTAAAAACTTTGCAT
Ca_LG_6:9102986	TCATTTTTTCCACACTAGAAGAAATCTATCTCTAAAATAAGATAAGTGTCATCAAAGTATTTGCCACCCCAACCTGCACTCCCACTAAGCAATTTTTTGC	C/A	TTGGCAACAATGTTTGTAGCTCAAAGAGATGACAAAAAATAGCTGTTTCAGATGCATCATTGTCAAGGCAGAAGAATTGGTGGTGCCACTAGCTGACGCA
Ca_LG_6:9689078	GCTAATATTCAAAATGGTTAATGACTTTTGGCACTATCTACACTCCCTGATGTGATCTCTGTAGATGTAGCTTAAGGGAATTGCTCCTCAATCCAGCATT	T/C	CTTGCCACATATTGGGCAAACTTTCATCGTTGGTTTCCGCCACCGCCGCCACTCCATTGAAACCTTTCCATACAGTTTTTGATCAAGTTCCTGTAAGATC
Ca_LG_6:9689117	ACACTCCCTGATGTGATCTCTGTAGATGTAGCTTAAGGGAATTGCTCCTCAATCCAGCATTCTTGCCACATATTGGGCAAACTTTCATCGTTGGTTTCCG	G/A	CCACCGCCGCCACTCCATTGAAACCTTTCCATACAGTTTTTGATCAAGTTCCTGTAAGATCTTAGCAAAGCCATCATTCGGCTGCGCTCGATGGTGAACT
Ca_LG_6:9826466	CACTCGCCAAGGCACTTTGTACAGCTTTTGTAATTCCTATGACACTCTGTGTTATCATCTACTCATTACTTTACTGCACGTACCCGAGAGACAGAGAGCG	G/A	AGCGAGAATGGTTGCATTAGAGGAATCAGAAATGCAGCAACTAGATGTGGAAGATTGCACAAGAGAAGAATATTGTGAAATTCATGTTTTAGAATCAAAT
Ca_LG_6:10242193	AAACACCTAAAATCATGAACCCTAAACAAATCAAAATCAAAAACATTCATTAACTCATCGTGTCCCAGATCTGATGTGTAATGAAACATCCATTGTGACG	G/A	ACTCGACTCCATTTTATCTTGCTCATTGAATGTTTACGCACCCCATATTACCAACGAGGTCTGGTGGTTCTGACGGTGGACGTTTAGGCAACATGTATGT
Ca_LG_6:10242230	CAAAAACATTCATTAACTCATCGTGTCCCAGATCTGATGTGTAATGAAACATCCATTGTGACGACTCGACTCCATTTTATCTTGCTCATTGAATGTTTAC	C/T	GCACCCCATATTACCAACGAGGTCTGGTGGTTCTGACGGTGGACGTTTAGGCAACATGTATGTTGGCCAATGCTCAATTAATAACCCATCTTTTGCAGCT
Ca_LG_6:10242248	CATCGTGTCCCAGATCTGATGTGTAATGAAACATCCATTGTGACGACTCGACTCCATTTTATCTTGCTCATTGAATGTTTACGCACCCCATATTACCAAC	C/T	GAGGTCTGGTGGTTCTGACGGTGGACGTTTAGGCAACATGTATGTTGGCCAATGCTCAATTAATAACCCATCTTTTGCAGCTACCCTACTTGATAATGGC
Ca_LG_6:10242249	ATCGTGTCCCAGATCTGATGTGTAATGAAACATCCATTGTGACGACTCGACTCCATTTTATCTTGCTCATTGAATGTTTACGCACCCCATATTACCAACG	G/C	AGGTCTGGTGGTTCTGACGGTGGACGTTTAGGCAACATGTATGTTGGCCAATGCTCAATTAATAACCCATCTTTTGCAGCTACCCTACTTGATAATGGCT

Positions	-100 bp flanking sequences	SNPs within *kabuli* cultivar	+100 bp flanking sequences
Ca_LG_6:10242531	TCGGGTTACTGCAATGGTGGTGGCGGAATCAGATTTCTGAGA GTACCACAAAGGAGTTGCAATTCTGCCCCTTCCGATTCTGAAA ATAACCCATTATCAT	T/G	AAACCTTCTCGTCATTCTTCCTCTTATGAGCGTCATTCTTCCT CTTATCGCTCATAATGTGATTGTTGCCCATTCCTTGTTTGTTC TCCACAAGGACCTT
Ca_LG_6:10719698	TTGCAATTCTGGTATCTTCTTCTCCTTCTATATGGATTTTTATA TCTTCATTGTTCTCAAGAAGATCCATTCTGGATAGTGAACATG GCTGCTATTTTCC	C/G	GACGTGACATCGGTGATTTTCCTTCCTCCAACCATTTCAAGC AGCAGCATCCCATAACTATAAACATCAGACTTATATGATACA TTGCCGAAGTTCCTAG
Ca_LG_6:10719700	GCAATTCTGGTATCTTCTTCTCCTTCTATATGGATTTTTATATC TTCATTGTTCTCAAGAAGATCCATTCTGGATAGTGAACATGGC TGCTATTTTCCGA	A/C	CGTGACATCGGTGATTTTCCTTCCTCCAACCATTTCAAGCAG CAGCATCCCATAACTATAAACATCAGACTTATATGATACATTG CCGAAGTTCCTAGAA
Ca_LG_6:10770645	TCCCCGTATCCACTTCATGCTTTCGTCCTACGCACCTGTGATC TCTGCTGCCAAGGCATACCATGAGCAGCTCTCTGTGCCTGAA ATCACCAATGCTGTA	A/G	TTTGAGCCTGCTAGCATGATGGCCAAGTGTGATCCAAGGCA CGGGAAATACATGGCTTGCTGCTTGATGTATCGTGGAGATG TTGTACCCAAGGATGTAA
Ca_LG_6:10782487	AACTTCAAAAATAAAATAAAAAACTAGTTAAACAGAAGCACAT CTAACCTGTACACGAGGATTCTGAAAATCATCCATCGCAGCA GCTAGTGCTGGCAAC	C/G	ACCCCTTGATGGTATTGAACTTGCAAATCTGGACCCAAATCA GTGGACAGTTGCCCGATTGCATTAATAGCTGCCCACCTTACA CGAGGATGTTGATCAG
Ca_LG_6:11104497	CTTCCTAGAAGATTGGCTGCTATATTAATCCATCTGCTGGTAA TGCTTCTATAGTGTTGCTATAGCAAGTAGGATCTATGTATAAT ATCTACAGGATCAT	T/G	AGAAAGCAAAAAATTAAGTCACATAATGACACTCTGAAGACT TCAGCAGCATAAGACGGCGTAGCAAACAATCAAATGAATAT GCTAAGGAAATGAGAAA
Ca_LG_6:11865385	TAAATGCATGAATGTGTTTCAGAAAATGGCTTCAGGCGGAGC AGCTCCCCAAAGAGGAAGTGCAGCAGCTACTGCAAGCATGA GGAGAAGGAAGACAGCT	T/C	GGTGGCGGGGCCTCTGGAGGAGCTGCAGGGACTATGCTTC AGTTTTACACTGATGATGCCCCTGGACTCAAGATCTCCCCAA ATGTGGTTCTTGTAATGA
Ca_LG_6:12035671	ACAAAATCCAGCTATGGGGGGAAATGGAACACTGATCTCTCA AACAAACGACACAACAGCTTCTGCAGCAGGAGCTAATGATTC TATGCAAAACCTGAAT	T/C	CAGGTCAGCAACTCCATTCAGAAAACCTTGGGCCTTATCCAC CAGCTTTACCTTACAGTGTCTACCTTCAACGCTGCCTTTCAA ATGCCACTCCTCCAAC
Ca_LG_6:12035743	AGCTAATGATTCTATGCAAAACCTGAATCAGGTCAGCAACTCC ATTCAGAAAACCTTGGGCCTTATCCACCAGCTTTACCTTACAG TGTCTACCTTCAAC	C/T	GCTGCCTTTCAAATGCCACTCCTCCAACGCATGTATTTCTAC ACAATCCCTTATAATGCATTTTTTCTGTTTTACTTTTTGTCTAT GTCCCAAATTGATA
Ca_LG_6:12036343	AACTTAACATTTCTGTTTGGGGCAGAATTTTCGCAAGCATCTT TTGGAGGAATTGGAGCAGAACTTCCCTGATGAGGTTGAAAGT TTTAGAGAGAGTCGC	C/T	GCTGCTTCCGCTGCTGTAAGTGTTACTTATTTGGTCTTTTATT TCAATGTATCCATAACTATTGAATAACCTTCCTACATCATTGG CTGTTCCCAATTTT
Ca_LG_6:12253096	TAAAATACTACAGTGTCAGATCAGGAAGCAGCTACTACGGCA AAGAAAGCATAGTCAAAGTTCAATTCCGTAAAGATCCAATAGG CATGGTTAAAGCTAG	G/T	GGTTTCAAGAAAGAGATAATAGTGACTATCGCTGGCCAGTAA ACTAGTTCAGTCTGCAACAGCAGCCACTACAGCCATGACAA CCGTCCCTCACATTAAA
Ca_LG_6:12277946	AACTGCAGCTAAATGTTTTGGAAATGAACTTTGTTCTATATATT ATTTAATTTAATATTCATATACATACAAAATTCAGAATTAGCAG TCATCCCGCAAC	C/T	GCTCCATCTTGCTTTAGTGTTTTGAGGTGGCTGCTACACACT GCTATCCAGGATCAATAACTGCCAATTATACTTGGTTTCTGT TTTATATAGTTGGGTT

Positions	-100 bp flanking sequences	SNPs within *kabuli* cultivar	+100 bp flanking sequences
Ca_LG_6:12279324	AGCTATGAATTCTTCACTTGCTTCATCCTCCCTCCCTTTGATGATGCAGCAAGCTGTTGCCATGCAACAAATGCAATTCCAGCAAGCGCTGCTTATGCAG	G/A	CAAACTATGACTGCACAGCAGGCTGCTAACCGAGCTGCAACCATGAAATCTGCCACAGATTTAGCAGCAGCACGAGCTGCAGAAATTAGTAAGAAATTGA
Ca_LG_6:12315924	ACTAGTATATTTATAGTTGAAGAGAGACACAGTGAAAAGCAAAAAATATGATGATAAAAGTATAATATTTTACGGTGGTACTTAACGAAAAAAAGATTCA	A/G	TTAAAGCAGGTTTTGAAAAGCTCACTAACCAAACAGTACCCACACTTCATTTCTCTCTTTCTCTCTCTCTCTCTGCGCACACACCCGCAGCCATGGCGTC
Ca_LG_6:12318605	TTGCAATAGCGGTCATCCCGGAATCCGCTATCCCATTTTTAGGGTTGGGCGTTCCGCGCTGCTATTTGAGATCGGCAACATAGGTAGTATGAGAGGGTAG	G/A	AAGATGACACAAACATTATTGGTTTGCTTAATCATTCCTGGGGAGTTTTTAAGCAGTCTTGCTGCATGTGGTGCTCCAGCAGCACCACTGGCACTAACTC
Ca_LG_6:12325718	GAGTTACTAGCAGCTAACTTGAAACTTTGTTGTTAGCATAAACTGGAACAGAAACTACTGTCGGAATTGAGCTAAGGATAATGCTCTTCTGATTGTACAA	A/G	ATACTTTTTGAGTACTTTTTTGGAAATGCTTCCCTTCTTTAAGCTTCACCAGCTGCTGCCAGTATATGTTACTTCTTTTGTTGGTTACTTTGTAATCGTA
Ca_LG_6:12325750	TTAGCATAAACTGGAACAGAAACTACTGTCGGAATTGAGCTAAGGATAATGCTCTTCTGATTGTACAAATACTTTTTGAGTACTTTTTTGGAAATGCTTC	C/T	CCTTCTTTAAGCTTCACCAGCTGCTGCCAGTATATGTTACTTCTTTTGTTGGTTACTTTGTAATCGTAGTAACCACAAATTTTATGGCTTTCTCTCACCT
Ca_LG_6:12330400	GATGGGATCCACGTGTCCACCTCCAATATCAAATGGGTCTGCTTCCTTGTGTGTGGGACCTTCTTCAGATATTAGGCTTCCATCAGTTCCACTTTGGGAG	G/T	GCTGCACTTTTCAATGTTACAATTAGTTAGTTTTGATAATGTGTTTGGATCATATTTTGATCTCGAATATAATTGATTTTGTAAAACTAATTTGATTATA
Ca_LG_6:12331372	GTGACCAAGTTGGGTGTTTGGATTTGATGAGTGCTGCTATTCCTGCCACATGGGGACAAGACATTGATGTTCCAGATAAAAATGTAAATCCACTGCTCTC	C/T	TTTACTGTTCTTTGGTGGAAATGCAGCCAAAATGTCCACACCAGGTGCAGCTATGTCAGGCTATATCTCCATGAACCATTCCCAGAAGAGTACAGGTTTT
Ca_LG_6:12362493	AAAACCCTGTTCCCGCCATTTACATACGTATAACTAGGCGCCAAATTTCACTGTTCGAAAGAGCGCAGCAGCCATGAACGTGACCCGAAACTCCCGCAAG	G/A	CGGAGCCACAAATCTCAGCCCATCTCTGGTAGATTCACCCGCAGCAGTTCTCAGCTCTTTTTACACCGTAACCGCTCCGGTCAAATTCGCCCCGACCCCG
Ca_LG_6:12362511	TTTACATACGTATAACTAGGCGCCAAATTTCACTGTTCGAAAGAGCGCAGCAGCCATGAACGTGACCCGAAACTCCCGCAAGCGGAGCCACAAATCTCAG	G/A	CCCATCTCTGGTAGATTCACCCGCAGCAGTTCTCAGCTCTTTTTACACCGTAACCGCTCCGGTCAAATTCGCCCCGACCCCGTTCGGTTTCCGAGACAGA
Ca_LG_6:12433128	AGCATCAAAGTATATTCCAAATGGTGGCTAATGGCTATGCTCAATTTCTGAAAAAGATGGACTAAGAGGAAAGAGATTGGGGGTAGTAATATTAAACAAT	T/G	ATTGGAAATTACACTTTTTGGCATGAAACTTTCAAGCTGCACCTGAAAATGTTAAGGTATCAAAAGTTATTATGTGACTGAATTCTAATACTTGTAAGGA
Ca_LG_6:12851342	TGGAATTGGAAAAAGTGTACTTGGTCTTTGAAATCTTTATATGGTTTTGGTTAATGAAACAAGGCTGGAGGCTACAAGGCAGCAACCAATCCTTTTTTTC	C/A	TGTTCTTTTTGGGCATTAGTAGAATATACGATGTGCAGCACAACAACAACCACTGATCCTTCTTTTCTCTTCTTTTTGGGAATTAGTGGGATGTACAATG
Ca_LG_6:12973204	CATTAAGTTACACATCCAATTATATTTTTCAAGATTTCATGCTCTTAAATACATATAAATAACTTTGAATAAGATGAAAAGGGTCAAAACTCAAATTTTC	C/A	CTTTTCAAATATGATTTCAATACCTTAGGAGCACAATATCCAAGTGTTTCTTCTATTGATGGATGATCGTCACCTAAAAGATGCCTAACAGTGCCCAAAA

Positions	-100 bp flanking sequences	SNPs within *kabuli* cultivar	+100 bp flanking sequences
Ca_LG_6:12973227	ATTTTTCAAGATTTCATGCTCTTAAATACATATAAATAACTTTG AATAAGATGAAAAGGGTCAAAACTCAAATTTTCCTTTTCAAATA TGATTTCAATAC	C/T	CTTAGGAGCACAATATCCAAGTGTTTCTTCTATTGATGGATG ATCGTCACCTAAAAGATGCCTAACAGTGCCCAAAATATCTTT TTTTTTCCAACATGAC
Ca_LG_6:13350032	NNNNNNNNNNNNNNNNNNNNNNNNNNNNNNNNNNNNNNNNN NNNNNNNNNNNNNNNNNNNNNNNNNNNNNGGCAGTAAGAAT CAAGCATTTTTCATAGGC	C/T	ATCTTTACTTATGAGTTATATATATATGTCCATTCATTCTATTC TATTTTTCCAGGCATATATTCAGCAACTTGAATCAAGCAGGA TTAGGCTTAATCAG
Ca_LG_6:13668978	GTTTTTTATACAAGCCCTTGTAGAACATGTGCCAATTGCCGAA ACATGTGTGAGCCGTGTGCAGCACACAGCCTTAGCAGTTAGC ACCCATAAACTCTTT	T/C	TGTAGTGTCAAATTGCTTTCCACTAATTTTGCGGTCTTGCTTT CACAAAGCTTGGGGACACTAGTTTTGAAGCATATGGATGAT GGAATCACAAAACCCG
Ca_LG_6:14673690	CAATCGAATACGAGAGAAGGTCTTCCTAGAAAGATCAATTGCT GTGATAACATCTTCCAAATTATCTCTCATCAACACATAGTTAGC AGCTTCTATTGCA	A/T	ATATCTGTCCCAGCACCAATTGCCATACCAACATCTGCAGCA GCTAATGCAGGAGAGTCATTTATACCATCACCCACCATTGCA ACTATACTTCCATCTT
Ca_LG_6:14673693	TCGAATACGAGAGAAGGTCTTCCTAGAAAGATCAATTGCTGT GATAACATCTTCCAAATTATCTCTCATCAACACATAGTTAGCA GCTTCTATTGCAATA	A/G	TCTGTCCCAGCACCAATTGCCATACCAACATCTGCAGCAGCT AATGCAGGAGAGTCATTTATACCATCACCCACCATTGCAACT ATACTTCCATCTTTTT
Ca_LG_6:14673717	AGAAAGATCAATTGCTGTGATAACATCTTCCAAATTATCTCTC ATCAACACATAGTTAGCAGCTTCTATTGCAATATCTGTCCCAG CACCAATTGCCATA	A/T	CCAACATCTGCAGCAGCTAATGCAGGAGAGTCATTTATACCA TCACCCACCATTGCAACTATACTTCCATCTTTTTGGAATGAA CGAACAACATCAGCTT
Ca_LG_6:14987662	TTTATATAGTCCCCCGGCTTATCACCGACATAAGCCGACAGTC TATTCAGGGTTCCAAACTCAATGTTGGCAACTAAACACAAAGG TTGCGCTCGTTGCA	A/G	GGACTTAACCCAACACCTTACGGCACGAGCTGACGACAGCC ATGCACCACCTGTCCGCGTTCCCGAAGGCATCCCTCTCTTT CAAGAGGATTCGCGACAT
Ca_LG_6:14987678	GCTTATCACCGACATAAGCCGACAGTCTATTCAGGGTTCCAA ACTCAATGTTGGCAACTAAACACAAAGGTTGCGCTCGTTGCA GGACTTAACCCAACAC	C/T	CTTACGGCACGAGCTGACGACAGCCATGCACCACCTGTCCG CGTTCCCGAAGGCATCCCTCTCTTTCAAGAGGATTCGCGAC ATGTCAAGCCTTGGTAAG
Ca_LG_6:14987681	TATCACCGACATAAGCCGACAGTCTATTCAGGGTTCCAAACTC AATGTTGGCAACTAAACACAAAGGTTGCGCTCGTTGCAGGAC TTAACCCAACACCTT	T/C	ACGGCACGAGCTGACGACAGCCATGCACCACCTGTCCGCG TTCCCGAAGGCATCCCTCTCTTTCAAGAGGATTCGCGACAT GTCAAGCCTTGGTAAGATT
Ca_LG_6:14987685	ACCGACATAAGCCGACAGTCTATTCAGGGTTCCAAACTCAAT GTTGGCAACTAAACACAAAGGTTGCGCTCGTTGCAGGACTTA ACCCAACACCTTACGG	G/A	CACGAGCTGACGACAGCCATGCACCACCTGTCCGCGTTCCC GAAGGCATCCCTCTCTTTCAAGAGGATTCGCGACATGTCAA GCCTTGGTAAGATTCTTC
Ca_LG_6:16141527	GAAATAACAACATTGTGATTTTGTGAAGGCTTAGGCAACCATA AAATCAAGCTAAATATTTAAGATTTTTGTGAGGGCTTTTTCAGC CACAAAATCAACT	T/C	CTTTGTGAGGGTAAAGGCTGCCACAAAGGCAAGCCATTTGC GGCCGCCCATGCCGCCACAAAGGCAAGCCATTTACGGCCG CCACAAAGGCAAGCCGTTT
Ca_LG_6:16141528	AAATAACAACATTGTGATTTTGTGAAGGCTTAGGCAACCATAA AATCAAGCTAAATATTTAAGATTTTTGTGAGGGCTTTTTCAGC CACAAAATCAACTC	C/T	TTTGTGAGGGTAAAGGCTGCCACAAAGGCAAGCCATTTGCG GCCGCCCATGCCGCCACAAAGGCAAGCCATTTACGGCCGC CACAAAGGCAAGCCGTTTG

Positions	-100 bp flanking sequences	SNPs within *kabuli* cultivar	+100 bp flanking sequences
Ca_LG_6:16141538	ATTGTGATTTTGTGAAGGCTTAGGCAACCATAAAATCAAGCTAAATATTTAAGATTTTTGTGAGGGCTTTTTCAGCCACAAAATCAACTCTTTGTGAGGG	G/T	TAAAGGCTGCCACAAAGGCAAGCCATTTGCGGCCGCCCATGCCGCCACAAAGGCAAGCCATTTACGGCCGCCACAAAGGCAAGCCGTTTGCGGCCGCCCA
Ca_LG_6:16158052	CGCACACAATGAAAGTGGTTGTGTCAGAAAACTTTTCCAGCAGCGATGGCAGGCAACAGTAGCAGACAGGGCAACAACGACGACAGGCAGCAGACGGCGG	G/A	CAGCGACAACTCTACTTGCATGAGAGATTAGCAACGCGTGTGACGGCTTTTGGCGACGCGTTTTCAGGCATGAACTGATCTGAAGATGACTGCTATTCAT
Ca_LG_6:16836434	TGGCTCATTCGCCGCCGCGATGAAGCTTCGTAATGTTACGGTAGTTACAACTACCATGAATGTTGCTGCGCCTTATAGTGAGACTGTTGCGTTGAGGGGG	G/A	CTTGTACCGCTTCACGTGCCGTTGCAGCAACGGTTGCCGATTTTCGACGGTGTTGTGGATATTGTTCGGTGTGGGCGTGCGGTGAACCGGTGGATTCCGG
Ca_LG_6:16939746	AAATCAAACTAATAGTAATTGTTCTGCCTAGGCCTGATTGTAAAATCCTTTCAACTTTAAATTAACGAATACTAATTATTAAGGCTAAGCTGCCTATGCC	C/T	TGCGGAACTAACTTTTCAAGACATCCTAGCCACACGTGTTTTCATTAGCTATATGTCCAAGCAGCTAGCTGTATTCCCAGCCAATCATGATTTCAAACCC
Ca_LG_6:17125227	CTCCTCTTGAGTTGAATGTGAAATATGCTTCTTCAGATGTTGTTCCTTTACCATATCTCACTTTGTATTGTACTTTGATTGACAGCTTGATGTATCTTAC	C/T	GATTACCAAACCTGATATTGCTTATGTTGTTCATGTTGTCAGTCAGTTTGTTGTGTCTTCCACTACAATACATTGTGATGTAGCTAAATATCACTAGAGG
Ca_LG_6:17125266	TTGTTCCTTTACCATATCTCACTTTGTATTGTACTTTGATTGACAGCTTGATGTATCTTACGATTACCAAACCTGATATTGCTTATGTTGTTCATGTTGT	T/A	CAGTCAGTTTGTTGTGTCTTCCACTACAATACATTGTGATGTAGCTAAATATCACTAGAGGGGGGGGGGGGGTTGAATATGGATTTTGCTGTTTAAAAATA
Ca_LG_6:17125282	TCTCACTTTGTATTGTACTTTGATTGACAGCTTGATGTATCTTACGATTACCAAACCTGATATTGCTTATGTTGTTCATGTTGTCAGTCAGTTTGTTGTG	G/C	TCTTCCACTACAATACATTGTGATGTAGCTAAATATCACTAGAGGGGGGGGGGGGTTGAATATGGATTTTGCTGTTTAAAAATAATTTTCAAGAGATTGT
Ca_LG_6:17125284	TCACTTTGTATTGTACTTTGATTGACAGCTTGATGTATCTTACGATTACCAAACCTGATATTGCTTATGTTGTTCATGTTGTCAGTCAGTTTGTTGTGTC	C/A	TTCCACTACAATACATTGTGATGTAGCTAAATATCACTAGAGGGGGGGGGGGTTGAATATGGATTTTGCTGTTTAAAAATAATTTTCAAGAGATTGTAA
Ca_LG_6:17462641	CATTTAATCATGTAAAAGCTGAAATTATTGTTTTGTTTGGGTGAATTATCTGCTGCCTAACAATCAAATGGTTGTGATAAGAAGTGTTCAATTTTCCGCA	A/G	CTCTTGCTACCATTTAGAGATCCTCCTTGGATAATTCACCCAGCTGAAGGGCAGCTATAGTCTCCTTAAGTTATCTGACTAATCAAGTAAACTCTAGTCT
Ca_LG_6:18143990	TCATAAGCTTTCTTTGAGGCTTTAAATGAATGAAACCTTTTCTATAGGCAATGGTAAGTGGTTAGTATGTTATGAGATTTGGATTTACTGCAGCCAGCAT	T/C	GTTAGTGGTCGACGATTCGAGATCAAAGGATCTAGATGTCAATTTAGATTTTGATTGTTTTAAAAAAAAACTGAAATATCAAGCTTAAAGTTTGGACCAT
Ca_LG_6:18395040	ATATTGTTGTTACTAAAGAAATAAAGGAGGCTATTTGGATGGGAGGAATGGTTAAGAGCATGGAGACAAAACAAGTTGTTACCAAGGTGTTCTGTGATAA	A/G	TCAAAGTAGTATTCACTTGGCAAGAAATCAAGTATACCATGATGAGAGGACAAAACACATAGATGACAAGCTACATTTTGTGAGAGAAGTCATTGCAAAA
Ca_LG_6:18395048	GTTACTAAAGAAATAAAGGAGGCTATTTGGATGGGAGGAATGGTTAAGAGCATGGAGACAAAACAAGTTGTTACCAAGGTGTTCTGTGATAATCAAAGTA	A/G	GTATTCACTTGGCAAGAAATCAAGTATACCATGATGAGAGGACAAAACACATAGATGACAAGCTACATTTTGTGAGAGAAGTCATTGCAAAAGGAAAGAT

Positions	-100 bp flanking sequences	SNPs within *kabuli* cultivar	+100 bp flanking sequences
Ca_LG_6:19731770	TCCAAGAAGGGATGACACAACAAGAGTCAACGACAATGGGGATGATCAAATGGCCGAAGCTATGAATAACATGGTTACTTCTGTTGCTGCATAGACTCTG	G/A	CGAGATCTGGAGAAGAGGGAAATAGAGATATGTGCTGCTGCGTCAAGGGGATTAGAAAATTTTCGTCGTTACCATCCTCCAAAGTTCAAGGGTGATGAGA
Ca_LG_6:21241988	AACCAATAATGGTGGTGGTGGGTTCACGGCTTTGGACGAAGGCAGCGCCTGGGTTCGCGGCAGTGAGAACGAGGAAAATTGAGAGTTGGTGAATGAGGGT	T/G	AAAAGAGAAACAAATCAAGAGGGTTTTCTGGTGAGTGAGGGAAAACGAGGGCGACACAATACGAAGAAGGAAGGAAAGAGGCGAAGGCGGCGCCTGCAAG
Ca_LG_6:21241993	ATAATGGTGGTGGTGGGTTCACGGCTTTGGACGAAGGCAGCGCCTGGGTTCGCGGCAGTGAGAACGAGGAAAATTGAGAGTTGGTGAATGAGGGTAAAAG	G/A	AGAAACAAATCAAGAGGGTTTTCTGGTGAGTGAGGGAAAACGAGGGCGACACAATACGAAGAAGGAAGGAAAGAGGCGAAGGCGGCGCCTGCAAGGCTGC
Ca_LG_6:21241997	TGGTGGTGGTGGGTTCACGGCTTTGGACGAAGGCAGCGCCTGGGTTCGCGGCAGTGAGAACGAGGAAAATTGAGAGTTGGTGAATGAGGGTAAAAGAGAA	A/G	ACAAATCAAGAGGGTTTTCTGGTGAGTGAGGGAAAACGAGGGCGACACAATACGAAGAAGGAAGGAAAGAGGCGAAGGCGGCGCCTGCAAGGCTGCCGCC
Ca_LG_6:21241998	GGTGGTGGTGGGTTCACGGCTTTGGACGAAGGCAGCGCCTGGGTTCGCGGCAGTGAGAACGAGGAAAATTGAGAGTTGGTGAATGAGGGTAAAAGAGAAA	A/G	CAAATCAAGAGGGTTTTCTGGTGAGTGAGGGAAAACGAGGGCGACACAATACGAAGAAGGAAGGAAAGAGGCGAAGGCGGCGCCTGCAAGGCTGCCGCCG
Ca_LG_6:21241999	GTGGTGGTGGGTTCACGGCTTTGGACGAAGGCAGCGCCTGGGTTCGCGGCAGTGAGAACGAGGAAAATTGAGAGTTGGTGAATGAGGGTAAAAGAGAAAC	C/G	AAATCAAGAGGGTTTTCTGGTGAGTGAGGGAAAACGAGGGCGACACAATACGAAGAAGGAAGGAAAGAGGCGAAGGCGGCGCCTGCAAGGCTGCCGCCGT
Ca_LG_6:21242042	TCGCGGCAGTGAGAACGAGGAAAATTGAGAGTTGGTGAATGAGGGTAAAAGAGAAACAAATCAAGAGGGTTTTCTGGTGAGTGAGGGAAAACGAGGGCGA	A/G	CACAATACGAAGAAGGAAGGAAAGAGGCGAAGGCGGCGCCTGCAAGGCTGCCGCCGTGGGATTAGGGTGAAAAAGATAGAAAGAACAAAAGTAAAGGAAA
Ca_LG_6:22563748	TAAAAATAAGAATGCAGGCTGCATAAAAATAAGAATGCATTGCATTAACCCCCAAATCCGATGACAGAAAAAATACATATAAGATTTATATCGAATTTTA	A/G	TTCCTTAAATAAAATTATATTTAATTTTGTTTGTGCCACAAGTTCTAATACAAGAAAGCTGCAGGCTGCATACTTTTAGCATCAAATAGAAAGTTGCAGG
Ca_LG_6:22628491	AATAGACATGCAGCAACAGCTATGACTGGAGCCAGAAGAACAGAAGCCATCACTGGAATTGGAAACAGGAGACCAATAACTCAAGCCATCCCATACAAAG	G/T	ACCTTTGCAAGAGCAGCATGATCATCATACCAATGGAGACATAAATGAAGAAAGAGTGGAGTCAAGAAGACCTCAAAGGGCAAGGCAACTACCAACACAT
Ca_LG_6:22687498	AGTTTTTCAATTATAAAAATAGGTCATGTTTGACACATATGCAGATTCTTCAGTATTTCTCTTTTTTCCGCTATTGCCGCTATGTCAGCTAGCAGAAACA	A/T	GGAAGCAGCGGCCGCTATTGCCCTTATAGTGAAACTAGGATAGCGCAGCGATTTTCATAGCAAACTCATGAAAATCTGCCTATGCTATAGGGCTATCGCC
Ca_LG_6:23853490	TTGCTGTCGAAGCGCTAAACTTAGCTAAAGATTTCAACATGTTGTCCTATATATAATTCTCTTCTGCGGCTACTACACTTTCTTTTAGCTTCTATTTTCT	T/C	TAAGTTGGATGAGGAAACATCATGTGAGTACAGAGATCTACCGGAGCCTATCAAAAAACCAGGTTGCATACCACTCCATATGGCAGCGATCTTCTTACAC
Ca_LG_6:23853533	GTCCTATATATAATTCTCTTCTGCGGCTACTACACTTTCTTTTAGCTTCTATTTTCTTAAGTTGGATGAGGAAACATCATGTGAGTACAGAGATCTACCG	G/A	GAGCCTATCAAAAAACCAGGTTGCATACCACTCCATATGGCAGCGATCTTCTTACACCGGCTCAAGATAGATGGAGTCAAGCTTACAAACACTTCCTCCA

**Positions**	**-100 bp flanking sequences**	**SNPs within *kabuli* cultivar**	**+100 bp flanking sequences**
Ca_LG_6:23853547	TCTCTTCTGCGGCTACTACACTTTCTTTTAGCTTCTATTTTCTTAAGTTGGATGAGGAAACATCATGTGAGTACAGAGATCTACCGGAGCCTATCAAAAA	A/T	ACCAGGTTGCATACCACTCCATATGGCAGCGATCTTCTTACACCGGCTCAAGATAGATGGAGTCAAGCTTACAAACACTTCCTCCAACATTCTAAAACCA
Ca_LG_6:23858669	AGCAGCCACAAAATCCACTTAAATTTTTAAGATTTTTGTGAAGGCTTTTTCAGCCACAAAATCTAAATTTTGTGAGGGTCAAGGTCGCCACAAAATCCTG	G/A	AAGTGACATTACAATTTGTGGCTGCAAAGGCCGCCACAAAAGCCTGAGTCGTTATATTTTGTGGCTGCAAAGGCCGCCACAAAGGAACCAAAGTAACGTT
Ca_LG_6:23858693	TTTTAAGATTTTTGTGAAGGCTTTTTCAGCCACAAAATCTAAATTTTGTGAGGGTCAAGGTCGCCACAAAATCCTGAAGTGACATTACAATTTGTGGCTG	G/A	CAAAGGCCGCCACAAAAGCCTGAGTCGTTATATTTTGTGGCTGCAAAGGCCGCCACAAAGGAACCAAAGTAACGTTGCATTTTTTGCTGTTCAGGCCGCC
Ca_LG_6:23858700	ATTTTTGTGAAGGCTTTTTCAGCCACAAAATCTAAATTTTGTGAGGGTCAAGGTCGCCACAAAATCCTGAAGTGACATTACAATTTGTGGCTGCAAAGGC	C/T	CGCCACAAAAGCCTGAGTCGTTATATTTTGTGGCTGCAAAGGCCGCCACAAAGGAACCAAAGTAACGTTGCATTTTTTGCTGTTCAGGCCGCCACAAAGT
Ca_LG_6:23858712	GCTTTTTCAGCCACAAAATCTAAATTTTGTGAGGGTCAAGGTCGCCACAAAATCCTGAAGTGACATTACAATTTGTGGCTGCAAAGGCCGCCACAAAAGC	C/T	CTGAGTCGTTATATTTTGTGGCTGCAAAGGCCGCCACAAAGGAACCAAAGTAACGTTGCATTTTTTGCTGTTCAGGCCGCCACAAAGTTTTAAACCGTTA
Ca_LG_6:23858718	TCAGCCACAAAATCTAAATTTTGTGAGGGTCAAGGTCGCCACAAAATCCTGAAGTGACATTACAATTTGTGGCTGCAAAGGCCGCCACAAAAGCCTGAGT	T/C	CGTTATATTTTGTGGCTGCAAAGGCCGCCACAAAGGAACCAAAGTAACGTTGCATTTTTTGCTGTTCAGGCCGCCACAAAGTTTTAAACCGTTACCTTTT
Ca_LG_6:23858724	ACAAAATCTAAATTTTGTGAGGGTCAAGGTCGCCACAAAATCCTGAAGTGACATTACAATTTGTGGCTGCAAAGGCCGCCACAAAAGCCTGAGTCGTTAT	T/C	ATTTTGTGGCTGCAAAGGCCGCCACAAAGGAACCAAAGTAACGTTGCATTTTTTGCTGTTCAGGCCGCCACAAAGTTTTAAACCGTTACCTTTTGTGGCT
Ca_LG_6:23858730	TCTAAATTTTGTGAGGGTCAAGGTCGCCACAAAATCCTGAAGTGACATTACAATTTGTGGCTGCAAAGGCCGCCACAAAAGCCTGAGTCGTTATATTTTG	G/T	TGGCTGCAAAGGCCGCCACAAAGGAACCAAAGTAACGTTGCATTTTTTGCTGTTCAGGCCGCCACAAAGTTTTAAACCGTTACCTTTTGTGGCTGCAAAG
Ca_LG_6:23965648	TATTATATTTTTGTAATTTGTCAGGAGTGAAGAGACCAAAATATTGTTCAGTTGCTGCACCAGGCTTCTGATTTTCATCAAACATAGCAAACAAATAAGC	C/T	CTCAATAGGTTGACCAGGCCGCATAGGAGTTCCAATCCCACTATTGACATGATTAATCAAATTAGTATAATATGCAGCAGCATTTTCAGTTGTGGCCCCT
Ca_LG_6:23965669	CAGGAGTGAAGAGACCAAAATATTGTTCAGTTGCTGCACCAGGCTTCTGATTTTCATCAAACATAGCAAACAAATAAGCCTCAATAGGTTGACCAGGCCG	G/T	CATAGGAGTTCCAATCCCACTATTGACATGATTAATCAAATTAGTATAATATGCAGCAGCATTTTCAGTTGTGGCCCCTTCCCCACCTGCAGATGGCCAT
Ca_LG_6:23965683	CCAAAATATTGTTCAGTTGCTGCACCAGGCTTCTGATTTTCATCAAACATAGCAAACAAATAAGCCTCAATAGGTTGACCAGGCCGCATAGGAGTTCCAA	A/T	TCCCACTATTGACATGATTAATCAAATTAGTATAATATGCAGCAGCATTTTCAGTTGTGGCCCCTTCCCCACCTGCAGATGGCCATCCACTCTCAGACAC
Ca_LG_6:24202956	TTTTTTCAGCCACAAAATCAACCATTTGTGTAGGTAAAGGCCGCTACAAAATTTCAAAGTGACGTTACCTTTTGTAGATGCAAAGGCCGCCACAAAAGCT	T/C	TGAGCTGTTACCTTTTGTGGCTGCAAAGGCCGCCACAAAATCCCAAAGTGACGTTGCCTTTTGTGACCGTCCAGGCCGCCACAAACGCTTATAGTCGTTG

Positions	-100 bp flanking sequences	SNPs within *kabuli* cultivar	+100 bp flanking sequences
Ca_LG_6:24222466	TGAATTTTGTACAAACCTGAAAGCAAGCACCACAAGCAGCTCCATCATAGAAAAGTGAAGAAACACCAGCTGCAAGTTGTCCACCACTAAAGTCCAATGC	C/G	TAAAGAACCATACCCACATGCCCCAGCTGCATAAAAATAAATATAAAAATTAAACAATGAAAAAATATTTGTTAACAAAAGAACTCTATATGAAATGAAA
Ca_LG_6:24222480	ACCTGAAAGCAAGCACCACAAGCAGCTCCATCATAGAAAAGTGAAGAAACACCAGCTGCAAGTTGTCCACCACTAAAGTCCAATGCTAAAGAACCATACC	C/T	CACATGCCCCAGCTGCATAAAAATAAATATAAAAATTAAACAATGAAAAAATATTTGTTAACAAAAGAACTCTATATGAAATGAAAATTGTTAATTACAT
Ca_LG_6:24222487	AGCAAGCACCACAAGCAGCTCCATCATAGAAAAGTGAAGAAACACCAGCTGCAAGTTGTCCACCACTAAAGTCCAATGCTAAAGAACCATACCCACATGC	C/T	CCCAGCTGCATAAAAATAAATATAAAAATTAAACAATGAAAAAATATTTGTTAACAAAAGAACTCTATATGAAATGAAAATTGTTAATTACATGAGAGAG
Ca_LG_6:25357840	CCCTACTCCAATTACCACCCACACCTTTGACAAGTCCTCCTGTTTTACAACCACCTCTGGTGAATCCAATGCCTTAGGCGAGCTCAGTGCTGCCTGCATT	T/C	AACCCTATCAACCTCTCTGCAAATTTGCAAAAACTCTCAGTGACAATAACTTTCTCTGGTGGAAGCAGCAAAGTAAGCTAGTTTTGAAGGGTCATCGCCA
Ca_LG_6:25407372	AACGCTATTTTCCATGATGCAACATTGGCTGCCGCTTGCTACAACTCAAACATTTCGAATTACTGGAATTGTTGTTGATGCATGAACAATAGGTTACTCG	G/A	ATCAGTGGAATTTGTTTCTAGTAAATGCAAGGTAACACTGCCTAGTACCTACAATATATTCACAATGAGTTTGACCCTTCACTTGACCCGATTACGGCAG
Ca_LG_6:25407394	CATTGGCTGCCGCTTGCTACAACTCAAACATTTCGAATTACTGGAATTGTTGTTGATGCATGAACAATAGGTTACTCGATCAGTGGAATTTGTTTCTAGT	T/C	AAATGCAAGGTAACACTGCCTAGTACCTACAATATATTCACAATGAGTTTGACCCTTCACTTGACCCGATTACGGCAGCAGCTCTATAGTATCATGTTTC
Ca_LG_6:25407408	TGCTACAACTCAAACATTTCGAATTACTGGAATTGTTGTTGATGCATGAACAATAGGTTACTCGATCAGTGGAATTTGTTTCTAGTAAATGCAAGGTAAC	C/G	ACTGCCTAGTACCTACAATATATTCACAATGAGTTTGACCCTTCACTTGACCCGATTACGGCAGCAGCTCTATAGTATCATGTTTCCCTATCTTAACTCT
Ca_LG_6:25407425	TTCGAATTACTGGAATTGTTGTTGATGCATGAACAATAGGTTACTCGATCAGTGGAATTTGTTTCTAGTAAATGCAAGGTAACACTGCCTAGTACCTACA	A/C	ATATATTCACAATGAGTTTGACCCTTCACTTGACCCGATTACGGCAGCAGCTCTATAGTATCATGTTTCCCTATCTTAACTCTCCCAAATTCAATAACAC
Ca_LG_6:25407427	CGAATTACTGGAATTGTTGTTGATGCATGAACAATAGGTTACTCGATCAGTGGAATTTGTTTCTAGTAAATGCAAGGTAACACTGCCTAGTACCTACAAT	T/C	ATATTCACAATGAGTTTGACCCTTCACTTGACCCGATTACGGCAGCAGCTCTATAGTATCATGTTTCCCTATCTTAACTCTCCCAAATTCAATAACACAA
Ca_LG_6:25407433	ACTGGAATTGTTGTTGATGCATGAACAATAGGTTACTCGATCAGTGGAATTTGTTTCTAGTAAATGCAAGGTAACACTGCCTAGTACCTACAATATATTC	C/T	ACAATGAGTTTGACCCTTCACTTGACCCGATTACGGCAGCAGCTCTATAGTATCATGTTTCCCTATCTTAACTCTCCCAAATTCAATAACACAAAAAAAA
Ca_LG_6:25599940	NNNNNNNNNNNNNNNNNNNNNNNNNNNNNNNNNNNNNNNNNNNNTTCTAAGTTTGCTGCCCCATTTTTAAAAAGGGGGAAAAAATTCAAATTTTTAAAA	A/T	TGTTAGAAAAATCTGGTATTCTAGTTTTTTGAAAAAGTGTATAATCTTAAGGGATAAAATCCGGTAAATTCTAAGTTTGCTGCCCTTTTTTTGAAAATCT
Ca_LG_6:25599980	NNNNNTTCTAAGTTTGCTGCCCCATTTTTAAAAAGGGGGAAAAAATTCAAATTTTTAAAATGTTAGAAAAATCTGGTATTCTAGTTTTTTGAAAAAGTGT	T/A	ATAATCTTAAGGGATAAAATCCGGTAAATTCTAAGTTTGCTGCCCTTTTTTTGAAAATCTGGTATTCTGTTTCAGATTTTTAAGGTGTAAAAAAATTTGGA

**Positions**	**-100 bp flanking sequences**	**SNPs within *kabuli* cultivar**	**+100 bp flanking sequences**
Ca_LG_6:25599994	TGCTGCCCCATTTTTAAAAAGGGGGAAAAAATTCAAATTTTTAAAATGTTAGAAAAATCTGGTATTCTAGTTTTTTGAAAAAGTGTATAATCTTAAGGGA	A/G	TAAAATCCGGTAAATTCTAAGTTTGCTGCCCTTTTTTTGAAAATCTGGTATTCTGTTTCAGATTTTTAAGGTGTAAAAAATTTGGAACATCTTTATAATC
Ca_LG_6:25600066	TTTTGAAAAAGTGTATAATCTTAAGGGATAAAATCCGGTAAATTCTAAGTTTGCTGCCCTTTTTTTGAAAATCTGGTATTCTGTTTCAGATTTTTAAGGT	T/G	GTAAAAAATTTGGAACATCTTTATAATCTTAACTCTTATTTTAAAATTTTTAAAGTTTTTAAATGAATTTGAGTTTTAATTTTAATATTATAAGTCTATT
Ca_LG_6:26811992	GATTATTCTTTTATATGTAATGTAGAAACATTAAAACAACTTATGAAAATGGTTTCGAATTTCTTAACCTTACTAATGTTGTTCCTTGCTGCTCTTATCT	T/C	TTGTTCCACAAGGACTTGCTGATGTAAGCCCACAAATCTCCAAGACACCAATTCCTAAACCACCTAATTACGACAAAAAAGGAGAACTCGACATTATTAG
Ca_LG_6:27599897	AGAAAGTCTAGCTTGTCAACCTTTCAGATATAGCGAGCCTCATTTACATCGATTAATCCAGGAAGTGCATAGAGGATGATAGCAGCAAGGATAGCCATAA	A/T	GGGTGTAGTATAAGAGCCTTGCAAATAATTGCAGGAACAAAATCACACTAATGGCCATCACAATATTTGAGACTGTTGTTTGACATTTTACACTATAGTT
Ca_LG_6:27599902	GTCTAGCTTGTCAACCTTTCAGATATAGCGAGCCTCATTTACATCGATTAATCCAGGAAGTGCATAGAGGATGATAGCAGCAAGGATAGCCATAAGGGTG	G/A	TAGTATAAGAGCCTTGCAAATAATTGCAGGAACAAAATCACACTAATGGCCATCACAATATTTGAGACTGTTGTTTGACATTTTACACTATAGTTAACTG
Ca_LG_6:27599910	TGTCAACCTTTCAGATATAGCGAGCCTCATTTACATCGATTAATCCAGGAAGTGCATAGAGGATGATAGCAGCAAGGATAGCCATAAGGGTGTAGTATAA	A/G	GAGCCTTGCAAATAATTGCAGGAACAAAATCACACTAATGGCCATCACAATATTTGAGACTGTTGTTTGACATTTTACACTATAGTTAACTGTTGTCCTT
Ca_LG_6:29978947	CTTGGATCCATTGATCCGCCTTCTCTGAATTCTCATCCCCCTTAAACTTGGGAGGATTGTAACGACGAAAGTCTTCTAATCCTCTTGACTCTGCAGCACG	G/A	GATCTCTCTTTCCCTCTTTTCAAGATCACGTCGAGTCTTGGCAGCAGTCTGTGCAGCAACAAAAGCAACCATGTTATTCATAGCTTCCGCCATTCGATCG
Ca_LG_6:29978955	CATTGATCCGCCTTCTCTGAATTCTCATCCCCCTTAAACTTGGGAGGATTGTAACGACGAAAGTCTTCTAATCCTCTTGACTCTGCAGCACGGATCTCTC	C/T	TTTCCCTCTTTTCAAGATCACGTCGAGTCTTGGCAGCAGTCTGTGCAGCAACAAAAGCAACCATGTTATTCATAGCTTCCGCCATTCGATCGTCCCTATT
Ca_LG_6:29978961	TCCGCCTTCTCTGAATTCTCATCCCCCTTAAACTTGGGAGGATTGTAACGACGAAAGTCTTCTAATCCTCTTGACTCTGCAGCACGGATCTCTCTTTCCC	C/A	TCTTTTCAAGATCACGTCGAGTCTTGGCAGCAGTCTGTGCAGCAACAAAAGCAACCATGTTATTCATAGCTTCCGCCATTCGATCGTCCCTATTAGCATT
Ca_LG_6:29978996	GGGAGGATTGTAACGACGAAAGTCTTCTAATCCTCTTGACTCTGCAGCACGGATCTCTCTTTCCCTCTTTTCAAGATCACGTCGAGTCTTGGCAGCAGTC	C/A	TGTGCAGCAACAAAAGCAACCATGTTATTCATAGCTTCCGCCATTCGATCGTCCCTATTAGCATTGGCTCTTGGAACGTCATTCCTTCTTGGCGGCATGG
Ca_LG_6:29979009	CGACGAAAGTCTTCTAATCCTCTTGACTCTGCAGCACGGATCTCTCTTTCCCTCTTTTCAAGATCACGTCGAGTCTTGGCAGCAGTCTGTGCAGCAACAA	A/T	AAGCAACCATGTTATTCATAGCTTCCGCCATTCGATCGTCCCTATTAGCATTGGCTCTTGGAACGTCATTCCTTCTTGGCGGCATGGTTTTCCTATAAAA
Ca_LG_6:29979042	GCACGGATCTCTCTTTCCCTCTTTTCAAGATCACGTCGAGTCTTGGCAGCAGTCTGTGCAGCAACAAAAGCAACCATGTTATTCATAGCTTCCGCCATTC	C/T	GATCGTCCCTATTAGCATTGGCTCTTGGAACGTCATTCCTTCTTGGCGGCATGGTTTTCCTATAAAACCATCATCAAGTAGAGTCTTAACACTACTTCAA

Positions	-100 bp flanking sequences	SNPs within *kabuli* cultivar	+100 bp flanking sequences
Ca_LG_6:29979074	ACGTCGAGTCTTGGCAGCAGTCTGTGCAGCAACAAAAGCAACCATGTTATTCATAGCTTCCGCCATTCGATCGTCCCTATTAGCATTGGCTCTTGGAACG	G/A	TCATTCCTTCTTGGCGGCATGGTTTTCCTATAAAACCATCATCAAGTAGAGTCTTAACACTACTTCAAATAATTCCATAACTAGCTTCCGACCACATCAA
Ca_LG_6:30005970	AAGCAAAACTTCCTTTTTCCCATCTGGTGCAAGATCAAGCATTTTTATGAAGTTCGCTGCTGATTCTAGCTGTTGTTCGTTTGGCTGACACGATGCTGGA	A/G	AAATTACTGAAAGAAGGAAATTGAAACTCTCGCACGTCCTCGGCAAAAGGAAGAACGTTGAAGTAAAACGAATCAGGCTGCACAGCATAATCAGAGAAGC
Ca_LG_6:30006012	TTTTATGAAGTTCGCTGCTGATTCTAGCTGTTGTTCGTTTGGCTGACACGATGCTGGAAAATTACTGAAAGAAGGAAATTGAAACTCTCGCACGTCCTCG	G/A	GCAAAAGGAAGAACGTTGAAGTAAAACGAATCAGGCTGCACAGCATAATCAGAGAAGCAAGAAATTATTGTTAAATGAAAAAATGAAATCGCAGAATAGA
Ca_LG_6:30214653	GAGTTGGGTTAGGTTCCTCTGTTGCTTGACGATCGATGTCTGCTATATCTTCAAATGACAGCTTTATGCCCAAATGCGCTTGAATTGCAGCCACATCCTA	A/G	AACAACTGAATTCAGGGAAGTCTGAAGAGTGGTCAAGGCAGCTTCCATTCTTGCCTGTGACTCTAGCATTTTGGCATTGTGAGCCATCTGAGCTACTATA
Ca_LG_6:30215552	TTGACGACCGATGTCTGCTATATCTTCAAATGACAGCTTTATGCCCAAATGCGCTTGAATTGCAGCCACATCTTGAACAACTGAATTCAGGAAAGTCTGA	A/G	AGAGTGGTCAAGGCAACTTCCATTCTTGCCTGTGACTCTAGCATTTTGGCATTGTGAGCCACCTGAGCTTCTATAAATTCCATTAATTTTGCGACAAACT
Ca_LG_6:30215558	ACCGATGTCTGCTATATCTTCAAATGACAGCTTTATGCCCAAATGCGCTTGAATTGCAGCCACATCTTGAACAACTGAATTCAGGAAAGTCTGAAGAGTG	G/T	GTCAAGGCAACTTCCATTCTTGCCTGTGACTCTAGCATTTTGGCATTGTGAGCCACCTGAGCTTCTATAAATTCCATTAATTTTGCGACAAACTCAGGAG
Ca_LG_6:30417319	ACGGTATTTGAAGAAGGCACCAAGACAAAGCTTATTTTTCCAATGGACTCAGAACTATAACTTAGTGGTTTTAGTGGTGCTGGCTGGGGAACATGCAAGA	A/G	ACACTAGAAGATCATATTTTGGAAGTAGTATCTTCTTAGGAAACTCATTAATAAGTTGGAAGACTAATAAGCAAACAATAGTAGCAAAATCATCATCAGT
Ca_LG_6:30417335	GCACCAAGACAAAGCTTATTTTTCCAATGGACTCAGAACTATAACTTAGTGGTTTTAGTGGTGCTGGCTGGGGAACATGCAAGAACACTAGAAGATCATA	A/T	TTTTGGAAGTAGTATCTTCTTAGGAAACTCATTAATAAGTTGGAAGACTAATAAGCAAACAATAGTAGCAAAATCATCATCAGTGGCTGAGTATAGGGCA
Ca_LG_6:30417338	CCAAGACAAAGCTTATTTTTCCAATGGACTCAGAACTATAACTTAGTGGTTTTAGTGGTGCTGGCTGGGGAACATGCAAGAACACTAGAAGATCATATTT	T/C	TGGAAGTAGTATCTTCTTAGGAAACTCATTAATAAGTTGGAAGACTAATAAGCAAACAATAGTAGCAAAATCATCATCAGTGGCTGAGTATAGGGCACTT
Ca_LG_6:30417340	AAGACAAAGCTTATTTTTCCAATGGACTCAGAACTATAACTTAGTGGTTTTAGTGGTGCTGGCTGGGGAACATGCAAGAACACTAGAAGATCATATTTTG	G/A	GAAGTAGTATCTTCTTAGGAAACTCATTAATAAGTTGGAAGACTAATAAGCAAACAATAGTAGCAAAATCATCATCAGTGGCTGAGTATAGGGCACTTGC
Ca_LG_6:30417346	AAGCTTATTTTTCCAATGGACTCAGAACTATAACTTAGTGGTTTTAGTGGTGCTGGCTGGGGAACATGCAAGAACACTAGAAGATCATATTTTGGAAGTA	A/T	GTATCTTCTTAGGAAACTCATTAATAAGTTGGAAGACTAATAAGCAAACAATAGTAGCAAAATCATCATCAGTGGCTGAGTATAGGGCACTTGCAACTGC
Ca_LG_6:30417349	CTTATTTTTCCAATGGACTCAGAACTATAACTTAGTGGTTTTAGTGGTGCTGGCTGGGGAACATGCAAGAACACTAGAAGATCATATTTTGGAAGTAGTA	A/G	TCTTCTTAGGAAACTCATTAATAAGTTGGAAGACTAATAAGCAAACAATAGTAGCAAAATCATCATCAGTGGCTGAGTATAGGGCACTTGCAACTGCCAC

Positions	-100 bp flanking sequences	SNPs within *kabuli* cultivar	+100 bp flanking sequences
Ca_LG_6:30417351	TATTTTTCCAATGGACTCAGAACTATAACTTAGTGGTTTTAGTGGTGCTGGCTGGGGAACATGCAAGAACACTAGAAGATCATATTTTGGAAGTAGTATC	C/T	TTCTTAGGAAACTCATTAATAAGTTGGAAGACTAATAAGCAAACAATAGTAGCAAAATCATCATCAGTGGCTGAGTATAGGGCACTTGCAACTGCCACTA
Ca_LG_6:30417365	ACTCAGAACTATAACTTAGTGGTTTTAGTGGTGCTGGCTGGGGAACATGCAAGAACACTAGAAGATCATATTTTGGAAGTAGTATCTTCTTAGGAAACTC	C/T	ATTAATAAGTTGGAAGACTAATAAGCAAACAATAGTAGCAAAATCATCATCAGTGGCTGAGTATAGGGCACTTGCAACTGCCACTAGTGAACTTCAATGA
Ca_LG_6:30835321	TTAATTGACTAACTAAATATAAGAAACAACATACTCAACACACACACACACACGCGTGCGCGTAAACACGCACACACGCGCACACACACGCGCGCACGCG	G/T	CACACACACACACGCACCCACGCACACACGCGCGCGCGCGCACACACACACACACACATACACACACCCCCACACACGCACACACGCGCACATGCAGACA
Ca_LG_6:31061072	ATTCTTTCATTCAACCACTTCAGTAATGTAGAAGAATTCACAATCATGTCTTCCTCCAAATTAAACATCATTGATTTAAGCAACAATGATCTATCAGGGT	T/C	CTTTTCCAACATCTATCTTACAGCTCGGTTCACTTTCTGTCCTCGATGTTTCCTCTAACAGGTTGAATGGGTCGCTGCAGCTAGATAAGTTTTTGGAGCT
Ca_LG_6:31061092	CAGTAATGTAGAAGAATTCACAATCATGTCTTCCTCCAAATTAAACATCATTGATTTAAGCAACAATGATCTATCAGGGTCTTTTCCAACATCTATCTTA	A/C	CAGCTCGGTTCACTTTCTGTCCTCGATGTTTCCTCTAACAGGTTGAATGGGTCGCTGCAGCTAGATAAGTTTTTGGAGCTTAGAAATTTAACGTATCTAG
Ca_LG_6:31061099	GTAGAAGAATTCACAATCATGTCTTCCTCCAAATTAAACATCATTGATTTAAGCAACAATGATCTATCAGGGTCTTTTCCAACATCTATCTTACAGCTCG	G/A	GTTCACTTTCTGTCCTCGATGTTTCCTCTAACAGGTTGAATGGGTCGCTGCAGCTAGATAAGTTTTTGGAGCTTAGAAATTTAACGTATCTAGACCTTTC
Ca_LG_6:31061111	ACAATCATGTCTTCCTCCAAATTAAACATCATTGATTTAAGCAACAATGATCTATCAGGGTCTTTTCCAACATCTATCTTACAGCTCGGTTCACTTTCTG	G/A	TCCTCGATGTTTCCTCTAACAGGTTGAATGGGTCGCTGCAGCTAGATAAGTTTTTGGAGCTTAGAAATTTAACGTATCTAGACCTTTCATTCAATAACTT
Ca_LG_6:31061120	TCTTCCTCCAAATTAAACATCATTGATTTAAGCAACAATGATCTATCAGGGTCTTTTCCAACATCTATCTTACAGCTCGGTTCACTTTCTGTCCTCGATG	G/C	TTTCCTCTAACAGGTTGAATGGGTCGCTGCAGCTAGATAAGTTTTTGGAGCTTAGAAATTTAACGTATCTAGACCTTTCATTCAATAACTTCTCAATCAA
Ca_LG_6:31061125	CTCCAAATTAAACATCATTGATTTAAGCAACAATGATCTATCAGGGTCTTTTCCAACATCTATCTTACAGCTCGGTTCACTTTCTGTCCTCGATGTTTCC	C/G	TCTAACAGGTTGAATGGGTCGCTGCAGCTAGATAAGTTTTTGGAGCTTAGAAATTTAACGTATCTAGACCTTTCATTCAATAACTTCTCAATCAATGTGA
Ca_LG_6:31979751	TCACGATCAGGCACGAGAATGACAACAACCACGATCAAGCAAGAATGAAGATCAAGGAAGCTAGCAACAATAGCGACAACCAATAACGAGCAAGAATGAC	C/G	GATCAACAAACCAGAATCAAGATCGAGCAGCAGCGAGAAACAACCCAGAAGCACAATCTCAACACTGAAAAATTGTGAAAGACAATAAGGAATCACAAAG
Ca_LG_6:31979771	GACAACAACCACGATCAAGCAAGAATGAAGATCAAGGAAGCTAGCAACAATAGCGACAACCAATAACGAGCAAGAATGACGATCAACAAACCAGAATCAA	A/C	GATCGAGCAGCAGCGAGAAACAACCCAGAAGCACAATCTCAACACTGAAAAATTGTGAAAGACAATAAGGAATCACAAAGATGAAACCAGGAGTCGACAG
Ca_LG_6:32153312	ACGATTGAATGAGGATCTCCTCATGAAAGTGACATGATGATGACGACGACGACATGTGTTTAAGTGCATGTGTCTAGGGAGTATTGTATTATTTAGTATC	C/T	TACAAATTGTGGTGATGATATTCACCATATATGCAACTATCTTTATAGTGTTCAACATAATAAGAGGAGCAATATAAATAGATTAATTGTTGAAATATTT

**Positions**	**-100 bp flanking sequences**	**SNPs within *kabuli* cultivar**	**+100 bp flanking sequences**
Ca_LG_6:32153321	TGAGGATCTCCTCATGAAAGTGACATGATGATGACGACGACGACATGTGTTTAAGTGCATGTGTCTAGGGAGTATTGTATTATTTAGTATCTACAAATTG	G/C	TGGTGATGATATTCACCATATATGCAACTATCTTTATAGTGTTCAACATAATAAGAGGAGCAATATAAATAGATTAATTGTTGAAATATTTTTCAGAATA
Ca_LG_6:32153322	GAGGATCTCCTCATGAAAGTGACATGATGATGACGACGACGACATGTGTTTAAGTGCATGTGTCTAGGGAGTATTGTATTATTTAGTATCTACAAATTGT	T/C	GGTGATGATATTCACCATATATGCAACTATCTTTATAGTGTTCAACATAATAAGAGGAGCAATATAAATAGATTAATTGTTGAAATATTTTTCAGAATAA
Ca_LG_6:32317480	AGATTTGATGTCAACAAGTTAATCATGGTGATTAGGTGAGCTGGCAGCTGCAGTACTTGTGTCAATTTCCAAAGGAGTAGCAGAACAAGATGCAGGGGAA	A/G	AGGGGAGGGGATGCAATATTATGAAAATCATCAAATACAAGACCATTGTTATTAGGGAGAGGATTTGGTTGCTGCAAAGTAGAATTAACTATAGAATAAG
Ca_LG_6:32317500	AATCATGGTGATTAGGTGAGCTGGCAGCTGCAGTACTTGTGTCAATTTCCAAAGGAGTAGCAGAACAAGATGCAGGGGAAAGGGGAGGGGATGCAATATT	T/C	ATGAAAATCATCAAATACAAGACCATTGTTATTAGGGAGAGGATTTGGTTGCTGCAAAGTAGAATTAACTATAGAATAAGGAAATATAAACTAAAAAAAA
Ca_LG_6:32317501	ATCATGGTGATTAGGTGAGCTGGCAGCTGCAGTACTTGTGTCAATTTCCAAAGGAGTAGCAGAACAAGATGCAGGGGAAAGGGGAGGGGATGCAATATTA	A/G	TGAAAATCATCAAATACAAGACCATTGTTATTAGGGAGAGGATTTGGTTGCTGCAAAGTAGAATTAACTATAGAATAAGGAAATATAAACTAAAAAAAAA
Ca_LG_6:33379159	TTGGTGAGAGTTATGATCGTTTAAATAGTTCATCAAACTGAAGAAGTGATGGATGAATGTGAGAATTGCAGTGGCGGCGGCATCTAGCGTGGAAGAAGAT	T/C	TGCATCTGGAAGAAGGTTGCGATGCAGATTTTTAATTTGTGCAGAAGTGGTGGAGCTAGCAATTATCTAGGTGCCCAAAGTAGCAGATTTAAAATACCTA
Ca_LG_6:33379166	GAGTTATGATCGTTTAAATAGTTCATCAAACTGAAGAAGTGATGGATGAATGTGAGAATTGCAGTGGCGGCGGCATCTAGCGTGGAAGAAGATTGCATCT	T/G	GGAAGAAGGTTGCGATGCAGATTTTTAATTTGTGCAGAAGTGGTGGAGCTAGCAATTATCTAGGTGCCCAAAGTAGCAGATTTAAAATACCTAAAACAGA
Ca_LG_6:33379175	TCGTTTAAATAGTTCATCAAACTGAAGAAGTGATGGATGAATGTGAGAATTGCAGTGGCGGCGGCATCTAGCGTGGAAGAAGATTGCATCTGGAAGAAGG	G/A	TTGCGATGCAGATTTTTAATTTGTGCAGAAGTGGTGGAGCTAGCAATTATCTAGGTGCCCAAAGTAGCAGATTTAAAATACCTAAAACAGAGTGGGTCAA
Ca_LG_6:33379176	CGTTTAAATAGTTCATCAAACTGAAGAAGTGATGGATGAATGTGAGAATTGCAGTGGCGGCGGCATCTAGCGTGGAAGAAGATTGCATCTGGAAGAAGGT	T/C	TGCGATGCAGATTTTTAATTTGTGCAGAAGTGGTGGAGCTAGCAATTATCTAGGTGCCCAAAGTAGCAGATTTAAAATACCTAAAACAGAGTGGGTCAAA
Ca_LG_6:33667124	TAGCTTCCATCCTTGCATTTGACTCTAATATTTTGGCGTTGTGTTCCATTTGAGCTGCCATAAATTCCATCAGCTTTGAAACAAATGCAGGAGCCTCAGC	C/T	TGCTGATTCAGATGCTTGTGGAGGAGTTGGTTCAACATTTGGCATTGGTCTAATCCAAACATTATCTACTTTCTTCAGTTTCATCTGATTCACAATCGCT
Ca_LG_6:34119146	TGTTCAAGATTCTTTTGTTCAAGTTCAATCGTTAAAGCTTCAATCTTAGCTTCCATAGTAGCAACTTTTCCTGATGATACATCAGAAACTGTAGACGATA	A/G	ATTTTATGAATGCTTTGGTAGTTGCAGCTTGAGATCCTAATCCAAATATGTGTCCTTTCTTGTTAATGCCTCCAACAACTTCCATGTATATACTATTATT
Ca_LG_6:34119147	GTTCAAGATTCTTTTGTTCAAGTTCAATCGTTAAAGCTTCAATCTTAGCTTCCATAGTAGCAACTTTTCCTGATGATACATCAGAAACTGTAGACGATAA	A/G	TTTTATGAATGCTTTGGTAGTTGCAGCTTGAGATCCTAATCCAAATATGTGTCCTTTCTTGTTAATGCCTCCAACAACTTCCATGTATATACTATTATTA

Positions	-100 bp flanking sequences	SNPs within *kabuli* cultivar	+100 bp flanking sequences
Ca_LG_6:34119159	TTTGTTCAAGTTCAATCGTTAAAGCTTCAATCTTAGCTTCCATAGTAGCAACTTTTCCTGATGATACATCAGAAACTGTAGACGATAATTTTATGAATGC	C/T	TTTGGTAGTTGCAGCTTGAGATCCTAATCCAAATATGTGTCCTTTCTTGTTAATGCCTCCAACAACTTCCATGTATATACTATTATTAAACAGTTGGCTA
Ca_LG_6:34119164	TCAAGTTCAATCGTTAAAGCTTCAATCTTAGCTTCCATAGTAGCAACTTTTCCTGATGATACATCAGAAACTGTAGACGATAATTTTATGAATGCTTTGG	G/A	TAGTTGCAGCTTGAGATCCTAATCCAAATATGTGTCCTTTCTTGTTAATGCCTCCAACAACTTCCATGTATATACTATTATTAAACAGTTGGCTACTACC
Ca_LG_6:34484889	TTCATATTTGGAGAAACTTTTCCGATTCTTGTAAATTATAATGACTCAATTATTTATCCAAAGGCATTTCCTTATTTATTTCTCTGTTTTAGTTTAATCT	T/C	CTTTTGAAAAAAAAAATATACCCTCGCTTTGAAAAACGGGGTGTTACAGTTAGCATAAGCATTTCTATTAGCATTTATGTTATAAGCAAATACACAAGTG
Ca_LG_6:35297705	GGATCTTGCTTAGGACAGAGGGGACACAACCACCCAAAAAAAAAAGTTTGGTAAAATGACCAAAATGCTCTTATTAAAATTACAAAATTACAATAATACT	T/C	TAAACCCTAAATCATATCATCTCTCTTCCTTTCTCTCCTGTGCGCTGCTCTCTCTTCCTCTCTCACTCTCCTGTTGTGTTGCGTCGCGTCGCTGTCGTGC
Ca_LG_6:35297710	TTGCTTAGGACAGAGGGGACACAACCACCCAAAAAAAAAAGTTTGGTAAAATGACCAAAATGCTCTTATTAAAATTACAAAATTACAATAATACTTAAAC	C/T	CCTAAATCATATCATCTCTCTTCCTTTCTCTCCTGTGCGCTGCTCTCTCTTCCTCTCTCACTCTCCTGTTGTGTTGCGTCGCGTCGCTGTCGTGCCTCCC
Ca_LG_6:35297711	TGCTTAGGACAGAGGGGACACAACCACCCAAAAAAAAAAGTTTGGTAAAATGACCAAAATGCTCTTATTAAAATTACAAAATTACAATAATACTTAAACC	C/T	CTAAATCATATCATCTCTCTTCCTTTCTCTCCTGTGCGCTGCTCTCTCTTCCTCTCTCACTCTCCTGTTGTGTTGCGTCGCGTCGCTGTCGTGCCTCCCT
Ca_LG_6:35297746	AAAAGTTTGGTAAAATGACCAAAATGCTCTTATTAAAATTACAAAATTACAATAATACTTAAACCCTAAATCATATCATCTCTCTTCCTTTCTCTCCTGT	T/C	GCGCTGCTCTCTCTTCCTCTCTCACTCTCCTGTTGTGTTGCGTCGCGTCGCTGTCGTGCCTCCCTCTTGCCGTCTTCACCGCTGCTACCTCGCCTCGGTC
Ca_LG_6:35297751	TTTGGTAAAATGACCAAAATGCTCTTATTAAAATTACAAAATTACAATAATACTTAAACCCTAAATCATATCATCTCTCTTCCTTTCTCTCCTGTGCGCT	T/G	GCTCTCTCTTCCTCTCTCACTCTCCTGTTGTGTTGCGTCGCGTCGCTGTCGTGCCTCCCTCTTGCCGTCTTCACCGCTGCTACCTCGCCTCGGTCGTTTG
Ca_LG_6:35297779	TAAAATTACAAAATTACAATAATACTTAAACCCTAAATCATATCATCTCTCTTCCTTTCTCTCCTGTGCGCTGCTCTCTCTTCCTCTCTCACTCTCCTGT	T/C	TGTGTTGCGTCGCGTCGCTGTCGTGCCTCCCTCTTGCCGTCTTCACCGCTGCTACCTCGCCTCGGTCGTTTGTCGTCAAAACTCCAAAGGTTTGGATTTT
Ca_LG_6:35297782	AATTACAAAATTACAATAATACTTAAACCCTAAATCATATCATCTCTCTTCCTTTCTCTCCTGTGCGCTGCTCTCTCTTCCTCTCTCACTCTCCTGTTGT	T/C	GTTGCGTCGCGTCGCTGTCGTGCCTCCCTCTTGCCGTCTTCACCGCTGCTACCTCGCCTCGGTCGTTTGTCGTCAAAACTCCAAAGGTTTGGATTTTGCT
Ca_LG_6:35297794	ACAATAATACTTAAACCCTAAATCATATCATCTCTCTTCCTTTCTCTCCTGTGCGCTGCTCTCTCTTCCTCTCTCACTCTCCTGTTGTGTTGCGTCGCGT	T/C	CGCTGTCGTGCCTCCCTCTTGCCGTCTTCACCGCTGCTACCTCGCCTCGGTCGTTTGTCGTCAAAACTCCAAAGGTTTGGATTTTGCTGATTTATTGAAC
Ca_LG_6:35791158	GTACTGTCAACCACAGAGCTGTTGAATTAAGAGTGATAAAATAACTTAAATTTTCTCGTACTTAATTCAATGGCTTTATAAGTGCTGCAGAAGATCCAGA	A/G	CACAAAAATGATAGGTTTAAATATAGGAGATAGAGAGTAAAAGTATGACAGAGAGAGAGAGAATGTAGATAGCGCAGCGAAAAATAGAATGCAAATTATC

Positions	-100 bp flanking sequences	SNPs within *kabuli* cultivar	+100 bp flanking sequences
Ca_LG_6:37224650	CGACAACAATATCCTGCGTTTTTGTTTTGACATAGCAGCAACTATCATCTCCCAACATTTTCATGCACTGCCTCCCAACATCCTACCCCTTACCAAATCA	A/G	GACTCCAAACACATTTGGTGTGGCGACTTTATCCCTCAACATAGACTTGTGATAAAATATGTCCTTAAATGAAACTGAGGCAAACCCTTGGATTTGTATC
Ca_LG_6:37224668	TTTTTGTTTTGACATAGCAGCAACTATCATCTCCCAACATTTTCATGCACTGCCTCCCAACATCCTACCCCTTACCAAATCAGACTCCAAACACATTTGG	G/T	TGTGGCGACTTTATCCCTCAACATAGACTTGTGATAAAATATGTCCTTAAATGAAACTGAGGCAAACCCTTGGATTTGTATCCGATGATTAATGGACTAC
Ca_LG_6:37224671	TTGTTTTGACATAGCAGCAACTATCATCTCCCAACATTTTCATGCACTGCCTCCCAACATCCTACCCCTTACCAAATCAGACTCCAAACACATTTGGTGT	T/C	GGCGACTTTATCCCTCAACATAGACTTGTGATAAAATATGTCCTTAAATGAAACTGAGGCAAACCCTTGGATTTGTATCCGATGATTAATGGACTACGGC
Ca_LG_6:37224694	TCATCTCCCAACATTTTCATGCACTGCCTCCCAACATCCTACCCCTTACCAAATCAGACTCCAAACACATTTGGTGTGGCGACTTTATCCCTCAACATAG	G/A	ACTTGTGATAAAATATGTCCTTAAATGAAACTGAGGCAAACCCTTGGATTTGTATCCGATGATTAATGGACTACGGCAATCCCTTCACTTTGCTATTGAG
Ca_LG_6:37224695	CATCTCCCAACATTTTCATGCACTGCCTCCCAACATCCTACCCCTTACCAAATCAGACTCCAAACACATTTGGTGTGGCGACTTTATCCCTCAACATAGA	A/T	CTTGTGATAAAATATGTCCTTAAATGAAACTGAGGCAAACCCTTGGATTTGTATCCGATGATTAATGGACTACGGCAATCCCTTCACTTTGCTATTGAGC
Ca_LG_6:37224701	CCAACATTTTCATGCACTGCCTCCCAACATCCTACCCCTTACCAAATCAGACTCCAAACACATTTGGTGTGGCGACTTTATCCCTCAACATAGACTTGTG	G/C	ATAAAATATGTCCTTAAATGAAACTGAGGCAAACCCTTGGATTTGTATCCGATGATTAATGGACTACGGCAATCCCTTCACTTTGCTATTGAGCCATGTT
Ca_LG_6:37225042	TTTTATAGCCCTCACTTCTTGACCTAATGGGCCCTTCTTTGGAAGTCCAATCCCATTAAGATAAATCTCTAAGATCCACACCACTTGTCCTACGAGTATT	T/A	ACAGCGCATGAAGCTGGCTGCCCCTTCTCCATGCAGTTTTTCCTTACAGGGGGCATGGAGCTGGAGCATGCGTAGCTCATGTTACTCGTCTGTGTAGGGA
Ca_LG_6:38186331	ATGCGATACACGTCATCCTTTTTTCCAATAGCAGCATACTTAGTTAGAAGAGATTCATATGCAGCTCTCTTTGACTTGCCTTTAATCAACTGTTCAGATT	T/C	TTTTCAACATTGCCAATGACTTGTCTAATTGGCCAGCTTTAATATATCCATCTGCTGCAACAGAATACAGGAACCAGCCCAAAATTACGCCGCGATCAAC
Ca_LG_6:38477318	TGACATCTTGGTTTATAGTAGAGATGAGGCTGCACATGAAGATCATTTGCGGAAAGTGCTGGAAGCATTAAGAGGAAATTCCTTACTGGTCAACAAAAAA	A/T	AAATGTAGTTTTGTTCAGCCAAGCCTAGAGTATTTGGGTCATGTTATCTCTGAGGCAGGAGTGGCAGCCGACCAGACAAAGGTAGAGGCTGTAAAATCTT
Ca_LG_6:38706508	ATCATTCACATTGATTGAAATGTTATTGAATGAAAGGTCTATTGCAGTTAAATTTATAAGCTTCAAAAGCTCATCTAGCTGCAGTGAACCATTCAACCTA	A/G	TTAGAGGAAAGATCCAAGAAAGAAAGTGAACTGAGCTGGAAGATAGATATTGGAAAAGGCCCTGATAGACTATTGCTACTTAAATCAAGAGTGTTCACTA
Ca_LG_6:38706514	CACATTGATTGAAATGTTATTGAATGAAAGGTCTATTGCAGTTAAATTTATAAGCTTCAAAAGCTCATCTAGCTGCAGTGAACCATTCAACCTATTAGAG	G/C	GAAAGATCCAAGAAAGAAAGTGAACTGAGCTGGAAGATAGATATTGGAAAAGGCCCTGATAGACTATTGCTACTTAAATCAAGAGTGTTCACTACAGATG
Ca_LG_6:38706523	TGAAATGTTATTGAATGAAAGGTCTATTGCAGTTAAATTTATAAGCTTCAAAAGCTCATCTAGCTGCAGTGAACCATTCAACCTATTAGAGGAAAGATCC	C/G	AAGAAAGAAAGTGAACTGAGCTGGAAGATAGATATTGGAAAAGGCCCTGATAGACTATTGCTACTTAAATCAAGAGTGTTCACTACAGATGAAGACAAAA

Positions	-100 bp flanking sequences	SNPs within *kabuli* cultivar	+100 bp flanking sequences
Ca_LG_6:38706525	AAATGTTATTGAATGAAAGGTCTATTGCAGTTAAATTTATAAGCTTCAAAAGCTCATCTAGCTGCAGTGAACCATTCAACCTATTAGAGGAAAGATCCAA	A/G	GAAAGAAAGTGAACTGAGCTGGAAGATAGATATTGGAAAAGGCCCTGATAGACTATTGCTACTTAAATCAAGAGTGTTCACTACAGATGAAGACAAAATT
Ca_LG_6:38706528	TGTTATTGAATGAAAGGTCTATTGCAGTTAAATTTATAAGCTTCAAAAGCTCATCTAGCTGCAGTGAACCATTCAACCTATTAGAGGAAAGATCCAAGAA	A/T	AGAAAGTGAACTGAGCTGGAAGATAGATATTGGAAAAGGCCCTGATAGACTATTGCTACTTAAATCAAGAGTGTTCACTACAGATGAAGACAAAATTATG
Ca_LG_6:38706557	AAATTTATAAGCTTCAAAAGCTCATCTAGCTGCAGTGAACCATTCAACCTATTAGAGGAAAGATCCAAGAAAGAAAGTGAACTGAGCTGGAAGATAGATA	A/G	TTGGAAAAGGCCCTGATAGACTATTGCTACTTAAATCAAGAGTGTTCACTACAGATGAAGACAAAATTATGAACTTGTCAAATTTACTAAACTGGTTGAA
Ca_LG_6:39323188	CCTCCAAGAAGGAACGACGCTGCAAGAGTCAACATCAACAGGGACGATCAAATGGCTGAAGCAATGAATTATATGGCTGCTTCTGTTGCTGCAAAGACTA	A/C	TGTGGGATCTGGAGAAGAGGAAAAGATAGATTTGTGCTGCTTAATCCAGGGGATTGGAAGATTCGTTGTTACAATCCTCCAAAGTTCAAGGGTGATGAGA
Ca_LG_6:39323221	ATCAACAGGGACGATCAAATGGCTGAAGCAATGAATTATATGGCTGCTTCTGTTGCTGCAAAGACTATGTGGGATCTGGAGAAGAGGAAAAGATAGATTT	T/C	GTGCTGCTTAATCCAGGGGATTGGAAGATTCGTTGTTACAATCCTCCAAAGTTCAAGGGTGATGAGAGCCCGAAAACGGACTGAAGTATGAGATTCAAGA
Ca_LG_6:39367917	CTAATGATGAAATACTCTTAAACTTTGCAATGAAGTTTGCTCGGAATTTGCTGCGGAATTTGCTGCAGAAGTTGCTGCGGACAATTGCCGCAGATAAATC	C/T	CTATGGAAAAAGATGAAGTTTGCTGCGGACGTATAGTCGTATGTAAATCCGCAGCAAATGAAAACGTTACCTGCCGTGTTACCTGCGGAACACGAAACCG
Ca_LG_6:39831795	GCAATTGCAGACGGGGTTGATATAATATCAGCCTCAATTGCATCAACATCAAATCAAACTGTCCATTTTAGAGATGCAATCTCTATTGGAGCATTCCATT	T/G	CTATGCAACGTGGAGTGCTTACAGTGTTGGCAGCAGGGAACGCATGTCCACATCCTTCCACTCTGCATAATTTTTCTCCTTGGGCTATTGTGGTGGGTGC
Ca_LG_6:39831815	TATAATATCAGCCTCAATTGCATCAACATCAAATCAAACTGTCCATTTTAGAGATGCAATCTCTATTGGAGCATTCCATTCTATGCAACGTGGAGTGCTT	T/G	ACAGTGTTGGCAGCAGGGAACGCATGTCCACATCCTTCCACTCTGCATAATTTTTCTCCTTGGGCTATTGTGGTGGGTGCTACCACATTAGACATGAGGT
Ca_LG_6:42495225	ATCTTGTGTAGTCTAATTAGACATACAAAAATACAAACAACAAAACAAAAGAAACATGTTTCTATTTTGACCACACCGGTTGATTAATTAAAAGCAGATT	T/A	GCTGGAAAGATCAACAAAATTATTAGAGATACAGATCTTCGAGAGCTTGGGCAGTTGGAGCAAGATCTTGTTTTTGGGGATGCAGCAGCCAAGGACGTTA
Ca_LG_6:42495249	ACAAAAATACAAACAACAAAACAAAAGAAACATGTTTCTATTTTGACCACACCGGTTGATTAATTAAAAGCAGATTGCTGGAAAGATCAACAAAATTATT	T/C	AGAGATACAGATCTTCGAGAGCTTGGGCAGTTGGAGCAAGATCTTGTTTTTGGGGATGCAGCAGCCAAGGACGTTATTAGTTTTTTAAGAACAAAACAGG
Ca_LG_6:42495276	AAACATGTTTCTATTTTGACCACACCGGTTGATTAATTAAAAGCAGATTGCTGGAAAGATCAACAAAATTATTAGAGATACAGATCTTCGAGAGCTTGGG	G/A	CAGTTGGAGCAAGATCTTGTTTTTGGGGATGCAGCAGCCAAGGACGTTATTAGTTTTTTAAGAACAAAACAGGTCTTGTACTGAATCCATCTGTTCGACT
Ca_LG_6:42495279	CATGTTTCTATTTTGACCACACCGGTTGATTAATTAAAAGCAGATTGCTGGAAAGATCAACAAAATTATTAGAGATACAGATCTTCGAGAGCTTGGGCAG	G/A	TTGGAGCAAGATCTTGTTTTTGGGGATGCAGCAGCCAAGGACGTTATTAGTTTTTTAAGAACAAAACAGGTCTTGTACTGAATCCATCTGTTCGACTAGA

Positions	-100 bp flanking sequences	SNPs within *kabuli* cultivar	+100 bp flanking sequences
Ca_LG_6:42594667	TCAGTTTTTACCGCTCCTAACTAGAAAAGTCGAATGCAGCCGCGAAGACATCATCTCTCATGGCGCTATACTATGATAGGCCCTTCGGAATTATGGGGTG	G/T	CCCGAGTCCTACATCGAGTAGTGCAGGATGCTCAGTGAAGTGTTTAAGTGACATGGTTGTGCCTCATGATAGCTAGTTGTTAAGGAAGGGTTTTCCCGAG
Ca_LG_6:42594670	GTTTTTACCGCTCCTAACTAGAAAAGTCGAATGCAGCCGCGAAGACATCATCTCTCATGGCGCTATACTATGATAGGCCCTTCGGAATTATGGGGTGCCC	C/T	GAGTCCTACATCGAGTAGTGCAGGATGCTCAGTGAAGTGTTTAAGTGACATGGTTGTGCCTCATGATAGCTAGTTGTTAAGGAAGGGTTTTCCCGAGTAT
Ca_LG_6:42594682	CCTAACTAGAAAAGTCGAATGCAGCCGCGAAGACATCATCTCTCATGGCGCTATACTATGATAGGCCCTTCGGAATTATGGGGTGCCCGAGTCCTACATC	C/T	GAGTAGTGCAGGATGCTCAGTGAAGTGTTTAAGTGACATGGTTGTGCCTCATGATAGCTAGTTGTTAAGGAAGGGTTTTCCCGAGTATTGCATAATGCAT
Ca_LG_6:43273560	TATGCACCCATTCCACTGTCTCCAACTTCCCCAAATGGCAAAGTATAAACCACAAGCTGCACAAGGAAAAGGAAAAGGAAAAAACAGATAAGATTAACAC	C/A	CATTGCATGACAAGGATATATGTACTTAATGCATTCATACAAAAGTTTTAAAAATAAAACAAGAGATTACATGTAAGGCAGTGTCATTAATAAGCAAACC
Ca_LG_6:43365171	CTAAATTAAAAATATCTGCAACCCAATACGTTTGACTACATATCAGTAGTCATAAAGACAATATTTAACCCTTCTCCTTGCTTTAAGAGTTGTAAACACC	C/G	AAGTTTTACAACCTTAAGATGTCATTCTCTTGCACAATGCATAAATCGAGACAAAATGCTTCTAACATTCAAAGTATCAAGTCGATTGGTTTTGAGATAC
Ca_LG_6:43365198	ACGTTTGACTACATATCAGTAGTCATAAAGACAATATTTAACCCTTCTCCTTGCTTTAAGAGTTGTAAACACCAAGTTTTACAACCTTAAGATGTCATTC	C/T	TCTTGCACAATGCATAAATCGAGACAAAATGCTTCTAACATTCAAAGTATCAAGTCGATTGGTTTTGAGATACATCAAACAACCAACTAGACTTCTAAAA
Ca_LG_6:43365199	CGTTTGACTACATATCAGTAGTCATAAAGACAATATTTAACCCTTCTCCTTGCTTTAAGAGTTGTAAACACCAAGTTTTACAACCTTAAGATGTCATTCT	T/A	CTTGCACAATGCATAAATCGAGACAAAATGCTTCTAACATTCAAAGTATCAAGTCGATTGGTTTTGAGATACATCAAACAACCAACTAGACTTCTAAAAT
Ca_LG_6:43365200	GTTTGACTACATATCAGTAGTCATAAAGACAATATTTAACCCTTCTCCTTGCTTTAAGAGTTGTAAACACCAAGTTTTACAACCTTAAGATGTCATTCTC	C/T	TTGCACAATGCATAAATCGAGACAAAATGCTTCTAACATTCAAAGTATCAAGTCGATTGGTTTTGAGATACATCAAACAACCAACTAGACTTCTAAAATA
Ca_LG_6:43365204	GACTACATATCAGTAGTCATAAAGACAATATTTAACCCTTCTCCTTGCTTTAAGAGTTGTAAACACCAAGTTTTACAACCTTAAGATGTCATTCTCTTGC	C/T	ACAATGCATAAATCGAGACAAAATGCTTCTAACATTCAAAGTATCAAGTCGATTGGTTTTGAGATACATCAAACAACCAACTAGACTTCTAAAATATGCT
Ca_LG_6:43365206	CTACATATCAGTAGTCATAAAGACAATATTTAACCCTTCTCCTTGCTTTAAGAGTTGTAAACACCAAGTTTTACAACCTTAAGATGTCATTCTCTTGCAC	C/T	AATGCATAAATCGAGACAAAATGCTTCTAACATTCAAAGTATCAAGTCGATTGGTTTTGAGATACATCAAACAACCAACTAGACTTCTAAAATATGCTAG
Ca_LG_6:44544817	CCCTCTTTTCTAGATCCTATCGAGTCTTTGCAGCAGTCTGGGCAGCAACAGAAGCAGCCATGTTATTCATAGCTTCTGCCATTTGGTCATCCCTATTGAC	C/T	ATTGACTCTTGGAGCGTCATTCCTTCTTGGCGGAATGGTTTCCTACAAAACCATAATGGAGAAGAGTCTTAACAACACTTCGAATAATTCCAAAACGAAC
Ca_LG_6:44544833	CTATCGAGTCTTTGCAGCAGTCTGGGCAGCAACAGAAGCAGCCATGTTATTCATAGCTTCTGCCATTTGGTCATCCCTATTGACATTGACTCTTGGAGCG	G/A	TCATTCCTTCTTGGCGGAATGGTTTCCTACAAAACCATAATGGAGAAGAGTCTTAACAACACTTCGAATAATTCCAAAACGAACTTCCGACTACATCAAC

Positions	-100 bp flanking sequences	SNPs within *kabuli* cultivar	+100 bp flanking sequences
Ca_LG_6:45164540	GTGGTGGCCTATGCAGCTACAAATGGACATGTCACTTCAGTCATTTGTGACGGCTTTGCCCCTCACAAATGTTATTTTTTGTGGCTGTAAAAGCCCTCAT	T/C	AAAATTCTTAAAAATTTAACTGGATTTTGTGGCTGTCTAAGCCCTCACAAAATCACAACGTTGTGATTTTGTGTGGGCTTAGGCAGCCACAAAATCCAGT
Ca_LG_6:45764381	ACCCGACAAAAATCGACTGAAATCCGTAGGCCAGGCTCAGACCTTTCAAAGTCTGACCTATTACCACCCCTAATTGCACATCACACCATAGAGCGACATG	G/T	TGGTAAGCTCGTTTTTAAGGCCAAACCATAAGCAGCACCTCTCGAATCAGGAAATATCCTCGGAAAACCACCATGTGCTATGACCAACGGCAGATTACCT
Ca_LG_6:45764382	CCCGACAAAAATCGACTGAAATCCGTAGGCCAGGCTCAGACCTTTCAAAGTCTGACCTATTACCACCCCTAATTGCACATCACACCATAGAGCGACATGT	T/C	GGTAAGCTCGTTTTTAAGGCCAAACCATAAGCAGCACCTCTCGAATCAGGAAATATCCTCGGAAAACCACCATGTGCTATGACCAACGGCAGATTACCTG
Ca_LG_6:45764396	ACTGAAATCCGTAGGCCAGGCTCAGACCTTTCAAAGTCTGACCTATTACCACCCCTAATTGCACATCACACCATAGAGCGACATGTGGTAAGCTCGTTTT	T/C	TAAGGCCAAACCATAAGCAGCACCTCTCGAATCAGGAAATATCCTCGGAAAACCACCATGTGCTATGACCAACGGCAGATTACCTGCAAAACAATCAACA
Ca_LG_6:45905256	CCTCCCAAGGGCAGGTTCTTACGCGTTACTCACCCGTCCGCCACTGGAAACACCACTTCCCGTCCGACTTGCATGTGTAAAGCATGCCGCCAGCGTTCAT	T/A	CCTGAGCCAGGATCAAACTCTCCATGAGATTCCTAGTTGCATTACTTATAGCTTCCTTGCTCGTAGACAAAGCTGATTCGGAATTTTATTTCATTTCAAG
Ca_LG_6:45905257	CTCCCAAGGGCAGGTTCTTACGCGTTACTCACCCGTCCGCCACTGGAAACACCACTTCCCGTCCGACTTGCATGTGTAAAGCATGCCGCCAGCGTTCATC	C/T	CTGAGCCAGGATCAAACTCTCCATGAGATTCCTAGTTGCATTACTTATAGCTTCCTTGCTCGTAGACAAAGCTGATTCGGAATTTTATTTCATTTCAAGG
Ca_LG_6:46091122	GAAGATGGGTGACAGAGATCAAAGACTCTATACAGAAAATTAGGCTCTGGCTCGGTACATTTGATACAGCTGAAGATGCAGCTCGTGCTTATGACACTGC	C/T	TGCATGAGCTTTAAGAGGTGCTAATGCATGAACAAACTTTGATTTGCCACAAGCTGCAACCAGTGGTGTTGGTGGTGGTGCCAAACGTGGTGCTAGTTCA
Ca_LG_6:46544744	TGTATGCAATATTTTTGTTCACTACATTATTTTACTGTTCTGATTATTTTCCACCCAATAAAAAGTATCTCTCTACCAACACAAGGCATTGAAGTACTTC	C/T	AAACCTGTTGCGGGAATGGGAATTCATTTATACGTAGCACATTAATTAATTTATTGATACAAAAACAAAAAAAAAAGACAACATGCAGCATATTGCTAGT
Ca_LG_6:46647588	CAGTCAAACTAAATTGATCACCATGAGTTGTAGCTTGATTGTTTGAATCTTGATCATCATGAGTTGCAGCTTGATTGTTTAAAGCATGATCATCATGAGT	T/C	TGCAGCTTGATTGTTTGAAGCTTGATCATTATTTGCAGCAGCTTGATGATCATGAGTTGAAGATTGATCATCATGAATTGCAGTTTGATCATGAGTTGCA
Ca_LG_6:46647651	TTGCAGCTTGATTGTTTAAAGCATGATCATCATGAGTTGCAGCTTGATTGTTTGAAGCTTGATCATTATTTGCAGCAGCTTGATGATCATGAGTTGAAGA	A/C	TTGATCATCATGAATTGCAGTTTGATCATGAGTTGCAGCTTGATCATCATGAATAGTTGGCAAAAATCCAGATGTTGGTGAAGGGCAATTAGTATGTCCT
Ca_LG_6:46647662	TTGTTTAAAGCATGATCATCATGAGTTGCAGCTTGATTGTTTGAAGCTTGATCATTATTTGCAGCAGCTTGATGATCATGAGTTGAAGATTGATCATCAT	T/C	GAATTGCAGTTTGATCATGAGTTGCAGCTTGATCATCATGAATAGTTGGCAAAAATCCAGATGTTGGTGAAGGGCAATTAGTATGTCCTTCTACAAATAG
Ca_LG_6:46647672	CATGATCATCATGAGTTGCAGCTTGATTGTTTGAAGCTTGATCATTATTTGCAGCAGCTTGATGATCATGAGTTGAAGATTGATCATCATGAATTGCAGT	T/C	TTGATCATGAGTTGCAGCTTGATCATCATGAATAGTTGGCAAAAATCCAGATGTTGGTGAAGGGCAATTAGTATGTCCTTCTACAAATAGTTTGGAATCT

Positions	-100 bp flanking sequences	SNPs within *kabuli* cultivar	+100 bp flanking sequences
Ca_LG_6:46647704	GAAGCTTGATCATTATTTGCAGCAGCTTGATGATCATGAGTTGAAGATTGATCATCATGAATTGCAGTTTGATCATGAGTTGCAGCTTGATCATCATGAA	A/G	TAGTTGGCAAAAATCCAGATGTTGGTGAAGGGCAATTAGTATGTCCTTCTACAAATAGTTTGGAATCTTCTCCAGGGATAACTTTTTCTGAATTTGAATT
Ca_LG_6:46647714	CATTATTTGCAGCAGCTTGATGATCATGAGTTGAAGATTGATCATCATGAATTGCAGTTTGATCATGAGTTGCAGCTTGATCATCATGAATAGTTGGCAA	A/T	AAATCCAGATGTTGGTGAAGGGCAATTAGTATGTCCTTCTACAAATAGTTTGGAATCTTCTCCAGGGATAACTTTTTCTGAATTTGAATTACCACCAAAC
Ca_LG_6:46979685	AAGAAATATGGAGAAGTCGGTAACTGATTTTCTGGAATCGGGGCATGCTGCTCGGTCACTATCACTAAAAGTTGAAATGTTGAAGAAGAAGAGAAGAATG	G/A	AACCTTTTCCGGGGCAGCCCTTAATATAATGAAGAATACGAGTAGCAACATGAAAATGTGAAGAAATAGGATTTGATACAAATTGACTAAGTTTGTGAGT
Ca_LG_6:46979688	AAATATGGAGAAGTCGGTAACTGATTTTCTGGAATCGGGGCATGCTGCTCGGTCACTATCACTAAAAGTTGAAATGTTGAAGAAGAAGAGAAGAATGAAC	C/T	CTTTTCCGGGGCAGCCCTTAATATAATGAAGAATACGAGTAGCAACATGAAAATGTGAAGAAATAGGATTTGATACAAATTGACTAAGTTTGTGAGTAGA
Ca_LG_6:46979693	TGGAGAAGTCGGTAACTGATTTTCTGGAATCGGGGCATGCTGCTCGGTCACTATCACTAAAAGTTGAAATGTTGAAGAAGAAGAGAAGAATGAACCTTTT	T/C	CCGGGGCAGCCCTTAATATAATGAAGAATACGAGTAGCAACATGAAAATGTGAAGAAATAGGATTTGATACAAATTGACTAAGTTTGTGAGTAGAAAAGG
Ca_LG_6:46979694	GGAGAAGTCGGTAACTGATTTTCTGGAATCGGGGCATGCTGCTCGGTCACTATCACTAAAAGTTGAAATGTTGAAGAAGAAGAGAAGAATGAACCTTTTC	C/A	CGGGGCAGCCCTTAATATAATGAAGAATACGAGTAGCAACATGAAAATGTGAAGAAATAGGATTTGATACAAATTGACTAAGTTTGTGAGTAGAAAAGGA
Ca_LG_6:46979695	GAGAAGTCGGTAACTGATTTTCTGGAATCGGGGCATGCTGCTCGGTCACTATCACTAAAAGTTGAAATGTTGAAGAAGAAGAGAAGAATGAACCTTTTCC	C/T	GGGGCAGCCCTTAATATAATGAAGAATACGAGTAGCAACATGAAAATGTGAAGAAATAGGATTTGATACAAATTGACTAAGTTTGTGAGTAGAAAAGGAG
Ca_LG_6:46979700	GTCGGTAACTGATTTTCTGGAATCGGGGCATGCTGCTCGGTCACTATCACTAAAAGTTGAAATGTTGAAGAAGAAGAGAAGAATGAACCTTTTCCGGGGC	C/T	AGCCCTTAATATAATGAAGAATACGAGTAGCAACATGAAAATGTGAAGAAATAGGATTTGATACAAATTGACTAAGTTTGTGAGTAGAAAAGGAGAGATT
Ca_LG_6:47577936	CACAATAGCTTTCATCTTCCAATTGGAGAGATGATCATTACTCTGGACGGTGTCTCCTCGCTGCTGCATATACCCATCACCGGTGCATTCTTCAGCGTTA	A/G	GTATATTTAACAAGGATGACGCTGCAAAATTATTGGGTGAGCTTCTTGGGGTTAGTCTGGCTATTGCATATGTTGGGTTTAACTTGACGCGGACAACCAC
Ca_LG_6:47577937	ACAATAGCTTTCATCTTCCAATTGGAGAGATGATCATTACTCTGGACGGTGTCTCCTCGCTGCTGCATATACCCATCACCGGTGCATTCTTCAGCGTTAG	G/A	TATATTTAACAAGGATGACGCTGCAAAATTATTGGGTGAGCTTCTTGGGGTTAGTCTGGCTATTGCATATGTTGGGTTTAACTTGACGCGGACAACCACT
Ca_LG_6:47803203	AGAAAAAGAGATCGATAGCGTCGAAAGGCGATTACAAGGAGGATGCCCTATGTCACCAAGAGAAGCAGCCATTTTTCTCAAGGCCATGGGATACCCTTCA	A/C	ACTACAACAATCTACATTGTTGCAGGCCCTATCTATGGAAGCAATAGTATGACAGCCTTTCGCGCCGAGTATCCTAATGTCTTTACTCATTCCACCCTTG
Ca_LG_6:47803225	GAAAGGCGATTACAAGGAGGATGCCCTATGTCACCAAGAGAAGCAGCCATTTTTCTCAAGGCCATGGGATACCCTTCAACTACAACAATCTACATTGTTG	G/A	CAGGCCCTATCTATGGAAGCAATAGTATGACAGCCTTTCGCGCCGAGTATCCTAATGTCTTTACTCATTCCACCCTTGCAACTGAGGAGGAGCTAGAGCC

**Positions**	**-100 bp flanking sequences**	**SNPs within *kabuli* cultivar**	**+100 bp flanking sequences**
Ca_LG_6:48317273	CAGAGTACATTATTAGATATTAATTTTGTATGTATCATAGCAGCATACCACAATTAATCAAGTCAGAATCAGATATCAGAATTGTCAATCAGAGCAATAT	T/G	CACAAGTTAAATTTGATACCAATCTCCCCCTTTTGTCATTAAATTCAAAAAGAAAAAAAAATACAAGAGACATACTGAAACAAAATTTACAAAACAATAA
Ca_LG_6:48646958	GTGGCGGCCAAAGCAGCCACAATGAGCAACTGATATACGACGTTTGTGGCGGCCAAAACGGTCAGAAATGCAGGGATTTGTGGGGGCCTGGGCGGCCACT	T/A	ACTGGCAACGGTCCTTTGGGTGTTTGTGGCGGCCTTTGCAGCCACAAAAGGTAACAGATATAAAACTTTGTGGCGGCCTGGGCGGCCAAAAAAAGCAACG
Ca_LG_6:48647035	TTGTGGGGGCCTGGGCGGCCACTACTGGCAACGGTCCTTTGGGTGTTTGTGGCGGCCTTTGCAGCCACAAAAGGTAACAGATATAAAACTTTGTGGCGGC	C/T	CTGGGCGGCCAAAAAAAGCAACGTCACTTTATTCGTTTGTGGCGGCCTTTGCAGCCATAAAAGGTAACGACTCAGGCTTTTGTGGCGGCCTTTGCAGCCA
Ca_LG_6:48647052	GCCACTACTGGCAACGGTCCTTTGGGTGTTTGTGGCGGCCTTTGCAGCCACAAAAGGTAACAGATATAAAACTTTGTGGCGGCCTGGGCGGCCAAAAAAA	A/G	GCAACGTCACTTTATTCGTTTGTGGCGGCCTTTGCAGCCATAAAAGGTAACGACTCAGGCTTTTGTGGCGGCCTTTGCAGCCACAAAGGGTAACATCACT
Ca_LG_6:48647078	TGTTTGTGGCGGCCTTTGCAGCCACAAAAGGTAACAGATATAAAACTTTGTGGCGGCCTGGGCGGCCAAAAAAAGCAACGTCACTTTATTCGTTTGTGGC	C/T	GGCCTTTGCAGCCATAAAAGGTAACGACTCAGGCTTTTGTGGCGGCCTTTGCAGCCACAAAGGGTAACATCACTTTAGGATTTTGTGGCGGCCTTTACAC
Ca_LG_6:48647093	TTGCAGCCACAAAAGGTAACAGATATAAAACTTTGTGGCGGCCTGGGCGGCCAAAAAAAGCAACGTCACTTTATTCGTTTGTGGCGGCCTTTGCAGCCAT	T/C	AAAAGGTAACGACTCAGGCTTTTGTGGCGGCCTTTGCAGCCACAAAGGGTAACATCACTTTAGGATTTTGTGGCGGCCTTTACACTCACAAAATTTAGAT
Ca_LG_6:48711058	TCCTTCTCAACCGTTTATCCACGAAAGGTAACTTGTTGAAGCGAGGTTCTTTGTAGCGAGTTCGAGGTTTTGTGTTGGGCTGCAGAGTGGATGAAGATGC	C/T	GCAAAATCTATTTTTTGATTGCCCCATGGCTGCTGGATGTGGGGGGATGTTTTGAAGTGGTGGAGTTTTTAGTGTGCTCTTCCAATTAGTGTGTTGGGTC
Ca_LG_6:48711063	CTCAACCGTTTATCCACGAAAGGTAACTTGTTGAAGCGAGGTTCTTTGTAGCGAGTTCGAGGTTTTGTGTTGGGCTGCAGAGTGGATGAAGATGCGCAAA	A/C	ATCTATTTTTTGATTGCCCCATGGCTGCTGGATGTGGGGGGATGTTTTGAAGTGGTGGAGTTTTTAGTGTGCTCTTCCAATTAGTGTGTTGGGTCATGCA
Ca_LG_6:48711123	GGTTTTGTGTTGGGCTGCAGAGTGGATGAAGATGCGCAAAATCTATTTTTTGATTGCCCCATGGCTGCTGGATGTGGGGGGATGTTTTGAAGTGGTGGAG	G/A	TTTTTAGTGTGCTCTTCCAATTAGTGTGTTGGGTCATGCAATTCAATGTAGTGAATTAGGGAGGCAGCGAAGGAATAGCCTACAAGTCTTGAATCTTATT
Ca_LG_6:49097971	CTTGAAGTCAAGGAAATTAAAATTGGAGAACTGAGGTTGGTGGTTTCTGTGGTGAAAATTGTTATGAACAGTATCAATAGCAGTAGAATTAATTAATTAT	T/G	CTACTCAATGGTCAAAATGTACTGTAGAAGAGAATGGCAGCCTTGGACTTATTGATGAGCTACTTCATACTCAAGTGCAGCCAGAGTAGAGACAGTGACC
Ca_LG_6:49280873	GGTTCATATTTTAGAATGATAGTAAGATTTGTAAAAAAAAAACAACGGTCATACTTTCAGCAGTAACAGTCATCAGTGTGTGCCACGTCTTTCATAACAC	C/T	TATCATCAACCTCTAACTGGTCAAACTTTTAAAATGATAGTAAGATTTGTAAACAAAAACAACAATCATACTTTCAGCAGCAACAGTTATCAGTGTGTGC
Ca_LG_6:49280875	TTCATATTTTAGAATGATAGTAAGATTTGTAAAAAAAAAACAACGGTCATACTTTCAGCAGTAACAGTCATCAGTGTGTGCCACGTCTTTCATAACACTA	A/G	TCATCAACCTCTAACTGGTCAAACTTTTAAAATGATAGTAAGATTTGTAAACAAAAACAACAATCATACTTTCAGCAGCAACAGTTATCAGTGTGTGCCA

Positions	-100 bp flanking sequences	SNPs within *kabuli* cultivar	+100 bp flanking sequences
Ca_LG_6:49280878	ATATTTTAGAATGATAGTAAGATTTGTAAAAAAAAAACAACGGTCATACTTTCAGCAGTAACAGTCATCAGTGTGTGCCACGTCTTTCATAACACTATCA	A/C	TCAACCTCTAACTGGTCAAACTTTTAAAATGATAGTAAGATTTGTAAACAAAAACAACAATCATACTTTCAGCAGCAACAGTTATCAGTGTGTGCCACCT
Ca_LG_6:50237614	TTTTGTTCAAACTATTTGCAGCCAAATTCTTTTCATGTTTTTGTCCAAATTATATGCAGCCAAAGTATAGATAACAAGTTATTTGCAGCCAAATAATTTG	G/T	GACAAATTAGTGTTTTTGTCCAAATTATTTGCAGCCAAATTATTTGGAGAAATTATTGCAGCCAAAGTATAGATAACAAGTTATTGCAGCCAAATTATTT
Ca_LG_6:50237632	CAGCCAAATTCTTTTCATGTTTTTGTCCAAATTATATGCAGCCAAAGTATAGATAACAAGTTATTTGCAGCCAAATAATTTGGACAAATTAGTGTTTTTG	G/A	TCCAAATTATTTGCAGCCAAATTATTTGGAGAAATTATTGCAGCCAAAGTATAGATAACAAGTTATTGCAGCCAAATTATTTGGACAAATGAGTGTTTTT
Ca_LG_6:50237663	TTATATGCAGCCAAAGTATAGATAACAAGTTATTTGCAGCCAAATAATTTGGACAAATTAGTGTTTTTGTCCAAATTATTTGCAGCCAAATTATTTGGAG	G/C	AAATTATTGCAGCCAAAGTATAGATAACAAGTTATTGCAGCCAAATTATTTGGACAAATGAGTGTTTTTGTCCAAATTATTTGCAGTCAAATTCTTTTCA
Ca_LG_6:50237676	AAGTATAGATAACAAGTTATTTGCAGCCAAATAATTTGGACAAATTAGTGTTTTTGTCCAAATTATTTGCAGCCAAATTATTTGGAGAAATTATTGCAGC	C/T	CAAAGTATAGATAACAAGTTATTGCAGCCAAATTATTTGGACAAATGAGTGTTTTTGTCCAAATTATTTGCAGTCAAATTCTTTTCATATTTTTGTCCAA
Ca_LG_6:50240576	TCCGTGACGGTTAAAAAACTCTTGTGACCAAAGAGAGTAATCATTTGCTGCTTTAAAATCAGCCAACTTATCTTGTTCAGCTATGATTTCAATGTCTAAC	C/G	ACTCTTGCCATGTCAGCAGCAGCTTGTTTGGCGTTTCCTTCAACGATTGCAGTGTAACGACCTGTTTCAGGCATTTTCATTCTCCAATAGTAAGTTAACA
Ca_LG_6:50356695	TTGTCATGTAACAATTAAAGTATTAATTACGGAAAATGGTTTTGAGATTTACCTTATTATGAGGAGCAGCACAAAAACCACAAGTAGGTGAAGAAGCCTG	G/T	TAAACACCTCCTACAATTCCATCTATCATTGTGAGGTCTCAAAACCATAGGTAAGTGAATTTGAGTCTCATGAAAAACAACAGTCAAAAGAGTCAAAGAG
Ca_LG_6:50356706	CAATTAAAGTATTAATTACGGAAAATGGTTTTGAGATTTACCTTATTATGAGGAGCAGCACAAAAACCACAAGTAGGTGAAGAAGCCTGTAAACACCTCC	C/A	TACAATTCCATCTATCATTGTGAGGTCTCAAAACCATAGGTAAGTGAATTTGAGTCTCATGAAAAACAACAGTCAAAAGAGTCAAAGAGAGAAAAACACC
Ca_LG_6:50466017	ATGTGGATGGTAATGGAGCTGCCATCAGCAAAGCAACAAGCTCAAAAAATGCTGCAGGGAGCAGCAAGGGGAGAACTGGGAGAGGAAAGGGAGCTTTCCG	G/A	AACCACCATGACCAGCAGAACGTCTGATGATAGTCACTGGTGCTGTGACCATTGTACCTCTACAAATGTCAGATCTGCCACTGTATGCCAGATTTGCAAT
Ca_LG_6:50965456	TGATACATGAGTTGGTTTCAAAATCTCAATCAGATTGAGAAACAGAAAAATCTGAAGTTCTGATCTAAATTAAGTAAATACAATTCACATGAATATATTG	G/C	ATACCTAAGACATTGCAGCAAACTCTCAGAAGCAGCAGCCTCTTGTATAGCCTCAGTTGCTGCCATTTGTGCTGCATCTCTGTGCTTCATTACTTCCTGT
Ca_LG_6:50965474	CAAAATCTCAATCAGATTGAGAAACAGAAAAATCTGAAGTTCTGATCTAAATTAAGTAAATACAATTCACATGAATATATTGATACCTAAGACATTGCAG	G/A	CAAACTCTCAGAAGCAGCAGCCTCTTGTATAGCCTCAGTTGCTGCCATTTGTGCTGCATCTCTGTGCTTCATTACTTCCTGTCAATATGTAAAAAAAATA
Ca_LG_6:51288474	CTTGGCTACCAAGGGCCTCCAGTTTTTCAAGCTTATCGTAGTCCAACAAACTATCAAAACCCATCCTAGGAGAATCAAAGTTCGCAGCCACACAATGTCA	A/G	GCCATCCTCTGTTATGGGAGTGTGAAATCGGGATTCAGAACTCATGCAACTCACGGCAGCCAAATCCACTAGATTCAGCCATGGGGAATGAAGTTCACGG

Positions	-100 bp flanking sequences	SNPs within *kabuli* cultivar	+100 bp flanking sequences
Ca_LG_6:51316854	TCGAATACAGCATCCACAACAAAAAACATGTATTTTGTGAGGGCCAAAAGCCCTCATAAAGGGGAAAAAACAGCCACAAAGGGGGCATTTGTGGCGGCCT	T/A	TAGGCAGCCACAAATAAGGTGGTCACAAACCTTTGTGGCGGCCAAAACGGCCACCAAGTGTGAAATTTACGTTGTCCTTTGTGAGGACAAATGCTGTCAC
Ca_LG_6:51422804	AATGCTGATCCAATTTGATTTGGCTGCTCCAAATCACATTCCACAATTTCAAGCTTCTCTACAGCTGGGCTAAAAACCAAAAACATTCTGTTATCCAACA	A/C	CAGCAGCATTTTTTGTCAATGAGAAAAAACTTTCTTGGCTTACCTTCACTCCCTCCACTTGTAGCAGCATCAAGCTTCAATTGTTTAACACTCTGCACAT
Ca_LG_6:51462417	ACAATAAAGCCACAATTAGTACAAAGTATTGTCAAATAGCAGCAGTATAGCCTTGTAGCATAGCAGAATTTGAACAAATTGCTATATTTTGCAATCCAAT	T/A	TTTCTAGCAGCCTGTCTGCCCACTTCCATAAAATAACATTGTGGCAATGTAGACCTTAGTGGTCATGTATATTGGATATTTGGGAGCCATAACAAGTGTG
Ca_LG_6:51914766	CAGCCAATAATTTAGCAAATCATTCTAAAATCAGCAATTTAGCCATCATTCTAATCAGACAACCAACAATTTAGCAAATCATTCTAAAATCAACAATTTA	A/G	ACAATTTAACAATTTATACATAGTTCTAAACAGACAGCCGACAATTTAGCAAATCATTCTAAAGAGACAACCAACACTTTAGCAATTTAACACTTTAGCA
Ca_LG_6:51914784	ATCATTCTAAAATCAGCAATTTAGCCATCATTCTAATCAGACAACCAACAATTTAGCAAATCATTCTAAAATCAACAATTTAACAATTTAACAATTTATA	A/C	CATAGTTCTAAACAGACAGCCGACAATTTAGCAAATCATTCTAAAGAGACAACCAACACTTTAGCAATTTAACACTTTAGCAAATCAGCAAACACCTTGT
Ca_LG_6:51946358	TCACTTTCCGCCGGTAATCGCCGCTAACCACCGTCAACCGCGCACTGCCACTGCCTTCTTTTTTTCGGTGTGTTTTTGGACATATAGCTGATGAAAACAT	T/C	GCGTGGCTAGAATGTCTTGAAAAGTTCCGCAGGCATAGGCAACTTAGCCTTAATAGTTAGTATTTGCTAGTTTAAAGCTGAAAAGATTTTGCAATCAGGC
Ca_LG_6:51946369	CGGTAATCGCCGCTAACCACCGTCAACCGCGCACTGCCACTGCCTTCTTTTTTTCGGTGTGTTTTTGGACATATAGCTGATGAAAACATGCGTGGCTAGA	A/G	ATGTCTTGAAAAGTTCCGCAGGCATAGGCAACTTAGCCTTAATAGTTAGTATTTGCTAGTTTAAAGCTGAAAAGATTTTGCAATCAGGCCTAGGCAGAAC
Ca_LG_6:52270736	GTGTGTATTCAGTAAAAAAATATATACAACATCGTCTAATGAAATGAAAGTTAGACATTCGTCTTTGACTGAATTCATTTCGAACAAAGTAGCTTCTCTG	G/A	AAAGTGGAGCATTTTGCTTGAAGATTATTCATTAATTGCATTCTTACAAAGATAATTCGGTAAAACGTGGAGTAGTGACTTCAGATAATTCTTTGTGTAT
Ca_LG_6:52270741	TATTCAGTAAAAAAATATATACAACATCGTCTAATGAAATGAAAGTTAGACATTCGTCTTTGACTGAATTCATTTCGAACAAAGTAGCTTCTCTGAAAGT	T/G	GGAGCATTTTGCTTGAAGATTATTCATTAATTGCATTCTTACAAAGATAATTCGGTAAAACGTGGAGTAGTGACTTCAGATAATTCTTTGTGTATTTTTG
Ca_LG_6:52270742	ATTCAGTAAAAAAATATATACAACATCGTCTAATGAAATGAAAGTTAGACATTCGTCTTTGACTGAATTCATTTCGAACAAAGTAGCTTCTCTGAAAGTG	G/T	GAGCATTTTGCTTGAAGATTATTCATTAATTGCATTCTTACAAAGATAATTCGGTAAAACGTGGAGTAGTGACTTCAGATAATTCTTTGTGTATTTTTGG
Ca_LG_6:52270780	TGAAAGTTAGACATTCGTCTTTGACTGAATTCATTTCGAACAAAGTAGCTTCTCTGAAAGTGGAGCATTTTGCTTGAAGATTATTCATTAATTGCATTCT	T/C	TACAAAGATAATTCGGTAAAACGTGGAGTAGTGACTTCAGATAATTCTTTGTGTATTTTTGGGTGTGGTGGCAATAAGACCATGCAACATGTATTCTTTG
Ca_LG_6:52270795	CGTCTTTGACTGAATTCATTTCGAACAAAGTAGCTTCTCTGAAAGTGGAGCATTTTGCTTGAAGATTATTCATTAATTGCATTCTTACAAAGATAATTCG	G/A	GTAAAACGTGGAGTAGTGACTTCAGATAATTCTTTGTGTATTTTTGGGTGTGGTGGCAATAAGACCATGCAACATGTATTCTTTGATTGCTTATTTTTTT

Positions	-100 bp flanking sequences	SNPs within *kabuli* cultivar	+100 bp flanking sequences
Ca_LG_6:52368625	AGATTTTTGTGAGGGCTTTTTCAGCCACAAAATACAAATTTTGTGAGGGTAAAGGCAGCCACAAATACTTGAAGTGACGTTGCAGTTTGTGGCTGTAAAG	G/T	GCCGCCACAAAAGCCTGACATGTTGCATTTTGTGGCTGCAAAGGCTGCCAGAAAGGAATAAAGAGACGTTGGCTTTTTTGGCTGTCCAGGCCGCCACAAA
Ca_LG_6:53602784	AATGTGAATTTCCTCTCAAATAGGTAAAGGAGTGGCTCGCAGACTAGGAGGCTGAAGCATTGAGATGCCAGAAGCTGCTGGTGGAGGAGGAGGAAGCTGT	T/C	ACAGACAAACACCACATGGACCACTCCTAAGCTGAATTTATTGTTGTCAATAAGCTGAATTCACGTGGAGAAGGGCATTGAGCATCGACCGAAGCTTCTT
Ca_LG_6:53602828	TAGGAGGCTGAAGCATTGAGATGCCAGAAGCTGCTGGTGGAGGAGGAGGAAGCTGTACAGACAAACACCACATGGACCACTCCTAAGCTGAATTTATTGT	T/C	TGTCAATAAGCTGAATTCACGTGGAGAAGGGCATTGAGCATCGACCGAAGCTTCTTAGCTGAGATTTCAACTCTTGGAAATACCAGGCACAGGAACATTG
Ca_LG_6:53602863	GGTGGAGGAGGAGGAAGCTGTACAGACAAACACCACATGGACCACTCCTAAGCTGAATTTATTGTTGTCAATAAGCTGAATTCACGTGGAGAAGGGCATT	T/C	GAGCATCGACCGAAGCTTCTTAGCTGAGATTTCAACTCTTGGAAATACCAGGCACAGGAACATTGTGAAGCTACATGAATTCTTTGAAAATAATTTTGAT
Ca_LG_6:53602904	CCACTCCTAAGCTGAATTTATTGTTGTCAATAAGCTGAATTCACGTGGAGAAGGGCATTGAGCATCGACCGAAGCTTCTTAGCTGAGATTTCAACTCTTG	G/A	GAAATACCAGGCACAGGAACATTGTGAAGCTACATGAATTCTTTGAAAATAATTTTGATGCGAAATTACCTGCGGAATTTGATTCAAAAAGTATTTTAGT
Ca_LG_6:53738002	ATAACCTTAGCATATAAAGAAAGTGAACTTCTGATTGACAAATTAGGGCACTGTTTAAACAATATTGGCAGCTTTCACTTTCTTTTTAATAGTTTTTTCA	A/G	TATTTGATTTGAATTTTAAACAATATTTGAAGTGTCAAAAAGGGTTGTTAAATAGCAGCTTTAGTGCTATGGCATAGCGGAATTCTAACCAATTGCTATT
Ca_LG_6:53740800	AGCCGTATCCCATGCAAAAGCAGCCAATGTACCAATTGTAGTTGCAATCAATAAATGTGATAAACCAGGTGCAAATCCTGAAAAAGTAAAACTACAGCTC	C/T	GCTTCTGAGGGCTTGCTGCTGGAGGAAATGGGTGGGGATATTCAGGTTGTTGAAGTTTCAGCGATTAAAAAGACTGGACTGGATAACCTAGAGGTAGCTG
Ca_LG_6:53824208	AAACAAGTTTCCCCCCCCCCCCCCCCCTATGTTAGTAAACATATGAACTGGGGATACTAATGAAAAAAATTTTATCAGAGCACAAACGATATGCAGCTTG	G/A	AACATAGTATGGCAATGTATATCAGAGAATAATGAATGAACTTGCTTATATATCATATAAAAACAAAACACAAAACAGCAAAAAAAACAAGACATCACAA
Ca_LG_6:54107084	AGACTTTTCATTTTTACGAAGGACAACTAGGATAGATGAACGACAATATTTATATTATAAATATGTTATCCTCATGGTGCTGCTAAGTGTAAGATCGTGG	G/A	TTGAATAGTTGGGTAGATTTGAAAACGCGTCTTGTTTTGGACCAATTAATTAACCAAACTAAATATAACAACCACACACATACCGTGCTGCTCTATAGTT
Ca_LG_6:54107089	TTTCATTTTTACGAAGGACAACTAGGATAGATGAACGACAATATTTATATTATAAATATGTTATCCTCATGGTGCTGCTAAGTGTAAGATCGTGGTTGAA	A/T	TAGTTGGGTAGATTTGAAAACGCGTCTTGTTTTGGACCAATTAATTAACCAAACTAAATATAACAACCACACACATACCGTGCTGCTCTATAGTTTCATT
Ca_LG_6:54107093	ATTTTTACGAAGGACAACTAGGATAGATGAACGACAATATTTATATTATAAATATGTTATCCTCATGGTGCTGCTAAGTGTAAGATCGTGGTTGAATAGT	T/C	TGGGTAGATTTGAAAACGCGTCTTGTTTTGGACCAATTAATTAACCAAACTAAATATAACAACCACACACATACCGTGCTGCTCTATAGTTTCATTAAAA
Ca_LG_6:54107096	TTTACGAAGGACAACTAGGATAGATGAACGACAATATTTATATTATAAATATGTTATCCTCATGGTGCTGCTAAGTGTAAGATCGTGGTTGAATAGTTGG	G/A	GTAGATTTGAAAACGCGTCTTGTTTTGGACCAATTAATTAACCAAACTAAATATAACAACCACACACATACCGTGCTGCTCTATAGTTTCATTAAAATCA

**Positions**	**-100 bp flanking sequences**	**SNPs within *kabuli* cultivar**	**+100 bp flanking sequences**
Ca_LG_6:54107103	AGGACAACTAGGATAGATGAACGACAATATTTATATTATAAATATGTTATCCTCATGGTGCTGCTAAGTGTAAGATCGTGGTTGAATAGTTGGGTAGATT	T/C	TGAAAACGCGTCTTGTTTTGGACCAATTAATTAACCAAACTAAATATAACAACCACACACATACCGTGCTGCTCTATAGTTTCATTAAAATCAAATATAG
Ca_LG_6:54107104	GGACAACTAGGATAGATGAACGACAATATTTATATTATAAATATGTTATCCTCATGGTGCTGCTAAGTGTAAGATCGTGGTTGAATAGTTGGGTAGATTT	T/A	GAAAACGCGTCTTGTTTTGGACCAATTAATTAACCAAACTAAATATAACAACCACACACATACCGTGCTGCTCTATAGTTTCATTAAAATCAAATATAGA
Ca_LG_6:54107108	AACTAGGATAGATGAACGACAATATTTATATTATAAATATGTTATCCTCATGGTGCTGCTAAGTGTAAGATCGTGGTTGAATAGTTGGGTAGATTTGAAA	A/C	ACGCGTCTTGTTTTGGACCAATTAATTAACCAAACTAAATATAACAACCACACACATACCGTGCTGCTCTATAGTTTCATTAAAATCAAATATAGATAAA
Ca_LG_6:54107127	CAATATTTATATTATAAATATGTTATCCTCATGGTGCTGCTAAGTGTAAGATCGTGGTTGAATAGTTGGGTAGATTTGAAAACGCGTCTTGTTTTGGACC	C/A	AATTAATTAACCAAACTAAATATAACAACCACACACATACCGTGCTGCTCTATAGTTTCATTAAAATCAAATATAGATAAAGAGACAAGAGTATCACACG
Ca_LG_6:54107135	ATATTATAAATATGTTATCCTCATGGTGCTGCTAAGTGTAAGATCGTGGTTGAATAGTTGGGTAGATTTGAAAACGCGTCTTGTTTTGGACCAATTAATT	T/A	AACCAAACTAAATATAACAACCACACACATACCGTGCTGCTCTATAGTTTCATTAAAATCAAATATAGATAAAGAGACAAGAGTATCACACGAATAATGT
Ca_LG_6:54124381	GGAAATCGATTGAATGAATAGCTGAGCCCCTGTGTTAAAGCCAATCGATTTCCAAATCGATTTTGTCAAAATGGCTGAGCTCATGTAATGCAGCCAATTA	A/G	ATTGCCAAAAATCGATTTTGTCAAAATGGCTGAGCTCCTGTAATGCAGCCAATCAATTGCCAAATCGATTTTGTCAAAATAGCATAGCTCTTGTAATGAA
Ca_LG_6:54238582	GGGGTAGTGGTCACTCCAAATATATATATATATATATATAAAATGACCAAAATGTAATACCTAAACCCTAAATTGTATCCTCTCTCTTCCTCTCTCACCT	T/C	GCGCACGGTGCCGCCCTCTTACCGTCTTGCGCTGCTGCTACCTCGCTTCAGCTCGCCTTGCCTTGATTTCGTCTCGCCACGCTTCAGCTCACCTTGATCG
Ca_LG_6:54238603	ATATATATATATATATATAAAATGACCAAAATGTAATACCTAAACCCTAAATTGTATCCTCTCTCTTCCTCTCTCACCTGCGCACGGTGCCGCCCTCTTA	A/G	CCGTCTTGCGCTGCTGCTACCTCGCTTCAGCTCGCCTTGCCTTGATTTCGTCTCGCCACGCTTCAGCTCACCTTGATCGTTTGTCGTCAAAACTTCAAAG
Ca_LG_6:54238606	TATATATATATATATAAAATGACCAAAATGTAATACCTAAACCCTAAATTGTATCCTCTCTCTTCCTCTCTCACCTGCGCACGGTGCCGCCCTCTTACCG	G/T	TCTTGCGCTGCTGCTACCTCGCTTCAGCTCGCCTTGCCTTGATTTCGTCTCGCCACGCTTCAGCTCACCTTGATCGTTTGTCGTCAAAACTTCAAAGGTT
Ca_LG_6:54255852	ATTTGCTATATGAAGAAAATCAAATGAGAGAAAATTTGCAATAAGCAAGCATGACATGCAATTCTTAAGGTTGTTTTCTTTTAGATTTTGAATTCCATAT	T/C	GCTGCTTCAGATATTCTATAACATTCTCCTCCTCCCTGGTCTTGAATATAAACAACATAAATGTTATATTGTTGATCTAAAATATAAGTAAATTTTAACT
Ca_LG_6:54267854	TTGTGTAACCTGATTTGAAAGGACCATTGCAGCCAATACATGCAACATCAGGACATATTTTTGAGCCACATTCAATAGGAATGTAGGATGCTGAATTGTA	A/G	GTGAGTGTCACAATCATACCAAAGATTTTCACCAGCTAGATCAATGGCTAGATTGAAGATTTGTGACATGCAACAAACCTCACTGATGATGCATTATCAT
Ca_LG_6:54267861	ACCTGATTTGAAAGGACCATTGCAGCCAATACATGCAACATCAGGACATATTTTTGAGCCACATTCAATAGGAATGTAGGATGCTGAATTGTAGTGAGTG	G/C	TCACAATCATACCAAAGATTTTCACCAGCTAGATCAATGGCTAGATTGAAGATTTGTGACATGCAACAAACCTCACTGATGATGCATTATCATGAACAGC

Positions	-100 bp flanking sequences	SNPs within *kabuli* cultivar	+100 bp flanking sequences
Ca_LG_6:54267878	CATTGCAGCCAATACATGCAACATCAGGACATATTTTTGAGCCACATTCAATAGGAATGTAGGATGCTGAATTGTAGTGAGTGTCACAATCATACCAAAG	G/A	ATTTTCACCAGCTAGATCAATGGCTAGATTGAAGATTTGTGACATGCAACAAACCTCACTGATGATGCATTATCATGAACAGCAGCATGGGTCAACCATC
Ca_LG_6:54267880	TTGCAGCCAATACATGCAACATCAGGACATATTTTTGAGCCACATTCAATAGGAATGTAGGATGCTGAATTGTAGTGAGTGTCACAATCATACCAAAGAT	T/A	TTTCACCAGCTAGATCAATGGCTAGATTGAAGATTTGTGACATGCAACAAACCTCACTGATGATGCATTATCATGAACAGCAGCATGGGTCAACCATCAA
Ca_LG_6:54267903	AGGACATATTTTTGAGCCACATTCAATAGGAATGTAGGATGCTGAATTGTAGTGAGTGTCACAATCATACCAAAGATTTTCACCAGCTAGATCAATGGCT	T/C	AGATTGAAGATTTGTGACATGCAACAAACCTCACTGATGATGCATTATCATGAACAGCAGCATGGGTCAACCATCAATTTCATCAATCTTAAATTCTCTC
Ca_LG_6:54423846	CAGTTACTCGCATGGACACTATTCGTTTCCTAATATCTGTAGTAGCTCAAAATAAGTGGAAACTTTATCAACTTGATGTAAAATCGGTATTTCTAAATGC	C/G	CTATCTTAAAGAAGAGGTCTATGTTGAGCAACCAATGAGGTACGTGATTAAAGGACATGAAGATAAAGTTTTAAGACTGAGGAGAACATTGTATGGATTA
Ca_LG_6:54423863	ACTATTCGTTTCCTAATATCTGTAGTAGCTCAAAATAAGTGGAAACTTTATCAACTTGATGTAAAATCGGTATTTCTAAATGCCTATCTTAAAGAAGAGG	G/A	TCTATGTTGAGCAACCAATGAGGTACGTGATTAAAGGACATGAAGATAAAGTTTTAAGACTGAGGAGAACATTGTATGGATTAAAGTAAGCCCCAACAAC
Ca_LG_6:54554915	ATGAACATTTAGGTCATTTTAGTCCTCCCTAAGCTTATTCTTGTTCAATTAGGGTTTTTAACCAATCTTGTGCATCCAATAAGTTCATTTGTGTCATTGG	G/C	ATGAACATTTAGGTCATTTTCGTCCTCCCTATGCTTATTCTTGTCCAATTAGGGTTTCTACCCACTCTTGTGCATCCAATAAGTTCATTTGTGTAATTGG
Ca_LG_6:54572328	ATTGGATGAACATTTAGGTCATTTTCGTCCTCCCTAGGCTTATTCTTGTCCAATTAGAATTTTTAATCAATGTTGTGAATCCAATAAGTTCATTTGTGTC	C/A	ATTGGATGAACATTTAGGTCATTTTCATCCTCCCTAGGCTTATTCTTTTCCAATAAGGGTTTTTAACCAATCTTGTGCATCCAATAGGTGTGTTTGTGTC
Ca_LG_6:54695492	ATCAACTCTGAAACTTTTTGTTCAATTTTAGTAATTAAATATAATTCGATAGTAAAATGCAATCCTTGAGTTTGCACTCGGTACGTTTCTAGGACCGACT	T/C	AACGCCTCACTTACACCGGTACCTATGTAGTGTAGTGTCGGTCGCACATTCACATAGCGTTCAGACACACGTGCCTTCCATAACCTAGGGTATTCAAGTC
Ca_LG_6:54695503	AACTTTTTGTTCAATTTTAGTAATTAAATATAATTCGATAGTAAAATGCAATCCTTGAGTTTGCACTCGGTACGTTTCTAGGACCGACTAACGCCTCACT	T/C	TACACCGGTACCTATGTAGTGTAGTGTCGGTCGCACATTCACATAGCGTTCAGACACACGTGCCTTCCATAACCTAGGGTATTCAAGTCAGCTGCATATG
Ca_LG_6:54695505	CTTTTTGTTCAATTTTAGTAATTAAATATAATTCGATAGTAAAATGCAATCCTTGAGTTTGCACTCGGTACGTTTCTAGGACCGACTAACGCCTCACTTA	A/G	CACCGGTACCTATGTAGTGTAGTGTCGGTCGCACATTCACATAGCGTTCAGACACACGTGCCTTCCATAACCTAGGGTATTCAAGTCAGCTGCATATGAT
Ca_LG_6:54695509	TTGTTCAATTTTAGTAATTAAATATAATTCGATAGTAAAATGCAATCCTTGAGTTTGCACTCGGTACGTTTCTAGGACCGACTAACGCCTCACTTACACC	C/A	GGTACCTATGTAGTGTAGTGTCGGTCGCACATTCACATAGCGTTCAGACACACGTGCCTTCCATAACCTAGGGTATTCAAGTCAGCTGCATATGATTAGG
Ca_LG_6:54695518	TTTAGTAATTAAATATAATTCGATAGTAAAATGCAATCCTTGAGTTTGCACTCGGTACGTTTCTAGGACCGACTAACGCCTCACTTACACCGGTACCTAT	T/C	GTAGTGTAGTGTCGGTCGCACATTCACATAGCGTTCAGACACACGTGCCTTCCATAACCTAGGGTATTCAAGTCAGCTGCATATGATTAGGTAGCTTTGC

**Positions**	**-100 bp flanking sequences**	**SNPs within *kabuli* cultivar**	**+100 bp flanking sequences**
Ca_LG_6:54695555	CCTTGAGTTTGCACTCGGTACGTTTCTAGGACCGACTAACGC<br>CTCACTTACACCGGTACCTATGTAGTGTAGTGTCGGTCGCAC<br>ATTCACATAGCGTTCA	A/G	GACACACGTGCCTTCCATAACCTAGGGTATTCAAGTCAGCT<br>GCATATGATTAGGTAGCTTTGCAGCTGCAAAAGGATTAGACA<br>TCTTCCCATGATATGGT
Ca_LG_6:54695559	GAGTTTGCACTCGGTACGTTTCTAGGACCGACTAACGCCTCA<br>CTTACACCGGTACCTATGTAGTGTAGTGTCGGTCGCACATTC<br>ACATAGCGTTCAGACA	A/T	CACGTGCCTTCCATAACCTAGGGTATTCAAGTCAGCTGCATA<br>TGATTAGGTAGCTTTGCAGCTGCAAAAGGATTAGACATCTTC<br>CCATGATATGGTTCCT
Ca_LG_6:54695562	TTTGCACTCGGTACGTTTCTAGGACCGACTAACGCCTCACTTA<br>CACCGGTACCTATGTAGTGTAGTGTCGGTCGCACATTCACAT<br>AGCGTTCAGACACAC	C/T	GTGCCTTCCATAACCTAGGGTATTCAAGTCAGCTGCATATGA<br>TTAGGTAGCTTTGCAGCTGCAAAAGGATTAGACATCTTCCCA<br>TGATATGGTTCCTTTT
Ca_LG_6:54695573	TACGTTTCTAGGACCGACTAACGCCTCACTTACACCGGTACCT<br>ATGTAGTGTAGTGTCGGTCGCACATTCACATAGCGTTCAGAC<br>ACACGTGCCTTCCAT	T/G	AACCTAGGGTATTCAAGTCAGCTGCATATGATTAGGTAGCTT<br>TGCAGCTGCAAAAGGATTAGACATCTTCCCATGATATGGTTC<br>CTTTTGGTCCGAACCT
Ca_LG_6:54695579	TCTAGGACCGACTAACGCCTCACTTACACCGGTACCTATGTA<br>GTGTAGTGTCGGTCGCACATTCACATAGCGTTCAGACACACG<br>TGCCTTCCATAACCTA	A/G	GGGTATTCAAGTCAGCTGCATATGATTAGGTAGCTTTGCAGC<br>TGCAAAAGGATTAGACATCTTCCCATGATATGGTTCCTTTTG<br>GTCCGAACCTGGTGCT
Ca_LG_6:54695588	GACTAACGCCTCACTTACACCGGTACCTATGTAGTGTAGTGT<br>CGGTCGCACATTCACATAGCGTTCAGACACACGTGCCTTCCA<br>TAACCTAGGGTATTCA	A/G	AGTCAGCTGCATATGATTAGGTAGCTTTGCAGCTGCAAAAG<br>GATTAGACATCTTCCCATGATATGGTTCCTTTTGGTCCGAAC<br>CTGGTGCTACATTAGGT
Ca_LG_6:54695591	TAACGCCTCACTTACACCGGTACCTATGTAGTGTAGTGTCGG<br>TCGCACATTCACATAGCGTTCAGACACACGTGCCTTCCATAAC<br>CTAGGGTATTCAAGT	T/C	CAGCTGCATATGATTAGGTAGCTTTGCAGCTGCAAAAGGATT<br>AGACATCTTCCCATGATATGGTTCCTTTTGGTCCGAACCTGG<br>TGCTACATTAGGTTAG
Ca_LG_7:190132	ACTAAAAGAATTCTGGGAAATTGGGGTGAAAACATTTGATGCA<br>TGCAAAAAAGAGTCATTCCAAATGCGTGTTGCTATCATGTGGA<br>CTATCAATGATTTT	T/C	CCGGCATATGCAAATTTGTCAGGCTGGAGCACTAAAGGTCG<br>ATACGCTTGTCCATGTTGTGGTTTAAAAACTGCGTCTCATTG<br>GTTGCGTCATAGTTGGA
Ca_LG_7:190135	AAAAGAATTCTGGGAAATTGGGGTGAAAACATTTGATGCATG<br>CAAAAAAGAGTCATTCCAAATGCGTGTTGCTATCATGTGGACT<br>ATCAATGATTTTCCG	G/A	GCATATGCAAATTTGTCAGGCTGGAGCACTAAAGGTCGATA<br>CGCTTGTCCATGTTGTGGTTTAAAAACTGCGTCTCATTGGTT<br>GCGTCATAGTTGGAAGT
Ca_LG_7:190177	CAAAAAAGAGTCATTCCAAATGCGTGTTGCTATCATGTGGACT<br>ATCAATGATTTTCCGGCATATGCAAATTTGTCAGGCTGGAGCA<br>CTAAAGGTCGATAC	C/T	GCTTGTCCATGTTGTGGTTTAAAAACTGCGTCTCATTGGTTG<br>CGTCATAGTTGGAAGTTTTGCTACGTGTGTCATCGTTGTTGG<br>TTAGAGCCTAATAGTA
Ca_LG_7:190195	AATGCGTGTTGCTATCATGTGGACTATCAATGATTTTCCGGCA<br>TATGCAAATTTGTCAGGCTGGAGCACTAAAGGTCGATACGCT<br>TGTCCATGTTGTGGT	T/A	TTAAAAACTGCGTCTCATTGGTTGCGTCATAGTTGGAAGTTT<br>TGCTACGTGTGTCATCGTTGTTGGTTAGAGCCTAATAGTAAG<br>TGGAGATACAATAGAA
Ca_LG_7:783804	GTTTCCAACACTTCCATACAAACTAAGGAGATAATGAAAAGGT<br>ACTCTATCTCGACCTAATATTCTACCCTCAACTTTTTTTAAGCA<br>CTCTTCGGCTTTG	G/T	TCAAACAATTCCATCTTAATGTACATTGCAGCCATTGTACTAA<br>ATGTGCTCCAGTTAGGCATAATGGTAGGATCCTTTGACATTT<br>GTTCAAATACTTGTT

Positions	-100 bp flanking sequences	SNPs within *kabuli* cultivar	+100 bp flanking sequences
Ca_LG_7:783812	CACTTCCATACAAACTAAGGAGATAATGAAAAGGTACTCTATCTCGACCTAATATTCTACCCTCAACTTTTTTTAAGCACTCTTCGGCTTTGTCAAACAA	A/G	TTCCATCTTAATGTACATTGCAGCCATTGTACTAAATGTGCTCCAGTTAGGCATAATGGTAGGATCCTTTGACATTTGTTCAAATACTTGTTCCATCTTT
Ca_LG_7:794077	AATCAAAAAATAAAAATAAACAGAATAATATTAAATAAAAGAGAATCAAATCTGATTTTCCTTGACTCTTCAACACTATTGATAGTTATAATTTACAACT	T/G	GCAATTTCTTGCTTTTCAGGAGTTGATTGCTGCTCTTCTTGTATGCTCATTTTTATGTCTATTCCCAGTCCATGACAGATATGAGAAACAACTTCAACCA
Ca_LG_7:794079	TCAAAAAATAAAAATAAACAGAATAATATTAAATAAAAGAGAATCAAATCTGATTTTCCTTGACTCTTCAACACTATTGATAGTTATAATTTACAACTGC	C/A	AATTTCTTGCTTTTCAGGAGTTGATTGCTGCTCTTCTTGTATGCTCATTTTTATGTCTATTCCCAGTCCATGACAGATATGAGAAACAACTTCAACCAGT
Ca_LG_7:803479	TCATCATATCCTGCAGGCTCAAAGACCTTTCATAGCATACTACACATTTGGCTTGGACGCATTGCACAACCTAGACAAGGTAACATGACATTTCCTTCAC	C/T	ACAGTGGGAGAATCTAAACATGTGATCAATGGCATTGCTGATAAGCATCTGCATCGAAAAGAGTTATATATTTTTCTTATTTCACGTTTGAATATTGATC
Ca_LG_7:1022258	ACAGTTGCATAAAATTAAAACAAAAAGCAAGCATGCTATAGTTTATCAGCTGCAATTCAAGTTTACTTTATGTGTATCCACGAATCTGGAACAAATTATG	G/C	TTTGACTTACATCGGAGTGCGAGTGAAAGGGTAAAAAGTGGCTGCGGAAAAAAGGATGAGTCTGAGGTTGTAGAACCAAGAGCTGCAGATCCCTGAAACA
Ca_LG_7:1103940	GTCTTCAAAACCAAACCTTGCAGCATGAATGGTTTCAAAAGCTTTCAGATAGTCCCCAACCTCAAGTGATTGAAGATGCATAGAGGCTTTCAAAAAAGTG	G/C	TCAACAGAGGCTGCTCTAAGTCCCAACTCGGTGAGCCTCTCAAAGTACAATATTTTCCATGTCTGCAAATGAATGAGCACATAAAACACATGAAGAGAAA
Ca_LG_7:1395487	TTACCTGTGCTTTGCCATTTGCAGGTTGCCTGCGGATAACATGCGGCCTCTCGTTAGGTGTTCCGGGTGCATATCCGGCCCCGCCTTGTTTCTCTTCTCT	T/G	CACTTTGTTGAAAATGTGAGTATAGCTGTCAGCTGATGCAGGGTTACTCACATCCCACTCCCCGAACTTTGGAACAGCAGCACCTTTATCAGGCTGATGT
Ca_LG_7:1395514	GCCTGCGGATAACATGCGGCCTCTCGTTAGGTGTTCCGGGTGCATATCCGGCCCCGCCTTGTTTCTCTTCTCTCACTTTGTTGAAAATGTGAGTATAGCT	T/C	GTCAGCTGATGCAGGGTTACTCACATCCCACTCCCCGAACTTTGGAACAGCAGCACCTTTATCAGGCTGATGTATCAGAATTCTAAAGTCAGAACATATT
Ca_LG_7:1395515	CCTGCGGATAACATGCGGCCTCTCGTTAGGTGTTCCGGGTGCATATCCGGCCCCGCCTTGTTTCTCTTCTCTCACTTTGTTGAAAATGTGAGTATAGCTG	G/A	TCAGCTGATGCAGGGTTACTCACATCCCACTCCCCGAACTTTGGAACAGCAGCACCTTTATCAGGCTGATGTATCAGAATTCTAAAGTCAGAACATATTT
Ca_LG_7:1395526	CATGCGGCCTCTCGTTAGGTGTTCCGGGTGCATATCCGGCCCCGCCTTGTTTCTCTTCTCTCACTTTGTTGAAAATGTGAGTATAGCTGTCAGCTGATGC	C/T	AGGGTTACTCACATCCCACTCCCCGAACTTTGGAACAGCAGCACCTTTATCAGGCTGATGTATCAGAATTCTAAAGTCAGAACATATTTGCAACAGAAAA
Ca_LG_7:1395532	GCCTCTCGTTAGGTGTTCCGGGTGCATATCCGGCCCCGCCTTGTTTCTCTTCTCTCACTTTGTTGAAAATGTGAGTATAGCTGTCAGCTGATGCAGGGTT	T/C	ACTCACATCCCACTCCCCGAACTTTGGAACAGCAGCACCTTTATCAGGCTGATGTATCAGAATTCTAAAGTCAGAACATATTTGCAACAGAAAATCTCAA
Ca_LG_7:1395537	TCGTTAGGTGTTCCGGGTGCATATCCGGCCCCGCCTTGTTTCTCTTCTCTCACTTTGTTGAAAATGTGAGTATAGCTGTCAGCTGATGCAGGGTTACTCA	A/T	CATCCCACTCCCCGAACTTTGGAACAGCAGCACCTTTATCAGGCTGATGTATCAGAATTCTAAAGTCAGAACATATTTGCAACAGAAAATCTCAAAATGA

**Positions**	**-100 bp flanking sequences**	**SNPs within *kabuli* cultivar**	**+100 bp flanking sequences**
Ca_LG_7:1395548	TCCGGGTGCATATCCGGCCCCGCCTTGTTTCTCTTCTCTCACTTTGTTGAAAATGTGAGTATAGCTGTCAGCTGATGCAGGGTTACTCACATCCCACTCC	C/A	CCGAACTTTGGAACAGCAGCACCTTTATCAGGCTGATGTATCAGAATTCTAAAGTCAGAACATATTTGCAACAGAAAATCTCAAAATGATTCCAAGCTAC
Ca_LG_7:1395551	GGGTGCATATCCGGCCCCGCCTTGTTTCTCTTCTCTCACTTTGTTGAAAATGTGAGTATAGCTGTCAGCTGATGCAGGGTTACTCACATCCCACTCCCCG	G/A	AACTTTGGAACAGCAGCACCTTTATCAGGCTGATGTATCAGAATTCTAAAGTCAGAACATATTTGCAACAGAAAATCTCAAAATGATTCCAAGCTACAAT
Ca_LG_7:1395554	TGCATATCCGGCCCCGCCTTGTTTCTCTTCTCTCACTTTGTTGAAAATGTGAGTATAGCTGTCAGCTGATGCAGGGTTACTCACATCCCACTCCCCGAAC	C/T	TTTGGAACAGCAGCACCTTTATCAGGCTGATGTATCAGAATTCTAAAGTCAGAACATATTTGCAACAGAAAATCTCAAAATGATTCCAAGCTACAATGAT
Ca_LG_7:1395557	ATATCCGGCCCCGCCTTGTTTCTCTTCTCTCACTTTGTTGAAAATGTGAGTATAGCTGTCAGCTGATGCAGGGTTACTCACATCCCACTCCCCGAACTTT	T/C	GGAACAGCAGCACCTTTATCAGGCTGATGTATCAGAATTCTAAAGTCAGAACATATTTGCAACAGAAAATCTCAAAATGATTCCAAGCTACAATGATATG
Ca_LG_7:1757610	ATGCACAACTTTTTCATTTTCATCAATGTGTTTCATTACACATTTTCTCATGCGCTTAATAGTAGGCAAAAACTCAATGGCTGCCCAAGTGAGGTGGTAG	G/C	TCGTTATCCTCCTGTGTGTGTCGATCATTCCACCAGAACACATTCGACTTTGCATTTGTGGTCACCTTCCAAACATCCATGAAACTAGTCGATGTCCAAC
Ca_LG_7:1757615	CAACTTTTTCATTTTCATCAATGTGTTTCATTACACATTTTCTCATGCGCTTAATAGTAGGCAAAAACTCAATGGCTGCCCAAGTGAGGTGGTAGTCGTT	T/C	ATCCTCCTGTGTGTGTCGATCATTCCACCAGAACACATTCGACTTTGCATTTGTGGTCACCTTCCAAACATCCATGAAACTAGTCGATGTCCAACTCCAA
Ca_LG_7:1757622	TTCATTTTCATCAATGTGTTTCATTACACATTTTCTCATGCGCTTAATAGTAGGCAAAAACTCAATGGCTGCCCAAGTGAGGTGGTAGTCGTTATCCTCC	C/T	TGTGTGTGTCGATCATTCCACCAGAACACATTCGACTTTGCATTTGTGGTCACCTTCCAAACATCCATGAAACTAGTCGATGTCCAACTCCAACGACTAC
Ca_LG_7:1757631	ATCAATGTGTTTCATTACACATTTTCTCATGCGCTTAATAGTAGGCAAAAACTCAATGGCTGCCCAAGTGAGGTGGTAGTCGTTATCCTCCTGTGTGTGT	T/C	CGATCATTCCACCAGAACACATTCGACTTTGCATTTGTGGTCACCTTCCAAACATCCATGAAACTAGTCGATGTCCAACTCCAACGACTACTCGTCGATA
Ca_LG_7:2021560	CTATGGCGATTACAACAATTAATGATATCAATTTAGGTACAAAATTTGATACAACCCCGACACCAATGAGGACAGTAACTCAGCAAAAGACAGTATCATG	G/C	CTGATAAAGTTCAAGGGATGTTGATGTTCCCTTCCCTACTCCAGTAGTCAGGAATTGTTGCCAGCTGCATTTTTAGTTGAGCCTTGAATAGTCTCCAAAA
Ca_LG_7:2021569	TTACAACAATTAATGATATCAATTTAGGTACAAAATTTGATACAACCCCGACACCAATGAGGACAGTAACTCAGCAAAAGACAGTATCATGCTGATAAAG	G/A	TTCAAGGGATGTTGATGTTCCCTTCCCTACTCCAGTAGTCAGGAATTGTTGCCAGCTGCATTTTTAGTTGAGCCTTGAATAGTCTCCAAAAGATGTTCAA
Ca_LG_7:2282369	TTGTTGTAAACAAATTAGATACGTGATTTATTGCTTAAGTGCATTTATGCAGCTATTAATTTTGTACATTTTACTCTGTTGTTCAATCTGTTATCAAACC	C/T	TGGCATTCAAGTTTAGTTTGTTTATCACTTGCCCATGCTGCTCCATGCCGGCAGACATTAGATGTTAAAAGTTCTAAGAATCTGAGCCTCTGTATGAAGT
Ca_LG_7:2282391	GTGATTTATTGCTTAAGTGCATTTATGCAGCTATTAATTTTGTACATTTTACTCTGTTGTTCAATCTGTTATCAAACCTGGCATTCAAGTTTAGTTTGTT	T/G	TATCACTTGCCCATGCTGCTCCATGCCGGCAGACATTAGATGTTAAAAGTTCTAAGAATCTGAGCCTCTGTATGAAGTAAATTATGTCAATTACTGACAC

**Positions**	**-100 bp flanking sequences**	**SNPs within *kabuli* cultivar**	**+100 bp flanking sequences**
Ca_LG_7:2286209	CAGGAACAAAATGTGCCAAGTGTATGAGTTAACAGTATAAGAACAAGGTAGCAGGGCTACTAGTATGCAGCAAAAAGTCCAAAAATATTACCTCTGGTGT	T/C	ATCTTTTTTGGAAGCAGTTTTCTTTTTCCTTCCAATTTCAACACCGTAGTGTTGAAGATACCACTGCTTGAAAGGAGCAGCATCAACCTGCACGATAGCA
Ca_LG_7:2320673	TTTTTTTAATTGAAACTGGATGCAGATGAAGGGAATGACTGTGGCAATGGTAGGTGATGGAATAAACGACTCTCCGGCTTTGGTTGCTGCTGATGTTGGC	C/A	ATGGCAATTGGTGCTGGAACTGACGTAGCTATAGAAGCAGCTGATATAGTTCTTGTCAAAAGCAACTTGGAAGATGTAATTACAGCCATAGATCTATCTA
Ca_LG_7:2320694	GCAGATGAAGGGAATGACTGTGGCAATGGTAGGTGATGGAATAAACGACTCTCCGGCTTTGGTTGCTGCTGATGTTGGCATGGCAATTGGTGCTGGAACT	T/A	GACGTAGCTATAGAAGCAGCTGATATAGTTCTTGTCAAAAGCAACTTGGAAGATGTAATTACAGCCATAGATCTATCTAGAAAGACCATGTCTCGTATCA
Ca_LG_7:2320698	ATGAAGGGAATGACTGTGGCAATGGTAGGTGATGGAATAAACGACTCTCCGGCTTTGGTTGCTGCTGATGTTGGCATGGCAATTGGTGCTGGAACTGACG	G/A	TAGCTATAGAAGCAGCTGATATAGTTCTTGTCAAAAGCAACTTGGAAGATGTAATTACAGCCATAGATCTATCTAGAAAGACCATGTCTCGTATCAGGCT
Ca_LG_7:2320703	GGGAATGACTGTGGCAATGGTAGGTGATGGAATAAACGACTCTCCGGCTTTGGTTGCTGCTGATGTTGGCATGGCAATTGGTGCTGGAACTGACGTAGCT	T/A	ATAGAAGCAGCTGATATAGTTCTTGTCAAAAGCAACTTGGAAGATGTAATTACAGCCATAGATCTATCTAGAAAGACCATGTCTCGTATCAGGCTGAACT
Ca_LG_7:2320719	ATGGTAGGTGATGGAATAAACGACTCTCCGGCTTTGGTTGCTGCTGATGTTGGCATGGCAATTGGTGCTGGAACTGACGTAGCTATAGAAGCAGCTGATA	A/G	TAGTTCTTGTCAAAAGCAACTTGGAAGATGTAATTACAGCCATAGATCTATCTAGAAAGACCATGTCTCGTATCAGGCTGAACTACATTTGGGCTCTTGG
Ca_LG_7:2320742	CTCTCCGGCTTTGGTTGCTGCTGATGTTGGCATGGCAATTGGTGCTGGAACTGACGTAGCTATAGAAGCAGCTGATATAGTTCTTGTCAAAAGCAACTTG	G/A	GAAGATGTAATTACAGCCATAGATCTATCTAGAAAGACCATGTCTCGTATCAGGCTGAACTACATTTGGGCTCTTGGCTACAACATTTTGGGCATGCCAG
Ca_LG_7:2320880	CATGTCTCGTATCAGGCTGAACTACATTTGGGCTCTTGGCTACAACATTTTGGGCATGCCAGTTGCTGCTGGTGTTTTGTACCCATTTACTGGAATCAGA	A/G	TTGCCACCATGGCTTGCTGGTGCTTGCATGGCTGCATCTTCCCTTAGTGTGGTTTCTTCGTCTCTTTTGCTGCAGTTCTATAAAAAACCTTTGCATGTAG
Ca_LG_7:2320886	TCGTATCAGGCTGAACTACATTTGGGCTCTTGGCTACAACATTTTGGGCATGCCAGTTGCTGCTGGTGTTTTGTACCCATTTACTGGAATCAGATTGCCA	A/T	CCATGGCTTGCTGGTGCTTGCATGGCTGCATCTTCCCTTAGTGTGGTTTCTTCGTCTCTTTTGCTGCAGTTCTATAAAAAACCTTTGCATGTAGAATCCA
Ca_LG_7:2364063	ATGCAATTTGAAGTTAATGTAATCAAAATGGATCGGCATTGTAATATCCAAACGTTGCCACCTAGTCAACAGAGGTTCTGAAGGCTGCAACTCCTTGACG	G/A	TACTGCTTACTCTTCTTCTACAATGAAAAGTGGATTATTGATGATATTAAATGCTTCATGCTGCCTTTCAACTCTAAATCACATCTTTAAACTAAGTGCC
Ca_LG_7:2561310	ATTGGGAGACGTACATATTATGAAGGACATGGGATATAGAGAAATAGACAGTATGAAACTTAACTCCAAATATAGAGAAATAGATAGTATGAAACTTAAC	C/T	TCCAAATATTCTCTAAATAATCTCTTGCATTCTTTCATGATATAATAATCTCAGCTAAGTTGTCATGTTCCCCTAATTAATTGATGCACTTGAATGGATT
Ca_LG_7:2685767	GTAATTTGATGTGCAGCCTCTTCCAACTAAACTTGTTCTAAGAAAAAAGAGGGCGAAAGAGGGAAGATCTGGTGATGAGGTTGAGCAATTTCCAATTCCG	G/T	GCTAGAGTGACTGTGAGGAGAAGGTCAAGTGTTGCTGCAATTGAGCGGAAGGATTCTGAGGTATGGCAACCACGTTATCTTACAAGCTTTCAATTTTATT

Positions	-100 bp flanking sequences	SNPs within *kabuli* cultivar	+100 bp flanking sequences
Ca_LG_7:3052903	TACCAAATAAAATCTTGTATGAAATAATTCAATGACTTGGACTTGATTAACAGTTCATGTAGGTGATAAGCTCTGCATTGCAGTAGGTTATGAAGTTCTG	G/T	CATTTTAGTAGTAGTCTTATGGGGTGTGTGTTGCTGCTACATTATGGGCATAAACACTGTTTCAAAGAGAAGTGTTTTACTTTTCTAGCATAGGATAAAG
Ca_LG_7:3168420	TCGCCGTCAAGTTCAACACCATACTTAAGCCACTGCCAATTCTGAACCCTGCTAATCTCGGCCGTTGCAGCATCCTCCATGAGATTGTAAAGAGGCACAG	G/A	AACCAGCTCCAGTGAGCCATGCTGCCACATACTGAATTCCAACCCGTGTATTCAACCGTAGACCTTCCATCGTTCGGACTCCTCTAGGTATCTGCAAGAG
Ca_LG_7:5196489	TACAAAGGATTAAGGTAGTTCTTTACTGGAAAGGCCTGAACAAGGATGTCAAAGCCTTCATACAACATTGCCCAACTTGTCAGAAATGCAAGTATGACAT	T/C	TGCTGCCTACCCAGGTTTATTACAACCTTTAACAATCCCTGATCATGTGTGGCAGCACATCACAATGGATTTCATTGAGGGATTGCCTATTTCTTATGGG
Ca_LG_7:5320476	TGCCTTCTCCATCTCTGGCGTCAACTGCATGTACCCAATAAGAGAATATCAATATACTGCAGGACAATTTCTACGAAGTAAATGCCAATTCAGAAATAAT	T/C	TATTACCGCAGGGGGCCTAAGAGGCTGATTAGCAGCACCCACCAGAACAGAAACCACAGATTGTGGTCCCAGAGGACCAGAATGCTGTACTAGATTTTCT
Ca_LG_7:6045997	CTTCAATAAAACCCATAATTTTGAAATAGTTTTTAATAAAGTATTTATAACTCAATAAATAAACATAAGACTTCTTGGTCAAGCCAAAAAGATTGCCGGG	G/C	AGAATGCTCCTACCTATATAAATTTGTATTGAACAATACTATAACAATAGTATAAGAAAAAAAAATACTAATGTTAAGAAAAAATAATACTAATATCGTA
Ca_LG_7:6639740	GAGGTGAGTGCGGTGGAAGAAGCGGCACCGTGAACGGCTGCCAGACGCGCCGTCACGCGCGGTGGCTATTGGCGGCGCGTGGATCTCACGCGCGTGAGGG	G/T	GGTTGCTGATGACGTAGCTGAAGGGTTTGAGTGATCTATGGTGTACGGCGCTGCTGGAGTGGTCGTTCGTTGAAAAAAATCATCAGAAACGGTGACGACA
Ca_LG_7:6815226	TGACATACCTAAAACATGTATGTGTACAGTGCTGCTATGATAAACCAAACAAAGAAGGTGGCTGCTAGCTACAACATGAAAGTAAAGAAAGTATATCAAT	T/C	AGAGGAAATAAAAACAATAAAAATGTAATCAATAAATAATAAATGATTTAATTAGCAAGAAAGTAGTAGAAAAAATAGAGAAAGAGTTGAAATTGATTAG
Ca_LG_7:6815228	ACATACCTAAAACATGTATGTGTACAGTGCTGCTATGATAAACCAAACAAAGAAGGTGGCTGCTAGCTACAACATGAAAGTAAAGAAAGTATATCAATAG	G/A	AGGAAATAAAAACAATAAAAATGTAATCAATAAATAATAAATGATTTAATTAGCAAGAAAGTAGTAGAAAAAATAGAGAAAGAGTTGAAATTGATTAGAA
Ca_LG_7:7666863	CTGCCGATCCCCTTCAGTTGGGTCTGGGTGAGCCTTCGCTTTTTGGATCGTCTTCTGCCCAATGTCAATGTGGTACCCTCCCGTTTTTGGTTCCCATTGA	A/G	GTTTACCTTGTTTACAGGTCGACCCCATGGCAGCAAGAAAAAAAAGGCAATCTTGTGCTTTACTCCAGTGATCTCTTTCATACCGAGGCACGCAAACCCC
Ca_LG_7:8133197	AGTTAGAGGCAATGACACTATCATTTGCTACAACTACAACTAGGCTTATGCACAATTAAGGCATTGAAATACCTGAGCAGCAAATAATGACAAAATACCG	G/A	CTGCCAGCACCCACATCAACTACTACGCGGCCAGTAAAATCAGCACGGTTCTCCATAACCGCGGCATAATAGGTTCCTAAAACAATATCACGAATGAAAT
Ca_LG_7:8133200	TAGAGGCAATGACACTATCATTTGCTACAACTACAACTAGGCTTATGCACAATTAAGGCATTGAAATACCTGAGCAGCAAATAATGACAAAATACCGCTG	G/A	CCAGCACCCACATCAACTACTACGCGGCCAGTAAAATCAGCACGGTTCTCCATAACCGCGGCATAATAGGTTCCTAAAACAATATCACGAATGAAATCAT
Ca_LG_7:8133209	TGACACTATCATTTGCTACAACTACAACTAGGCTTATGCACAATTAAGGCATTGAAATACCTGAGCAGCAAATAATGACAAAATACCGCTGCCAGCACCC	C/A	ACATCAACTACTACGCGGCCAGTAAAATCAGCACGGTTCTCCATAACCGCGGCATAATAGGTTCCTAAAACAATATCACGAATGAAATCATTAGGTAAGA

Positions	-100 bp flanking sequences	SNPs within *kabuli* cultivar	+100 bp flanking sequences
Ca_LG_7:8133218	CATTTGCTACAACTACAACTAGGCTTATGCACAATTAAGGCATTGAAATACCTGAGCAGCAAATAATGACAAAATACCGCTGCCAGCACCCACATCAACT	T/C	ACTACGCGGCCAGTAAAATCAGCACGGTTCTCCATAACCGCGGCATAATAGGTTCCTAAAACAATATCACGAATGAAATCATTAGGTAAGAGTAAAATAA
Ca_LG_7:8133224	CTACAACTACAACTAGGCTTATGCACAATTAAGGCATTGAAATACCTGAGCAGCAAATAATGACAAAATACCGCTGCCAGCACCCACATCAACTACTACG	G/A	CGGCCAGTAAAATCAGCACGGTTCTCCATAACCGCGGCATAATAGGTTCCTAAAACAATATCACGAATGAAATCATTAGGTAAGAGTAAAATAAATATAT
Ca_LG_7:8415484	GGTGATGTAGCGGTGCAGTGTGTTGTAGCAGTATGACAATATTGCAGTGGAGCAGCAGCGGTGTGGTGGTGCTATGCAGTGGAGCAGCGGTGTGGTGGTG	G/A	CTATGCAGTGGAGCAGCAGTGCGGTGGTGTTGTACCTGCGATGGGAGGGGCGACGAAGGAAGGAAAAGGGGTTTTATGTTTCTGATGTGATTTTATGTTT
Ca_LG_7:8421007	GCTTTGCCATGATGTTAGAAGATGTTTCAGATTGTGCAGGTGGTTGGGCACTTGCTTGGGCTTGATCAGCACTGGGTTGTGCCTGATCAGCAGTAGATGA	A/G	AGCTCTTGTCCATACTCCATTTATGCGTCGCAGTTGCATTTGGTTCACGATTGATTCACCAAATGTCAGTGTGGATTTGAGAGATTCTTCATTGGCTAGA
Ca_LG_7:8519553	AATTGTAGATAACTTTTTAATATTGAATGCAATTAACCCTACTAACATATTTGCATTGCAGTCTAAACTCATATGTACCATTGCTAACTCAAAGTGAGAG	G/C	AATGGCACAGTGGACTTAATCTTCTTAGTTTACTATTCATGACATGAATTCTACAAATCCCAAATTTTAGCTTAACCGAAATAAACCAGAAACAAATGGT
Ca_LG_7:8519563	AACTTTTTAATATTGAATGCAATTAACCCTACTAACATATTTGCATTGCAGTCTAAACTCATATGTACCATTGCTAACTCAAAGTGAGAGAATGGCACAG	G/C	TGGACTTAATCTTCTTAGTTTACTATTCATGACATGAATTCTACAAATCCCAAATTTTAGCTTAACCGAAATAAACCAGAAACAAATGGTACTTATTATA
Ca_LG_7:8519578	AATGCAATTAACCCTACTAACATATTTGCATTGCAGTCTAAACTCATATGTACCATTGCTAACTCAAAGTGAGAGAATGGCACAGTGGACTTAATCTTCT	T/C	TAGTTTACTATTCATGACATGAATTCTACAAATCCCAAATTTTAGCTTAACCGAAATAAACCAGAAACAAATGGTACTTATTATATAATTGTTTAATATT
Ca_LG_7:8519630	CCATTGCTAACTCAAAGTGAGAGAATGGCACAGTGGACTTAATCTTCTTAGTTTACTATTCATGACATGAATTCTACAAATCCCAAATTTTAGCTTAACC	C/T	GAAATAAACCAGAAACAAATGGTACTTATTATATAATTGTTTAATATTCAACCAATGAACTTTTTATCACAATTTTTTTACCGTTAAAATATTATTCTTA
Ca_LG_7:9520523	AGACCACGGTCTCAATTTGCAAGCATGACAAAGCCTTTGAACTTTCCAAATTATCTTTAGTTTTTGGATCATAGGTAGGTTCAGGTGGCTGCACTGCAGC	C/A	CAAGGAAGATGTCTCCACAACAGGATCTGGGTGTGGAGGTCCAATAGATACTTTTGGTGGTCTCTGCATATGGAGACAATTTAATACCACAATAGTGTGC
Ca_LG_7:9520544	AGCATGACAAAGCCTTTGAACTTTCCAAATTATCTTTAGTTTTTGGATCATAGGTAGGTTCAGGTGGCTGCACTGCAGCCAAGGAAGATGTCTCCACAAC	C/T	AGGATCTGGGTGTGGAGGTCCAATAGATACTTTTGGTGGTCTCTGCATATGGAGACAATTTAATACCACAATAGTGTGCTTGCGTATTTTGAAATAACAA
Ca_LG_7:9520545	GCATGACAAAGCCTTTGAACTTTCCAAATTATCTTTAGTTTTTGGATCATAGGTAGGTTCAGGTGGCTGCACTGCAGCCAAGGAAGATGTCTCCACAACA	A/T	GGATCTGGGTGTGGAGGTCCAATAGATACTTTTGGTGGTCTCTGCATATGGAGACAATTTAATACCACAATAGTGTGCTTGCGTATTTTGAAATAACAAA
Ca_LG_7:9520557	CTTTGAACTTTCCAAATTATCTTTAGTTTTTGGATCATAGGTAGGTTCAGGTGGCTGCACTGCAGCCAAGGAAGATGTCTCCACAACAGGATCTGGGTGT	T/C	GGAGGTCCAATAGATACTTTTGGTGGTCTCTGCATATGGAGACAATTTAATACCACAATAGTGTGCTTGCGTATTTTGAAATAACAAACTTCTAGCATAC

Positions	-100 bp flanking sequences	SNPs within *kabuli* cultivar	+100 bp flanking sequences
Ca_LG_7:9881077	CAAATGCATAATGATAGTAGGGTTGAGATATCCTCGCCCTCC GAACCCAAACGTGAAGGTCTCCCCTCATCTGAATCTTTGCAG GGGAATCTCCACTCAT	T/C	TGCTTCCCTTAATATCCTCCACCACATCATGGTGGTTTACAG GAGATCCCAGAGGCTCGCTCAGAAAGGCTGTTACCATACAT TTTGGCACAACTTTACT
Ca_LG_7:9881086	AATGATAGTAGGGTTGAGATATCCTCGCCCTCCGAACCCAAA CGTGAAGGTCTCCCCTCATCTGAATCTTTGCAGGGGAATCTC CACTCATTGCTTCCCT	T/C	TAATATCCTCCACCACATCATGGTGGTTTACAGGAGATCCCA GAGGCTCGCTCAGAAAGGCTGTTACCATACATTTTGGCACA ACTTTACTCAAGTAGCC
Ca_LG_7:9881110	TCGCCCTCCGAACCCAAACGTGAAGGTCTCCCCTCATCTGAA TCTTTGCAGGGGAATCTCCACTCATTGCTTCCCTTAATATCCT CCACCACATCATGGT	T/G	GGTTTACAGGAGATCCCAGAGGCTCGCTCAGAAAGGCTGTT ACCATACATTTTGGCACAACTTTACTCAAGTAGCCACTAGAC TCACTATAGTAGTTCGT
Ca_LG_7:9881140	CCCTCATCTGAATCTTTGCAGGGGAATCTCCACTCATTGCTTC CCTTAATATCCTCCACCACATCATGGTGGTTTACAGGAGATCC CAGAGGCTCGCTCA	A/G	GAAAGGCTGTTACCATACATTTTGGCACAACTTTACTCAAGT AGCCACTAGACTCACTATAGTAGTTCGTTCGTCTGTCCTTGT TGAAATCAATGCTTTT
Ca_LG_7:9881265	CACAACTTTACTCAAGTAGCCACTAGACTCACTATAGTAGTTC GTTCGTCTGTCCTTGTTGAAATCAATGCTTTTCATTCTTTTGTG CTCTAGAAATTGT	T/C	GCTCCCTAGACAAACCATGTAGTTAGGTAGAGACAATCAATA CACTTATACGCAATGAAGGGAATCTGGGATCCCTATCGATAT AAAAAACGAATAATAT
Ca_LG_7:9881375	CAAACCATGTAGTTAGGTAGAGACAATCAATACACTTATACGC AATGAAGGGAATCTGGGATCCCTATCGATATAAAAAACGAATA ATATGTGTATAGAT	T/G	AAGCTACCTTTCTCACACTCAATAGATATATCTGGTACAGTTA AGTACAATACGAGACAATGGAATGCTATGGGATGGATGGTA TAGGGTTGTTTGTGCC
Ca_LG_7:9881390	GGTAGAGACAATCAATACACTTATACGCAATGAAGGGAATCT GGGATCCCTATCGATATAAAAAACGAATAATATGTGTATAGAT AAGCTACCTTTCTCA	A/T	CACTCAATAGATATATCTGGTACAGTTAAGTACAATACGAGA CAATGGAATGCTATGGGATGGATGGTATAGGGTTGTTTGTG CCCAAAAGTGACGATTC
Ca_LG_7:9881403	AATACACTTATACGCAATGAAGGGAATCTGGGATCCCTATCGA TATAAAAAACGAATAATATGTGTATAGATAAGCTACCTTTCTCA CACTCAATAGATA	A/G	TATCTGGTACAGTTAAGTACAATACGAGACAATGGAATGCTA TGGGATGGATGGTATAGGGTTGTTTGTGCCCAAAAGTGACG ATTCTCTTGCCCCCTTG
Ca_LG_7:9881414	ACGCAATGAAGGGAATCTGGGATCCCTATCGATATAAAAAAC GAATAATATGTGTATAGATAAGCTACCTTTCTCACACTCAATA GATATATCTGGTACA	A/G	GTTAAGTACAATACGAGACAATGGAATGCTATGGGATGGAT GGTATAGGGTTGTTTGTGCCCAAAAGTGACGATTCTCTTGCC CCCTTGTCCATAAGGAC
Ca_LG_7:9881417	CAATGAAGGGAATCTGGGATCCCTATCGATATAAAAAACGAAT AATATGTGTATAGATAAGCTACCTTTCTCACACTCAATAGATAT ATCTGGTACAGTT	T/A	AAGTACAATACGAGACAATGGAATGCTATGGGATGGATGGT ATAGGGTTGTTTGTGCCCAAAAGTGACGATTCTCTTGCCCCC TTGTCCATAAGGACCTT
Ca_LG_7:9881418	AATGAAGGGAATCTGGGATCCCTATCGATATAAAAAACGAATA ATATGTGTATAGATAAGCTACCTTTCTCACACTCAATAGATATA TCTGGTACAGTTA	A/C	AGTACAATACGAGACAATGGAATGCTATGGGATGGATGGTA TAGGGTTGTTTGTGCCCAAAAGTGACGATTCTCTTGCCCCCT TGTCCATAAGGACCTTG
Ca_LG_7:9881434	GATCCCTATCGATATAAAAAACGAATAATATGTGTATAGATAA GCTACCTTTCTCACACTCAATAGATATATCTGGTACAGTTAAG TACAATACGAGACA	A/G	ATGGAATGCTATGGGATGGATGGTATAGGGTTGTTTGTGCC CAAAAGTGACGATTCTCTTGCCCCCTTGTCCATAAGGACCTT GTGGCATACATGTGAAA

Positions	-100 bp flanking sequences	SNPs within *kabuli* cultivar	+100 bp flanking sequences
Ca_LG_7:10260548	TTTCAAGTTAATTTCATTGAACCTTATCTGGCTCAACCCACAGGTCAGATTTCAAAAAAACCTATCGATATACTTGTTTTGATGTAGCGTAAATGCCTGA	A/C	TGTAGATGCTATCACTTATTCTGCCTCGGTCGGATGTTGCCTTAGTTGATGTGATAGAATCCCCCTTGCTTCCTAATCATTTACTATGGTCTTGCTCAGT
Ca_LG_7:10264601	ATATTCAATTCCATTCCTCCATAACCATGCTTTCCAACTCGATCCAGCTCTCAAACTATACGATCCTCGTCACCAGTCAGCAGCTAATACTGATAGCTCA	A/C	TGCGGACCCTTGCCTTCCATCTTCCTTCCTAGGCTCGGATGTTACATCCGAATGTTGCAGCCTAGTCAAATCGGTATTTCCCCGCCTACCATTAAATCAA
Ca_LG_7:10264620	CATAACCATGCTTTCCAACTCGATCCAGCTCTCAAACTATACGATCCTCGTCACCAGTCAGCAGCTAATACTGATAGCTCATGCGGACCCTTGCCTTCCA	A/C	TCTTCCTTCCTAGGCTCGGATGTTACATCCGAATGTTGCAGCCTAGTCAAATCGGTATTTCCCCGCCTACCATTAAATCAAAATAAAGAAGTAGGGGCTT
Ca_LG_7:10266295	GAATGCTTAGCTCAGAACCTAAACTACTGGCTTTCTCGCATTGAAGGTATCTGTTGAGGGAATCTATCCCAAAGGTAGGTCCAAGGCTGCTTAATGAGCA	A/G	CCCCTTGAAAGAACAAGAGCTGCATTCATCCCACCTGCGGAAGTCTCTTAACCACCTATAAATGTCCCTTTTTGGCTAGTATTATCCAAAATCTAGAAAG
Ca_LG_7:10267740	CTAGACTAATAAGGCGAGACTTAGGGACCTAGCTAGATTCACTAGCACCGTCTACTCTCACAGCGGATAGGGATTCCGTTCCCTTTCTAATTGTAAGAGT	T/C	TGCTACGGATATAAGTCCCACAGACCCCCCTTCTCGAGCTTATTCTTTTTAAGAGACTGATGCCAGCTATGAATATACGATCGAATGCGCTCTTGTCTAT
Ca_LG_7:10267765	GACCTAGCTAGATTCACTAGCACCGTCTACTCTCACAGCGGATAGGGATTCCGTTCCCTTTCTAATTGTAAGAGTTGCTACGGATATAAGTCCCACAGAC	C/T	CCCCCTTCTCGAGCTTATTCTTTTTAAGAGACTGATGCCAGCTATGAATATACGATCGAATGCGCTCTTGTCTATTTTTAGGTTTCTTACTTTTATTTTT
Ca_LG_7:10267806	ATAGGGATTCCGTTCCCTTTCTAATTGTAAGAGTTGCTACGGATATAAGTCCCACAGACCCCCCTTCTCGAGCTTATTCTTTTTAAGAGACTGATGCCAG	G/A	CTATGAATATACGATCGAATGCGCTCTTGTCTATTTTTAGGTTTCTTACTTTTATTTTTGCATTTTCTCTTTAAAGCCTTCCCCAACGAAACAAATGATG
Ca_LG_7:10282318	CGGGCAGGAGTGGGACATTCCATAAAAATATTTCTGATCCTCTAGCTCTCACTCCTTGCCCTATTCGGTCAAGCAGCTTCACTCCTCTCAAATCCTATTA	A/T	TTTCATCGACAAGTAGGGTTAATTATAAGGTTCCTTATTCTGGTTGTCAAGCTGAAGAAGAGGGAGAAAGAATGGGCACAACCCCACCAGCAGCCCGTGT
Ca_LG_7:10282330	GGACATTCCATAAAAATATTTCTGATCCTCTAGCTCTCACTCCTTGCCCTATTCGGTCAAGCAGCTTCACTCCTCTCAAATCCTATTATTTCATCGACAA	A/G	GTAGGGTTAATTATAAGGTTCCTTATTCTGGTTGTCAAGCTGAAGAAGAGGGAGAAAGAATGGGCACAACCCCACCAGCAGCCCGTGTTTTCGACCTGAA
Ca_LG_7:10282343	AAATATTTCTGATCCTCTAGCTCTCACTCCTTGCCCTATTCGGTCAAGCAGCTTCACTCCTCTCAAATCCTATTATTTCATCGACAAGTAGGGTTAATTA	A/C	TAAGGTTCCTTATTCTGGTTGTCAAGCTGAAGAAGAGGGAGAAAGAATGGGCACAACCCCACCAGCAGCCCGTGTTTTCGACCTGAAAGGAATGGAAAAT
Ca_LG_7:10282399	CTCCTCTCAAATCCTATTATTTCATCGACAAGTAGGGTTAATTATAAGGTTCCTTATTCTGGTTGTCAAGCTGAAGAAGAGGGAGAAAGAATGGGCACAA	A/G	CCCCACCAGCAGCCCGTGTTTTCGACCTGAAAGGAATGGAAAATGAAATAAGAATTCGTGTTCACTATCTATGGAGCGGAGTGTCTATCCTTAATCTCCT
Ca_LG_7:10341739	TCATAGGGTCTTTCTGTCCAGGTGCAGGTAGTCCGCATCTTCACAGACATGTCTATTTCACCGAGCCTCTCTCCGAGACAGTGCCCAGATCGTTACGCCT	T/A	TTCGTGCGGGTCGGAACTTACCCGACAAGGAATTTCGCTACCTTAGGACCGTTATAGTTACGGCCGCCGTTCACCGGGGCTTCGGTCGCTGGCTTCCCTG

Positions	-100 bp flanking sequences	SNPs within *kabuli* cultivar	+100 bp flanking sequences
Ca_LG_7:10341747	TCTTTCTGTCCAGGTGCAGGTAGTCCGCATCTTCACAGACATGTCTATTTCACCGAGCCTCTCTCCGAGACAGTGCCCAGATCGTTACGCCTTTCGTGCG	G/A	GGTCGGAACTTACCCGACAAGGAATTTCGCTACCTTAGGACCGTTATAGTTACGGCCGCCGTTCACCGGGGCTTCGGTCGCTGGCTTCCCTGTCATCAGG
Ca_LG_7:10478324	AGATCCAATTGATGATGCTCCTCGTGCATATGCAGCAAATATATACAATCCACAAGTCAACTATGATCTATCATCTAACAGGTACAATCCTGGTTGGAGA	A/G	AACCATCCCAACCTGAGATGGGAGAATTCATCTGCACAACATCAACGTAGGCAGCATCAACAACAAAATATTCCTCAAAAAAAAAATGCACCTCCATTAG
Ca_LG_7:10503469	AGCACTTTATTCCTTTGTGGTGGCCTTTGCAGCCACAAAAGGTAACAGTCTCAGGCGTTTGTGGCGGCCTGTGCAGCCACAAATGGACATATCCCTTCAG	G/A	GCTTTTGTGGCGGATTTGACCCTCACAAATTTTAGATTTTGTGGCTGAAAAAGCCCTCACAAAAATTTTAAAAATTTAAGTGGATTTTGTGGCTGCTTAA
Ca_LG_7:10503482	TTTGTGGTGGCCTTTGCAGCCACAAAAGGTAACAGTCTCAGGCGTTTGTGGCGGCCTGTGCAGCCACAAATGGACATATCCCTTCAGGCTTTTGTGGCGG	G/A	ATTTGACCCTCACAAATTTTAGATTTTGTGGCTGAAAAAGCCCTCACAAAAATTTTAAAAATTTAAGTGGATTTTGTGGCTGCTTAAGCCCTCACAAAAT
Ca_LG_7:10503536	CCTGTGCAGCCACAAATGGACATATCCCTTCAGGCTTTTGTGGCGGATTTGACCCTCACAAATTTTAGATTTTGTGGCTGAAAAAGCCCTCACAAAAATT	T/C	TTAAAAATTTAAGTGGATTTTGTGGCTGCTTAAGCCCTCACAAAATCACAGCGTTGTGATTTTGTGAGGGCTTAGGCAGCCAAAAATTCCACCTAAATTT
Ca_LG_7:11185996	CAACCCTGCATTCCAGTATCCTATGATGCCAGCATGTAGAGCTACAACGGCTTCCCCTATCTTTGTTGCAGCTCCCGCCGAGAAAGGAAACTTTGTTGTC	C/T	GACTTTCTCATGGGTGGAGTTTCAGCTGCTGTGTCTAAAACTGCTGCTGCTCCAATTGAACGAATTAAGCTTTTGATCCAGAACCAAGATGAGATGATTA
Ca_LG_7:11292544	TGTAACGGATGGTGGTGTTGAGCAGAAATATCCTGCTAAGGACTGTGCTTCGAGTGAAAAAAACTTTTGTTGCTGCCGTGCTAGCTGCATTCGCATTTCA	A/C	CGATTCTCCTTTTGTAGCTCAAGTACTAGCTTGGCCTGGTATGTTCCTGTTTCAGTTACTGGAAATAGATCCTCATTTGCGTCATTTGCCTGCACCCATG
Ca_LG_7:11293067	GCTGCATGTTCCTCCTAGTGAATCTTTGAGAAGCTGAGTCAATTTTGAATTTCGGTATGGTATATGCTTCTTTCCATCTACAAGAGCATTGATGCAGCTA	A/G	CTTAGTGCAAGAAGAGACCGGTTAATGTTGGCACCCTCAAGAGATCTAAGTGTTATTTGATATGTGGCAAGAGCTCTCTCTGACCCTGCAAGATCAACCA
Ca_LG_7:11293088	ATCTTTGAGAAGCTGAGTCAATTTTGAATTTCGGTATGGTATATGCTTCTTTCCATCTACAAGAGCATTGATGCAGCTACTTAGTGCAAGAAGAGACCGG	G/A	TTAATGTTGGCACCCTCAAGAGATCTAAGTGTTATTTGATATGTGGCAAGAGCTCTCTCTGACCCTGCAAGATCAACCAACGAGAGCTTTCCCACATTTT
Ca_LG_7:11293446	TGAAGATGTTTCATTTGCACGAGTTGGTTCTGTAGTTCTGTTATGATTTCCTTGCTGAAGCAATATCATCACCTGAAGCAGCAAAAGCAAATTAAAACTC	C/T	AAAAAATTCATTCAGCAATCTTCCAATATACCAGAATTGTTCAAAATATTACTTCATCAGTAGAGTAAACTCTGTATTGTGTAAGACCTACTGCCACAAT
Ca_LG_7:11293453	GTTTCATTTGCACGAGTTGGTTCTGTAGTTCTGTTATGATTTCCTTGCTGAAGCAATATCATCACCTGAAGCAGCAAAAGCAAATTAAAACTCAAAAAAT	T/A	TCATTCAGCAATCTTCCAATATACCAGAATTGTTCAAAATATTACTTCATCAGTAGAGTAAACTCTGTATTGTGTAAGACCTACTGCCACAATCCCCTAA
Ca_LG_7:11293484	TGTTATGATTTCCTTGCTGAAGCAATATCATCACCTGAAGCAGCAAAAGCAAATTAAAACTCAAAAAATTCATTCAGCAATCTTCCAATATACCAGAATT	T/G	GTTCAAAATATTACTTCATCAGTAGAGTAAACTCTGTATTGTGTAAGACCTACTGCCACAATCCCCTAAAATATAGTTAGACAAGGTACGATCAGATTGT

Positions	-100 bp flanking sequences	SNPs within *kabuli* cultivar	+100 bp flanking sequences
Ca_LG_7:11333084	AGAATGTTTTTGCTGTCGCAGTTGCAACAGAATATTCTTTCCCTGAGGATGACAAGGTCAAGGAGTTCCTAAAGGACCCAAGCAAGTTTGCTGTAGCTGT	T/C	AGTTGCTTCCCCAGCTGCTGATTCTGGTACTGCCCCTTCTGCTGCTGCCAAGGAGGAGGAGAAGAAGGATGAATTGATGGTATAGTCTATGTTACTGATC
Ca_LG_7:11333092	TTTGCTGTCGCAGTTGCAACAGAATATTCTTTCCCTGAGGATGACAAGGTCAAGGAGTTCCTAAAGGACCCAAGCAAGTTTGCTGTAGCTGTAGTTGCTT	T/G	CCCCAGCTGCTGATTCTGGTACTGCCCCTTCTGCTGCTGCCAAGGAGGAGGAGAAGAAGGATGAATTGATGGTATAGTCTATGTTACTGATCCTGGATTT
Ca_LG_7:11882749	CTATTTTTACAAAAGATATGAAGGCTGTGAGGGTGTTCTTGAACACTGAATCCCTGTATTTCATACCCATCGGGAACTTGCCCAGGCTGCGCAACCTCTA	A/G	GTTGAGGTGTGGGCTTTCCTCTGGGATGTTCTAAGTCCCTAAAGATAGACTCCCCACTTATGCCCAAGGAATTTCTTTTGAGCGCACCATGAAGCCGTTG
Ca_LG_7:11882759	AAAAGATATGAAGGCTGTGAGGGTGTTCTTGAACACTGAATCCCTGTATTTCATACCCATCGGGAACTTGCCCAGGCTGCGCAACCTCTAGTTGAGGTGT	T/C	GGGCTTTCCTCTGGGATGTTCTAAGTCCCTAAAGATAGACTCCCCACTTATGCCCAAGGAATTTCTTTTGAGCGCACCATGAAGCCGTTGACTAATTCCC
Ca_LG_7:11882765	TATGAAGGCTGTGAGGGTGTTCTTGAACACTGAATCCCTGTATTTCATACCCATCGGGAACTTGCCCAGGCTGCGCAACCTCTAGTTGAGGTGTGGGCTT	T/C	TCCTCTGGGATGTTCTAAGTCCCTAAAGATAGACTCCCCACTTATGCCCAAGGAATTTCTTTTGAGCGCACCATGAAGCCGTTGACTAATTCCCTAAGAA
Ca_LG_7:11882771	GGCTGTGAGGGTGTTCTTGAACACTGAATCCCTGTATTTCATACCCATCGGGAACTTGCCCAGGCTGCGCAACCTCTAGTTGAGGTGTGGGCTTTCCTCT	T/C	GGGATGTTCTAAGTCCCTAAAGATAGACTCCCCACTTATGCCCAAGGAATTTCTTTTGAGCGCACCATGAAGCCGTTGACTAATTCCCTAAGAAGGCTGT
Ca_LG_7:11882775	GTGAGGGTGTTCTTGAACACTGAATCCCTGTATTTCATACCCATCGGGAACTTGCCCAGGCTGCGCAACCTCTAGTTGAGGTGTGGCTTTCCTCTGGGA	A/C	TGTTCTAAGTCCCTAAAGATAGACTCCCCACTTATGCCCAAGGAATTTCTTTTGAGCGCACCATGAAGCCGTTGACTAATTCCCTAAGAAGGCTGTGAAT
Ca_LG_7:11882781	GTGTTCTTGAACACTGAATCCCTGTATTTCATACCCATCGGGAACTTGCCCAGGCTGCGCAACCTCTAGTTGAGGTGTGGGCTTTCCTCTGGGATGTTCT	T/C	AAGTCCCTAAAGATAGACTCCCCACTTATGCCCAAGGAATTTCTTTTGAGCGCACCATGAAGCCGTTGACTAATTCCCTAAGAAGGCTGTGAATTTATTT
Ca_LG_7:11882802	CTGTATTTCATACCCATCGGGAACTTGCCCAGGCTGCGCAACCTCTAGTTGAGGTGTGGGCTTTCCTCTGGGATGTTCTAAGTCCCTAAAGATAGACTCC	C/G	CCACTTATGCCCAAGGAATTTCTTTTGAGCGCACCATGAAGCCGTTGACTAATTCCCTAAGAAGGCTGTGAATTTATTTATGAAAACAACTCTGTTTAAA
Ca_LG_7:11882805	TATTTCATACCCATCGGGAACTTGCCCAGGCTGCGCAACCTCTAGTTGAGGTGTGGGCTTTCCTCTGGGATGTTCTAAGTCCCTAAAGATAGACTCCCCA	A/G	CTTATGCCCAAGGAATTTCTTTTGAGCGCACCATGAAGCCGTTGACTAATTCCCTAAGAAGGCTGTGAATTTATTTATGAAAACAACTCTGTTTAAAACC
Ca_LG_7:11882815	CCATCGGGAACTTGCCCAGGCTGCGCAACCTCTAGTTGAGGTGTGGGCTTTCCTCTGGGATGTTCTAAGTCCCTAAAGATAGACTCCCCACTTATGCCCA	A/G	AGGAATTTCTTTTGAGCGCACCATGAAGCCGTTGACTAATTCCCTAAGAAGGCTGTGAATTTATTTATGAAAACAACTCTGTTTAAAACCACAACAACAC
Ca_LG_7:11882816	CATCGGGAACTTGCCCAGGCTGCGCAACCTCTAGTTGAGGTGTGGGCTTTCCTCTGGGATGTTCTAAGTCCCTAAAGATAGACTCCCCACTTATGCCCAA	A/G	GGAATTTCTTTTGAGCGCACCATGAAGCCGTTGACTAATTCCCTAAGAAGGCTGTGAATTTATTTATGAAAACAACTCTGTTTAAAACCACAACAACACA

Positions	-100 bp flanking sequences	SNPs within *kabuli* cultivar	+100 bp flanking sequences
Ca_LG_7:11882828	GCCCAGGCTGCGCAACCTCTAGTTGAGGTGTGGGCTTTCCTCTGGGATGTTCTAAGTCCCTAAAGATAGACTCCCCACTTATGCCCAAGGAATTTCTTTT	T/C	GAGCGCACCATGAAGCCGTTGACTAATTCCCTAAGAAGGCTGTGAATTTATTTATGAAAACAACTCTGTTTAAAACCACAACAACACATGTGAGAAATTT
Ca_LG_7:11882839	GCAACCTCTAGTTGAGGTGTGGGCTTTCCTCTGGGATGTTCTAAGTCCCTAAAGATAGACTCCCCACTTATGCCCAAGGAATTTCTTTTGAGCGCACCAT	T/G	GAAGCCGTTGACTAATTCCCTAAGAAGGCTGTGAATTTATTTATGAAAACAACTCTGTTTAAAACCACAACAACACATGTGAGAAATTTCCAAAATATTG
Ca_LG_7:12548370	GGTGAACTGGGAATCATTCAAAAGGAAGTTTTTAGACAAATACTTTCCGATGAGTACCAGGACTAAACTGGGTGATGATTTTCTGAAGCTTCACCAGGGG	G/A	AGCATGACCGTGGGCGAATATGCTGCTAAGTTTGAATCGCTATCAAGGCATTTCATGCTCTTCCGTGAAGAAATTGATGAACCATTCATGTGTCATCGTT
Ca_LG_7:12552938	TGTAAGCTTTTATGTGCCCAAAAAGAACCAAGCAAATCCACCACCTTCAAAGGGTCTTCAAGTTCAAAAATGGAAAAATGTGTATGCAGTATTTAGACAG	G/A	TTTGCTGGATATGTGAATGACAAAAATGTTGCAGAACAAGCTGCAACATTGAAGGATAGTATCAATGGTACTAAATGGCTATCTGCTATTGAAAAAAGTC
Ca_LG_7:12552943	GCTTTTATGTGCCCAAAAAGAACCAAGCAAATCCACCACCTTCAAAGGGTCTTCAAGTTCAAAAATGGAAAAATGTGTATGCAGTATTTAGACAGTTTGC	C/G	TGGATATGTGAATGACAAAAATGTTGCAGAACAAGCTGCAACATTGAAGGATAGTATCAATGGTACTAAATGGCTATCTGCTATTGAAAAAAGTCATAAA
Ca_LG_7:12552970	CAAATCCACCACCTTCAAAGGGTCTTCAAGTTCAAAAATGGAAAAATGTGTATGCAGTATTTAGACAGTTTGCTGGATATGTGAATGACAAAAATGTTGC	C/T	AGAACAAGCTGCAACATTGAAGGATAGTATCAATGGTACTAAATGGCTATCTGCTATTGAAAAAAGTCATAAAGCTCCTAATTTCTATGTGGCTCGGTAT
Ca_LG_7:14030720	ATAAAATATTCATTGATTACTGGTTAACGTGATAAACAGAATAACAGTGAAGTATCAAAAGAAAAATATGGATGGCAGGCTACGCTCACGTCCTTATACA	A/G	AAAACCAACAAAATAATAATAGGGATAACAAATAGGGATATTAGCTAGTAGTGATAGCATGCAGTAAAATCTTTATAAAATAATAAATAAAAAAACAAAG
Ca_LG_7:14571742	CGGTGTTTGTTGGTTTTGGATGCGCACTAGAGAGGAGGCAGCTGGTGTTGACACATCTGTAACAGAGTAAAAAAAAAATAGACAGAATAGTGACAGCTGA	A/G	TGTAAAGCACAGTTGTGTTTGTAAGAAAAAAAAAGTCACTGGAAAAACGCATAAGGAAGGCAGCGCTTTTAAGGCAGCTGTAGCGTTGTGGCTGGTTGCT
Ca_LG_7:14571761	ATGCGCACTAGAGAGGAGGCAGCTGGTGTTGACACATCTGTAACAGAGTAAAAAAAAAATAGACAGAATAGTGACAGCTGATGTAAAGCACAGTTGTGTT	T/C	TGTAAGAAAAAAAAAGTCACTGGAAAAACGCATAAGGAAGGCAGCGCTTTTAAGGCAGCTGTAGCGTTGTGGCTGGTTGCTAGAAAGGCAGGCGGAGTTG
Ca_LG_7:14571764	CGCACTAGAGAGGAGGCAGCTGGTGTTGACACATCTGTAACAGAGTAAAAAAAAAATAGACAGAATAGTGACAGCTGATGTAAAGCACAGTTGTGTTTGT	T/A	AAGAAAAAAAAAGTCACTGGAAAAACGCATAAGGAAGGCAGCGCTTTTAAGGCAGCTGTAGCGTTGTGGCTGGTTGCTAGAAAGGCAGGCGGAGTTGACA
Ca_LG_7:14571783	CTGGTGTTGACACATCTGTAACAGAGTAAAAAAAAAATAGACAGAATAGTGACAGCTGATGTAAAGCACAGTTGTGTTTGTAAGAAAAAAAAAGTCACTG	G/T	GAAAAACGCATAAGGAAGGCAGCGCTTTTAAGGCAGCTGTAGCGTTGTGGCTGGTTGCTAGAAAGGCAGGCGGAGTTGACAGTGACAGATGATTGTATGA
Ca_LG_7:14694733	CTTAAGCAACCACAAAATCCACTTTAATTTTTAAGATTTTTGTGAGGGCTTTTTAGCCACAAAATTTAAAATTTGTGAGGGTCAAATCCGTCACAAAAGC	C/T	CTGAACTGACATGTCCATTTGTGGCTGCATAGGCCGCCACAAACGCATGAGACCGTTACCTTTTGTGGCTGCAAATGCCGCCACAAAGGAATAAAGTGCT

Positions	-100 bp flanking sequences	SNPs within *kabuli* cultivar	+100 bp flanking sequences
Ca_LG_7:14694780	GCTTTTTAGCCACAAAATTTAAAATTTGTGAGGGTCAAATCCG TCACAAAAGCCTGAACTGACATGTCCATTTGTGGCTGCATAG GCCGCCACAAACGCA	A/T	TGAGACCGTTACCTTTTGTGGCTGCAAATGCCGCCACAAAG GAATAAAGTGCTACGTGTTTTTCTGGCCGTCCAGGCTACCAC AAATATTTATATCCATT
Ca_LG_7:14694809	GAGGGTCAAATCCGTCACAAAAGCCTGAACTGACATGTCCAT TTGTGGCTGCATAGGCCGCCACAAACGCATGAGACCGTTACC TTTTGTGGCTGCAAAT	T/G	GCCGCCACAAAGGAATAAAGTGCTACGTGTTTTTCTGGCCG TCCAGGCTACCACAAATATTTATATCCATTACATCTTGTGGC GGCAAAGGCCGCCACAA
Ca_LG_7:14694833	CTGAACTGACATGTCCATTTGTGGCTGCATAGGCCGCCACAA ACGCATGAGACCGTTACCTTTTGTGGCTGCAAATGCCGCCAC AAAGGAATAAAGTGCT	T/A	ACGTGTTTTTCTGGCCGTCCAGGCTACCACAAATATTTATAT CCATTACATCTTGTGGCGGCAAAGGCCGCCACAAACGCCCA AAGGACCGTTGCCAGTT
Ca_LG_7:14694835	GAACTGACATGTCCATTTGTGGCTGCATAGGCCGCCACAAAC GCATGAGACCGTTACCTTTTGTGGCTGCAAATGCCGCCACAA AGGAATAAAGTGCTAC	C/T	GTGTTTTTCTGGCCGTCCAGGCTACCACAAATATTTATATCC ATTACATCTTGTGGCGGCAAAGGCCGCCACAAACGCCCAAA GGACCGTTGCCAGTTGT
Ca_LG_7:15469155	TGGTTGGTATACTCCTAAAAAGATATCAGCTTCGTTACAGTGG CCGAAGGACTCACCTGTAGTAAGAAGCAAAAATTATGTCATTT CATACAAGAAGCAA	A/G	ATTGGATGGAAATTTGTTCCTATGTTTCATCGTCTTTTGCAG GCTGTTCCGTATACTTTCTGCTGCTGCTAGACTTCATATTTG GTATTAAGAATCTTCA
Ca_LG_7:15469169	CTAAAAAGATATCAGCTTCGTTACAGTGGCCGAAGGACTCAC CTGTAGTAAGAAGCAAAAATTATGTCATTTCATACAAGAAGCA AATTGGATGGAAATT	T/A	TGTTCCTATGTTTCATCGTCTTTTGCAGGCTGTTCCGTATACT TTCTGCTGCTGCTAGACTTCATATTTGGTATTAAGAATCTTCA GATTTCATTCTTTG
Ca_LG_7:15469173	AAAGATATCAGCTTCGTTACAGTGGCCGAAGGACTCACCTGT AGTAAGAAGCAAAAATTATGTCATTTCATACAAGAAGCAAATT GGATGGAAATTTGTT	T/C	CCTATGTTTCATCGTCTTTTGCAGGCTGTTCCGTATACTTTCT GCTGCTGCTAGACTTCATATTTGGTATTAAGAATCTTCAGATT TCATTCTTTGTTTG
Ca_LG_7:15469177	ATATCAGCTTCGTTACAGTGGCCGAAGGACTCACCTGTAGTA AGAAGCAAAAATTATGTCATTTCATACAAGAAGCAAATTGGAT GGAAATTTGTTCCTA	A/G	TGTTTCATCGTCTTTTGCAGGCTGTTCCGTATACTTTCTGCT GCTGCTAGACTTCATATTTGGTATTAAGAATCTTCAGATTTCA TTCTTTGTTTGATAC
Ca_LG_7:15584199	TTCCAACAACTCCGTCAGCATCGGTAACGGATTGGAGGTGAA AACAGCAAGCTTCAAGGGCAGCATTACAAGCAGAAACATCAG GTATCATGAAATCAAA	A/G	CTTCTCATCGGAAGCAACCCTGCAATTATGGATGAAAAATCT GTTACGGAAGAGAGAGGTGAGAAGTGAAAAAGCAGGGGGA GCAGTGTTGGCAGATTTC
Ca_LG_7:17162291	CAACTAAAATTGTTTTCCCCAAATTCCTTAAAATTATGGTTCTA AATTTCTAGGTTTTGAAGTATCAATGTCAACTGTGAACATGTC TTACAGCAGCGAC	C/T	GGAACCAAATCGTGCCGTTACCCACGGCCACAATCATCTCC AATTTAAGATGCTGCTATTGTTTAACACAGTCATTCATAATTA TTATGAAATTTTTTTA
Ca_LG_7:17601974	GAGCCCCAATCGTTGTCTTCTCCTTCTCGCCGTTCTCACTCAA AACTGTCGTTCTCACCGTTCTAGCCGTCAATCAATCAGCTGCA CTCGCCGTCGAACC	C/T	AGTCGCGCACTCACCGTCGAACCGGAGCTGCACTCGCCGT CGAACCGGAGCTGCACTCGTCGTCGAACCAGTCGCGCCGT CGAACCCGAGCTGCACTCAC
Ca_LG_7:17602002	GCCGTTCTCACTCAAAACTGTCGTTCTCACCGTTCTAGCCGTC AATCAATCAGCTGCACTCGCCGTCGAACCAGTCGCGCACTCA CCGTCGAACCGGAGC	C/T	TGCACTCGCCGTCGAACCGGAGCTGCACTCGTCGTCGAACC AGTCGCGCCGTCGAACCCGAGCTGCACTCACCGTCGTTCTC ACCGTTCTCGCACCACTG

Positions	-100 bp flanking sequences	SNPs within *kabuli* cultivar	+100 bp flanking sequences
Ca_LG_7:17602025	TTCTCACCGTTCTAGCCGTCAATCAATCAGCTGCACTCGCCGTCGAACCAGTCGCGCACTCACCGTCGAACCGGAGCTGCACTCGCCGTCGAACCGGAGC	C/T	TGCACTCGTCGTCGAACCAGTCGCGCCGTCGAACCCGAGCTGCACTCACCGTCGTTCTCACCGTTCTCGCACCACTGGTAATTTCTTGAACCACCATTAT
Ca_LG_7:17602034	TTCTAGCCGTCAATCAATCAGCTGCACTCGCCGTCGAACCAGTCGCGCACTCACCGTCGAACCGGAGCTGCACTCGCCGTCGAACCGGAGCTGCACTCGT	T/C	CGTCGAACCAGTCGCGCCGTCGAACCCGAGCTGCACTCACCGTCGTTCTCACCGTTCTCGCACCACTGGTAATTTCTTGAACCACCATTATGTTCTCGCA
Ca_LG_7:20919524	ATATGGGCTCAAACAATCGTCACAAACATATCCAAGGGGGCAAGTCCATATAGATCTCTTCTTCATGATCTCCATGGAGAAAAACATTTTTTACATCAAA	A/T	TTGATGTAAGTTCCAACTATAATTTGCTGCTAGAGATAATATTACCTCGTCTGCTTTCATTTTTGCAACCAGAGCAAATGTCTCCGAGTAATCCACCCCA
Ca_LG_7:20919528	GGGCTCAAACAATCGTCACAAACATATCCAAGGGGGCAAGTCCATATAGATCTCTTCTTCATGATCTCCATGGAGAAAAACATTTTTTACATCAAATTGA	A/G	TGTAAGTTCCAACTATAATTTGCTGCTAGAGATAATATTACCTCGTCTGCTTTCATTTTTGCAACCAGAGCAAATGTCTCCGAGTAATCCACCCCATATG
Ca_LG_7:20919531	CTCAAACAATCGTCACAAACATATCCAAGGGGGCAAGTCCATATAGATCTCTTCTTCATGATCTCCATGGAGAAAAACATTTTTTACATCAAATTGATGT	T/C	AAGTTCCAACTATAATTTGCTGCTAGAGATAATATTACCTCGTCTGCTTTCATTTTTGCAACCAGAGCAAATGTCTCCGAGTAATCCACCCCATATGTTT
Ca_LG_7:21583375	ATATATGGCAAACCATGTTTGGATGAAAGTTAGAACCAATGCCAGAGATGCAGCTAAAAAGGCAATCATAGCCCATGGCGTATTGAAATGAGTGTTGTAA	A/T	GCTTGAGCCAACCATGTCTTCCATTTATTTATGTAATGTTTTTCAATTTGACGCTTGATGAGAATATATTTCTTACTATAGGCCACTGCATTTGTGTACG
Ca_LG_7:21583406	AGAACCAATGCCAGAGATGCAGCTAAAAAGGCAATCATAGCCCATGGCGTATTGAAATGAGTGTTGTAAGCTTGAGCCAACCATGTCTTCCATTTATTTA	A/C	TGTAATGTTTTTCAATTTGACGCTTGATGAGAATATATTTCTTACTATAGGCCACTGCATTTGTGTACGAAGTGTTGCAATACATTTTTGTTGGCAAATC
Ca_LG_7:21583440	TCATAGCCCATGGCGTATTGAAATGAGTGTTGTAAGCTTGAGCCAACCATGTCTTCCATTTATTTATGTAATGTTTTTCAATTTGACGCTTGATGAGAAT	T/C	ATATTTCTTACTATAGGCCACTGCATTTGTGTACGAAGTGTTGCAATACATTTTTGTTGGCAAATCGTCCCCTAATTCATTGAAGAGCTTTGCCAAATCT
Ca_LG_7:21583450	TGGCGTATTGAAATGAGTGTTGTAAGCTTGAGCCAACCATGTCTTCCATTTATTTATGTAATGTTTTTCAATTTGACGCTTGATGAGAATATATTTCTTA	A/G	CTATAGGCCACTGCATTTGTGTACGAAGTGTTGCAATACATTTTTGTTGGCAAATCGTCCCCTAATTCATTGAAGAGCTTTGCCAAATCTTCATCACTTC
Ca_LG_7:21583455	TATTGAAATGAGTGTTGTAAGCTTGAGCCAACCATGTCTTCCATTTATTTATGTAATGTTTTTCAATTTGACGCTTGATGAGAATATATTTCTTACTATA	A/C	GGCCACTGCATTTGTGTACGAAGTGTTGCAATACATTTTTGTTGGCAAATCGTCCCCTAATTCATTGAAGAGCTTTGCCAAATCTTCATCACTTCCAAGC
Ca_LG_7:21921405	GCCATATGGATCCCACAATTGTAAGGAGAAAGGCTCCTTCACCACAATCTCCTTTGCTGCCACCGCCGAGATTAACTTGTTAATTATTCTGCACAATTGT	T/C	ATATGTCCCACATTAGACCGGAATAAGCCACCTTTATTGTGTTGGACTTGCACACTTTCAGCTTCATTAAGGTCACACAGTAGCTCAATCTCAGAAACTT
Ca_LG_7:21921419	ACAATTGTAAGGAGAAAGGCTCCTTCACCACAATCTCCTTTGCTGCCACCGCCGAGATTAACTTGTTAATTATTCTGCACAATTGTATATGTCCCACATT	T/C	AGACCGGAATAAGCCACCTTTATTGTGTTGGACTTGCACACTTTCAGCTTCATTAAGGTCACACAGTAGCTCAATCTCAGAAACTTAGTGATCAGTCCTA

Positions	-100 bp flanking sequences	SNPs within *kabuli* cultivar	+100 bp flanking sequences
Ca_LG_7:21921423	TTGTAAGGAGAAAGGCTCCTTCACCACAATCTCCTTTGCTGCCACCGCCGAGATTAACTTGTTAATTATTCTGCACAATTGTATATGTCCCACATTAGAC	C/T	CGGAATAAGCCACCTTTATTGTGTTGGACTTGCACACTTTCAGCTTCATTAAGGTCACACAGTAGCTCAATCTCAGAAACTTAGTGATCAGTCCTACCTT
Ca_LG_7:21921424	TGTAAGGAGAAAGGCTCCTTCACCACAATCTCCTTTGCTGCCACCGCCGAGATTAACTTGTTAATTATTCTGCACAATTGTATATGTCCCACATTAGACC	C/A	GGAATAAGCCACCTTTATTGTGTTGGACTTGCACACTTTCAGCTTCATTAAGGTCACACAGTAGCTCAATCTCAGAAACTTAGTGATCAGTCCTACCTTT
Ca_LG_7:21921441	CTTCACCACAATCTCCTTTGCTGCCACCGCCGAGATTAACTTGTTAATTATTCTGCACAATTGTATATGTCCCACATTAGACCGGAATAAGCCACCTTTA	A/T	TTGTGTTGGACTTGCACACTTTCAGCTTCATTAAGGTCACACAGTAGCTCAATCTCAGAAACTTAGTGATCAGTCCTACCTTTCGCTTCTTCTAACATGC
Ca_LG_7:22604587	AATTCAAGTGTTGCATCTTTGCTTGAATGCTATTTATAACAGCTGCAGAATTAAACATCTCAAAAAATGAACTTGGACTCTTCCATTTGGTTAGGGAATA	A/G	CAGGCCTTAATTGCACTTATCCAAATATGCACTCCATCCTTGAAGACGGTATTCACGAGCTGCCGCAGCCTGATTCTCCGCCTTGATGATACCGCGTCCC
Ca_LG_7:22646952	CAGGCACTTTGTGGCTGCAAAAGCCCTCACAAAATTTTGTGACCAGCTTCTTTGTGGCTGCCAAAAGCCCTCACAAAATCCACCTTTGTGGCTGATTATA	A/G	CCCCTTTGTGAGGGTAAAAGCCCTCACAAAATGCTTGTTTTCTTGTTGTGAACGGGGGTTCTAGACTTTCTGCATAGCACTTCATAAATGCAACTTTGTT
Ca_LG_7:22785096	ACCAGATGATTTTAACCGAATATTTAAAATAAATTTGGATCTGATTTTGAATTTGGGCCTTATAACCGGATTTTTTGCACAACCCTAGCTAGCAGTGTCA	A/G	GGCTTTGCTCGCAATTTGAGTGGAACAAAAGACCTGAATTAAAGTTCAGAGTTCTCTTGTGCTGCTATCTAAAGAATGCAGAGTAAGTAAGACCCTAACG
Ca_LG_7:22785097	CCAGATGATTTTAACCGAATATTTAAAATAAATTTGGATCTGATTTTGAATTTGGGCCTTATAACCGGATTTTTTGCACAACCCTAGCTAGCAGTGTCAG	G/A	GCTTTGCTCGCAATTTGAGTGGAACAAAAGACCTGAATTAAAGTTCAGAGTTCTCTTGTGCTGCTATCTAAAGAATGCAGAGTAAGTAAGACCCTAACGA
Ca_LG_7:22785109	AACCGAATATTTAAAATAAATTTGGATCTGATTTTGAATTTGGGCCTTATAACCGGATTTTTTGCACAACCCTAGCTAGCAGTGTCAGGCTTTGCTCGCA	A/G	ATTTGAGTGGAACAAAAGACCTGAATTAAAGTTCAGAGTTCTCTTGTGCTGCTATCTAAAGAATGCAGAGTAAGTAAGACCCTAACGAGATAAAGATTAA
Ca_LG_7:22785113	GAATATTTAAAATAAATTTGGATCTGATTTTGAATTTGGGCCTTATAACCGGATTTTTTGCACAACCCTAGCTAGCAGTGTCAGGCTTTGCTCGCAATTT	T/C	GAGTGGAACAAAAGACCTGAATTAAAGTTCAGAGTTCTCTTGTGCTGCTATCTAAAGAATGCAGAGTAAGTAAGACCCTAACGAGATAAAGATTAAAAAA
Ca_LG_7:22785116	TATTTAAAATAAATTTGGATCTGATTTTGAATTTGGGCCTTATAACCGGATTTTTTGCACAACCCTAGCTAGCAGTGTCAGGCTTTGCTCGCAATTTGAG	G/C	TGGAACAAAAGACCTGAATTAAAGTTCAGAGTTCTCTTGTGCTGCTATCTAAAGAATGCAGAGTAAGTAAGACCCTAACGAGATAAAGATTAAAAAACCT
Ca_LG_7:22785123	AATAAATTTGGATCTGATTTTGAATTTGGGCCTTATAACCGGATTTTTTGCACAACCCTAGCTAGCAGTGTCAGGCTTTGCTCGCAATTTGAGTGGAACA	A/T	AAAGACCTGAATTAAAGTTCAGAGTTCTCTTGTGCTGCTATCTAAAGAATGCAGAGTAAGTAAGACCCTAACGAGATAAAGATTAAAAAACCTACTTCTA
Ca_LG_7:22785143	TGAATTTGGGCCTTATAACCGGATTTTTTGCACAACCCTAGCTAGCAGTGTCAGGCTTTGCTCGCAATTTGAGTGGAACAAAAGACCTGAATTAAAGTTC	C/A	AGAGTTCTCTTGTGCTGCTATCTAAAGAATGCAGAGTAAGTAAGACCCTAACGAGATAAAGATTAAAAAACCTACTTCTAAACAAGGGCGAGTGAAGTAA

Positions	-100 bp flanking sequences	SNPs within *kabuli* cultivar	+100 bp flanking sequences
Ca_LG_7:22815849	AACAATGGTTGTTGCTACAAGTACTGAGGCCTCGAATATTGTTGTTGTTGGTGAGGCGAGAACTGCTGATGAAACGCAAGAGGAAATGAAAATTGTTGCA	A/T	GTTGAAATTGTTGTTGTTGCTACTCTTGAAACTTTTGTTTCCATTCTTCGTTTAGTTTATCAATTGCTACACGTATCAACTGTTGTGTCTTTTATTTTTG
Ca_LG_7:24035480	TCTGATTTTGCTGCCATATTCATATAGATATCATATTAAGGATCACAAGAAGTATAAACAGATTTATCAGAGGATGCTACGCCTTACCGATGCAAGGAGG	G/A	TAAATTTTTAAAATGACAATGCAGCTTGTAACTGGAAATGGCAATGCAGCTTGTAACTGGAAATGGCAATGCACTATGTAATGTACGTCCACTAATTAAT
Ca_LG_7:24035496	TATTCATATAGATATCATATTAAGGATCACAAGAAGTATAAACAGATTTATCAGAGGATGCTACGCCTTACCGATGCAAGGAGGTAAATTTTTAAAATGA	A/G	CAATGCAGCTTGTAACTGGAAATGGCAATGCAGCTTGTAACTGGAAATGGCAATGCACTATGTAATGTACGTCCACTAATTAATCCAAATTTATGCTAAT
Ca_LG_7:24035531	GTATAAACAGATTTATCAGAGGATGCTACGCCTTACCGATGCAAGGAGGTAAATTTTTAAAATGACAATGCAGCTTGTAACTGGAAATGGCAATGCAGCT	T/A	TGTAACTGGAAATGGCAATGCACTATGTAATGTACGTCCACTAATTAATCCAAATTTATGCTAATAATAGTATACTCAAATTCAAATTCACATCTTCTAT
Ca_LG_7:24121490	AAGAAGCTAAGACGACAACAGGAAGCAATGACGGCAGCTGGAACGATGACTCATGGAGAAGATGAAGGCTGGGAAGACAGCGGCTAGCAAAGACAAGGGC	C/A	TGCGATGACATCTGCTGGTTTGCAGCAGAGAAAGATATAAGAGAATGTGAAGAGAAATAGAATTGGGTTGCTGGTTCAAAGTGAAGAGGTAAAATAGAGT
Ca_LG_7:24178892	TACTGATGCGATGACAGGGCAACCAAAAGGGTAATAATTTTCAGATGTTTATGGTCAAGATTTTTGTATTTCTCACTATATTGATTAACTTTTTTTTGGA	A/G	ACAGGGCAGCTTATGTGGAGTTCATGAGTAAAGAAGCAGCAGATAATGCGTTATCACTGGATGGAACTTCCTTCATGTCACGTATTCTTAAGGTATTGGT
Ca_LG_7:24467013	CGATCTATATATTACATTTATAGGGTTATGAAGCTGCTATTGCAGCTGGAGCTAGAGAAGTAGCTATTTTTGCATCAGCTTCGGAATCTTTCTCAAAATC	C/T	AAACATTAATTGTAGTATTGAAGAGAGTCTTGCCCGCTATCGTGCTGTTACTCGTGCTGCTAAAGAGCTCTCAATTCCAGTCCGAGGGTATGCATTTTTG
Ca_LG_7:24666413	CGTTACCTTTTGTGGCTGCAAAGGCCGCCACAAAAGCCTGAGCCGTTACCTTTTGTGGCTGCAAAGGCCGCCACAAAATCCCAAAGTGACGTTGCCATTT	T/A	GTGGCCGCCCAGGCCGCCACAAATGCTTATAGTCGTTACCTTTTGTGTCTGCAAAGGCCGCCACAAACTCCAAAAGGACCGTTGCCATTTGTGGCCGCCC
Ca_LG_7:24666429	TGCAAAGGCCGCCACAAAAGCCTGAGCCGTTACCTTTTGTGGCTGCAAAGGCCGCCACAAAATCCCAAAGTGACGTTGCCATTTGTGGCCGCCCAGGCCG	G/T	CCACAAATGCTTATAGTCGTTACCTTTTGTGTCTGCAAAGGCCGCCACAAACTCCAAAAGGACCGTTGCCATTTGTGGCCGCCCAAGCCGCCACAAACAC
Ca_LG_7:24666439	GCCACAAAAGCCTGAGCCGTTACCTTTTGTGGCTGCAAAGGCCGCCACAAAATCCCAAAGTGACGTTGCCATTTGTGGCCGCCCAGGCCGCCACAAATGC	C/G	TTATAGTCGTTACCTTTTGTGTCTGCAAAGGCCGCCACAAACTCCAAAAGGACCGTTGCCATTTGTGGCCGCCCAAGCCGCCACAAACACATGCCTTTCT
Ca_LG_7:24666446	AAGCCTGAGCCGTTACCTTTTGTGGCTGCAAAGGCCGCCACAAAATCCCAAAGTGACGTTGCCATTTGTGGCCGCCCAGGCCGCCACAAATGCTTATAGT	T/C	CGTTACCTTTTGTGTCTGCAAAGGCCGCCACAAACTCCAAAAGGACCGTTGCCATTTGTGGCCGCCCAAGCCGCCACAAACACATGCCTTTCTAACCGTT
Ca_LG_7:24825034	TTGGATGTCAAGACACGCCCCGTATTTAGCAAGGCATGGCTGACTTATTTTAGGAGCAGCAGCAGCAGCGACCGATTTTCCCCGATCCAGTGTTCTTGCA	A/T	GCAACTTTGGCAGCTTTATTACCCTCCAGCCTTTGTAGTTTGCCAGCTCTATTACCCTCCAATTTTTGAGGTTTGCTGGCTTTTTTTGGCTGTACGGGTG

Positions	-100 bp flanking sequences	SNPs within *kabuli* cultivar	+100 bp flanking sequences
Ca_LG_7:24852480	TGGTGGCTGCCTCTAGTCTAGTAAACTTACCTCTGGTCCAGTAAACTTGCTTCTAGTGGCTGCCTTTGGTCCAATAATTACACTTGCCTTTGGTCATTGC	C/G	CTCTAGTCTAATAATTATGCCTCTGGTTGCTCCACGCTTATGATCAAGTGAAGTTGATTCTGCTTCATACATGGTCAAGAAATCATGCCTTTGATTGTTC
Ca_LG_7:24911538	GAGAATCACTTTCATTACTAACATGCTTTGCCCCAACATCATCACCAACATTACCAAAAAGAAGAACAATAATTCCAACAAGTGCAGCAACAAAAGCAGA	A/C	TCCAAAAGAAGTTCTAATTTGTCCAGGAACAACTGAAGCAACAGTGAAACCCAAACACAAACCAGCAGCTCTAATCCAACCATACCAAACACATAAAACA
Ca_LG_7:26684734	GATCCCAGCTCGACGGGGAAGCATAATTAACACAGCTAGTGTTTGTGGAAGCATAGGTGGTGGTGCTTCACATGCCTGTACAAGTTCAAAGCACGCCGTC	C/T	TTAGGTCTAATGAAAAACACGGCGGTCGAGCTTGGACCGTACGGTATTCGGGTGAATTGTGTGTCACCTTATGTTGTTGCAACCCCATTGGCTAAGAAAT
Ca_LG_7:26684748	GGGGAAGCATAATTAACACAGCTAGTGTTTGTGGAAGCATAGGTGGTGGTGCTTCACATGCCTGTACAAGTTCAAAGCACGCCGTCTTAGGTCTAATGAA	A/G	AAACACGGCGGTCGAGCTTGGACCGTACGGTATTCGGGTGAATTGTGTGTCACCTTATGTTGTTGCAACCCCATTGGCTAAGAAATTCTTTAAGCTTGAT
Ca_LG_7:27314623	AATTGTATTTAACTTGTTACTATCTCGTCAATTAATAATAATGAATTATTCGTCTGTTTTCGTTCTTTTTGTGGAAGGAAGCAGCCATTTGTTATATAAA	A/T	GGAACAAACTCTACCTTCTTTGTGCGGAGATACTGTTAATGTTAATAATTAAATTAAATAAATAGATGTAATTATAATTCGTGCAGCTACCATTGATAAC
Ca_LG_7:27314624	ATTGTATTTAACTTGTTACTATCTCGTCAATTAATAATAATGAATTATTCGTCTGTTTTCGTTCTTTTTGTGGAAGGAAGCAGCCATTTGTTATATAAAG	G/T	GAACAAACTCTACCTTCTTTGTGCGGAGATACTGTTAATGTTAATAATTAAATTAAATAAATAGATGTAATTATAATTCGTGCAGCTACCATTGATAACA
Ca_LG_7:27314648	CGTCAATTAATAATAATGAATTATTCGTCTGTTTTCGTTCTTTTTGTGGAAGGAAGCAGCCATTTGTTATATAAAGGAACAAACTCTACCTTCTTTGTGC	C/G	GGAGATACTGTTAATGTTAATAATTAAATTAAATAAATAGATGTAATTATAATTCGTGCAGCTACCATTGATAACAAAGTTGTAGGATTCCGTGAAGATT
Ca_LG_7:27314670	ATTCGTCTGTTTTCGTTCTTTTTGTGGAAGGAAGCAGCCATTTGTTATATAAAGGAACAAACTCTACCTTCTTTGTGCGGAGATACTGTTAATGTTAATA	A/G	ATTAAATTAAATAAATAGATGTAATTATAATTCGTGCAGCTACCATTGATAACAAAGTTGTAGGATTCCGTGAAGATTCATAAAACTTTGTTCTTTACGC
Ca_LG_7:27327551	AATATACCTGTGTTCGCAAGTCATCAGGGCCAAGTTTTGCCCTCAGAATTTGCAAAGTTGTTTGTTCATGCTGCACACTCAATGGATATGCTTCCATCAA	A/G	CGAGAGTGCTATTGCTATTGCGTGGTAACTTGCAGCTGTCTGCAACGAATCAGCATCCAAGAAAGATGCAAATGTTATAACACAAATGAGAAGCTTTATT
Ca_LG_7:27327573	ATCAGGGCCAAGTTTTGCCCTCAGAATTTGCAAAGTTGTTTGTTCATGCTGCACACTCAATGGATATGCTTCCATCAACGAGAGTGCTATTGCTATTGCG	G/A	TGGTAACTTGCAGCTGTCTGCAACGAATCAGCATCCAAGAAAGATGCAAATGTTATAACACAAATGAGAAGCTTTATTGCATAAGTAATACAAGCCTATT
Ca_LG_7:27671648	TTGAAGATCCCTTGAGCGTACAAGATATTGAATCTCCAAGAGATTCAACTGGCCATAGGACACATACAACTTCAACTAGGAGGGATACCGATTAAGAAGA	A/G	CAAGCATGGTAGGCCTTGCACGAGGTACAACAAGAGGTGGAGCTCCATCTGCACGTATTGTTGTCTACAAATGACAGTATGTATTTTAGAGGTGCAATTT
Ca_LG_7:27671671	GATATTGAATCTCCAAGAGATTCAACTGGCCATAGGACACATACAACTTCAACTAGGAGGGATACCGATTAAGAAGACAAGCATGGTAGGCCTTGCACGA	A/G	GGTACAACAAGAGGTGGAGCTCCATCTGCACGTATTGTTGTCTACAAATGACAGTATGTATTTTAGAGGTGCAATTTCCATCGGATCATTCCATGCTATG

Positions	-100 bp flanking sequences	SNPs within *kabuli* cultivar	+100 bp flanking sequences
Ca_LG_7:27671678	AATCTCCAAGAGATTCAACTGGCCATAGGACACATACAACTTCAACTAGGAGGGATACCGATTAAGAAGACAAGCATGGTAGGCCTTGCACGAGGTACAA	A/G	CAAGAGGTGGAGCTCCATCTGCACGTATTGTTGTCTACAAATGACAGTATGTATTTTAGAGGTGCAATTTCCATCGGATCATTCCATGCTATGCAACATG
Ca_LG_7:27671709	ACATACAACTTCAACTAGGAGGGATACCGATTAAGAAGACAAGCATGGTAGGCCTTGCACGAGGTACAACAAGAGGTGGAGCTCCATCTGCACGTATTGT	T/C	TGTCTACAAATGACAGTATGTATTTTAGAGGTGCAATTTCCATCGGATCATTCCATGCTATGCAACATGGAGTGTTGACAGTATTGGCAGCTGGAAATCG
Ca_LG_7:27671713	ACAACTTCAACTAGGAGGGATACCGATTAAGAAGACAAGCATGGTAGGCCTTGCACGAGGTACAACAAGAGGTGGAGCTCCATCTGCACGTATTGTTGTC	C/G	TACAAATGACAGTATGTATTTTAGAGGTGCAATTTCCATCGGATCATTCCATGCTATGCAACATGGAGTGTTGACAGTATTGGCAGCTGGAAATCGTGTC
Ca_LG_7:27671725	AGGAGGGATACCGATTAAGAAGACAAGCATGGTAGGCCTTGCACGAGGTACAACAAGAGGTGGAGCTCCATCTGCACGTATTGTTGTCTACAAATGACAG	G/C	TATGTATTTTAGAGGTGCAATTTCCATCGGATCATTCCATGCTATGCAACATGGAGTGTTGACAGTATTGGCAGCTGGAAATCGTGTCCTCGTCCTTCAA
Ca_LG_7:27671740	TAAGAAGACAAGCATGGTAGGCCTTGCACGAGGTACAACAAGAGGTGGAGCTCCATCTGCACGTATTGTTGTCTACAAATGACAGTATGTATTTTAGAGG	G/A	TGCAATTTCCATCGGATCATTCCATGCTATGCAACATGGAGTGTTGACAGTATTGGCAGCTGGAAATCGTGTCCTCGTCCTTCAACCTTAGGCAATTGTT
Ca_LG_7:27671766	CACGAGGTACAACAAGAGGTGGAGCTCCATCTGCACGTATTGTTGTCTACAAATGACAGTATGTATTTTAGAGGTGCAATTTCCATCGGATCATTCCATG	G/A	CTATGCAACATGGAGTGTTGACAGTATTGGCAGCTGGAAATCGTGTCCTCGTCCTTCAACCTTAGGCAATTGTTGTGGCGGCTTGCACATTAGATAGAAT
Ca_LG_7:27671767	ACGAGGTACAACAAGAGGTGGAGCTCCATCTGCACGTATTGTTGTCTACAAATGACAGTATGTATTTTAGAGGTGCAATTTCCATCGGATCATTCCATGC	C/T	TATGCAACATGGAGTGTTGACAGTATTGGCAGCTGGAAATCGTGTCCTCGTCCTTCAACCTTAGGCAATTGTTGTGGCGGCTTGCACATTAGATAGAATG
Ca_LG_7:27671792	CCATCTGCACGTATTGTTGTCTACAAATGACAGTATGTATTTTAGAGGTGCAATTTCCATCGGATCATTCCATGCTATGCAACATGGAGTGTTGACAGTA	A/G	TTGGCAGCTGGAAATCGTGTCCTCGTCCTTCAACCTTAGGCAATTGTTGTGGCGGCTTGCACATTAGATAGAATGTTTGTGACCAAGGTCAAATTAGGAG
Ca_LG_7:27671810	GTCTACAAATGACAGTATGTATTTTAGAGGTGCAATTTCCATCGGATCATTCCATGCTATGCAACATGGAGTGTTGACAGTATTGGCAGCTGGAAATCGT	T/G	GTCCTCGTCCTTCAACCTTAGGCAATTGTTGTGGCGGCTTGCACATTAGATAGAATGTTTGTGACCAAGGTCAAATTAGGAGTTAATATTATAAATAATA
Ca_LG_7:27671816	AAATGACAGTATGTATTTTAGAGGTGCAATTTCCATCGGATCATTCCATGCTATGCAACATGGAGTGTTGACAGTATTGGCAGCTGGAAATCGTGTCCTC	C/A	GTCCTTCAACCTTAGGCAATTGTTGTGGCGGCTTGCACATTAGATAGAATGTTTGTGACCAAGGTCAAATTAGGAGTTAATATTATAAATAATAATGTTT
Ca_LG_7:28281119	NNNNNNNNNNNNNNNNNNNNNNNNNNNNNNNNNNNNNNNNNNNNTGTGGCTGCAAAAGCCCTCACAAATGCCTGTCTTTTGTGGCTGCAAAAGCCCTCC	C/A	CAAATGCCAACCTGTTTCAGGCACTTTGTGGCTGCAAAAGCCCTCACAAATGCCAACCTGTTTCAGGCACTTTGTGGCTGCAAAAGCCCTCACAAATGCC
Ca_LG_7:28592202	AGATCAATTAACAAAGTTTTTGGCCGATACAACAAGAATTGCTTACTTAAATGATAATAGATTAAGAATCTACTTATGTAATAAAGTGGATCCCCTAAGA	A/G	TTTTGAGCAGCGTTGTAGTATCATATTCCAAAGATAGCAAGTCTTTTCTTTTCTCATTAAGAAAAGTCTTTCTCAAAGATTCTATAGACATGAATATATA

Positions	-100 bp flanking sequences	SNPs within *kabuli* cultivar	+100 bp flanking sequences
Ca_LG_7:28592215	AAGTTTTTGGCCGATACAACAAGAATTGCTTACTTAAATGATAATAGATTAAGAATCTACTTATGTAATAAAGTGGATCCCCTAAGATTTTGAGCAGCGT	T/G	TGTAGTATCATATTCCAAAGATAGCAAGTCTTTTCTTTTCTCATTAAGAAAAGTCTTTCTCAAAGATTCTATAGACATGAATATATAAAACATATATTTT
Ca_LG_7:28592226	CGATACAACAAGAATTGCTTACTTAAATGATAATAGATTAAGAATCTACTTATGTAATAAAGTGGATCCCCTAAGATTTTGAGCAGCGTTGTAGTATCAT	T/G	ATTCCAAAGATAGCAAGTCTTTTCTTTTCTCATTAAGAAAAGTCTTTCTCAAAGATTCTATAGACATGAATATATAAAACATATATTTTGCATATACAAA
Ca_LG_7:28592229	TACAACAAGAATTGCTTACTTAAATGATAATAGATTAAGAATCTACTTATGTAATAAAGTGGATCCCCTAAGATTTTGAGCAGCGTTGTAGTATCATATT	T/C	CCAAAGATAGCAAGTCTTTTCTTTTCTCATTAAGAAAAGTCTTTCTCAAAGATTCTATAGACATGAATATATAAAACATATATTTTGCATATACAAAAGT
Ca_LG_7:28592240	TTGCTTACTTAAATGATAATAGATTAAGAATCTACTTATGTAATAAAGTGGATCCCCTAAGATTTTGAGCAGCGTTGTAGTATCATATTCCAAAGATAGC	C/T	AAGTCTTTTCTTTTCTCATTAAGAAAAGTCTTTCTCAAAGATTCTATAGACATGAATATATAAAACATATATTTTGCATATACAAAAGTAAAACTTTGAT
Ca_LG_7:28592260	AGATTAAGAATCTACTTATGTAATAAAGTGGATCCCCTAAGATTTTGAGCAGCGTTGTAGTATCATATTCCAAAGATAGCAAGTCTTTTCTTTTCTCATT	T/C	AAGAAAAGTCTTTCTCAAAGATTCTATAGACATGAATATATAAAACATATATTTTGCATATACAAAAGTAAAACTTTGATTAGCTAGAACTGTACACCCT
Ca_LG_7:28592269	ATCTACTTATGTAATAAAGTGGATCCCCTAAGATTTTGAGCAGCGTTGTAGTATCATATTCCAAAGATAGCAAGTCTTTTCTTTTCTCATTAAGAAAAGT	T/C	CTTTCTCAAAGATTCTATAGACATGAATATATAAAACATATATTTTGCATATACAAAAGTAAAACTTTGATTAGCTAGAACTGTACACCCTACAAAATGC
Ca_LG_7:28592295	CCTAAGATTTTGAGCAGCGTTGTAGTATCATATTCCAAAGATAGCAAGTCTTTTCTTTTCTCATTAAGAAAAGTCTTTCTCAAAGATTCTATAGACATGA	A/G	ATATATAAAACATATATTTTGCATATACAAAAGTAAAACTTTGATTAGCTAGAACTGTACACCCTACAAAATGCAGTAGGTATGCTTTCGTTGCATAGTC
Ca_LG_7:30085012	ATAGATATTAGCACCCTTTATTTGCTGCCACATTTTCAACACGCACACTCAACATGAAGGAACCTGACAATACACCTCAATGAGAAGGAACCTGGAAATA	A/G	GTATCCCTCCCCATTAATAGTCCCTTTTCTCACTCAAGCTCTCTCTATCTCTACTCTCAGCCTCTATCCTAACAGACGCTCCAACCAGCAGCAGCGAGCC
Ca_LG_7:30085070	GGAACCTGACAATACACCTCAATGAGAAGGAACCTGGAAATAGTATCCCTCCCCATTAATAGTCCCTTTTCTCACTCAAGCTCTCTCTATCTCTACTCTC	C/A	AGCCTCTATCCTAACAGACGCTCCAACCAGCAGCAGCGAGCCCCCACCCCATTGCTTGTCCACCACCACCAATGCCTTGTTTCACCCGTTGGCCCTCTCT
Ca_LG_7:30085080	AATACACCTCAATGAGAAGGAACCTGGAAATAGTATCCCTCCCCATTAATAGTCCCTTTTCTCACTCAAGCTCTCTCTATCTCTACTCTCAGCCTCTATC	C/A	CTAACAGACGCTCCAACCAGCAGCAGCGAGCCCCCACCCCATTGCTTGTCCACCACCACCAATGCCTTGTTTCACCCGTTGGCCCTCTCTCTCGGCATGA
Ca_LG_7:30085349	AAAGCTTATGGGGAGTGTAACTAACTATCACAATGTCATCTCCATAGATGAGGAGGGCTGTATATTAAGAGGTGTGGTGGCAGATTGGGAAATCTATTAG	G/A	AGAAACTGGTGCAACAGAATCTGTCGATATGGAAAGACAGGTACAAACAAAATCACTGATTTGAATTGATGAATGGAATTGGTTCAAGTGGCAGCAGTCA
Ca_LG_7:30085366	TAACTAACTATCACAATGTCATCTCCATAGATGAGGAGGGCTGTATATTAAGAGGTGTGGTGGCAGATTGGGAAATCTATTAGAGAAACTGGTGCAACAG	G/T	AATCTGTCGATATGGAAAGACAGGTACAAACAAAATCACTGATTTGAATTGATGAATGGAATTGGTTCAAGTGGCAGCAGTCAGCAGGGCTTGGATAGGA

**Positions**	**-100 bp flanking sequences**	**SNPs within *kabuli* cultivar**	**+100 bp flanking sequences**
Ca_LG_7:30514448	TTTTGAACAAAGCAGCAACGCGATCTGGGGACAGATGAAGTTGCGGCGAACAGAGAGGCGAGAGCGAACGGCCACGGAGGCAGTCAGGCAGAGGCGGCCG	G/A	CAGGCCTGCAGCGAATAGAGGAGCGATTGATTCCTGATCTGGCAGAGAAGCAGAGAAGTGATCTGAGGCGGCAGCGCGATTGTTGTGATTTCTGGAGAAG
Ca_LG_7:30615984	ATCACATTTTGTGGTCTTGACGAAGCTGCTGGAATTCATGGAGATTCAAGCTGCTCATAACGCAAGAGTAGAAGCATCTCTCAAGACTCTCCAGGCATCA	A/G	CTTACTTCTGTCGCTCAGGATGTAGCTGCAATATAAGAACATCTAGGCCTTCAAATGTCATTTGAAGATATTGCAGATATAGGCCGACAAGCAACCGAAG
Ca_LG_7:30739580	CCCAGATCGTACGCTAAGGTCCCTAAGCAATCACTTAGTGGAAAAGGAAGTGATCGAGCGATGACAACCAGGAGGTGGGCTTGGAAGCAGCCATCCTTTG	G/A	AAGAAAGCGTAATAGCTCACTGGTCTAGCTCCATGGCACCGAAAATGTATCAGGGCTCAAGTGATTCACCGAAGCGACGAGACCTTGAAAGTTGTTCAAG
Ca_LG_7:30815261	GGCATATGGACTAGAGCTCAACCGAATACTGAACAAGCGCAGCCAACTGCAGAGCAAGCTGAGCCAGTCGAAGCACATTCTGTGGTCTTAACGAAGCTGT	T/A	TGGAATTCATGGAGATTCAAGCTGCTCATAAGGCAAGGGTGGAAGCCTCTCTGAAGAAGCTCCAAGCAACTTTGAACTCCGTAGTTCAAGACGTGGATGC
Ca_LG_7:30836883	NNNNNNNNNNNNNNNNNNNNNNNNNNNNNCTTTCTAGTTCTGAATCATCTAGTCGATGCAACTCCATTTCGTGCTCCCTGAGTTTTCCAAATAGTGTTGC	C/T	TGTGGTCATTTTTGCTAGATCTTTAGATTCAGCAATAGCAGTTATTTTGGGCTGCCATTCTCTGGTTAGACACCTCATAATCTTTCCGATCTGCTGTTCA
Ca_LG_7:32204740	GGGAGGCGGCCAGGCAGTTCAGAACACCTCAATGGCTAGGCAATGAACAAAAAGAAGCGAAAACGAAGAAGGACGAAGAAACGGCGAGCAGCGACAACGG	G/A	CGGGCAGTGGCCACGGCGAGCAGCGACCACGGAGAGCAGCGACCACGAAGAGCAGCGACAACGGCGACGACCAGGATGAGCAGCGACAACGGCGACGACC
Ca_LG_7:32507631	CATATCTAAGTTTGATGGACATGCTCACCAATCTTACAATTGGTAGTTGTGATGGTGTGGAGTCCTTCCCCAATAAGGGTTTTGCGTTGCTGCCTCCCTC	C/T	CCTTACCTCTCTAACGCTAAGCAGTATGTCAAGTTTGCACACGTTGGAATGCACGGAGCTTCTCCACCTCACGTCCCTCCAACACTTAGCAATTTTGACT
Ca_LG_7:32724929	CTTATAGGAAAACTATGCTGCCAAGAAGGAATGATGCTCCAAGAGTAAATGTCAATAAGGATGACCAAATGGCGGAAGCTATGAATAACATGGCTGCTTC	C/A	TGTTGCTACCCAAACTATGGCAAAGACTCGACGGGATCAAGAAAAGAGGGAAAAAGAGATCCTTGTTGCTGAGTCAAGAGGATTAGAATATTTTCATCAT
Ca_LG_7:33464331	GCAAAGAGAAAAGTGAGAGAGGAAGAGCAAACCTTATTTTCGCATAAAACAGATCAGCTGCACTAGATTCGCGATTTCTCCTTCGTTCACCGTCGGATCA	A/G	GGCTGAAATTTGGACAGCGGGTTCGTGACTCGTGATACTTCATTCTAACCGTTTGGATCGTCAATCAGAGGTCTGAGGTGGGAGCAATTGAACTCGGACA
Ca_LG_7:35890994	ATACCACACATGCTGTTACGACCAAATGTCAATGTTCCAGCAGGCGATTGCACGGGCATTTTTCCGTCAGCACCCCATTCAACAAACGGCGTTGCTTCAT	T/C	TTATGTCATATCCACTTGTCCATGTAACTGTCATCTGCACAAGACAATAACAACAATTCCTGAAATCACTTGCATAGCTCTAATAGACATAGTGGTTCCA
Ca_LG_7:35891017	AAATGTCAATGTTCCAGCAGGCGATTGCACGGGCATTTTTCCGTCAGCACCCCATTCAACAAACGGCGTTGCTTCATTTATGTCATATCCACTTGTCCAT	T/G	GTAACTGTCATCTGCACAAGACAATAACAACAATTCCTGAAATCACTTGCATAGCTCTAATAGACATAGTGGTTCCAATGACATCAATTATTATTCCTAT
Ca_LG_7:36171779	GACCATATTTTCGGGACATGTTGATGGTAATCTTCGTTTATGGGACATTCAGACTGGAAGGCTACTGAGCGAGGTTGCTGCACATTCACTTGCCGTCACG	G/A	TCGATATCCCTATCTCGAAACGGAAATATTGCATTGACCAGTGGAAGGGATAATTTGCACAATTTGTTTGACGTGCGATCTCTTGAAGTTTGTGGCACAT

Positions	-100 bp flanking sequences	SNPs within *kabuli* cultivar	+100 bp flanking sequences
Ca_LG_7:36247658	GAGGAATAGTTATATTTATAGGAGAACAACGACACTTTGTGACAGCCAAAACAACCACAAAATTGGGTGTTTGTGGCGGCCTTTGTGGCCAGAAAAACTG	G/T	ACTTTGTGGCGGCCAAAGCAGCCATAAATGCCAACGGTACACTACGCCTTTTGTGAGGGCTTTTGCAGTCACAAACGAGTGACTTTGTGGCGGCATTTGC
Ca_LG_7:36247689	ACACTTTGTGACAGCCAAAACAACCACAAAATTGGGTGTTTGTGGCGGCCTTTGTGGCCAGAAAAACTGACTTTGTGGCGGCCAAAGCAGCCATAAATGC	C/T	CAACGGTACACTACGCCTTTTGTGAGGGCTTTTGCAGTCACAAACGAGTGACTTTGTGGCGGCATTTGCAGGCACAAATGAGTAACTTTGTGGCGGCTTT
Ca_LG_7:36247698	GACAGCCAAAACAACCACAAAATTGGGTGTTTGTGGCGGCCTTTGTGGCCAGAAAAACTGACTTTGTGGCGGCCAAAGCAGCCATAAATGCCAACGGTAC	C/T	ACTACGCCTTTTGTGAGGGCTTTTGCAGTCACAAACGAGTGACTTTGTGGCGGCATTTGCAGGCACAAATGAGTAACTTTGTGGCGGCTTTTGCACTCAT
Ca_LG_7:36247704	CAAAACAACCACAAAATTGGGTGTTTGTGGCGGCCTTTGTGGCCAGAAAAACTGACTTTGTGGCGGCCAAAGCAGCCATAAATGCCAACGGTACACTACG	G/A	CCTTTTGTGAGGGCTTTTGCAGTCACAAACGAGTGACTTTGTGGCGGCATTTGCAGGCACAAATGAGTAACTTTGTGGCGGCTTTTGCACTCATAAATGC
Ca_LG_7:36247706	AAACAACCACAAAATTGGGTGTTTGTGGCGGCCTTTGTGGCCAGAAAAACTGACTTTGTGGCGGCCAAAGCAGCCATAAATGCCAACGGTACACTACGCC	C/A	TTTTGTGAGGGCTTTTGCAGTCACAAACGAGTGACTTTGTGGCGGCATTTGCAGGCACAAATGAGTAACTTTGTGGCGGCTTTTGCACTCATAAATGCGT
Ca_LG_7:37423848	GGCATTCACATTGCTCGATGTATAATTTTAGGTTGAGGACGAAGCAGTGCTAGTCGAAGATGTGCCAACAATGAAGTCAAAAAGAAGACTTGTCTTGACA	A/T	ACACAGCTTATGCAGCAACTACTTAACCCTCCGCCAACATCAGTTCTCTGTGCAGATGTCAAGTTGCATCATGAAAGTGTGGTTTACTCTGTTGCTAGAT
Ca_LG_7:37423851	ATTCACATTGCTCGATGTATAATTTTAGGTTGAGGACGAAGCAGTGCTAGTCGAAGATGTGCCAACAATGAAGTCAAAAAGAAGACTTGTCTTGACAACA	A/G	CAGCTTATGCAGCAACTACTTAACCCTCCGCCAACATCAGTTCTCTGTGCAGATGTCAAGTTGCATCATGAAAGTGTGGTTTACTCTGTTGCTAGATTAG
Ca_LG_7:37423855	ACATTGCTCGATGTATAATTTTAGGTTGAGGACGAAGCAGTGCTAGTCGAAGATGTGCCAACAATGAAGTCAAAAAGAAGACTTGTCTTGACAACACAGC	C/G	TTATGCAGCAACTACTTAACCCTCCGCCAACATCAGTTCTCTGTGCAGATGTCAAGTTGCATCATGAAAGTGTGGTTTACTCTGTTGCTAGATTAGTGTT
Ca_LG_7:38157474	GTTTGTGGCGGCCTGAGCGGCCACAAATGACAACGTCACTTTGGAATTTTGTGGCGGCCTTTGCAGCCACAAAAGGTAATGACTCAGGCTTTTGTGGCGA	A/G	CCTTTGCAGCCACAAAAGGTAACGTCACTTTGGGATTTTGTGGCGGCCTTTACCCTCACAAATGGTTGTATTTTGTGGCTGAAAAACCTCACAAAAATC
Ca_LG_7:42029368	GAAATCGTCGGAAATGCAGAAGATGAACTTGCAGCATCAGTAGTTGCTGACTGTAGACTTGAGAGAATCTCATATAATACGCTCTAGAATGAACAAGAGA	A/C	AAATTAGTTTTTGTTCTTCAAATAACACTGTCATAATATAGCTGAATCATGCAGCCATATATAAAACAAGGTCACCAAAAACAAAAAACGAAACAAAAAA
Ca_LG_7:42029379	AAATGCAGAAGATGAACTTGCAGCATCAGTAGTTGCTGACTGTAGACTTGAGAGAATCTCATATAATACGCTCTAGAATGAACAAGAGAAAATTAGTTTT	T/C	TGTTCTTCAAATAACACTGTCATAATATAGCTGAATCATGCAGCCATATATAAAACAAGGTCACCAAAAACAAAAAACGAAACAAAAAACTAACAATAAT
Ca_LG_7:42056387	AATTAATATCTACAATTTTATTATTTTTATTATTTTTTCTCAAACCCTATTAACTTCAACAGTATCATTTTGTTAGTTTTTCATAAGCTGCATTATGGGC	C/G	ATAAAATACAAATACAAGTATATTTCTTGTGGGTCAGGCTTNNNNNNNNNNNNNNNNNNNNNNNNNNNNNNNNNNNNNNNNNNNNNNNNNNNNNNNNNNN

Positions	-100 bp flanking sequences	SNPs within *kabuli* cultivar	+100 bp flanking sequences
Ca_LG_7:42143183	ACTCTGTTACAGCTTGGTTCCGTACCTTCTTCAGATCTTACTCAGTCAAGCAGCAGCAGGAGGCAGAGCTGCAAAACAACCGAAGATGATCCATCAAGTA	A/G	CTTCAAGAGACATTACTTCTTTCAAGCACAAGTCAGACCTCCAATATACGCCTCACAACTCAACATCCCCTGCTTCCCACCAAAAAGCAGTAAACGGCAT
Ca_LG_7:42169378	AGTGAGGTTGATGTTATTAATTTCAATTTGAACGGAAAATCAAAGATGCAGCGATAATTGTAGTGGAGAAATTAAGGGATTGTTTCTCTCTCTCTCTCTC	C/A	TCTATATGTGTGTGTGTGTAAGGCAGGCAGGCAGGCAGGCAGCCATGTTTTGTCCTCACCACTATCTGACACACTGTTCCCCTCTTTCTCTCTTTCTCTC
Ca_LG_7:42169380	TGAGGTTGATGTTATTAATTTCAATTTGAACGGAAAATCAAAGATGCAGCGATAATTGTAGTGGAGAAATTAAGGGATTGTTTCTCTCTCTCTCTCTCTC	C/A	TATATGTGTGTGTGTGTAAGGCAGGCAGGCAGGCAGGCAGCCATGTTTTGTCCTCACCACTATCTGACACACTGTTCCCCTCTTTCTCTCTTTCTCTCTC
Ca_LG_7:42169384	GTTGATGTTATTAATTTCAATTTGAACGGAAAATCAAAGATGCAGCGATAATTGTAGTGGAGAAATTAAGGGATTGTTTCTCTCTCTCTCTCTCTCTATA	A/G	TGTGTGTGTGTGTAAGGCAGGCAGGCAGGCAGGCAGCCATGTTTTGTCCTCACCACTATCTGACACACTGTTCCCCTCTTTCTCTCTTTCTCTCTCACTC
Ca_LG_7:42170111	GATTCATCATCATAATCAACTACACAATGTTATAACATCTTCCACAGTGGCAGTGGCAGTGGCAGTAGCAGCACCTCATAACAACCCTTGTTCAAATGGG	G/C	ACAACAGTTGGAGAGGTAGATGAGTCAAGCTACAAGAAAGTGGATGTGGTAGTTGTAAGGTACAGAGAATGCCTAAAGAACCATGCTGCTGCAATGGGAG
Ca_LG_7:42192196	TACAACAGCTCGACGATGCTGCTGGTGATGTTTCTGGACTTGCTGTCAAATGGTACTACATCTTCTATCTCTCTCATTAATATTTTTTAATCTATATCAT	T/A	TAACTTTTTTAATCAAATTTTGCAGCCTTGCTCCATTAGTTAGGAAGATGAGTGAGTCAAGGGTTGTGGAAATGAGCAGTCAACTCTGTGACAAAATACT
Ca_LG_7:42845017	AAACACAAATAATAGAAAATATTATAATTTTAAGATGCAGCAGCTATAAAGATTAATAAACTTTGAGACATAAAATATGTATCTCTCAAATTCAAATTGA	A/G	TGTCTCGCAAAGCTGCTATAAAGAATGTAAATACTATTTTTTCGGGGCAAGTTTACAACTATTAAGCATTGTGTATTATTAAAATATTGAATATCACTAT
Ca_LG_7:42883120	ACATTTGAAGTCTCTCATGGGAAAAGTGCAACATTTTGGTGTTCCATCTCTTGGCGGTTGCTTCGATAGCTGCCGCGACCATACACAAGGCTCGGGATTC	C/T	GGGACGAGGATATGGAACCTCAGTGATAAGCCAGTGGAGCTGCAAATAAGGGTAGGATCAATACTAAAGAAGATTTATACATTGAAGCCAGGGTCTTCAA
Ca_LG_7:43536774	AGTAGATACAATAGACTTGGTTGATCTGAAAGATGTAAAGTTTGTCCACTTATAAGATGTTTGTTCATTTAAAAAACTCAAATTTAAATTATGTTATTGG	G/C	TGTTGGGTTAGCCGTTGGACTTGCTTCTATTGGACCTTTAATTGGTCAAAACACAACTGCAGGATAAGTTGTAGATTACCATTCATAAAATTTAATATTT
Ca_LG_7:43536777	AGATACAATAGACTTGGTTGATCTGAAAGATGTAAAGTTTGTCCACTTATAAGATGTTTGTTCATTTAAAAAACTCAAATTTAAATTATGTTATTGGTGT	T/C	TGGGTTAGCCGTTGGACTTGCTTCTATTGGACCTTTAATTGGTCAAAACACAACTGCAGGATAAGTTGTAGATTACCATTCATAAAATTTAATATTTACT
Ca_LG_7:43536784	ATAGACTTGGTTGATCTGAAAGATGTAAAGTTTGTCCACTTATAAGATGTTTGTTCATTTAAAAAACTCAAATTTAAATTATGTTATTGGTGTTGGGTTA	A/G	GCCGTTGGACTTGCTTCTATTGGACCTTTAATTGGTCAAAACACAACTGCAGGATAAGTTGTAGATTACCATTCATAAAATTTAATATTTACTTTATAAA
Ca_LG_7:43536812	AGTTTGTCCACTTATAAGATGTTTGTTCATTTAAAAAACTCAAATTTAAATTATGTTATTGGTGTTGGGTTAGCCGTTGGACTTGCTTCTATTGGACCTT	T/G	TAATTGGTCAAAACACAACTGCAGGATAAGTTGTAGATTACCATTCATAAAATTTAATATTTACTTTATAAAATTTATCGTCATTAAAATATATATAAAA

**Positions**	**-100 bp flanking sequences**	**SNPs within *kabuli* cultivar**	**+100 bp flanking sequences**
Ca_LG_7:43536813	GTTTGTCCACTTATAAGATGTTTGTTCATTTAAAAAACTCAAATTTAAATTATGTTATTGGTGTTGGGTTAGCCGTTGGACTTGCTTCTATTGGACCTTT	T/G	AATTGGTCAAAACACAACTGCAGGATAAGTTGTAGATTACCATTCATAAAATTTAATATTTACTTTATAAAATTTATCGTCATTAAAATATATATAAAAG
Ca_LG_7:43536825	ATAAGATGTTTGTTCATTTAAAAAACTCAAATTTAAATTATGTTATTGGTGTTGGGTTAGCCGTTGGACTTGCTTCTATTGGACCTTTAATTGGTCAAAA	A/G	CACAACTGCAGGATAAGTTGTAGATTACCATTCATAAAATTTAATATTTACTTTATAAAATTTATCGTCATTAAAATATATATAAAAGATGTTTGACCTC
Ca_LG_7:43540275	ACATTGTTGCTAAATCTGGTGCTTTTCTAAAATTTTACACTATCTCAGGTTTTGGTCACACATTGTAGCTGCTTATGCATTTACCTTCTGGACATGCTAT	T/A	ATTTTGATGAGAGAGTATGAAAAAGTTGCCTCAATGAGATTACAATTTGTTGCAGCAGAAAAGCGTCGTCCTGATCAATTTTCGGTAATATTTTGTTCTC
Ca_LG_7:43615860	TGTAACTGTATGGTAATAATATTGATGGAGAGGCAGGGAGATAGTCAAAAACTGTAAGCAACTAAGAATAGACTCACATTTGTACCACGAAGCATATGAG	G/C	CAGAAGTAGAGACACCAGCTGCAACAGACAGGACCAATGGCAAGCCAGAAGAGTAAAGTAGCCAAAATAAATATGTGGAGAAGGCAAGAGAGCAAGACAA
Ca_LG_7:43615877	AATATTGATGGAGAGGCAGGGAGATAGTCAAAAACTGTAAGCAACTAAGAATAGACTCACATTTGTACCACGAAGCATATGAGCAGAAGTAGAGACACCA	A/G	GCTGCAACAGACAGGACCAATGGCAAGCCAGAAGAGTAAAGTAGCCAAAATAAATATGTGGAGAAGGCAAGAGAGCAAGACAAGAACAGGTATGGTACCA
Ca_LG_7:43615878	ATATTGATGGAGAGGCAGGGAGATAGTCAAAAACTGTAAGCAACTAAGAATAGACTCACATTTGTACCACGAAGCATATGAGCAGAAGTAGAGACACCAG	G/A	CTGCAACAGACAGGACCAATGGCAAGCCAGAAGAGTAAAGTAGCCAAAATAAATATGTGGAGAAGGCAAGAGAGCAAGACAAGAACAGGTATGGTACCAT
Ca_LG_7:43615910	ACTGTAAGCAACTAAGAATAGACTCACATTTGTACCACGAAGCATATGAGCAGAAGTAGAGACACCAGCTGCAACAGACAGGACCAATGGCAAGCCAGAA	A/T	GAGTAAAGTAGCCAAAATAAATATGTGGAGAAGGCAAGAGAGCAAGACAAGAACAGGTATGGTACCATACCAGCTGCTGTATTGAGTAGGTTGGAGGATC
Ca_LG_7:43617812	AAAGATAGAAATCCAGAGTACATTATAGGGAGAAAGAACTACAAAGCCTTTTCATAAAGAAAAAAATAGAAGTGGGAAGTCTATCACACTAATTGGTTTG	G/A	CACTCACTCACCAAAGAGATATAAGCAAGCCAGCAAGCTGCCTAGAACCCATACGCTCAAAAGCTTTTCCTTCTTCCAAGGCCTTCCCTATTGTATCCAG
Ca_LG_7:43617821	AATCCAGAGTACATTATAGGGAGAAAGAACTACAAAGCCTTTTCATAAAGAAAAAAATAGAAGTGGGAAGTCTATCACACTAATTGGTTTGCACTCACTC	C/T	ACCAAAGAGATATAAGCAAGCCAGCAAGCTGCCTAGAACCCATACGCTCAAAAGCTTTTCCTTCTTCCAAGGCCTTCCCTATTGTATCCAGCCACCACTG
Ca_LG_7:43643955	TTGCTTCTTATCTTCTTCCCCATGTACCCCAGCAGGTTCTCGCAGCACTTAGTGTTGAAATTCTCAAAGCTGCTGGTGAACGCAGTGGTGGGTCTCTTGA	A/T	TGGTGTAGCATTTCTCCTCGAATCTGACTTCTTAGGTGATCCTGCAGCTACTTATGCTGTGGCAGACATTATTGCTAAATTGGAGGATGAGGCTGTTGCT
Ca_LG_7:44887538	ATTATGTGTTTTTGCAGCTGTTGTTGGTGATGGATTTATTGCACAAGATATCACATTTAGGAACACTGCTGGTGCAAACAACCATCAAGCGGTTGCATTG	G/C	CGTTCTGGATCAGACTTATCTGTGTTTTACAGATGTAGCTTTGAAGGTTATCAAGACACACTATATGTGCATTCAGATAGACAATTCTATAAGGAATGTA
Ca_LG_7:44887547	TTTTGCAGCTGTTGTTGGTGATGGATTTATTGCACAAGATATCACATTTAGGAACACTGCTGGTGCAAACAACCATCAAGCGGTTGCATTGCGTTCTGGA	A/C	TCAGACTTATCTGTGTTTTACAGATGTAGCTTTGAAGGTTATCAAGACACACTATATGTGCATTCAGATAGACAATTCTATAAGGAATGTAACATTTATG

Positions	-100 bp flanking sequences	SNPs within *kabuli* cultivar	+100 bp flanking sequences
Ca_LG_7:44887553	AGCTGTTGTTGGTGATGGATTTATTGCACAAGATATCACATTTAGGAACACTGCTGGTGCAAACAACCATCAAGCGGTTGCATTGCGTTCTGGATCAGAC	C/T	TTATCTGTGTTTTACAGATGTAGCTTTGAAGGTTATCAAGACACACTATATGTGCATTCAGATAGACAATTCTATAAGGAATGTAACATTTATGGCACAG
Ca_LG_7:44887554	GCTGTTGTTGGTGATGGATTTATTGCACAAGATATCACATTTAGGAACACTGCTGGTGCAAACAACCATCAAGCGGTTGCATTGCGTTCTGGATCAGACT	T/C	TATCTGTGTTTTACAGATGTAGCTTTGAAGGTTATCAAGACACACTATATGTGCATTCAGATAGACAATTCTATAAGGAATGTAACATTTATGGCACAGT
Ca_LG_7:44887559	TGTTGGTGATGGATTTATTGCACAAGATATCACATTTAGGAACACTGCTGGTGCAAACAACCATCAAGCGGTTGCATTGCGTTCTGGATCAGACTTATCT	T/A	GTGTTTTACAGATGTAGCTTTGAAGGTTATCAAGACACACTATATGTGCATTCAGATAGACAATTCTATAAGGAATGTAACATTTATGGCACAGTTGACT
Ca_LG_7:44887562	TGGTGATGGATTTATTGCACAAGATATCACATTTAGGAACACTGCTGGTGCAAACAACCATCAAGCGGTTGCATTGCGTTCTGGATCAGACTTATCTGTG	G/A	TTTTACAGATGTAGCTTTGAAGGTTATCAAGACACACTATATGTGCATTCAGATAGACAATTCTATAAGGAATGTAACATTTATGGCACAGTTGACTTCA
Ca_LG_7:44887570	GATTTATTGCACAAGATATCACATTTAGGAACACTGCTGGTGCAAACAACCATCAAGCGGTTGCATTGCGTTCTGGATCAGACTTATCTGTGTTTTACAG	G/A	ATGTAGCTTTGAAGGTTATCAAGACACACTATATGTGCATTCAGATAGACAATTCTATAAGGAATGTAACATTTATGGCACAGTTGACTTCATATTTGGT
Ca_LG_7:44887601	CACTGCTGGTGCAAACAACCATCAAGCGGTTGCATTGCGTTCTGGATCAGACTTATCTGTGTTTTACAGATGTAGCTTTGAAGGTTATCAAGACACACTA	A/G	TATGTGCATTCAGATAGACAATTCTATAAGGAATGTAACATTTATGGCACAGTTGACTTCATATTTGGTAACGCTGCTGCAGTGTTCCAAAATTGTAATA
Ca_LG_7:44941905	GTTCACAAAAAACAGAAAAACTAATTTTGATAACTATTATGTTCATCCAACTCTTTTATGTGAAAGCTGAGAAGCAGCTTAAGAGTTAGTTGGTAGAAGT	T/A	GATTATAATTGGTTGGAATGAATAATGTCTTATTCAAAGGAAAAACATATATAGAAACGTAATTATTTCATCTCTATATATACGTAGAAAATATGAATTA
Ca_LG_7:44941931	TTGATAACTATTATGTTCATCCAACTCTTTTATGTGAAAGCTGAGAAGCAGCTTAAGAGTTAGTTGGTAGAAGTGATTATAATTGGTTGGAATGAATAAT	T/C	GTCTTATTCAAAGGAAAAACATATATAGAAACGTAATTATTTCATCTCTATATATACGTAGAAAATATGAATTAACATGTTTTGAGATGGGAAATATAGC
Ca_LG_7:44942016	GTTGGAATGAATAATGTCTTATTCAAAGGAAAAACATATATAGAAACGTAATTATTTCATCTCTATATATACGTAGAAAATATGAATTAACATGTTTTGA	A/C	GATGGGAAATATAGCTGCTCAAATAAGTTTTCTTTATTCAGATAGAGCCCATATCATGTAATTAACCAAACATCAAACAAGTTCACTATATGTCTCTAAT
Ca_LG_8:21335	TATAGCACAAGAAAATAGATTCTTTTTTTTGAGACAGTAAGCCATTCCATTAGATAATACAAACAGGAACAGCAGCAGAAACATTTGGGATATAACCCAG	G/A	CCATGTTTTGGTGCCAATAGTTATTGCTAAATATAACCGAAGCAATCCTAAAATTACTTAAAAGATCAATGCAGTCCTGAATTATTGGATCAACAGCTGC
Ca_LG_8:21393	ACAAACAGGAACAGCAGCAGAAACATTTGGGATATAACCCAGCCATGTTTTGGTGCCAATAGTTATTGCTAAATATAACCGAAGCAATCCTAAAATTACT	T/C	TAAAAGATCAATGCAGTCCTGAATTATTGGATCAACAGCTGCAATGCACCGTTTTCCTTGCAAACATCCCACCACCACCAGTTTGTCTGATTGAATTTCT
Ca_LG_8:106826	CAATTTGATACCGTAAATGATCTATACCGTTGATTCAAAATCAAACGATTCAGATTACACGATGAATTTTATCCGAATAACTGAGCTGCAATATTGTGTC	C/A	TAATTCAAAGCCTGTATATTTTCATTATCAACTCGAAGGAGGAGGATAGCCACTGGTATCTGACCATGTTGAAGGTGGTAAACCCGACCCACCACCAACA

**Positions**	**-100 bp flanking sequences**	**SNPs within *kabuli* cultivar**	**+100 bp flanking sequences**
Ca_LG_8:107418	TTTTGCTCTGCTACCTCTGAATTTCAAAGCAGCTTCATCGTAAGCTCTTGCTGCAGCTTCAGCAGTTTCGAATGTACCAAGCCATACTCTTGCTGCTTTA	A/G	TGTGGATCTCTAATCTCTGCTGCCCATTTTCCCCATGGTCTCTGTCTCACTCCTCTGTATCTTCTTCTTCTCTCTCCACTTTCTTCTTCATTTGACTCTC
Ca_LG_8:549667	TATCATTGGCCCAGCCAACCATAGTCGTTAGTTCAAAGTATAAAAAAGATTAGTGATGATTTTACATGTTTCCGTGGCACTATATTTGAAAATGGAATAC	C/A	ATGCAGCATTTGCTTCTCATAAGACTAATGAAGCATAATTTTTCAAAGGAGCGTACTATGTACGCGTGGCAGTTGTTCCAGGCTCCACTCGTGACAAACT
Ca_LG_8:933265	TGATGAAACAATAAAACTTATTTTCGATGAACCAAAACATGCAAGCAGAAAATGTATTGTTACTTTCATACATGGTTTGGCTGCATAACTTTTTTAAGAA	A/C	CACAAATTAAAGAAAATGTTTCCGTTGAAAGAAGCATCTTCTGAATCCATAAACAATTCTTGCAAAAGAAACATTTTGACGCACAAATTTGACACTAAAT
Ca_LG_8:1083826	AATGGTCAAGATGGGGGAGCTCACTGGTCACACTTCCAGAGTTCTTTACATGGCTCAGAGTCCAGATGGTTGCACAGTGGCATCAGCAGCGGCCGATGAA	A/G	ACACTGAGGTTTTGGAATGCTTTTGGCACTCCAGAAGTAGTAACCAAAGCTGCTCCAAAAGCTAGAGCTGAGCCATTTTCACATCTTAGTCGCATTCGAT
Ca_LG_8:1683247	ATAATCAAACAACTTTGCTATCCCTTCAGTGCCACACTTGTTTGCAGTGATAGGAAATAAAAAATTCATGCATTGCTTACAGAAGCAGCAAACATATTTC	C/A	AATTCTATTAGTAACTCAGCTAGTATAACACATAGAGATAGTTTAATTTCGCTTGAATGCGATGACAATTGCAGAGCAGTTATCTTTGCAGCGACGCTCA
Ca_LG_8:2318046	TATCTGCGCAGCTATCTCCTTAGAGTGTTGAGACAAGACATAGGTGCAACCTCTAGATGGACGAGAATATGCGATTAGACTTGCGACACCTGGTCTATAT	T/C	AGCGAGGATCTATCTCCAACACCATAAATACGTTCTTTATTCTTTCCCCCAACAGATTGGACCCATATGTCTAAGCCAGCTTCTGCATTAACCACTTGGC
Ca_LG_8:2318070	GTGTTGAGACAAGACATAGGTGCAACCTCTAGATGGACGAGAATATGCGATTAGACTTGCGACACCTGGTCTATATAGCGAGGATCTATCTCCAACACCA	A/G	TAAATACGTTCTTTATTCTTTCCCCCAACAGATTGGACCCATATGTCTAAGCCAGCTTCTGCATTAACCACTTGGCTACAACTTCTAGATGATTCCCTAC
Ca_LG_8:2318080	AAGACATAGGTGCAACCTCTAGATGGACGAGAATATGCGATTAGACTTGCGACACCTGGTCTATATAGCGAGGATCTATCTCCAACACCATAAATACGTT	T/C	CTTTATTCTTTCCCCCAACAGATTGGACCCATATGTCTAAGCCAGCTTCTGCATTAACCACTTGGCTACAACTTCTAGATGATTCCCTACCTTTGAAGGC
Ca_LG_8:2318083	ACATAGGTGCAACCTCTAGATGGACGAGAATATGCGATTAGACTTGCGACACCTGGTCTATATAGCGAGGATCTATCTCCAACACCATAAATACGTTCTT	T/C	TATTCTTTCCCCCAACAGATTGGACCCATATGTCTAAGCCAGCTTCTGCATTAACCACTTGGCTACAACTTCTAGATGATTCCCTACCTTTGAAGGCCTG
Ca_LG_8:2318084	CATAGGTGCAACCTCTAGATGGACGAGAATATGCGATTAGACTTGCGACACCTGGTCTATATAGCGAGGATCTATCTCCAACACCATAAATACGTTCTTT	T/G	ATTCTTTCCCCCAACAGATTGGACCCATATGTCTAAGCCAGCTTCTGCATTAACCACTTGGCTACAACTTCTAGATGATTCCCTACCTTTGAAGGCCTGT
Ca_LG_8:3905321	AGGAAACAATGCCTCTAAGAAGGAACGACGCTGCAAGAGTCAACATCAACAGGGACGATCAAATGGCTGAAGCAATGAATAATATGGCTGCTTCTGTTGT	T/C	TGCACAGACAACTGCAAAGACTTTGCGGGATCTGGAGAAGAGGGAAAGAGAGATCCGTGCTGCTGAATCTAGGGGATTGGAAGATTTTCGTCATTACGAT
Ca_LG_8:3905332	CCTCTAAGAAGGAACGACGCTGCAAGAGTCAACATCAACAGGGACGATCAAATGGCTGAAGCAATGAATAATATGGCTGCTTCTGTTGTTGCACAGACAA	A/G	CTGCAAAGACTTTGCGGGATCTGGAGAAGAGGGAAAGAGAGATCCGTGCTGCTGAATCTAGGGGATTGGAAGATTTTCGTCATTACGATCCTCCAAAGTT

**Positions**	**-100 bp flanking sequences**	**SNPs within *kabuli* cultivar**	**+100 bp flanking sequences**
Ca_LG_8:4297461	TAAGATGCGCTTCGTTATGCAGCGAAGAGAATACAAGCCCTGTTTCTCAGGATCAGACCTTAAGCAGTTGTGCCTCAGAGGATGATATGAATTCTTCTCA	A/G	TGAGCTGCGTCGAGAGGCATCTTCAAGTTTGAGTCAGGACGATTCTACAGCTCTGAAGTTAAGTGGAAAATCAATATCTAGTAGAGGTACCGCTACGGAT
Ca_LG_8:4369258	TGATAGCACTGCCCTGAAAAAGGTACCATTACTACTTCCGTCGCTACTAGAAGCATTATTTCCCGCAGTTCGAATTTTTGTTTCATCACCATCATCTAAC	C/T	CTAGACACATATTCTGCCATTTGATCCCATTCTCCCATGTTCCAAGCAGCATTAGCAGCCTAAACCATAAAACAATAGTAACATAAACACGTGAAGTGGA
Ca_LG_8:4369261	TAGCACTGCCCTGAAAAAGGTACCATTACTACTTCCGTCGCTACTAGAAGCATTATTTCCCGCAGTTCGAATTTTTGTTTCATCACCATCATCTAACCTA	A/G	GACACATATTCTGCCATTTGATCCCATTCTCCCATGTTCCAAGCAGCATTAGCAGCCTAAACCATAAAACAATAGTAACATAAACACGTGAAGTGGAAAT
Ca_LG_8:4369288	ACTACTTCCGTCGCTACTAGAAGCATTATTTCCCGCAGTTCGAATTTTTGTTTCATCACCATCATCTAACCTAGACACATATTCTGCCATTTGATCCCAT	T/C	TCTCCCATGTTCCAAGCAGCATTAGCAGCCTAAACCATAAAACAATAGTAACATAAACACGTGAAGTGGAAATTGACCATCACACATAAAAAGAGTATAT
Ca_LG_8:4369297	GTCGCTACTAGAAGCATTATTTCCCGCAGTTCGAATTTTTGTTTCATCACCATCATCTAACCTAGACACATATTCTGCCATTTGATCCCATTCTCCCATG	G/A	TTCCAAGCAGCATTAGCAGCCTAAACCATAAAACAATAGTAACATAAACACGTGAAGTGGAAATTGACCATCACACATAAAAAGAGTATATAACCCACCA
Ca_LG_8:4961561	TTGGTGGGACACCCCAACCAGTGCATTGAAGTCCACCAGTGCAATCACCTGTTTGGCAGCGGCCACGACCGCTGCCATCGAAATTGCAACCAGTACGACT	T/C	CCAAATACGAGCCATTGAAGTGCCAGCGTTAACCCAAAGATTCCATGTTTGACCGCGGTCAAGGCGACGACCACCGCCCGGACTAGCAGCTGCCCAAACG
Ca_LG_8:5053713	GGGATCTTCATTAACACACTGTTGATCACGCTGCTTGTTAATTTCATCTTCAGCTGCAATTAATTCAGCAGCTTTCATAGCCGCTTTTCTTATCTCCACT	T/C	GCATCTGTCGATTCAGGCAACGTTAACCGCCATGCTGAGTCAGCAAAGTTGAGACAAGCTGATGTTCCACGAAGAGTGAGTGAGGCAAGGTCGTGTGCAC
Ca_LG_8:6691873	TTTAGCATAGATCTAGCCATCTAGGGTTAGTGGTAAGGTAGTTCTTAGGTAAAAGAGATTGAGAGTCCCATAAACATTAGCTGCCATCTTGTTTTTTTGA	A/G	TAGTATTTTTAATCTGTTTTCATCCATATGTAGGATGTTGAGCTGAACCATGTTATATTGCGTACAATTTGTTTGTGCTGCCTTTTGCTATTATTTAGTA
Ca_LG_8:7345832	TTTTTTTAAGGCATTACCGGATTGTTAGCAGCCACACCACCATCTACGAGATTGTATTCTTTGATGACTTTCCCTTGTTCATCTTTTTTTTCGAAATAGT	T/A	GAGCTGGCAAATATGTTGGAGCTGCCGACGTGGCTATGCAAATATCTGACAGTGGTACATCGAAACATGGATCACATCCTATCTGCATAACATCGTTGAA
Ca_LG_8:7465932	GTTCTGACTACTGTTACTGCGGCTACACAGATGGTGATTAGTGACCTTCAACCACCACCGTCTCAACATCCAATCTTGGAAGCTCAGCAATTAGCTGCTG	G/C	CTGTTGTTGCAAGAGAGCAACAAGAGATGTTTAGAAGTTTCGAACATCAACAACAGCAGCAAGAGTTTTTGAGGTTCAGTGGTGGACTTGATGTGGATTC
Ca_LG_8:7534257	ATCTATTTTCCTCATCGATAAACAAAAGCATAATATTCCGATCCAAACTACTATGCAGTTCAGTGCACTAGGTTCTATATTCTTTAACTTAACCAATTTA	A/C	ACAGTGACAGAACTGAATTTTCTGTGTATGCAGAACCAGACTGAAACTTGGTCTTAGTTCTTCTGAAGCAAAGCCACATGCCAGTGGCTTGATTCAACAA
Ca_LG_8:7534262	TTTTCCTCATCGATAAACAAAAGCATAATATTCCGATCCAAACTACTATGCAGTTCAGTGCACTAGGTTCTATATTCTTTAACTTAACCAATTTAACAGT	T/C	GACAGAACTGAATTTTCTGTGTATGCAGAACCAGACTGAAACTTGGTCTTAGTTCTTCTGAAGCAAAGCCACATGCCAGTGGCTTGATTCAACAACACAA

Positions	-100 bp flanking sequences	SNPs within *kabuli* cultivar	+100 bp flanking sequences
Ca_LG_8:7534285	CATAATATTCCGATCCAAACTACTATGCAGTTCAGTGCACTAGGTTCTATATTCTTTAACTTAACCAATTTAACAGTGACAGAACTGAATTTTCTGTGTA	A/G	TGCAGAACCAGACTGAAACTTGGTCTTAGTTCTTCTGAAGCAAAGCCACATGCCAGTGGCTTGATTCAACAACACAAATACTGATCCGATAACACAGATA
Ca_LG_8:8145459	GTGGATGATACCGTTGCATTTTGCATTTGCGTTACAATTTCCTCTCAATAGGTTTCTACAGAGCCAGCTTAAAACCGCTGCTATAGCTTGGGTGTCTTTC	C/G	GTGGCACTTTTGGTTCATGTTTTTGTTAGTTGGTTGTTTGTGTTTAAGTTTCAGTTTGGTGTTATTGGAACCGCTGCTACTTTGAATTTCTCTTGGTGGG
Ca_LG_8:8314641	TCAACCAAATTTCTCTCCGTTGATCTTAAACCTCTTCCCAAAATCTTCAATAACATCAGCCAACACAACATTTTACCTCATTGTGGCAGCCACGTATTTG	G/A	TTTCTGATAATGACACTCTAGTCATCTATCACCTTCTTCATTGCAAGAAGATAAATATGCCCTACCTGATAATCCAGCATATGATTACTGCAATCCAAAA
Ca_LG_8:8314655	CTCCGTTGATCTTAAACCTCTTCCCAAAATCTTCAATAACATCAGCCAACACAACATTTTACCTCATTGTGGCAGCCACGTATTTGTTTCTGATAATGAC	C/T	ACTCTAGTCATCTATCACCTTCTTCATTGCAAGAAGATAAATATGCCCTACCTGATAATCCAGCATATGATTACTGCAATCCAAAAGGATTGTAAGAGAA
Ca_LG_8:8314656	TCCGTTGATCTTAAACCTCTTCCCAAAATCTTCAATAACATCAGCCAACACAACATTTTACCTCATTGTGGCAGCCACGTATTTGTTTCTGATAATGACA	A/G	CTCTAGTCATCTATCACCTTCTTCATTGCAAGAAGATAAATATGCCCTACCTGATAATCCAGCATATGATTACTGCAATCCAAAAGGATTGTAAGAGAAA
Ca_LG_8:8314661	TGATCTTAAACCTCTTCCCAAAATCTTCAATAACATCAGCCAACACAACATTTTACCTCATTGTGGCAGCCACGTATTTGTTTCTGATAATGACACTCTA	A/G	GTCATCTATCACCTTCTTCATTGCAAGAAGATAAATATGCCCTACCTGATAATCCAGCATATGATTACTGCAATCCAAAAGGATTGTAAGAGAAACATCG
Ca_LG_8:8314697	CAGCCAACACAACATTTTACCTCATTGTGGCAGCCACGTATTTGTTTCTGATAATGACACTCTAGTCATCTATCACCTTCTTCATTGCAAGAAGATAAAT	T/C	ATGCCCTACCTGATAATCCAGCATATGATTACTGCAATCCAAAAGGATTGTAAGAGAAACATCGTTCCTTATGACATGGTTTTCTCAAAATAATTCAGGC
Ca_LG_8:8314709	CATTTTACCTCATTGTGGCAGCCACGTATTTGTTTCTGATAATGACACTCTAGTCATCTATCACCTTCTTCATTGCAAGAAGATAAATATGCCCTACCTG	G/A	ATAATCCAGCATATGATTACTGCAATCCAAAAGGATTGTAAGAGAAACATCGTTCCTTATGACATGGTTTTCTCAAAATAATTCAGGCATTTTCAGATTC
Ca_LG_8:8314716	CCTCATTGTGGCAGCCACGTATTTGTTTCTGATAATGACACTCTAGTCATCTATCACCTTCTTCATTGCAAGAAGATAAATATGCCCTACCTGATAATCC	C/T	AGCATATGATTACTGCAATCCAAAAGGATTGTAAGAGAAACATCGTTCCTTATGACATGGTTTTCTCAAAATAATTCAGGCATTTTCAGATTCCTCTATT
Ca_LG_8:9386225	TCTTGAGATCAAAACAAAGTAAAACAACATGGTTCCAACAATTTGTTCCAACAATGAATGTGGTTCCTACGATGCTGCTGGTATCAAGAACATTTGTGGG	G/A	AAGTGTTTTAGAGAATGGTTCGACGGCACCACTAAATCAAATAATGAAGAACATGTTTTGGAAAAACCCGTATTTGCACCGTTTTGTTCGAATGAAAAAT
Ca_LG_8:10398083	AAAAAATTAACACCAATTGCAGCAACAAGAATTTTTAATACAGGACGAGTAGGTGCAATTAGCAATTCCTTCCACACCCCCTTATTCAAATTTTTGTTAG	G/A	AATTTGAAGCTGCTTGCGAAATTTCACTAAGTCTTAATTCAGCTTCACCTTTATTCTCCGAAGTTCTAATCAAAACTTGTTTAGCTTCATCAAATTTACC
Ca_LG_8:11277186	TATTTTGACAGCTTGAATAGGTGTGTGTGCGCACGTGTGACACTGACAGATAATATAGATATACCTCCGAAGGTGCAGCCCCCCATATGAAGACACGCAA	A/G	GCGAAACTGAGGAAGTCCATAAGCTCCAGCAGCCATAATTCCCAAGCGACTTTGATAGTTCATTTGAACAAGACGACCCAATGCATATCTTCCAAGAAAT

Positions	-100 bp flanking sequences	SNPs within *kabuli* cultivar	+100 bp flanking sequences
Ca_LG_8:12073505	TGAAAACAATGACGCCATCCTAGTTTCGCTATTAGGAAAGTAG<br>TTGTCGCTATTATAGTTTCGACTAGCAAATGTAGCAGCAATAA<br>TACTTAAGCTGGTG	G/T	CTCTACAATCATTTCCTTTTTTATTTGTAAGCAACATGTCCAC<br>TACTGCAACGCTAAAGCTATCCTCTATTTCACACAATGGATT<br>ATTGGCGTTCTCTAT
Ca_LG_8:12879270	ACATTCCACGTCACCTTCACCACCTCCTCCAAGCCCTCACTCT<br>TTTCTGCAATACAACCATCGCAGCCAACACTAAAACGAAACAC<br>GCCAGGGGGATCCG	G/A	CCACCATTTCATCCGCGGTTCTCGATGCAGCGGCTACAACG<br>CTAGAGTCTCGCTTGGAAGAAATCGAAACCGCAAAGATGAT<br>ATCGCCGAGGGATAGGAT
Ca_LG_8:13423557	CGCCGAAACCGCGAGTCCCAACGACGAAGGAAAAACGTAAAT<br>CAACGACGTCGTTTGAATTAAAATCCCCATAGCAGCAACAGTA<br>GCAGTGGGGTCCACA	A/C	AGTACCCCACATAAAACAATAACTATCTCATACCACCACCAT<br>TCTAAACAAACCGAAACACAGCTCGGCGCAGCGAGCCGAAT<br>CAACGGCTCCCAGCCGG
Ca_LG_8:13526876	AAGATAGCTCTTTTACAAGAGTTCCTTGAAGATTTGCATGGTT<br>TAGATCTATGCCTGCAACAACTACTGAACTAGAAGCTGATGCA<br>GCAGCAGTAGCAGC	C/A	AGAAGAAGATGAAGACATTCCATCATCTTCAAGATTTGCACA<br>GAAAACTCCTTTGTAAGAACACACATTTGAACCAACCCAAGA<br>AGCTAAAATCTTTAAA
Ca_LG_8:13532638	AAAAGTAAATTATACAAACATTTTCAAAAAAAAAAAAAATAATAA<br>CATATTGGATAATCAAAATAATATAGTGATTATTTTCATGCATC<br>TGGATGGATACT	T/A	TTTGAATTGAGTCTTTGGTTCTCTCATTCCATGTTTCAACTTT<br>CAAGGTGACAGCAGCCTTTTCTTGTGTTTCTTTTTCTTTTGTT<br>TTTTCATCTTTGAT
Ca_LG_8:13532647	TTATACAAACATTTTCAAAAAAAAAAAAAATAATAACATATTGGA<br>TAATCAAAATAATATAGTGATTATTTTCATGCATCTGGATGGAT<br>ACTTTTGAATTG	G/T	AGTCTTTGGTTCTCTCATTCCATGTTTCAACTTTCAAGGTGAC<br>AGCAGCCTTTTCTTGTGTTTCTTTTTCTTTTGTTTTTTCATCTT<br>TGATTTGATTTTG
Ca_LG_8:13796464	CCGTGGTATGCGCCAATAAGACCACCAAAAGCCTTTGTGGCA<br>CTAGTGGTACACAGAAGTCATGGGTGATCATTGGTCCGATGC<br>TTCGGGCGAAACCAAT	T/C	TCCCAGGGTGTGACGGGCGGTGTGTACAGGGCCCGGGTAC<br>ATATTCACCGCGGCATGCTGATCCGCGATTACTAGCGATTC<br>CAACTTCATGTTCCCGAGT
Ca_LG_8:13796470	TATGCGCCAATAAGACCACCAAAAGCCTTTGTGGCACTAGTG<br>GTACACAGAAGTCATGGGTGATCATTGGTCCGATGCTTCGGG<br>CGAAACCAATTCCCAG	G/T	GGTGTGACGGGCGGTGTGTACAGGGCCCGGGTACATATTC<br>ACCGCGGCATGCTGATCCGCGATTACTAGCGATTCCAACTT<br>CATGTTCCCGAGTTGCAGA
Ca_LG_8:13796493	AGCCTTTGTGGCACTAGTGGTACACAGAAGTCATGGGTGATC<br>ATTGGTCCGATGCTTCGGGCGAAACCAATTCCCAGGGTGTGA<br>CGGGCGGTGTGTACAG	G/A	GGCCCGGGTACATATTCACCGCGGCATGCTGATCCGCGATT<br>ACTAGCGATTCCAACTTCATGTTCCCGAGTTGCAGAGAACAA<br>TCCGAACTGAGGCAATC
Ca_LG_8:13796502	GGCACTAGTGGTACACAGAAGTCATGGGTGATCATTGGTCCG<br>ATGCTTCGGGCGAAACCAATTCCCAGGGTGTGACGGGCGGT<br>GTGTACAGGGCCCGGGT	T/A	ACATATTCACCGCGGCATGCTGATCCGCGATTACTAGCGATT<br>CCAACTTCATGTTCCCGAGTTGCAGAGAACAATCCGAACTG<br>AGGCAATCTTTCCGGAT
Ca_LG_8:13796505	ACTAGTGGTACACAGAAGTCATGGGTGATCATTGGTCCGATG<br>CTTCGGGCGAAACCAATTCCCAGGGTGTGACGGGCGGTGTG<br>TACAGGGCCCGGGTACA	A/G	TATTCACCGCGGCATGCTGATCCGCGATTACTAGCGATTCC<br>AACTTCATGTTCCCGAGTTGCAGAGAACAATCCGAACTGAG<br>GCAATCTTTCCGGATTCG
Ca_LG_8:13796519	GAAGTCATGGGTGATCATTGGTCCGATGCTTCGGGCGAAACC<br>AATTCCCAGGGTGTGACGGGCGGTGTGTACAGGGCCCGGGT<br>ACATATTCACCGCGGCA	A/G	TGCTGATCCGCGATTACTAGCGATTCCAACTTCATGTTCCCG<br>AGTTGCAGAGAACAATCCGAACTGAGGCAATCTTTCCGGAT<br>TCGCTCCGCCTTACAGC

**Positions**	**-100 bp flanking sequences**	**SNPs within *kabuli* cultivar**	**+100 bp flanking sequences**
Ca_LG_8:13796528	GGTGATCATTGGTCCGATGCTTCGGGCGAAACCAATTCCCAGGGTGTGACGGGCGGTGTGTACAGGGCCCGGGTACATATTCACCGCGGCATGCTGATCC	C/T	GCGATTACTAGCGATTCCAACTTCATGTTCCCGAGTTGCAGAGAACAATCCGAACTGAGGCAATCTTTCCGGATTCGCTCCGCCTTACAGCCTTGCTTCC
Ca_LG_8:13796658	CCGAGTTGCAGAGAACAATCCGAACTGAGGCAATCTTTCCGGATTCGCTCCGCCTTACAGCCTTGCTTCCCATTGTAATTGCCATTGTAGCACGTGTGTG	G/A	GCCCAGCCCATAAGGGCCATGCGGACTTGACGTCATCCCCACCTTCCTCCAGTATATCACTGGCAGTCCCTCGTGAGTGCGGCATGCACCTTTTAGTTTG
Ca_LG_8:13796666	CAGAGAACAATCCGAACTGAGGCAATCTTTCCGGATTCGCTCCGCCTTACAGCCTTGCTTCCCATTGTAATTGCCATTGTAGCACGTGTGTGGCCCAGCC	C/G	CATAAGGGCCATGCGGACTTGACGTCATCCCCACCTTCCTCCAGTATATCACTGGCAGTCCCTCGTGAGTGCGGCATGCACCTTTTAGTTTGTTTCGGAG
Ca_LG_8:13796668	GAGAACAATCCGAACTGAGGCAATCTTTCCGGATTCGCTCCGCCTTACAGCCTTGCTTCCCATTGTAATTGCCATTGTAGCACGTGTGTGGCCCAGCCCA	A/G	TAAGGGCCATGCGGACTTGACGTCATCCCCACCTTCCTCCAGTATATCACTGGCAGTCCCTCGTGAGTGCGGCATGCACCTTTTAGTTTGTTTCGGAGCC
Ca_LG_8:13796680	AACTGAGGCAATCTTTCCGGATTCGCTCCGCCTTACAGCCTTGCTTCCCATTGTAATTGCCATTGTAGCACGTGTGTGGCCCAGCCCATAAGGGCCATGC	C/A	GGACTTGACGTCATCCCCACCTTCCTCCAGTATATCACTGGCAGTCCCTCGTGAGTGCGGCATGCACCTTTTAGTTTGTTTCGGAGCCGTTTGTTTTGGC
Ca_LG_8:13797322	GACACGAGCTGACGACAGCCATGCAGCACCTGTATGAAAGTCAGTACCATCCCGTTAAGGACAGGTTTTCTTGTTCATATGTCAAGGGCTGGTAAGGTTT	T/C	TGCGCGTTGTATCGAATTAAACCACATGCTCCACCGCTTGTGCAGGCCCCCGTCAATTCCTTTGAGTTTCGGTCTTGCGACCGTACTCCCCAGGCGGAGT
Ca_LG_8:13797332	GACGACAGCCATGCAGCACCTGTATGAAAGTCAGTACCATCCCGTTAAGGACAGGTTTTCTTGTTCATATGTCAAGGGCTGGTAAGGTTTTGCGCGTTGT	T/C	ATCGAATTAAACCACATGCTCCACCGCTTGTGCAGGCCCCCGTCAATTCCTTTGAGTTTCGGTCTTGCGACCGTACTCCCCAGGCGGAGTGTTTCACGCG
Ca_LG_8:13797333	ACGACAGCCATGCAGCACCTGTATGAAAGTCAGTACCATCCCGTTAAGGACAGGTTTTCTTGTTCATATGTCAAGGGCTGGTAAGGTTTTGCGCGTTGTA	A/T	TCGAATTAAACCACATGCTCCACCGCTTGTGCAGGCCCCCGTCAATTCCTTTGAGTTTCGGTCTTGCGACCGTACTCCCCAGGCGGAGTGTTTCACGCGT
Ca_LG_8:13797366	TACCATCCCGTTAAGGACAGGTTTTCTTGTTCATATGTCAAGGGCTGGTAAGGTTTTGCGCGTTGTATCGAATTAAACCACATGCTCCACCGCTTGTGCA	A/G	GGCCCCCGTCAATTCCTTTGAGTTTCGGTCTTGCGACCGTACTCCCCAGGCGGAGTGTTTCACGCGTTAGCTGGGCCCCTGATCCACGTAGACCAAGGGC
Ca_LG_8:13797392	TTGTTCATATGTCAAGGGCTGGTAAGGTTTTGCGCGTTGTATCGAATTAAACCACATGCTCCACCGCTTGTGCAGGCCCCCGTCAATTCCTTTGAGTTTC	C/T	GGTCTTGCGACCGTACTCCCCAGGCGGAGTGTTTCACGCGTTAGCTGGGCCCCTGATCCACGTAGACCAAGGGCGAACACTCATCGTTTACGGCATGGAC
Ca_LG_8:13797393	TGTTCATATGTCAAGGGCTGGTAAGGTTTTGCGCGTTGTATCGAATTAAACCACATGCTCCACCGCTTGTGCAGGCCCCCGTCAATTCCTTTGAGTTTCG	G/A	GTCTTGCGACCGTACTCCCCAGGCGGAGTGTTTCACGCGTTAGCTGGGCCCCTGATCCACGTAGACCAAGGGCGAACACTCATCGTTTACGGCATGGACT
Ca_LG_8:13797394	GTTCATATGTCAAGGGCTGGTAAGGTTTTGCGCGTTGTATCGAATTAAACCACATGCTCCACCGCTTGTGCAGGCCCCCGTCAATTCCTTTGAGTTTCGG	G/A	TCTTGCGACCGTACTCCCCAGGCGGAGTGTTTCACGCGTTAGCTGGGCCCCTGATCCACGTAGACCAAGGGCGAACACTCATCGTTTACGGCATGGACTA

Positions	-100 bp flanking sequences	SNPs within *kabuli* cultivar	+100 bp flanking sequences
Ca_LG_8:13797395	TTCATATGTCAAGGGCTGGTAAGGTTTTGCGCGTTGTATCGAA<br>TTAAACCACATGCTCCACCGCTTGTGCAGGCCCCCGTCAATT<br>CCTTTGAGTTTCGGT	T/C	CTTGCGACCGTACTCCCCAGGCGGAGTGTTTCACGCGTTAG<br>CTGGGCCCCTGATCCACGTAGACCAAGGGCGAACACTCATC<br>GTTTACGGCATGGACTAC
Ca_LG_8:13797487	GTTTCGGTCTTGCGACCGTACTCCCCAGGCGGAGTGTTTCAC<br>GCGTTAGCTGGGCCCCTGATCCACGTAGACCAAGGGCGAAC<br>ACTCATCGTTTACGGCA	A/G	TGGACTACCAGGGTATCTAATCCCGTTCGCTCCCCATGCTTT<br>CGCACTCCAGCGTCGGTAGGGACCCAGAGAGCTGCCTTCG<br>CTTTTGGCGTTCCTTCGT
Ca_LG_8:13797511	CCAGGCGGAGTGTTTCACGCGTTAGCTGGGCCCCTGATCCA<br>CGTAGACCAAGGGCGAACACTCATCGTTTACGGCATGGACTA<br>CCAGGGTATCTAATCCC	C/T	GTTCGCTCCCCATGCTTTCGCACTCCAGCGTCGGTAGGGAC<br>CCAGAGAGCTGCCTTCGCTTTTGGCGTTCCTTCGTAGATCT<br>GCGGATTTCACCCCTACA
Ca_LG_8:13797515	GCGGAGTGTTTCACGCGTTAGCTGGGCCCCTGATCCACGTAG<br>ACCAAGGGCGAACACTCATCGTTTACGGCATGGACTACCAGG<br>GTATCTAATCCCGTTC	C/T	GCTCCCCATGCTTTCGCACTCCAGCGTCGGTAGGGACCCAG<br>AGAGCTGCCTTCGCTTTTGGCGTTCCTTCGTAGATCTGCGG<br>ATTTCACCCCTACACACG
Ca_LG_8:13797524	TTCACGCGTTAGCTGGGCCCCTGATCCACGTAGACCAAGGGC<br>GAACACTCATCGTTTACGGCATGGACTACCAGGGTATCTAAT<br>CCCGTTCGCTCCCCAT	T/C	GCTTTCGCACTCCAGCGTCGGTAGGGACCCAGAGAGCTGC<br>CTTCGCTTTTGGCGTTCCTTCGTAGATCTGCGGATTTCACCC<br>CTACACACGAAATTCCAC
Ca_LG_8:13797885	GCCGGGGCTTCTTCCTCGAGTCCTGTCATGATCGCGCACTCG<br>ACGAAAGAGCTTTACAAGCGGCATTGCCCTTCTTCACTCACG<br>CGATATTGCTGGATCG	G/A	GGCTTTCGCCCATTGTCCAAGATTCCCCACTGCTGCCCCCC<br>GTGGGAGTCCGGGCCGTGTCTCAGTCCCAGTGTGGCTGAT<br>CATCCGAAAAGACCAGCTA
Ca_LG_8:13797923	CTCGACGAAAGAGCTTTACAAGCGGCATTGCCCTTCTTCACT<br>CACGCGATATTGCTGGATCGGGCTTTCGCCCATTGTCCAAGA<br>TTCCCCACTGCTGCCC	C/T	CCCGTGGGAGTCCGGGCCGTGTCTCAGTCCCAGTGTGGCT<br>GATCATCCGAAAAGACCAGCTAAGCATCATTGGCTTGGTCA<br>GCCTTTACCTTACCAACTA
Ca_LG_8:13797929	GAAAGAGCTTTACAAGCGGCATTGCCCTTCTTCACTCACGCG<br>ATATTGCTGGATCGGGCTTTCGCCCATTGTCCAAGATTCCCCA<br>CTGCTGCCCCCCGTG	G/A	GGAGTCCGGGCCGTGTCTCAGTCCCAGTGTGGCTGATCATC<br>CGAAAAGACCAGCTAAGCATCATTGGCTTGGTCAGCCTTTA<br>CCTTACCAACTACCTAAT
Ca_LG_8:13797936	CTTTACAAGCGGCATTGCCCTTCTTCACTCACGCGATATTGCT<br>GGATCGGGCTTTCGCCCATTGTCCAAGATTCCCCACTGCTGC<br>CCCCCGTGGGAGTCC	C/T	GGGCCGTGTCTCAGTCCCAGTGTGGCTGATCATCCGAAAAG<br>ACCAGCTAAGCATCATTGGCTTGGTCAGCCTTTACCTTACCA<br>ACTACCTAATACTACGC
Ca_LG_8:15577061	GCCATTTTTTGGCGGTACGTTAGCAGCACGTCTATCGATTGT<br>GGCTATGCTTGTGAAATACAATTTGAATTTTAAATTGACAAATT<br>GTTTGTTGTCTACT	T/A	TTAGCAGCATGGCTAAAATTGAGCTGCCATATTTTGCAACTG<br>AGATTACAACTCCATCAAGCCCCTTTGCACTATCTCTAGCAA<br>AGTATTTGCATTCCAT
Ca_LG_8:16665228	GCTACTAATGTCTGTTTAGCGTCGACCTCACGGACGCTAAATT<br>TGCTATATTGTTATTCATTGACATGGTATCAACGCATCTATTGA<br>CCAAGTGGTCAAA	A/G	AGTTCAATCCATGCAGCTCAACTATCTTCACATTTAATTACAT<br>TAGATTATAGCAAATTGTATGTTAGAGATGAAAATCATTCTTG<br>GTTTTATACCAATT
Ca_LG_8:16855154	CGAACATAATTCACTAGGAACCAACCAACCCAATAGACCCCA<br>AGACCAAGCATAAGCTGCAACATAGCCATATATGAAAAATAAG<br>AGGAGATTGGCTTCA	A/T	GCTTTAGTTAACGAGCCTTCACCACTTAGTCCAAACTTTACA<br>ACAATCATTCCTCCAATAGAAATCTACAAGTAATTAATGGTTG<br>TAACATTTATATATA

**Positions**	**-100 bp flanking sequences**	**SNPs within *kabuli* cultivar**	**+100 bp flanking sequences**
Ca_LG_8:16855155	GAACATAATTCACTAGGAACCAACCAACCCAATAGACCCCAAGACCAAGCATAAGCTGCAACATAGCCATATATGAAAAATAAGAGGAGATTGGCTTCAG	G/C	CTTTAGTTAACGAGCCTTCACCACTTAGTCCAAACTTTACAACAATCATTCCTCCAATAGAAATCTACAAGTAATTAATGGTTGTAACATTTATATATAC
Ca_LG_8:17316273	GAATATAAGGAAGAGCCCTTTACATCAACCCAATTTCGGCAACAATGCTGCTAGACAACAGCCTCAGCCTCAGGTTTACAATATAAGCAAGAACGATTTT	T/C	CGGGATATTGTTCAGCAGCTTACTGGATCACCTTCACAGGATCCTCCACCTCGGCCTCCACATAATCCGCCAAAACCGCAGAGCATGCGTTTGCAGAAGA
Ca_LG_8:17354072	TGCTGTTTTCTGCCATCAATAAAAACATTATAAAGCCGAAACTCTGCATTCAATGTTAGAGATTTTTTTTTATTGAATTATGAAGGGGAGTAGAGAAATG	G/A	AGGCATACATACCTGTGCAACAGGGGCATTGTCATCTATAAATGGTGCTGCAAGACTTATGAAGGAAGCCTCACCAACACCAACTAGCCTGGGAAACAAA
